# Selective inhibition of the K^+^ efflux sensitive NLRP3 pathway by Cl^−^ channel modulation†

**DOI:** 10.1039/d0sc03828h

**Published:** 2020-10-12

**Authors:** Tessa Swanton, James A. Beswick, Halah Hammadi, Lucy Morris, Daniel Williams, Stephane de Cesco, Lina El-Sharkawy, Shi Yu, Jack Green, John B. Davis, Catherine B. Lawrence, David Brough, Sally Freeman

**Affiliations:** Division of Neuroscience and Experimental Psychology, School of Biological Sciences, Faculty of Biology, Medicine and Health, Manchester Academic Health Science Centre, University of Manchester AV Hill Building, Oxford Road Manchester M13 9PT UK David.brough@manchester.ac.uk; Lydia Becker Institute of Immunology and Inflammation, University of Manchester Manchester M13 9PT UK; Division of Pharmacy and Optometry, School of Health Sciences, Faculty of Biology, Medicine and Health, Manchester Academic Health Science Centre, University of Manchester Stopford Building, Oxford Road Manchester M13 9PT UK Sally.freeman@manchester.ac.uk; Alzheimer's Research UK Oxford Drug Discovery Institute, Target Discovery Institute NDM Building, Roosevelt Drive Oxford OX3 7FZ UK

## Abstract

The NLRP3 inflammasome regulates production of the pro-inflammatory cytokines interleukin-1β (IL-1β) and IL-18, and contributes to inflammation exacerbating disease. Fenamate non-steroidal anti-inflammatory drugs (NSAIDs) were recently described as NLRP3 inflammasome inhibitors *via* chloride channel inhibition. Fenamate NSAIDs inhibit cyclooxygenase (COX) enzymes, limiting their potential as therapeutics for NLRP3-associated diseases due to established side effects. The aim here was to develop properties of the fenamates that inhibit NLRP3, and at the same time to reduce COX inhibition. We synthesised a library of analogues, with feedback from *in silico* COX docking potential, and IL-1β release inhibitory activity. Through iterative screening and rational chemical design, we established a collection of chloride channel inhibiting active lead molecules with potent activity at the canonical NLRP3 inflammasome and no activity at COX enzymes, but only in response to stimuli that activated NLRP3 by a K^+^ efflux-dependent mechanism. This study identifies a model for the isolation and removal of unwanted off-target effects, with the enhancement of desired activity, and establishes a new chemical motif for the further development of NLRP3 inflammasome inhibitors.

## Abstract 2

The NLRP3 inflammasome regulates production of the pro-inflammatory cytokines interleukin-1β (IL-1β) and IL-18, and contributes to inflammation exacerbating disease.

## Graphical abstract

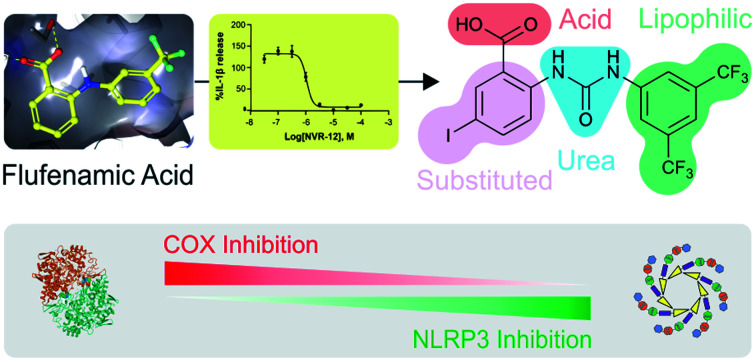

## Introduction

Inflammation is known to contribute to the worsening of many diseases, and is frequently associated with the activation of the NOD-like receptor pyrin domain-containing protein 3 (NLRP3) inflammasome.^1^ NLRP3 is studied mainly in cells of the innate immune system such as macrophages where it responds to danger in the form of pathogen or damage-associated molecular patterns (PAMPs or DAMPs respectively). Upon sensing danger NLRP3 interacts with an adaptor protein called apoptosis-associated speck-like protein containing a CARD (ASC) causing its oligomerisation into an activating platform for the protease caspase-1. Caspase-1 then cleaves pro-inflammatory cytokine precursors pro-IL-18 and pro-IL-1β into active forms that are then secreted from the cell.^1^ Caspase-1 also cleaves the pore-forming protein gasdermin D (GSDMD) which subsequently forms membrane pores causing pyroptotic cell death.^2,3^ A number of inhibitors of the NLRP3 inflammasome have been described such as CRID3/MCC950/CP-456773 which binds directly to NLRP3.^4,5^ We previously reported that fenamate NSAIDs are also able to inhibit NLRP3 inflammasome activation *in vitro* and *in vivo* by virtue of their ability to act on Cl^−^ channels.^6^ Targeting the regulatory pathways of NLRP3 may complement strategies to inhibit the protein directly. Fenamate targeting of Cl^−^ channels however is complicated by their primary effects at cyclooxygenase (COX) enzymes which is associated with significant side effects with long term use.

Thus, the aim of this study was to develop the NLRP3 inhibiting properties of the fenamate scaffold and deselect the COX inhibiting properties. Using cycles of iterative chemistry, computational modelling of COX inhibition and biological measurement of effects against the NLRP3 inflammasome, we have developed inhibitors that block NLRP3 *via* Cl^−^ channel inhibition and that are devoid of activity against COX. Furthermore, targeting Cl^−^ channels only inhibited K^+^-dependent canonical NLRP3 activation. This offers the advantage now of selective NLRP3 pathway modulation in diseases which may help mitigate potentially immunosuppressive effects of a blanket NLRP3 inhibition.

## Experimental

### Chemistry

Full details of the synthesis and characterisation for all compounds are provided in the ESI Chemistry file.† All other pharmacological reagents were obtained from Sigma (Niflumic acid (NFA), Tolfenamic acid (TFA), Flufenamic acid (FFA), Clonixin, Furosemide, SB 225002, U-104, S4, Celecoxib, Mefenamic acid, Bay 11-7082, MCC950 and Ac-Tyr–Val–Ala–Asp-chloromethylketone (Ac-YVAD-CMK)).

### Cell culture

#### Primary BMDMs

For the preparation of primary bone marrow derived macrophages (BMDMs), wild-type C57BL/6 (Charles River) mice were euthanized by rising CO_2_, followed by cervical dislocation. All procedures were carried out in accordance with the Home Office (Animals) Scientific Procedures Act (1986). Bone marrow was isolated from the femur and tibia bones of C57BL/6 mice and re-suspended in ACK lysis buffer (Fisher Scientific) for lysis of red blood cells. The remaining cells were cultured in L929-containing Dulbecco's Modified Eagle's Medium (DMEM) with 10% (vol/vol) fetal bovine serum (FBS, Life Technologies), 100 U ml^−1^ penicillin and 100 μg ml^−1^ streptomycin (1% P/S, Sigma). On day 6–7, cells were scraped and seeded overnight at a density of 1 × 10^6^ ml^−1^ in 24- or 96-well plates.

#### ASC-mCherry iBMDMs

Immortalised BMDMs stably expressing ASC conjugated to mCherry^6^ were cultured in DMEM with 10% (vol/vol) FBS and 1% (vol/vol) P/S and seeded overnight at a density of 0.75 × 10^6^ ml^−1^ in black-walled, clear bottom 96 well plates.

#### CD14^+^ monocytes

Full consent from human volunteers (National Health Service Blood and Transplant, Manchester, UK) and ethical approval from the Research Governance, Ethics, and Integrity Committee at The University of Manchester was obtained prior to experiments (ref. 2018-2696-5711). Blood was collected from healthy human donors and peripheral blood mononuclear cells (PBMCs) were isolated using Ficoll (Thermofisher) gradient density centrifugation at 500 × *g* for 40 min at room temperature. The PBMC layer was collected and platelets, plasma proteins and further contaminants were removed by washing with MACS buffer (PBS containing 0.5% (w/vol) BSA and 2 mM EDTA) followed by centrifugation at 500 × *g* for 10 min at room temperature. PBMCs were incubated with MACS CD14^+^ MicroBeads (Miltenyi) for 15 min at 4 °C and CD14^+^ monocytes were pulled out using a LS column (Miltenyi). CD14^+^ monocytes were cultured in RPMI-1640 (Thermofisher) supplemented with 1% (vol/vol) FBS, 2 mM l-glutamine (Sigma) and 1% (vol/vol) P/S and seeded at a density of 2 × 10^6^ ml^−1^ in round-bottom 96-well plates.

#### HeLa cells

HeLa cells were cultured in DMEM containing 10% (vol/vol) FBS and 1% (vol/vol) P/S and seeded at a density of 1 × 10^5^ ml^−1^ overnight before transfection the following morning.

### Inflammasome assays

BMDMs were primed with 1 μg ml^−1^ lipopolysaccharide (LPS) from *Escherichia coli* (serotype O26:B6, Sigma) in DMEM containing 10% (vol/vol) FBS and 1% (vol/vol) P/S for 4 h, followed by pre-treatment with NVR compound (10 μM), NS3728 (10 μM), MCC950 (10 μM) or vehicle (DMSO (0.5%, vol/vol), Sigma) in serum-free DMEM at indicated concentrations for 15 min. To induce canonical NLRP3 activation, 5 mM ATP (Sigma) or 75 μM imiquimod (InvivoGen) was added directly to wells for 1 or 2 h, respectively. Alternatively, hypotonic buffer (27 mM NaCl, 0.54 mM KCl, 0.3 mM KH_2_PO_4_, 0.5 mM MgCl_2_, 0.9 mM CaCl_2_, 20 mM HEPES, 5 mM NaHCO_3_ and 3 mM glucose, pH 7.4, 117 mOsm kg^−1^ (ref. 7)) was added to LPS-primed (1 μg ml^−1^; 4 h) BMDMs for 4 h pre-treated with NVR compound (10 μM), NS3728 (10 μM), MCC950 (10 μM) or vehicle (DMSO (0.5%)) to induce NLRP3 activation. Isotonic buffer (132 mM NaCl, 2.6 mM KCl, 1.4 mM KH_2_PO_4_, 0.5 mM MgCl_2_, 0.9 mM CaCl_2_, 20 mM HEPES, 5 mM NaHCO_3_ and 3 mM glucose, pH 7.4, 340 mOsm kg^−1^ (ref. 7)) was used as control. For NLRC4 or AIM2 inflammasome activation, LPS-primed (1 μg ml^−1^; 4 h) BMDMs were pre-treated with NVR compound (10 μM), NS3728 (10 μM), MCC950 (10 μM), Ac-YVADCMK (100 μM) or vehicle (DMSO (0.5%)) for 15 min in serum-free DMEM and then transfected with either 1 μg ml^−1^ flagellin from *Salmonella typhimurium* (Invivogen) or 1 μg ml^−1^ poly(deoxyadenylic-thymidylic) acid sodium salt (poly(dA:dT), Sigma), respectively, using Lipofectamine 3000 (Thermofisher) per manufacturer's instructions, for 4 h. For BMDM priming experiments, cells were pre-treated with NVR compound (10 μM), MCC950 (10 μM) or vehicle (DMSO (0.5%)) in serum-free DMEM for 15 min followed by 4 h treatment with 1 μg ml^−1^ LPS. For alternative inflammasome activation, primary human CD14^+^ monocytes were seeded and immediately treated with 1 μg ml^−1^ LPS in RPMI-1640 supplemented with 1% FBS, 2 mM l-glutamine and 1% P/S for 20 h in the presence of NVR compound (10 μM), NS3728 (10 μM), MCC950 (10 μM) or vehicle (DMSO (0.5%).

### ELISA analysis

IL-1β, IL-6 and TNF, were analysed by ELISA according to manufacturer's instructions (DuoSet, R&D Systems).

### Western blot

Lysates and/or supernatants were assessed by western blot for NLRP3, IL-1β, caspase-1, and GSDMD. Samples were run on SDS polyacrylamide gels and transferred onto nitrocellulose or PVDF membranes using a semi-dry Trans-blot Turbo system (Bio-Rad) at 25 V. Membranes were blocked with 2.5% BSA in phosphate-buffered saline, 0.1% Tween 20 (Sigma) (PBST) for 1 h before overnight incubation at 4 °C with mouse anti-NLRP3 monoclonal antibody (Cryo2, Adipogen), goat anti-IL-1β polyclonal antibody (AF-401, R&D Systems), rabbit anti-caspase-1 + p10 + p12 monoclonal antibody (EPR16883, Abcam), or rabbit anti-GSDMD antibody (EPR19828, Abcam) in 2.5% BSA PBS-T. The following morning, membranes were washed (5 min, ×3) in PBST and subsequently incubated with either rabbit anti-mouse, rabbit anti-goat or goat anti-rabbit HRP antibodies (Dako) in 2.5% PBST for 1 h at room temperature. β-Actin was used as a sample loading control using a monoclonal anti-β-Actin-peroxidase antibody (Sigma). After washing, Amersham ECL prime detection reagent (GE Healthcare) was added to membranes and images were taken with a G:Box Chemi XX6 (Syngene) scanner.

### ASC speck imaging

ASC-mCherry iBMDMs were primed with 1 μg ml^−1^ LPS in DMEM containing 10% FBS and 1% P/S for 2 h. Media was replaced with OptiMEM (Thermofisher) containing the irreversible caspase-1 inhibitor Ac-YVAD-CMK (100 μM; Sigma) to prevent pyroptosis and either NVR compound (10 μM), NS3728 (10 μM), MCC950 (10 μM) or vehicle (DMSO (0.5%)) for 15 min followed by stimulation with ATP (5 mM). ASC speck formation was assessed in real-time with an IncuCyte ZOOM system (Essen Bioscience). Images were acquired using a 10×/0.3 s Plan Fluor objective every 10 min for a period of 90 min. ASC speck formation was compared between treatments at the 90 min time point. Specks were counted using ImageJ and speck formation was calculated as a percentage of vehicle-treated cells.

### COX2 *in silico* selectivity model

The structure of COX2 (PDB CODE 5IKQ) was prepared using Schrodinger 2019.3. All water and ligands were removed, and protonation states were adjusted (pH = 7.4) along with the optimization of the side chain orientations. The grid was then prepared using Schrodinger grid preparation tool and was used in a KNIME workflow for further docking and classification tasks. Ligands were prepared using LigPrep and docking performed using Glide SP. Five known COX inhibitors (Niflumic acid, Tolfenamic acid, Clonixin, Flufenamic acid and Tromaril) were docked and their average docking score and standard deviation were determined (average docking score = −8.35; *σ* = 1.61). The standard deviation (*σ*) was then used to classify other docked ligands into different categories: high risk < −8.35 + 1*σ*, −8.35 + 1*σ* < medium high < −8.35 + 2*σ*, −8.35 + 2*σ* < medium low risk < −8.35 + 3*σ*, and low risk > −8.35 + 3*σ*. Physicochemical parameters were also computed using ChemAxon software to calculate the MPO and BBB score.

### COX assays

A Cayman chemical COX activity assay (Cayman chemical, catalogue number 760111) was used to assay COX inhibition for each drug (Mefenamic acid, Celecoxib, NVR compound or NS3728 (10 μM)) or vehicle (DMSO (0.5%)). Drugs were assayed in duplicate in a 96 well plate in independent experiments. Purified COX1 or COX2 were incubated with each drug or vehicle at indicated concentrations for 30 min before addition of arachidonic acid, KOH and colorimetric substrate as per the manufacturer's instructions. After 2 min, absorbance at 590 nm was read using a plate reader (Synergy HT, BioTek). For calculation of COX activity, background absorbance from wells containing no COX1 or COX2 was first subtracted from each value. Total COX activity for each drug was then calculated by comparison of absorbance values to wells containing vehicle (reference value set as 100% to which all other absorbance values were compared) and expressed as the percentage of total COX activity.

### Iodide quenching assay

HeLa cells were transfected with pcDNA3.1EYFP H148Q, kindly provided by Peter Haggie (Addgene #25872), for 18–24 h using Lipofectamine 3000 (Thermofisher) as per manufacturer's instructions. Cells were washed twice with isotonic buffer (310 mOsm kg^−1^, 140 mM NaCl, 5 mM KCl, 20 mM HEPES, pH 7.4) and then incubated in 50 μl isotonic buffer containing NVR compound (10 μM) or vehicle (DMSO (0.5%)) at 37 °C for 5 min. 50 μl hypotonic (120 mOsm kg^−1^, 5 mM KCl, 20 mM HEPES, 90 mM mannitol, pH 7.4) solution containing either NVR compound (10 μM) or vehicle (DMSO (0.5%)) was then added directly to wells for 5 min. Wells treated with isotonic buffer were used as controls. To induce quenching of YFP fluorescence, 25 μl of 200 mM sodium iodide (NaI; Sigma) was then spiked directly into the well and fluorescence readings were taken every 2 s for 1 min using the FlexStation 3 plate reader. Baseline fluorescence readings (*F*_0_) before the addition of NaI were taken in order to calculate the fluorescence intensity ratio (*F*/*F*_0_).

### Statistical analyses

All data are presented as mean ± S.E.M unless stated otherwise. Statistical analyses were performed on GraphPad Prism 8. Data were assessed for normality and homoscedasticity using Shapiro Wilk's and Levene's test, respectively, and transformed where appropriate. Where data is normalised as a percentage, a one-tailed, one-sample *t*-test was performed to determine significant difference from 100%, followed by Holm–Sidak correction. A one-way or two-way ANOVA with either Dunnett's or Holm–Sidak *post hoc* analysis was used to compare treatments against a vehicle control, or selected treatments, respectively, where accepted levels of significance were *p < 0.05, **p < 0.01, ***p < 0.001 and ****p < 0.0001.

## Results and discussion

In the first instance we synthesised fenamate analogues (designated as NVR compounds) where we (A) linked the phenyl rings by an amine (ESI Fig. 1†), (B) linked the phenyl rings by an amide (ESI Fig. 2†), and (C) linked the phenyl rings by a urea. The carboxylic acid group in the fenamates was also replaced with a tetrazole ring, and analogues with a range of substituents on the phenyl rings were prepared (ESI Fig. 3†). Other modifications were made including ureas lacking an acidic group (ESI Fig. 4†), cyclised urea analogues (ESI Fig. 5†), a series of unsubstituted linker analogues (ESI Fig. 6†), cyclic boron-containing analogues (ESI Fig. 7†), as well as three commercially available bioactive ureas and furosemide (ESI Fig. 8†). These molecules were screened in LPS (1 μg ml^−1^, 4 h)-primed mouse primary bone marrow-derived macrophages (BMDMs), being added at 10 μM just before the addition of ATP (5 mM, 60 min) to activate NLRP3. IL-1β release was quantified by ELISA. From this experiment the class of molecule with the greatest inhibition of NLRP3-dependent IL-1β release were those with the urea linker (ESI Fig. 3†), with the amine and amide linker series, and the other analogues possessing minimal inhibitory activity at this concentration (ESI Fig. 1–2 and 4–8).†

**Fig. 1:**
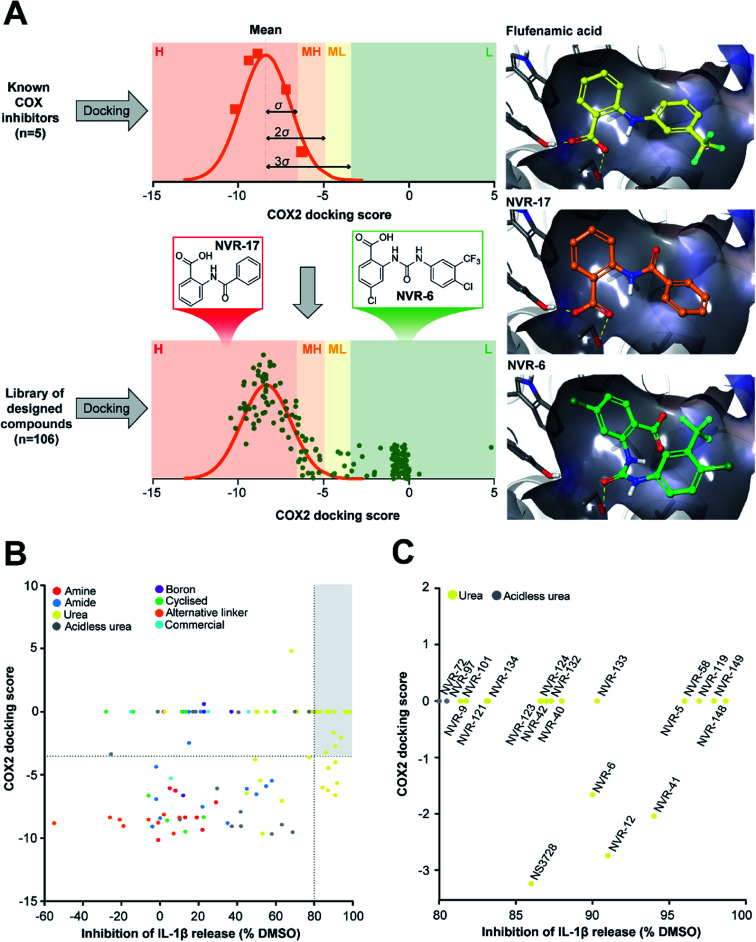
Identification of a sub-set of urea-based molecules with potent NLRP3 inhibition and low COX2 activity. (A) Representation of the workflow used to classify the risk of COX2 inhibition. The known COX inhibitors Niflumic acid, Tolfenamic acid, Clonixin, Flufenamic acid and Tromaril were docked into the COX2 crystal structure (PDB code 5IKQ). Their docking score was averaged (mean docking score = −8.35) and was used along with the standard deviation (*σ*, *σ* = 1.61) to classify the designed compounds (*n* = 106) in to various risk categories: high (H), medium high (MH), medium low (ML) and low (L) risk. (B) Murine bone marrow-derived macrophages (BMDMs) were primed with LPS (1 μg ml^−1^, 4 h) followed by treatment with NVR compound (10 μM), NS3728 (10 μM), or vehicle (DMSO, 0.5%) for 15 min before stimulation with ATP (5 mM, 1 h). Supernatants were collected and IL-1β release was determined by ELISA. Docking scores were then plotted against the % inhibition of IL-1β release at 10 μM. Compounds in the low-risk COX2 category that inhibited IL-1β release by more than 80% (compared to vehicle) are shown in the grey quadrant. (C) Highlighted compounds from B with high IL-1β release inhibition and low COX activity. Data are presented as mean percentage inhibition of IL-1β release compared to vehicle control of at least two experiments.

As stated above, NSAIDs are established COX inhibitors thus the fenamates cannot be considered as selective NLRP3 inhibitors. To screen the potential for COX inhibition of the new analogues we established a work-flow that used docking as a proxy for the likelihood of interaction with COX enzymes (Fig. 1A). Given that NSAID-related side effects are most closely associated with inhibition of COX2 enzymes, all molecules, including the known COX inhibitors Niflumic acid, Tolfenamic acid, Clonixin, Flufenamic acid and Tromaril, were docked in the COX2 crystal structure (PDB code 5IKQ) (Fig. 1A). The docking score of the known COX inhibitors was calculated (average docking score = −8.35) and the standard deviation (*σ* = 1.61) was used to classify molecules into risk categories. A score of < −8.35 + 1*σ* was considered high-risk, whereas the other risk categories were as follows: −8.35 + 1*σ* < medium high < −8.35 + 2*σ*, −8.35 + 2*σ* < medium low risk < −8.35 + 3*σ*, and Low risk > −8.35 + 3*σ* (Fig. 1A and ESI Fig. 9†). Docking scores were then plotted against the % inhibition of IL-1β release at 10 μM as determined above (Fig. 1B). From this we were able to determine a sub-set of molecules with high (>80%) inhibition of IL-1β release at 10 μM and low predicted COX activity (Fig. 1C). For this sub-set COX inhibitory activity was measured using recombinant COX1 and COX2 enzymes and confirmed that at 10 μM, where we observed significant inhibition of IL-1β release in a cellular assay, these NVR molecules were devoid of COX inhibitory activity (ESI Fig. 10†).

**Fig. 2:**
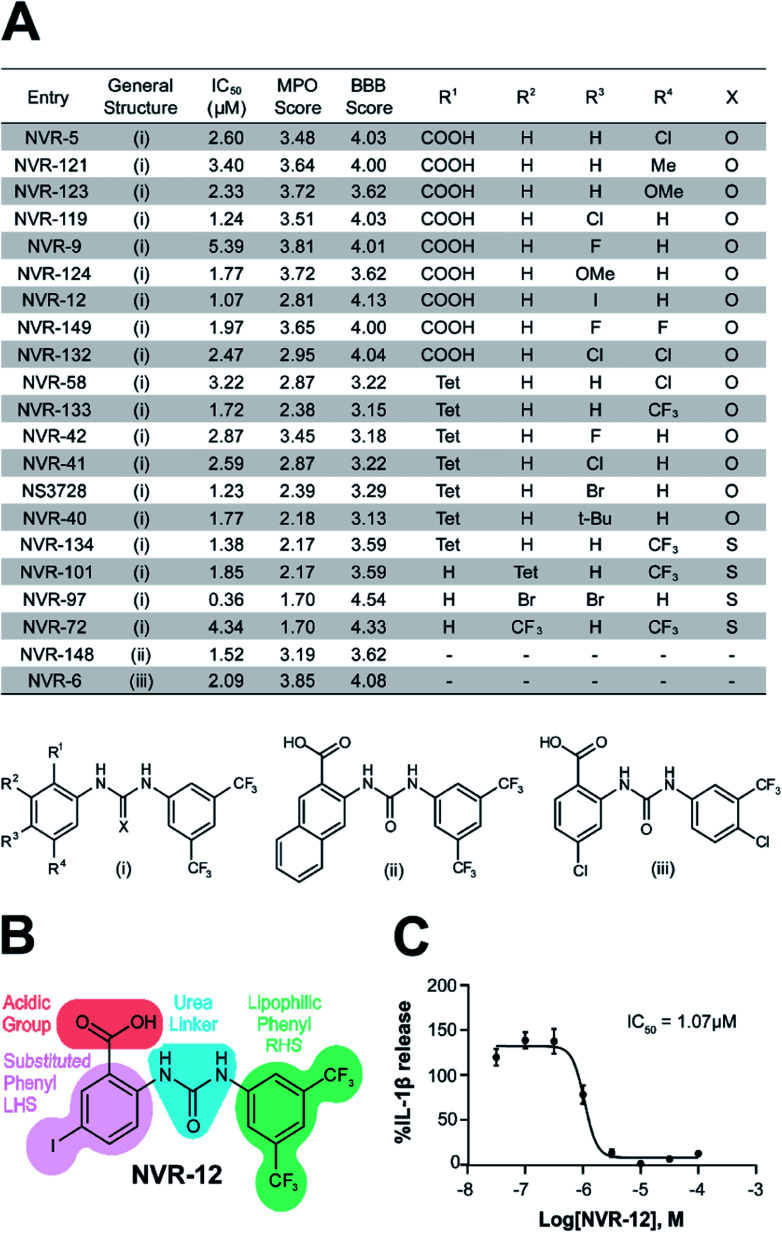
Characterisation of the properties of the improved inflammasome inhibiting sub-set. (A) Table of the most active urea-based sub-set of NLRP3 inhibitors with the general structure type **(i)**, **(ii)** or **(iii)**. Tet = 1H-tetrazol-5-yl. To obtain IL-1β half-maximal inhibitory concentration (IC_50_) values for these inhibitors, supernatants were collected from LPS-primed BMDMs (1 μg ml^−1^; 4 h) pre-treated with either NVR compound, NS3728 (0.03–100 μM) or vehicle (DMSO, 0.5%) for 15 min before stimulation with ATP (5 mM; 1 h). IL-1β release was assessed by ELISA and data are expressed as a mean percentage *versus* vehicle of at least three independent experiments. Dose–response curves were fitted using either a 3- or 4-parameter logistical sigmoidal model. Physicochemical properties were also calculated using ChemAxon software to generate the multiparameter optimisation (MPO) and blood–brain barrier (BBB) score, predictors of CNS permeability. (B) Structure–activity relationship of inhibition of NLRP3-dependent IL-1β release as exemplified by the structure of NVR-12. (C) IC_50_ graph for NVR-12, a representative molecule from the sub-set of urea-based inhibitors.

We screened this sub-set of molecules with high IL-1β inhibition and no COX activity at a range of concentrations in order to determine their IC_50_ values against LPS and ATP-induced IL-1β release in primary BMDMs (Fig. 2A and ESI Fig. 11†). NLRP3 activation is associated with a number of diseases, including those of the central nervous system (CNS), making it an attractive therapeutic target.^1,8^ To obtain information about the likelihood of CNS permeability of these molecules, multiparameter optimisation (MPO,^9^) and blood–brain barrier (BBB,^10^) scores were also generated based on physicochemical properties (Fig. 2A and ESI Fig. 9†), where scores ≥ 4 indicate greater likelihood of CNS activity.^9,10^ Further structure activity relationship (SAR) analysis of this subset showed the importance of an acid group, with substituents well tolerated on the left hand side (LHS), and the requirement for a lipophilic aryl ring on the right hand side (RHS) (Fig. 2B).

**Fig. 3:**
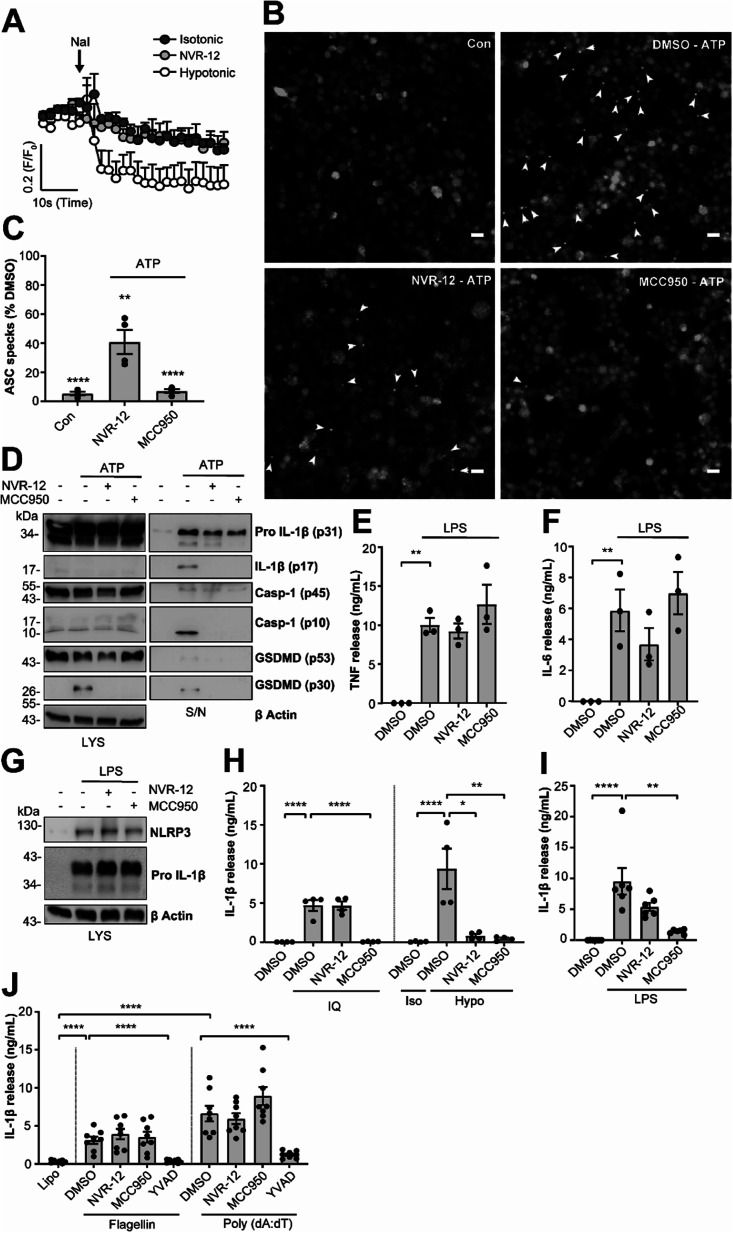
NVRs are selective inhibitors of the K^+^ efflux sensitive NLRP3 activation pathway. (A) HeLa cells were transfected with the halide-sensitive YFP mutant (EYFP H148Q/I152L). Cells were placed in either isotonic (310 mOsm kg^−1^) or hypotonic buffer (215 mOsm kg^−1^) containing either NVR-12 (10 μM) or vehicle (DMSO; 0.5%) for 5 min. Fluorescence readings were taken every 2 s before the addition of sodium iodide (NaI) to obtain an average baseline fluorescence value (*F*_0_). NaI (200 mM) was then spiked directly into the well to induce quenching of YFP fluorescence, and fluorescence readings were maintained every 2 s for a further 1 min. Data are expressed as mean *F*/*F*_0_ + S.E.M (*n* = 4). (B) LPS-primed (1 μg ml^−1^; 2 h) ASC-mCherry iBMDMs were pre-treated with NVR-12 (10 μM), MCC950 (10 μM) or vehicle (DMSO; 0.5%), and Ac-YVAD-CMK (100 μM) to prevent pyroptosis, for 15 min prior to the addition of ATP (5 mM; 90 min) under live microscopy. Scale bars are 20 μm. Specks are denoted by white arrows and (C) quantified and presented as a percentage *versus* vehicle (*n* = 4). Control (Con) is no ATP stimulation (DMSO; 0.5%). (D) Western blot of BMDM cell lysates and supernatants assessing IL-1β, caspase-1 and GSDMD processing. Primary murine BMDMs were treated with LPS (1 μg ml^−1^; 4 h) prior to treatment with either NVR-12 (10 μM), MCC950 (10 μM) or vehicle (DMSO; 0.5%) for 15 min before stimulation with ATP (5 mM, 1 h) (*n* = 3). (E–G) Primary murine BMDMs were pre-treated with either NVR-12 (10 μM), or MCC950 (10 μM) for 15 min prior to the addition of LPS (1 μg ml^−1^; 4 h) (*n* = 3); (E) TNF and (F) IL-6 release was measured by ELISA (*n* = 3), and (G) NLRP3 and pro-IL-1β expression was assessed by western blot in cell lysates (*n* = 4). (H) LPS-primed (1 μg ml^−1^; 4 h) BMDMs were pre-treated with either NVR-12 (10 μM), MCC950 (10 μM) or vehicle (DMSO; 0.5%) for 15 min prior to the addition of imiquimod (IQ, 75 μM; 2 h) (left), or hypotonic solution (117 mOsm kg^−1^; 4 h) (right). For hypotonicity experiments, isotonic buffer (340 mOsm kg^−1^) was used as a control (*n* = 4). (I) Human CD14^+^ monocytes were treated with either NVR-12 (10 μM), MCC950 (10 μM) or vehicle (DMSO; 0.5%) and LPS (1 μg ml^−1^; 20 h) (*n* = 6). (J) LPS-primed BMDMs (1 μg ml^−1^; 4 h) were pre-treated with NVR-12 (10 μM), MCC950 (10 μM), Ac-YVAD-CMK (100 μM) for 15 min followed by transfection with flagellin (1 μg ml^−1^), poly(dA:dT) (1 μg ml^−1^) or treated with lipofectamine alone for 4 h (*n* = 8). (E, F, H–J) Supernatants were collected and (E) TNF, (F) IL-6 or (H–J) IL-1β release was assessed by ELISA, where data are shown as mean ± S.E.M. (C) **p < 0.01, ****p < 0.0001 significant difference from 100% speck formation determined by a one-tailed, one-sample *t*-test with Holm–Sidak correction. (E, F, I and J) **p < 0.01, and ****p < 0.0001 determined by one-way ANOVA with Dunnett's (E, F and I) or Holm–Sidak (J) *post hoc* analysis. (H) *p < 0.05, **p < 0.01 and ****p < 0.0001 determined by two-way ANOVA with Dunnett's correction. Data were assessed for normality and homoscedasticity by performing a Shapiro–Wilks and Levene's test, respectively, and transformed where appropriate.

The mechanism of action of the selected sub-set of NVRs was then further characterised. NVR-12 (which inhibited LPS and ATP induced IL-1β release with an IC_50_ of 1 μM (Fig. 2A and C)) is presented as a representative compound in this characterisation, with the other selected best compounds included in ESI.† To test if NVR12 inhibited Cl^−^ channels we tested its effects against hypotonicity-induced Cl^−^ flux using I^−^ quenching of halide-sensitive YFP H148Q/I152L^11^ in HeLa cells (Fig. 3A). In this model, I^−^ enters the cell through open Cl^−^ channels to induce quenching of YFP. In response to hypotonic shock YFP fluorescence was immediately quenched, which was inhibited by NVR-12 (Fig. 3A). NVR-12 prevented the formation of ASC specks in LPS-primed-ASC-mCherry iBMDMs stimulated with ATP (Fig. 3B, C and ESI Fig. 12†). The inhibitory effects of NVR-12 on ATP-induced processing of pro-IL-1β, caspase-1, and gasdermin D were also shown by western blot (Fig. 3D). Significant levels of TNF and IL-6 were produced in response to LPS alone and in the presence of NVR-12 or MCC950 (Fig. 3E and F), and NVR-12 did not inhibit LPS-induced NLRP3 and pro-IL-1β expression (Fig. 3G), suggesting that the NVRs act downstream of priming.

Interestingly NVR-12 did not inhibit NLRP3 activation in response to the K^+^ efflux-independent NLRP3 agonist imiquimod, but did inhibit hypotonicity-induced NLRP3 activation (Fig. 3H and ESI Fig. 13†). So far we have focussed on the inhibition of the canonical NLRP3 activation pathway. The ‘alternative’ NLRP3 pathway has been described in primary human monocytes where LPS treatment alone activates NLRP3-dependent IL-1β release.^12^ LPS-induced IL-1β release occurs independently of K^+^ ion efflux.^12^ We tested the effects of our NVR sub-set against NLRP3-dependent IL-1β release from primary human monocytes in response to LPS. LPS-induced IL-1β release was inhibited by the direct NLRP3 inhibitor MCC950 but not by NVRs (Fig. 3I and ESI Fig. 14†). AIM2 and NLRC4 inflammasomes can be activated by transfection of poly(dA:dT) and flagellin respectively.^13^ The effects of the NVRs were specific to NLRP3 as NVRs had no effect on AIM2 or NLRC4 inflammasome dependent IL-1β release (Fig. 3J and ESI Fig. 15†) suggesting the NVRs specifically inhibit NLRP3. These data suggesting a K^+^ dependence are consistent with our recent report using ion substitution experiments to isolate the Cl^−^ dependence of ASC polymerisation, a feature of the canonical K^+^ efflux-dependent NLRP3 activation pathway.^14^ This discovery offers an advantage over directly targeting the NLRP3 protein where we can now target the canonical K^+^-dependent pathway in diseases where ASC speck formation is considered to drive disease pathology such as Alzheimer's disease,^15,16^ without targeting systemic peripheral NLRP3 responses to infection, thus reducing the potential of immune-suppression.

Understanding and targeting the NLRP3 inflammasome in disease has become an intensely researched subject.^17^ Initially studied as it inhibited the anion exchange activity of the ABC transporter ABC1, the sulfonylurea glyburide was found to inhibit ATP-induced IL-1β release from macrophages.^18^ Further work showing that diarylsulfonylureas were potent inhibitors of IL-1β release,^19^ eventually led to their direct target being identified to be NLRP3.^4,20^ Our work here showing that the NVRs do not inhibit K^+^ efflux-independent NLRP3 activation confirm that they are not direct NLRP3 inhibitors. Other NLRP3 inhibiting molecules have been described, many of which target parts of the activation pathway rather than NLRP3 directly (*e.g.*^21^). We discovered that fenamate NSAIDs have the potential to be repurposed as NLRP3 inhibitors by virtue of their ability to inhibit Cl^−^ channels.^6^ Starting with a fenamate scaffold, we isolated and enhanced the Cl^−^ channel and NLRP3 inhibiting potential to develop new urea analogues that have no effect on COX enzymes, thus negating a potentially harmful side effect of these molecules.

There are several pathways leading to the activation of NLRP3 which have been termed the canonical, non-canonical, and alternative pathways.^17^ The fact that the NVRs do not inhibit the alternative pathway in LPS treated primary human monocytes strongly suggests that they are not direct inhibitors, but rather work by inhibiting Cl^−^ channels that link to the K^+^ efflux dependent canonical pathway of NLRP3 activation. This aspect now allows the possibility of inhibiting only Cl^−^ sensitive pathways when they are known to contribute to disease and avoid inhibiting NLRP3 responses to infection such as the alternative pathway in monocytes. Our previous research showed that Cl^−^ efflux is essential for NLRP3-dependent ASC oligomerisation and formation of the ASC speck.^14^ Thus NVR based molecules could be used to inhibit NLRP3-dependent inflammation where the K^+^ efflux-dependent ASC speck is known to be important. NLRP3-dependent ASC specks are thought to underpin the damaging inflammation that occurs in Alzheimer's disease^16^ and Parkinson's disease.^22^ Although the MPO and BBB scores described above suggest that the NVRs would not easily penetrate the brain, they can form the basis for subsequent molecules.

## Conclusions

In summary, we present a model for how a repurposed drug can be further developed to enhance desirable properties and remove unwanted effects. In doing so we discovered that inhibition of Cl^−^ sensitive pathways provides a way of inhibiting K^+^ efflux-dependent pathways specifically, which may offer therapeutic advantages in certain contexts. The specific target of the NVRs remains unknown although chloride intracellular channel proteins (CLICs 1–6)^23,24^ are known to contribute to NLRP3 inflammasome activation.^25,26^ Future studies will determine whether the CLICs or alternative Cl^−^ channels or transporters are the molecular targets of the NVRs, and will also address the mechanisms through which Cl^−^ regulates NLRP3.

## Conflicts of interest

The authors have no conflicts to declare.

## Author contributions

All authors (TS, JAB, HH, LM, DW, SdC, LES, SY, JG, JBD, CBL, DB, SF) have made substantial contributions to the conception or design of the work, the acquisition, analysis, and interpretation of data. Specifically the synthetic chemistry has been led by JAB, HH, LES, and SF. The computational chemistry has been led by SdC and JBD. The biology has been led by TS, LM, DW, SY, JG, CBL, and DB. All authors contributing to the drafting of the manuscript and approve the final version. The authors are accountable for the accuracy of the work.

## Supplementary Material

SC-011-D0SC03828H-s001

SC-011-D0SC03828H-s002
