# Supplementary material for: Selective inhibition of the K+ efflux sensitive NLRP3 pathway by Cl− channel modulation
Source: Chem Sci. 2020 Oct 12;11(43):11720–8. doi: 10.1039/d0sc03828h (PMC8162947; doi:10.1039/d0sc03828h)
Supplement: SC-011-D0SC03828H-s001 [file SC-011-D0SC03828H-s001.pdf]

Electronic Supplementary Material (ESI) for Chemical Science.
This journal is © The Royal Society of Chemistry 2020

Chemistry Methods, Experimental and Supplementary Information

Selective inhibition of the K^+ efflux sensitive NLRP3 pathway by Cl^- channel modulation

Tessa Swanton*, James A. Beswick*, Halah Hammadi, Lucy Morris, Daniel Williams, Stephane de Cesco, Lina El-Sharkawy, Shi Yu, Jack Green, John B. Davis, Catherine B. Lawrence, David Brough* and Sally Freeman*

Methods

All chemicals, solvents and deuterated solvents were purchased from Sigma-Aldrich, Alfa-Aesar, Fluorochem or Fisher Scientific. ^{1}H, ^{13}C, and ^{19}F NMR spectra were recorded on a Bruker Avance 400 or 300 MHz spectrometer. Chemical shifts (δ) are defined in parts per million (ppm). ^{1}H NMR spectra were referenced to residual undeuterated solvent ($CDCl_3$, δ = 7.27 ppm; DMSO-d_6, δ = 2.50 ppm). ^{13}C NMR spectra were referenced to residual undeuterated solvent ($CDCl_3$, δ = 77.16 ppm, DMSO-d_6 = 39.51) as an internal reference. ^{19}F NMR chemical shifts were referenced using the deuterium lock signal of the solvent. Accurate mass determination was carried out on a Thermo Exactive™ Plus EMR Orbitrap™ LC-MS system. Molecular ion peaks are defined as mass/charge (m/z) ratios. Infrared spectroscopy was recorded on a JASCO FT/IR-4100 spectrophotometer using the Spectra Manager II (JASCO) software package. Analytical thin-layer chromatography (TLC) was performed using silica gel 60 on aluminium sheets coated with F254 indicator. All spots were visualised with $KMnO_4$ or ultraviolet light using a MV Mineralight lamp (254/365) UVGL-58. Flash column chromatography was performed using silica gel with particle size 40-63 µm. Evaporation of solvents was conducted on a Buchi Rotavapor R-200. LC data was obtained using a Waters ACQUITY UPLC PDA detector scanning between 210-400 nm. Mass spectrometry data was acquired using a Waters ACQUITY QDa detector scanning in the positive (ES^+) and negative ($ES^–$) modes between *m/z* 100-1000. Separation of components was achieved using a Waters ACQUITY UPLC BEH C18 1.7 µm 2.1 × 50 mm column coupled to a Waters ACQUTY UPLC BEH C18 1.7 µm VanGuard pre-column 2.1 × 5 mm. Columns were maintained at 40 °C throughout acquisition. The table below details a schematic for the standard four-minute run on the instrument using a flow rate of 0.60 mL min^{-1}:

Start Time / min.	End Time / min.	H_2O : MeCN (%v/v)
0.0	0.5	95 : 5
0.5	2.5	95 : 5 → 5 : 95
2.5	3.0	5 : 95
3.0	3.1	5 : 95 → 95 : 5
3.1	4.0	95 : 5

All solvents obtained were of LC-MS grade (Fisher Optima) and were modified by the addition of 0.1%v/v formic acid (Fisher Optima). Samples were prepared in MeOH (Fisher Optima) and a 2.0 μL aliquot was extracted from each sample for analysis. Samples were maintained at 10 °C prior to acquisition. Solvents were combined using a Waters ACQUITY UPLC H-Class Quaternary Solvent Manager (QSM) with in-built degasser. Inert gas was provided using a Genius NM32LA nitrogen generator.The switch method consisted of 95:5 H_2O:MeCN (with appropriate acidic or basic modifiers) running at 0.60 mL min^{-1} for two minutes immediately prior to acquisition.Data was processed using MassLynx V4.1. Values of purity were obtained through analysis of the peak areas in the LC trace between 0.40 min. and 3.50 min.

With thanks to the School of Chemistry, University of Manchester NMR and Mass Spectrometry facilities, and Dr S Butterworth, Division of Pharmacy and Optometry, for use of the LCMS.

The following compounds were purchased from Sigma – Aldrich Co Limited:

- **2-((3-(Trifluoromethyl)phenyl)amino)nicotinic acid (NFA).**
- **2-((3-Chloro-2-methylphenyl)amino)benzoic acid (TFA).**
- **4-Chloro-2-((furan-2-ylmethyl)amino)-5-sulfamoylbenzoic acid (Furosemide).**
- **1-(2-Bromophenyl)-3-(2-hydroxy-4-nitrophenyl)urea (SB225002).**
- **4-(3-(4-Fluorophenyl)ureido)benzenesulfonamide (U-104).**
- **4-(3-(3,5-Dimethylphenyl)ureido)phenyl sulfamate (S4).**
- **2-Benzylbenzoic acid (NVR-51).**
- **2-Phenoxybenzoic acid (NVR-49).**

The following compounds were purchased from Fisher Scientific UK Ltd:

- **2-(Phenylamino)benzoic acid (NVR-48).**

- **2-((3-(Trifluoromethyl)phenyl)amino)benzoic acid (FFA).**

The following compounds were purchased from Fluorochem Ltd:

- **2-(3-Phenyl-1,2,4-oxadiazol-5-yl)benzoic acid (NVR-61).**
- **[1,1'-Biphenyl]-2-carboxylic acid (NVR-50).**

2-((3-Cyanophenyl)amino)nicotinic acid (NVR-1)

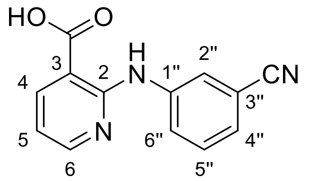

2-Chloronicotinic acid (200.0 mg, 1.3 mmol) and 3-aminobenzonitrile (194.7 mg, 1.7 mmol) were added to deionized water - methanol (1:1) (5 mL). This mixture was stirred for 15 h at 100 °C. The reaction mixture was cooled at room temperature and the precipitate collected by filtration. The residue was collected and stirred with hexane for 30 min. This mixture was filtered and the residue was collected and dissolved in DCM (10 mL). This mixture was filtered and the filtrate was collected and concentrated by evaporation *in vacuo* to yield **2-((3-cyanophenyl)amino)nicotinic acid.** White solid; yield 13% (40 mg, 0.17 mmol); mp: 228 – 232 °C; ^{1}H NMR (500 MHz, [D$_6$]DMSO): δ ppm 13.73 (br s, 1H, COOH), 10.61 (s, 1H, NH), 8.45 (br d, $^{3}J_{H6–H5}$ = 4.4 Hz, 1H, H6), 8.38 (s, 1H, H2″), 8.28 (br d, $^{3}J_{H6''–H5''}$ = 7.8 Hz, 1H, H6″), 7.88 (br d, $^{3}J_{H4–H5}$ = 8.2 Hz, 1H, H4), 7.49 (t, $^{3}J_{H5''–H4''}$ = $^{3}J_{H5''–H6''}$ = 7.9 Hz, 1H, H5″), 7.42 (br d, $^{3}J_{H4''–H5''}$ = 7.6 Hz, 1H, H4″), 6.95 (dd, $^{3}J_{H5–H4}$ = 7.2 Hz, $^{3}J_{H5–H6}$ = 5.2 Hz, 1H, H5); ^{13}C NMR (126 MHz, [D$_6$]DMSO): δ ppm 168.9 (s, COOH), 155.0 (s, C2), 152.5 (s, C6), 140.7 (s, C4), 140.6 (s, C1″), 130.0 (s, C5″), 125.4 (s, C4″), 124.4 (s, C6″), 122.2 (s, C2″), 119.0 (s, CN), 115.0 (s, C5), 111.5 (s, C3″), 108.4 (s, C3); MS (m/z): [M−H, 100%]$^{-}$, 238.1.

2-((3-Chloro-2-methylphenyl)amino)nicotinic acid (Clonixin)

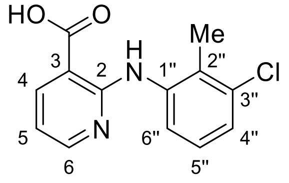

2-Chloronicotinic acid (200.0 mg, 1.26 mmol) and 3-chloro-2-methylaniline (269.6 mg, 1.9 mmol) were added to deionized water-methanol (1:1) (5 mL). This mixture was stirred for 15 h at 100 °C after which it was cooled to room temperature. The precipitate was collected by filtration and stirred with hexane for 30 min. The mixture was filtered and the residue was purified by flash chromatography (0.5% methanol in DCM) to yield **2-((3-chloro-2-methylphenyl)amino)nicotinic acid.** White solid; yield 7% (23.4 mg, 0.0891 mmol); mp: 235

– 238 °C (lit. 234°C)[1] ; ^{1}H NMR (500 MHz, [D$_6$]DMSO): δ ppm 13.63 (br s, 1H, COOH), 10.30 (s, 1H, NH), 8.34 (dd, $^3J_{H6–H5}$ = 4.7 Hz, $^4J_{H6–H4}$ = 2.0 Hz, 1H, H6), 8.26 (dd, $^3J_{H4–H5}$ = 7.7 Hz, $^4J_{H4–H6}$ = 1.9 Hz, 1H, H4), 8.11 (dd, $^3J_{H6''–H5''}$ = 7.9 Hz, $^4J_{H6''–H4''}$ = 0.8 Hz, 1H, H6"), 7.21 (t, $^3J_{H5''–H4''}$ = $^3J_{H5''–H6''}$ = 7.9 Hz, 1H, H5"), 7.16 (dd, $^3J_{H4''–H5''}$ = 7.9 Hz, $^4J_{H4''–H6''}$ = 1.2 Hz, 1H, H4"), 6.87 (dd, $^3J_{H5–H4}$ = 7.8 Hz, $^3J_{H5–H6}$ = 4.7 Hz, 1H, H5), 2.32 (s, 3H, Me); ^{13}C NMR (126 MHz, [D$_6$]DMSO): δ ppm 169.2 (s, COOH), 155.8 (s, C2), 152.7 (s, C6), 140.6 (s, C4), 139.8 (s, C1"), 133.5 (s, C3"), 126.9 (s, C5"), 123.7 (s, C6"), 121.1 (s, C4"), 114.1 (s, C5), 107.8 (s, C3), 14.7 (s, Me), C2" not observed; MS (m/z): [M−H, ^{35}Cl, 100%]$^-$, 261.1, [M−H, ^{37}Cl, 30%]$^-$, 263.0.

5-Nitro-2-((3-(trifluoromethyl)phenyl)amino)benzoic acid (NVR-3)

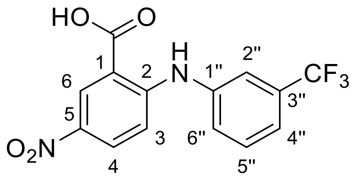

5-Nitro-2-chloro-benzoic acid (200.0 mg, 0.99 mmol) and 3-trifluoromethylaniline (962.0 mg, 6.0 mmol) were added to a microwave reaction sealed vessel (5 mL) equipped with a magnetic stirring bar. This mixture was irradiated in a microwave oven for 10 min at 200 °C, after which the reaction mixture was cooled to room temperature. The organic solution was extracted with NaOH (0.1 M, 3mL). This extraction was repeated 3 times until the aqueous layer became faint yellow. This layer was collected and acidified using concentrated HCl (38% v/v) until the desired compound precipitated. The yellow precipitate was collected by filtration and washed twice with water (2 × 10 mL) in order to remove the remaining sodium chloride and hydrochloric acid. The product was dried in an oven at 100 °C to yield **5-nitro-2-((3-(trifluoromethyl)phenyl)amino)benzoic acid.** Yellow solid; yield 6% (20 mg, 0.061 mmol); mp: 200 – 204 °C; ^{1}H NMR (300 MHz, CDCl$_3$): δ ppm 9.72 (s, 1H, NH), 8.35 (s, 1H, H6), 7.41 – 7.64 (m, 5H, H2", H4", H5", H6", H4), 7.23 (d, $^3J_{H3–H4}$ = 8.9 Hz, 1H, H3), COOH not observed; ^{13}C NMR (75 MHz, [D$_6$]DMSO): δ ppm 168.3 (s, COOH), 151.6 (s, C2), 139.5 (s, C1"), 137.4 (s, C5), 130.9 (s, C4), 129.4 (s, C6), 128.3 (s, C5"), 127.6 (s, C6"), 121.9 (q,$^3J_{C-F}$ = 4.4 Hz, C2"), 120.4 ($^3J_{C-F}$ = 4.4 Hz, C4"), 113.7 (s, C1), 112.1 (s, C3), CF$_3$, C3" not observed; MS (m/z): [M−H, 100%]$^-$, 325.0; HRMS (m/z): [M−H]$^-$ calcd. for $C_{14}H_8O_4N_2F_3$, 325.0431; found 325.0433; error, 0.71 ppm.

5-(Trifluoromethyl)-2-((3-(trifluoromethyl)phenyl)amino)benzoic acid (NVR-4)

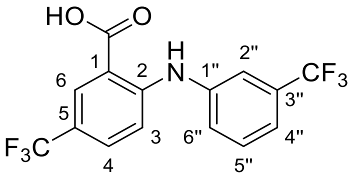

5-Trifluoromethyl-2-chloro-benzoic acid (165.0 mg, 0.735 mmol), 3-trifluoromethylaniline (189.4 mg, 1.175 mmol), Copper powder (3.9 mg, 0.0615 mmol), Copper (II) oxide (3.91 mg, 0.0275 mmol), sodium carbonate (100 mg, 80 mmol) and DMF (3 mL) were added to a microwave reaction sealed vessel (5 mL) equipped with a magnetic stirring bar. This mixture was irradiated in a microwave oven for 10 min at 200 °C, after which the reaction mixture was cooled to room temperature. The organic solution was extracted with NaOH (0.1 M, 3 × 3 mL) when the aqueous layer became faint yellow. This layer was collected and acidified using concentrated HCl (38% v/v) until the desired compound precipitated. The white precipitate was collected by filtration and washed with water (2 × 10 mL) in order to remove the remaining sodium chloride and hydrochloric acid. The product was dried in an oven at 100 °C to yield **5-(trifluoromethyl)-2-((3-(trifluoromethyl)phenyl)amino)benzoic acid.** White solid; yield 21% (55 mg, 0.16 mmol); mp: 210 – 212 °C; ^{1}H NMR (500 MHz, [D$_6$]DMSO): δ ppm 13.67 (br s, 1H, COOH), 10.03 (s, 1H, NH), 8.16 (d, $^{4}J_{H6\text{–}H4}$ = 2.0 Hz, 1H, H6), 7.71 (dd, $^{3}J_{H4\text{–}H3}$ = 9.0, $^{4}J_{H4\text{–}H6}$ = 2.3 Hz, 1H, H4), 7.59 – 7.67 (m, 3H, H2″, H5″, H6″), 7.50 (d, $^{3}J_{H4''\text{–}H5''}$ = 6.9 Hz, 1H, H4″), 7.31 (d, $^{3}J_{H3\text{–}H4}$ = 8.9 Hz, 1H, H3); ^{13}C NMR (126 MHz, [D$_6$]DMSO): δ ppm 168.7 (s, COOH), 149.2 (s, C2), 140.4 (s, C1″), 130.7 (s, C5″), 130.7 (q, $^{3}J_{C\text{-}F}$ = 4.7 Hz, C4), 130.4 (q, $^{2}J_{C\text{-}F}$ = 31.8 Hz, C3″), 128.9 (q, $^{3}J_{C\text{-}F}$ = 4.2 Hz, C6), 126.2 (s, C6″), 124.3 (q, $^{1}J_{C\text{-}F}$ = 271.0 Hz, CF$_3$), 123.9 (q, $^{1}J_{C\text{-}F}$ = 272.4 Hz, CF$_3$), 120.6 (br q, $^{3}J_{C\text{-}F}$ = 3.9 Hz, C2″), 119.0 (q, $^{3}J_{C\text{-}F}$ = 4.4 Hz, C4″), 117.9 (q, $^{2}J_{C\text{-}F}$ = 31.6 Hz, C5), 114.4 (s, C3), 112.9 (s, C1); ^{19}F NMR (471 MHz, [D$_6$]DMSO): δ ppm −60.24 (s, CF$_3$), −61.23 (s, CF$_3$); MS (m/z): [M−H, 100%]$^-$, 348.1; HRMS (m/z): [M−H]$^-$ calcd. for $C_{15}H_8O_2NF_6$, 348.0465; found 348.0464; error, −0.20 ppm.

2-Benzamidobenzoic acid (NVR-17)

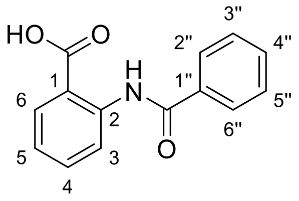

To a stirred solution of anthranilic acid (100 mg, 0.729 mmol, 1 eq) and benzoyl chloride (0.1 mL, 121 mg, 0.861 mmol, 1.2 eq) in DCM (5 mL) was added a 10% solution of NaOH (1 mL). The reaction mixture was stirred for 3h at room temperature. Solvent was removed *in vacuo* and the white residue was taken up in EtOAc (5 mL) before the product was extracted with 2 × 5 mL 0.1M NaOH. The combined aqueous layers were acidified by dropwise addition of concentrated HCl, and the resultant precipitate was isolated by filtration and dried to yield **2-benzamidobenzoic acid.** White solid; yield 59% (104 mg, 0.431 mmol); mp: 179 – 180 °C (lit 179 – 181 °C)[2] ^{1}H NMR (400 MHz, [D$_6$]DMSO): δ ppm 13.80 (br s, 1H, COOH), 12.18 (s, 1H, CONH), 8.72 (d, $^{3}J_{H3\text{-}H4}$ = 8.0 Hz, 1H, H3), 8.06 (dd, $^{3}J_{H6\text{-}H5}$ = 8.0 Hz, $^{4}J_{H6\text{-}H4}$ = 1.5 Hz, 1H, H6), 7.96 (d, $^{3}J_{H2''/H6''\text{-}H3''/H5''}$ = 7.0 Hz, 2H, H2″ + H6″), 7.54 - 7.73 (m, 4H, H4 + H3″ + H4″ + H5″), 7.22 (ddd, $^{3}J_{H5\text{-}H6}$ = 8.2 Hz, $^{3}J_{H5\text{-}H4}$ = 6.9 Hz, $^{4}J_{H5\text{-}H3}$ = 1.0 Hz, 1H, H5); ^{13}C NMR (101 MHz, [D$_6$]DMSO): δ ppm 170.0 (s, COOH), 164.7 (s, CONH), 141.1 (s, C1″), 134.5 (s, C2), 134.3 (s, C6), 132.2 (s, C5), 131.3 (s, C4), 129.0 (s, C3″ + C5″), 127.0 (s, C2″+ C6″), 123.0 (s, C4″), 119.9 (s, C3), 116.6 (s, C1); NMR assignments made with the assistance of COSY, HSQC and HMBC; MS (m/z): [M−H, 100%]$^{-}$, 240.1.

***N*-(2-(1*H*-Tetrazol-5-yl)phenyl)benzamide (NVR-15)**

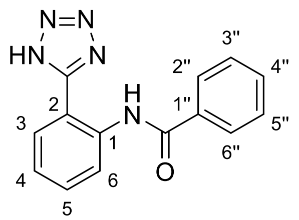

Benzoyl chloride (104.2 mg, 0.744 mmol) was added dropwise to a solution of 2-(1*H*-tetrazol-5-yl)-aniline (100.0 mg, 0.620 mmol) and DIPEA (136.3 mg, 1.0 mmol) in anhydrous DCM (3 mL) at 0 °C under N_2. This mixture was stirred at 0 °C for 3 h then at room temperature overnight. The reaction mixture was concentrated and extracted with EtOAc (2x 10 mL) and

NaOH (0.1 M, 3 mL). The aqueous layer was acidified with 1M HCl, the precipitate was filtered and washed with water to yield ***N*-(2-(1*H*-tetrazol-5-yl)phenyl)benzamide.** White solid; yield 39% (62 mg, 0.234 mmol); mp: 150 °C, (lit 226 – 227 °C)[3]; ^{1}H NMR (500 MHz, [D$_6$]DMSO): δ ppm 11.54 (s, 1H, CONH), 8.59 (dd, $^{3}J_{H6–H5}$ = 8.4 Hz, $^{4}J_{H6–H4}$ = 0.8 Hz, 1H, H6), 8.02 – 8.08 (m, 3H, H3 + H2″ + H6″), 7.57 – 7.68 (m, 4H, H5 + H3″ + H4″ + H5″), 7.37 (td, $^{3}J_{H4–H3}$ = $^{3}J_{H4–H5}$ = 7.6 Hz, $^{4}J_{H4–H6}$ = 1.1 Hz, 1H, H4), tetrazole NH not observed; ^{13}C NMR (126 MHz, [D$_6$]DMSO): δ ppm 165.1 (s, CONH), 154.6 (br s, Tet-C), 137.2 (s, C1), 134.4 (s, C1″), 132.2 (s, C4″), 131.9 (s, C5), 128.9 (s, C3″ + C5″), 128.7 (s, C4), 127.3 (s, C2″ + C6″), 124.2 (s, C3), 121.9 (s, C6), 113.4 (s, C2); MS (m/z): [M−H, 100%]$^-$, 264.1.

***N*-(2-(1*H*-Tetrazol-5-yl)phenyl)-4-methoxybenzamide (NVR-29)**

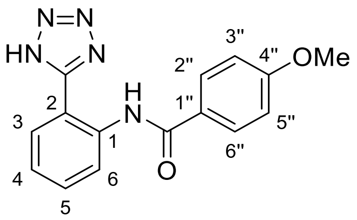

4-Methoxybenzoyl chloride (152.0 mg, 0.89 mmol) was added dropwise to a solution of 2-(1*H*-tetrazol-5-yl)-aniline (120.0 mg, 0.74 mmol) and DIPEA (162.6 mg, 1.26 mmol) in anhydrous DCM (3 mL) at 0 °C under N_2. This mixture was stirred at 0 °C for 3 h then at room temperature overnight. The reaction mixture was concentrated and extracted with EtOAc (2 × 5 mL), NaOH (0.1 M, 3 mL). The aqueous layer was acidified with 1M HCl; the precipitate was filtered and washed with water to yield ***N*-(2-(1*H*-tetrazol-5-yl)phenyl)-4-methoxybenzamide.** White solid; yield 18% (40 mg, 0.14 mmol); mp: 122 – 124 °C (lit 181 – 183 °C)[3]; ^{1}H NMR (500 MHz, [D$_6$]DMSO): δ ppm 11.50 (s, 1H, CONH), 8.60 (dd, $^{3}J_{H6–H5}$ = 8.4 Hz, $^{3}J_{H6–H4}$ = 0.8 Hz, 1H, H6), 8.08 (dd, $^{3}J_{H3–H4}$ = 7.9 Hz, $^{3}J_{H3–H5}$ = 1.4 Hz, 1H, H3), 8.02 (br d, $^{3}J_{H2″/H6″–H3″/H5″}$ = 9.2 Hz, 2H, H2″ + H6″), 7.60 (ddd, $^{3}J_{H5–H6}$ = 8.4 Hz, $^{3}J_{H5–H4}$ = 7.4 Hz, $^{3}J_{H5–H3}$ = 1.4 Hz, 1H, H5), 7.32 (td, $^{3}J_{H4–H3}$ = $^{3}J_{H4–H5}$ = 7.6 Hz, $^{3}J_{H4–H6}$ = 1.1 Hz, 1H, H4), 7.12 (br d, $^{3}J_{H3″/H5″–H2″/H6″}$ = 8.9 Hz, 2H, H3″ + H5″), 3.85 (s, 3H, OMe), tetrazole NH not observed; ^{13}C NMR (126 MHz, [D$_6$]DMSO): δ ppm 164.6 (s, CONH), 162.4 (s, C4″), 154.6 (br s, Tet-C), 137.5 (s, C1), 132.0 (s, C5), 129.3 (s, C2″ + C6″), 128.8 (s, C3), 126.5 (s, C1″), 123.9 (s, C4), 121.7 (s, C6), 114.2 (s, C3″ + C5″), 112.9 (s, C2), 55.6 (s, OMe); MS (m/z): [M−H, 100%]$^-$, 294.0; HRMS (m/z): [M−H]$^-$ calcd. for $C_{15}H_{12}O_2N_5$, 294.0996; found 294.0995; error, −0.50 ppm.

N-(5-Chloro-2-(1H-tetrazol-5-yl)phenyl)benzamide (NVR-16)

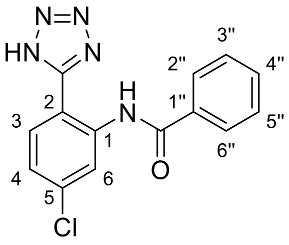

Benzoyl chloride (85.9 mg, 0.613 mmol) was added dropwise to a solution of 5-chloro-2-(1*H*-tetrazol-5-yl)-aniline (100.0 mg, 0.511 mmol) and DIPEA (112.0 mg, 0.868 mmol) in anhydrous DCM (3 mL) at 0 °C under N_2. This mixture was stirred at 0 °C for 3 h then at room temperature overnight. The reaction mixture was concentrated and extracted with EtOAc (2 × 5 mL) and NaOH (0.1 M, 3 mL). The aqueous layer was acidified with 1M HCl, the precipitate was filtered and washed with water to yield ***N*-(5-chloro-2-(1*H*-tetrazol-5-yl)phenyl)benzamide.** White solid; yield 30% (45.7 mg, 0.153 mmol); mp: 165 – 168 °C; ^{1}H NMR (500 MHz, [D$_6$]DMSO): δ ppm 11.75 (s , 1H, COOH), 8.74 (d, $^4J_{H6–H4}$ = 2.1 Hz, 1H, H6), 8.09 (d, $^3J_{H3–H4}$ = 8.4 Hz, 1H, H3), 8.06 (br d, $^3J_{H2''/H6''–H3''/H5''}$ = 7.0 Hz, 2H, H2″ + H6″), 7.68 (br t, $^3J_{H4''–H3''}$ = $^3J_{H4''–H5''}$ = 7.0 Hz, 1H, H4″), 7.62 (br t, $^3J_{H3''/H5''–H2''/H6''}$ = $^3J_{H3''/H5''–H4''}$ = 6.9 Hz, 2H, H3″ + H5″), 7.46 (dd, $^3J_{H4–H3}$ = 8.4 Hz, $^4J_{H4–H6}$ = 2.1 Hz, 1H, H4), tetrazole NH not observed; ^{13}C NMR (126 MHz, [D$_6$]DMSO): δ ppm 165.3 (s, CONH), 154.4 (s, Tet-C), 138.3 (s, C1), 136.2 (s, C1″), 134.0 (s, C5), 132.5 (s, C4″), 130.2 (s, C3), 129.0 (s, C3″ + C5″), 127.4 (s, C2″ + C6″), 124.0 (s, C4), 120.9 (s, C6), 112.2 (s, C2); MS (m/z): [M−H, ^{35}Cl, 100%]$^-$, 298.1, [M−H, ^{37}Cl, 90%]$^-$, 300.1.

5-Iodo-2-(3-(4-methoxyphenyl)propanamido)benzoic acid (NVR-31)

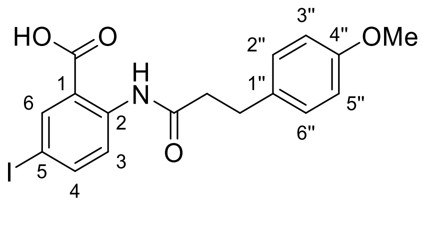

3-(4-Methoxyphenyl)propanoyl chloride (162.1 mg, 0.82 mmol) was added dropwise to a solution of 2-amino-5-iodobenzoic acid (180.0 mg, 0.68 mmol) and DIPEA (150.0 mg, 1.2 mmol) in anhydrous DCM (5 mL) at 0 °C under N_2. This mixture was stirred at 0 °C for 3 h then at room temperature overnight. The reaction mixture was concentrated and extracted with EtOAc (2 × 10 mL), NaOH (0.1 M, 5mL). The aqueous layer was acidified with 1M HCl, the

precipitate was filtered and washed with water to yield **5-iodo-2-(3-(4-methoxyphenyl)propanamido)benzoic acid.** White solid; yield 7% (20.4 mg, 0.0480 mmol); mp: 228 – 230 °C; ^{1}H NMR (500 MHz, [D$_6$]DMSO): δ ppm 11.02 (s, **1H**, CONH), 8.28 (d, $^{3}J_{H3-H4}$ = 8.9 Hz, 1H, H3), 8.20 (d, $^{4}J_{H6-H4}$ = 2.3 Hz, 1H, H6), 7.88 (dd, $^{3}J_{H4-H3}$ = 8.8 Hz, $^{4}J_{H4-H6}$ = 2.2 Hz, 1H, H4), 7.15 (br d, $^{3}J_{H2''/H6''-H3''/H5''}$ = 8.5 Hz, 2H, H2″ + H6″), 6.82 (br d, $^{3}J_{H3''/H5''-H2''/H6''}$ = 8.5 Hz, 2H, H3″ + H5″), 3.70 (s, 3H, OMe), 2.86 (t, $^{3}J_{HH}$ =7.6 Hz, 2H, CH$_2$Ph), 2.66 (t, $^{3}J_{HH}$ =7.6 Hz, 2H, COCH$_2$), COOH not observed; ^{13}C NMR (126 MHz, [D$_6$]DMSO): δ ppm 170.7 (s, CONH), 168.1 (s, COOH), 157.6 (s, C4″), 142.2 (s, C4), 140.3 (s, C2), 138.9 (s, C6), 132.5 (s, C1″), 129.2 (s, C2″ + C6″), 122.2 (s, C3), 118.7 (s, C1), 113.7 (s, C3″ + C5″), 85.7 (s, C5), 55.0 (s, OMe), 29.6 (s, CH$_2$Ar), CH$_2$CO obscured by DMSO peak; MS (m/z): [M−H, 100%]$^-$, 424.1; HRMS (m/z): [M−H]$^-$ calcd. for $C_{17}H_{15}O_4NI$, 424.0051; found 424.0052; error, 0.81 ppm.

***N*-(2-(1*H*-Tetrazol-5-yl)phenyl)cinnamamide (NVR-23)**

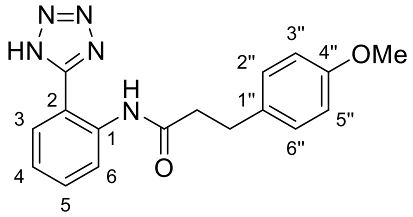

3-(4-Methoxyphenyl)propanoyl chloride (177.0 mg, 0.89 mmol) was added dropwise to a solution of 2-(1*H*-tetrazol-5-yl)-aniline (120 mg, 0.74 mmol) and DIPEA (163.3 mg, 1.26 mmol) in anhydrous DCM (3 mL) at 0 °C under N_2. This mixture was stirred at 0 °C for 3 h then at room temperature overnight. The reaction mixture was concentrated and extracted with EtOAc (2 × 5 mL) and NaOH (0.1 M, 3 mL). The aqueous layer was acidified with 1M HCl, the precipitate was filtered and washed with water to yield ***N*-(2-(1*H*-tetrazol-5-yl)phenyl)cinnamamide.** White solid; yield 14% (34.1 mg, 0.106 mmol); mp: 182 – 185 °C; ^{1}H NMR (500 MHz, [D$_6$]DMSO): δ ppm 10.55 (s , 1H, CONH) , 8.27 (d, $^{3}J_{H6-H5}$ = 8.2 Hz, 1H, H6), 7.89 (dd, $^{3}J_{H3-H4}$ = 7.9 Hz, $^{4}J_{H3-H5}$ = 1.2 Hz, 1H, H3), 7.56 (br t, $^{3}J_{H5-H4}$ = $^{3}J_{H5-H6}$ = 8.2 Hz, 1H, H5), 7.30 (br t, $^{3}J_{H4-H3}$ = $^{3}J_{H4-H5}$ = 7.6 Hz, 1H, H4), 7.16 (d, $^{3}J_{H2''/H6''-H3''/H5''}$ = 8.5 Hz, 2H, H2″ + H6″), 6.81 (br d, $^{3}J_{H3''/H5''-H2''/H6''}$ = 8.5 Hz, 2H, H3″ + H5″), 3.69 (s, 3H, OMe), 2.88 (t, $^{3}J_{HH}$ = 7.6 Hz, 2H, CH$_2$Ar), 2.67 (t, $^{3}J_{HH}$ = 7.6 Hz, 2H, COCH$_2$), tetrazole NH not observed; ^{13}C NMR (126 MHz, [D$_6$]DMSO): δ ppm 170.7 (s, COOH), 157.5 (s, C4″), 154.3 (br s, Tet-C), 137.0 (s, C1), 132.6 (s, C1″), 131.7 (s, C6), 129.2 (s, C2″ + C6″), 128.9 (s, C3), 124.0 (s, C4), 122.2 (s, C5), 113.7 (s, C3″

+ C5″), 54.9 (s, OMe), 29.8 (s, CH_2Ar), C2 not observed, CH_2CO obscured by DMSO peak; MS (m/z): [M−H, 100%]$^-$, 322.2; HRMS (m/z): [M−H]$^-$ calcd. for $C_{17}H_{16}O_2N_5$, 322.1309; found 322.1305; error, –1.39 ppm.

_N_-(5-Chloro-2-(1_H_-tetrazol-5-yl)phenyl)-3-(4-methoxyphenyl)propanamide (NVR-21)

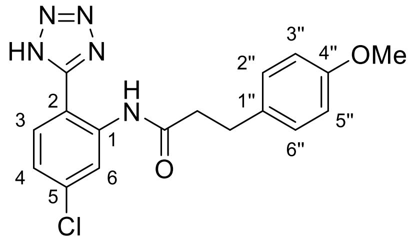

3-(4-Methoxyphenyl)propanoyl chloride (146.2 mg, 0.740 mmol) was added dropwise to a solution of 5-chloro-2-(1*H*-tetrazol-5-yl)-aniline (120.0 mg, 0.610 mmol) and DIPEA (134.0 mg, 1.04 mmol) in anhydrous DCM (3 mL) at 0 °C under N_2. This mixture was stirred at 0 °C for 3 h then at room temperature overnight. The reaction mixture was concentrated and extracted with EtOAc (2 × 5 mL) and NaOH (0.1 M, 3 mL). The aqueous layer was acidified with 1M HCl, the precipitate was filtered and washed with water to yield **_N_-(5-chloro-2-(1_H_-tetrazol-5-yl)phenyl)-3-(4-methoxyphenyl)propanamide.** White solid; yield 15% (32.7 mg, 0.0914 mmol); mp: 228 – 230 °C; ^{1}H NMR (500 MHz, [D$_6$]DMSO): δ ppm 10.83 (s, 1H, CONH), 8.44 (d, $^4J_{H6–H4}$ = 2.1 Hz, 1H, H6), 8.00 (d, $^3J_{H3–H4}$ = 8.4 Hz, 1H, H3), 7.39 (dd, $^3J_{H4–H3}$ = 8.5 Hz, $^4J_{H4–H6}$ = 2.2 Hz, 1H, H4), 7.16 (d, $^3J_{H2''/H6''–H3''/H5''}$ = 8.5 Hz, 2H, H2″ + H6″), 6.82 (br d, $^3J_{H3''/H5''–H2''/H6''}$ = 8.7 Hz, 2H, H3″+ H5″), 3.69 (s, 3H, OMe), 2.89 (t, $^3J_{HH}$ = 7.5 Hz, 2H, CH_2Ar), 2.71 (t, $^3J_{HH}$ = 7.5 Hz, 2H, $COCH_2$), tetrazole NH not observed; ^{13}C NMR (126 MHz, [D$_6$]DMSO): δ ppm 171.1 (s, CONH), 157.6 (s, Tet-C), 138.0 (s, C1), 135.8 (s, C5), 132.5 (s, C1″), 130.4 (s, C3), 129.2 (s, C2″ + C6″), 123.7 (s, C4), 121.0 (s, C6), 113.7 (s, C3″ + C5″), 54.9 (s, OMe), 29.6 (s, CH_2Ar), CH_2CO obscured by DMSO peak; MS (m/z): [M−H, ^{35}Cl, 100%]$^-$, 356.2, [M−H, ^{37}Cl, 30%]$^-$, 358.2; HRMS (m/z): [M−H]$^-$ calcd. for $C_{17}H_{15}O_2N_5{}^{35}Cl$, 356.0918; found 356.0918; error, –0.49 ppm.

N-(4-Bromo-2-(1H-tetrazol-5-yl)phenyl)-3-(4-methoxyphenyl)propanamide (NVR-30)

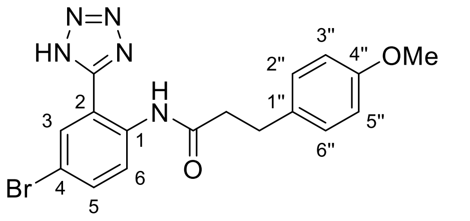

3-(4-Methoxyphenyl)propanoyl chloride (119.2 mg, 0.60 mmol) was added dropwise to a solution of 4-bromo-2-(1*H*-tetrazol-5-yl)aniline (120.0 mg, 0.50 mmol) and DIPEA (109.9 mg, 0.85 mmol) in anhydrous DCM (3 mL) at 0 °C under N_2. The mixture was stirred at 0° C for 3 h then at room temperature overnight. The reaction mixture was concentrated and extracted with EtOAc (2 × 5 mL) and NaOH (0.1 M, 3 mL). The aqueous layer was acidified with 1M HCl, the precipitate was filtered and washed with water to yield ***N*-(4-bromo-2-(1*H*-tetrazol-5-yl)phenyl)-3-(4-methoxyphenyl)propanamide.** Cream solid; yield 9% (17.8 mg, 0.0443 mmol); mp: 170 – 172 °C; ^{1}H NMR (500 MHz, [D$_6$]DMSO): δ ppm 11.01 (s, 1H, C6), 8.67 (d, $^3J_{H6–H5}$ = 9.0 Hz, 1H, H6), 8.56 (d, $^4J_{H3–H5}$ = 2.3 Hz, 1H, H3), 8.16 (dd, $^3J_{H5–H6}$ = 8.9 Hz, $^4J_{H5–H3}$ = 2.4 Hz, 1H, H5), 7.57 (br d, $^3J_{H2''/H6''–H3''/H5''}$ = 8.7 Hz, 2H, H2″ + H6″), 7.23 (br d, $^3J_{H3''/H5''–H2''/H6''}$ = 8.7 Hz, 2H, H3″ + H5″), 4.11 (s, 3H, OMe), 3.29 (t, $^3J_{HH}$ = 7.6 Hz, 2H, CH_2Ph), 3.10 (t, $^3J_{HH}$ = 7.8 Hz, 2H, $COCH_2$), tetrazole NH not observed; ^{13}C NMR (126 MHz, [D$_6$]DMSO): δ ppm 170.8 (s, CONH), 157.6 (s, C4″), 153.5 (br s, Tet-C), 136.2 (s, C1), 134.2 (s, C3), 132.5 (s, C1″), 131.1 (s, C5), 129.2 (s, C2″ + C6″), 124.0 (s, C6), 118.0 (s, C4), 115.4 (s, C2), 113.7 (s, C3″ + C5″), 54.9 (s, OMe), 30.7 (s, CH_2Ar), CH_2CO obscured by DMSO peak; MS (m/z): [M−H, ^{79}Br, 90%]$^-$, 400.1, [M−H, ^{81}Br, 100%]$^-$, 402.1; HRMS (m/z): [M−H]$^-$ calcd. for $C_{17}H_{15}O_2N_5{}^{79}Br$, 400.0415; found 400.0414; error, –0.15 ppm.

N-(2-(1H-Tetrazol-5-yl)phenyl)cinnamamide (NVR-25)

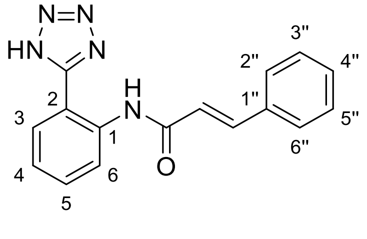

Cinnamoyl chloride (148.0 mg, 0.891 mmol) was added dropwise to a solution of 2-(1*H*-tetrazol-5-yl)-aniline (120.0 mg, 0.74 mmol) and DIPEA (163.0 mg, 1.3 mmol) in anhydrous

DCM (3 mL) at 0 °C under N_2. This mixture was stirred at 0 °C for 3 h then at room temperature overnight. The reaction mixture was concentrated and extracted with EtOAc (2 × 5 mL), HCl (0.1 M, 5 mL). The product in the organic layer was extracted with 0.1 M NaOH (5 mL). The aqueous layer was acidified with 1M HCl; the precipitate was filtered and washed with water to yield ***N*-(2-(1*H*-tetrazol-5-yl)phenyl)cinnamamide.** White solid; yield 15% (32.2 mg, 0.111 mmol); mp: 242 °C; ^{1}H NMR (500 MHz, [D$_6$]DMSO): δ ppm 10.92 (s, 1H, CONH), 8.41 (d, $^3J_{H6–H5}$ = 8.1 Hz, 1H, H6), 8.01 (d, $^3J_{H3–H4}$ = 7.8 Hz, 1H, H3), 7.70 (dd, $^3J_{H2''/H6''–H3''/H5''}$ = 7.9 Hz, $^4J_{H2''/H6''–H4''}$ = 1.3 Hz, 2H, H2″ + H6″), 7.64 (d, $^3J_{HH}$ = 15.7 Hz, 1H, CHPh), 7.59 (ddd, $^3J_{H5–H6}$ = 8.4 Hz, $^3J_{H5–H4}$ = 7.5 Hz, $^4J_{H5–H3}$ = 1.4 Hz, 1H, H5), 7.39 – 7.48 (m, 3H, H3″ + H4″ + H5″), 7.33 (ddd, $^3J_{H4–H3}$ = 7.9 Hz, $^3J_{H4–H5}$ = 7.0 Hz, $^4J_{H4–H6}$ = 0.9 Hz, 1H, H4), 6.89 (d, $^3J_{HH}$ = 15.7 Hz, 1H, COCH), tetrazole NH not observed; ^{13}C NMR (126 MHz, [D$_6$]DMSO): δ ppm 163.9 (s, CONH), 154.5 (s, Tet-C), 141.0 (s, CHPh), 137.0 (s, C1), 134.4 (s, C1″), 131.5 (s, C5), 130.0 (s, C4″), 129.1 (s, C3), 129.0 (s, C3″ + C5″), 128.0 (s, C2″ + C6″), 124.2 (s, C4), 122.5 (s, CHCO), 122.2 (s, C6), 114.3 (s, C2); MS (m/z): [M−H, 100%]$^-$, 290.2. HRMS (m/z): [M−H]$^-$ calcd. for $C_{16}H_{12}ON_5$, 290.1047; found 290.1045; error, –0.80 ppm.

(*E*)-*N*-(2-(1*H*-Tetrazol-5-yl)phenyl)-3-(4-methoxyphenyl)acrylamide (NVR-38)

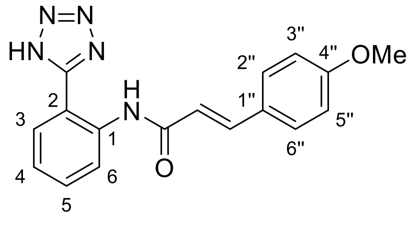

(*E*)-3-(4-Methoxyphenyl)acryloyl chloride (175.8 mg, 0.894 mmol) was added dropwise to a solution of 2-(1*H*-tetrazol-5-yl)-aniline (120.0 mg, 0.74 mmol) and DIPEA (163.5 mg, 1.26 mmol) in anhydrous DCM (5 mL) at 0 °C under N_2. This mixture was stirred at 0 °C for 3 h then at room temperature overnight. The reaction mixture was concentrated and extracted with EtOAc (2 x 5 mL) and HCl (0.1 M, 5 mL). The product in the organic layer was extracted with 0.1 M NaOH (5 mL). The aqueous layer was acidified with 1M HCl; the precipitate was filtered and washed with water to yield **(*E*)-*N*-(2-(1*H*-tetrazol-5-yl)phenyl)-3-(4-methoxyphenyl)acrylamide.** Yellow solid; yield 10% (24 mg, 0.075 mmol); mp: 162 – 166 °C; ^{1}H NMR (400 MHz, [D$_6$]DMSO): δ ppm 10.90 (br s, 1H, CONH), 8.42 (br d, $^3J_{H6–H5}$ = 8.3 Hz, 1H, H6), 7.96 (br d, $^3J_{H3–H4}$ = 7.3 Hz, 1H, H3), 7.66 (br d, $^3J_{H2''/H6''–H3''/H5''}$ = 8.4 Hz, 2H, H2″ + H6″),

7.50 – 7.63 (m, 2H, H5 + CHAr), 7.31 (br t, $^3J_{H4–H3}$ = $^3J_{H4–H5}$ = 7.4 Hz, 1H, H4), 7.01 (br d, $^3J_{H3''/H5''–H2''/H6''}$ = 8.4 Hz, 2H, H3″ + H5″), 6.72 (br d, $^3J_{HC=CH}$ = 15.6 Hz, 1H, COCH), 3.81 (s, 3H, OMe), tetrazole NH not observed; ^{13}C NMR (101 MHz, [D$_6$]DMSO): δ ppm 164.1 (s, CONH), 160.8 (s, C4″), 140.9 (s, CHAr), 137.1 (s, C1), 131.3 (s, C5), 129.7 (s, C2″ + C6″), 128.9 (s, C3), 127.0 (s, C1″), 124.0 (s, C4), 122.2 (s, CHCO), 119.5 (s, C6), 114.4 (s, C3″ + C5″), 55.3 (s, OMe), C2 + tetrazole carbon not observed; HRMS (m/z): [M−H]$^-$ calcd. for $C_{17}H_{14}O_2N_5$, 320.1153; found 320.1146; error, −2.18 ppm.

(*E*)-*N*-(2-(1*H*-Tetrazol-5-yl)phenyl)-3-(3,4-dimethoxyphenyl)acrylamide (NVR-27)

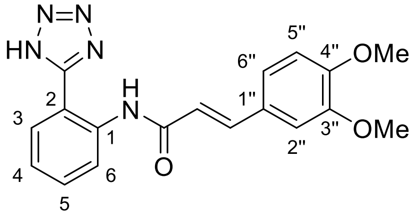

(*E*)-3-(3-4-Dimethoxyphenyl)acryloyl chloride (252.9 mg, 1.1 mmol) was added dropwise to a solution of 2-(1*H*-tetrazol-5-yl)-aniline (150.0 mg, 0.93 mmol) and DIPEA (204.4 mg, 1.5 mmol) in anhydrous DCM (3 mL) at 0 °C under N_2. This mixture was stirred at 0 °C for 3 h then at room temperature overnight. The reaction mixture was concentrated and extracted with EtOAc (2 × 5 mL), HCl (0.1 M, 5 mL). The product in the organic layer was extracted with 0.1 M NaOH (5 mL). The aqueous layer was acidified with 1M HCl, the precipitate was filtered and washed with water to yield (*E*)-*N*-(2-(1*H*-tetrazol-5-yl)phenyl)-3-(3,4-dimethoxyphenyl)acrylamide**.** White solid; yield 6% (18 mg, 0.051 mmol); mp: 190 – 192 °C; ^{1}H NMR (300 MHz, [D$_6$]DMSO): δ ppm 10.76 (s , CONH) , 8.41 (d, $^3J_{H6–H5}$ = 8.2 Hz, 1H, H6), 8.03 (dd, $^3J_{H3–H4}$ = 7.9 Hz, $^4J_{H3–H5}$ = 0.9 Hz, 1H, H3), 7.52 – 7.64 (m, 2H, H5 + CHAr), 7.29 – 7.38 (m, 2H, H4 + H2″), 7.25 (br d, $^3J_{H6''–H5''}$ =8.3 Hz, 1H, H6″), 7.01 (d, $^3J_{H5''–H6''}$ = 8.3 Hz, 1H, H5″), 6.79 (d, $^3J_{HC=CH}$ = 15.6 Hz, 1H, COCH), 3.84 (s, 3H, OMe), 3.80 (s, 3H, OMe), tetrazole NH not observed; ^{13}C NMR (126 MHz, [D$_6$]DMSO): δ ppm 164.3 (s, CONH), 154.2 (br s, Tet-C), 150.7 (s, C3″), 149.0 (s, C4″), 141.4 (s, CHAr), 137.2 (s, C1), 131.7 (s, C5), 129.3 (s, C1″), 127.3 (s, C3), 124.1 (s, C4), 122.5 (s, C6″), 122.4 (s, C6), 119.7 (s, CHCO), 113.9 (s, C2), 111.7 (s, C5″), 110.4 (s, 2″), 55.65 (s, OMe), 55.62 (s, OMe); MS (m/z): [M−H, 100%]$^-$, 350.3.

(*E*)-*N*-(4-Bromo-2-(1*H*-tetrazol-5-yl)phenyl)-3-(3,4-dimethoxyphenyl)acrylamide (NVR-32)

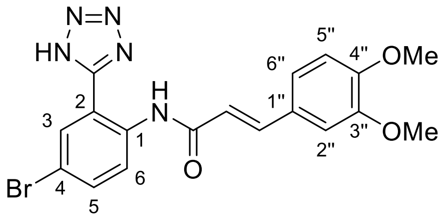

(*E*)-3-(3-4-Dimethoxyphenyl)acryloyl chloride (136.0 mg, 0.60 mmol) was added dropwise to a solution of 4-bromo-2-(1*H*-tetrazol-5-yl)aniline (120.0 mg, 0.50 mmol) and DIPEA (109.9 mg, 0.843 mmol) in anhydrous DCM (3 mL) at 0 °C under N_2. This mixture was stirred at 0 °C for 3 h then at room temperature overnight. The reaction mixture was concentrated and extracted with EtOAc (2 × 5 mL), HCl (0.1 M, 5 mL). The product in the organic layer was extracted with 0.1 M NaOH (5 mL). The aqueous layer was acidified with 1M HCl, the precipitate was filtered and washed with water to yield **(*E*)-*N*-(4-bromo-2-(1*H*-tetrazol-5-yl)phenyl)-3-(3,4-dimethoxyphenyl)acrylamide.** Yellow solid; yield 9% (19.3 mg, 0.0449 mmol); mp: 232 – 235 °C; ^{1}H NMR (500 MHz, [D$_6$]DMSO): δ ppm 10.81 (s, 1H, CONH) , 8.38 (d, $^{3}J_{H6–H5}$ = 8.9 Hz, 1H, H6), 8.17 (s, 1H, H3), 7.76 (br d, $^{3}J_{H5–H6}$ = 9.0 Hz, 1H, H5), 7.58 (d, $^{3}J_{HC=CH}$ = 15.6 Hz, 1H, CHAr), 7.31 (s, 1H, H2”), 7.24 (br d, $^{3}J_{H6''–H5''}$ = 8.4 Hz, 1H, H6”), 7.01 (d, $^{3}J_{H5''–H6''}$ = 8.4 Hz, 1H, H5”), 6.76 (d, $^{3}J_{HC=CH}$ =15.6 Hz, 1H, COCH), 3.83 (s, 3H, OMe), 3.80 (s, 3H, OMe), tetrazole NH not observed; ^{13}C NMR (126 MHz, [D$_6$]DMSO): δ ppm 164.4 (s, CONH), 153.8 (s, Tet-C), 150.7 (s, C3”), 149.0 (s, C4”), 141.8 (s, CHAr), 136.5 (s, C1), 134.1 (s, C3), 131.3 (s, C5), 127.2 (s, C1”), 124.2 (s, C6), 122.5 (s, C6”), 119.4 (s, COCH), 116.0 (s, C2), 115.6 (s, C4), 111.7 (s, C5”), 110.5 (s, C2”), 55.7 (s, OMe), 55.6 (s, OMe); MS (m/z): [M−H, ^{79}Br, 85%]$^-$, 428.2, [M−H, ^{81}Br, 100%]$^-$, 430.2; HRMS (m/z): [M−H]$^-$ calcd. for $C_{18}H_{15}O_3N_5{}^{79}Br$, 428.0364; found 428.0364; error, 0.06 ppm.

***N*-(2-(1*H*-Tetrazol-5-yl)phenyl)thiophene-2-carboxamide (NVR-19)**

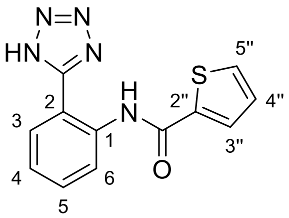

2-Thiophenecarbonyl chloride (163.0 mg, 1.12 mmol) was added dropwise to a solution of 2-(1*H*-tetrazol-5-yl)-aniline (120.0 mg, 0.744 mmol) and DIPEA (191.9 mg, 1.48 mmol) in anhydrous DCM (3 mL) at 0 °C under N_2. This mixture was stirred at 0 °C for 3 h then at room temperature overnight. The reaction mixture was concentrated and extracted with EtOAc (2 × 5 mL) and NaOH (0.1 M, 3 mL). The aqueous layer was acidified with 1M HCl; the precipitate was filtered and washed with water to yield ***N*-(2-(1*H*-tetrazol-5-yl)phenyl)thiophene-2-carboxamide.** White solid; yield 26% (51.5 mg, 0.190 mmol); mp: 230 – 231 °C; ^{1}H NMR (500 MHz, [D$_6$]DMSO): δ ppm 11.53 (s , 1H, CONH) , 8.47 (d, $^{3}J_{H6–H5}$ = 8.1 Hz, 1H, H6), 8.01 (dd, $^{3}J_{H3–H4}$ = 7.9 Hz, $^{4}J_{H3–H5}$ = 1.2 Hz, 1H, H3), 7.92 – 7.97 (m, 2H, H3″ + H5″), 7.62 (ddd, $^{3}J_{H5–H6}$ = 8.2 Hz, $^{3}J_{H5–H4}$ = 7.4 Hz, $^{4}J_{H5–H3}$ = 1.2 Hz, 1H, H5), 7.36 (ddd, $^{3}J_{H4–H3}$ = 7.9 Hz, $^{3}J_{H4–H5}$ = 7.2 Hz, $^{4}J_{H4–H6}$ = 0.9 Hz, 1H, H4), 7.31 (dd, $^{3}J_{H4''–H5''}$ = 4.9 Hz, $^{3}J_{H4''–H3''}$ = 3.8 Hz, 1H, H4″), tetrazole NH not observed; ^{13}C NMR (126 MHz, [D$_6$]DMSO): δ ppm 159.8 (s, CONH), 154.6 (br s, Tet-C), 139.6 (s, C2″), 136.8 (s, C1), 132.6 (s, C3″), 131.9 (s, C5″), 129.0 (s, C4″), 128.7 (s, C5), 128.4 (s, C3), 124.3 (s, C4), 121.9 (s, C6), 113.4 (s, C2); MS (m/z): [M−H, 100%]$^{-}$, 270.2.

***N*-(5-Chloro-2-(1*H*-tetrazol-5-yl)phenyl)thiophene-2-carboxamide (NVR-20)**

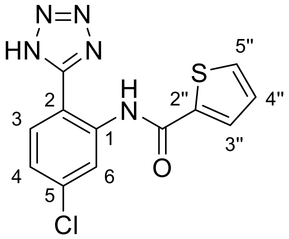

2-Thiophenecarbonyl chloride (134.9 mg, 0.92 mmol) was added dropwise to a solution of 5-chloro-2-(1*H*-tetrazol-5-yl)-aniline (120 mg, 0.61 mmol) and DIPEA (157.7 mg, 1.22 mmol) in anhydrous DCM (3 mL) at 0 °C under N_2. This mixture was stirred at 0 °C for 3 h then at room temperature overnight. The reaction mixture was concentrated and extracted with EtOAc (2

× 5 mL) and NaOH (0.1 M, 3 mL). The aqueous layer was acidified with 1M HCl, the precipitate was filtered and washed with water to yield ***N*-(5-chloro-2-(1*H*-tetrazol-5-yl)phenyl)thiophene-2-carboxamide.** White solid; yield 29% (53.8 mg, 0.176 mmol); mp: 250 – 253 °C; ^{1}H NMR (500 MHz, [D$_6$]DMSO): δ ppm 11.74 (s, 1H, CONH), 8.59 (s, 1H, H6), 8.03 (d, $^3J_{H3–H4}$ = 8.4 Hz, 1H, H3), 7.96 (dd, $^3J_{H3''–H4''}$ = 5.0 Hz, $^4J_{H3''–H5''}$ = 0.9 Hz, 1H, H3''), 7.92 (dd, $^3J_{H5''–H4''}$ = 3.7 Hz, $^4J_{H5''–H3''}$ = 0.9 Hz, 1H, H5''), 7.40 (dd, $^3J_{H4–H3}$ = 8.5 Hz, $^4J_{H4–H6}$ = 2.2 Hz, 1H, H4), 7.31 (dd, $^3J_{H4''–H3''}$ = 4.9 Hz, $^3J_{H4''–H5''}$ = 3.8 Hz, 1H, H4''), tetrazole NH not observed; ^{13}C NMR (126 MHz, [D$_6$]DMSO): δ ppm 159.9 (s, CONH), 154.3 (br s, Tet-C), 139.1 (s, C2''), 138.0 (s, C1), 136.2 (s, C5), 133.1 (s, C3''), 130.0 (s, C4''), 129.2 (s, C5''), 128.5 (s, C3), 123.9 (s, C4), 120.6 (s, C6), 111.6 (s, C2); MS (m/z): [M−H, ^{35}Cl, 100%]$^-$, 304.1, [M−H, ^{37}Cl, 35%]$^-$, 306.1.

2-(3-(3,5-Bis(trifluoromethyl)phenyl)ureido)benzoic acid (NVR-7)

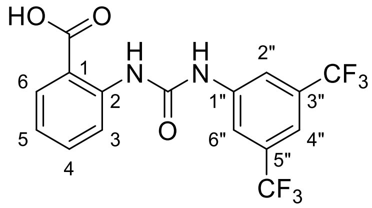

3,5-Bis(trifluoromethyl)phenyl isocyanate (0.23 mL, 1.31 mmol, 1.2 eq) was added dropwise to a stirred solution of 2-aminobenzoic acid (150 mg, 1.09 mmol, 1.0 eq) in acetonitrile (25 mL). The reaction was stirred for 16 hours at room temperature. The white precipitate was filtered and then washed with acetonitrile, before being dried to yield **2-(3-(3,5-bis(trifluoromethyl)phenyl)ureido)benzoic acid.** White solid; yield 47% (199 mg, 0.507 mmol); mp: 196-197 °C; ^{1}H NMR (400 MHz, [D$_6$]DMSO): δ ppm 13.54 (br s, 1H, COOH), 10.66 (s, 1H, NH), 10.53 (s, 1H, NH''), 8.42 (dd, $^3J_{H3–H4}$ = 8.5 Hz, $^4J_{H3–H5}$ = 0.8 Hz, 1H, H3), 8.20 (br s, 2H, H2'' + H6''), 7.98 (dd, $^3J_{H6–H5}$ = 7.9 Hz, $^4J_{H6–H4}$ = 1.6 Hz, 1H, H6), 7.63 (br s, 1H, H4''), 7.56 (ddd, $^3J_{H4–H3}$ = 8.7 Hz, $^3J_{H4–H5}$ = 7.2 Hz, $^4J_{H4–H6}$ = 1.8 Hz, 1H, H4), 7.08 (ddd, $^3J_{H5–H6}$ = 7.9 Hz, $^3J_{H5–H4}$ = 7.2 Hz, $^4J_{H5–H3}$ = 0.8 Hz, 1H, H5); ^{13}C NMR (101 MHz, [D$_6$]DMSO): δ ppm 169.6 (s, COOH), 152.1 (s, CO), 141.9 (s, C1''), 141.6 (s, C2), 133.9 (s, C4), 131.1 (s, C6), 130.7 (q, $^2J_{C\text{-}F}$ = 32.3 Hz, C3'' + C5''), 121.5 (s, C5), 119.7 (s, C3), 123.3 (q, $^1J_{C\text{-}F}$ = 272.9 Hz, 2 × CF_3), 118.0 (br q, $^3J_{C\text{-}F}$ = 3.9 Hz, C2'' + C6''), 115.6 (s, C1), 114.5 (br septet, $^3J_{C\text{-}F}$ = 3.9 Hz, C4''); ^{19}F NMR (376 MHz, [D$_6$]DMSO): δ ppm −61.80 (s, 2 × CF_3); NMR assignments made with the assistance of COSY,

HSQC and HMBC; LCMS (m/z): 391.1; RT = 2.86 min; HRMS (m/z): $[M-H]^-$ calcd. for $C_{16}H_9O_3N_2F_6$, 391.0523; found 391.0520; error, −0.73 ppm.

2-(3-(3,5-Bis(trifluoromethyl)phenyl)ureido)-4-chlorobenzoic acid (NVR-5)

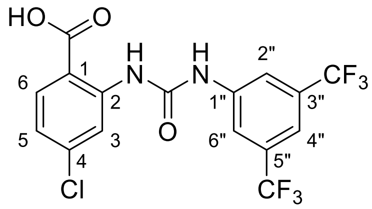

3,5-Bis(trifluoromethyl)phenyl isocyanate (0.36 mL, 2.10 mmol, 1.2 eq) was added dropwise to a stirred solution of 2-amino-4-chlorobenzoic acid (300 mg, 1.75 mmol, 1.0 eq) in acetonitrile (25 mL). The reaction was stirred for 16 hours at room temperature. The precipitate was filtered and then washed with acetonitrile, before being dried to yield **2-(3-(3,5-bis(trifluoromethyl)phenyl)ureido)-4-chlorobenzoic acid.** White solid; yield 23% (172 mg, 0.403 mmol); mp: 211 – 212 °C; ^1H NMR (400 MHz, $[D_6]$DMSO): δ ppm 13.75 (br s, 1H, COOH), 10.77 (s, 1H, NH), 10.63 (s, 1H, NH″), 8.53 (d, $^4J_{H3-H5}$ = 2.3 Hz, 1H, H3), 8.17 (br s, 2H, H2″ + H6″), 7.95 (d, $^3J_{H6-H5}$ = 8.5 Hz, 1H, H6), 7.63 (br s, 1H, H4″), 7.11 (dd, $^3J_{H5-H6}$ = 8.7 Hz, $^4J_{H5-H3}$ = 2.1 Hz, 1H, H5); ^{13}C NMR (101 MHz, $[D_6]$DMSO): δ ppm 168.9 (s, COOH), 151.9 (s, CO), 142.8 (s, C2), 141.6 (s, C1″), 138.5 (s, C4), 132.7 (s, C6), 130.7 (q, $^2J_{C-F}$ = 32.6 Hz, C3″ + C5″), 121.3 (s, C5), 123.3 (q, $^1J_{C-F}$ = 272.9 Hz, 2 × CF_3), 118.7 (s, C3), 118.1 (br q, $^3J_{C-F}$ = 3.9 Hz, C2″ + C6″), 114.8 (br septet, C4″), 114.1 (s, C1); ^{19}F NMR (376 MHz, $[D_6]$DMSO): δ ppm −61.81 (s, 2 × CF_3); NMR assignments made with the assistance of COSY, HSQC and HMBC; MS (m/z): $[M-H, {}^{35}Cl, 100\%]^-$, 425.1, $[M-H, {}^{37}Cl, 45\%]^-$, 427.2.

2-(3,5-Bis(Trifluoromethyl)phenyl)ureido)-4-methylbenzoic acid (NVR-121)

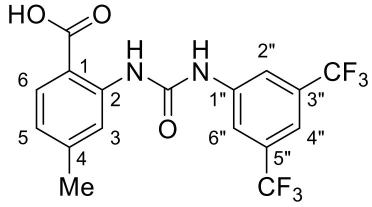

3,5-Bis(trifluoromethyl)phenyl isocyanate (0.41 mL, 2.38 mmol, 1.2 eq) was added dropwise to a stirred solution of 2-amino-4-methylbenzoic acid (300 mg, 1.98 mmol, 1.0 eq) in

acetonitrile (25 mL). The reaction was stirred for 16 hours at room temperature. The precipitate was filtered and then washed with acetonitrile, before being dried to yield **2-(3-(3,5-bis(trifluoromethyl)phenyl)ureido)-4-methylbenzoic acid.** White solid; yield 59% (471 mg, 1.16 mmol); mp: 196 – 197 °C; ^{1}H NMR (400 MHz, [D$_6$]DMSO): δ ppm 13.36 (br s, 1H, COOH), 10.73 (br s, 1H, NH), 10.52 (s, 1H, NH″), 8.28 (s, 1H, H3), 8.21 (br s, 2H, H2″ + H6″), 7.87 (d, $^3J_{H6–H5}$ = 8.0 Hz, 1H, H6), 7.65 (s, 1H, H4″), 6.91 (br d, $^3J_{H5–H6}$ = 8.0 Hz, 1H, H5), 2.35 (s, 3H, Me); ^{13}C NMR (101 MHz, [D$_6$]DMSO): δ ppm 169.6 (s, COOH), 152.0 (s, CO), 144.4 (s, C4), 142.0 (s, C1″), 141.7 (s, C2), 131.1 (s, C6), 130.7 (q, $^2J_{C-F}$ = 32.9 Hz, C3″ + C5″), 122.4 (s, C5), 119.8 (s, C3), 123.3 (q, $^1J_{C-F}$ = 272.9 Hz, 2 × CF_3), 118.0 (br q, $^3J_{C-F}$ = 3.9 Hz, C2″ + C6″), 114.5 (br septet, C4″), 112.9 (s, C1), 21.6 (s, Me); ^{19}F NMR (376 MHz, [D$_6$]DMSO): δ ppm −61.72 (s, 2 × CF_3); NMR assignments made with the assistance of COSY, HSQC and HMBC; LCMS (m/z): [M−H, 100%]$^-$, 405.2; RT = 2.91 min; HRMS (m/z): [M]$^+$ calcd. for $C_{17}H_{12}O_3N_2F_6$, 406.0747; found, 406.0757; error, 2.55 ppm.

2-(3-(3,5-Bis(trifluoromethyl)phenyl)ureido)-4-fluorobenzoic acid (NVR-120)

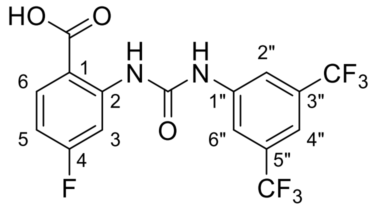

3,5-Bis(trifluoromethyl)phenyl isocyanate (0.40 mL, 2.32 mmol, 1.2 eq) was added dropwise to a stirred solution of 2-amino-4-fluorobenzoic acid (300 mg, 1.93 mmol, 1.0 eq) in acetonitrile (25 mL). The reaction was stirred for 16 hours at room temperature. The precipitate was filtered and then washed with acetonitrile, before being dried to yield **2-(3-(3,5-bis(trifluoromethyl)phenyl)ureido)-4-fluorobenzoic acid.** White solid; yield 38% (298 mg, 0.726 mmol); mp: 200 - 201°C; ^{1}H NMR (400 MHz, [D$_6$]DMSO): δ ppm 13.67 (br s, 1H, COOH), 10.91 (s, 1H, NH), 10.67 (s , 1H, NH″) , 8.29 (dd, $^3J_{H3–F}$ = 12.7 Hz, $^4J_{H3–H5}$ = 2.6 Hz, 1H, H3), 8.20 (br s, 2H, H2″ + H6″), 8.06 (dd, $^3J_{H6–H5}$ = 8.9 Hz, $^4J_{H6–F}$ = 6.9 Hz, 1H, H6), 7.69 (br s, 1H, H4″), 6.93 (ddd, $^3J_{H5–H6}$ = 8.8 Hz, $^3J_{H5–F}$ = 7.8 Hz, $^4J_{H5–H3}$ = 2.6 Hz, 1H, H5); ^{13}C NMR (101 MHz, [D$_6$]DMSO): δ ppm 168.9 (s, COOH), 165.0 (d, $^1J_{C-F}$ = 248.4 Hz, C4), 152.0 (s, CO), 144.0 (d, $^3J_{C-F}$ = 12.7 Hz, C2), 141.6 (s, C1″), 133.8 (d, $^3J_{C-F}$ = 11.7 Hz, C6), 130.7 (q, $^2J_{C-F}$ = 33.3 Hz, C3″ + C5″), 123.3 (q, $^1J_{C-F}$ = 272.9 Hz, 2 × CF_3), 118.1 (br q, $^3J_{C-F}$ = 3.9 Hz, C2″ + C6″), 114.8 (br septet, $^3J_{C-F}$

= 3.9 Hz, C4″), 111.8 (d, $^4J_{C\text{-}F}$ = 2.9 Hz, C1), 108.5 (d, $^2J_{C\text{-}F}$ = 22.5 Hz, C5), 105.9 (d, $^2J_{C\text{-}F}$ = 28.4 Hz, C3); ^{19}F NMR (376 MHz, [D$_6$]DMSO): δ ppm −61.71 (s, 2 × CF_3), -103.34 (ddd, $^3J_{F\text{-}H3}$ = 12.7 Hz, $^3J_{F\text{-}H5}$ = 7.8 Hz, $^4J_{F\text{-}H6}$ = 6.9 Hz, F); NMR assignments made with the assistance of COSY, HSQC and HMBC; LCMS (m/z): [M+H, 100%]$^+$, 411.2; RT = 3.00 min.

2-(3-(3,5-Bis(trifluoromethyl)phenyl)ureido)-4-methoxybenzoic acid (NVR-123)

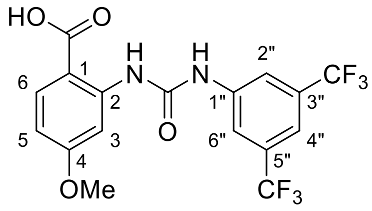

3,5-Bis(trifluoromethyl)phenyl isocyanate (0.37 mL, 2.15 mmol, 1.2 eq) was added dropwise to a stirred solution of 2-amino-4-methoxybenzoic acid (300 mg, 1.79 mmol, 1.0 eq) in acetonitrile (25 mL). The reaction was stirred for 16 hours at room temperature. The precipitate was filtered and then washed with acetonitrile, before being dried to yield **2-(3-(3,5-bis(trifluoromethyl)phenyl)ureido)-4-methoxybenzoic acid.** White solid; yield 35% (261 mg, 0.618 mmol); mp: 198 – 199 °C; ^{1}H NMR (400 MHz, [D$_6$]DMSO): δ ppm 13.20 (br s, 1H, COOH), 10.92 (s, 1H, NH), 10.56 (s, 1H, NH″), 8.21 (br s, 2H, H2″ + H6″), 8.09 (d, $^4J_{H3\text{-}H5}$ = 2.5 Hz, 1H, H3), 7.93 (d, $^3J_{H6\text{-}H5}$ = 9.0 Hz, 1H, H6), 7.66 (br s, 1H, H4″), 6.66 (dd, $^3J_{H5\text{-}H6}$ = 8.9 Hz, $^4J_{H5\text{-}H3}$ = 2.6 Hz, 1H, H5), 3.83 (s, 3H, OMe); ^{13}C NMR (101 MHz, [D$_6$]DMSO): δ ppm 169.5 (s, COOH), 163.6 (s, C4), 152.1 (s, CO), 143.8 (s, C2), 141.9 (s, C1″), 133.0 (s, C6), 130.7 (q, $^2J_{C\text{-}F}$ = 32.3 Hz, C3″ + C5″), 123.3 (q, $^1J_{C\text{-}F}$ = 272.6 Hz, 2 × CF_3), 118.1 (br q, $^3J_{C\text{-}F}$ = 3.9 Hz, C2″ + C6″), 114.6 (br septet, $^3J_{C\text{-}F}$ = 3.9 Hz, C4″), 107.8 (s, C1 + C5), 103.8 (s, C3), 55.4 (s, OMe); ^{19}F NMR (376 MHz, [D$_6$]DMSO): δ ppm −61.68 (s, 2 × CF_3); NMR assignments made with the assistance of COSY, HSQC and HMBC; LCMS (m/z): [M−H, 100%]$^-$, 421.2; RT = 2.84 min; HRMS (m/z): calcd. for $C_{17}H_{13}O_4N_2F_6$ [M+H]$^+$, 423.0774; found, 423.0772; error, 0.5 ppm.

2-(3-(3,5-Bis(trifluoromethyl)phenyl)ureido)-5-chlorobenzoic acid (NVR-119)

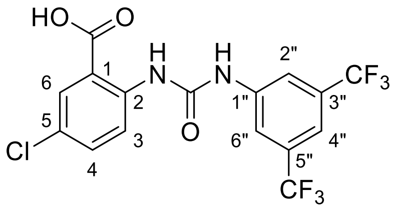

3,5-Bis(trifluoromethyl)phenyl isocyanate (0.36 mL, 2.10 mmol, 1.2 eq) was added dropwise to a stirred solution of 2-amino-4-chlorobenzoic acid (300 mg, 1.75 mmol, 1.0 eq) in acetonitrile (25 mL). The reaction was stirred for 16 hours at room temperature. The precipitate was filtered and then washed with acetonitrile, before being dried to yield **2-(3-(3,5-bis(trifluoromethyl)phenyl)ureido)-5-chlorobenzoic acid.** White solid; yield 35% (261 mg, 0.612 mmol); mp: 211 – 212 °C; ^{1}H NMR (400 MHz, [D$_6$]DMSO): δ ppm 13.89 (br s, 1H, COOH), 10.59 (s, 1H, NH), 10.56 (s, 1H, NH″), 8.45 (d, $^{3}J_{H3–H4}$ = 9.0 Hz, 1H, H3), 8.16 (br s, 2H, H2″ + H6″), 7.88 (d, $^{4}J_{H6–H4}$ = 2.8 Hz, 1H, H6), 7.61 (br s, 1H, H4″), 7.58 (dd, $^{3}J_{H4–H3}$ = 9.2 Hz, $^{4}J_{H4–H6}$ = 2.6 Hz, 1H, H4); ^{13}C NMR (101 MHz, [D$_6$]DMSO): δ ppm 168.4 (s, COOH), 151.9 (s, CO), 141.8 (s, C1″), 140.5 (s, C2), 133.5 (s, C4), 130.7 (q, $^{2}J_{C-F}$ = 32.3 Hz, C3″ + C5″), 130.1 (s, C6), 125.1 (s, C5), 121.5 (s, C3), 123.3 (q, $^{1}J_{C-F}$ = 272.9 Hz, 2 × CF$_3$), 118.0 (br q, $^{3}J_{C-F}$ = 3.9 Hz, C2″ + C6″), 117.2 (s, C1), 114.6 (br septet, $^{3}J_{C-F}$ = 3.9 Hz, C4″); ^{19}F NMR (376 MHz, [D$_6$]DMSO): δ ppm −61.86 (s, 2 × CF$_3$); NMR assignments made with the assistance of COSY, HSQC and HMBC; LCMS (m/z): [M+H, ^{35}Cl, 100%]$^+$, 427.1, [M+H, ^{37}Cl, 100%]$^+$, 427.1; RT = 2.95 min.

2-(3-(3,5-Bis(trifluoromethyl)phenyl)ureido)-5-methylbenzoic acid (NVR-122)

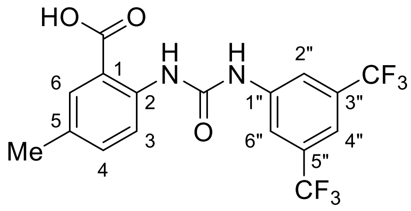

3,5-Bis(trifluoromethyl)phenyl isocyanate (0.41 mL, 2.38 mmol, 1.2 eq) was added dropwise to a stirred solution of 2-amino-5-methylbenzoic acid (300 mg, 1.98 mmol, 1.0 eq) in acetonitrile (25 mL). The reaction was stirred for 16 hours at room temperature. The precipitate was filtered and then washed with acetonitrile, before being dried to yield **2-(3-(3,5-bis(trifluoromethyl)phenyl)ureido)-5-methylbenzoic acid.** White solid; yield 75% (607

mg, 1.49 mmol); mp: 199 – 200 °C; ^{1}H NMR (400 MHz, [D$_6$]DMSO): δ ppm 13.48 (br s, 1H, COOH), 10.54 (br s, 1H, NH), 10.49 (s, 1H, NH″), 8.30 (d, $^3J_{H3–H4}$ = 8.5 Hz, 1H, H3), 8.19 (br s, 2H, H2″ + H6″), 7.79 (d, $^4J_{H6–H4}$ = 2.0 Hz, 1H, H6), 7.65 (br s, 1H, H4″), 7.39 (dd, $^3J_{H4–H3}$ = 8.8 Hz, $^4J_{H4–H6}$ = 2.0 Hz, 1H, H4), 2.29 (s, 3H, Me); ^{13}C NMR (101 MHz, [D$_6$]DMSO): δ ppm 169.6 (s, COOH), 152.1 (s, CO), 142.0 (s, C1″), 139.2 (s, C2), 134.6 (s, C4), 131.0 (s, C6), 130.5 (s, C5), 130.7 (q, $^2J_{C-F}$ = 32.3 Hz, C3″ + C5″), 119.7 (s, C3), 123.3 (q, $^1J_{C-F}$ = 272.6 Hz, 2 × CF_3), 117.9 (br q, $^3J_{C-F}$ = 3.9 Hz, C2″ + C6″), 115.5 (s, C1), 114.4 (br septet, $^3J_{C-F}$ = 3.9 Hz, C4″), 20.0 (s, Me); ^{19}F NMR (376 MHz, [D$_6$]DMSO): δ ppm −61.72 (s, 2 × CF_3); NMR assignments made with the assistance of COSY, HSQC and HMBC; LCMS (m/z): [M−H, 100%]$^-$, 405.2; RT = 2.90 min; HRMS (m/z): calcd. for $C_{17}H_{12}O_3N_2F_6$ [M]$^+$, 406.0747; found, 406.0750; error, 0.83 ppm.

2-(3-(3,5-Bis(trifluoromethyl)phenyl)ureido)-5-fluorobenzoic acid (NVR-9)

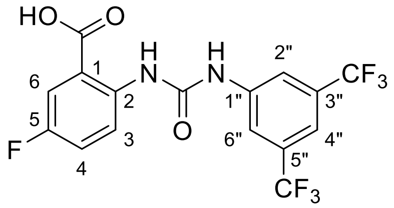

3,5-Bis(trifluoromethyl)phenyl isocyanate (0.40 mL, 2.32 mmol, 1.2 eq) was added dropwise to a stirred solution of 2-amino-4-fluorobenzoic acid (300 mg, 1.93 mmol, 1.0 eq) in acetonitrile (25 mL). The reaction was stirred for 16 hours at room temperature. The precipitate was filtered and then a small portion (≈50 mg) triturated with hot toluene, before being dried to yield **2-(3-(3,5-bis(trifluoromethyl)phenyl)ureido)-5-fluorobenzoic acid.** White solid; yield 4% (31 mg, 0.0756 mmol); mp: 204 – 205 °C; ^{1}H NMR (400 MHz, [D$_6$]DMSO): δ ppm 13.89 (br s, 1H, COOH), 10.54 (s, 1H, NH″), 10.48 (s, 1H, NH), 8.41 (dd, $^3J_{H3–H4}$ = 9.4 Hz, $^4J_{H3–F}$ = 5.1 Hz, 1H, H3), 8.18 (br s, 2H, H2″ + H6″), 7.67 (dd, $^3J_{H6–F}$ = 9.4 Hz, $^4J_{H6–H4}$ = 3.1 Hz, 1H, H6), 7.64 (br s, 1H, H4″), 7.45 (ddd, $^3J_{H4–H3}$ = 9.3 Hz, $^3J_{H4–F}$ = 7.8 Hz, $^4J_{H4–H6}$ = 3.1 Hz, 1H, H4); ^{13}C NMR (101 MHz, [D$_6$]DMSO): δ ppm 168.4 (d, $^4J_{C-F}$ = 2.0 Hz, COOH), 156.1 (d, $^1J_{C-F}$ = 239.6 Hz, C5), 152.1 (s, CO), 141.9 (s, C1″), 138.0 (d, $^4J_{C-F}$ = 2.0 Hz, C2), 130.7 (q, $^2J_{C-F}$ = 32.6 Hz, C3″ + C5″), 121.9 (br d, $^3J_{C-F}$ = 3.9 Hz, C3), 120.9 (d, $^2J_{C-F}$ = 22.5 Hz, C4), 123.3 (q, $^1J_{C-F}$ = 274.2 Hz, 2 × CF_3), 118.0 (q, $^3J_{C-F}$ = 3.9 Hz, C2″ + C6″), 117.4 (d, $^3J_{C-F}$ = 6.8 Hz, C1), 116.6 (d, $^2J_{C-F}$ = 23.5 Hz, C6), 114.6 (br septet, $^3J_{C-F}$ = 3.9 Hz, C4″); ^{19}F NMR (376 MHz, [D$_6$]DMSO): δ ppm −61.78 (s, 2 × CF_3), −120.86 (ddd, $^3J_{F-H6}$ = 9.2 Hz, $^3J_{F-H4}$ = 8.2 Hz, $^4J_{F-H3}$ = 5.1 Hz, F); NMR assignments made

with the assistance of COSY, HSQC and HMBC; LCMS (m/z): [M−H, 100%]$^-$, 409.1; RT = 2.85 min.

2-(3-(3,5-Bis(trifluoromethyl)phenyl)ureido)-5-methoxybenzoic acid (NVR-124)

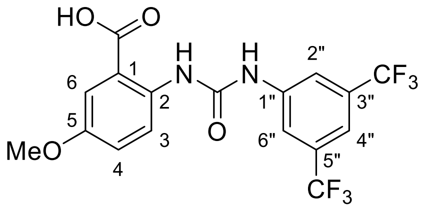

3,5-Bis(trifluoromethyl)phenyl isocyanate (0.37 mL, 2.15 mmol, 1.2 eq) was added dropwise to a stirred solution of 2-amino-5-methoxybenzoic acid (300 mg, 1.79 mmol, 1.0 eq) in acetonitrile (25 mL). The reaction was stirred for 16 hours at room temperature. The precipitate was filtered and then washed with acetonitrile, before being dried to yield **2-(3-(3,5-bis(trifluoromethyl)phenyl)ureido)-5-methoxybenzoic acid.** White solid; yield 59% (446 mg, 1.06 mmol); mp: 202 – 203 °C; ^{1}H NMR (400 MHz, [D$_6$]DMSO): δ ppm 13.62 (br s, 1H, COOH), 10.43 (s, 1H, NH″), 10.30 (s, 1H, NH), 8.29 (d, $^3J_{H3–H4}$ = 9.3 Hz, 1H, H3), 8.18 (br s, 2H, H2″ + H6″), 7.63 (br s, 1H, H4″), 7.45 (d, $^4J_{H6–H4}$ = 3.3 Hz, 1H, H6), 7.20 (dd, $^3J_{H4–H3}$ = 9.2 Hz, $^4J_{H4–H6}$ = 3.1 Hz, 1H, H4), 3.77 (s, 3H, OMe); ^{13}C NMR (101 MHz, [D$_6$]DMSO): δ ppm 169.2 (s, COOH), 153.4 (s, C5), 152.2 (s, CO), 142.1 (s, C1″), 134.8 (s, C2), 130.7 (q, $^2J_{C-F}$ = 32.9 Hz, C3″ + C5″), 121.7 (s, C3), 120.3 (s, C4), 123.3 (q, $^1J_{C-F}$ = 272.9 Hz, 2 × CF$_3$), 117.8 (br q, $^3J_{C-F}$ = 3.9 Hz, C2″ + C6″), 117.0 (s, C1), 114.4 (s, C6), 114.3 (br septet, $^3J_{C-F}$ = 3.9 Hz, C4″), 55.3 (s, OMe); ^{19}F NMR (376 MHz, [D$_6$]DMSO): δ ppm −61.75 (s, 2 × CF$_3$); NMR assignments made with the assistance of COSY, HSQC and HMBC; LCMS (m/z): [M−H, 100%]$^-$, 421.2; RT = 2.79 min; HRMS (m/z): [M+H]$^+$ calcd. for $C_{17}H_{13}O_4N_2F_6$, 423.0774; found, 423.0766; error, −1.90 ppm.

2-(3-(3,5-Bis(trifluoromethyl)phenyl)ureido)-5-iodobenzoic acid (NVR-12)

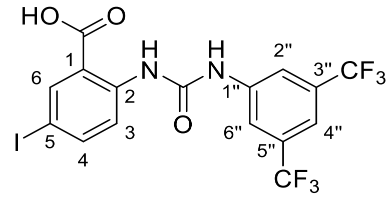

2-Amino-5-iodobenzoic acid (150.0 mg, 0.570 mmol) and 3,5-bis(trifluoromethyl)phenyl isocyanate (145.5 mg, 0.570 mmol) were suspended in THF (3 mL). This mixture was stirred at room temperature for 2 h then stored in a refrigerator overnight. The white solid that precipitated was filtered and washed with DCM to yield **2-(3-(3,5-bis(trifluoromethyl)phenyl)ureido)-5-iodobenzoic acid.** White solid; yield 15% (43 mg, 0.083 mmol); mp: 208 – 210 °C; ^{1}H NMR (500 MHz, [D$_6$]DMSO): δ ppm 13.88 (br s, 1H, COOH), 10.60 (s, 2H, NH, NH″), 8.26 (d, $^{3}J_{H3–H4}$ = 8.9 Hz, 1H, H3), 8.21 (d, $^{4}J_{H6–H4}$ = 2.3 Hz, 1H, H6), 8.18 (br s, 2H, H2″ + H6″), 7.87 (dd, $^{3}J_{H4–H3}$ = 8.9 Hz, $^{4}J_{H4–H6}$ = 2.2 Hz, 1H, H4), 7.66 (s, 1H, H4″); ^{13}C NMR (126 MHz, [D$_6$]DMSO): δ ppm 168.2 (s, COOH), 151.8 (s, CO), 142.1 (s, C6), 141.7 (s, C2), 141.3 (s, C1″), 138.9 (s, C4), 130.7 (q, $^{2}J_{C\text{-}F}$ = 32.6 Hz, C3″ + C5″), 121.8 (s, C3), 123.3 (q, $^{1}J_{C\text{-}F}$ = 272.6 Hz, CF_3 × 2), 118.0 (br q, $^{3}J_{C\text{-}F}$ = 3.9 Hz, C2″ + C6″), 117.7 (s, C1), 114.7 (br septet, $^{3}J_{C\text{-}F}$ = 3.9 Hz, C4″), 84.3 (s, C5); ^{19}F NMR (471 MHz, [D$_6$]DMSO): δ ppm −61.75 (s, 2 × CF_3); MS (m/z): [M−H, 100%]$^{-}$, 517.0.

2-(3-(3,5-Bis(trifluoromethyl)phenyl)ureido)-4,5-dichlorobenzoic acid (NVR-132)

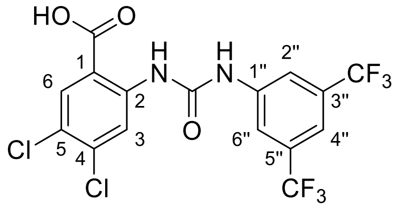

3,5-Bis(trifluoromethyl)phenyl isocyanate (740 mg, 2.9 mmol) was added dropwise to a solution of 2-amino-4,5-dichlorobenzoic acid (300 mg, 1.47 mmol) in a mixture of anhydrous THF (3 mL) and toluene (5 mL) at room temperature under N_2. This mixture was stirred overnight at room temperature; then stored in a refrigerator overnight. The white solid that precipitated was filtered and washed with DCM to yield **2-(3-(3,5-**

bis(trifluoromethyl)phenyl)ureido)-4,5-dichlorobenzoic acid. White solid; yield 66% (400 mg, 0.867 mmol); mp: 218 – 220 °C; ^{1}H NMR (400 MHz, [D$_6$]DMSO): δ ppm 10.68 (s, 2H, NH, NH″), 8.70 (s, 1H, H3), 8.14 (br s, 2H, H2″ + H6″), 8.01 (s, 1H, H6), 7.63 (br s, 1H, H4″), COOH not observed; ^{13}C NMR (101 MHz, [D$_6$]DMSO): δ ppm 167.8 (s, COOH), 151.8 (s, CO), 141.5 (s, C2), 141.2 (s, C1″), 136.3 (s, C4), 132.0 (s, C6), 130.7 (q, $^{2}J_{C\text{-}F}$ = 32.3 Hz, C3″ + C5″), 123.0 (s, C5), 120.7 (s, C3), 123.3 (q, $^{1}J_{C\text{-}F}$ = 272.9 Hz, 2 × CF_3), 118.1 (br q, $^{3}J_{C\text{-}F}$ = 3.9 Hz, C2″ + C6″), 115.8 (s, C1), 114.9 (br septet, $^{3}J_{C\text{-}F}$ = 3.9 Hz, C4″); ^{19}F NMR (376 MHz, [D$_6$]DMSO): δ ppm −61.79 (s, 2 × CF_3); MS (m/z): [M−H, ^{35}Cl^{35}Cl, 100%]$^{-}$, 459.1, [M−H, ^{35}Cl^{37}Cl, 85%]$^{-}$, 461.0, [M−H, ^{37}Cl^{37}Cl, 15%]$^{-}$, 463.0.

2-(3-(3,5-Bis(trifluoromethyl)phenyl)ureido)-4,5-difluorobenzoic acid (NVR-149)

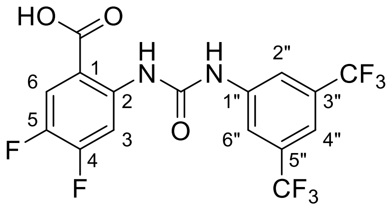

3,5-Bis(trifluoromethyl)phenyl isocyanate (0.36 mL, 2.08 mmol, 1.2 eq) was added dropwise to a stirred solution of 2-amino-4,5-fluorobenzoic acid (300 mg, 1.73 mmol, 1.0 eq) in acetonitrile (25 mL). The reaction was stirred for 16 hours at room temperature. The precipitate was filtered and then washed with acetonitrile, before being dried to yield **2-(3-(3,5-bis(trifluoromethyl)phenyl)ureido)-4,5-difluorobenzoic acid.** White solid; yield 38% (283 mg, 0.661 mmol), mp: 211 – 212 °C; ^{1}H NMR (400 MHz, [D$_6$]DMSO): δ ppm 13.94 (br s, 1H, COOH), 10.71 (s, 1H, NH), 10.62 (s , 1H, NH″), 8.45 (dd, $^{3}J_{H3\text{–}F4}$ = 14.2, $^{4}J_{H3\text{–}F5}$ = 7.7 Hz, 1H, H3), 8.14 (br s, 2H, H2″ + H6″), 7.87 (dd, $^{3}J_{H6\text{–}F5}$ = 11.2 Hz, $^{4}J_{H6\text{–}F4}$ = 9.4 Hz, 1H, H6), 7.62 (br s, 1H, H4″); ^{13}C NMR (101 MHz, [D$_6$]DMSO): δ ppm 167.9 (s, COOH), 151.9 (s, CO), 152.2 (dd, $^{1}J_{C\text{–}F}$ = 250.4 Hz, $^{2}J_{C\text{-}F}$ = 13.7 Hz, C4), 143.4 (dd, $^{1}J_{C\text{-}F}$ = 242.1 Hz, $^{2}J_{C\text{-}F}$ = 13.2 Hz, C5), 141.6 (s, C1″), 139.5 (dd, $^{3}J_{C\text{-}F}$ = 11.7 Hz, $^{4}J_{C\text{-}F}$ = 2.0 Hz, C2), 130.7 (q, $^{2}J_{C\text{-}F}$ = 32.3 Hz, C3″ + C5″), 126.0 (q, $^{1}J_{C\text{-}F}$ = 272.9 Hz, 2 × CF_3), 119.3 (d, $^{2}J_{C\text{-}F}$ = 18.6 Hz, $^{3}J_{C\text{-}F}$ = 2.0 Hz, C6), 118.0 (br q, $^{3}J_{C\text{-}F}$ = 3.9 Hz, C2″ + C6″), 114.8 (br sept, $^{3}J_{C\text{-}F}$ = 3.9 Hz, C4″), 112.2 (dd, $^{3}J_{C\text{-}F}$ = $^{4}J_{C\text{-}F}$ = 3.5 Hz, C1), 108.2 (d, $^{2}J_{C\text{-}F}$ = 24.5 Hz, C3); ^{19}F NMR (376 MHz, [D$_6$]DMSO): δ ppm −61.83 (s, 2 × CF_3), −128.41 (dd, $^{3}J_{FH}$ = 14.0 Hz, $^{4}J_{FH}$ = 9.5 Hz, F), −128.47 (dd, $^{3}J_{FH}$ = 14.0 Hz, $^{4}J_{FH}$ = 9.2 Hz); NMR assignments made

with the assistance of COSY, HSQC and HMBC; LCMS (m/z): [M−H, 100%]$^-$, 427.3; RT = 3.04 min; HRMS (m/z): [MH]$^+$ calcd. for $C_{16}H_7O_3N_2F_8$, 427.0334; found, 427.0320; error, −3.37 ppm.

2-(3-(3,5-Bis(trifluoromethyl)phenyl)ureido)-4,5-dimethoxybenzoic acid (NVR-150)

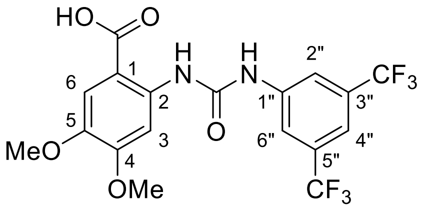

3,5-Bis(trifluoromethyl)phenyl isocyanate (0.32 mL, 1.83 mmol, 1.2 eq) was added dropwise to a stirred solution of 2-amino-4,5-methoxybenzoic acid (300 mg, 1.52 mmol, 1.0 eq) in acetonitrile (25 mL). The reaction was stirred for 16 hours at room temperature. The precipitate was filtered and then washed with acetonitrile, before being dried to yield **2-(3-(3,5-bis(trifluoromethyl)phenyl)ureido)-4,5-dimethoxybenzoic acid.** Off white solid; yield 76% (523 mg, 1.16 mmol); mp: 187 – 188 °C; ^{1}H NMR (400 MHz, [D$_6$]DMSO): δ ppm 13.26 (br s, 1H, COOH), 10.75 (s, 1H, NH), 10.49 (s, 1H, NH″), 8.20 (br s, 2H, H2″ + H6″), 8.16 (s, 1H, H3), 7.63 (br s, 1H, H4″), 7.42 (s, 1H, H6), 3.84 (s, 3H, OMe), 3.75 (s, 3H, OMe); ^{13}C NMR (101 MHz, [D$_6$]DMSO): δ ppm 169.3 (s, COOH), 153.3 (s, C4), 152.2 (s, CO), 142.9 (s, C5), 142.0 (s, C1″), 137.6 (s, C2), 130.7 (q, $^2J_{C-F}$ = 32.6 Hz, C3″ + C5″), 123.4 (q, $^1J_{C-F}$ = 272.9 Hz, 2 × CF_3), 117.9 (br q, $^3J_{C-F}$ = 3.9 Hz, C2″ + C6″), 114.4 (br septet, $^3J_{C-F}$ = 3.9 Hz, C4″), 112.7 (s, C6), 106.7 (s, C1), 102.8 (s, C3), 55.6 (s, 2 × OMe); ^{19}F NMR (376 MHz, [D$_6$]DMSO): δ ppm −61.72 (s, 2 × CF_3); NMR assignments made with the assistance of COSY, HSQC and HMBC; LCMS (m/z): [M−H, 100%]$^-$, 451.3; RT = 2.85 min; HRMS (m/z): [M−H]$^-$ calcd. for $C_{18}H_{13}O_5N_2F_6$, 451.0734; found, 451.0726; error, −1.80 ppm.

3-(3-(3,5-Bis(trifluoromethyl)phenyl)ureido)benzoic acid (NVR-118)

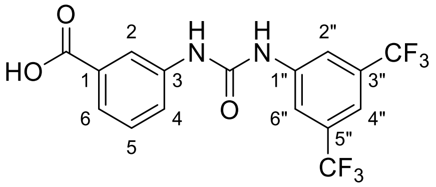

3,5-Bis(trifluoromethyl)phenyl isocyanate (0.46 mL, 2.63 mmol, 1.2 eq) was added dropwise to a stirred solution of 3-aminobenzoic acid (300 mg, 2.19 mmol, 1.0 eq) in acetonitrile (25 mL). The reaction was stirred for 16 hours at room temperature. The white precipitate was filtered and then washed with acetonitrile, before being dried to yield **3-(3-(3,5-bis(trifluoromethyl)phenyl)ureido)benzoic acid.** White solid; yield 70% (596 mg, 1.52 mmol); mp: >300°C; ^{1}H NMR (400 MHz, [D$_6$]DMSO): δ ppm 13.03 (s, 1H, COOH), 9.47 (s, 1H, NH″), 9.28 (s, 1H, NH), 8.13 – 8.18 (m, 3H, H2 + H2″ + H6″), 7.67 (s, 2H, H4 + H4″), 7.59 (br d, $^{3}J_{H6–H5}$ = 7.8 Hz, 1H, H6), 7.43 (t, $^{3}J_{H5–H4}$ = $^{3}J_{H5–H6}$ = 7.9 Hz, 1H, H5); ^{13}C NMR (101 MHz, [D$_6$]DMSO): δ ppm 167.2 (s, COOH), 152.5 (s, CO), 141.8 (s, C1″), 139.4 (s, C3), 131.4 (s, C1), 130.7 (q, $^{2}J_{C\text{-}F}$ = 33.3 Hz, C3″ + C5″), 129.0 (s, C5), 123.4 (s, C6), 123.1 (s, C4), 119.6 (s, C2), 123.3 (q, $^{1}J_{C\text{-}F}$ = 272.9 Hz, 2 × CF_3), 118.2 (br q, $^{3}J_{C\text{-}F}$ = 3.9 Hz, C2″ + C6″), 114.5 (br septet, $^{3}J_{C\text{-}F}$ = 3.9 Hz, C4″); ^{19}F NMR (376 MHz, [D$_6$]DMSO): δ ppm −61.60 (s, 2 × CF_3); NMR assignments made with the assistance of COSY, HSQC and HMBC; LCMS (m/z): [M+H, 100%]$^+$, 393.3; RT = 3.03 min; HRMS (m/z): [M−H]$^-$ calcd. for $C_{16}H_9O_3N_2F_6$, 391.0523; found 391.0527; error, 1.06 ppm.

4-(3-(3,5-Bis(trifluoromethyl)phenyl)ureido)benzoic acid (NVR-117)

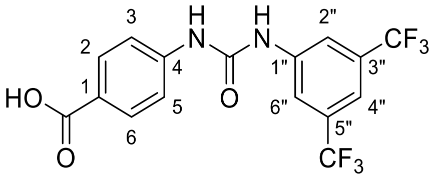

3,5-Bis(trifluoromethyl)phenyl isocyanate (0.46 mL, 2.63 mmol, 1.2 eq) was added dropwise to a stirred solution of 4-aminobenzoic acid (300 mg, 2.19 mmol, 1.0 eq) in acetonitrile (25 mL). The reaction was stirred for 16 hours at room temperature. The white precipitate was filtered and then washed with acetonitrile, before being dried to yield **4-(3-(3,5-bis(trifluoromethyl)phenyl)ureido)benzoic acid.** White solid; yield 57% (486 mg, 1.24 mmol); mp: >300 °C; ^{1}H NMR (400 MHz, [D$_6$]DMSO): δ ppm 12.65 (br s, 1H, COOH), 9.50 (s, 1H, NH″),

9.38 (s, 1H, NH), 8.15 (br s, 2H, H2″ + H6″), 7.89 (br d, $^3J_{H2/H6-H3/H5}$ = 8.8 Hz, 2H, H2 + H6), 7.67 (br s, 1H, H4″), 7.61 (br d, $^3J_{H3/H5-H2/H6}$ = 8.8 Hz, 2H, H3 + H5); ^{13}C NMR (101 MHz, [D$_6$]DMSO): δ ppm 167.0 (s, COOH), 152.2 (s, CO), 143.3 (s, C4), 141.6 (s, C1″), 130.5 (s, C2 + C6), 130.8 (q, $^2J_{C-F}$ = 32.3 Hz, C3″ + C5″), 124.3 (s, C1), 123.3 (q, $^1J_{C-F}$ = 272.9 Hz, 2 × CF_3), 118.2 (br q, $^3J_{C-F}$ = 3.9 Hz, C2″ + C6″), 117.9 (s, C3 + C5), 114.7 (br septet, $^3J_{C-F}$ = 3.9 Hz, C4″); ^{19}F NMR (376 MHz, [D$_6$]DMSO): δ ppm −61.61 (s, 2 × CF_3); NMR assignments made with the assistance of COSY, HSQC and HMBC; LCMS (m/z): [M−H, 100%]$^-$, 391.2; RT = 3.03 min; HRMS (m/z): [M−H]$^-$ calcd. for $C_{16}H_9O_3N_2F_6$, 391.0523; found 391.0530; error, 1.83 ppm.

1-(3,5-Bis(trifluoromethyl)phenyl)-3-(5-chloro-2-(1*H*-tetrazol-5-yl)phenyl)urea (NVR-58)

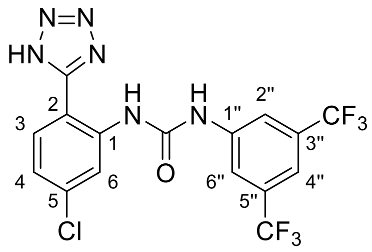

To a stirred solution of 5-chloro-2-(1*H*-tetrazol-5-yl)aniline (300 mg, 1.53 mmol. 1 eq) in toluene (25 mL) was added 3,5-bis(trifluoromethyl)phenyl isocyanate (470 mg, 1.84 mmol, 1.2 eq). The reaction mixture was stirred at room temperature for 16 h. The resultant white precipitate was filtered and triturated with toluene (10 mL) before filtering and drying to yield **1-(3,5-bis(trifluoromethyl)phenyl)-3-(5-chloro-2-(1*H*-tetrazol-5-yl)phenyl)urea.** White solid; yield 36% (250 mg, 0.556 mmol). ^{1}H NMR (400 MHz, [D$_6$]DMSO): δ ppm 10.62 (s, 1H, NH″), 10.28 (s, 1H, NH), 8.48 (d, $^4J_{H6-H4}$ = 2.3 Hz, 1H, H6), 8.21 (s, 2H, H2″ + H6″), 7.95 (d, $^3J_{H3-H4}$ = 8.5 Hz, 1H, H3), 7.69 (s, 1H, H4″), 7.36 (dd, $^3J_{H4-H3}$ = 8.4 Hz, $^4J_{H4-H6}$ = 2.1 Hz, 1H, H4), tetrazole NH not observed; ^{13}C NMR (101 MHz, [D$_6$]DMSO): δ ppm 152.1 (s, C7″), 141.6 (s, C1″), 138.8 (s, C1), 136.1 (s, C5), 130.7 (q, $^2J_{C-F}$ = 32.3 Hz, C3″ + C5″), 130.0 (s, C3), 122.6 (s, C4), 120.4 (s, C6), 123.3 (q, $^1J_{C-F}$ = 273.6 Hz, 2 × CF_3), 118.4 (br q, $^3J_{C-F}$ = 3.9 Hz, C2″+ C6″), 115.0 (br septet, $^3J_{C-F}$ = 3.9 Hz, C4″), 110.7 (s, C2), tetrazole carbon not observed; ^{19}F NMR (376 MHz, [D$_6$]DMSO): δ ppm −61.69 (s, 2 × CF_3);

1-(2-(1*H*-Tetrazol-5-yl)-5-(trifluoromethyl)phenyl)-3-(3,5-bis(trifluoromethyl)phenyl)urea (NVR-133)

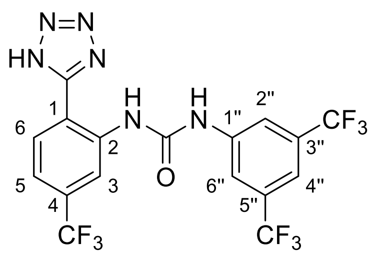

3,5-Bis(trifluoromethyl)phenyl isocyanate (250 mg, 1.1mmol) was added dropwise to a solution of 5-trifluoromethyl-2-(1*H*-tetrazol-5-yl)aniline (200 mg, 0.873 mmol) in a mixture of anhydrous THF (3 mL) and toluene (5 mL) at room temperature under N_2. This mixture was stirred overnight at room temperature; then stored in a refrigerator overnight. The creamy solid that precipitated was filtered and washed with DCM to yield **1-(2-(1*H*-tetrazol-5-yl)-5-(trifluoromethyl)phenyl)-3-(3,5-bis(trifluoromethyl)phenyl)urea.** Cream solid; yield 36% (150 mg, 0.310 mmol); mp: 197 – 199 °C; ^{1}H NMR (400 MHz, [D$_6$]DMSO): δ ppm 10.67 (s, 1H, NH″), 10.36 (s, 1H, NH), 8.76 (d, $^4J_{H3-H5}$ = 1.0 Hz, 1H, H3), 8.19 (s, 2H, H2″ + H6″), 8.18 (d, $^3J_{H6-H5}$ = 8.1 Hz, 1H, H6), 7.68 (s, 1H, H4″), 7.62 (dd, $^3J_{H5-H6}$ = 8.3 Hz, $^4J_{H5-H3}$ = 1.2 Hz, 1H, H5), tetrazole NH not observed; ^{13}C NMR (101 MHz, [D$_6$]DMSO): δ ppm 152.2 (s, CO), 141.6 (s, C1″), 138.1 (s, C2), 131.2 (q, $^2J_{C-F}$ = 32.0 Hz, C4), 130.7 (q, $^2J_{C-F}$ = 33.3 Hz, C3″ + C5″), 129.8 (s, C6), 123.7 (q, $^1J_{C-F}$ = 271.3 Hz, CF_3), 123.3 (q, $^1J_{C-F}$ = 272.6 Hz, 2 × CF_3), 118.9 (br q, $^3J_{C-F}$ = 3.9 Hz, C5), 118.4 (br q, $^3J_{C-F}$ = 3.9 Hz, C2″ + C6″), 117.4 (br q, $^3J_{C-F}$ = 3.9 Hz, C3), 115.6 (s, C1), 115.0 (br septet, $^3J_{C-F}$ = 3.9 Hz, C4″), tetrazole carbon not observed; ^{19}F NMR (376 MHz, [D$_6$]DMSO): δ ppm −61.73 (s, 2 × CF_3), -61.85 (s, CF_3); MS (m/z): [M−H, 100%]$^-$, 483.2.

1-(3,5-Bis(trifluoromethyl)phenyl)-3-(4-chloro-2-(1*H*-tetrazol-5-yl)phenyl)urea (NVR-41)

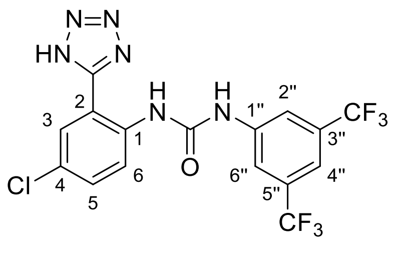

3,5-Bis(trifluoromethyl)phenyl isocyanate (130.0 mg, 0.511 mmol) was added dropwise to a solution of 4-chloro-2-(1*H*-tetrazol-5-yl)aniline (100.0 mg, 0.511 mmol) and DIPEA (264.2 mg,

2.0 mmol) in anhydrous DMF (3 mL) at 0 °C under N_2. This mixture was stirred at 0 °C for 3 h then at room temperature overnight before heating at 70 °C for 1 h. The reaction mixture was concentrated and extracted with EtOAc (2 × 5 mL) and NaOH (0.1 M, 3 mL). The aqueous layer was acidified with 1M HCl, the precipitate was filtered and washed with water to yield **1-(3,5-bis(trifluoromethyl)phenyl)-3-(4-chloro-2-(1*H*-tetrazol-5-yl)phenyl)urea.** Yellow solid; yield 26% (60 mg, 0.13 mmol); mp: 198 – 201 °C; ^{1}H NMR (500 MHz, [D$_6$]DMSO): δ ppm 10.54 (s, 1H, NH″), 10.11 (s, 1H, NH), 8.37 (d, $^3J_{H6–H5}$ = 9.0 Hz, 1H, H6), 8.19 (s, 2H, H2″ + H6″), 8.00 (s, 1H, H3), 7.64 (s, 1H, H4″), 7.58 (br d, $^3J_{H5–H6}$ = 9.2 Hz, 1H, H5), tetrazole NH not observed; ^{13}C NMR (126 MHz, [D$_6$]DMSO): δ ppm 153.6 (s, Tet-C), 152.1 (s, CO), 141.7 (s, C1″), 136.5 (s, C1), 131.2 (s, C5), 130.7 (q, $^2J_{C-F}$ = 32.8 Hz, C3″ + C5″), 127.8 (s, C3), 126.4 (s, C4), 123.1 (s, C6), 123.3 (q, $^1J_{C-F}$ = 272.9 Hz, 2 × CF_3), 118.2 (br q, $^3J_{C-F}$ = 3.9 Hz, C2″ + C6″), 114.6 - 114.8 (septet, $^3J_{C-F}$ = 3.9 Hz, C4″), 113.6 (s, C2); MS (m/z): [M−H, ^{35}Cl, 90%]$^-$, 449.0, [M−H, ^{37}Cl, 100%]$^-$, 451.0.

1-(3,5-Bis(trifluoromethyl)phenyl)-3-(4-fluoro-2-(1*H*-tetrazol-5-yl)phenyl)urea (NVR-42)

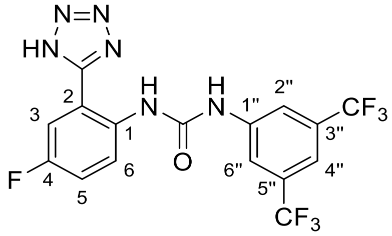

3,5-Bis(trifluoromethyl)phenyl isocyanate (142.4 mg, 0.558 mmol) was added dropwise to a solution of 4-fluoro-2-(1*H*-tetrazol-5-yl)aniline (100.0 mg, 0.558 mmol) and DIPEA (288.5 mg, 2.2 mmol) in anhydrous DMF (3 mL) at 0 °C under N_2. The reaction mixture was stirred at 0 °C for 3 h then at room temperature overnight before heated at 70 °C for 1 h. This mixture was concentrated and extracted with EtOAc (2 × 5 mL) and NaOH (0.1 M, 3 mL). The aqueous layer was acidified with 1M HCl; the precipitate was filtered and washed with water to yield **1-(3,5-bis(trifluoromethyl)phenyl)-3-(4-fluoro-2-(1*H*-tetrazol-5-yl)phenyl)urea.** White solid; yield 23% (55 mg, 0.13 mmol); mp: 195 – 200 °C; ^{1}H NMR (400 MHz, [D$_6$]DMSO): δ ppm 10.48 (s, 1H, NH″), 9.97 (s, 1H, NH), 8.29 (dd, $^3J_{H6–H5}$ = 9.3 Hz, $^4J_{H6–F}$ = 5.4 Hz, 1H, H6), 8.19 (br s, 2H, H2″ + H6″), 7.79 (dd, $^3J_{H3–F}$ = 9.3 Hz, $^4J_{H3–H5}$ = 3.0 Hz, 1H, H3), 7.66 (br s, 1H, H4″), 7.44 (td, $^3J_{H5–F}$ = $^3J_{H5–H6}$ = 8.6, $^4J_{H5–H3}$ = 3.0 Hz, 1H, H5), tetrazole NH not observed; ^{13}C NMR (101 MHz, [D$_6$]DMSO): δ ppm 156.0 (s, Tet-C), 152.3 (s, CO), 141.8 (s, C1″), 133.8 (d, $^4J_{C-F}$ = 2.4 Hz, C1),

130.7 (q, $^2J_{C-F}$ = 32.6 Hz, C3″ + C5″), 124.1 (d, $^3J_{C-F}$ = 8.3 Hz, C6), 123.3 (q, $^1J_{C-F}$ = 272.3 Hz, 2 × CF_3), 118.3 (d, $^2J_{C-F}$ = 22.0 Hz, C3), 118.1 (br q, $^3J_{C-F}$ = 3.9 Hz, C2″ + C6″), 114.9 (s, C2), 114.6 (s, C4″), C4 + C5 in the noise; ^{19}F NMR (376 MHz, [D$_6$]DMSO): δ ppm −61.72 (s, 2 × CF_3), -119.64 (s, F); LCMS (m/z): [M−H, 100%]$^-$, 433.1; RT = 2.81 min.

1-(3,5-Bis(trifluoromethyl)phenyl)-3-(4-bromo-2-(1*H*-tetrazol-5-yl)phenyl)urea (NS3728)

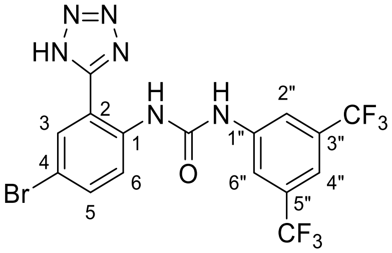

3,5-Bis(trifluoromethyl)phenyl isocyanate (0.24 mL, 1.37 mmol, 1.1 eq) was added dropwise to a stirred solution of 4-bromo-2-(1*H*-tetrazol-5-yl)aniline (300 mg, 1.25 mmol, 1.0 eq) in acetonitrile (20 mL). The reaction mixture was stirred for 24 hours at room temperature, after which a solid precipitated. This solid was filtered and dried to yield **1-(3,5-bis(trifluoromethyl)phenyl)-3-(4-bromo-2-(1*H*-tetrazol-5-yl)phenyl)urea.** White solid; yield 40% (250 mg, 0.505 mmol); decomposition at 263 °C (lit 268 – 271 °C)[4]; ^{1}H NMR (400 MHz, [D$_6$]DMSO): δ ppm 10.56 (s, 1H, NH″), 10.14 (s, 1H, NH), 8.32 (d, $^3J_{H6–H5}$ = 9.0 Hz, 1H, H6), 8.20 (br s, 2H, H2″ + H6″), 8.15 (d, $^4J_{H3–H5}$ = 2.3 Hz, 1H, H3), 7.73 (dd, $^3J_{H5–H6}$ = 9.0 Hz, $^4J_{H5–H3}$ = 2.3 Hz, 1H, H5), 7.68 (br s, 1H, H4″); ^{13}C NMR (101 MHz, [D$_6$]DMSO): δ ppm 152.1 (s, CO), 141.7 (s, C1″), 136.8 (s, C1), 134.0 (s, C3), 130.7 (s, C5), 130.7 (q, $^2J_{C-F}$ = 32.9 Hz, C3″ + C5″), 123.4 (s, C6), 123.3 (q, $^1J_{C-F}$ = 272.9 Hz, 2 × CF_3), 118.3 (br q, $^3J_{C-F}$ = 3.9 Hz, C2″ + C6″), 114.8 (br septet, $^3J_{C-F}$ = 3.9 Hz, C4″), 114.2 (s, C2); ^{19}F NMR (376 MHz, [D$_6$]DMSO): δ ppm −61.70 (s, 2 × CF_3); NMR assignments made with the assistance of COSY, HSQC and HMBC; MS (m/z): [M−H, ^{79}Br, 90%]$^-$, 493.1, [M−H, ^{81}Br, 100%]$^-$, 495.1; HRMS (m/z): [M−H]$^-$ calcd. for $C_{16}H_8ON_6{}^{79}BrF_6$, 492.9858; found 492.9853; error, 1.09 ppm.

1-(3,5-Bis(trifluoromethyl)phenyl)-3-(4-(*tert*-butyl)-2-(1*H*-tetrazol-5-yl)phenyl)urea (NVR-40)

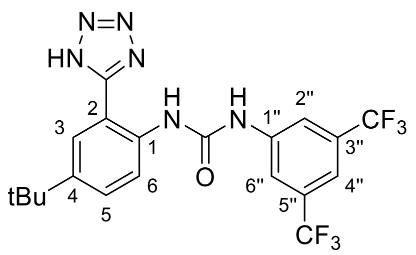

3,5-Bis(trifluoromethyl)phenyl isocyanate (58.7 mg, 0.23 mmol) was added dropwise to a solution of 4-(*tert*-butyl)-2-(1*H*-tetrazol-5-yl)aniline (50.0 mg, 0.23 mmol) and DIPEA (118.9 mg, 0.92 mmol) in anhydrous DMF (3 mL) at 0 °C under N_2. The reaction mixture was heated at 70 ^{0}C for 1 h before being concentrated and extracted with EtOAc (2 × 5 mL) and NaOH (0.1 M, 3 mL). The aqueous layer was acidified with 1M HCl; the precipitate was filtered and washed with water to yield **1-(3,5-bis(trifluoromethyl)phenyl)-3-(4-(*tert*-butyl)-2-(1*H*-tetrazol-5-yl)phenyl)urea.** Yellow solid; yield 8% (15 mg, 0.032 mmol); decomposition at 204 °C; ^{1}H NMR (400 MHz, [D$_6$]DMSO): δ ppm 10.45 (s, 1H, NH″), 10.01 (s, 1H, NH), 8.23 (d, $^3J_{H6–H5}$ = 8.9 Hz, 1H, H6), 8.20 (br s, 2H, H2″ + H6″), 7.93 (d, $^4J_{H3–H5}$ = 2.3 Hz, 1H, H3), 7.65 (br s, 1H, H4″), 7.60 (dd, $^3J_{H5–H6}$ = 8.8 Hz, $^4J_{H5–H3}$ = 2.3 Hz, 1H, H5), 1.34 (s, 9H, ^{t}Bu), tetrazole NH not observed; ^{13}C NMR (101 MHz, [D$_6$]DMSO): δ ppm 152.3 (s, CO), 145.4 (s, C4), 141.9 (s, C1″), 135.0 (s, C1), 130.7 (q, $^2J_{C\text{-}F}$ = 32.8 Hz, C3″ + C5″), 128.8 (s, C5), 125.2 (s, C3), 121.5 (s, C6), 123.3 (q, $^1J_{C\text{-}F}$ = 272.9 Hz, 2 × CF_3), 118.0 (br q, $^3J_{C\text{-}F}$ = 3.9 Hz, C2″+C6″), 114.5 (br septet, br q, $^3J_{C\text{-}F}$ = 3.9 Hz, C4″), 34.3 (s, *C*(CH_3)$_3$), 31.0 (s, 3 × CH_3), C2 + tetrazole carbon not observed; ^{19}F NMR (376 MHz, [D$_6$]DMSO): δ ppm −61.71 (s, 2 × CF_3); MS (m/z): [M−H, 100%]$^-$, 471.2; HRMS (m/z): [M−H]$^-$ calcd. for $C_{20}H_{17}ON_6F_6$, 471.1374; found 471.1372; error, –0.32 ppm.

1-(3,5-Bis(trifluoromethyl)phenyl)-3-(4-chloro-5-fluoro-2-(1*H*-tetrazol-5-yl)phenyl)urea (NVR-131)

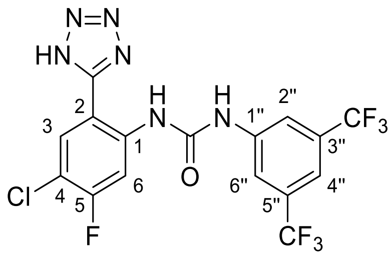

3,5-Bis(trifluoromethyl)phenyl isocyanate (740 mg, 2.9mmol) was added dropwise to a solution of 4-chloro-5-fluoro-2-(1*H*-tetrazol-5-yl)aniline (300 mg, 1.47mmol) in a mixture of anhydrous THF (3 mL) and toluene (5 mL) at room temperature under N_2. This mixture was stirred overnight at room temperature; then stored in a refrigerator overnight. The white solid that precipitated was filtered and washed with DCM to yield **1-(3,5-bis(trifluoromethyl)phenyl)-3-(4-chloro-5-fluoro-2-(1*H*-tetrazol-5-yl)phenyl)urea.** White solid; yield 34% (223 mg, 0.476 mmol); mp: 202 – 203 °C; ^{1}H NMR (500 MHz, [D$_6$]DMSO): δ ppm 10.62 (s, 1H, NH), 10.37 (s, 1H, NH″), 8.40 (d, $^{3}J_{H6–F}$ = 12.8 Hz, 1H, H6), 8.15 (s, 2H, H2″ + H6″), 8.11 (d, $^{3}J_{H3–F}$ = 7.9 Hz, 1H, H3), 7.59 (s, 1H, H4″), tetrazole NH not observed; ^{13}C NMR (126 MHz, [D$_6$]DMSO): δ ppm 157.4 (d, $^{1}J_{C-F}$ = 248.4 Hz, C5), 153.2 (s, Tet-C), 151.8 (s, CO), 141.5 (s, 1″), 138.5 (d, $^{3}J_{C-F}$ = 11.7 Hz, C1), 130.6 (q, $^{2}J_{C-F}$ = 32.9 Hz, C3″ + C5″), 129.9 (s, C3), 123.3 (q, $^{1}J_{C-F}$ = 272.9 Hz, 2 × CF_3), 117.9 (br q, $^{3}J_{C-F}$ = 4.0 Hz, C2″ + C6″), 114.9 (br septet, $^{3}J_{C-F}$ = 3.9 Hz, C4″), 112.7 (d, $^{2}J_{C-F}$ = 18.6 Hz, C4), 108.8 (d, $^{4}J_{C-F}$ = 2.0 Hz, C2), 108.5 (d, $^{2}J_{C-F}$ = 27.4 Hz, C6); MS (m/z): [M−H, ^{35}Cl, 100%]$^-$, 467.1, [M−H, ^{37}Cl, 50%]$^-$, 469.1.

1-(3-(1*H*-Tetrazol-5-yl)-5-(trifluoromethyl)phenyl)-3-(3,5-bis(trifluoromethyl)phenyl)urea (NVR-135)

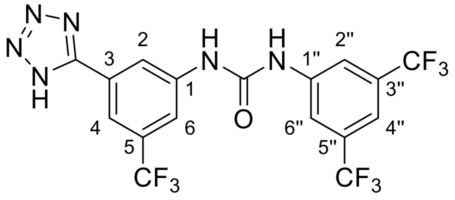

3,5-Bis(trifluoromethyl)phenyl isocyanate (550 mg, 2.0 mmol) was added dropwise to a solution of 3-(1*H*-tetrazol-5-yl)-5-(trifluoromethyl)aniline (250 mg, 1.1 mmol) in a mixture of

anhydrous THF (3 mL) and toluene (5 mL) at room temperature under N_2. This mixture was stirred overnight at room temperature; then stored in a refrigerator overnight. The creamy solid that precipitated was filtered and washed with DCM to yield **1-(3-(1*H*-tetrazol-5-yl)-5-(trifluoromethyl)phenyl)-3-(3,5-bis(trifluoromethyl)phenyl)urea.** Cream solid; yield 85% (490 mg, 1.01 mmol); mp: 190 – 191 °C; ^{1}H NMR (400 MHz, [D$_6$]DMSO): δ ppm 9.74 (s, 1H, NH), 9.70 (s, 1H, NH″), 8.47 (s, 1H, H6), 8.17 (s, 2H, H2″ + H6″), 8.12 (s, 1H, H2), 7.97 (s, 1H, H4), 7.67 (s, 1H, H4″), tetrazole NH not observed; ^{13}C NMR (101 MHz, [D$_6$]DMSO): δ ppm 163.0 (s, Tet-C), 152.5 (s, CO), 141.4 (s, C1), 141.2 (s, 1C , C1″), 130.6 (q, $^{2}J_{C-F}$ = 32.9 Hz, C5), 130.7 (q, $^{2}J_{C-F}$ = 32.0 Hz, C3″ + C5″), 120.3 (s, C2), 123.7 (q, $^{1}J_{C-F}$ = 271.9 Hz, 5-CF_3), 123.3 (q, $^{1}J_{C-F}$ = 274.9 Hz, 2 × CF_3), 118.4 (br q, $^{3}J_{C-F}$ = 3.9 Hz, C2″ + C6″), 117.0 (q, $^{3}J_{C-F}$ = 3.9 Hz, C4), 116.8 (q, $^{3}J_{C-F}$ = 3.9 Hz, C6), 114.9 (br septet, $^{3}J_{C-F}$ = 3.9 Hz, C4″), C3 not observed; ^{19}F NMR (376 MHz, [D$_6$]DMSO): δ ppm −61.68 (s, 2 × CF_3), -61.70 (s, CF_3); MS (m/z): [M−H, 100%]$^-$, 483.2.

1-(3,5-Bis(trifluoromethyl)phenyl)-3-(3-bromo-5-(1*H*-tetrazol-5-yl)phenyl)urea (NVR-139)

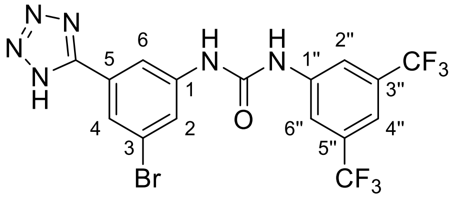

3,5-Bis(trifluoromethyl)phenyl isocyanate (415 mg, 1.6 mmol) was added dropwise to a solution of 3-bromo-5-(1*H*-tetrazol-5-yl)aniline (200 mg, 0.8mmol) in a mixture of anhydrous THF (3 mL) and toluene (5 mL) at room temperature under N_2. This mixture was stirred overnight at room temperature; then stored in a refrigerator overnight. The yellow solid that precipitated was filtered and washed with DCM to yield **1-(3,5-bis(trifluoromethyl)phenyl)-3-(3-bromo-5-(1*H*-tetrazol-5-yl)phenyl)urea.** Brown solid; yield 37% (150 mg, 0.303 mmol); mp: 194 – 199 °C; ^{1}H NMR (500 MHz, [D$_6$]DMSO): δ ppm 10.96 (br s, 1H, NH), 10.89 (br s, 1H, NH″), 8.30 (s, 2H, H2″ + H6″), 8.21 (br s, 1H, H6), 8.04 (br s, 1H, H4), 8.02 (br s, 1H, H2), 7.82 (s, 1H, H4″), tetrazole NH not observed; ^{13}C NMR (126 MHz, [D$_6$]DMSO): δ ppm 153.5 (s, Tet-C), 152.1 (s, CO), 141.8 (s, C1″), 136.9 (s, C1), 134.0 (s, C4), 130.7 - 130.9 (s, C6), 130.7 (q, $^{2}J_{C-F}$ = 32.3 Hz, C3″ + C5″), 123.3 (s, C2), 123.3 (q, $^{1}J_{C-F}$ = 272.9 Hz, 2 × CF_3), 118.2 (br q, $^{3}J_{C-F}$ = 3.9 Hz, C2″ + C6″), 114.7 (br septet, $^{3}J_{C-F}$ = 3.9 Hz, C4″), 114.2 (s, C3), 114.1 (s, C5); ^{19}F NMR (471

MHz, [D$_6$]DMSO): δ ppm −61.54 (s, 2 × CF_3); MS (m/z): [M−H, ^{79}Br, 100%]$^-$, 493.1, [M−H, ^{81}Br, 95%]$^-$, 495.1.

1-(2-(1*H*-Tetrazol-5-yl)-5-(trifluoromethyl)phenyl)-3-(3,5-bis(trifluoromethyl)phenyl)-thiourea (NVR-134)

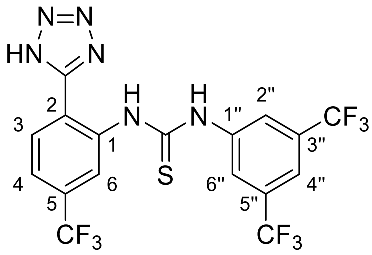

3,5-Bis(trifluoromethyl)phenyl isothiocyanate (4.70 mg, 1.7mmol) was added dropwise to a solution of 5-trifluoromethyl-2-(1*H*-tetrazol-5-yl)aniline (200 mg, 1.0 mmol) in a mixture of anhydrous THF (3 mL) and toluene (5 mL) at room temperature under N_2. This mixture was stirred overnight at room temperature; then stored in a refrigerator overnight. The white solid that precipitated was filtered and washed with DCM to yield **1-(2-(1*H*-tetrazol-5-yl)-5-(trifluoromethyl)phenyl)-3-(3,5-bis(trifluoromethyl)phenyl)thiourea.** White solid; yield 61% (265 mg, 0.530 mmol); mp: 180 – 181 °C; ^{1}H NMR (400 MHz, [D$_6$]DMSO): δ ppm 10.90 (s, 1H, NH), 10.55 (s, 1H, NH″), 8.27 (s, 2H, H2″ + H6″), 8.17 (br s, 1H, H6), 8.12 (d, $^3J_{H3–H4}$ = 8.2 Hz, 1H, H3), 7.85 (br s, 1H, H4″), 7.82 (dd, $^3J_{H4–H3}$ = 8.3 Hz, $^4J_{H4–H6}$ = 1.3 Hz, 1H, H4), tetrazole NH not observed; ^{13}C NMR (101 MHz, [D$_6$]DMSO): δ ppm 180.6 (s, CS), 141.2 (s, C1″), 138.0 (s, C1), 130.6 (br s, C3), 130.4 (br q, $^2J_{C\text{-}F}$ = 33.3 Hz, C5, C3″ + C5″), 124.6 (br q, $^3J_{C\text{-}F}$ = 3.9 Hz, C4), 123.5 (br q, $^3J_{C\text{-}F}$ = 3.9 Hz, C2″ + C6″), 122.7 (br q, $^3J_{C\text{-}F}$ = 3.9 Hz, C6), 123.2 (q, $^1J_{C\text{-}F}$ = 273.5 Hz, 3 × CF_3), 117.5 (br septet, $^3J_{C\text{-}F}$ = 3.9 Hz, C4″), C2 and tetrazole carbon not observed; ^{19}F NMR (376 MHz, [D$_6$]DMSO): δ ppm −61.55 (s, 2 × CF_3), -61.63 (s, CF_3); MS (m/z): [M−H, 100%]$^-$, 499.1.

1-(2-(1*H*-Tetrazol-5-yl)-3,5-bis(trifluoromethyl)phenyl)-3-(3,5-bis(trifluoromethyl)phenyl)thiourea (NVR-105)

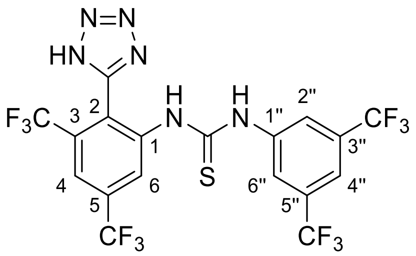

3,5-Bis(trifluoromethyl)phenyl isothiocyanate (337 mg, 1.2 mmol) was added dropwise to a solution of 2-(1*H*-tetrazol-5-yl)-3,5-bis(trifluoromethyl)aniline (185 mg, 1.0 mmol) in a mixture of anhydrous THF (3 mL) and toluene (5 mL) at room temperature under N_2. This mixture was stirred overnight at room temperature; then stored in a refrigerator overnight. The creamy solid that precipitated was filtered and washed with DCM to yield **1-(2-(1*H*-tetrazol-5-yl)-3,5-bis(trifluoromethyl)phenyl)-3-(3,5-bis(trifluoromethyl)phenyl)thiourea.** Cream solid; yield 44% (157 mg, 0.276 mmol); mp: 161 – 162 °C; ^{1}H NMR (500 MHz, [D$_6$]DMSO): δ ppm 10.48 (br s, 1H, NH″), 9.97 (br s, 1H, NH), 8.41 (s, 1H, H6), 8.22 (s, 1H, H4″), 8.10 (s, 2H, H2″ + H6″), 7.84 (s, 1H, H4), tetrazole NH not observed; ^{19}F NMR (471 MHz, [D$_6$]DMSO): δ ppm −57.85 (s, CF_3), -61.54 (s, 2 × CF_3), -61.75 (s, CF_3); MS (m/z): [M−H, 100%]$^-$, 567.0.

1-(3-(1*H*-Tetrazol-5-yl)-5-(trifluoromethyl)phenyl)-3-(3,5-bis(trifluoromethyl)phenyl)-thiourea (NVR-101)

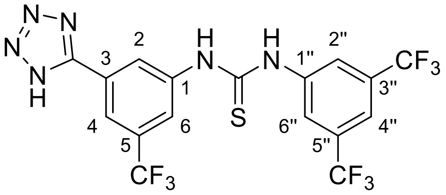

3,5-Bis(trifluoromethyl)phenyl isothiocyanate (500 mg, 2.0 mmol) was added dropwise to a solution of 3-(1*H*-tetrazol-5-yl)-5-(trifluoromethyl)aniline (200 mg, 1.0 mmol) in a mixture of anhydrous THF (3 mL) and toluene (5 mL) at room temperature under N_2. This mixture was stirred overnight at room temperature; then stored in a refrigerator overnight. The white solid that precipitated was filtered and washed with DCM to yield **1-(3-(1*H*-tetrazol-5-yl)-5-**

(trifluoromethyl)phenyl)-3-(3,5-bis(trifluoromethyl)phenyl)thiourea. White solid; yield 86% (370 mg, 0.740 mmol); mp: 163 – 164 °C; ^{1}H NMR (500 MHz, [D$_6$]DMSO): δ ppm 10.67 (s, 1H, NH), 10.60 (s, 1H, NH″), 8.45 (s, 1H, H4), 8.23 (br s, 2H, H2″ + H6″), 8.13 (s, 2H, H2 + H6), 7.85 (br s, 1H, H4″), tetrazole NH not observed; ^{13}C NMR (126 MHz, [D$_6$]DMSO): δ ppm 180.5 (s, CS), 141.3 (s, C1), 141.0 (s, C1″), 130.3 (q, $^{2}J_{C\text{-}F}$ = 32.3 Hz, C5), 130.3 (q, $^{2}J_{C\text{-}F}$ = 32.6 Hz, C3″ + C5″), 125.7 (s, C2), 124.0 (br q, $^{3}J_{C\text{-}F}$ = 3.9 Hz, C2″ + C6″), 122.6 (br q, $^{3}J_{C\text{-}F}$ = 3.9 Hz, C4), 123.2 (q, $^{1}J_{C\text{-}F}$ = 272.9 Hz, 3 × CF_3), 119.3 (s, C6), 117.6 (br septet, $^{3}J_{C\text{-}F}$ = 3.9 Hz, C4″), C3 and tetrazole carbon not observed; ^{19}F NMR (471 MHz, [D$_6$]DMSO): δ ppm −61.53 (s, 2 × CF_3), -61.57 (s, CF_3); MS (m/z): [M−H, 100%]$^-$, 499.1.

1-(4-(1*H*-Tetrazol-5-yl)-3-(trifluoromethyl)phenyl)-3-(3,5-bis(trifluoromethyl)phenyl)-thiourea (NVR-96)

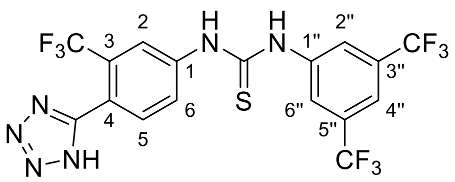

3,5-Bis(trifluoromethyl)phenyl isothiocyanate (500 mg, 2.0 mmol) was added dropwise to a solution of 4-(1*H*-tetrazol-5-yl)-3-(trifluoromethyl)aniline (200 mg, 1.0 mmol) in a mixture of anhydrous THF (3 mL) and toluene (5 mL) at room temperature under N_2. This mixture was stirred overnight at room temperature; then stored in a refrigerator overnight. The yellow solid that precipitated was filtered and washed with DCM to yield **1-(4-(1*H*-tetrazol-5-yl)-3-(trifluoromethyl)phenyl)-3-(3,5-bis(trifluoromethyl)phenyl)thiourea.** Yellow solid; yield 29% (128 mg, 0.256 mmol); mp: 166 – 167 °C; ^{1}H NMR (400 MHz, [D$_6$]DMSO): δ ppm 11.06 (br s, 2H, NH + NH″), 8.31 (br s, 2H, H2″ + H6″), 8.26 (br s, 1H, H2), 8.02 (br d, $^{3}J_{H5\text{–}H6}$ = 7.8 Hz, 1H, H5), 7.87 (br s, 1H, H4″), 7.80 (br d, $^{3}J_{H6\text{–}H5}$ = 8.8 Hz, 1H, H6), tetrazole NH not observed; ^{13}C NMR (101 MHz, [D$_6$]DMSO): δ ppm 180.5 (s, CS), 141.3 (s, C1), 141.0 (s, C1″), 130.3 (q, $^{2}J_{C\text{-}F}$ = 32.8 Hz, C3 + C3″ + C5″), 125.7 (s, C6), 124.5 (s, C5), 124.0 (br q, $^{3}J_{C\text{-}F}$ = 3.9 Hz, C2), 119.3 (br q, $^{3}J_{C\text{-}F}$ = 3.9 Hz, C2″ + C6″), 117.6 (br septet, $^{3}J_{C\text{-}F}$ = 3.9 Hz, C4″), 3 × CF_3, C4 and tetrazole carbon in the noise; ^{19}F NMR (376 MHz, [D$_6$]DMSO): δ ppm −61.53 (s, 2 × CF_3), -61.57 (s, CF_3); MS (m/z): [M−H, 100%]$^-$, 499.1.

(3-(3-(3,5-Bis(trifluoromethyl)phenyl)thioureido)phenyl)boronic acid (NVR-141)

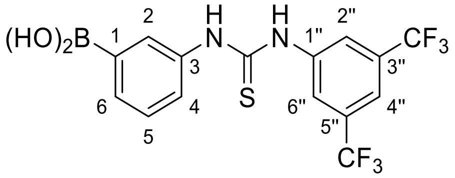

3,5-Bis(trifluoromethyl)phenyl isothiocyanate (1500 mg, 5.8mmol) was added dropwise to a solution of (3-aminophenyl)boronic acid (200 mg, 1.0 mmol) in a mixture of anhydrous THF (3 mL) and toluene (5 mL) at room temperature under N_2. This mixture was stirred overnight at room temperature; then stored in a refrigerator overnight. The white solid that precipitated was filtered and washed with DCM to yield **(3-(3-(3,5-bis(trifluoromethyl)phenyl)thioureido)phenyl)boronic acid.** White solid; yield 63% (300 mg, 0.735 mmol); mp: 119 – 121 °C; ^{1}H NMR (400 MHz, [D$_6$]DMSO): δ ppm 10.27 (s, 1H, NH″), 10.14 (s, 1H, NH), 8.24 (s, 2H, B(OH)$_2$, 8.11 (s, 2H, H2″ + H6″), 7.79 (s, 1H, H2), 7.73 (s, 1H, H4″), 7.63 (d, $^{3}J_{H6–H5}$ = 7.3 Hz, 1H, H6), 7.49 (br d, $^{3}J_{H4–H5}$ = 8.8 Hz, 1H, H4), 7.35 (t, $^{3}J_{H5–H4}$ = $^{3}J_{H5–H6}$ =7.9 Hz, 1H, H5); ^{13}C NMR (101 MHz, [D$_6$]DMSO): δ ppm 179.9 (s, CS), 141.9 (s, C1″), 137.8 (s, C3), 131.3 (s, C6), 130.1 (s, C5), 129.9 (q, $^{2}J_{C\text{-}F}$ = 32.3 Hz, C3″ + C5″), 127.9 (s, C2), 126.4 (s, C4), 123.6 (br q, $^{3}J_{C\text{-}F}$ = 3.9 Hz, C2″ + C6″), 123.3 (q, $^{1}J_{C\text{-}F}$ = 272.9 Hz, 2 × CF_3), 116.7 (s, $^{3}J_{C\text{-}F}$ = 3.9 Hz, C4″), C1 not observed; ^{19}F NMR (376 MHz, [D$_6$]DMSO): δ ppm −61.52 (s, 2 × CF_3).

3-(3-(3,5-Bis(trifluoromethyl)phenyl)ureido)-2-naphthoic acid (NVR-148)

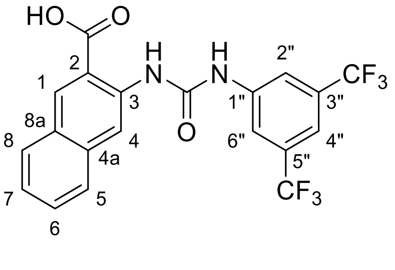

3,5-Bis(trifluoromethyl)phenyl isocyanate (0.17 mL, 0.96 mmol, 1.2 eq) was added dropwise to a stirred solution of 3-amino-2-naphthoic acid (150 mg, 0.80 mmol, 1.0 eq) in acetonitrile (25 mL). The reaction was stirred for 16 hours at room temperature. The precipitate was filtered and then washed with acetonitrile, before being dried to yield **3-(3-(3,5-bis(trifluoromethyl)phenyl)ureido)-2-naphthoic acid.** White solid; yield 63% (224 mg, 0.506 mmol); decomposition at 230 °C; ^{1}H NMR (400 MHz, [D$_6$]DMSO): δ ppm 13.80 (br s, 1H,

COOH), 10.63 (s, 1H, NH), 10.55 (s, 1H, NH″), 8.85 (s, 1H, H4), 8.70 (s, 1H, H1), 8.25 (br s, 2H, H2″ + H6″), 8.00 (br d, $^{3}J_{H8–H7}$ = 8.0 Hz, 1H, H8), 7.89 (br d, $^{3}J_{H5–H6}$ = 8.0 Hz, 1H, H5), 7.65 (br s, 1H, H4″), 7.60 (br dd, $^{3}J_{H6–H5}$ = 8.0 Hz, $^{3}J_{H6–H7}$ = 7.5 Hz, 1H, H6), 7.45 (br t, $^{3}J_{H7–H6}$ = $^{3}J_{H7–H8}$ = 7.5 Hz, 1H, H7); ^{13}C NMR (101 MHz, [D$_6$]DMSO): δ ppm 169.6 (s, COOH), 152.2 (s, CO), 142.1 (s, C1″), 136.6 (s, C3), 135.7 (s, C4a), 133.0 (s, C1), 130.7 (q, $^{2}J_{C-F}$ = 32.3 Hz, C3″ + C5″), 129.2 (s, C6), 129.0 (s, C8), 127.6 (s, C8a), 127.0 (s, C5), 125.2 (s, C7), 123.4 (q, $^{1}J_{C-F}$ = 272.2 Hz, 2 × CF_3), 117.9 (br q, $^{3}J_{C-F}$ = 3.9 Hz, C2″ + C6″), 116.9 (s, C2), 116.0 (s, C4), 114.4 (br septet, $^{3}J_{C-F}$ = 3.9 Hz, C4″); ^{19}F NMR (376 MHz, [D$_6$]DMSO): δ ppm −61.73 (s, 2 × CF_3); NMR assignments made with the assistance of COSY, HSQC and HMBC; HRMS (m/z): $[M+H]^+$ calcd. for $C_{17}H_{13}O_4N_2F_6$, 423.0774; found, 423.0766; error, 1.3 ppm.

2-(3-(4-Chloro-3-(trifluoromethyl)phenyl)ureido)benzoic acid (NVR-13)

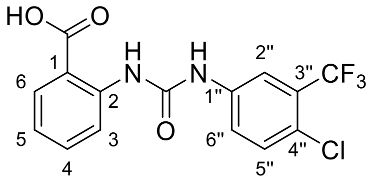

Anthranilic acid (120.0 mg, 0.875 mmol) and 4-chloro-3-(trifluoromethyl)phenyl isocyanate (154.9 mg, 0.699 mmol) were suspended in THF (3 mL). This mixture was stirred at room temperature for 2 h and then stored in a refrigerator overnight. The white solid that precipitated was filtered and purified by flash column chromatography (2% methanol in DCM) to yield **2-(3-(4-chloro-3-(trifluoromethyl)phenyl)ureido)benzoic acid.** White solid; yield 9% (27.3 mg, 0.0761 mmol); mp: 197 – 200 °C; ^{1}H NMR (400 MHz, [D$_6$]DMSO): δ ppm 10.54 (s, 1H, NH), 10.29 (s, 1H, NH″), 8.37 (dd, $^{3}J_{H3–H4}$ = 8.5 Hz, $^{4}J_{H3–H5}$ = 0.8 Hz, 1H, H3), 8.11 (d, $^{4}J_{H2''–H6''}$ = 2.5 Hz, 1H, H2″), 7.97 (dd, $^{3}J_{H6–H5}$ = 8.0 Hz, $^{4}J_{H6–H4}$ = 1.6 Hz, 1H, H6), 7.77 (dd, $^{3}J_{H6''–H5''}$ = 8.9 Hz, $^{4}J_{H6''–H2''}$ = 2.5 Hz, 1H, H6″), 7.62 (d, $^{3}J_{H5''–H6''}$ = 8.8 Hz, 1H, H5″), 7.56 (ddd, $^{3}J_{H4–H3}$ = 8.6 Hz, $^{3}J_{H4–H5}$ = 7.2 Hz, $^{4}J_{H4–H6}$ = 1.6 Hz , 1H, H4), 7.07 (ddd, $^{3}J_{H5–H6}$ =8.0 Hz, $^{3}J_{H5–H4}$ 7.2 Hz, $^{4}J_{H5–H3}$ = 1.0 Hz, 1H, H5), COOH not observed; ^{13}C NMR (101 MHz, [D$_6$]DMSO): δ ppm 169.5 (s, COOH), 152.1 (s, CO), 141.8 (s, C2), 139.4 (s, C1″), 133.9 (s, C4), 132.0 (s, C6), 131.1 (s, C6″), 126.7 (q, $^{2}J_{C-F}$ = 30.8 Hz, C3″), 123.2 (br s, C5″), 122.5 (br q, $^{3}J_{C-F}$ = 1.5 Hz, C4″), 121.4 (s, C5), 119.8 (s, C3), 117.1 (q, $^{3}J_{C-F}$ = 5.5 Hz, C2″), 115.6 (s, C1), CF_3 in the noise; ^{19}F NMR (376 MHz, [D$_6$]DMSO): δ ppm −61.50 (s, CF_3); LCMS (m/z): [M−H, ^{35}Cl, 100%]$^-$, 357.0, [M−H, ^{37}Cl, 40%]$^-$, 359.0 RT = 2.73

min; HRMS (m/z): [M−H]⁻ calcd. for $C_{15}H_9O_3N_2{}^{35}ClF_3$, 357.0260; found, 357.0259; error, 0.20 ppm.

4-Chloro-2-(3-(4-chloro-3-(trifluoromethyl)phenyl)ureido)benzoic acid (NVR-6)

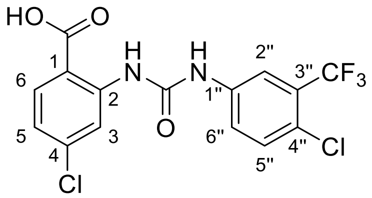

2-Amino-4-chlorobenzoic acid (120.0 mg, 0.699 mmol) and 4-chloro-3-(trifluoromethyl)phenyl isocyanate (154.9 mg, 0.699 mmol) were suspended in THF (3 mL). This mixture was stirred at room temperature for 2 h, then stored in a refrigerator overnight. The white solid that precipitated was filtered and washed with DCM to yield **4-chloro-2-(3-(4-chloro-3-(trifluoromethyl)phenyl)ureido)benzoic acid.** White solid; yield 12% (32 mg, 0.081 mmol); mp: 214 °C (lit 187 - 188°C)[5]; ^{1}H NMR (500 MHz, [D$_6$]DMSO): δ ppm 13.74 (br s, 1H, COOH), 10.67 (s, 1H, NH), 10.40 (s, 1H, NH″), 8.52 (d, $^{4}J_{H3–H5}$ = 2.1 Hz, 1H, H3), 8.09 (d, $^{4}J_{H2″–H6″}$ = 2.6 Hz, 1H, H2″), 7.96 (d, $^{3}J_{H6–H5}$ = 8.5 Hz, 1H, H6), 7.77 (dd, $^{3}J_{H6″–H5″}$ =8.8 Hz, $^{4}J_{H6″–H2″}$ = 2.5 Hz, 1H, H6″) , 7.62 (d, $^{3}J_{H5″–H6″}$ = 8.9 Hz, 1H, H5″), 7.12 (dd, $^{3}J_{H5–H6}$ = 8.5 Hz, $^{4}J_{H5–H3}$ = 2.1 Hz, 1H, H5); ^{13}C NMR (126 MHz, [D$_6$]DMSO): δ ppm 168.8 (s, COOH), 151.9 (s, CO), 143.0 (s, C4), 139.1 (s, C2), 138.5 (s, C1″), 132.8 (s, C6), 132.0 (s, C6″), 126.7 (q, $^{2}J_{C-F}$ = 30.3 Hz, C3″), 123.3 (s, C5″), 122.8 (br q, $^{3}J_{C-F}$ = 1.5 Hz, C4″), 121.2 (s, C5), 122.8 (q, $^{1}J_{C-F}$ = 273.1 Hz, CF$_3$), 118.8 (s, C3), 117.2 (q, $^{3}J_{C-F}$ = 5.9 Hz, C2″), 114.1 (s, C1); IR: 3359 (N-H), 3129 (CO<u>OH</u>), 1698 (<u>CO</u>OH), 1670 (NH-<u>C=O</u>), 1320 (C-F), cm^{-1}; MS (m/z): [M−H, ^{35}Cl^{35}Cl, 100%]⁻, 391.1, [M−H, ^{35}Cl^{37}Cl, 75%]⁻, 393.1, [M−H, ^{37}Cl^{37}Cl, 10%]⁻, 395.1; HRMS (m/z): [M−H]⁻ calcd. for $C_{15}H_8O_3N_2{}^{35}Cl_2F_3$, 390.9870; found 390.9870; error, 0.11 ppm.

4-(*tert*-Butyl)-2-(3-(4-chloro-3-(trifluoromethyl)phenyl)ureido)benzoic acid (NVR-158)

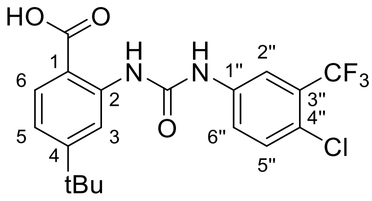

4-Chloro-3-(trifluoromethyl)phenyl isocyanate (1.15g, 5.2 mmol, 1 eq) was added to a stirred solution of 4-*tert*-butylanthranilic acid (1.00 g, 5.2 mmol, 1 eq) in THF (20 mL). The reaction mixture was stirred at room temperature for 2 hours. Solvent was removed *in vacuo* before the resultant solid residue was washed with minimal DCM to yield **4-(*tert*-butyl)-2-(3-(4-chloro-3-(trifluoromethyl)phenyl)ureido)benzoic acid**. Orange solid; yield 79% (1.690 g, 4.163 mmol); mp: 163 – 165 °C; ^{1}H NMR (500 MHz, [D$_6$]DMSO): δ ppm 13.32 (br s, 1H, COOH), 10.54 (s, 1H, NH), 10.26 (s, 1H, NH″), 8.50 (d, $^{4}J_{H3\text{-}H5}$ = 1.5 Hz, 1H, H3), 8.06 (d, $^{4}J_{H2''\text{-}H6''}$ = 2.1 Hz, 1H, H2″), 7.89 (d, $^{3}J_{H6\text{-}H5}$ = 8.4 Hz, 1H, H6), 7.82 (dd, $^{3}J_{H6''\text{-}H5''}$ = 8.7 Hz, $^{4}J_{H6''\text{-}H2''}$ = 2.1 Hz, 1H, H6″), 7.62 (d, $^{3}J_{H5''\text{-}H6''}$ = 8.9 Hz, 1H, H5″), 7.11 (br d, $^{3}J_{H5\text{-}H6}$ = 8.4 Hz, 1H, H5), 1.29 (s, 9H, 3 × Me); ^{13}C NMR (126 MHz, [D$_6$]DMSO): δ ppm 169.4 (s, COOH), 157.0 (s, C4), 152.2 (s, CO), 141.8 (s, C2), 139.5 (s, C1″), 132.0 (s, C6), 130.8 (s, C5″), 127.0 (s, C6″), 126.6 (q, $^{2}J_{C\text{-}F}$ = 30.8 Hz, C3″), 123.2 (br s, C4″), 122.8 (q, $^{1}J_{C\text{-}F}$ = 272.9 Hz, CF$_3$), 118.6 (s, C5), 117.1 (br q, $^{3}J_{C\text{-}F}$ = 4.9 Hz, C2″), 116.4 (s, C3), 112.8 (s, C1), 35.0 (s, *C*(CH$_3$)$_3$), 30.7 (s, 3 × Me); MS (m/z): [M+H, ^{35}Cl, 100%]$^+$, 415.1, [M+H, ^{37}Cl, 32%]$^+$, 417.1; HRMS (m/z): [M+H]$^+$ calcd. for $C_{19}H_{19}O_3N_2{}^{35}ClF_3$, 415.1031; found, 415.1024; error, –1.64 ppm.

2-(3-(4-Chloro-3-(trifluoromethyl)phenyl)ureido)-5-fluorobenzoic acid (NVR-10)

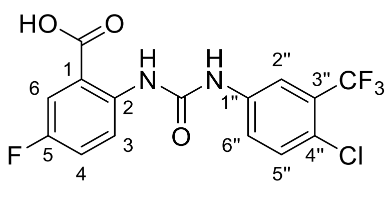

2-Amino-5-fluorobenzoic acid (120.0 mg, 0.77 mmol) and 4-chloro-3-(trifluoromethyl)phenyl isocyanate (171.2 mg, 0.773 mmol) were suspended in THF (3 mL). This mixture was stirred at room temperature for 2 h; then stored in a refrigerator overnight. The white solid that precipitated was filtered and washed with DCM to yield **2-(3-(4-chloro-3-(trifluoromethyl)phenyl)ureido)-5-fluorobenzoic acid.** White solid; yield 10% (28.4 mg,

0.0754 mmol); mp: 195 – 199 °C; ^{1}H NMR (500 MHz, [D$_6$]DMSO): δ ppm 13.83 (br s, 1H, COOH), 10.36 (s, 1H, NH″), 10.28 (s, 1H, NH), 8.38 (dd, $^{3}J_{H3–H4}$ = 9.3 Hz, $^{4}J_{H3''–F}$ = 5.2 Hz, 1H, H3), 8.09 (d, $^{4}J_{H2''–H6''}$ = 2.6 Hz, 1H, H2″), 7.75 (dd, $^{3}J_{H6''–H5''}$ = 8.8 Hz, $^{4}J_{H6''–H2''}$ = 2.5 Hz, 1H, H6″), 7.67 (dd, $^{3}J_{H6–F}$ = 9.3 Hz, $^{4}J_{H6–H4}$ = 3.2 Hz, 1H, H6), 7.61 (d, $^{3}J_{H5''–H6''}$ = 8.7 Hz, 1H, H5″), 7.45 (ddd, $^{3}J_{H4–H3''}$ = 9.3 Hz, $^{3}J_{H4–F}$ = 7.9 Hz, $^{4}J_{H4–H6}$ = 3.2 Hz, 1H, H4); ^{13}C NMR (126 MHz, [D$_6$]DMSO): δ ppm 168.4 (s, COOH), 156.0 (d, $^{1}J_{C-F}$ = 239.6 Hz, C5), 152.1 (s, CO), 139.4 (s, C1″), 138.2 (d, $^{4}J_{FC}$ = 2.0 Hz, C2), 132.0 (s, C5″), 126.7 (q, $^{2}J_{C-F}$ = 30.3 Hz, C3″), 123.1 (s, C6″), 122.5 (br q, $^{3}J_{C-F}$ = 1.5 Hz, C4″), 122.0 (d, $^{3}J_{C-F}$ = 7.3 Hz, C3), 120.9 (d, $^{2}J_{C-F}$ = 22.0 Hz, C4), 122.8 (q, $^{1}J_{C-F}$ = 272.9 Hz, CF_3), 117.2 (d, $^{3}J_{C-F}$ = 6.8 Hz, C1), 117.0 (q, $^{3}J_{C-F}$ = 5.7 Hz, C2″), 116.5 (d, $^{2}J_{C-F}$ = 23.5 Hz, C6); ^{19}F NMR (471 MHz, [D$_6$]DMSO): δ ppm −61.54 (s, CF_3), -121.09 (ddd, $^{3}J_{F-H6}$ = 9.2 Hz, $^{3}J_{F-H4}$ = 8.2 Hz, $^{4}J_{F-H3}$ = 5.1 Hz, F); MS (m/z): [M−H, ^{35}Cl, 100%]$^{-}$, 375.1, [M−H, ^{37}Cl, 30%]$^{-}$, 377.1.

5-Chloro-2-(3-(3-(trifluoromethyl)phenyl)ureido)benzoic acid (NVR-162)

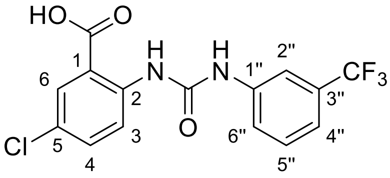

3-Trifluorophenyl isocyanate (1.87 g, 9.99 mmol, 1.25 eq) was added to a stirred solution of 5-chloroanthranilic acid (1.37 g, 7.99 mmol, 1 eq) in toluene (50 mL). The reaction mixture was stirred at room temperature for 72 h. Solvent was removed *in vacuo*, and the resultant solid was purified by flash column chromatography using a 3:1 DCM:MeOH solvent system. Pure fractions were combined and solvent removed *in vacuo* to yield the desired product. Impure fractions were also combined, solvent removed *in vacuo* and the resultant solid washed with a 1:1:1 mixture of DCM:hexane:toluene to furnish the desired product. The two portions were combined to yield **5-chloro-2-(3-(3-(trifluoromethyl)phenyl)ureido)benzoic acid.** White solid; yield 86% (2.45 g, 6.830 mmol); mp: 167 – 169 °C; ^{1}H NMR (500 MHz, [D$_6$]DMSO): δ ppm 10.44 (s, 1H, NH″), 10.24 (s, 1H, NH), 8.43 (d, $^{3}J_{H3-H4}$ = 9.2 Hz, 1H, H3), 8.00 (s, 1H, H2″), 7.90 (d, $^{4}J_{H6-H4}$ = 2.1 Hz, 1H, H6), 7.69 (br d, $^{3}J_{H6''-H5''}$ = 8.4 Hz, 1H, H6″), 7.61 (dd, $^{3}J_{H4-H3}$ = 9.1 Hz, $^{4}J_{H4-H6}$ = 2.0 Hz, 1H, H4), 7.52 (t, $^{3}J_{H5''-H4''}$ = $^{3}J_{H5''-H6''}$ = 8.0 Hz, 1H, H5″), 7.33 (br d, $^{3}J_{H4''-H5''}$ = 7.8 Hz, 1H, H4″); ^{13}C NMR (126 MHz, [D$_6$]DMSO): δ ppm 168.3 (s, COOH), 152.1 (s, CO), 140.8 (s, C1″), 140.5 (s, C2), 133.5 (s, C4), 130.1 (s, C6), 130.0 (s, C5″), 129.5 (q, $^{2}J_{C-F}$ =

31.6 Hz, C3″), 124.8 (s, C5), 122.2 (s, C3), 121.7 (s, C6″), 124.2 (q, $^{1}J_{C\text{-}F}$ = 272.1 Hz, CF_3), 118.5 (br q, $^{3}J_{C\text{-}F}$ = 3.9 Hz, C2″), 117.3 (s, C1), 114.6 (br q, $^{3}J_{C\text{-}F}$ = 2.9 Hz, C4″); MS (m/z): [M+H, ^{35}Cl, 100%]$^{+}$, 359.2, [M+H, ^{37}Cl, 32%]$^{+}$, 361.2; HRMS (m/z): [M+H]$^{+}$ calcd. for $C_{15}H_{10}O_3N_2{}^{35}ClF_3$, 359.0405; found, 359.0399; error, –1.62 ppm.

2-(3-(4-Chloro-3-(trifluoromethyl)phenyl)ureido)-5-iodobenzoic acid (NVR-11)

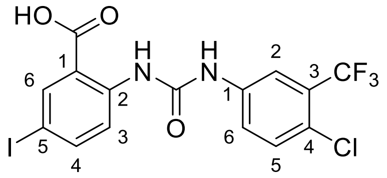

2-Amino-5-iodobenzoic acid (150.0 mg, 0.570 mmol) and 4-chloro-3-(trifluoromethyl)phenyl isocyanate (126.4 mg, 0.570 mmol) were suspended in THF (3 mL). This mixture was stirred at room temperature for 2 h then stored in a refrigerator overnight. The precipitate was purified by flash column chromatography (0.5% methanol in DCM) to yield **2-(3-(4-chloro-3-(trifluoromethyl)phenyl)ureido)-5-iodobenzoic acid.** White solid; yield 9% (25.6 mg, 0.0528 mmol); mp: 195 – 198 °C; ^{1}H NMR (500 MHz, [D$_6$]DMSO): δ ppm 13.83 (br s, 1H, COOH), 10.48 (s, 1H, NH), 10.34 (s, 1H, NH″), 8.23 (d, $^{3}J_{H3\text{–}H4}$ = 9.0 Hz, 1H, H3), 8.20 (br s, 1H, H6), 8.09 (br s, 1H, H2″), 7.85 (br d, $^{3}J_{H4\text{–}H6}$ = 9.0 Hz, 1H, H4), 7.75 (br d, $^{3}J_{H6''\text{–}H5''}$ = 8.9 Hz, 1H, H6″), 7.61 (d, $^{3}J_{H5''\text{–}H6''}$ = 8.9 Hz, 1H, H5″); ^{13}C NMR (126 MHz, [D$_6$]DMSO): δ ppm 168.6 (s, COOH), 152.2 (s, CO), 141.3 (s, C4), 140.2 (s, C1″), 139.6 (s, C2), 139.2 (s, C6), 131.9 (s, C5″), 126.6 (q, $^{2}J_{C\text{-}F}$ = 30.5 Hz, C3″), 123.1 (s, C3 + C4″), 122.3 (s, C1), 121.5 (s, C6″), 122.8 (q, $^{1}J_{C\text{-}F}$ = 272.9 Hz, CF_3), 117.1 (q, $^{3}J_{C\text{-}F}$ = 5.4 Hz, C2″), 83.7 (s, C5); ^{19}F NMR (471 MHz, [D$_6$]DMSO): δ ppm −61.51 (s, CF_3); MS (m/z): [M−H, ^{35}Cl, 100%]$^{-}$, 482.9, [M−H, ^{37}Cl, 75%]$^{-}$, 484.9; HRMS (m/z): [M−H]$^{-}$ calcd. for $C_{15}H_8O_3N_2{}^{35}ClF_3I$, 482.9215; found 482.9233; error, 3.78 ppm.

1-(5-Chloro-2-(1*H*-tetrazol-5-yl)phenyl)-3-(4-chloro-3-(trifluoromethyl)phenyl)urea (NVR-43)

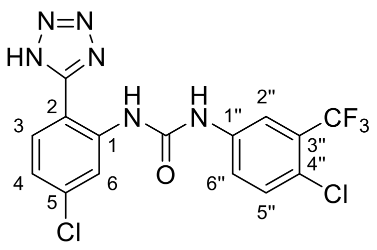

4-Chloro-3-(trifluoromethyl)phenyl isocyanate (163.0 mg, 0.736 mmol) was added dropwise to a solution of 5-chloro-2-(1*H*-tetrazol-5-yl)-phenylamine (120 mg, 0.613 mmol) and DIPEA (237.2 mg, 2.5 mmol) in anhydrous DMF (3 mL) at 0 °C under N_2. This mixture was stirred at 0 °C for 3 h then at room temperature overnight. The reaction mixture was heated at 70 °C for 1 h before concentration and extraction with EtOAc (2 × 5 mL) and NaOH (0.1 M, 3 mL). The aqueous layer was acidified with 1M HCl, the precipitate was filtered and washed with water. The filtrate was purified by HPLC (eluting with 50% acetonitrile and 50% water) to yield **1-(5-chloro-2-(1*H*-tetrazol-5-yl)phenyl)-3-(4-chloro-3-(trifluoromethyl)phenyl)urea.** Cream solid; yield 8% (25 mg, 0.060 mmol), mp: 192 $^{°C;}$ ^{1}H NMR (500 MHz, CD_3CN) δ ppm 10.34 (br s, 1H, NH), 8.57 (s, 1H, NH″), 8.46 (br s, 1H, H6), 8.04 (s, 1H, H2″), 7.83 (br d, $^3J_{H3–H4}$ = 8.4 Hz, 1H, H3), 7.70 (br d, $^3J_{H6''–H5''}$ = 8.7 Hz, 1H, H6″), 7.50 (br d, $^3J_{H5''–H6''}$ = 8.7 Hz, 1H, H5″), 7.21 (br d, $^3J_{H4–H3}$ = 8.5 Hz, 1H, H4), tetrazole NH not observed; ^{13}C NMR (126 MHz, CD_3CN): δ ppm 155.3 (s, Tet-C), 153.1 (s, CO), 140.6 (s, C1), 139.8 (s, C1″), 138.3 (s, C5), 132.9 (s, C5″), 130.7 (s, C3), 124.6 (s, C6), 123.5 (s, C4), 121.4 (s, C6″), 119.0 (q, $^3J_{C-F}$ = 6.0 Hz, C2″), 110.8 (s, C2) , C3″ + C4″ not observed; IR: 3265 (N-H), 3106 (N-H), 1678 (C=O), 1322 (C-F) cm^{-1};^{19}F NMR (471 MHz, CD_3CN): δ ppm −63.22 (s, CF_3); MS (m/z): [M−H, ^{35}Cl^{35}Cl, 70%]$^-$, 415.1, [M−H, ^{35}Cl^{37}Cl, 100%]$^-$, 417.1, [M−H, ^{37}Cl^{37}Cl, 20%]$^-$, 419.1.

1-(4-Bromo-2-(1*H*-tetrazol-5-yl)phenyl)-3-(4-chloro-3-(trifluoromethyl)phenyl)urea (NVR-44)

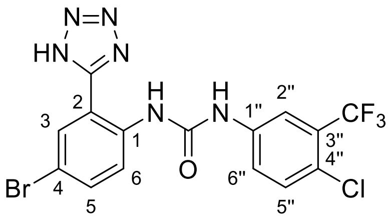

4-Chloro-3-(trifluoromethyl)phenyl isocyanate (92.3 mg, 0.42 mmol) was added dropwise to a solution of 4-bromo-2-(1*H*-tetrazol-5-yl)-phenylamine (100.0 mg, 0.42 mmol) and DIPEA (215.1 mg, 1.7 mmol) in anhydrous DMF (3 mL) at 0 °C under N_2. This mixture was stirred at 0 °C for 3 h then at room temperature overnight. The reaction mixture was heated at 70 °C for 1 h before concentration and extraction with EtOAc (2 × 5 mL) and NaOH (0.1 M, 3 mL). The aqueous layer was acidified with 1M HCl; the precipitate was filtered and washed with water to yield **1-(4-bromo-2-(1*H*-tetrazol-5-yl)phenyl)-3-(4-chloro-3-(trifluoromethyl)phenyl)urea.** Cream solid; yield 23% (40 mg, 0.086 mmol); mp: 208 – 210 °C (lit 259 – 261 °C)[6;] ^{1}H NMR (500 MHz, [D$_6$]DMSO): δ ppm 10.34 (s, 1H, NH″), 10.00 (s, 1H, NH), 8.27 (d, $^3J_{H6–H5}$ = 9.0 Hz, 1H, H6), 8.15 (br d, $^4J_{H3–H5}$ = 1.7 Hz, 1H, H3), 8.11 (br s, 1H, H2″), 7.75 (br d, $^3J_{H6''–H5''}$ = 8.5 Hz, 1H, H6″), 7.72 (br d, $^3J_{H5–H6}$ =8.9 Hz, 1H, H5), 7.63 (br d, $^3J_{H5''–H6''}$ = 8.7 Hz, 1H, H5″), tetrazole NH not observed; ^{13}C NMR (126 MHz, [D$_6$]DMSO): δ ppm 152.0 (s, CO), 139.2 (s, C1″), 137.0 (s, C1), 134.0 (s, C6″), 132.0 (s, C3), 130.8 (s, C5″), 126.7 (q, $^2J_{C\text{-}F}$ = 30.3 Hz, C3″), 123.4 (s, C6), 123.3 (s, C5), 122.7 (br q, $^3J_{C\text{-}F}$ = 1.5 Hz, C4″), 122.8 (q, $^1J_{C\text{-}F}$ = 272.9 Hz, CF_3), 117.2 (q, $^3J_{C\text{-}F}$ = 6.2 Hz, C2″), 114.1 (s, C4), 114.0 (s, C2), tetrazole carbon not observed; ^{19}F NMR (471 MHz, CD_3OD): δ ppm −64.10 (s, CF_3); MS (m/z): [M−H, ^{35}Cl, ^{79}Br, 75%]$^-$, 459.0, [M−H, ^{35}Cl^{81}Br/^{37}Cl^{79}Br, 100%]$^-$, 461.0, [M−H, ^{37}Cl, ^{81}Br, 25%]$^-$, 463.0.

4-Chloro-2-(3-(4-(*N*-(3,4-dibromophenyl)sulfamoyl)phenyl)ureido)benzoic acid (NVR-47)

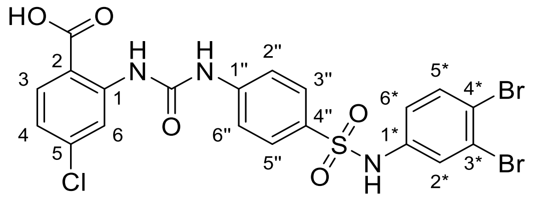

2-Amino-4-chloro-benzoic acid (150.0 mg, 0.87 mmol) and 4-isocyanatobenzenesulfonyl chloride (190.3 mg, 0.874 mmol) were suspended in THF (3 mL). This mixture was stirred at 0 °C for 4 h and stored in a refrigerator overnight. The yellow solid that precipitated was filtered and washed with hexane to give crude 4-chloro-2-(3-(4-(chlorosulfonyl)phenyl)ureido)benzoic acid**.** This precipitate (100.0 mg, 0.26 mmol) was concentrated and suspended in anhydrous DCM (3 mL) at 0 °C under N_2. This suspension was added dropwise to a solution of 3,4-dibromoaniline (64.3 mg, 0.26 mmol) and pyridine (23.2 mg, 0.3 mmol) in anhydrous DCM (3 mL) at 0 °C under N_2. This mixture was stirred at 0 °C under N_2 for 5 h at room temperature overnight. The solution was concentrated and purified by flash column chromatography (2% methanol in DCM) to yield **4-chloro-2-(3-(4-(*N*-(3,4-dibromophenyl)sulfamoyl)phenyl)ureido)benzoic acid.** White solid; yield 4% (20 mg, 0.033 mmol). ^{1}H NMR (400 MHz, [D$_6$]DMSO): δ ppm 10.75 (br s, 2H, NH + NH″), 10.40 (br s, 1H, NH*), 8.48 (br d, $^4J_{H6–H4}$ = 1.6 Hz, 1H, H6), 7.96 (br d, $^3J_{H3–H4}$ = 8.7 Hz, 1H, H3), 7.65 – 7.77 (m, 4H, H2″ + H3″ + H5″ + H6″), 7.60 (br d, $^3J_{H5*–H6*}$ = 8.7 Hz, 1H, H5*), 7.50 (br d, $^4J_{H2*–H6*}$ = 1.6 Hz, 1H, H2*), 7.38 (d, $^3J_{H4–H3}$ = 6.3 Hz, 1H, H4), 7.12 (dd, $^3J_{H6*–H5*}$ =8.1 Hz, $^4J_{H6*–H2*}$ = 1.5 Hz, 1H, H6*), COOH not observed; ^{13}C NMR (126 MHz, [D$_6$]DMSO): δ ppm 168.9 (s, COOH), 152.4 (s, CO), 144.0 (s, C5), 142.8 (s, C1), 138.7 (s, C1*), 138.4 (s, C1″), 138.2 (s, C4″), 134.2 (s, C5*), 133.1 (s, C3″ + C5″), 131.5 (s, C3), 124.1 (s, C4), 123.7 (s, C3*), 121.3 (s, C6), 120.0 (s, C6*), 118.4 (s, C2*), 115.0 (s, C2″ + C6″), 114.5 (s, C4*), 108.5 (s, C2); MS (m/z): [M−H, ^{35}Cl^{79}Br^{79}Br, 48%]$^-$, 599.9, [M−H, ^{37}Cl^{79}Br^{79}Br + ^{35}Cl^{79}Br^{81}Br, 100%]$^-$, 601.9, [M−H, ^{35}Cl^{81}Br^{81}Br + ^{37}Cl^{79}Br^{81}Br, 52%]$^-$, 604.2, [M−H, ^{37}Cl^{81}Br^{81}Br, 5%]$^-$, 605.9.

1-([1,1'-Biphenyl]-2-yl)-3-(3,5-bis(trifluoromethyl)phenyl)urea (NVR-113)

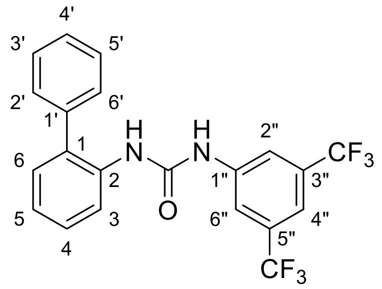

3,5-Bis(trifluoromethyl)phenyl isocyanate (0.34 mL, 1.95 mmol, 1.1 eq) was added dropwise to a stirred solution of 2-aminobiphenyl (300 mg, 1.18 mmol, 1.0 eq) in acetonitrile (25 mL). The reaction was stirred for 1 hour at room temperature. Solvent was removed *in vacuo*, the white residue triturated with cold acetonitrile, and the precipitate was dried to yield **1-([1,1'-biphenyl]-2-yl)-3-(3,5-bis(trifluoromethyl)phenyl)urea.** White solid; yield 45% (337 mg, 0.794 mmol); mp: 185 – 186 °C; ^{1}H NMR (400 MHz, [D$_6$]DMSO): δ ppm 9.67 (br s, 1H, NH″), 8.02 (br s, 2H, H2″ + H6″), 7.88 (s, 1H, NH), 7.87 (dd, $^{3}J_{H3–H4}$ = 8.3 Hz, $^{4}J_{H3–H5}$ = 1.2 Hz, 1H, H3), 7.61 (br s, 1H, H4″), 7.49 (br t, $^{3}J_{H3'/H5'–H4'}$ = $^{3}J_{H3'/H5'–H2'/H6'}$ = 7.5 Hz, 2H, H3′ + H5′), 7.39 – 7.45 (m, 3H, H2′ + H4′ + H6′), 7.36 (ddd, $^{3}J_{H4–H3}$ = 8.4 Hz, $^{3}J_{H4–H5}$ = 6.9 Hz, $^{4}J_{H4–H6}$ = 1.8 Hz, 1H, H4), 7.26 (dd, $^{3}J_{H6–H5}$ = 7.5 Hz, $^{4}J_{H6–H4}$ = 1.8 Hz, 1H, H6), 7.20 (td, $^{3}J_{H5–H4}$ = $^{3}J_{H5–H6}$ = 7.3 Hz, $^{4}J_{H5–H3}$ = 1.3 Hz, 1H, H5); ^{13}C NMR (101 MHz, [D$_6$]DMSO): δ ppm 152.7 (s, CO), 141.9 (s, C1″), 138.4 (s, C1′), 134.9 (s, C2), 133.8 (s, C1), 130.4 (s, C6), 130.7 (q, $^{2}J_{C-F}$ = 32.6 Hz, C3″ + C5″), 129.1 (s, C2′ + C6′), 128.8 (s, C3′ + C5′), 127.9 (s, C4), 127.5 (s, C4′), 124.2 (s, C5), 123.5 (s, C3), 123.3 (q, $^{1}J_{C-F}$ = 272.6 Hz, 2 × CF_3), 117.6 (br q, $^{3}J_{C-F}$ = 2.0 Hz, C2″ + C6″), 114.2 (br septet, $^{3}J_{C-F}$ = 3.9 Hz, C4″); ^{19}F NMR (376 MHz, [D$_6$]DMSO): δ ppm −61.72 (s, 2 × CF_3); NMR assignments made with the assistance of COSY, HSQC and HMBC; LCMS (m/z): [M−H, 100%]$^-$, 423.3; RT = 3.23 min; HRMS (m/z): [M−H]$^-$ calcd. for $C_{21}H_{13}ON_2F_6$, 423.0938; found, 423.0918; error, –4.62 ppm.

1-(3,5-Bis(trifluoromethyl)phenyl)-3-(2-morpholinophenyl)urea (NVR-114)

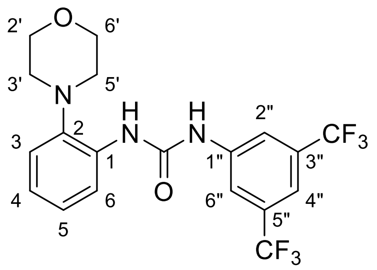

3,5-Bis(trifluoromethyl)phenyl isocyanate (0.32 mL, 1.85 mmol, 1.1 eq) was added dropwise to a stirred solution of 2-morpholinoaniline (300 mg, 1.68 mmol, 1.0 eq) in acetonitrile (25 mL). The reaction was stirred for 1 hour at room temperature. The reaction mixture was filtered and the precipitate washed with cold acetonitrile, before being dried to yield **1-(3,5-bis(trifluoromethyl)phenyl)-3-(2-morpholinophenyl)urea.** White solid; yield 54% (392 mg, 0.905 mmol); mp: 198 – 199 °C; ^{1}H NMR (400 MHz, [D$_6$]DMSO): δ ppm 10.22 (s, 1H, NH″), 8.26 (s, 1H, NH), 8.13 (br s, 2H, H2″ + H6″), 8.06 (dd, $^{3}J_{H6–H5}$ = 8.0 Hz, $^{4}J_{H6–H4}$ = 1.5 Hz, 1H, H6), 7.65 (br s, 1H, H4″), 7.23 (dd, $^{3}J_{H3–H4}$ = 7.8 Hz, $^{4}J_{H3–H5}$ = 1.5 Hz, 1H, H3), 7.10 (td, $^{3}J_{H5–H4}$ = $^{3}J_{H5–H6}$ = 7.8 Hz, $^{4}J_{H5–H3}$ = 1.5 Hz, 1H, H5), 7.03 (td, $^{3}J_{H4–H3}$ = $^{3}J_{H4–H5}$ = 7.5 Hz, $^{4}J_{H4–H6}$ = 1.8 Hz, 1H, H4), 3.82 – 3.89 (m, 4H, H2′ + H6′), 2.77 – 2.86 (m, 4H, H3′ + H5′); ^{13}C NMR (101 MHz, [D$_6$]DMSO): δ ppm 152.2 (s, CO), 141.9 (s, C1″), 141.4 (s, C2), 133.3 (s, C1), 130.8 (q, $^{2}J_{C-F}$ = 33.3 Hz, C3″ + C5″), 124.6 (s, C5), 122.9 (s, C4), 120.4 (s, C3), 119.3 (s, C6), 123.3 (q, $^{1}J_{C-F}$ = 272.9 Hz, 2 × CF_3), 117.6 (br q, $^{3}J_{C-F}$ = 3.9 Hz, C2″ + C6″), 114.3 (br septet, $^{3}J_{C-F}$ = 3.9 Hz, C4″), 66.3 (s, C2′ + C6′), 52.0 (s, C3′ + C5′); ^{19}F NMR (376 MHz, [D$_6$]DMSO): δ ppm −61.70 (s, 2 × CF_3); NMR assignments made with the assistance of COSY, HSQC and HMBC; LCMS (m/z): $[M−H, 100\%]^-$, 432.3; RT = 3.14 min; HRMS (m/z): $[M+H]^+$ calcd. for $C_{19}H_{18}O_2N_3F_6$, 434.1298; found, 434.1290; error, −1.78 ppm.

1-(2-(1*H*-Pyrrol-1-yl)phenyl)-3-(3,5-bis(trifluoromethyl)phenyl)urea (NVR-115)

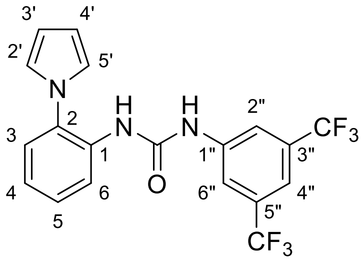

3,5-Bis(trifluoromethyl)phenyl isocyanate (0.36 mL, 2.09 mmol, 1.1 eq) was added dropwise to a stirred solution of 2-(1*H*-pyrrol-1-yl)aniline (300 mg, 2.09 mmol, 1.0 eq) in acetonitrile (25 mL). The reaction was stirred for 1 hour at room temperature. Solvent was removed *in vacuo*, the white residue triturated with cold acetonitrile, and finally the precipitate was dried to yield **1-(2-(1*H*-pyrrol-1-yl)phenyl)-3-(3,5-bis(trifluoromethyl)phenyl)urea.** Off-white solid; yield 29% (229 mg, 0.554 mmol); mp: 182 – 183 °C; ^{1}H NMR (400 MHz, [D$_6$]DMSO): δ ppm 9.85 (s, 1H, NH″), 8.05 (br s, 2H, H2″ + H6″), 7.96 (dd, $^{3}J_{H6–H5}$ = 8.0 Hz, $^{4}J_{H6–H4}$ = 1.3 Hz, 1H, H6), 7.94 (br s, 1 H), 7.62 (br s, 1H, NH), 7.37 (ddd, $^{3}J_{H5–H6}$ = 8.2 Hz, $^{3}J_{H5–H4}$ = 7.5 Hz, $^{4}J_{H5–H3}$ = 1.8 Hz, 1H, H5), 7.27 (dd, $^{3}J_{H3–H4}$ = 7.8 Hz, $^{4}J_{H3–H5}$ = 1.8 Hz, 1H, H3), 7.21 (td, $^{4}J_{H4–H3}$ = $^{4}J_{H4–H5}$ = 7.5 Hz, $^{4}J_{H4–H6}$ = 1.3 Hz, 1H, H4), 6.99 (t, $^{3}J_{H2'/H5'–H3'/H4'}$ = 2.1 Hz, 2H, H2′ + H5′), 6.29 (t, $^{3}J_{H3'/H4'–H2'/H5'}$ = 2.1 Hz, 2H, H3′ + H4′); ^{13}C NMR (101 MHz, [D$_6$]DMSO): δ ppm 152.6 (s, CO), 141.7 (s, C1″), 132.6 (s, C1), 132.5 (s, C2), 130.7 (q, $^{2}J_{C-F}$ = 32.6 Hz, C3″ + C5″), 127.6 (s, C5), 126.8 (s, C3), 124.4 (s, C4), 123.9 (s, C6), 122.0 (s, C2′ + C5′), 123.3 (q, $^{1}J_{C-F}$ = 272.6 Hz, 2 × CF_3), 117.7 (br q, $^{3}J_{C-F}$ = 3.9 Hz, C2″ + C6″), 114.4 (br septet, $^{3}J_{C-F}$ = 3.9 Hz, C4″), 109.7 (s, C3′ + C4′); ^{19}F NMR (376 MHz, [D$_6$]DMSO): δ ppm −61.71 (s, 2 × CF_3); NMR assignments made with the assistance of COSY, HSQC and HMBC; LCMS (m/z): [M−H, 100%]$^-$, 412.3; RT = 3.33 min; HRMS (ES$^+$): [M−H]$^+$ calcd. for $C_{19}H_{12}ON_3F_6$, 412.0890; found 412.0872; error, –4.38 ppm.

1-(2-(1*H*-Benzo[d]imidazol-2-yl)phenyl)-3-(3,5-bis(trifluoromethyl)phenyl)urea (NVR-116)

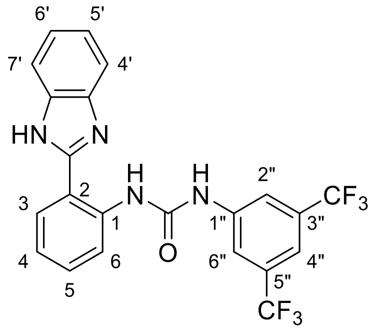

3,5-Bis(trifluoromethyl)phenyl isocyanate (0.27 mL, 1.57 mmol, 1.1 eq) was added dropwise to a stirred solution of 2-(2-aminophenyl)benzimidazole (300 mg, 1.43 mmol, 1.0 eq) in acetonitrile (25 mL). The reaction was stirred for 1 hour at room temperature. The white precipitate was filtered and then washed with acetonitrile, before being dried to yield **1-(2-(1*H*-benzo[d]imidazol-2-yl)phenyl)-3-(3,5-bis(trifluoromethyl)phenyl)urea.** White solid; yield 69% (457 mg, 0.984 mmol); mp: >300 °C; ^{1}H NMR (400 MHz, [D$_6$]DMSO): δ ppm 13.12 (br s, 1H, Benz-NH), 12.45 (s, 1H, NH), 10.42 (s , 1H, NH″) , 8.44 (dd, $^{3}J_{H6–H5}$ = 8.5 Hz, $^{4}J_{H6–H4}$ = 1.0 Hz, 1H, H6), 8.29 (br s, 2H, H2″ + H6″), 8.10 (dd, $^{3}J_{H3–H4}$ = 7.9 Hz, $^{4}J_{H3–H5}$ = 1.4 Hz, 1H, H3), 7.73 (br s, 2H, H4′ + H7′), 7.66 (br s, 1H, H4″), 7.48 (ddd, $^{3}J_{H5–H6}$ = 8.5 Hz, $^{3}J_{H5–H4}$ = 7.2 Hz, $^{4}J_{H5–H3}$ = 1.4 Hz, 1H, H5), 7.26 – 7.34 (m, 2H, H5′ + H6′), 7.22 (ddd, $^{3}J_{H4–H3}$ = 7.9 Hz, $^{3}J_{H4–H5}$ = 7.2 Hz, $^{4}J_{H4–H6}$ = 1.0 Hz, 1H, H4); ^{13}C NMR (101 MHz, [D$_6$]DMSO): δ ppm 152.5 (s, CO), 150.9 (s, C2′), 142.1 (s, C1″), 138.7 (s, C1), 130.4 (s, C5), 130.6 (q, $^{2}J_{C\text{-}F}$ = 32.3 Hz, C3″ + C5″), 127.3 (s, C3), 122.7 (br s, C5′ + C6′), 126.1 (q, $^{1}J_{C\text{-}F}$ = 272.9 Hz, 2 × CF$_3$), 122.0 (s, C4), 120.4 (s, C6), 119.3 (s, C2), 118.3 (br q, $^{3}J_{C\text{-}F}$ = 3.9 Hz, C2″ + C6″), 115.4 (s, C4′ + C7′), 114.4 (br septet, $^{3}J_{C\text{-}F}$ = 3.9 Hz, C4″); ^{19}F NMR (376 MHz, [D$_6$]DMSO): δ ppm −61.57 (s, 2 × CF$_3$); NMR assignments made with the assistance of COSY, HSQC and HMBC; LCMS (m/z): [M+H, 100%]$^-$, 465.3; RT = 3.48 min; HRMS (m/z): [M−H]$^-$ calcd. for $C_{22}H_{13}ON_4F_6$, 463.0999 Found, 463.0985; error, −3.03 ppm.

1-(3,5-Bis(trifluoromethyl)phenyl)-3-phenylurea (NVR-35)

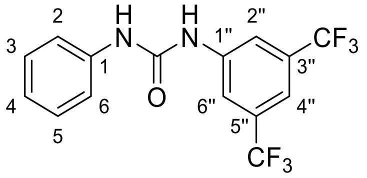

Aniline (120.0 mg, 1.29 mmol) and 3,5-bis(trifluoromethyl)phenyl isocyanate (329.0 mg, 1.29 mmol) were suspended in THF (5 mL). This mixture was stirred at room temperature for 2 h and stored in a refrigerator overnight. The white solid that precipitated was filtered and washed with hexane to yield **1-(3,5-bis(trifluoromethyl)phenyl)-3-phenylurea.** White solid; yield 11% (50 mg, 0.14 mmol); mp: 183 – 184 °C; ^{1}H NMR (500 MHz, CD_3CN): δ ppm 9.37 (s, 1H, NH″), 8.96 (s, 1H, NH), 8.13 (s, 2H, H2″ + H6″), 7.61 (s, 1H, H4″), 7.48 (br d, $^{3}J_{H2'/H6–H3/H5}$ = 7.6 Hz, 2H, H2 + H6), 7.30 (t, $^{3}J_{H3/H5–H2/H6}$ = 7.9 Hz, 2H, H3 + H5), 7.01 (t, $^{3}J_{H4–H3}$ = $^{3}J_{H4–H5}$ = 7.3 Hz, 1H, H4); ^{13}C NMR (126 MHz, [D$_6$]DMSO): δ ppm 152.4 (s, CO), 141.9 (s, C1), 139.0 (s, C1″), 130.7 (q, $^{2}J_{C-F}$ = 32.6 Hz, C3″ + C5″), 128.8 (s, C3 + C5), 122.5 (s, C4), 123.3 (q, $^{1}J_{C-F}$ = 272.9 Hz, 2 × CF_3), 118.9 (s, C2 + C6), 117.9 (br q, $^{3}J_{C-F}$ = 2.9 Hz, C2″+ C6″), 114.3 (br septet, $^{3}J_{C-F}$ = 3.9 Hz, C4″); MS (m/z): [M−H, 100%]$^{-}$, 347.0.

1-(3,5-Bis(trifluoromethyl)phenyl)-3-(4-bromophenyl)urea (NVR-36)

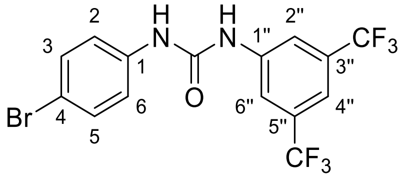

4-Bromoaniline (120.0 mg, 0.69 mmol) and 3,5-bis(trifluoromethyl)phenyl isocyanate (177.9 mg, 0.69 mmol) were suspended in THF (5 mL). This mixture was stirred at room temperature for 2 h and stored in a refrigerator overnight. The white solid that precipitated was filtered and washed with hexane to yield **1-(3,5-bis(trifluoromethyl)phenyl)-3-(4-bromophenyl)urea.** White solid; yield 16% (48.2 mg, 0.113 mmol); mp: 225 °C; ^{1}H NMR (500 MHz, [D$_6$]DMSO): δ ppm 9.41 (s, 1H, NH″), 9.12 (s, 1H, NH), 8.12 (s, 2H, H2″ + H6″), 7.62 (s, 1H, H4″), 7.46 (s, 4H, H2 + H3 + H5 + H6); ^{13}C NMR (126 MHz, [D$_6$]DMSO): δ ppm 152.3 (s, CO), 141.7 (s, C1), 138.5 (s, C1″), 131.5 (s, C3 + C5), 130.7 (q, $^{2}J_{C-F}$ = 32.6 Hz, C3″ + C5″), 120.8 (s, C2 + C6), 123.3 (q, $^{1}J_{C-F}$ = 272.4 Hz, 2 × CF_3), 118.1 (br q, $^{3}J_{C-F}$ = 3.9 Hz, C2″ + C6″), 114.5 br septet,

$^3J_{C\text{-}F}$ = 3.9 Hz, C4″), 114.0 (s, C4); ^{19}F NMR (471 MHz, [D$_6$]DMSO): δ ppm −61.74 (s, 2 × CF_3); MS (m/z): [M−H, ^{79}Br, 100%]$^-$, 425.0, [M−H, ^{81}Br, 70%]$^-$, 427.0.

1-(3,5-Bis(trifluoromethyl)phenyl)-3-(3,4-dibromophenyl)urea (NVR-142)

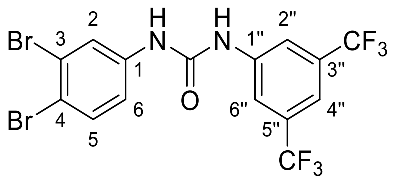

3,5-Bis(trifluoromethyl)phenyl isocyanate (408 mg, 1.6 mmol) was added dropwise to a solution of 3,4-dibromoaniline (200 mg, 1.0 mmol) in a mixture of anhydrous THF (3 mL) and toluene (5 mL) at room temperature under N_2. This mixture was stirred overnight at room temperature; then stored in a refrigerator overnight. The white solid that precipitated was filtered and washed with DCM to yield **1-(3,5-bis(trifluoromethyl)phenyl)-3-(3,4-dibromophenyl)urea.** White solid; yield 77% (308 mg, 0.609 mmol); mp: 220 – 223 °C; ^{1}H NMR (400 MHz, [D$_6$]DMSO): δ ppm 9.51 (s, 1H, NH), 9.28 (s, 1H, NH″), 8.13 (s, 2H, H2″ + H6″), 8.02 (d, $^4J_{H2\text{–}H6}$ = 2.4 Hz, 1H, H2), 7.62 – 7.67 (m, 2H, H5 + H4″), 7.36 (dd, $^3J_{H6\text{–}H5}$ = 8.8 Hz, $^4J_{H6\text{–}H2}$ = 2.4 Hz, 1H, H6); ^{13}C NMR (101 MHz, [D$_6$]DMSO): δ ppm 152.2 (s, CO), 141.5 (s, C1″), 139.8 (s, C1), 133.6 (s, C5), 130.7 (q, $^2J_{C\text{-}F}$ = 32.9 Hz, C3″ + C5″), 123.8 (s, C3), 123.1 (s, C2), 119.7 (s, C6), 123.3 (q, $^1J_{C\text{-}F}$ = 272.6 Hz, 2 × CF_3), 118.3 (br q, $^3J_{C\text{-}F}$ = 3.9 Hz, C2″ + C6″), 115.9 (s, C4), 114.7 (br septet, $^3J_{C\text{-}F}$ = 3.9 Hz, C4″); ^{19}F NMR (376 MHz, [D$_6$]DMSO): δ ppm −61.70 (s, 2 × CF_3); MS (m/z): [M−H, ^{79}Br^{79}Br, 15%]$^-$, 502.8, [M−H, ^{79}Br^{81}Br, 85%]$^-$, 504.8, [M−H, ^{81}Br^{81}Br, 100%]$^-$, 507.0.

1-(3,5-Bis(trifluoromethyl)phenyl)-3-(3-bromo-5-cyanophenyl)urea (NVR-140)

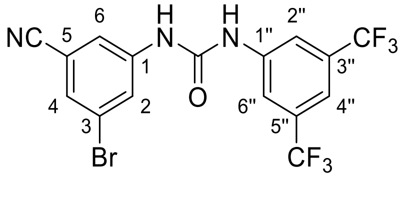

3,5-Bis(trifluoromethyl)phenyl isocyanate (500 mg, 2.0mmol) was added dropwise to a solution of 5-amino-3-bromobenzonitrile (200 mg, 1.0 mmol) in a mixture of anhydrous THF (3 mL) and toluene (5 mL) at room temperature under N_2. This mixture was stirred overnight at room temperature; then stored in a refrigerator overnight. The white solid that

precipitated was filtered and washed with DCM to yield **1-(3,5-bis(trifluoromethyl)phenyl)-3-(3-bromo-5-cyanophenyl)urea.** White solid; yield 63% (290 mg, 0.641 mmol); mp: 179 – 180 °C; ^{1}H NMR (400 MHz, [D$_6$]DMSO): δ ppm 9.68 (br s, 1H, NH), 9.46 (br s, 1H, NH″), 8.13 (br s, 2H, H2″ + H6″), 8.06 (br s, 1H, H4), 7.88 (br s, 1H, H6), 7.73 (br s, 1H, H2), 7.67 (br s, 1H, H4″); ^{13}C NMR (101 MHz, [D$_6$]DMSO): δ ppm 152.2 (s, CO), 141.4 (s, C1″), 141.3 (s, C1), 130.7 (q, $^{2}J_{C\text{-}F}$ = 32.3 Hz, C3″ + C5″), 127.8 (s, C4), 125.7 (s, C2), 122.3 (s, C3), 120.8 (s, C6), 123.4 (br q, $^{1}J_{C\text{-}F}$ = 238.2 Hz, 2 × CF_3), 118.4 (br q, $^{3}J_{C\text{-}F}$ = 3.9 Hz, C2″ + C6″), 117.3 (s, CN), 114.9 (br septet, $^{3}J_{C\text{-}F}$ = 3.9 Hz, C4″), 113.4 (s, C5); ^{19}F NMR (376 MHz, [D$_6$]DMSO): δ ppm −61.70 (s, 2 × CF_3); MS (m/z): [M−H, ^{79}Br, 60%]$^{-}$, 450.0, [M−H, ^{81}Br, 100%]$^{-}$, 452.1.

1,3-Bis(3,5-bis(trifluoromethyl)phenyl)urea (NVR-111)

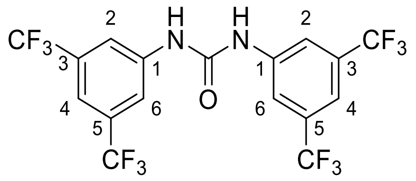

3,5-Bis(trifluoromethyl)phenyl isocyanate (0.20 mL, 1.18 mmol, 1.0 eq) was added dropwise to a stirred solution of 3,5-bis(trifluoromethyl)aniline (0.18 mL, 1.18 mmol, 1.0 eq) in acetonitrile (25 mL). The reaction was stirred for 16 hours at room temperature. The reaction mixture was filtered, the precipitate triturated with hexane, and the precipitate was dried to yield **1,3-bis(3,5-bis(trifluoromethyl)phenyl)urea.** White solid; yield 86% (491 mg, 1.014 mmol); mp: 241 – 242 °C (lit 236 - 238 °C)[7]; ^{1}H NMR (400 MHz, [D$_6$]DMSO): δ ppm 9.75 (br s, 2H, NH), 8.18 (br s, 4H, H2 + H6), 7.71 (br s, 2H, H4); ^{19}F NMR (376 MHz, [D$_6$]DMSO): δ ppm −61.60 (s, 4 x CF_3); LCMS (m/z): [M−H, 100%]$^{-}$, 483.1; RT = 3.29 min.

1-(3,5-Bis(trifluoromethyl)phenyl)-3-(3-cyano-5-(trifluoromethyl)phenyl)urea (NVR-136)

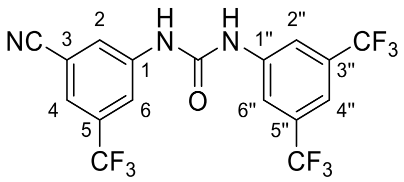

3,5-Bis(trifluoromethyl)phenyl isocyanate (550 mg, 2.0 mmol) was added dropwise to a solution of 5-Amino-3-trifluoromethylbenzonitrile (800 mg, 3.2 mmol) in a mixture of

anhydrous THF (3 mL) and toluene (5 mL) at room temperature under N_2. This mixture was stirred overnight at room temperature; then stored in a refrigerator overnight. The white solid that precipitated was filtered and washed with DCM to yield **1-(3,5-bis(trifluoromethyl)phenyl)-3-(3-cyano-5-(trifluoromethyl)phenyl)urea.** White solid; yield 88% (350 mg, 0.793 mmol); mp: 210 – 213 °C; ^{1}H NMR (500 MHz, [D$_6$]DMSO): δ ppm 9.74 (s, 1H, NH″), 9.63 (s, 1H, NH), 8.21 (br s, 1H, H6), 8.12 – 8.19 (m, 3H, H4 + H2″ + H6″), 7.93 (br s, 1H, H2), 7.68 (br s, 1H, H4″); ^{13}C NMR (126 MHz, [D$_6$]DMSO): δ ppm 152.4 (s, CO), 141.3 (s, C1″), 141.1 (s, C1), 130.8 (q, $^2J_{C\text{-}F}$ = 33.3 Hz, C5), 130.7 (q, $^2J_{C\text{-}F}$ = 32.3 Hz, C3″ + C5″), 125.4 (s, C2), 122.3 (br q, $^3J_{C\text{-}F}$ = 2.0 Hz, C4), 123.2 (q, $^1J_{C\text{-}F}$ = 272.9 Hz, 2 × CF_3), 123.0 (q, $^1J_{C\text{-}F}$ = 272.9 Hz, CF_3), 119.3 (br q, $^2J_{C\text{-}F}$ = 4.9 Hz, C6), 118.6 (br q, $^2J_{C\text{-}F}$ = 3.9 Hz, C2″ + C6″), 117.4 (s, CN), 115.1 (br septet, $^2J_{C\text{-}F}$ = 3.9 Hz, C4″), 113.0 (s, C3); ^{19}F NMR (471 MHz, [D$_6$]DMSO): δ ppm −61.69 (s, 2 × CF_3), -61.82 (s, CF_3); MS (m/z): [M−H, 100%]$^-$, 440.1.

3-(3-(3,5-Bis(trifluoromethyl)phenyl)ureido)-5-(trifluoromethyl)benzoate (NVR-137)

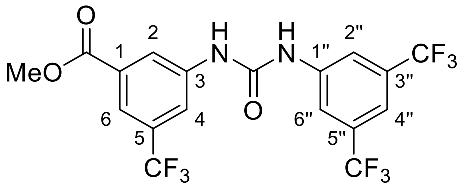

3,5-Bis(trifluoromethyl)phenyl isocyanate (680 mg, 2.7 mmol) was added dropwise to a solution of methyl 3-amino-5-(trifluoromethyl)benzoate (300 mg, 1.4 mmol) in a mixture of anhydrous THF (3 mL) and toluene (5 mL) at room temperature under N_2. This mixture was stirred overnight at room temperature; then stored in a refrigerator overnight. The white solid that precipitated was filtered and washed with DCM to yield **methyl 3-(3-(3,5-bis(trifluoromethyl)phenyl)ureido)-5-(trifluoromethyl)benzoate.** White solid; yield 63% (410 mg, 0.865 mmol); mp: 183 – 185 °C; ^{1}H NMR (400 MHz, [D$_6$]DMSO): δ ppm 9.65 (s, 1H, NH), 9.59 (s, 1H, NH″), 8.34 (br s, 1H, H4), 8.18 (br s, 1H, H6), 8.16 (s, 2H, H2″ + H6″), 7.80 (br s, 1H, H2), 7.67 (br s, 1H, H4″), 3.90 (s, 3H, COOMe); ^{13}C NMR (101 MHz, [D$_6$]DMSO): δ ppm 164.9 (s, COOMe), 152.4 (s, CO), 141.4 (s, C1″), 140.8 (s, C3), 131.4 (s, C1), 130.7 (q, $^2J_{C\text{-}F}$ = 32.3 Hz, C3″ + C5″), 130.0 (q, $^2J_{C\text{-}F}$ = 32.3 Hz, C5), 122.7 (s, C2), 123.6 (q, $^1J_{C\text{-}F}$ = 272.9 Hz, 5-CF_3), 123.3 (q, $^1J_{C\text{-}F}$ = 272.9 Hz, 2 × CF_3), 118.9 (br q, $^3J_{C\text{-}F}$ = 4.9 Hz, C6), 118.7 (br q, $^3J_{C\text{-}F}$ = 3.9 Hz, C4), 118.5 (br q, $^3J_{C\text{-}F}$ = 3.9 Hz, C2″ + C6″), 114.9 (br septet, $^3J_{C\text{-}F}$ = 4.9 Hz, C4″), 52.7 (s, MeOOC); ^{19}F

NMR (376 MHz, [D$_6$]DMSO): δ ppm −61.67 (s, CF_3), -61.68 (s, 2 × CF_3); MS (m/z): [M−H, 100%]$^-$, 473.1.

1-(3,5-Bis(trifluoromethyl)phenyl)-3-(3,5-dichloro-4-fluorophenyl)urea (NVR-143)

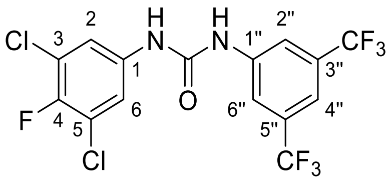

3,5-Bis(trifluoromethyl)phenyl isocyanate (840 mg, 3.3 mmol) was added dropwise to a solution of 3,5-dichloro-4-fluoroaniline (300 mg, 1.6 mmol) in a mixture of anhydrous THF (3 mL) and toluene (5 mL) at room temperature under N_2. This mixture was stirred overnight at room temperature; then stored in a refrigerator overnight. The white solid that precipitated was filtered and washed with DCM to yield **1-(3,5-bis(trifluoromethyl)phenyl)-3-(3,5-dichloro-4-fluorophenyl)urea.** White solid; yield 69% (482 mg, 1.11 mmol); mp: 178 – 179 °C; ^{1}H NMR (500 MHz, [D$_6$]DMSO): δ ppm 9.55 (br s, 1H, NH), 9.23 (br s, 1H, NH"), 8.10 (br s, 2H, H2" + H6"), 7.65 (d, $^4J_{H2/H6-F}$ = 6.0 Hz, 2H, H2 + H6), 7.61 (br s, 1H, H4"); ^{13}C NMR (126 MHz, [D$_6$]DMSO): δ ppm 152.2 (s, CO), 148.5 (d, $^1J_{C-F}$ = 242.6 Hz, C4), 141.4 (s, C1"), 136.5 (d, $^4J_{C-F}$ = 3.9 Hz, C1), 130.7 (q, $^2J_{C-F}$ = 32.3 Hz, C3" + C5"), 120.7 (d, $^2J_{C-F}$ = 17.6 Hz, C3 + C5), 123.3 (q, $^1J_{C-F}$ = 272.6 Hz, 2 × CF_3), 119.0 (br s, C2 + C6), 118.3 (br q, $^3J_{C-F}$ = 3.9 Hz, C2" + C6"), 114.7 (br septet, $^3J_{C-F}$ = 3.9 Hz, C4"); ^{19}F NMR (376 MHz, [D$_6$]DMSO): δ ppm −61.72 (s, 2 × CF_3), −126.00 (t, $^4J_{F-H2/H6}$ = 6.1 Hz, F); MS (m/z): [M−H, ^{35}Cl^{35}Cl, 65%]$^-$, 433.0, [M−H, ^{35}Cl^{37}Cl, 100%]$^-$, 435.1, [M−H, ^{37}Cl^{37}Cl, 35%]$^-$, 437.1.

1-(3,5-Bis(trifluoromethyl)phenyl)-3-(3,4-dibromophenyl)thiourea (NVR-97)

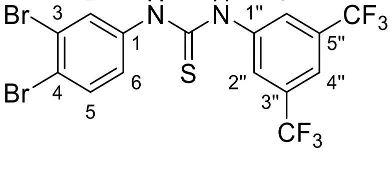

3,5-Bis(trifluoromethyl)phenyl isothioisocyanate (324 mg, 1.2 mmol) was added dropwise to a solution of 3,4-dibromoaniline (200 mg, 1.0 mmol) in a mixture of anhydrous THF (3 mL) and toluene (5 mL) at room temperature under N_2. This mixture was stirred overnight at room

temperature; then stored in a refrigerator overnight. The white solid that precipitated was filtered and washed with DCM to yield **1-(3,5-bis(trifluoromethyl)phenyl)-3-(3,4-dibromophenyl)thiourea.** White solid; yield 83% (330 mg, 0.632 mmol); mp: 140 – 141 °C; ^{1}H NMR (400 MHz, [D$_6$]DMSO): δ ppm 10.38 (br s, 2H, NH + NH″), 8.22 (s, 2H, H2 + H6), 7.94 (d, $^{4}J_{H2–H6}$ = 2.4 Hz, 1H, H2), 7.82 (br s, 1H, H4), 7.74 (d, $^{3}J_{H5–H6}$ = 8.7 Hz, 1H, H5), 7.40 (dd, $^{3}J_{H6–H5}$ = 8.7 Hz, $^{4}J_{H6–H2}$ = 2.4 Hz, 1H, H6); ^{13}C NMR (101 MHz, [D$_6$]DMSO): δ ppm 180.0 (s, CS), 141.5 (s, C1″), 139.4 (s, C1), 133.6 (s, C5), 130.1 (q, $^{2}J_{C-F}$ = 32.8 Hz, C3″ + C5″), 128.5 (s, C2), 124.8 (s, C6), 123.8 (br q, $^{3}J_{C-F}$ = 3.9 Hz, C2″ + C6″), 123.5 (s, C3), 119.3 (s, C4), 123.2 (q, $^{1}J_{C-F}$ = 272.7 Hz, 2 × CF$_3$), 117.3 (br septet, $^{3}J_{C-F}$ = 3.9 Hz, C4″); ^{19}F NMR (376 MHz, [D$_6$]DMSO): δ ppm −61.54 (s, 2 × CF$_3$); MS (m/z): [M−H, ^{79}Br^{79}Br, 40%]$^-$, 518.9, [M−H, ^{79}Br^{81}Br, 100%]$^-$, 520.9, [M−H, ^{81}Br^{81}Br, 30%]$^-$, 522.9.

1-(3,5-Bis(trifluoromethyl)phenyl)-3-(3-bromo-5-cyanophenyl)thiourea (NVR-104)

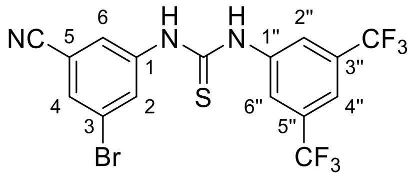

3,5-Bis(trifluoromethyl)phenyl isothiocyanate (550 mg, 2.5mmol) was added dropwise to a solution of 5-amino-3-bromobenzonitrile (200 mg, 1.0 mmol) in a mixture of anhydrous THF (3 mL) and toluene (5 mL) at room temperature under N$_2$. This mixture was stirred overnight at room temperature; then stored in a refrigerator overnight. The faint brown solid that precipitated was filtered and washed with DCM to yield 320 mg (64%) of **1-(3,5-bis(trifluoromethyl)phenyl)-3-(3-bromo-5-cyanophenyl)thiourea.** Beige solid; yield 64% (320 mg, 0.683 mmol); mp: 140 – 141 °C; ^{1}H NMR (500 MHz, [D$_6$]DMSO): δ ppm 10.54 (br s, 2H, NH + NH″), 8.21 (br s, 2H, H2″ + H6″), 8.04 (br s, 1H, H4), 7.97 (br s, 1H, H6), 7.94 (br s, 1H, H2), 7.86 (br s, 1H, H4″); MS (m/z): [M−H, ^{79}Br, 100%]$^-$, 466.0, [M−H, ^{81}Br, 85%]$^-$, 468.1.

1,3-Bis(3,5-bis(trifluoromethyl)phenyl)thiourea (NVR-72)

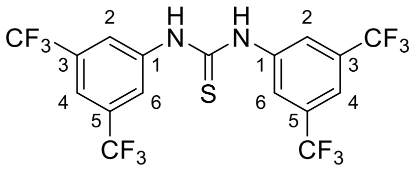

3,5-Bis(trifluoromethyl)phenyl isothiocyanate (350 mg, 1.3mmol) was added dropwise to a solution of 3,5-bis(trifluoromethyl)aniline (200 mg, 1.0mmol) in a mixture of anhydrous THF (3 mL) and toluene (5 mL) at room temperature under N_2. This mixture was stirred overnight at room temperature; then stored in a refrigerator overnight. The white solid that precipitated was filtered and washed with DCM to yield **1,3-bis(3,5-bis(trifluoromethyl)phenyl)thiourea.** White solid; yield 66% (290 mg, 0.580 mmol); mp: 160 – 162 °C (lit 170 -172 °C)[7]; ^{1}H NMR (400 MHz, [D$_6$]DMSO): δ ppm 10.65 (s, 2H, NH), 8.21 (s, 4H, H2 + H6), 7.83 (s, 2H, H4); MS (m/z): [M−H, 100%]$^-$, 499.1.

Methyl 3-(3-(3,5-bis(trifluoromethyl)phenyl)thioureido)-5-(trifluoromethyl)benzoate (NVR-138)

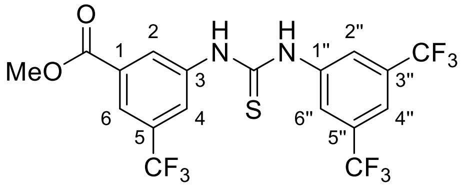

3,5-Bis(trifluoromethyl)phenyl isocyanate (620 mg, 2.0 mmol) was added dropwise to a solution of methyl 3-amino-5-(trifluoromethyl)benzoate (250 mg, 1.0 mmol) in a mixture of anhydrous THF (3 mL) and toluene (5 mL) at room temperature under N_2. This mixture was stirred overnight at room temperature; then stored in a refrigerator overnight. The white solid that precipitated was filtered and washed with DCM to yield **methyl 3-(3-(3,5-bis(trifluoromethyl)phenyl)thioureido)-5-(trifluoromethyl)benzoate.** White solid; yield 80% (432 mg, 0.881 mmol); mp: 161 – 162 °C; ^{1}H NMR (400 MHz, [D$_6$]DMSO): δ ppm 10.62 (s, 1H, NH), 10.59 (s, 1H, NH″), 8.34 (br s, 1H, H6), 8.23 (br s, 1H, H4), 8.21 (s, 2H, H2″ + H6″), 7.95 (br s, 1H, H2), 7.85 (br s, 1H, H4″), 3.90 (s, 3H, COOMe); ^{13}C NMR (101 MHz, [D$_6$]DMSO): δ ppm 180.5 (s, CS), 164.7 (s, COOMe), 141.2 (s, C1″), 140.7 (s, C3), 131.1 (s, C1), 130.3 (q, $^2J_{C-F}$ = 33.3 Hz, C3″ + C5″), 129.7 (q, $^2J_{C-F}$ = 32.3 Hz, C5), 127.9 (s, C2), 124.5 (br q, $^3J_{C-F}$ = 3.9 Hz, C6), 124.0

(br q, $^3J_{C\text{-}F}$ = 3.9 Hz, C2″+C6″), 121.2 (br q, $^3J_{C\text{-}F}$ = 3.9 Hz, C4), 123.4 (q, $^1J_{C\text{-}F}$ = 271.9 Hz, 5-CF_3), 123.2 (q, $^1J_{C\text{-}F}$ = 271.9 Hz, 2 × CF_3), 117.6 (br septet, $^3J_{C\text{-}F}$ = 3.9 Hz, C4″), 52.8 (s, MeOOC); ^{19}F NMR (376 MHz, [D$_6$]DMSO): δ ppm −61.57 (s, 2 × CF_3), −61.58 (s, CF_3); MS (m/z): [M−H, 100%]$^-$, 489.1.

1-(3,5-Bis(trifluoromethyl)phenyl)-3-(3,5-dichloro-4-fluorophenyl)thiourea (NVR-144)

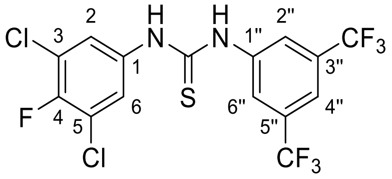

3,5-Bis(trifluoromethyl)phenyl isothiocyanate (890 mg, 3.3 mmol) was added dropwise to a solution of 3,5-dichloro-4-fluoroaniline (400 mg, 2.2 mmol) in a mixture of anhydrous THF (3 mL) and toluene (5 mL) at room temperature under N_2. This mixture was stirred overnight at room temperature; then stored in a refrigerator overnight. The white solid that precipitated was filtered and washed with DCM to yield **1-(3,5-bis(trifluoromethyl)phenyl)-3-(3,5-dichloro-4-fluorophenyl)thiourea.** White solid; yield 35% (340 mg, 0.754 mmol); mp: 158 – 159 °C; ^{1}H NMR (400 MHz, [D$_6$]DMSO): δ ppm 10.39 (s, 1H, NH), 10.37 (s, 1H, NH″), 8.21 (br s, 2H, H2″ + H6″), 7.84 (br s, 1H, H4″), 7.70 (d, $^4J_{H2/H6\text{–}F}$ = 6.3 Hz, 2H, H2 + H6); ^{13}C NMR (101 MHz, [D$_6$]DMSO): δ ppm 180.5 (s, CS), 150.6 (d, $^1J_{C\text{-}F}$ = 245.5 Hz, C4), 141.4 (s, C1″), 136.0 (s, C1), 130.1 (q, $^2J_{C\text{-}F}$ = 32.9 Hz, C3″ + C5″), 125.3 (s, C2 + C6), 124.1 (br q, $^3J_{C\text{-}F}$ = 3.9 Hz, C2″ + C6″), 120.6 (d, $^2J_{C\text{-}F}$ = 18.6 Hz, C3 + C5), 123.2 (q, $^1J_{C\text{-}F}$ = 272.4 Hz, 2 × CF_3), 117.5 (br septet, $^3J_{C\text{-}F}$ = 3.9 Hz, C4″); ^{19}F NMR (376 MHz, [D$_6$]DMSO): δ ppm −61.53 (s, 2 × CF_3),−122.02 (t, $^4J_{H2/H6\text{-}F}$ = 6.1 Hz, F); MS (m/z): [M−H, ^{35}Cl^{35}Cl, 90%]$^-$, 449.0, [M−H, ^{35}Cl^{37}Cl, 100%]$^-$, 451.0, [M−H, ^{37}Cl^{37}Cl, 20%]$^-$, 453.0.

3-(3,5-Bis(trifluoromethyl)phenyl)-7-chloroquinazoline-2,4(1*H*,3*H*)-dione (NVR-66)

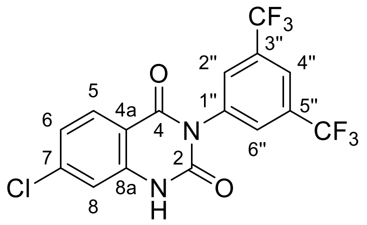

2-(3-(3,5-Bis(trifluoromethyl)phenyl)ureido)-4-chlorobenzoic acid (170 mg, 0.40 mmol, 1 eq) was stirred in concentrated H_2SO_4 (20 mL) at room temperature for 24 h. The reaction mixture was poured into a mixture of ice and distilled water, and neutralised with a saturated solution of $NaHCO_3$. The subsequent precipitate was isolated by filtration and washed with diethyl ether then ethanol before being dried to yield **3-(3,5-bis(trifluoromethyl)phenyl)-7-chloroquinazoline-2,4(1*H*,3*H*)-dione.** Beige solid; yield 57% (92 mg, 0.225 mmol); mp: >250 °C; ^{1}H NMR (500 MHz, [D$_6$]DMSO): δ ppm 11.85 (s, 1H, NH), 8.24 (s, 3H, H2″ + H4″ + H6″), 7.96 (d, $^{3}J_{H5\text{-}H6}$ = 8.5 Hz, 1H, H5), 7.31 (dd, $^{3}J_{H6\text{-}H5}$ = 8.5 Hz, $^{4}J_{H6\text{-}H8}$ = 1.9 Hz, 1H, H6), 7.28 (d, $^{4}J_{H8\text{-}H6}$ = 1.8 Hz, 1H, H8); ^{13}C NMR (126 MHz, [D$_6$]DMSO): δ ppm 161.5 (s, C4), 149.8 (s, C2), 140.9 (s, C8a), 139.8 (s, C7), 137.7 (s, C1″), 130.8 (br q, $^{3}J_{C\text{-}F}$ = 3.9 Hz, C2″ + C6″), 130.9 (q, $^{2}J_{C\text{-}F}$ = 33.7 Hz, C3″ + C5″), 129.6 (s, C5), 122.9 (s, C6), 123.0 (q, $^{1}J_{C\text{-}F}$ = 272.9 Hz, 2 × CF_3), 122.4 (br septet, $^{3}J_{C\text{-}F}$ = 3.9 Hz, C4″), 114.7 (s, C4″), 113.3 (s, C4a); MS (m/z): [M+H, ^{35}Cl, 100%]$^{+}$, 409.0, [M+H, ^{37}Cl, 32%]$^{+}$, 411.0; HRMS (m/z): [M+H]$^{+}$ calcd. for $C_{16}H_8O_2N_2{}^{35}ClF_6$, 409.0173; found, 409.0180; error, 1.71 ppm.

7-Chloro-3-(4-chloro-3-(trifluoromethyl)phenyl)quinazoline-2,4(1*H*,3*H*)-dione (NVR-91)

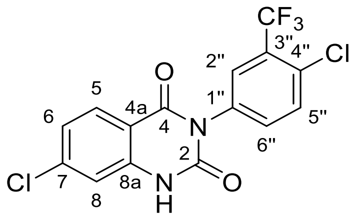

A solution of 7-chloro-2-((4-chloro-3-(trifluoromethyl)phenyl)amino)-4*H*-benzo[d][1,3]oxazin-4-one (187 mg, 0.498 mmol, 1 eq) in formamide (20 mL) was heated to reflux with stirring at 220 °C for 2 h. The reaction mixture was poured into a mixture of ice and distilled water, before the product was extracted with EtOAc. Solvent was removed *in vacuo* to yield **7-chloro-3-(4-chloro-3-(trifluoromethyl)phenyl)quinazoline-2,4(1*H*,3*H*)-**

dione. Yellow solid; yield 59% (110 mg, 0.293 mmol); mp: > 250 °C; ^{1}H NMR (500 MHz, [D$_6$]DMSO): δ ppm 11.79 (br s, 1H, NH), 7.99 (d, $^4J_{H2''-H6''}$ = 2.3 Hz, 1H, H2''), 7.94 (d, $^3J_{H5-H6}$ = 8.4 Hz, 1H, H5), 7.88 (d, $^3J_{H5''-H6''}$ = 8.4 Hz, 1H, H5''), 7.73 (dd, $^3J_{H6-H5}$ = 8.5 Hz, $^4J_{H6-H8}$ = 2.4 Hz, 1H, H6), 7.28 (dd, $^3J_{H6''-H5''}$ = 8.5 Hz, $^4J_{H6''-H2''}$ = 2.0 Hz, 1H, H6''), 7.25 (d, $^4J_{H8-H6}$ = 1.8 Hz, 1H, H8); ^{13}C NMR (126 MHz, [D$_6$]DMSO): δ ppm 161.5 (s, C4), 149.9 (s, C2), 140.9 (s, C8a), 139.7 (s, C7), 135.2 (s, C1''), 135.0 (s, C5), 132.3 (s, C6''), 130.6 (br q, $^3J_{C-F}$ = 3.9 Hz, C5''), 129.6 (s, C2''), 129.0 (q, $^3J_{C-F}$ = 5.2 Hz, C4''), 127.1 (q, $^2J_{C-F}$ = 31.1 Hz, C3''), 122.8 (s, C6), 122.5 (q, $^1J_{C-F}$ = 273.1 Hz, CF_3), 114.6 (s, C4a), 113.3 (s, C8); MS (m/z): [M+H, ^{35}Cl^{35}Cl, 100%]$^+$, 375.0, [M+H, ^{35}Cl^{37}Cl, 64%]$^+$, 377.0, [M+H, ^{37}Cl^{37}Cl, 16%]$^+$, 379.0; HRMS (m/z): [M+H]$^+$ calcd. for $C_{15}H_8O_2N_2{}^{35}Cl_2F_3$, 374.9909; found, 374.9916; error, 1.75 ppm.

7-Chloro-3-(3-(trifluoromethyl)phenyl)quinazoline-2,4(1*H*,3*H*)-dione (NVR-92)

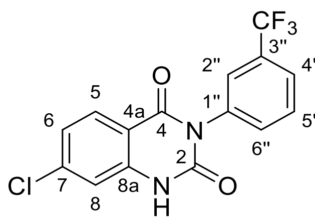

A solution of 7-chloro-2-((3-(trifluoromethyl)phenyl)amino)-4H-benzo[d][1,3]oxazin-4-one (120 mg, 0.352 mmol, 1 eq) in formamide (20 mL) was heated to reflux with stirring at 220 °C for 2 h. The reaction mixture was poured into a mixture of ice and distilled water, before the product was extracted with EtOAc. Solvent was removed *in vacuo* to yield **7-chloro-3-(3-(trifluoromethyl)phenyl)quinazoline-2,4(1*H*,3*H*)-dione.** Beige solid; yield 35% (42 mg, 0.123 mmol); mp: >250 °C; ^{1}H NMR (500 MHz, [D$_6$]DMSO): δ ppm 7.93 (s, 1H, NH), 7.78 - 7.83 (m, 2H, H2'' + H8), 7.73 (t, $^3J_{H5''-H4''}$ = $^3J_{H5''-H6''}$ = 8.1 Hz, 1H, H5''), 7.67 (br d, $^3J_{H6''-H5''}$ = 7.9 Hz, 1H, H6''), 7.25 - 7.30 (m, 3H, H5 + H6 + H4''); ^{13}C NMR (126 MHz, [D$_6$]DMSO): δ ppm 161.8 (s, 1C, C4), 150.2 (s, C2), 141.1 (s, C8a), 139.9 (s, C7), 136.5 (s, C1''), 133.7 (s, 1C, C6''), 130.3 (s, C5''), 129.8 (s, C5), 126.3 (q, $^3J_{C-F}$ = 3.9 Hz, 1C, C2''), 125.3 (q, $^3J_{C-F}$ =3.9 Hz, C4''), 123.0 (s, 1C, C6), 114.8 (s, 1C, C8), 113.5 (s, 1C, C4a), CF_3 and C3'' in the noise; MS (m/z): [M−H, ^{35}Cl, 100%]$^-$, 339.0, [M−H, ^{37}Cl, 32%]$^-$, 341.0; HRMS (m/z): [M+H]$^+$ calcd. for $C_{15}H_9O_2N_2{}^{35}ClF_3$, 341.0299; found, 341.0298; error, −0.34 ppm.

3-(4-Chloro-3-(trifluoromethyl)phenyl)-7-methoxyquinazoline-2,4(1*H*,3*H*)-dione (NVR-93)

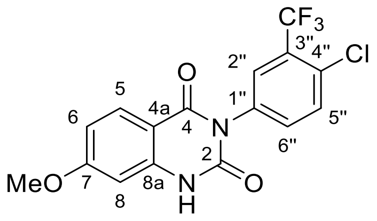

A solution of 2-((4-chloro-3-(trifluoromethyl)phenyl)amino)-7-methoxy-4H-benzo[d][1,3]oxazin-4-one (20 mg, 0.054 mmol, 1 eq) in formamide (20 mL) was heated to reflux with stirring at 220 °C for 2 h. The reaction mixture was poured into a mixture of ice and distilled water, before the product was extracted with EtOAc. Solvent was removed *in vacuo* to yield **3-(4-chloro-3-(trifluoromethyl)phenyl)-7-methoxyquinazoline-2,4(1*H*,3*H*)-dione.** Grey solid; yield 15% (2.9 mg, 0.008 mmol); mp: >250 °C; ^{1}H NMR (500 MHz, [D$_6$]DMSO): δ ppm 11.49 (br s, 1H, NH), 7.97 (d, $^4J_{H2''-H6''}$ = 2.1 Hz, 1H, H2''), 7.86 (br d, $^3J_{H5-H6}$ = 8.4 Hz, 1H, H5), 7.86 (br d, $^3J_{H5''-H6''}$ = 8.9 Hz, 1H, H5''), 7.72 (dd, $^3J_{H6''-H5''}$ = 8.5 Hz, $^4J_{H6''-H2''}$ = 2.1 Hz, 1H, H6''), 6.84 (dd, $^3J_{H6-H5}$ = 8.9 Hz, $^4J_{H6-H8}$ = 2.1 Hz, 1H, H6), 6.69 (d, $^4J_{H8-H6}$ = 2.3 Hz, 1H, H8), 3.86 (s, 3H, OMe); ^{13}C NMR (126 MHz, [D$_6$]DMSO): δ ppm 164.7 (s, C7), 161.6 (s, C4), 150.2 (s, C2), 141.8 (s, C8a), 135.3 (s, C1''), 132.1 (s, C6''), 130.3 (s, C5''), 129.5 (s, C2''), 129.2 (br q, $^3J_{C-F}$ = 5.2 Hz, C4''), 115.0 (s, C4a), 110.8 (s, C5), 107.6 (s, C6), 98.3 (s, C8), 55.7 (s, OMe), C3'' and CF$_3$ lost in the noise. MS (m/z): [M+H, ^{35}Cl, 100%]$^+$, 371.0, [M+H, ^{37}Cl, 32%]$^+$, 373.0; HRMS (m/z): [M+H]$^+$ calcd. for $C_{16}H_{11}O_3N_2{}^{35}ClF_3$, 371.0405 found, 371.0407; error, 0.59 ppm.

3-(3,5-Bis(trifluoromethyl)phenyl)-6-fluoroquinazoline-2,4(1*H*,3*H*)-dione (NVR-125)

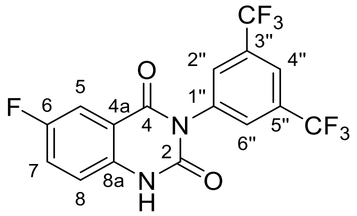

3,5-Bis(trifluoromethyl)phenyl isocyanate (0.40 mL, 2.32 mmol, 1.2 eq) was added dropwise to a stirred solution of 2-amino-4-fluorobenzoic acid (300 mg, 1.93 mmol, 1.0 eq) in acetonitrile (25 mL). The reaction was stirred for 16 hours at room temperature. The precipitate was filtered and then a small portion (≈50 mg) triturated with hot toluene. The filtrate was concentrated *in vacuo* to yield **3-(3,5-bis(trifluoromethyl)phenyl)-6-**

fluoroquinazoline-2,4(1*H*,3*H*)-dione. White solid; yield 1% (8 mg, 0.02 mmol). ^{1}H NMR (300 MHz, [D$_6$]DMSO): δ ppm 11.00 (s, 1H, NH), 8.47 (s, 2H, H2″ + H6″), 7.78 (s, 1H, H4″), 7.73 - 7.77 (m, 1H, H5), 7.69 (dd, $^{3}J_{H8-H7}$ = 8.7 Hz, $^{4}J_{H8-F}$ = 3.2 Hz, 1H, H8), 7.46 (dd, $^{3}J_{H7-H8}$ = 8.8 Hz, $^{3}J_{H7-F}$ = 4.8 Hz, 1H, H7).

6-Chloro-2-thioxo-3-(3-(trifluoromethyl)phenyl)-2,3-dihydroquinazolin-4(1*H*)-one (NVR-159)

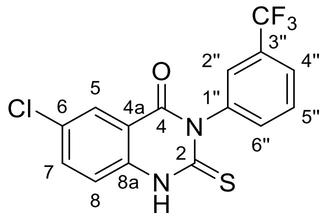

To 3-trifluoromethylphenyl thioisocyanate (0.25 mL, 1.68 mmol, 1.15 eq) was added a stirred solution of 5-chloroanthranilic acid (250 mg, 1.46 mmol, 1 eq) in dry THF (10 mL). The reaction mixture was heated to reflux with stirring at 85 °C for 16 h. Solvent was removed *in vacuo*, and the resultant solid residue was washed with DCM. To the residue was added 0.1M HCl (2 mL), followed by 2 mL trifluoroacetic acid. The product was extracted with EtOAc and the organic layer concentrated *in vacuo*, washed with minimal DCM and hexane to yield the desired product. Further product was obtained after removing solvent from filtrate *in vacuo*, before washing the solid residue with minimal toluene and THF to furnish **6-chloro-2-thioxo-3-(3-(trifluoromethyl)phenyl)-2,3-dihydroquinazolin-4(1*H*)-one.** White solid; yield 19% (100 mg, 0.280 mmol); mp: >250 °C; ^{1}H NMR (300 MHz, $CDCl_3$) δ ppm 10.75 (br s, 1H, NH), 8.14 (br s, 1H, H5), 7.78 (br d, $^{3}J_{H7-H8}$ = 7.2 Hz, 1H, H7), 7.60 - 7.74 (m, 2H, H2″+ H5″), 7.56 (br s, 1H, H8), 7.48 (br d, $^{3}J_{H6''-H5''}$ = 7.2 Hz, 1H, H6″), 7.14 (br d, $^{3}J_{H4''-H5''}$ = 8.9 Hz, 1H, H4″); ^{13}C NMR (126 MHz, $CDCl_3$) δ ppm 176.2 (s, C2), 158.8 (s, C4), 138.6 (s, C8a), 137.2 (s, C1″), 136.3 (s, C5″), 132.1 (s, C6″), 132.2 (br q, $^{2}J_{C-F}$ = 33.3 Hz, C3″), 131.0 (s, C5), 130.2 (s, C6), 128.3 (s, C7), 126.1 (br q, $^{3}J_{C-F}$ = 2.9 Hz, C2″), 125.9 (br q, $^{3}J_{C-F}$ = 2.9 Hz, C4″), 123.5 (q, $^{1}J_{C-F}$ = 272.9 Hz, CF_3), 117.4 (s, C8), 116.4 (s, C4a); MS (m/z): [M+H, ^{35}Cl, 100%]$^+$, 357.2, [M+H, ^{37}Cl, 32%]$^+$, 359.2; HRMS (m/z): [M+H]$^+$ calcd. for $C_{15}H_9O_2N_2S^{35}ClF_3$, 357.0071; found, 357.0075; error, 1.20 ppm.

2-((4-Chloro-3-(trifluoromethyl)phenyl)amino)-7-methoxy-4H-benzo[d][1,3]oxazin-4-one (NVR-65)

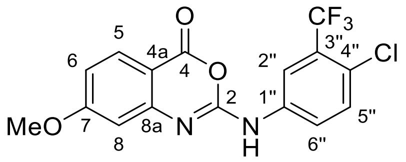

2-(3-(4-Chloro-3-(trifluoromethyl)phenyl)ureido)-4-methoxybenzoic acid (340 mg, 0.88 mmol, 1 eq) was stirred in concentrated H_2SO_4 (20 mL) at room temperature for 24 h. The reaction mixture was poured into a mixture of ice and distilled water, and neutralised with a saturated solution of $NaHCO_3$. The subsequent precipitate was isolated by filtration and washed with diethyl ether then ethanol before being dried to yield **2-((4-chloro-3-(trifluoromethyl)phenyl)amino)-7-methoxy-4H-benzo[d][1,3]oxazin-4-one.** Brown solid; yield 49% (160 mg, 0.43 mmol); mp: 244 °C; ^{1}H NMR (500 MHz, [D$_6$]DMSO): δ ppm 8.26 (d, $^4J_{H2''-H6''}$ = 2.4 Hz, 1H, H2”), 8.10 (dd, $^3J_{H6''-H5''}$ = 8.8 Hz, $^4J_{H6''-H2''}$ = 2.4 Hz, 1H, H6”), 7.88 (d, $^3J_{H5-H6}$ = 8.9 Hz, 1H, H5), 7.69 (d, $^3J_{H5''-H6''}$ = 8.9 Hz, 1H, H5”), 6.89 (dd, $^3J_{H6-H5}$ = 8.9 Hz, $^4J_{H6-H8}$ = 2.4 Hz, 1H, H6), 6.79 (d, $^4J_{H8-H6}$ = 2.4 Hz, 1H, H8), 3.89 (s, 3H, OMe); MS (m/z): [M+H, ^{35}Cl, 100%]$^+$, 371.0, [M+H, ^{37}Cl, 32%]$^+$, 373.0; HRMS (m/z): [M+H]$^+$ calcd. for $C_{16}H_{11}O_3N_2{}^{35}ClF_3$, 371.0405; found, 371.0412; error, 1.94 ppm.

7-(*tert*-Butyl)-2-((4-chloro-3-(trifluoromethyl)phenyl)amino)-4*H*-benzo[d][1,3]oxazin-4-one (NVR-68)

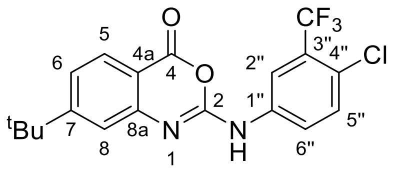

4-(*tert*-Butyl)-2-(3-(4-chloro-3-(trifluoromethyl)phenyl)ureido)benzoic acid (740 mg, 1.784 mmol, 1 eq) was stirred in concentrated H_2SO_4 (20 mL) at room temperature for 72 h. The reaction mixture was poured into a mixture of ice and distilled water, and neutralised with a saturated solution of $NaHCO_3$. The subsequent precipitate was isolated by filtration and washed with diethyl ether then ethanol before being dried to yield **7-(*tert*-butyl)-2-((4-chloro-3-(trifluoromethyl)phenyl)amino)-4*H*-benzo[d][1,3]oxazin-4-one.** Beige solid; yield 25%

(180 mg, 0.454 mmol); mp: >250 °C; ^{1}H NMR (400 MHz, [D$_6$]DMSO): δ ppm 10.68 (br s, 1H, NH), 8.30 (d, $^4J_{H2''-H6''}$ = 2.5 Hz, 1H, H2''), 8.16 (dd, $^3J_{H6''-H5''}$ = 8.9 Hz, $^4J_{H6''-H2''}$ = 2.4 Hz, 1H, H6''), 7.93 (d, $^3J_{H5-H6}$ = 8.4 Hz, 1H, H5), 7.72 (d, $^3J_{H5''-H6''}$ = 8.8 Hz, 1H, H5''), 7.44 (dd, $^3J_{H6-H5}$ = 8.4 Hz, $^4J_{H6-H8}$ = 1.9 Hz, 1H, H6), 7.32 (d, $^4J_{H8-H6}$ = 1.7 Hz, 1H, H8), 1.33 (s, 9H, ^{t}Bu); MS (m/z): [M+H, ^{35}Cl, 100%]$^+$, 397.0, [M+H, ^{37}Cl, 32%]$^+$, 399.0; HRMS (m/z): [M+H]$^+$ calcd. for $C_{19}H_{17}O_2N_2{}^{35}ClF_3$, 397.0925; found, 397.0920; error, −1.30 ppm.

6-Chloro-2-((3-(trifluoromethyl)phenyl)amino)-4*H*-benzo[d][1,3]oxazin-4-one (NVR-160)

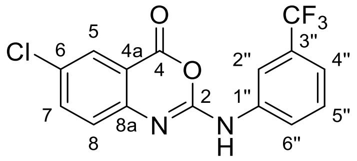

To 3-trifluoromethylphenyl thioisocyanate (0.255 mL, 1.68 mmol, 1.15 eq) was added a stirred solution of 5-chloroanthranilic acid (250 mg, 1.46 mmol, 1 eq) in dry THF (10 mL). The reaction mixture was heated to reflux with stirring at 85 °C for 16 h. Due to unconsumed starting material identified by TLC, a further portion of 3-trifluoromethylphenyl thioisocyanate (0.2 mL, 1.32 mmol, 1.1 eq) was added, and the reaction heated at 85 °C until TLC indicated reaction completion. Solvent was removed from the reaction mixture *in vacuo*, and the resultant solid residue was filtered and washed with minimal MeCN to yield **6-chloro-2-((3-(trifluoromethyl)phenyl)amino)-4*H*-benzo[d][1,3]oxazin-4-one.** White solid; yield 4% (21 mg, 0.062 mmol); mp: 233 – 234 °C; ^{1}H NMR (300 MHz, [D$_6$]DMSO): δ ppm 10.74 (s, 1H, NH), 8.24 (s, 1H, H2''), 8.01 (br d, $^3J_{H4''-H5''}$ = 8.1 Hz, 1H, H4''), 7.93 (d, $^4J_{H5-H7}$ = 2.5 Hz, 1H, H5), 7.80 (dd, $^3J_{H7-H8}$ = 8.7 Hz, $^4J_{H7-H5}$ = 2.5 Hz, 1H, H7), 7.61 (t, $^3J_{H5''-H4''}$ = $^3J_{H5''-H6''}$ = 8.0 Hz, 1H, H5''), 7.43 (d, $^3J_{H6''-H5''}$ = 7.8 Hz, 1H, H6''), 7.40 (d, $^3J_{H8-H7}$ = 8.8 Hz, 1H, H8); ^{13}C NMR (126 MHz, [D$_6$]DMSO): δ ppm 158.0 (s, C4), 150.6 (s, C2), 147.7 (s, C8a), 138.8 (s, C1''), 136.6 (s, C7), 130.0 (s, C6), 129.6 (q, $^2J_{C-F}$ = 31.5 Hz, C3''), 128.2 (s, C5), 126.9 (q, $^4J_{C-F}$ = 2.0 Hz, C5''), 126.8 (s, C8), 122.9 (s, C6''), 124.1 (q, $^1J_{C-F}$ = 272.1 Hz, CF$_3$), 119.4 (br q, $^3J_{C-F}$ = 3.9 Hz, C2''), 115.7 (s, C4a), 115.4 (br q, $^3J_{C-F}$ = 3.9 Hz, C4''); MS (m/z): [M+H, ^{35}Cl, 100%]$^+$, 341.2, [M+H, ^{37}Cl, 32%]$^+$, 343.2; HRMS (m/z): [M+H]$^+$ calcd. for $C_{15}H_9O_2N_2{}^{35}ClF_3$, 341.0299; found, 341.0305; error, 1.71 ppm.

2-(Phenethylamino)benzoic acid (Tromaril)

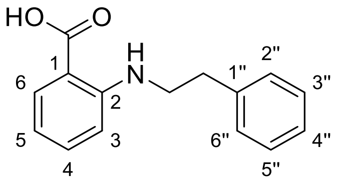

A solution of anthranilic acid (226.0 mg, 1.65 mmol, 1 eq) and phenylacetaldehyde (198.2 mg, 1.65 mmol, 1 eq) in anhydrous methanol (5 mL) was stirred for 30 min at room temperature. To this was added a stirred suspension of zinc chloride (260 mg, 1.91 mmol, 1.15 eq) and sodium cyanoborohydride (207 mg, 5.4 mmol, 3.3 eq) in anhydrous methanol (5 mL), before the reaction mixture was stirred at room temperature for 20 h. The solution was concentrated *in vacuo* and purified by flash column chromatography (hexane:ethyl acetate, 9:1). Appropriate fractions were combined and solvent removed *in vacuo* to yield **2-(phenethylamino)benzoic acid.** Yellow solid; yield 5% (20.2 mg, 0.084 mmol); mp: 134 – 135 °C (lit 182 -183 °C)[8]; ^{1}H NMR (300 MHz, $CDCl_3$): δ ppm 7.95 (dd, $^{3}J_{H6\text{-}H5}$ = 8.1 Hz, $^{4}J_{H6\text{-}H4}$ = 1.5 Hz, 1H, H6), 7.16 - 7.40 (m, 6H, H4 + H2″ + H3″ + H4″ + H5″ + H6″), 6.69 (d, $^{3}J_{H3\text{-}H4}$ = 8.5 Hz, 1H, H3), 6.59 (br t, $^{3}J_{H5\text{-}H3}$ = $^{3}J_{H5\text{-}H4}$ = 7.5 Hz, 1H, H5), 3.45 (t, $^{3}J_{HH}$ = 7.2 Hz, 2H, C$\underline{H}_2$NH), 2.96 (t, $^{3}J_{HH}$ = 7.2 Hz, 2H, CH_2Ph); ^{13}C NMR (75 MHz, $CDCl_3$) δ ppm 173.0 (s, COOH), 151.5 (s, C2), 139.1 (s, C1″), 135.6 (s, C4), 132.6 (s, C6), 128.8 (s, C2″ + C6″), 128.6 (s, C3″ + C5″), 126.5 (s, C4″), 114.7 (s, C5), 111.3 (s, C3), 108.6 (s, C1), 44.4 (s, CH_2NH), 35.5 (s, CH_2Ph); MS (m/z): [M−H, 100%]$^{-}$, 240.0.

2-(Phenylcarbamoyl)benzoic acid (NVR-53)

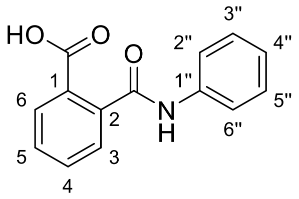

Aniline (208 mg, 2.29 mmol, 1.1 eq) was added to a stirred solution of phthalic anhydride (300 mg, 2.025 mmol, 1 eq) in EtOAc (5 mL). The reaction mixture was stirred at room temperature for 3 h. The reaction mixture was then extracted with 3 × 5 mL 0.1M NaOH, before the combined aqueous layers were acidified with concentrated HCl, precipitating a white solid which was dried to yield **2-(phenylcarbamoyl)benzoic acid.** White solid; yield 43% (211 mg, 0.875 mmol); mp: 209 – 210 °C; ^{1}H NMR (400 MHz, [D$_6$]DMSO): δ ppm 13.00 (br s, 1H, COOH), 10.32 (s, 1H, CONH), 7.88 (dd, $^3J_{H6\text{-}H5}$ = 7.8 Hz, $^4J_{H6\text{-}H4}$ = 0.8 Hz, 1H, H6), 7.69 (br d, $^3J_{H2''/H6''\text{-}H3''/H5''}$ = 7.5 Hz, 2H, H2″ + H6″), 7.65 (dd, $^3J_{H3\text{-}H4}$ = 7.4 Hz, $^4J_{H3\text{-}H5}$ = 1.1 Hz, 1H, H3), 7.57 (td, $^3J_{H5\text{-}H4}$ = $^3J_{H5\text{-}H6}$ = 7.5 Hz, $^4J_{H5\text{-}H3}$ = 1.0 Hz, 1H, H5), 7.55 (t, $^3J_{H4\text{-}H3}$ = $^3J_{H4\text{-}H5}$ = 7.3 Hz, 1H, H4), 7.33 (t, $^3J_{H3''/H5''\text{-}H2''/H6''}$ = 7.9 Hz, 2H, H3″ + H5″), 7.07 (t, $^3J_{H4''\text{-}H3''/H5''}$ = 7.3 Hz, 1H, H4″); ^{13}C NMR (101 MHz, [D$_6$]DMSO): δ ppm 167.5 (s, COOH), 167.4 (s, CONH), 139.6 (s, C1″), 138.9 (s, C2), 131.7 (s, C6), 129.9 (s, C1), 129.4 (s, C4), 128.6 (s, C3″ + C5″), 127.8 (s, C3), 123.3 (s, C4″), 119.5 (s, C2″+ C6″).

2-(3-Phenylureido)benzoic acid (NVR-54)

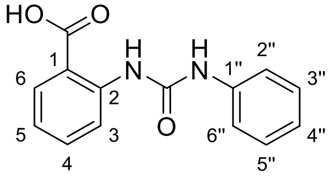

To a stirred solution of anthranilic acid (300 mg, 2.19 mmol, 1 eq) in MeCN (5 mL) was added phenyl isocyanate (0.290 mL, 316 mg, 2.65 mmol, 1.2 eq). The reaction mixture was stirred at room temperature for 3 h. The resultant precipitate was filtered and washed with minimal MeCN before being dried to yield **2-(3-phenylureido)benzoic acid.** White solid; yield 57% (317 mg, 1.23 mmol); mp: 174 – 175 °C (lit 168 – 169 °C)[9]; ^{1}H NMR (400 MHz, [D$_6$]DMSO): δ ppm 13.37 (br s, 1H, COOH), 10.34 (s, 1H, NH), 9.78 (s, 1H, NH″), 8.36 (d, $^3J_{H3\text{-}H4}$ = 8.3 Hz, 1H, H3), 7.95 (dd, $^3J_{H6\text{-}H5}$ = 7.8 Hz, $^4J_{H6\text{-}H4}$ = 1.5 Hz, 1H, H6), 7.54 (ddd, $^3J_{H4\text{-}H3}$ = 8.5 Hz, $^3J_{H4\text{-}H5}$ = 7.3 Hz,

$^{4}J_{H4-H6}$ = 1.5 Hz, 1H, H4), 7.51 (d, $^{3}J_{H2''/H6''-H3''/H5''}$ = 7.3 Hz, 2H, H2″+ H6″), 7.28 (t, $^{3}J_{H3''/H5''-H2''/H6''}$ = 7.9 Hz, 2H, H3″ + H5″), 7.04 (t, $^{3}J_{H5-H4}$ = $^{3}J_{H5-H6}$ = 7.7 Hz, 1H, H5), 6.98 (t, $^{3}J_{H4''-H3''/H5''}$ = 7.3 Hz, 1H, H4″); ^{13}C NMR (101 MHz, [D$_6$]DMSO): δ ppm 169.4 (s, COOH), 152.3 (s, CO), 142.2 (s, C2), 139.7 (s, C1″), 133.7 (s, C4), 131.0 (s, C6), 128.7 (s, C3″ + C5″), 122.1 (s, C5), 120.9 (s, C4″), 119.8 (s, C3), 118.8 (s, C2″ + C6″), 115.4 (s, C1).

2-((Phenoxycarbonyl)amino)benzoic acid (NVR-60)

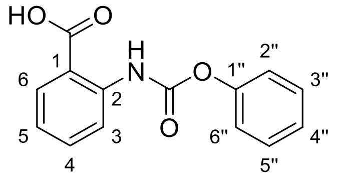

Phenyl chloroformate (380 mg, 2.43 mmol, 1.1 eq) was added to a stirred solution of anthranilic acid (300 mg, 2.19 mmol, 1 eq) in THF (5 mL). The reaction mixture was stirred at room temperature for 1 h after which time a white precipitate formed. Solvent was removed *in vacuo* prior to taking up the residue in EtOAc (10 mL). The product was extracted with 3 × 5 mL 0.1M NaOH, before the combined aqueous layers were acidified with concentrated HCl, precipitating a beige solid which was dried to yield **2-((phenoxycarbonyl)amino)benzoic acid.** Beige solid; yield 40% (228 mg, 0.882 mmol); mp: decomposition at 137 °C; ^{1}H NMR (400 MHz, [D$_6$]DMSO): δ ppm 13.07 (br s, 1H, COOH), 9.65 (br s, 1H, CONH), 7.91 (dd, $^{3}J_{H6-H5}$ = 7.9 Hz, $^{4}J_{H6-H4}$ = 1.6 Hz, 1H, H6), 7.31 - 7.41 (m, 3H, H3 + H3″ + H5″), 7.20 - 7.28 (m, 3H, H4 + H2″ + H6″), 7.07 (tt, $^{3}J_{H4''-H3''/H5''}$ = 7.3 Hz, $^{4}J_{H4''-H2''/H6''}$ = 1.1 Hz, 1H, H4″), 6.78 (td, $^{3}J_{H5-H4}$ = $^{3}J_{H5-H6}$ =7.5 Hz, $^{4}J_{H5-H3}$ = 1.0 Hz, 1H, H5); ^{13}C NMR (101 MHz, [D$_6$]DMSO): δ ppm 170.0 (s, COOH), 147.0 (s, C1″ + CONH), 140.5 (s, C2), 134.2 (s, C4), 131.9 (s, C6), 129.5 (s, C3″ + C5″), 123.1 (s, C4″), 121.4 (s, C2″+ C6″), 117.4 (s, C5), 113.7 (s, C3), 112.6 (s, C1).

2-(5-Phenyl-1,3,4-oxadiazol-2-yl)benzoic acid (NVR-59)

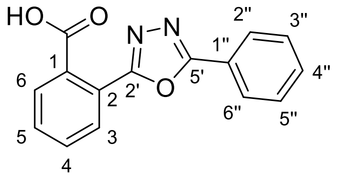

A mixture of methyl hydrogen phthalate (500 mg, 2.78 mmol, 1 eq) and $SOCl_2$ (1.5 mL) was heated to reflux with stirring at 75 °C. Upon cooling, the excess $SOCl_2$ was removed *in vacuo* to yield a residue which was added dropwise to a stirred 0 °C solution of 5-phenyl-1*H*-tetrazole (406 mg, 2.78 mmol, 1 eq) and pyridine (3 mL). The reaction mixture was heated to 150 °C with stirring for 2 h, after which solvent was removed *in vacuo*. The reaction mixture was diluted with EtOAc (5 mL) and H_2O (5 mL), the product was extracted with 2 × 5 mL EtOAc, and solvent from the combined organic layers was removed *in vacuo*. The subsequent residue was dissolved in a 9:1 mixture of MeOH, H_2O (10 mL), before addition of 0.2M NaOH (3 mL). The reaction was stirred overnight before solvent was removed *in vacuo*. The product was extracted with 0.1M NaOH (3 × 3 mL) and the combined aqueous layers acidified with concentrated HCl and the subsequent precipitate filtered and dried to yield **2-(5-phenyl-1,3,4-oxadiazol-2-yl)benzoic acid.** White solid; yield 5% (38 mg, 0.14 mmol); mp: 151 – 152 °C; ^{1}H NMR (400 MHz, [D$_6$]DMSO): δ ppm 13.68 (br s, 1H, COOH), 8.04 (dd, $^3J_{H2''/H6''-H3''/H5''}$ = 7.9 Hz, $^4J_{H2''/H6''-H4''}$ = 1.6 Hz, 2H, H2″ + H6″), 7.89 - 7.97 (m, 2H, H3 + H6), 7.74 - 7.81 (m, 2H, H4 + H4″), 7.60 - 7.68 (m, 3H, H5 + H3″+ H5″); ^{13}C NMR (101 MHz, [D$_6$]DMSO): δ ppm 167.7 (s, COOH), 164.3 (s, C2′), 163.9 (s, C5′), 132.9 (s, C1), 132.1 (s, C4), 131.9 (s, C6), 131.7 (s, C4″), 130.5 (s, C5), 129.7 (s, C3), 129.5 (s, C3″+ C5″), 126.5 (s, C2″ + C6″), 123.3 (s, C2), 123.0 (s, C1″).

3-((3,5-Bis(trifluoromethyl)phenyl)amino)-1*H*-benzo[c][1,5,2]oxazaborinin-1-ol (NVR-154)

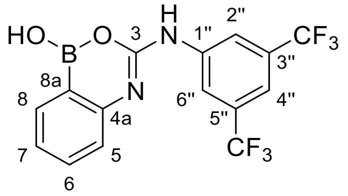

To a stirred solution of (2-aminophenyl)boronic acid (200 mg, 1.46 mmol, 1 eq) in MeCN (50 mL) was added 3,5-bis(trifluoromethyl)phenyl isocyanate (0.303 mL, 447 mg, 1.75 mmol, 1.2 eq). The reaction mixture was stirred at room temperature for 24 h, before the resultant

precipitate was filtered, washed with minimal MeCN and dried to yield **3-((3,5-bis(trifluoromethyl)phenyl)amino)-1*H*-benzo[c][1,5,2]oxazaborinin-1-ol.** White solid; yield 86% (472 mg, 1.26 mmol); mp: 168 - 170 °C; ^{1}H NMR (400 MHz, [D$_6$]DMSO): δ ppm 10.59 (s, 1H, BOH), 9.40 (br s, 1H, NH), 8.06 (s, 1H, H4″), 8.02 (s, 2H, H2″ + H6″), 7.99 (br d, $^3J_{H8-H7}$ = 7.5 Hz, 1H, H8), 7.50 (ddd, $^3J_{H6-H5}$ = 8.2 Hz, $^3J_{H6-H7}$ = 7.1, $^4J_{H6-H8}$ = 1.3 Hz, 1H, H6), 7.11 (d, $^3J_{H5-H6}$ = 8.0 Hz, 1H, H5), 7.07 (t, $^3J_{H7-H6}$ = $^3J_{H7-H8}$ = 7.3 Hz, 1H, H7); ^{13}C NMR (101 MHz, [D$_6$]DMSO): δ ppm 153.7 (s, C3), 145.4 (s, C4a), 141.5 (s, C1″), 132.8 (s, C6), 132.7 (s, C8), 130.4 (br q, $^3J_{C-F}$ = 2.9 Hz, C2″ + C6″), 130.3 (q, $^2J_{C-F}$ = 32.9 Hz, C3″ + C5″), 121.0 (s, C7), 120.2 (br septet, $^3J_{C-F}$ = 2.9 Hz, C5″), 123.3 (q, $^1J_{C-F}$ = 272.2 Hz, 2 × CF_3), 114.5 (s, C5), 114.2 (br s, C8a); ^{19}F NMR (376 MHz, [D$_6$]DMSO): δ ppm −61.09 (s, 2 × CF_3); NMR assignments made with the assistance of COSY, HSQC and HMBC; LCMS (m/z): [M−H, 100%]$^-$, 373.2; RT = 2.48 min; HRMS (m/z): [M+H]$^+$ calcd. for $C_{15}H_{10}O_2N_2BF_6$, 375.0734; found, 375.0734; error, −1.08 ppm.

3-((4-Fluorophenyl)amino)-1*H*-benzo[c][1,5,2]oxazaborinin-1-ol (NVR-156)

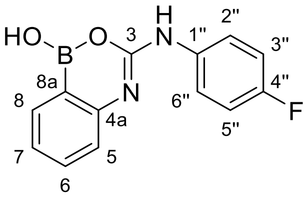

To a stirred solution of (2-aminophenyl)boronic acid (200 mg, 1.46 mmol, 1 eq) in MeCN (50 mL) was added 4-fluorophenyl isocyanate (0.200 mL, 240 mg, 1.75 mmol, 1.2 eq). The reaction mixture was stirred at room temperature for 24 h, before the resultant precipitate was filtered, washed with minimal MeCN and dried to yield **3-((4-fluorophenyl)amino)-1*H*-benzo[c][1,5,2]oxazaborinin-1-ol.** White solid; yield 78% (291 mg, 1.14 mmol); mp: 218 – 220 °C; ^{1}H NMR (400 MHz, [D$_6$]DMSO): δ ppm 10.45 (s, 1H, BOH), 9.09 (s, 1H, NH), 7.99 (d, $^3J_{H8-H7}$ = 6.8 Hz, 1H, H8), 7.47 (ddd, $^3J_{H6-H5}$ = 8.3 Hz, $^3J_{H6-H7}$ = 7.2 Hz, $^4J_{H6-H8}$ = 1.4 Hz, 1H, H6), 7.20 (d, J = 7.0 Hz, 4H, H2″+ H3″ + H5″ + H6″), 7.08 (d, $^3J_{H5-H6}$ = 8.0 Hz, 1H, H5), 7.04 (t, $^3J_{H7-H6}$ = $^3J_{H7-H8}$ = 7.4 Hz, 1H, H7); ^{13}C NMR (101 MHz, [D$_6$]DMSO): δ ppm 160.6 (d, $^1J_{C-F}$ = 241.6 Hz, C4″), 154.2 (s, C3), 145.6 (s, C4a), 135.4 (d, $^4J_{C-F}$ = 2.9 Hz, C1″), 132.7 (s, C6 + C8), 130.6 (d, $^3J_{C-F}$ = 8.8 Hz, C2″ + C6″), 120.8 (s, C7), 115.0 (d, $^2J_{C-F}$ = 22.5 Hz, C3″+ C5″), 114.3 (s, C5); ^{19}F NMR (376 MHz, [D$_6$]DMSO): δ ppm −117.05 (dt, $^3J_{F-H3''/H5''}$ = 13.3 Hz, $^4J_{F-H2''/H6''}$ = 6.8 Hz, F); NMR assignments made with the assistance of COSY, HSQC and HMBC; LCMS (m/z): [M−H, 100%]$^-$, 255.2; RT =

2.01 min; HRMS (m/z): [M+Na]$^+$ calcd. for $C_{13}H_{10}O_2N_2BFNa$, 279.0712; found, 279.0705; error, −2.36 ppm.

1-(3,5-Bis(trifluoromethyl)phenyl)-3-(2-(4,4,5,5-tetramethyl-1,3,2-dioxaborolan-2-yl)phenyl)urea (NVR-155)

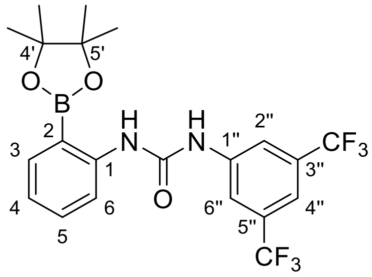

To a stirred solution of 2-(4,4,5,5-tetramethyl-1,3,2-dioxaborolan-2-yl)aniline (200 mg, 0.91 mmol, 1 eq) in MeCN (50 mL) was added 3,5-bis(trifluoromethyl)phenyl isocyanate (0.190 mL, 280 mg, 1.10 mmol, 1.2 eq). The reaction mixture was stirred for 24 h, before the resultant precipitate was filtered, washed with minimal MeCN and dried to yield **1-(3,5-bis(trifluoromethyl)phenyl)-3-(2-(4,4,5,5-tetramethyl-1,3,2-dioxaborolan-2-yl)phenyl)urea.** White solid; yield 79% (342 mg, 0.721 mmol); mp: 217 – 218 °C; ^{1}H NMR (400 MHz, [D$_6$]DMSO): δ ppm 9.96 (s, 1H, NH), 9.25 (s, 1H, NH″), 8.15 (s, 2H, H2″+ H6″), 7.71 (s, 1H, H4″), 7.51 (d, $^3J_{H3\text{-}H4}$ = 7.0 Hz, 1H, H3), 7.32 - 7.43 (m, 2H, H5 + H6), 7.06 (td, $^3J_{H4\text{-}H3}$ = $^3J_{H4\text{-}H5}$ = 7.1 Hz, $^4J_{H4\text{-}H6}$ = 1.4 Hz, 1H, H4), 1.23 (s, 12H, 4 × Me); ^{13}C NMR (101 MHz, [D$_6$]DMSO): δ ppm 153.6 (s, CO), 141.7 (s, C1), 141.2 (s, C1″), 134.2 (s, C3), 130.7 (q, $^2J_{C\text{-}F}$ = 32.0 Hz, C3″ + C5″), 130.3 (s, C5), 122.9 (s, C4), 119.2 (s, C6), 119.0 (br q, $^3J_{C\text{-}F}$ = 3.0 Hz, C2″+ C6″), 123.3 (q, $^1J_{C\text{-}F}$ = 272.9 Hz, 2 × CF_3), 115.1 (br s, C4″), 82.4 (s, C4′ + C5′), 25.0 (s, 4C, 4 × Me); ^{19}F NMR (376 MHz, [D$_6$]DMSO): δ ppm −61.65 (s, 2 × CF_3); NMR assignments made with the assistance of COSY, HSQC and HMBC; LCMS (m/z): [M−H, 100%]$^-$, 373.2; RT = 2.46 min; HRMS (m/z): [M−H]$^-$ calcd. for $C_{21}H_{20}O_3N_2BF_6$, 473.1477; found 473.1468; error, –1.83 ppm.

1-(4-Fluorophenyl)-3-(2-(4,4,5,5-tetramethyl-1,3,2-dioxaborolan-2-yl)phenyl)urea (NVR-157)

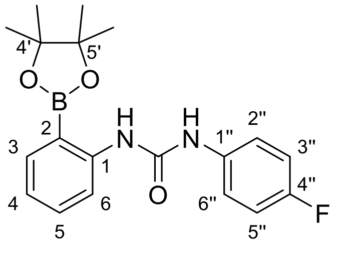

4-Fluorophenyl isocyanate (0.125 mL, 151 mg, 1.10 mmol, 1.2 eq) was added to a stirred solution of 2-(4,4,5,5-tetramethyl-1,3,2-dioxaborolan-2-yl)aniline (200 mg, 0.91 mmol, 1 eq) in MeCN (50 mL). The reaction mixture was stirred for 24 h, before the resultant precipitate was filtered, washed with minimal MeCN and dried to yield **1-(4-fluorophenyl)-3-(2-(4,4,5,5-tetramethyl-1,3,2-dioxaborolan-2-yl)phenyl)urea.** White solid; yield 77% (250 mg, 0.702 mmol); mp: 221 – 222 °C; ^{1}H NMR (400 MHz, [D$_6$]DMSO): δ ppm 9.36 (br s, 1H, NH″), 9.35 (br s, 1H, NH), 7.37 - 7.50 (m, 3H, H6 + H2″ + H6″), 7.24 - 7.30 (m, 1H, H3), 7.21 - 7.24 (m, 1H, H5), 7.18 (br t, $^{3}J_{H3''/H5''-H2''/H6''}$ = 8.9 Hz, 2H, H3″+ H5″), 7.00 (td, $^{3}J_{H4-H3}$ = $^{3}J_{H4-H5}$ = 7.1 Hz, $^{4}J_{H4-H6}$ = 1.4 Hz, 1H, H4), 1.19 (s, 12H, 4 × Me); ^{13}C NMR (101 MHz, [D$_6$]DMSO): δ ppm 158.4 (d, $^{1}J_{C-F}$ = 239.6 Hz, C4″), 154.2 (s, C7″), 141.2 (s, C1), 134.2 (d, $^{4}J_{C-F}$ = 2.0 Hz, C1″), 133.4 (s, C6), 129.1 (s, C3), 122.6 (s, C4), 122.5 (br d, $^{3}J_{C-F}$ = 3.9 Hz, C2″ + C6″), 117.1 (s, C5), 115.4 (d, $^{2}J_{C-F}$ = 22.5 Hz, C3″+ C5″), 81.2 (s, C4′ + C5′), 25.4 (s, 4C, 4 × Me); ^{19}F NMR (376 MHz, [D$_6$]DMSO): δ ppm −119.43 (br s, F); NMR assignments made with the assistance of COSY, HSQC and HMBC; LCMS (m/z): [M−H, 100%]$^{-}$, 255.2; RT = 2.01 min; HRMS (m/z): [M+Na]$^{+}$ calcd. for $C_{19}H_{22}O_3N_2BFNa$, 379.1600; found, 379.1591; error, −2.30 ppm.

References

1 Jean-Claude, B., Antony, W. & Andrew, L. *Jean-Claude Bradley Open Melting Point Dataset*. (2014).

2 Hinsberger, S. *et al.* Discovery of Novel Bacterial RNA Polymerase Inhibitors: Pharmacophore-Based Virtual Screening and Hit Optimization. *J. Med. Chem.* **56**, 8332-8338, doi:10.1021/jm400485e (2013).

3 Köhler, S. C., Vahdati, S., Scholz, M. S. & Wiese, M. Structure activity relationships, multidrug resistance reversal and selectivity of heteroarylphenyl ABCG2 inhibitors. *Eur. J. Med. Chem.* **146**, 483-500, doi:https://doi.org/10.1016/j.ejmech.2018.01.012 (2018).

4 Dahl, B. & Christophersen, P. Subsituted phenyl derivatives, their preparation and use. WO 00/24707 (2000).

5 Christophersen, P. & Dahl, B. Use of malaria parasite anion channel blockers for treating malaria. WO 02/39887 (2002).

6 Dahl, B. & Christophersen, P. Subsituted phenyl derivatives, their preparation and use. US 2002/0037905 (2002).

7 Busschaert, N. *et al.* Squaramides as Potent Transmembrane Anion Transporters. *Angew Chem Int Edit* **51**, 4426-4430, doi:10.1002/anie.201200729 (2012).

8 Zeng, L., Fu, H., Qiao, R., Jiang, Y. & Zhao, Y. Efficient Copper-Catalyzed Synthesis of N-Alkylanthranilic Acids via an ortho-Substituent Effect of the Carboxyl Group of 2-Halobenzoic Acids at Room Temperature. *Adv. Synth. Catal.* **351**, 1671-1676, doi:10.1002/adsc.200900065 (2009).

9 Valgeirsson, J. *et al.* 2-Arylureidobenzoic Acids: Selective Noncompetitive Antagonists for the Homomeric Kainate Receptor Subtype GluR5. *J. Med. Chem.* **46**, 5834-5843, doi:10.1021/jm030428j (2003).

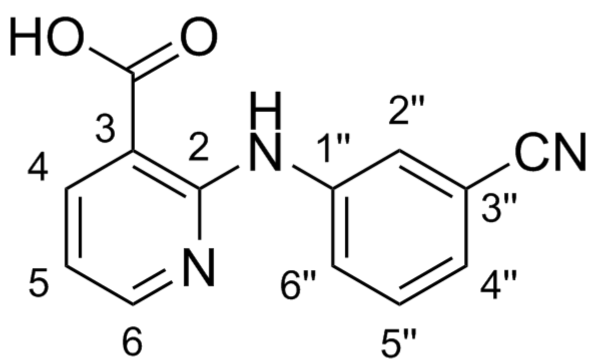

Shift (ppm)	H	m	J (Hz)	Assign
13.73	1	br s	-	COOH
10.61	1	s	-	NH
8.45	1	br d	4.4	H6
8.38	1	s	-	H2''
8.28	1	br d	7.8	H6''
7.88	1	br d	8.2	H4
7.49	1	t	7.9	H5''
7.42	1	br d	7.6	H4''
6.95	1	dd	7.2, 5.2	H5

Acquisition Time (sec)	3.2768
Date	09 Mar 2018 12:29:13
Date Stamp	09 Mar 2018 12:29:13
Frequency (MHz)	500.1900
Nucleus	1H
Number of Transients	16
Solvent	DMSO-d6
Temperature (degree C)	25.001

^{1}H NMR (500 MHz, *DMSO-d*$_6$) δ ppm 13.73 (br s, 1 H), 10.61 (s, 1 H), 8.45 (br d, *J*=4.4 Hz, 1 H), 8.38 (s, 1 H), 8.28 (br d, *J*=7.8 Hz, 1 H), 7.88 (br d, *J*=8.2 Hz, 1 H), 7.49 (t, *J*=7.9 Hz, 1 H), 7.42 (br d, *J*=7.6 Hz, 1 H), 6.95 (dd, *J*=7.2, 5.2 Hz, 1 H)

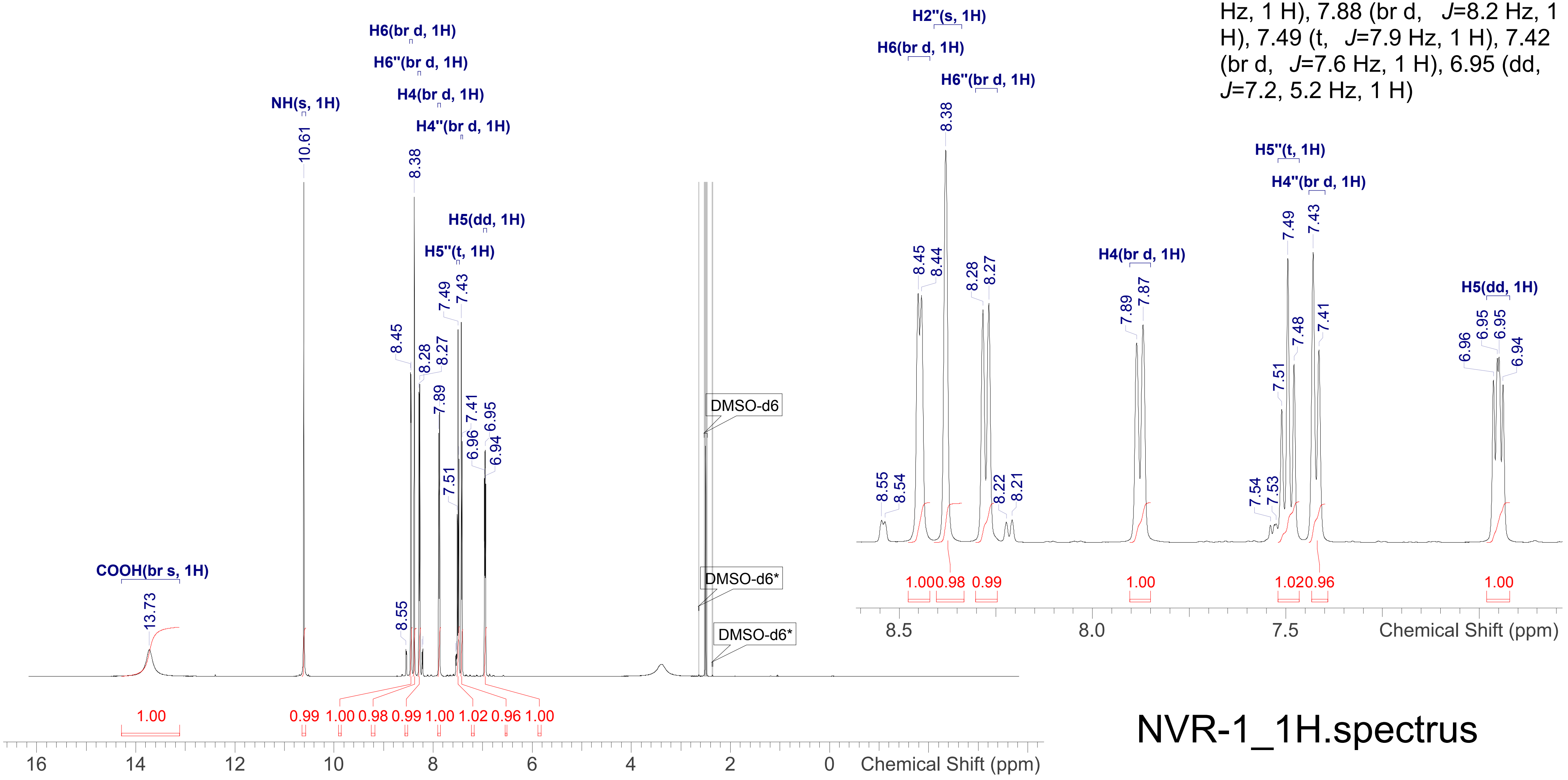

NVR-1_1H.spectrus

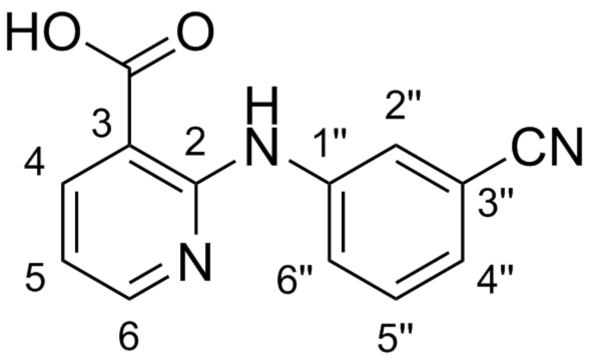

Shift (ppm)	C	m	Assign
168.9	1	s	COOH
155.0	1	s	2
152.5	1	s	6
140.7	1	s	4
140.6	1	s	1''
130.0	1	s	5''
125.4	1	s	4''
124.4	1	s	6''
122.2	1	s	2''
119.0	1	s	CN
115.0	1	s	5
111.5	1	s	3''
108.4	1	s	3

Acquisition Time (sec)	2.0447
Date	09/03/2018 12:33:00
Date Stamp	09/03/2018 12:33:00
Frequency (MHz)	125.7870
Nucleus	13C
Number of Transients	64
Solvent	DMSO-d6

^{13}C NMR (126 MHz, *DMSO-d_6*) δ ppm 168.9 (s, 1 C), 155.0 (s, 1 C), 152.5 (s, 1 C), 140.7 (s, 1 C), 140.6 (s, 1 C), 130.0 (s, 1 C), 125.4 (s, 1 C), 124.4 (s, 1 C), 122.2 (s, 1 C), 119.0 (s, 1 C), 115.0 (s, 1 C), 111.5 (s, 1 C), 108.4 (s, 1 C)

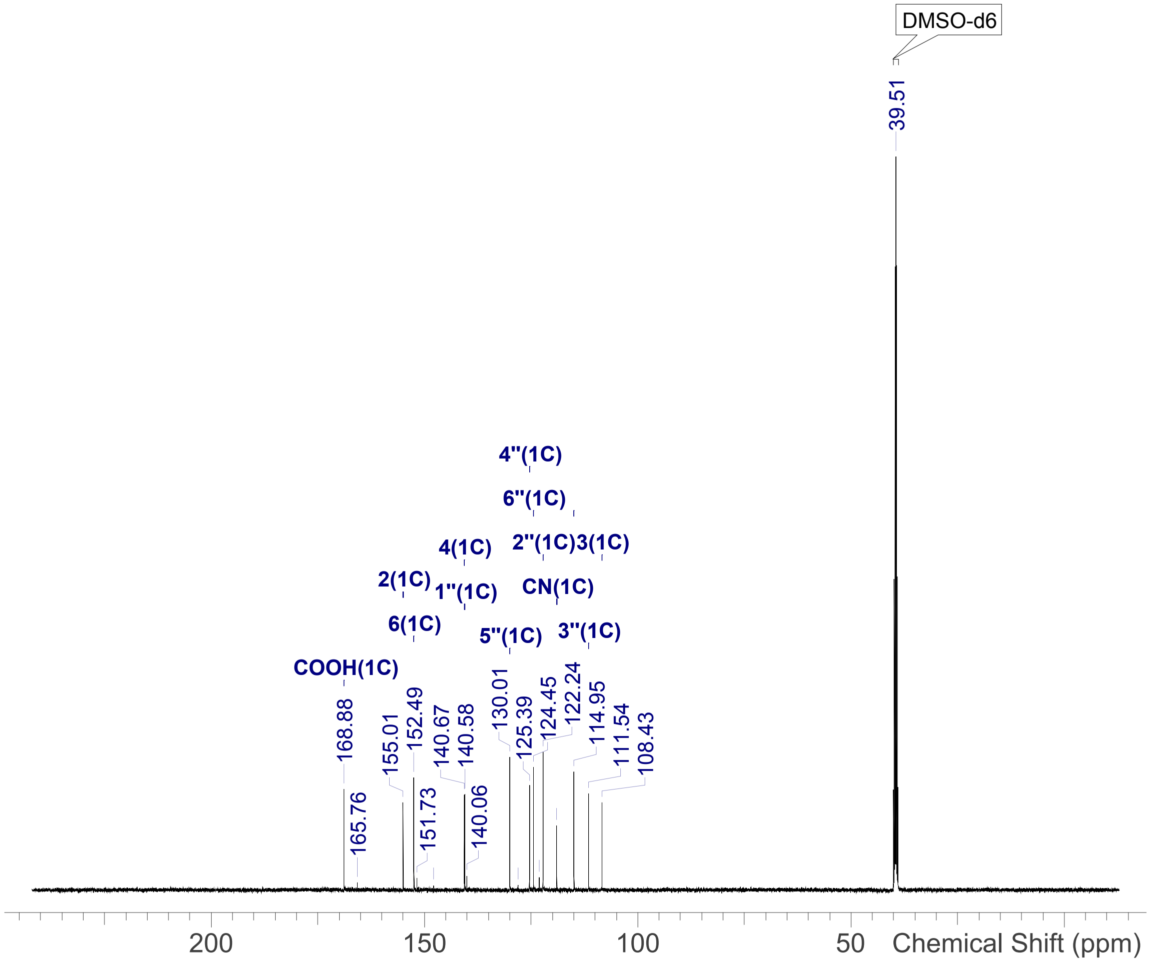

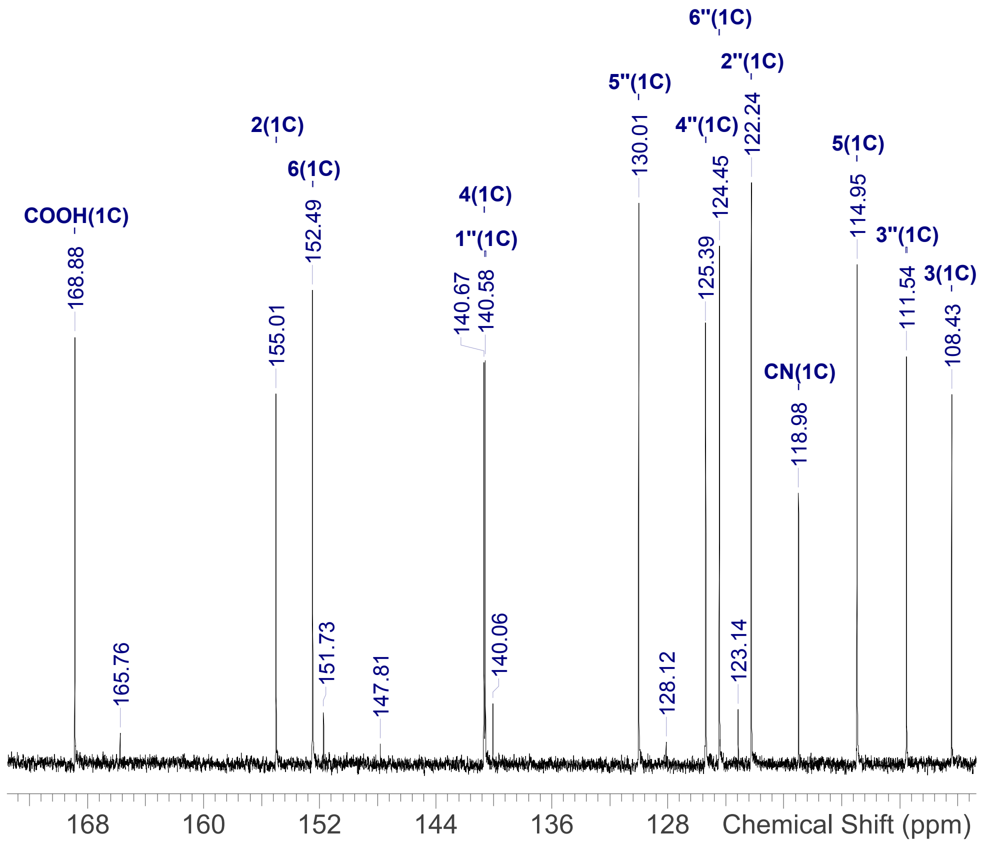

NVR-1_13C.spectrus

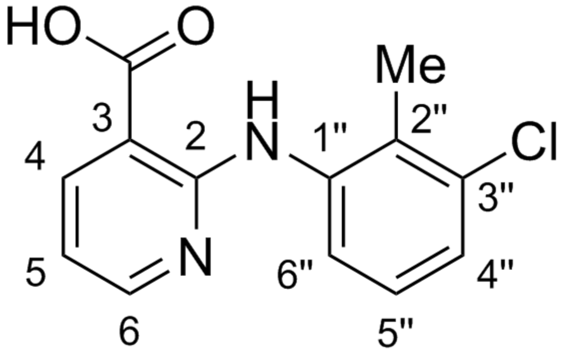

Shift (ppm)	H	m	J (Hz)	Assign
13.63	1	br s	-	COOH
10.30	1	s	-	NH
8.34	1	dd	4.7, 2.0	H6
8.26	1	dd	7.7, 1.9	H4
8.11	1	dd	7.9, 0.8	H6''
7.21	1	t	7.9	H5''
7.16	1	dd	7.9, 1.2	H4''
6.87	1	dd	7.8, 4.7	H5
2.32	3	s	-	Me

Acquisition Time (sec)	6.5536
Date	10/04/2018 11:15:00
Date Stamp	10/04/2018 11:15:00
Frequency (MHz)	500.1930
Nucleus	1H
Number of Transients	16
Solvent	DMSO-d6

^{1}H NMR (500 MHz, *DMSO-d*$_6$) δ ppm 13.63 (br s, 1 H), 10.30 (s, 1 H), 8.34 (dd, *J*=4.7, 2.0 Hz, 1 H), 8.26 (dd, *J*=7.7, 1.9 Hz, 1 H), 8.11 (dd, *J*=7.9, 0.8 Hz, 1 H), 7.21 (t, *J*=7.9 Hz, 1 H), 7.16 (dd, *J*=7.9, 1.2 Hz, 1 H), 6.87 (dd, *J*=7.8, 4.7 Hz, 1 H), 2.32 (s, 3 H)

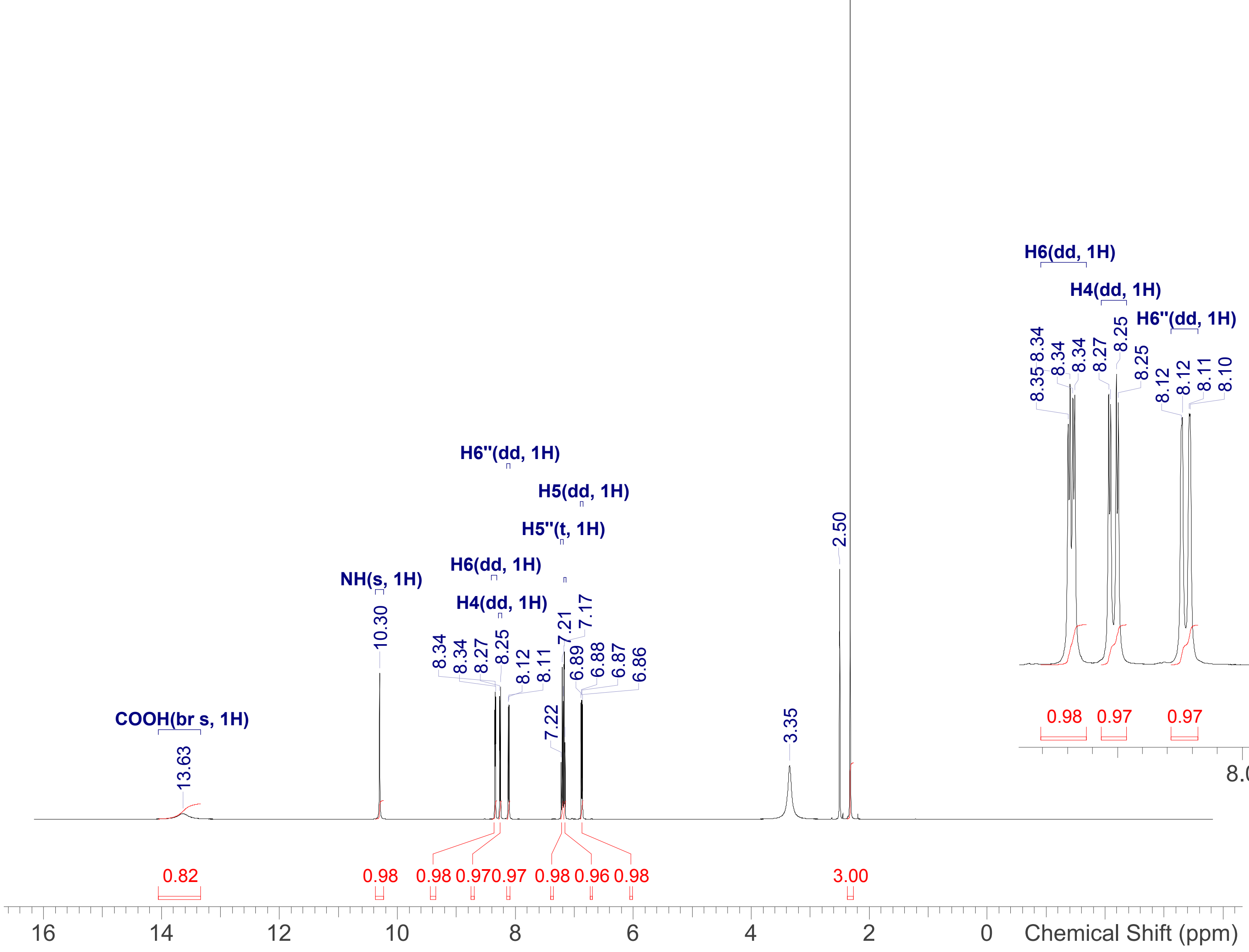

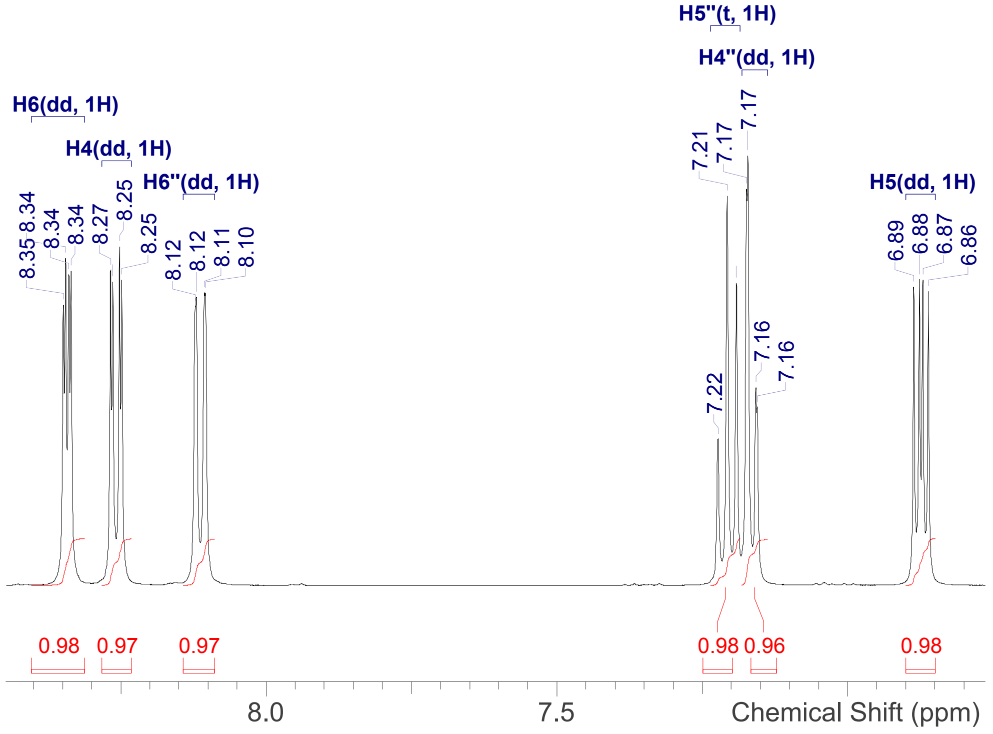

Clonixin_1H.spectrus

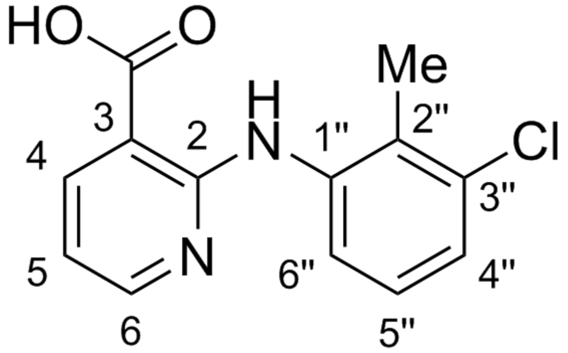

Shift (ppm)	C	m	Assign
169.2	1	s	COOH
155.8	1	s	2
152.7	1	s	6
140.6	1	s	4
139.8	1	s	1''
133.5	1	s	3''
126.9	1	s	5''
123.7	1	s	6''
121.1	1	s	4''
114.1	1	s	5
107.8	1	s	3
14.7	1	s	Me

Acquisition Time (sec)	2.0447
Date	10/04/2018 11:20:00
Date Stamp	10/04/2018 11:20:00
Frequency (MHz)	125.7870
Nucleus	13C
Number of Transients	64
Solvent	DMSO-d6

^{13}C NMR (126 MHz, *DMSO-d_6*) δ ppm 169.2 (s, 1 C), 155.8 (s, 1 C), 152.7 (s, 1 C), 140.6 (s, 1 C), 139.8 (s, 1 C), 133.5 (s, 1 C), 126.9 (s, 1 C), 123.7 (s, 1 C), 121.1 (s, 1 C), 114.1 (s, 1 C), 107.8 (s, 1 C), 14.7 (s, 1 C)

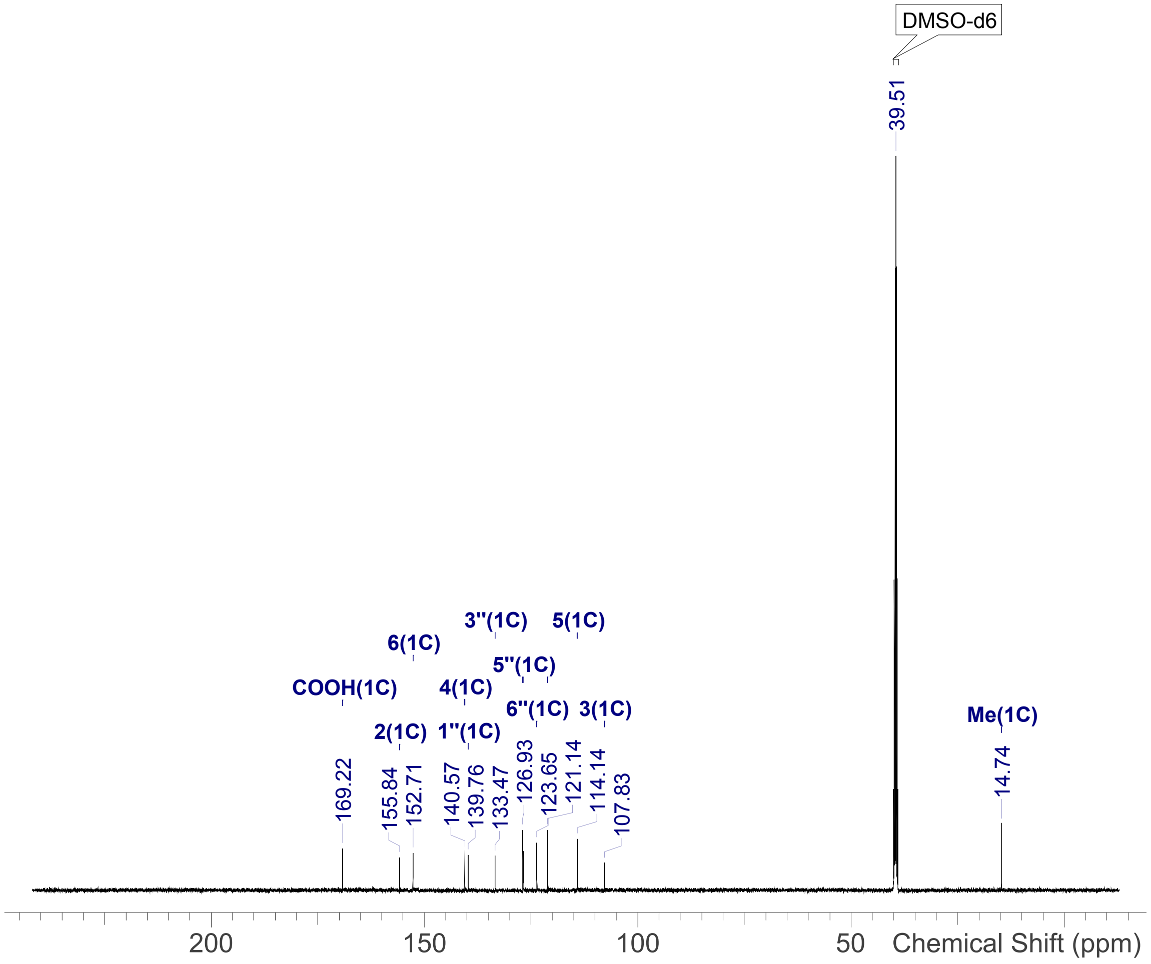

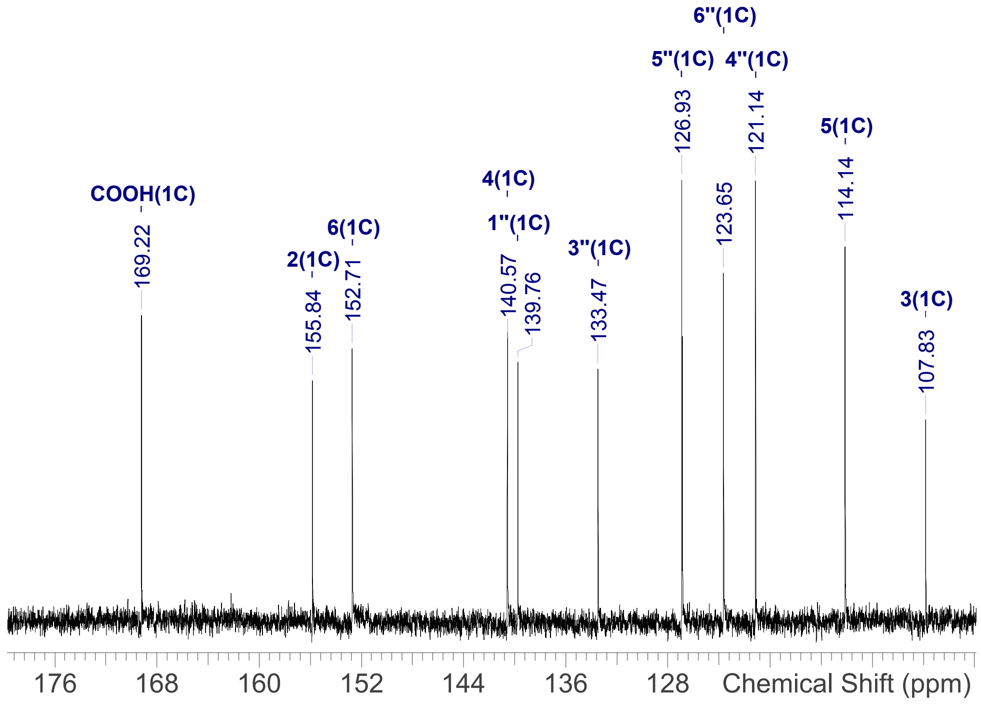

Clonixin_13C.spectrus

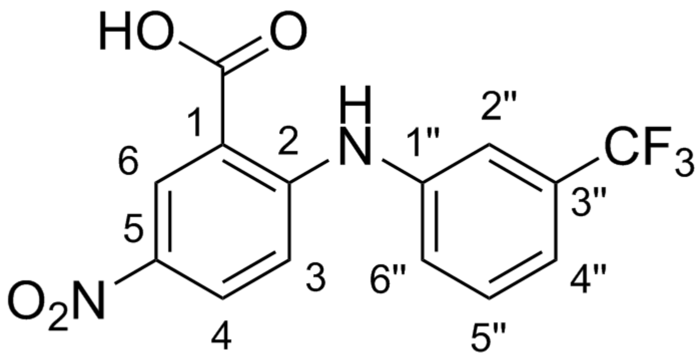

Shift (ppm)	H	m	J (Hz)	Assign
9.72	1	s	-	NH
8.35	1	s	-	H6
7.53	5	m	-	H4, H2", H4", H5", H6"
7.23	1	d	8.9	H3

Acquisition Time (sec)	10.6168
Date	10/01/2017 14:41:00
Date Stamp	10/01/2017 14:41:00
Frequency (MHz)	300.1310
Nucleus	1H
Number of Transients	16
Solvent	CHLOROFORM-d

^{1}H NMR (300 MHz, *CHLOROFORM-d*) δ ppm 9.72 (s, 1 H), 8.35 (s, 1 H), 7.41 - 7.64 (m, 5 H), 7.23 (d, *J*=8.9 Hz, 1 H)

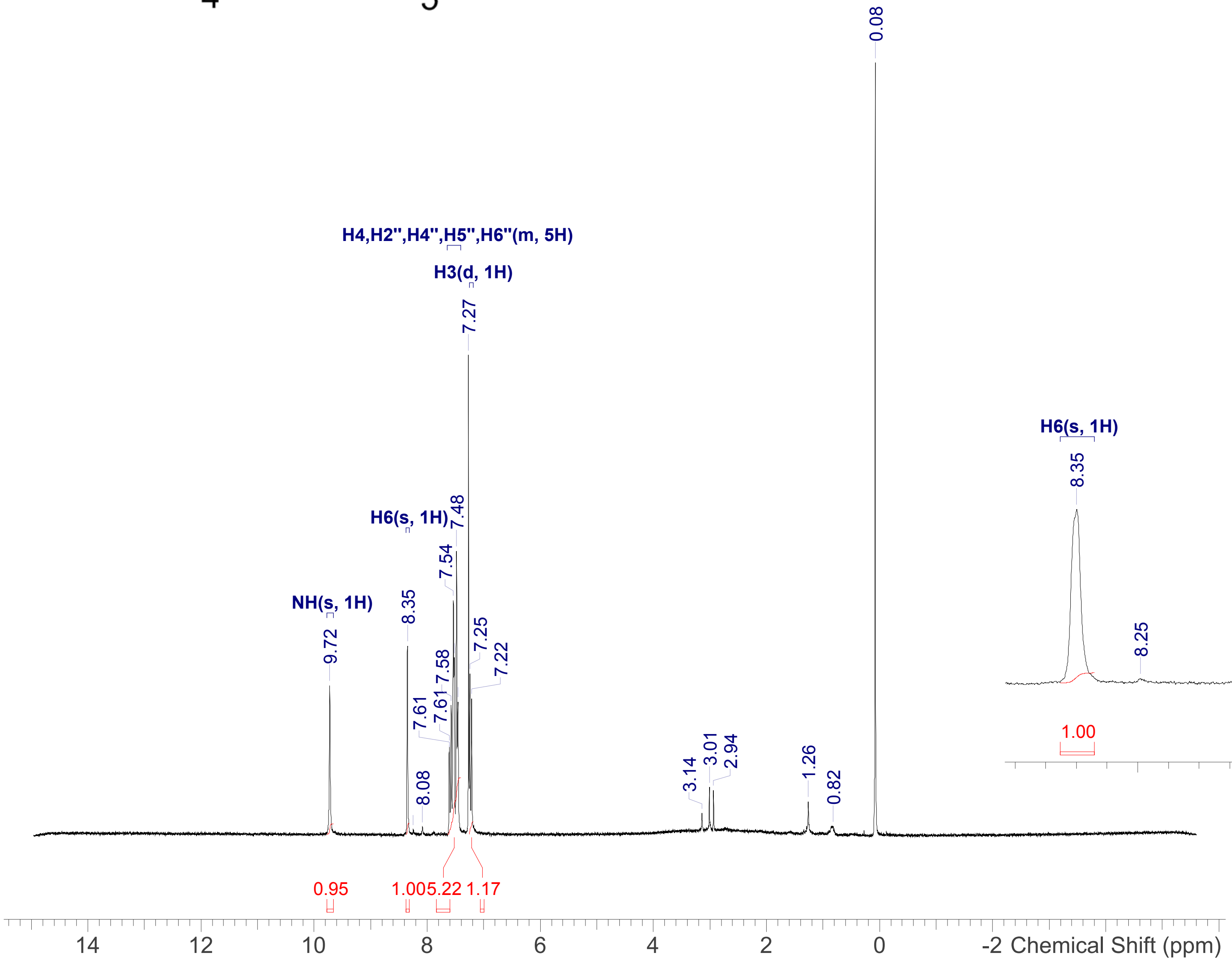

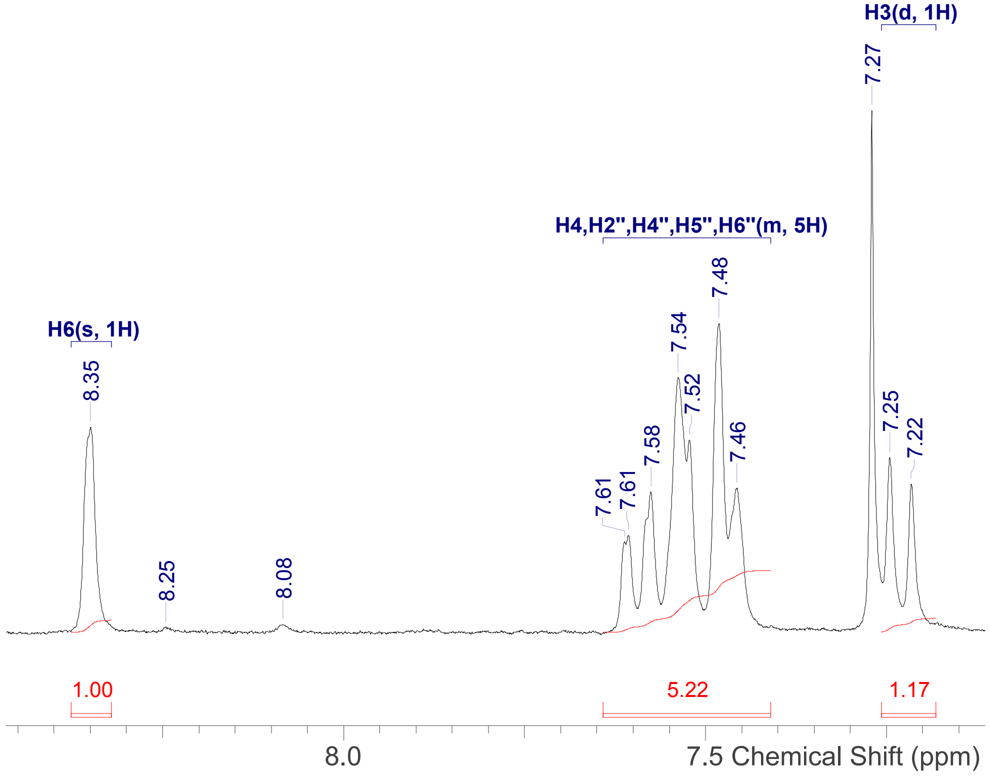

NVR-3_1H.spectrus

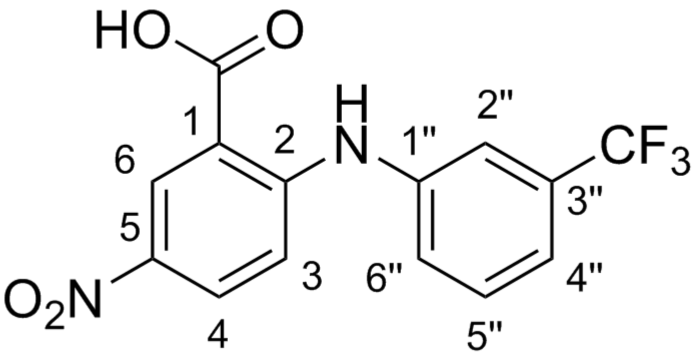

Shift (ppm)	C	m	J (Hz)	Assign
168.3	1	s	-	COOH
151.6	1	s	-	2
139.5	1	s	-	1''
137.4	1	s	-	5
130.9	1	s	-	4
129.4	1	s	-	6
128.3	1	s	-	5''
127.6	1	s	-	6''
121.9	1	q	4.1	2''
120.4	1	q	4.4	4''
113.7	1	s	-	1
112.1	1	s	-	3

Acquisition Time (sec)	3.6438
Date	10/08/2016 08:00:00
Date Stamp	10/08/2016 08:00:00
Frequency (MHz)	75.4753
Nucleus	13C
Number of Transients	12000
Solvent	DMSO-d6

^{13}C NMR (75 MHz, *DMSO-d$_6$*) δ ppm 168.3 (s, 1 C), 151.6 (s, 1 C), 139.5 (s, 1 C), 137.4 (s, 1 C), 130.9 (s, 1 C), 129.4 (s, 1 C), 128.3 (s, 1 C), 127.6 (s, 1 C), 121.9 (q, *J*=4.1 Hz, 1 C), 120.4 (q, *J*=4.4 Hz, 1 C), 113.7 (s, 1 C), 112.1 (s, 1 C)

NVR-3_13C

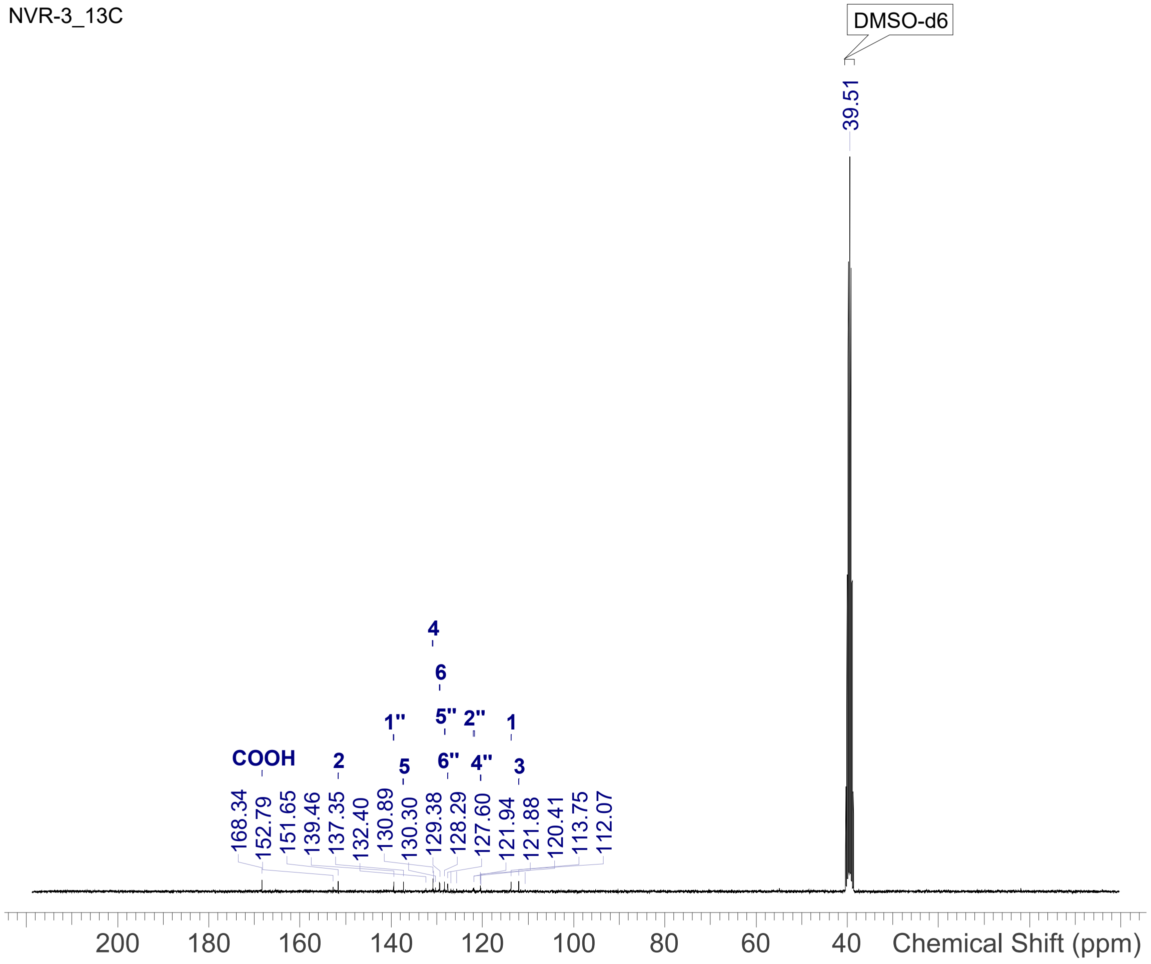

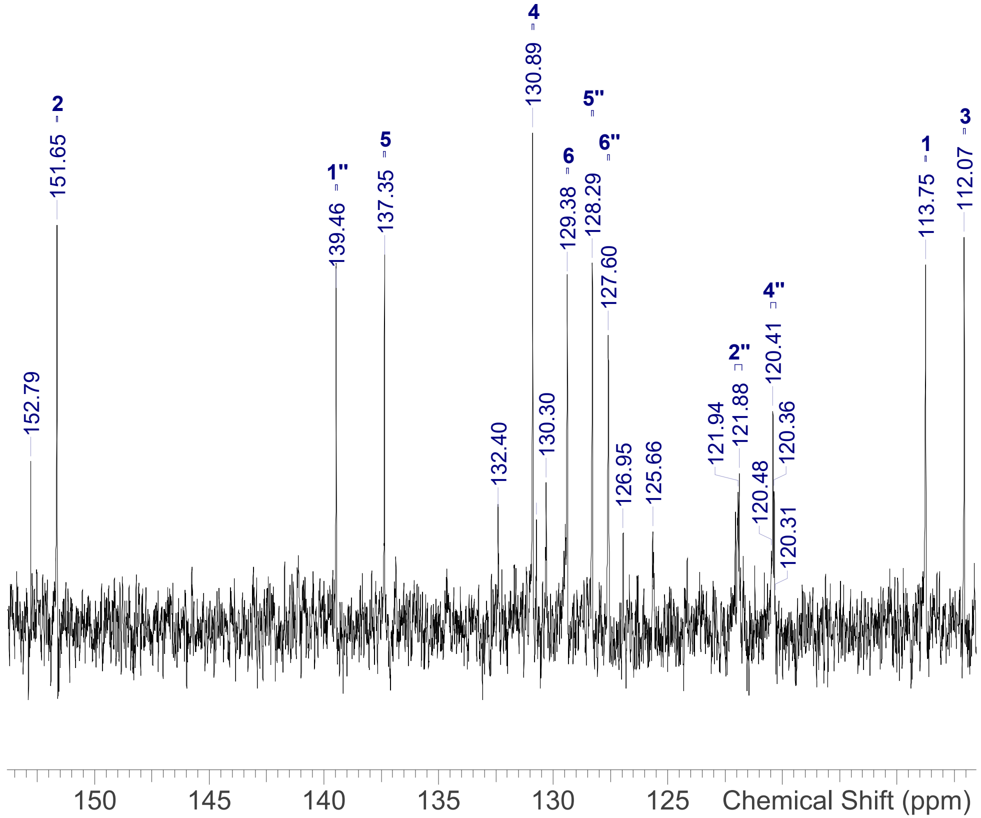

NVR-3_13C.spectrus

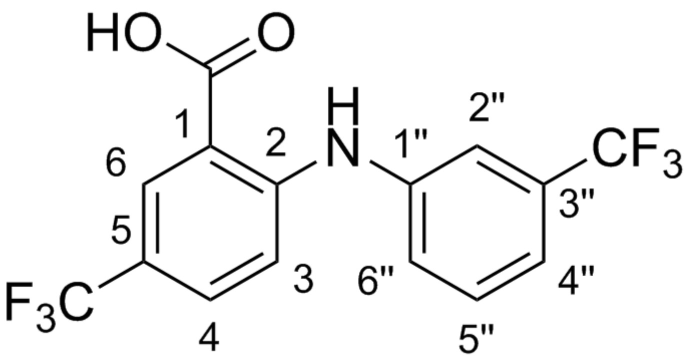

Shift (ppm)	H	m	J (Hz)	Assign
13.67	1	br s	-	COOH
10.03	1	s	-	NH
8.16	1	d	2.0	H6
7.71	1	dd	9.0, 2.3	H4
7.64	3	m	-	H2", H5", H6"
7.50	1	d	6.9	H4"
7.31	1	d	8.9	H3

Acquisition Time (sec)	3.2768
Date	28 Aug 2017 17:31:44
Date Stamp	28 Aug 2017 17:31:44
Frequency (MHz)	500.1900
Nucleus	1H
Number of Transients	16
Solvent	DMSO-d6
Temperature (degree C)	24.999

^{1}H NMR (500 MHz, *DMSO-d_6*) δ ppm 13.67 (br s, 1 H), 10.03 (s, 1 H), 8.16 (d, *J*=2.0 Hz, 1 H), 7.71 (dd, *J*=9.0, 2.3 Hz, 1 H), 7.59 - 7.67 (m, 3 H), 7.50 (d, *J*=6.9 Hz, 1 H), 7.31 (d, *J*=8.9 Hz, 1 H)

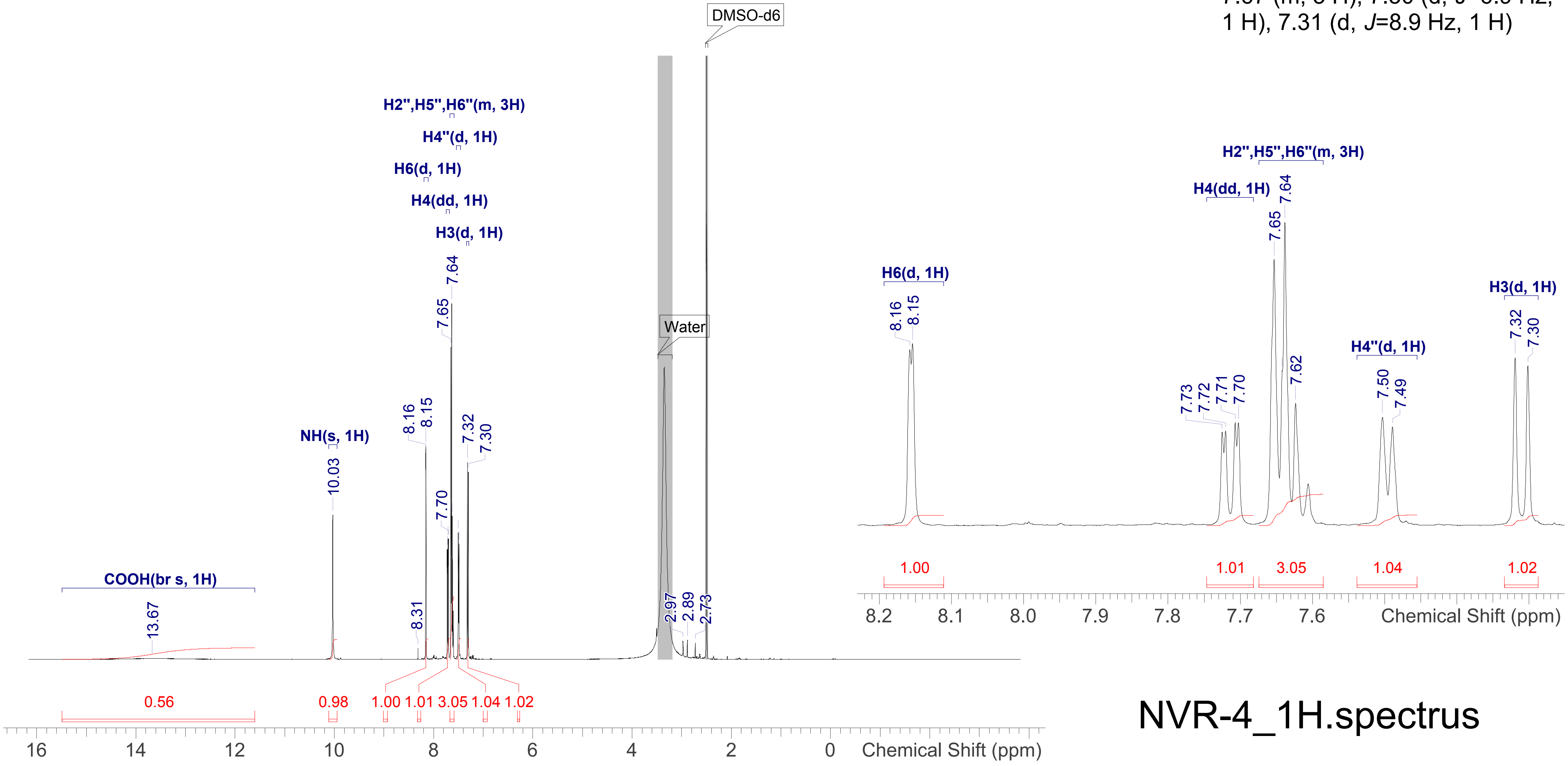

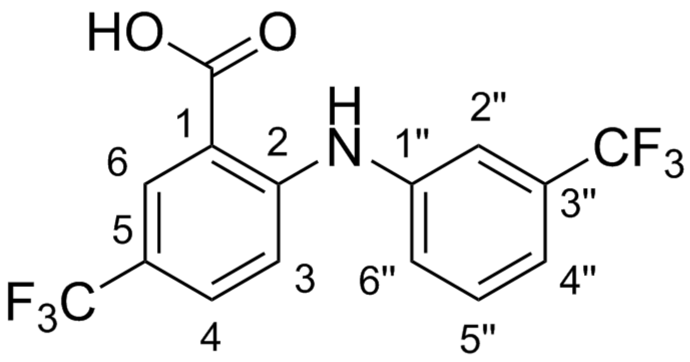

Shift (ppm)	C	m	J (Hz)	Assign
168.7	1	s	-	COOH
149.2	1	s	-	2
140.4	1	s	-	1''
130.7	1	s	-	5''
130.7	1	q	4.7	4
130.4	1	q	31.8	3''
128.9	1	q	4.2	6
126.2	1	s	-	6''
124.3	1	q	271.0	CF3
123.9	1	q	272.4	CF3
120.6	1	q	3.9	4''
119.0	1	q	4.4	2''
117.9	1	q	31.6	5
114.4	1	s	-	3
112.9	1	s	-	1

**Acquisition Time (sec)**	2.0447
**Date**	28/08/2017 17:36:00
**Date Stamp**	28/08/2017 17:36:00
**Frequency (MHz)**	125.7870
**Nucleus**	13C
**Number of Transients**	64
**Solvent**	DMSO-d6

^{13}C NMR (126 MHz, *DMSO-d$_6$*) δ ppm 168.7 (s, 1 C), 149.2 (s, 1 C), 140.4 (s, 1 C), 130.7 (s, 1 C), 130.7 (q, *J*=4.7 Hz, 1 C), 130.4 (q, *J*=31.8 Hz, 1 C), 128.9 (q, *J*=4.2 Hz, 1 C), 126.2 (s, 1 C), 124.3 (q, *J*=271.0 Hz, 1 C), 123.9 (q, *J*=272.4 Hz, 1 C), 120.6 (q, *J*=3.9 Hz, 1 C), 119.0 (q, *J*=4.4 Hz, 1 C), 117.9 (q, *J*=31.6 Hz, 1 C), 114.4 (s, 1 C), 112.9 (s, 1 C)

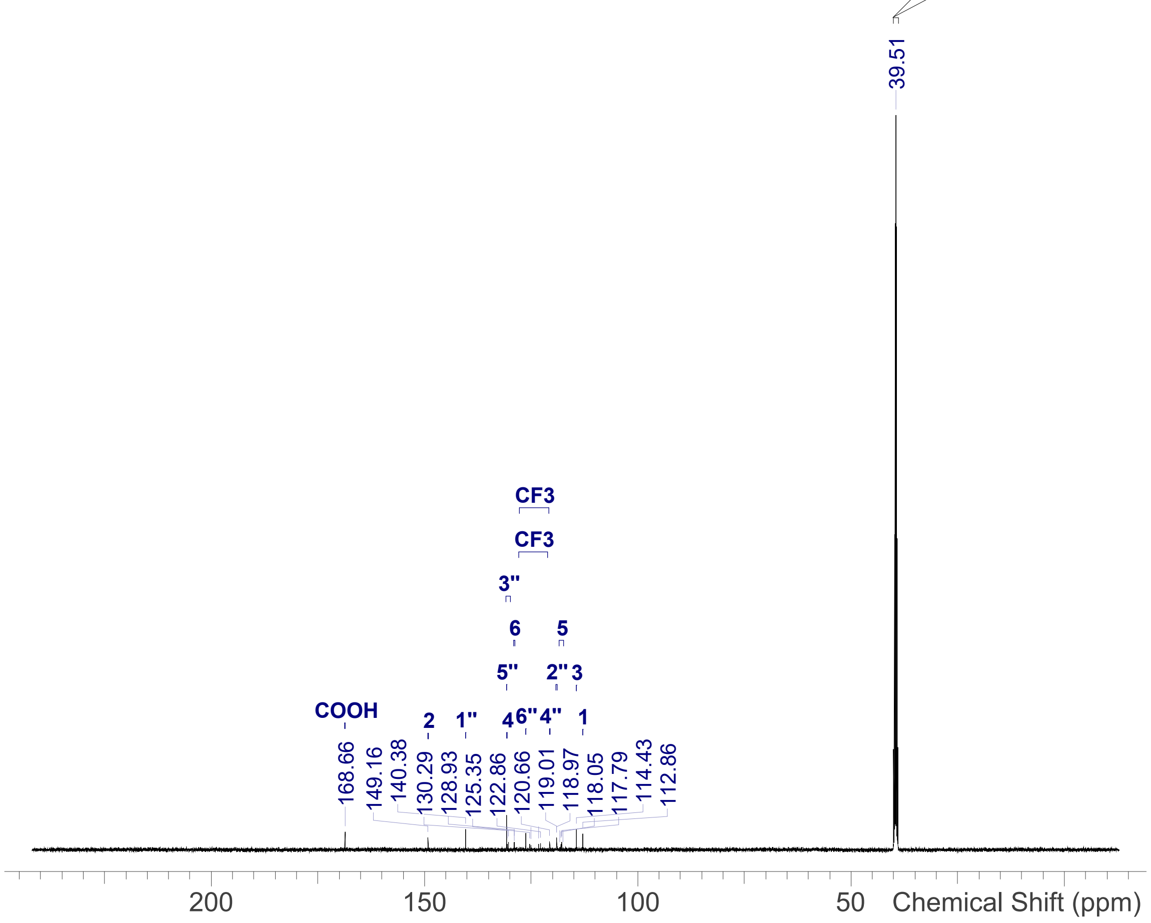

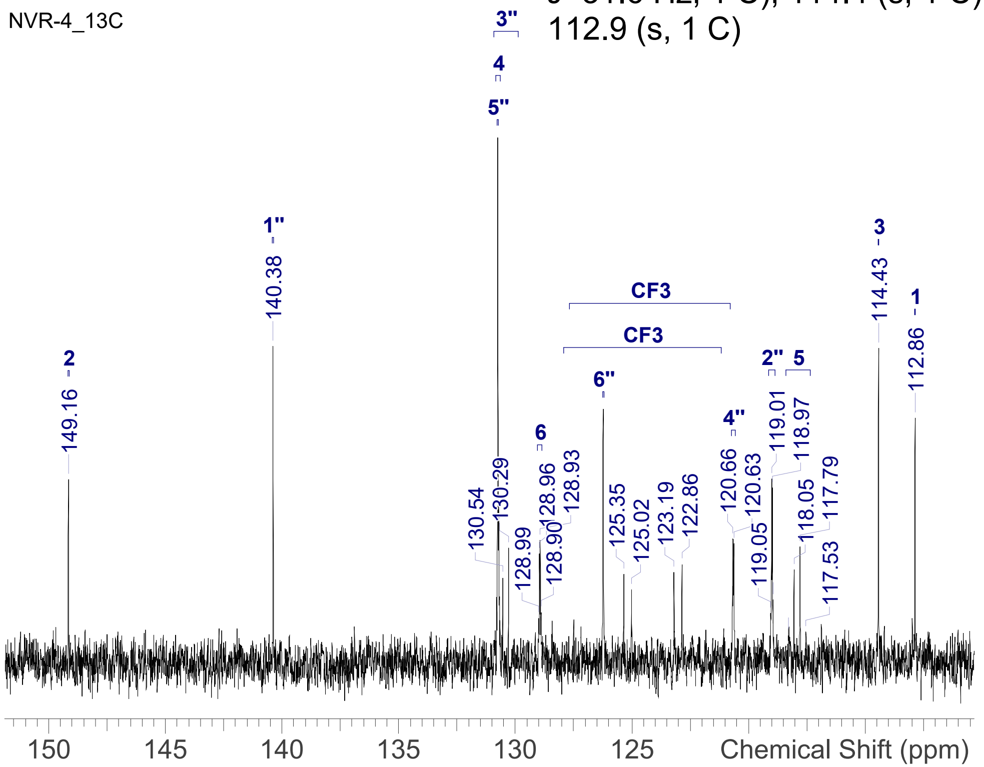

NVR-4_13C.spectrus

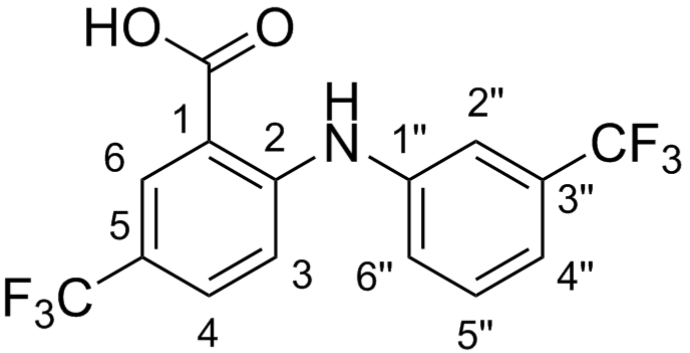

Shift (ppm)	F	m
-60.24	3	s
-61.23	3	s

Acquisition Time (sec)	2.9360
Date	28/08/2017 17:43:00
Date Stamp	28/08/2017 17:43:00
Frequency (MHz)	470.6020
Nucleus	19F
Number of Transients	16
Solvent	DMSO-d6

^{19}F NMR (471 MHz, *DMSO-d_6*) δ ppm -60.24 (s, 3 F), -61.23 (s, 3 F)

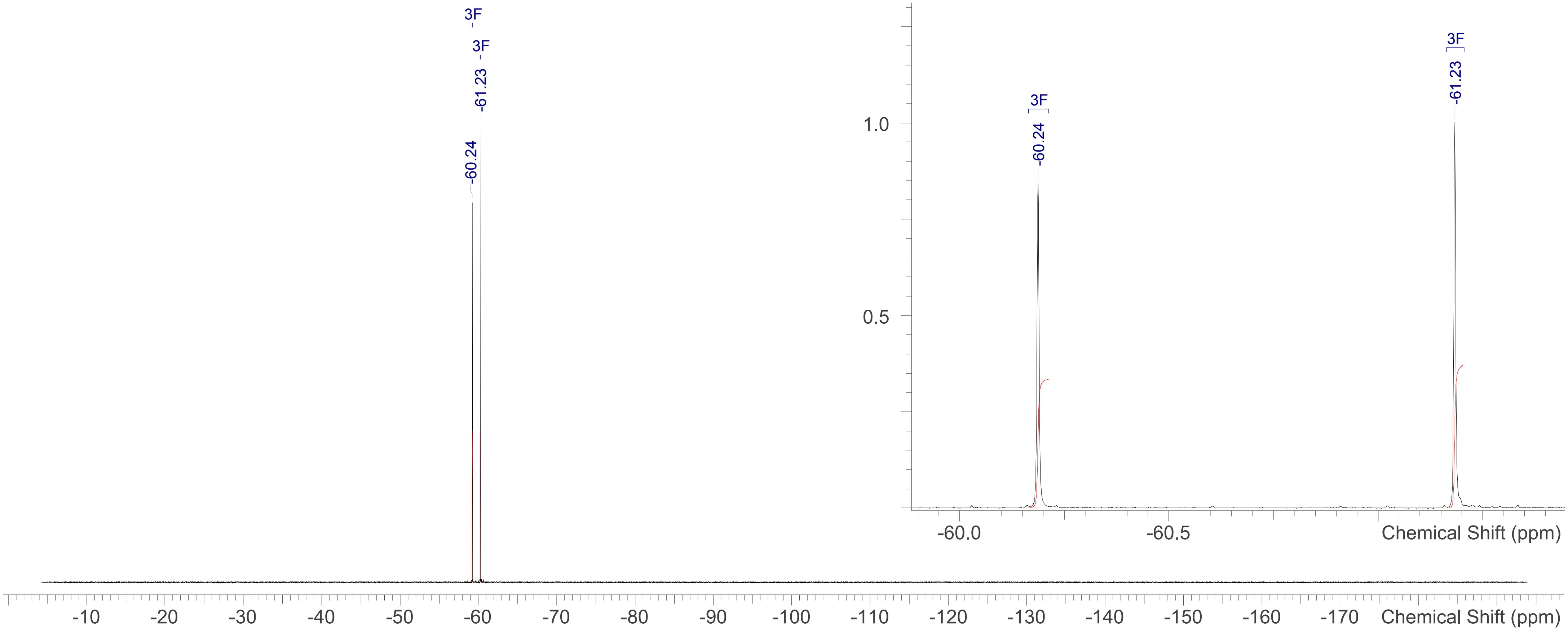

NVR-4_19F.spc

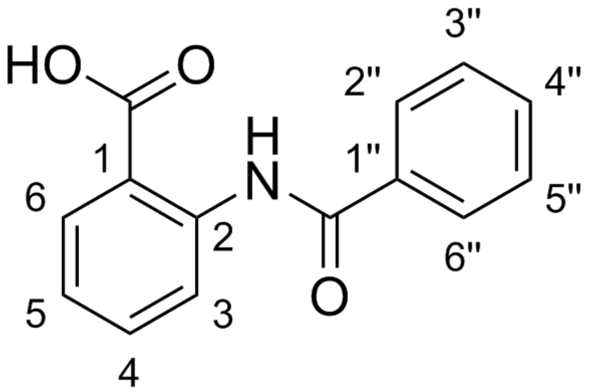

Shift (ppm)	H	m	J (Hz)	Assign
13.80	1	br s	-	COOH
12.18	1	s	-	CONH
8.72	1	d	8.0	3
8.06	1	dd	8.0, 1.5	6
7.96	2	d	7.0	2", 6"
7.63	4	m	-	4", 3", 5", 4
7.22	1	ddd	8.2, 6.9, 1.0	5

Acquisition Time (sec)	3.9846
Date	30 Apr 2018 12:35:27
Date Stamp	30 Apr 2018 12:35:27
Frequency (MHz)	400.0700
Nucleus	1H
Number of Transients	16
Solvent	DMSO-d6
Temperature (degree C)	25.007

^{1}H NMR (400 MHz, *DMSO-d_6*) δ ppm 13.80 (br s, 1 H), 12.18 (s, 1 H), 8.72 (d, *J*=8.0 Hz, 1 H), 8.06 (dd, *J*=8.0, 1.5 Hz, 1 H), 7.96 (d, *J*=7.0 Hz, 2 H), 7.54 - 7.73 (m, 4 H), 7.22 (ddd, *J*=8.2, 6.9, 1.0 Hz, 1 H)

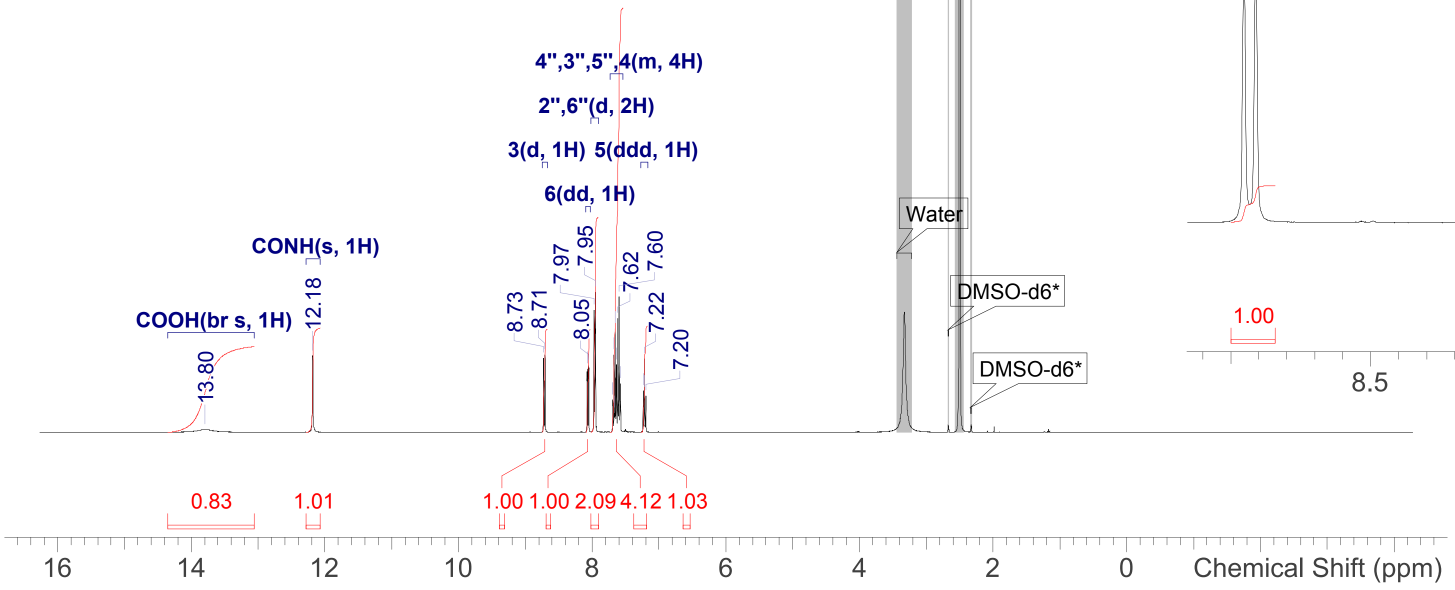

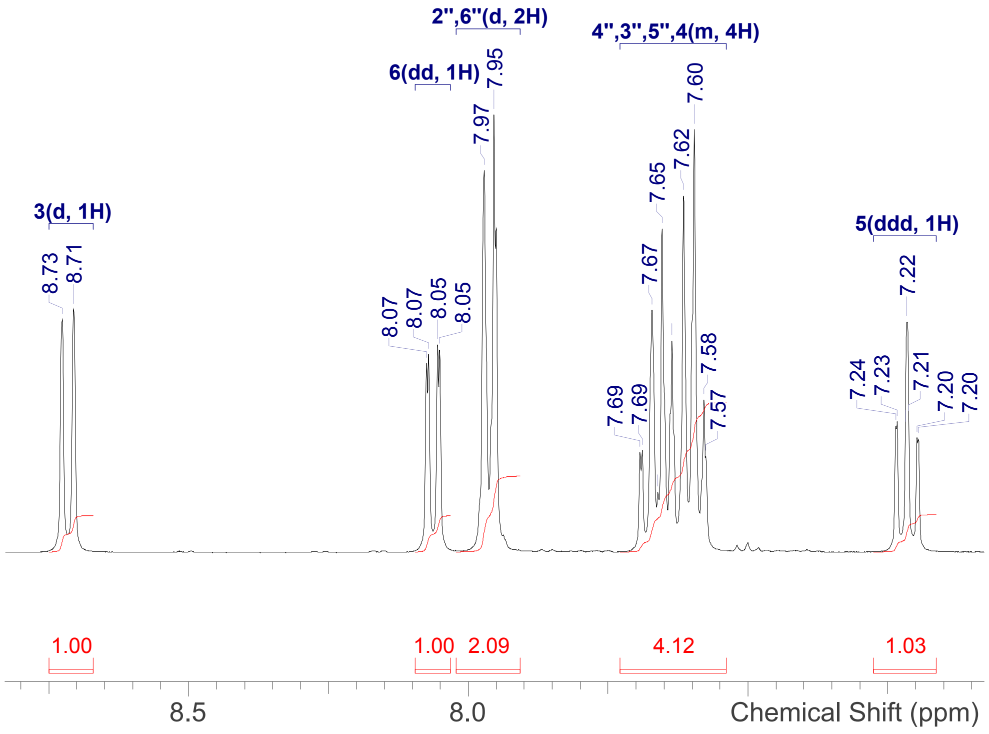

NVR-17_1H.spectrus

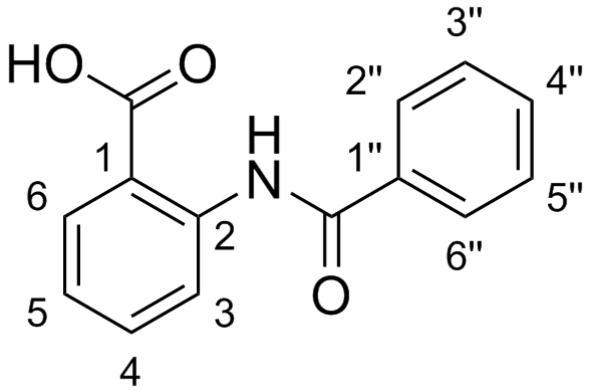

Shift (ppm)	C	m	Assign
170.0	1	s	COOH
164.7	1	s	CONH
141.1	1	s	1''
134.5	1	s	2
134.3	1	s	6
132.2	1	s	5
131.3	1	s	4
129.0	2	s	3'', 5''
127.0	2	s	2'', 6''
123.0	1	s	4''
119.9	1	s	3
116.6	1	s	1

Acquisition Time (sec)	1.0224
Date	30 Apr 2018 12:39:51
Date Stamp	30 Apr 2018 12:39:51
Frequency (MHz)	100.5977
Nucleus	13C
Number of Transients	64
Solvent	DMSO-d6
Temperature (degree C)	25.005

^{13}C NMR (101 MHz, *DMSO-d_6*) δ ppm 170.0 (s, 1 C), 164.7 (s, 1 C), 141.1 (s, 1 C), 134.5 (s, 1 C), 134.3 (s, 1 C), 132.2 (s, 1 C), 131.3 (s, 1 C), 129.0 (s, 2 C), 127.0 (s, 2 C), 123.0 (s, 1 C), 119.9 (s, 1 C), 116.6 (s, 1 C)

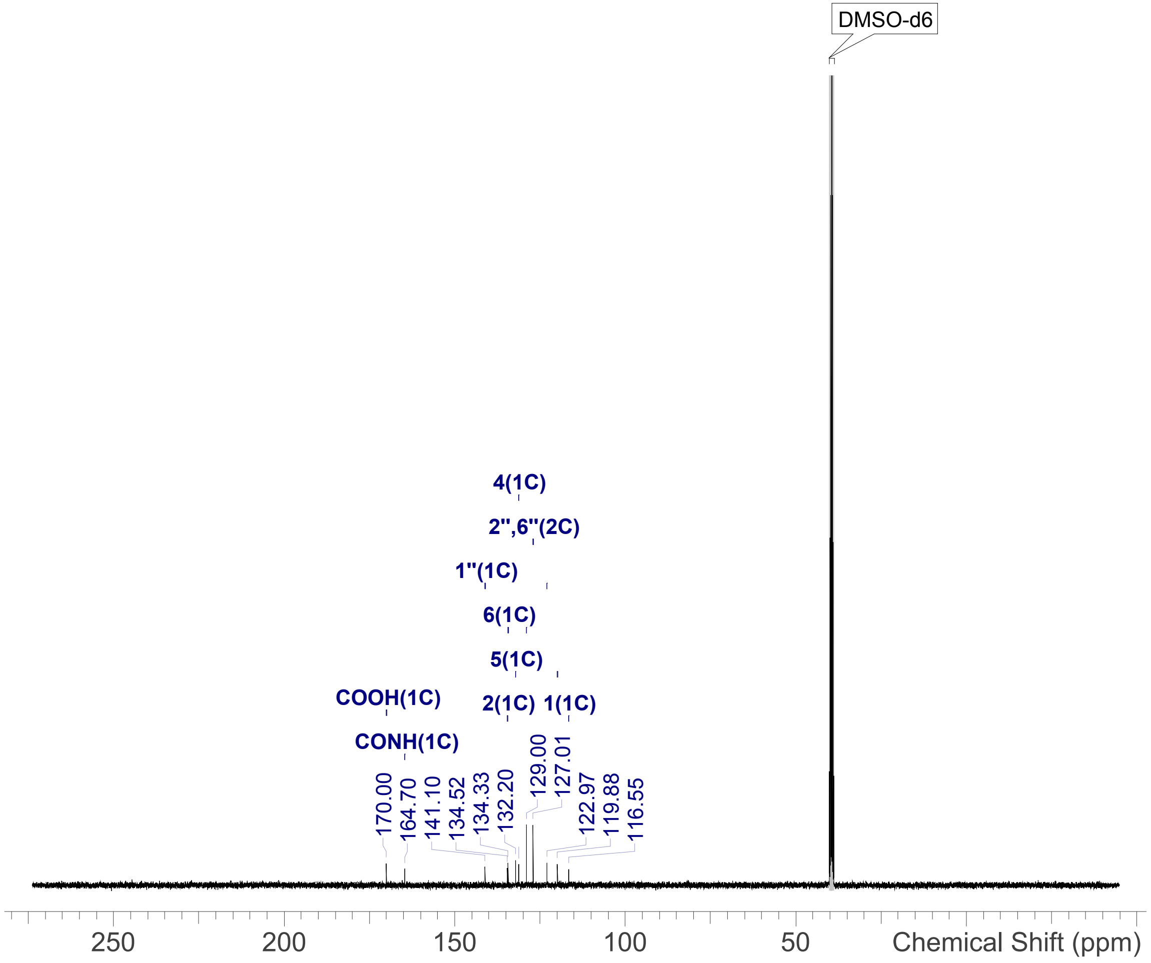

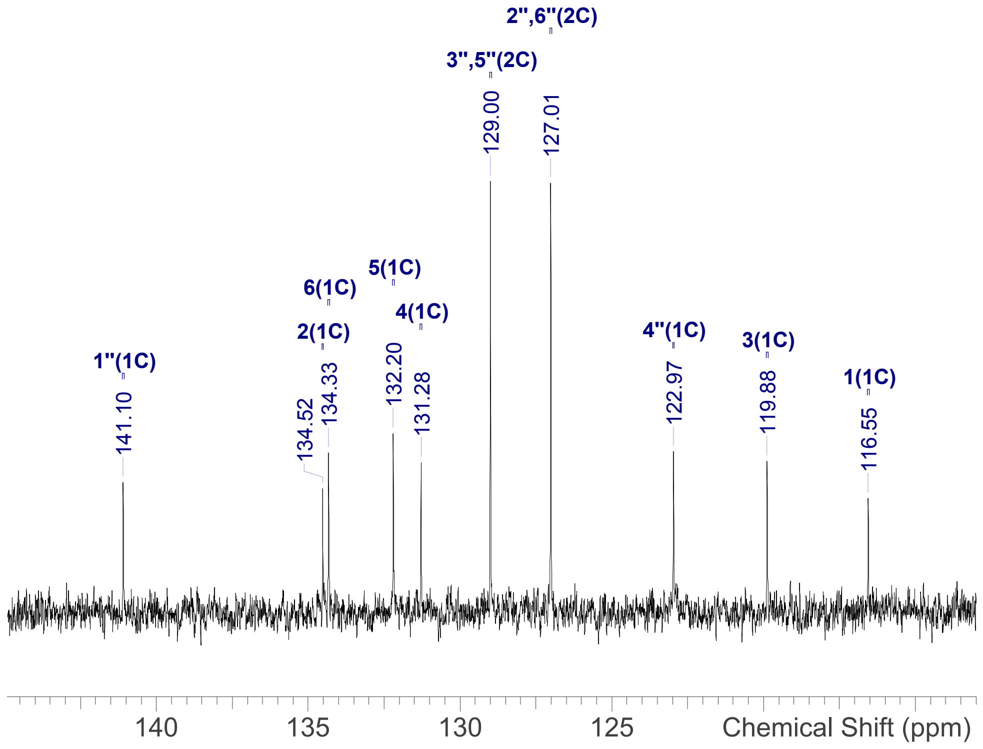

NVR-17_13C.spectrus

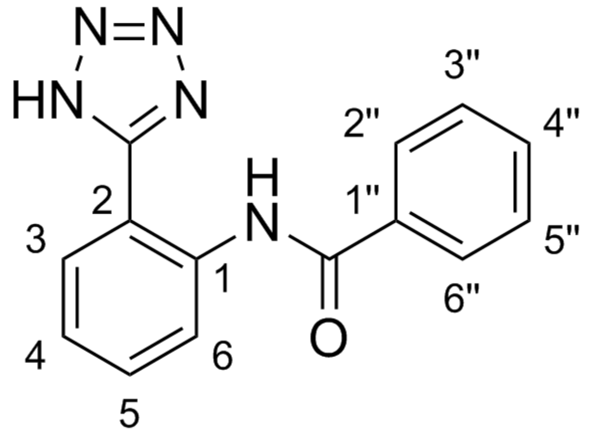

Shift (ppm)	H	m	J (Hz)	Assign
11.54	1	s	-	CONH
8.59	1	dd	8.4, 0.8	H6
8.05	3	m	-	H3, H6'', H2''
7.62	4	m	-	H5, H4'', H5'', H3''
7.37	1	td	7.6, 1.1	H4

Acquisition Time (sec)	6.5536
Date	31/07/2017 13:04:00
Date Stamp	31/07/2017 13:04:00
Frequency (MHz)	500.1930
Nucleus	1H
Number of Transients	16
Solvent	DMSO-d6

^{1}H NMR (500 MHz, *DMSO-d$_6$*) δ ppm 11.54 (s, 1 H), 8.59 (dd, *J*=8.4, 0.8 Hz, 1 H), 8.02 - 8.08 (m, 3 H), 7.57 - 7.68 (m, 4 H), 7.37 (td, *J*=7.6, 1.1 Hz, 1 H)

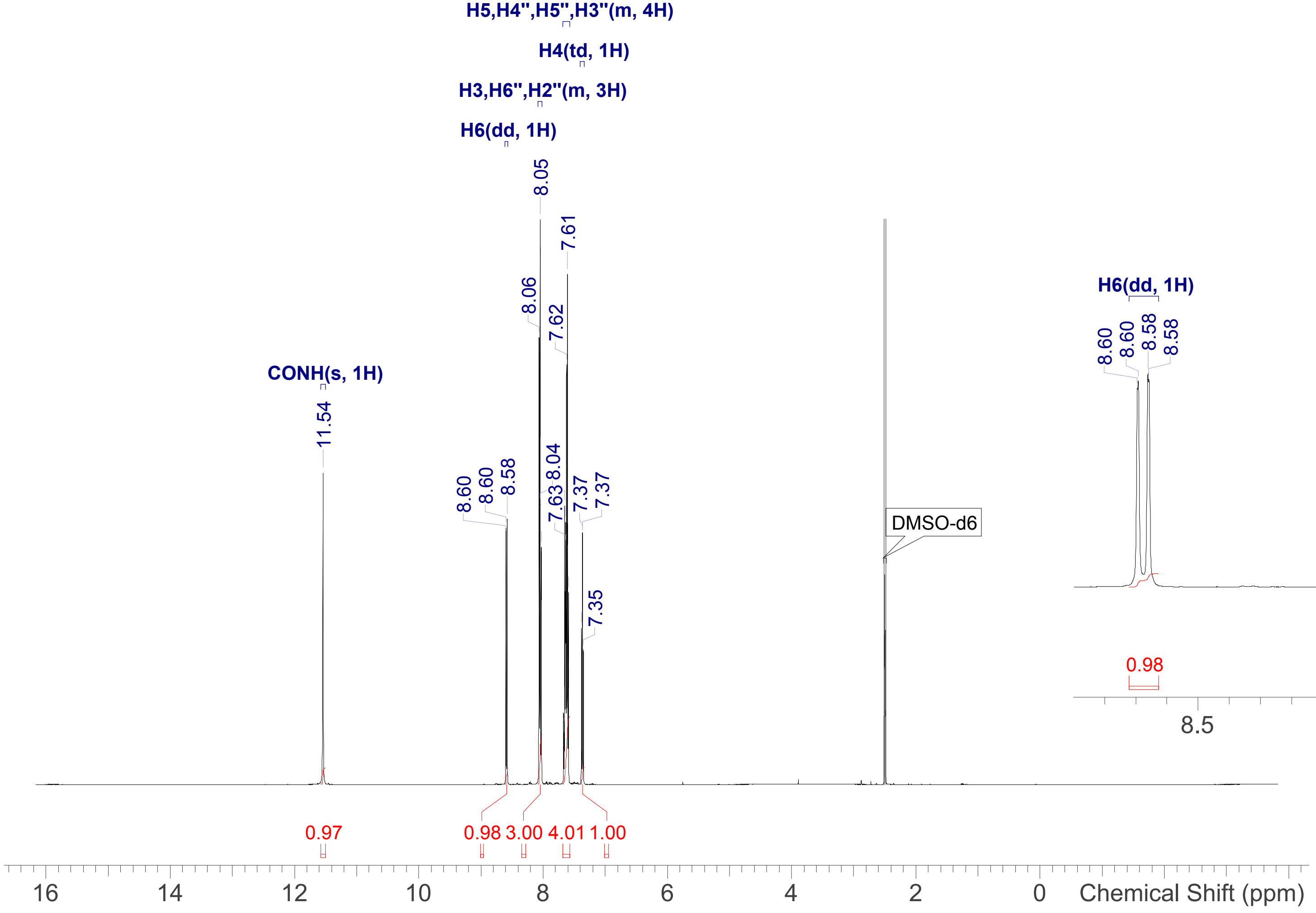

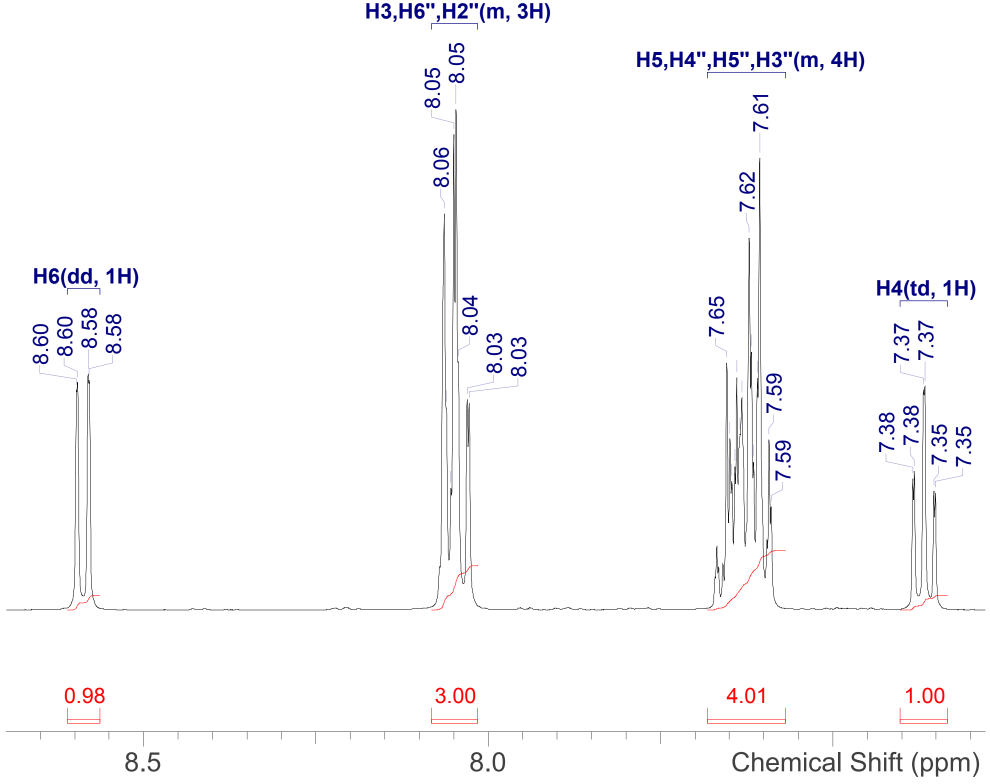

NVR-15_1H.spectrus

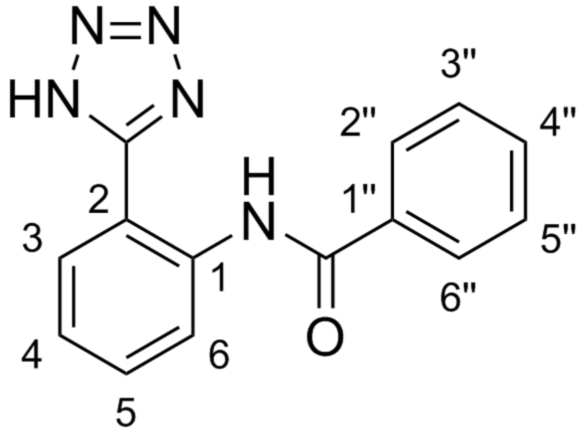

Shift (ppm)	C	m	Assign
165.1	1	s	CONH
154.6	1	br s	Tet-C
137.2	1	s	1
134.4	1	s	1''
132.2	1	s	4''
131.9	1	s	5
128.9	2	s	3'', 5''
128.7	1	s	4
127.3	2	s	2'', 6''
124.2	1	s	3
121.9	1	s	6
113.4	1	s	2

Acquisition Time (sec)	2.0447
Date	31/07/2017 18:48:00
Date Stamp	31/07/2017 18:48:00
Frequency (MHz)	125.7870
Nucleus	13C
Number of Transients	256
Solvent	DMSO-d6

^{13}C NMR (126 MHz, *DMSO-d$_6$*) δ ppm 165.1 (s, 1 C), 154.6 (br s, 1 C), 137.2 (s, 1 C), 134.4 (s, 1 C), 132.2 (s, 1 C), 131.9 (s, 1 C), 128.9 (s, 2 C), 128.7 (s, 1 C), 127.3 (s, 2 C), 124.2 (s, 1 C), 121.9 (s, 1 C), 113.4 (s, 1 C)

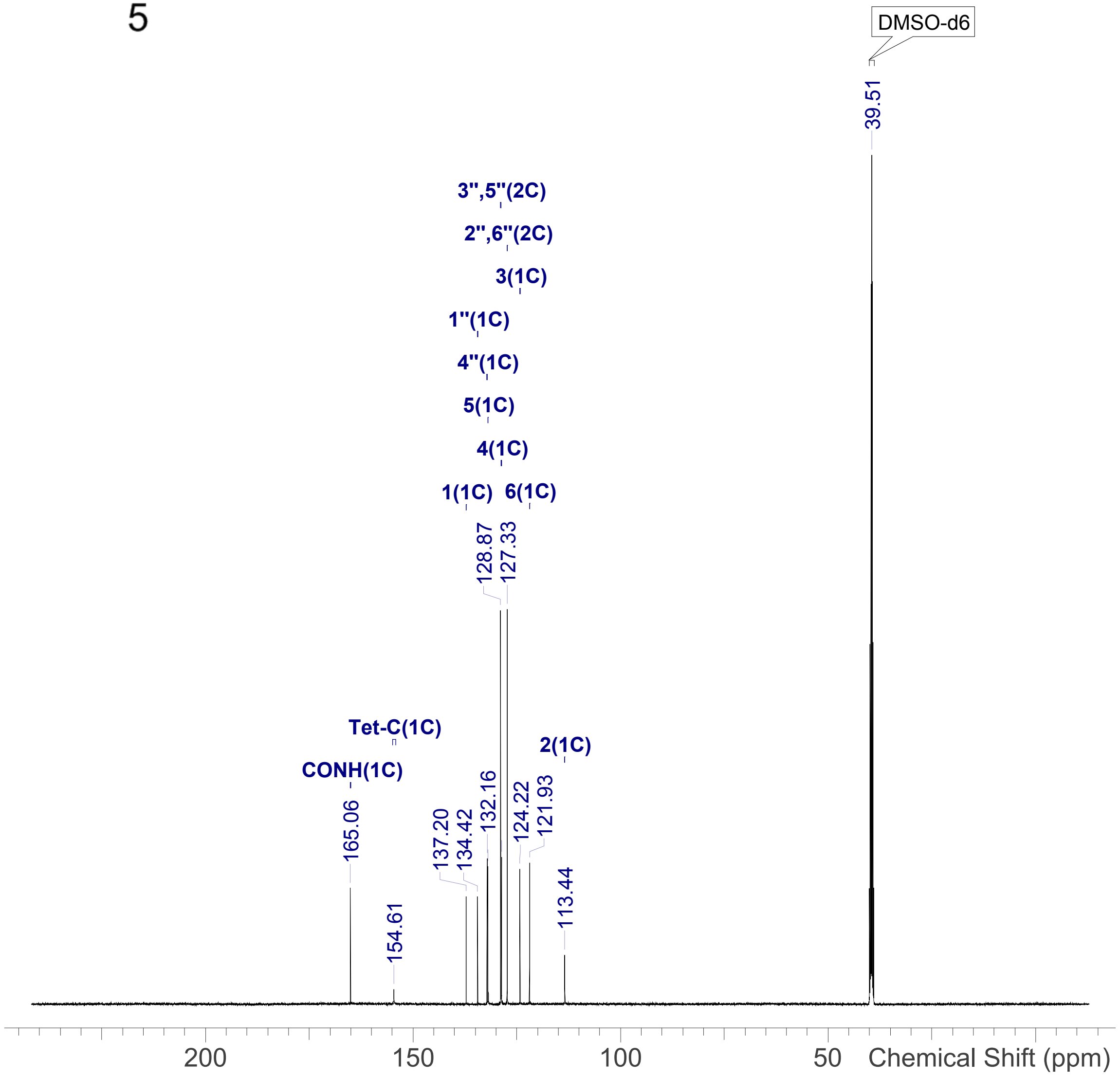

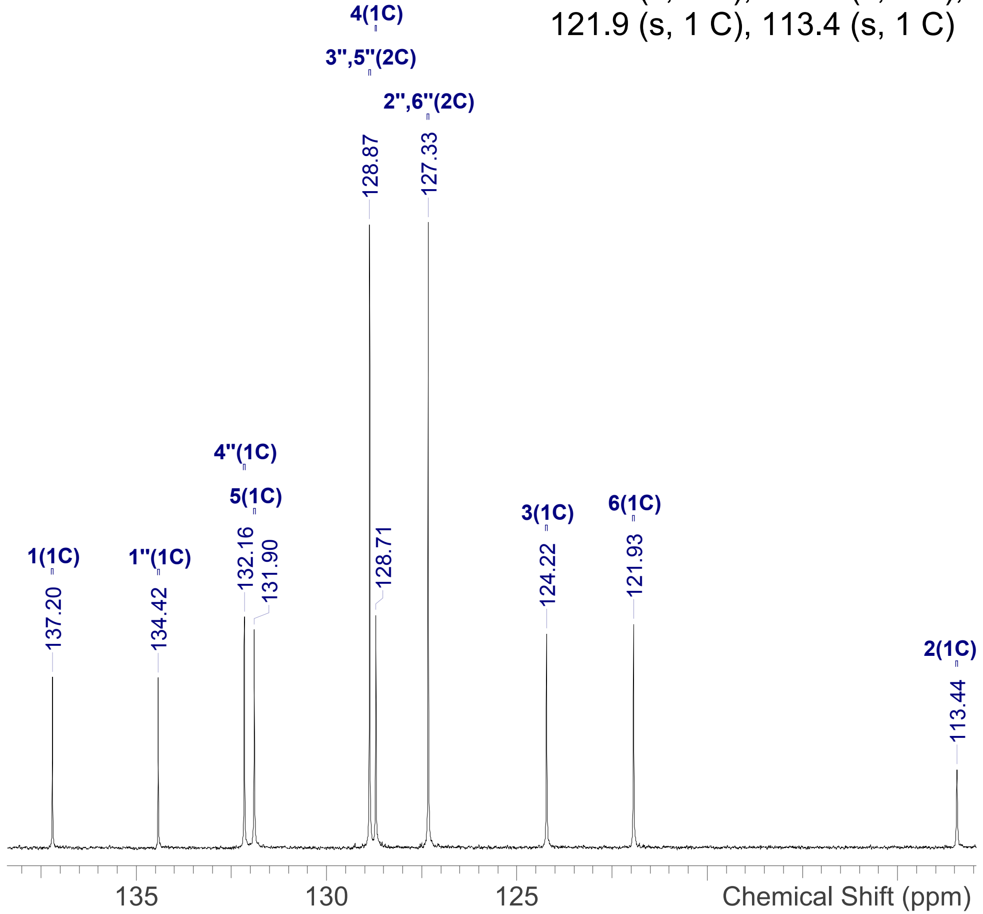

NVR-15_13C.spectrus

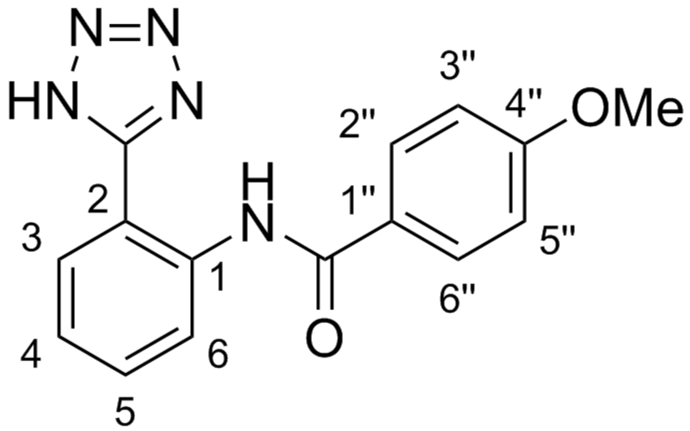

Shift (ppm)	H	m	J (Hz)	Assign
11.50	1	s	-	CONH
8.60	1	dd	8.4, 0.8	H6
8.08	1	dd	7.9, 1.4	H3
8.02	2	br d	9.2	H2'', H6''
7.60	1	ddd	8.4, 7.4, 1.4	H5
7.32	1	td	7.6, 1.1	H4
7.12	2	br d	8.9	H3'', H5''
3.85	3	s	-	OMe

Acquisition Time (sec)	6.5536
Date	31/07/2017 12:54:00
Date Stamp	31/07/2017 12:54:00
Frequency (MHz)	500.1930
Nucleus	1H
Number of Transients	16
Solvent	DMSO-d6

^{1}H NMR (500 MHz, *DMSO-d*$_6$) δ ppm 11.50 (s, 1 H), 8.60 (dd, *J*=8.4, 0.8 Hz, 1 H), 8.08 (dd, *J*=7.9, 1.4 Hz, 1 H), 8.02 (br d, *J*=9.2 Hz, 2 H), 7.60 (ddd, *J*=8.4, 7.4, 1.4 Hz, 1 H), 7.32 (td, *J*=7.6, 1.1 Hz, 1 H), 7.12 (br d, *J*=8.9 Hz, 2 H), 3.85 (s, 3 H)

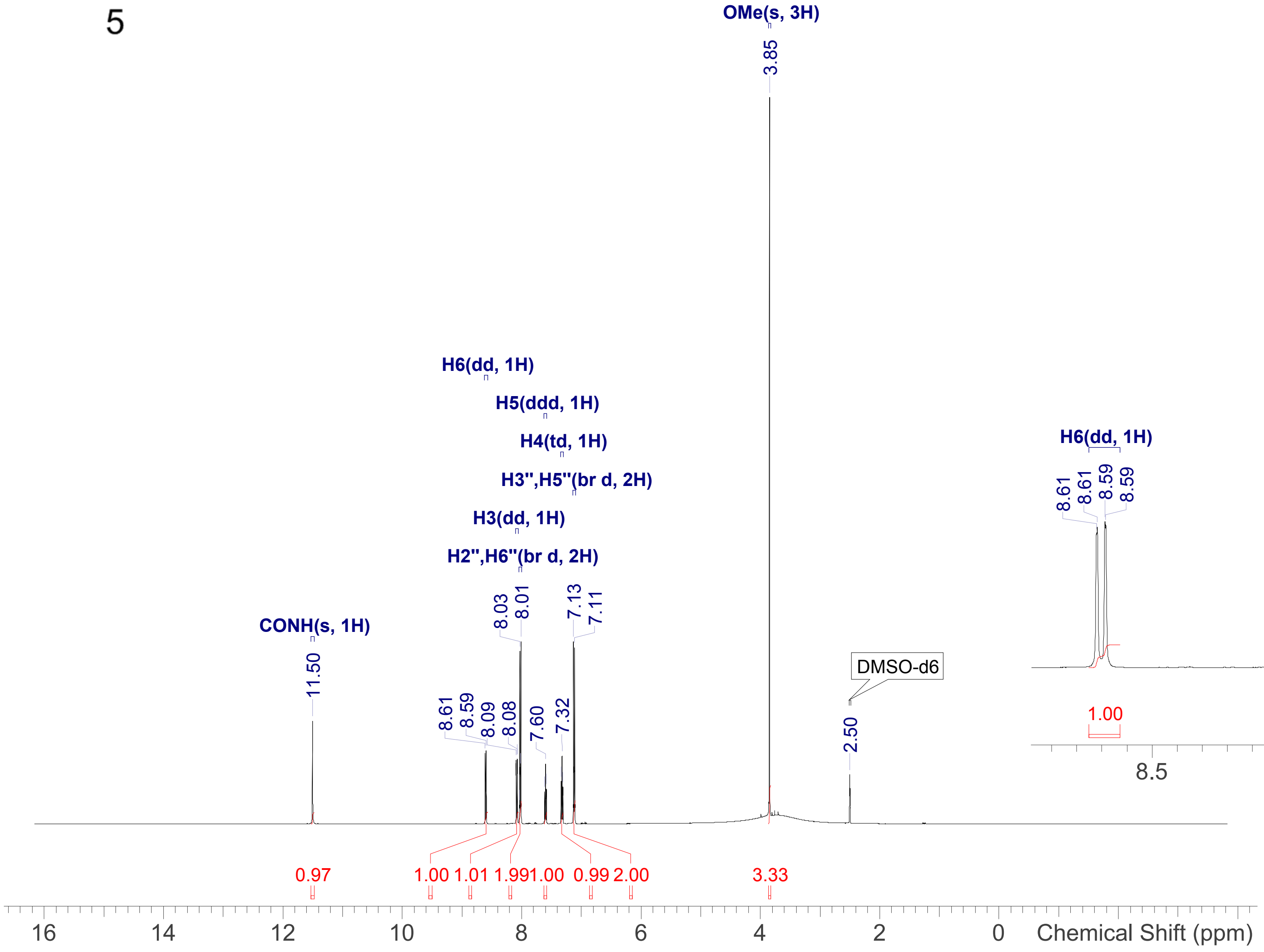

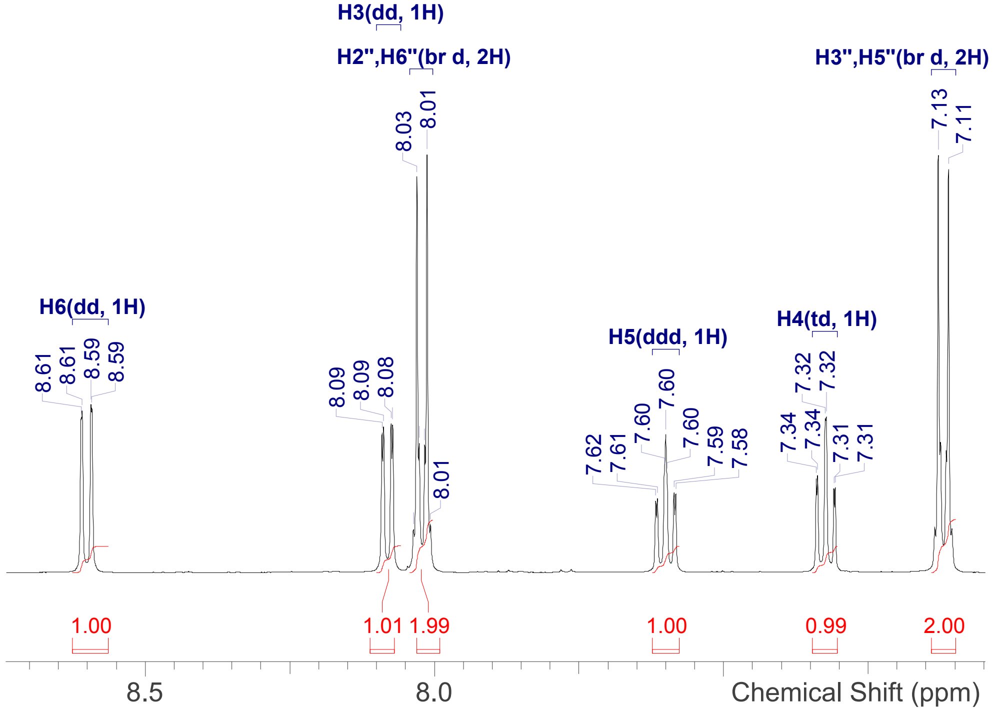

NVR-29_1H.spectrus

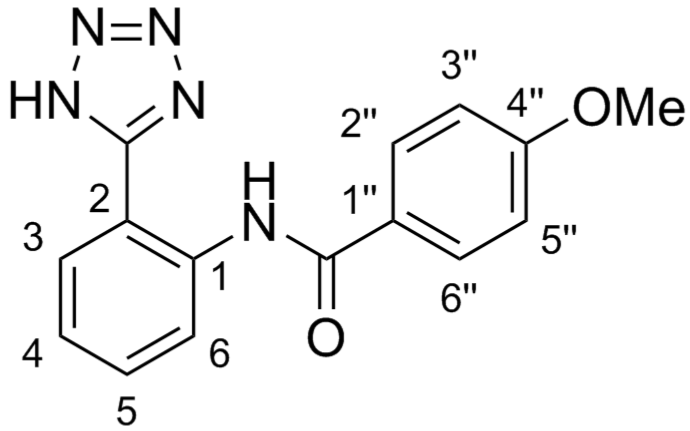

Shift (ppm)	C	m	Assign
164.6	1	s	CONH
162.4	1	s	4''
154.6	1	br s	Tet-C
137.5	1	s	1
132.0	1	s	5
129.3	2	s	2'', 6''
128.8	1	s	3
126.5	1	s	1''
123.9	1	s	4
121.7	1	s	6
114.2	2	s	3'', 5''
112.9	1	s	2
55.6	1	s	OMe

Acquisition Time (sec)	2.0447
Date	31/07/2017 17:20:00
Date Stamp	31/07/2017 17:20:00
Frequency (MHz)	125.7870
Nucleus	13C
Number of Transients	256
Solvent	DMSO-d6

^{13}C NMR (126 MHz, *DMSO-d_6*) δ ppm 164.6 (s, 1 C), 162.4 (s, 1 C), 154.6 (br s, 1 C), 137.5 (s, 1 C), 132.0 (s, 1 C), 129.3 (s, 2 C), 128.8 (s, 1 C), 126.5 (s, 1 C), 123.9 (s, 1 C), 121.7 (s, 1 C), 114.2 (s, 2 C), 112.9 (s, 1 C), 55.6 (s, 1 C)

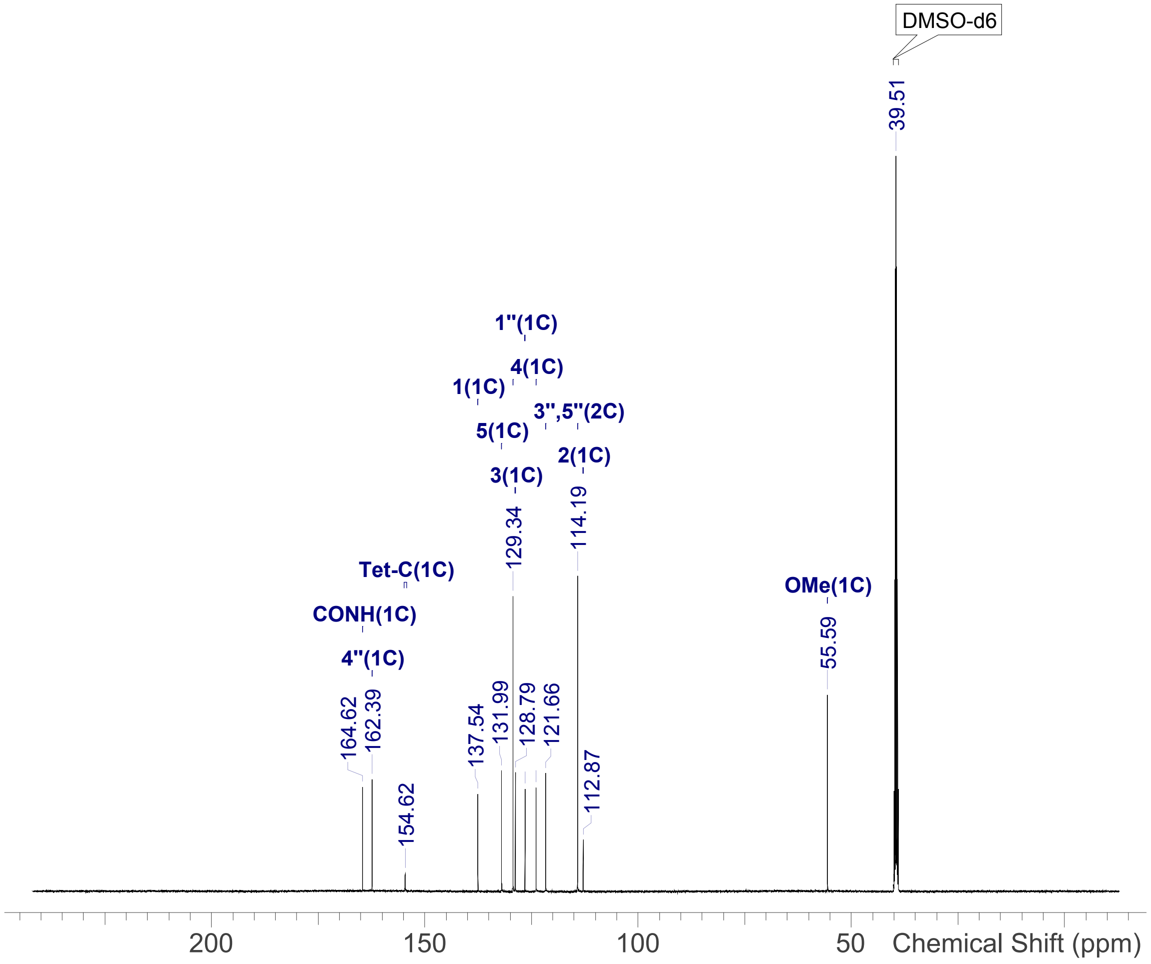

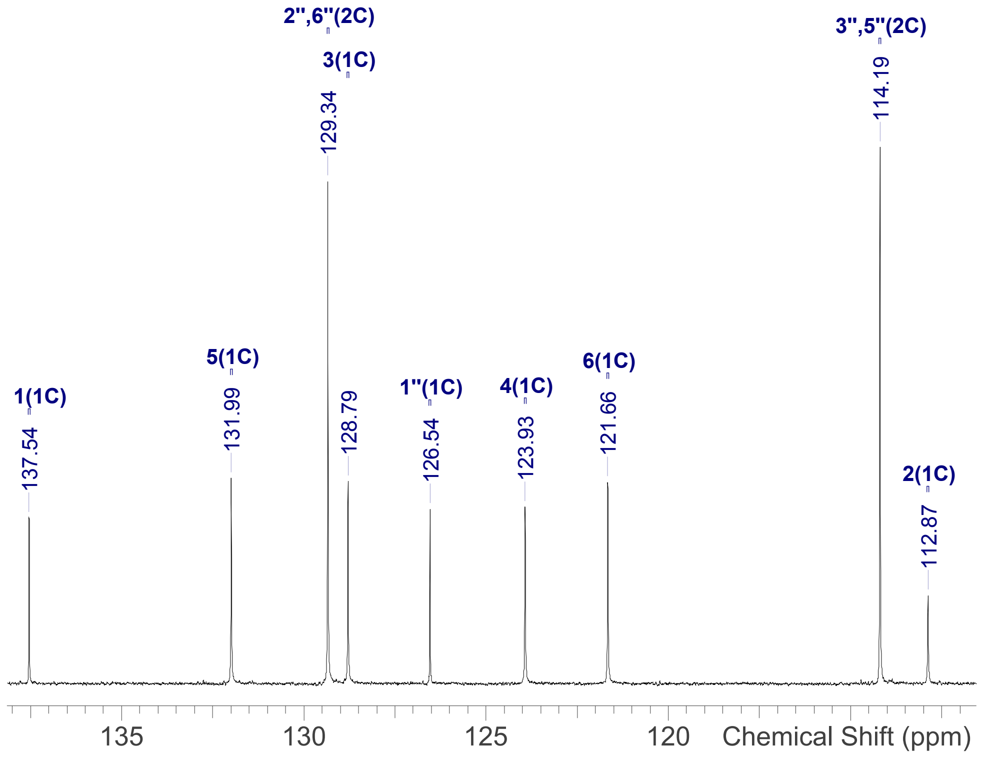

NVR-29_13C.spectrus

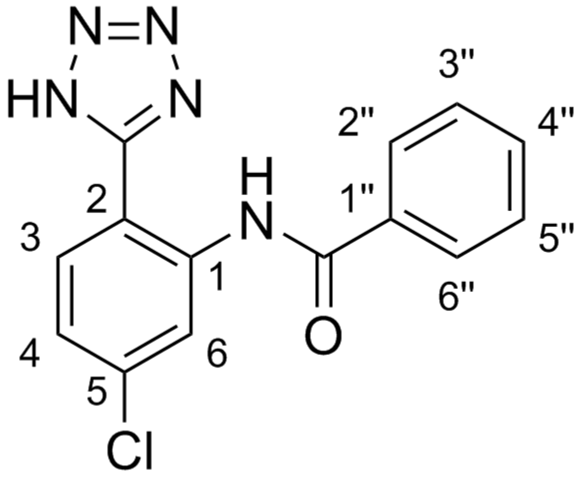

Shift (ppm)	H	m	J (Hz)	Assign
11.75	5	s	-	NH
8.74	1	d	2.1	H6
8.09	1	d	8.4	H3
8.06	2	br d	7.0	H6", H2"
7.68	1	br t	7.0	H4"
7.62	2	br t	6.9	H5", H3"
7.46	1	dd	8.4, 2.1	H4

Acquisition Time (sec)	6.5536
Date	03/08/2017 16:02:00
Date Stamp	03/08/2017 16:02:00
Frequency (MHz)	500.1930
Nucleus	1H
Number of Transients	16
Solvent	DMSO-d6

^{1}H NMR (500 MHz, *DMSO-d_6*) δ ppm 11.75 (s, 5 H), 8.74 (d, *J*=2.1 Hz, 1 H), 8.09 (d, *J*=8.4 Hz, 1 H), 8.06 (br d, *J*=7.0 Hz, 2 H), 7.68 (br t, *J*=7.0 Hz, 1 H), 7.62 (br t, *J*=6.9 Hz, 2 H), 7.46 (dd, *J*=8.4, 2.1 Hz, 1 H)

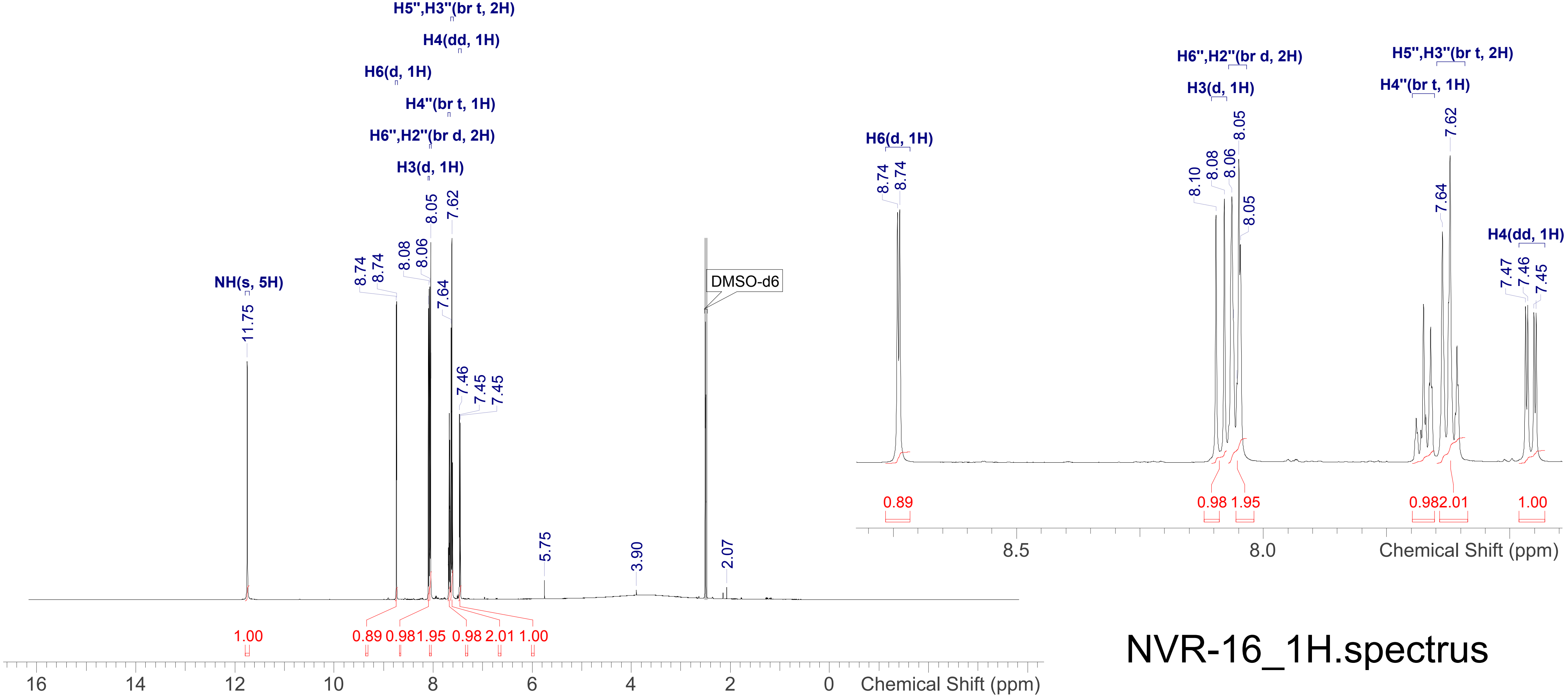

NVR-16_1H.spectrus

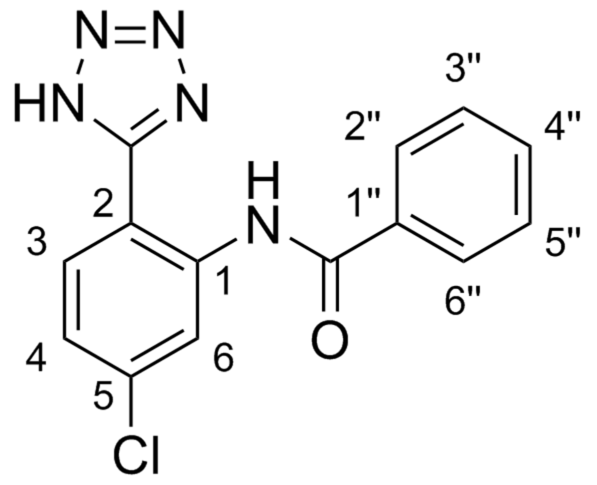

Shift (ppm)	C	m	Assign
165.3	1	s	CONH
154.4	1	br s	Tet-C
138.3	1	s	1
136.2	1	s	1"
134.0	1	s	5
132.5	1	s	4"
130.2	1	s	3
129.0	2	s	5", 3"
127.4	2	s	6", 2"
124.0	1	s	4
120.9	1	s	6
112.2	1	s	2

Acquisition Time (sec)	2.0447
Date	05/08/2017 06:51:00
Date Stamp	05/08/2017 06:51:00
Frequency (MHz)	125.7870
Nucleus	13C
Number of Transients	256
Solvent	DMSO-d6

^{13}C NMR (126 MHz, *DMSO-d_6*) δ ppm 165.3 (s, 1 C), 154.4 (br s, 1 C), 138.3 (s, 1 C), 136.2 (s, 1 C), 134.0 (s, 1 C), 132.5 (s, 1 C), 130.2 (s, 1 C), 129.0 (s, 2 C), 127.4 (s, 2 C), 124.0 (s, 1 C), 120.9 (s, 1 C), 112.2 (s, 1 C)

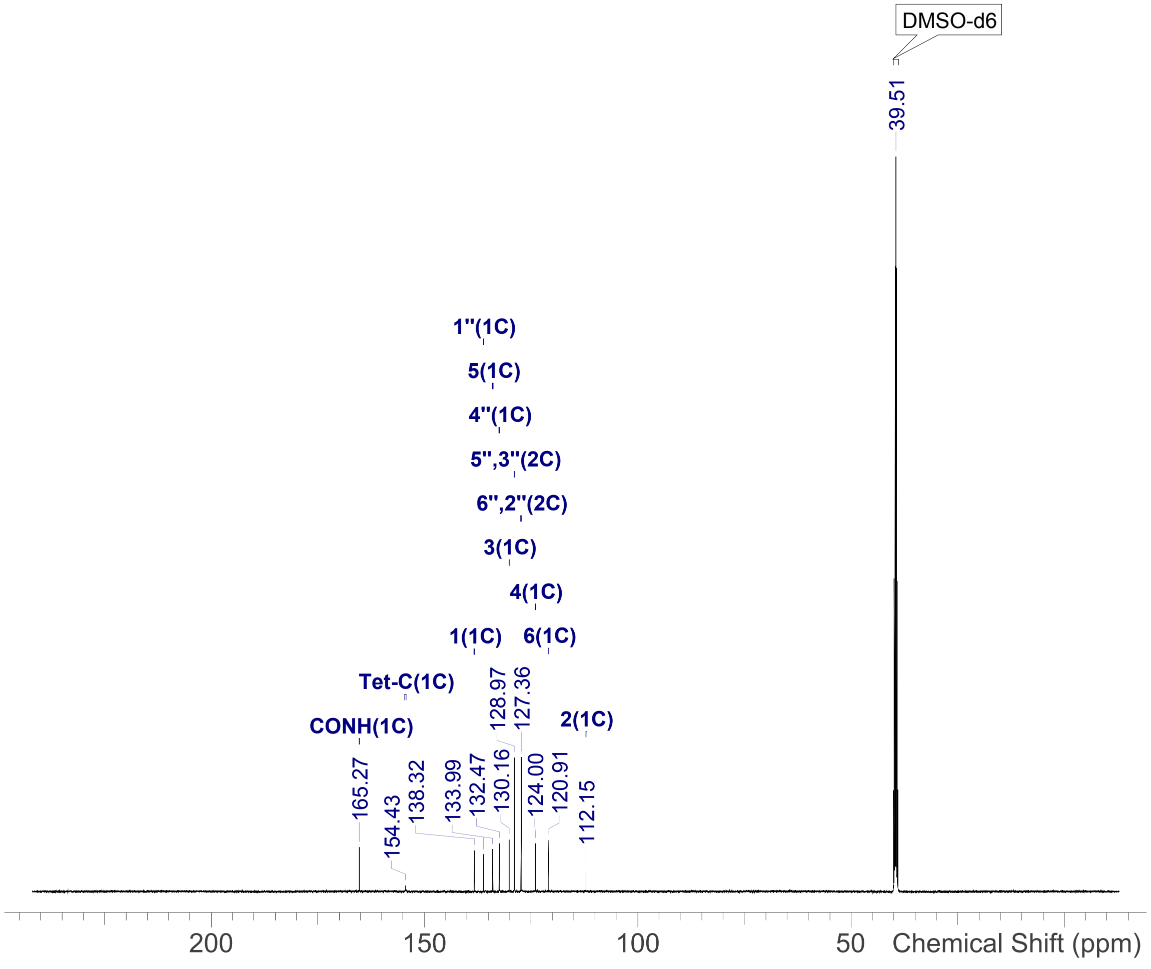

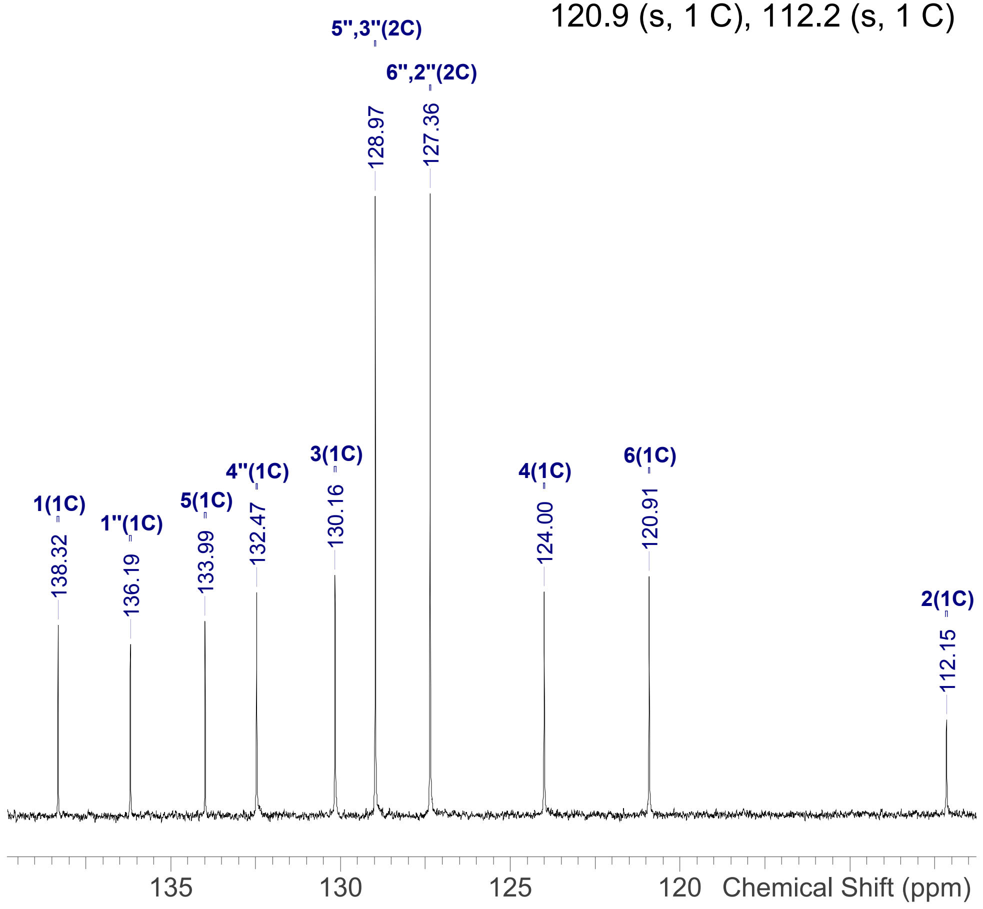

NVR-16_13C.spectrus

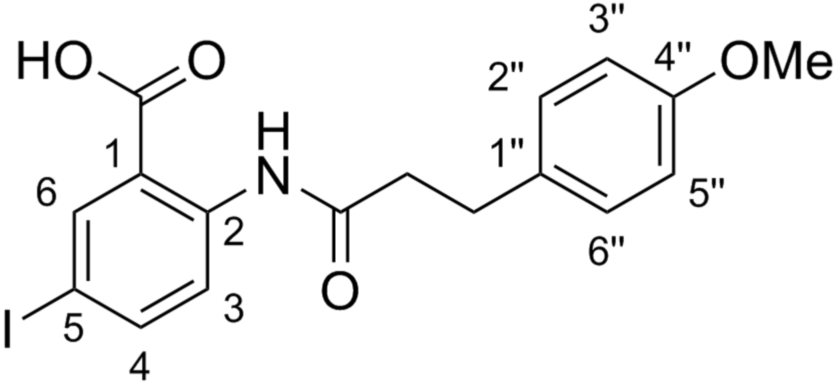

Shift (ppm)	H	m	J (Hz)	Assign
11.02	1	s	-	NH
8.28	1	d	8.9	3
8.20	1	d	2.3	6
7.88	1	dd	8.8, 2.2	4
7.15	2	br d	8.5	6", 2"
6.82	2	br d	8.5	5", 3"
3.70	3	s	-	OMe
2.86	2	t	7.6	CH2Ph
2.66	2	t	7.6	COCH2

Acquisition Time (sec)	6.5536
Date	19/07/2017 10:55:00
Date Stamp	19/07/2017 10:55:00
Frequency (MHz)	500.1930
Nucleus	1H
Number of Transients	16
Solvent	DMSO-d6

^{1}H NMR (500 MHz, *DMSO-d_6*) δ ppm 11.02 (s, 1 H), 8.28 (d, *J*=8.9 Hz, 1 H), 8.20 (d, *J*=2.3 Hz, 1 H), 7.88 (dd, *J*=8.8, 2.2 Hz, 1 H), 7.15 (br d, *J*=8.5 Hz, 2 H), 6.82 (br d, *J*=8.5 Hz, 2 H), 3.70 (s, 3 H), 2.86 (t, *J*=7.6 Hz, 2 H), 2.66 (t, *J*=7.6 Hz, 2 H)

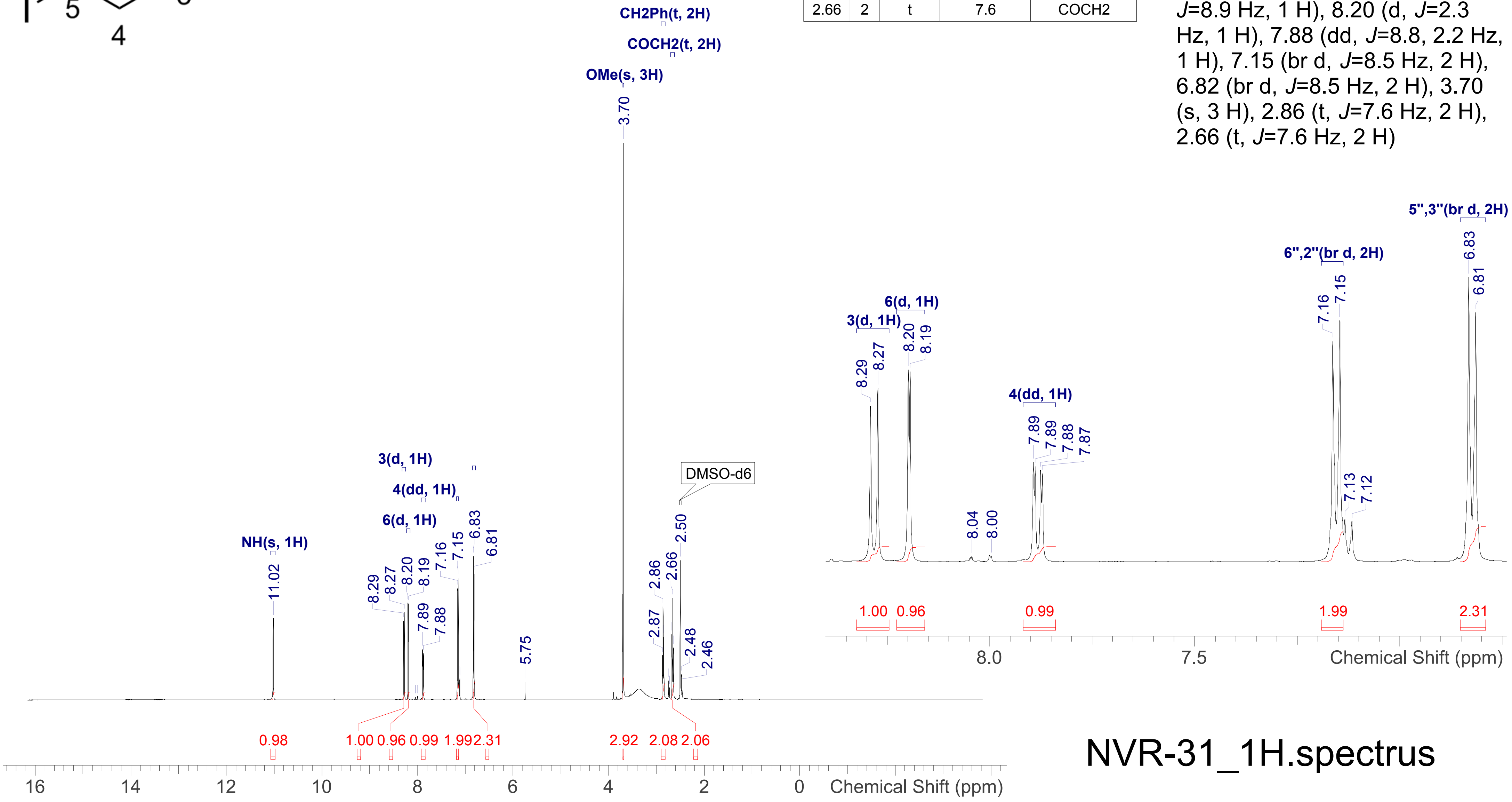

NVR-31_1H.spectrus

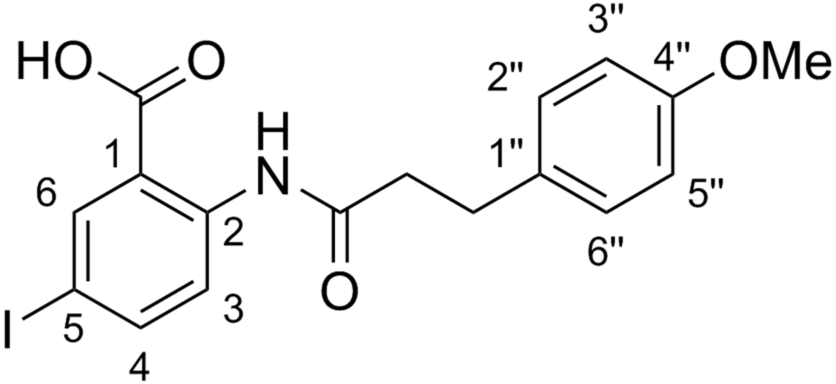

Shift (ppm)	C	m	Assign
170.7	1	s	CONH
168.1	1	s	COOH
157.6	1	s	4''
142.2	1	s	4
140.3	1	s	2
138.9	1	s	6
132.5	1	s	1''
129.2	2	s	2'', 6''
122.2	1	s	3
118.7	1	s	1
113.7	2	s	3'', 5''
85.7	1	s	5
55.0	1	s	OMe
29.6	1	s	CH2Ph

Acquisition Time (sec)	2.0447
Date	20/07/2017 01:47:00
Date Stamp	20/07/2017 01:47:00
Frequency (MHz)	125.7870
Nucleus	13C
Number of Transients	2048
Solvent	DMSO-d6

^{13}C NMR (126 MHz, *DMSO-d_6*) δ ppm 170.7 (s, 1 C), 168.1 (s, 1 C), 157.6 (s, 1 C), 142.2 (s, 1 C), 140.3 (s, 1 C), 138.9 (s, 1 C), 132.5 (s, 1 C), 129.2 (s, 2 C), 122.2 (s, 1 C), 118.7 (s, 1 C), 113.7 (s, 2 C), 85.7 (s, 1 C), 55.0 (s, 1 C), 29.6 (s, 1 C)

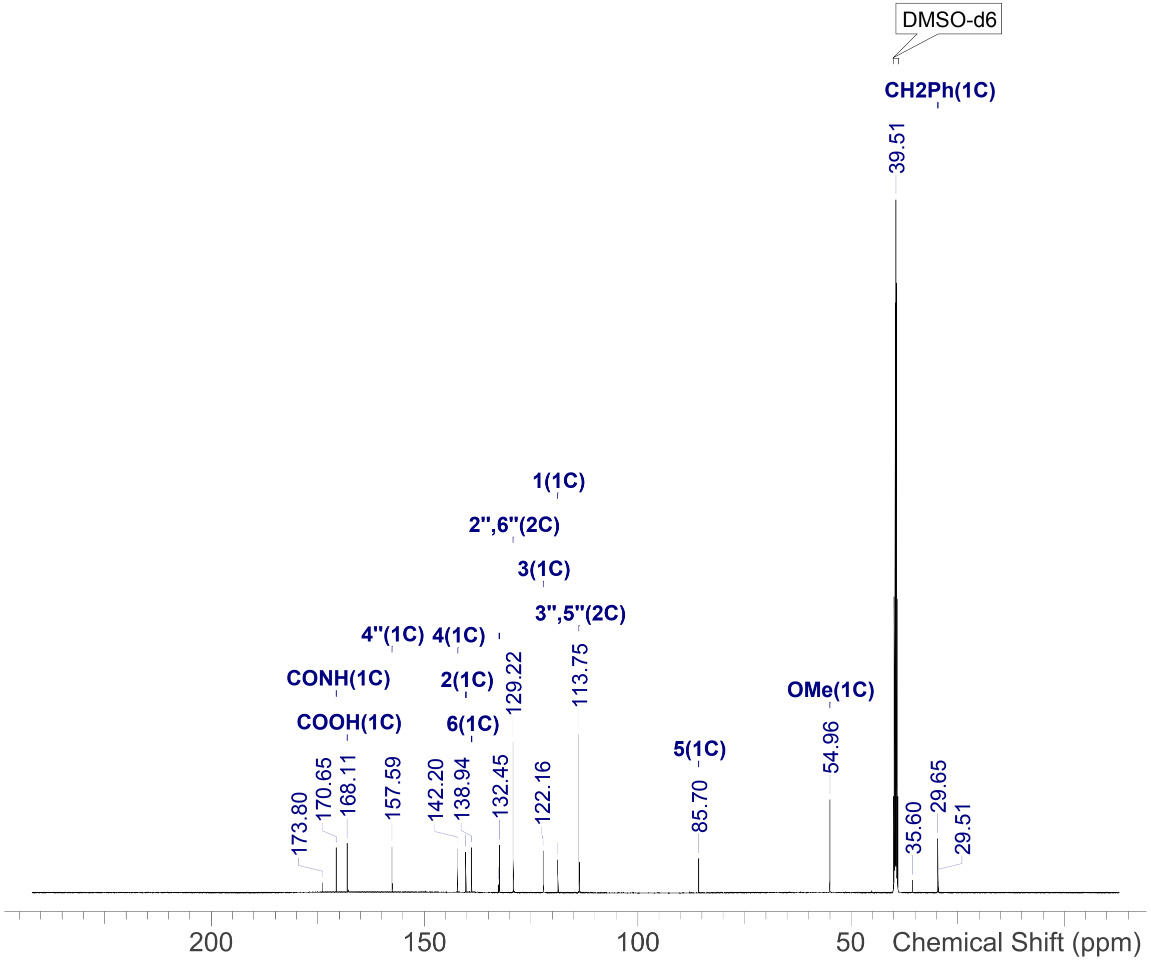

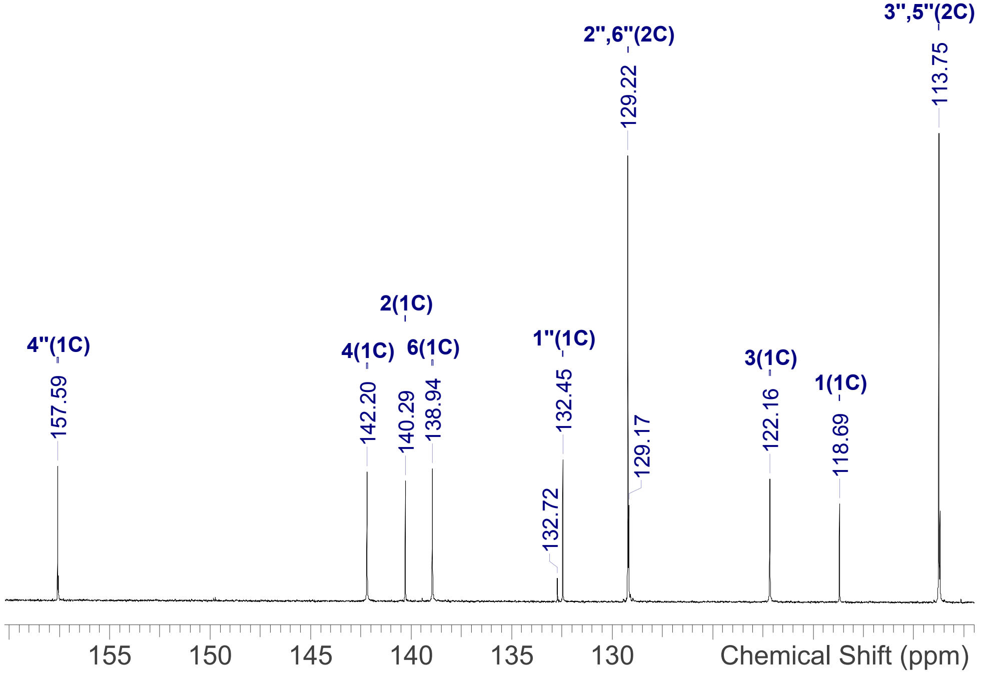

NVR-31_13C.spectrus

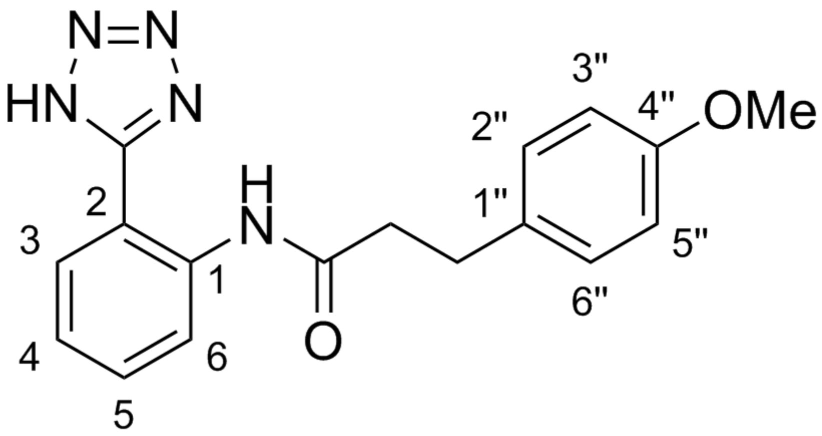

Shift (ppm)	H	m	J (Hz)	Assign
10.55	1	s	-	CONH
8.27	1	d	8.2	H6
7.89	1	dd	7.9, 1.2	H3
7.56	1	br t	8.2	H5
7.30	1	br t	7.6	H4
7.16	2	d	8.5	H2", H6"
6.81	2	br d	8.5	H3", H5"
3.69	3	s	-	OMe
2.88	2	t	7.6	CH2Ar
2.67	2	t	7.6	COCH2

Acquisition Time (sec)	6.5536
Date	02/08/2017 15:18:00
Date Stamp	02/08/2017 15:18:00
Frequency (MHz)	500.1930
Nucleus	1H
Number of Transients	16
Solvent	DMSO-d6

^{1}H NMR (500 MHz, *DMSO-d*$_6$) δ ppm 10.55 (s, 1 H), 8.27 (d, *J*=8.2 Hz, 1 H), 7.89 (dd, *J*=7.9, 1.2 Hz, 1 H), 7.56 (br t, *J*=8.2 Hz, 1 H), 7.30 (br t, *J*=7.6 Hz, 1 H), 7.16 (d, *J*=8.5 Hz, 2 H), 6.81 (br d, *J*=8.5 Hz, 2 H), 3.69 (s, 3 H), 2.88 (t, *J*=7.6 Hz, 2 H), 2.67 (t, *J*=7.6 Hz, 2 H)

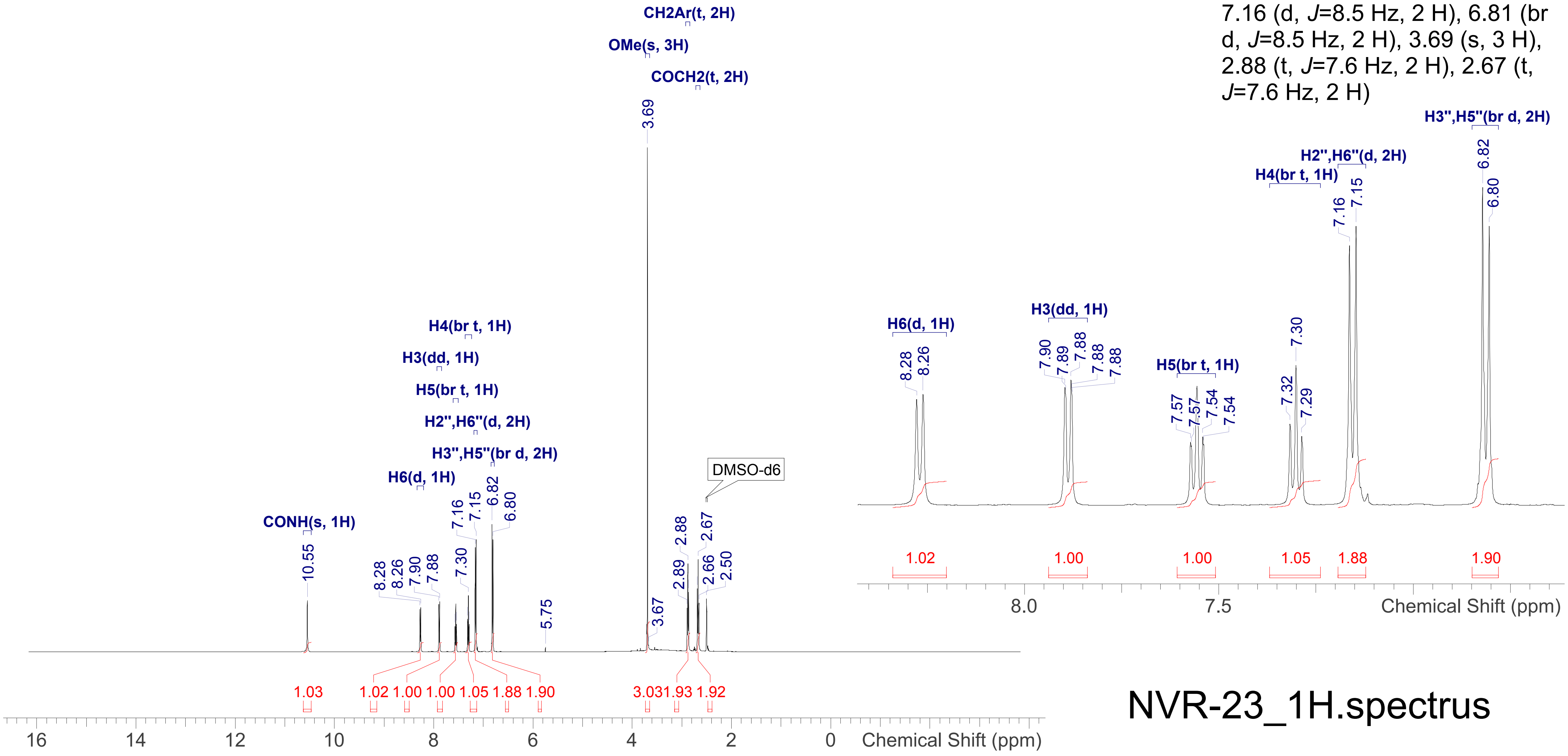

NVR-23_1H.spectrus

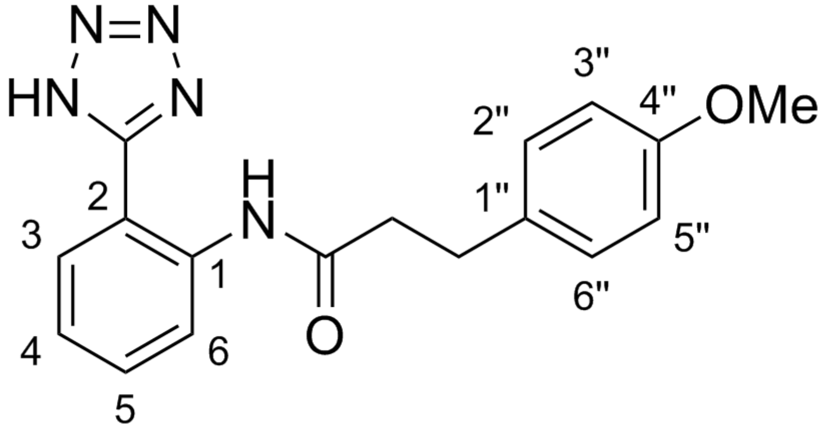

Shift (ppm)	C	m	Assign
170.7	1	s	CONH
157.5	1	s	4"
154.3	1	br s	Tet-C
137.0	1	s	1
132.6	1	s	1"
131.7	1	s	6
129.2	2	s	2", 6"
128.9	1	s	3
124.0	1	s	4
122.2	1	s	5
113.7	2	s	3", 5"
54.9	1	s	OMe
29.8	1	s	CH2Ar

Acquisition Time (sec)	2.0447
Date	02/08/2017 22:05:00
Date Stamp	02/08/2017 22:05:00
Frequency (MHz)	125.7870
Nucleus	13C
Number of Transients	256
Solvent	DMSO-d6

^{13}C NMR (126 MHz, *DMSO-d_6*) δ ppm 170.7 (s, 1 C), 157.5 (s, 1 C), 154.3 (br s, 1 C), 137.0 (s, 1 C), 132.6 (s, 1 C), 131.7 (s, 1 C), 129.2 (s, 2 C), 128.9 (s, 1 C), 124.0 (s, 1 C), 122.2 (s, 1 C), 113.7 (s, 2 C), 54.9 (s, 1 C), 29.8 (s, 1 C)

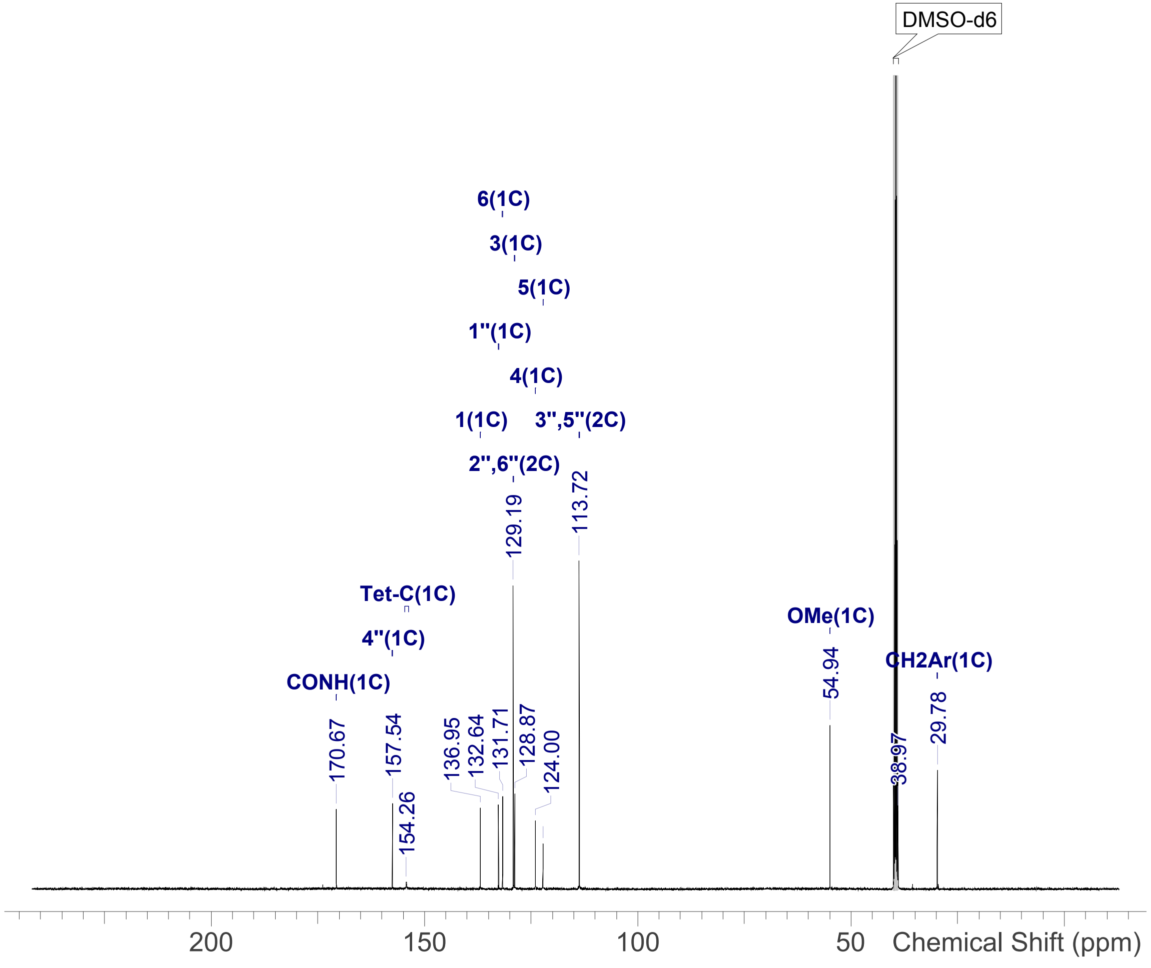

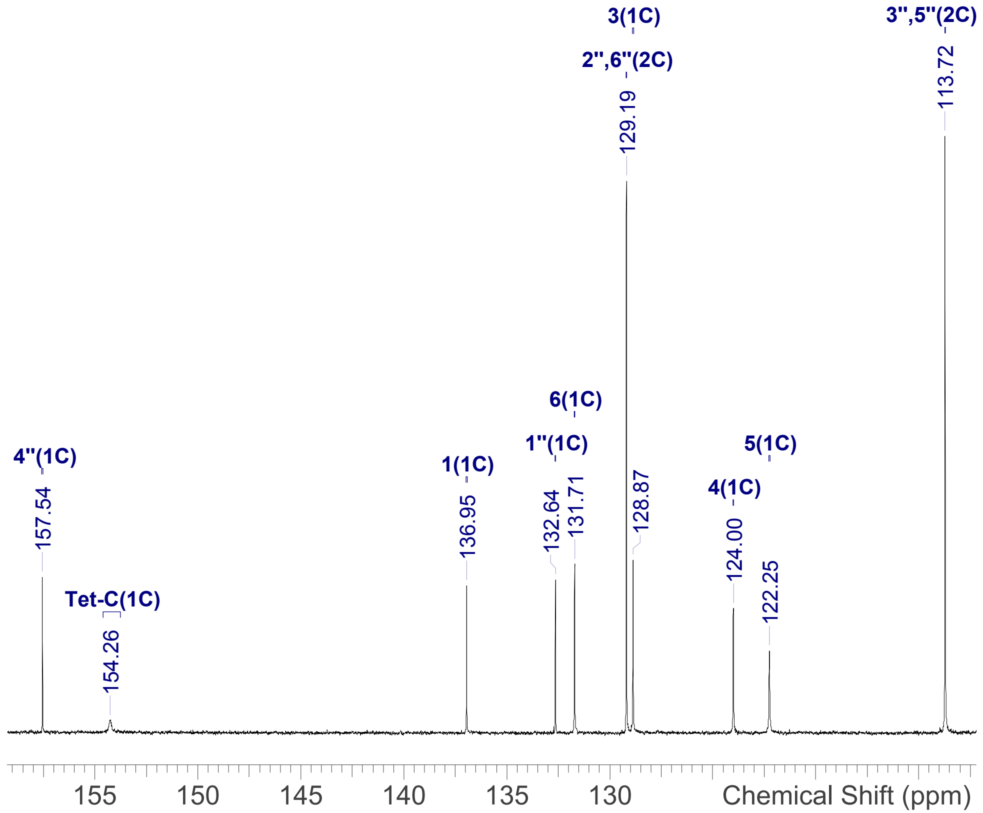

NVR-23_13C.spectrus

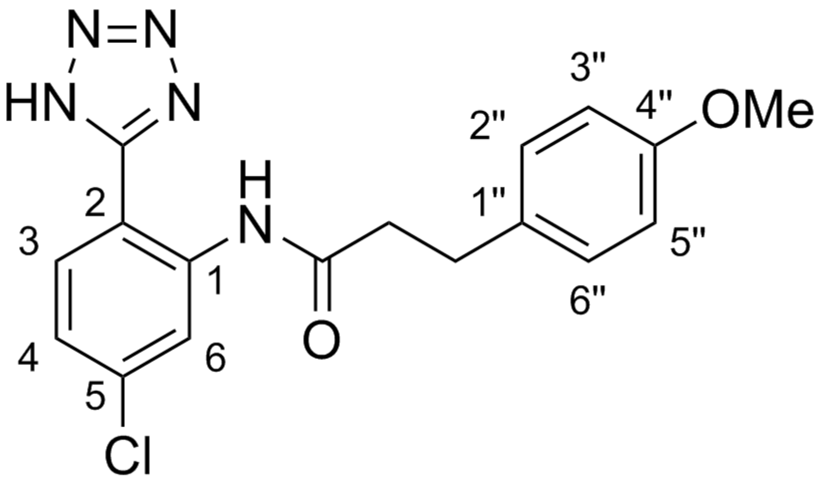

Shift (ppm)	H	m	J (Hz)	Assign
10.83	1	s	-	NH
8.44	1	d	2.1	6
8.00	1	d	8.4	3
7.39	1	dd	8.5, 2.2	4
7.16	2	d	8.5	6", 2"
6.82	2	br d	8.7	5", 3"
3.69	3	s	-	OMe
2.89	2	t	7.5	CH2Ph
2.71	2	t	7.5	COCH2

Acquisition Time (sec)	6.5536
Date	03/08/2017 02:13:00
Date Stamp	03/08/2017 02:13:00
Frequency (MHz)	500.1930
Nucleus	1H
Number of Transients	16
Solvent	DMSO-d6

^{1}H NMR (500 MHz, *DMSO-d_6*) δ ppm 10.83 (s, 1 H), 8.44 (d, *J*=2.1 Hz, 1 H), 8.00 (d, *J*=8.4 Hz, 1 H), 7.39 (dd, *J*=8.5, 2.2 Hz, 1 H), 7.16 (d, *J*=8.5 Hz, 2 H), 6.82 (br d, *J*=8.7 Hz, 2 H), 3.69 (s, 3 H), 2.89 (t, *J*=7.5 Hz, 2 H), 2.71 (t, *J*=7.5 Hz, 2 H)

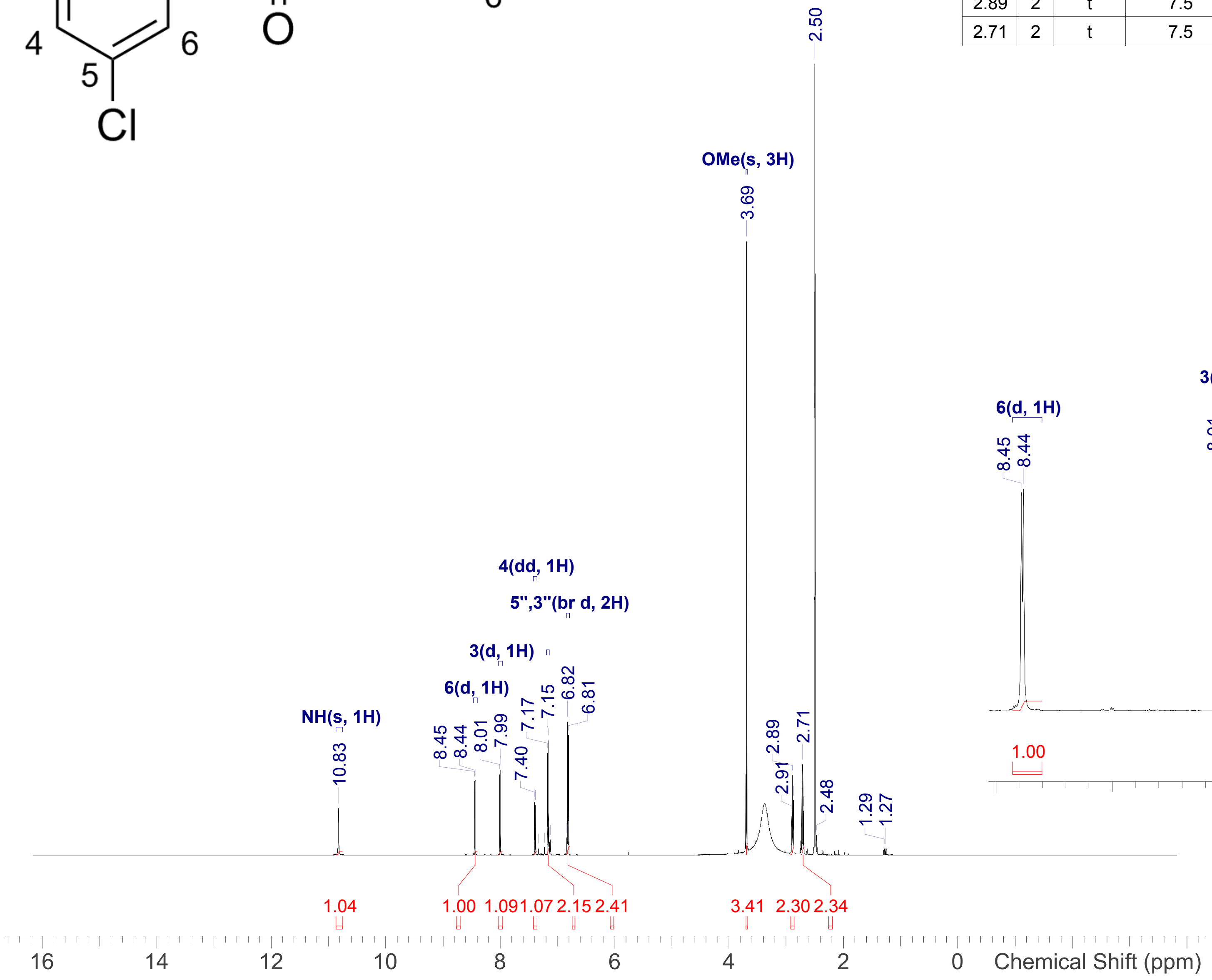

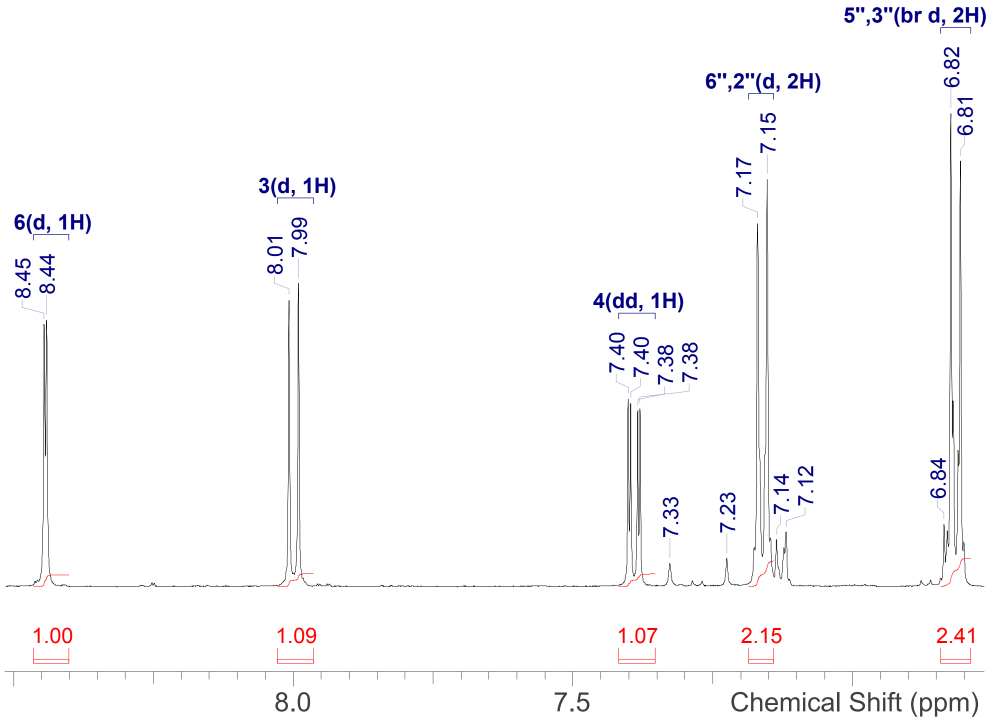

NVR-21_1H.spectrus

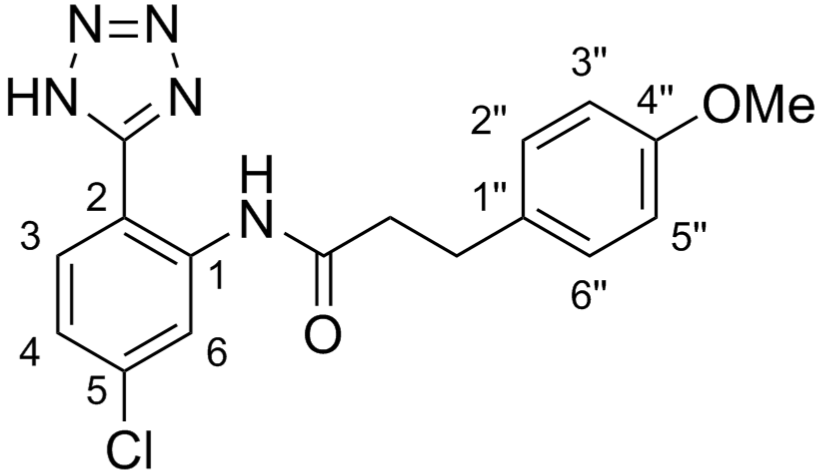

Shift (ppm)	C	m	Assign
171.1	1	s	CONH
157.6	1	s	Tet-C
138.0	1	s	1
135.8	1	s	5
132.5	1	s	1"
130.4	1	s	3
129.2	2	s	2", 6"
123.7	1	s	4
121.0	1	s	6
113.7	2	s	3", 5"
112.4	1	s	2
54.9	1	s	OMe
29.6	1	s	CH2Ar

Acquisition Time (sec)	2.0447
Date	03/08/2017 02:27:00
Date Stamp	03/08/2017 02:27:00
Frequency (MHz)	125.7870
Nucleus	13C
Number of Transients	256
Solvent	DMSO-d6

^{13}C NMR (126 MHz, *DMSO-d$_6$*) δ ppm 171.1 (s, 1 C), 157.6 (s, 1 C), 138.0 (s, 1 C), 135.8 (s, 1 C), 132.5 (s, 1 C), 130.4 (s, 1 C), 129.2 (s, 2 C), 123.7 (s, 1 C), 121.0 (s, 1 C), 113.7 (s, 2 C), 112.4 (s, 1 C), 54.9 (s, 1 C), 29.6 (s, 1 C)

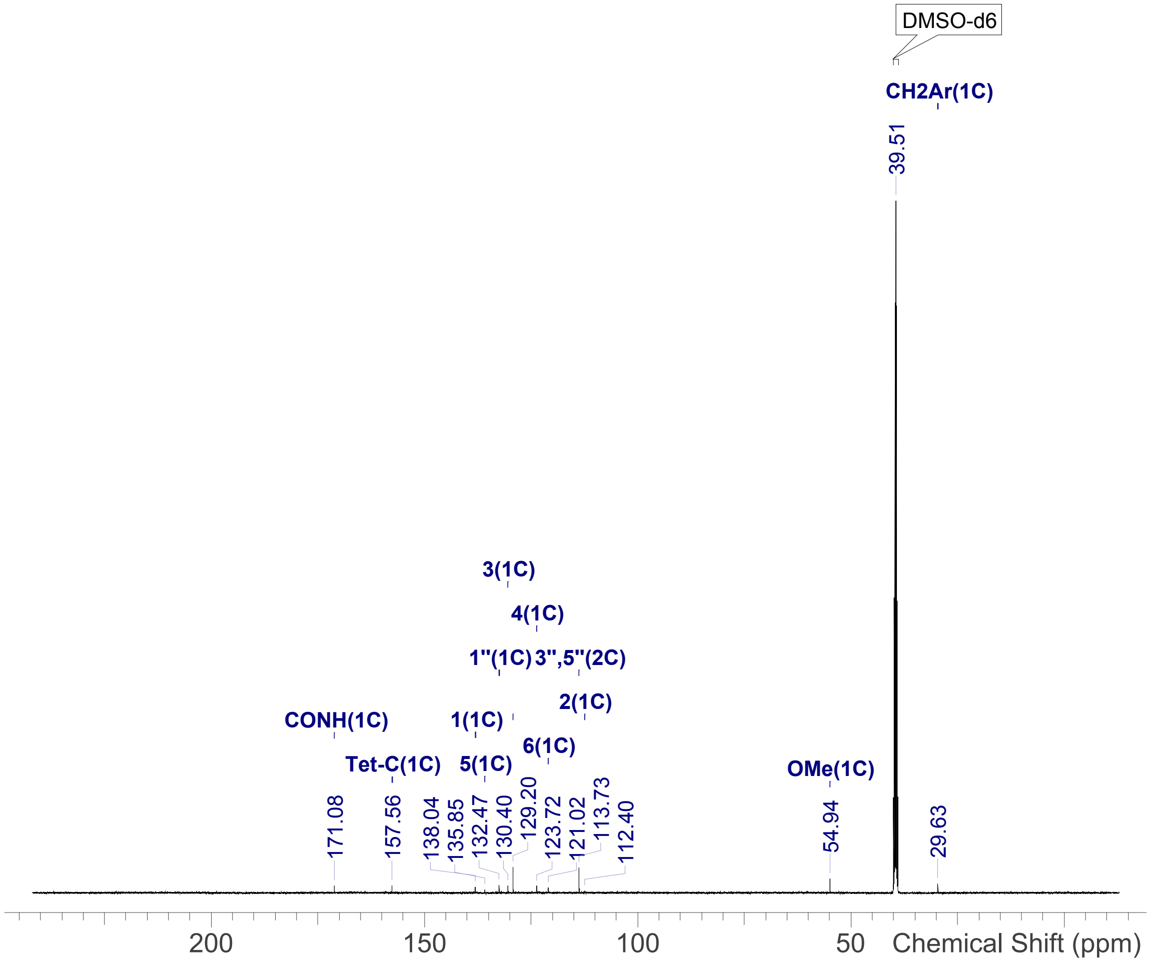

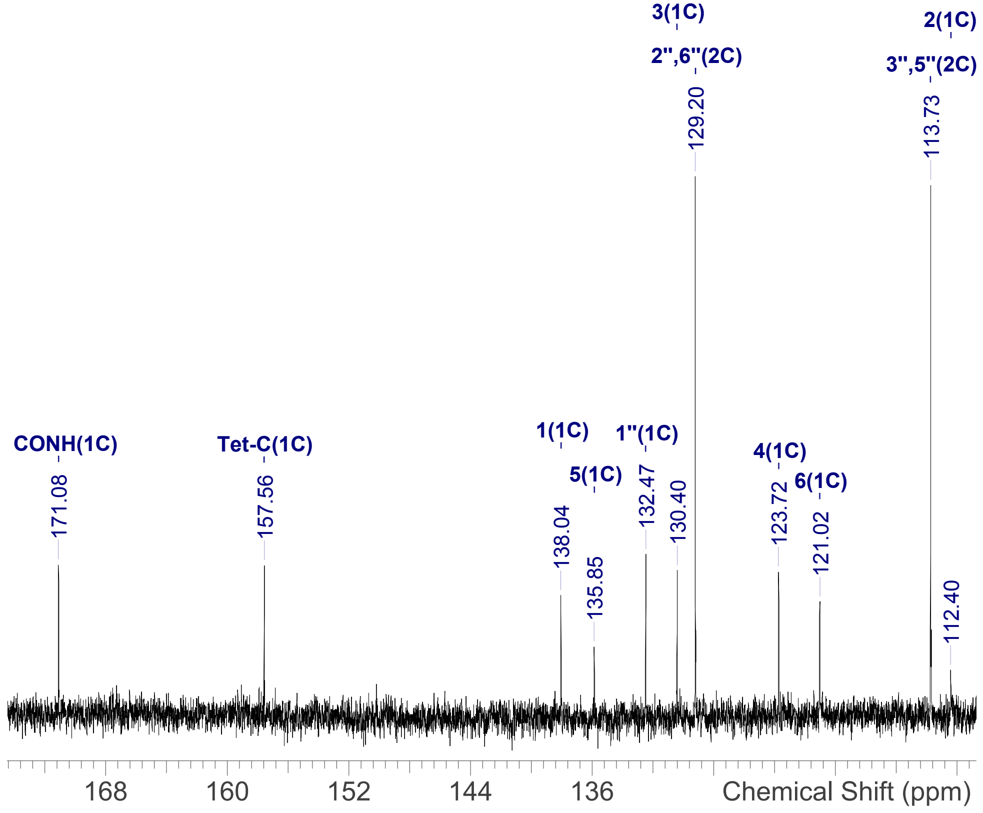

NVR-21_13C.spectrus

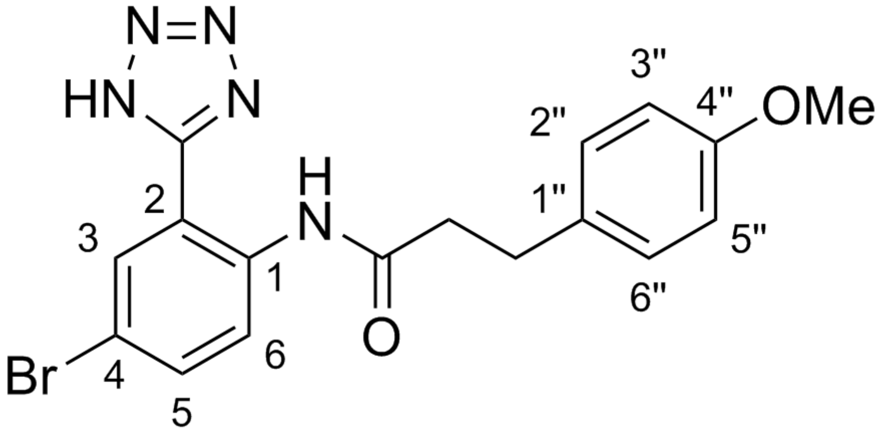

Shift (ppm)	H	m	J (Hz)	Assign
11.01	1	s	-	NH
8.67	1	d	9.0	6
8.56	1	d	2.3	3
8.16	1	dd	8.9, 2.4	5
7.57	2	br d	8.7	6", 2"
7.23	2	br d	8.7	5", 3"
4.11	3	s	-	OMe
3.29	2	t	7.6	CH2Ph
3.10	2	t	7.8	COCH2

Acquisition Time (sec)	6.5536
Date	19/07/2017 10:51:00
Date Stamp	19/07/2017 10:51:00
Frequency (MHz)	500.1930
Nucleus	1H
Number of Transients	128
Solvent	DMSO-d6

^{1}H NMR (500 MHz, *DMSO-d_6*) δ ppm 11.01 (s, 1 H), 8.67 (d, *J*=9.0 Hz, 1 H), 8.56 (d, *J*=2.3 Hz, 1 H), 8.16 (dd, *J*=8.9, 2.4 Hz, 1 H), 7.57 (br d, *J*=8.7 Hz, 2 H), 7.23 (br d, *J*=8.7 Hz, 2 H), 4.11 (s, 3 H), 3.29 (t, *J*=7.6 Hz, 2 H), 3.10 (t, *J*=7.8 Hz, 2 H)

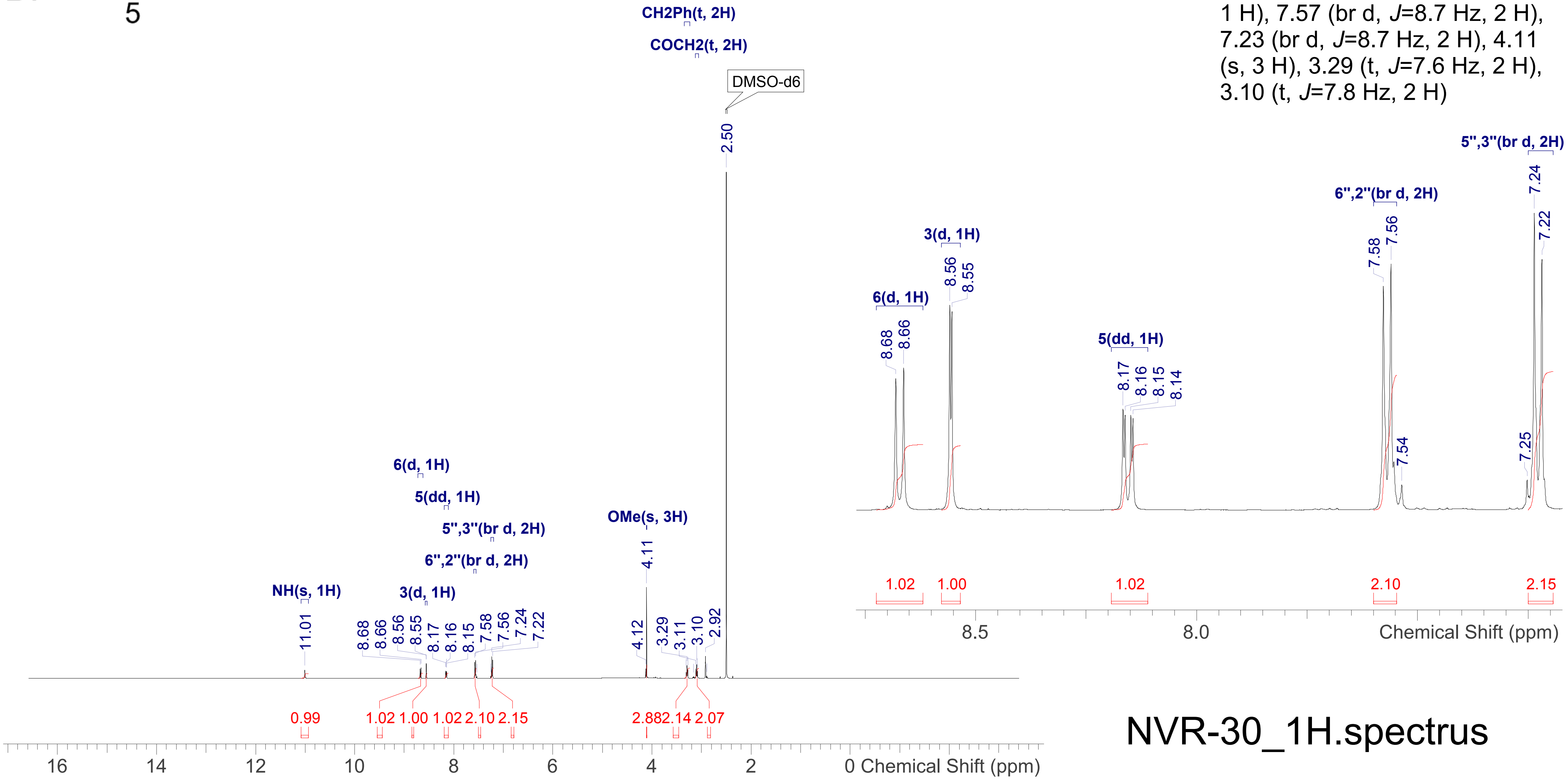

NVR-30_1H.spectrus

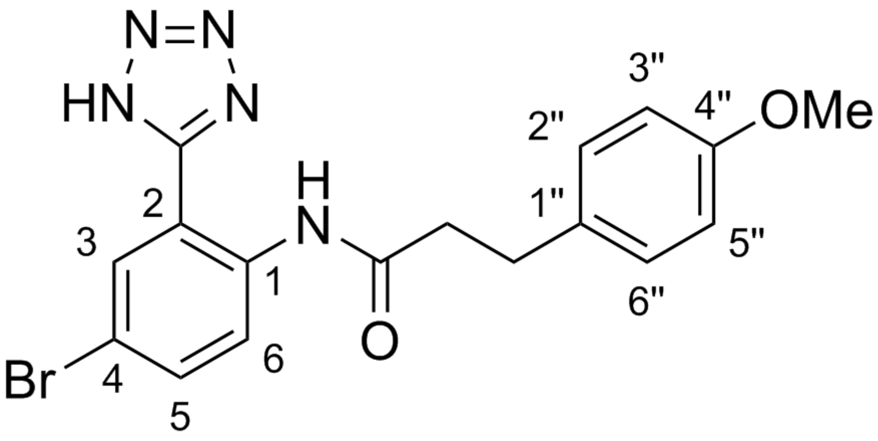

Shift (ppm)	C	m	Assign
170.8	1	s	CONH
157.6	1	s	4"
153.5	1	br s	Tet-C
136.2	1	s	1
134.2	1	s	3
132.5	1	s	1"
131.1	1	s	5
129.2	2	s	2", 6"
124.0	1	s	6
118.0	1	s	4
115.4	1	s	2
113.7	2	s	3", 5"
54.9	1	s	OMe
30.7	1	s	CH2Ar

Acquisition Time (sec)	2.0447
Date	19/07/2017 23:59:00
Date Stamp	19/07/2017 23:59:00
Frequency (MHz)	125.7870
Nucleus	13C
Number of Transients	2048
Solvent	DMSO-d6

^{13}C NMR (126 MHz, *DMSO-d$_6$*) δ ppm 170.8 (s, 1 C), 157.6 (s, 1 C), 153.5 (br s, 1 C), 136.2 (s, 1 C), 134.2 (s, 1 C), 132.5 (s, 1 C), 131.1 (s, 1 C), 129.2 (s, 2 C), 124.0 (s, 1 C), 118.0 (s, 1 C), 115.4 (s, 1 C), 113.7 (s, 2 C), 54.9 (s, 1 C), 30.7 (s, 1 C)

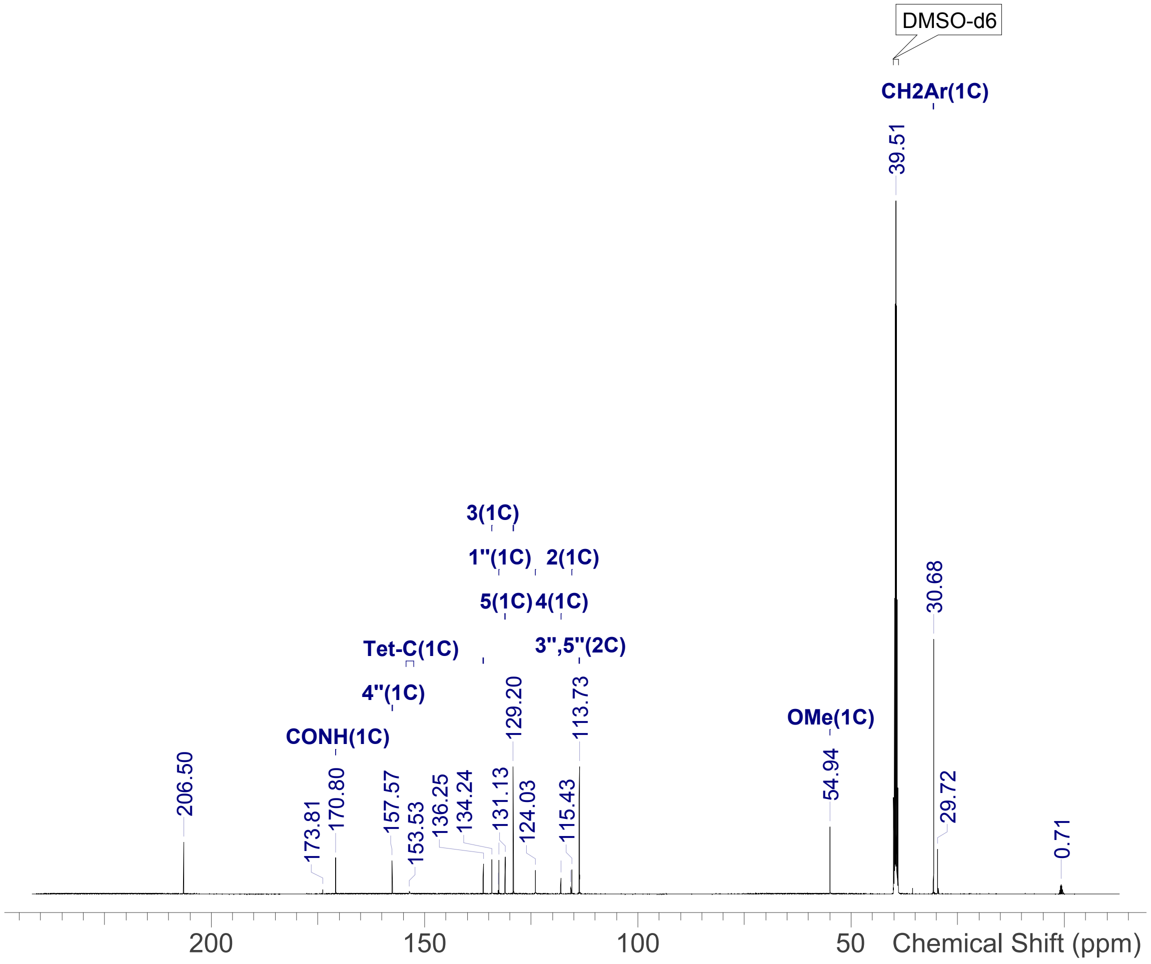

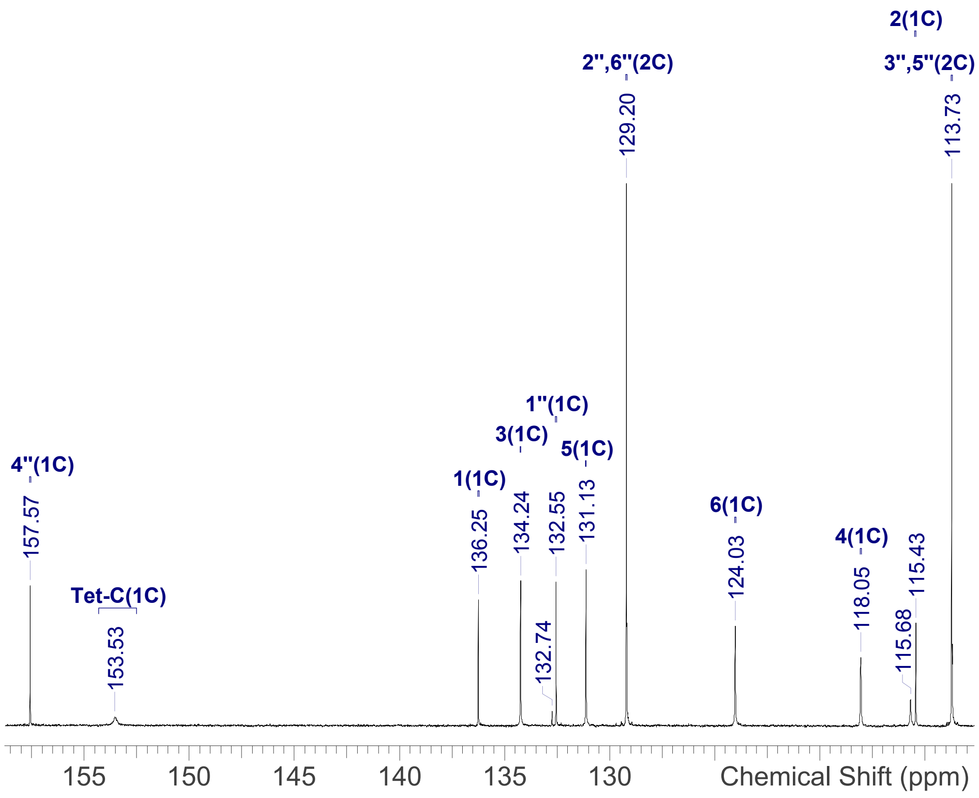

NVR-30_13C.spectrus

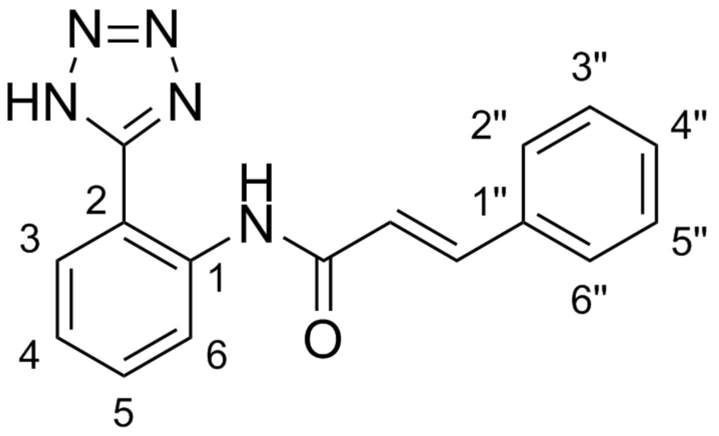

Shift (ppm)	H	m	J (Hz)	Assign
10.92	1	s	-	CONH
8.41	1	d	8.1	6
8.01	1	d	7.8	3
7.70	2	dd	7.9, 1.3	2", 6"
7.64	1	d	15.7	CHPh
7.59	1	ddd	8.4, 7.5, 1.4	5
7.43	3	m	-	4", 3", 5"
7.33	1	ddd	7.9, 7.0, 0.9	4
6.89	1	d	15.7	COCH

Acquisition Time (sec)	6.5536
Date	01/08/2017 16:12:00
Date Stamp	01/08/2017 16:12:00
Frequency (MHz)	500.1930
Nucleus	1H
Number of Transients	16
Solvent	DMSO-d6

^{1}H NMR (500 MHz, *DMSO-d_6*) δ ppm 10.92 (s, 1 H), 8.41 (d, *J*=8.1 Hz, 1 H), 8.01 (d, *J*=7.8 Hz, 1 H), 7.70 (dd, *J*=7.9, 1.3 Hz, 2 H), 7.64 (d, *J*=15.7 Hz, 1 H), 7.59 (ddd, *J*=8.4, 7.5, 1.4 Hz, 1 H), 7.39 - 7.48 (m, 3 H), 7.33 (ddd, *J*=7.9, 7.0, 0.9 Hz, 1 H), 6.89 (d, *J*=15.7 Hz, 1 H)

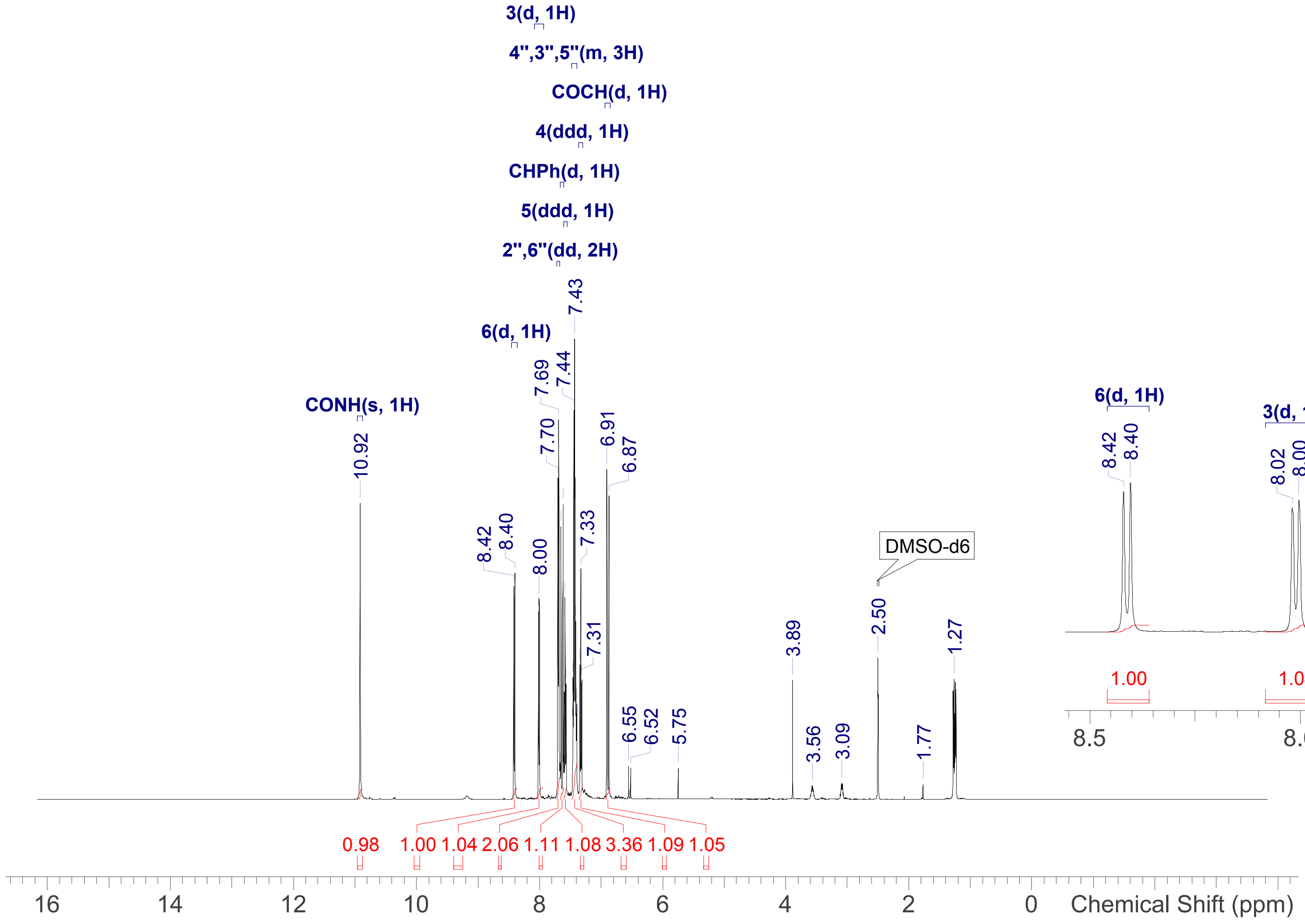

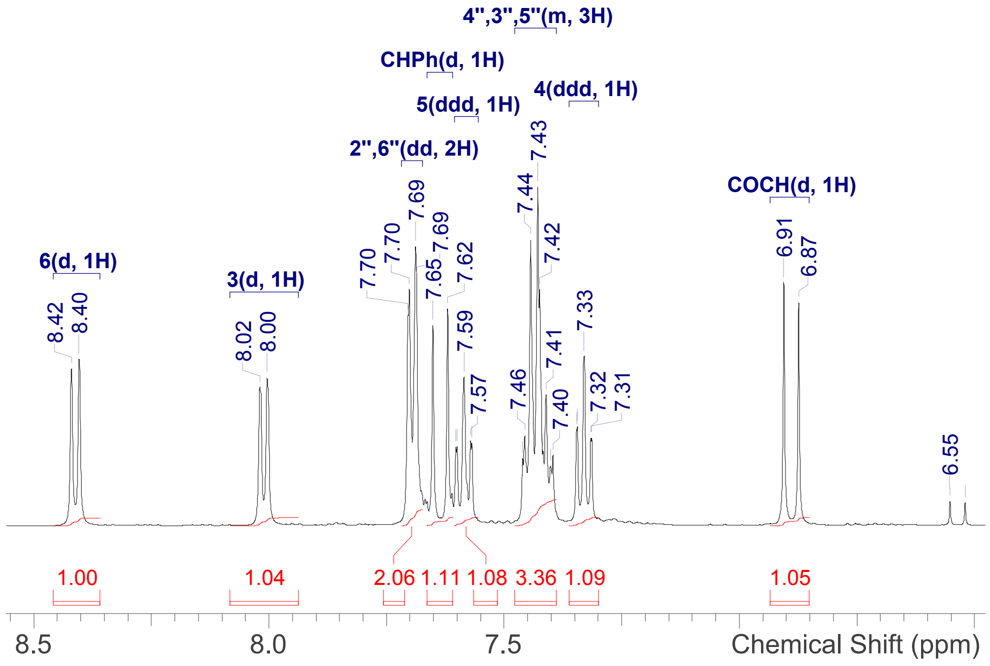

NVR-25_1H.spectrus

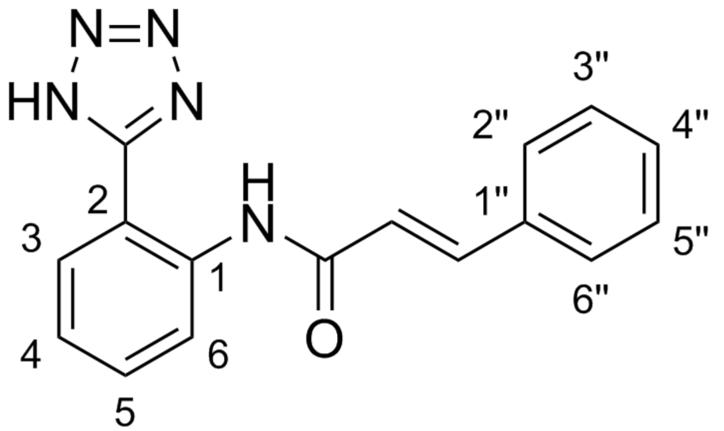

Shift (ppm)	C	m	Assign
163.9	1	s	CONH
154.5	1	s	Tet-C
141.0	1	s	CHPh
137.0	1	s	1
134.4	1	s	1''
131.5	1	s	5
130.0	1	s	4''
129.1	1	s	3
129.0	2	s	5'', 3''
128.0	2	s	6'', 2''
124.2	1	s	4
122.5	1	s	CHCO
122.2	1	s	6
114.3	1	s	2

Acquisition Time (sec)	2.0447
Date	02/08/2017 07:14:00
Date Stamp	02/08/2017 07:14:00
Frequency (MHz)	125.7870
Nucleus	13C
Number of Transients	256
Solvent	DMSO-d6

^{13}C NMR (126 MHz, *DMSO-d_6*) δ ppm 163.9 (s, 1 C), 154.5 (s, 1 C), 141.0 (s, 1 C), 137.0 (s, 1 C), 134.4 (s, 1 C), 131.5 (s, 1 C), 130.0 (s, 1 C), 129.1 (s, 1 C), 129.0 (s, 2 C), 128.0 (s, 2 C), 124.2 (s, 1 C), 122.5 (s, 1 C), 122.2 (s, 1 C), 114.3 (s, 1 C)

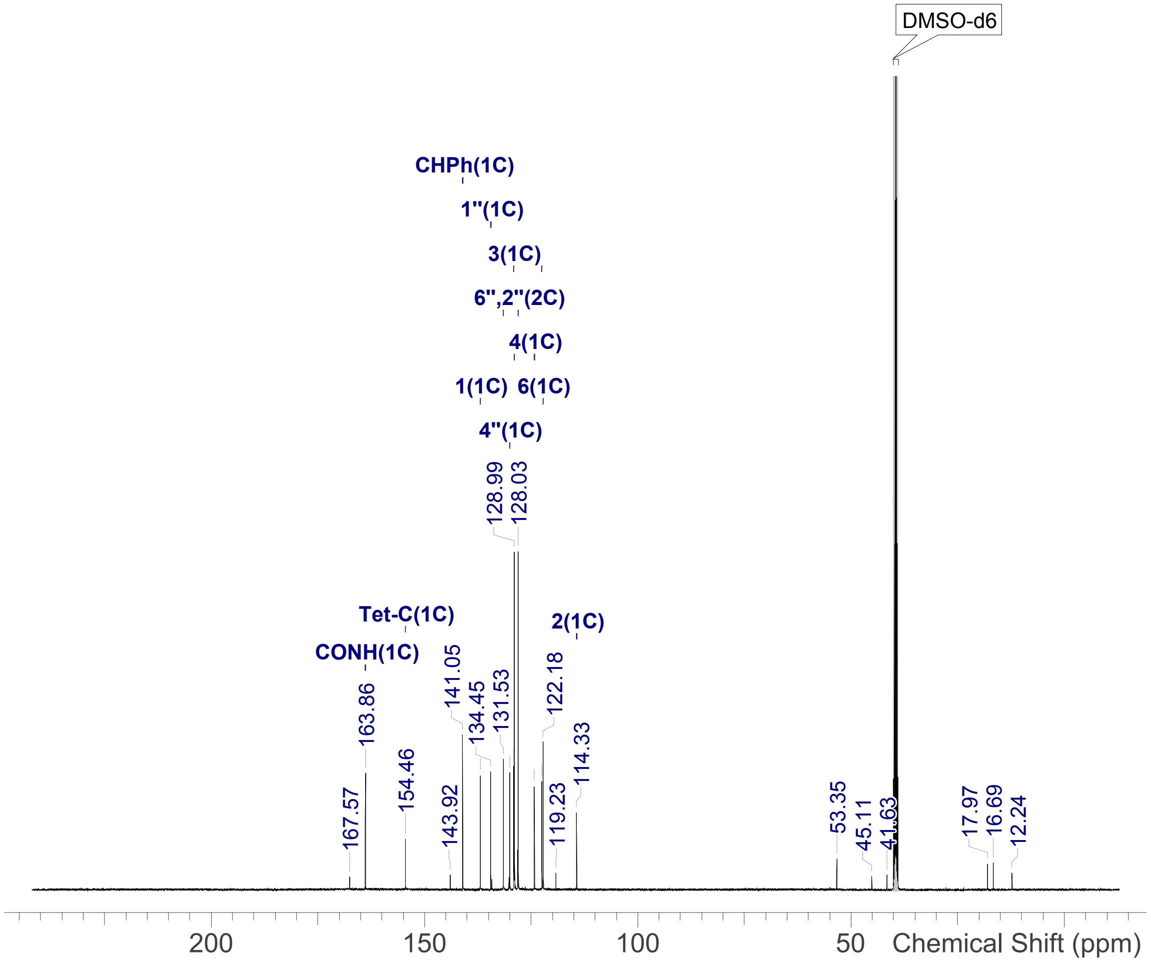

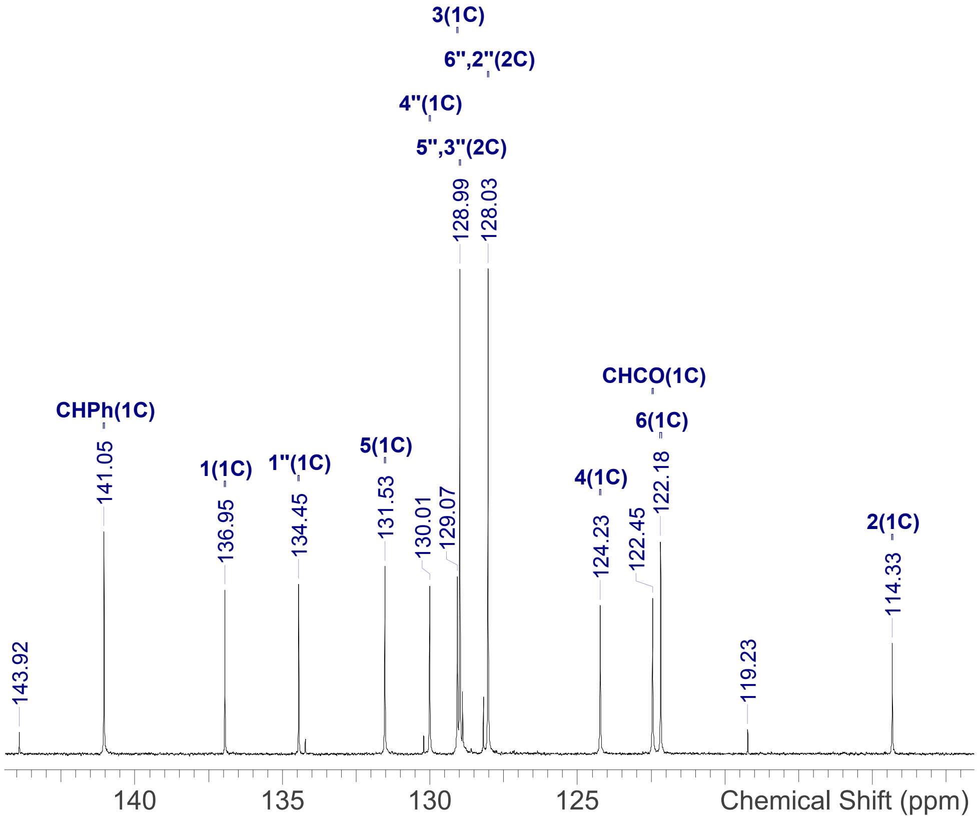

NVR-25_13C.spectrus

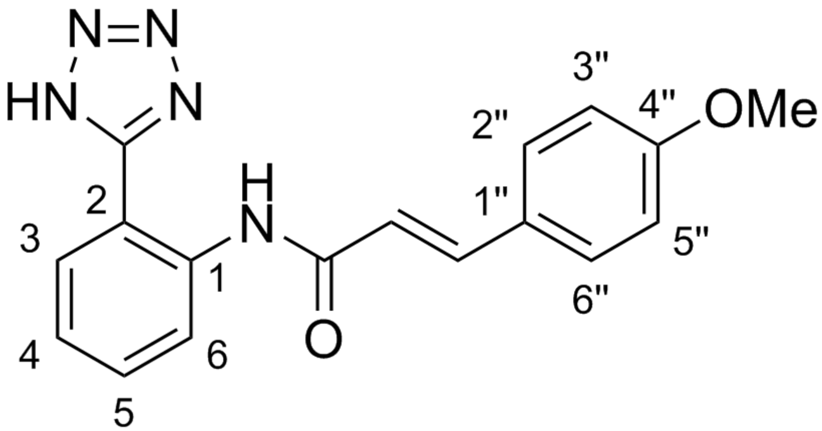

Shift (ppm)	H	m	J (Hz)	Assign
10.90	1	br s	-	CONH
8.42	1	br d	8.3	H6
7.96	1	br d	7.3	H3
7.66	2	br d	8.4	H2'', H6''
7.58	2	m	-	H5, CHAr
7.31	1	br t	7.4	H4
7.01	2	br d	8.4	H3'', H5''
6.72	1	br d	15.6	COCH
3.81	3	s	-	OMe

Acquisition Time (sec)	8.1920
Date	20/12/2017 15:33:00
Date Stamp	20/12/2017 15:33:00
Frequency (MHz)	400.1320
Nucleus	1H
Number of Transients	16
Solvent	DMSO-d6

^{1}H NMR (400 MHz, *DMSO-d$_6$*) δ ppm 10.90 (br s, 1 H), 8.42 (br d, *J*=8.3 Hz, 1 H), 7.96 (br d, *J*=7.3 Hz, 1 H), 7.66 (br d, *J*=8.4 Hz, 2 H), 7.50 - 7.63 (m, 2 H), 7.31 (br t, *J*=7.4 Hz, 1 H), 7.01 (br d, *J*=8.4 Hz, 2 H), 6.72 (br d, *J*=15.6 Hz, 1 H), 3.81 (s, 3 H)

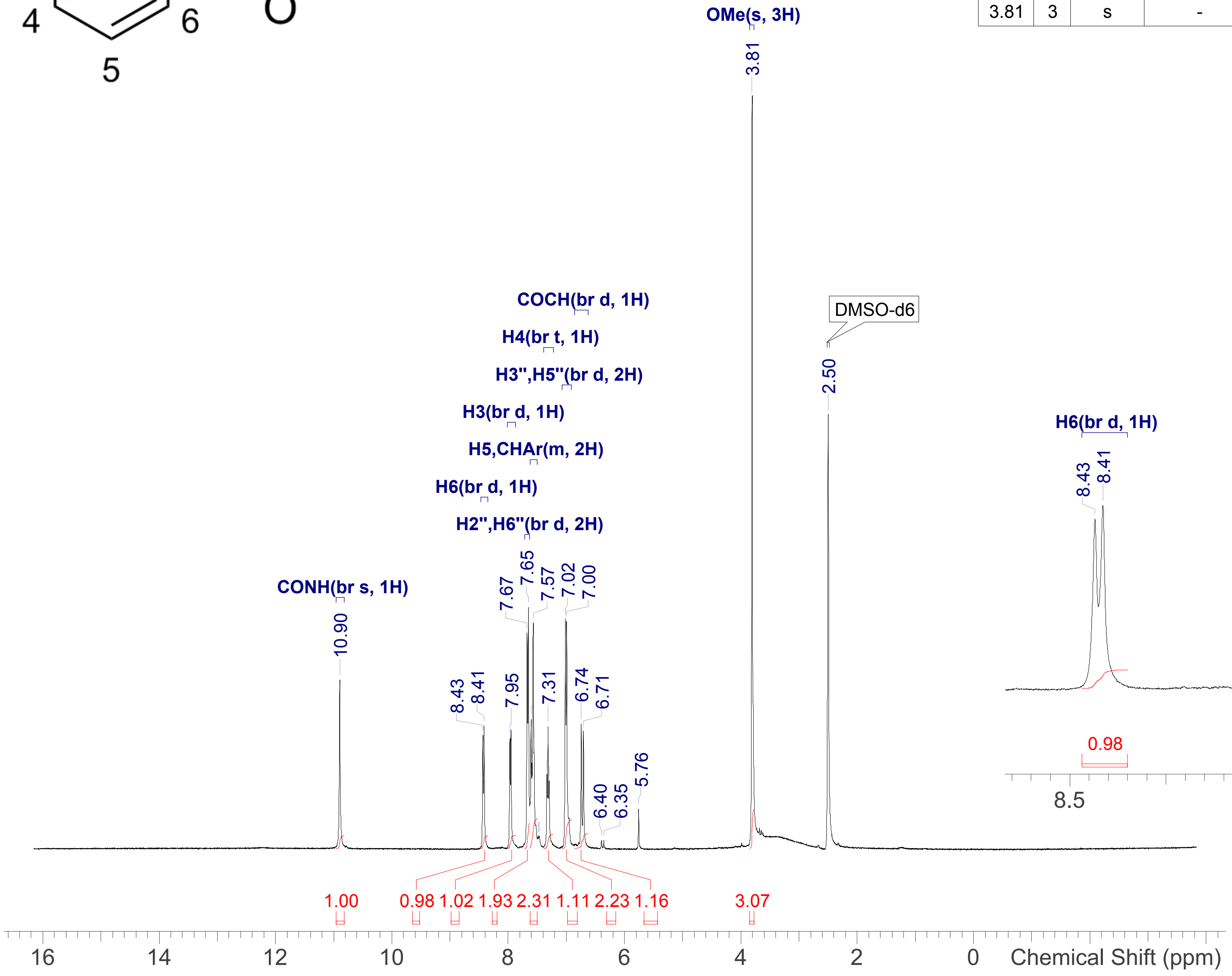

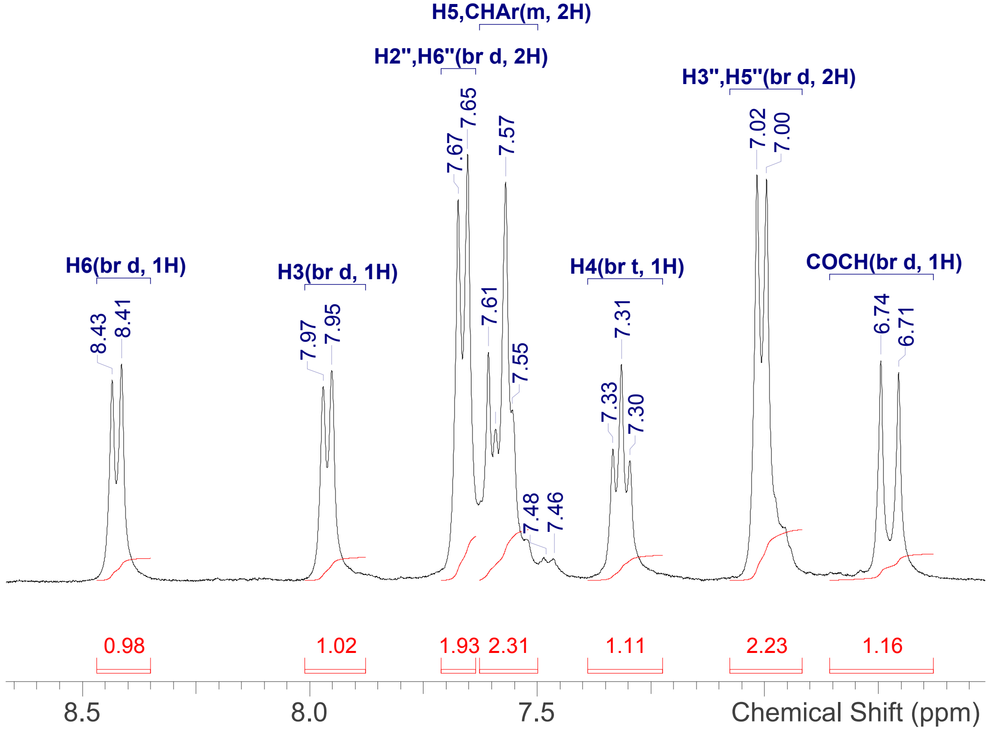

NVR-38_1H.spectrus

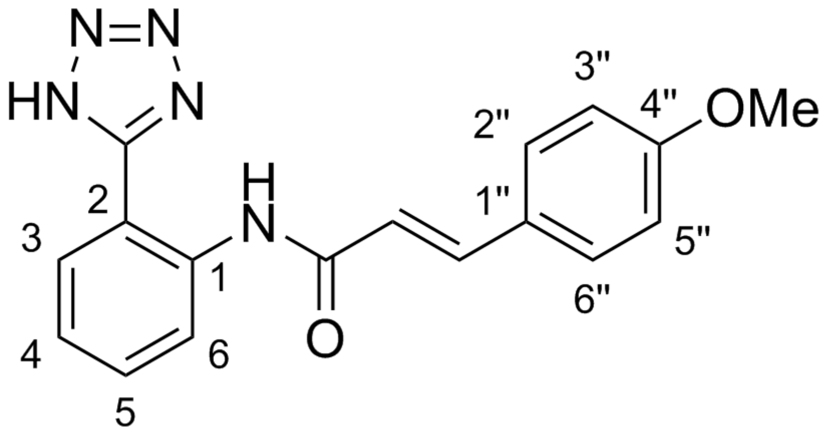

Shift (ppm)	C	m	Assign
164.1	1	s	CONH
160.8	1	s	4''
140.9	1	s	CHAr
137.1	1	s	1
131.3	1	s	5
129.7	2	s	2'', 6''
128.9	1	s	3
127.0	1	s	1''
124.0	1	s	4
122.2	1	s	CHCO
119.5	1	s	6
114.4	2	s	3'', 5''
55.3	1	s	OMe

Acquisition Time (sec)	2.0447
Date	20/12/2017 21:32:00
Date Stamp	20/12/2017 21:32:00
Frequency (MHz)	100.6230
Nucleus	13C
Number of Transients	256
Solvent	DMSO-d6

^{13}C NMR (101 MHz, *DMSO-d_6*) δ ppm 164.1 (s, 1 C), 160.8 (s, 1 C), 140.9 (s, 1 C), 137.1 (s, 1 C), 131.3 (s, 1 C), 129.7 (s, 2 C), 128.9 (s, 1 C), 127.0 (s, 1 C), 124.0 (s, 1 C), 122.2 (s, 1 C), 119.5 (s, 1 C), 114.4 (s, 2 C), 55.3 (s, 1 C)

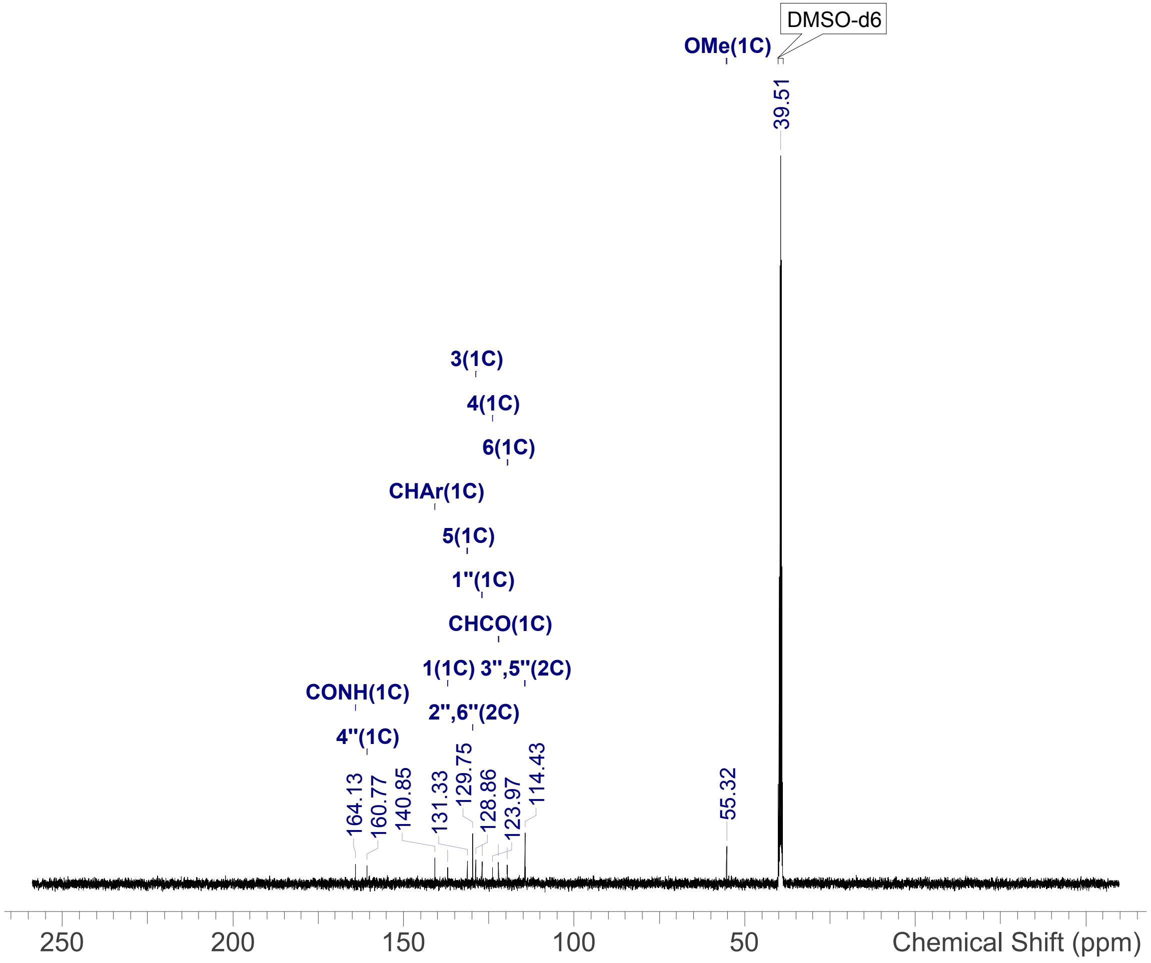

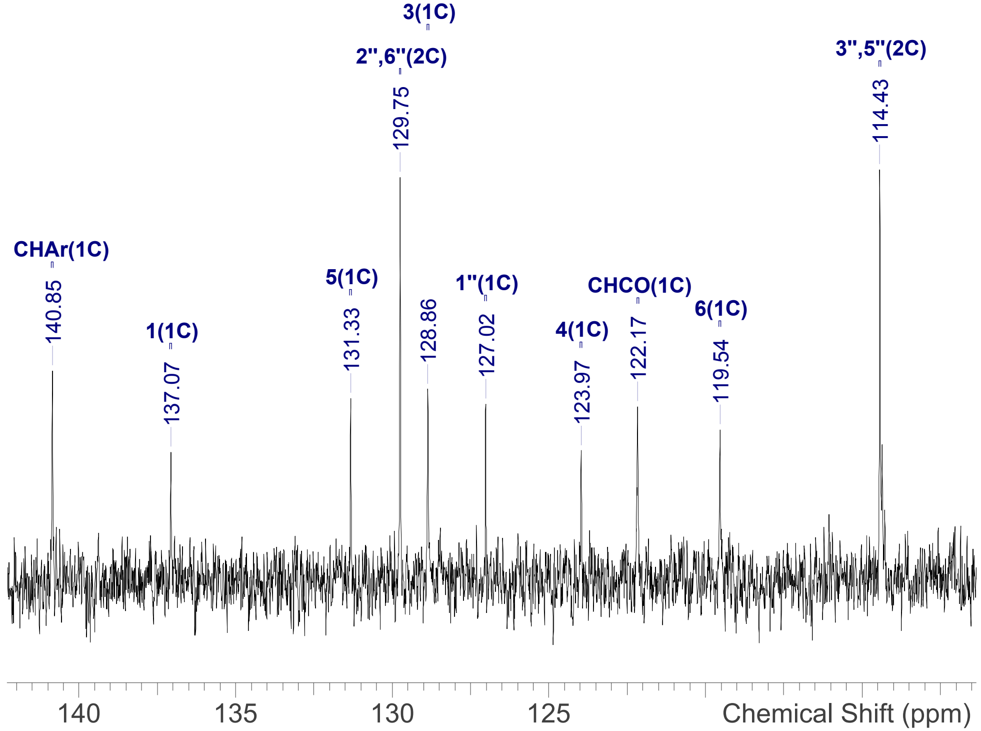

NVR-38_13C.spectrus

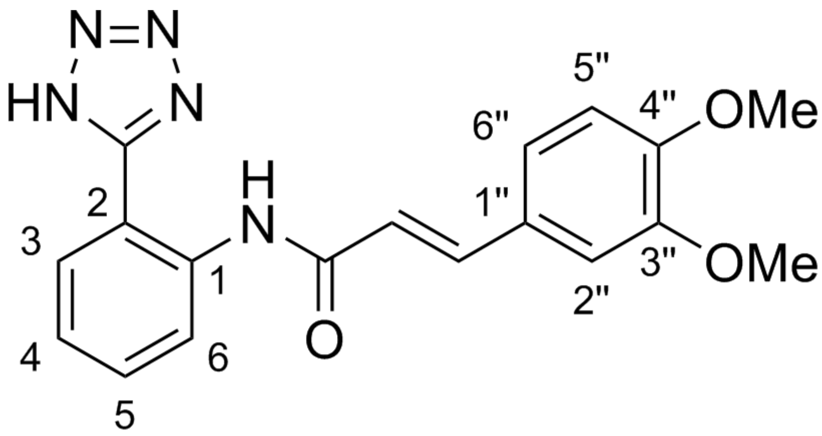

Shift (ppm)	H	m	J (Hz)	Assign
10.76	1	s	-	CONH
8.41	1	d	8.2	6
8.03	1	dd	7.9, 0.9	3
7.58	2	m	-	5, CHAr
7.33	2	m	-	2", 4
7.25	1	br d	8.3	6"
7.01	1	d	8.3	5"
6.79	1	d	15.6	COCH
3.84	3	s	-	OMe
3.80	3	s	-	OMe

Acquisition Time (sec)	10.6168
Date	27/05/2017 13:40:00
Date Stamp	27/05/2017 13:40:00
Frequency (MHz)	300.1310
Nucleus	1H
Number of Transients	16
Solvent	DMSO-d6

^{1}H NMR (300 MHz, *DMSO-d_6*) δ ppm 10.76 (s, 1 H), 8.41 (d, *J*=8.2 Hz, 1 H), 8.03 (dd, *J*=7.9, 0.9 Hz, 1 H), 7.52 - 7.64 (m, 2 H), 7.29 - 7.38 (m, 2 H), 7.25 (br d, *J*=8.3 Hz, 1 H), 7.01 (d, *J*=8.3 Hz, 1 H), 6.79 (d, *J*=15.6 Hz, 1 H), 3.84 (s, 3 H), 3.80 (s, 3 H)

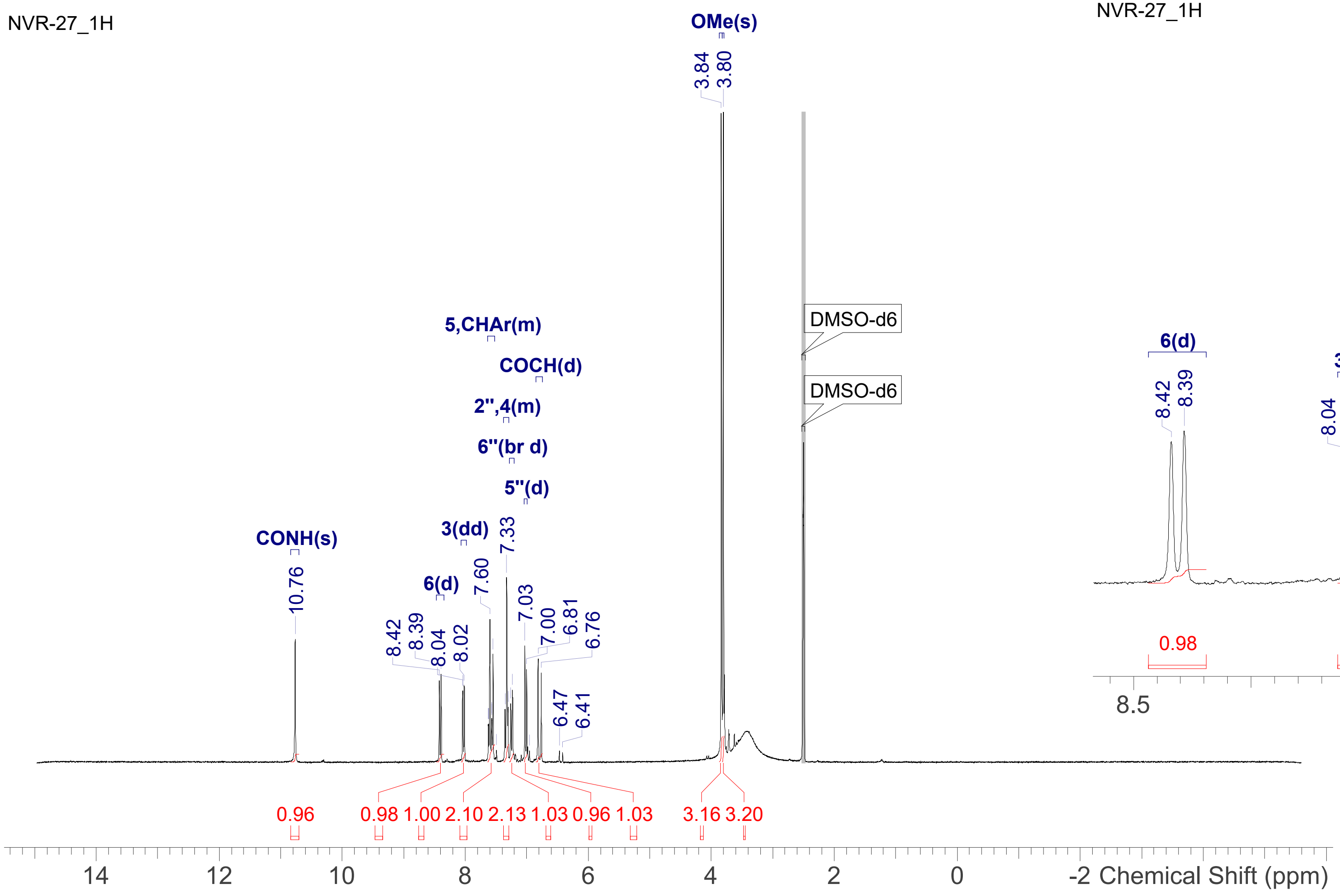

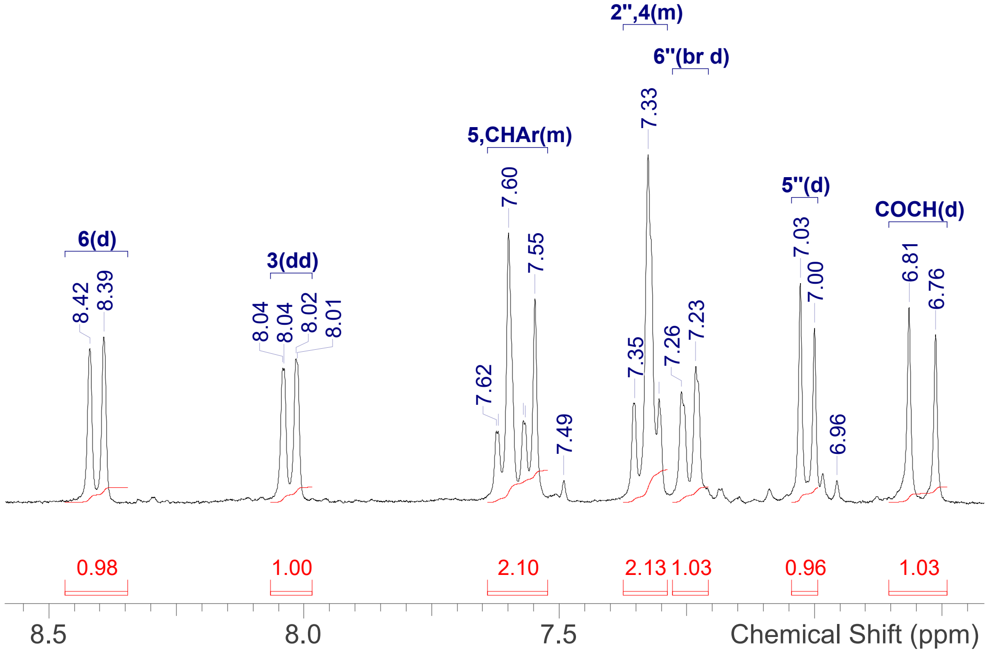

NVR-27_1H.spectrus

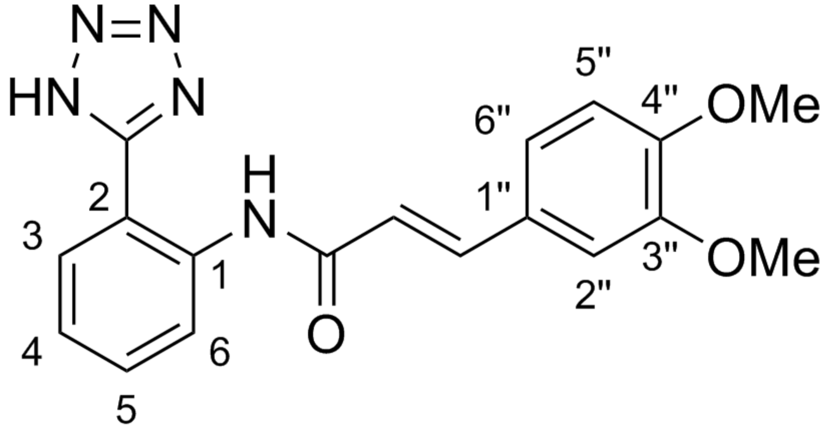

NVR-27_13C

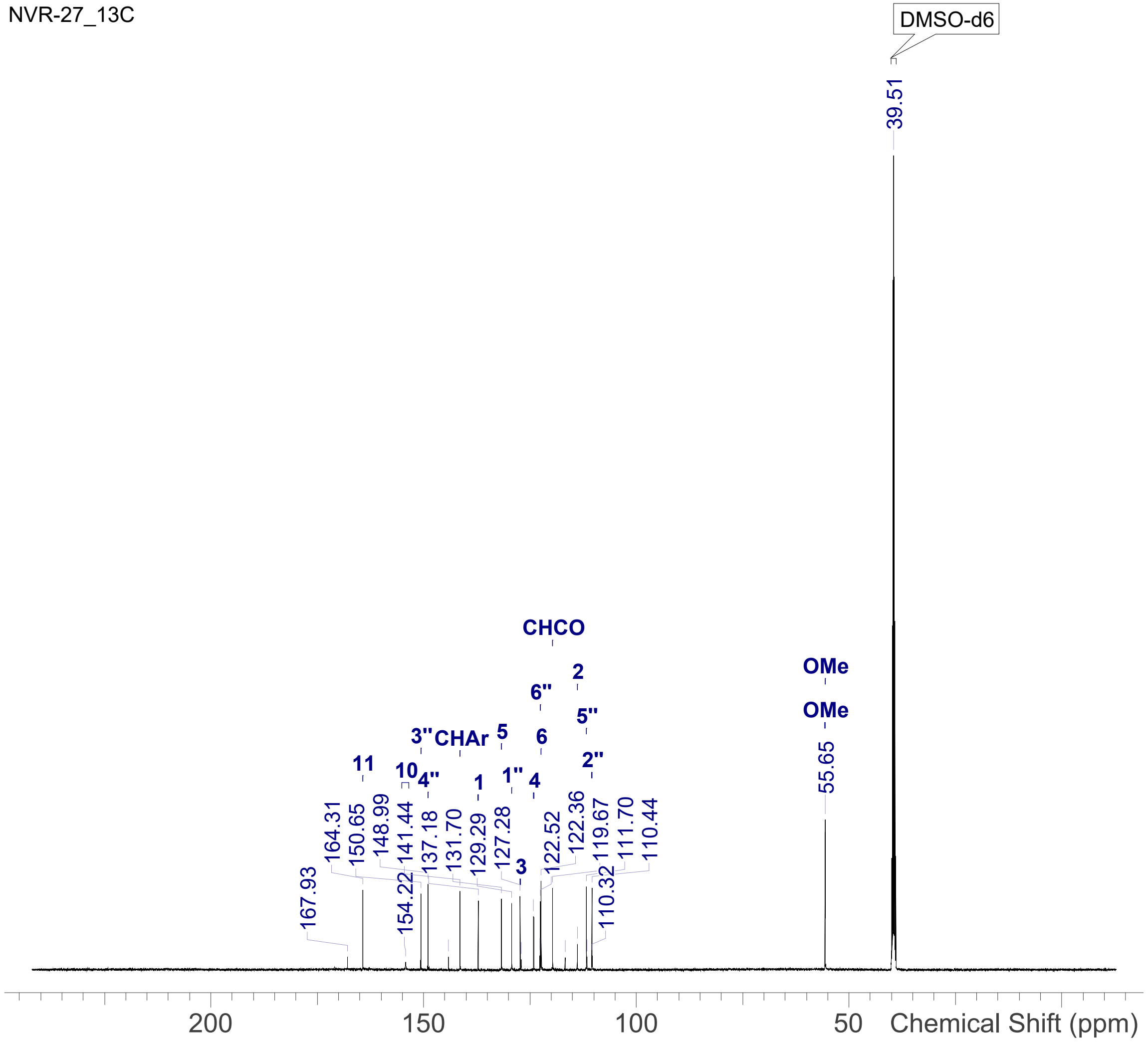

Shift (ppm)	C	m	Assign
164.3	1	s	11
154.2	1	br s	10
150.7	1	s	3"
149.0	1	s	4"
141.4	1	s	CHAr
137.2	1	s	1
131.7	1	s	5
129.3	1	s	1"
127.3	1	s	3
124.1	1	s	4
122.5	1	s	6"
122.4	1	s	6
119.7	1	s	CHCO
113.9	1	s	2
111.7	1	s	5"
110.4	1	s	2"
55.6	1	s	OMe
55.6	1	s	OMe

Acquisition Time (sec)	2.0447
Date	02/08/2017 03:05:00
Date Stamp	02/08/2017 03:05:00
Frequency (MHz)	125.7870
Nucleus	13C
Number of Transients	256
Solvent	DMSO-d6

^{13}C NMR (126 MHz, *DMSO-d_6*) δ ppm 164.3 (s, 1 C), 154.2 (br s, 1 C), 150.7 (s, 1 C), 149.0 (s, 1 C), 141.4 (s, 1 C), 137.2 (s, 1 C), 131.7 (s, 1 C), 129.3 (s, 1 C), 127.3 (s, 1 C), 124.1 (s, 1 C), 122.5 (s, 1 C), 122.4 (s, 1 C), 119.7 (s, 1 C), 113.9 (s, 1 C), 111.7 (s, 1 C), 110.4 (s, 1 C), 55.6 (s, 1 C), 55.6 (s, 1 C)

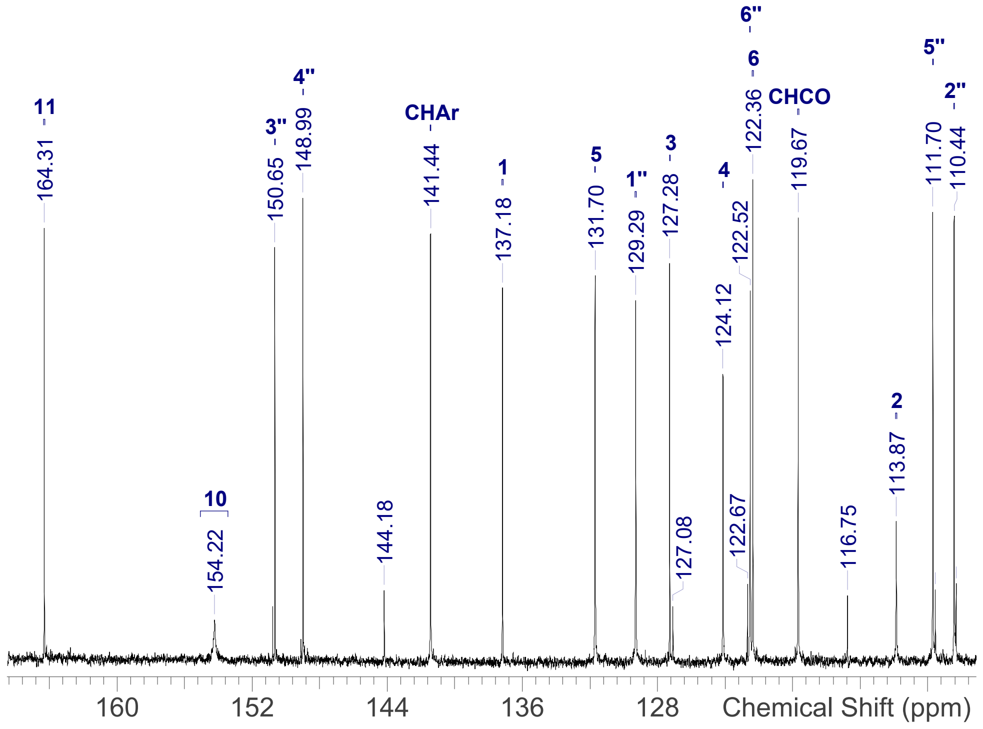

NVR-27_13C.spectrus

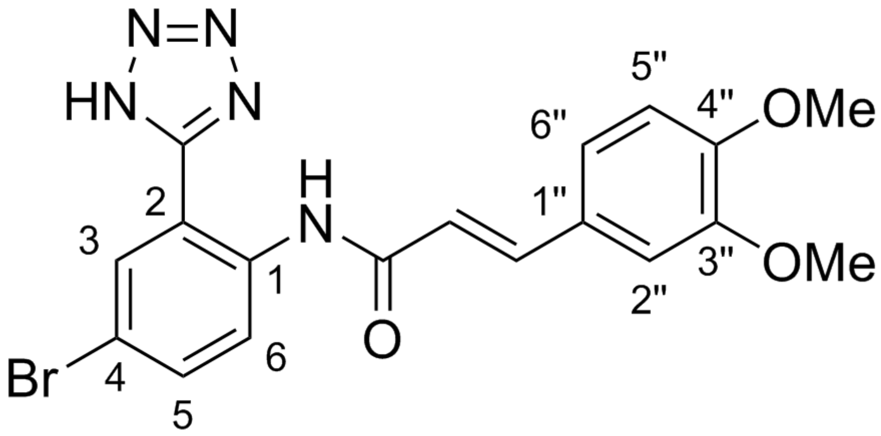

Shift (ppm)	H	m	J (Hz)	Assign
10.81	1	s	-	CONH
8.38	1	d	8.9	6
8.17	1	s	-	3
7.76	1	br d	9.0	5
7.58	1	d	15.6	CHAr
7.31	1	s	-	2"
7.24	1	br d	8.4	6"
7.01	1	d	8.4	5"
6.76	1	d	15.6	COCH
3.83	3	s	-	OMe
3.80	3	s	-	OMe

Acquisition Time (sec)	6.5536
Date	16/06/2017 03:58:00
Date Stamp	16/06/2017 03:58:00
Frequency (MHz)	500.1930
Nucleus	1H
Number of Transients	128
Solvent	DMSO-d6

^{1}H NMR (500 MHz, *DMSO-d_6*) δ ppm 10.81 (s, 1 H), 8.38 (d, *J*=8.9 Hz, 1 H), 8.17 (s, 1 H), 7.76 (br d, *J*=9.0 Hz, 1 H), 7.58 (d, *J*=15.6 Hz, 1 H), 7.31 (s, 1 H), 7.24 (br d, *J*=8.4 Hz, 1 H), 7.01 (d, *J*=8.4 Hz, 1 H), 6.76 (d, *J*=15.6 Hz, 1 H), 3.83 (s, 3 H), 3.80 (s, 3 H)

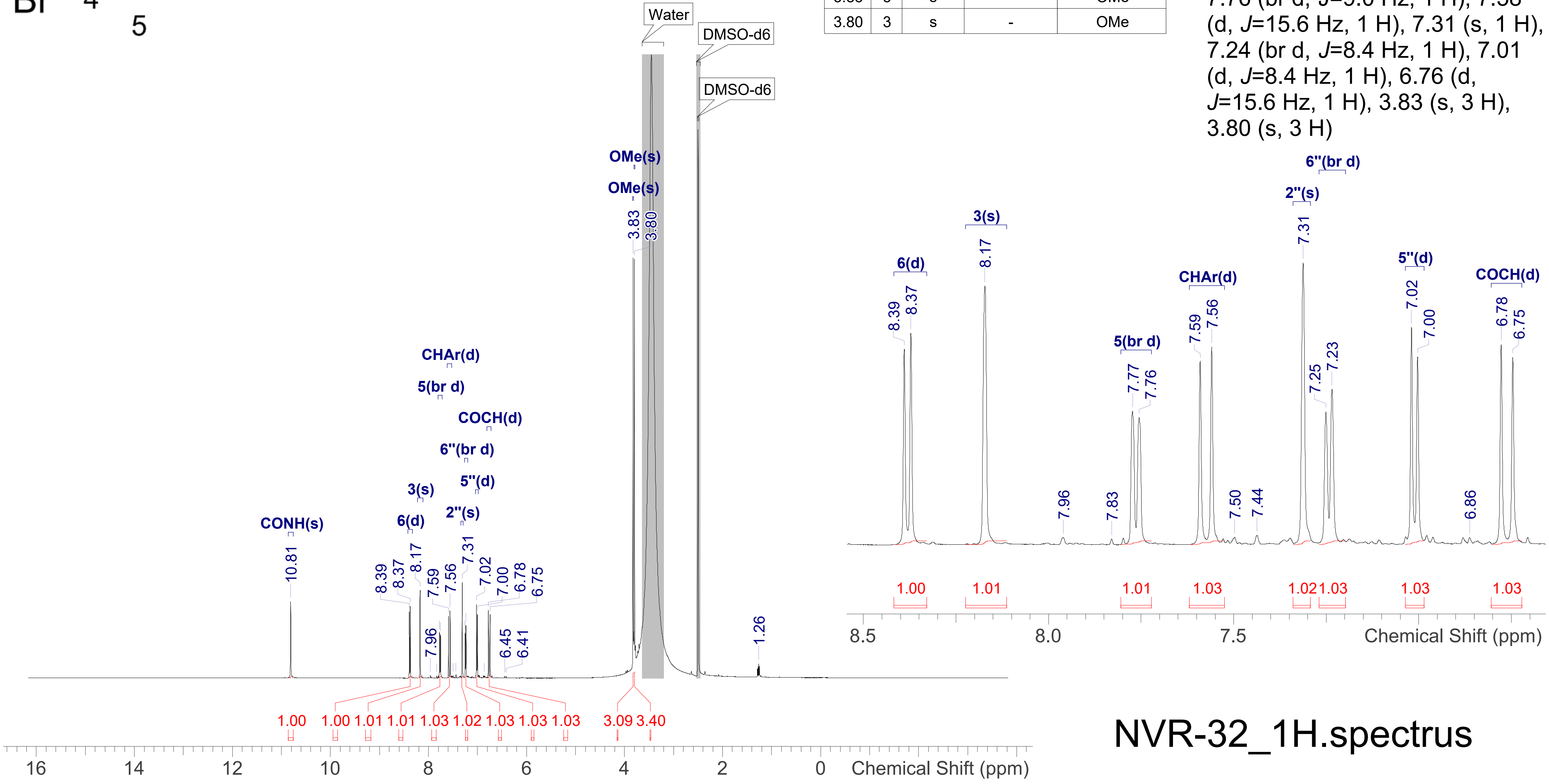

NVR-32_1H.spectrus

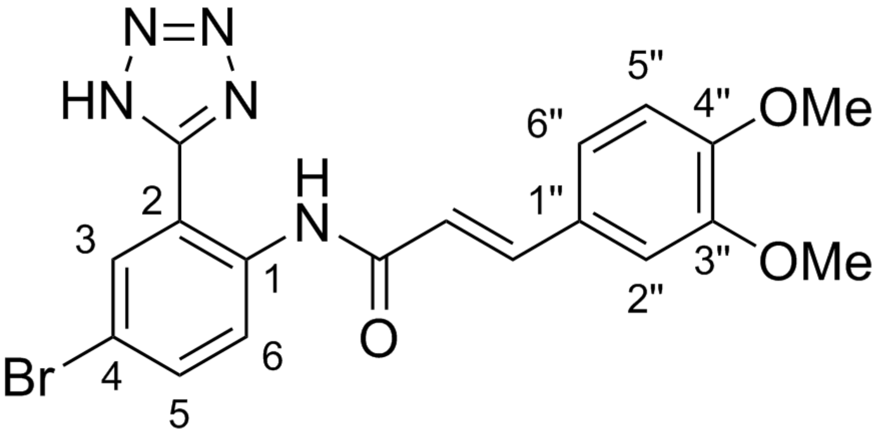

Shift (ppm)	C	m	Assign
164.4	1	s	CONH
153.8	1	s	Tet-C
150.7	1	s	3"
149.0	1	s	4"
141.8	1	s	CHAr
136.5	1	s	1
134.1	1	s	3
131.3	1	s	5
127.2	1	s	1"
124.2	1	s	6
122.5	1	s	6"
119.4	1	s	COCH
116.0	1	s	2
115.6	1	s	4
111.7	1	s	5"
110.5	1	s	2"
55.7	1	s	OMe
55.6	1	s	OMe

Acquisition Time (sec)	2.0447
Date	25/07/2017 03:58:00
Date Stamp	25/07/2017 03:58:00
Frequency (MHz)	125.7870
Nucleus	13C
Number of Transients	2048
Solvent	DMSO-d6

^{13}C NMR (126 MHz, *DMSO-d_6*) δ ppm 164.4 (s, 1 C), 153.8 (s, 1 C), 150.7 (s, 1 C), 149.0 (s, 1 C), 141.8 (s, 1 C), 136.5 (s, 1 C), 134.1 (s, 1 C), 131.3 (s, 1 C), 127.2 (s, 1 C), 124.2 (s, 1 C), 122.5 (s, 1 C), 119.4 (s, 1 C), 116.0 (s, 1 C), 115.6 (s, 1 C), 111.7 (s, 1 C), 110.5 (s, 1 C), 55.7 (s, 1 C), 55.6 (s, 1 C)

NVR-32_13C

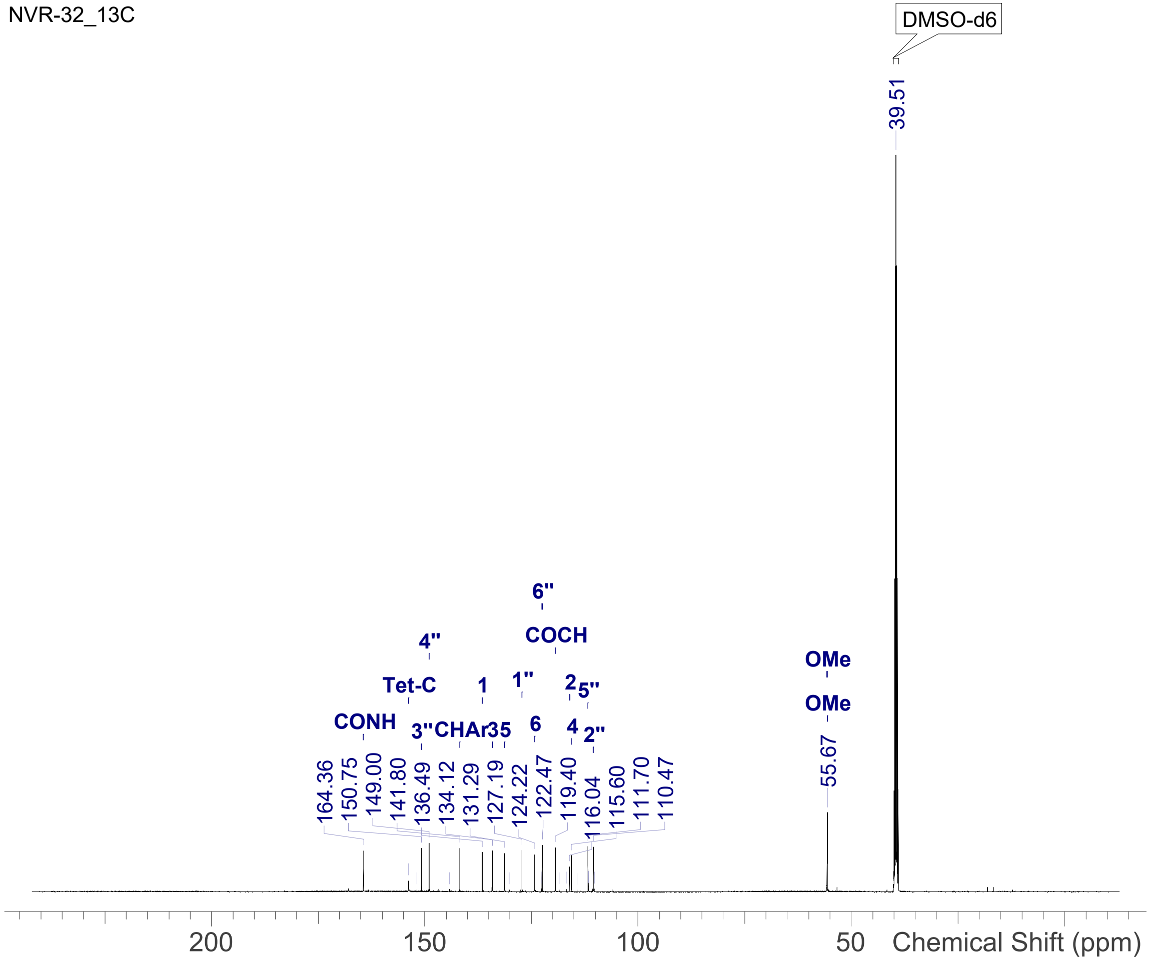

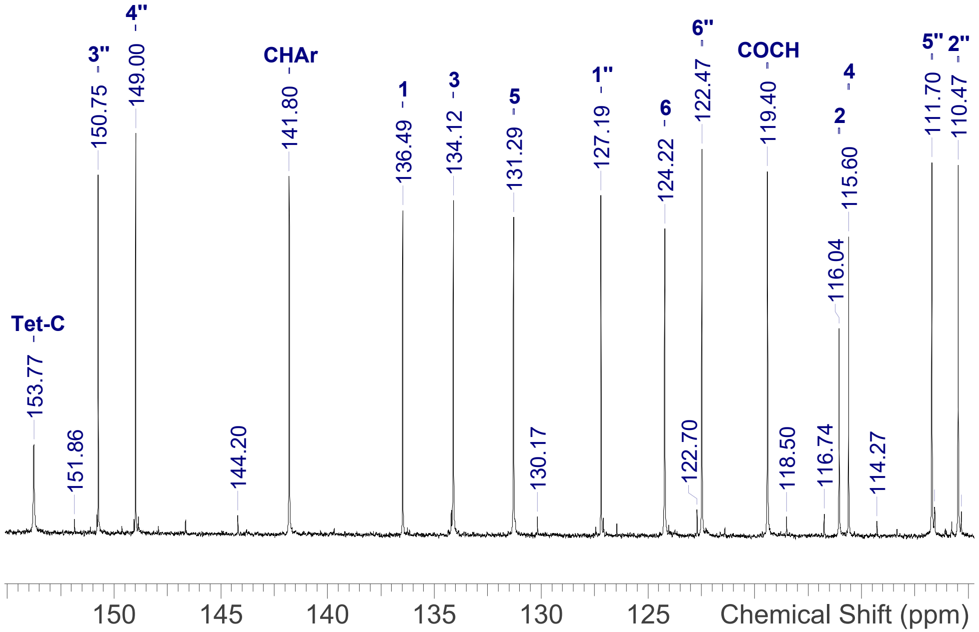

NVR-32_13C.spectrus

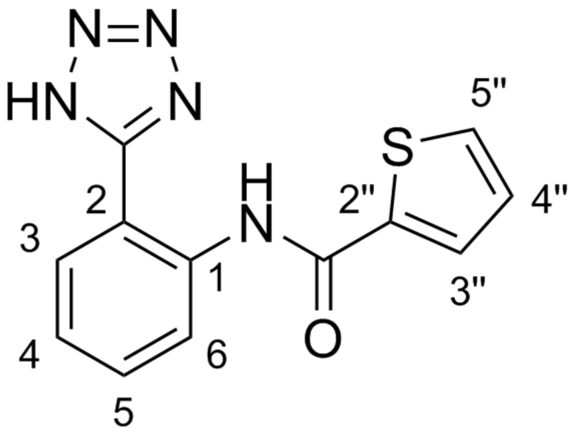

Shift (ppm)	H	m	J (Hz)	Assign
11.53	1	s	-	CONH
8.47	1	d	8.1	H6
8.01	1	dd	7.9, 1.2	H3
7.95	2	m	-	H3'', H5''
7.62	1	ddd	8.2, 7.4, 1.2	H5
7.36	1	ddd	7.9, 7.2, 0.9	H4
7.31	1	dd	4.9, 3.8	H4''

Acquisition Time (sec)	6.5536
Date	03/08/2017 16:31:00
Date Stamp	03/08/2017 16:31:00
Frequency (MHz)	500.1930
Nucleus	1H
Number of Transients	16
Solvent	DMSO-d6

^{1}H NMR (500 MHz, *DMSO-d_6*) δ ppm 11.53 (s, 1 H), 8.47 (d, *J*=8.1 Hz, 1 H), 8.01 (dd, *J*=7.9, 1.2 Hz, 1 H), 7.92 - 7.97 (m, 2 H), 7.62 (ddd, *J*=8.2, 7.4, 1.2 Hz, 1 H), 7.36 (ddd, *J*=7.9, 7.2, 0.9 Hz, 1 H), 7.31 (dd, *J*=4.9, 3.8 Hz, 1 H)

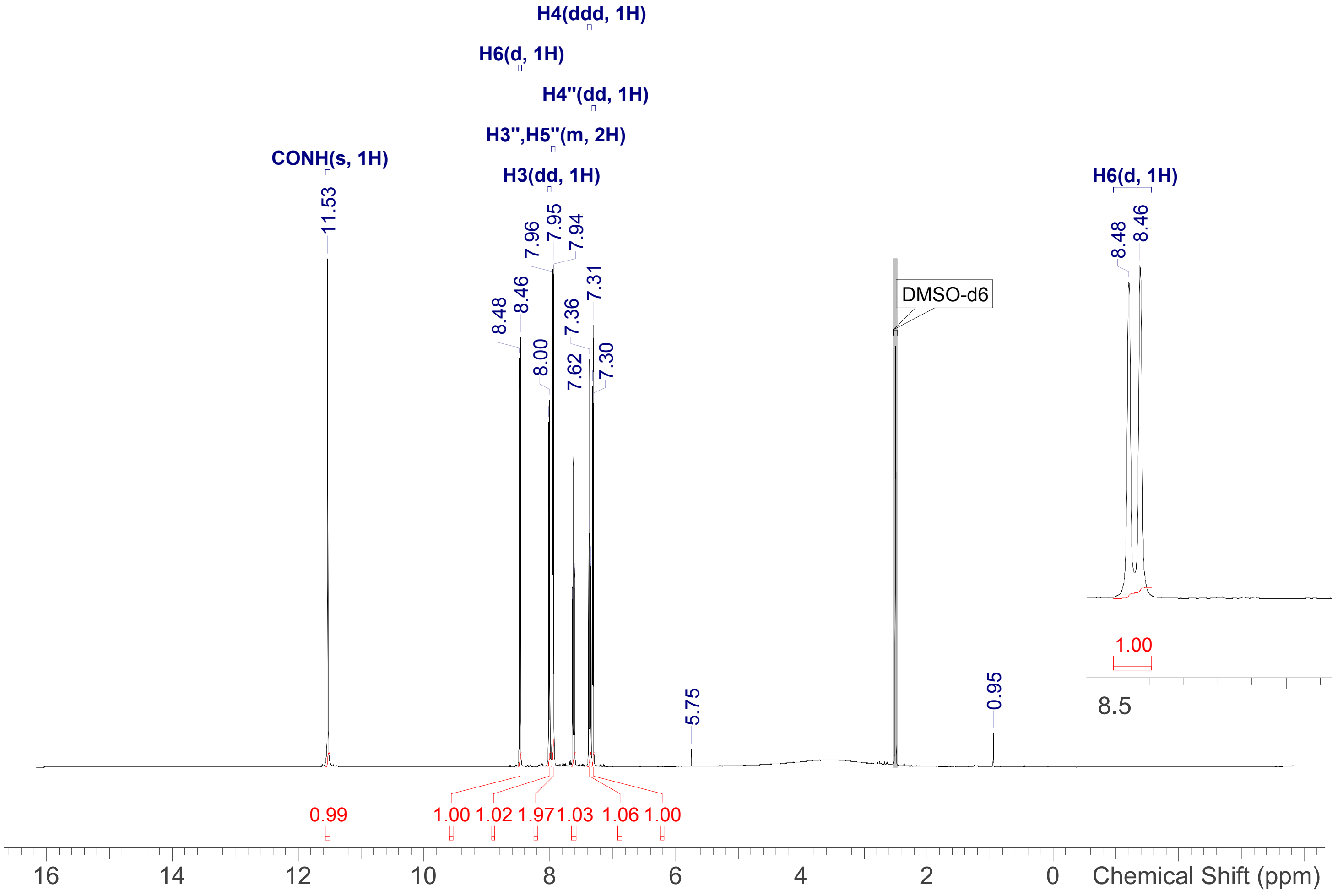

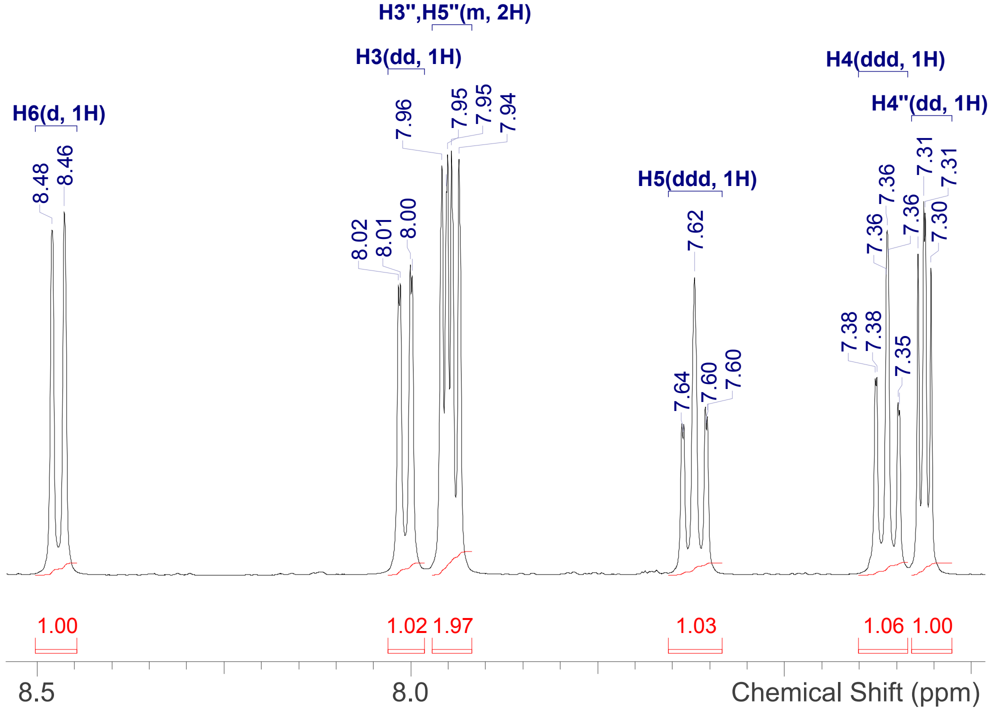

NVR-19_1H.spectrus

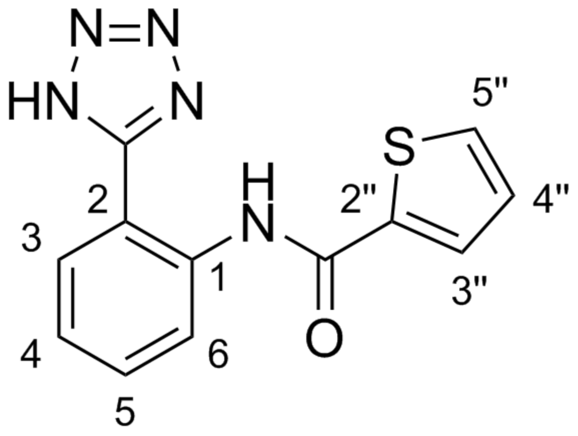

Shift (ppm)	C	m	Assign
159.8	1	s	CONH
154.6	1	br s	Tet-C
139.6	1	s	2''
136.8	1	s	1
132.6	1	s	3''
131.9	1	s	5''
129.0	1	s	4''
128.7	1	s	5
128.4	1	s	3
124.3	1	s	4
121.9	1	s	6
113.4	1	s	2

Acquisition Time (sec)	2.0447
Date	05/08/2017 08:18:00
Date Stamp	05/08/2017 08:18:00
Frequency (MHz)	125.7870
Nucleus	13C
Number of Transients	256
Solvent	DMSO-d6

^{13}C NMR (126 MHz, *DMSO-d$_6$*) δ ppm 159.8 (s, 1 C), 154.6 (br s, 1 C), 139.6 (s, 1 C), 136.8 (s, 1 C), 132.6 (s, 1 C), 131.9 (s, 1 C), 129.0 (s, 1 C), 128.7 (s, 1 C), 128.4 (s, 1 C), 124.3 (s, 1 C), 121.9 (s, 1 C), 113.4 (s, 1 C)

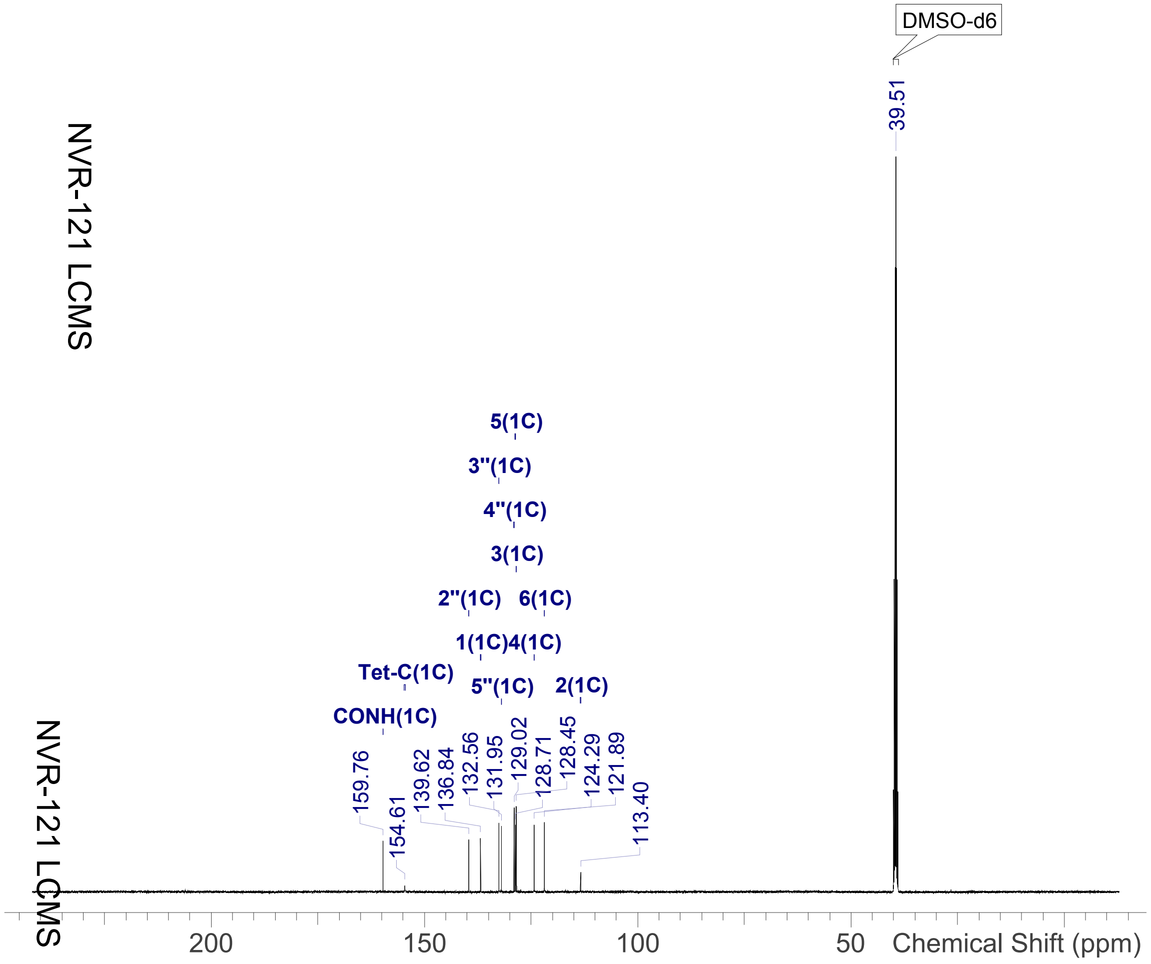

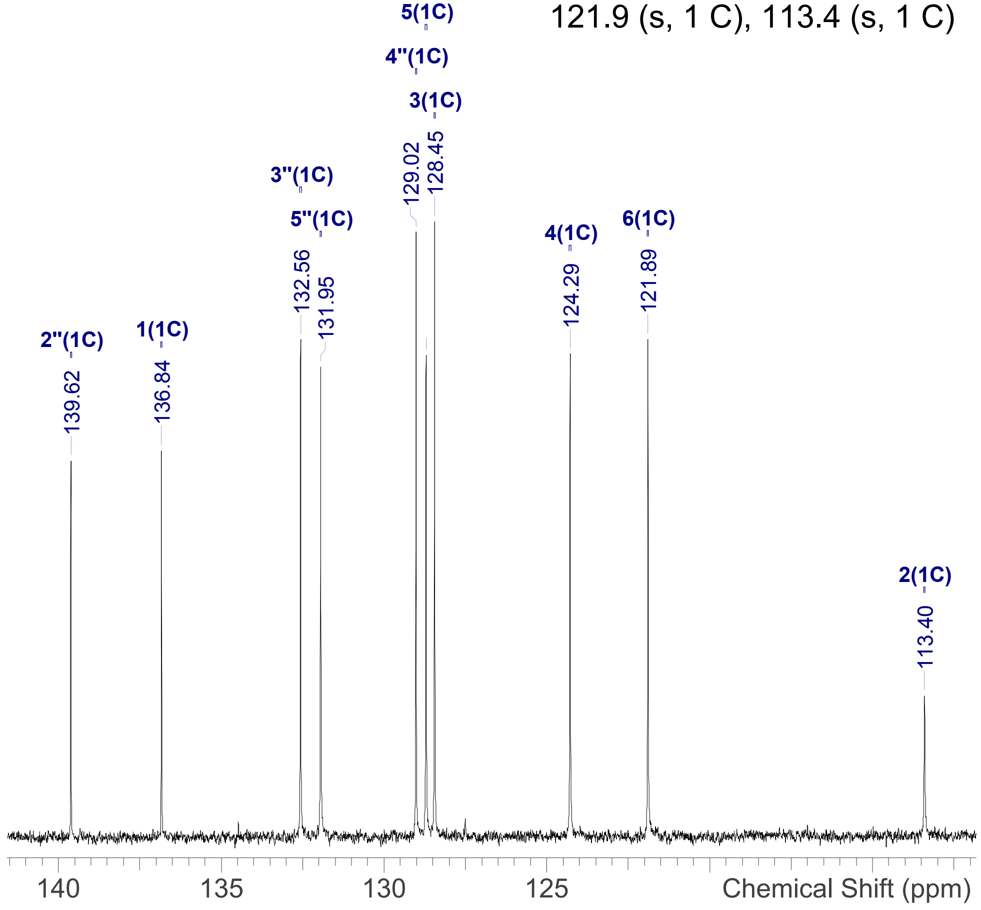

NVR-19_13C.spectrus

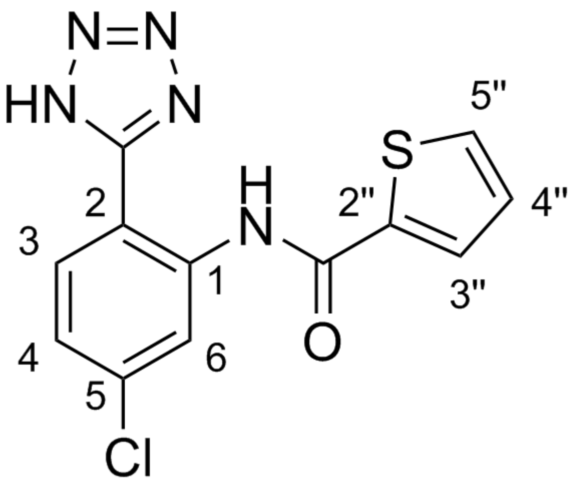

Shift (ppm)	H	m	J (Hz)	Assign
11.74	1	s	-	CONH
8.59	1	d	2.1	H6
8.03	1	d	8.4	H3
7.96	1	dd	5.0, 0.9	H3''
7.92	1	dd	3.7, 0.9	H5''
7.40	1	dd	8.5, 2.2	H4
7.31	1	dd	4.9, 3.8	H4''

Acquisition Time (sec)	6.5536
Date	03/08/2017 16:35:00
Date Stamp	03/08/2017 16:35:00
Frequency (MHz)	500.1930
Nucleus	1H
Number of Transients	16
Solvent	DMSO-d6

^{1}H NMR (500 MHz, *DMSO-d_6*) δ ppm 11.74 (s, 1 H), 8.59 (d, *J*=2.1 Hz, 1 H), 8.03 (d, *J*=8.4 Hz, 1 H), 7.96 (dd, *J*=5.0, 0.9 Hz, 1 H), 7.92 (dd, *J*=3.7, 0.9 Hz, 1 H), 7.40 (dd, *J*=8.5, 2.2 Hz, 1 H), 7.31 (dd, *J*=4.9, 3.8 Hz, 1 H)

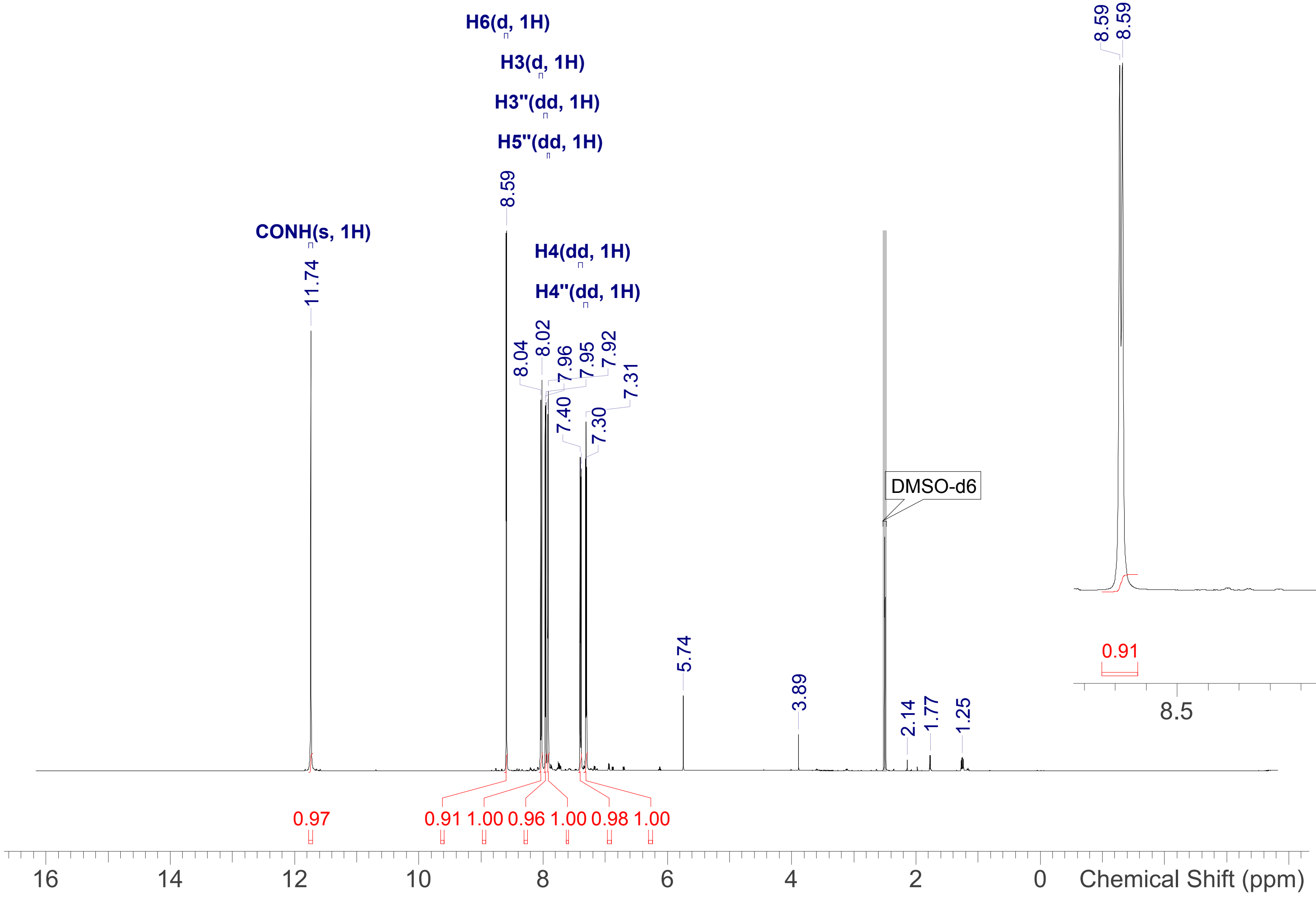

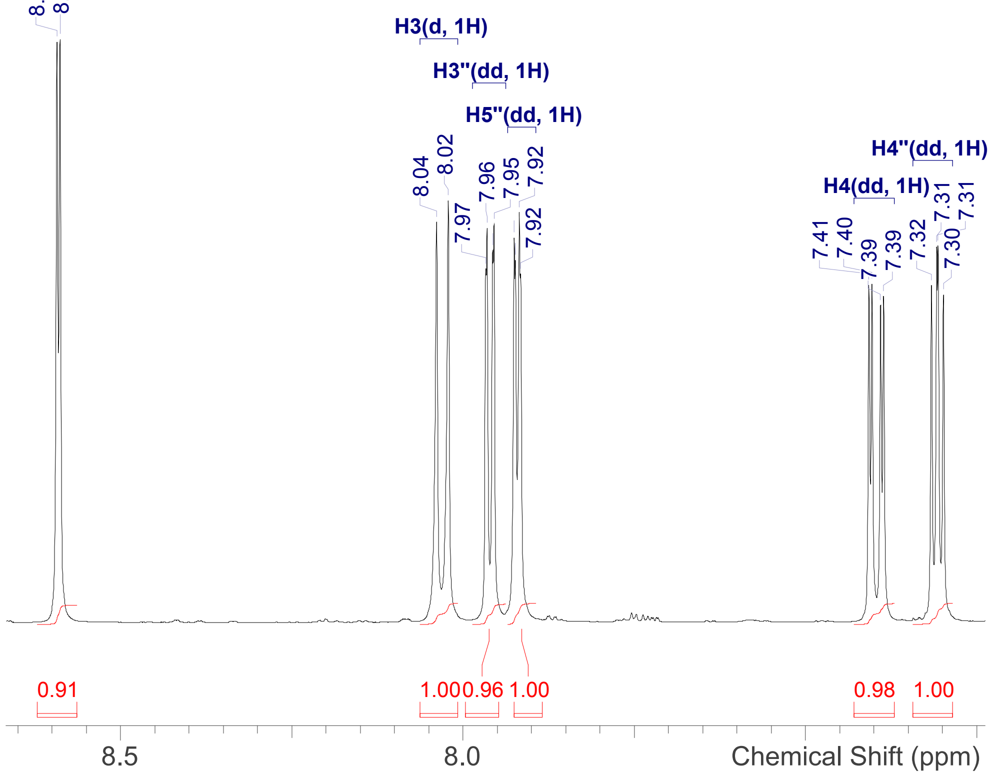

NVR-20_1H.spectrus

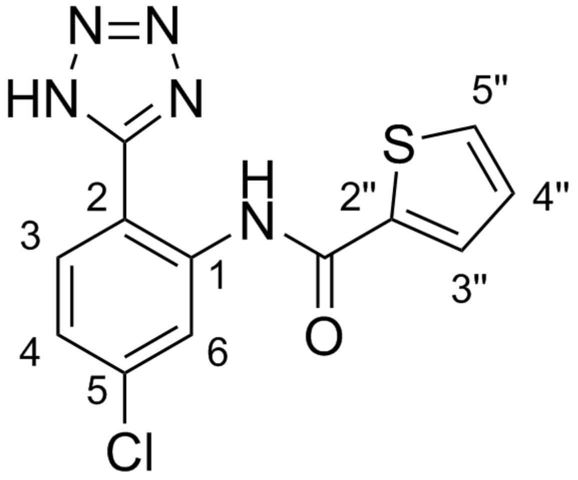

Shift (ppm)	C	m	Assign
159.9	1	s	CONH
154.3	1	br s	Tet-C
139.1	1	s	2''
138.0	1	s	1
136.2	1	s	5
133.1	1	s	3''
130.0	1	s	4''
129.2	1	s	5''
128.5	1	s	3
123.9	1	s	4
120.6	1	s	6
111.6	1	s	2

Acquisition Time (sec)	2.0447
Date	05/08/2017 09:02:00
Date Stamp	05/08/2017 09:02:00
Frequency (MHz)	125.7870
Nucleus	13C
Number of Transients	256
Solvent	DMSO-d6

^{13}C NMR (126 MHz, *DMSO-d$_6$*) δ ppm 159.9 (s, 1 C), 154.3 (br s, 1 C), 139.1 (s, 1 C), 138.0 (s, 1 C), 136.2 (s, 1 C), 133.1 (s, 1 C), 130.0 (s, 1 C), 129.2 (s, 1 C), 128.5 (s, 1 C), 123.9 (s, 1 C), 120.6 (s, 1 C), 111.6 (s, 1 C)

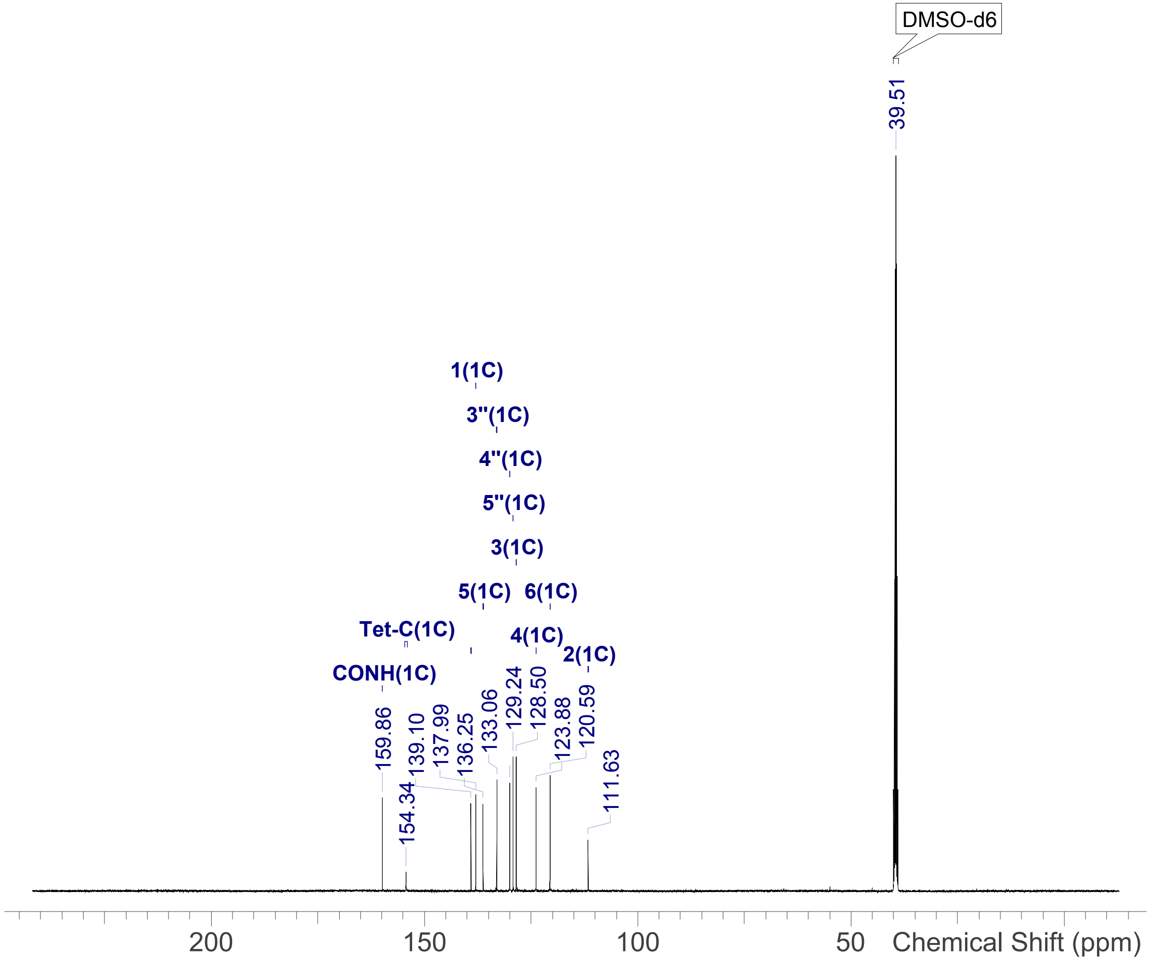

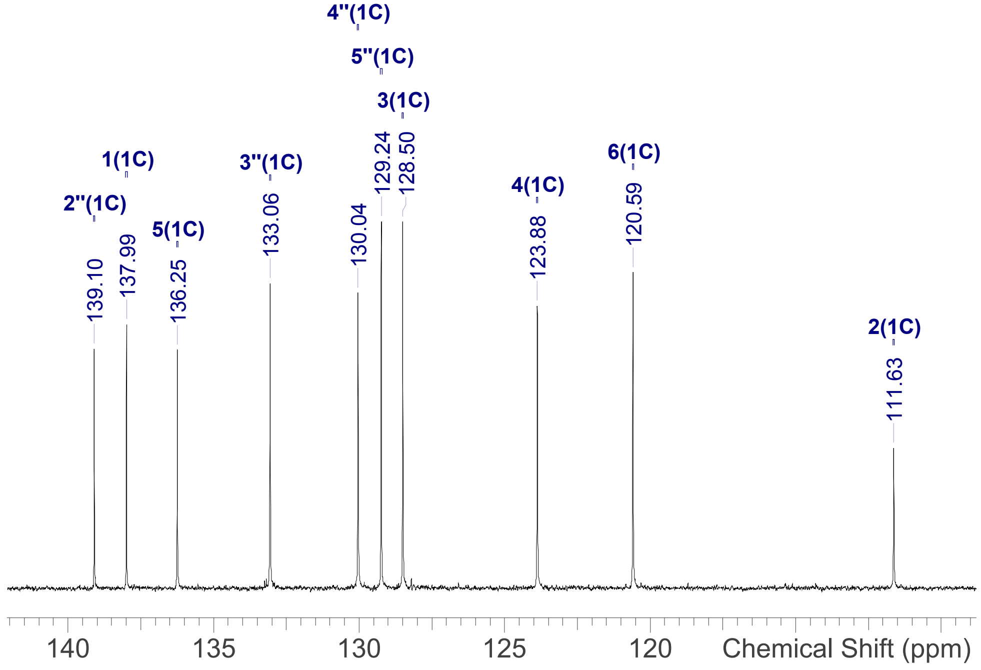

NVR-20_13C.spectrus

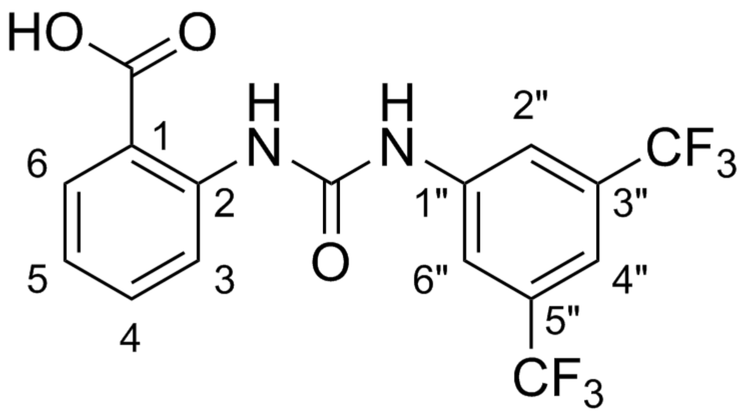

Shift (ppm)	H	m	J (Hz)	Assign
13.54	1	br s	-	COOH
10.66	1	s	-	NH
10.53	1	s	-	NH"
8.42	1	dd	8.5, 0.8	3
8.20	2	br s	-	2", 6"
7.98	1	dd	7.9, 1.6	6
7.63	1	br s	-	4"
7.56	1	ddd	8.7, 7.2, 1.8	4
7.08	1	ddd	7.9, 7.2, 0.8	5

Acquisition Time (sec)	3.9846
Date	15 May 2019 16:55:57
Date Stamp	15 May 2019 16:55:57
Frequency (MHz)	400.0700
Nucleus	1H
Number of Transients	4
Solvent	DMSO-d6
Temperature (degree C)	24.999

^{1}H NMR (400 MHz, *DMSO-d_6*) δ ppm 13.54 (br s, 1 H), 10.66 (s, 1 H), 10.53 (s, 1 H), 8.42 (dd, *J*=8.5, 0.8 Hz, 1 H), 8.20 (br s, 2 H), 7.98 (dd, *J*=7.9, 1.6 Hz, 1 H), 7.63 (br s, 1 H), 7.56 (ddd, *J*=8.7, 7.2, 1.8 Hz, 1 H), 7.08 (ddd, *J*=7.9, 7.2, 0.8 Hz, 1 H)

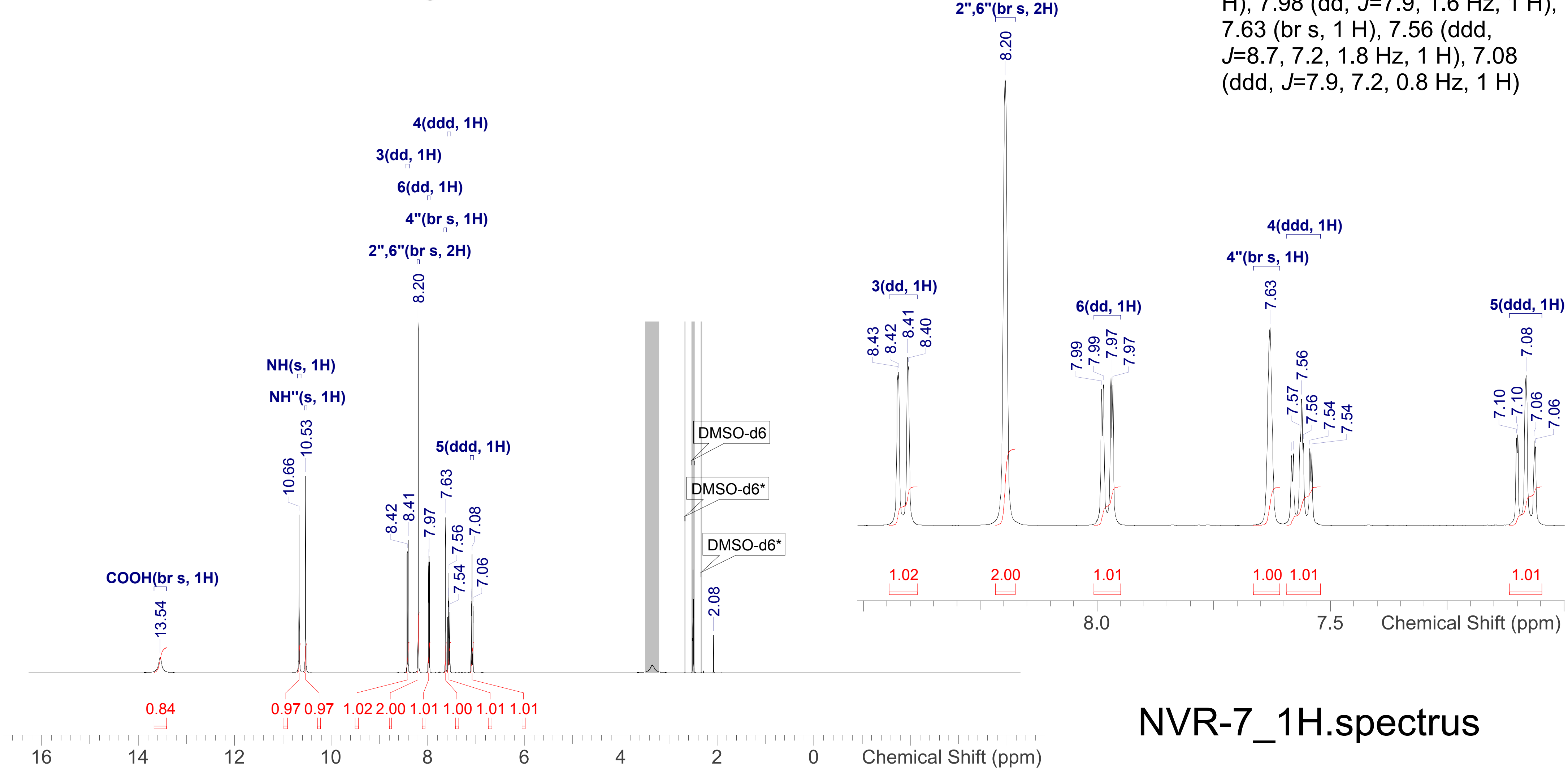

NVR-7_1H.spectrus

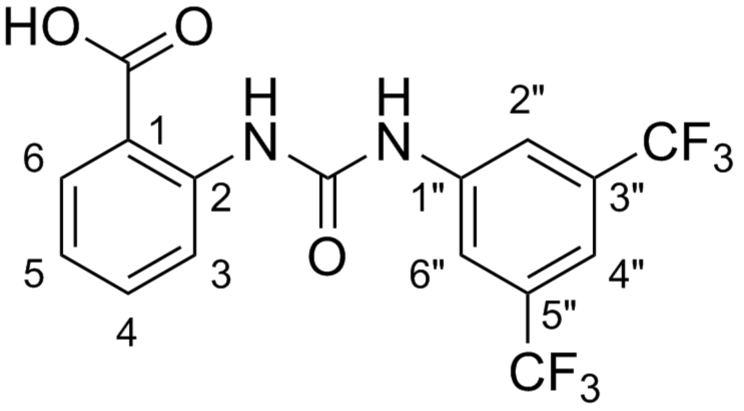

Shift (ppm)	C	m	J (Hz)	Assign
169.6	1	s	-	COOH
152.1	1	s	-	CO
141.9	1	s	-	1"
141.6	1	s	-	2
133.9	1	s	-	4
131.1	1	s	-	6
130.7	2	q	32.3	5", 3"
123.3	2	q	272.9	3"-CF3, 5"-CF3
121.5	1	s	-	5
119.7	1	s	-	3
118.0	2	br q	3.9	6", 2"
115.6	1	s	-	1
114.5	1	br spt	3.9	4"

Acquisition Time (sec)	1.0224
Date	16 May 2019 00:00:15
Date Stamp	16 May 2019 00:00:15
Frequency (MHz)	100.5977
Nucleus	13C
Number of Transients	256
Solvent	DMSO-d6
Temperature (degree C)	25.001

^{13}C NMR (101 MHz, *DMSO-d$_6$*) δ ppm 169.6 (s, 1 C), 152.1 (s, 1 C), 141.9 (s, 1 C), 141.6 (s, 1 C), 133.9 (s, 1 C), 131.1 (s, 1 C), 130.7 (q, *J*=32.3 Hz, 2 C), 121.5 (s, 1 C), 119.7 (s, 1 C), 123.3 (q, *J*=272.9 Hz, 2 C), 118.0 (br q, *J*=3.9 Hz, 2 C), 115.6 (s, 1 C), 114.5 (br spt, *J*=3.9 Hz, 1 C)

NVR-7_13C

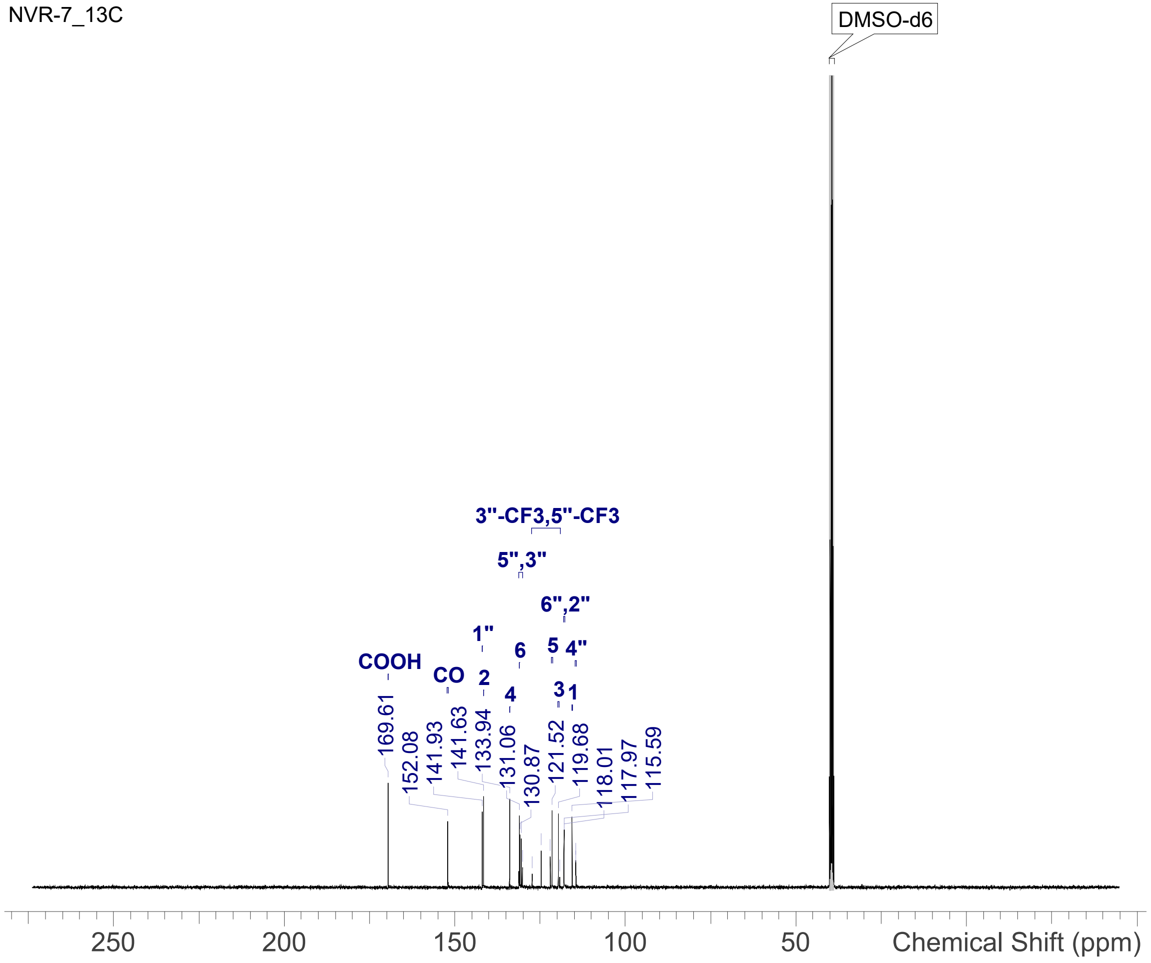

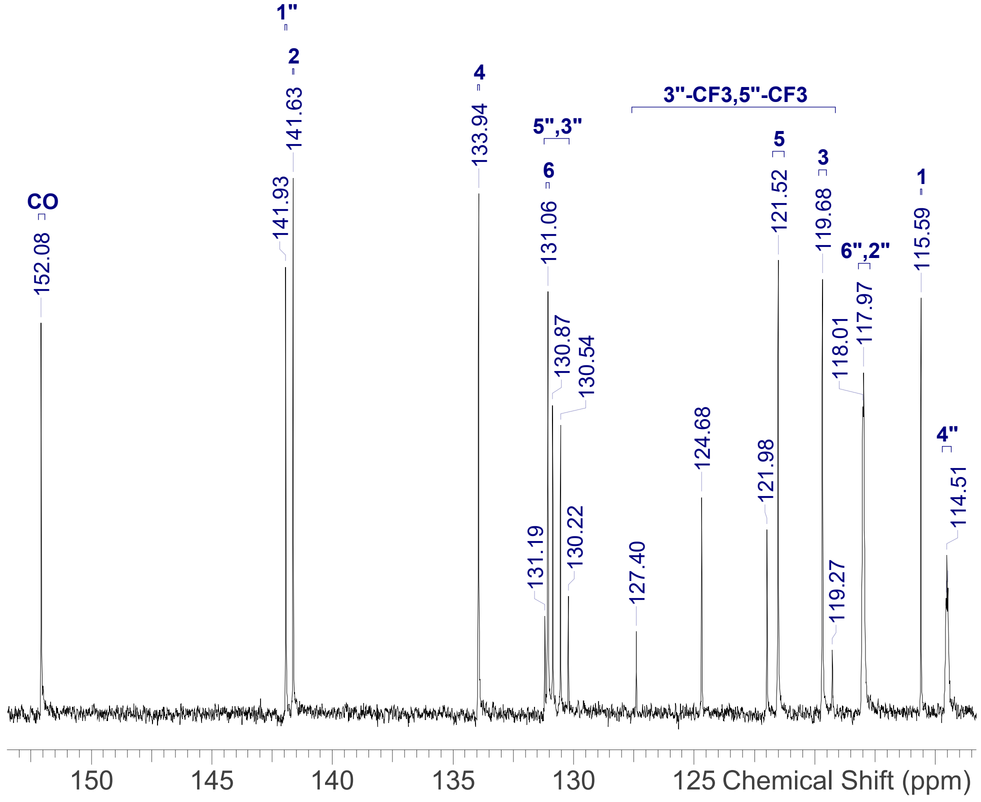

NVR-7_13C.spectrus

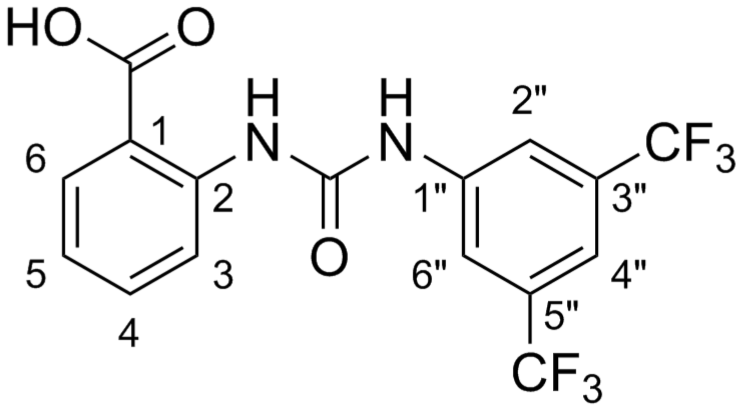

Shift (ppm)	F	m
-61.80	6	s

Acquisition Time (sec)	1.4680
Date	16 May 2019 01:06:28
Date Stamp	16 May 2019 01:06:28
Frequency (MHz)	376.4419
Nucleus	19F
Number of Transients	16
Solvent	DMSO-d6
Temperature (degree C)	24.999

^{19}F NMR (376 MHz, *DMSO-d_6*) δ ppm -61.80 (s, 6 F)

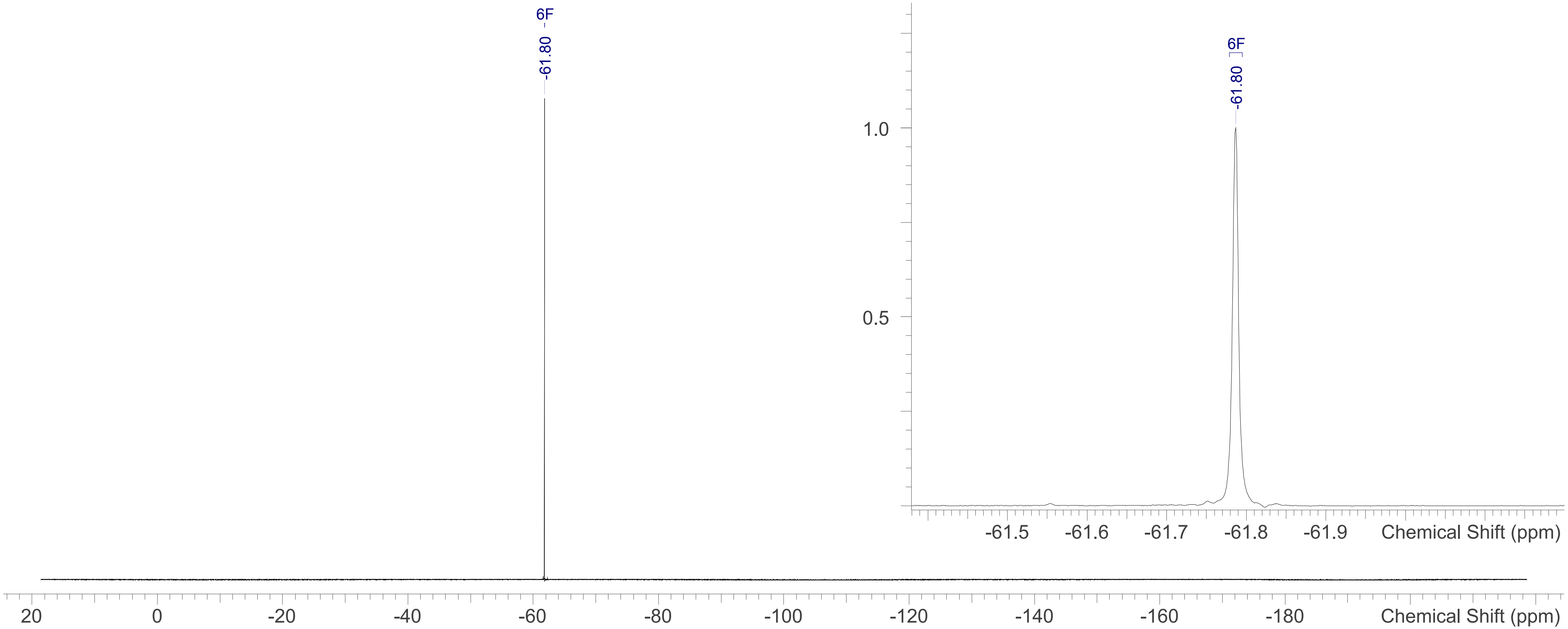

NVR-7_19F.spectrus

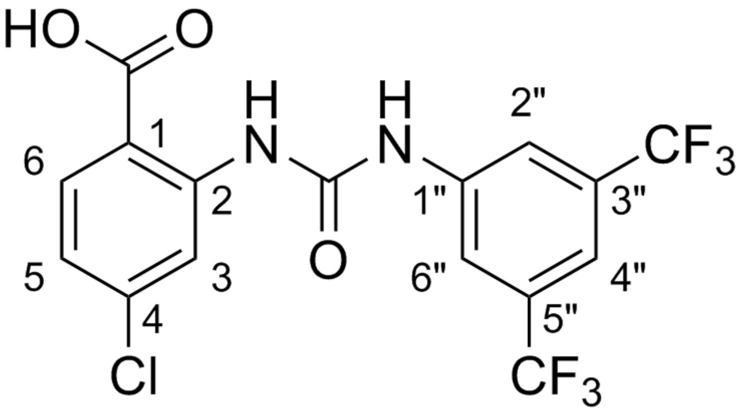

Shift (ppm)	H	m	J (Hz)	Assign
13.75	1	br s	-	COOH
10.77	1	s	-	NH
10.63	1	s	-	NH"
8.53	1	d	2.3	3
8.17	2	br s	-	6", 2"
7.95	1	d	8.5	6
7.63	1	br s	-	4"
7.11	1	dd	8.7, 2.1	5

Acquisition Time (sec)	3.9846
Date	11 May 2019 02:13:44
Date Stamp	11 May 2019 02:13:44
Frequency (MHz)	400.0700
Nucleus	1H
Number of Transients	16
Solvent	DMSO-d6
Temperature (degree C)	24.999

^{1}H NMR (400 MHz, *DMSO-d_6*) δ ppm 13.75 (br s, 1 H), 10.77 (s, 1 H), 10.63 (s, 1 H), 8.53 (d, *J*=2.3 Hz, 1 H), 8.17 (br s, 2 H), 7.95 (d, *J*=8.5 Hz, 1 H), 7.63 (br s, 1 H), 7.11 (dd, *J*=8.7, 2.1 Hz, 1 H)

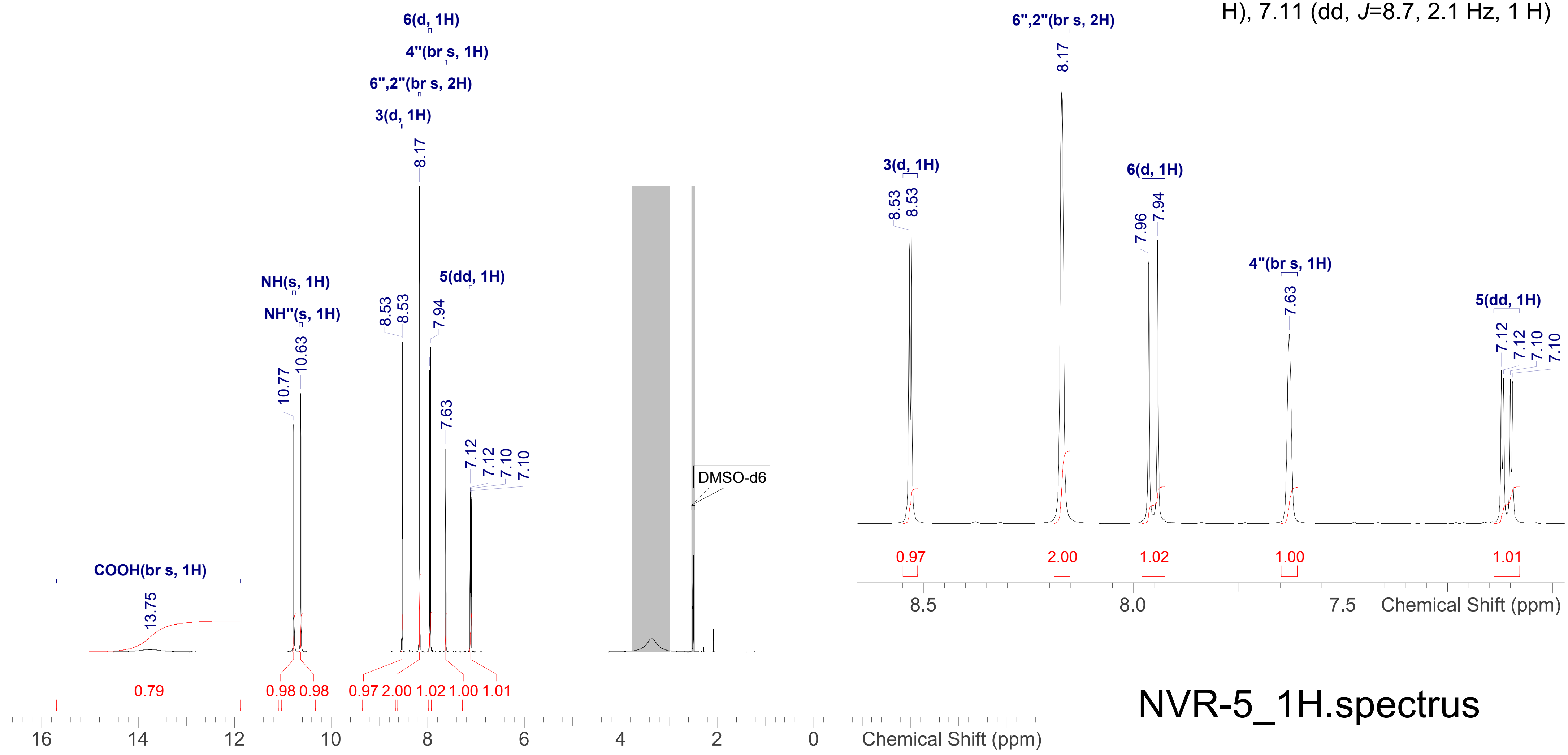

NVR-5_1H.spectrus

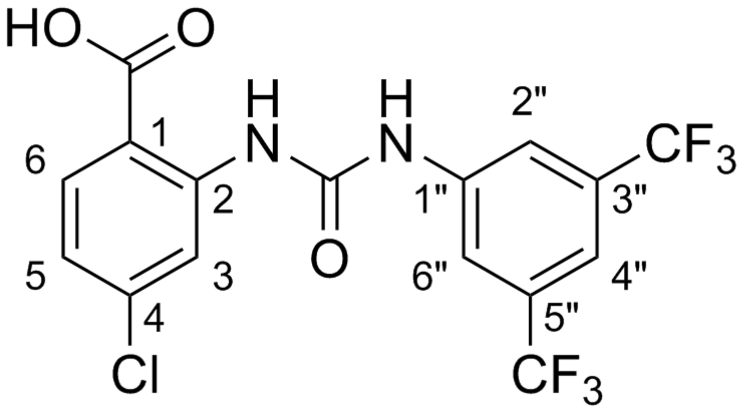

Shift (ppm)	C	m	J (Hz)	Assign
168.9	1	s	-	COOH
151.9	1	s	-	CO
142.8	1	s	-	2
141.6	1	s	-	1"
138.5	1	s	-	4
132.7	1	s	-	6
130.7	2	q	32.6	5", 3"
123.3	2	q	272.9	3"-CF3, 5"-CF3
121.3	1	s	-	5
118.7	1	s	-	3
118.1	2	br q	3.9	6", 2"
114.8	1	br spt	3.9	4"
114.1	1	s	-	1

Acquisition Time (sec)	1.0224
Date	11 May 2019 02:27:59
Date Stamp	11 May 2019 02:27:59
Frequency (MHz)	100.5977
Nucleus	13C
Number of Transients	256
Solvent	DMSO-d6
Temperature (degree C)	24.999

^{13}C NMR (101 MHz, *DMSO-d_6*) δ ppm 168.9 (s, 1 C), 151.9 (s, 1 C), 142.8 (s, 1 C), 141.6 (s, 1 C), 138.5 (s, 1 C), 132.7 (s, 1 C), 130.7 (q, *J*=32.6 Hz, 2 C), 121.3 (s, 1 C), 123.3 (q, *J*=272.9 Hz, 2 C), 118.7 (s, 1 C), 118.1 (br q, *J*=3.9 Hz, 2 C), 114.8 (br spt, *J*=3.9 Hz, 1 C), 114.1 (s, 1 C)

NVR-5_13C

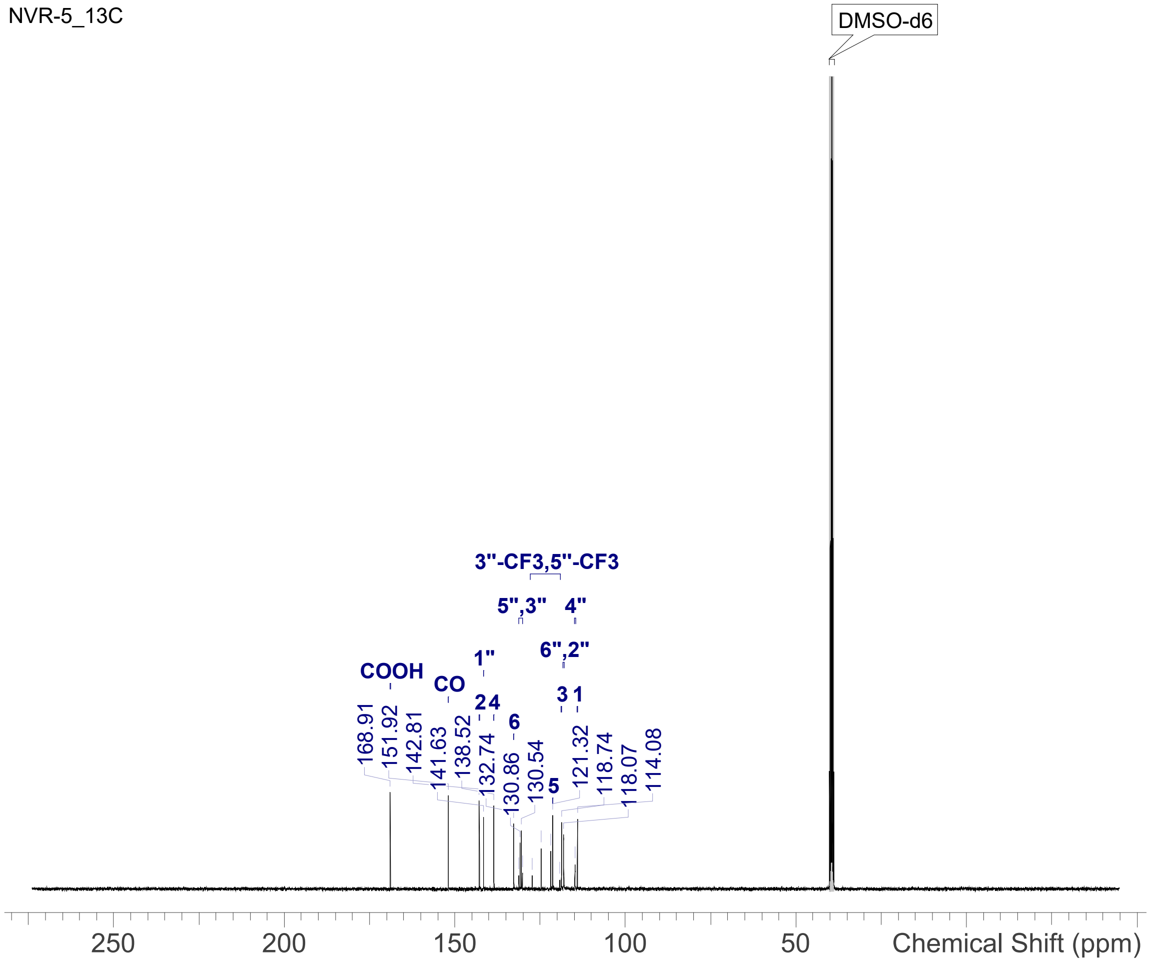

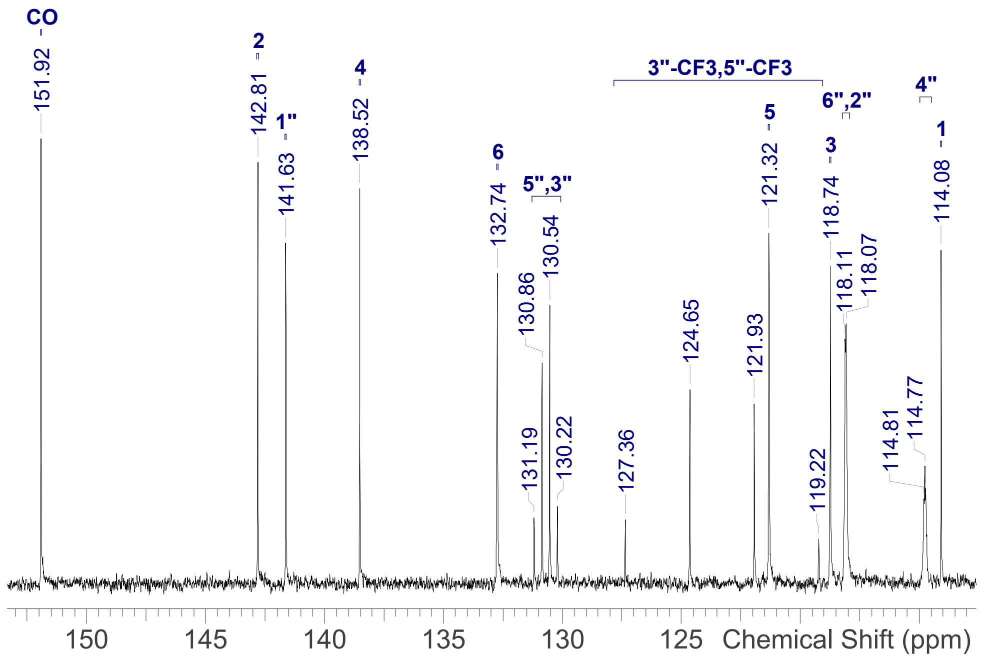

NVR-5_13C.spectrus

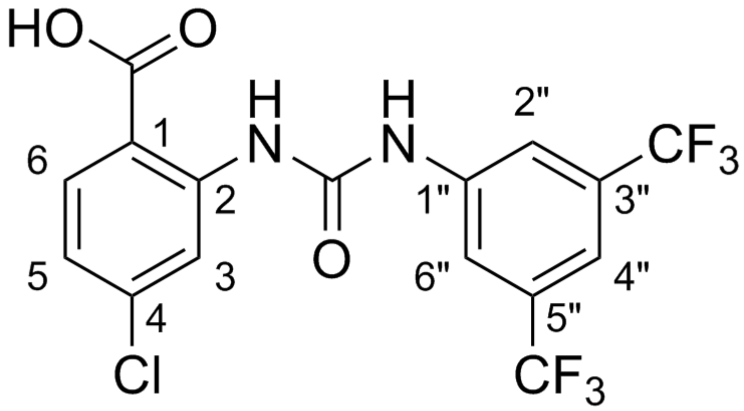

Shift (ppm)	F	m
-61.81	6	s

Acquisition Time (sec)	1.4680
Date	11 May 2019 03:34:05
Date Stamp	11 May 2019 03:34:05
Frequency (MHz)	376.4419
Nucleus	19F
Number of Transients	16
Solvent	DMSO-d6
Temperature (degree C)	24.999

^{19}F NMR (376 MHz, *DMSO-d_6*) δ ppm -61.81 (s, 6 F)

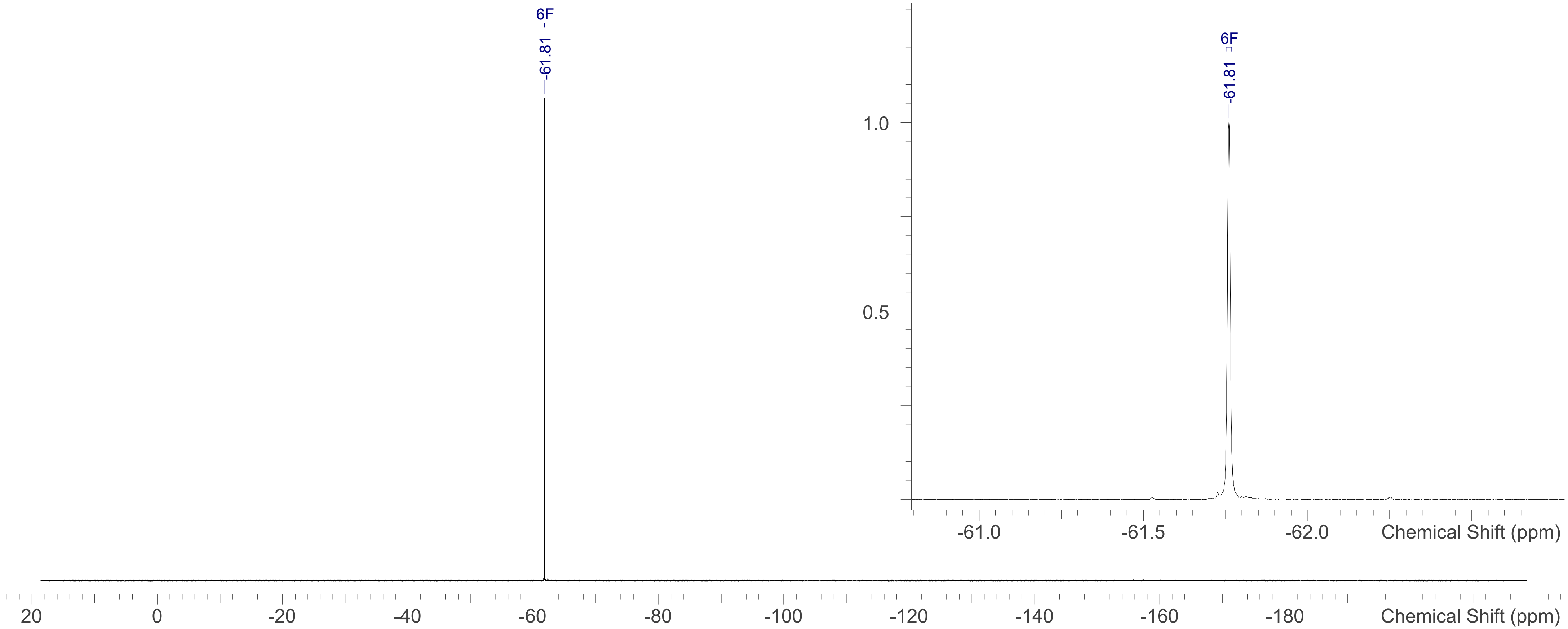

NVR-5_19F.spectrus

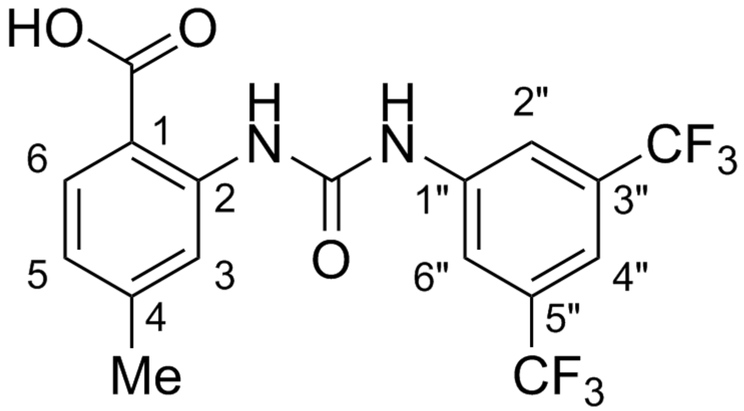

No.	Shift (ppm)	H	m	J (Hz)	Assign
1	13.36	1	br s	-	COOH
2	10.73	1	br s	-	NH
3	10.52	1	s	-	NH"
4	8.28	1	s	-	3
5	8.21	2	br s	-	6", 2"
6	7.87	1	d	8.0	6
7	7.65	1	s	-	4"
8	6.91	1	br d	8.0	5
9	2.35	3	s	-	Me

Acquisition Time (sec)	3.9846
Date	01 Apr 2019 19:50:29
Date Stamp	01 Apr 2019 19:50:29
Frequency (MHz)	400.0700
Nucleus	1H
Number of Transients	16
Solvent	DMSO-d6
Temperature (degree C)	24.999

^{1}H NMR (400 MHz, *DMSO-d_6*) δ ppm 13.36 (br s, 1 H), 10.73 (br s, 1 H), 10.52 (s, 1 H), 8.28 (s, 1 H), 8.21 (br s, 2 H), 7.87 (d, *J*=8.0 Hz, 1 H), 7.65 (s, 1 H), 6.91 (br d, *J*=8.0 Hz, 1 H), 2.35 (s, 3 H)

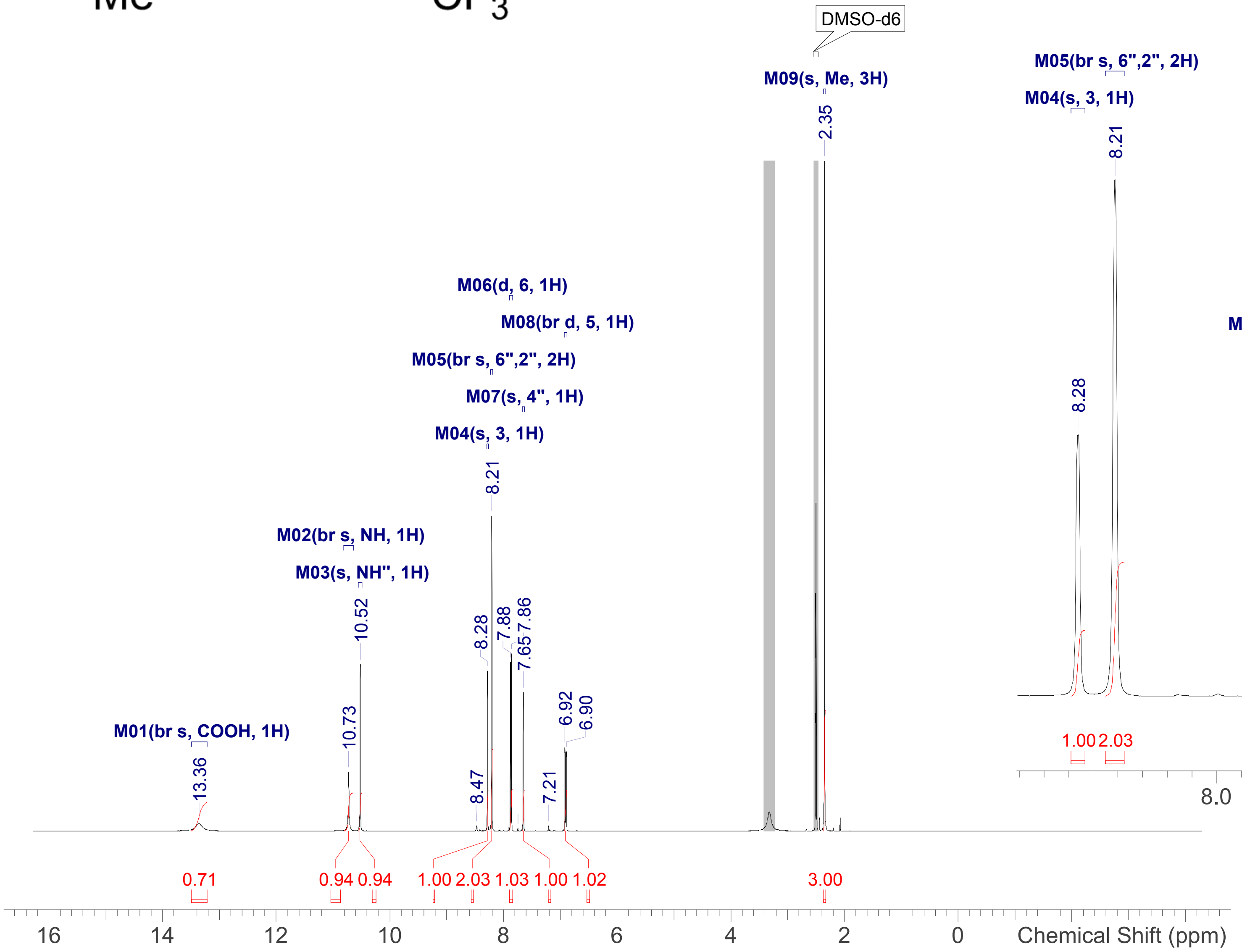

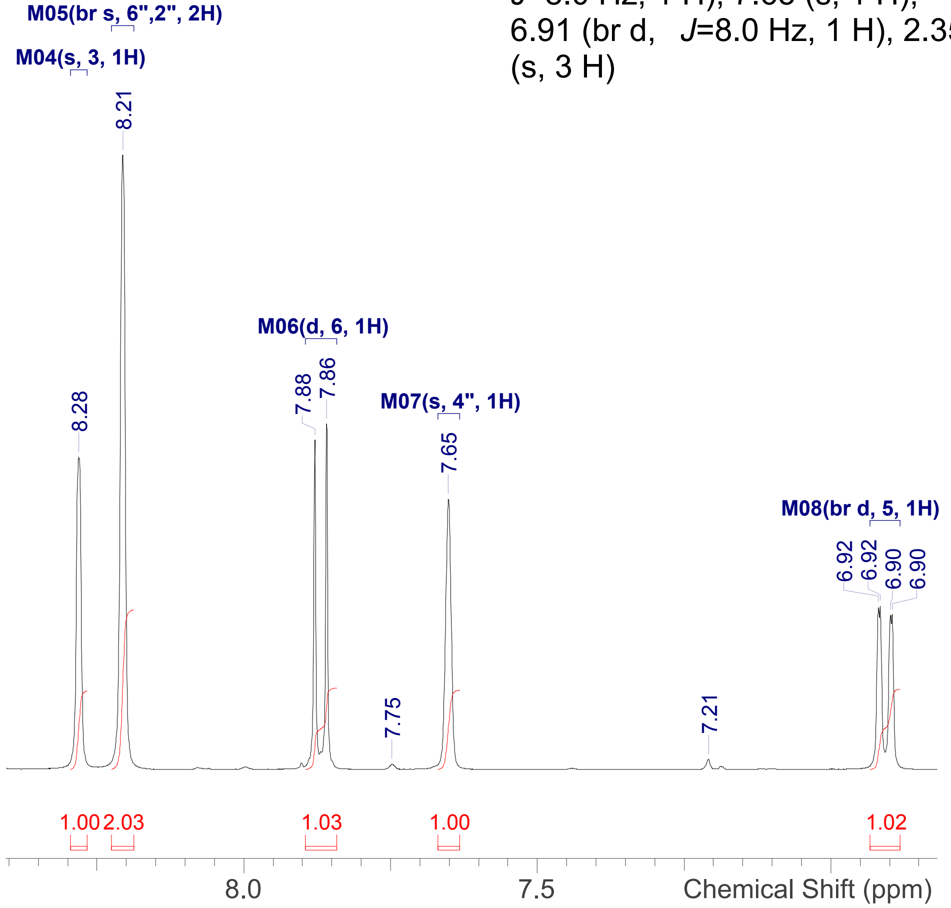

NVR-121_1H.spectrus

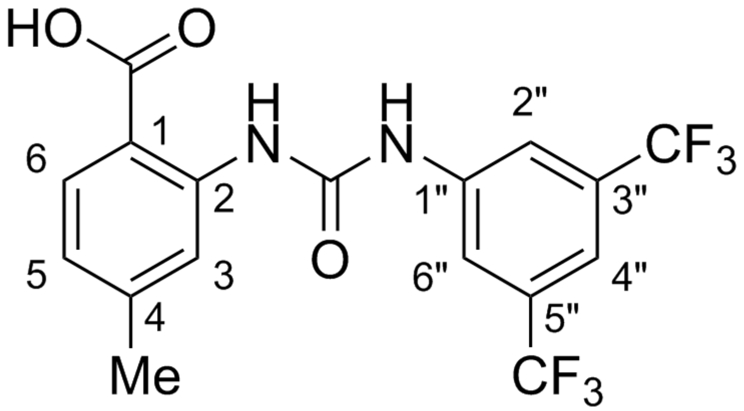

Shift (ppm)	C	m	J (Hz)	Assign
169.6	1	s	-	COOH
152.0	1	s	-	CO
144.4	1	s	-	4
142.0	1	s	-	1"
141.7	1	s	-	2
131.1	1	s	-	6
130.7	2	q	32.9	5", 3"
123.3	2	q	272.9	3"-CF3, 5"-CF3
122.4	1	s	-	5
119.8	1	s	-	3
118.0	2	br q	3.9	6", 2"
114.5	1	br spt	3.9	4"
112.9	1	s	-	1
21.6	1	s	-	Me

Acquisition Time (sec)	1.0224
Date	11 May 2019 01:26:03
Date Stamp	11 May 2019 01:26:03
Frequency (MHz)	100.5977
Nucleus	13C
Number of Transients	256
Solvent	DMSO-d6
Temperature (degree C)	25.000

^{13}C NMR (101 MHz, *DMSO-d_6*) δ ppm 169.6 (s, 1 C), 152.0 (s, 1 C), 144.4 (s, 1 C), 142.0 (s, 1 C), 141.7 (s, 1 C), 131.1 (s, 1 C), 130.7 (q, *J*=32.9 Hz, 2 C), 122.4 (s, 1 C), 119.8 (s, 1 C), 123.3 (q, *J*=272.9 Hz, 2 C), 118.0 (br q, *J*=3.9 Hz, 2 C), 114.5 (br spt, *J*=3.9 Hz, 1 C), 112.9 (s, 1 C), 21.6 (s, 1 C)

NVR-121_13C

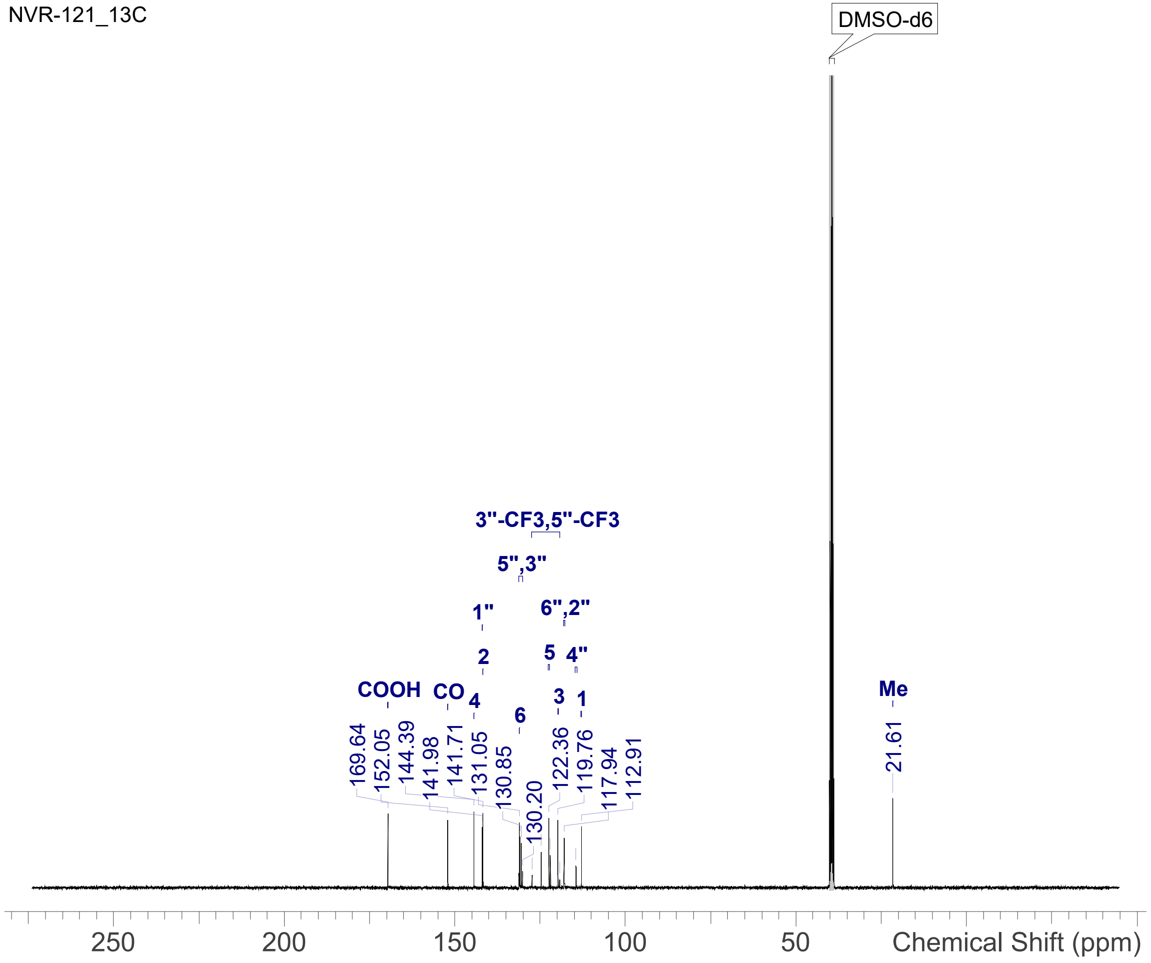

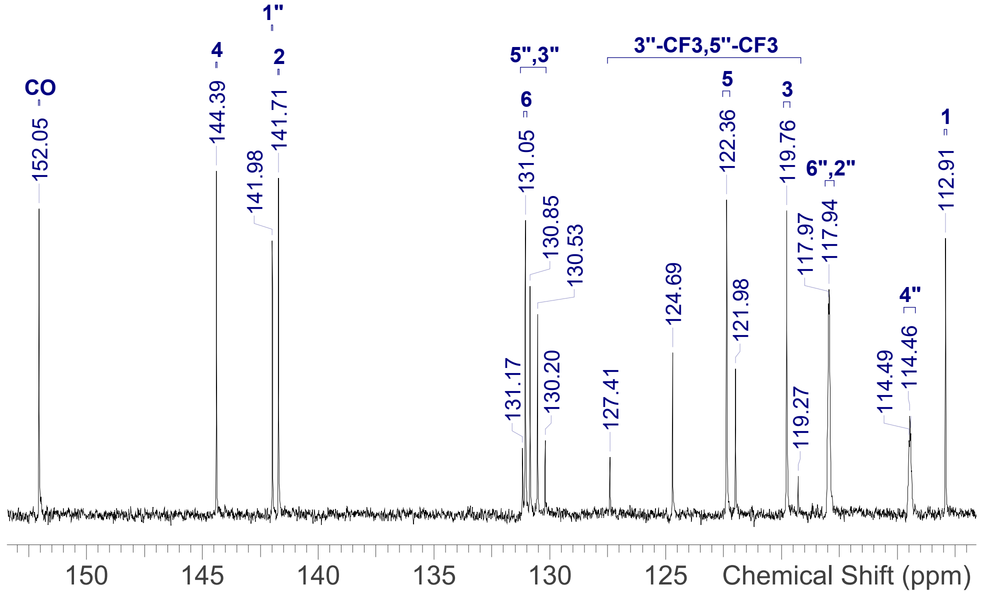

NVR-121_13C.spectrus

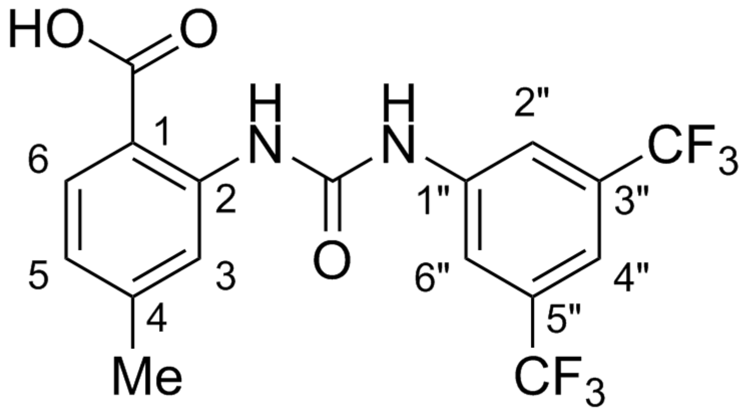

Shift (ppm)	F	m
-61.72	6	s

Acquisition Time (sec)	1.4680
Date	01 Apr 2019 21:10:40
Date Stamp	01 Apr 2019 21:10:40
Frequency (MHz)	376.4419
Nucleus	19F
Number of Transients	16
Solvent	DMSO-d6
Temperature (degree C)	25.001

^{19}F NMR (376 MHz, *DMSO-d_6*) δ ppm -61.72 (s, 6 F)

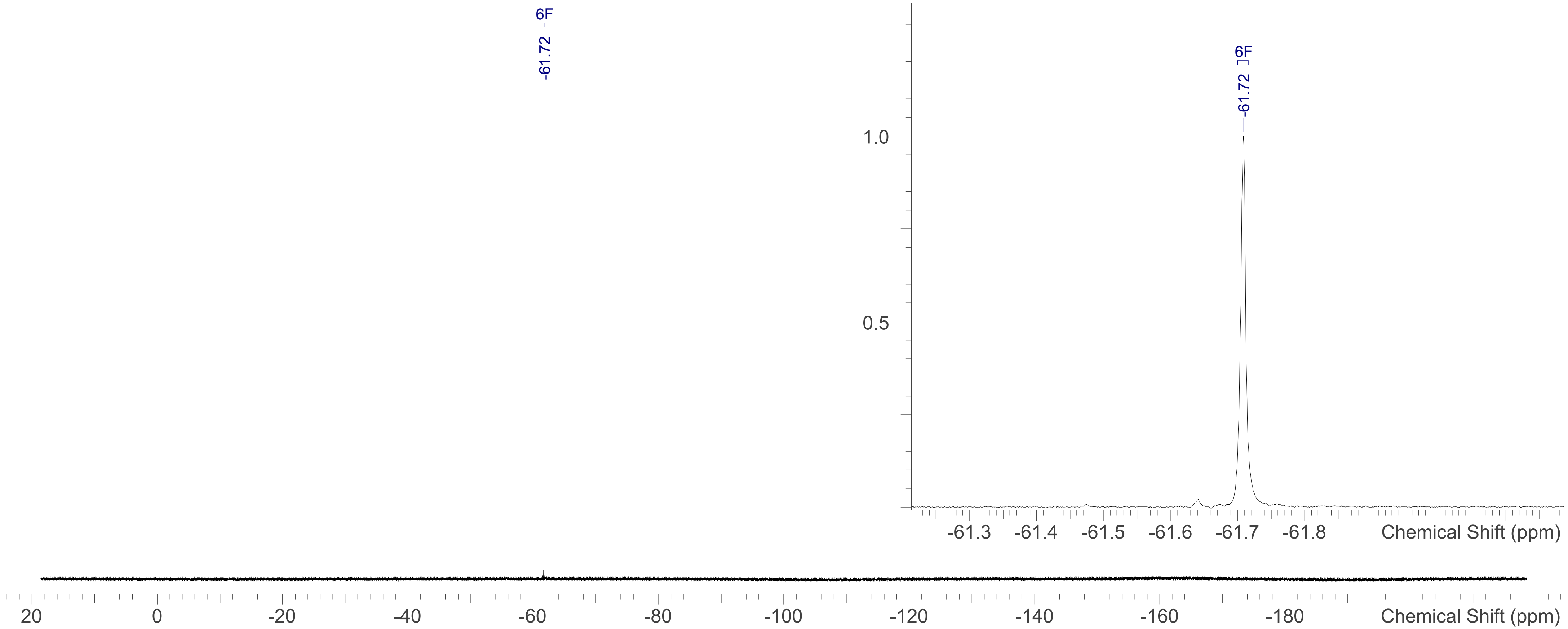

NVR-121_19F.spectrus

Openlynx Report - James B

Sample: 1 | Vial:2:37 | ID:JB153-1
File:JB153-1 | Date:04-Apr-2019 | Time:15:53:22
Description:

Printed: Mon Apr 15 12:24:18 2019

3: UV Detector: TAC: Wavelength Range: (210 - 400) 3.092e+2 Range: 3.197e+2

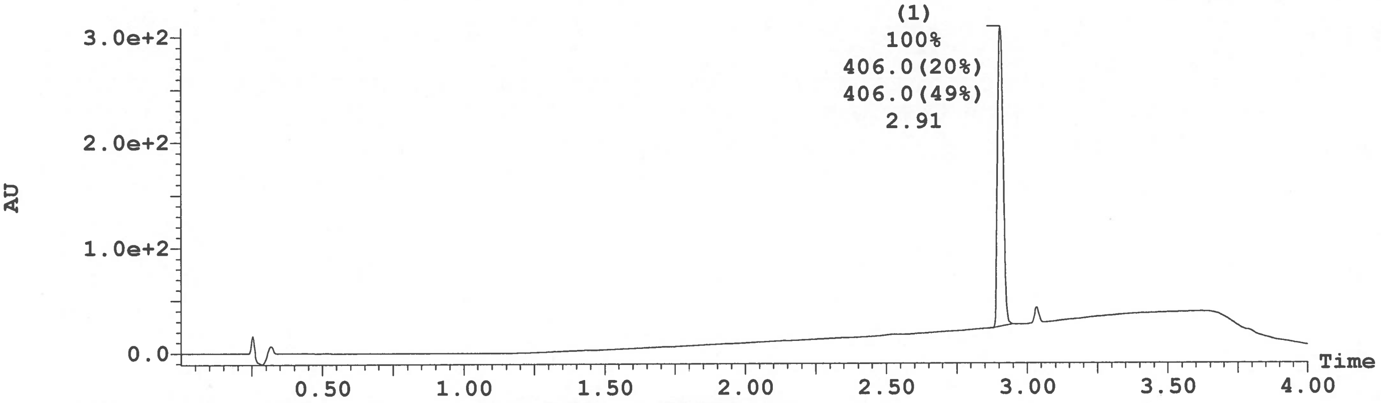

Peak Number	Compound	Time	Area %Total	Mass Found
1	Found	2.91	100.00	406.0, 406.0

1: MS ES+ :TIC Smooth (Mn, 2x2) 1.9e+007

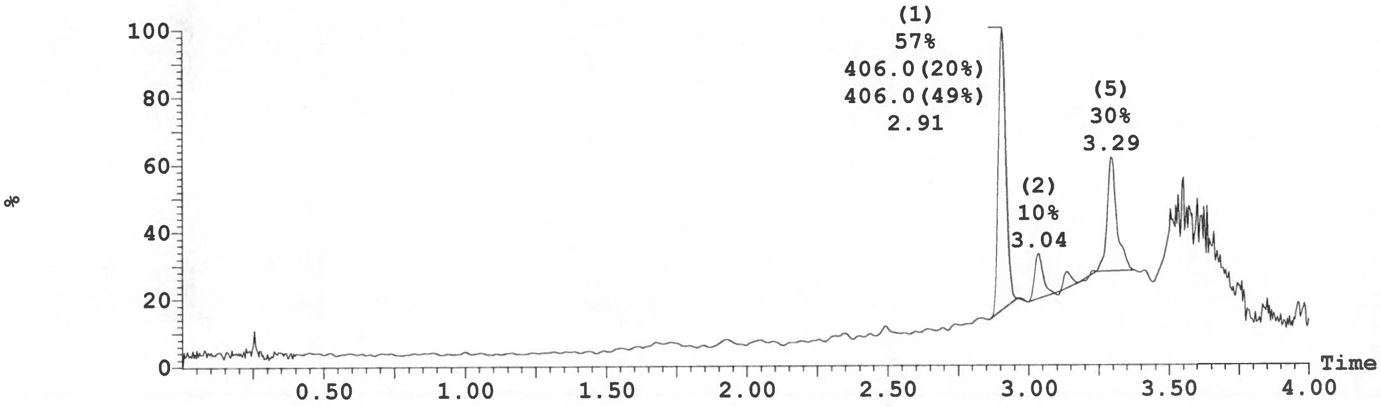

2: MS ES- :TIC Smooth (Mn, 2x2) 8.5e+006

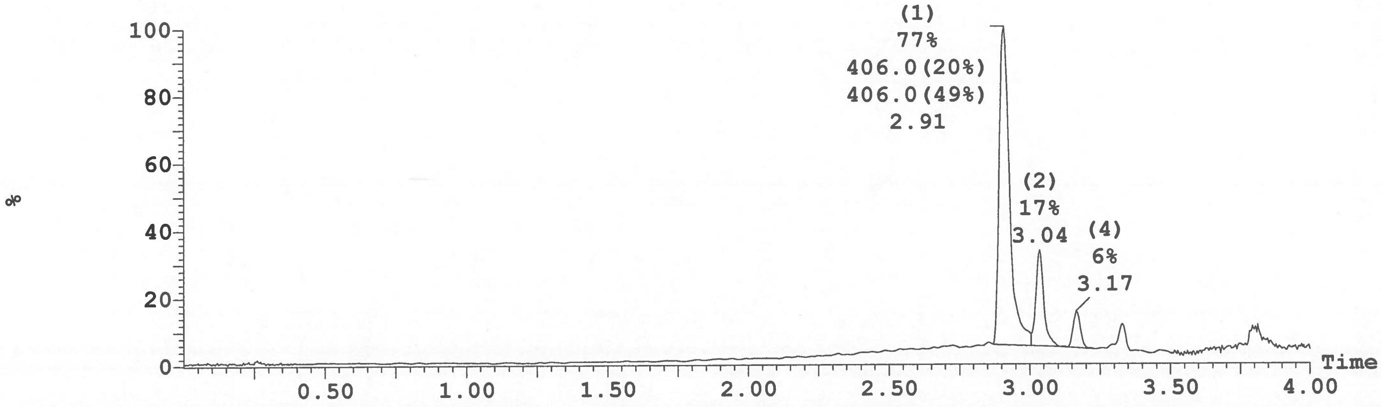

NVR-121 LCMS

Sample: 1	Vial:2:37	ID:JB153-1
File:JB153-1	Date:04-Apr-2019	Time:15:53:22
Description:		

Printed: Mon Apr 15 12:24:18 2019

Peak ID	Compound	Time	Mass Found
1	Found	2.91	407

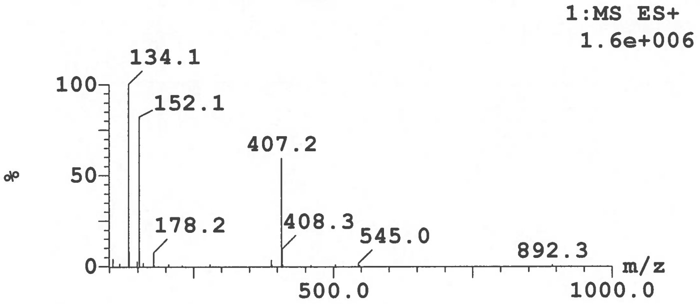

Peak ID	Compound	Time	Mass Found
1	Found	2.91	405

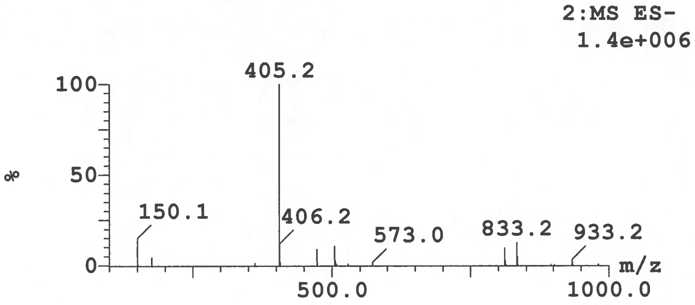

Peak ID	Compound	Time	Mass Found
1		2.91	Not Found

1: (Time: 2.91) Combine (1744) 3:UV Detector 3.088 AU

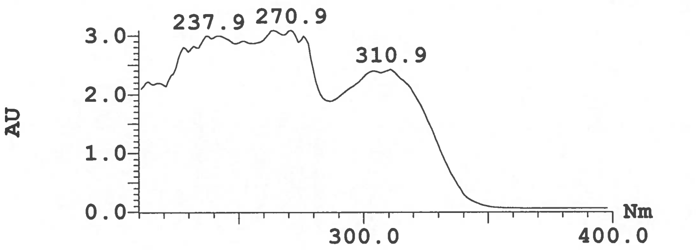

NVR-121 LCMS

Thermo Exactive Plus EMR Orbitrab ASAP pos

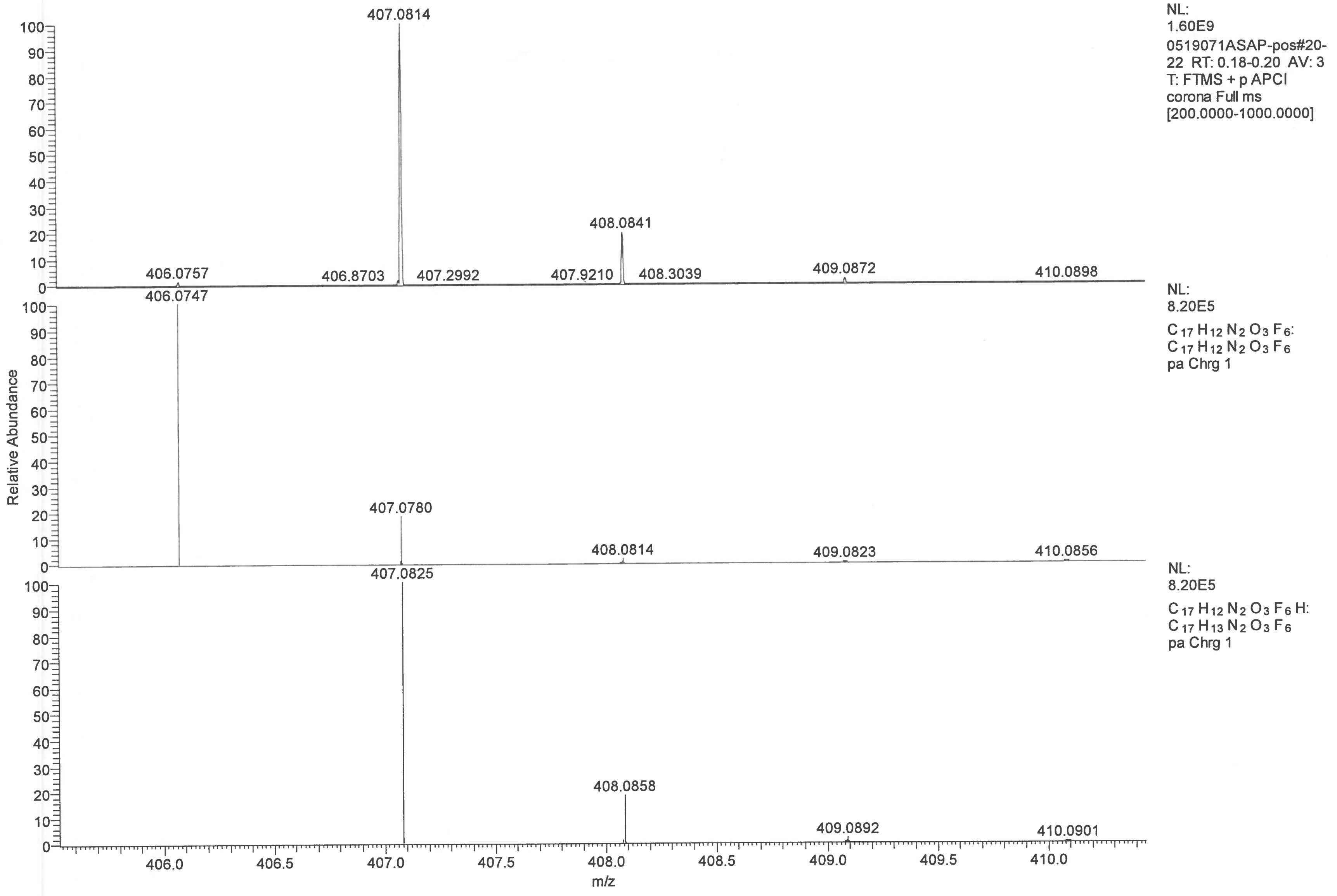

NVR-121 HRMS

Thermo Exactive Plus EMR Orbitrab ASAP pos

m/z	Theo. Mass	Delta (ppm)	RDB equiv.	Composition
406.0757	406.0747	2.55	10.0	C17 H12 O3 N2 F6 ←
	406.0773	-4.05	14.5	C20 H10 N3 F6

NVR-121 HRMS

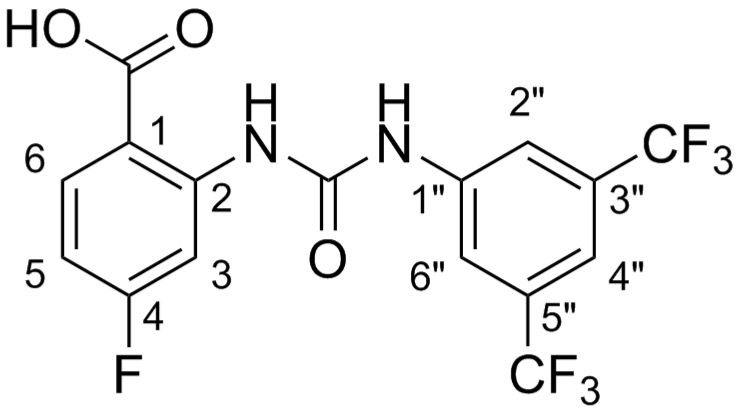

No.	Shift (ppm)	H	m	J (Hz)	Assign
1	13.67	1	br s	-	COOH
2	10.91	1	s	-	NH
3	10.67	1	s	-	NH"
4	8.29	1	dd	12.7, 2.6	3
5	8.20	2	br s	-	6", 2"
6	8.06	1	dd	8.9, 6.9	6
7	7.69	1	br s	-	4"
8	6.93	1	ddd	8.8, 7.8, 2.6	5

Acquisition Time (sec)	3.9846
Date	29 Mar 2019 02:28:25
Date Stamp	29 Mar 2019 02:28:25
Frequency (MHz)	400.0700
Nucleus	1H
Number of Transients	16
Solvent	DMSO-d6
Temperature (degree C)	25.000

^{1}H NMR (400 MHz, *DMSO-d_6*) δ ppm 13.67 (br s, 1 H), 10.91 (s, 1 H), 10.67 (s, 1 H), 8.29 (dd, *J*=12.7, 2.6 Hz, 1 H), 8.20 (br s, 2 H), 8.06 (dd, *J*=8.9, 6.9 Hz, 1 H), 7.69 (br s, 1 H), 6.93 (ddd, *J*=8.8, 7.8, 2.6 Hz, 1 H)

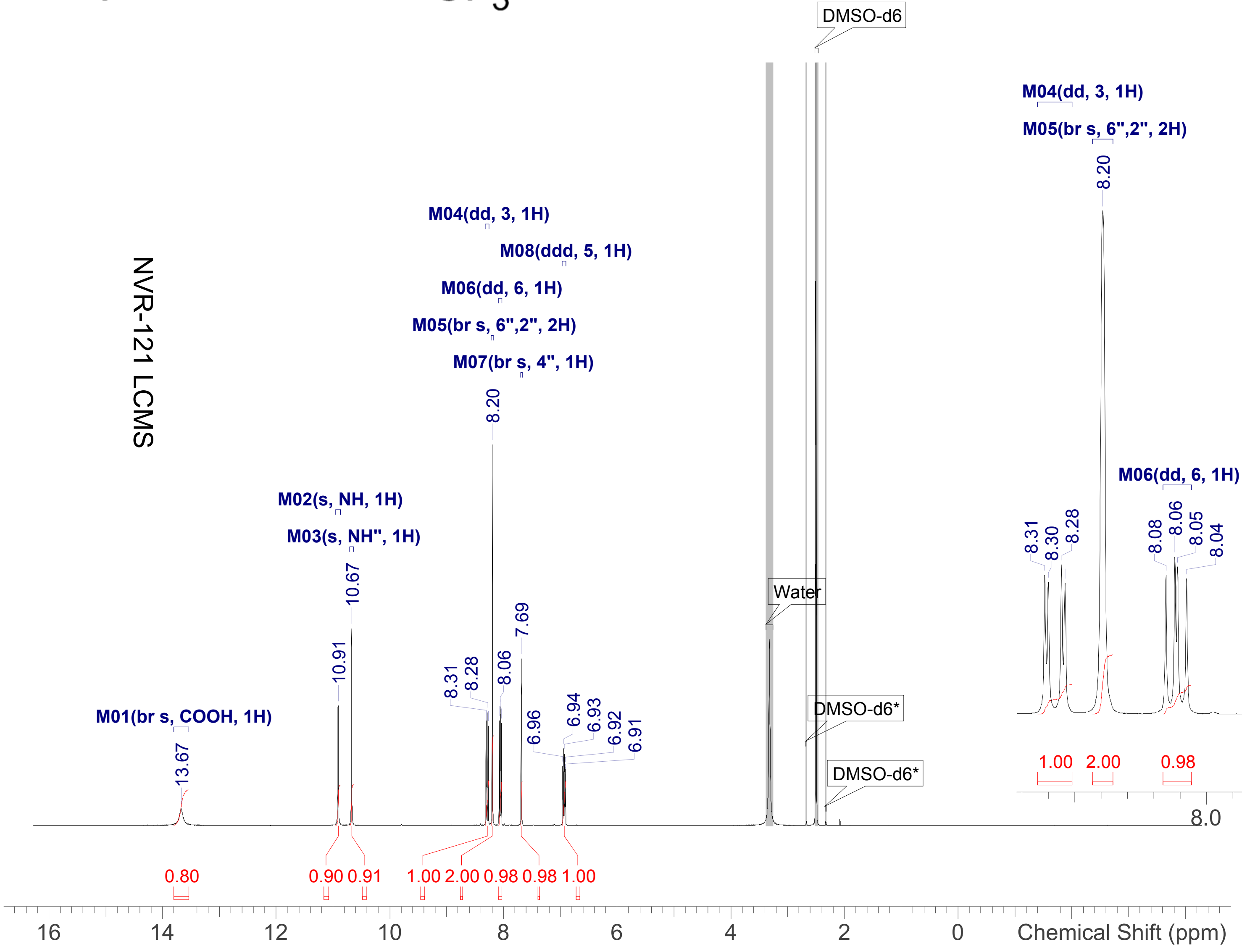

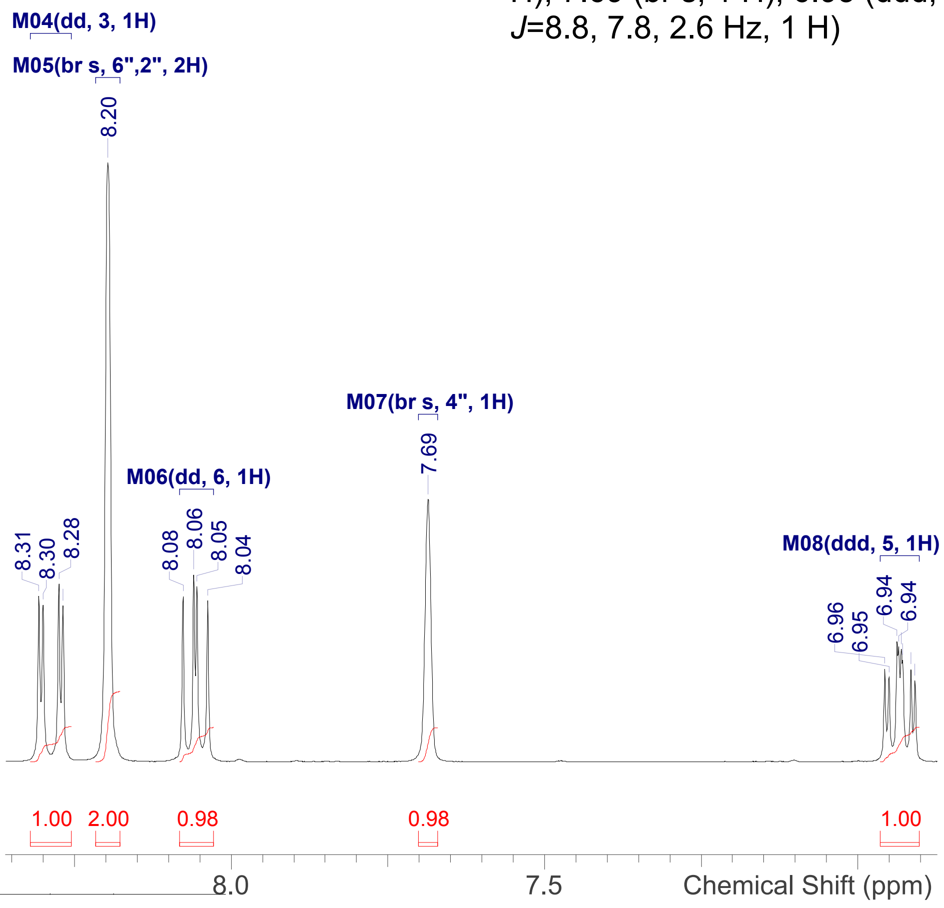

NVR-120_1H.spectrus

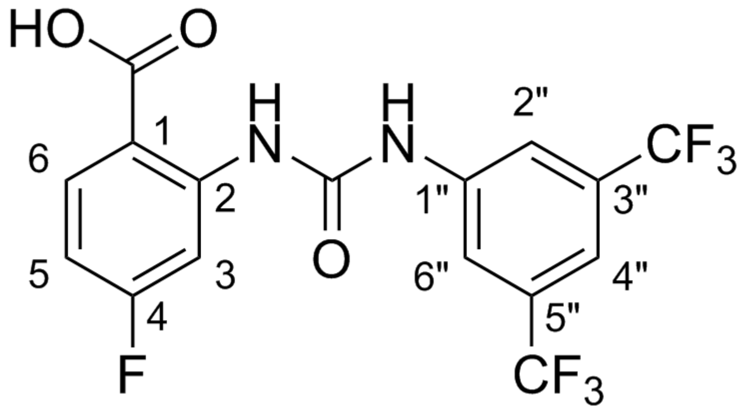

Shift (ppm)	C	m	J (Hz)	Assign
168.9	1	s	-	COOH
165.0	1	d	248.4	4
152.0	1	s	-	CO
144.0	1	d	12.7	2
141.6	1	s	-	1"
133.8	1	d	11.7	6
130.7	2	q	33.3	5", 3"
123.3	2	q	272.9	3"-CF3, 5"-CF3
118.1	2	br q	3.9	6", 2"
114.8	1	br spt	3.9	4"
111.8	1	d	2.9	1
108.5	1	d	22.5	5
105.9	1	d	28.4	3

Acquisition Time (sec)	1.0224
Date	10 May 2019 18:21:52
Date Stamp	10 May 2019 18:21:52
Frequency (MHz)	100.5977
Nucleus	13C
Number of Transients	256
Solvent	DMSO-d6
Temperature (degree C)	24.999

^{13}C NMR (101 MHz, *DMSO-d_6*) δ ppm 168.9 (s, 1 C), 165.0 (d, *J*=248.4 Hz, 1 C), 152.0 (s, 1 C), 144.0 (d, *J*=12.7 Hz, 1 C), 141.6 (s, 1 C), 133.8 (d, *J*=11.7 Hz, 1 C), 130.7 (q, *J*=33.3 Hz, 2 C), 123.3 (q, *J*=272.9 Hz, 2 C), 118.1 (br q, *J*=3.9 Hz, 2 C), 114.8 (br spt, *J*=3.9 Hz, 1 C), 111.8 (d, *J*=2.9 Hz, 1 C), 108.5 (d, *J*=22.5 Hz, 1 C), 105.9 (d, *J*=28.4 Hz, 1 C)

NVR-120_13C

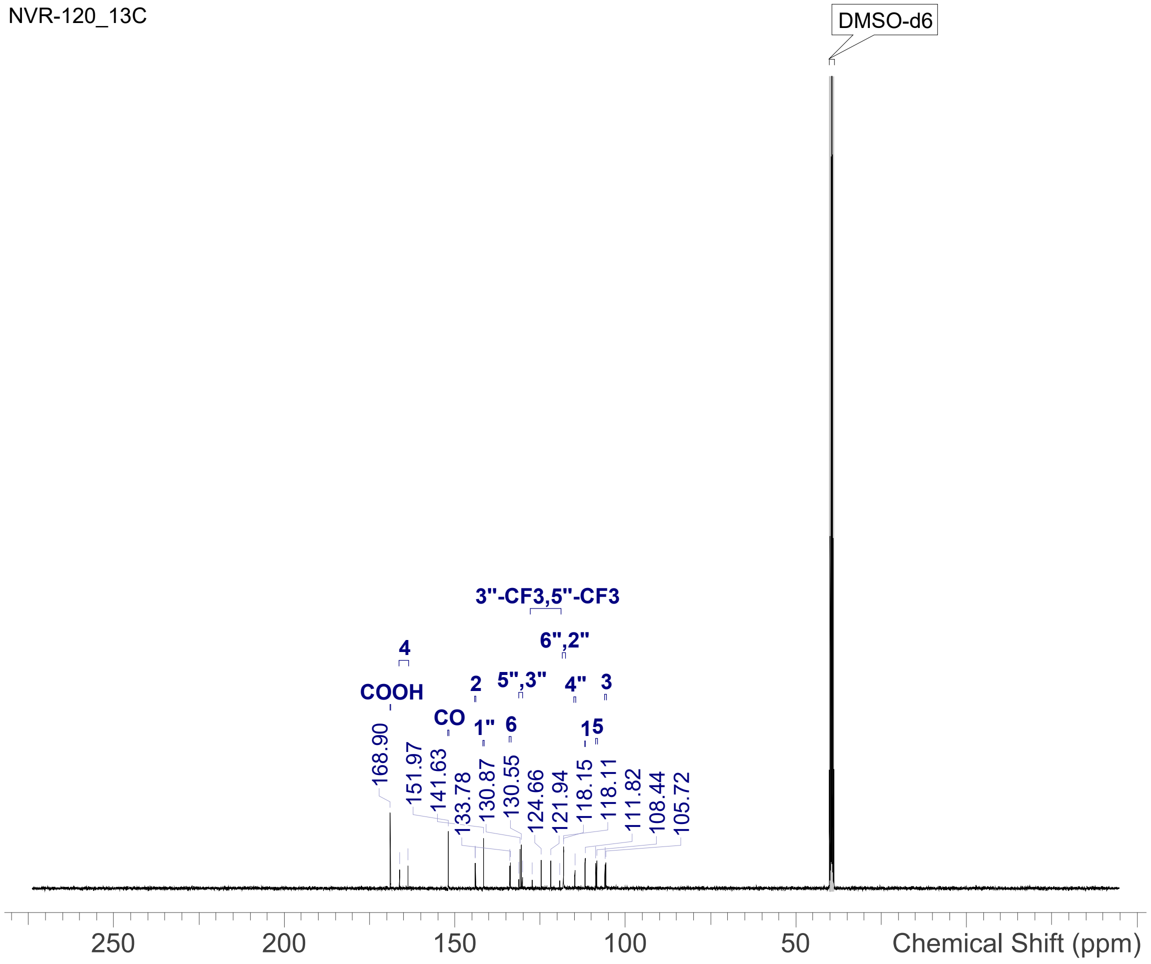

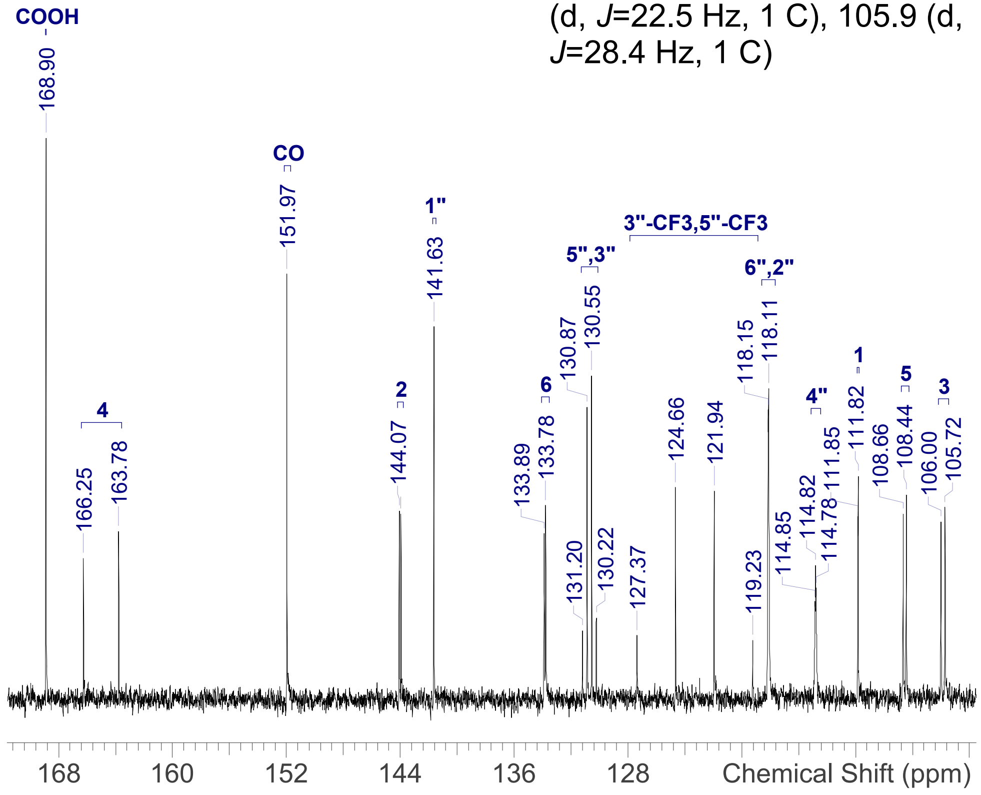

NVR-120_13C.spectrus

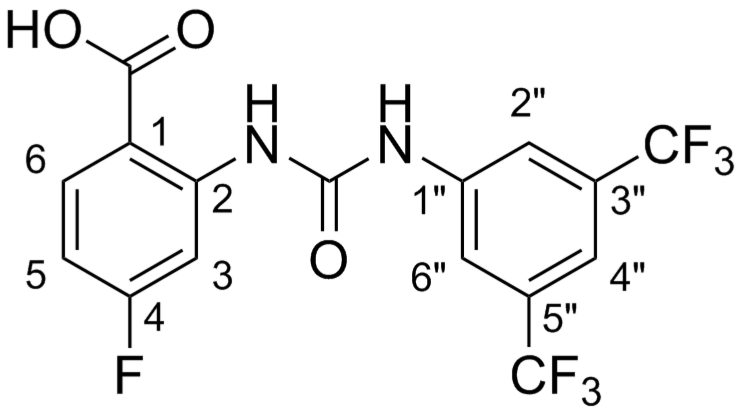

Shift (ppm)	F	m	J (Hz)
-61.71	6	s	-
-103.34	1	ddd	12.7, 7.8, 6.9

Acquisition Time (sec)	1.4680
Date	29 Mar 2019 03:49:20
Date Stamp	29 Mar 2019 03:49:20
Frequency (MHz)	376.4419
Nucleus	19F
Number of Transients	16
Solvent	DMSO-d6
Temperature (degree C)	25.001

^{19}F NMR (376 MHz, *DMSO-d_6*) δ ppm -61.71 (s, 6 F), -103.34 (ddd, *J*=12.7, 7.8, 6.9 Hz, 1 F)

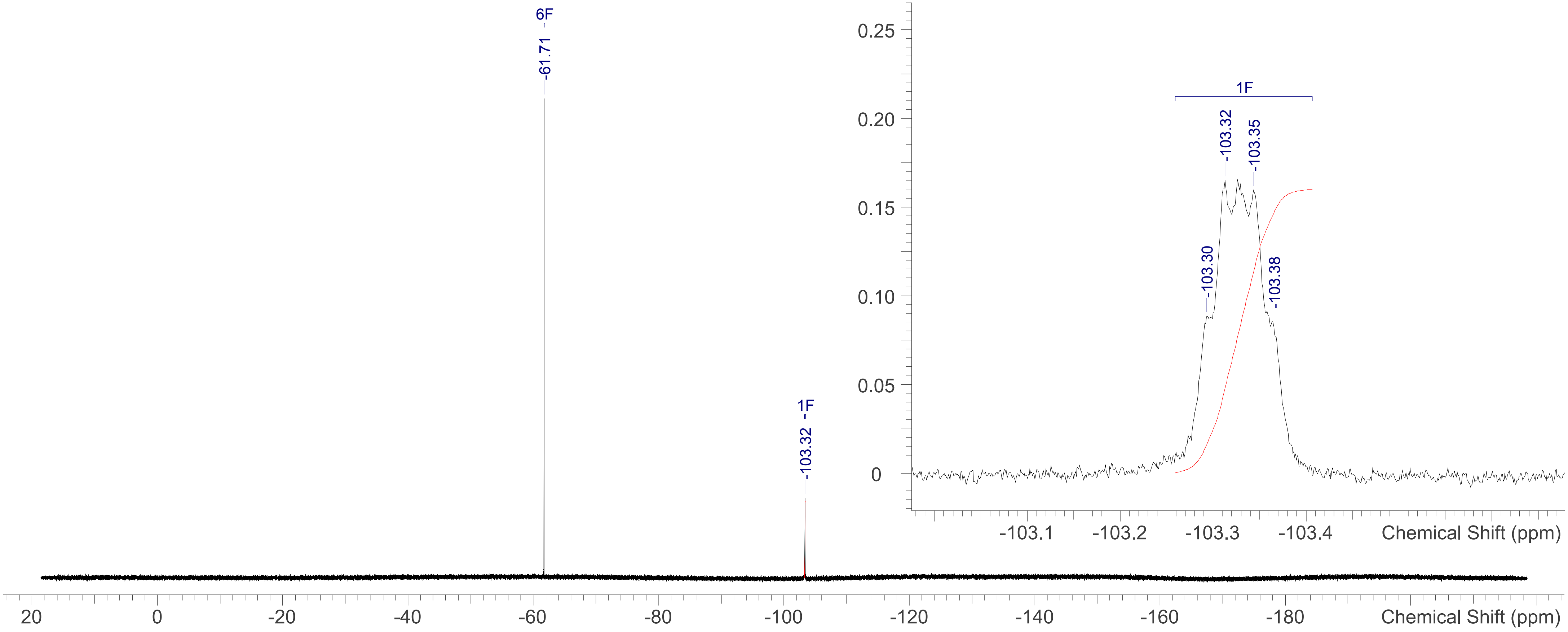

NVR-120_19F.spectrus

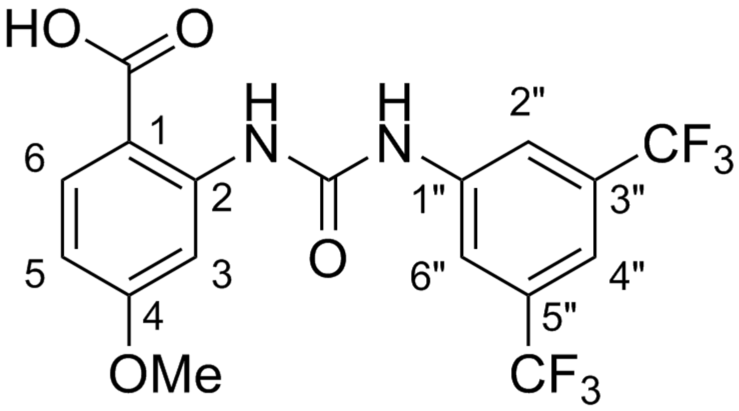

No.	Shift (ppm)	H	m	J (Hz)	Assign
1	13.20	1	br s	-	COOH
2	10.92	1	s	-	NH
3	10.56	1	s	-	NH"
4	8.21	2	br s	-	6", 2"
5	8.09	1	d	2.5	3
6	7.93	1	d	9.0	6
7	7.66	1	br s	-	4"
8	6.66	1	dd	8.9, 2.6	5
9	3.83	3	s	-	OMe

Acquisition Time (sec)	3.9846
Date	03 Apr 2019 07:57:45
Date Stamp	03 Apr 2019 07:57:45
Frequency (MHz)	400.0700
Nucleus	1H
Number of Transients	16
Solvent	DMSO-d6
Temperature (degree C)	25.000

^{1}H NMR (400 MHz, *DMSO-d_6*) δ ppm 13.20 (br s, 1 H), 10.92 (s, 1 H), 10.56 (s, 1 H), 8.21 (br s, 2 H), 8.09 (d, *J*=2.5 Hz, 1 H), 7.93 (d, *J*=9.0 Hz, 1 H), 7.66 (br s, 1 H), 6.66 (dd, *J*=8.9, 2.6 Hz, 1 H), 3.83 (s, 3 H)

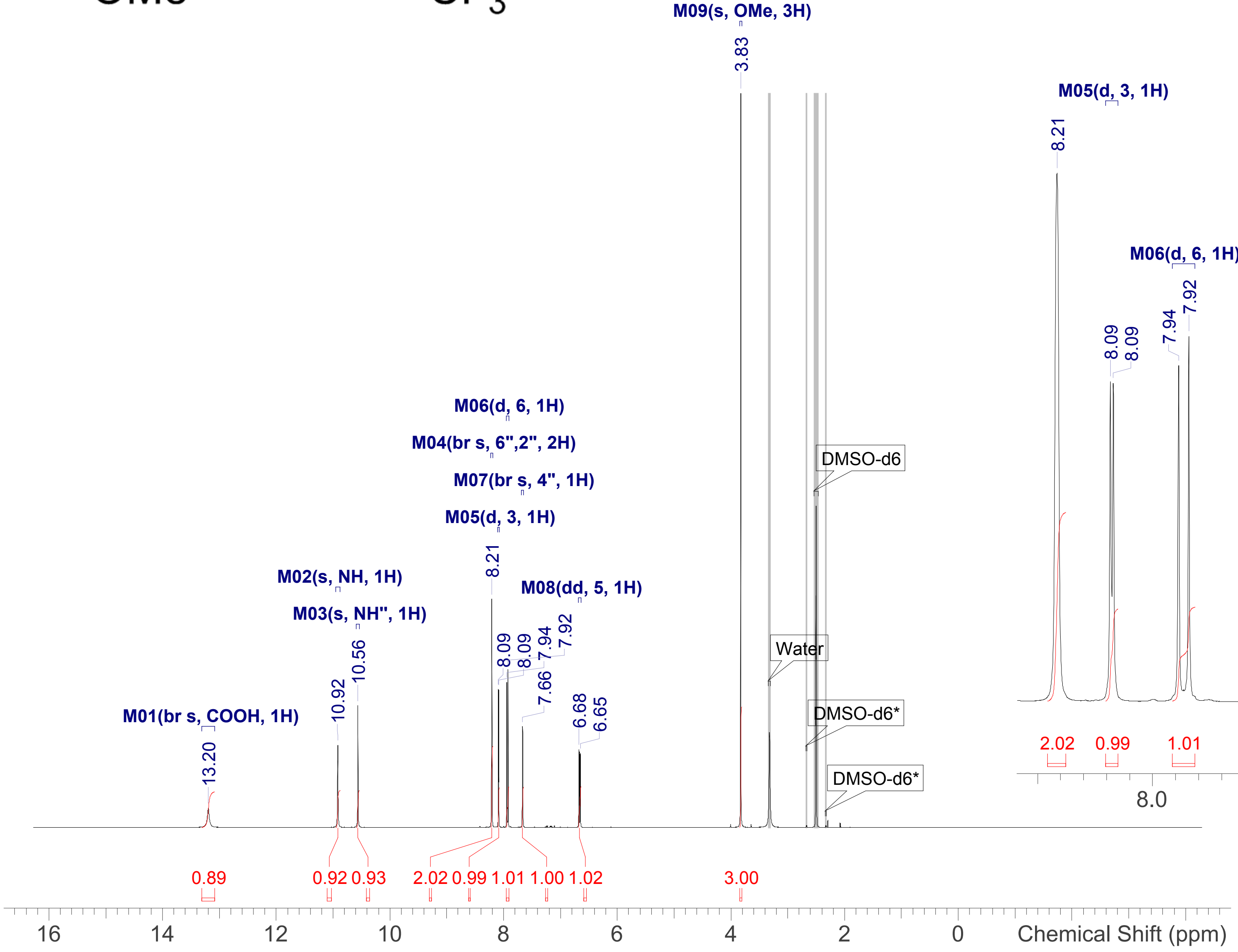

NVR-123_1H.spectrus

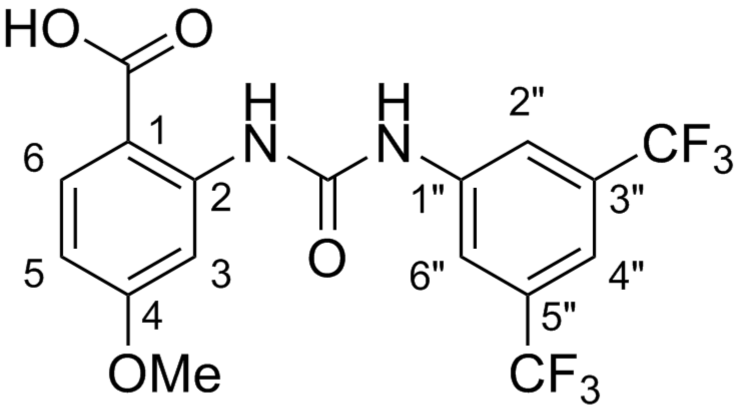

Shift (ppm)	C	m	J (Hz)	Assign
169.5	1	s	-	COOH
163.6	1	s	-	4
152.1	1	s	-	CO
143.8	1	s	-	2
141.9	1	s	-	1"
133.0	1	s	-	6
130.7	2	q	32.3	3", 5"
123.3	2	q	272.6	3"-CF3, 5"-CF3
118.1	2	br q	3.9	2", 6"
114.6	1	br spt	3.9	4"
107.8	1	s	-	1, 5
103.8	1	s	-	3
55.4	1	s	-	OMe

Acquisition Time (sec)	1.0224
Date	11 May 2019 01:47:39
Date Stamp	11 May 2019 01:47:39
Frequency (MHz)	100.5977
Nucleus	13C
Number of Transients	256
Solvent	DMSO-d6
Temperature (degree C)	24.999

^{13}C NMR (101 MHz, *DMSO-d$_6$*) δ ppm 169.5 (s, 1 C), 163.6 (s, 1 C), 152.1 (s, 1 C), 143.8 (s, 1 C), 141.9 (s, 1 C), 133.0 (s, 1 C), 130.7 (q, *J*=32.3 Hz, 2 C), 123.3 (q, *J*=272.6 Hz, 2 C), 118.1 (br q, *J*=3.9 Hz, 2 C), 114.6 (br spt, *J*=3.9 Hz, 1 C), 107.8 (s, 1 C), 103.8 (s, 1 C), 55.4 (s, 1 C)

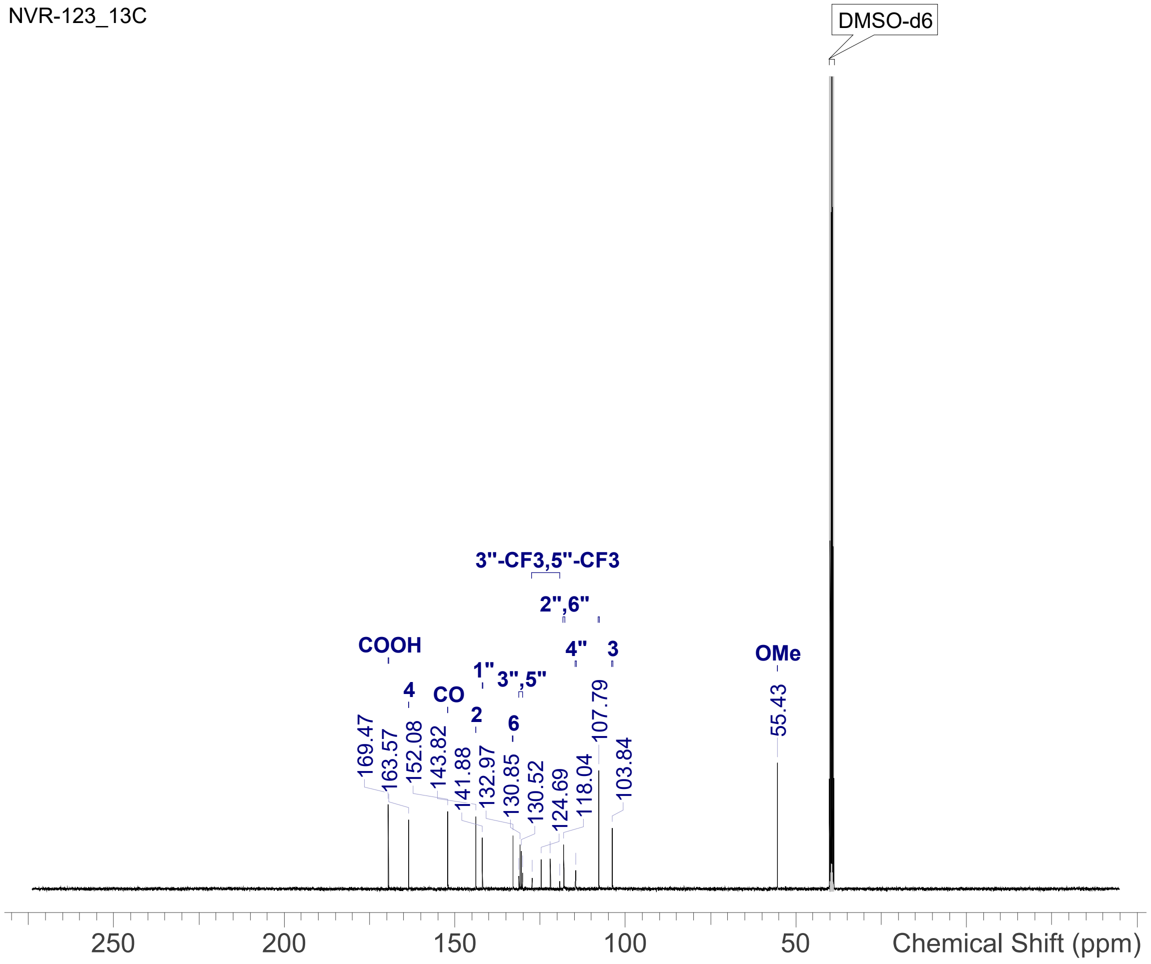

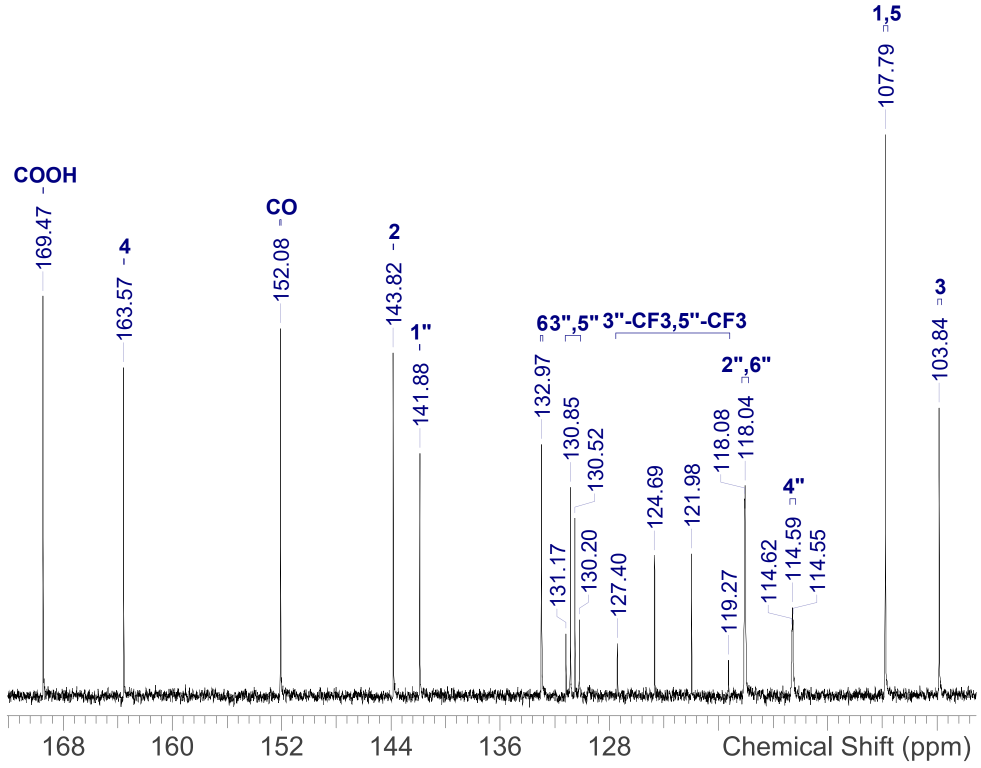

NVR-123_13C.spectrus

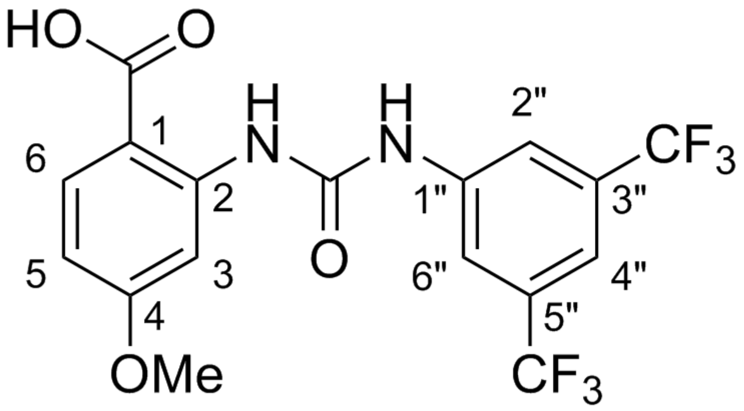

Shift (ppm)	F	m
-61.68	6	s

Acquisition Time (sec)	1.4680
Date	02 Apr 2019 17:47:58
Date Stamp	02 Apr 2019 17:47:58
Frequency (MHz)	376.4419
Nucleus	19F
Number of Transients	16
Solvent	DMSO-d6
Temperature (degree C)	25.002

^{19}F NMR (376 MHz, *DMSO-d_6*) δ ppm -61.68 (s, 6 F)

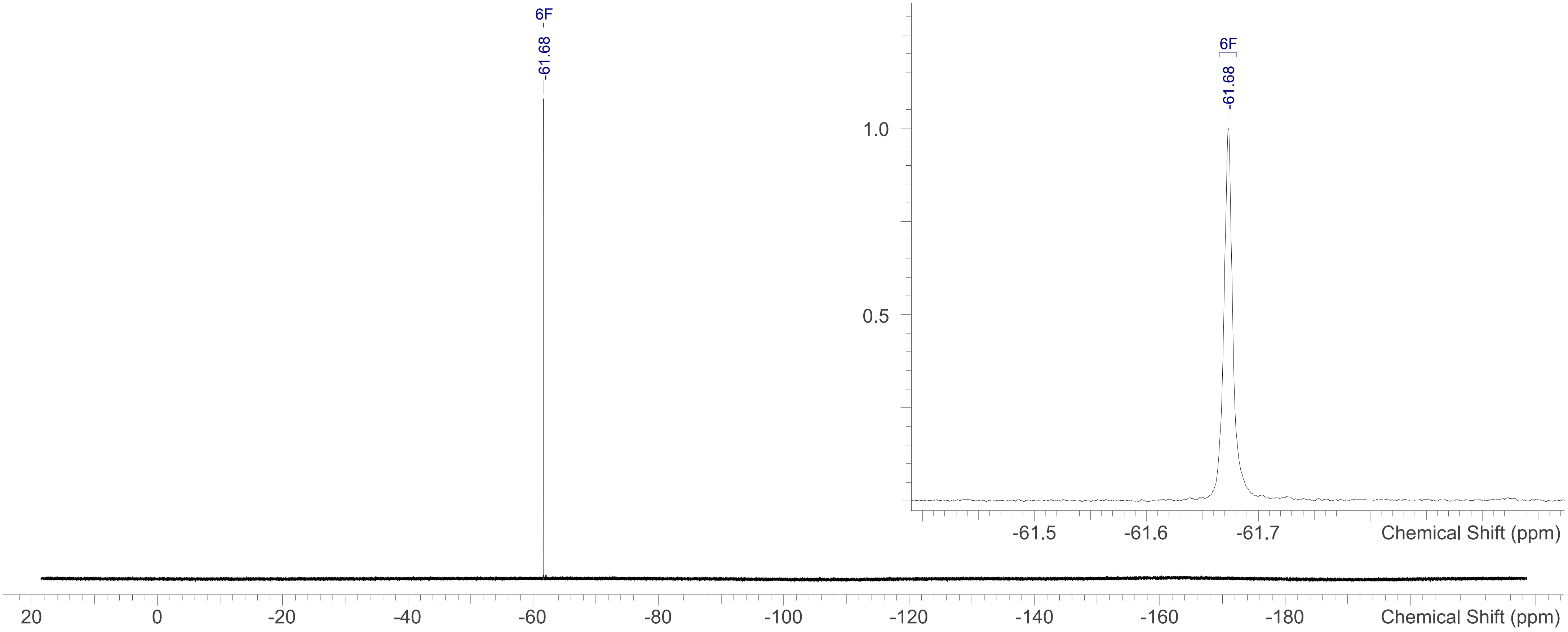

NVR-123_19F.spectrus

Openlynx Report - James B Page 1

Sample: 1	Vial:2:3	ID:JB154-1
File:JB154-1	Date:01-Apr-2019	Time:16:06:20
Description:		

Printed: Tue Apr 02 15:23:08 2019

3: UV Detector: TAC: Wavelength Range: (210 - 400) 3.047e+2
Range: 3.171e+2

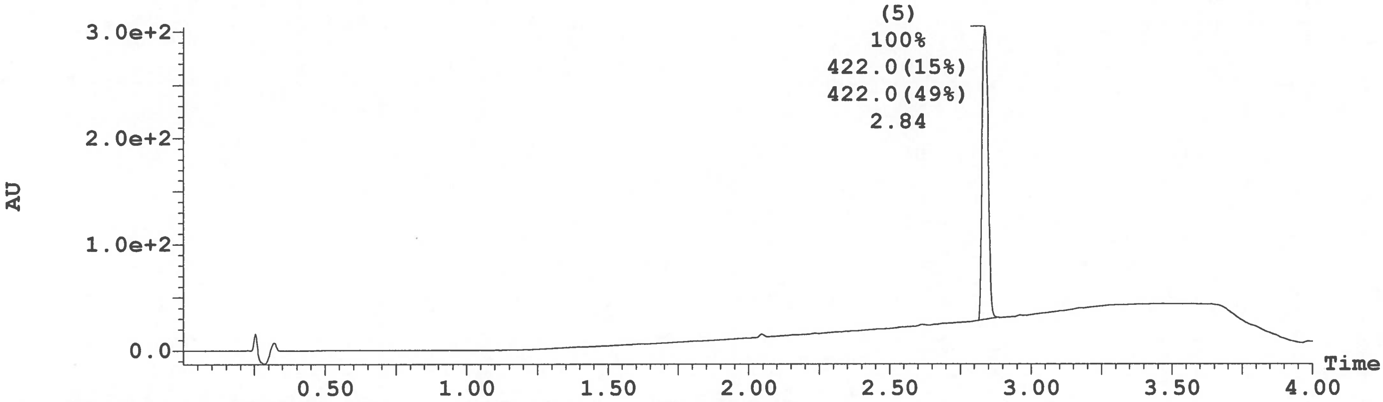

Peak Number	Compound	Time	Area %Total	Mass Found
5	Tentative	2.84	100.00	422.0, 422.0

1: MS ES+ :TIC Smooth (Mn, 2x2) 1.7e+007

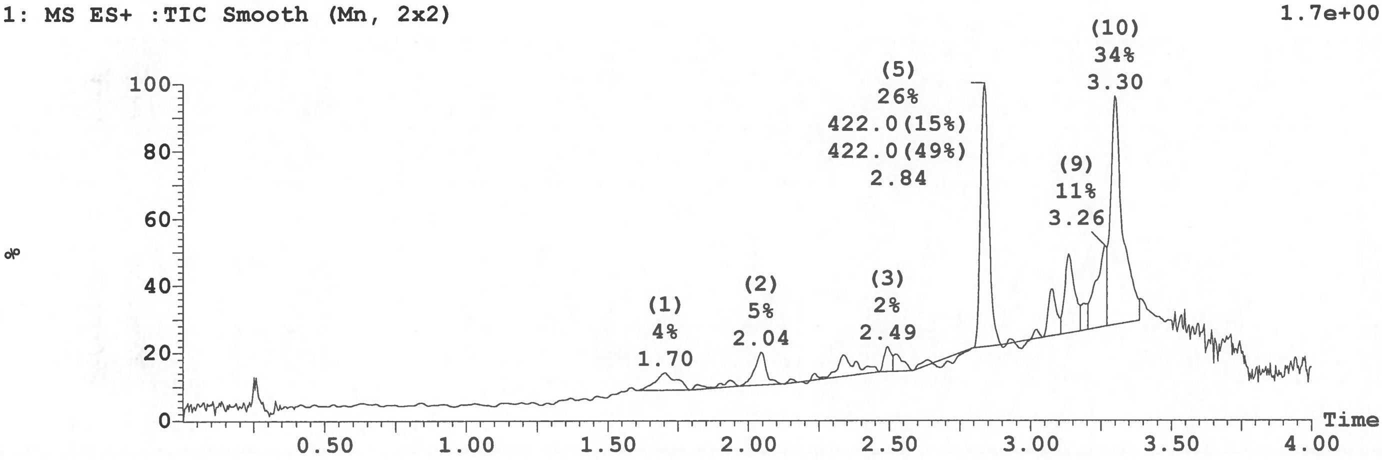

2: MS ES- :TIC Smooth (Mn, 2x2) 1.1e+007

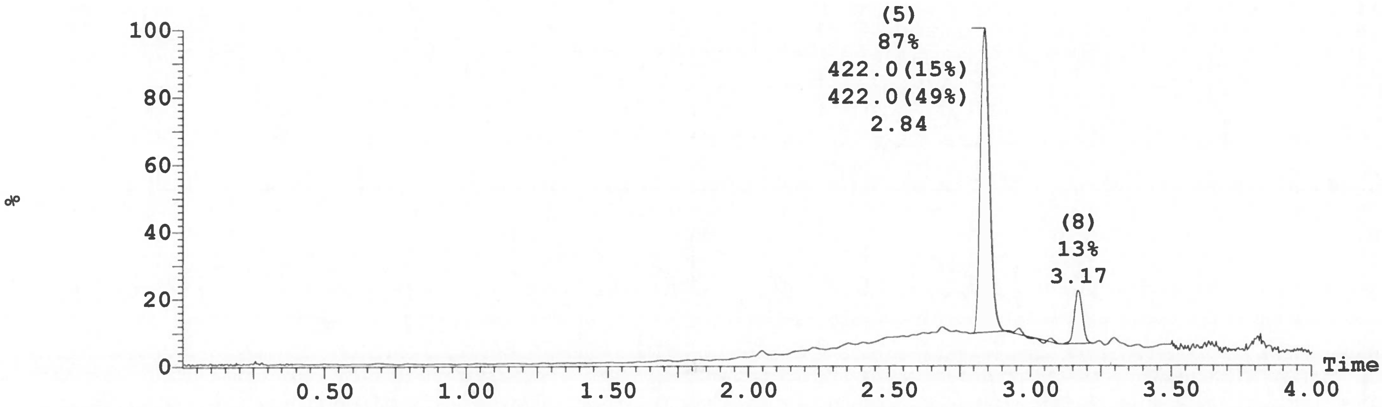

NVR-123 LCMS

Sample: 1	Vial:2:3	ID:JB154-1
File:JB154-1	Date:01-Apr-2019	Time:16:06:20
Description:		

Printed: Tue Apr 02 15:23:08 2019

Peak ID	Compound	Time	Mass Found
5	Tentative	2.84	423

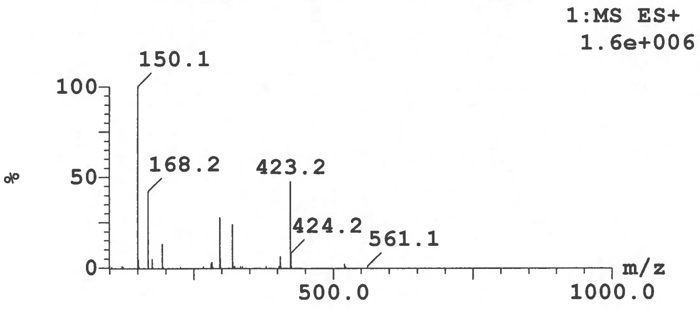

Peak ID	Compound	Time	Mass Found
5	Found	2.84	421

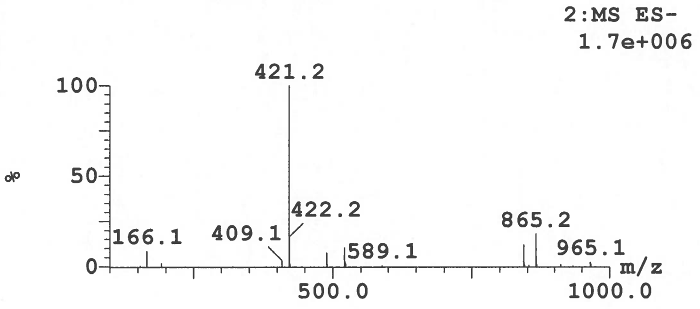

Peak ID	Compound	Time	Mass Found
5		2.84	Not Found

5: (Time: 2.84) Combine (1702)

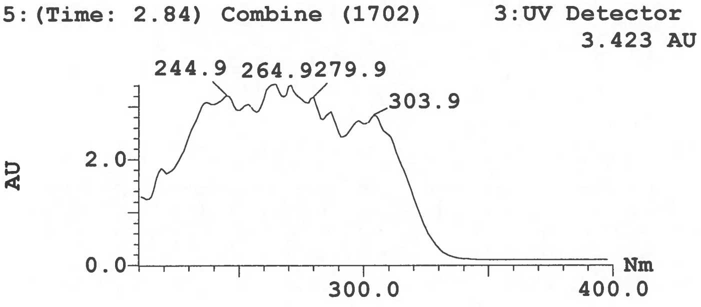

Thermo Exactive Plus EMR Orbitrab ASAP pos

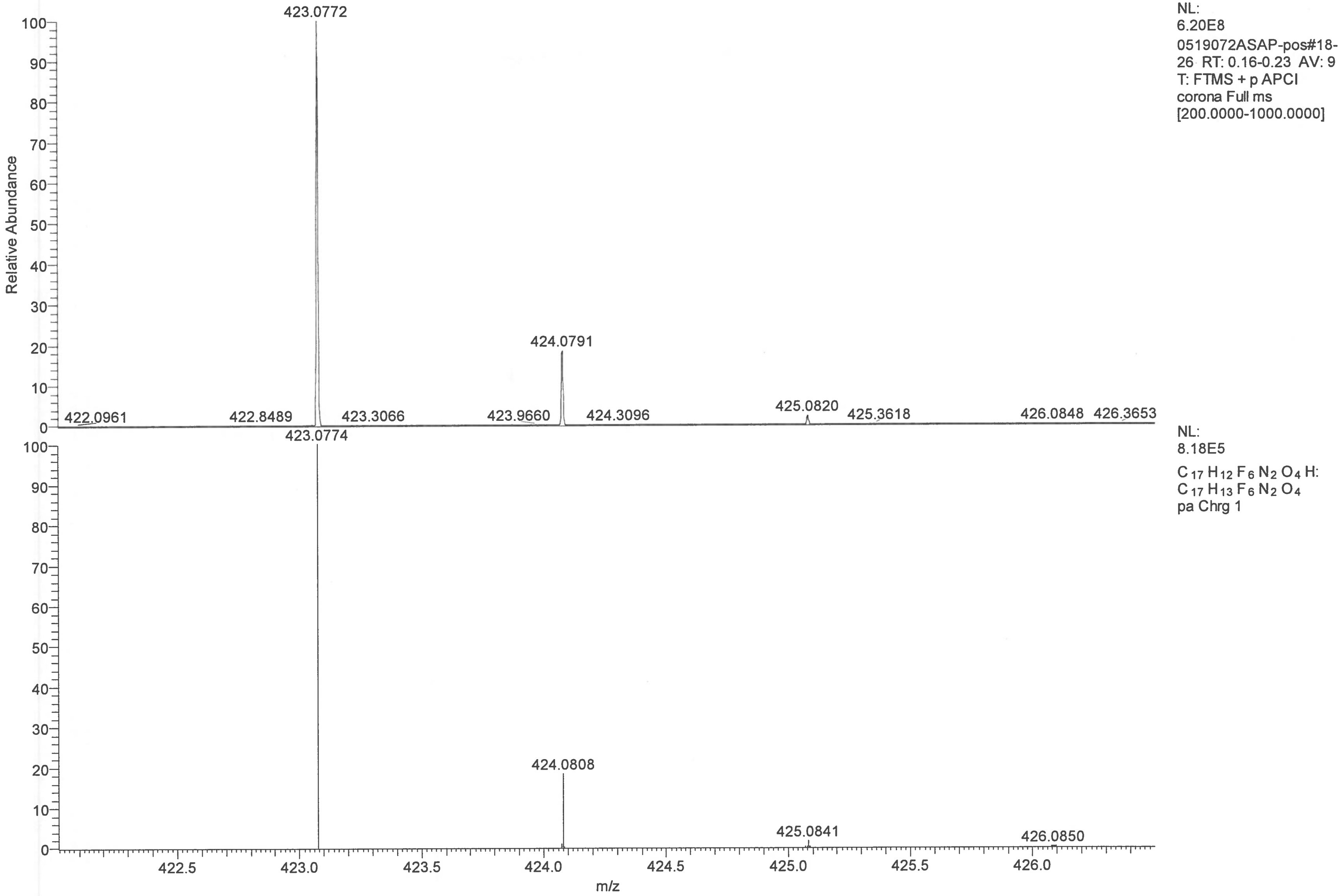

Type text here

Thermo Exactive Plus EMR Orbitrab ASAP pos

m/z	Theo. Mass	Delta (ppm)	RDB equiv.	Composition
423.0772	423.0774	-0.48	9.5	C17 H13 O4 N2 F6 ←
	423.0761	2.69	10.0	C15 H11 O3 N5 F6
	423.0787	-3.64	14.5	C18 H9 N6 F6

NVR-123 HRMS

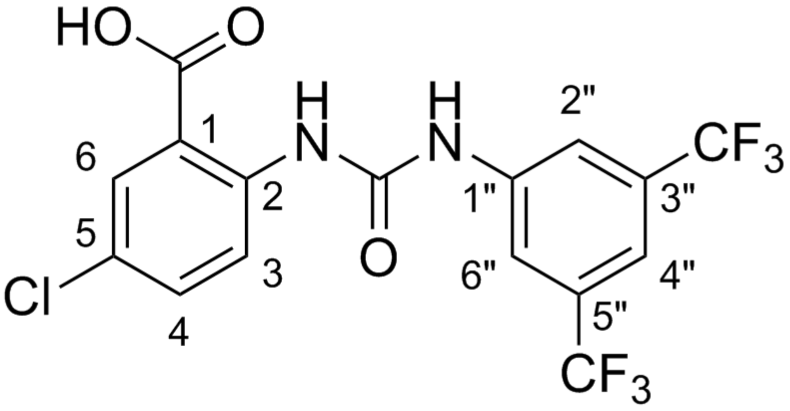

No.	Shift (ppm)	H	m	J (Hz)	Assign
1	13.89	1	br s	-	COOH
2	10.59	1	s	-	NH
3	10.56	1	s	-	NH"
4	8.45	1	d	9.0	3
5	8.16	2	br s	-	6", 2"
6	7.88	1	d	2.8	6
7	7.61	1	br s	-	4"
8	7.58	1	dd	9.2, 2.6	4

Acquisition Time (sec)	3.9846
Date	11 May 2019 03:38:27
Date Stamp	11 May 2019 03:38:27
Frequency (MHz)	400.0700
Nucleus	1H
Number of Transients	16
Solvent	DMSO-d6
Temperature (degree C)	25.000

^{1}H NMR (400 MHz, *DMSO-d_6*) δ ppm 13.89 (br s, 1 H), 10.59 (s, 1 H), 10.56 (s, 1 H), 8.45 (d, *J*=9.0 Hz, 1 H), 8.16 (br s, 2 H), 7.88 (d, *J*=2.8 Hz, 1 H), 7.61 (br s, 1 H), 7.58 (dd, *J*=9.2, 2.6 Hz, 1 H)

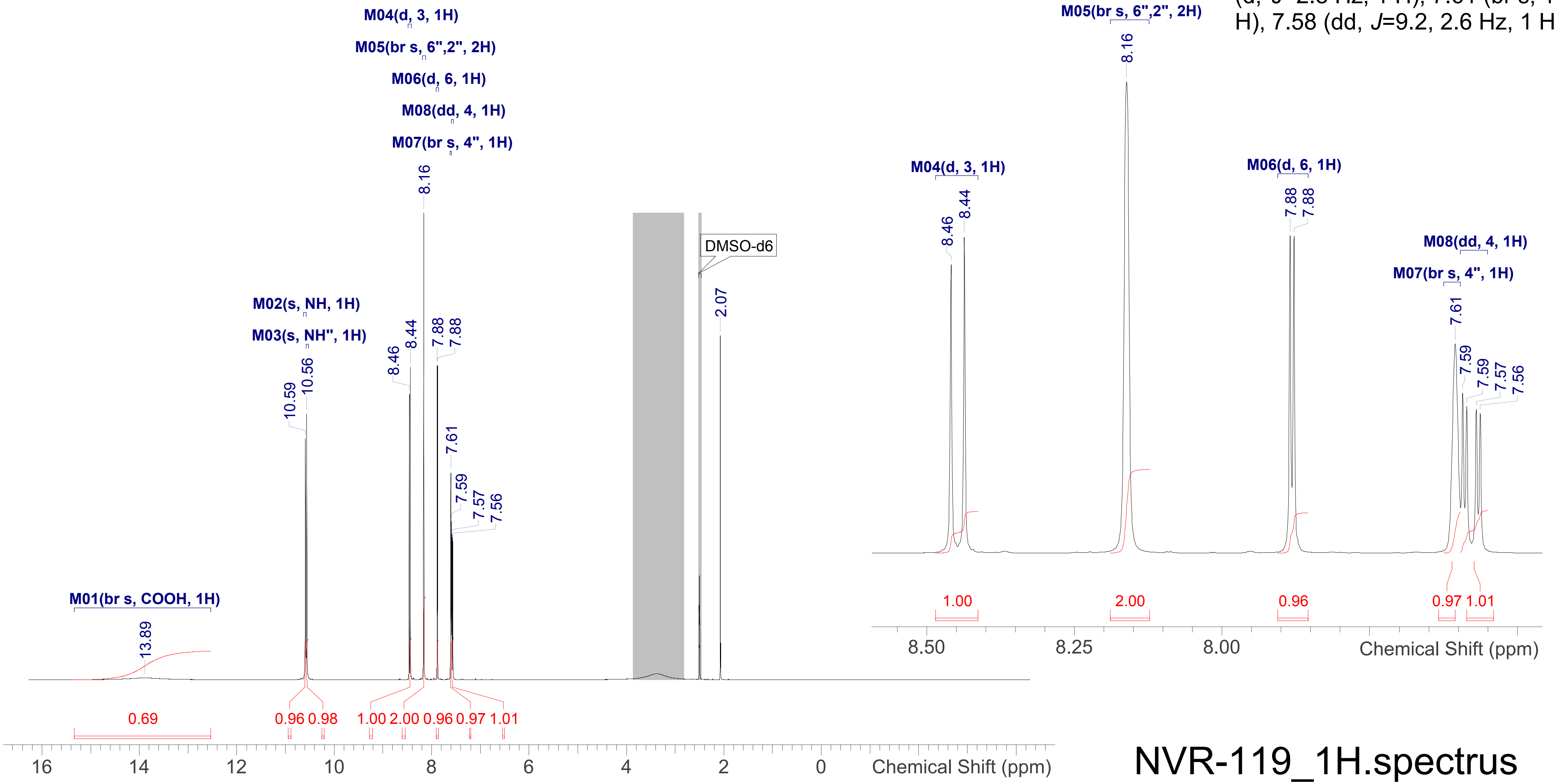

NVR-119_1H.spectrus

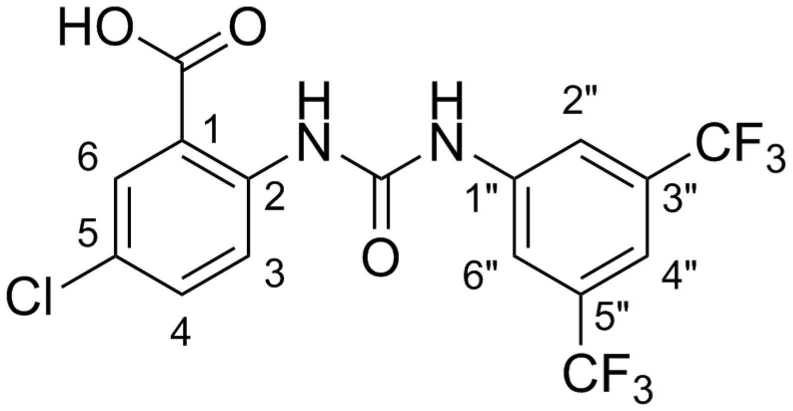

Shift (ppm)	C	m	J (Hz)	Assign
168.4	1	s	-	COOH
151.9	1	s	-	CO
141.8	1	s	-	1"
140.5	1	s	-	2
133.5	1	s	-	4
130.7	2	q	32.3	5", 3"
130.1	1	s	-	6
125.1	1	s	-	5
123.3	2	q	272.9	5"-CF3, 3"-CF3
121.5	1	s	-	3
118.0	2	br q	3.9	6", 2"
117.2	1	s	-	1
114.6	1	br spt	3.9	4"

Acquisition Time (sec)	1.0224
Date	11 May 2019 03:52:48
Date Stamp	11 May 2019 03:52:48
Frequency (MHz)	100.5977
Nucleus	13C
Number of Transients	256
Solvent	DMSO-d6
Temperature (degree C)	25.001

^{13}C NMR (101 MHz, *DMSO-d$_6$*) δ ppm 168.4 (s, 1 C), 151.9 (s, 1 C), 141.8 (s, 1 C), 140.5 (s, 1 C), 133.5 (s, 1 C), 130.7 (q, *J*=32.3 Hz, 2 C), 130.1 (s, 1 C), 125.1 (s, 1 C), 121.5 (s, 1 C), 123.3 (q, *J*=272.9 Hz, 2 C), 118.0 (br q, *J*=3.9 Hz, 2 C), 117.2 (s, 1 C), 114.6 (br spt, *J*=3.9 Hz, 1 C)

NVR-119_13C

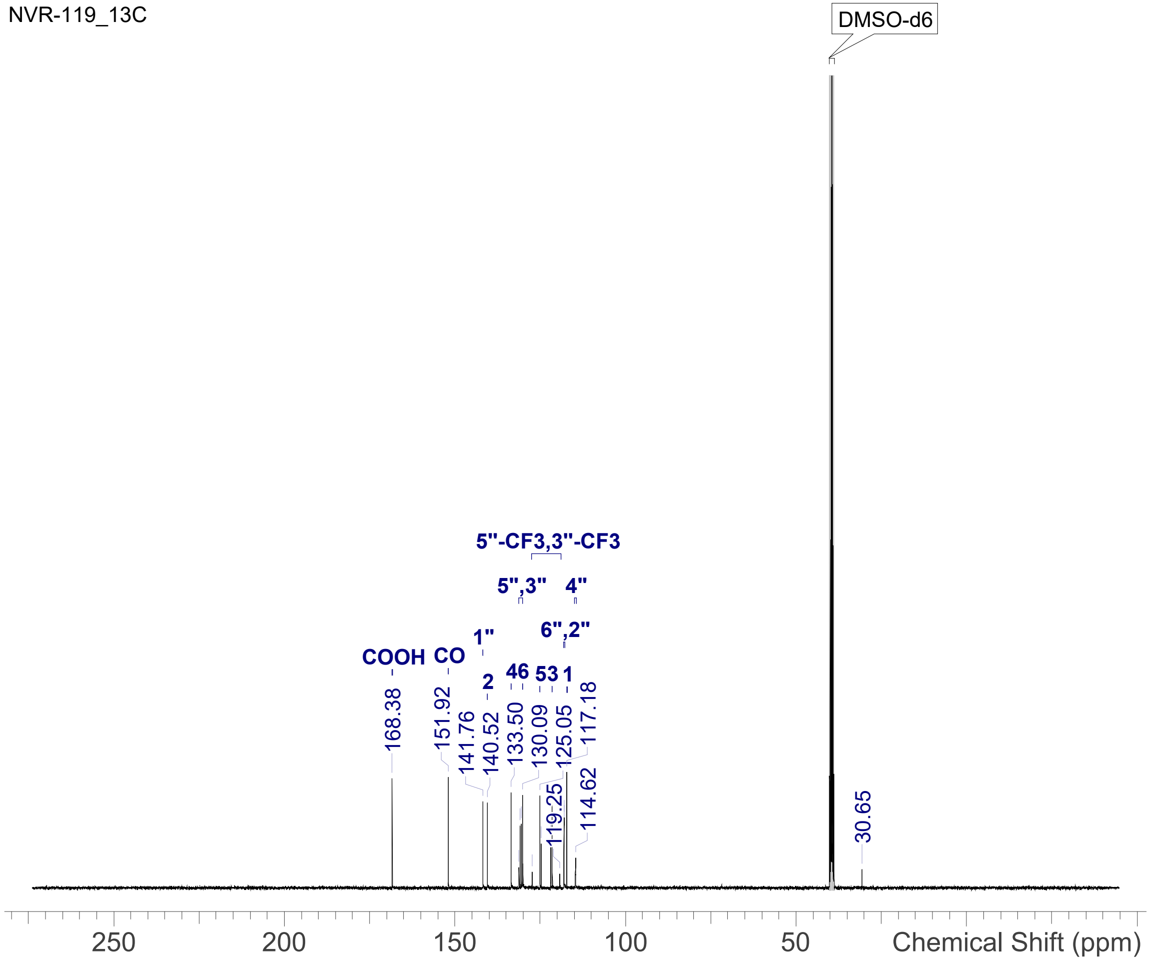

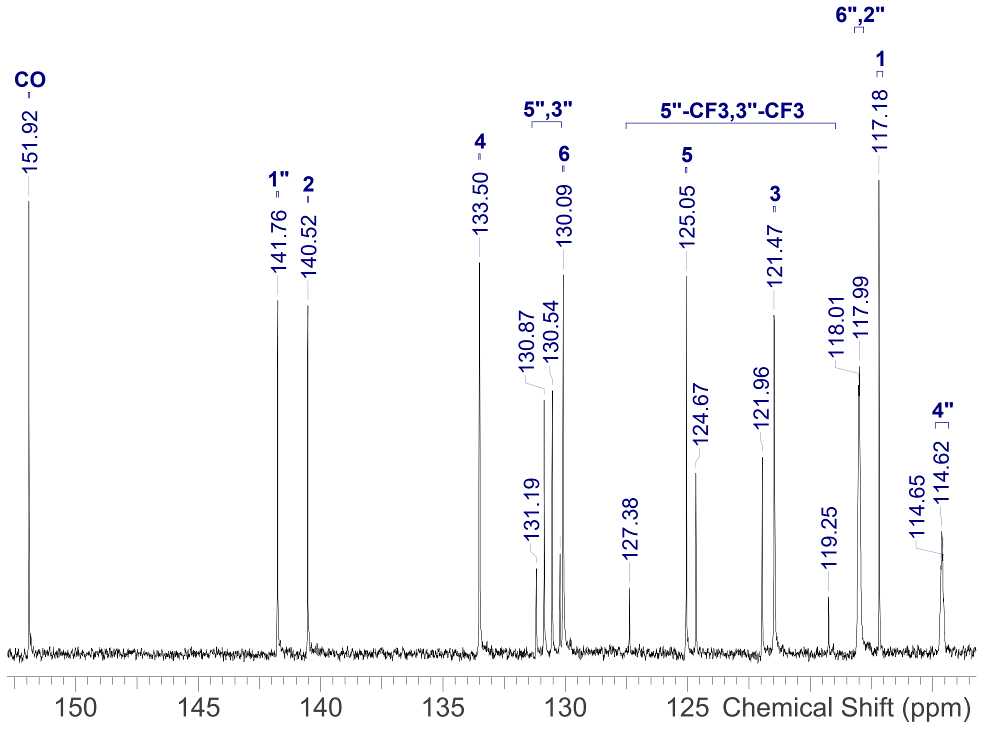

NVR-119_13C.spectrus

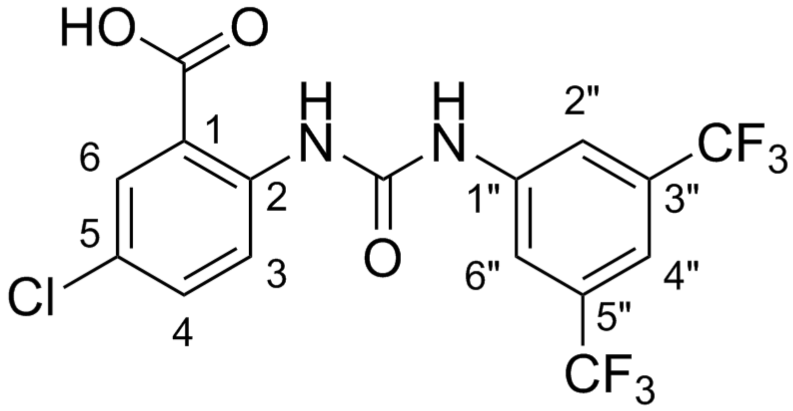

Shift (ppm)	F	m
-61.86	6	s

Acquisition Time (sec)	1.4680
Date	11 May 2019 04:58:51
Date Stamp	11 May 2019 04:58:51
Frequency (MHz)	376.4419
Nucleus	19F
Number of Transients	16
Solvent	DMSO-d6
Temperature (degree C)	25.000

^{19}F NMR (376 MHz, *DMSO-d_6*) δ ppm -61.86 (s, 6 F)

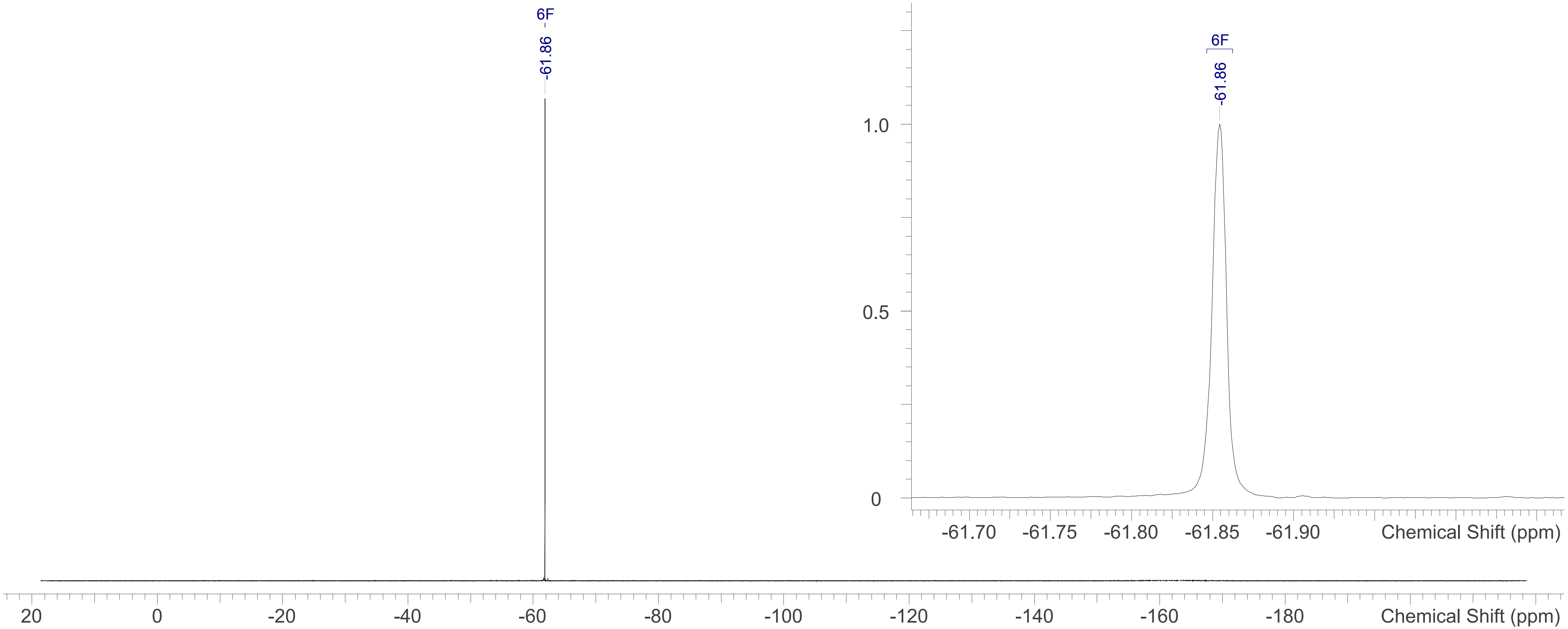

NVR-119_19F.spectrus

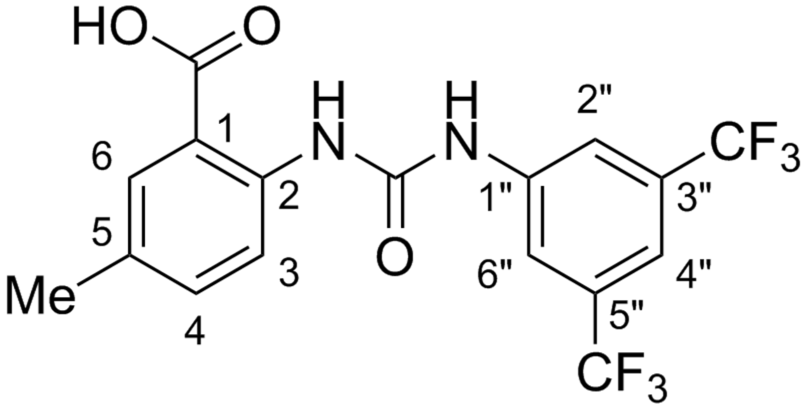

No.	Shift (ppm)	H	m	J (Hz)	Assign
1	13.48	1	br s	-	COOH
2	10.54	1	br s	-	NH
3	10.49	1	s	-	NH"
4	8.30	1	d	8.5	3
5	8.19	2	br s	-	6", 2"
6	7.79	1	d	2.0	6
7	7.65	1	br s	-	4"
8	7.39	1	dd	8.8, 2.0	4
9	2.29	3	s	-	Me

Acquisition Time (sec)	3.9846
Date	29 Mar 2019 04:06:50
Date Stamp	29 Mar 2019 04:06:50
Frequency (MHz)	400.0700
Nucleus	1H
Number of Transients	16
Solvent	DMSO-d6
Temperature (degree C)	25.000

^{1}H NMR (400 MHz, *DMSO-d_6*) δ ppm 13.48 (br s, 1 H), 10.54 (br s, 1 H), 10.49 (s, 1 H), 8.30 (d, *J*=8.5 Hz, 1 H), 8.19 (br s, 2 H), 7.79 (d, *J*=2.0 Hz, 1 H), 7.65 (br s, 1 H), 7.39 (dd, *J*=8.8, 2.0 Hz, 1 H), 2.29 (s, 3 H)

NVR-121

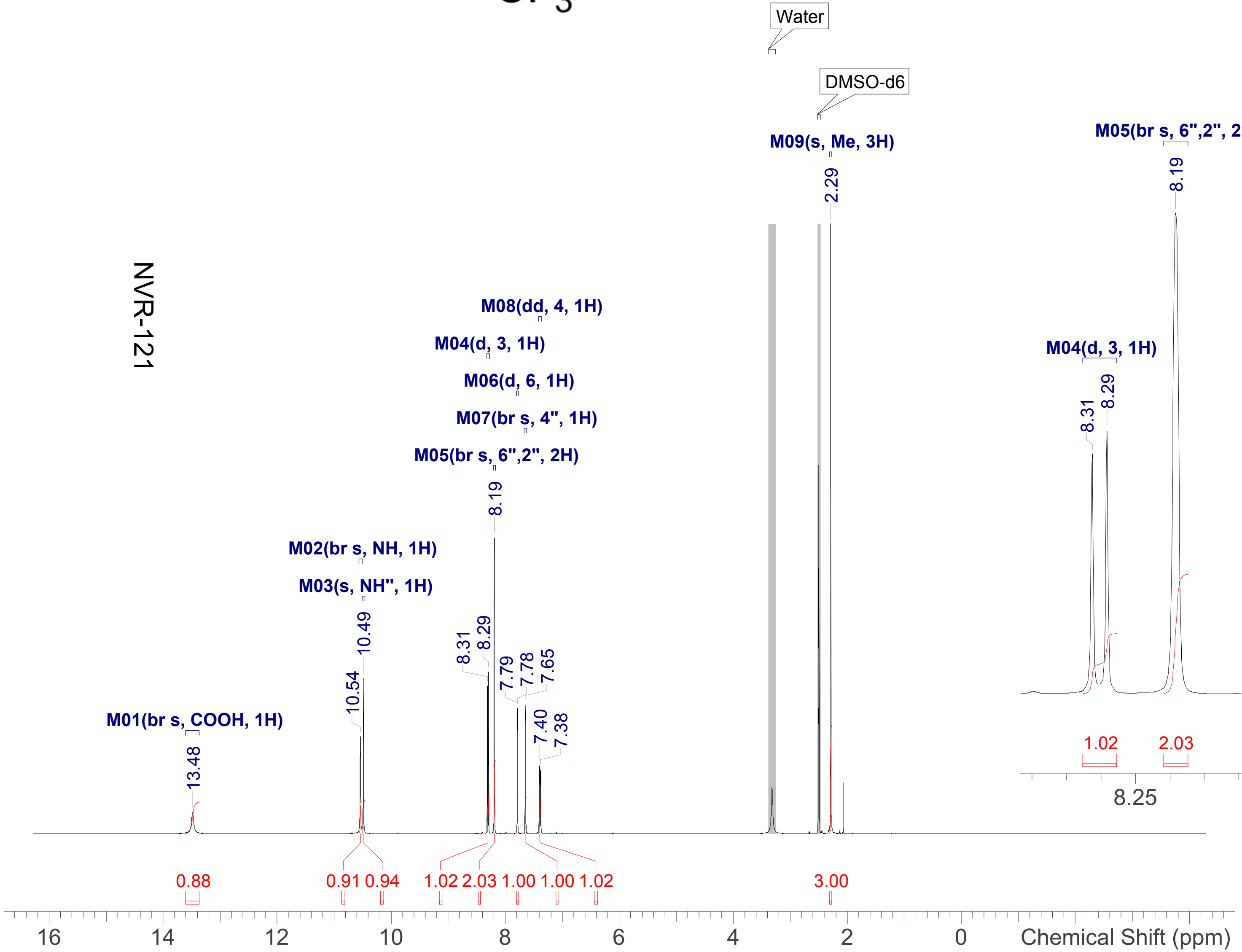

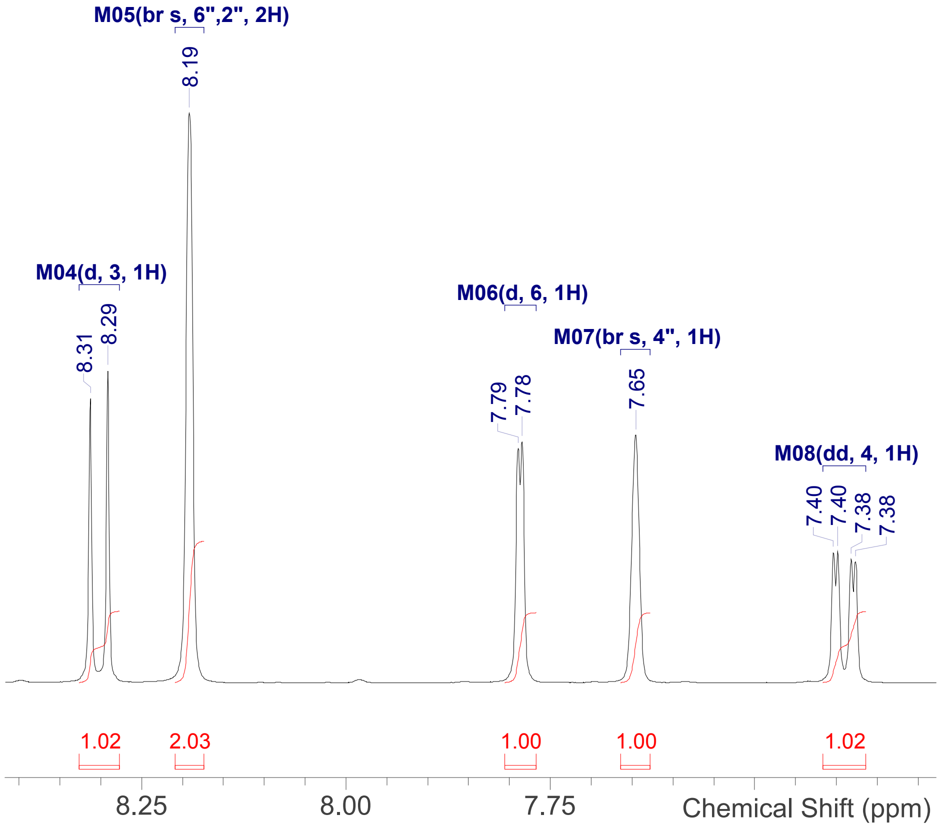

NVR-122_1H.spectrus

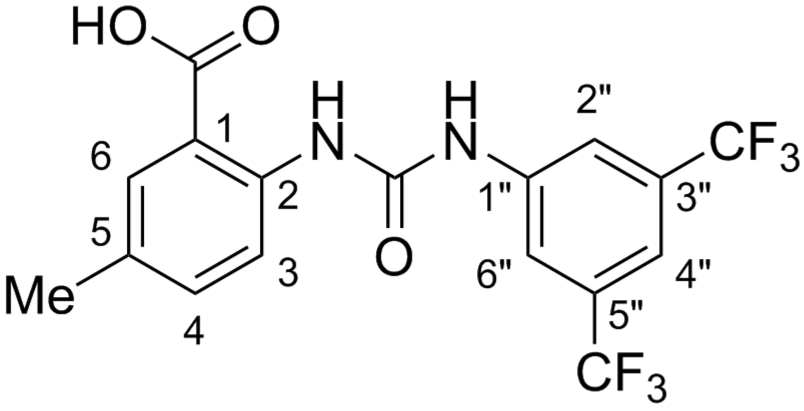

Shift (ppm)	C	m	J (Hz)	Assign
169.6	1	s	-	COOH
152.1	1	s	-	CO
142.0	1	s	-	1"
139.2	1	s	-	2
134.6	1	s	-	4
131.0	1	s	-	6
130.7	2	q	32.3	5", 3"
130.5	1	s	-	5
123.3	2	q	272.6	3"-CF3, 5"-CF3
119.7	1	s	-	3
117.9	2	br q	3.9	6", 2"
115.5	1	s	-	1
114.3	1	br spt	3.9	4"
20.0	1	s	-	Me

Acquisition Time (sec)	1.0224
Date	11 May 2019 01:04:15
Date Stamp	11 May 2019 01:04:15
Frequency (MHz)	100.5977
Nucleus	13C
Number of Transients	256
Solvent	DMSO-d6
Temperature (degree C)	25.001

^{13}C NMR (101 MHz, *DMSO-d*$_6$) δ ppm 169.6 (s, 1 C), 152.1 (s, 1 C), 142.0 (s, 1 C), 139.2 (s, 1 C), 134.6 (s, 1 C), 131.0 (s, 1 C), 130.5 (s, 1 C), 130.7 (q, *J*=32.3 Hz, 2 C), 119.7 (s, 1 C), 123.3 (q, *J*=272.6 Hz, 2 C), 117.9 (br q, *J*=3.9 Hz, 2 C), 115.5 (s, 1 C), 114.3 (br spt, *J*=3.9 Hz, 1 C), 20.0 (s, 1 C)

NVR-122_13C

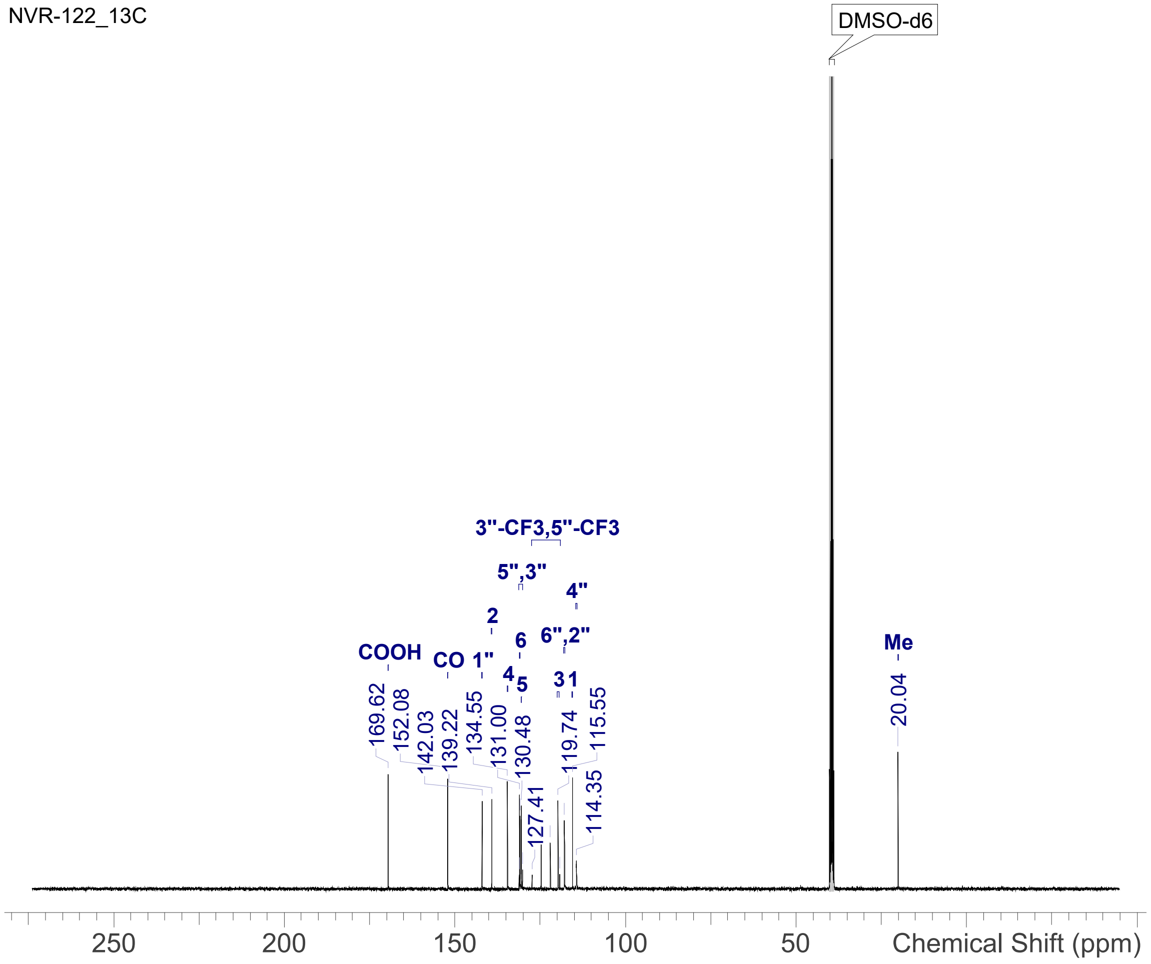

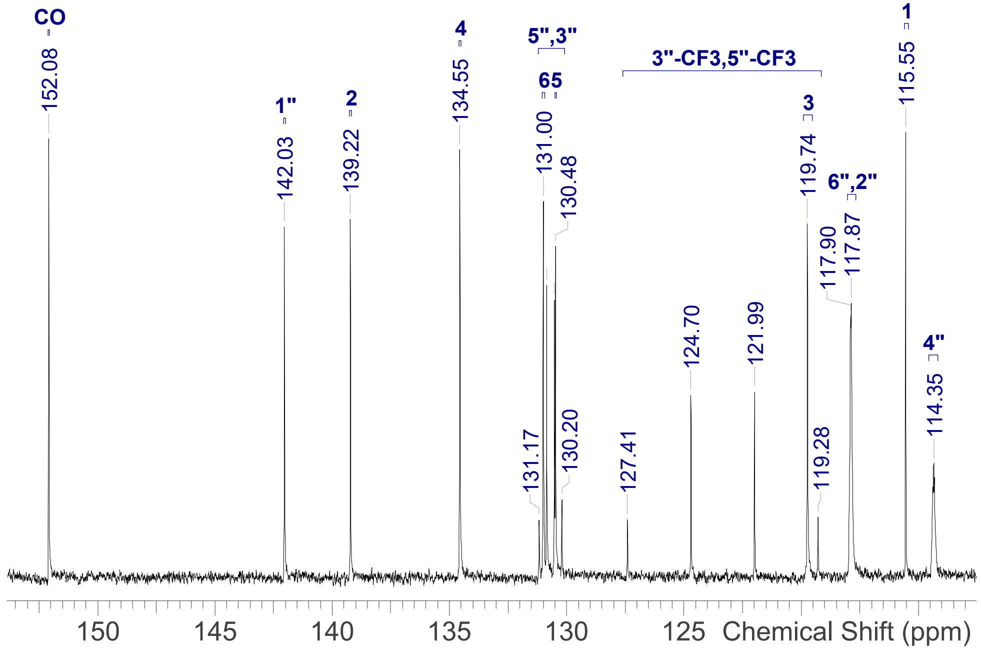

NVR-122_13C.spectrus

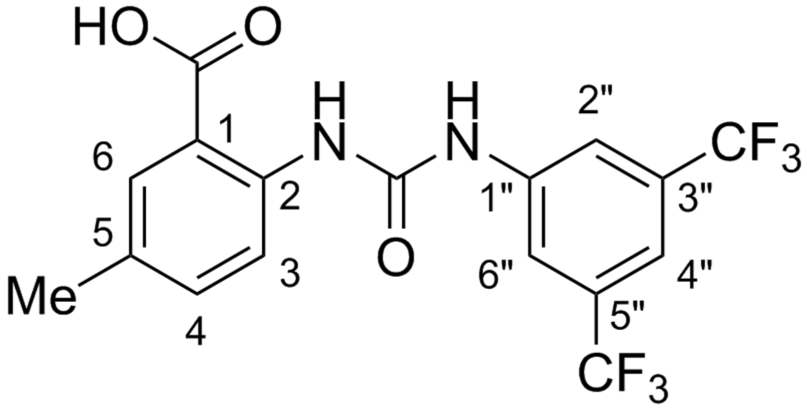

Shift (ppm)	F	m
-61.72	6	s

Acquisition Time (sec)	1.4680
Date	29 Mar 2019 05:27:06
Date Stamp	29 Mar 2019 05:27:06
Frequency (MHz)	376.4419
Nucleus	19F
Number of Transients	16
Solvent	DMSO-d6
Temperature (degree C)	25.001

^{19}F NMR (376 MHz, *DMSO-d_6*) δ ppm -61.72 (s, 6 F)

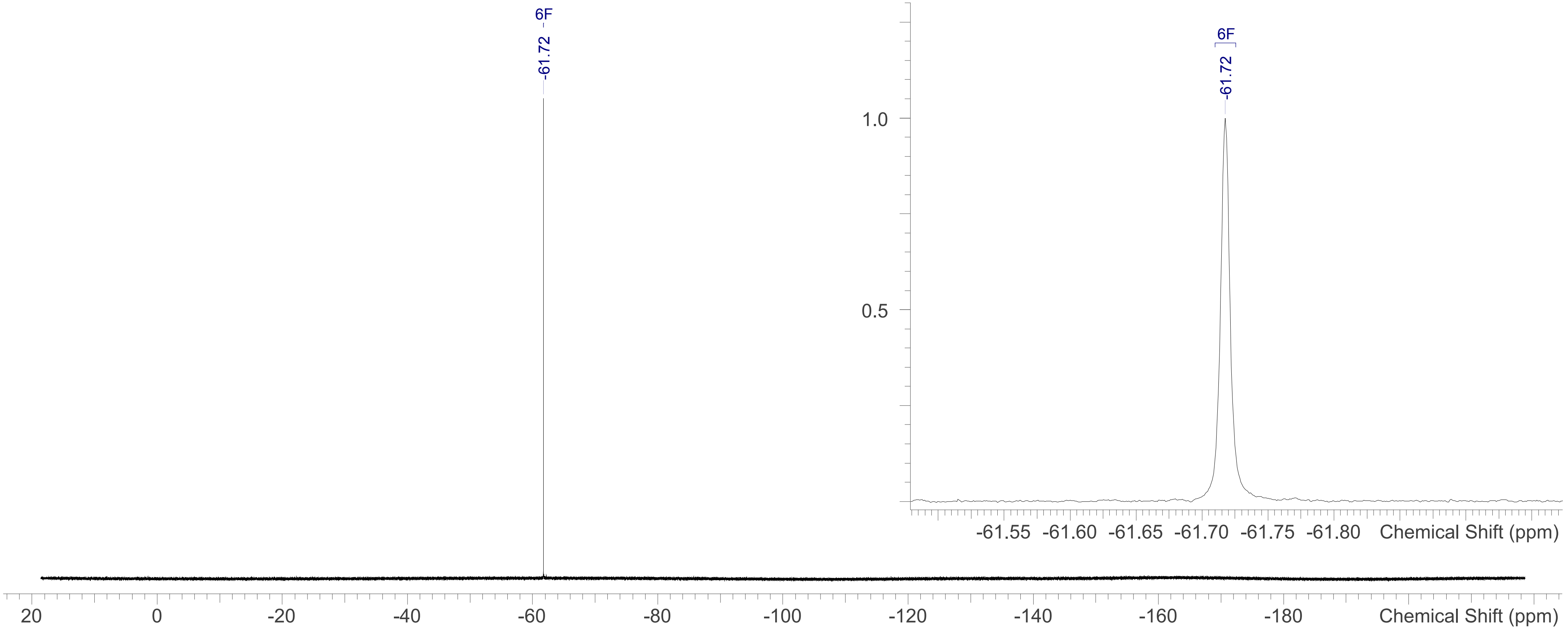

NVR-122_19F.spectrus

Openlynx Report - James B

Sample: 1 | Vial:2:34 | ID:JB152-1

File:JB152-1 | Date:04-Apr-2019 | Time:15:36:49

Description:

Printed: Mon Apr 15 12:24:00 2019

3: UV Detector: TAC: Wavelength Range: (210 - 400)

3.873e+2
Range: 3.974e+2

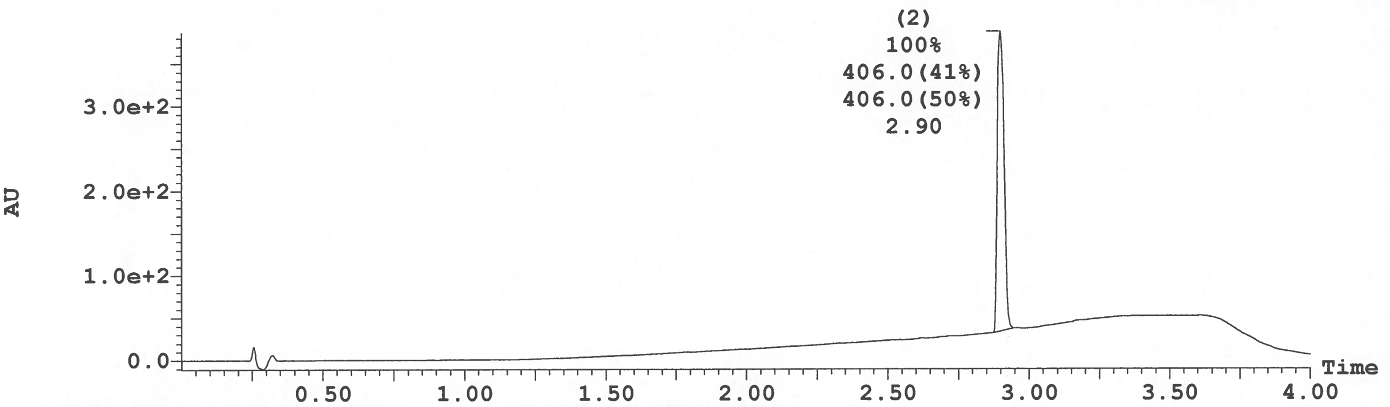

Peak Number	Compound	Time	Area %Total	Mass Found
2	Found	2.90	100.00	406.0, 406.0

1: MS ES+ :TIC Smooth (Mn, 2x2)

2.4e+007

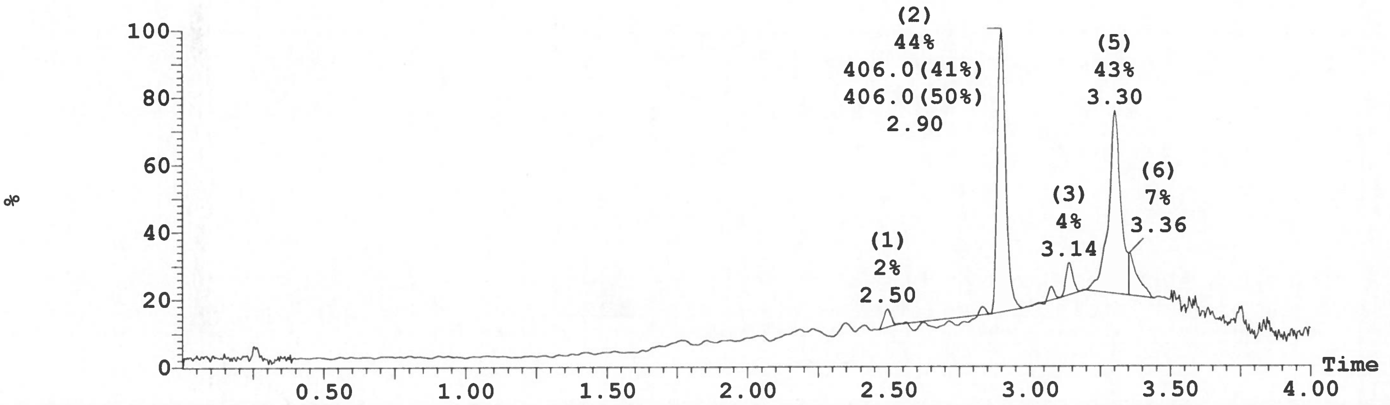

2: MS ES- :TIC Smooth (Mn, 2x2)

8.8e+006

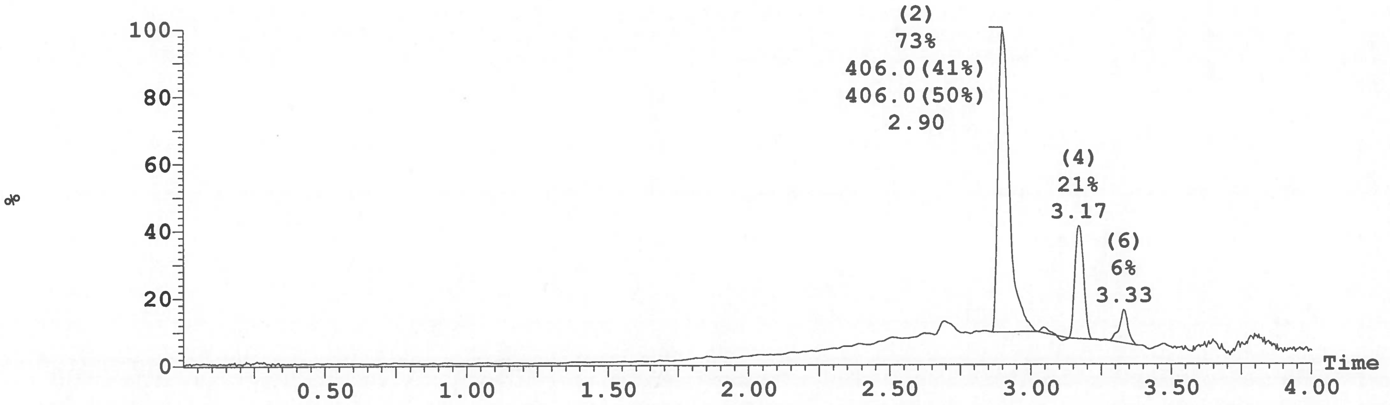

NVR-122 LCMS

Sample: 1 | Vial:2:34 | ID:JB152-1
File:JB152-1 | Date:04-Apr-2019 | Time:15:36:49
Description:

Printed: Mon Apr 15 12:24:00 2019

Peak ID	Compound	Time	Mass Found
2	Found	2.90	407

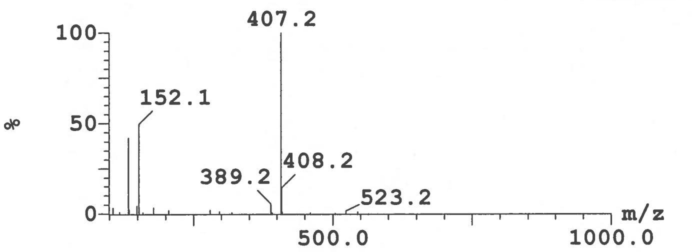

Peak ID	Compound	Time	Mass Found
2	Found	2.90	405

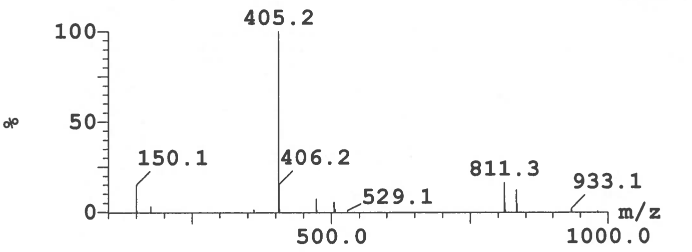

Peak ID	Compound	Time	Mass Found
2		2.90	Not Found

2:(Time: 2.90) Combine (1740) 3:UV Detector
3.345 AU

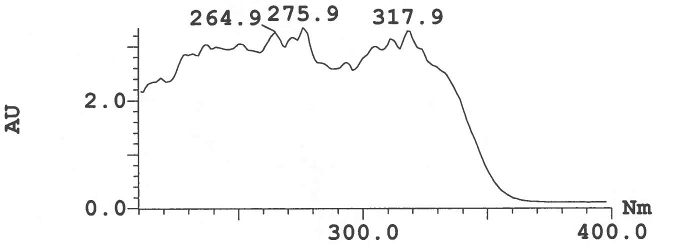

NVR-122 LCMS

Thermo Exactive Plus EMR Orbitrab ASAP pos

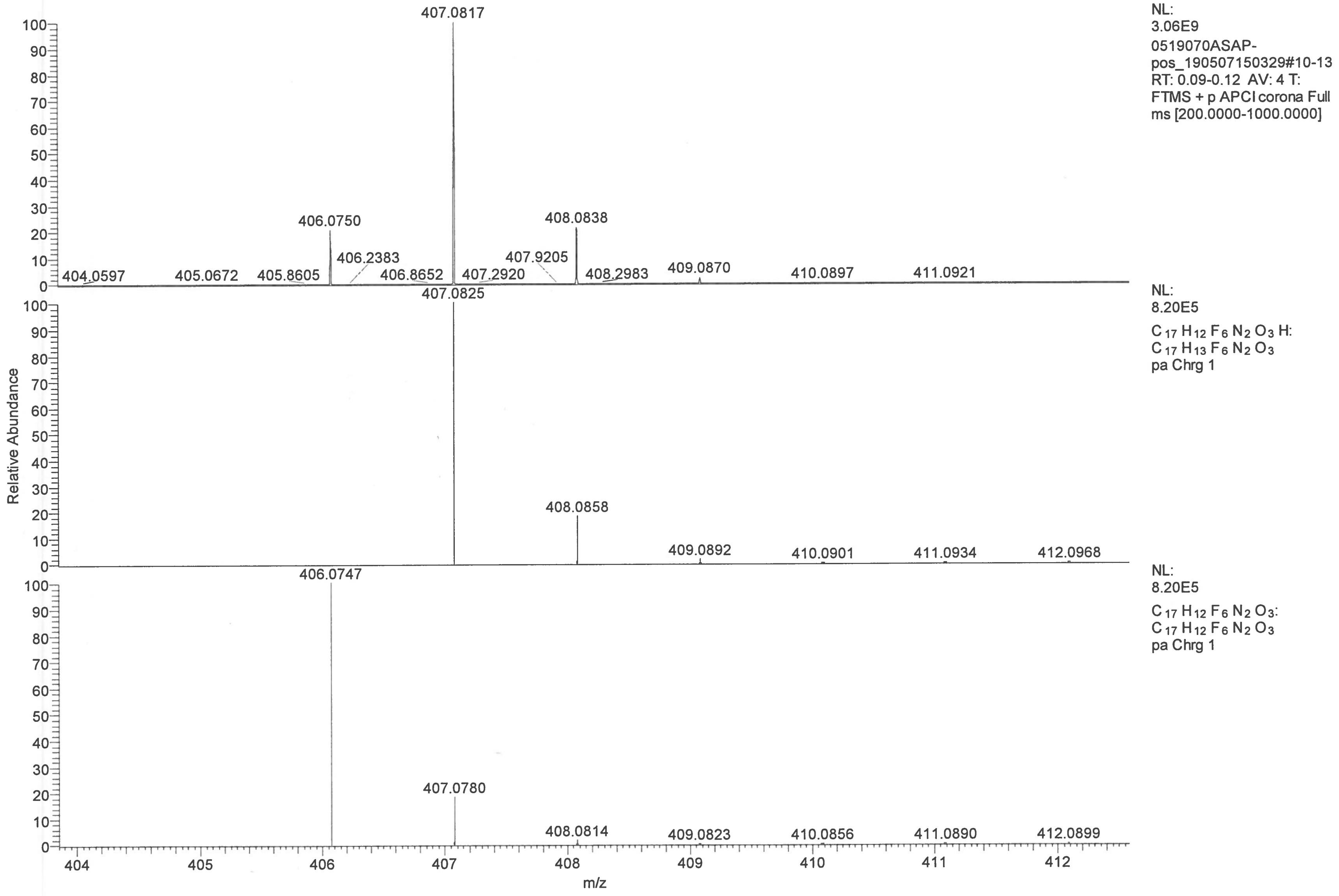

NVR-122 HRMS

Thermo Exactive Plus EMR Orbitrab ASAP pos

m/z	Theo. Mass	Delta (ppm)	RDB equiv.	Composition
406.0750	406.0747	0.83	10.0	C17 H12 O3 N2 F6 ←
	406.0733	4.14	10.5	C15 H10 O2 N5 F6

NVR-122 HRMS

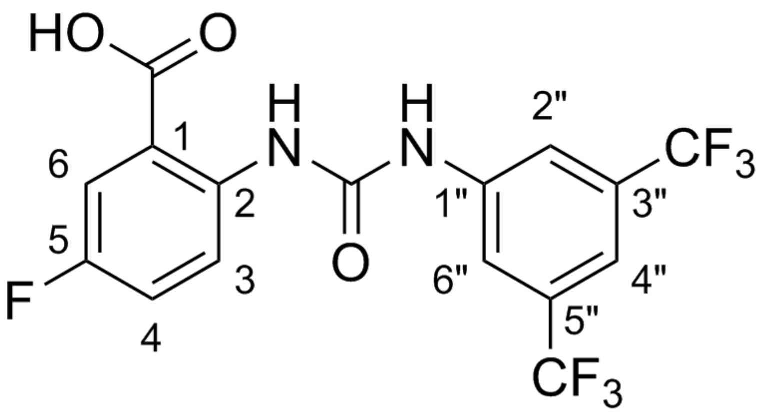

Shift (ppm)	H	m	J (Hz)	Assign
13.89	1	br s	-	COOH
10.54	1	s	-	NH"
10.48	1	s	-	NH
8.41	1	dd	9.4, 5.1	3
8.18	2	br s	-	2", 6"
7.67	1	dd	9.4, 3.1	6
7.64	1	br s	-	4"
7.45	1	ddd	9.3, 7.8, 3.1	4

Acquisition Time (sec)	3.9846
Date	10 May 2019 23:17:19
Date Stamp	10 May 2019 23:17:19
Frequency (MHz)	400.0700
Nucleus	1H
Number of Transients	16
Solvent	DMSO-d6
Temperature (degree C)	25.000

^{1}H NMR (400 MHz, *DMSO-d_6*) δ ppm 13.89 (br s, 1 H), 10.54 (s, 1 H), 10.48 (s, 1 H), 8.41 (dd, *J*=9.4, 5.1 Hz, 1 H), 8.18 (br s, 2 H), 7.67 (dd, *J*=9.4, 3.1 Hz, 1 H), 7.64 (br s, 1 H), 7.45 (ddd, *J*=9.3, 7.8, 3.1 Hz, 1 H)

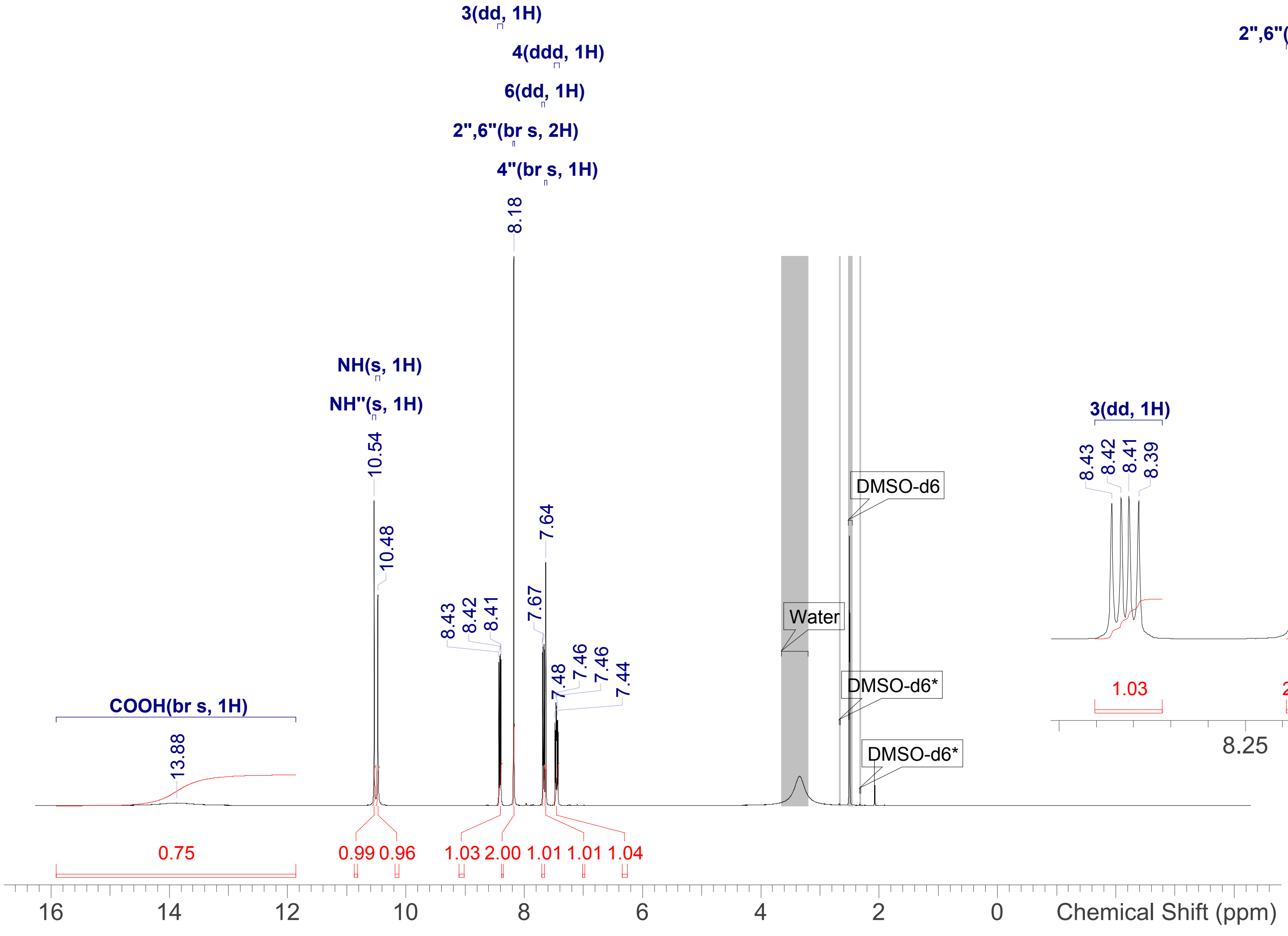

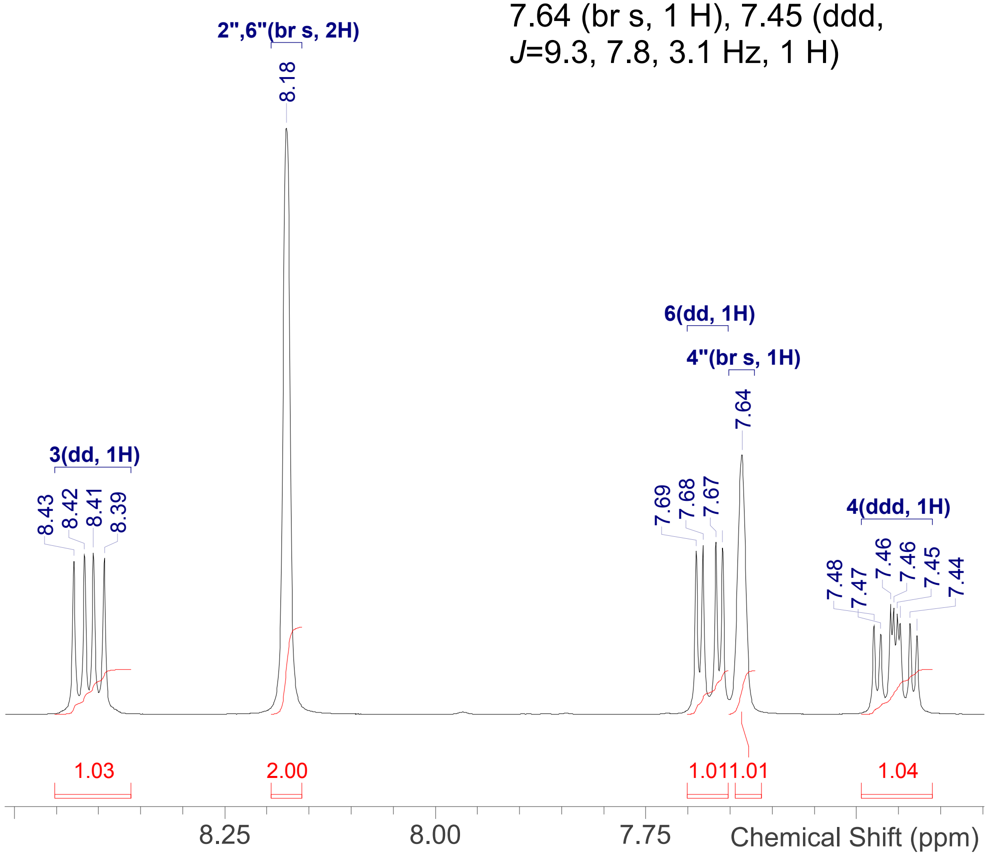

NVR-9_1H.spectrus

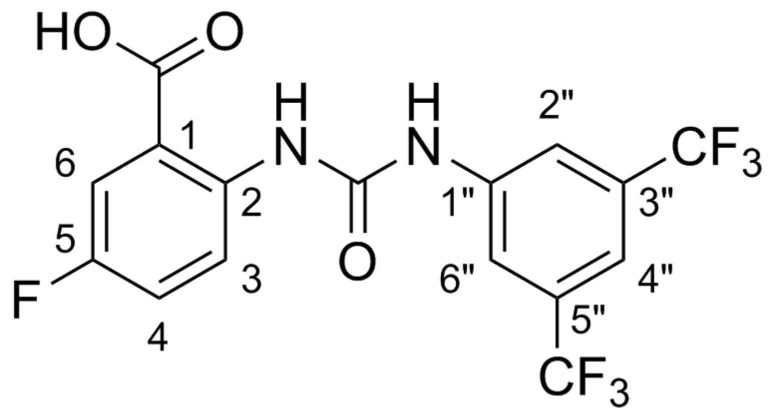

Shift (ppm)	C	m	J (Hz)	Assign
168.4	1	d	2.0	COOH
156.1	1	d	239.6	5
152.1	1	s	-	CO
141.9	1	s	-	1"
138.0	1	d	2.0	2
130.7	2	q	32.6	5", 3"
123.3	2	q	274.2	3"-CF3, 5"-CF3
121.9	1	d	3.9	3
120.9	1	d	22.5	4
118.0	2	br q	3.9	6", 2"
117.4	1	d	6.8	1
116.6	1	d	23.5	6
114.6	1	br spt	3.9	4"

Acquisition Time (sec)	1.0224
Date	10 May 2019 23:31:38
Date Stamp	10 May 2019 23:31:38
Frequency (MHz)	100.5977
Nucleus	13C
Number of Transients	256
Solvent	DMSO-d6
Temperature (degree C)	25.000

^{13}C NMR (101 MHz, *DMSO-d_6*) δ ppm 168.4 (d, *J*=2.0 Hz, 1 C), 156.1 (d, *J*=239.6 Hz, 1 C), 152.1 (s, 1 C), 141.9 (s, 1 C), 138.0 (d, *J*=2.0 Hz, 1 C), 130.7 (q, *J*=32.6 Hz, 2 C), 121.9 (d, *J*=3.9 Hz, 1 C), 120.9 (d, *J*=22.5 Hz, 1 C), 123.3 (q, *J*=274.2 Hz, 2 C), 118.0 (br q, *J*=3.9 Hz, 2 C), 117.4 (d, *J*=6.8 Hz, 1 C), 116.6 (d, *J*=23.5 Hz, 1 C), 114.6 (br spt, *J*=3.9 Hz, 1 C)

NVR-9_13C

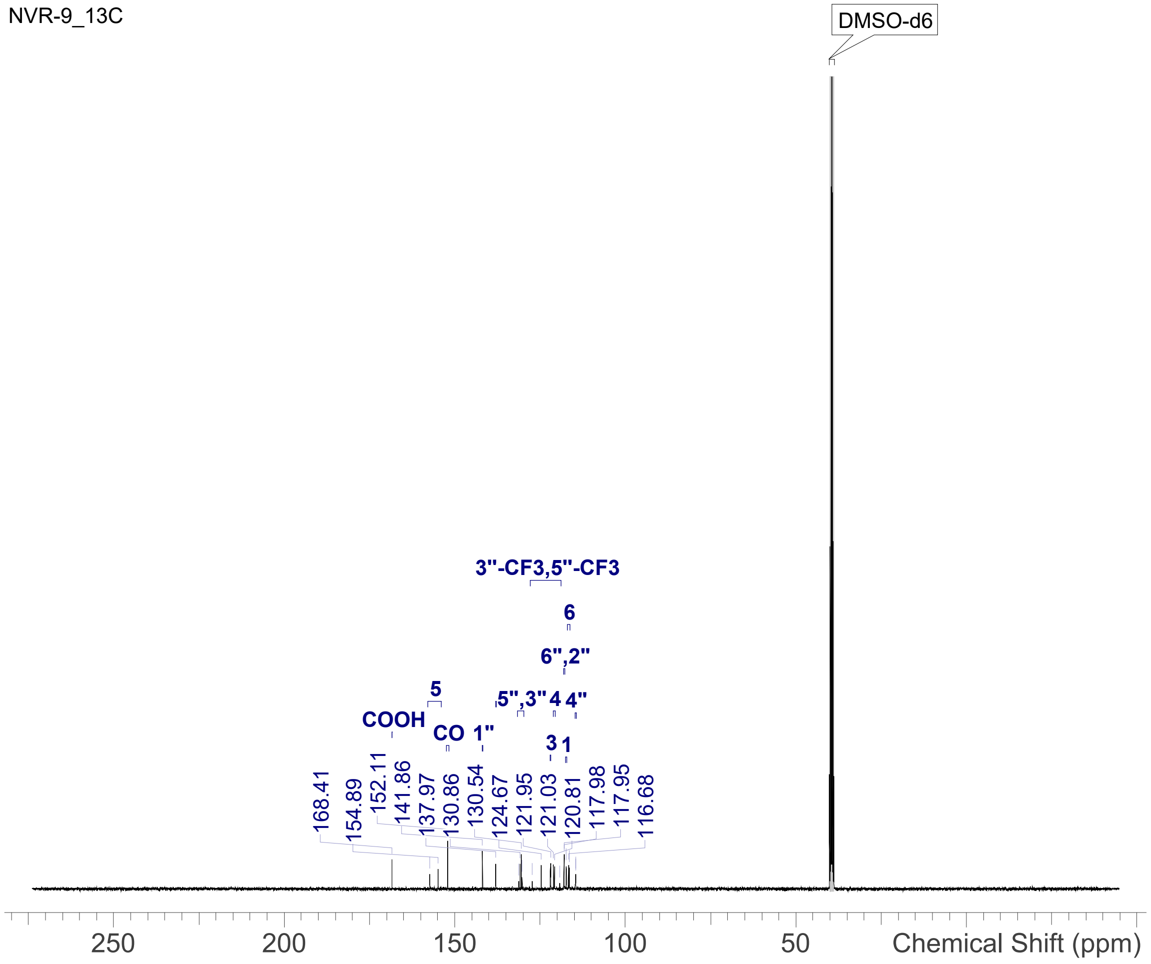

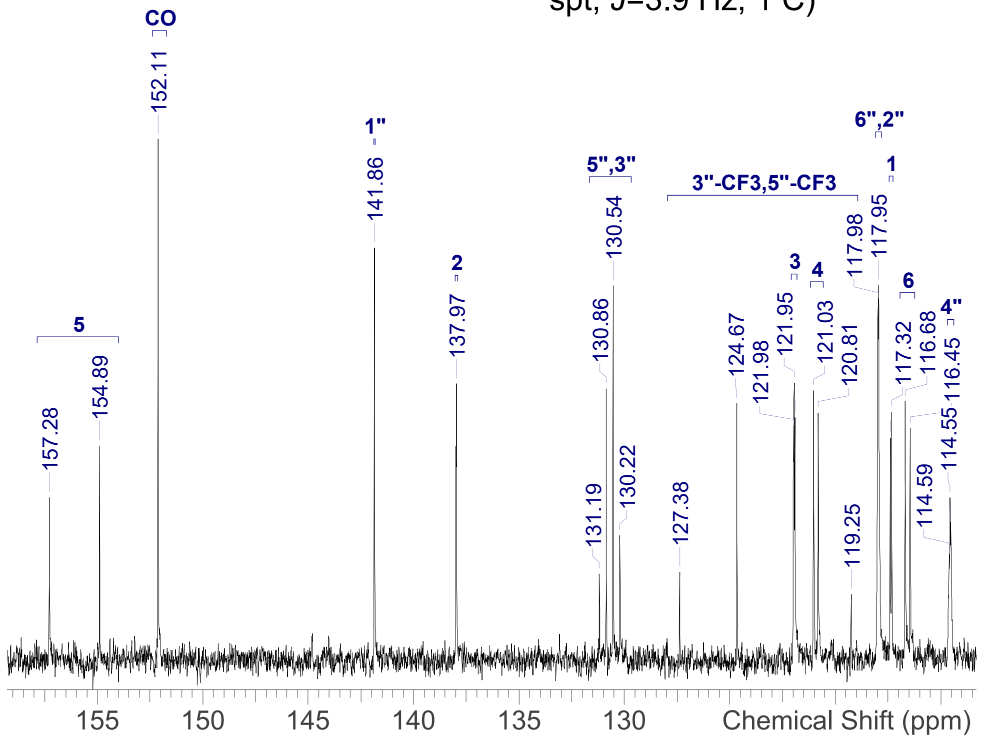

NVR-9_13C.spectrus

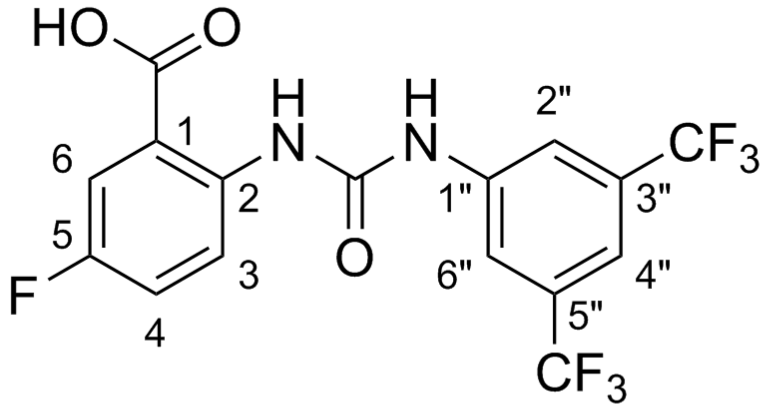

Shift (ppm)	F	m	J (Hz)
-61.78	6	s	-
-120.86	1	ddd	9.2, 8.2, 5.1

Acquisition Time (sec)	1.4680
Date	11 May 2019 00:42:25
Date Stamp	11 May 2019 00:42:25
Frequency (MHz)	376.4419
Nucleus	19F
Number of Transients	128
Solvent	DMSO-d6
Temperature (degree C)	25.000

^{19}F NMR (376 MHz, *DMSO-d_6*) δ ppm -61.78 (s, 6 F), -120.86 (ddd, *J*=9.2, 8.2, 5.1 Hz, 1 F)

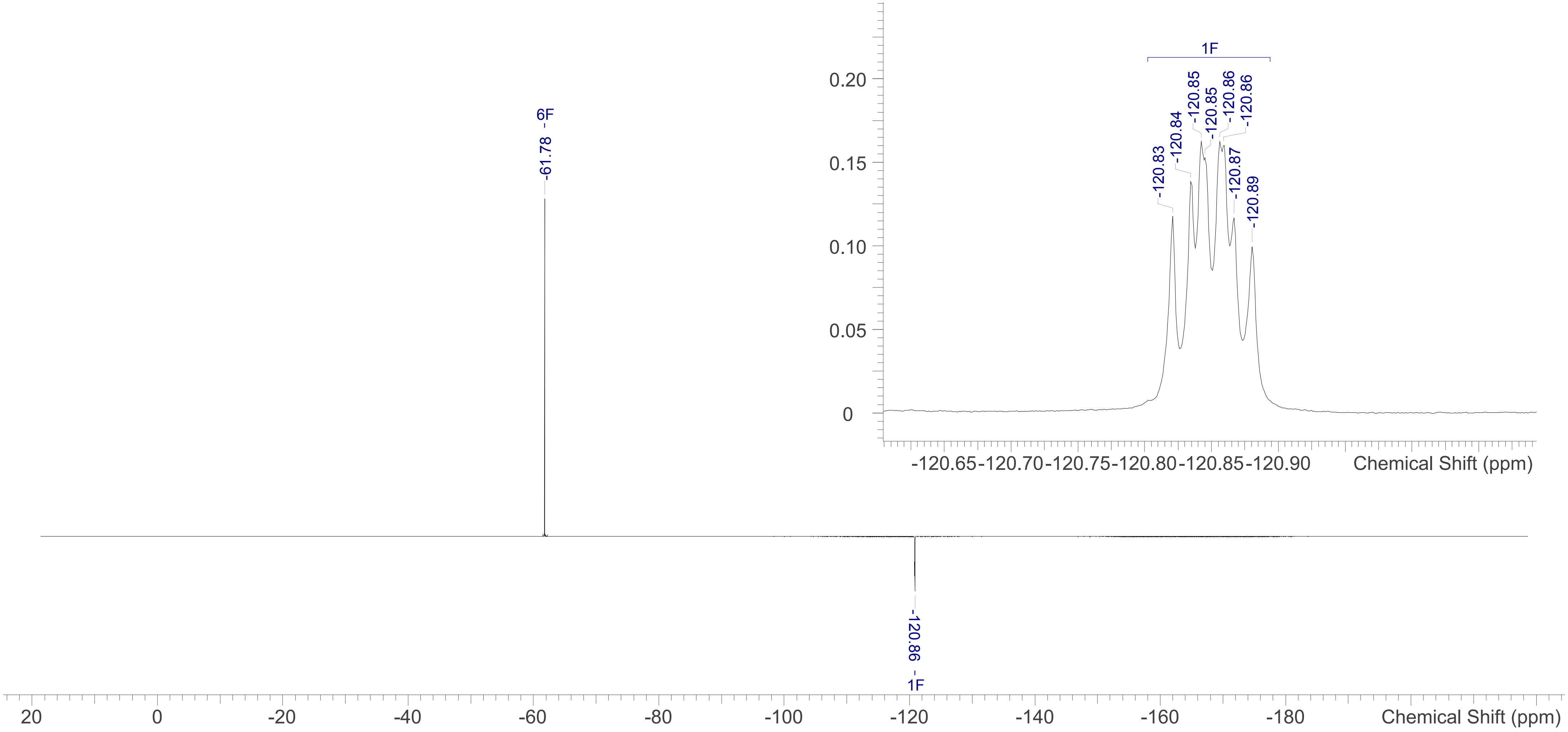

NVR-9_19F.spectrus

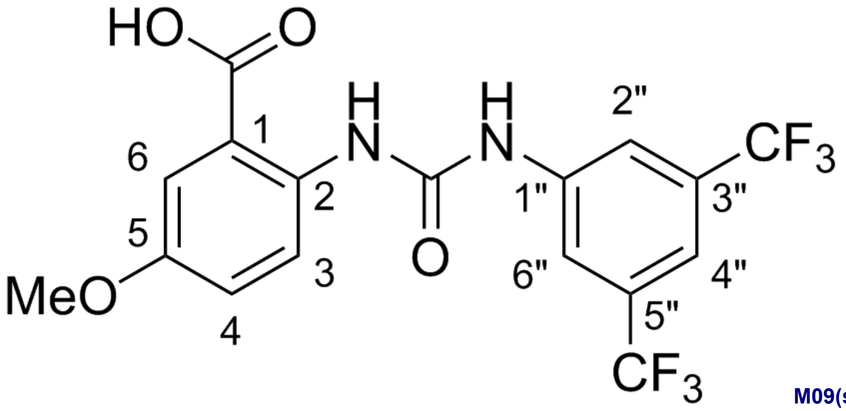

No.	Shift (ppm)	H	m	J (Hz)	Assign
1	13.62	1	br s	-	COOH
2	10.43	1	s	-	NH"
3	10.30	1	s	-	NH
4	8.29	1	d	9.3	3
5	8.18	2	br s	-	6", 2"
6	7.63	1	br s	-	4"
7	7.45	1	d	3.3	6
8	7.20	1	dd	9.2, 3.1	4
9	3.77	3	s	-	OMe

Acquisition Time (sec)	3.9846
Date	03 Apr 2019 08:45:02
Date Stamp	03 Apr 2019 08:45:02
Frequency (MHz)	400.0700
Nucleus	1H
Number of Transients	16
Solvent	DMSO-d6
Temperature (degree C)	25.001

^{1}H NMR (400 MHz, *DMSO-d_6*) δ ppm 13.62 (br s, 1 H), 10.43 (s, 1 H), 10.30 (s, 1 H), 8.29 (d, *J*=9.3 Hz, 1 H), 8.18 (br s, 2 H), 7.63 (br s, 1 H), 7.45 (d, *J*=3.3 Hz, 1 H), 7.20 (dd, *J*=9.2, 3.1 Hz, 1 H), 3.77 (s, 3 H)

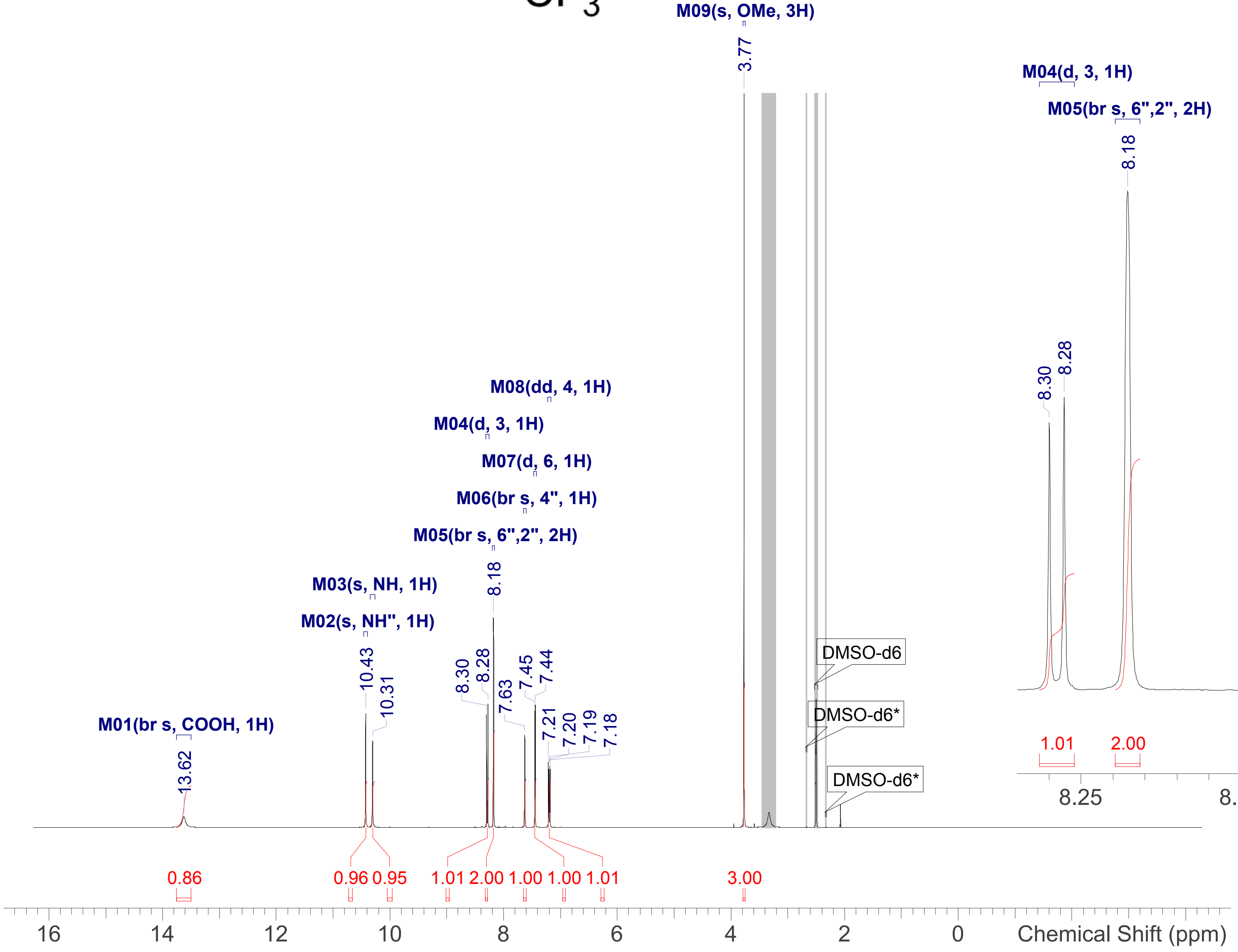

NVR-124_1H.spectrus

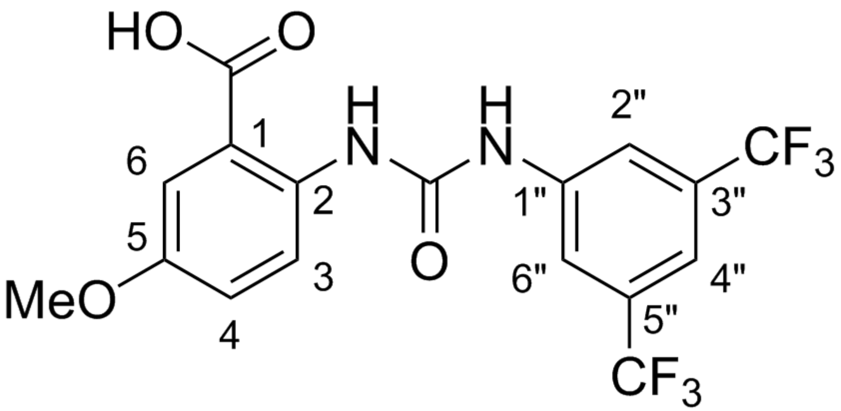

Shift (ppm)	C	m	J (Hz)	Assign
169.2	1	s	-	COOH
153.4	1	s	-	5
152.2	1	s	-	CO
142.1	1	s	-	1"
134.8	1	s	-	2
130.7	2	q	32.9	5", 3"
123.3	2	q	272.9	3"-CF3, 5"-CF3
121.7	1	s	-	3
120.3	1	s	-	4
117.8	2	br q	3.9	6", 2"
117.0	1	s	-	1
114.4	1	s	-	6
114.3	1	spt	3.9	4"
55.3	1	s	-	OMe

Acquisition Time (sec)	1.0224
Date	11 May 2019 02:09:23
Date Stamp	11 May 2019 02:09:23
Frequency (MHz)	100.5977
Nucleus	13C
Number of Transients	256
Solvent	DMSO-d6
Temperature (degree C)	25.001

^{13}C NMR (101 MHz, *DMSO-d$_6$*) δ ppm 169.2 (s, 1 C), 153.4 (s, 1 C), 152.2 (s, 1 C), 142.1 (s, 1 C), 134.8 (s, 1 C), 130.7 (q, *J*=32.9 Hz, 2 C), 121.7 (s, 1 C), 120.3 (s, 1 C), 123.3 (q, *J*=272.9 Hz, 2 C), 117.8 (br q, *J*=3.9 Hz, 2 C), 117.0 (s, 1 C), 114.4 (s, 1 C), 114.3 (spt, *J*=3.9 Hz, 1 C), 55.3 (s, 1 C)

NVR-124_13C

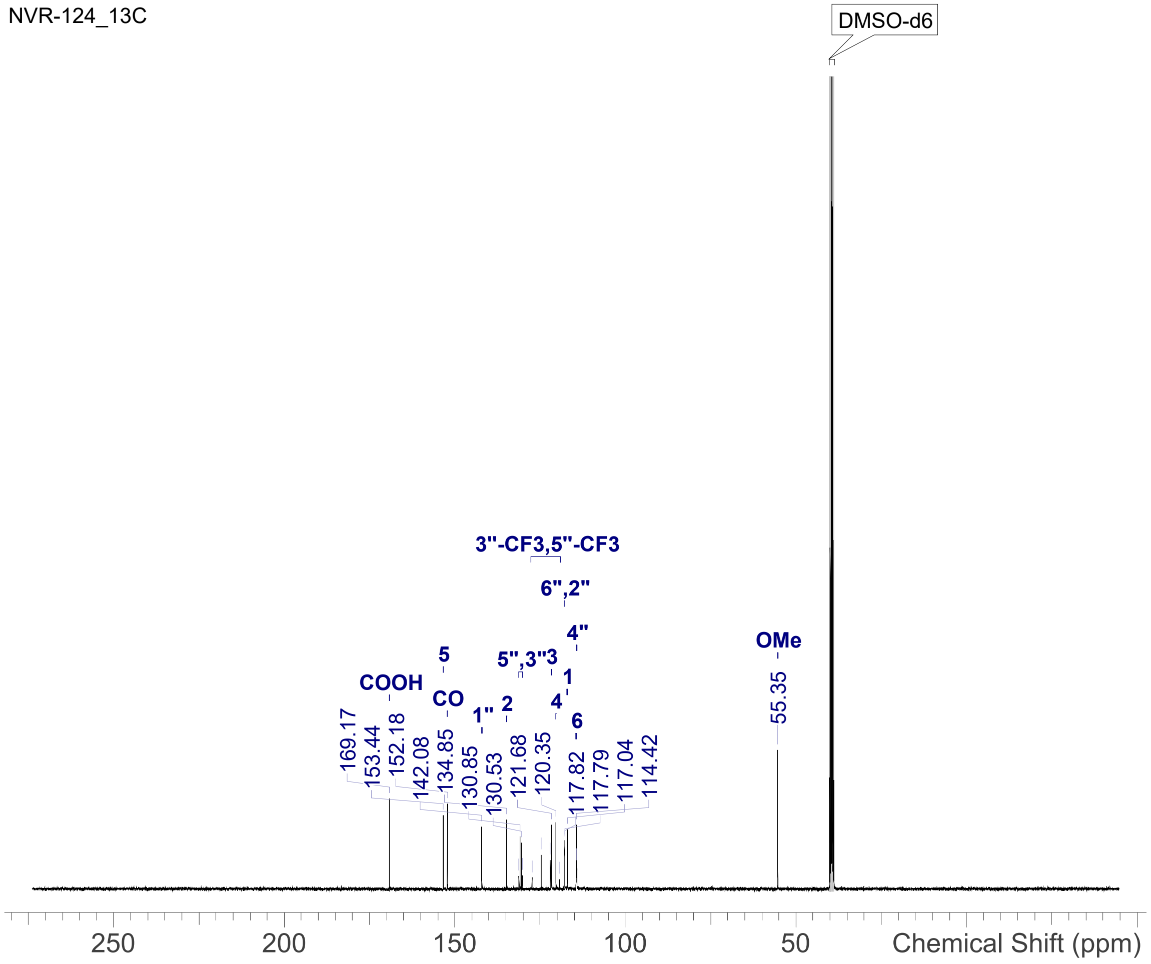

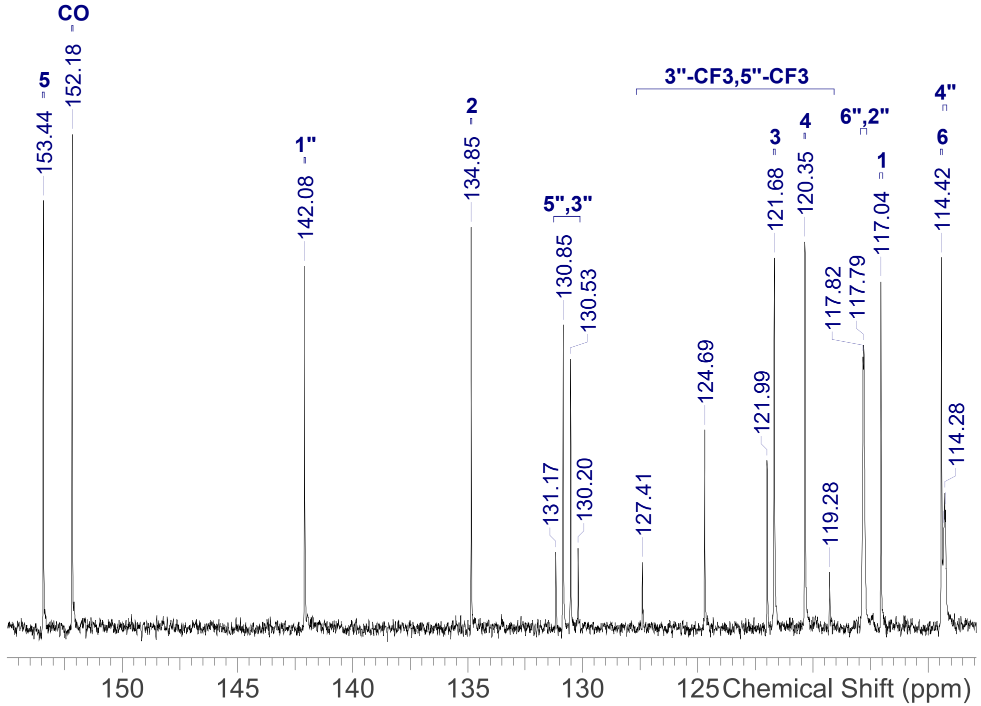

NVR-124_13C.spectrus

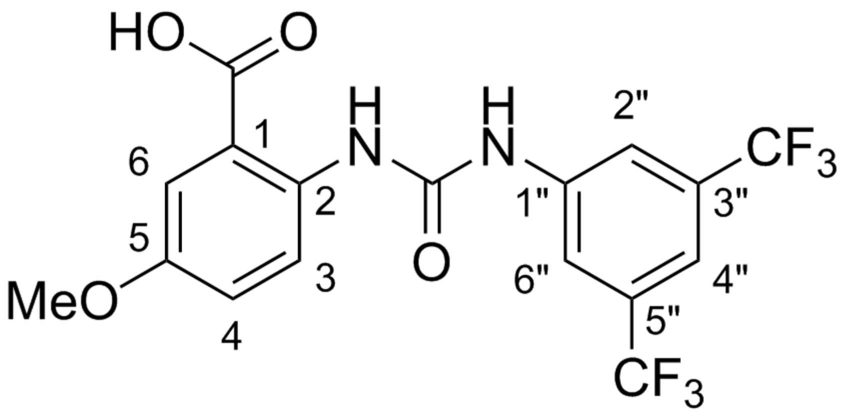

Shift (ppm)	F	m
-61.75	6	s

Acquisition Time (sec)	1.4680
Date	02 Apr 2019 17:51:21
Date Stamp	02 Apr 2019 17:51:21
Frequency (MHz)	376.4419
Nucleus	19F
Number of Transients	16
Solvent	DMSO-d6
Temperature (degree C)	25.001

^{19}F NMR (376 MHz, *DMSO-d_6*) δ ppm -61.75 (s, 6 F)

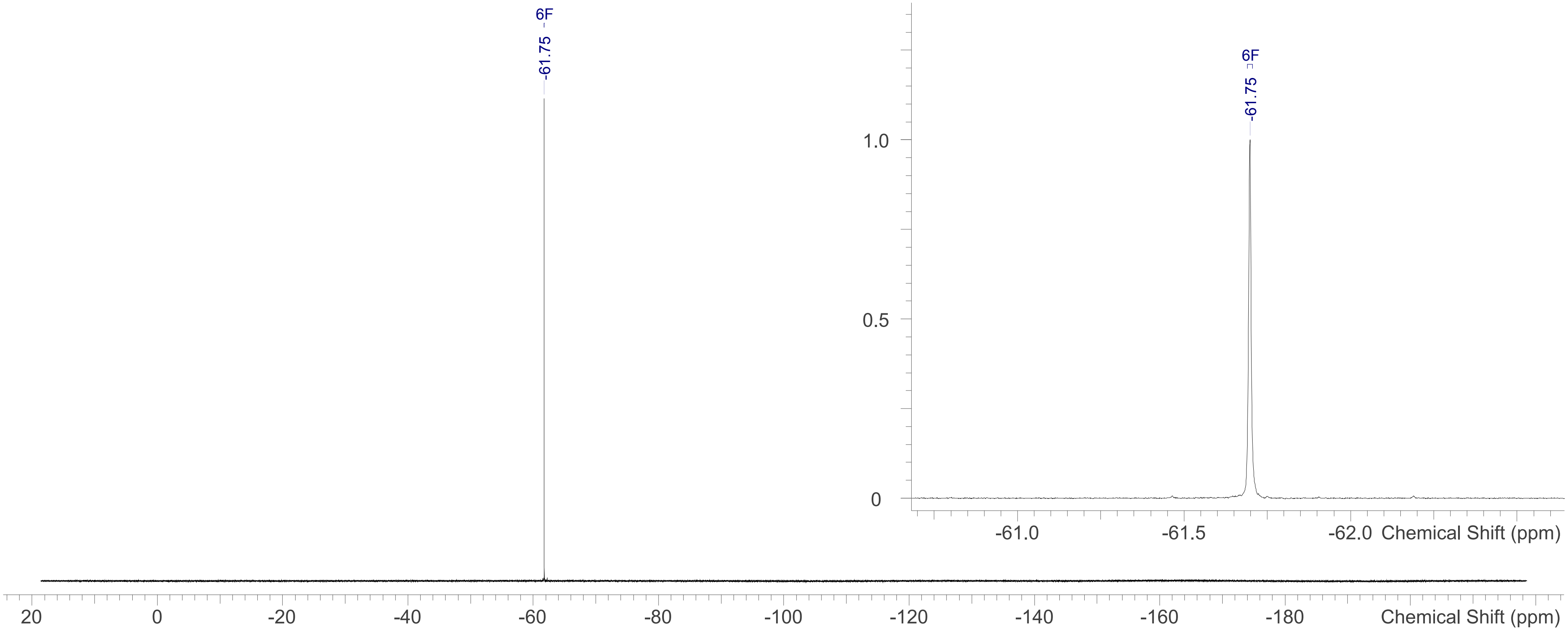

NVR-124_19F.spectrus

Openlynx Report - James B

Sample: 1 | Vial:2:4 | ID:JB155-1
File:JB155-1 | Date:01-Apr-2019 | Time:16:13:36
Description:

Printed: Tue Apr 02 15:22:54 2019

3: UV Detector: TAC: Wavelength Range: (210 - 400)

3.631e+2
Range: 3.753e+2

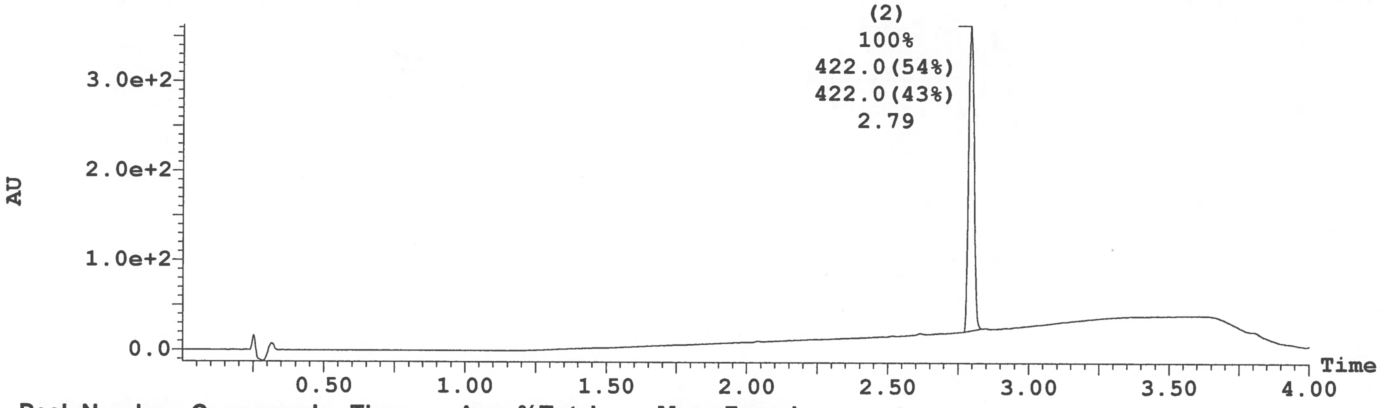

Peak Number	Compound	Time	Area %Total	Mass Found
2	Found	2.79	100.00	422.0, 422.0

1: MS ES+ :TIC Smooth (Mn, 2x2)

1.9e+007

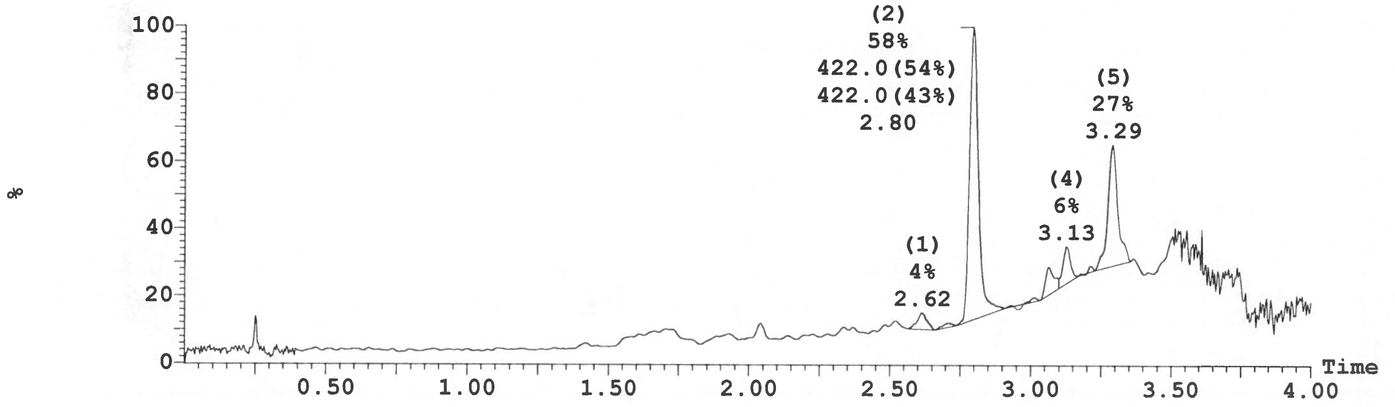

2: MS ES- :TIC Smooth (Mn, 2x2)

1.1e+007

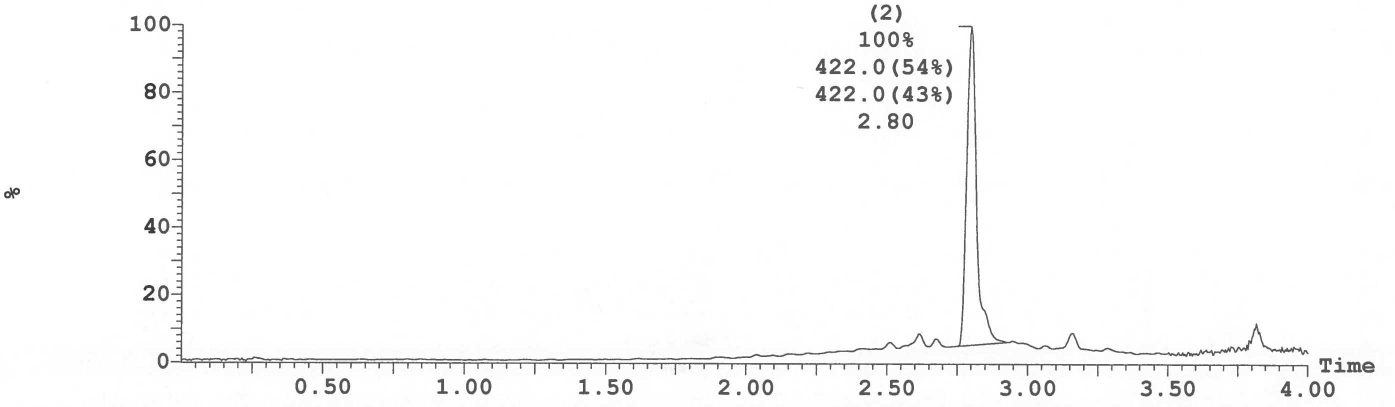

NVR-124 LCMS

Sample: 1 Vial:2:4 ID:JB155-1
File:JB155-1 Date:01-Apr-2019 Time:16:13:36
Description:

Printed: Tue Apr 02 15:22:54 2019

Peak ID	Compound	Time	Mass Found
2	Found	2.80	423

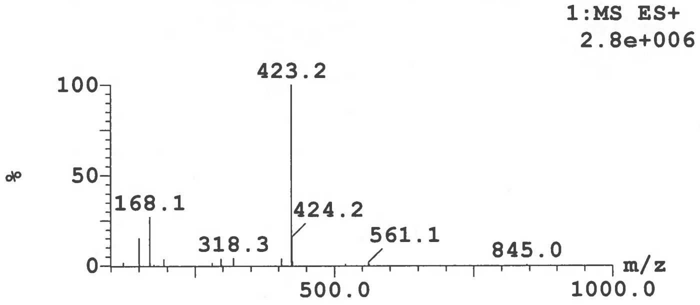

Peak ID	Compound	Time	Mass Found
2	Found	2.80	421

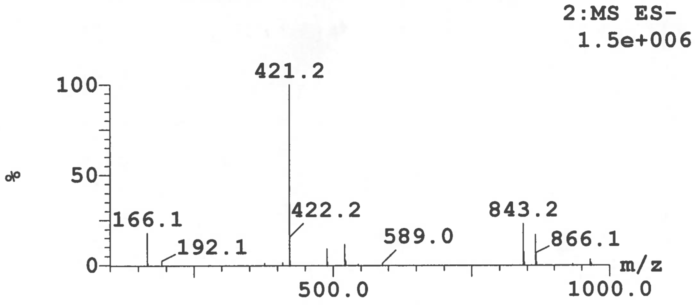

Peak ID	Compound	Time	Mass Found
2		2.80	Not Found

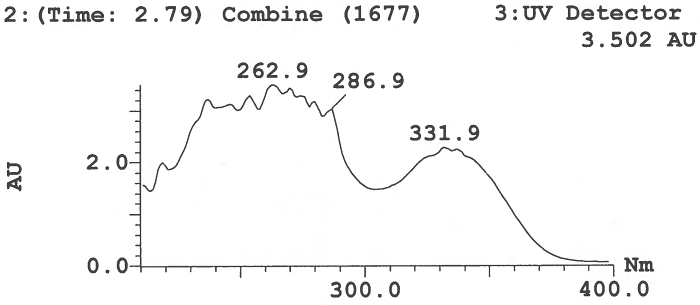

NVR-124 LCMS

Thermo Exactive Plus EMR Orbitrab ASAP pos

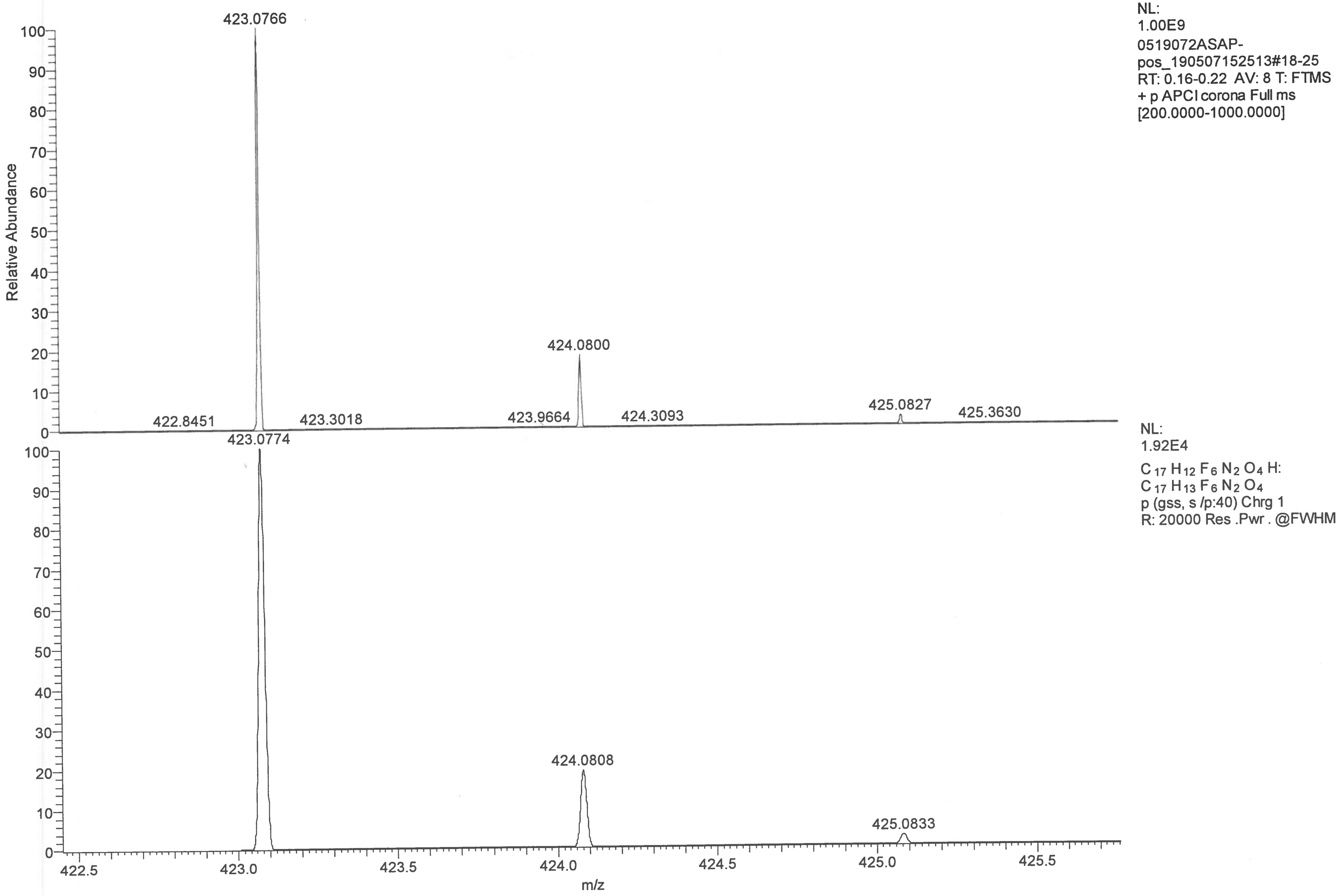

NVR-124 HRMS

Thermo Exactive Plus EMR Orbitrab ASAP pos

m/z	Theo. Mass	Delta (ppm)	RDB equiv.	Composition
423.0766	423.0761	1.28	10.0	C15 H11 O3 N5 F6
	423.0774	-1.90	9.5	C17 H13 O4 N2 F6 ←
	423.0747	4.44	5.0	C14 H15 O7 N F6
	423.0747	4.45	10.5	C13 H9 O2 N8 F6

NVR-124 HRMS

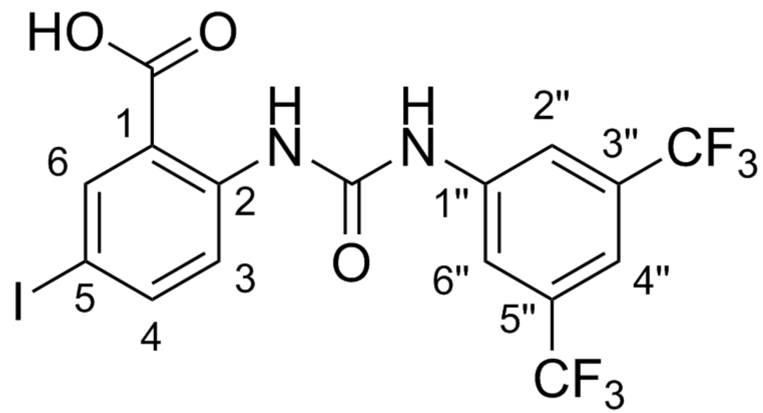

Shift (ppm)	H	m	J (Hz)	Assign
13.88	1	br s	-	COOH
10.60	2	s	-	NH'', NH
8.26	1	d	8.9	H3
8.21	1	d	2.3	H6
8.18	2	br s	-	H2'', H6''
7.87	1	dd	8.9, 2.2	H4
7.66	1	s	-	H4''

Acquisition Time (sec)	6.5536
Date	08/08/2017 09:02:00
Date Stamp	08/08/2017 09:02:00
Frequency (MHz)	500.1930
Nucleus	1H
Number of Transients	16
Solvent	DMSO-d6

^{1}H NMR (500 MHz, *DMSO-d$_6$*) δ ppm 13.88 (br s, 1 H), 10.60 (s, 2 H), 8.26 (d, *J*=8.9 Hz, 1 H), 8.21 (d, *J*=2.3 Hz, 1 H), 8.18 (br s, 2 H), 7.87 (dd, *J*=8.9, 2.2 Hz, 1 H), 7.66 (s, 1 H)

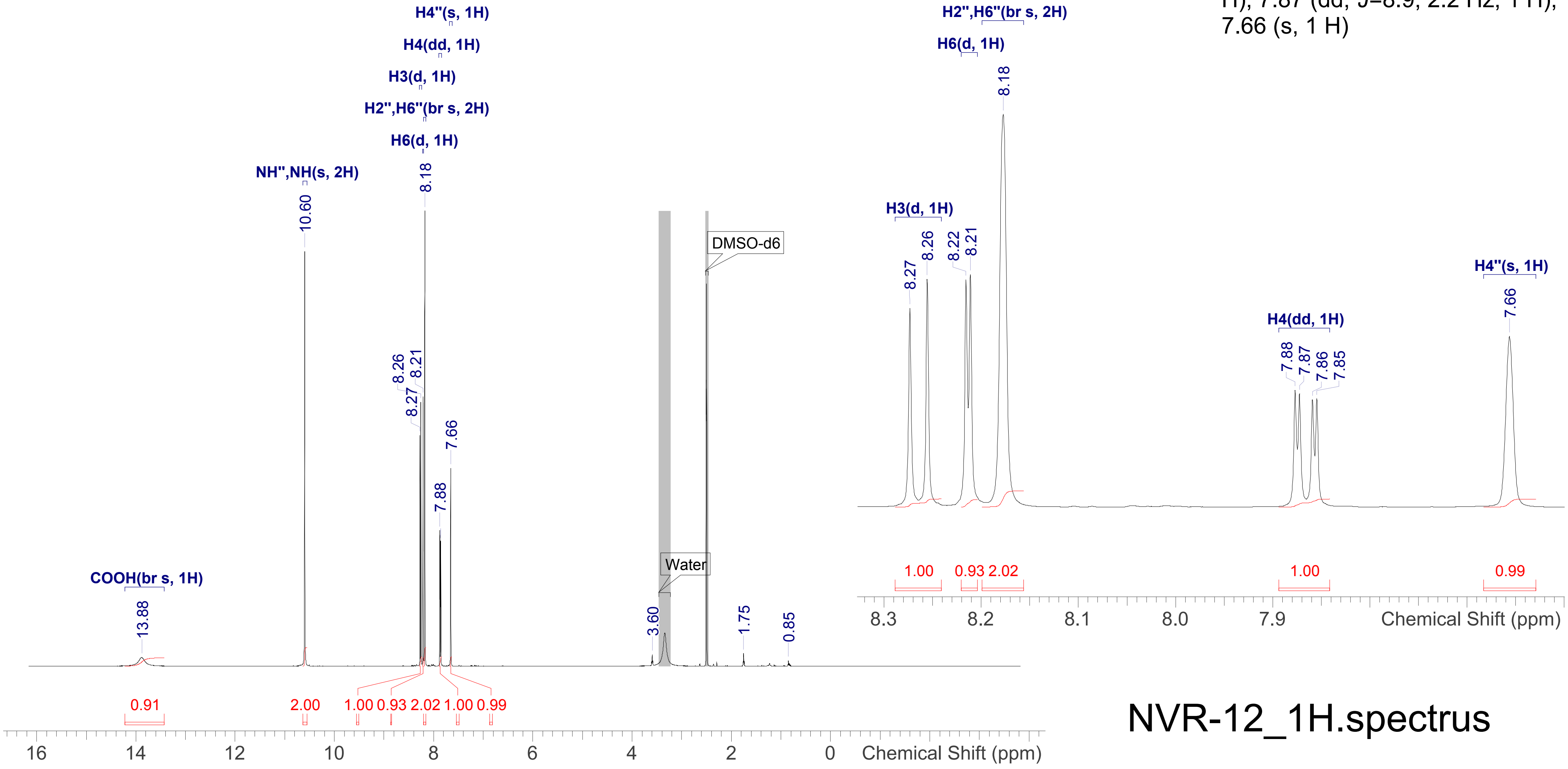

NVR-12_1H.spectrus

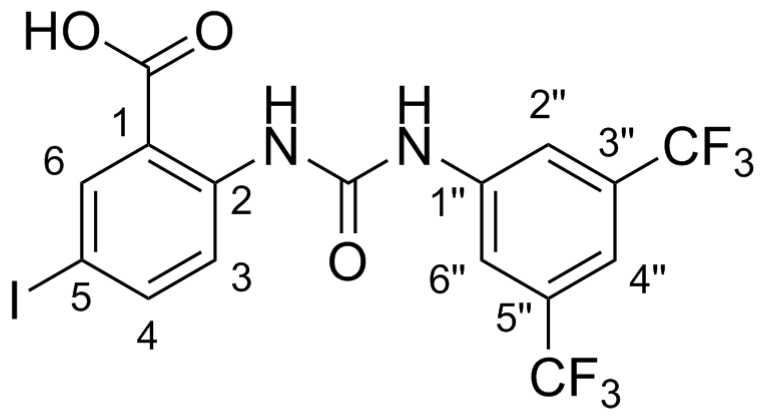

Shift (ppm)	C	m	J (Hz)	Assign
168.2	1	s	-	COOH
151.8	1	s	-	CO
142.1	1	s	-	6
141.7	1	s	-	2
141.3	1	s	-	1"
138.9	1	s	-	4
130.7	2	q	32.6	5", 3"
123.3	2	q	272.6	CF3", CF3"
121.8	1	s	-	3
118.0	2	br q	3.9	6", 2"
117.7	1	s	-	1
114.7	1	br spt	3.9	4"
84.3	1	s	-	5

Acquisition Time (sec)	2.0447
Date	08/08/2017 15:23:00
Date Stamp	08/08/2017 15:23:00
Frequency (MHz)	125.7870
Nucleus	13C
Number of Transients	256
Solvent	DMSO-d6

^{13}C NMR (126 MHz, *DMSO-d_6*) δ ppm 168.2 (s, 1 C), 151.8 (s, 1 C), 142.1 (s, 1 C), 141.7 (s, 1 C), 141.3 (s, 1 C), 138.9 (s, 1 C), 130.7 (q, *J*=32.6 Hz, 2 C), 121.8 (s, 1 C), 123.3 (q, *J*=272.6 Hz, 2 C), 118.0 (br q, *J*=3.9 Hz, 2 C), 117.7 (s, 1 C), 114.7 (br spt, *J*=3.9 Hz, 1 C), 84.3 (s, 1 C)

NVR-12_13C

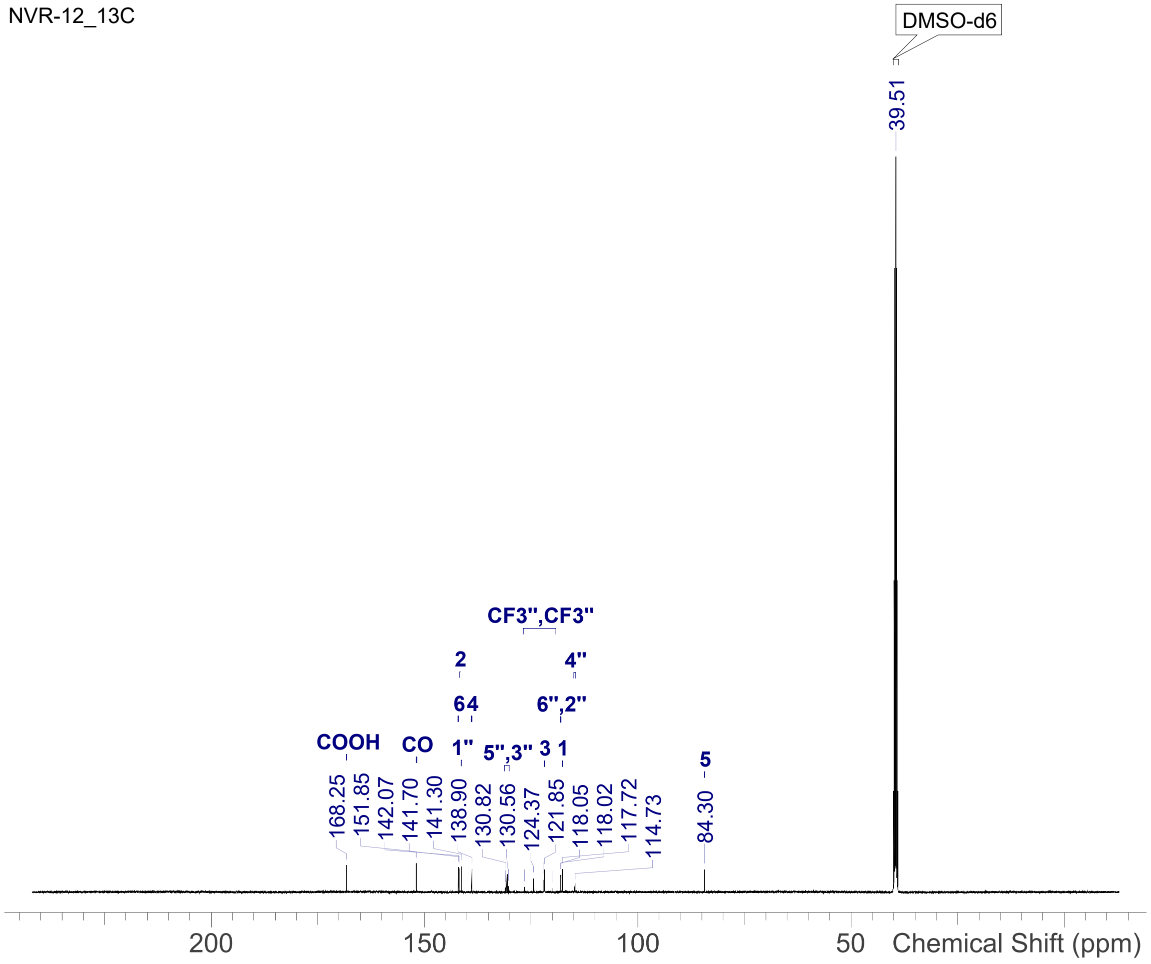

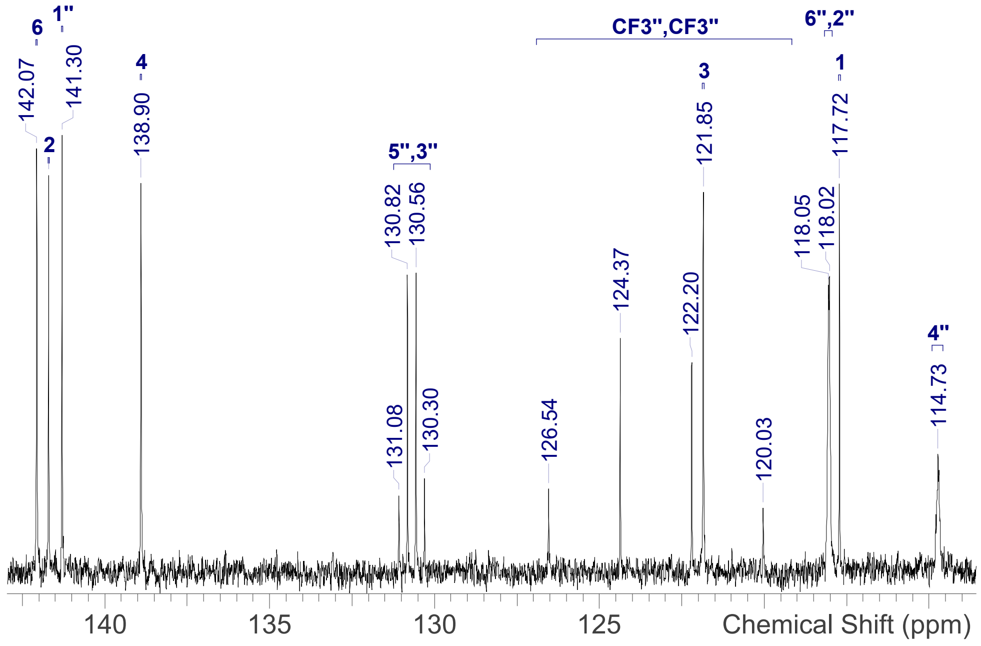

NVR-12_13C.spectrus

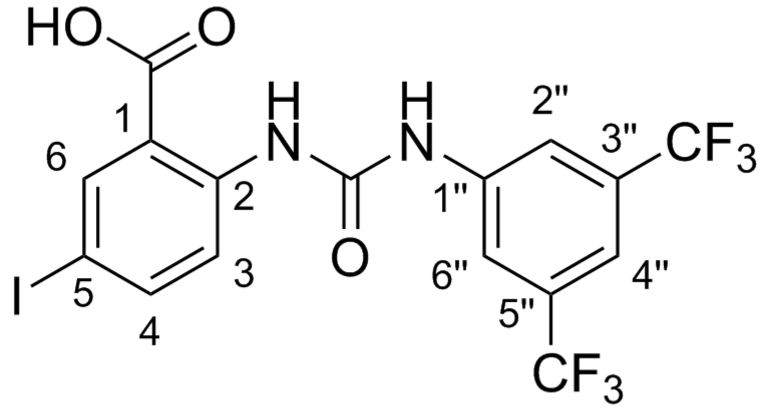

Shift (ppm)	F	m
-61.75	6	s

Acquisition Time (sec)	2.9360
Date	08/08/2017 15:57:00
Date Stamp	08/08/2017 15:57:00
Frequency (MHz)	470.6020
Nucleus	19F
Number of Transients	128
Solvent	DMSO-d6

^{19}F NMR (471 MHz, *DMSO-d_6*) δ ppm -61.75 (s, 6 F)

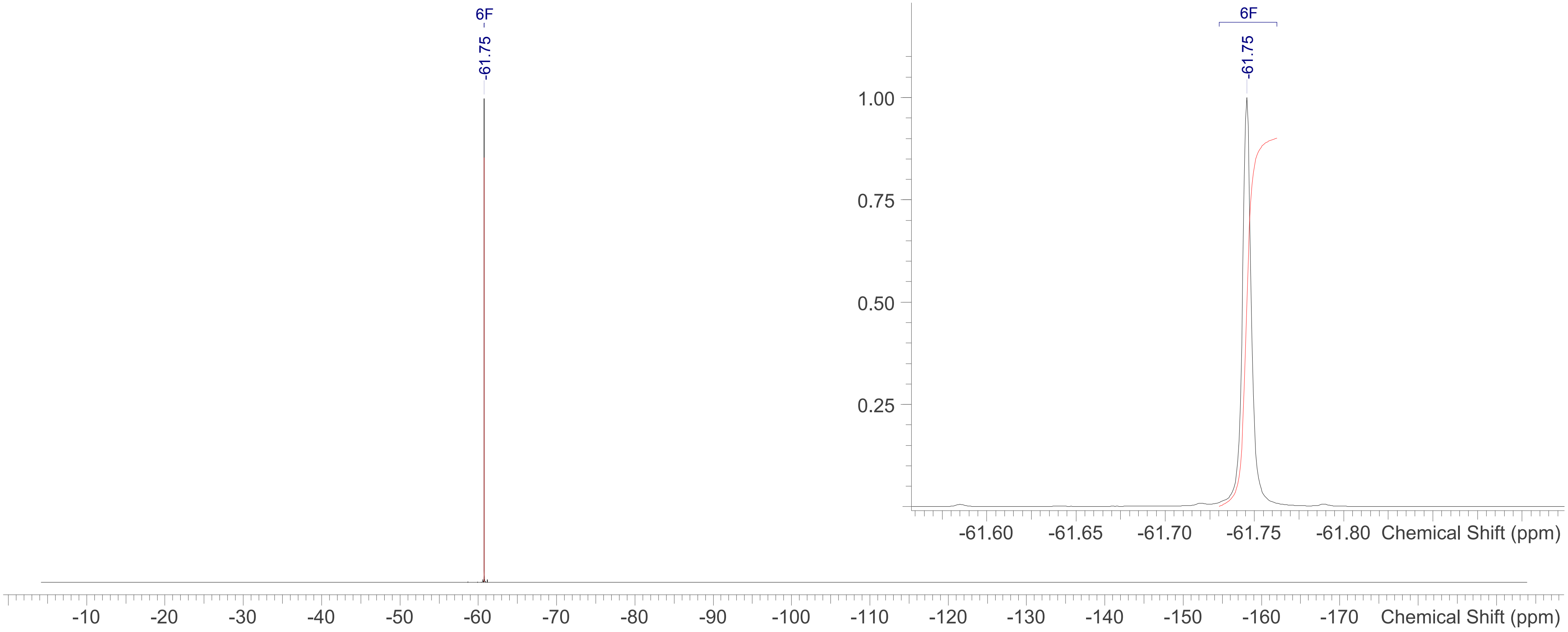

NVR-12_19F.spc

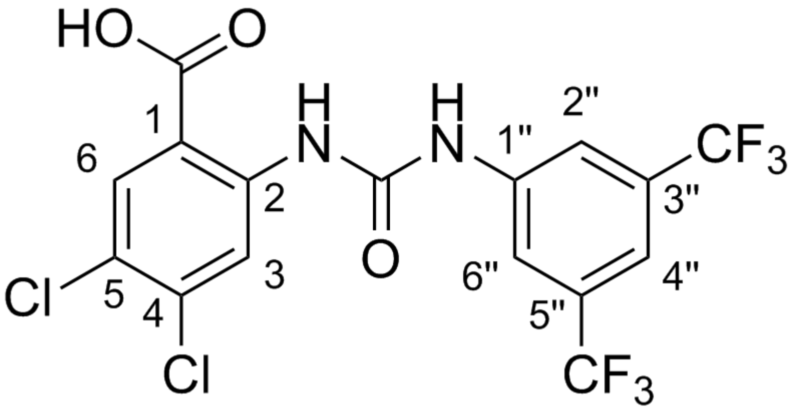

No.	Shift (ppm)	H	m	Assign
1	10.68	2	s	NH", NH
2	8.70	1	s	3
3	8.14	2	br s	2", 6"
4	8.01	1	s	6
5	7.63	1	br s	4"

Acquisition Time (sec)	8.1789
Date	20/06/2019 17:11:00
Date Stamp	20/06/2019 17:11:00
Frequency (MHz)	400.1320
Nucleus	1H
Number of Transients	16
Solvent	DMSO-d6

^{1}H NMR (400 MHz, *DMSO-d$_6$*) δ ppm 10.68 (s, 2 H), 8.70 (s, 1 H), 8.14 (br s, 2 H), 8.01 (s, 1 H), 7.63 (br s, 1 H)

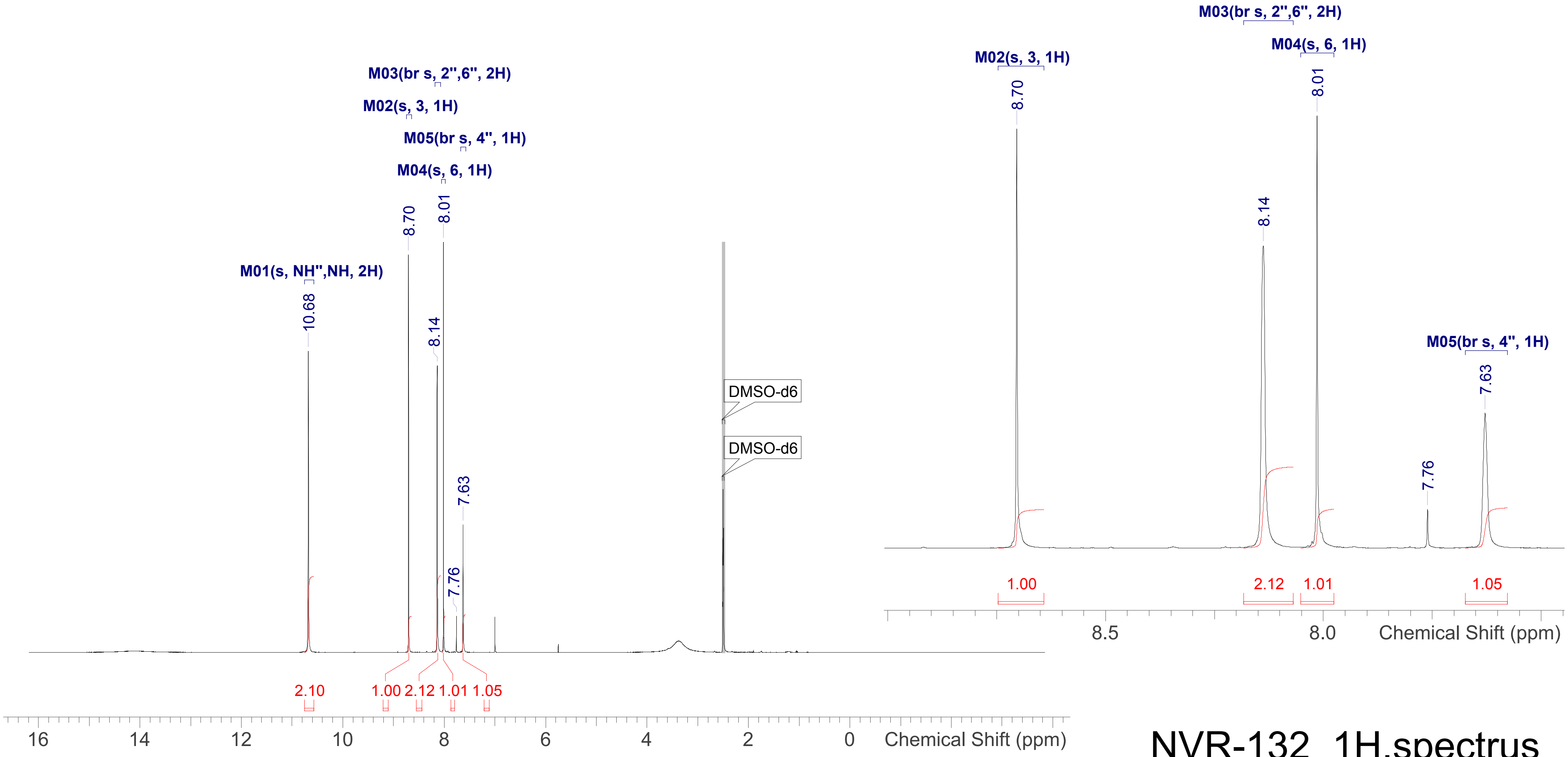

NVR-132_1H.spectrus

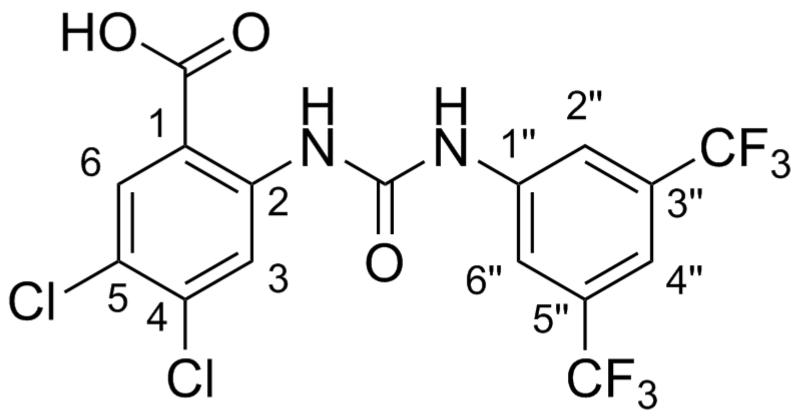

Shift (ppm)	C	m	J (Hz)	Assign
167.8	1	s	-	COOH
151.8	1	s	-	CO
141.5	1	s	-	2
141.2	1	s	-	1"
136.3	1	s	-	4
132.0	1	s	-	6
130.7	2	q	32.3	3", 5"
123.3	2	q	272.9	5"-CF3, 3"-CF3
123.0	1	s	-	5
120.7	1	s	-	3
118.1	2	br q	3.9	2", 6"
115.8	1	s	-	1
114.9	1	br spt	3.9	4"

Acquisition Time (sec)	1.0224
Date	20 Jun 2019 23:03:15
Date Stamp	20 Jun 2019 23:03:15
Frequency (MHz)	100.6128
Nucleus	13C
Number of Transients	256
Solvent	DMSO-d6
Temperature (degree C)	22.700

^{13}C NMR (101 MHz, *DMSO-d$_6$*) δ ppm 167.8 (s, 1 C), 151.8 (s, 1 C), 141.5 (s, 1 C), 141.2 (s, 1 C), 136.3 (s, 1 C), 132.0 (s, 1 C), 130.7 (q, *J*=32.3 Hz, 2 C), 123.0 (s, 1 C), 120.7 (s, 1 C), 123.3 (q, *J*=272.9 Hz, 2 C), 118.1 (br q, *J*=3.9 Hz, 2 C), 115.8 (s, 1 C), 114.9 (br spt, *J*=3.9 Hz, 1 C)

NVR-132_13C

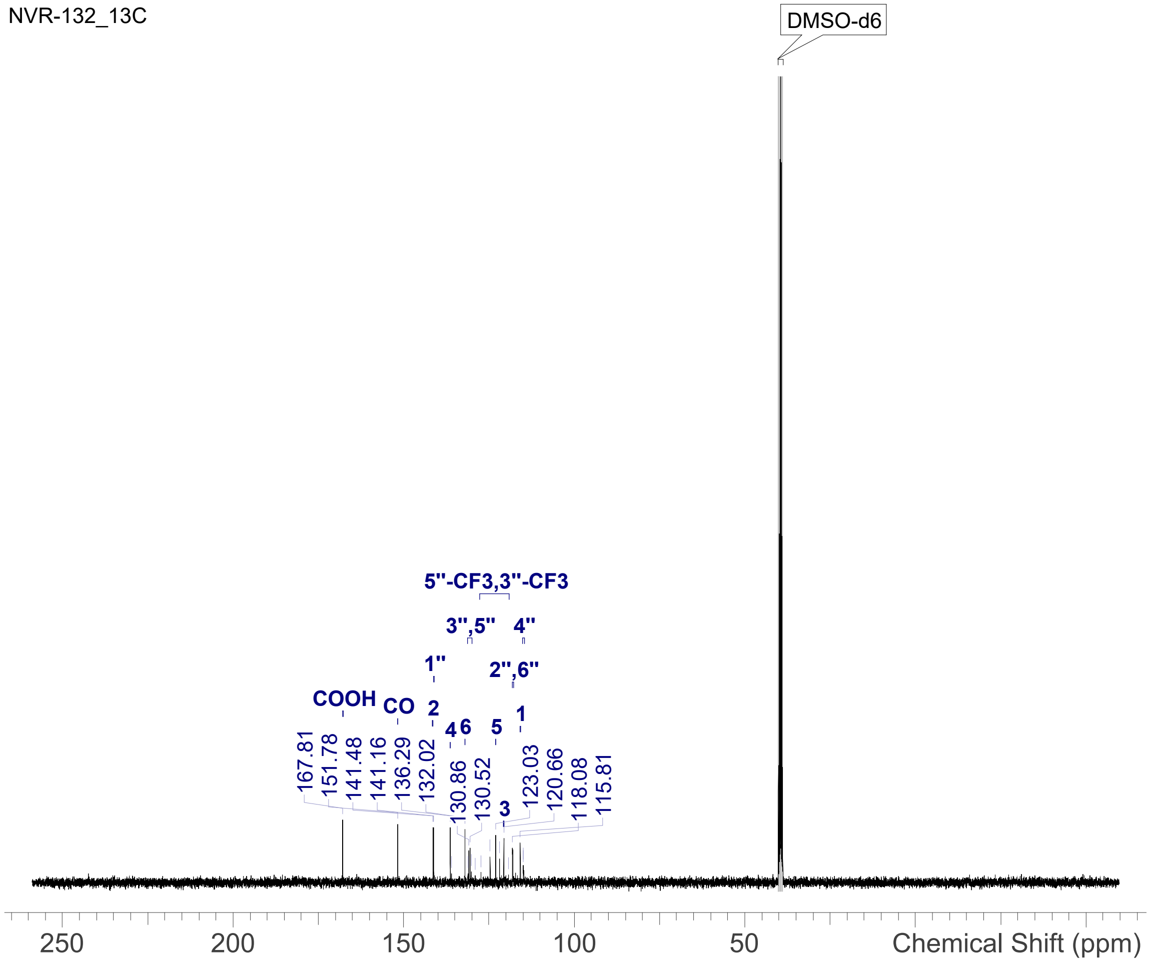

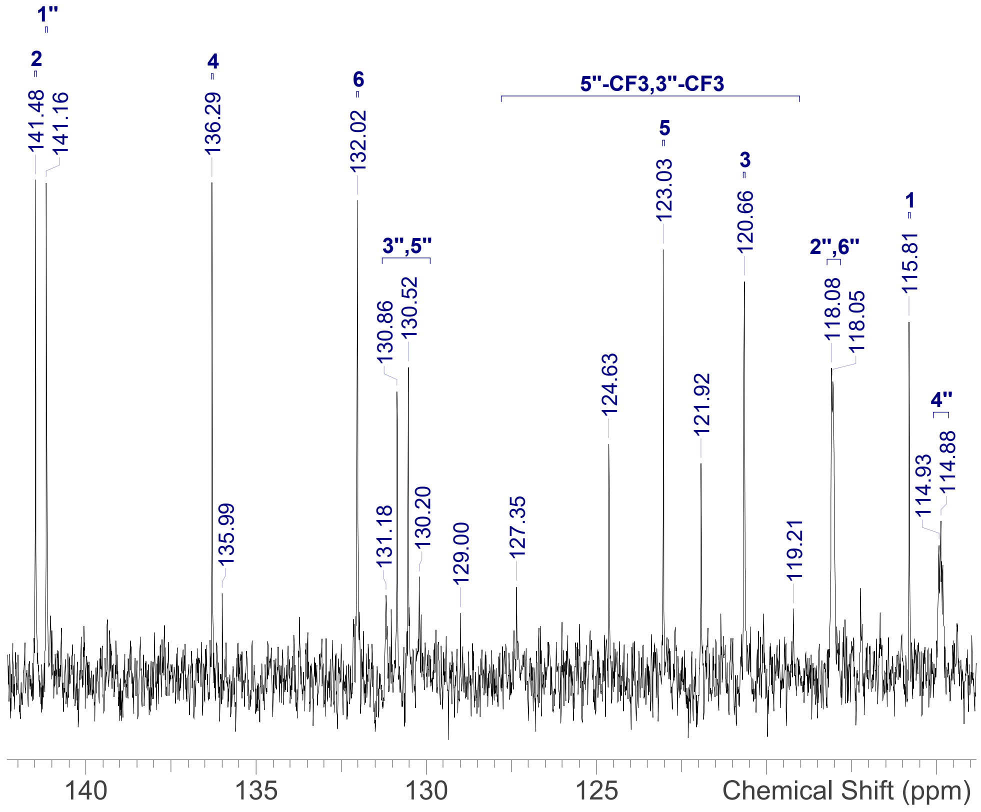

NVR-132_13C.spectrus

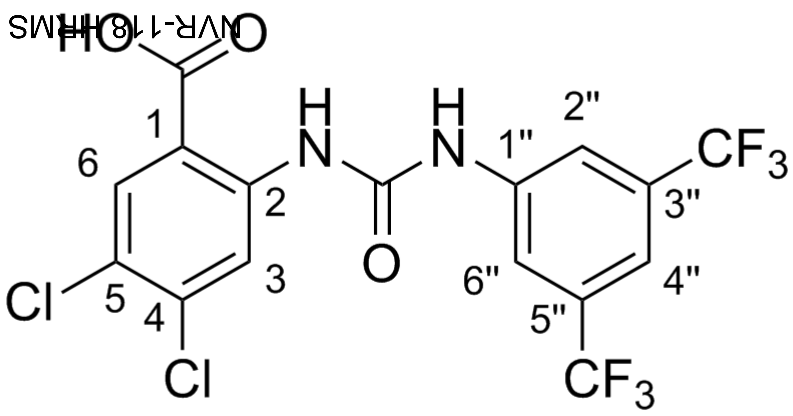

Shift (ppm)	F	m
-61.79	6	s

Acquisition Time (sec)	2.9360
Date	20/06/2019 23:14:00
Date Stamp	20/06/2019 23:14:00
Frequency (MHz)	376.4610
Nucleus	19F
Number of Transients	16
Solvent	DMSO-d6

^{19}F NMR (376 MHz, *DMSO-d_6*) δ ppm -61.79 (s, 6 F)

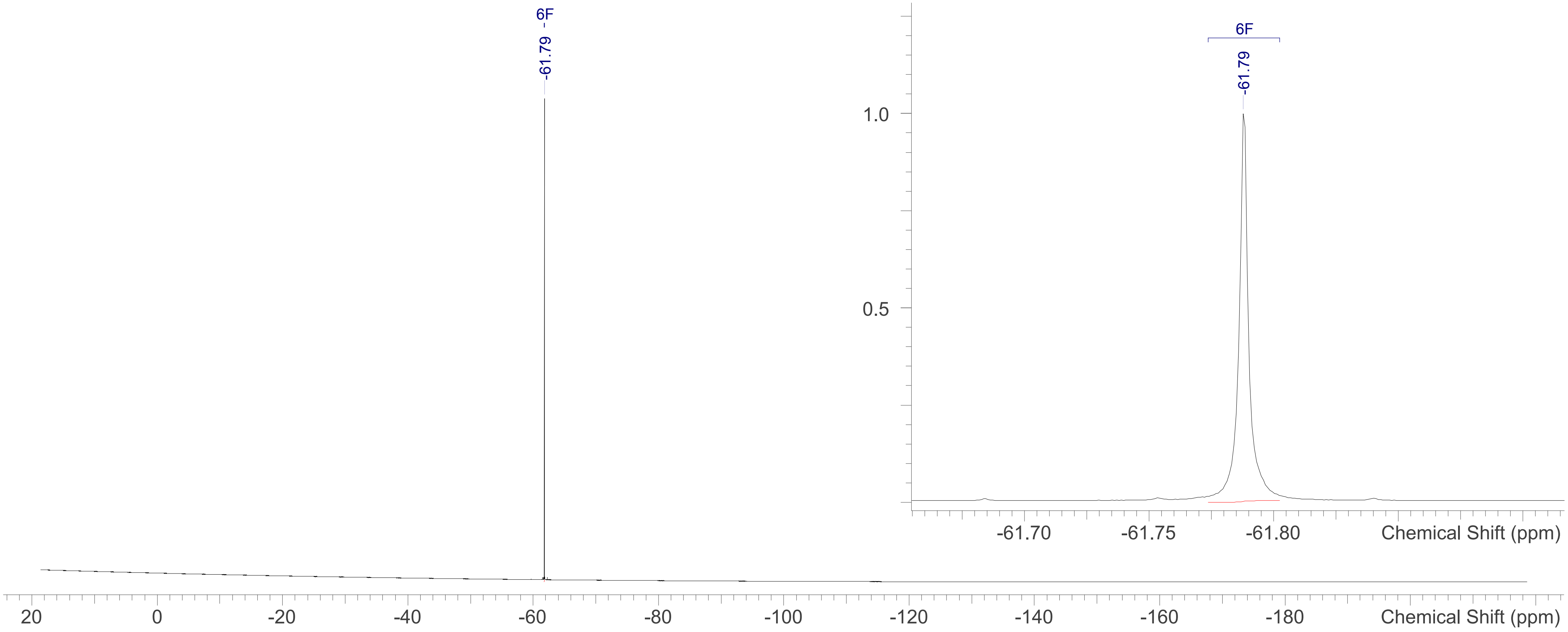

NVR-132_19F.spectrus

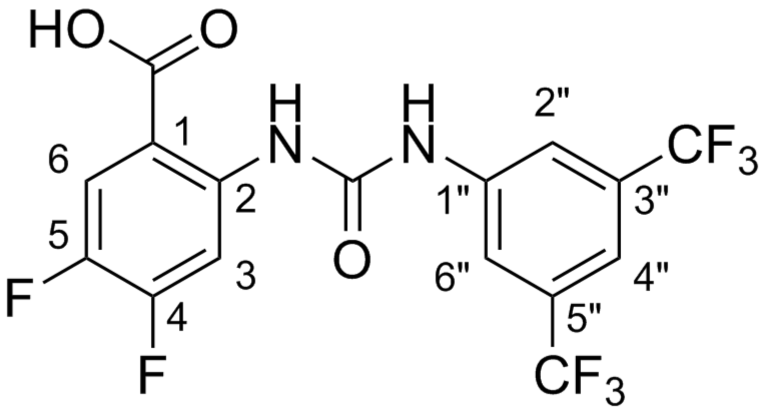

No.	Shift (ppm)	H	m	J (Hz)	Assign
1	13.94	1	br s	-	COOH
2	10.71	1	s	-	NH
3	10.62	1	s	-	NH"
4	8.45	1	dd	14.2, 7.7	3
5	8.14	2	br s	-	6", 2"
6	7.87	1	dd	11.2, 9.4	6
7	7.62	1	br s	-	4"

Acquisition Time (sec)	3.9846
Date	01 Jul 2019 20:29:25
Date Stamp	01 Jul 2019 20:29:25
Frequency (MHz)	400.0700
Nucleus	1H
Number of Transients	16
Solvent	DMSO-d6
Temperature (degree C)	25.001

^{1}H NMR (400 MHz, *DMSO-d_6*) δ ppm 13.94 (br s, 1 H), 10.71 (s, 1 H), 10.62 (s, 1 H), 8.45 (dd, *J*=14.2, 7.7 Hz, 1 H), 8.14 (br s, 2 H), 7.87 (dd, *J*=11.2, 9.4 Hz, 1 H), 7.62 (br s, 1 H)

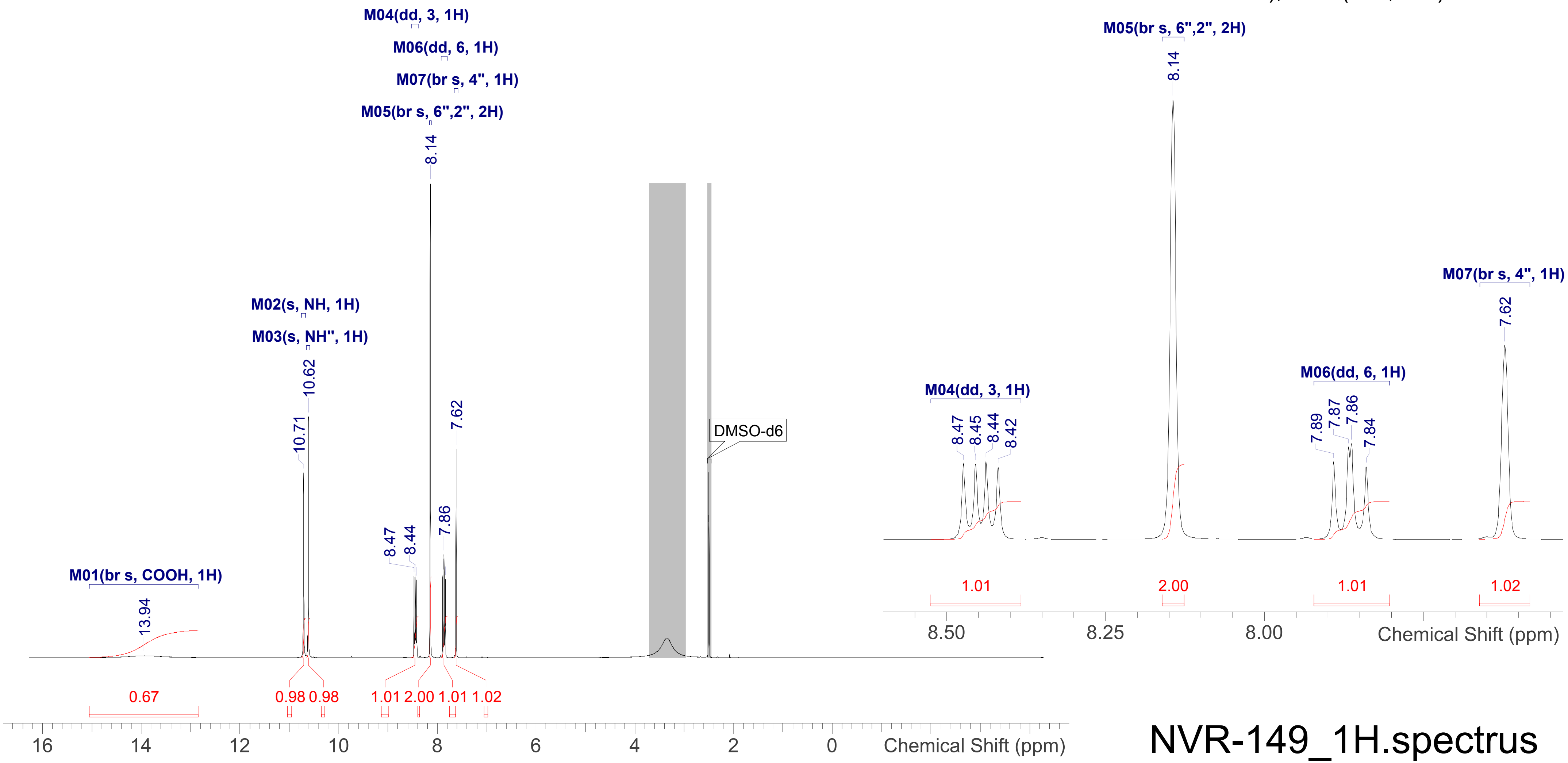

NVR-149_1H.spectrus

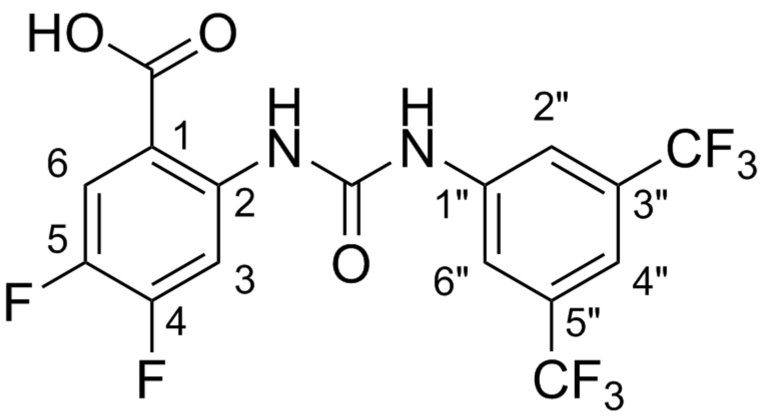

Shift (ppm)	C	m	J (Hz)	Assign
167.9	1	s	-	COOH
152.2	1	dd	250.4, 13.7	4
151.9	1	s	-	CO
143.4	1	dd	242.1, 13.2	5
141.6	1	s	-	1"
139.5	1	dd	11.7, 2.0	2
130.7	2	q	32.3	5", 3"
126.0	2	q	272.9	5"-CF3, 3"-CF3
119.3	1	d	18.6	6
118.0	2	br q	3.9	6", 2"
114.8	1	br spt	3.9	4"
112.2	1	dd	3.5	1
108.2	1	d	24.5	3

Acquisition Time (sec)	1.0224
Date	01 Jul 2019 20:43:40
Date Stamp	01 Jul 2019 20:43:40
Frequency (MHz)	100.5977
Nucleus	13C
Number of Transients	256
Solvent	DMSO-d6
Temperature (degree C)	24.998

^{13}C NMR (101 MHz, *DMSO-d_6*) δ ppm 167.9 (s, 1 C), 151.9 (s, 1 C), 152.2 (dd, *J*=250.4, 13.7 Hz, 1 C), 143.4 (dd, *J*=242.1, 13.2 Hz, 1 C), 141.6 (s, 1 C), 139.5 (dd, *J*=11.7, 2.0 Hz, 1 C), 130.7 (q, *J*=32.3 Hz, 2 C), 126.0 (q, *J*=272.9 Hz, 2 C), 119.3 (d, *J*=18.6 Hz, 1 C), 118.0 (br q, *J*=3.9 Hz, 2 C), 114.8 (br spt, *J*=3.9 Hz, 1 C), 112.2 (dd, *J*=3.5 Hz, 1 C), 108.2 (d, *J*=24.5 Hz, 1 C)

NVR-149_13C

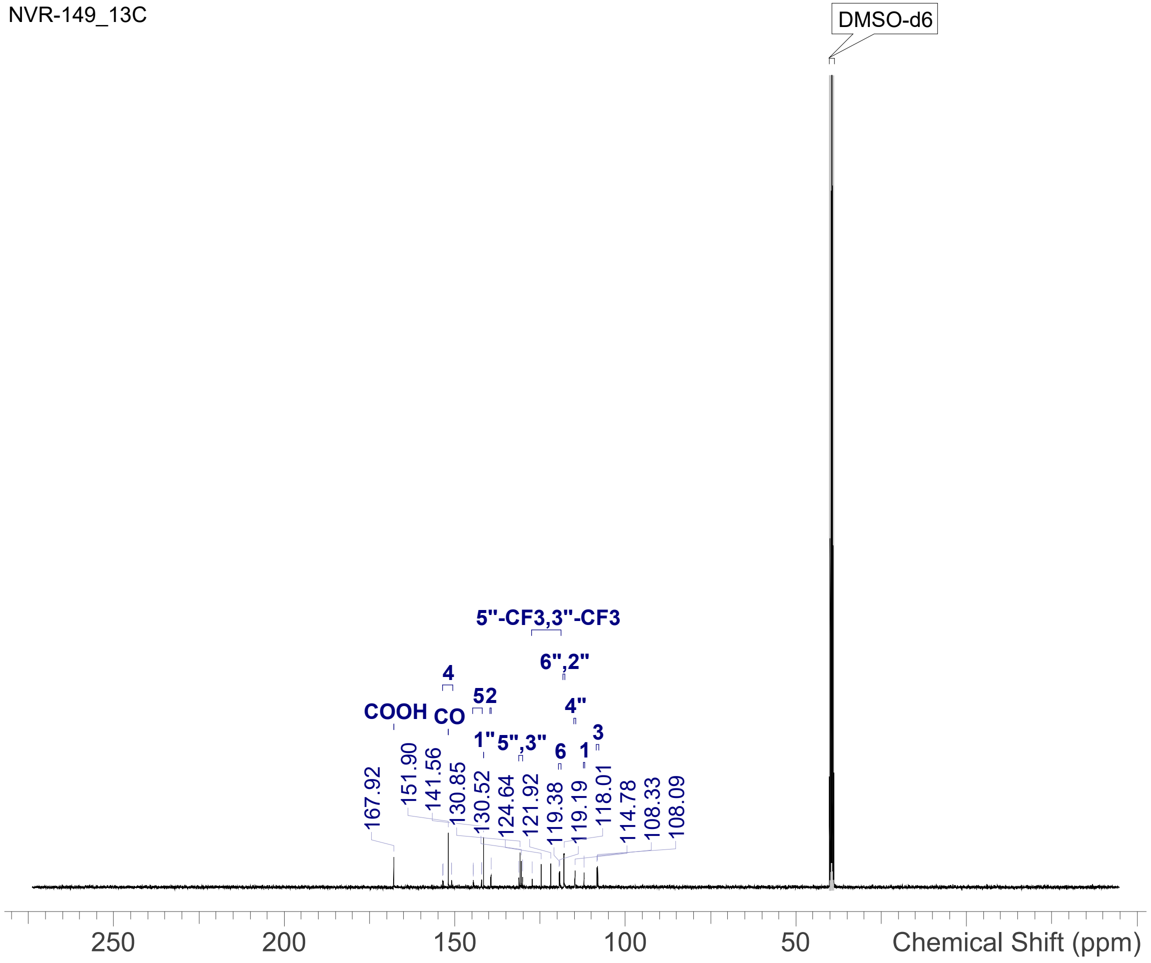

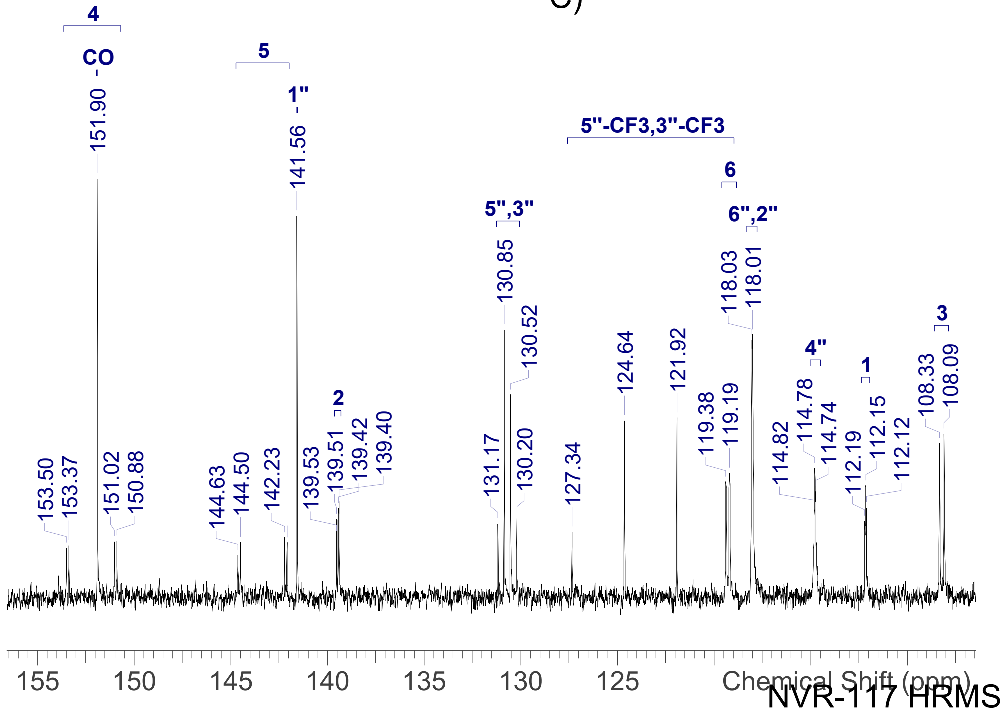

NVR-117 HRMS

NVR-149_13C.spectrus

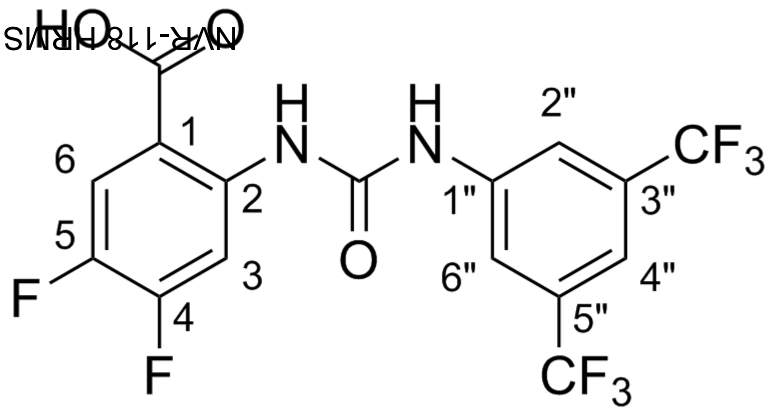

Shift (ppm)	F	m	J (Hz)
-61.83	6	s	-
-128.41	1	dd	14.0, 9.5
-128.47	1	dd	14.0, 9.2

Acquisition Time (sec)	1.4680
Date	01 Jul 2019 21:49:47
Date Stamp	01 Jul 2019 21:49:47
Frequency (MHz)	376.4419
Nucleus	19F
Number of Transients	16
Solvent	DMSO-d6
Temperature (degree C)	24.999

^{19}F NMR (376 MHz, *DMSO-d_6*) δ ppm -61.83 (s, 6 F), -128.41 (dd, *J*=14.0, 9.5 Hz, 1 F), -128.47 (dd, *J*=14.0, 9.2 Hz, 1 F)

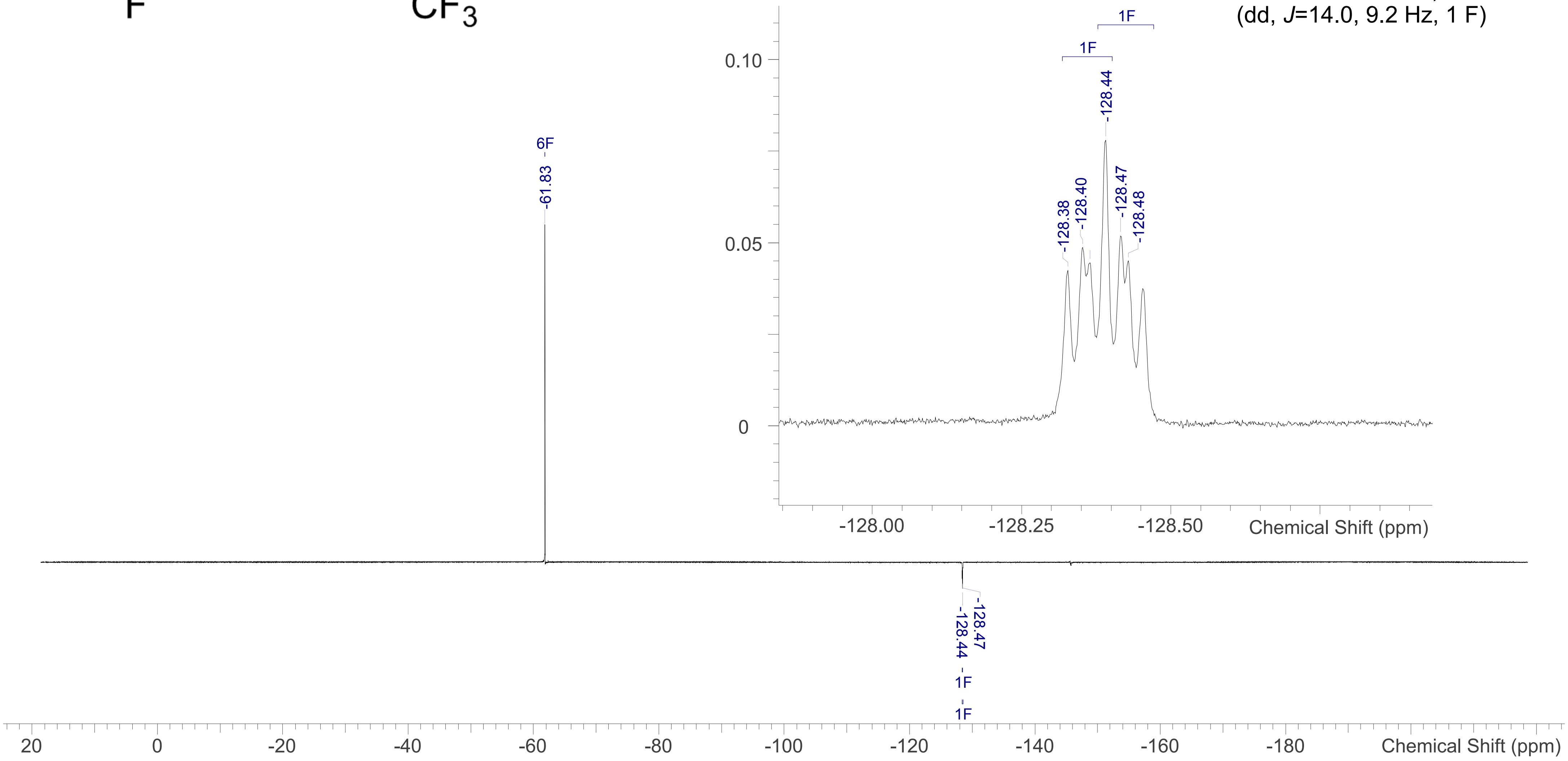

NVR-149_19F.spectrus

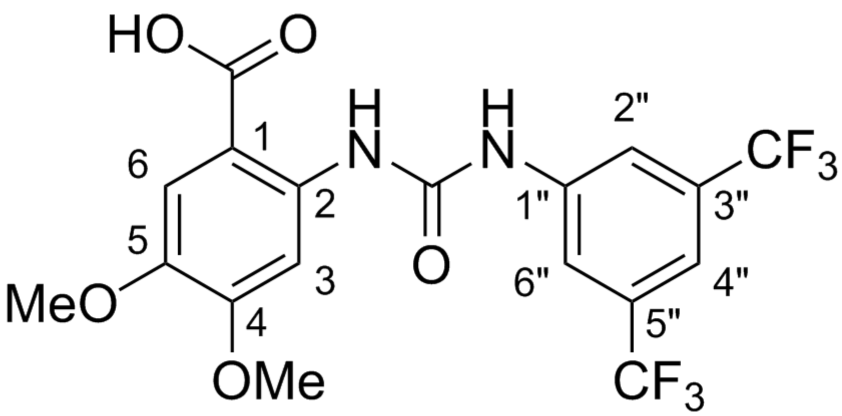

Shift (ppm)	H	m	Assign
13.26	1	br s	COOH
10.75	1	s	NH
10.49	1	s	NH"
8.20	2	br s	2", 6"
8.16	1	s	3
7.63	1	br s	4"
7.42	1	s	6
3.84	3	s	OMe
3.75	3	s	OMe

Acquisition Time (sec)	3.9846
Date	05 Jul 2019 01:21:41
Date Stamp	05 Jul 2019 01:21:41
Frequency (MHz)	400.0700
Nucleus	1H
Number of Transients	4
Solvent	DMSO-d6
Temperature (degree C)	25.000

^{1}H NMR (400 MHz, *DMSO-d$_6$*) δ ppm 13.26 (br s, 1 H), 10.75 (s, 1 H), 10.49 (s, 1 H), 8.20 (br s, 2 H), 8.16 (s, 1 H), 7.63 (br s, 1 H), 7.42 (s, 1 H), 3.84 (s, 3 H), 3.75 (s, 3 H)

NVR-150_1H

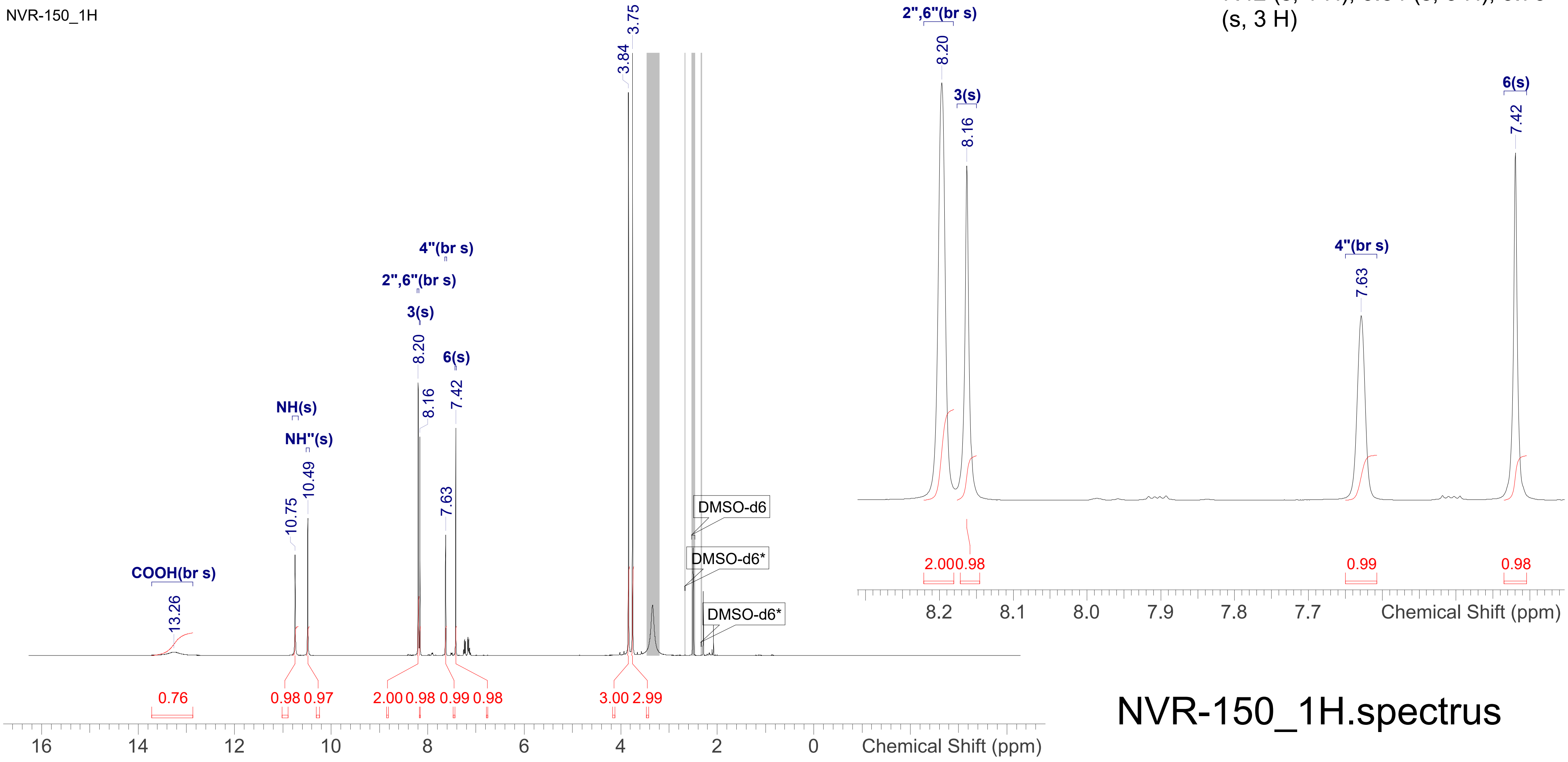

NVR-150_1H.spectrus

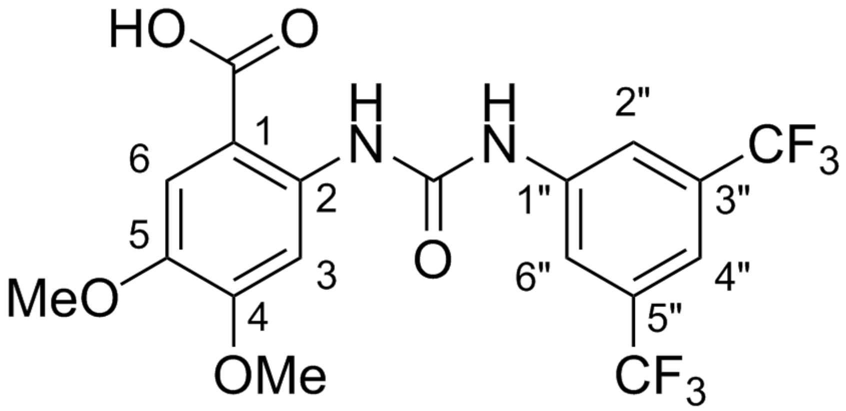

Shift (ppm)	C	m	J (Hz)	Assign
169.3	1	s	-	COOH
153.3	1	s	-	4
152.2	1	s	-	CO
142.9	1	s	-	5
142.0	1	s	-	1"
137.6	1	s	-	2
130.7	2	q	32.6	3", 5"
123.4	2	q	272.9	5"-CF3, 3"-CF3
117.9	2	br q	3.9	2", 6"
114.4	1	br spt	3.9	4"
112.7	1	s	-	6
106.7	1	s	-	1
102.8	1	s	-	3
55.6	2	s	-	OMe, OMe

Acquisition Time (sec)	1.0224
Date	05 Jul 2019 01:38:19
Date Stamp	05 Jul 2019 01:38:19
Frequency (MHz)	100.5977
Nucleus	13C
Number of Transients	256
Solvent	DMSO-d6
Temperature (degree C)	25.000

^{13}C NMR (101 MHz, *DMSO-d_6*) δ ppm 169.3 (s, 1 C), 153.3 (s, 1 C), 152.2 (s, 1 C), 142.9 (s, 1 C), 142.0 (s, 1 C), 137.6 (s, 1 C), 130.7 (q, *J*=32.6 Hz, 2 C), 123.4 (q, *J*=272.9 Hz, 2 C), 117.9 (br q, *J*=3.9 Hz, 2 C), 114.4 (br spt, *J*=3.9 Hz, 1 C), 112.7 (s, 1 C), 106.7 (s, 1 C), 102.8 (s, 1 C), 55.6 (s, 2 C)

NVR-150_13C

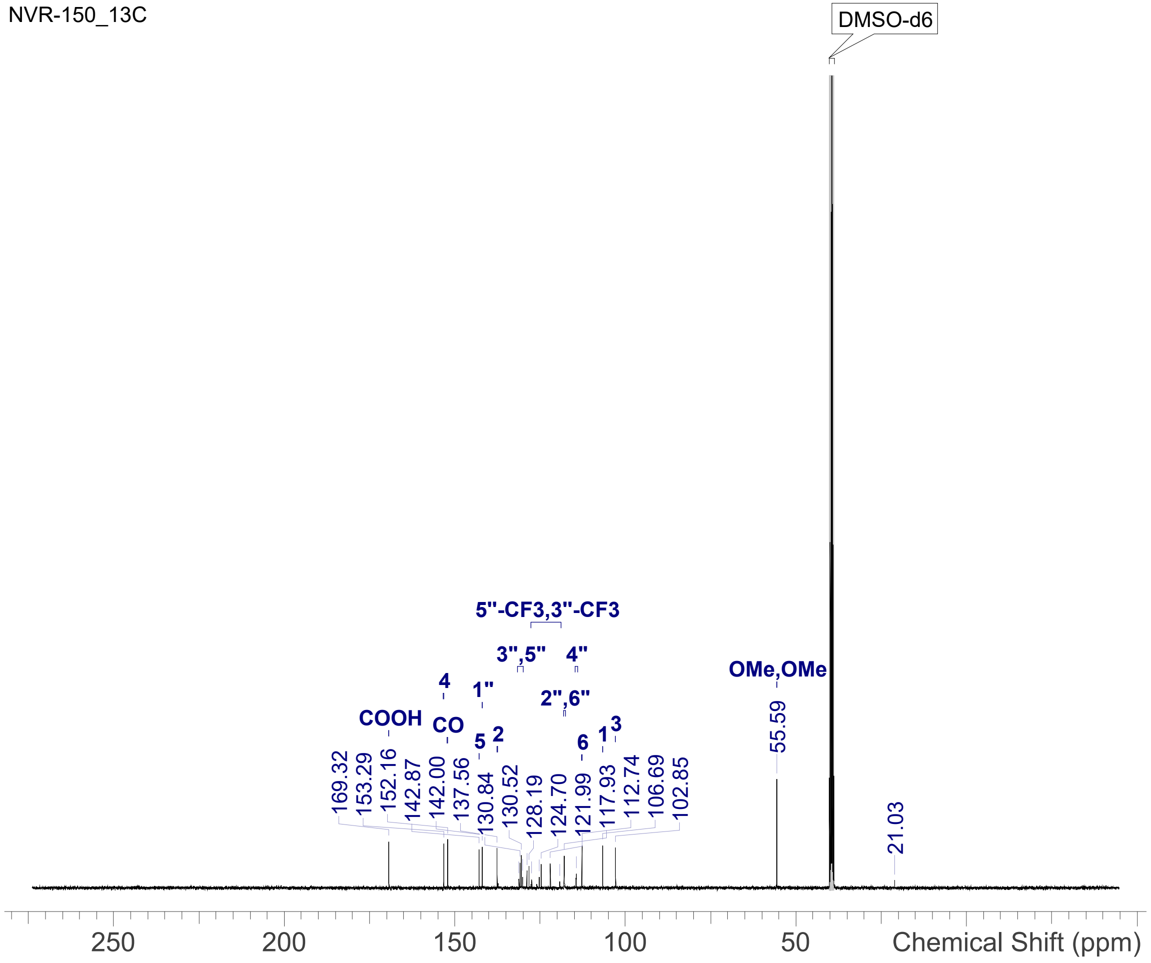

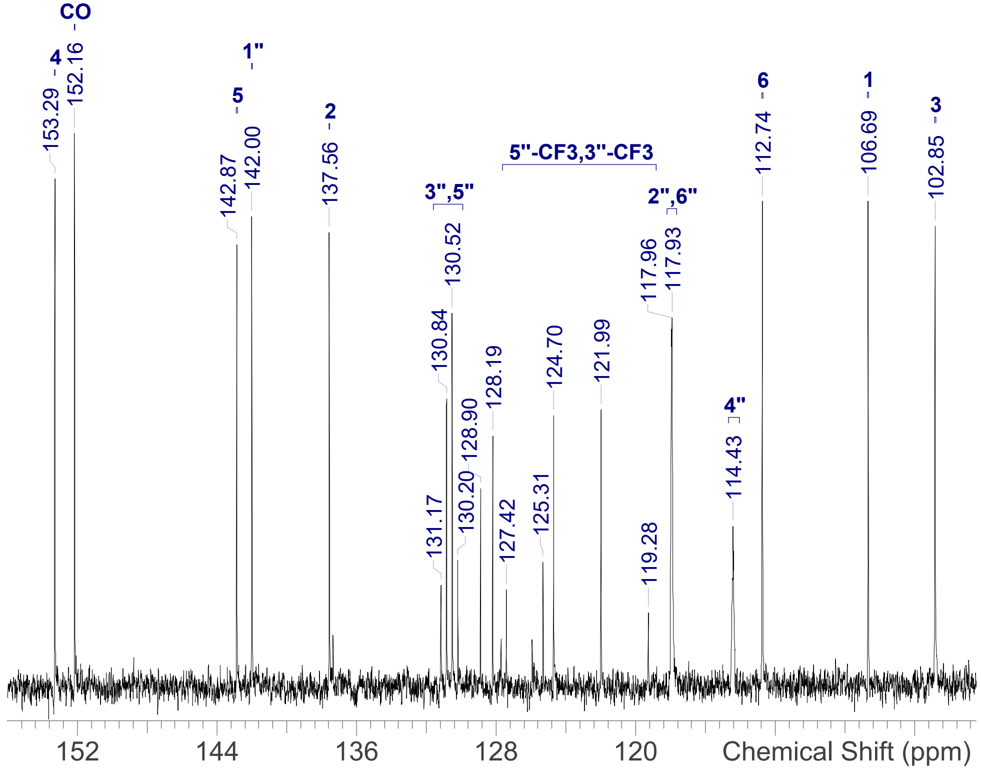

NVR-150_13C.spectrus

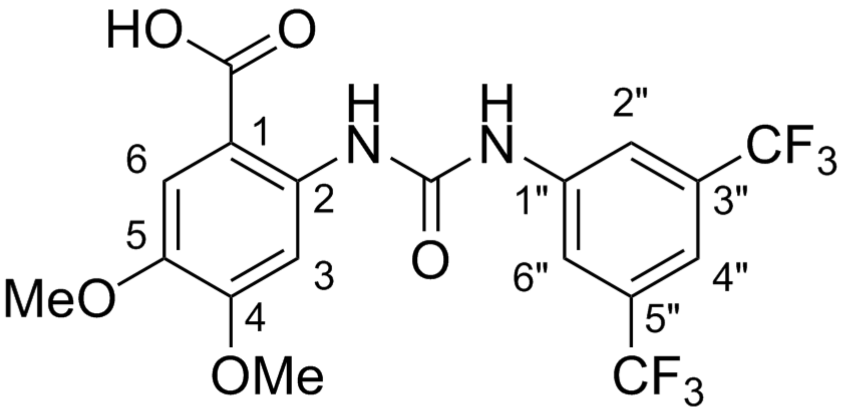

Shift (ppm)	F	m
-61.72	6	s

Acquisition Time (sec)	1.4680
Date	05 Jul 2019 02:44:24
Date Stamp	05 Jul 2019 02:44:24
Frequency (MHz)	376.4419
Nucleus	19F
Number of Transients	16
Solvent	DMSO-d6
Temperature (degree C)	24.999

^{19}F NMR (376 MHz, *DMSO-d_6*) δ ppm -61.72 (s, 6 F)

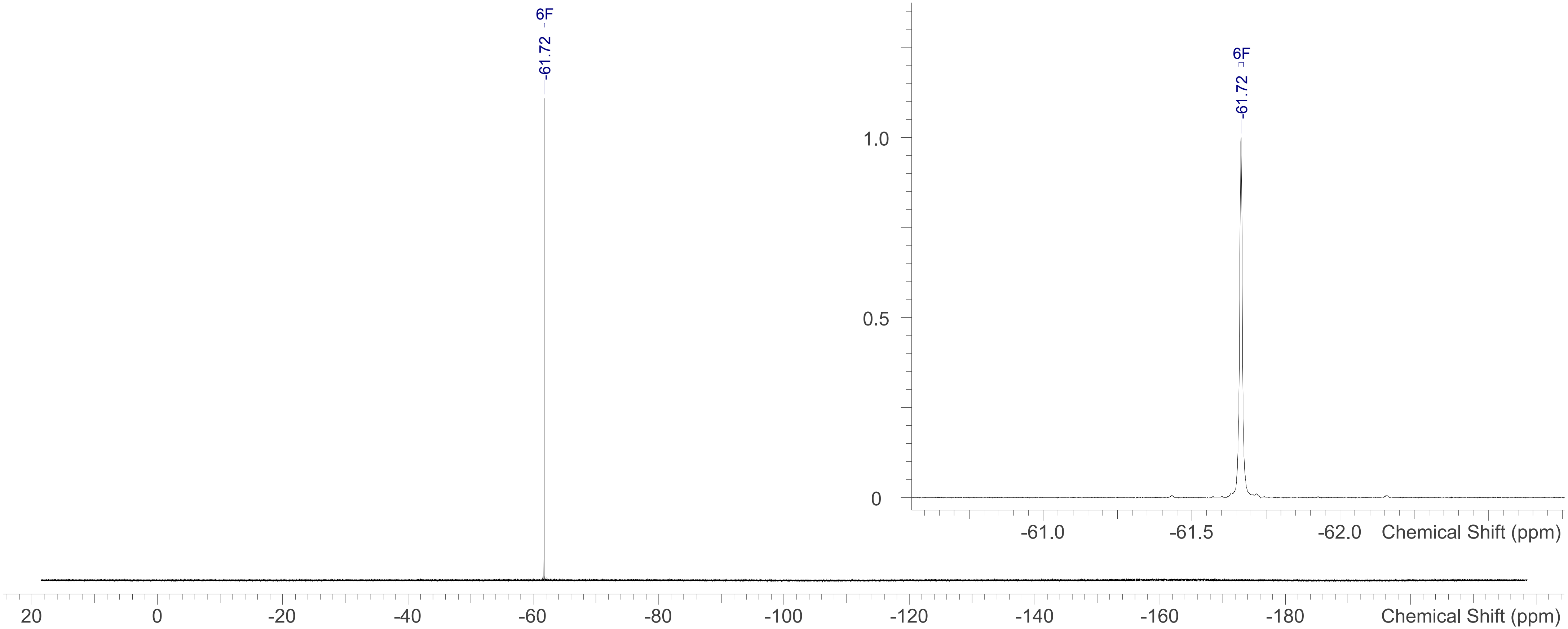

NVR-150_19F.spectrus

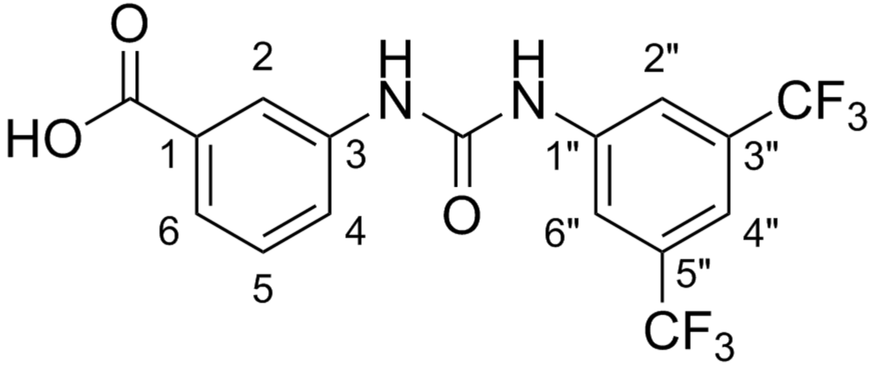

No.	Shift (ppm)	H	m	J (Hz)	Assign
1	13.03	1	s	-	COOH
2	9.47	1	s	-	NH"
3	9.28	1	s	-	NH
4	8.16	3	m	-	2", 6", 2
5	7.67	2	s	-	4", 4
6	7.59	1	br d	7.8	6
7	7.43	1	t	7.9	5

Acquisition Time (sec)	3.9846
Date	25 Mar 2019 23:00:30
Date Stamp	25 Mar 2019 23:00:30
Frequency (MHz)	400.0700
Nucleus	1H
Number of Transients	16
Solvent	DMSO-d6
Temperature (degree C)	-273.000

^{1}H NMR (400 MHz, *DMSO-d_6*) δ ppm 13.03 (s, 1 H), 9.47 (s, 1 H), 9.28 (s, 1 H), 8.13 - 8.18 (m, 3 H), 7.67 (s, 2 H), 7.59 (br d, *J*=7.8 Hz, 1 H), 7.43 (t, *J*=7.9 Hz, 1 H)

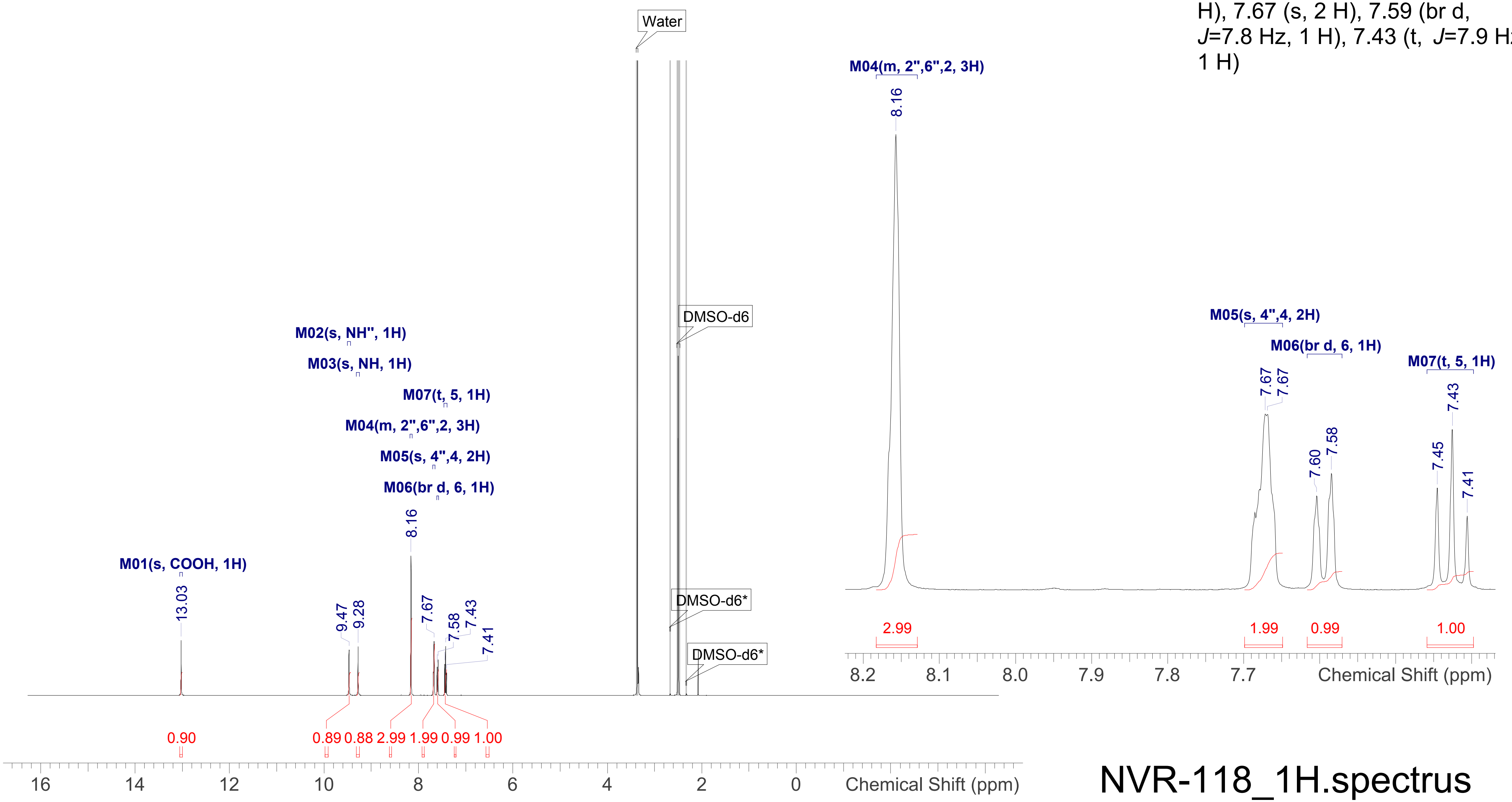

NVR-118_1H.spectrus

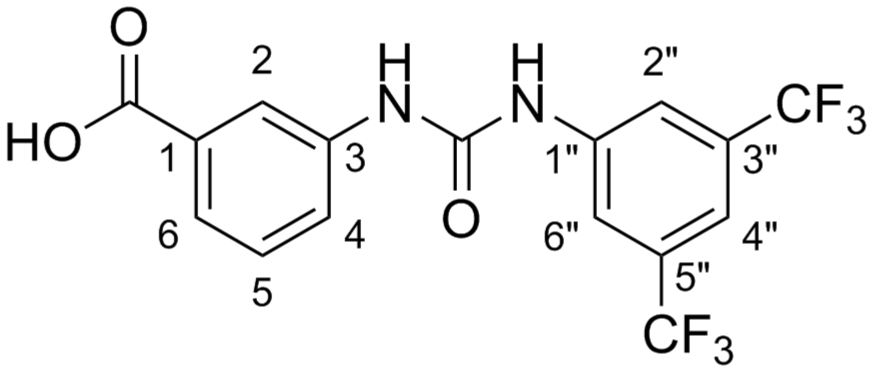

Shift (ppm)	C	m	J (Hz)	Assign
167.2	1	s	-	COOH
152.5	1	s	-	CO
141.8	1	s	-	1"
139.4	1	s	-	3
131.4	1	s	-	1
130.7	2	q	33.3	3", 5"
129.0	1	s	-	5
123.4	1	s	-	6
123.3	2	q	272.9	5"-CF3, 3"-CF3
123.1	1	s	-	4
119.6	1	s	-	2
118.2	2	br q	3.9	2", 6"
114.5	1	br spt	3.9	4"

Acquisition Time (sec)	1.0224
Date	08 May 2019 18:16:35
Date Stamp	08 May 2019 18:16:35
Frequency (MHz)	100.5977
Nucleus	13C
Number of Transients	256
Solvent	DMSO-d6
Temperature (degree C)	25.000

^{13}C NMR (101 MHz, *DMSO-d_6*) δ ppm 167.2 (s, 1 C), 152.5 (s, 1 C), 141.8 (s, 1 C), 139.4 (s, 1 C), 131.4 (s, 1 C), 130.7 (q, *J*=33.3 Hz, 2 C), 129.0 (s, 1 C), 123.4 (s, 1 C), 123.1 (s, 1 C), 119.6 (s, 1 C), 123.3 (q, *J*=272.9 Hz, 2 C), 118.2 (br q, *J*=3.9 Hz, 2 C), 114.5 (br spt, *J*=3.9 Hz, 1 C)

NVR-118_13C

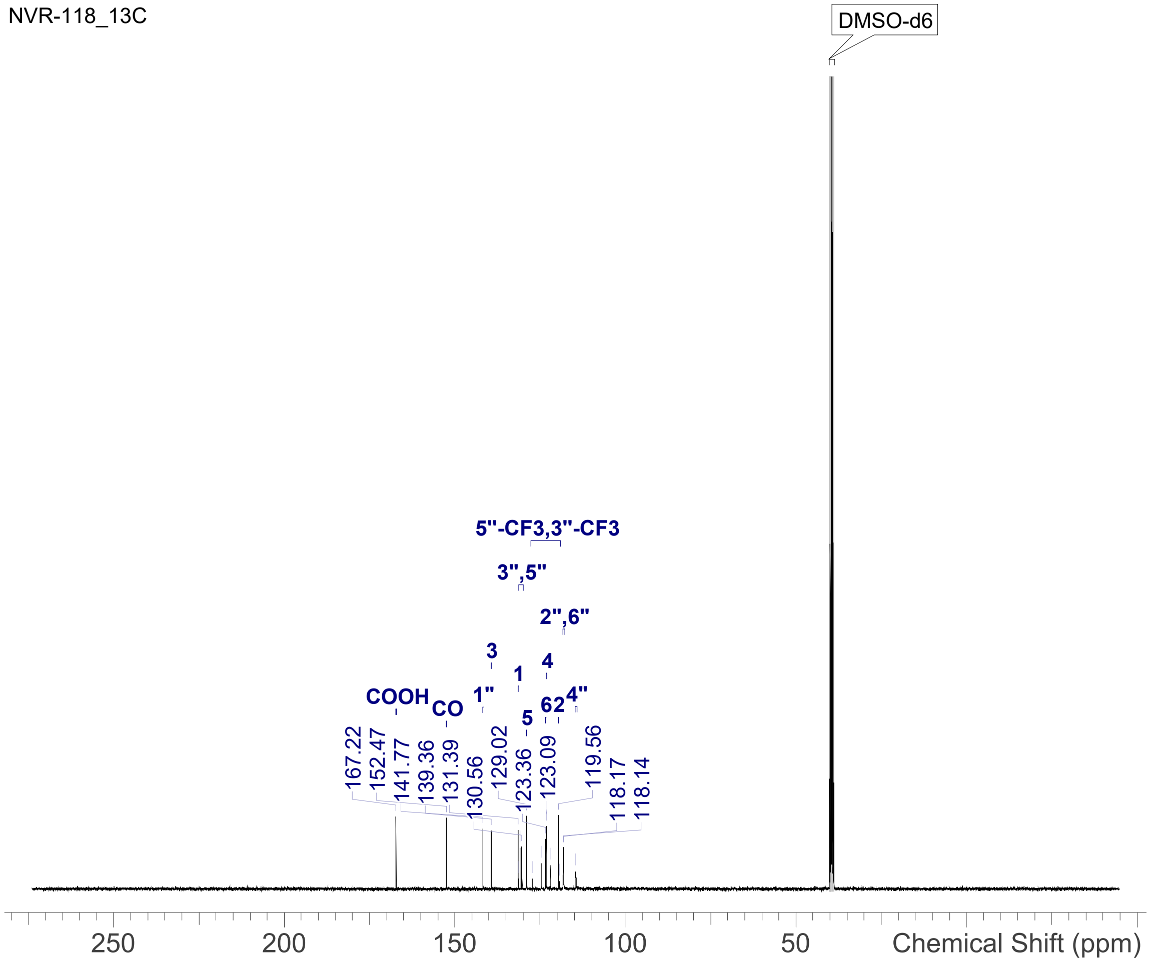

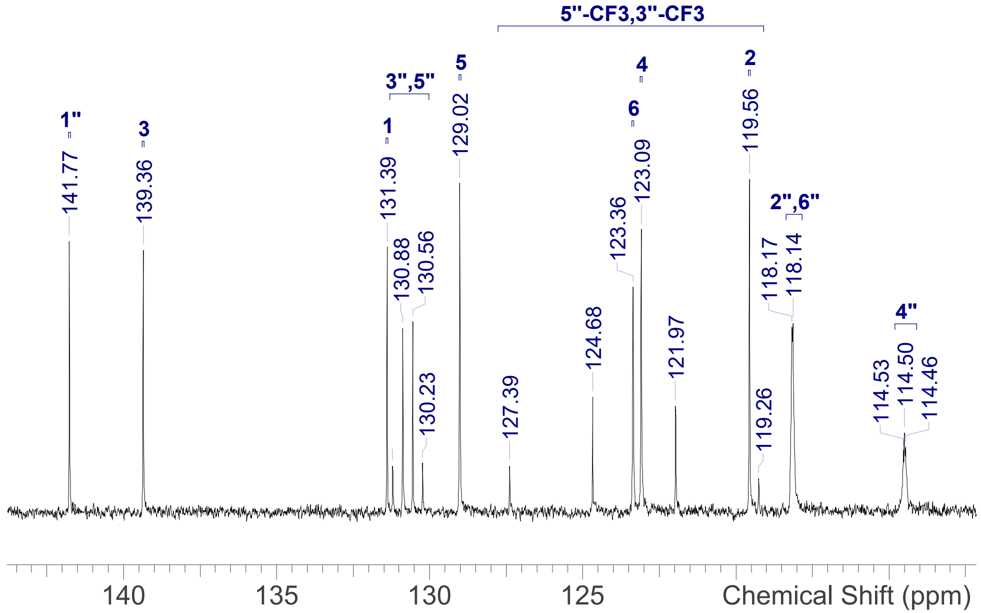

NVR-118_13C.spectrus

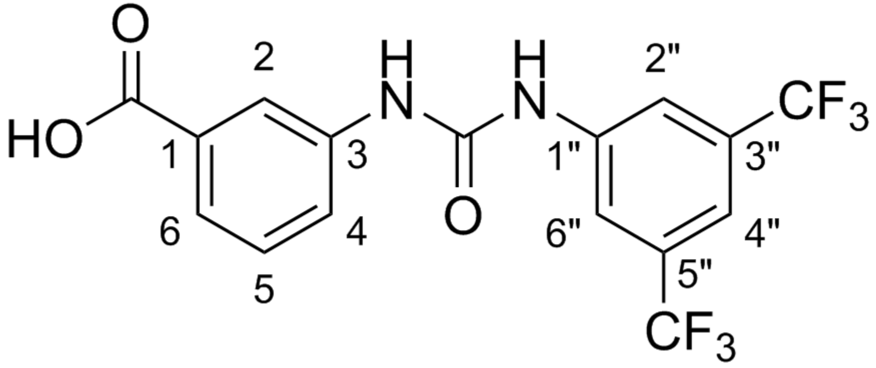

Shift (ppm)	F	m
-61.60	6	s

Acquisition Time (sec)	1.4680
Date	26 Mar 2019 00:20:55
Date Stamp	26 Mar 2019 00:20:55
Frequency (MHz)	376.4419
Nucleus	19F
Number of Transients	16
Solvent	DMSO-d6
Temperature (degree C)	-273.000

^{19}F NMR (376 MHz, *DMSO-d_6*) δ ppm -61.60 (s, 6 F)

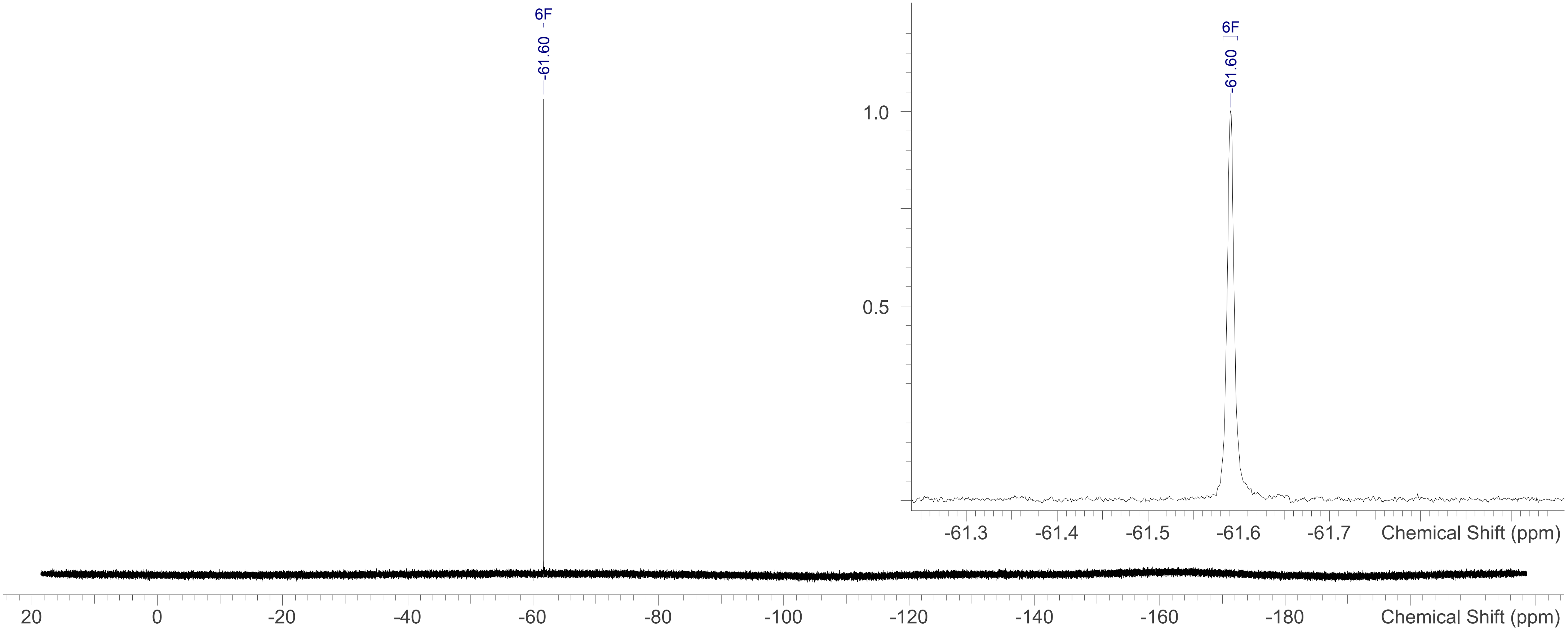

NVR-118_19F.spectrus

Openlynx Report - James B

Page 1

Sample: 1
File:JB149 HRMS-1
Description:

Vial:2:22
Date:02-Oct-2019

ID:JB149 HRMS-1
Time:12:43:29

Printed: Wed Oct 02 12:56:03 2019

3: UV Detector: TAC: Wavelength Range: (210 - 400)

1.348e+2
Range: 1.446e+2

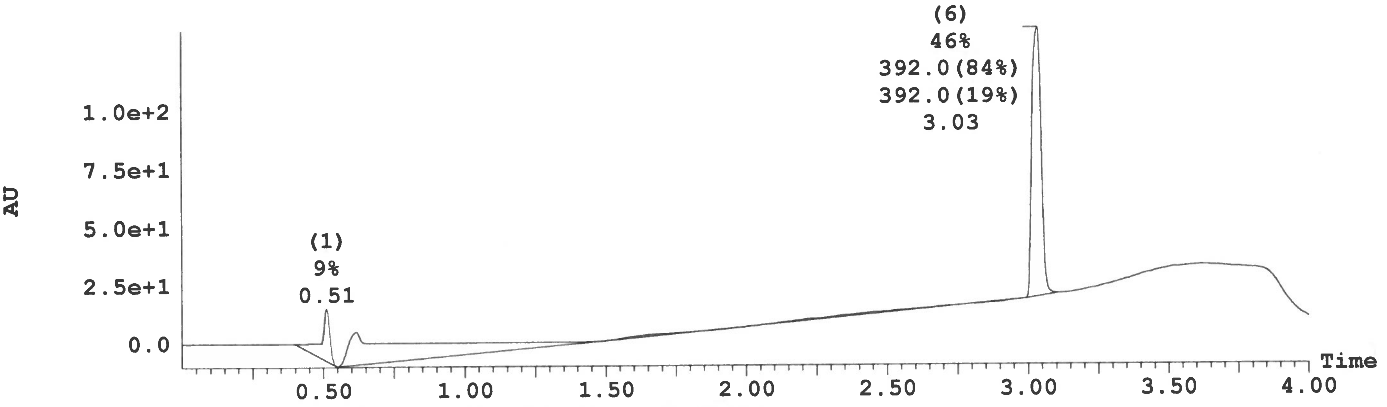

Peak Number	Compound	Time	Area %Total	Mass Found
1		0.51	8.58	Not Found
2		0.62	45.70	Not Found
6	Found	3.03	45.71	392.0, 392.0

1: MS ES+ :TIC Smooth (Mn, 2x2)

5.8e+006

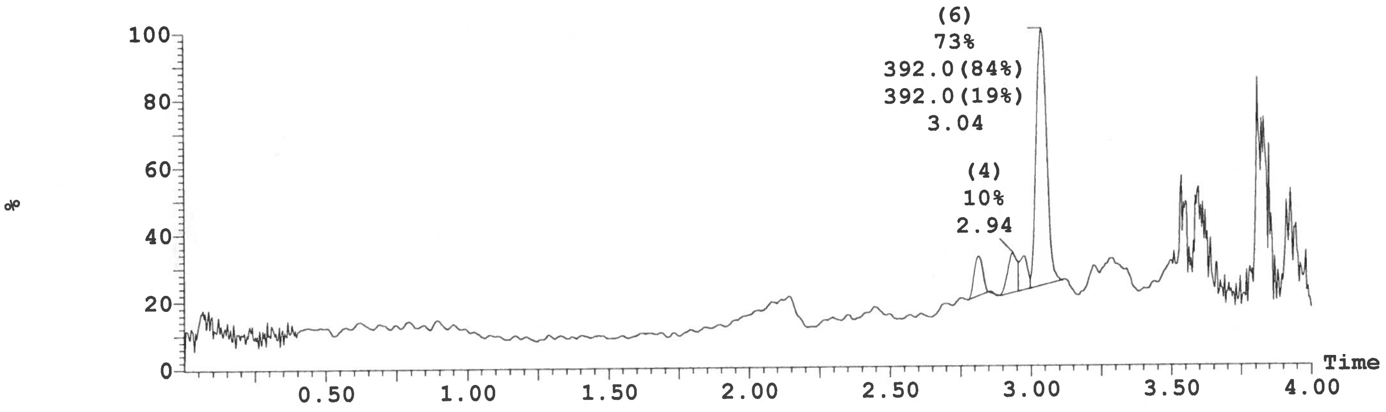

2: MS ES- :TIC Smooth (Mn, 2x2)

3.7e+006

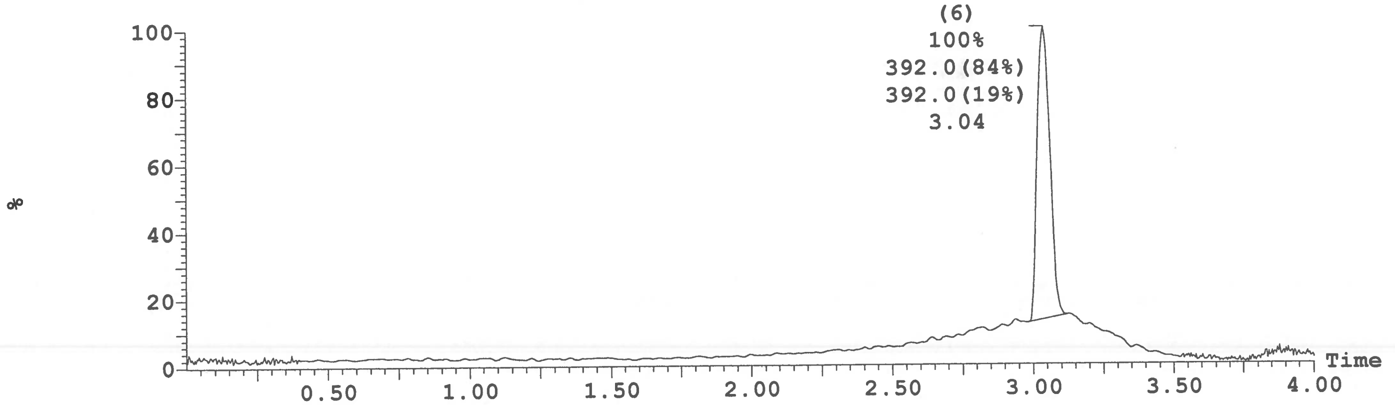

NVR-118 LCMS

Openlynx Report - James B

Sample: 1 | Vial:2:22 | ID:JB149 HRMS-1

File:JB149 HRMS-1 | Date:02-Oct-2019 | Time:12:43:29

Description:

Printed: Wed Oct 02 12:56:03 2019

Peak ID	Compound	Time	Mass Found
1		0.51	Not Found

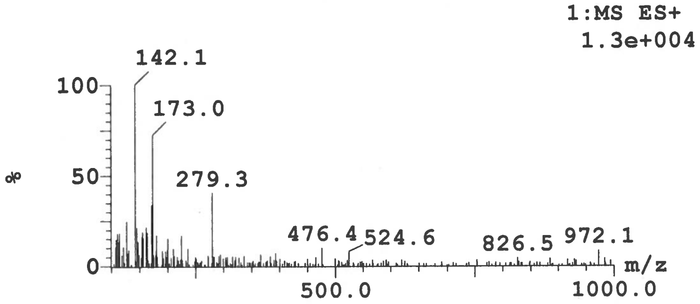

Peak ID	Compound	Time	Mass Found
1		0.51	Not Found

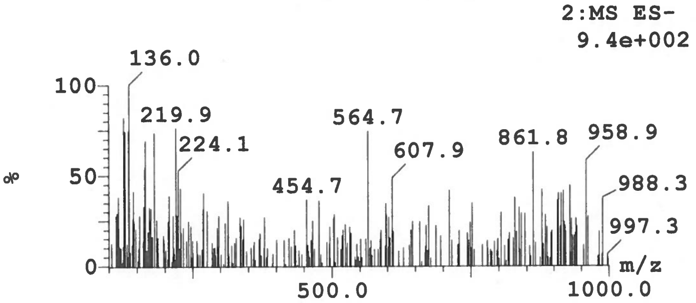

Peak ID	Compound	Time	Mass Found
1		0.51	Not Found

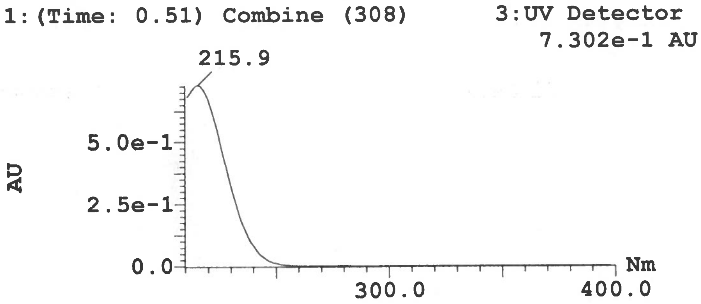

Peak ID	Compound	Time	Mass Found
2		0.62	Not Found

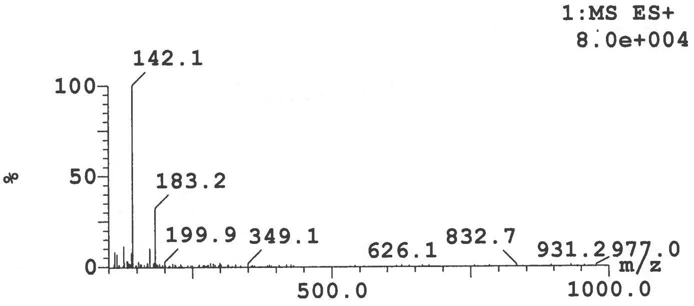

Peak ID	Compound	Time	Mass Found
2		0.62	Not Found

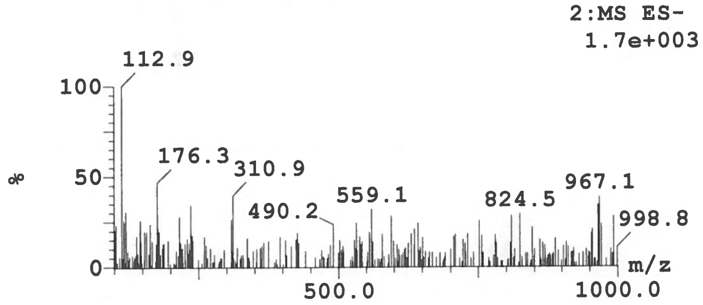

Peak ID	Compound	Time	Mass Found
2		0.62	Not Found

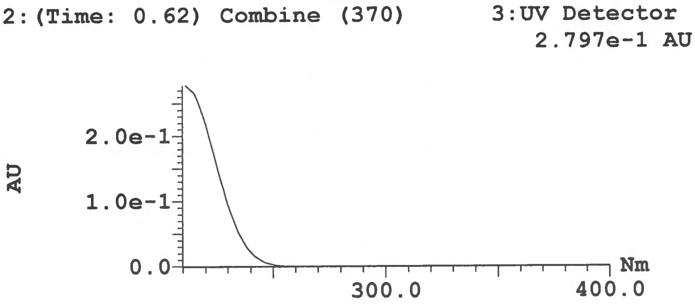

Peak ID	Compound	Time	Mass Found
6	Found	3.04	393

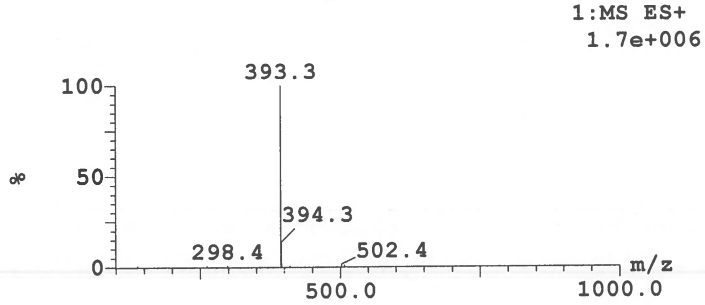

Peak ID	Compound	Time	Mass Found
6	Tentative	3.04	391

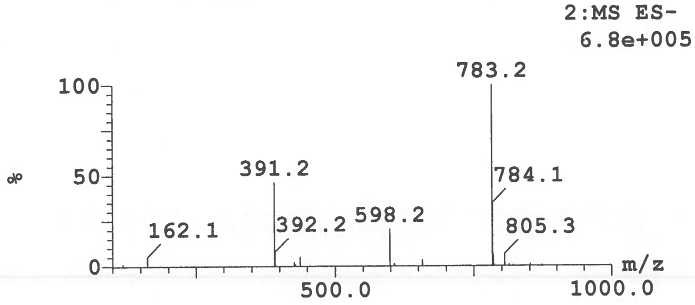

NVR-118 LCMS

Peak ID	Compound	Time	Mass Found
6		3.04	Not Found

6:(Time: 3.03) Combine (1821) 3:UV Detector
2.47 AU

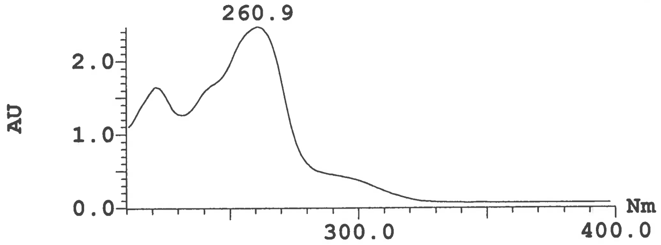

Thermo Exactive Plus EMR Orbitrab HESI pos

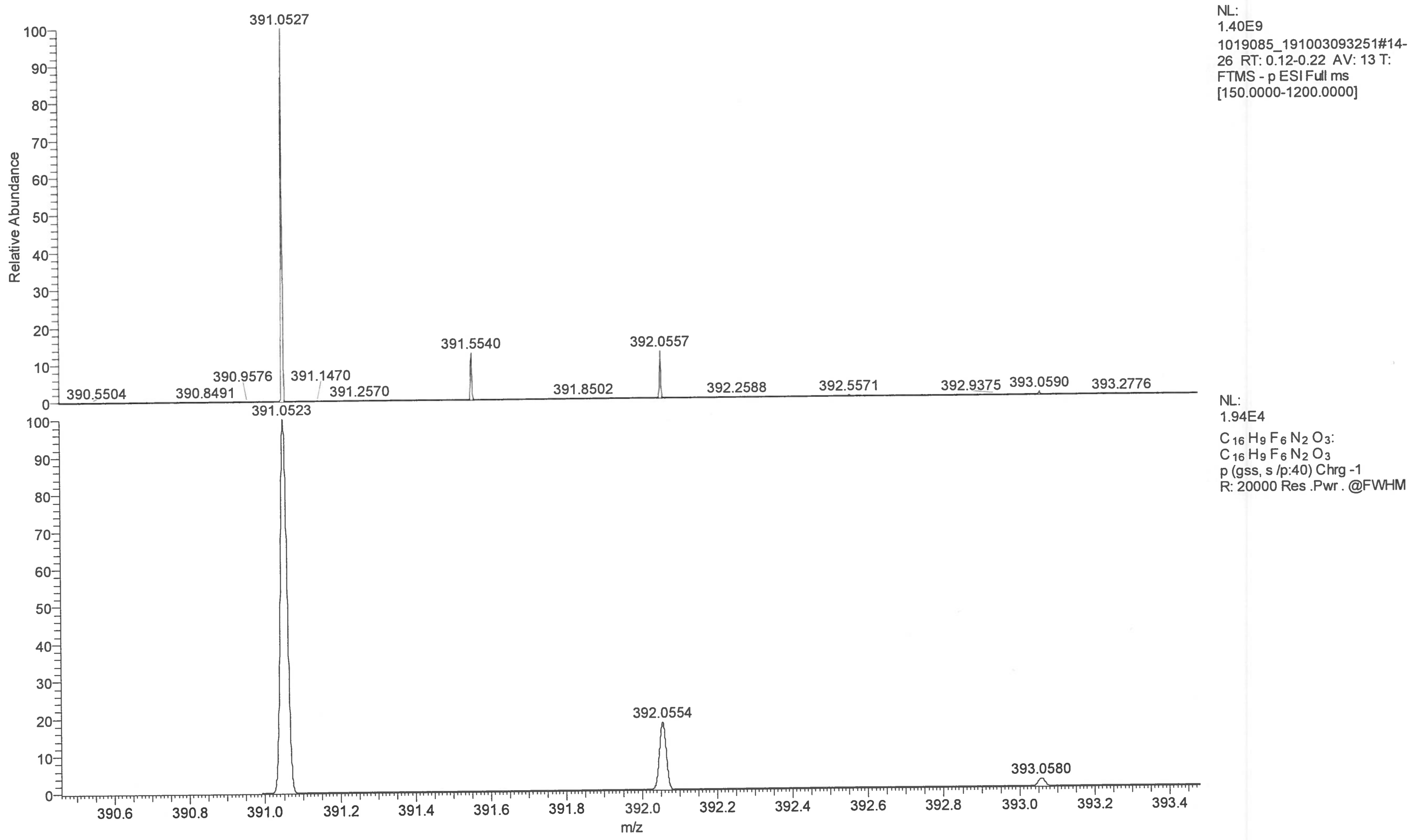

NVR-118 HRMS

Thermo Exactive Plus EMR Orbitrab HESI pos

m/z	Theo. Mass	Delta (ppm)	RDB equiv.	Composition
391.0527	391.0523	1.06	10.5	C16 H9 O3 N2 F6

NVR-118 HRMS

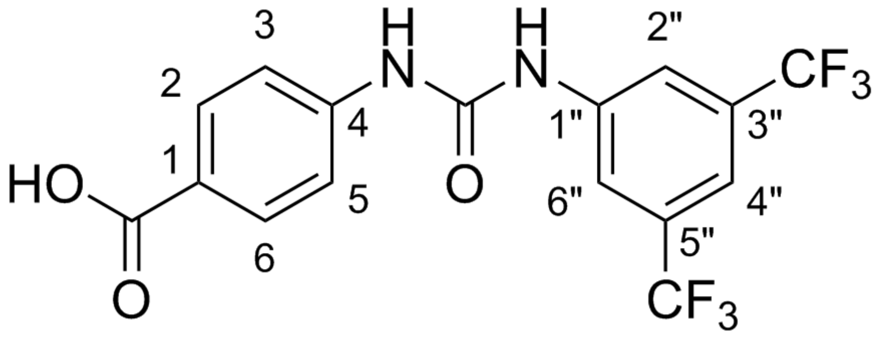

No.	Shift (ppm)	H	m	J (Hz)	Assign
1	12.65	1	br s	-	COOH
2	9.50	1	s	-	NH"
3	9.38	1	s	-	NH
4	8.15	2	br s	-	6", 2"
5	7.89	2	br d	8.8	6, 2
6	7.67	1	br s	-	4"
7	7.61	2	br d	8.8	3, 5

Acquisition Time (sec)	3.9846
Date	27 Mar 2019 21:55:09
Date Stamp	27 Mar 2019 21:55:09
Frequency (MHz)	400.0700
Nucleus	1H
Number of Transients	16
Solvent	DMSO-d6
Temperature (degree C)	25.000

^{1}H NMR (400 MHz, *DMSO-d_6*) δ ppm 12.65 (br s, 1 H), 9.50 (s, 1 H), 9.38 (s, 1 H), 8.15 (br s, 2 H), 7.89 (br d, *J*=8.8 Hz, 2 H), 7.67 (br s, 1 H), 7.61 (br d, *J*=8.8 Hz, 2 H)

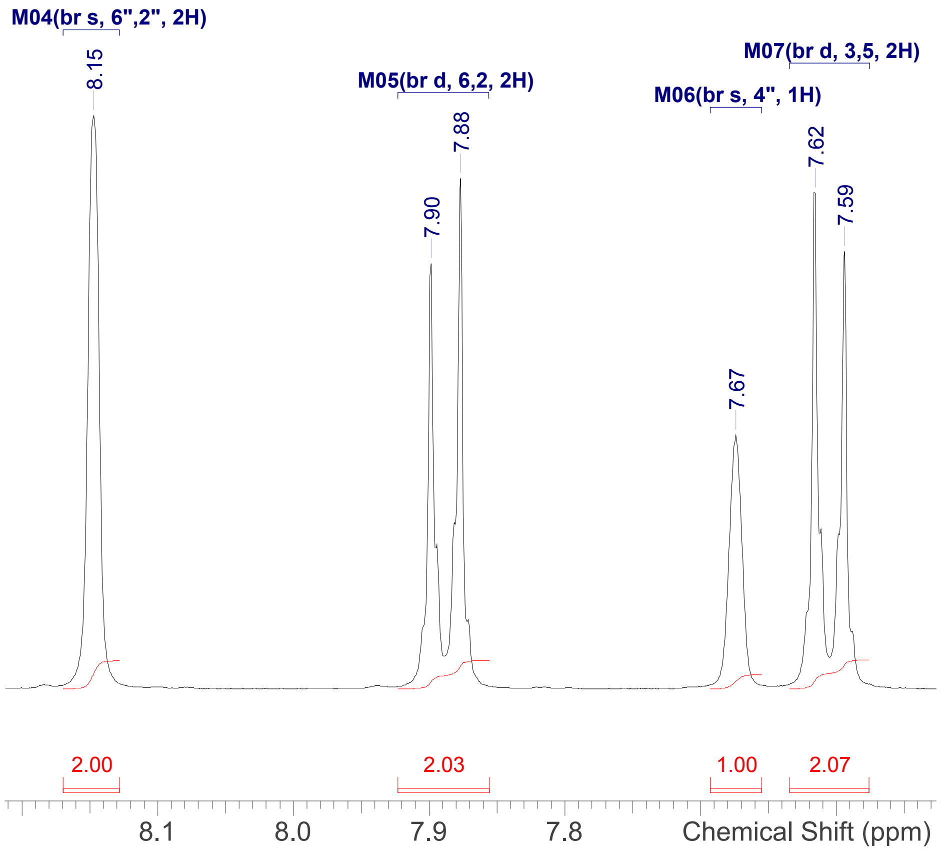

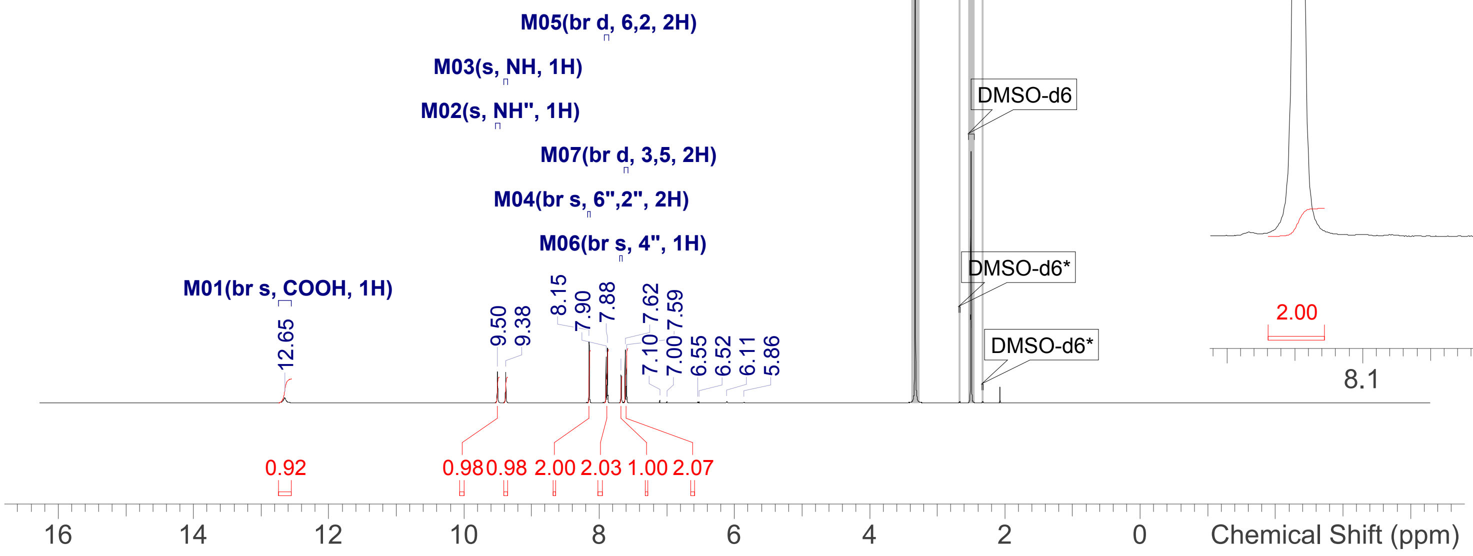

NVR-117_1H.spectrus

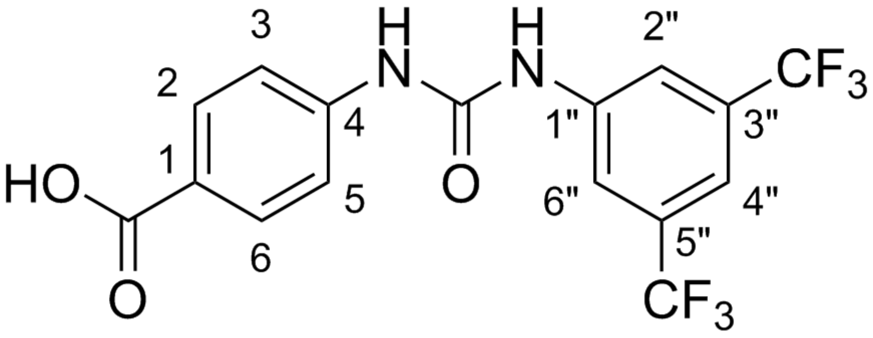

Shift (ppm)	C	m	J (Hz)	Assign
167.0	1	s	-	COOH
152.2	1	s	-	CO
143.3	1	s	-	4
141.6	1	s	-	1"
130.8	2	q	32.3	3", 5"
130.5	2	s	-	2, 6
124.3	1	s	-	1
123.3	2	q	272.9	5"-CF3, 3"-CF3
118.2	2	br q	3.9	2", 6"
117.9	2	s	-	5, 3
114.7	1	br spt	3.9	4"

Acquisition Time (sec)	1.0224
Date	09 May 2019 18:40:39
Date Stamp	09 May 2019 18:40:39
Frequency (MHz)	100.5977
Nucleus	13C
Number of Transients	256
Solvent	DMSO-d6
Temperature (degree C)	25.001

^{13}C NMR (101 MHz, *DMSO-d_6*) δ ppm 167.0 (s, 1 C), 152.2 (s, 1 C), 143.3 (s, 1 C), 141.6 (s, 1 C), 130.5 (s, 2 C), 130.8 (q, *J*=32.3 Hz, 2 C), 124.3 (s, 1 C), 123.3 (q, *J*=272.9 Hz, 2 C), 118.2 (br q, *J*=3.9 Hz, 2 C), 117.9 (s, 2 C), 114.7 (br spt, *J*=3.9 Hz, 1 C)

NVR-117_13C

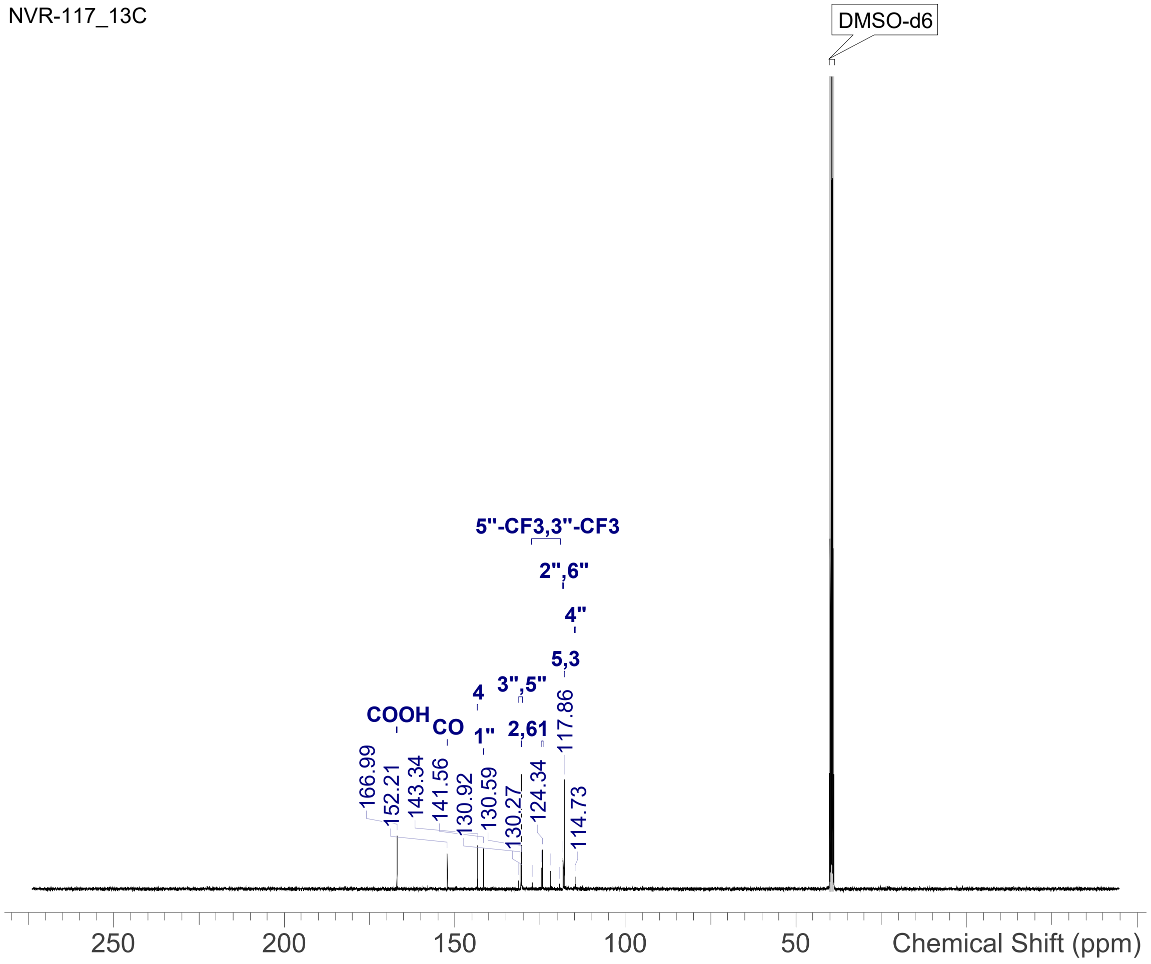

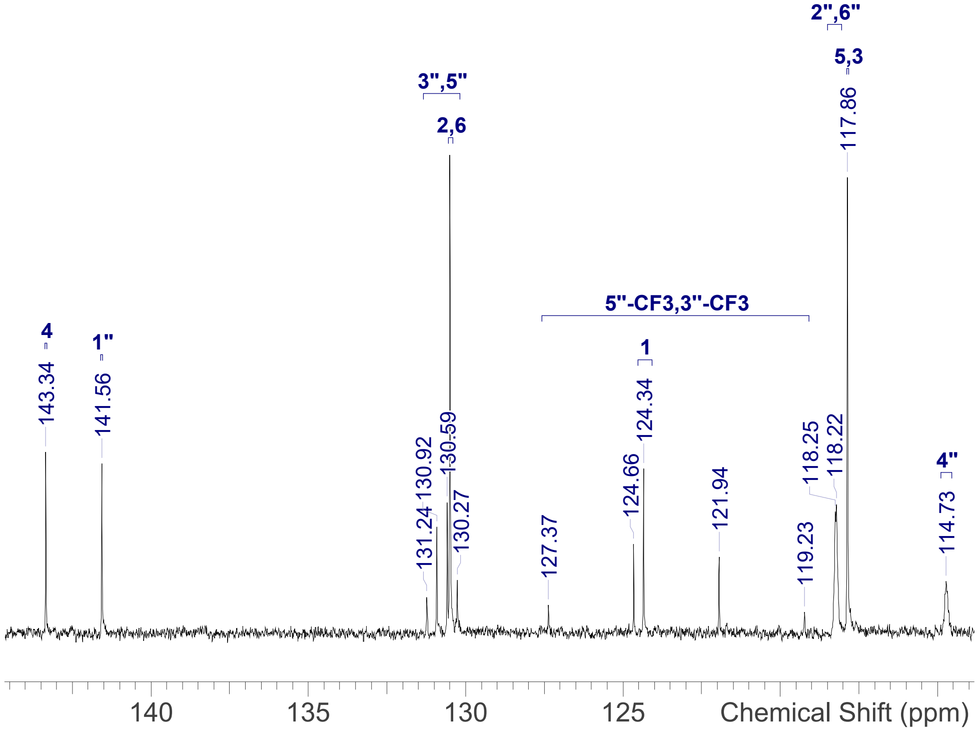

NVR-117_13C.spectrus

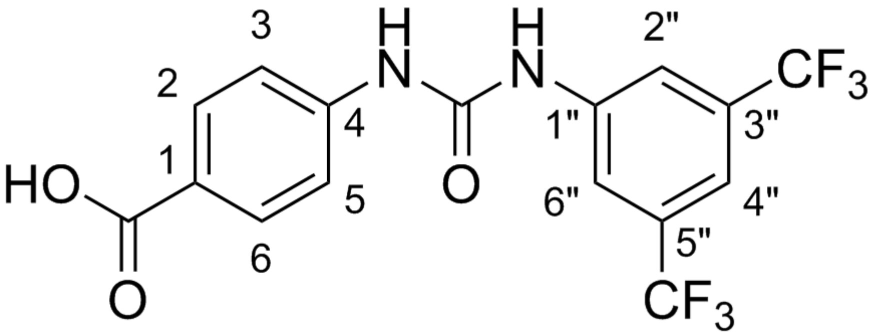

Shift (ppm)	F	m
-61.61	6	s

Acquisition Time (sec)	1.4680
Date	25 Mar 2019 22:56:18
Date Stamp	25 Mar 2019 22:56:18
Frequency (MHz)	376.4419
Nucleus	19F
Number of Transients	16
Solvent	DMSO-d6
Temperature (degree C)	-273.000

^{19}F NMR (376 MHz, *DMSO-d_6*) δ ppm -61.61 (s, 6 F)

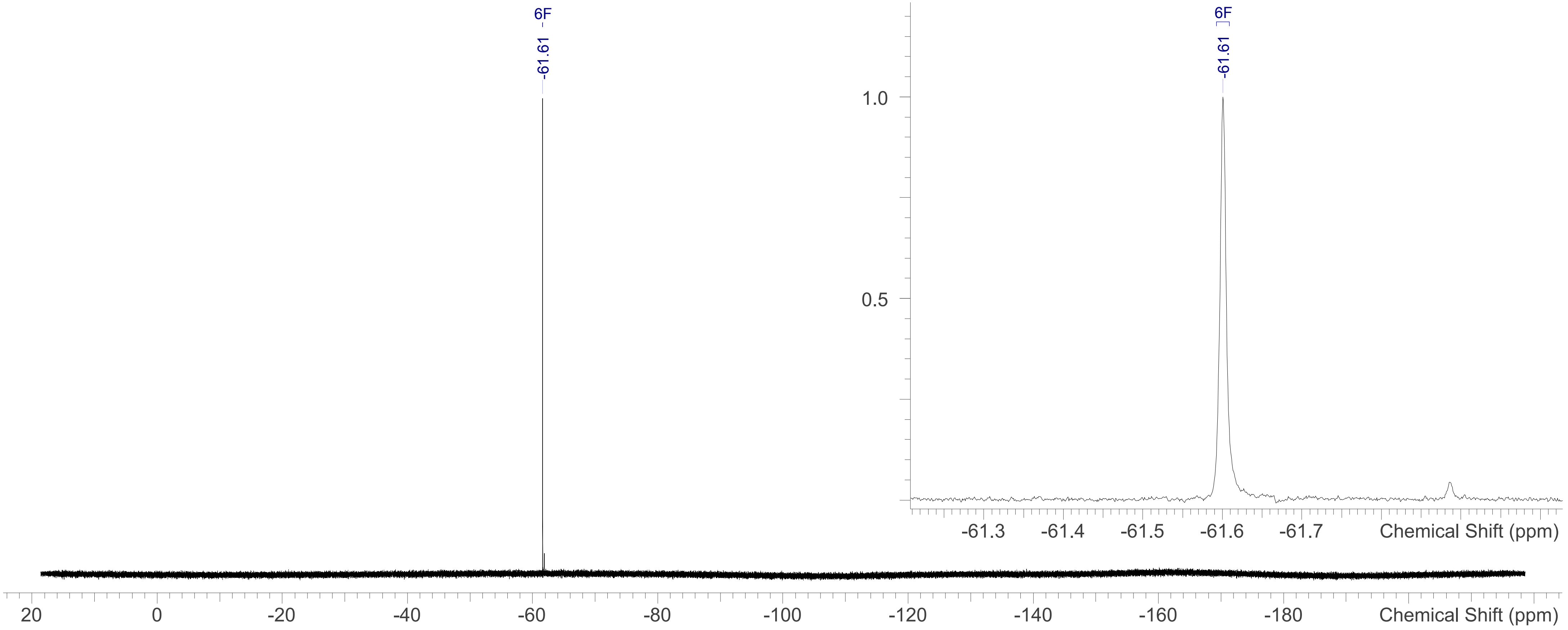

NVR-117_19F.spectrus

Openlynx Report - James B

Page 1

Sample: 1 | Vial:2:21 | ID:JB148 HRMS-1
File:JB148 HRMS-1 | Date:02-Oct-2019 | Time:12:31:13
Description:

Printed: Wed Oct 02 12:55:48 2019

3: UV Detector: TAC: Wavelength Range: (210 - 400)

1.074e+2
Range: 1.176e+2

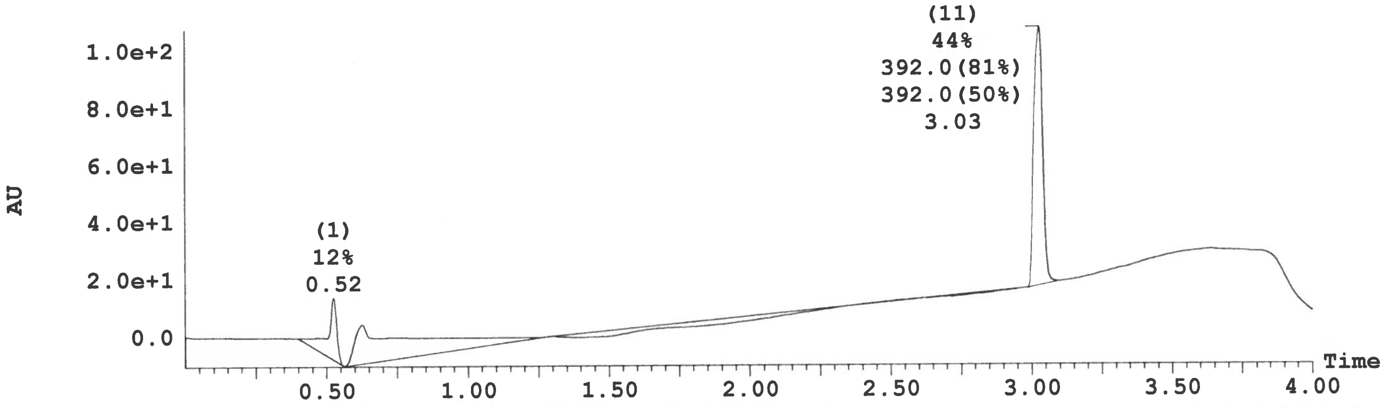

Peak Number	Compound	Time	Area %Total	Mass Found
1		0.52	11.72	Not Found
2		0.63	44.36	Not Found
11	Found	3.03	43.92	392.0, 392.0

1: MS ES+ :TIC Smooth (Mn, 2x2)

3.8e+006

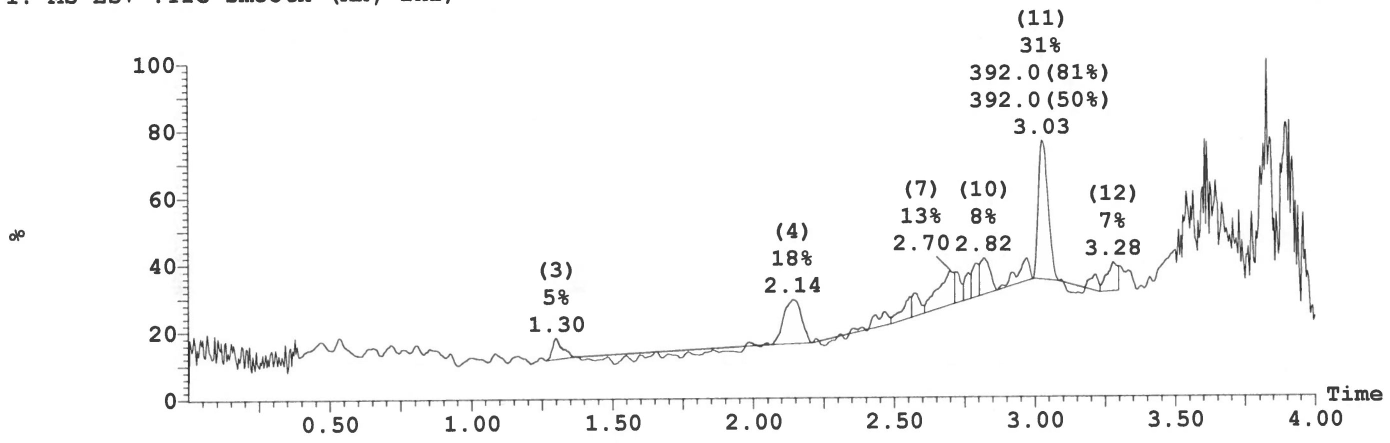

2: MS ES- :TIC Smooth (Mn, 2x2)

3.3e+006

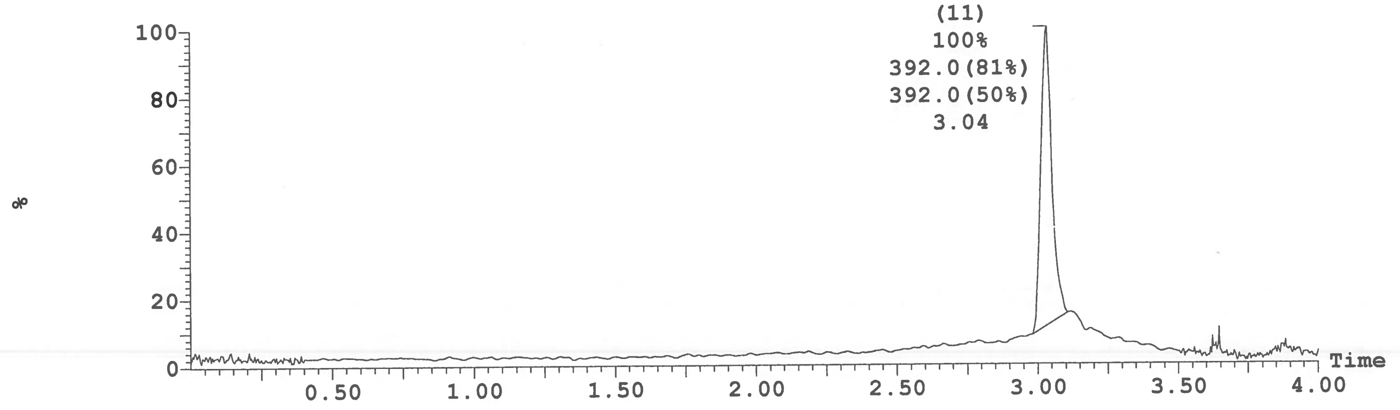

NVR-117 LCMS

Openlynx Report - James B

Sample: 1 | Vial:2:21 | ID:JB148 HRMS-1
File:JB148 HRMS-1 | Date:02-Oct-2019 | Time:12:31:13
Description:

Printed: Wed Oct 02 12:55:48 2019

Peak ID	Compound	Time	Mass Found
1		0.52	Not Found

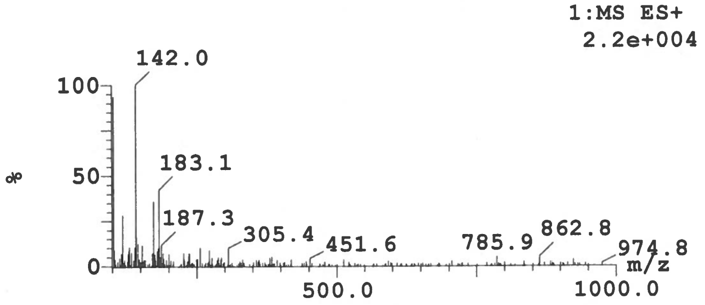

Peak ID	Compound	Time	Mass Found
1		0.52	Not Found

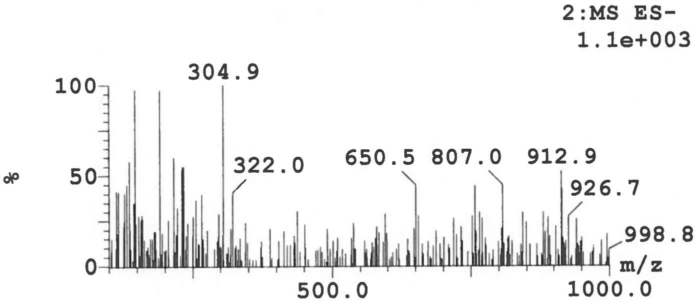

Peak ID	Compound	Time	Mass Found
1		0.52	Not Found

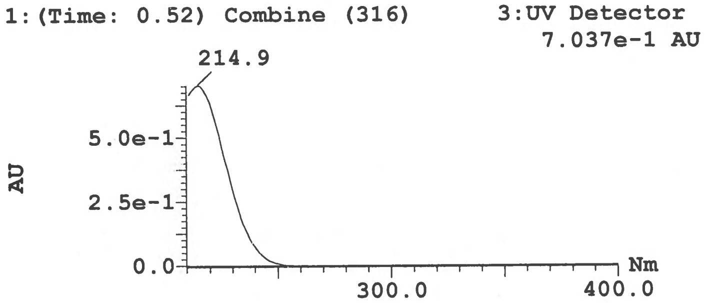

Peak ID	Compound	Time	Mass Found
2		0.63	Not Found

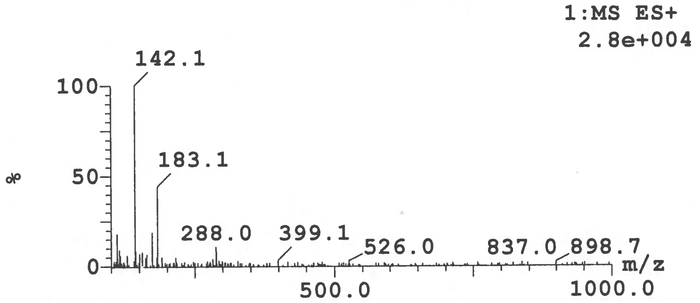

Peak ID	Compound	Time	Mass Found
2		0.63	Not Found

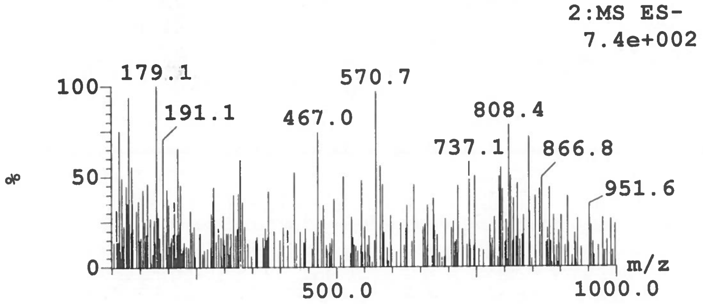

Peak ID	Compound	Time	Mass Found
2		0.63	Not Found

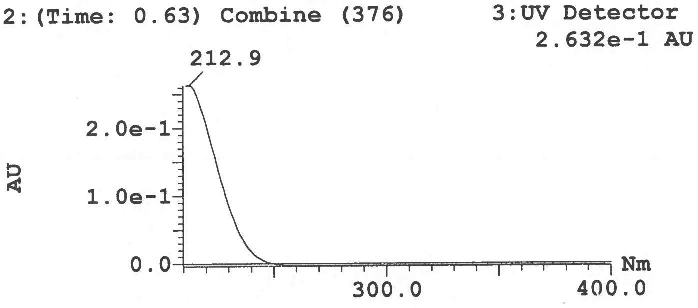

Peak ID	Compound	Time	Mass Found
11	Found	3.03	393

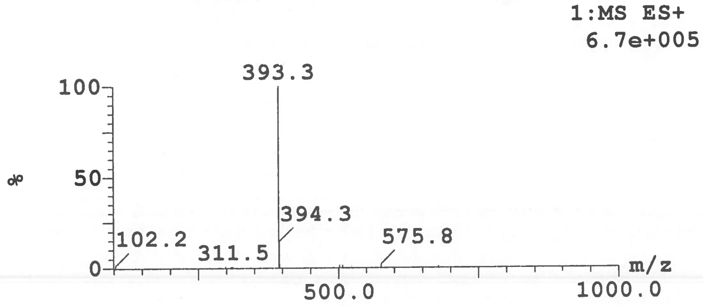

Peak ID	Compound	Time	Mass Found
11	Found	3.03	391

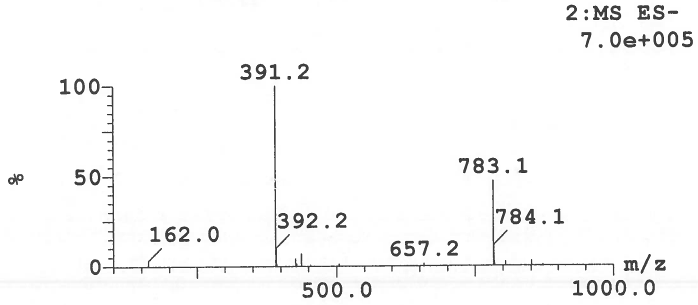

NVR-117 LCMS

Sample: 1 Vial:2:21 ID:JB148 HRMS-1
File:JB148 HRMS-1 Date:02-Oct-2019 Time:12:31:13
Description:

Printed: Wed Oct 02 12:55:48 2019

Peak ID	Compound	Time	Mass Found
11		3.03	Not Found

11: (Time: 3.03) Combine (1818) 3:UV Detector
2.035 AU

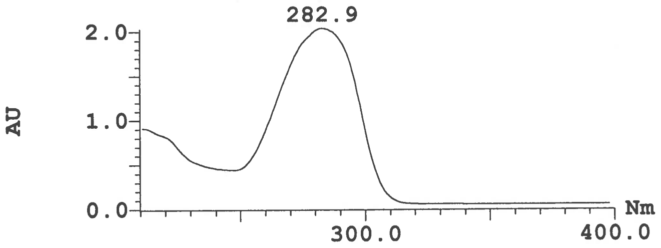

Thermo Exactive Plus EMR Orbitrab HESI neg

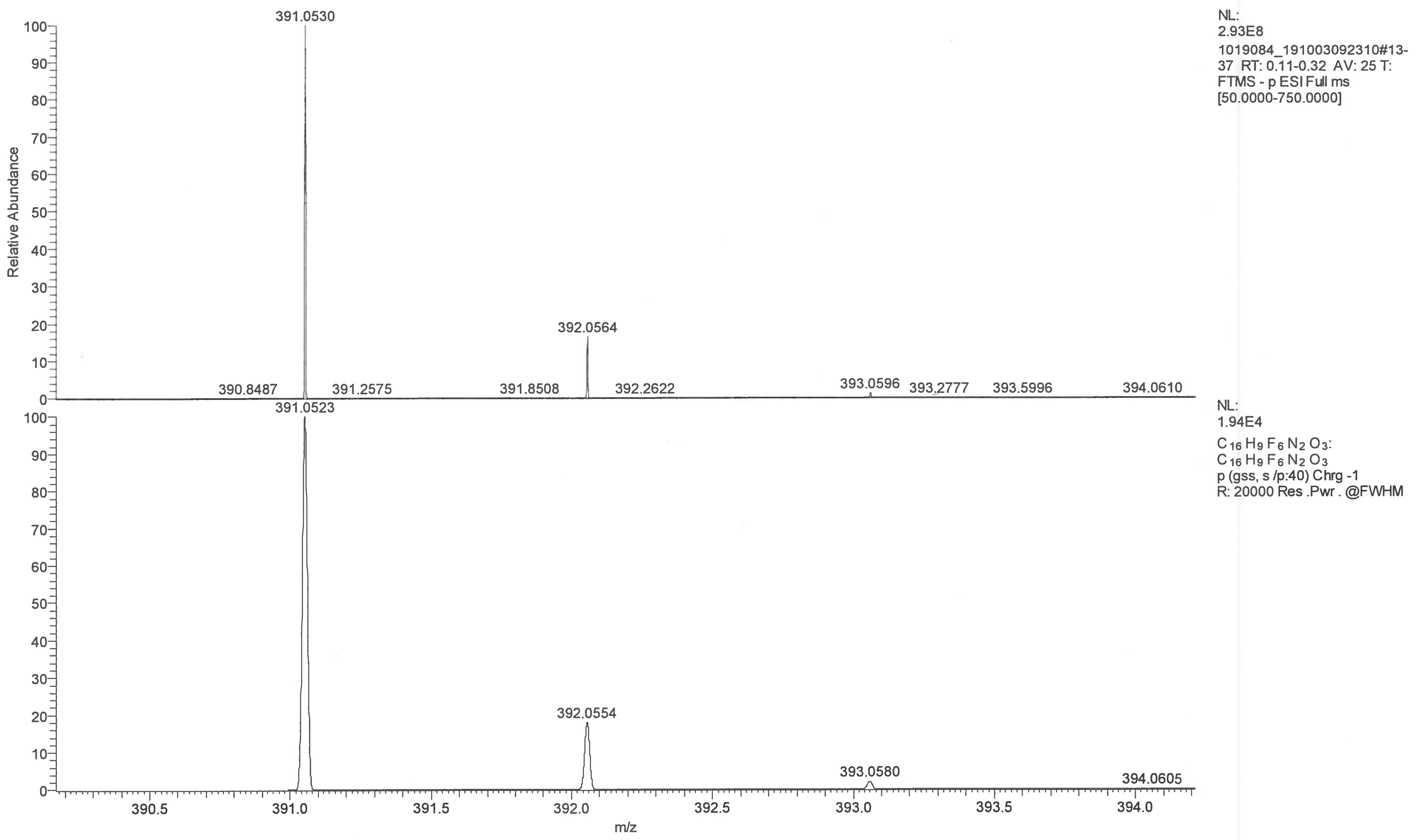

NVR-117 HRMS

Thermo Exactive Plus EMR Orbitrab HESI neg

m/z	Theo. Mass	Delta (ppm)	RDB equiv.	Composition
391.0530	391.0523	1.83	10.5	C16 H9 O3 N2 F6

NVR-117 HRMS

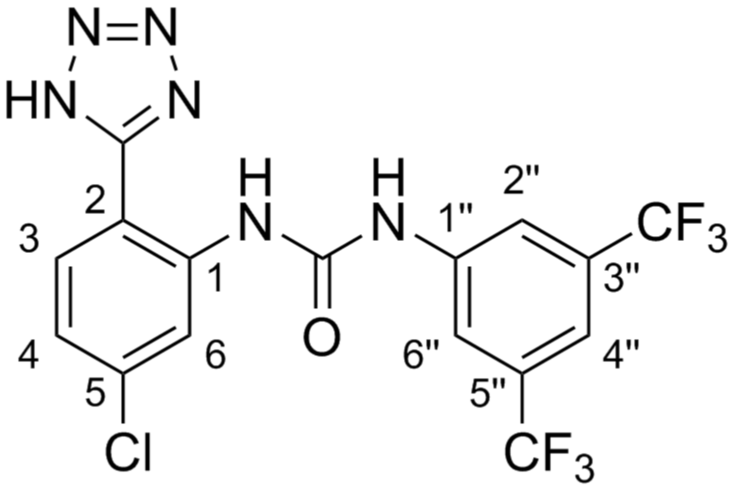

Shift (ppm)	H	m	J (Hz)	Assign
10.62	1	s	-	NH"
10.28	1	s	-	NH
8.48	1	d	2.3	6
8.21	2	s	-	2", 6"
7.95	1	d	8.5	3
7.69	1	s	-	4"
7.36	1	dd	8.4, 2.1	4

Acquisition Time (sec)	3.9846
Date	28 Apr 2018 04:49:14
Date Stamp	28 Apr 2018 04:49:14
Frequency (MHz)	400.0700
Nucleus	1H
Number of Transients	16
Solvent	DMSO-d6
Temperature (degree C)	24.998

^{1}H NMR (400 MHz, *DMSO-d_6*) δ ppm 10.62 (s, 1 H), 10.28 (s, 1 H), 8.48 (d, *J*=2.3 Hz, 1 H), 8.21 (s, 2 H), 7.95 (d, *J*=8.5 Hz, 1 H), 7.69 (s, 1 H), 7.36 (dd, *J*=8.4, 2.1 Hz, 1 H)

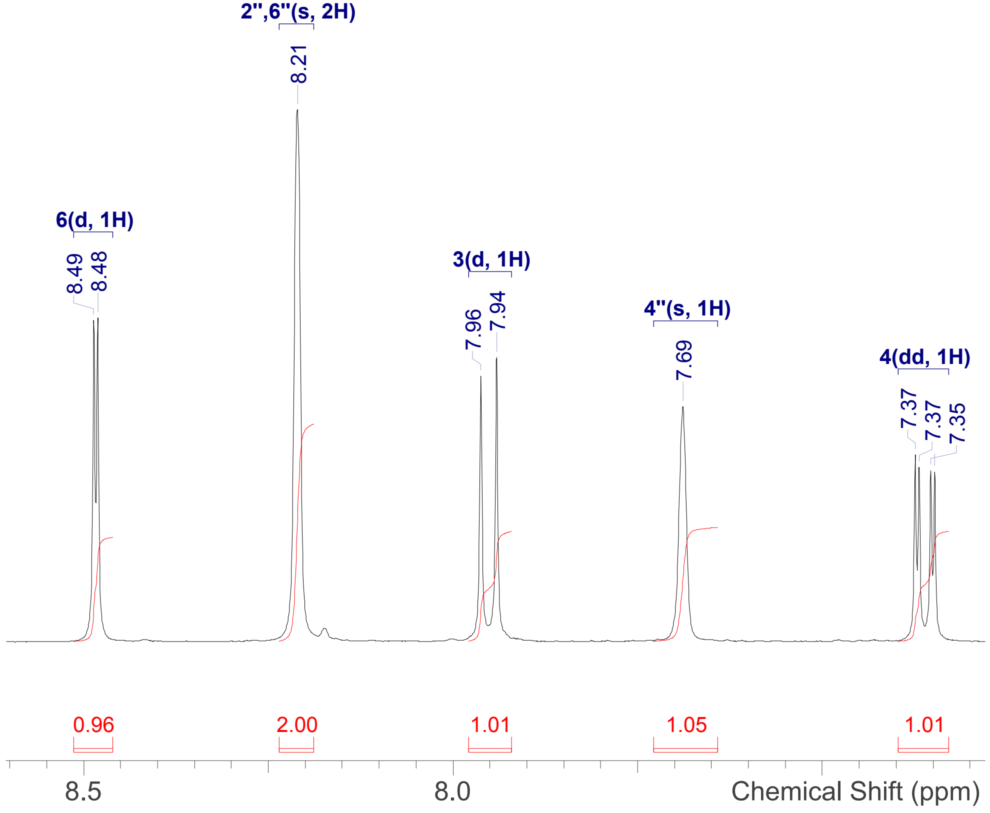

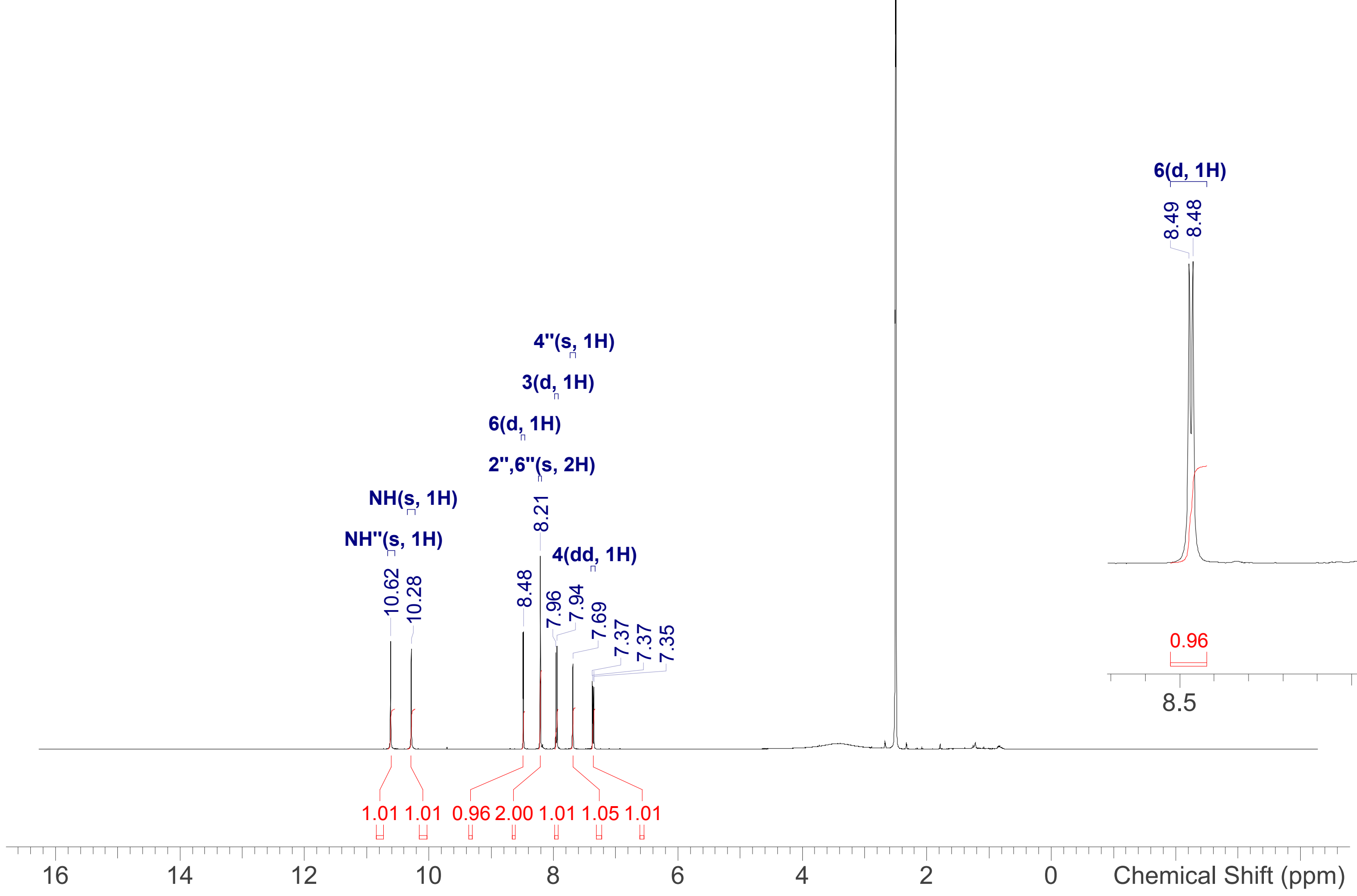

NVR-58_1H.spectrus

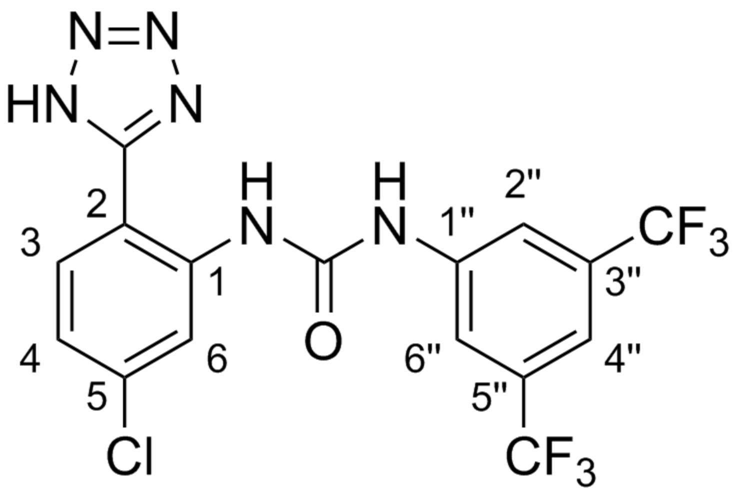

Shift (ppm)	C	m	J (Hz)	Assign
152.1	1	s	-	7"
141.6	1	s	-	1"
138.8	1	s	-	1
136.1	1	s	-	5
130.7	2	q	32.3	5", 3"
130.0	1	s	-	3
123.3	2	q	273.6	3"-CF3, 5"-CF3
122.6	1	s	-	4
120.4	1	s	-	6
118.4	2	br q	3.9	6", 2"
115.0	1	br spt	3.9	4"
110.7	1	s	-	2

Acquisition Time (sec)	1.0224
Date	28 Apr 2018 05:03:37
Date Stamp	28 Apr 2018 05:03:37
Frequency (MHz)	100.5977
Nucleus	13C
Number of Transients	256
Solvent	DMSO-d6
Temperature (degree C)	25.002

^{13}C NMR (101 MHz, *DMSO-d_6*) δ ppm 152.1 (s, 1 C), 141.6 (s, 1 C), 138.8 (s, 1 C), 136.1 (s, 1 C), 130.7 (q, *J*=32.3 Hz, 2 C), 130.0 (s, 1 C), 122.6 (s, 1 C), 120.4 (s, 1 C), 123.3 (q, *J*=273.6 Hz, 2 C), 118.4 (br q, *J*=3.9 Hz, 2 C), 115.0 (br spt, *J*=3.9 Hz, 1 C), 110.7 (s, 1 C)

NVR-58_13C

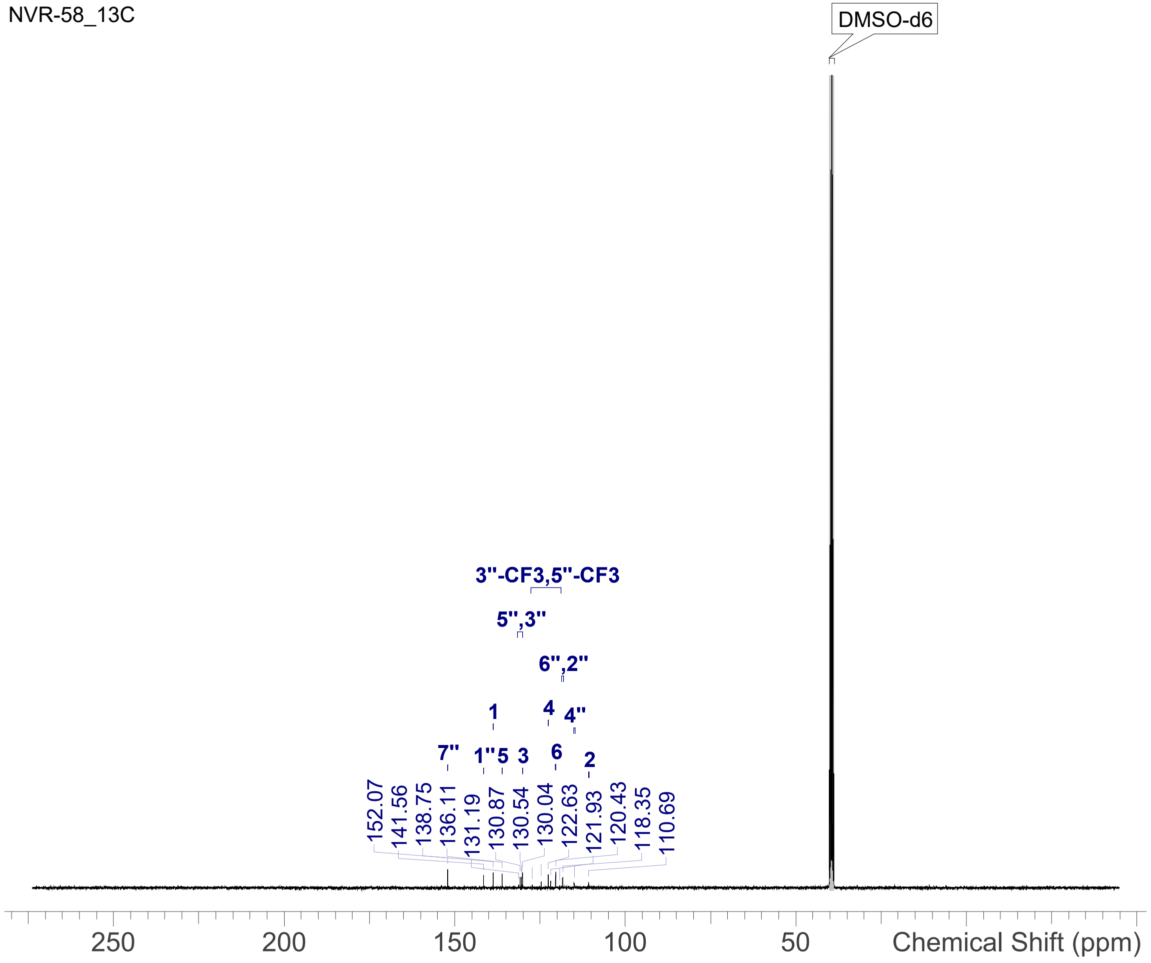

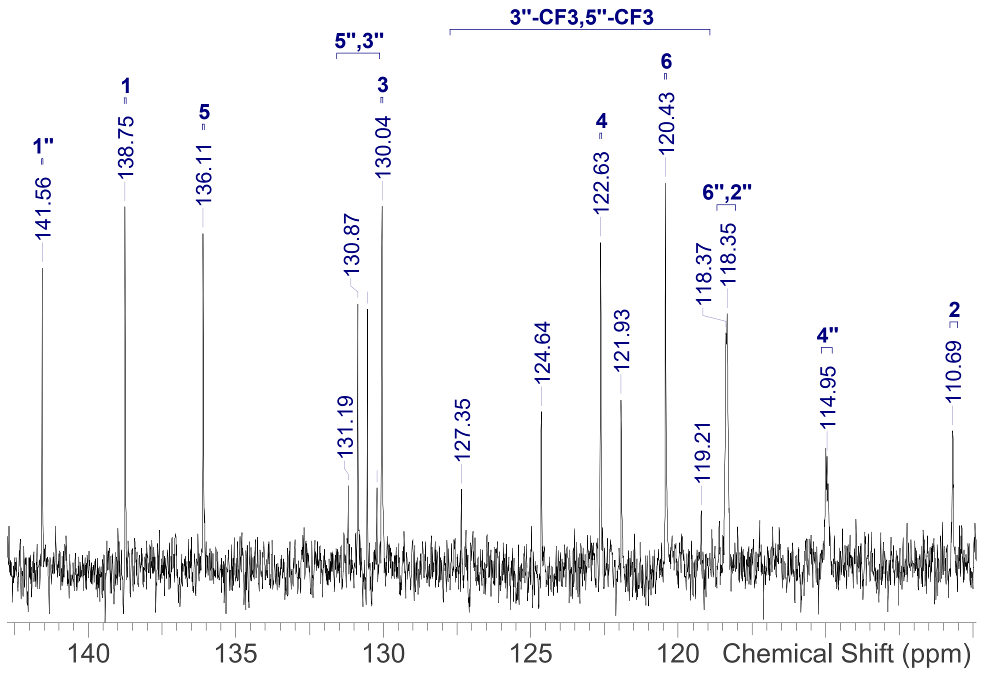

NVR-58_13C.spectrus

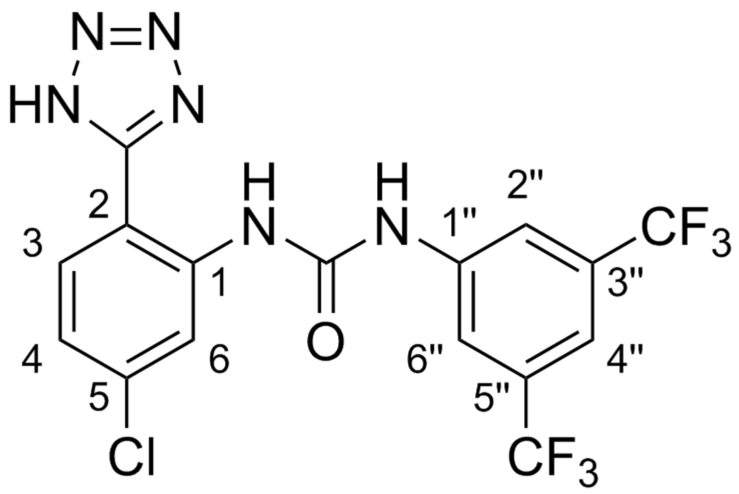

Shift (ppm)	F	m
-61.69	6	s

Acquisition Time (sec)	1.4680
Date	28 Apr 2018 05:48:16
Date Stamp	28 Apr 2018 05:48:16
Frequency (MHz)	376.4419
Nucleus	19F
Number of Transients	16
Solvent	DMSO-d6
Temperature (degree C)	25.001

^{19}F NMR (376 MHz, *DMSO-d_6*) δ ppm -61.69 (s, 6 F)

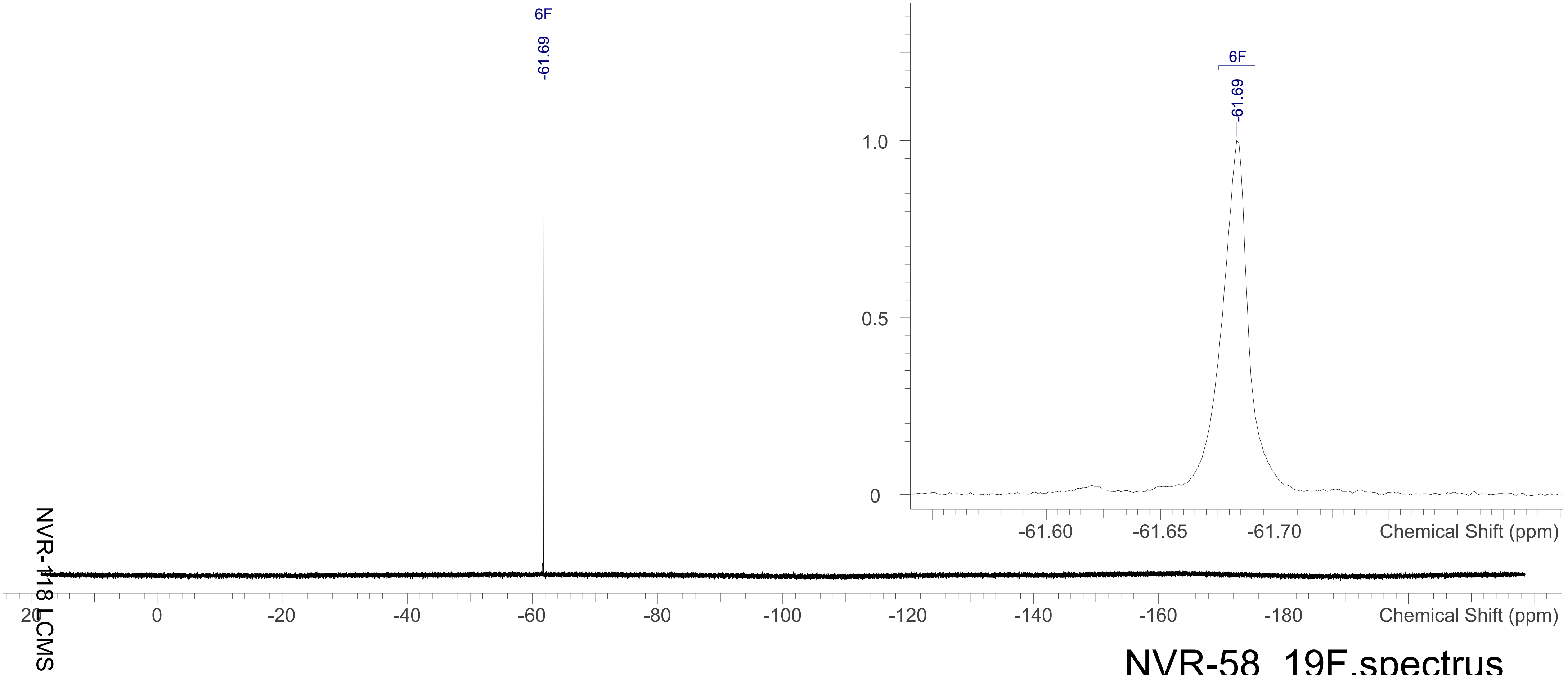

NVR-58_19F.spectrus

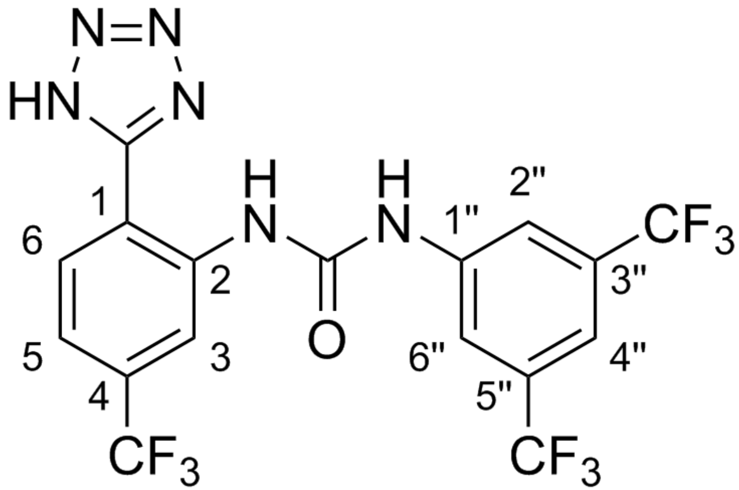

Shift (ppm)	H	m	J (Hz)	Assign
10.67	1	s	-	NH''
10.36	1	s	-	NH
8.76	1	d	1.0	3
8.19	2	s	-	2'', 6''
8.18	1	d	8.1	6
7.68	1	s	-	4''
7.62	1	dd	8.3, 1.2	5

Acquisition Time (sec)	4.0894
Date	20 Jun 2019 17:07:36
Date Stamp	20 Jun 2019 17:07:36
Frequency (MHz)	400.1300
Nucleus	1H
Number of Transients	16
Solvent	DMSO-d6
Temperature (degree C)	21.100

^{1}H NMR (400 MHz, *DMSO-d_6*) δ ppm 10.67 (s, 1 H), 10.36 (s, 1 H), 8.76 (d, *J*=1.0 Hz, 1 H), 8.19 (s, 2 H), 8.18 (d, *J*=8.1 Hz, 1 H), 7.68 (s, 1 H), 7.62 (dd, *J*=8.3, 1.2 Hz, 1 H)

NVR-133_1H

NVR-133_1H

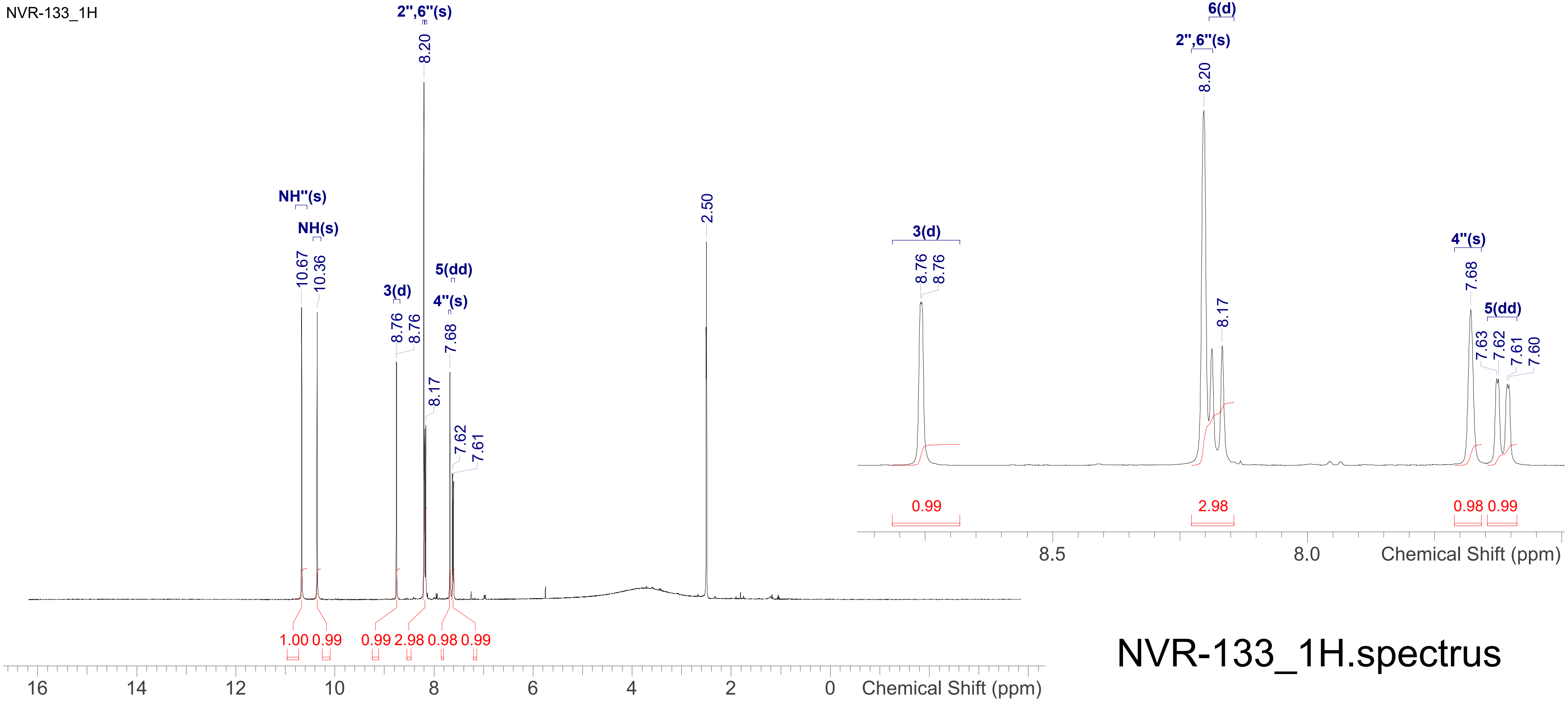

NVR-133_1H.spectrus

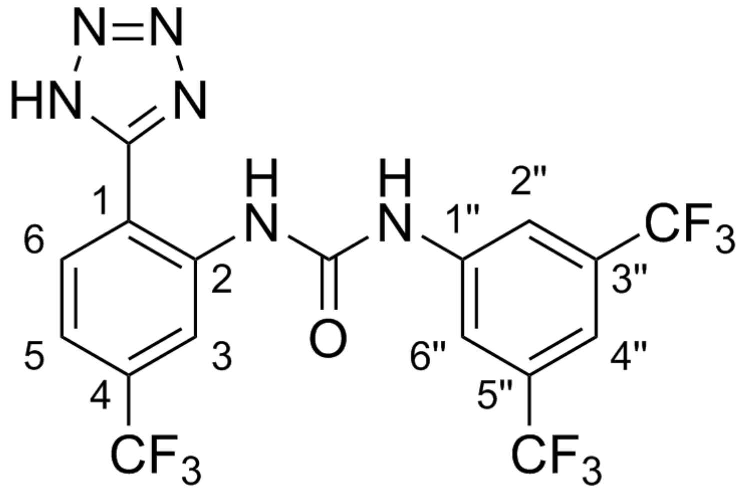

Shift (ppm)	C	m	J (Hz)	Assign
152.2	1	s	-	CO
141.6	1	s	-	1''
138.1	1	s	-	2
131.2	1	q	32.0	4
130.7	2	q	33.3	3'', 5''
129.8	1	s	-	6
123.7	1	q	271.3	4-CF3
123.3	2	q	272.6	5''-CF3, 3''-CF3
119.0	1	br q	3.9	5
118.4	2	br q	3.9	2'', 6''
117.4	1	br q	3.9	3
115.6	1	s	-	1
115.0	1	spt	3.9	4''

Acquisition Time (sec)	1.0224
Date	20 Jun 2019 22:31:08
Date Stamp	20 Jun 2019 22:31:08
Frequency (MHz)	100.6128
Nucleus	13C
Number of Transients	256
Solvent	DMSO-d6
Temperature (degree C)	22.700

^{13}C NMR (101 MHz, *DMSO-d_6*) δ ppm 152.2 (s, 1 C), 141.6 (s, 1 C), 138.1 (s, 1 C), 131.2 (q, *J*=32.0 Hz, 1 C), 130.7 (q, *J*=33.3 Hz, 2 C), 129.8 (s, 1 C), 123.7 (q, *J*=271.3 Hz, 1 C), 123.3 (q, *J*=272.6 Hz, 2 C), 119.0 (br q, *J*=3.9 Hz, 1 C), 118.4 (br q, *J*=3.9 Hz, 2 C), 117.4 (br q, *J*=3.9 Hz, 1 C), 115.6 (s, 1 C), 115.0 (spt, *J*=3.9 Hz, 1 C)

NVR-133_13C

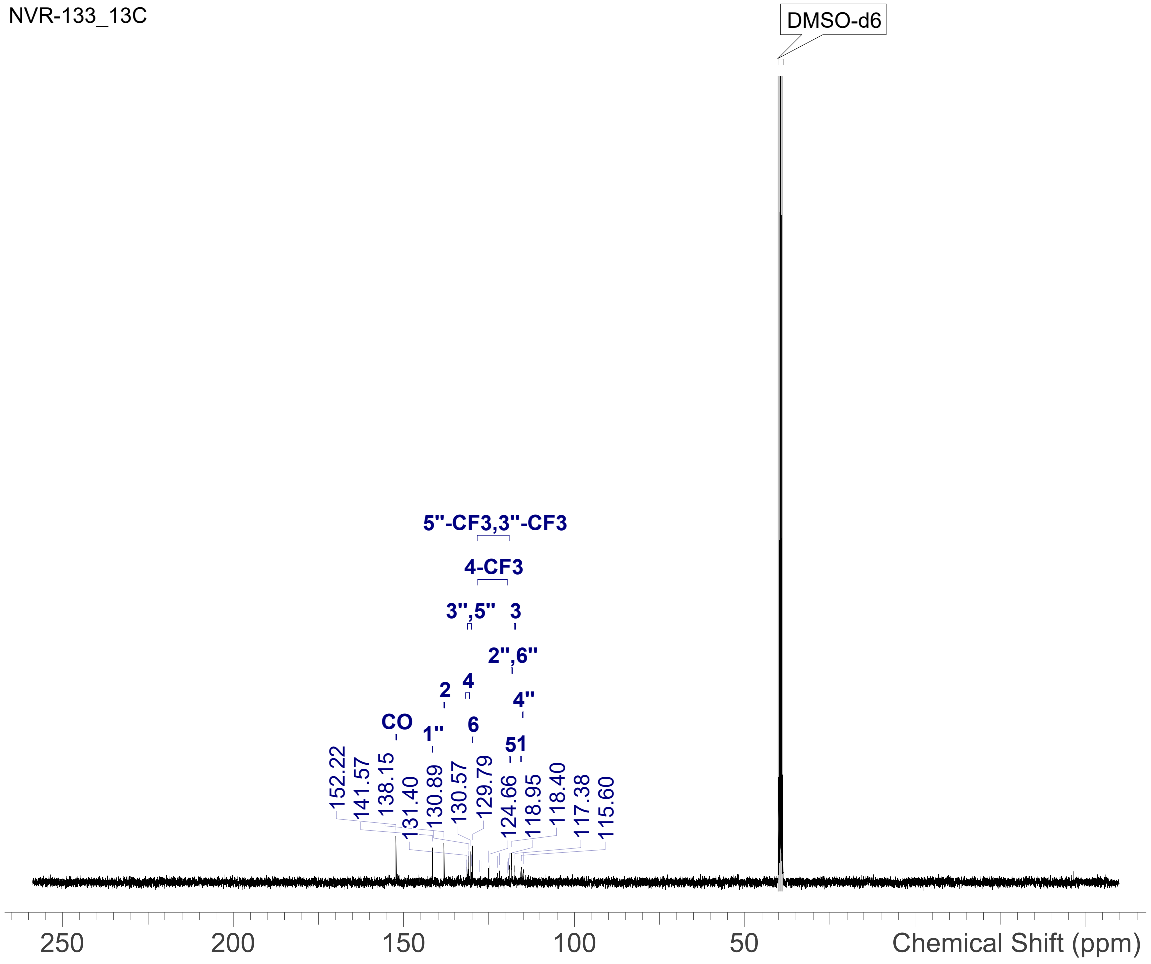

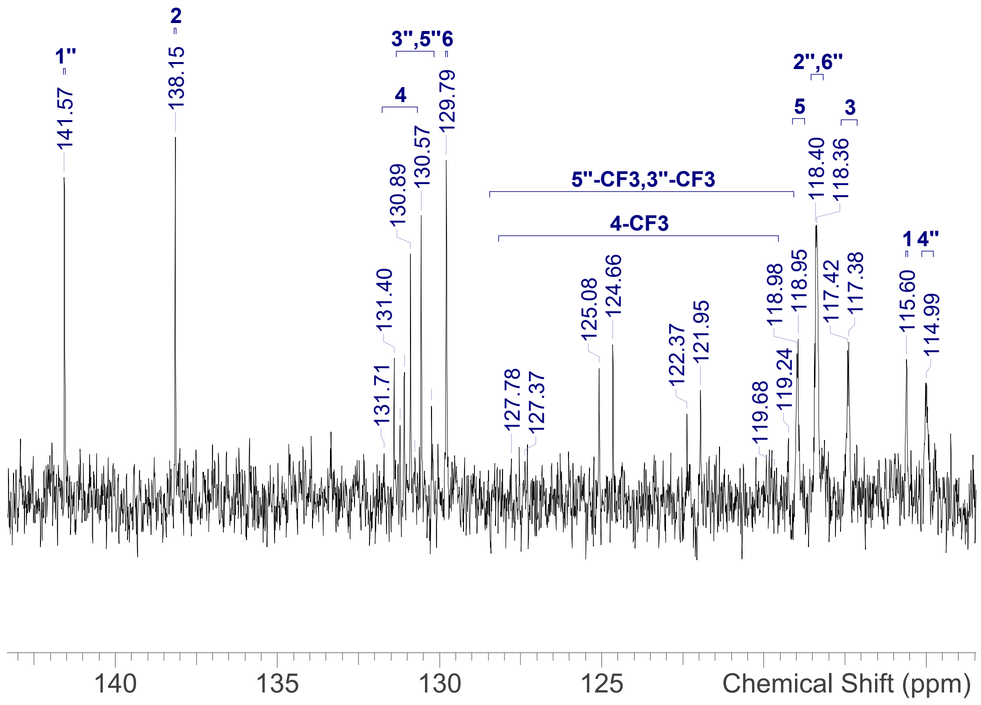

NVR-133_13C.spectrus

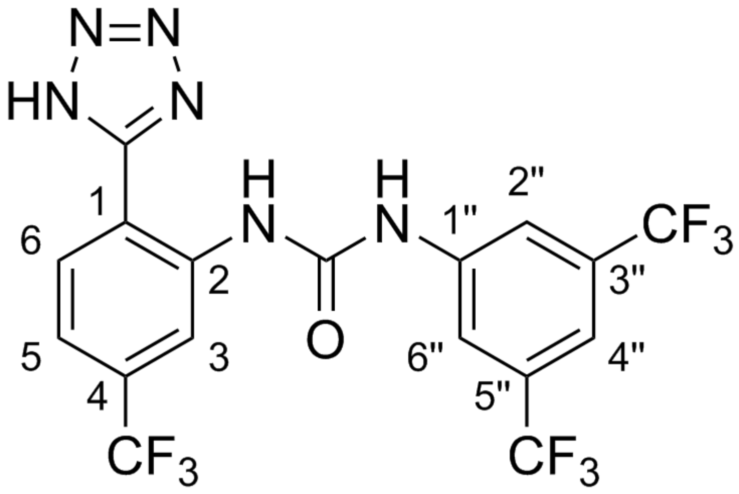

Shift (ppm)	F	m
-61.73	6	s
-61.85	3	s

Acquisition Time (sec)	2.9360
Date	20/06/2019 22:47:00
Date Stamp	20/06/2019 22:47:00
Frequency (MHz)	376.4610
Nucleus	19F
Number of Transients	128
Solvent	DMSO-d6

^{19}F NMR (376 MHz, *DMSO-d*$_6$) δ ppm -61.73 (s, 6 F), -61.85 (s, 3 F)

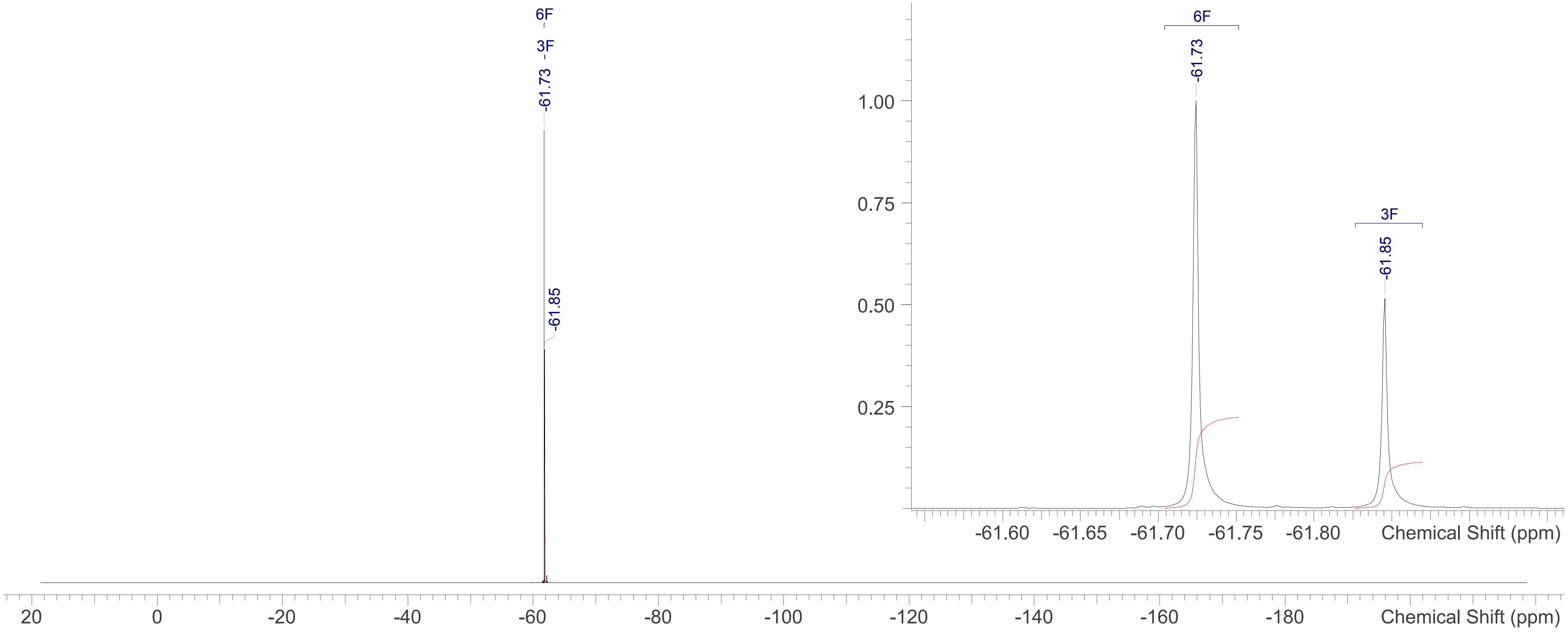

NVR-133_19F.spectrus

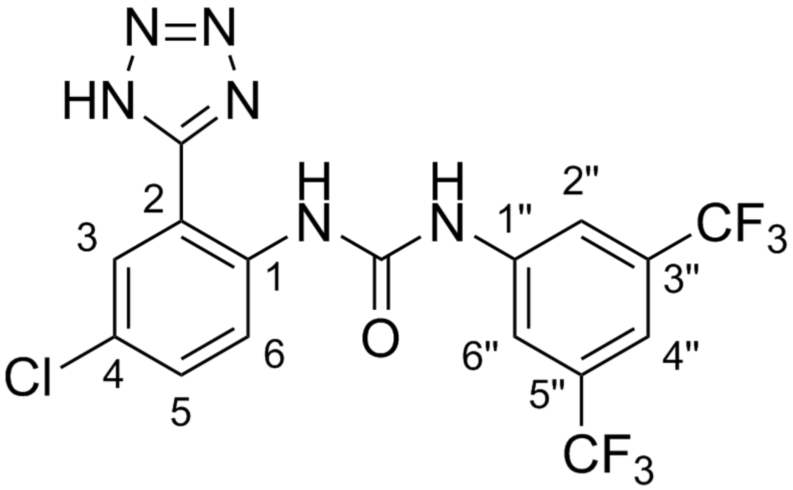

Shift (ppm)	H	m	J (Hz)	Assign
10.54	1	s	-	NH"
10.11	1	s	-	NH
8.37	1	d	9.0	H6
8.19	2	s	-	H2", H6"
8.00	1	br s	-	H3
7.64	1	s	-	H4"
7.58	1	br d	9.2	H5

Acquisition Time (sec)	6.5536
Date	21/03/2018 17:18:00
Date Stamp	21/03/2018 17:18:00
Frequency (MHz)	500.1930
Nucleus	1H
Number of Transients	16
Solvent	DMSO-d6

^{1}H NMR (500 MHz, *DMSO-d_6*) δ ppm 10.54 (s, 1 H), 10.11 (s, 1 H), 8.37 (d, *J*=9.0 Hz, 1 H), 8.19 (s, 2 H), 8.00 (br s, 1 H), 7.64 (s, 1 H), 7.58 (br d, *J*=9.2 Hz, 1 H)

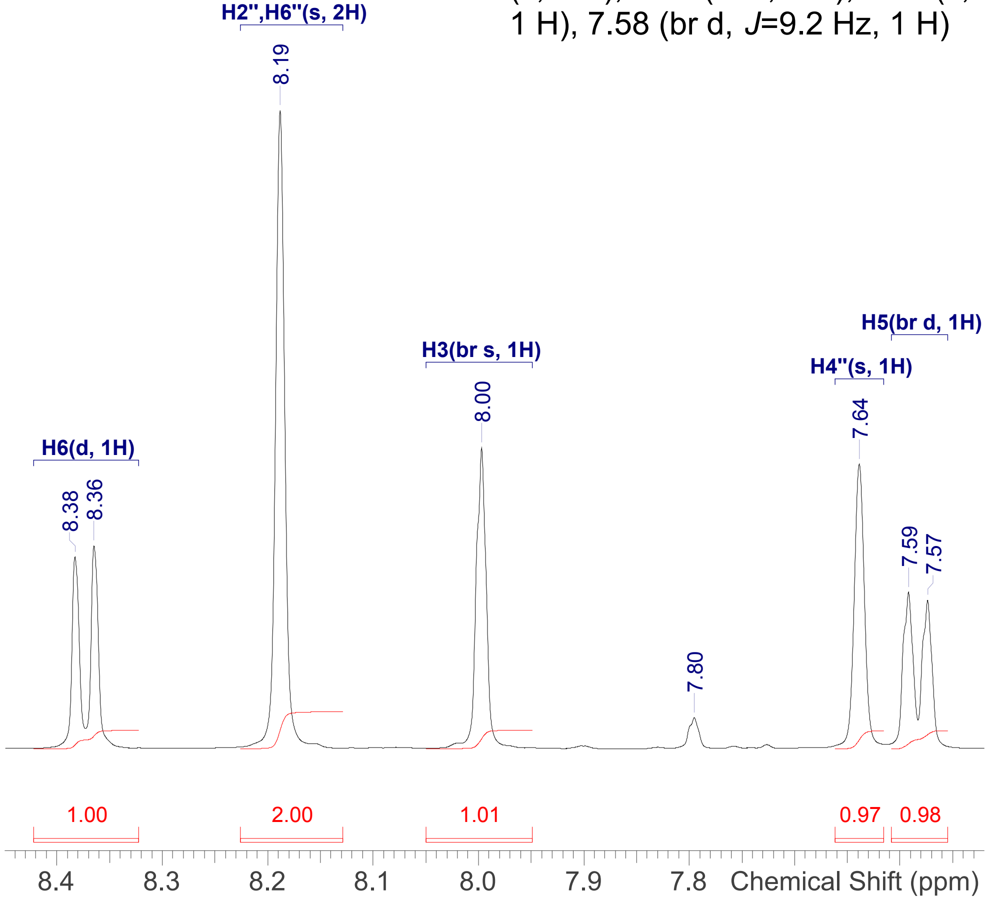

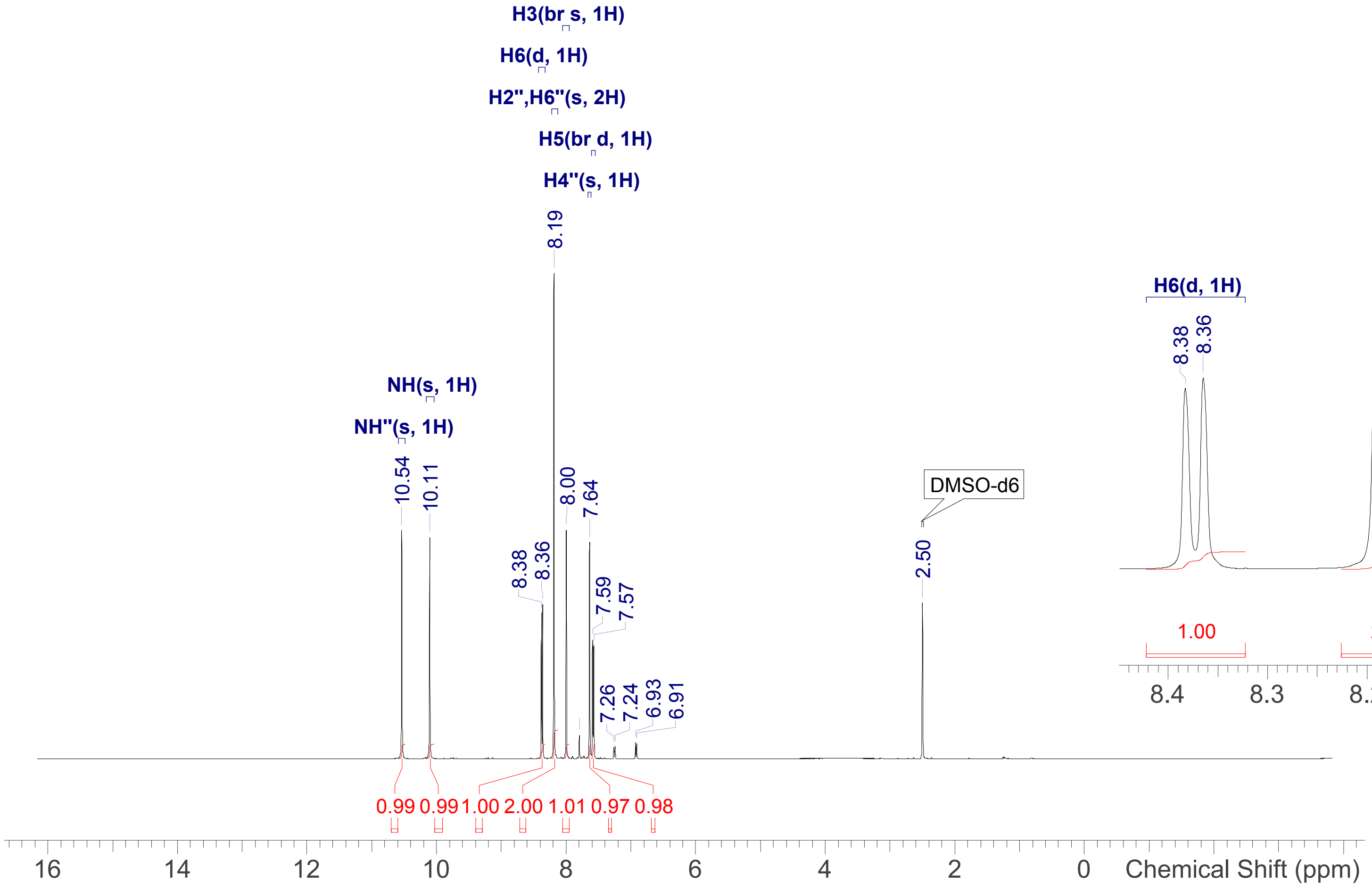

NVR-41_1H.spectrus

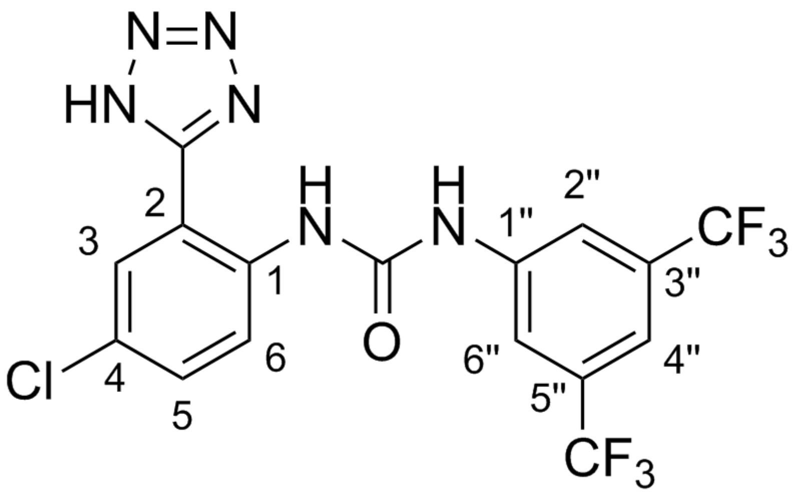

Shift (ppm)	C	m	J (Hz)	Assign
153.6	1	s	-	Tet-C
152.1	1	s	-	CO
141.7	1	s	-	1"
136.5	1	s	-	1
131.2	1	s	-	5
130.7	2	q	32.8	3", 5"
127.8	1	s	-	3
126.4	1	s	-	4
123.3	2	q	272.9	5"-CF3, 3"-CF3
123.1	1	s	-	6
118.2	2	br q	3.9	2", 6"
114.7	1	br spt	3.8	4"
113.6	1	s	-	2

Acquisition Time (sec)	2.0447
Date	21/03/2018 17:22:00
Date Stamp	21/03/2018 17:22:00
Frequency (MHz)	125.7870
Nucleus	13C
Number of Transients	64
Solvent	DMSO-d6

^{13}C NMR (126 MHz, *DMSO-d_6*) δ ppm 153.6 (s, 1 C), 152.1 (s, 1 C), 141.7 (s, 1 C), 136.5 (s, 1 C), 131.2 (s, 1 C), 130.7 (q, *J*=32.8 Hz, 2 C), 127.8 (s, 1 C), 126.4 (s, 1 C), 123.1 (s, 1 C), 123.3 (q, *J*=272.9 Hz, 2 C), 118.2 (br q, *J*=3.9 Hz, 2 C), 114.7 (br spt, *J*=3.8 Hz, 1 C), 113.6 (s, 1 C)

NVR-41_13C

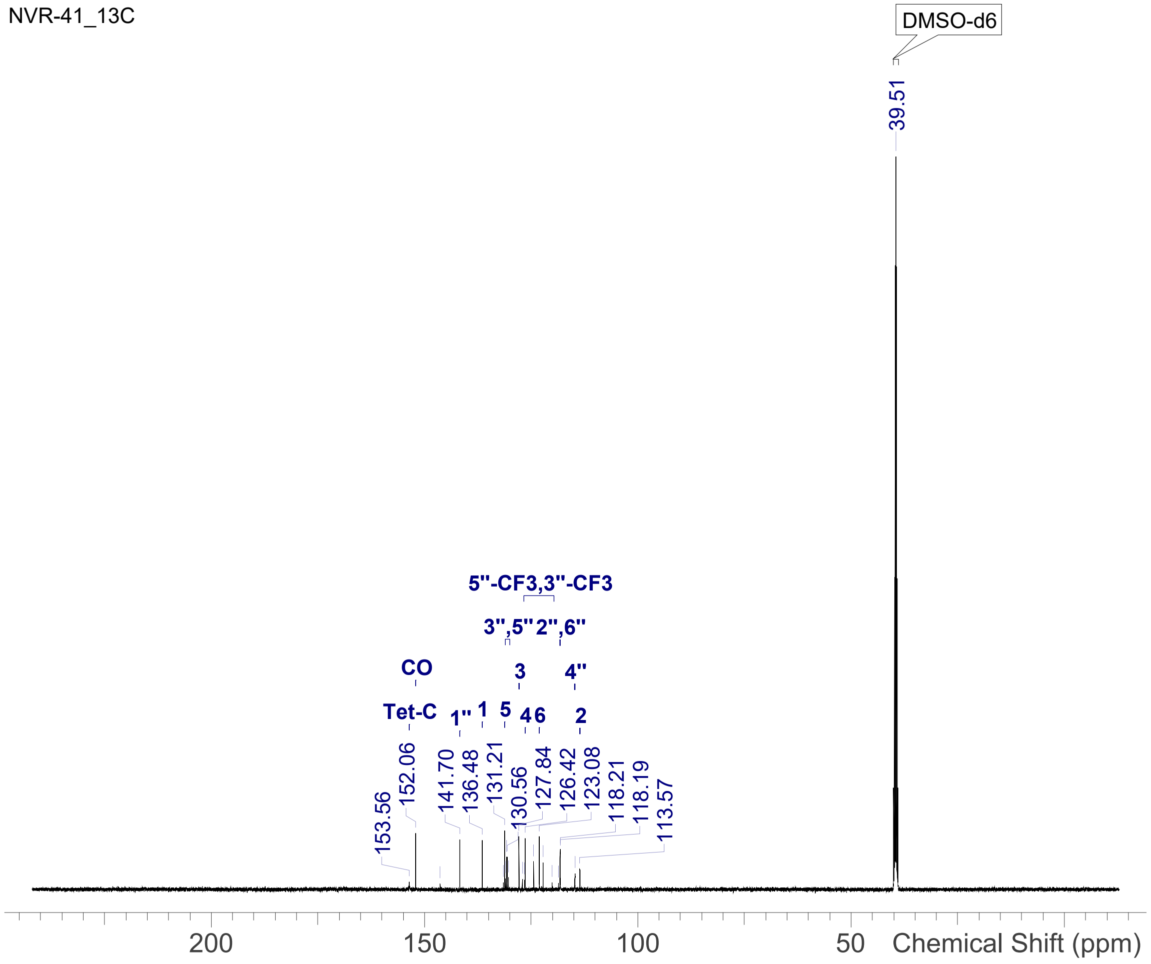

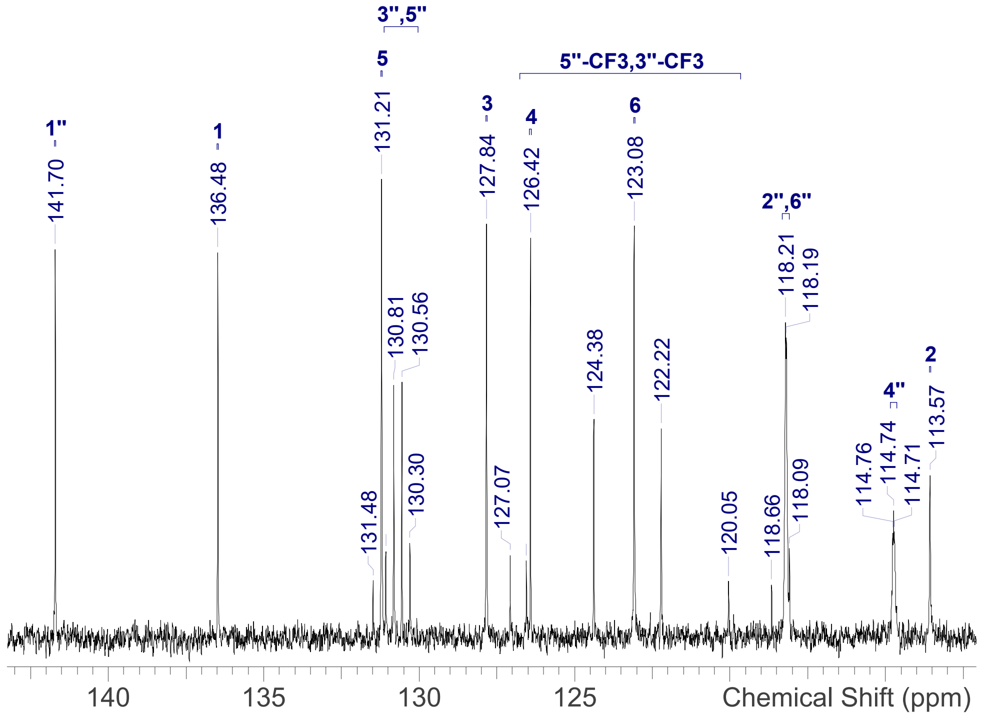

NVR-41_13C.spectrus

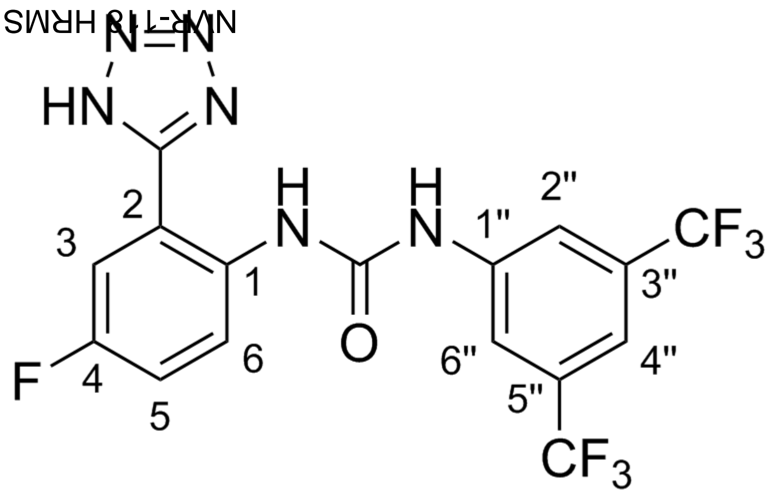

Shift (ppm)	H	m	J (Hz)	Assign
10.48	1	s	-	NH"
9.97	1	s	-	NH
8.29	1	dd	9.3, 5.4	H6
8.19	2	br s	-	H2", H6"
7.79	1	dd	9.3, 3.0	H3
7.66	1	br s	-	H4"
7.44	1	td	8.6, 3.0	H5

Acquisition Time (sec)	8.1920
Date	17/11/2017 14:59:00
Date Stamp	17/11/2017 14:59:00
Frequency (MHz)	400.1320
Nucleus	1H
Number of Transients	16
Solvent	DMSO-d6

^{1}H NMR (400 MHz, *DMSO-d_6*) δ ppm 10.48 (s, 1 H), 9.97 (s, 1 H), 8.29 (dd, *J*=9.3, 5.4 Hz, 1 H), 8.19 (br s, 2 H), 7.79 (dd, *J*=9.3, 3.0 Hz, 1 H), 7.66 (br s, 1 H), 7.44 (td, *J*=8.6, 3.0 Hz, 1 H)

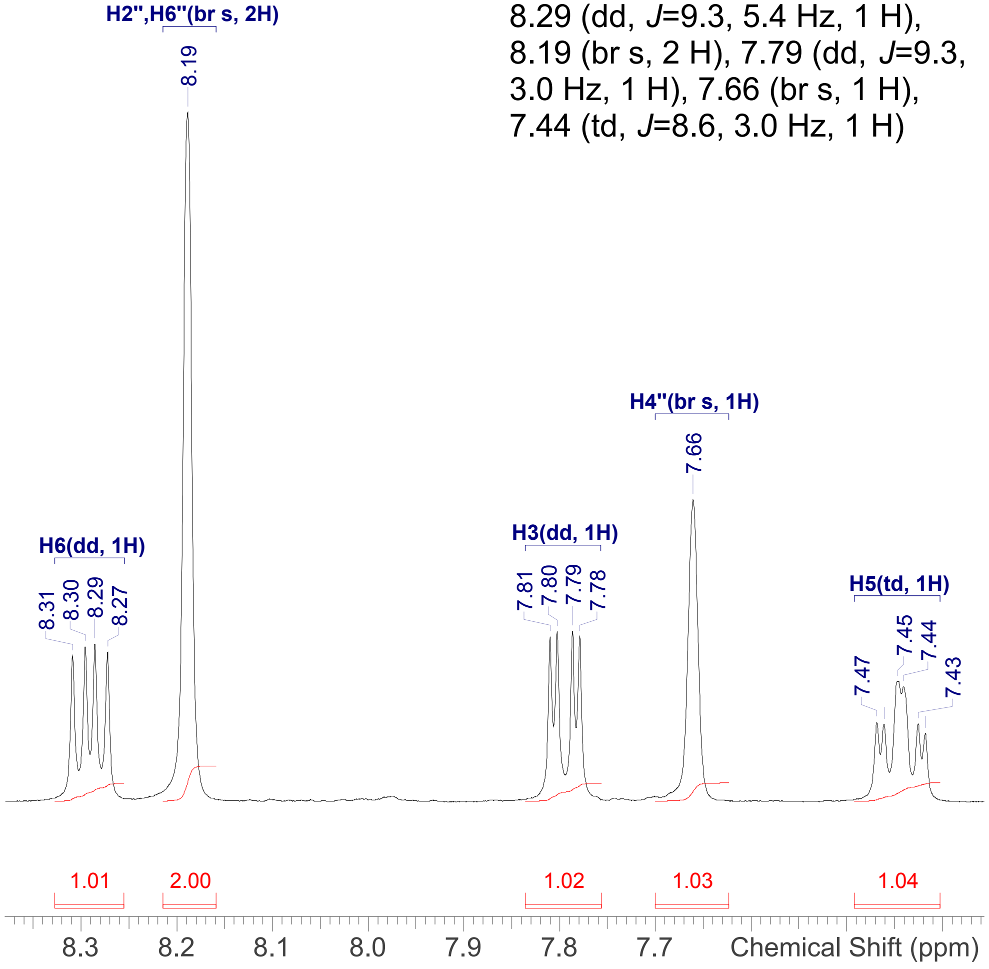

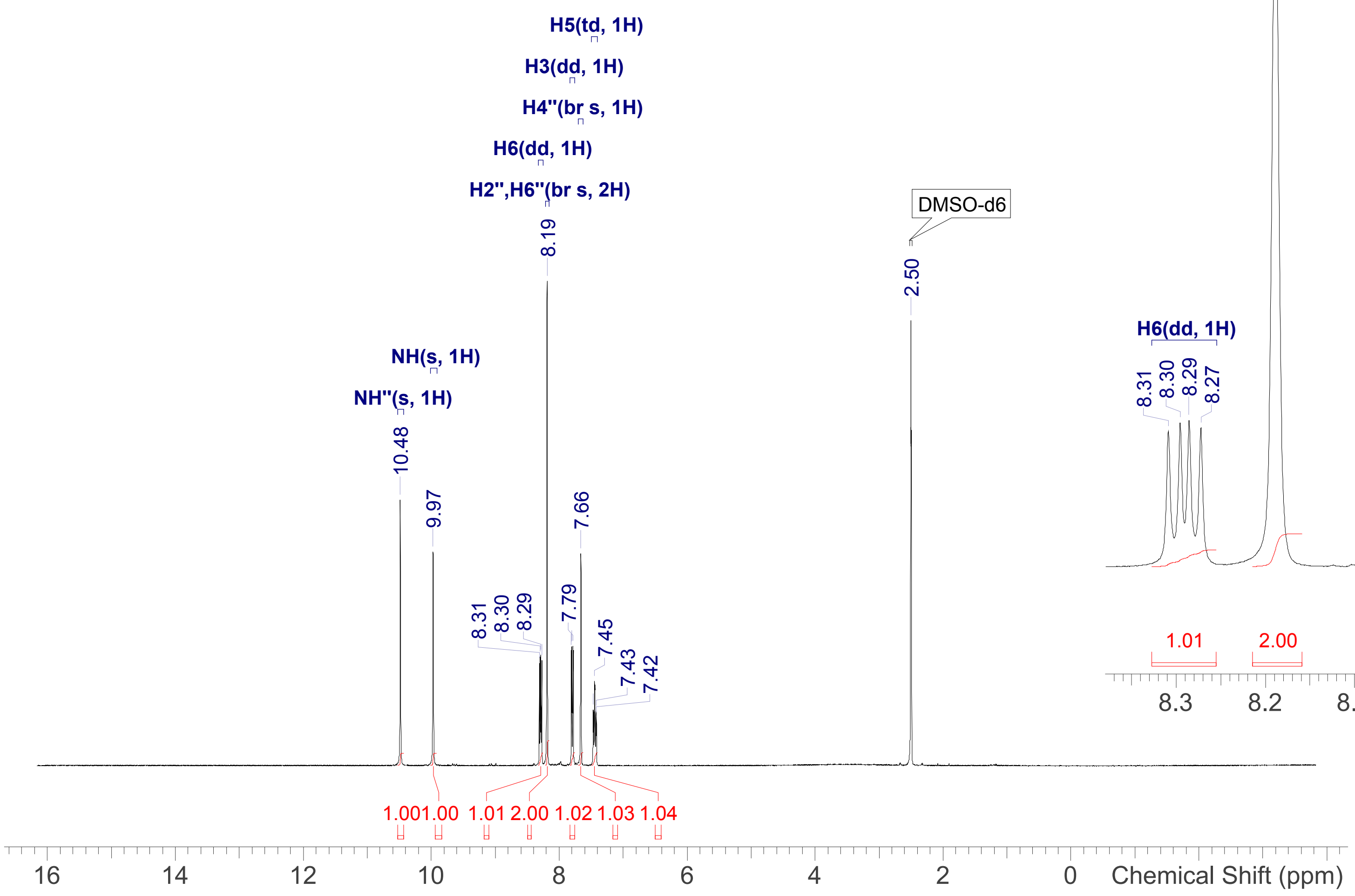

NVR-42_1H.spectrus

NVR-118L

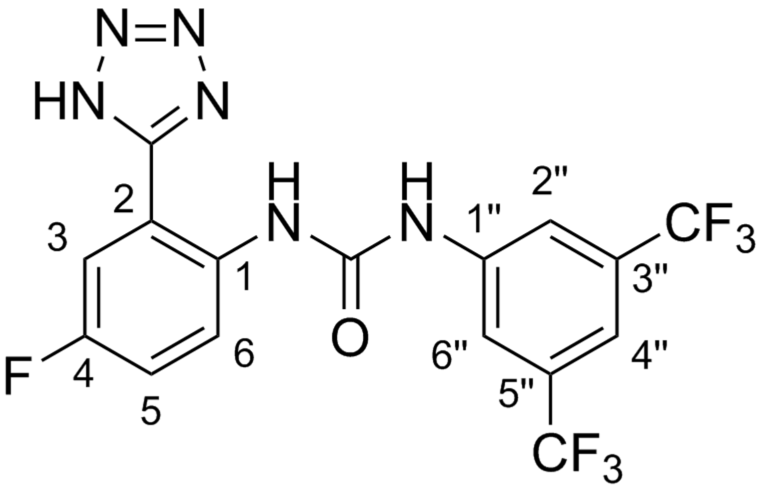

Shift (ppm)	C	m	J (Hz)	Assign
156.0	1	s	-	Tet-C
152.3	1	s	-	CO
141.8	1	s	-	1''
133.8	1	d	2.4	1
130.7	2	q	32.6	3'', 5''
124.1	1	d	8.3	6
123.3	2	q	272.3	5''-CF3, 3''-CF3
118.3	1	d	22.0	3
118.1	2	br q	3.9	2'', 6''
114.9	1	s	-	2
114.6	1	br s	-	

Acquisition Time (sec)	2.0447
Date	18/11/2017 23:56:00
Date Stamp	18/11/2017 23:56:00
Frequency (MHz)	100.6230
Nucleus	13C
Number of Transients	256
Solvent	DMSO-d6

^{13}C NMR (101 MHz, *DMSO-d_6*) δ ppm 156.0 (s, 1 C), 152.3 (s, 1 C), 141.8 (s, 1 C), 133.8 (d, *J*=2.4 Hz, 1 C), 130.7 (q, *J*=32.6 Hz, 2 C), 124.1 (d, *J*=8.3 Hz, 1 C), 123.3 (q, *J*=272.3 Hz, 2 C), 118.3 (d, *J*=22.0 Hz, 1 C), 118.1 (br q, *J*=3.9 Hz, 2 C), 114.9 (s, 1 C), 114.6 (br s, 1 C)

NVR-42_13C

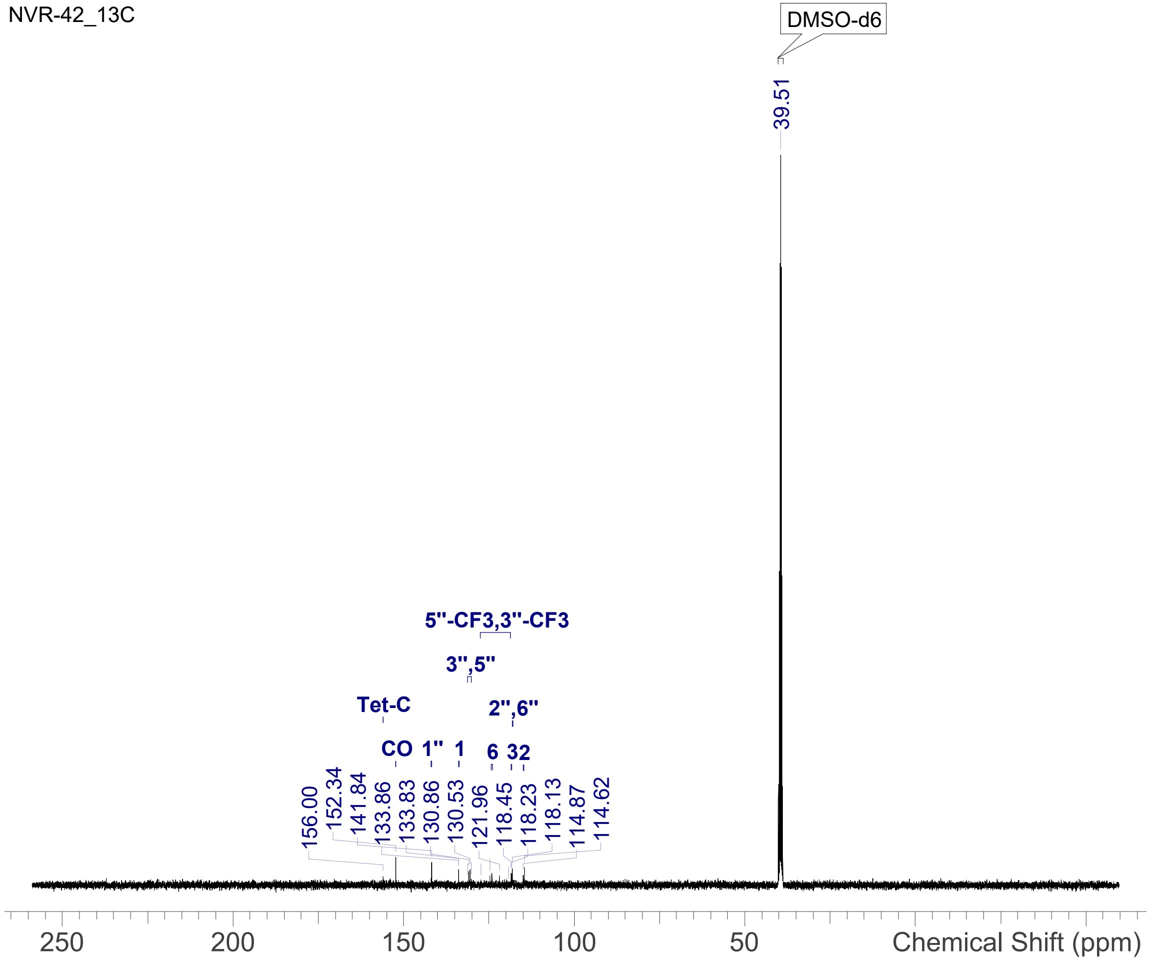

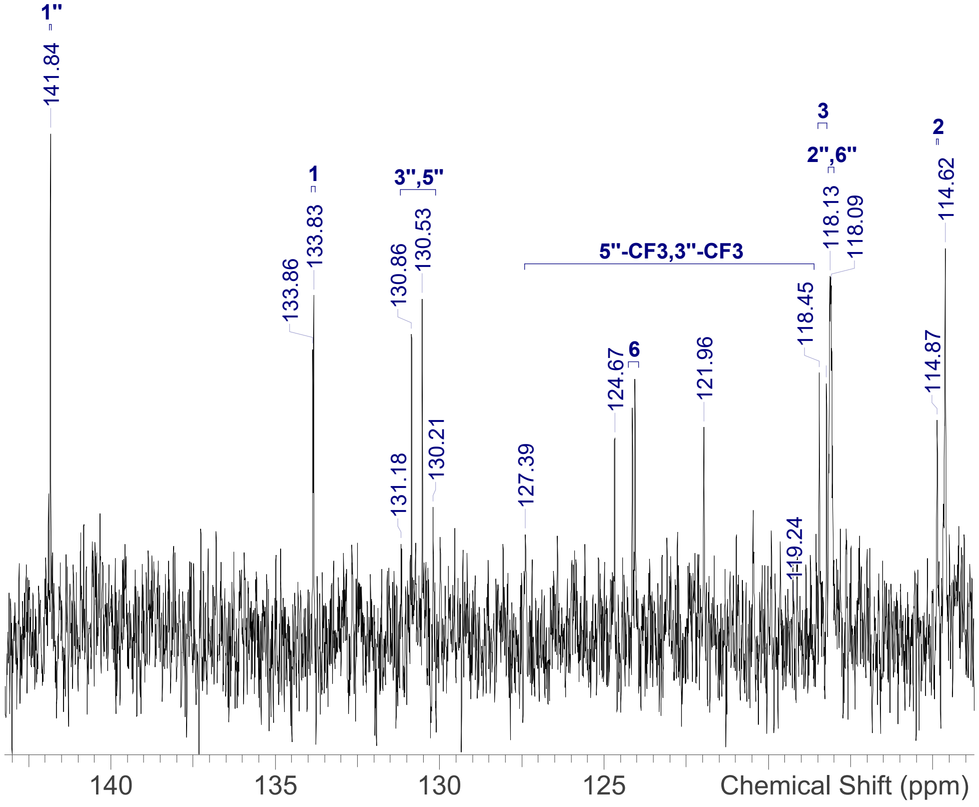

NVR-42_13C.spectrus

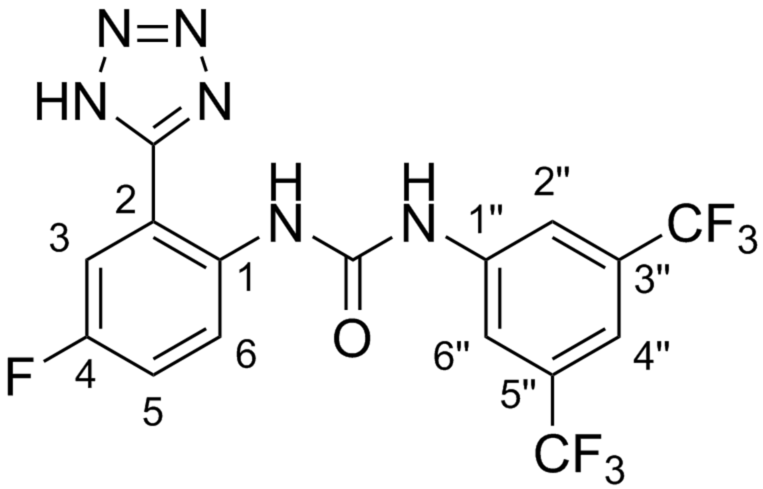

Shift (ppm)	F	m
-61.72	6	s
-119.64	1	s

Acquisition Time (sec)	2.9360
Date	19/11/2017 00:07:00
Date Stamp	19/11/2017 00:07:00
Frequency (MHz)	376.4610
Nucleus	19F
Number of Transients	16
Solvent	DMSO-d6

^{19}F NMR (376 MHz, *DMSO-d$_6$*) δ ppm -61.72 (s, 6 F), -119.64 (s, 1 F)

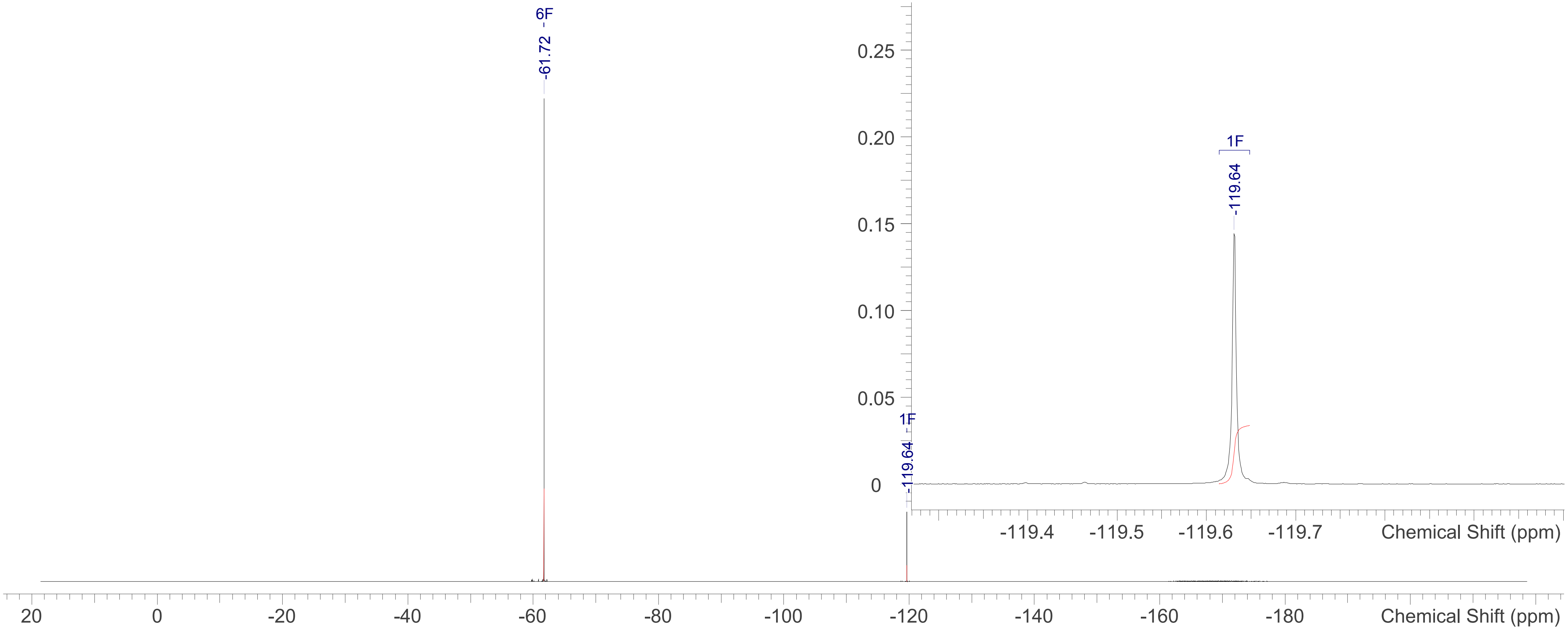

NVR-42_19F.spc

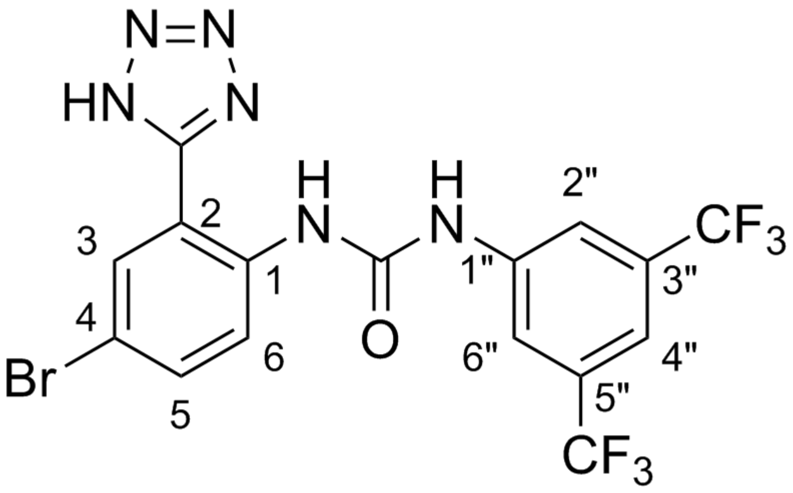

Shift (ppm)	H	m	J (Hz)	Assign
10.56	1	s	-	NH"
10.14	1	s	-	NH
8.32	1	d	9.0	6
8.20	2	br s	-	2", 6"
8.15	1	d	2.3	3
7.73	1	dd	9.0, 2.3	5
7.68	1	br s	-	4"

Acquisition Time (sec)	3.9846
Date	27 Mar 2019 13:35:10
Date Stamp	27 Mar 2019 13:35:10
Frequency (MHz)	400.0700
Nucleus	1H
Number of Transients	16
Solvent	DMSO-d6
Temperature (degree C)	25.000

^{1}H NMR (400 MHz, *DMSO-d$_6$*) δ ppm 10.56 (s, 1 H), 10.14 (s, 1 H), 8.32 (d, *J*=9.0 Hz, 1 H), 8.20 (br s, 2 H), 8.15 (d, *J*=2.3 Hz, 1 H), 7.73 (dd, *J*=9.0, 2.3 Hz, 1 H), 7.68 (br s, 1 H)

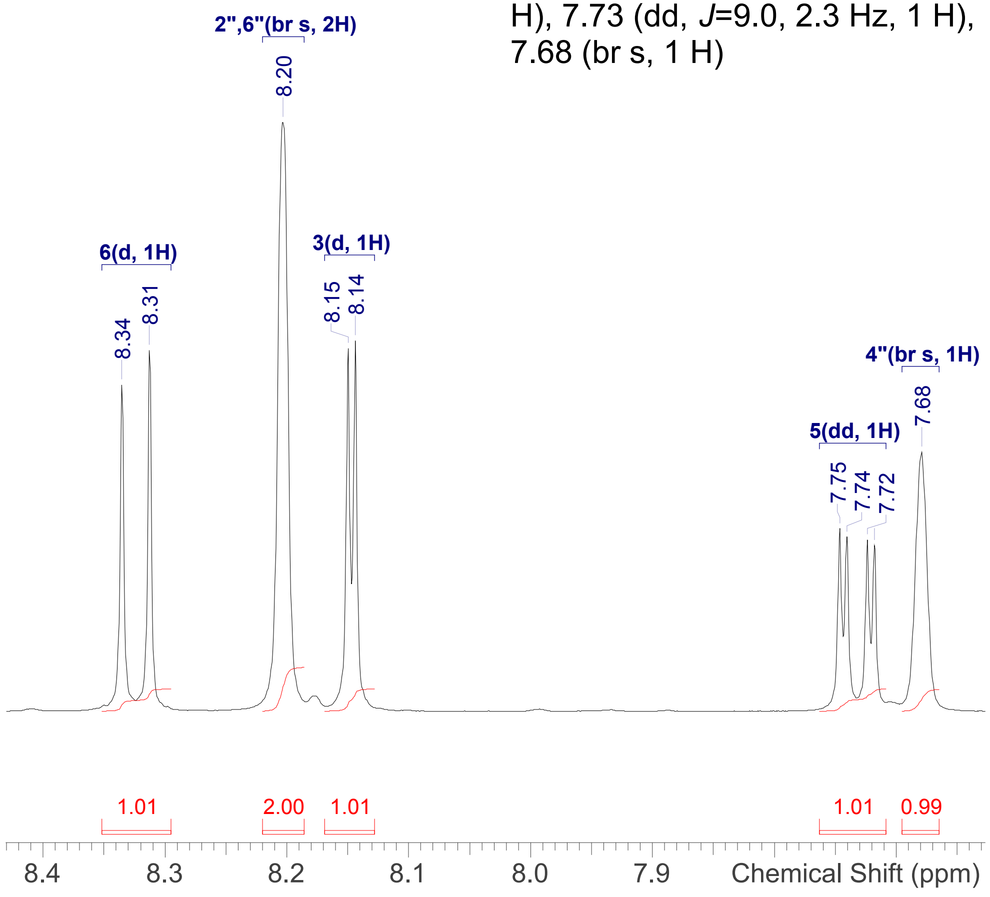

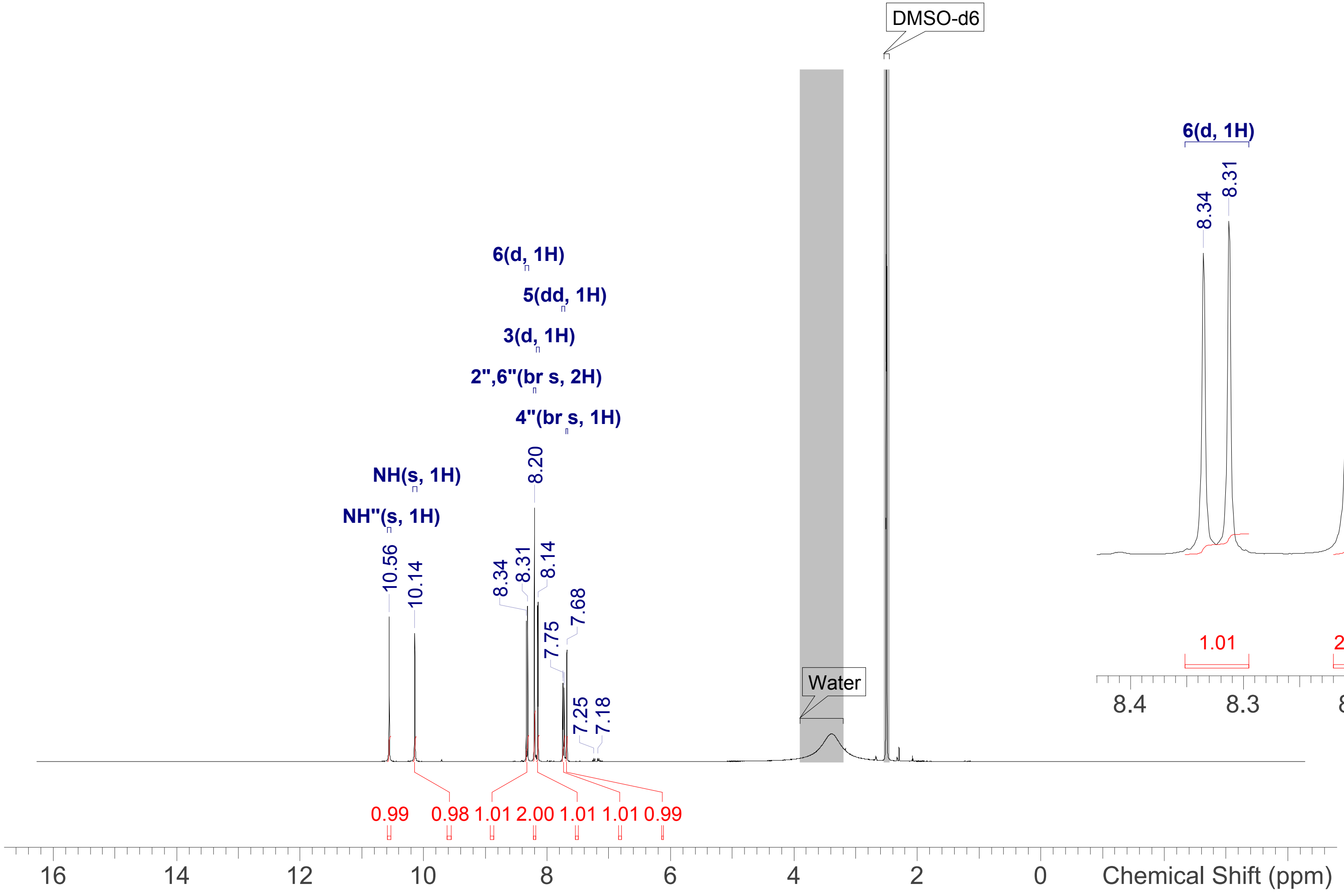

NS3728_1H.spectrus

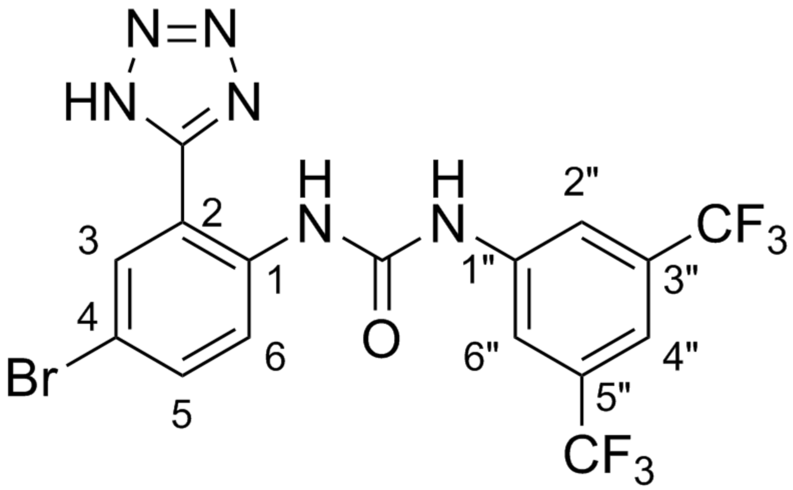

Shift (ppm)	C	m	J (Hz)	Assign
152.1	1	s	-	CO
141.7	1	s	-	1"
136.8	1	s	-	1
134.0	1	s	-	3
130.7	2	q	32.9	3", 5"
130.7	1	s	-	5
123.4	1	s	-	6
123.3	2	q	272.9	5"-CF3, 3"-CF3
118.3	2	br q	3.9	2", 6"
114.8	1	br spt	3.9	4"
114.2	1	s	-	2

Acquisition Time (sec)	1.0224
Date	27 Mar 2019 20:31:27
Date Stamp	27 Mar 2019 20:31:27
Frequency (MHz)	100.5977
Nucleus	13C
Number of Transients	256
Solvent	DMSO-d6
Temperature (degree C)	25.000

^{13}C NMR (101 MHz, *DMSO-d$_6$*) δ ppm 152.1 (s, 1 C), 141.7 (s, 1 C), 136.8 (s, 1 C), 134.0 (s, 1 C), 130.7 (s, 1 C), 130.7 (q, *J*=32.9 Hz, 2 C), 123.4 (s, 1 C), 123.3 (q, *J*=272.9 Hz, 2 C), 118.3 (br q, *J*=3.9 Hz, 2 C), 114.8 (br spt, *J*=3.9 Hz, 1 C), 114.2 (s, 1 C)

NS3728_13C

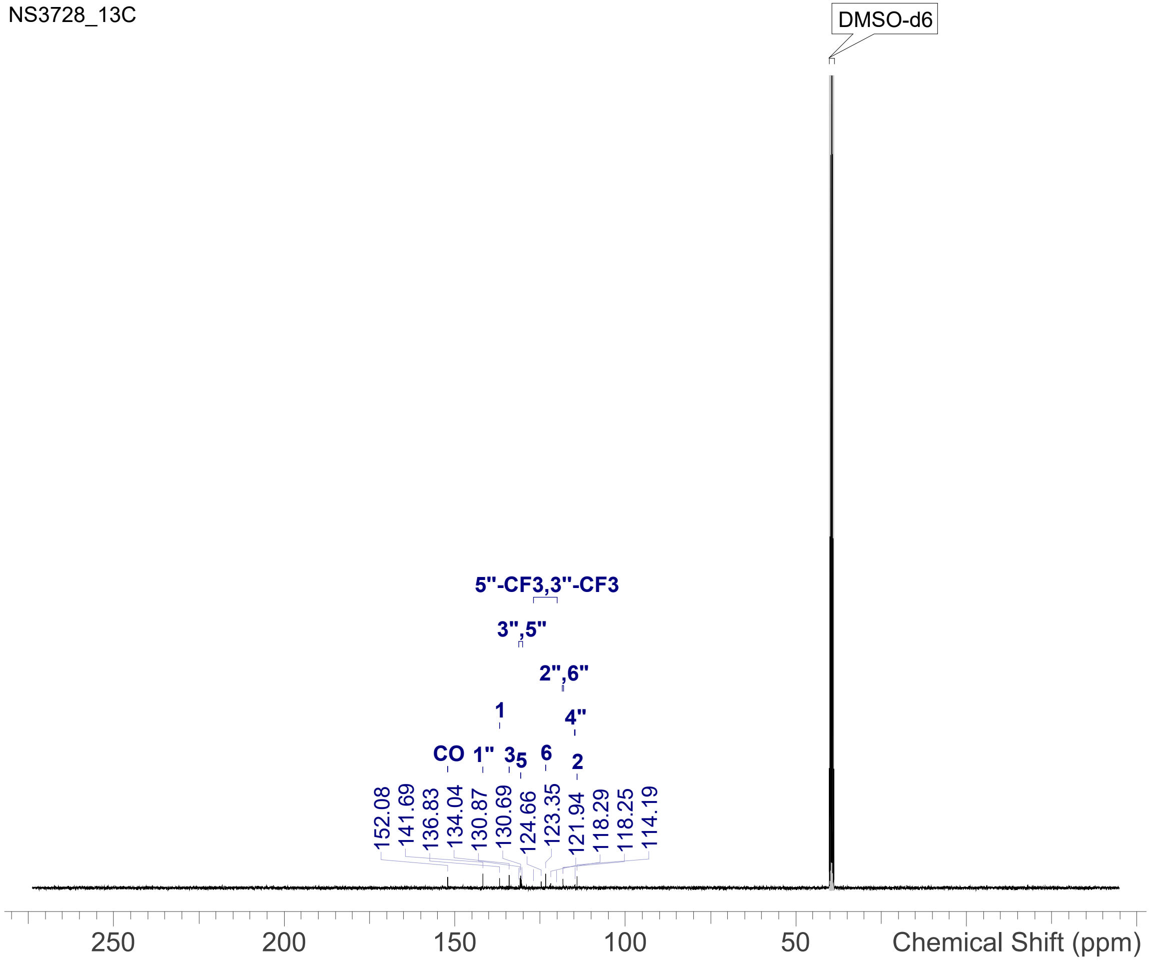

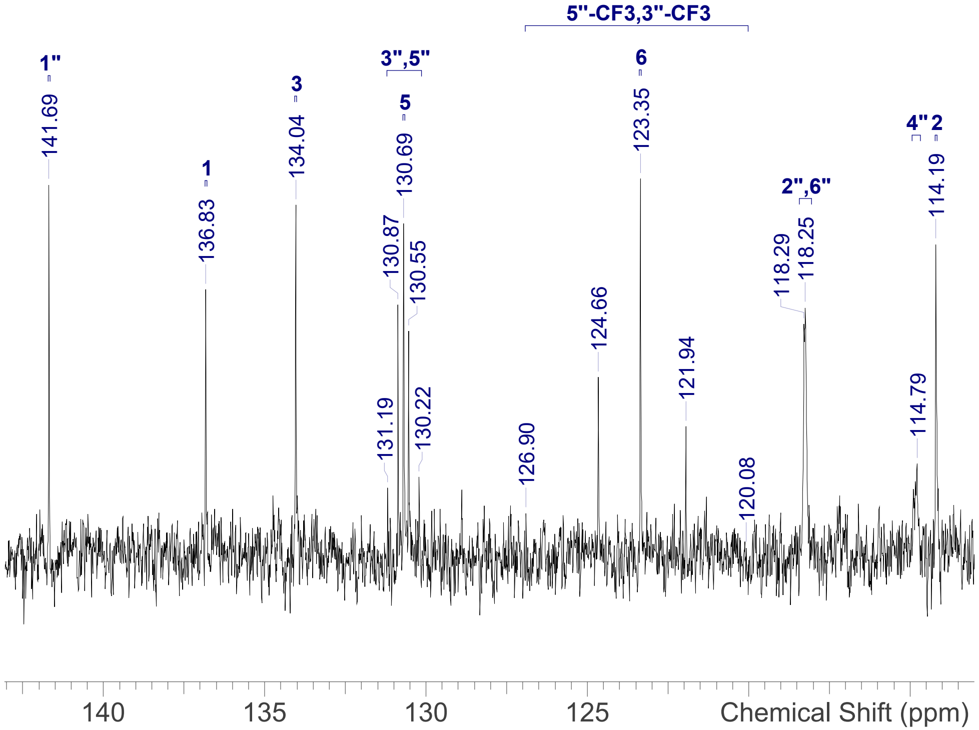

NS3728_13C.spectrus

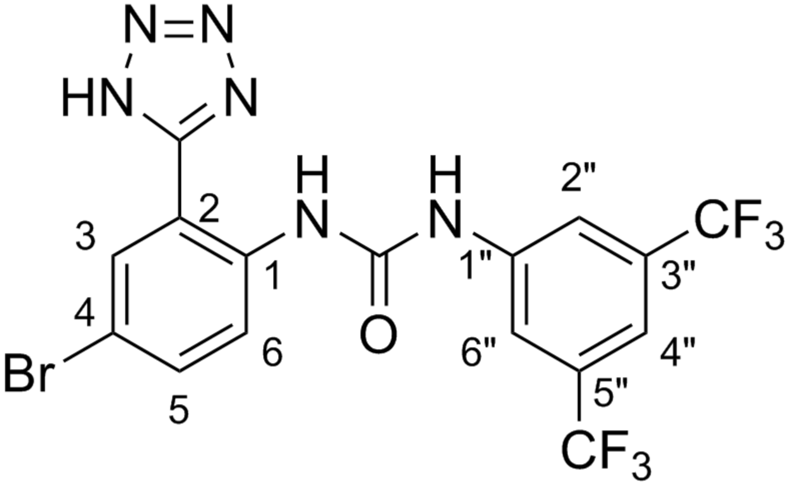

Shift (ppm)	F	m
-61.70	6	s

Acquisition Time (sec)	1.4680
Date	27 Mar 2019 21:37:36
Date Stamp	27 Mar 2019 21:37:36
Frequency (MHz)	376.4419
Nucleus	19F
Number of Transients	16
Solvent	DMSO-d6
Temperature (degree C)	24.999

^{19}F NMR (376 MHz, *DMSO-d_6*) δ ppm -61.70 (s, 6 F)

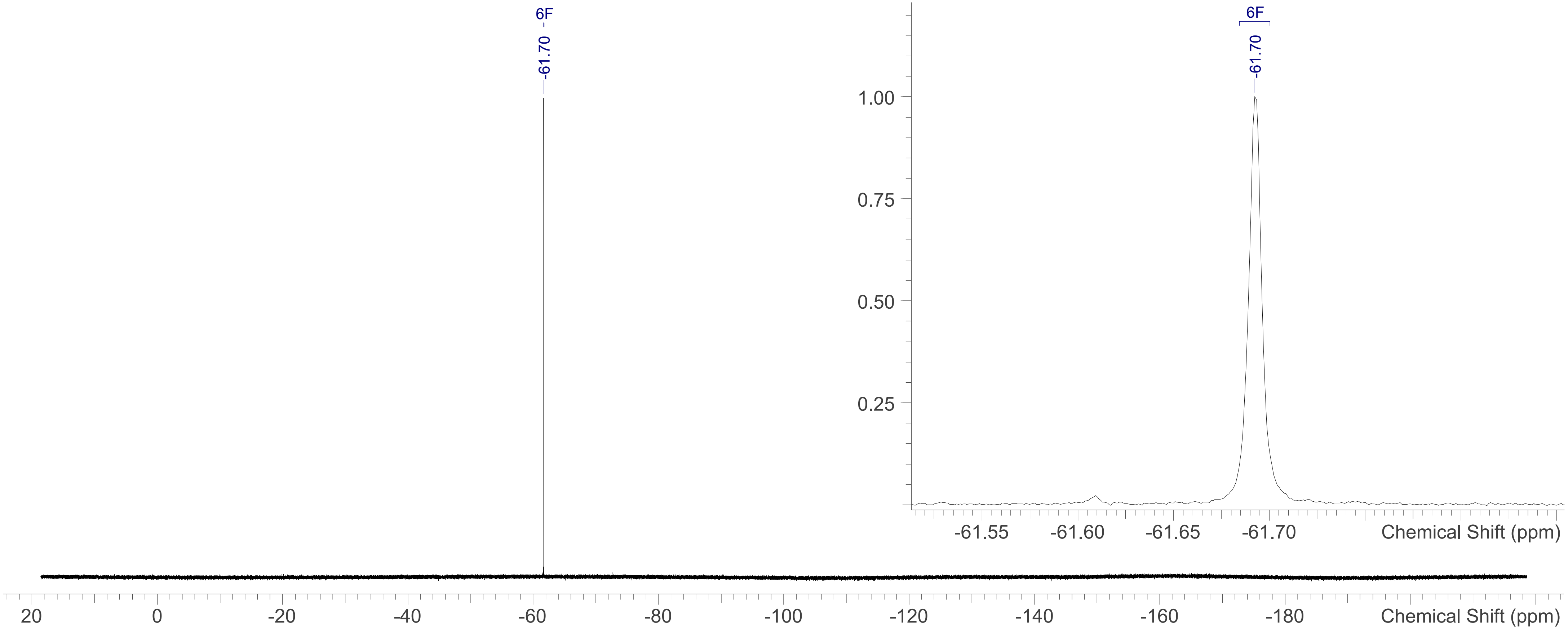

NS3728_19F.spectrus

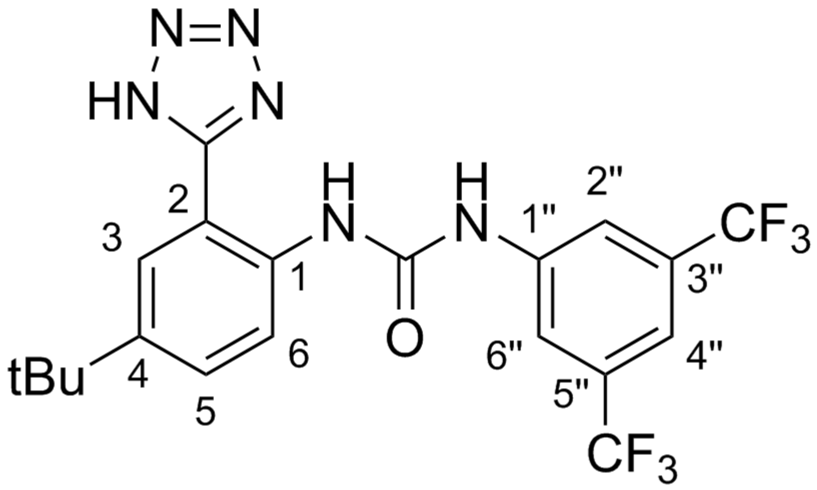

Shift (ppm)	H	m	J (Hz)	Assign
10.45	1	s	-	NH"
10.01	1	s	-	NH
8.23	1	d	8.9	H6
8.20	2	br s	-	H2", H6"
7.93	1	d	2.3	H3
7.65	1	br s	-	H4"
7.60	1	dd	8.8, 2.3	H5
1.34	9	s	-	Me, Me, Me

Acquisition Time (sec)	7.9692
Date	26/04/2018 17:48:00
Date Stamp	26/04/2018 17:48:00
Frequency (MHz)	400.0720
Nucleus	1H
Number of Transients	4
Solvent	DMSO-d6

^{1}H NMR (400 MHz, *DMSO-d_6*) δ ppm 10.45 (s, 1 H), 10.01 (s, 1 H), 8.23 (d, *J*=8.9 Hz, 1 H), 8.20 (br s, 2 H), 7.93 (d, *J*=2.3 Hz, 1 H), 7.65 (br s, 1 H), 7.60 (dd, *J*=8.8, 2.3 Hz, 1 H), 1.34 (s, 9 H)

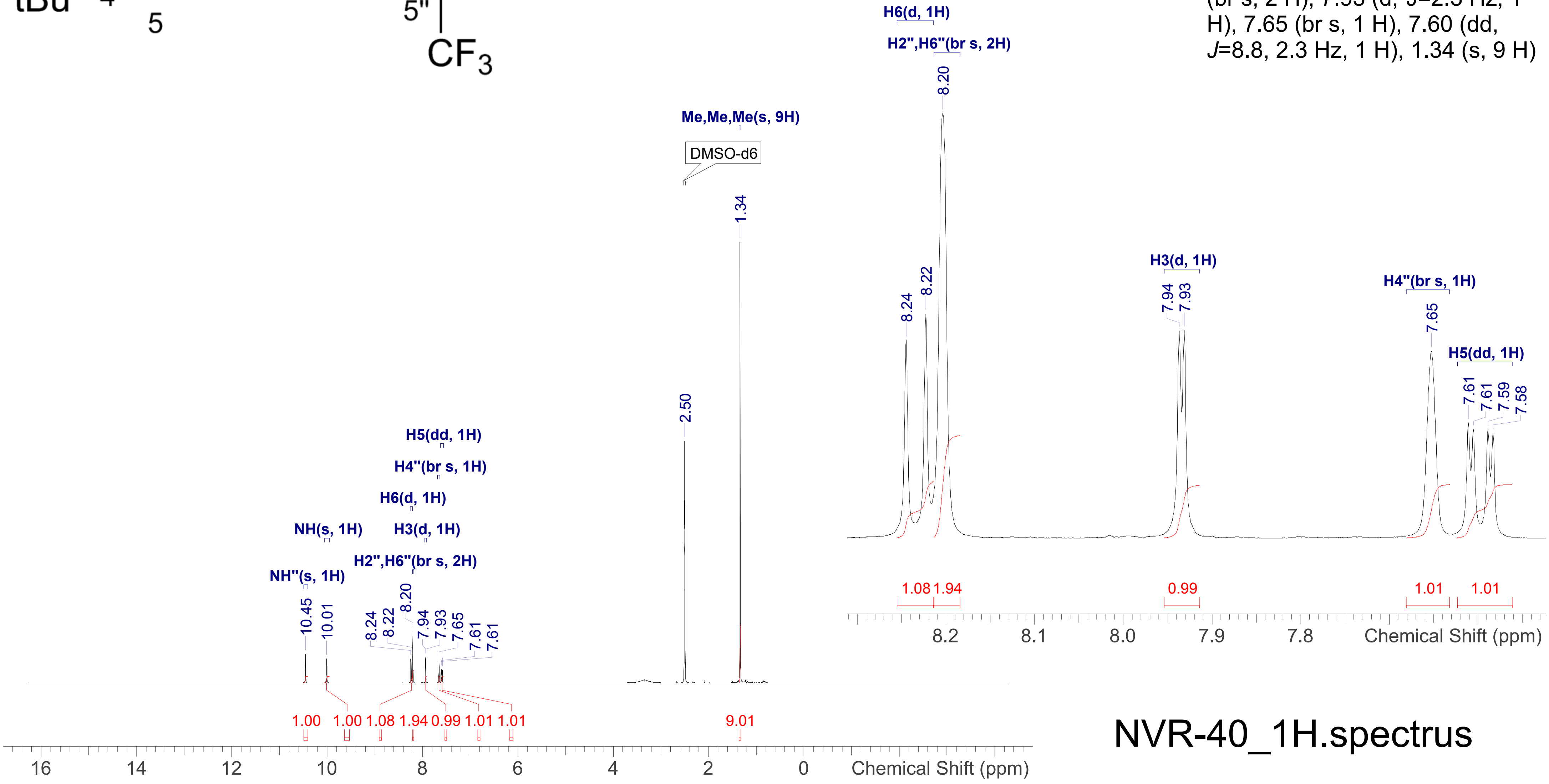

NVR-40_1H.spectrus

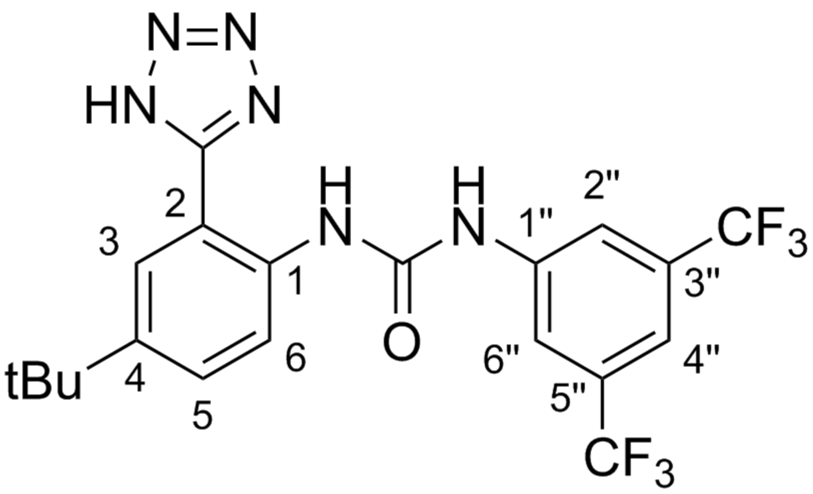

Shift (ppm)	C	m	J (Hz)	Assign
152.3	1	s	-	CO
145.4	1	s	-	4
141.9	1	s	-	1"
135.0	1	s	-	1
130.7	2	q	32.8	3", 5"
128.8	1	s	-	5
125.2	1	s	-	3
123.3	2	q	272.9	5"-CF3, 3"-CF3
121.5	1	s	-	6
118.0	2	br q	3.9	2", 6"
114.5	1	br spt	3.9	4"
34.3	1	s	-	C(CH3)3
31.0	3	s	-	Me, Me, Me

**Acquisition Time (sec)**	2.0447
**Date**	26/04/2018 17:53:00
**Date Stamp**	26/04/2018 17:53:00
**Frequency (MHz)**	100.6090
**Nucleus**	13C
**Number of Transients**	64
**Solvent**	DMSO-d6

^{13}C NMR (101 MHz, *DMSO-d_6*) δ ppm 152.3 (s, 1 C), 145.4 (s, 1 C), 141.9 (s, 1 C), 135.0 (s, 1 C), 130.7 (q, *J*=32.8 Hz, 2 C), 128.8 (s, 1 C), 125.2 (s, 1 C), 121.5 (s, 1 C), 123.3 (q, *J*=272.9 Hz, 2 C), 118.0 (br q, *J*=3.9 Hz, 2 C), 114.5 (br spt, *J*=3.9 Hz, 1 C), 34.3 (s, 1 C), 31.0 (s, 3 C)

NVR-40_13C

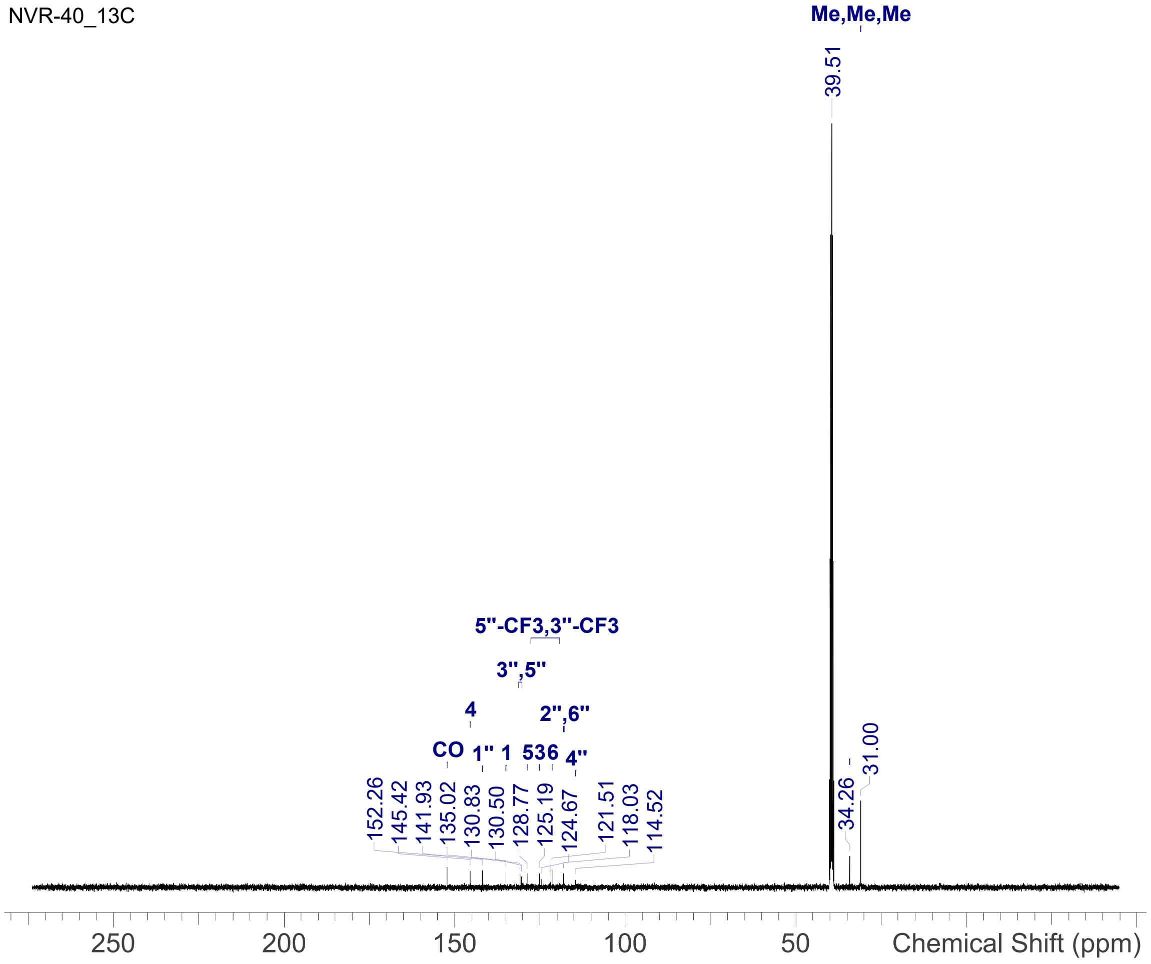

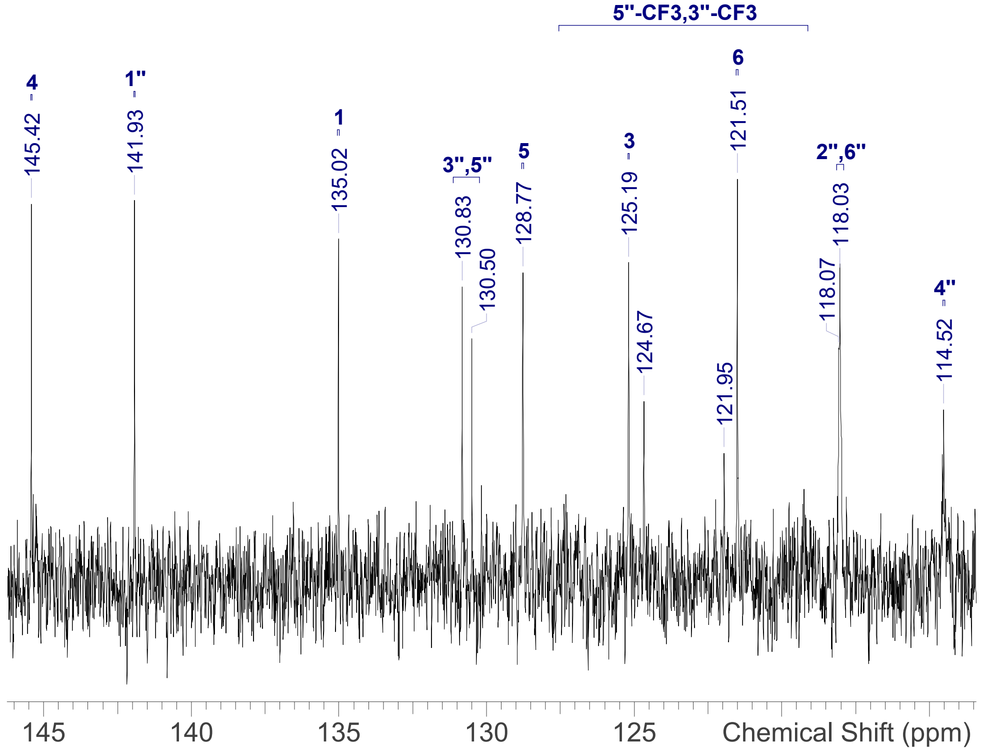

NVR-40_13C.spectrus

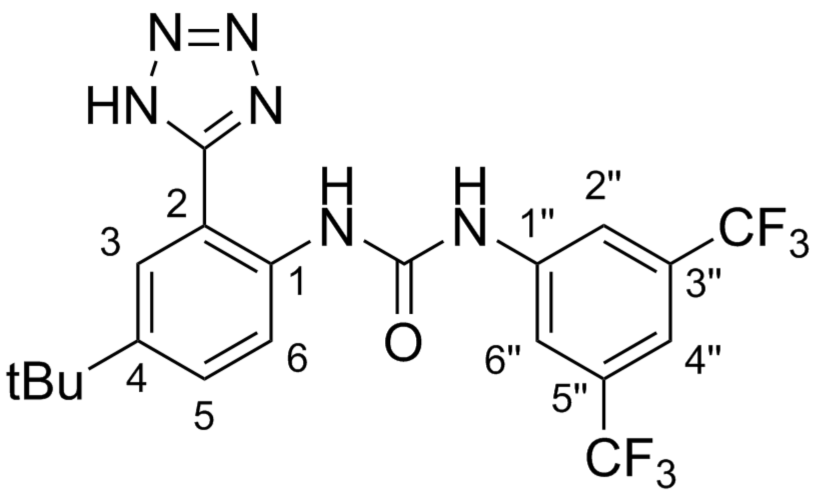

Shift (ppm)	F	m
-61.71	6	s

Acquisition Time (sec)	2.9360
Date	26/04/2018 18:00:00
Date Stamp	26/04/2018 18:00:00
Frequency (MHz)	376.4040
Nucleus	19F
Number of Transients	16
Solvent	DMSO-d6

^{19}F NMR (376 MHz, *DMSO-d$_6$*) δ ppm -61.71 (s, 6 F)

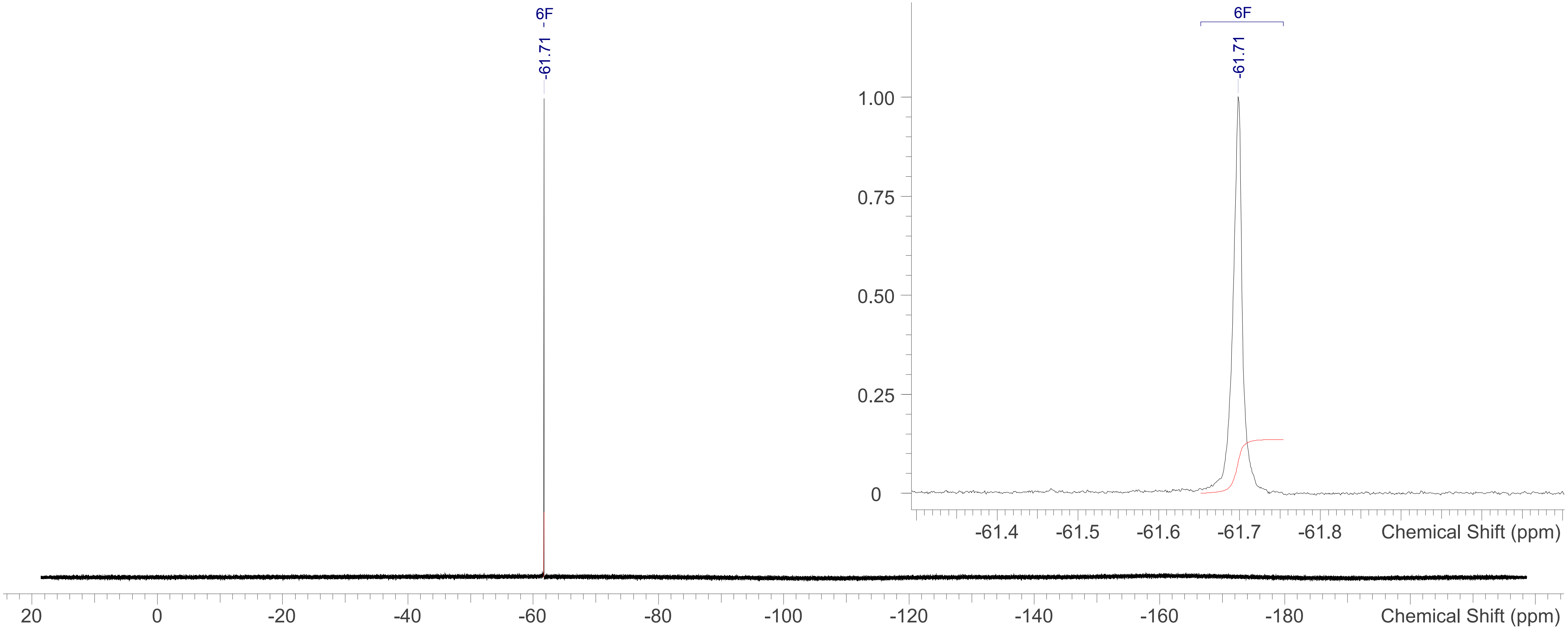

NVR-40_19F.spectrus

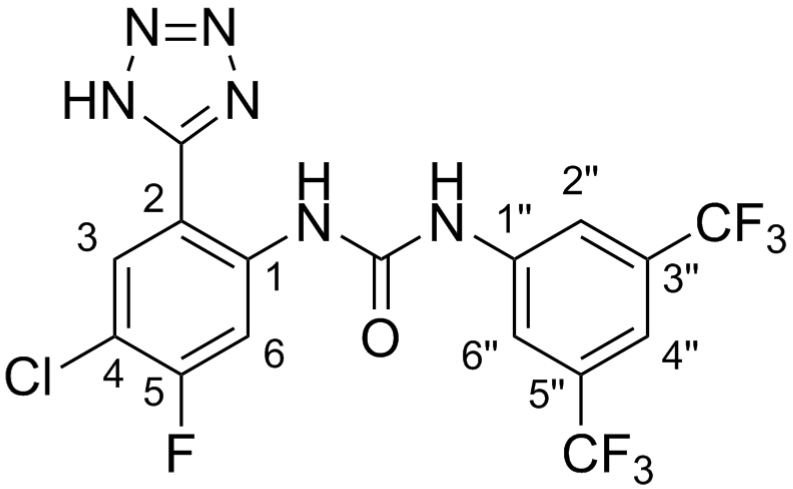

No.	Shift (ppm)	H	m	J (Hz)	Assign
1	10.62	1	s	-	NH
2	10.37	1	s	-	NH"
3	8.40	1	d	12.8	6
4	8.15	2	s	-	2", 6"
5	8.11	1	d	7.9	3
6	7.59	1	s	-	4"

Acquisition Time (sec)	6.5536
Date	26/07/2019 14:09:00
Date Stamp	26/07/2019 14:09:00
Frequency (MHz)	500.1930
Nucleus	1H
Number of Transients	16
Solvent	DMSO-d6

^{1}H NMR (500 MHz, *DMSO-d$_6$*) δ ppm 10.62 (s, 1 H), 10.37 (s, 1 H), 8.40 (d, *J*=12.8 Hz, 1 H), 8.15 (s, 2 H), 8.11 (d, *J*=7.9 Hz, 1 H), 7.59 (s, 1 H)

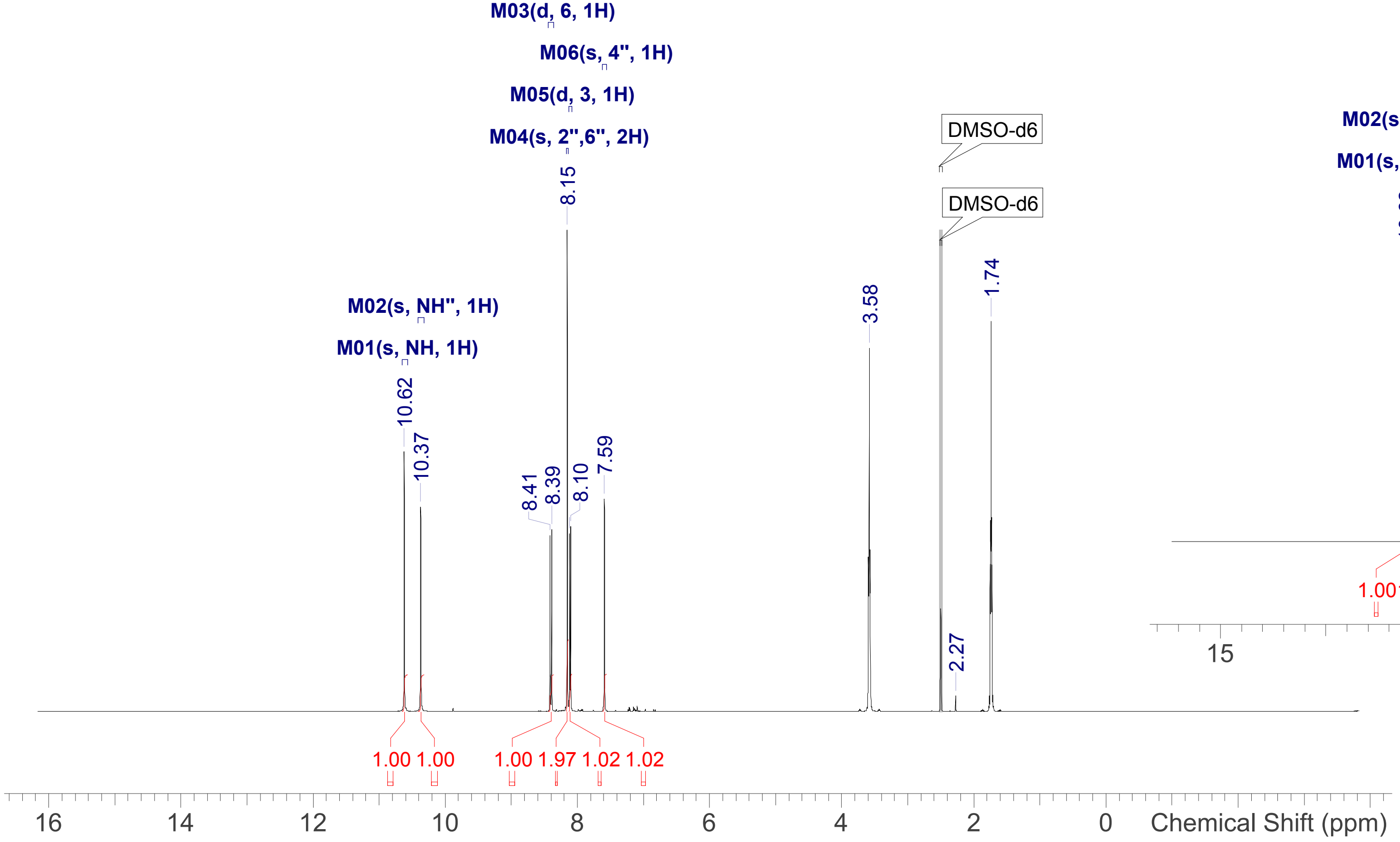

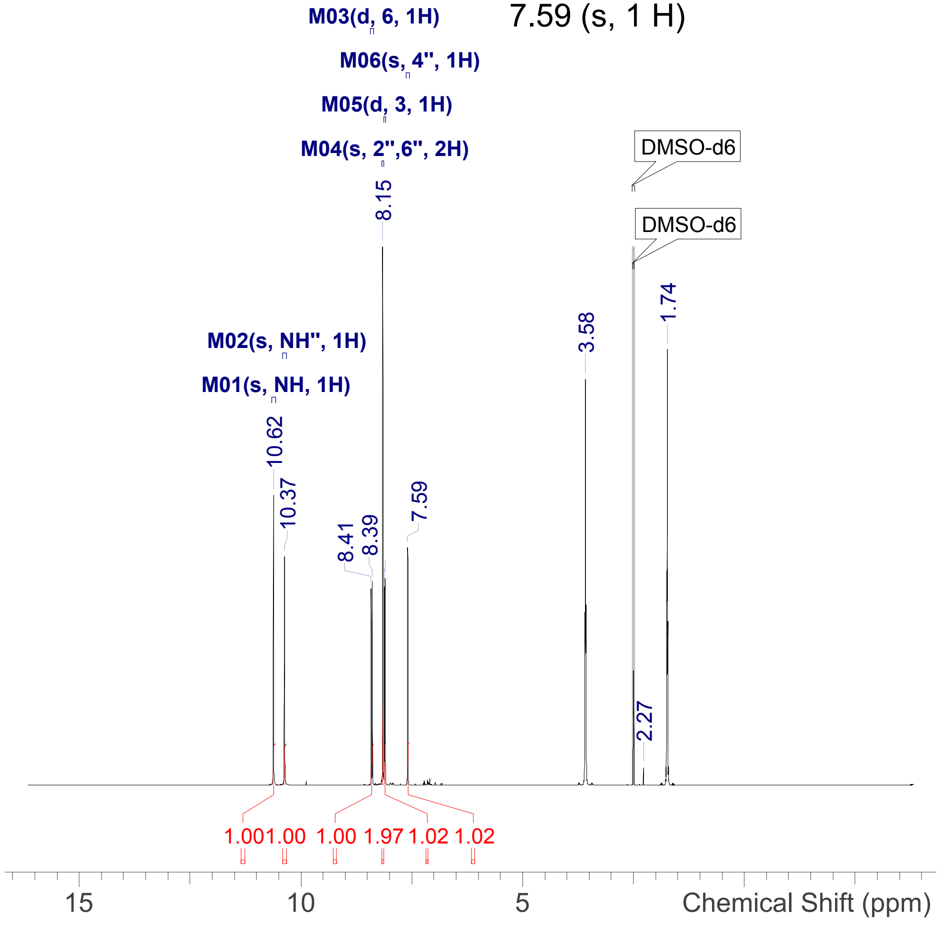

NVR-131_1H.spectrus

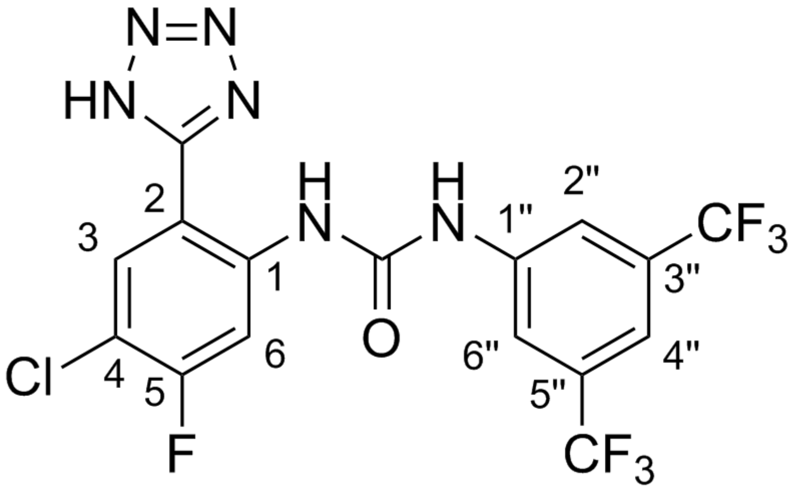

Shift (ppm)	C	m	J (Hz)	Assign
157.4	1	d	248.4	5
153.2	1	s	-	Tet-C
151.8	1	s	-	CO
141.5	1	s	-	1"
138.5	1	d	11.7	1
130.6	2	q	32.9	3", 5"
129.9	1	s	-	3
123.3	2	q	272.9	5"-CF3, 3"-CF3
118.2	2	br q	4.0	2", 6"
114.9	1	br spt	3.9	4"
112.7	1	d	18.6	4
109.0	1	d	2.0	2
108.5	1	d	27.4	6

Acquisition Time (sec)	1.0224
Date	26 Jul 2019 14:12:10
Date Stamp	26 Jul 2019 14:12:10
Frequency (MHz)	125.7729
Nucleus	13C
Number of Transients	32
Solvent	DMSO-d6
Temperature (degree C)	25.000

^{13}C NMR (126 MHz, *DMSO-d$_6$*) δ ppm 157.4 (d, *J*=248.4 Hz, 1 C), 153.2 (s, 1 C), 151.8 (s, 1 C), 141.5 (s, 1 C), 138.5 (d, *J*=11.7 Hz, 1 C), 130.6 (q, *J*=32.9 Hz, 2 C), 129.9 (s, 1 C), 123.3 (q, *J*=272.9 Hz, 2 C), 118.2 (br q, *J*=4.0 Hz, 2 C), 114.9 (br spt, *J*=3.9 Hz, 1 C), 112.7 (d, *J*=18.6 Hz, 1 C), 109.0 (d, *J*=2.0 Hz, 1 C), 108.5 (d, *J*=27.4 Hz, 1 C)

NVR-131_13C

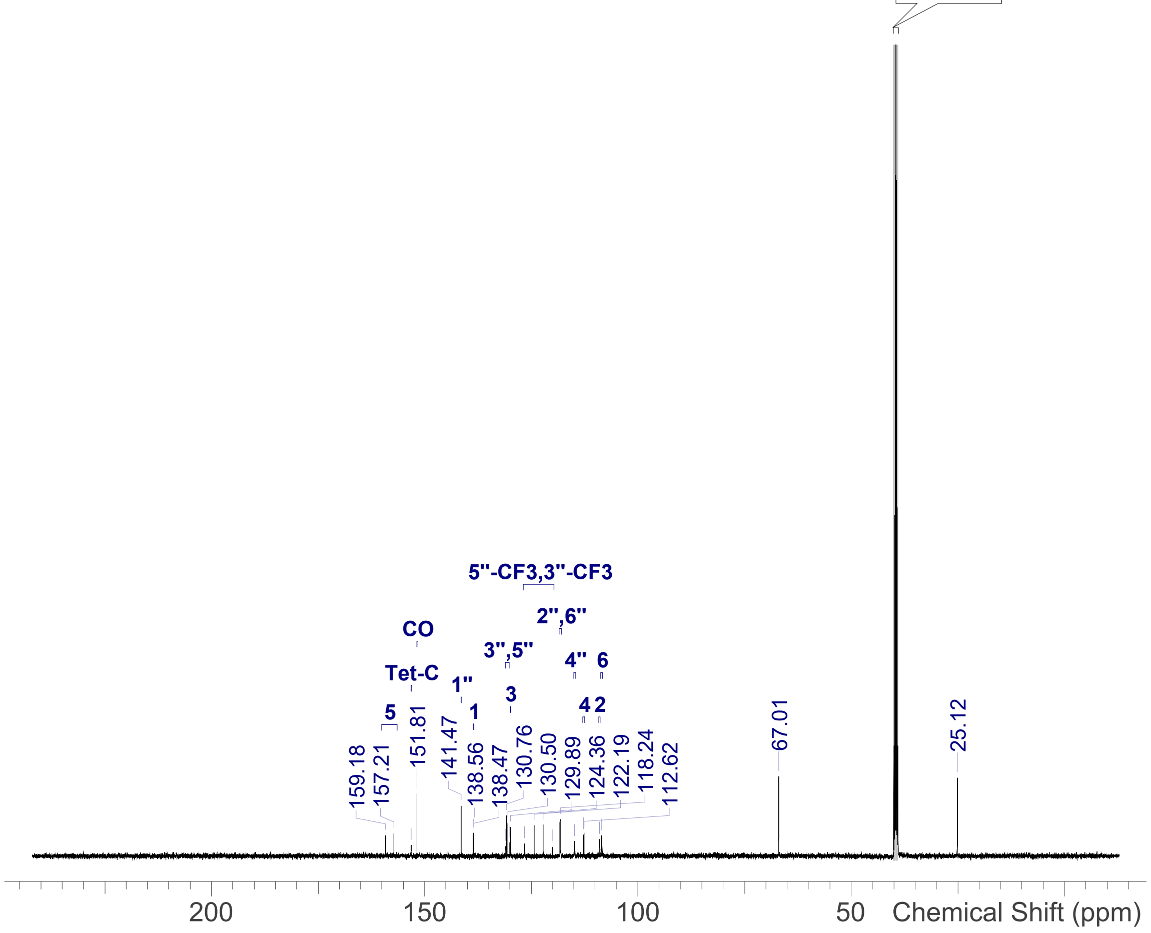

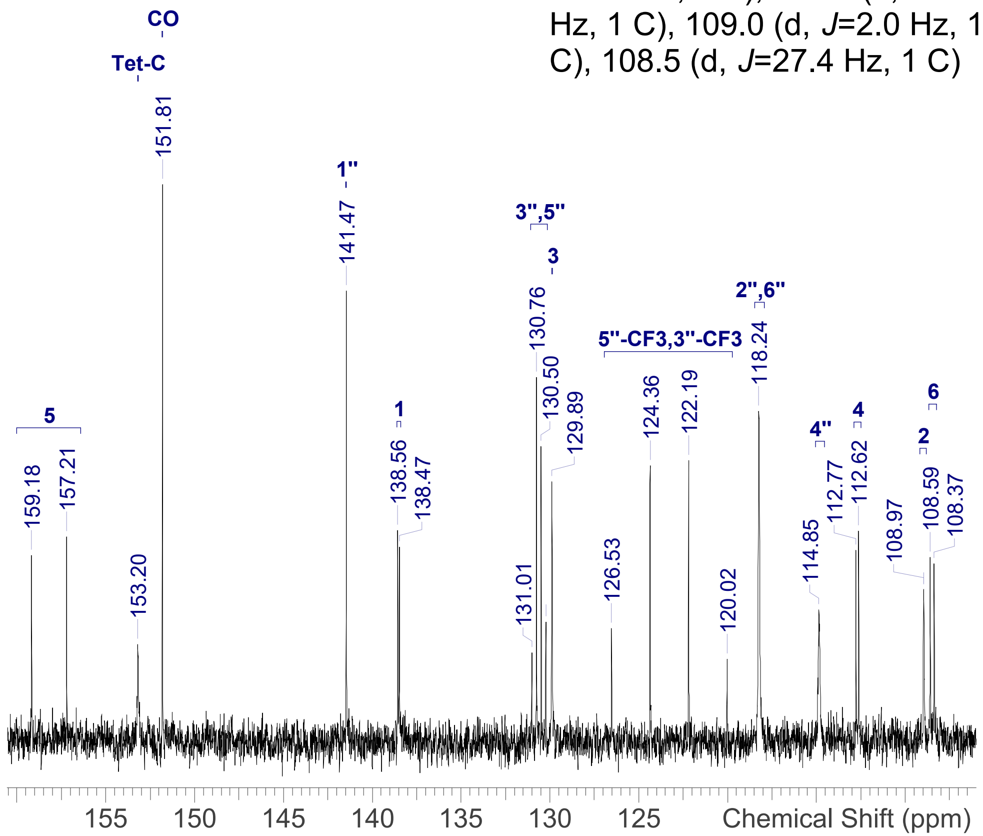

NVR-131_13C.spectrus

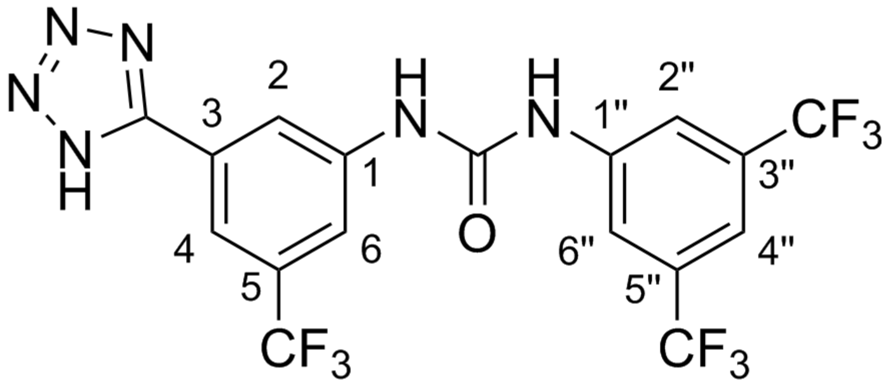

No.	Shift (ppm)	H	m	Assign
1	9.74	1	s	NH
2	9.70	1	s	NH''
3	8.47	1	s	6
4	8.17	2	s	2'', 6''
5	8.12	1	s	2
6	7.97	1	s	4
7	7.67	1	s	4''

Acquisition Time (sec)	8.1789
Date	21/06/2019 11:51:00
Date Stamp	21/06/2019 11:51:00
Frequency (MHz)	400.1320
Nucleus	1H
Number of Transients	16
Solvent	DMSO-d6

^{1}H NMR (400 MHz, *DMSO-d_6*) δ ppm 9.74 (s, 1 H), 9.70 (s, 1 H), 8.47 (s, 1 H), 8.17 (s, 2 H), 8.12 (s, 1 H), 7.97 (s, 1 H), 7.67 (s, 1 H)

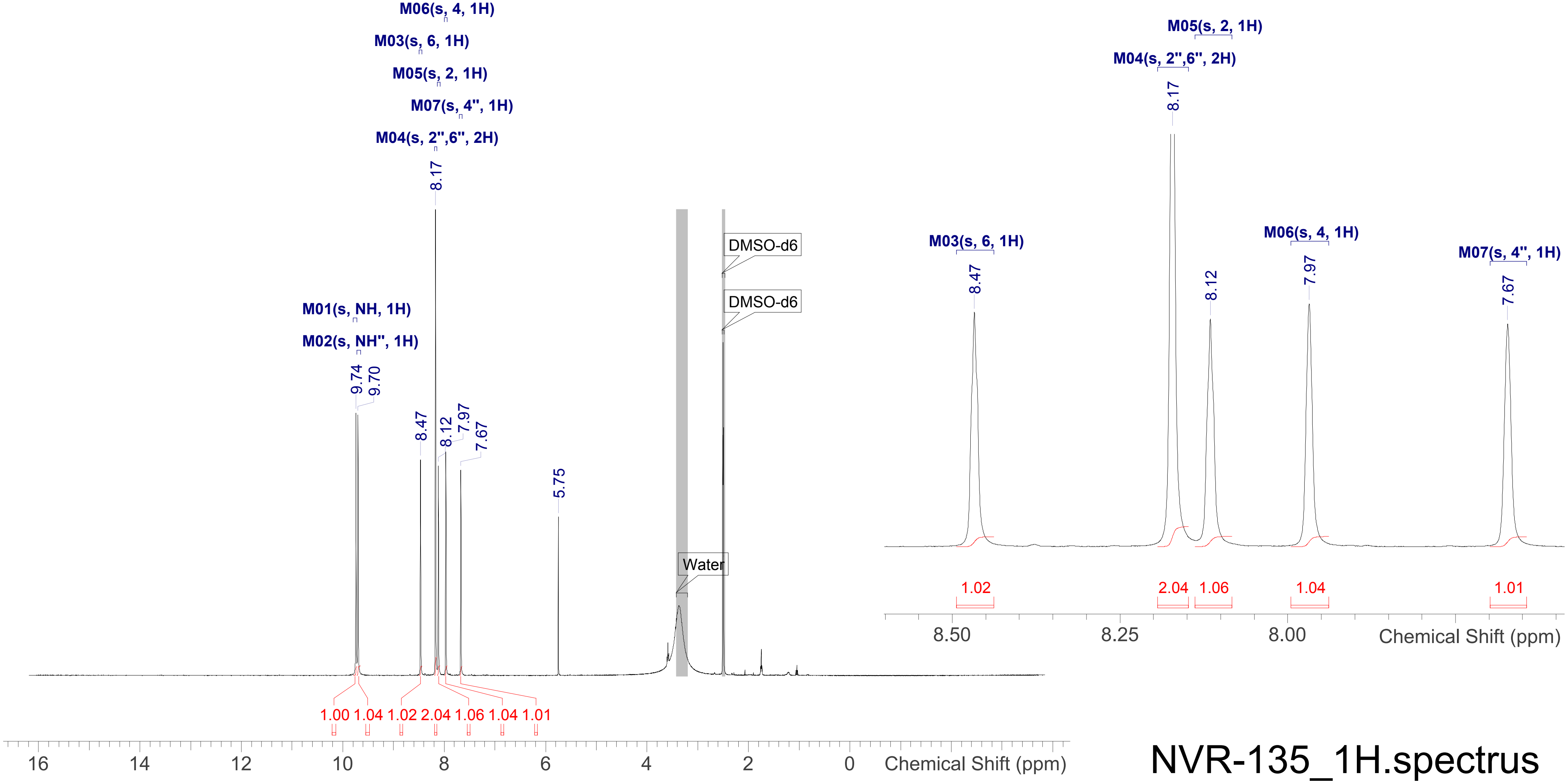

NVR-135_1H.spectrus

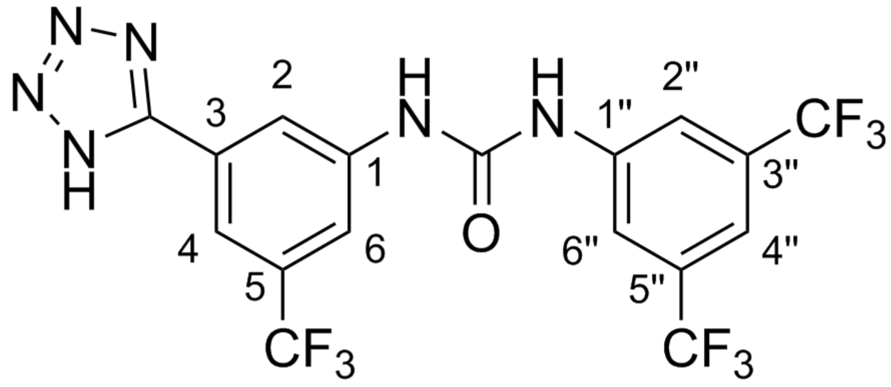

Shift (ppm)	C	m	J (Hz)	Assign
163.0	1	s	-	Tet-C
152.5	1	s	-	CO
141.4	1	s	-	1
141.2	1	s	-	1"
130.7	2	q	32.0	3", 5"
130.6	1	q	32.9	5
123.7	1	q	271.9	5-CF3
123.3	2	q	274.9	5"-CF3, 3"-CF3
120.3	1	s	-	2
118.4	2	br q	3.9	2", 6"
117.0	1	q	3.9	4
116.8	1	q	3.9	6
114.9	1	br spt	3.9	4"

Acquisition Time (sec)	1.0224
Date	22 Jun 2019 21:27:52
Date Stamp	22 Jun 2019 21:27:52
Frequency (MHz)	100.6128
Nucleus	13C
Number of Transients	256
Solvent	DMSO-d6
Temperature (degree C)	23.200

^{13}C NMR (101 MHz, *DMSO-d*$_6$) δ ppm 163.0 (s, 1 C), 152.5 (s, 1 C), 141.4 (s, 1 C), 141.2 (s, 1 C), 130.6 (q, *J*=32.9 Hz, 1 C), 130.7 (q, *J*=32.0 Hz, 2 C), 120.3 (s, 1 C), 123.7 (q, *J*=271.9 Hz, 1 C), 123.3 (q, *J*=274.9 Hz, 2 C), 118.4 (br q, *J*=3.9 Hz, 2 C), 117.0 (q, *J*=3.9 Hz, 1 C), 116.8 (q, *J*=3.9 Hz, 1 C), 114.9 (br spt, *J*=3.9 Hz, 1 C)

NVR-135_13C

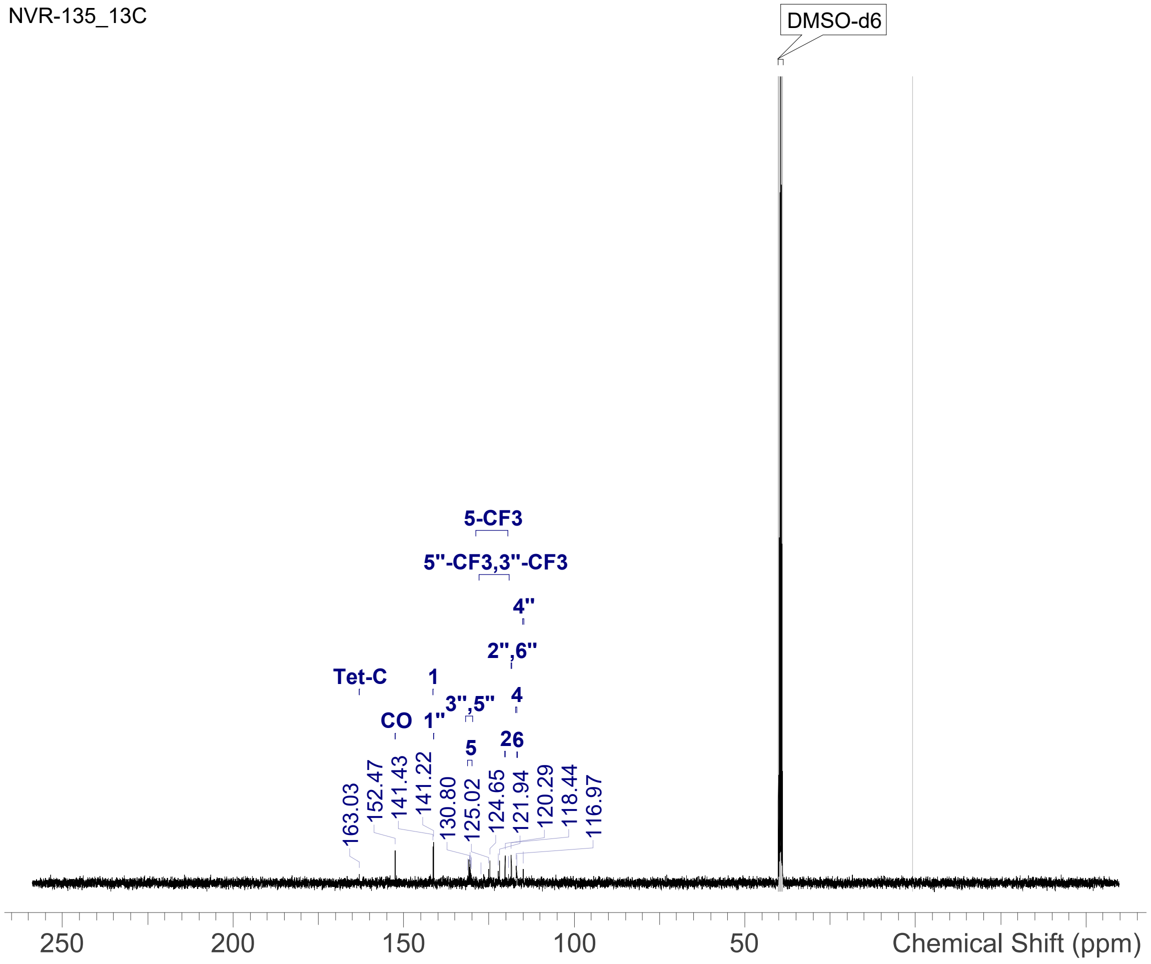

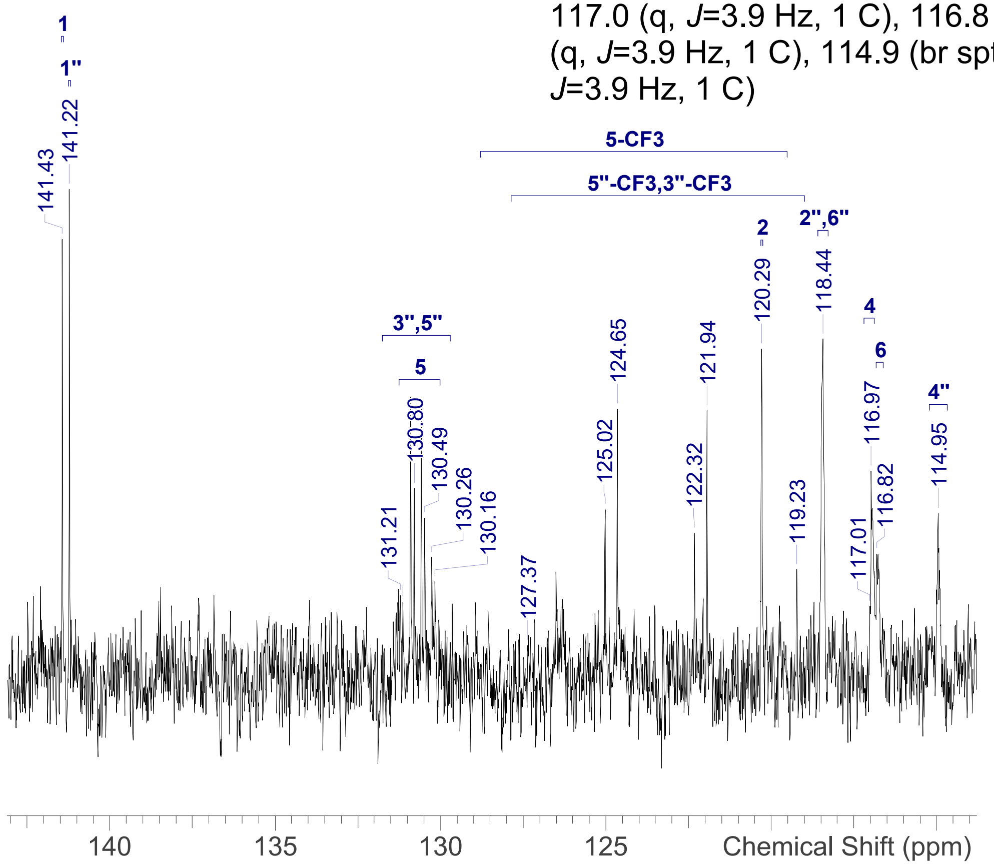

NVR-135_13C.spectrus

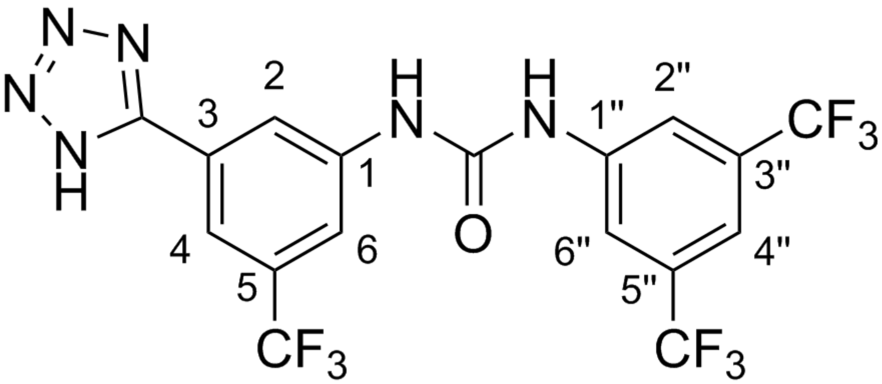

Shift (ppm)	F	m
-61.68	6	s
-61.70	3	s

Acquisition Time (sec)	2.9360
Date	22/06/2019 21:38:00
Date Stamp	22/06/2019 21:38:00
Frequency (MHz)	376.4610
Nucleus	19F
Number of Transients	16
Solvent	DMSO-d6

^{19}F NMR (376 MHz, *DMSO-d_6*) δ ppm -61.68 (s, 6 F), -61.70 (s, 3 F)

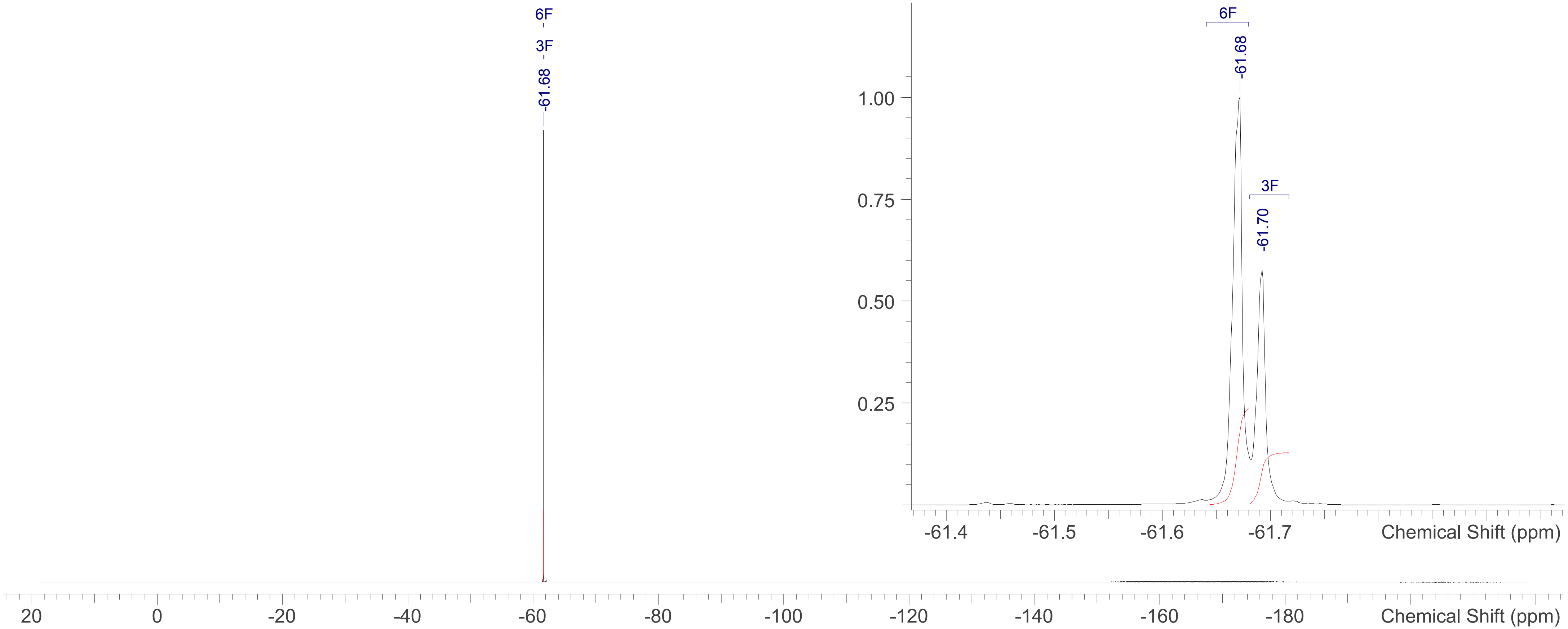

NVR-135_19F.spectrus

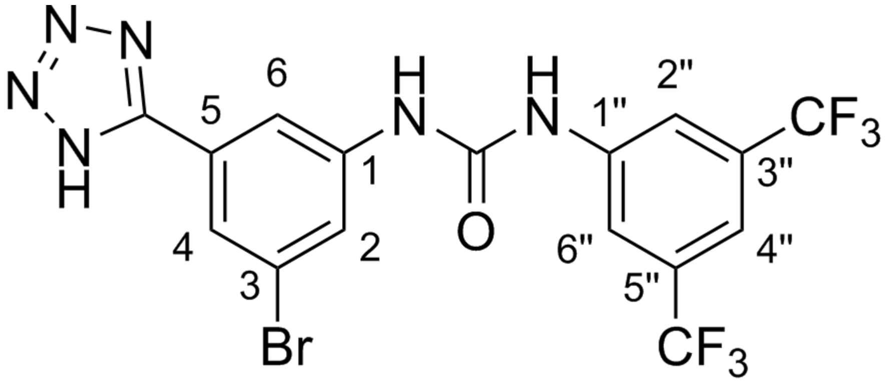

No.	Shift (ppm)	H	m	Assign
1	10.96	1	br s	NH
2	10.89	1	br s	NH"
3	8.30	2	s	6", 2"
4	8.21	1	br s	6
5	8.04	1	br s	4
6	8.02	1	br s	2
7	7.82	1	s	4"

Acquisition Time (sec)	6.5536
Date	29/07/2019 14:31:00
Date Stamp	29/07/2019 14:31:00
Frequency (MHz)	500.1930
Nucleus	1H
Number of Transients	16
Solvent	DMSO-d6

^{1}H NMR (500 MHz, *DMSO-d_6*) δ ppm 10.96 (br s, 1 H), 10.89 (br s, 1 H), 8.30 (s, 2 H), 8.21 (br s, 1 H), 8.04 (br s, 1 H), 8.02 (br s, 1 H), 7.82 (s, 1 H)

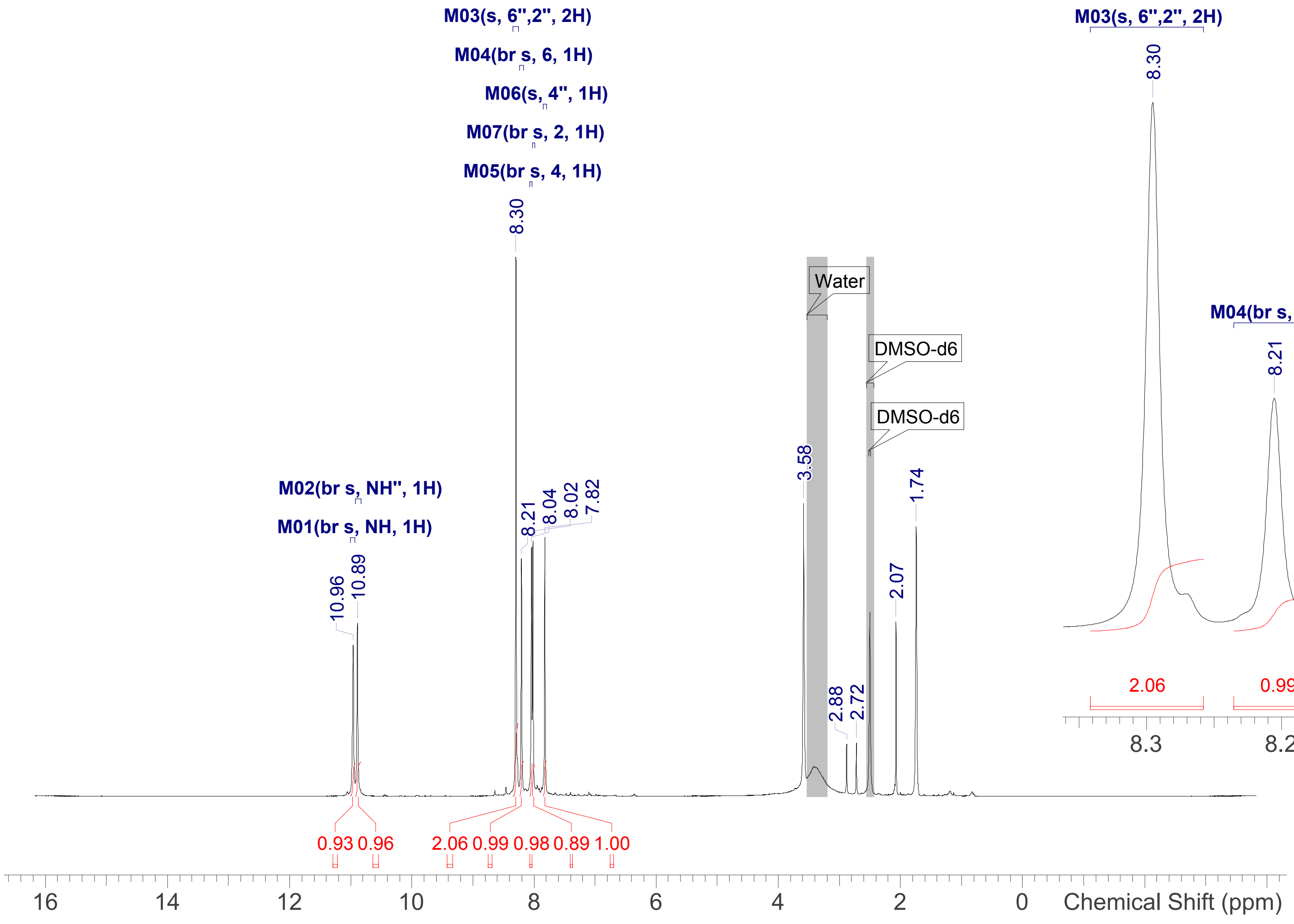

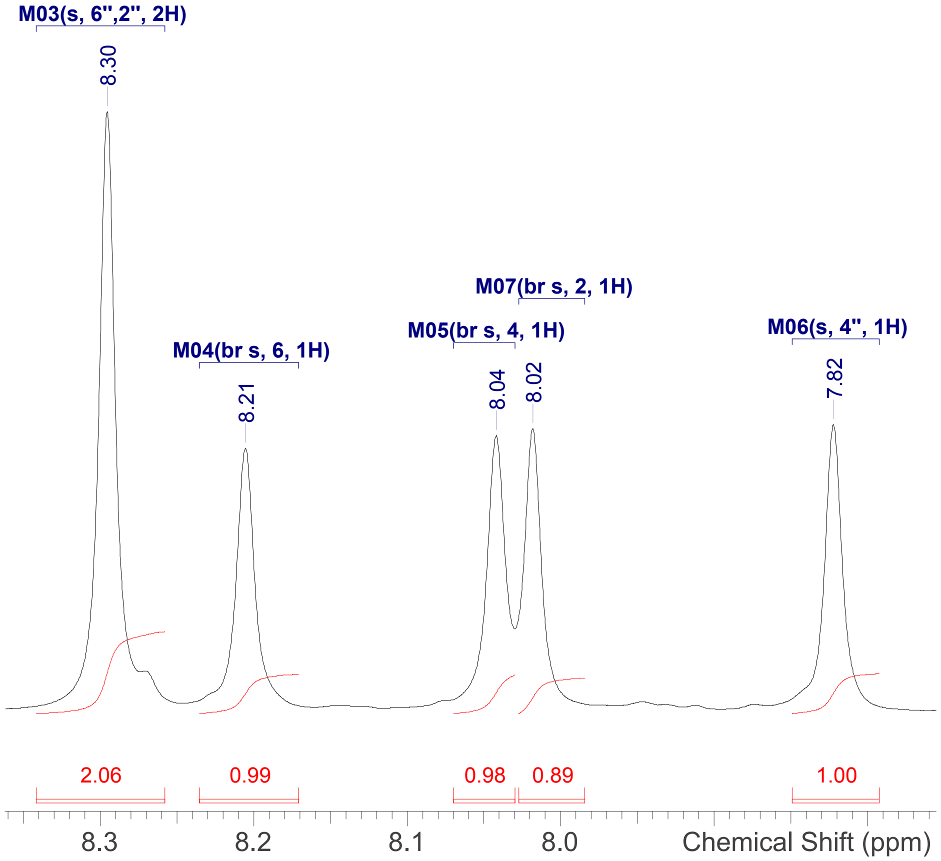

NVR-139_1H.spectrus

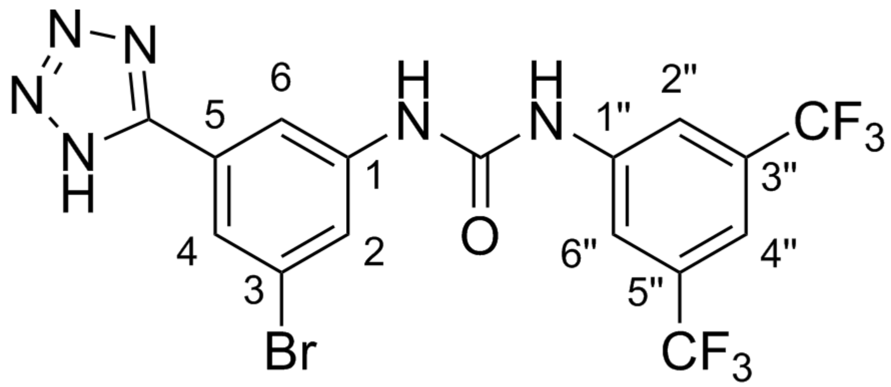

Shift (ppm)	C	m	J (Hz)	Assign
153.5	1	s	-	Tet-C
152.1	1	s	-	CO
141.8	1	s	-	1''
136.9	1	s	-	1
134.0	1	s	-	4
130.8	1	s	-	6
130.7	2	q	32.3	3'', 5''
123.3	1	s	-	2
123.3	2	q	272.9	5''-CF3, 3''-CF3
118.2	2	br q	3.9	2'', 6''
114.7	1	br spt	3.9	4''
114.2	1	s	-	3
114.1	1	s	-	5

Acquisition Time (sec)	1.0224
Date	02 Aug 2019 13:38:33
Date Stamp	02 Aug 2019 13:38:33
Frequency (MHz)	125.7729
Nucleus	13C
Number of Transients	32
Solvent	DMSO-d6
Temperature (degree C)	25.000

^{13}C NMR (126 MHz, *DMSO-d_6*) δ ppm 153.5 (s, 1 C), 152.1 (s, 1 C), 141.8 (s, 1 C), 136.9 (s, 1 C), 134.0 (s, 1 C), 130.8 (s, 1 C), 130.7 (q, *J*=32.3 Hz, 2 C), 123.3 (s, 1 C), 123.3 (q, *J*=272.9 Hz, 2 C), 118.2 (br q, *J*=3.9 Hz, 2 C), 114.7 (br spt, *J*=3.9 Hz, 1 C), 114.2 (s, 1 C), 114.1 (s, 1 C)

NVR-139_13C

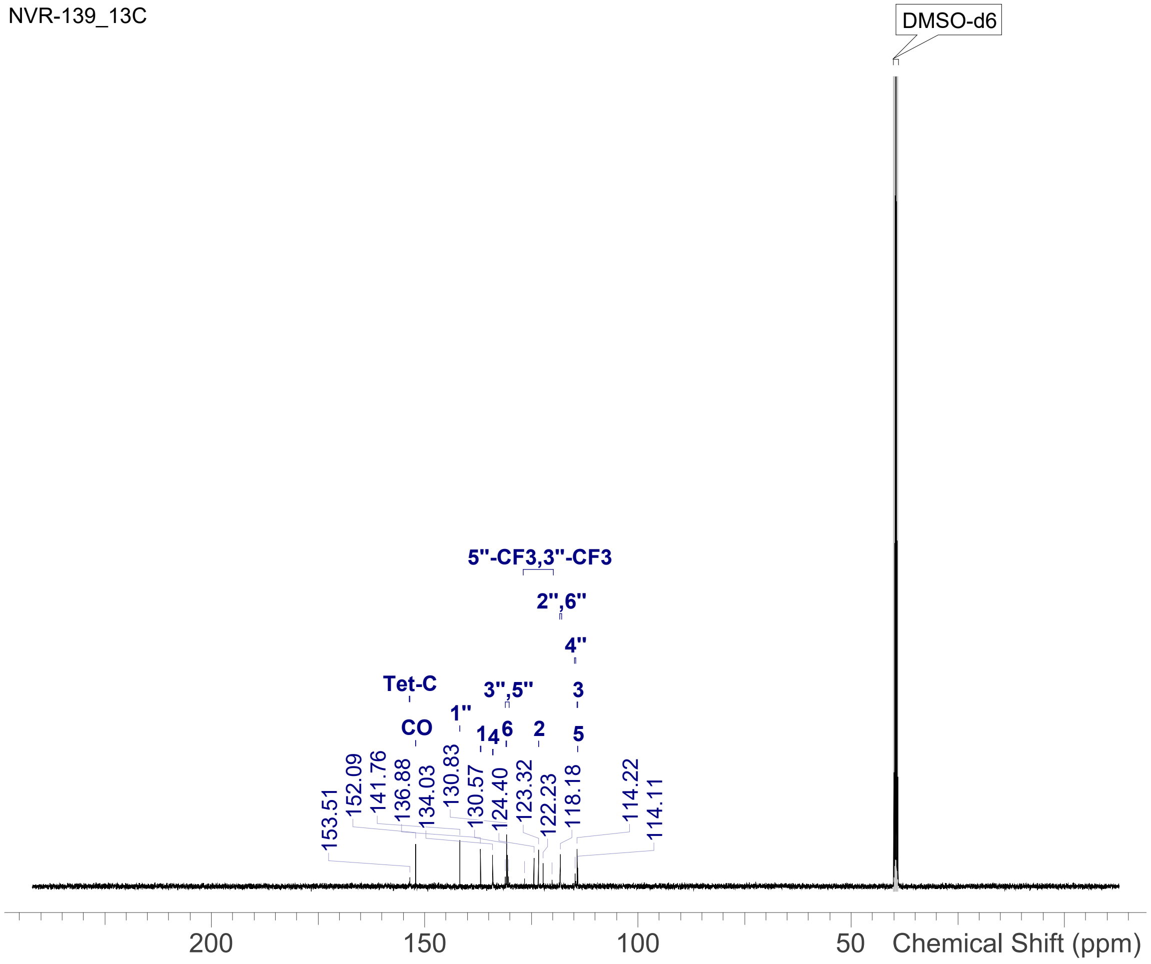

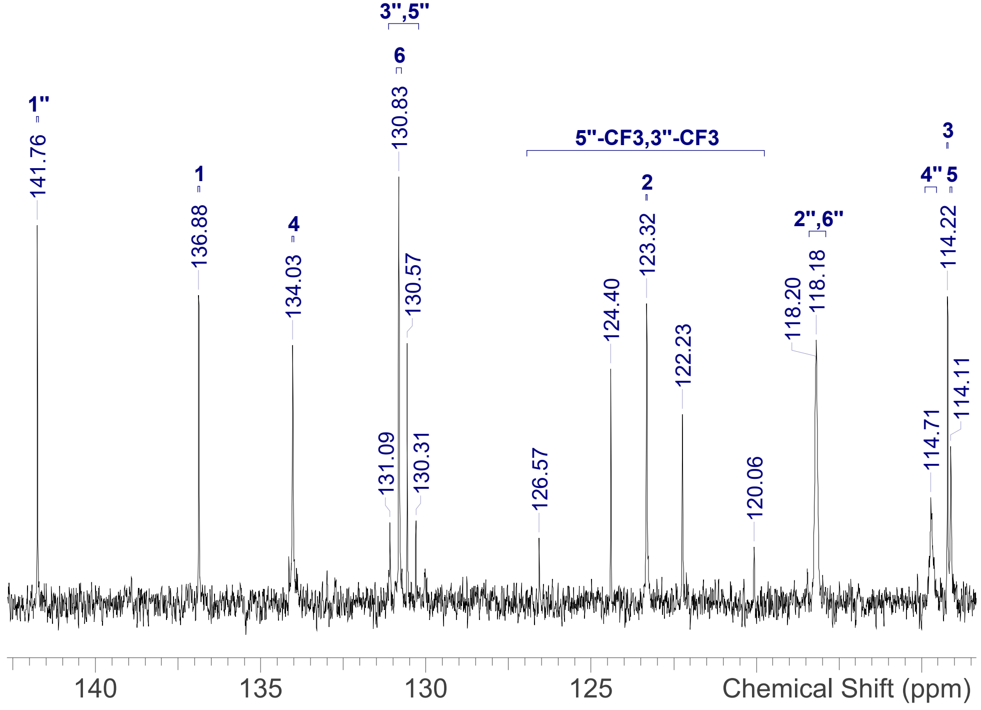

NVR-139_13C.spectrus NVR-117 HRMS

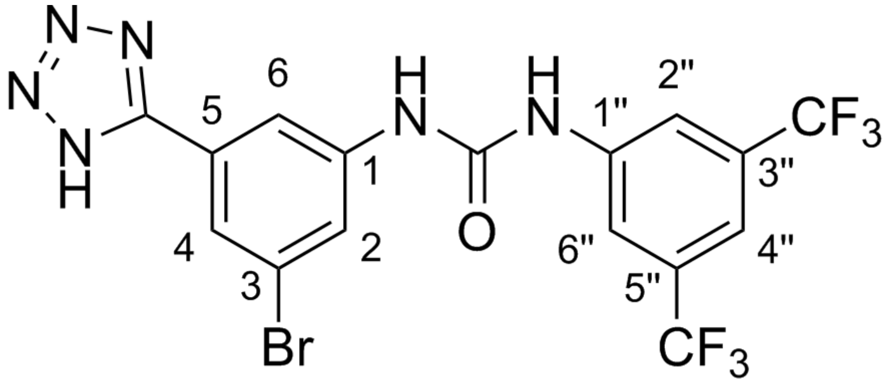

Shift (ppm)	F	m
-61.54	6	s

Acquisition Time (sec)	0.3670
Date	29 Jul 2019 14:39:27
Date Stamp	29 Jul 2019 14:39:27
Frequency (MHz)	470.6488
Nucleus	19F
Number of Transients	16
Solvent	DMSO-d6
Temperature (degree C)	25.002

^{19}F NMR (471 MHz, *DMSO-d_6*) δ ppm -61.54 (s, 6 F)

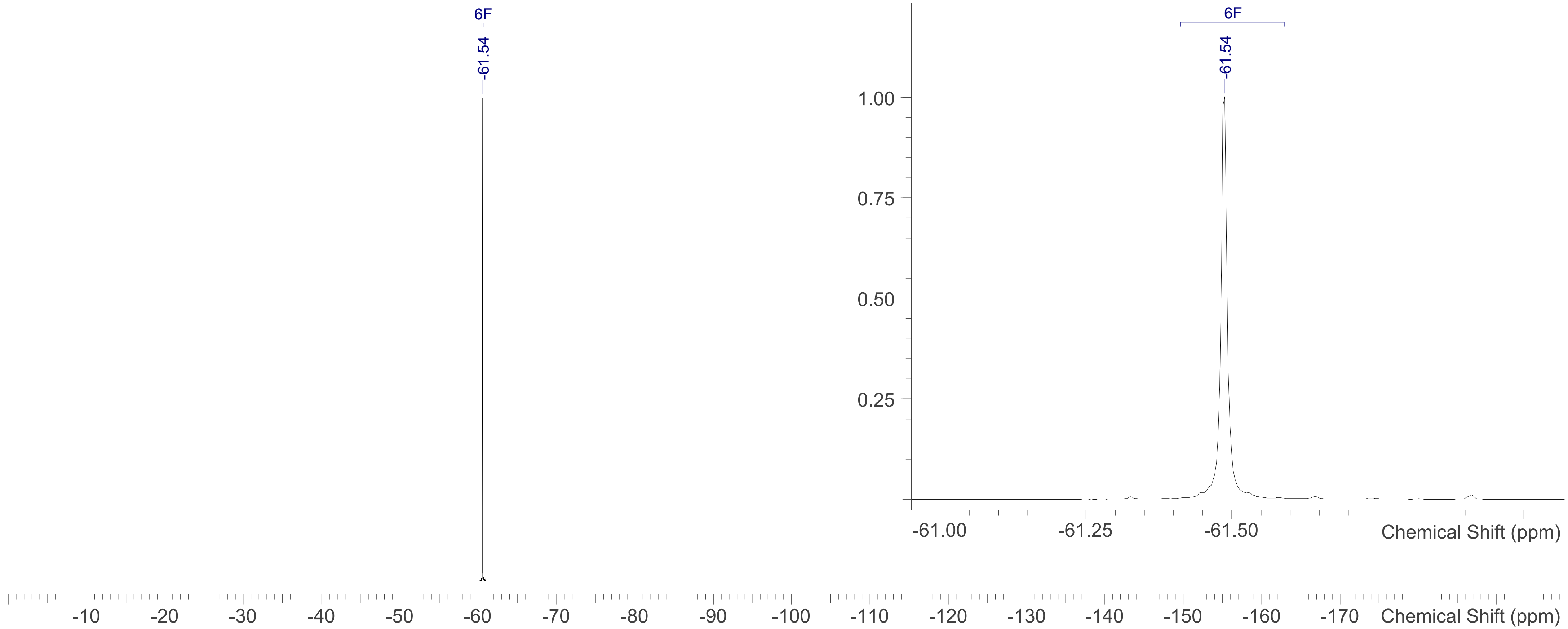

NVR-139_19F.spectrus

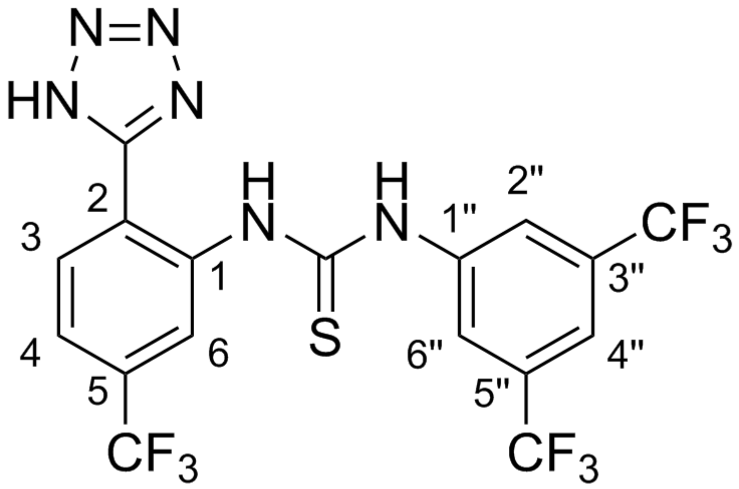

No.	Shift (ppm)	H	m	J (Hz)	Assign
1	10.90	1	s	-	NH
2	10.55	1	s	-	NH"
3	8.27	2	s	-	6", 2"
4	8.17	1	br s	-	6
5	8.12	1	d	8.2	3
6	7.85	1	br s	-	4"
7	7.82	1	dd	8.3, 1.3	4

Acquisition Time (sec)	8.1789
Date	20/06/2019 17:21:00
Date Stamp	20/06/2019 17:21:00
Frequency (MHz)	400.1320
Nucleus	1H
Number of Transients	16
Solvent	DMSO-d6

^{1}H NMR (400 MHz, *DMSO-d_6*) δ ppm 10.90 (s, 1 H), 10.55 (s, 1 H), 8.27 (s, 2 H), 8.17 (br s, 1 H), 8.12 (d, *J*=8.2 Hz, 1 H), 7.85 (br s, 1 H), 7.82 (dd, *J*=8.3, 1.3 Hz, 1 H)

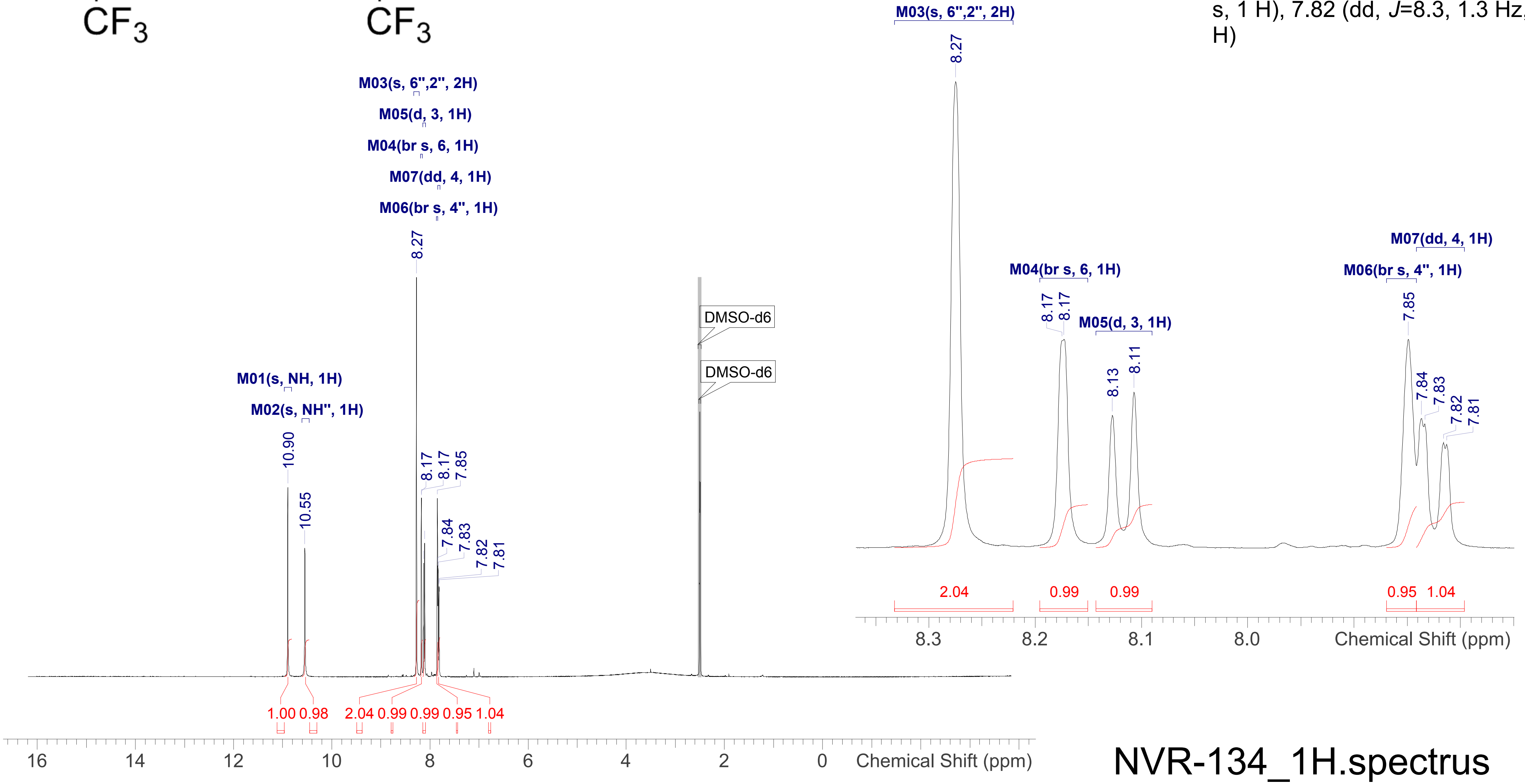

NVR-134_1H.spectrus

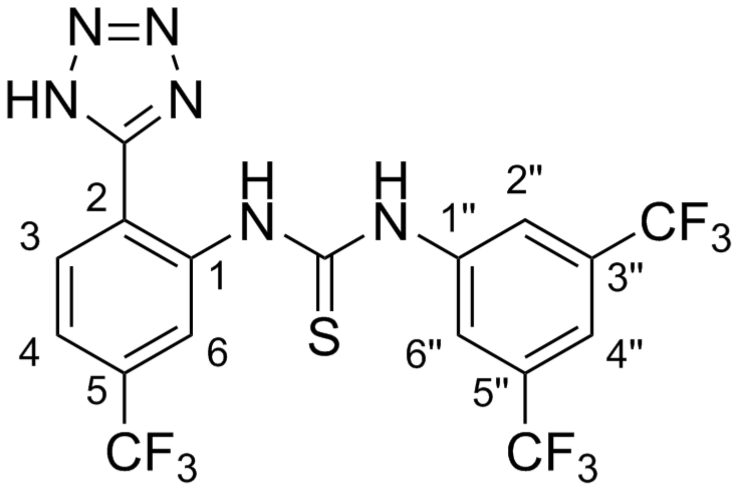

Shift (ppm)	C	m	J (Hz)	Assign
180.6	1	s	-	CS
141.2	1	s	-	1"
138.0	1	s	-	1
130.6	1	br s	-	3
130.4	3	br q	33.3	3", 5", 5
124.6	1	br q	3.9	4
123.5	2	br q	3.9	2", 6"
123.2	3	q	273.5	5-CF3, 5"-CF3, 3"-CF3
122.7	1	br q	3.9	6
117.5	1	br spt	3.9	4"

Acquisition Time (sec)	1.0224
Date	20 Jun 2019 23:58:36
Date Stamp	20 Jun 2019 23:58:36
Frequency (MHz)	100.6128
Nucleus	13C
Number of Transients	256
Solvent	DMSO-d6
Temperature (degree C)	22.700

^{13}C NMR (101 MHz, *DMSO-d_6*) δ ppm 180.6 (s, 1 C), 141.2 (s, 1 C), 138.0 (s, 1 C), 130.6 (br s, 1 C), 130.4 (br q, *J*=33.3 Hz, 3 C), 124.6 (br q, *J*=3.9 Hz, 1 C), 123.5 (br q, *J*=3.9 Hz, 2 C), 122.7 (br q, *J*=3.9 Hz, 1 C), 123.2 (q, *J*=273.5 Hz, 3 C), 117.5 (br spt, *J*=3.9 Hz, 1 C)

NVR-134_13C

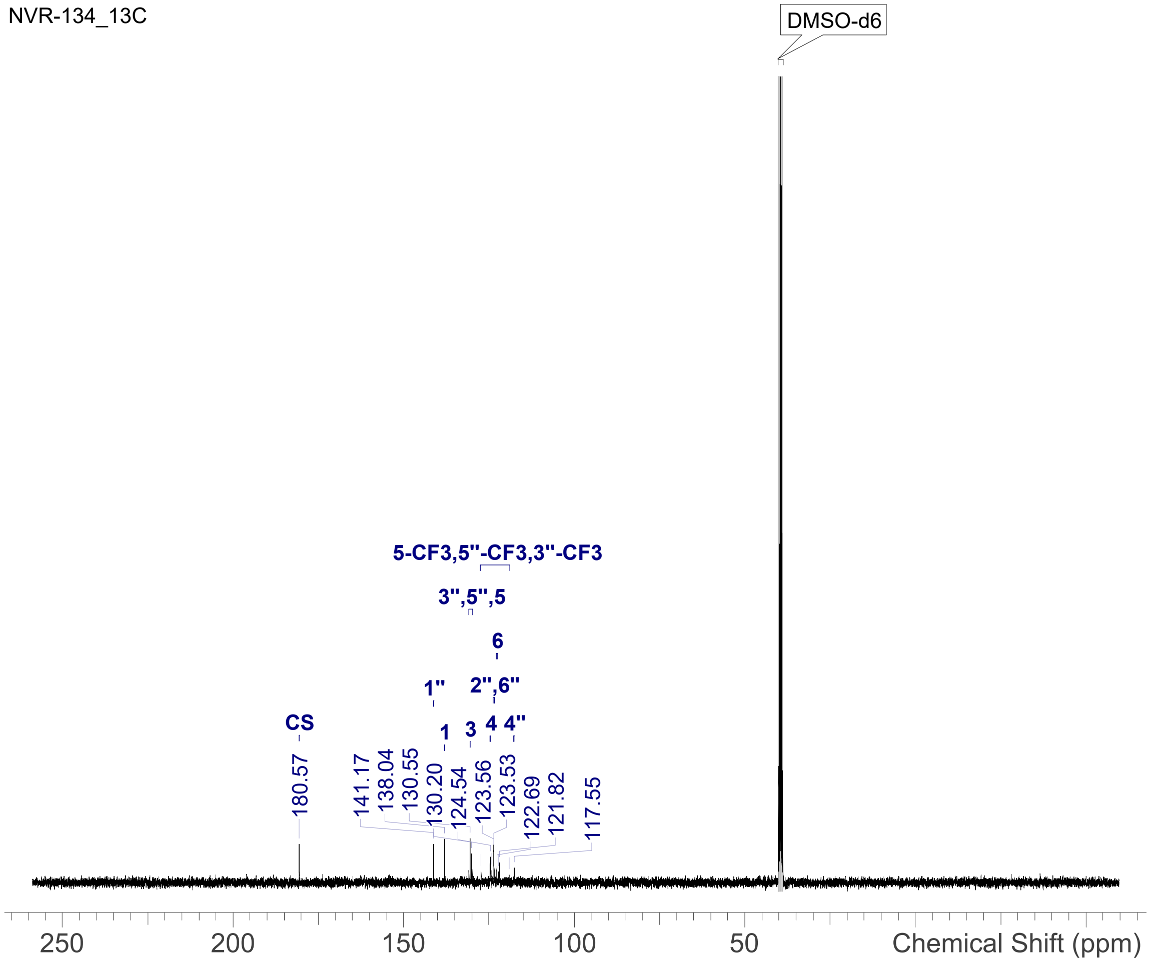

NVR-134_13C

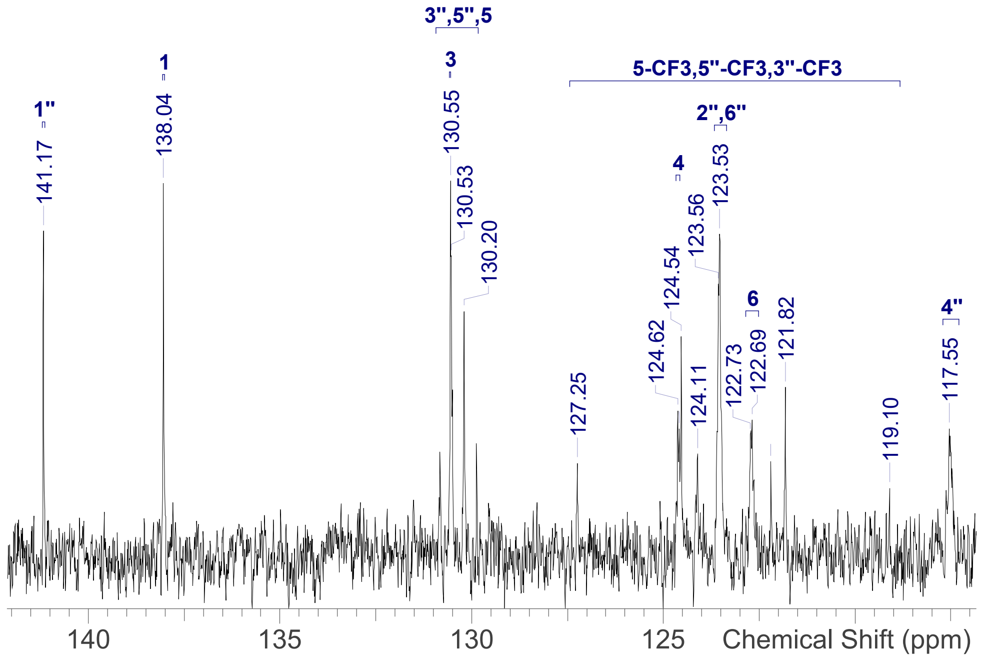

NVR-134_13C.spectrus

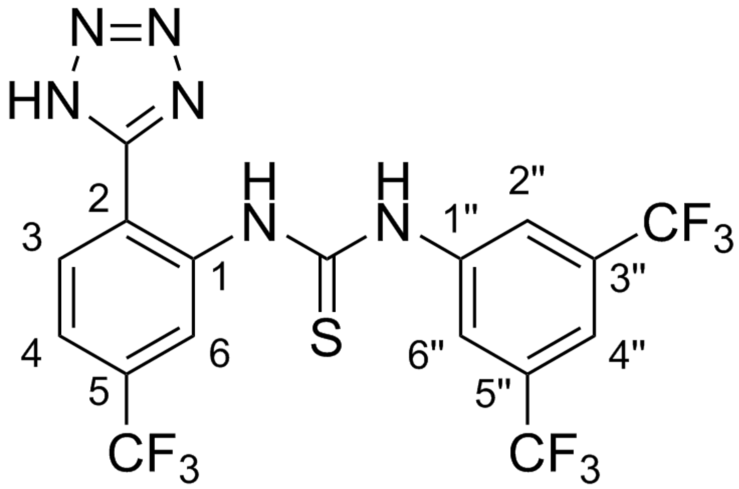

Shift (ppm)	F	m
-61.55	6	s
-61.63	3	s

Acquisition Time (sec)	2.9360
Date	21/06/2019 00:49:00
Date Stamp	21/06/2019 00:49:00
Frequency (MHz)	376.4610
Nucleus	19F
Number of Transients	16
Solvent	DMSO-d6

^{19}F NMR (376 MHz, *DMSO-d$_6$*) δ ppm -61.55 (s, 6 F), -61.63 (s, 3 F)

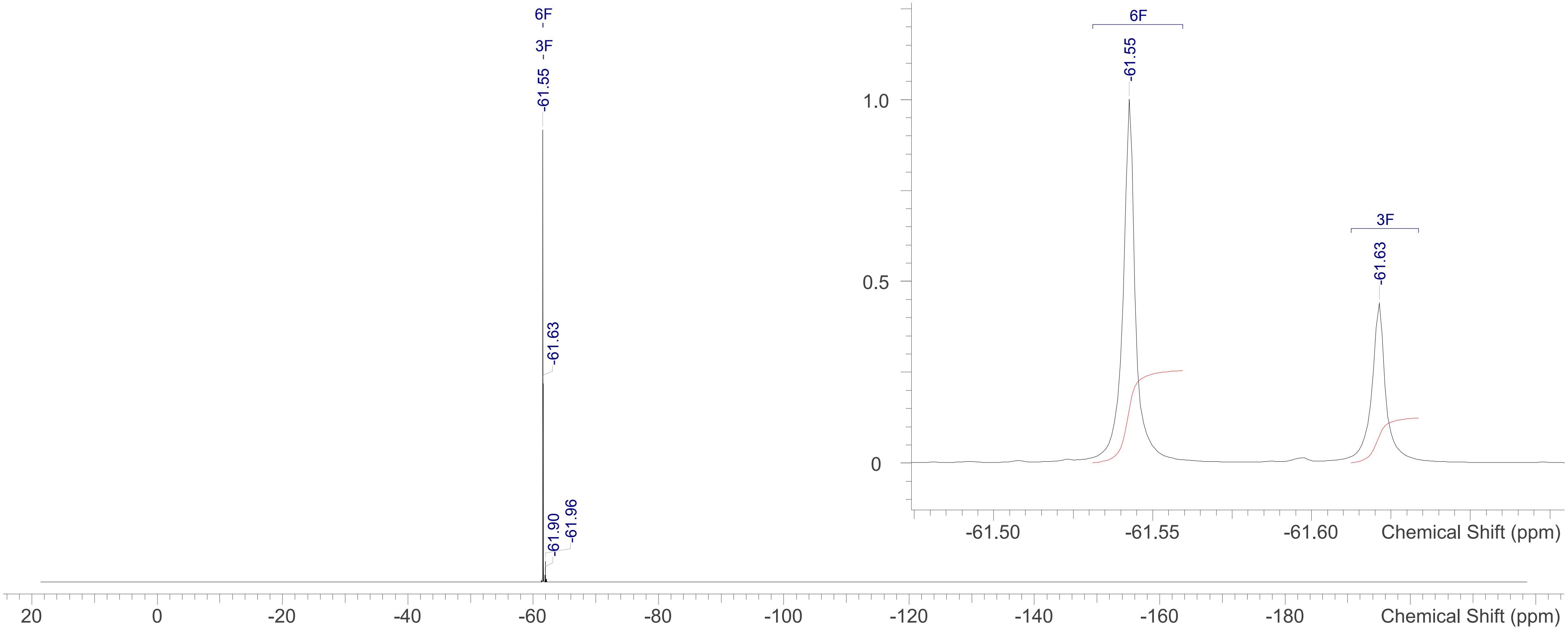

NVR-134_19F.spectrus

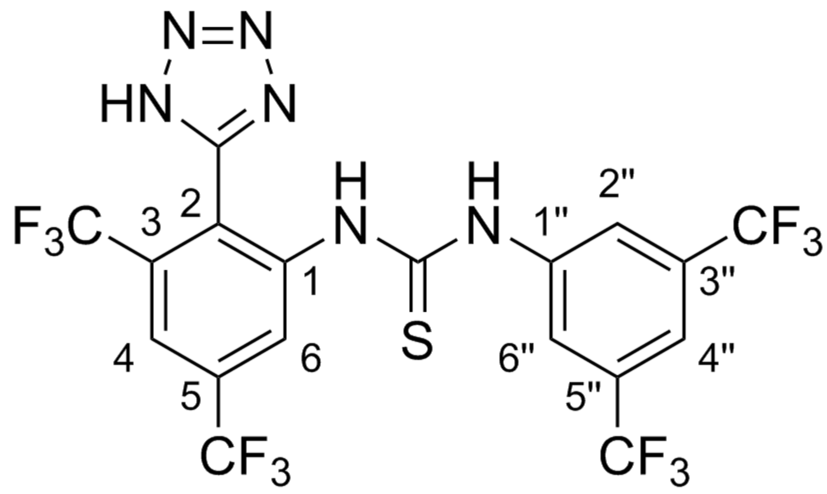

Shift (ppm)	H	m	Assign
10.48	1	br s	NH"
9.97	1	br s	NH
8.41	1	s	6
8.22	1	s	4"
8.10	2	s	2", 6"
7.84	1	s	4

Acquisition Time (sec)	6.5536
Date	10/01/2019 13:56:00
Date Stamp	10/01/2019 13:56:00
Frequency (MHz)	500.1930
Nucleus	1H
Number of Transients	16
Solvent	DMSO-d6

^{1}H NMR (500 MHz, *DMSO-d_6*) δ ppm 10.48 (br s, 1 H), 9.97 (br s, 1 H), 8.41 (s, 1 H), 8.22 (s, 1 H), 8.10 (s, 2 H), 7.84 (s, 1 H)

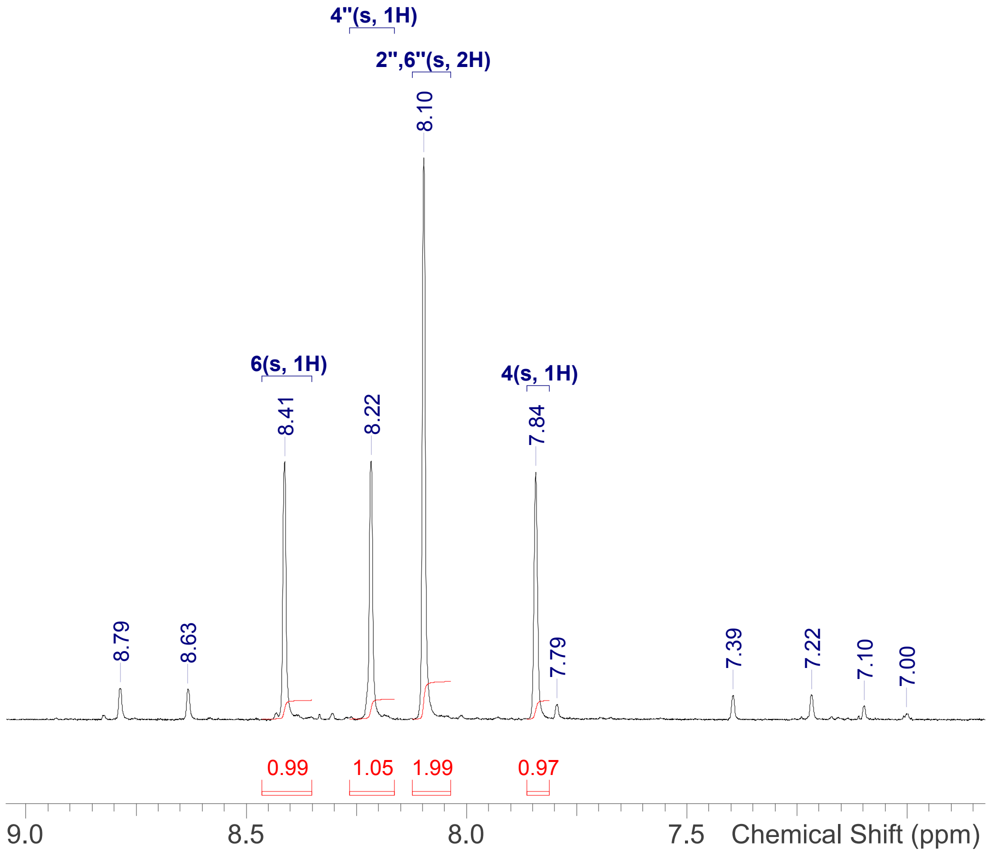

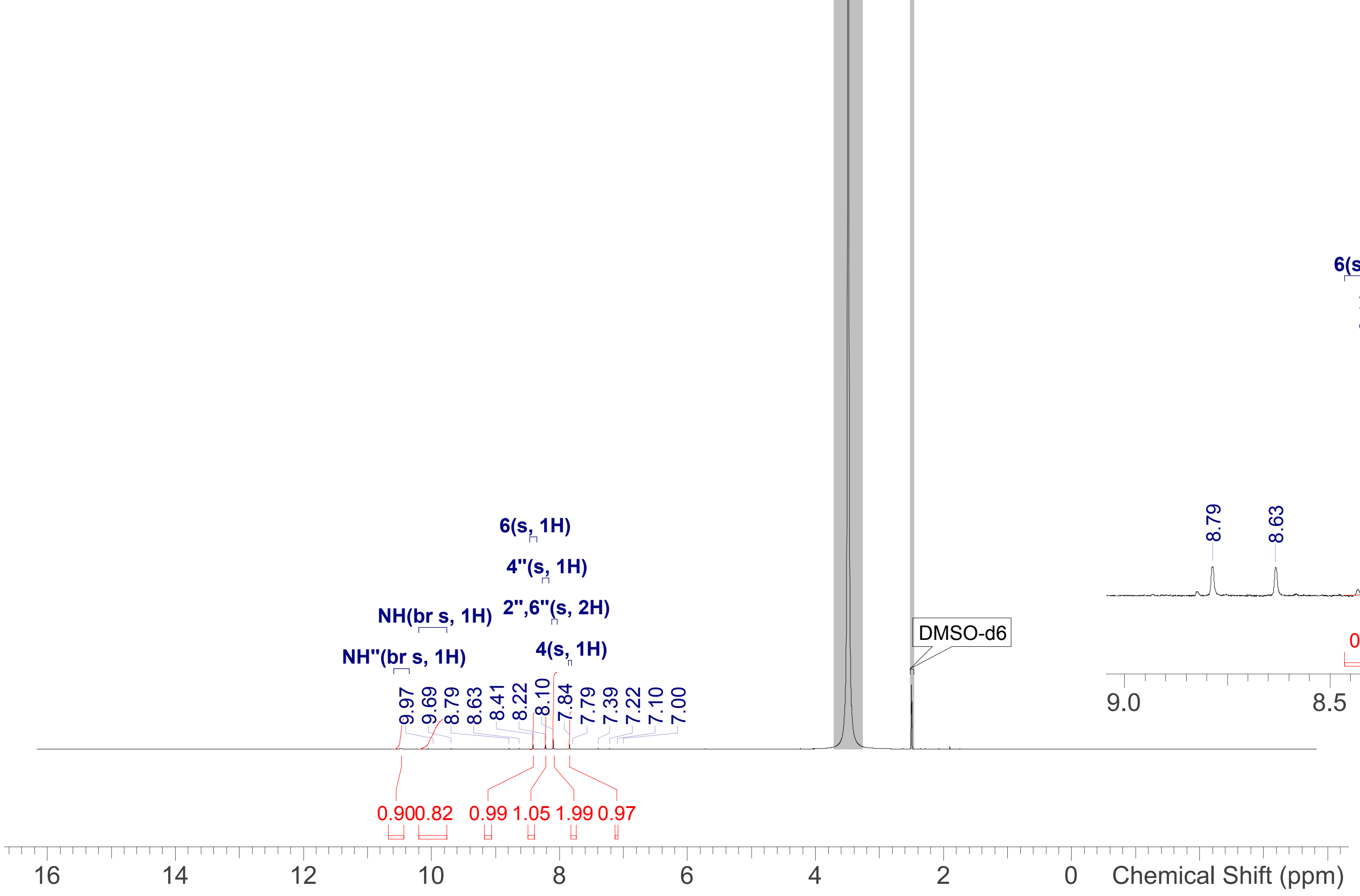

NVR-105_1H.spectrus

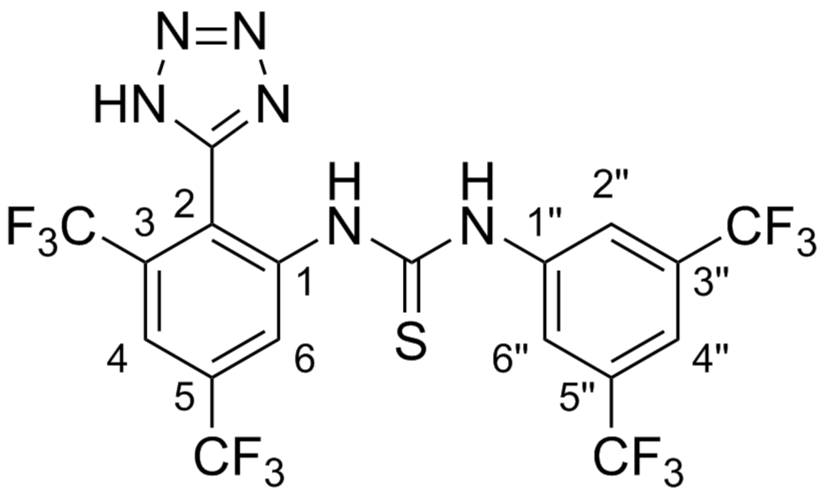

Shift (ppm)	F	m
-57.85	3	s
-61.54	6	s
-61.75	3	s

Acquisition Time (sec)	0.7340
Date	16/07/2019 09:06:00
Date Stamp	16/07/2019 09:06:00
Frequency (MHz)	470.6020
Nucleus	19F
Number of Transients	16
Solvent	DMSO-d6

^{19}F NMR (471 MHz, *DMSO-d_6*) δ ppm -57.85 (s, 3 F), -61.54 (s, 6 F), -61.75 (s, 3 F)

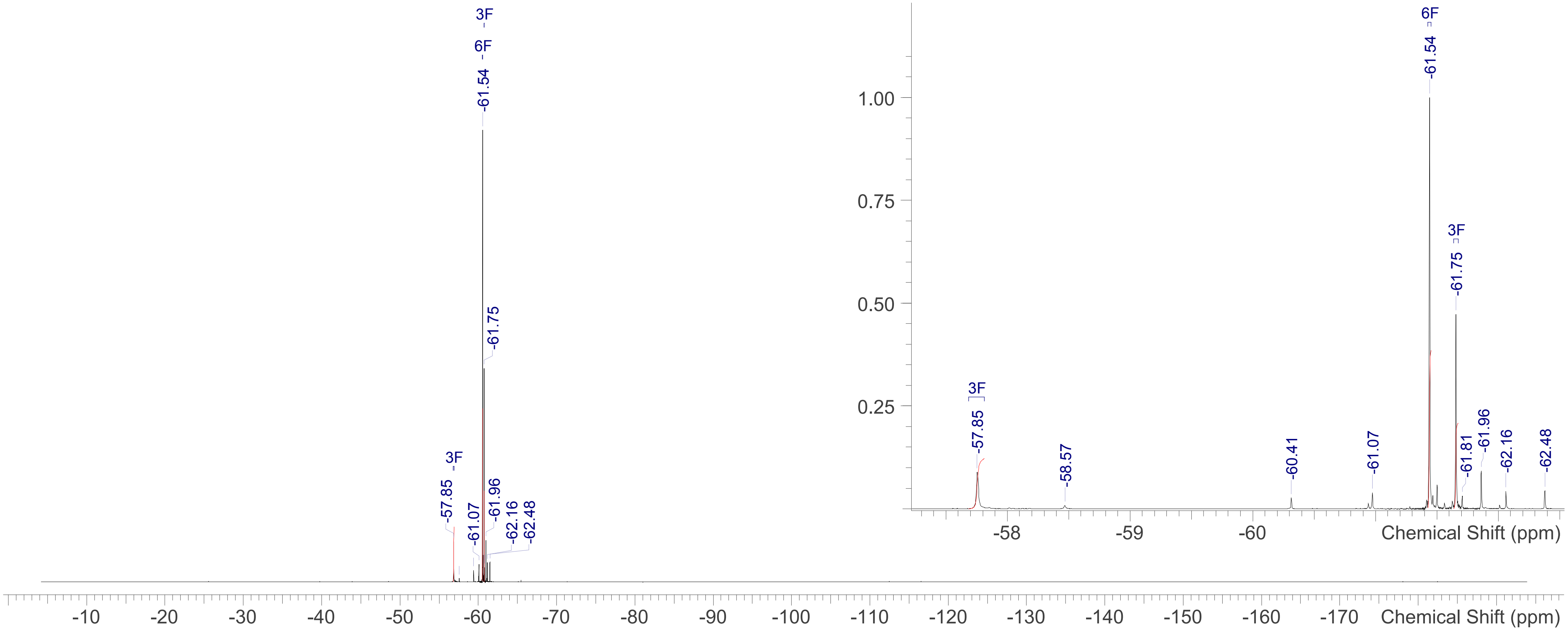

NVR-105_19F.spectrus

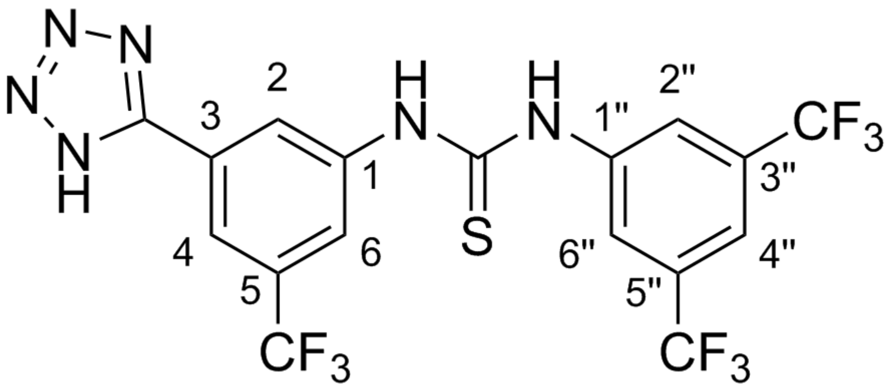

No.	Shift (ppm)	H	m	Assign
1	10.67	1	s	NH
2	10.60	1	s	NH''
3	8.45	1	s	4
4	8.23	2	br s	6'', 2''
5	8.13	2	s	6, 2
6	7.85	1	br s	4''

Acquisition Time (sec)	6.5536
Date	23/07/2019 10:07:00
Date Stamp	23/07/2019 10:07:00
Frequency (MHz)	500.1930
Nucleus	1H
Number of Transients	16
Solvent	DMSO-d6

^{1}H NMR (500 MHz, *DMSO-d_6*) δ ppm 10.67 (s, 1 H), 10.60 (s, 1 H), 8.45 (s, 1 H), 8.23 (br s, 2 H), 8.13 (s, 2 H), 7.85 (br s, 1 H)

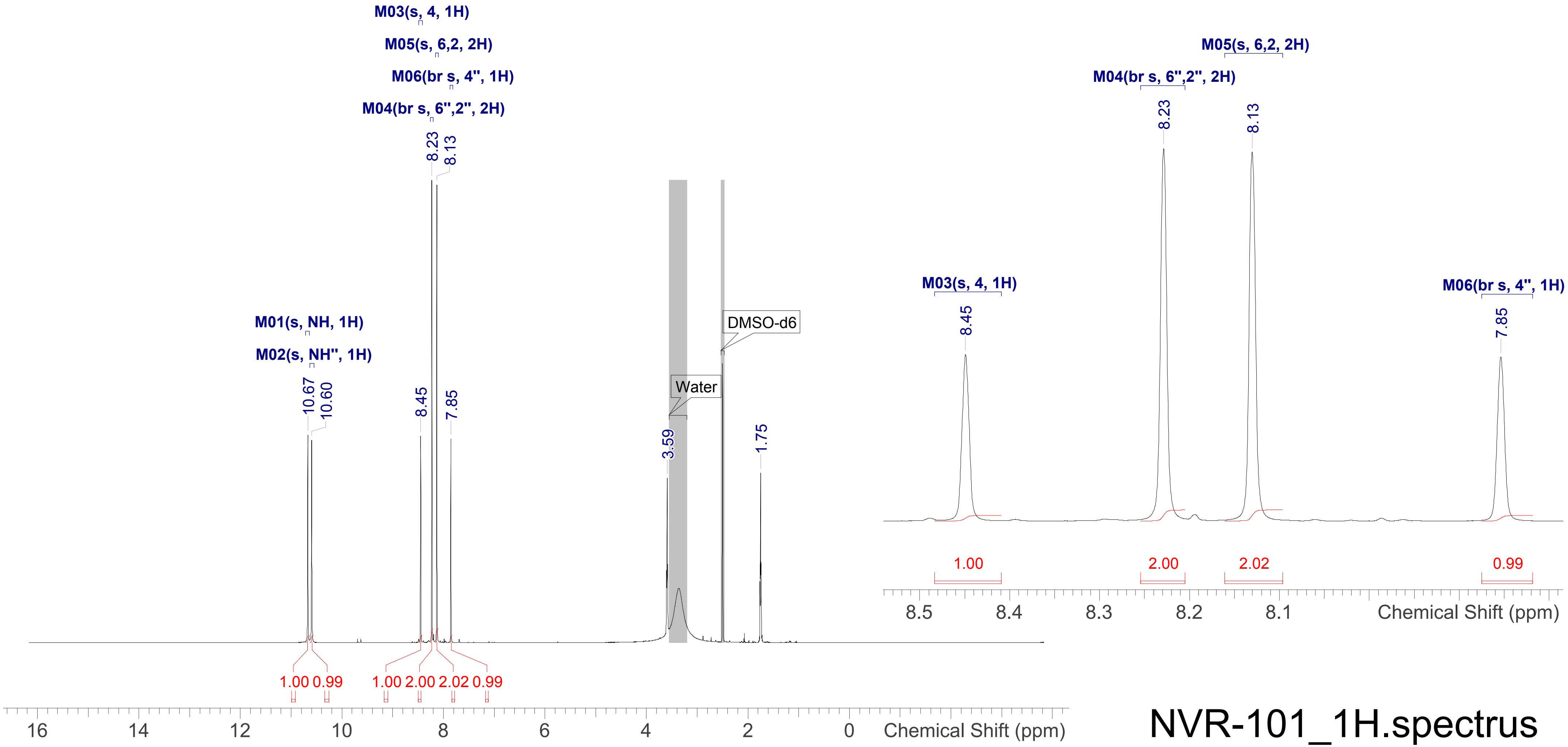

NVR-101_1H.spectrus

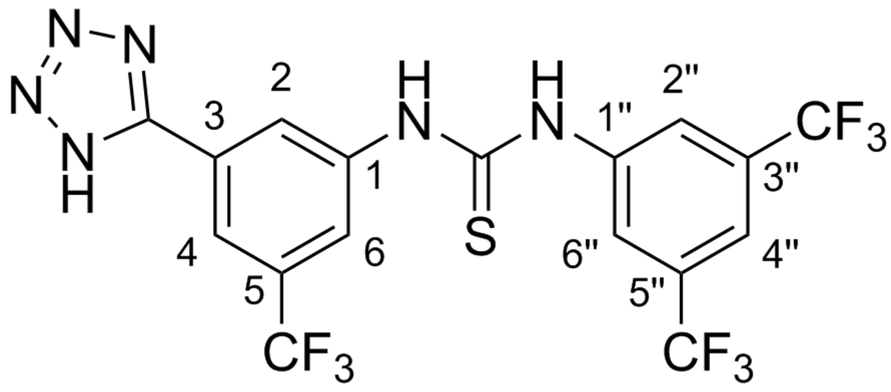

Shift (ppm)	C	m	J (Hz)	Assign
180.5	1	s	-	CS
141.3	1	s	-	1
141.0	1	s	-	1''
130.3	1	q	32.3	5
130.3	2	q	32.6	3'', 5''
125.7	1	s	-	2
124.0	2	br q	3.9	2'', 6''
123.2	3	q	272.9	5-CF3, 5''-CF3, 3''-CF3
122.6	1	br q	3.9	4
119.3	1	br q	3.9	6
117.6	1	br spt	3.9	4''

Acquisition Time (sec)	1.0224
Date	23 Jul 2019 10:13:22
Date Stamp	23 Jul 2019 10:13:22
Frequency (MHz)	125.7729
Nucleus	13C
Number of Transients	32
Solvent	DMSO-d6
Temperature (degree C)	25.000

^{13}C NMR (126 MHz, *DMSO-d_6*) δ ppm 180.5 (s, 1 C), 141.3 (s, 1 C), 141.0 (s, 1 C), 130.3 (q, *J*=32.3 Hz, 1 C), 130.3 (q, *J*=32.6 Hz, 2 C), 125.7 (s, 1 C), 124.0 (br q, *J*=3.9 Hz, 2 C), 122.6 (br q, *J*=3.9 Hz, 1 C), 123.2 (q, *J*=272.9 Hz, 3 C), 119.3 (br q, *J*=3.9 Hz, 1 C), 117.6 (br spt, *J*=3.9 Hz, 1 C)

NVR-101_13C

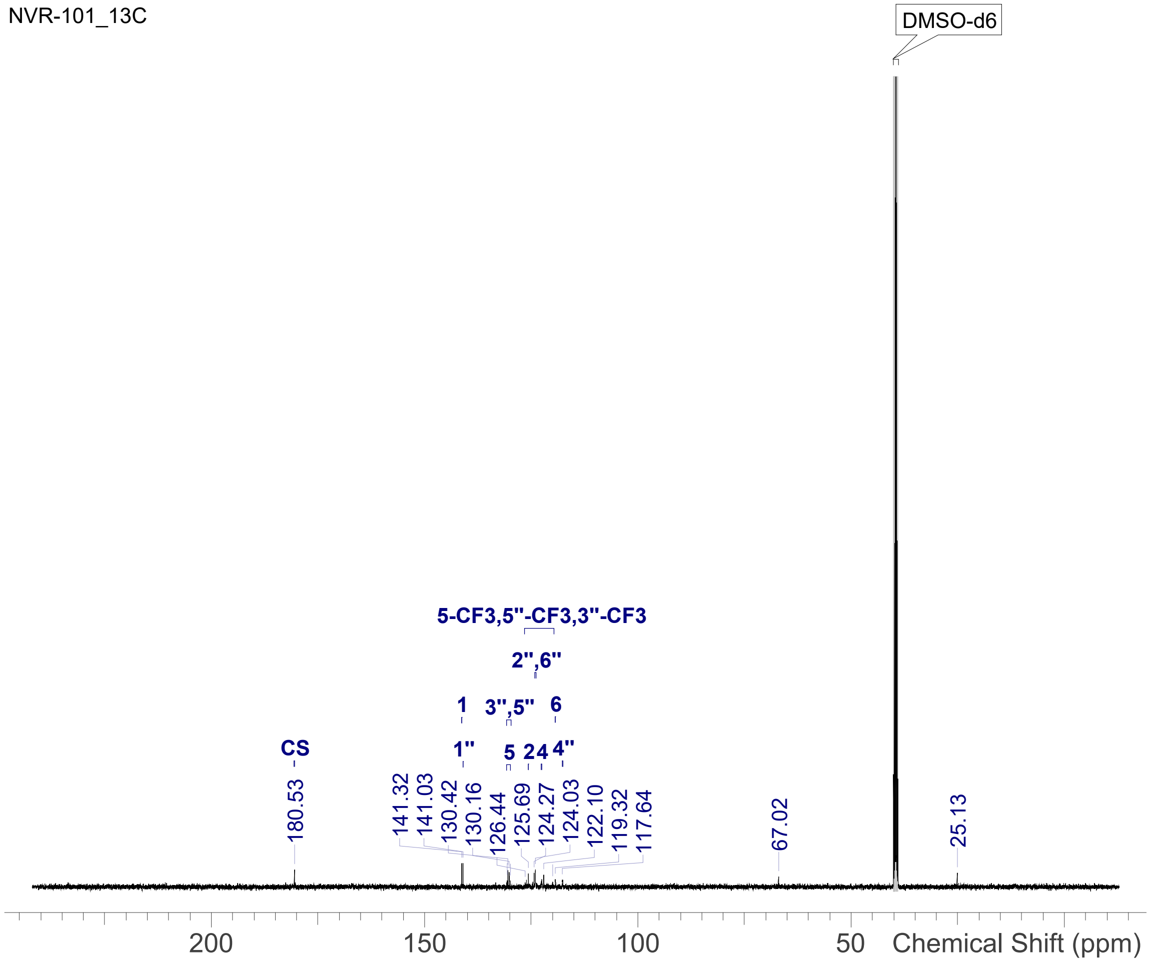

NVR-101_13C

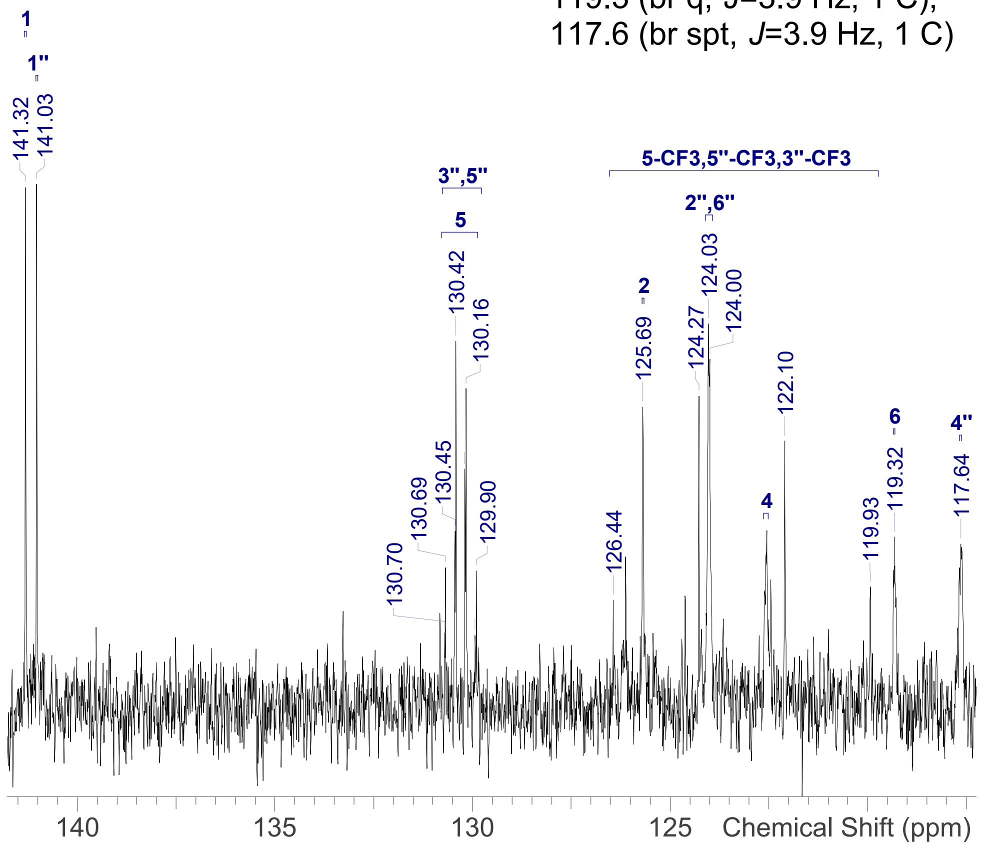

NVR-101_13C.spectrus

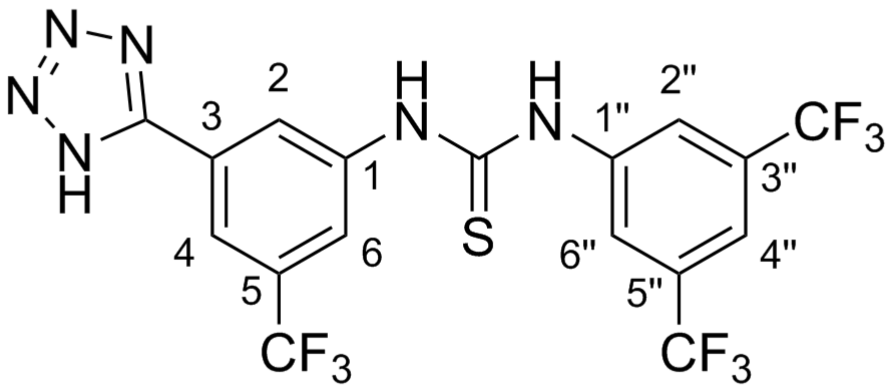

Shift (ppm)	F	m
-61.53	6	s
-61.57	3	s

Acquisition Time (sec)	0.3670
Date	23 Jul 2019 10:22:00
Date Stamp	23 Jul 2019 10:22:00
Frequency (MHz)	470.6488
Nucleus	19F
Number of Transients	16
Solvent	DMSO-d6
Temperature (degree C)	25.000

^{19}F NMR (471 MHz, *DMSO-d_6*) δ ppm -61.53 (s, 6 F), -61.57 (s, 3 F)

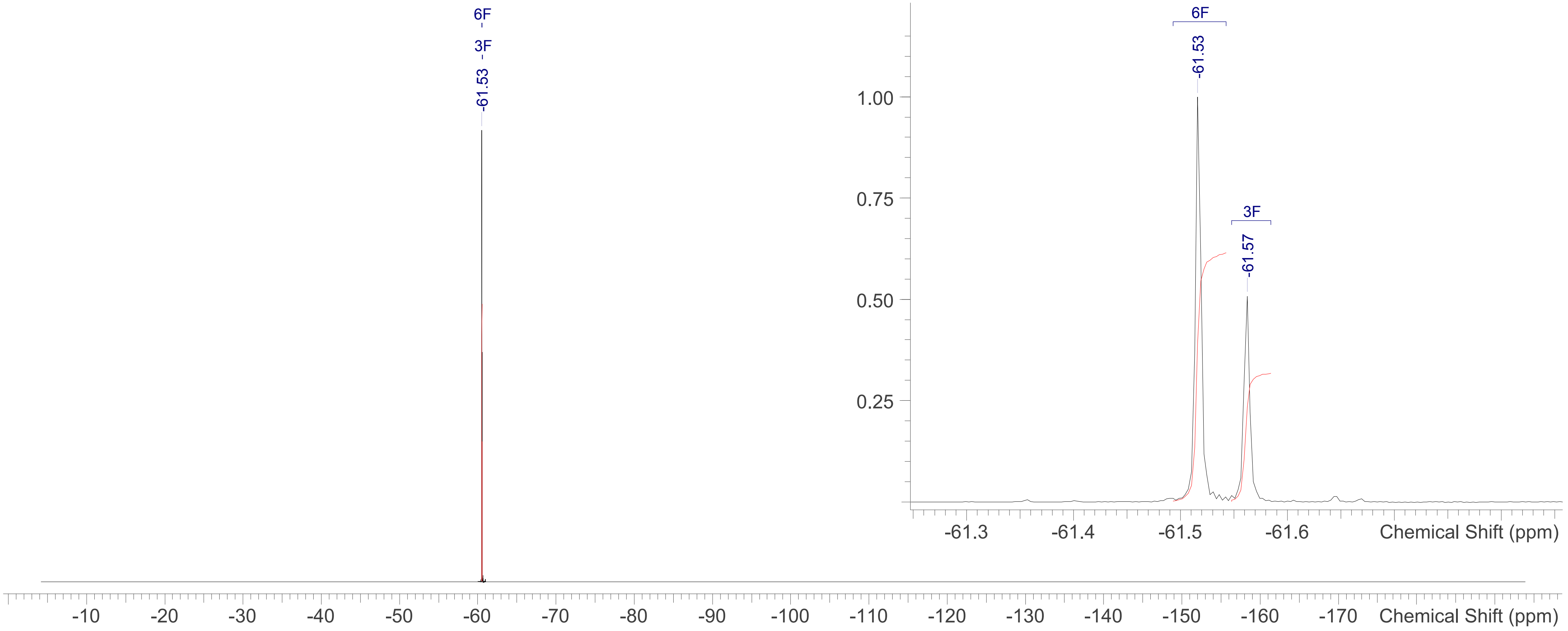

NVR-101_19F.spectrus

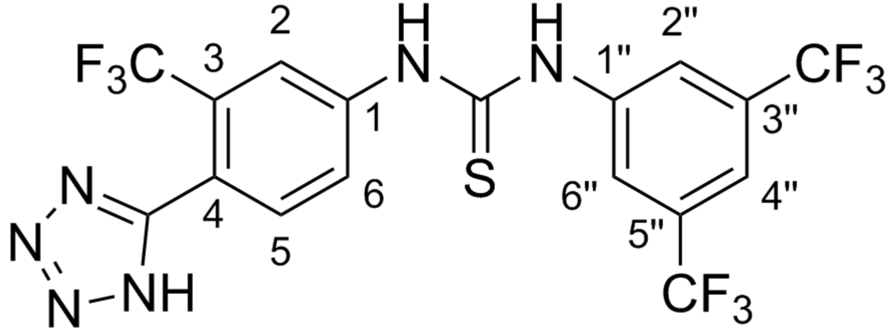

Shift (ppm)	H	m	J (Hz)	Assign
11.06	2	br s	-	NH, NH''
8.31	2	br s	-	2'', 6''
8.26	1	br s	-	2
8.02	1	br d	7.8	5
7.87	1	br s	-	4''
7.80	1	br d	8.8	6

Acquisition Time (sec)	8.1789
Date	11/12/2018 18:06:00
Date Stamp	11/12/2018 18:06:00
Frequency (MHz)	400.1320
Nucleus	1H
Number of Transients	16
Solvent	DMSO-d6

^{1}H NMR (400 MHz, *DMSO-d_6*) δ ppm 11.06 (br s, 2 H), 8.31 (br s, 2 H), 8.26 (br s, 1 H), 8.02 (br d, *J*=7.8 Hz, 1 H), 7.87 (br s, 1 H), 7.80 (br d, *J*=8.8 Hz, 1 H)

NVR-96_1H

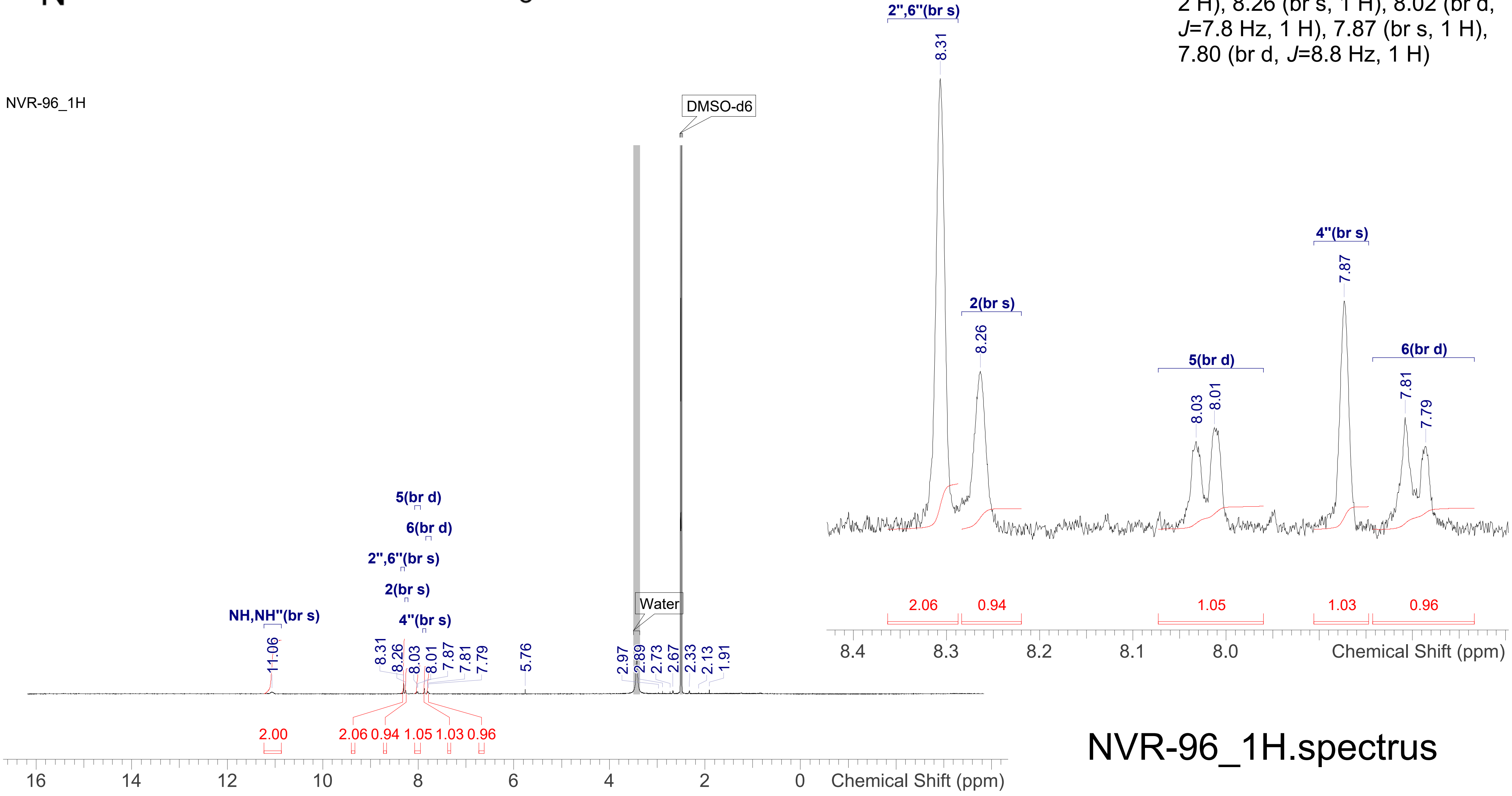

NVR-96_1H.spectrus

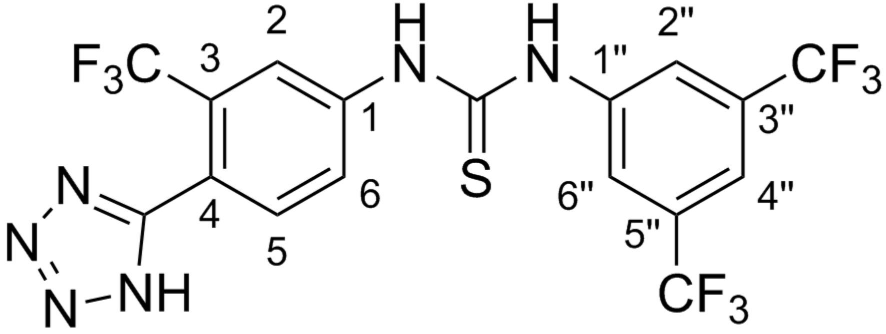

Shift (ppm)	C	m	J (Hz)	Assign
180.5	1	s	-	CS
141.3	1	s	-	1
141.0	1	s	-	1"
130.3	3	q	32.8	3, 3", 5"
125.7	1	s	-	6
124.5	1	s	-	5
124.0	1	br q	3.9	2
119.3	2	q	3.9	2", 6"
117.6	1	br spt	3.9	4"

Acquisition Time (sec)	1.0224
Date	05 Jul 2019 12:38:43
Date Stamp	05 Jul 2019 12:38:43
Frequency (MHz)	100.5977
Nucleus	13C
Number of Transients	16
Solvent	DMSO-d6
Temperature (degree C)	25.002

^{13}C NMR (101 MHz, *DMSO-d$_6$*) δ ppm 180.5 (s, 1 C), 141.3 (s, 1 C), 141.0 (s, 1 C), 130.3 (q, *J*=32.8 Hz, 3 C), 125.7 (s, 1 C), 124.5 (s, 1 C), 124.0 (br q, *J*=3.9 Hz, 1 C), 119.3 (q, *J*=3.9 Hz, 2 C), 117.6 (br spt, *J*=3.9 Hz, 1 C)

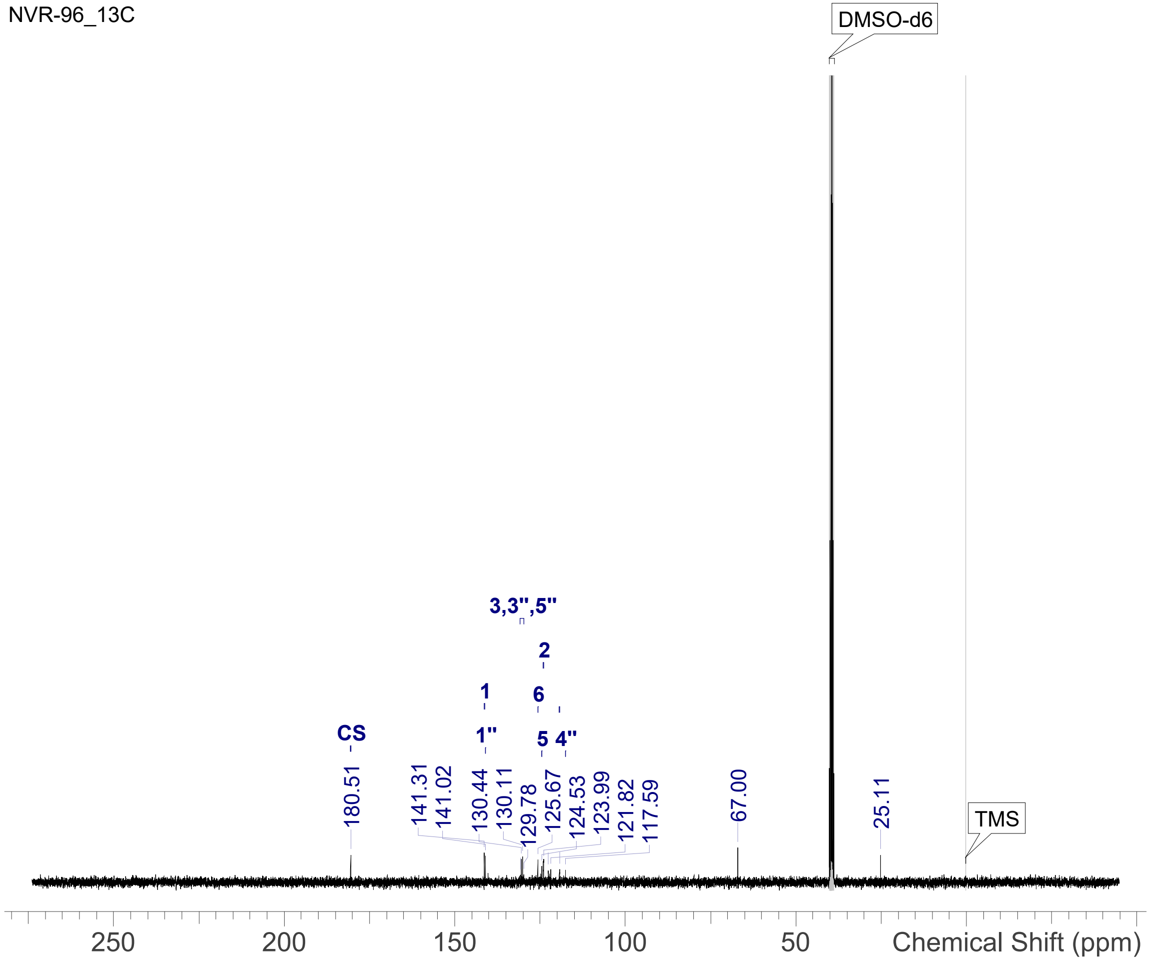

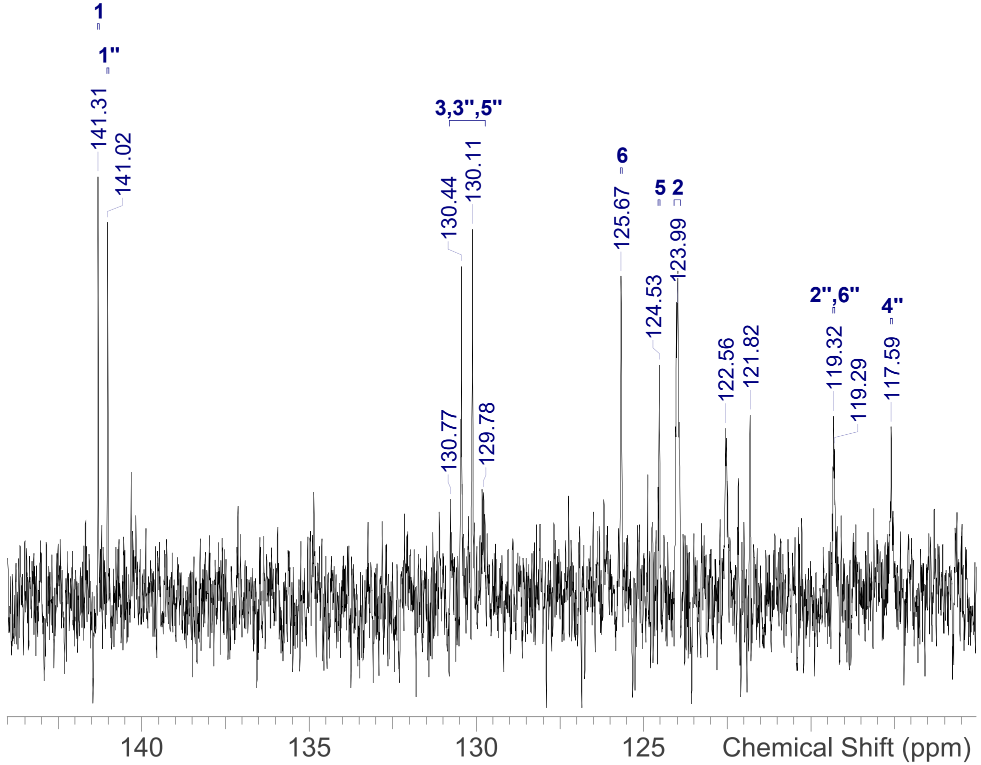

NVR-96_13C.spectrus

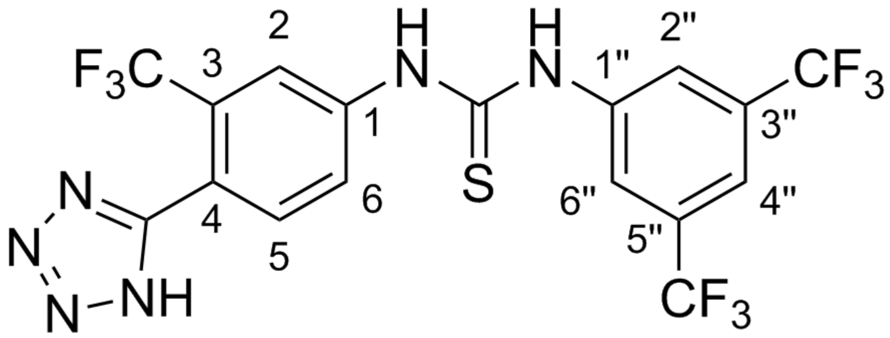

Shift (ppm)	F	m
-61.53	6	s
-61.57	3	s

Acquisition Time (sec)	1.4680
Date	05 Jul 2019 12:45:06
Date Stamp	05 Jul 2019 12:45:06
Frequency (MHz)	376.4419
Nucleus	19F
Number of Transients	16
Solvent	DMSO-d6
Temperature (degree C)	25.001

^{19}F NMR (376 MHz, *DMSO-d_6*) δ ppm -61.53 (s, 6 F), -61.57 (s, 3 F)

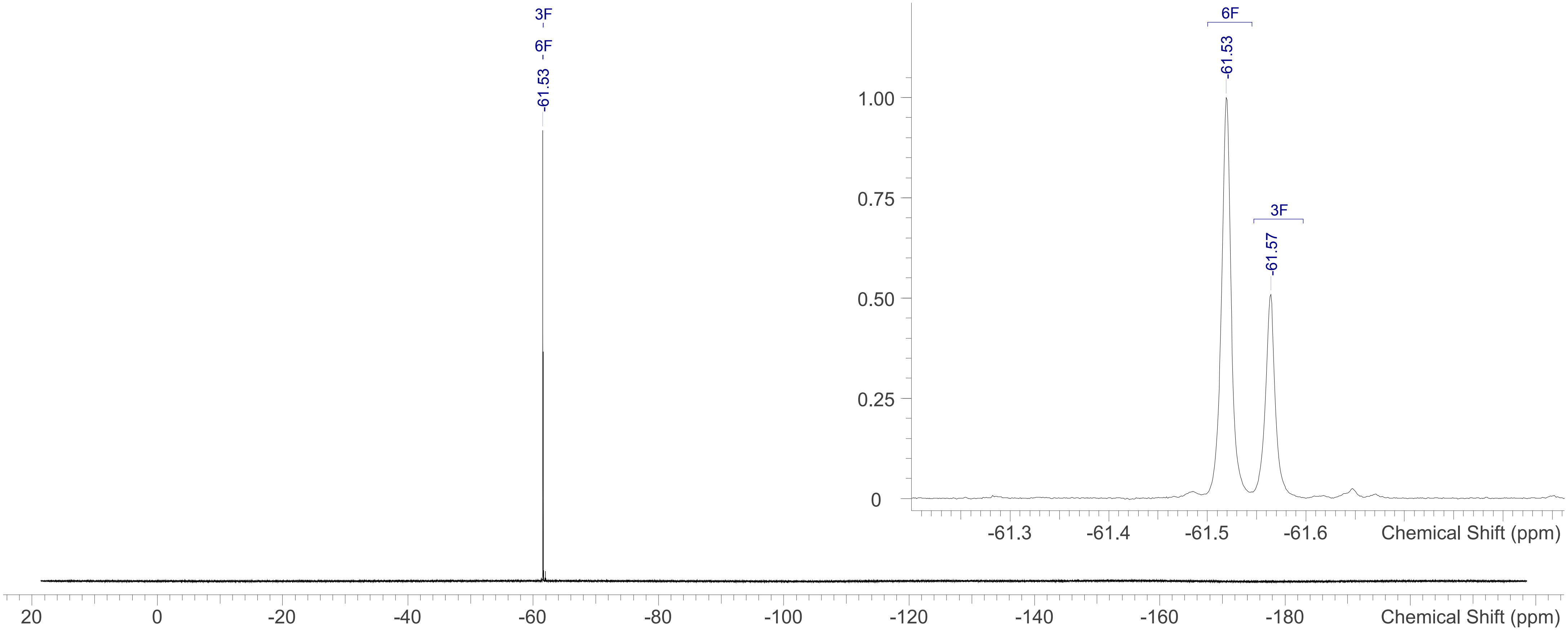

NVR-96_19F.spectrus

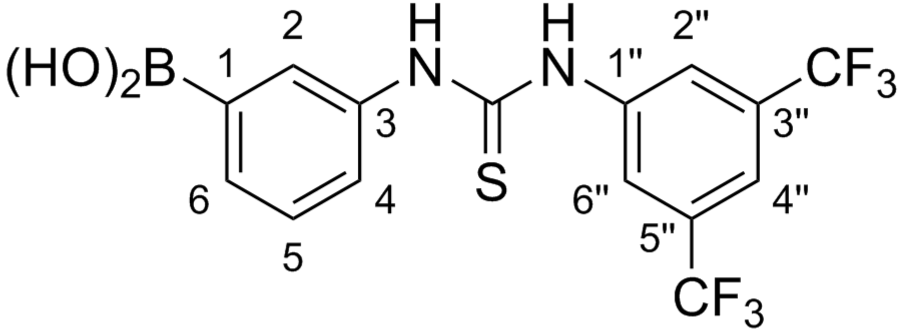

Shift (ppm)	H	m	J (Hz)	Assign
10.27	1	s	-	NH"
10.14	1	s	-	NH
8.24	2	s	-	B(OH), B(OH)
8.11	2	s	-	6", 2"
7.79	1	s	-	2
7.73	1	s	-	4"
7.63	1	d	7.3	6
7.49	1	br d	8.8	4
7.35	1	t	7.9	5

Acquisition Time (sec)	8.1789
Date	20/06/2019 17:25:00
Date Stamp	20/06/2019 17:25:00
Frequency (MHz)	400.1320
Nucleus	1H
Number of Transients	16
Solvent	DMSO-d6

^{1}H NMR (400 MHz, *DMSO-d_6*) δ ppm 10.27 (s, 1 H), 10.14 (s, 1 H), 8.24 (s, 2 H), 8.11 (s, 2 H), 7.79 (s, 1 H), 7.73 (s, 1 H), 7.63 (d, *J*=7.3 Hz, 1 H), 7.49 (br d, *J*=8.8 Hz, 1 H), 7.35 (t, *J*=7.9 Hz, 1 H)

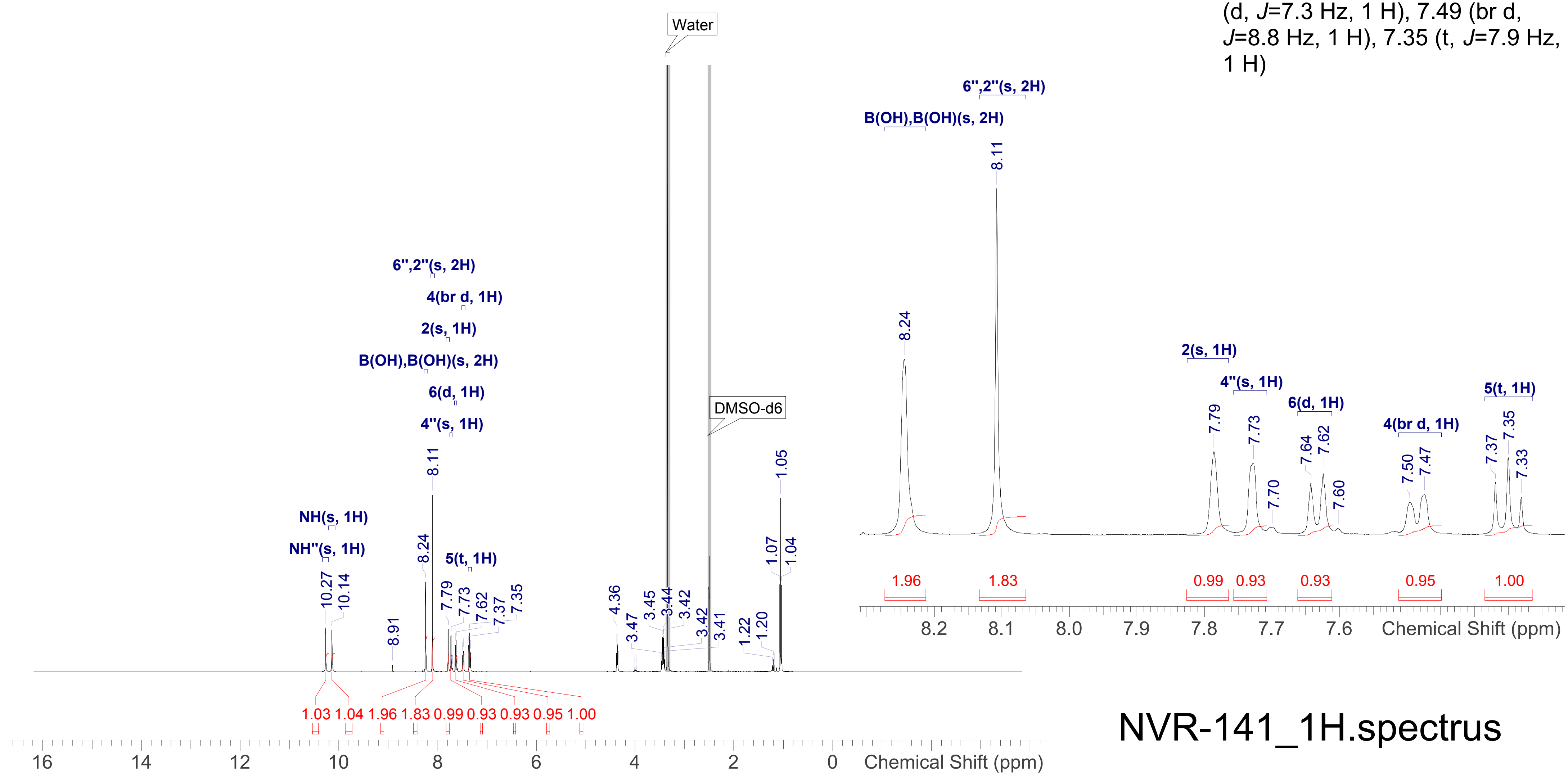

NVR-141_1H.spectrus

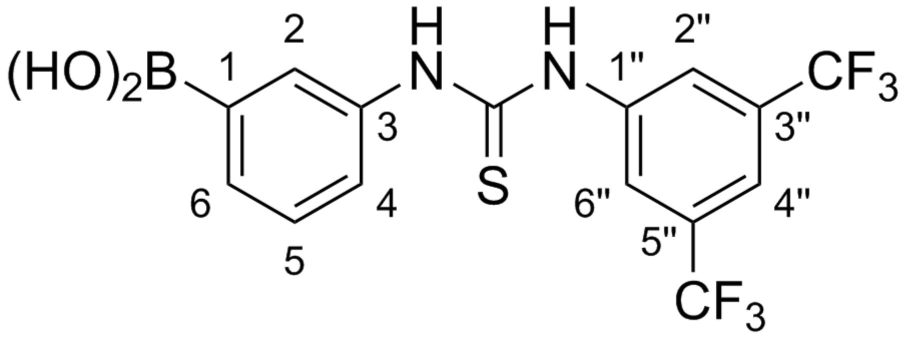

Shift (ppm)	C	m	J (Hz)	Assign
179.9	1	s	-	CS
141.9	1	s	-	1''
137.8	1	s	-	3
131.3	1	s	-	6
130.1	1	s	-	5
129.9	2	q	32.3	3'', 5''
127.9	1	s	-	2
126.4	1	s	-	4
123.6	2	br q	3.9	2'', 6''
123.3	2	q	272.9	5''-CF3, 3''-CF3
116.7	1	br spt	3.9	4''

Acquisition Time (sec)	1.0224
Date	21 Jun 2019 01:05:42
Date Stamp	21 Jun 2019 01:05:42
Frequency (MHz)	100.6128
Nucleus	13C
Number of Transients	256
Solvent	DMSO-d6
Temperature (degree C)	22.700

^{13}C NMR (101 MHz, *DMSO-d$_6$*) δ ppm 179.9 (s, 1 C), 141.9 (s, 1 C), 137.8 (s, 1 C), 131.3 (s, 1 C), 130.1 (s, 1 C), 129.9 (q, *J*=32.3 Hz, 2 C), 127.9 (s, 1 C), 126.4 (s, 1 C), 123.6 (br q, *J*=3.9 Hz, 2 C), 123.3 (q, *J*=272.9 Hz, 2 C), 116.7 (br spt, *J*=3.9 Hz, 1 C)

NVR-141_13C

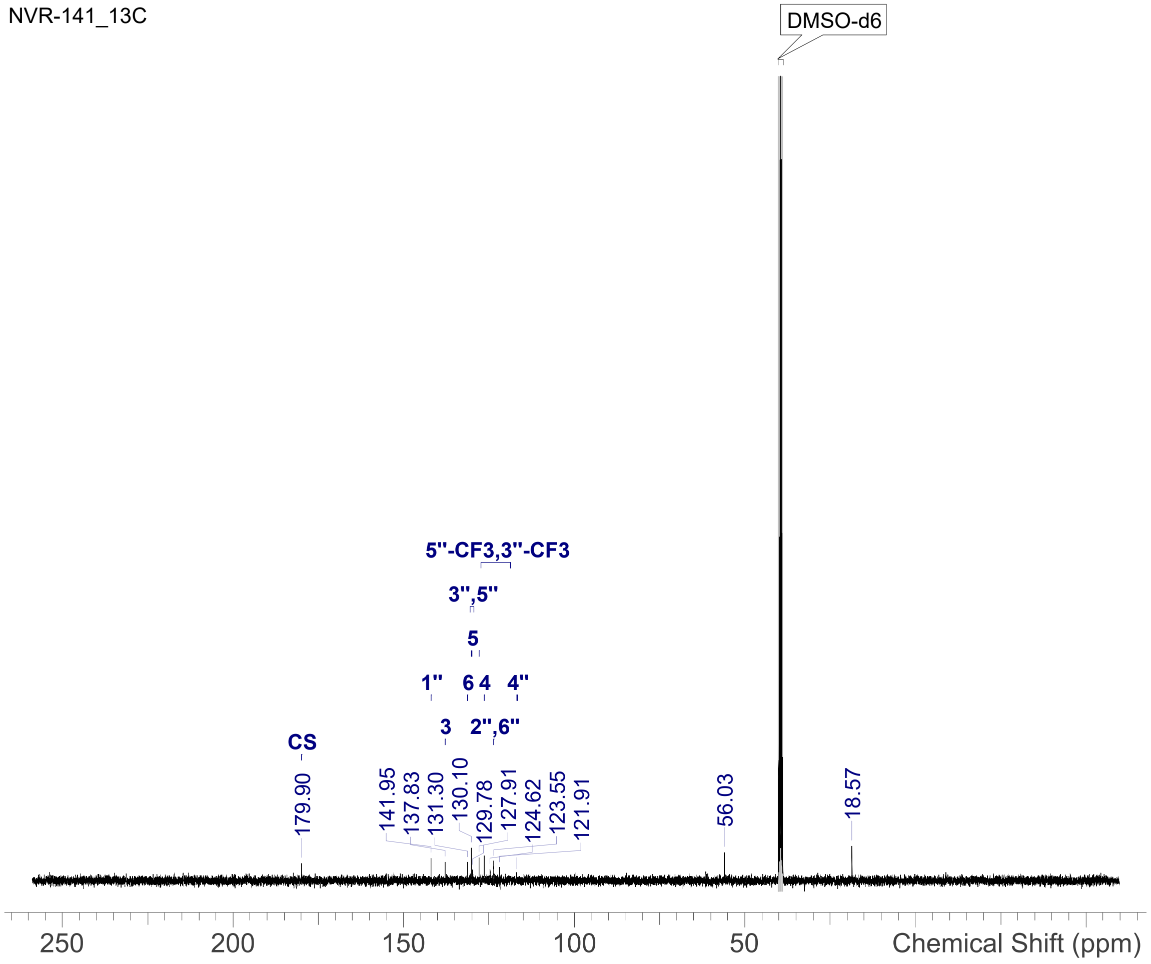

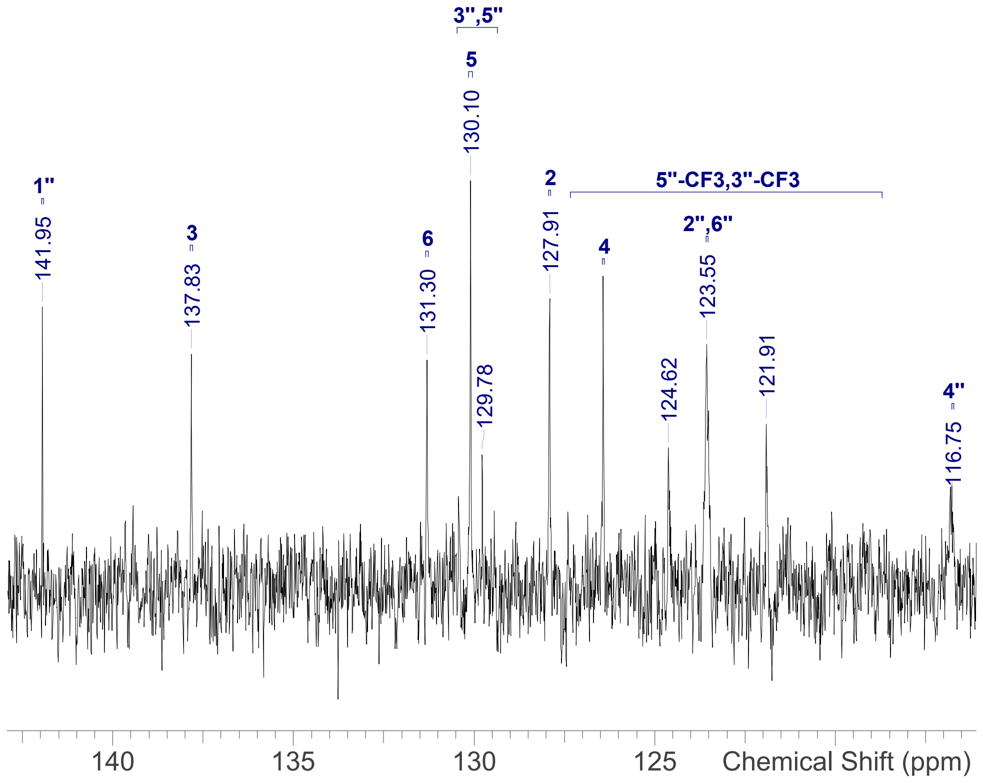

NVR-141_13C.spectrus

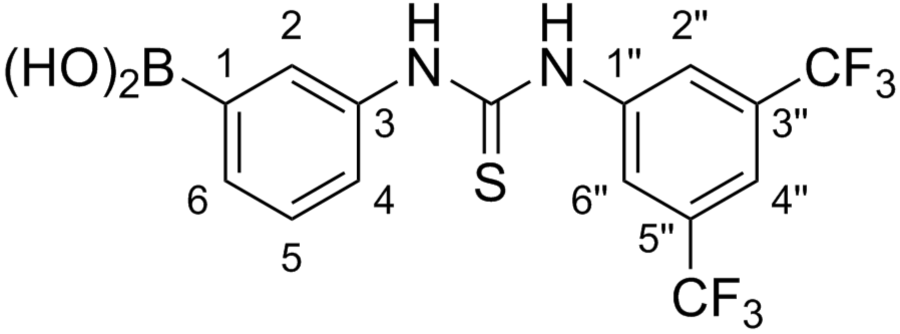

Shift (ppm)	F	m
-61.52	6	s

Acquisition Time (sec)	2.9360
Date	21/06/2019 01:16:00
Date Stamp	21/06/2019 01:16:00
Frequency (MHz)	376.4610
Nucleus	19F
Number of Transients	16
Solvent	DMSO-d6

^{19}F NMR (376 MHz, *DMSO-d_6*) δ ppm -61.52 (s, 6 F)

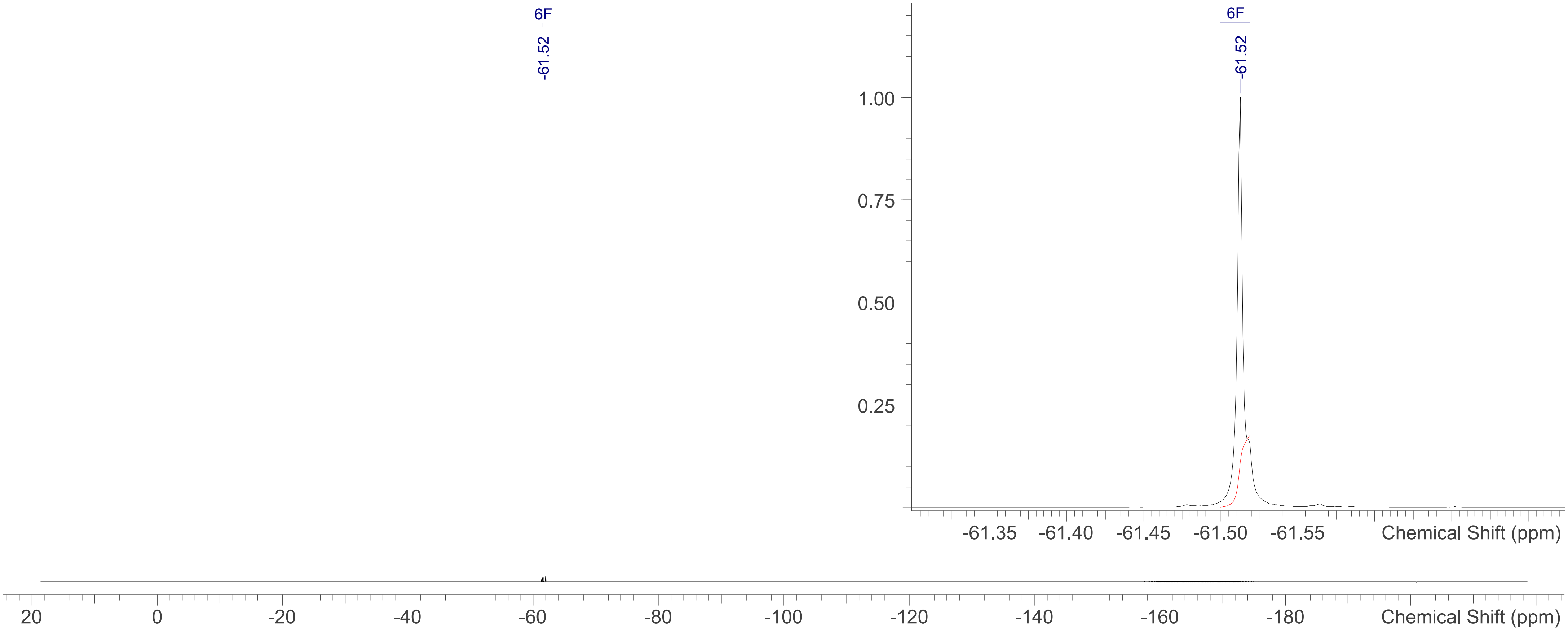

NVR-141_19F.spectrus

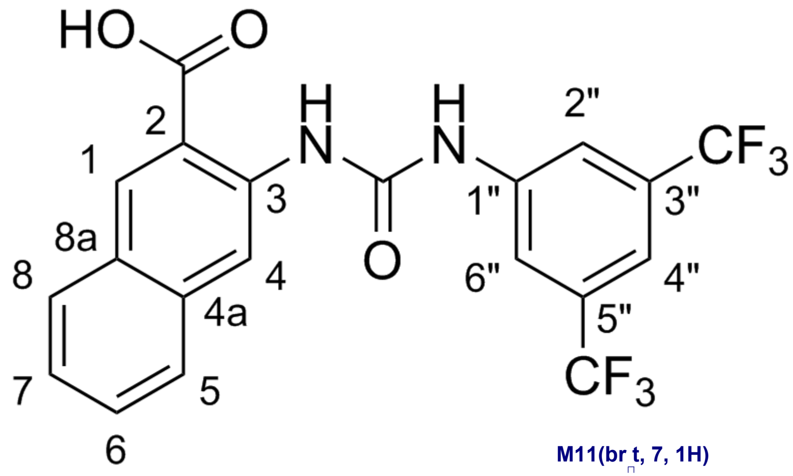

No.	Shift (ppm)	H	m	J (Hz)	Assign
1	13.80	1	br s	-	COOH
2	10.63	1	s	-	NH
3	10.55	1	s	-	NH"
4	8.85	1	s	-	4
5	8.70	1	s	-	1
6	8.25	2	br s	-	2", 6"
7	8.00	1	br d	8.0	8
8	7.89	1	br d	8.0	5
9	7.65	1	br s	-	4"
10	7.60	1	br dd	8.0, 7.5	6
11	7.45	1	br t	7.5	7

Acquisition Time (sec)	3.9846
Date	15 May 2019 16:48:13
Date Stamp	15 May 2019 16:48:13
Frequency (MHz)	400.0700
Nucleus	1H
Number of Transients	4
Solvent	DMSO-d6
Temperature (degree C)	25.000

^{1}H NMR (400 MHz, *DMSO-d_6*) δ ppm 13.80 (br s, 1 H), 10.63 (s, 1 H), 10.55 (s, 1 H), 8.85 (s, 1 H), 8.70 (s, 1 H), 8.25 (br s, 2 H), 8.00 (br d, *J*=8.0 Hz, 1 H), 7.89 (br d, *J*=8.0 Hz, 1 H), 7.65 (br s, 1 H), 7.60 (br dd, *J*=8.0, 7.5 Hz, 1 H), 7.45 (br t, *J*=7.5 Hz, 1 H)

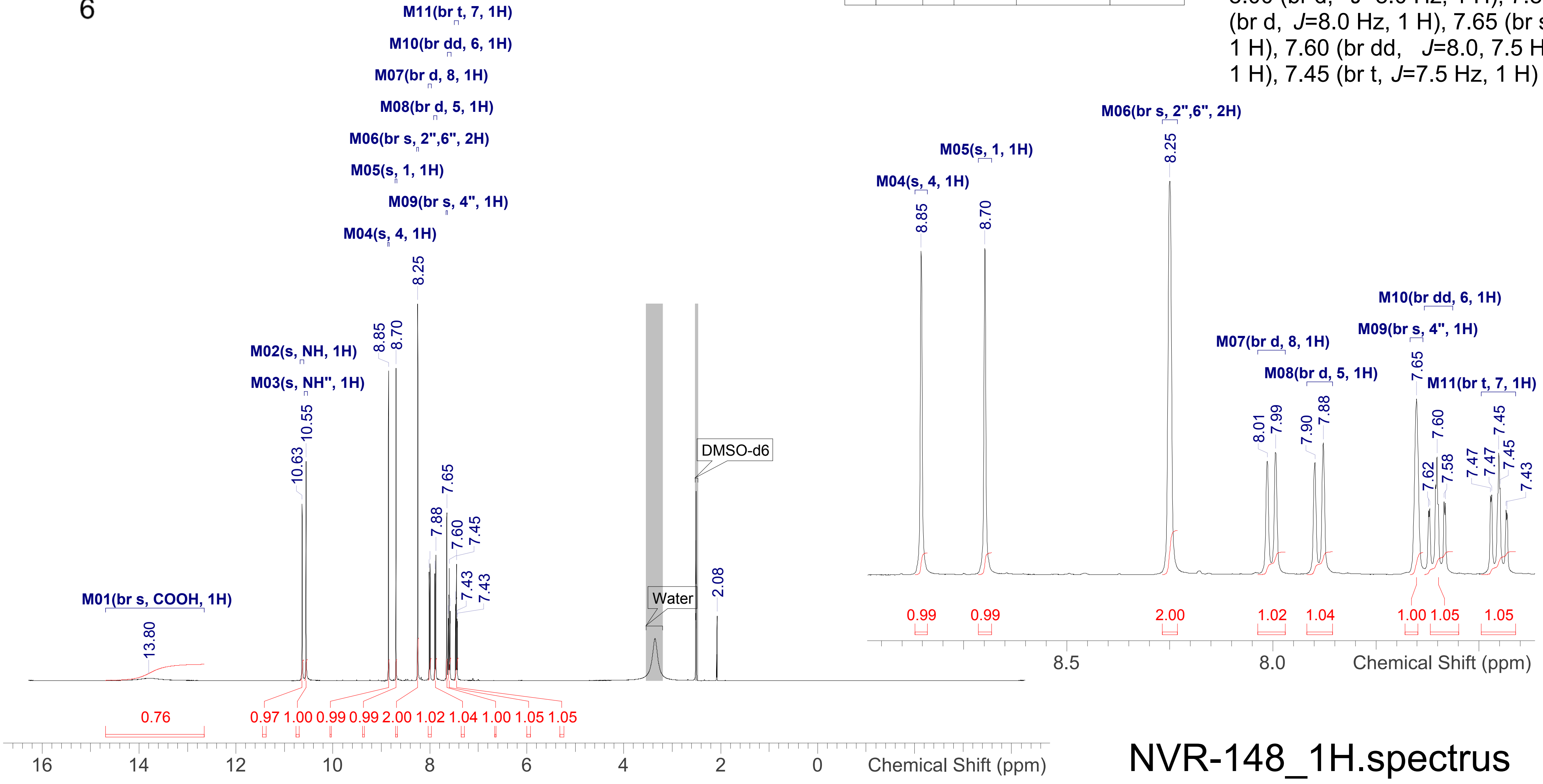

NVR-148_1H.spectrus

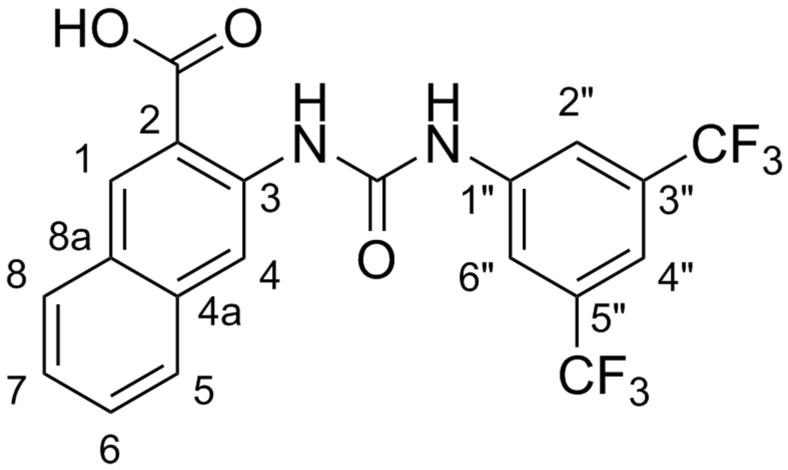

Shift (ppm)	C	m	J (Hz)	Assign
169.6	1	s	-	COOH
152.2	1	s	-	CO
142.1	1	s	-	1"
136.6	1	s	-	3
135.7	1	s	-	4a
133.0	1	s	-	1
130.7	2	q	32.3	3", 5"
129.2	1	s	-	6
129.0	1	s	-	8
127.6	1	s	-	8a
127.0	1	s	-	5
125.2	1	s	-	7
123.4	2	q	272.2	3"-CF3, 5"-CF3
117.9	2	br q	3.9	6", 2"
116.9	1	s	-	2
116.0	1	s	-	4
114.4	1	br spt	3.9	4"

Acquisition Time (sec)	1.0224
Date	15 May 2019 22:35:43
Date Stamp	15 May 2019 22:35:43
Frequency (MHz)	100.5977
Nucleus	13C
Number of Transients	256
Solvent	DMSO-d6
Temperature (degree C)	25.000

^{13}C NMR (101 MHz, *DMSO-d*$_6$) δ ppm 169.6 (s, 1 C), 152.2 (s, 1 C), 142.1 (s, 1 C), 136.6 (s, 1 C), 135.7 (s, 1 C), 133.0 (s, 1 C), 130.7 (q, *J*=32.3 Hz, 2 C), 129.2 (s, 1 C), 129.0 (s, 1 C), 127.6 (s, 1 C), 127.0 (s, 1 C), 125.2 (s, 1 C), 123.4 (q, *J*=272.2 Hz, 2 C), 117.9 (br q, *J*=3.9 Hz, 2 C), 116.9 (s, 1 C), 116.0 (s, 1 C), 114.4 (br spt, *J*=3.9 Hz, 1 C)

NVR-148_13C

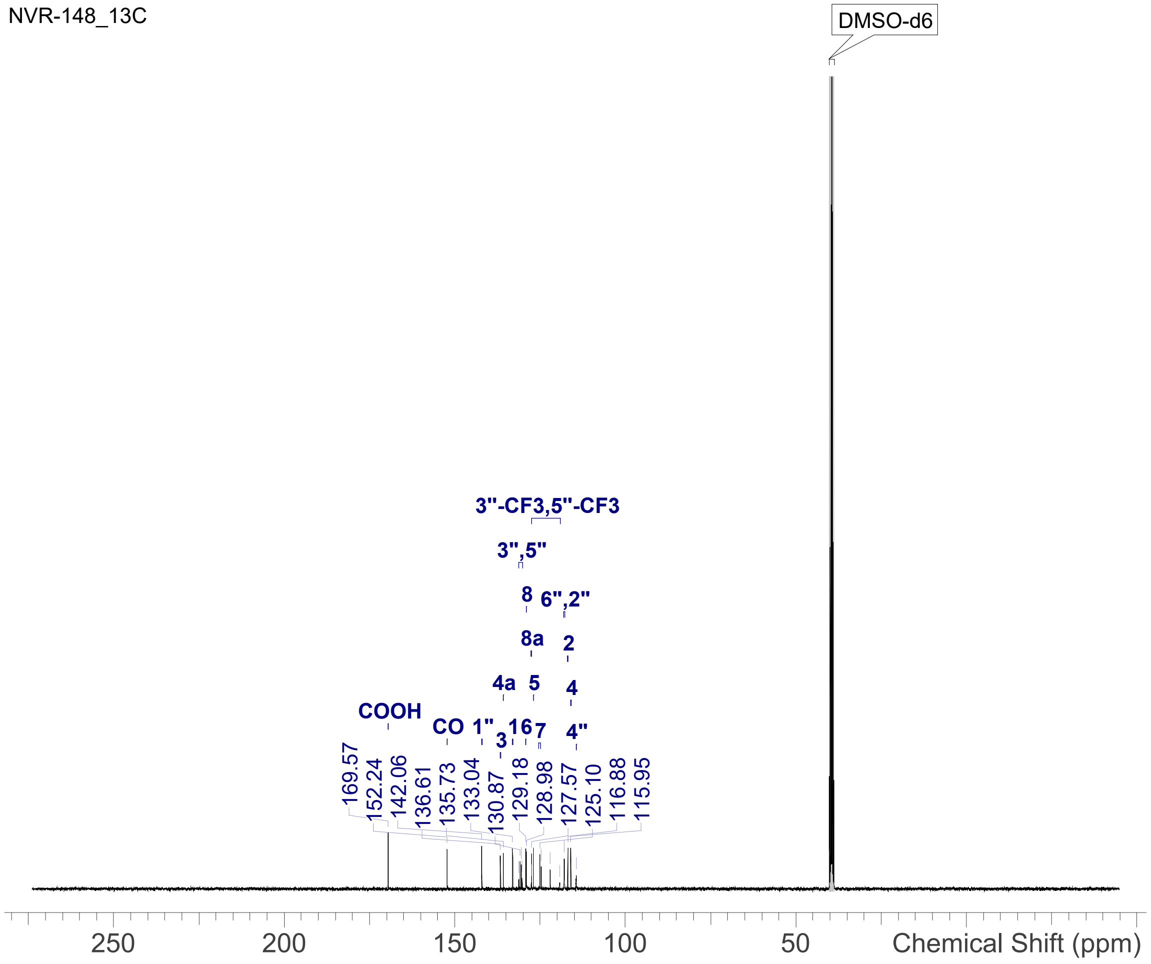

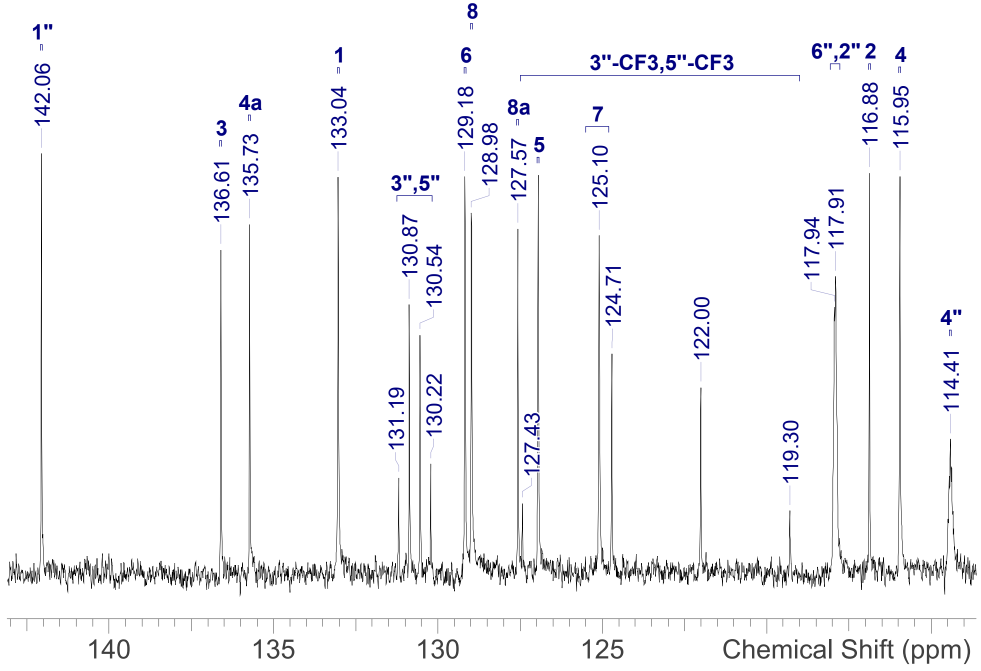

NVR-148_13C.spectrus

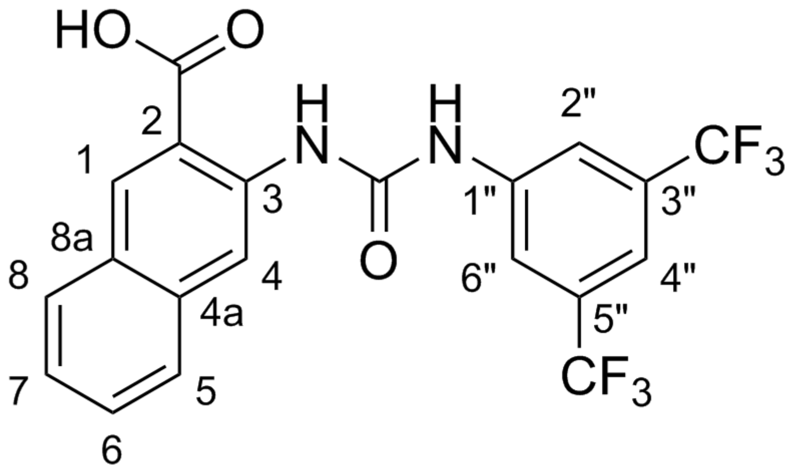

Shift (ppm)	F	m
-61.73	6	s

Acquisition Time (sec)	1.4680
Date	15 May 2019 23:41:47
Date Stamp	15 May 2019 23:41:47
Frequency (MHz)	376.4419
Nucleus	19F
Number of Transients	16
Solvent	DMSO-d6
Temperature (degree C)	25.000

^{19}F NMR (376 MHz, *DMSO-d_6*) δ ppm -61.73 (s, 6 F)

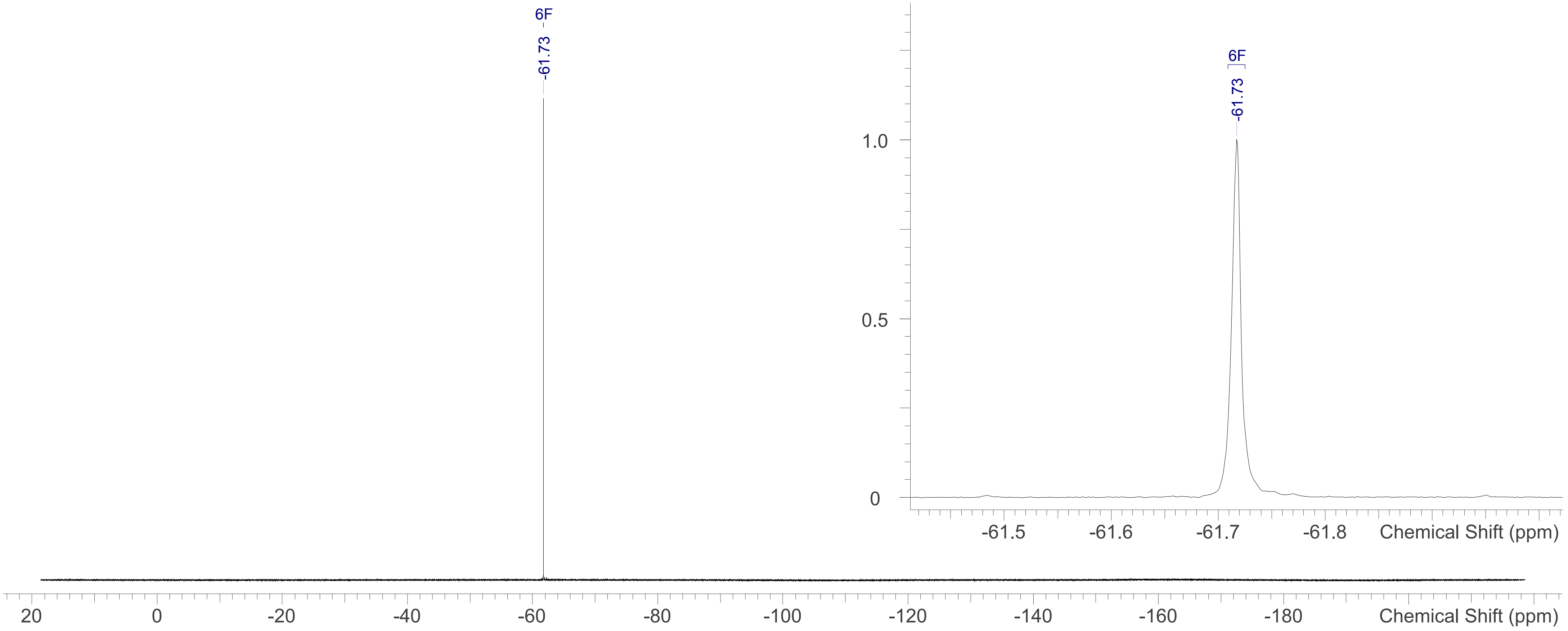

NVR-148_19F.spectrus

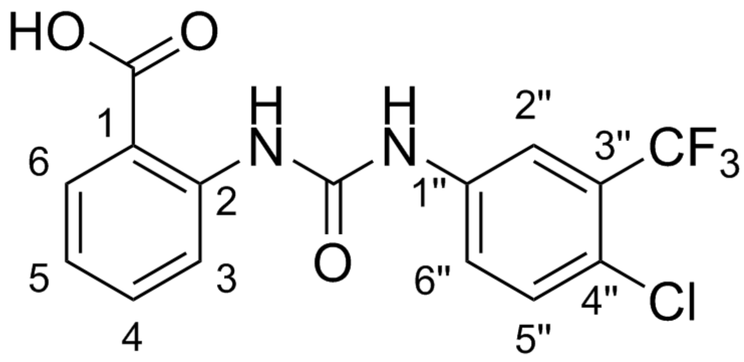

Shift (ppm)	H	m	J (Hz)	Assign
10.54	1	s	-	NH
10.29	1	s	-	NH''
8.37	1	dd	8.5, 0.8	H3
8.11	1	d	2.5	H2''
7.97	1	dd	8.0, 1.6	H6
7.77	1	dd	8.9, 2.5	H6''
7.62	1	d	8.8	H5''
7.56	1	ddd	8.6, 7.2, 1.6	H4
7.07	1	ddd	8.0, 7.2, 1.0	H5

Acquisition Time (sec)	7.9692
Date	10/05/2018 12:30:00
Date Stamp	10/05/2018 12:30:00
Frequency (MHz)	400.0720
Nucleus	1H
Number of Transients	16
Solvent	DMSO-d6

^{1}H NMR (400 MHz, *DMSO-d_6*) δ ppm 10.54 (s, 1 H), 10.29 (s, 1 H), 8.37 (dd, *J*=8.5, 0.8 Hz, 1 H), 8.11 (d, *J*=2.5 Hz, 1 H), 7.97 (dd, *J*=8.0, 1.6 Hz, 1 H), 7.77 (dd, *J*=8.9, 2.5 Hz, 1 H), 7.62 (d, *J*=8.8 Hz, 1 H), 7.56 (ddd, *J*=8.6, 7.2, 1.6 Hz, 1 H), 7.07 (ddd, *J*=8.0, 7.2, 1.0 Hz, 1 H)

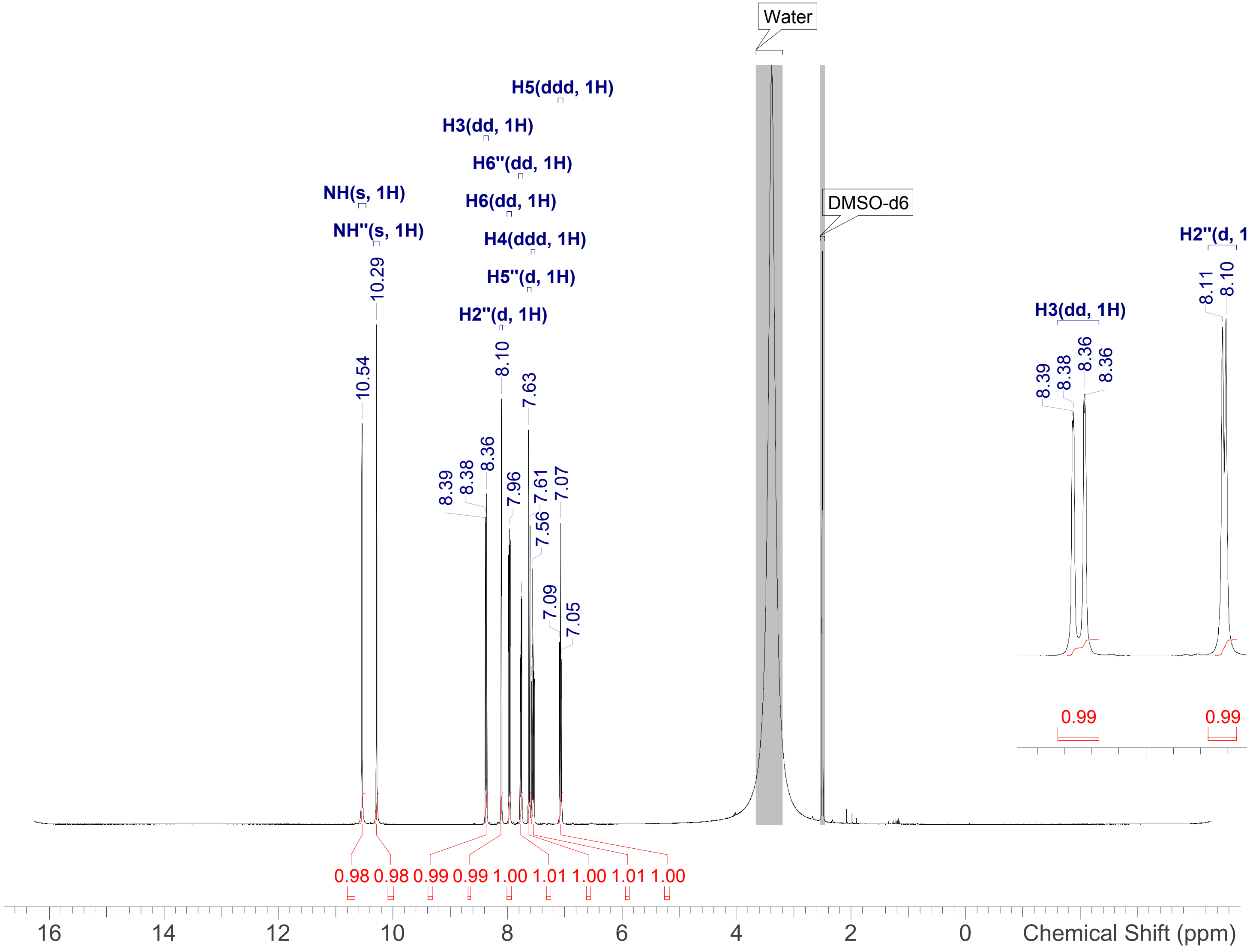

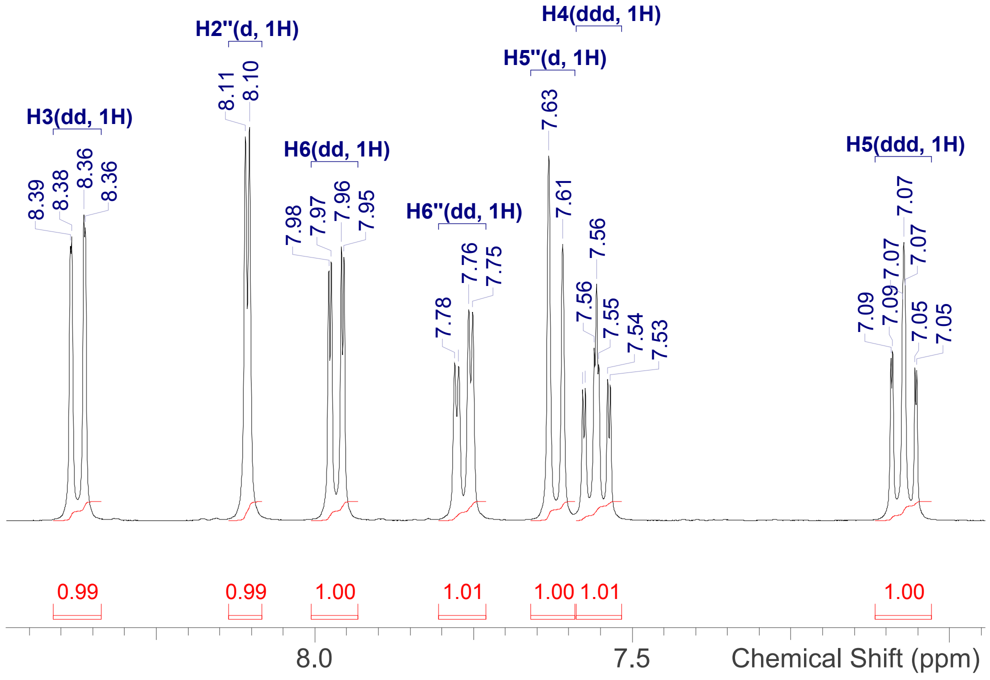

NVR-13_1H.spectrus

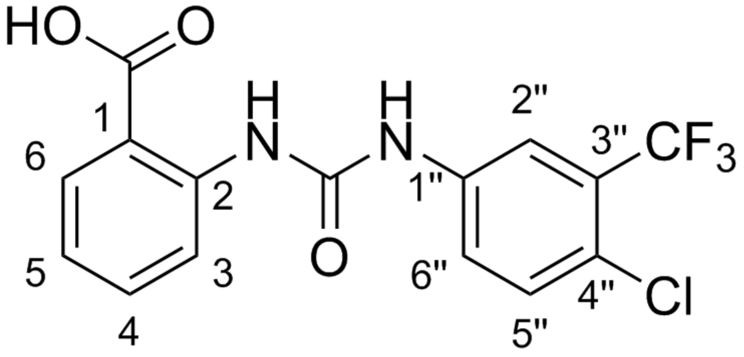

Shift (ppm)	C	m	J (Hz)	Assign
169.5	1	s	-	COOH
152.1	1	s	-	CO
141.8	1	s	-	2
139.4	1	s	-	1''
133.9	1	s	-	4
132.0	1	s	-	6
131.1	1	s	-	6''
126.7	1	q	30.8	3''
123.2	1	br s	-	5''
122.5	1	br q	1.5	4''
121.4	1	s	-	5
119.8	1	s	-	3
117.1	1	q	5.5	2''
115.6	1	s	-	1

Acquisition Time (sec)	2.0447
Date	10/05/2018 12:34:00
Date Stamp	10/05/2018 12:34:00
Frequency (MHz)	100.6090
Nucleus	13C
Number of Transients	64
Solvent	DMSO-d6

^{13}C NMR (101 MHz, *DMSO-d$_6$*) δ ppm 169.5 (s, 1 C), 152.1 (s, 1 C), 141.8 (s, 1 C), 139.4 (s, 1 C), 133.9 (s, 1 C), 132.0 (s, 1 C), 131.1 (s, 1 C), 126.7 (q, *J*=30.8 Hz, 1 C), 123.2 (br s, 1 C), 122.5 (br q, *J*=1.5 Hz, 1 C), 121.4 (s, 1 C), 119.8 (s, 1 C), 117.1 (q, *J*=5.5 Hz, 1 C), 115.6 (s, 1 C)

NVR-13_13C

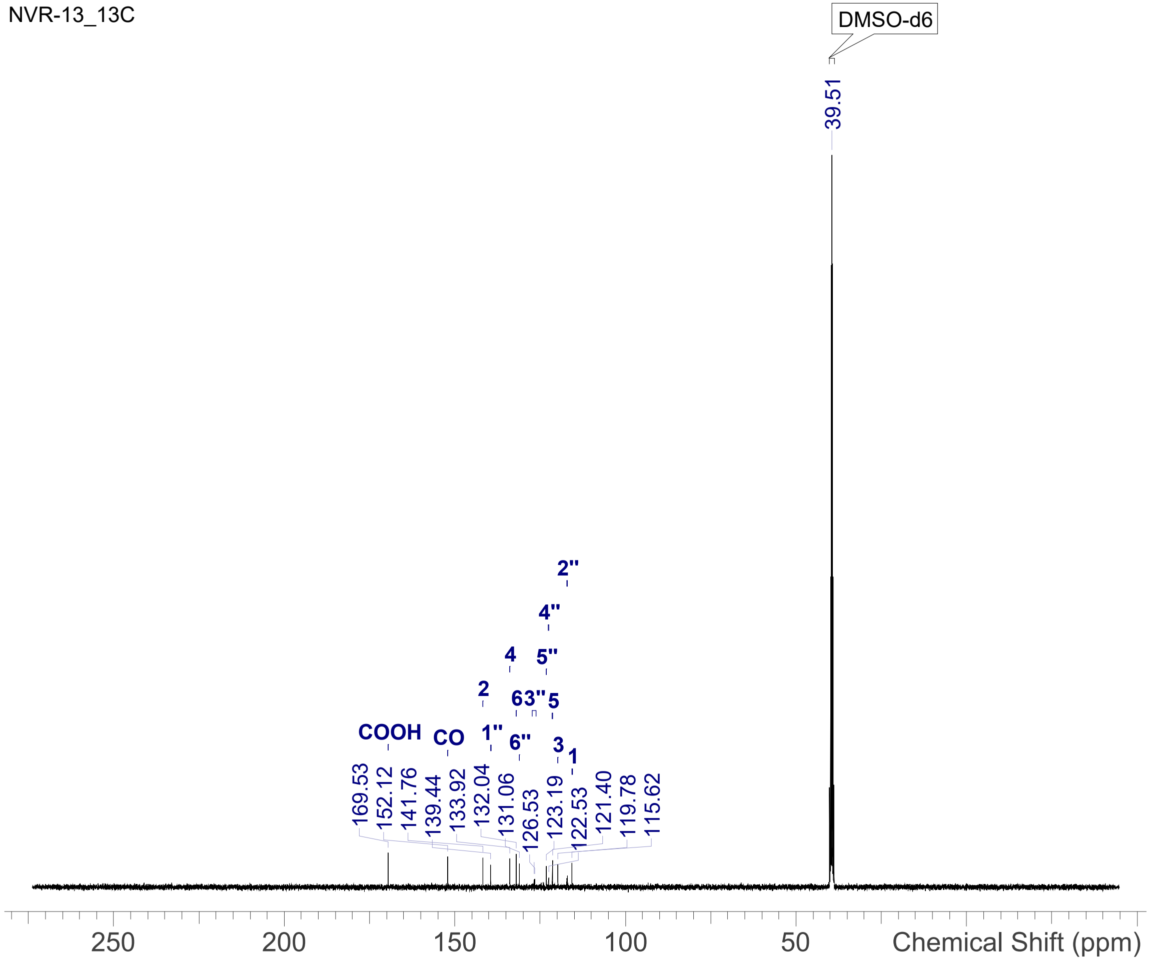

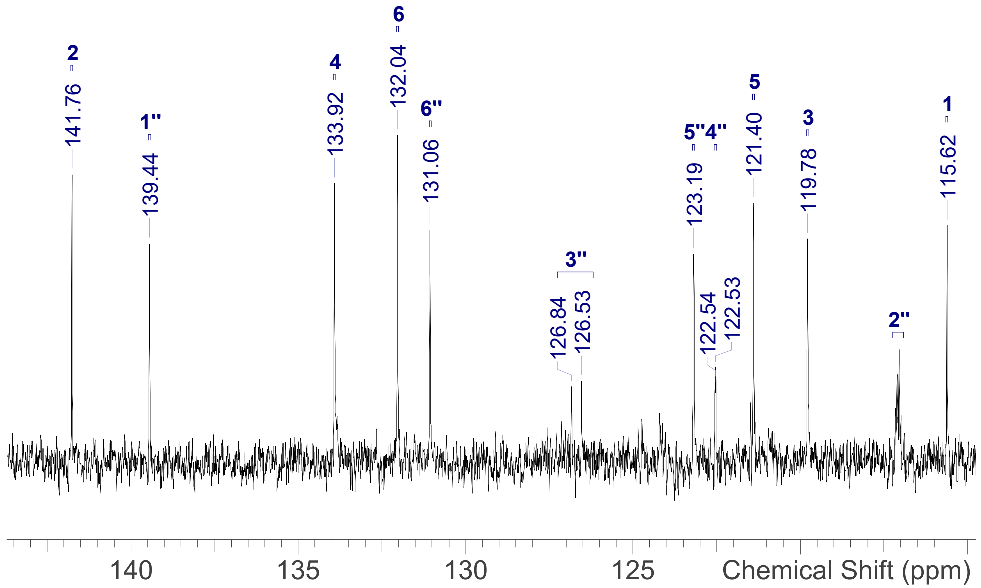

NVR-13_13C.spectrus

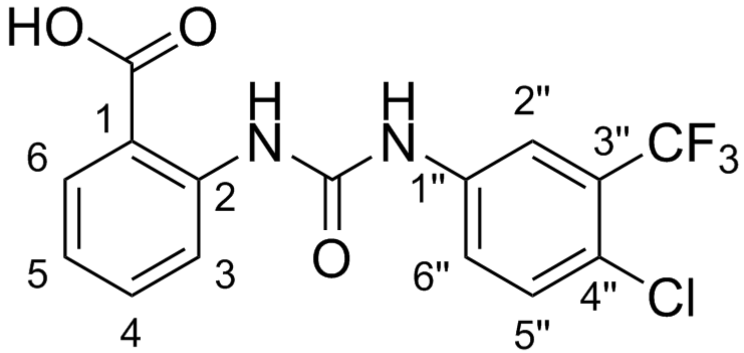

Shift (ppm)	F	m
-61.50	3	s

Acquisition Time (sec)	2.9360
Date	27/04/2018 10:30:00
Date Stamp	27/04/2018 10:30:00
Frequency (MHz)	376.4040
Nucleus	19F
Number of Transients	16
Solvent	DMSO-d6

^{19}F NMR (376 MHz, *DMSO-d_6*) δ ppm -61.50 (s, 3 F)

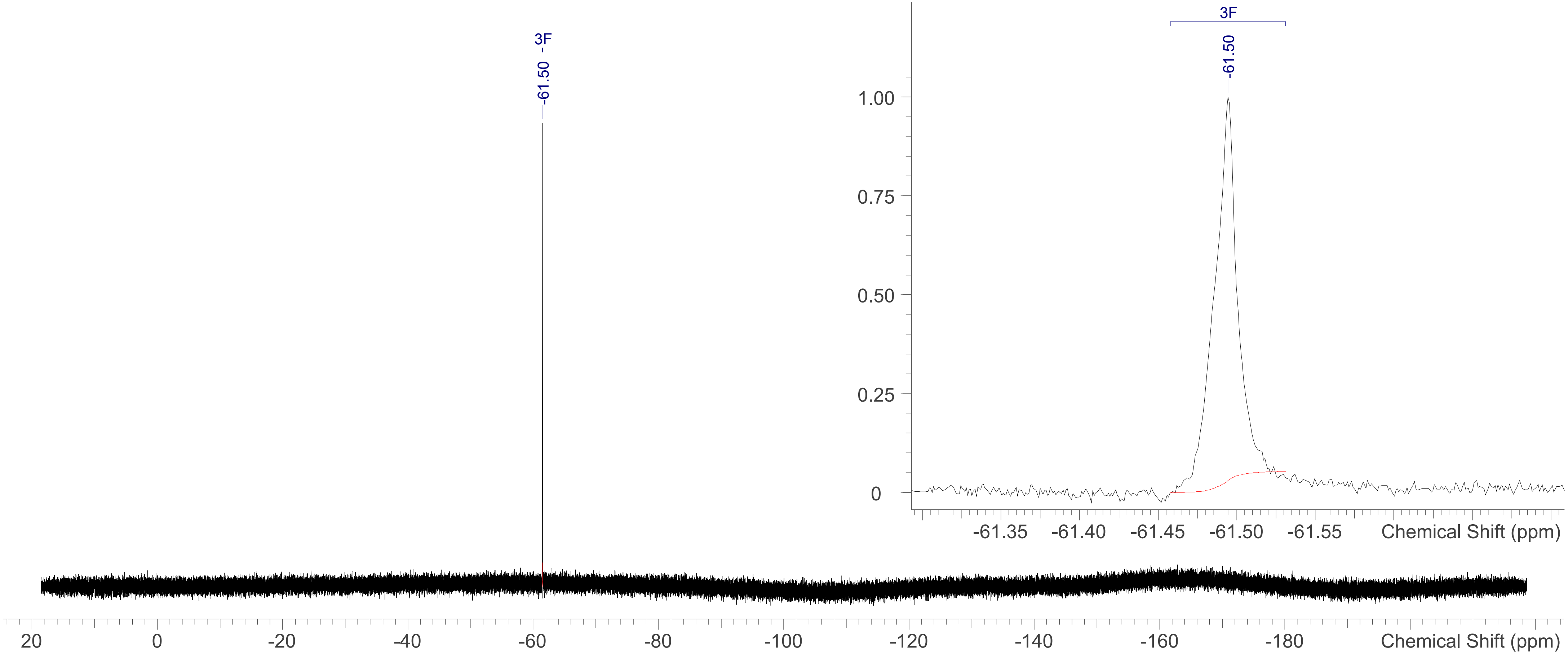

NVR-13_19F.spectrus

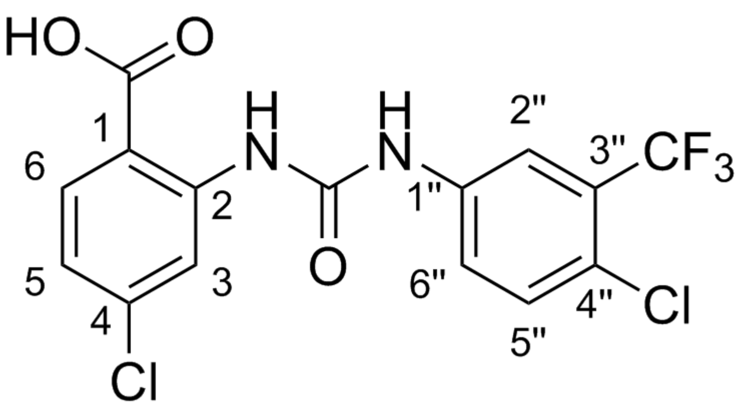

Shift (ppm)	H	m	J (Hz)	Assign
13.74	1	br s	-	COOH
10.67	1	s	-	NH
10.40	1	s	-	NH''
8.52	1	d	2.1	H3
8.09	1	d	2.6	H2''
7.96	1	d	8.5	H6
7.77	1	dd	8.8, 2.5	H6''
7.62	1	d	8.9	H5''
7.12	1	dd	8.5, 2.1	H5

Acquisition Time (sec)	3.2768
Date	05 Aug 2017 20:02:25
Date Stamp	05 Aug 2017 20:02:25
Frequency (MHz)	500.1900
Nucleus	1H
Number of Transients	16
Solvent	DMSO-d6
Temperature (degree C)	25.001

^{1}H NMR (500 MHz, *DMSO-d_6*) δ ppm 13.74 (br s, 1 H), 10.67 (s, 1 H), 10.40 (s, 1 H), 8.52 (d, *J*=2.1 Hz, 1 H), 8.09 (d, *J*=2.6 Hz, 1 H), 7.96 (d, *J*=8.5 Hz, 1 H), 7.77 (dd, *J*=8.8, 2.5 Hz, 1 H), 7.62 (d, *J*=8.9 Hz, 1 H), 7.12 (dd, *J*=8.5, 2.1 Hz, 1 H)

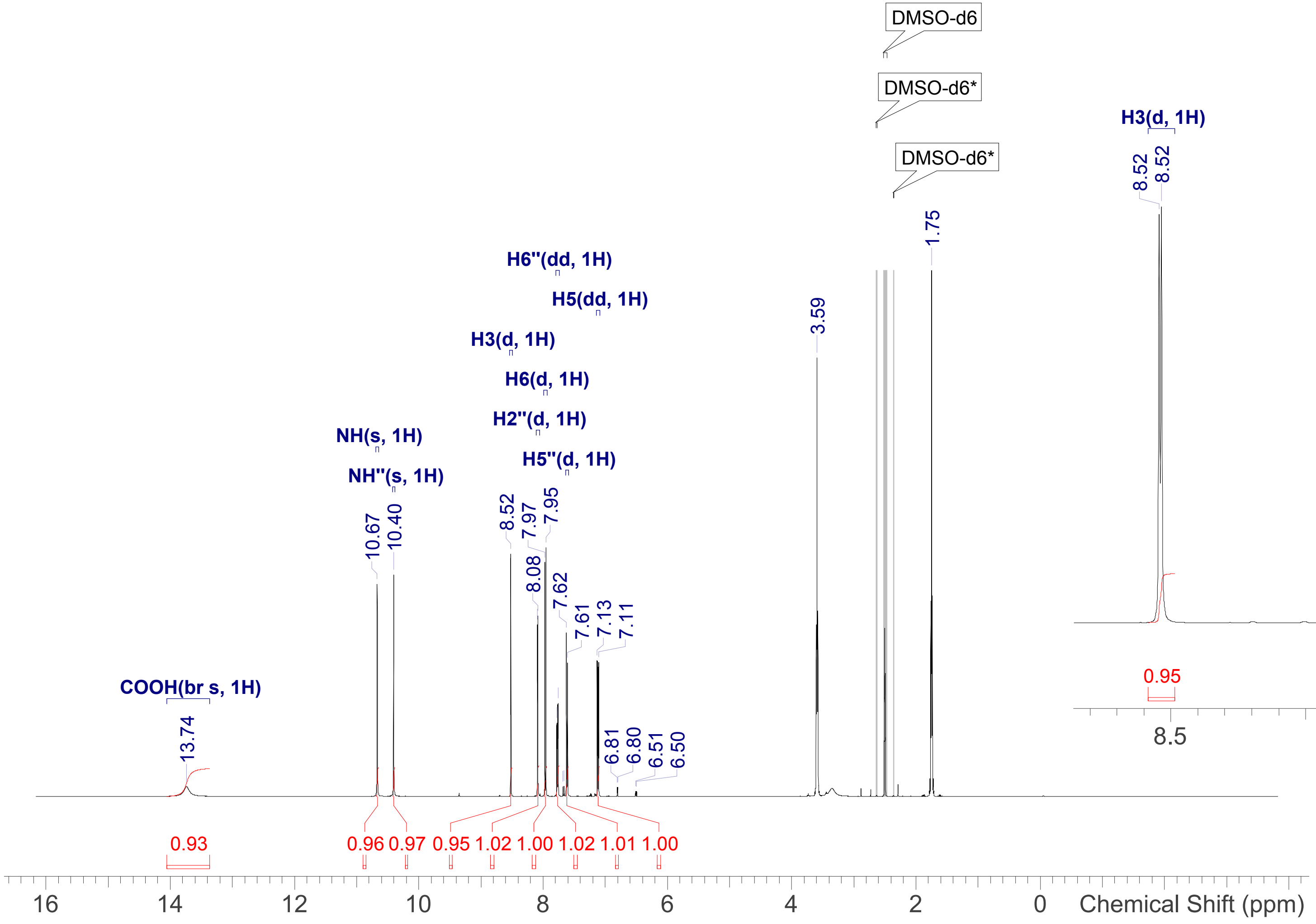

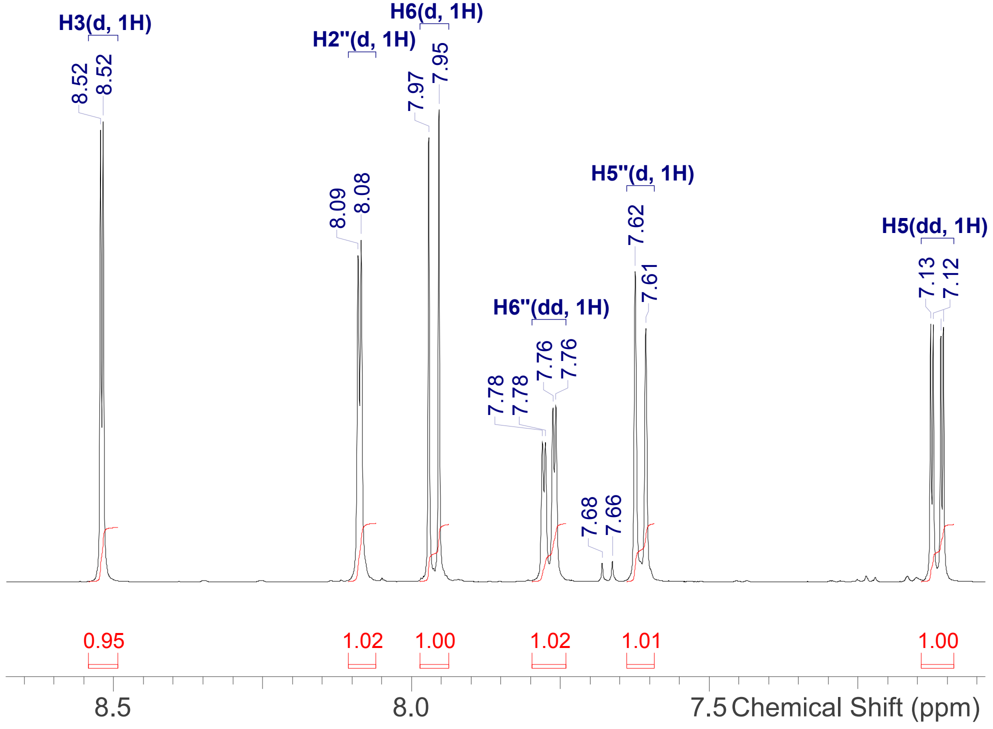

NVR-6_1H.spectrus

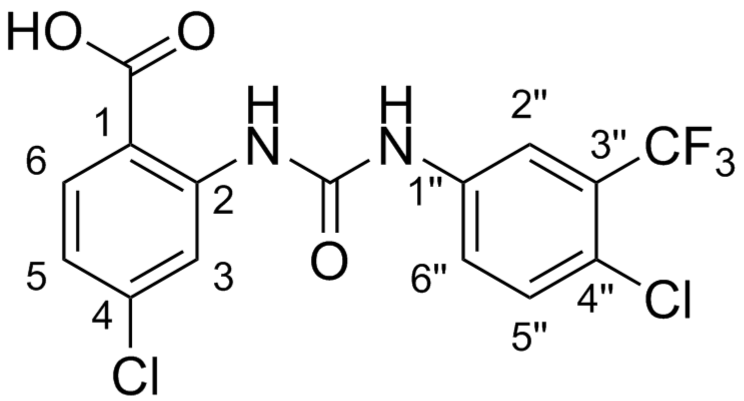

Shift (ppm)	C	m	J (Hz)	Assign
168.8	1	s	-	COOH
151.9	1	s	-	CO
143.0	1	s	-	4
139.1	1	s	-	2
138.5	1	s	-	1''
132.8	1	s	-	6
132.0	1	s	-	6''
126.7	1	q	30.3	3''
123.3	1	s	-	5''
122.8	1	br q	1.5	4''
122.8	1	q	273.1	CF3''
121.2	1	s	-	5
118.8	1	s	-	3
117.2	1	q	5.9	2''
114.1	1	s	-	1

**Acquisition Time (sec)**	2.0447
**Date**	05/08/2017 20:16:00
**Date Stamp**	05/08/2017 20:16:00
**Frequency (MHz)**	125.7870
**Nucleus**	13C
**Number of Transients**	256
**Solvent**	DMSO-d6

^{13}C NMR (126 MHz, *DMSO-d$_6$*) δ ppm 168.8 (s, 1 C), 151.9 (s, 1 C), 143.0 (s, 1 C), 139.1 (s, 1 C), 138.5 (s, 1 C), 132.8 (s, 1 C), 132.0 (s, 1 C), 126.7 (q, *J*=30.3 Hz, 1 C), 123.3 (s, 1 C), 122.8 (br q, *J*=1.5 Hz, 1 C), 121.2 (s, 1 C), 122.8 (q, *J*=273.1 Hz, 1 C), 118.8 (s, 1 C), 117.2 (q, *J*=5.9 Hz, 1 C), 114.1 (s, 1 C)

NVR-6_13C

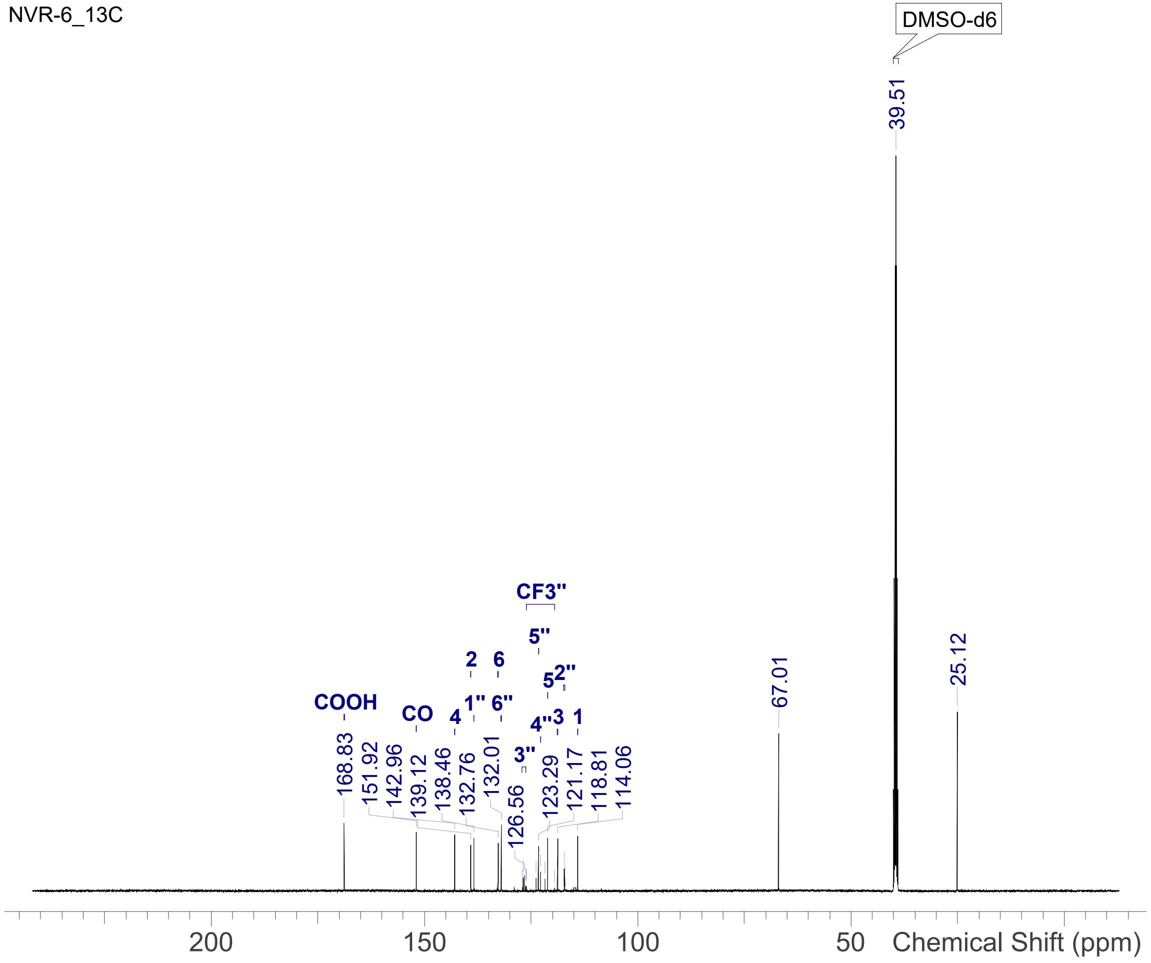

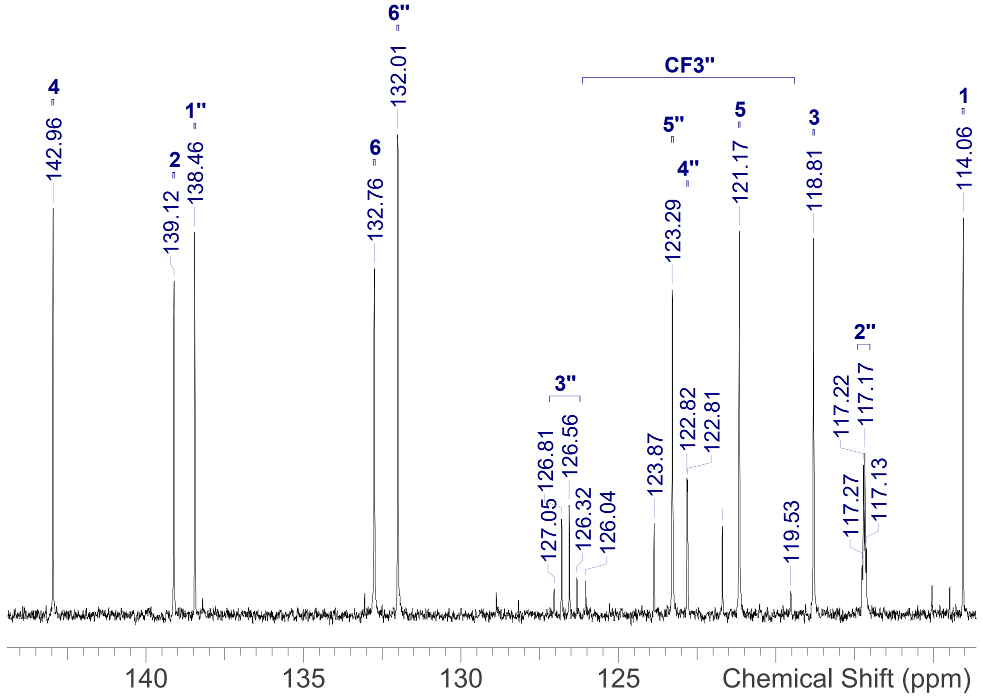

NVR-6_13C.spectrus

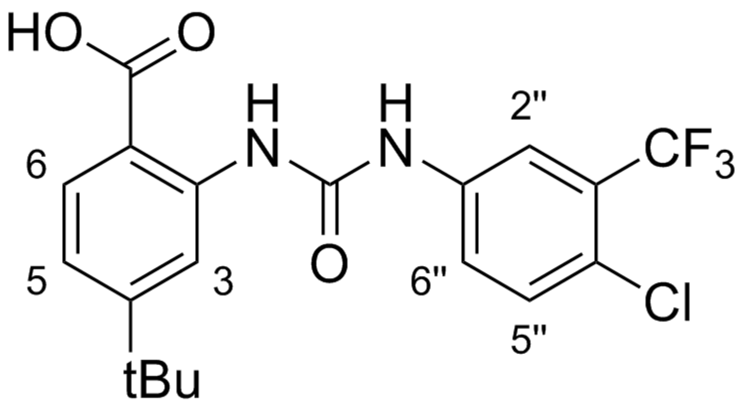

Shift (ppm)	H	m	J (Hz)	Assign
13.32	1	br s	-	COOH
10.54	1	s	-	NH
10.26	1	s	-	NH''
8.50	1	d	1.5	3
8.06	1	d	2.1	2''
7.89	1	d	8.4	6
7.82	1	dd	8.7, 2.1	6''
7.62	1	d	8.9	5''
7.11	1	br d	8.4	5
1.29	9	s	-	Me, Me, Me

Acquisition Time (sec)	6.5536
Date	24/10/2018 18:02:00
Date Stamp	24/10/2018 18:02:00
Frequency (MHz)	500.1930
Nucleus	1H
Number of Transients	16
Solvent	DMSO-d6

^{1}H NMR (500 MHz, *DMSO-d_6*) δ ppm 13.32 (br s, 1 H), 10.54 (s, 1 H), 10.26 (s, 1 H), 8.50 (d, *J*=1.5 Hz, 1 H), 8.06 (d, *J*=2.1 Hz, 1 H), 7.89 (d, *J*=8.4 Hz, 1 H), 7.82 (dd, *J*=8.7, 2.1 Hz, 1 H), 7.62 (d, *J*=8.9 Hz, 1 H), 7.11 (br d, *J*=8.4 Hz, 1 H), 1.29 (s, 9 H)

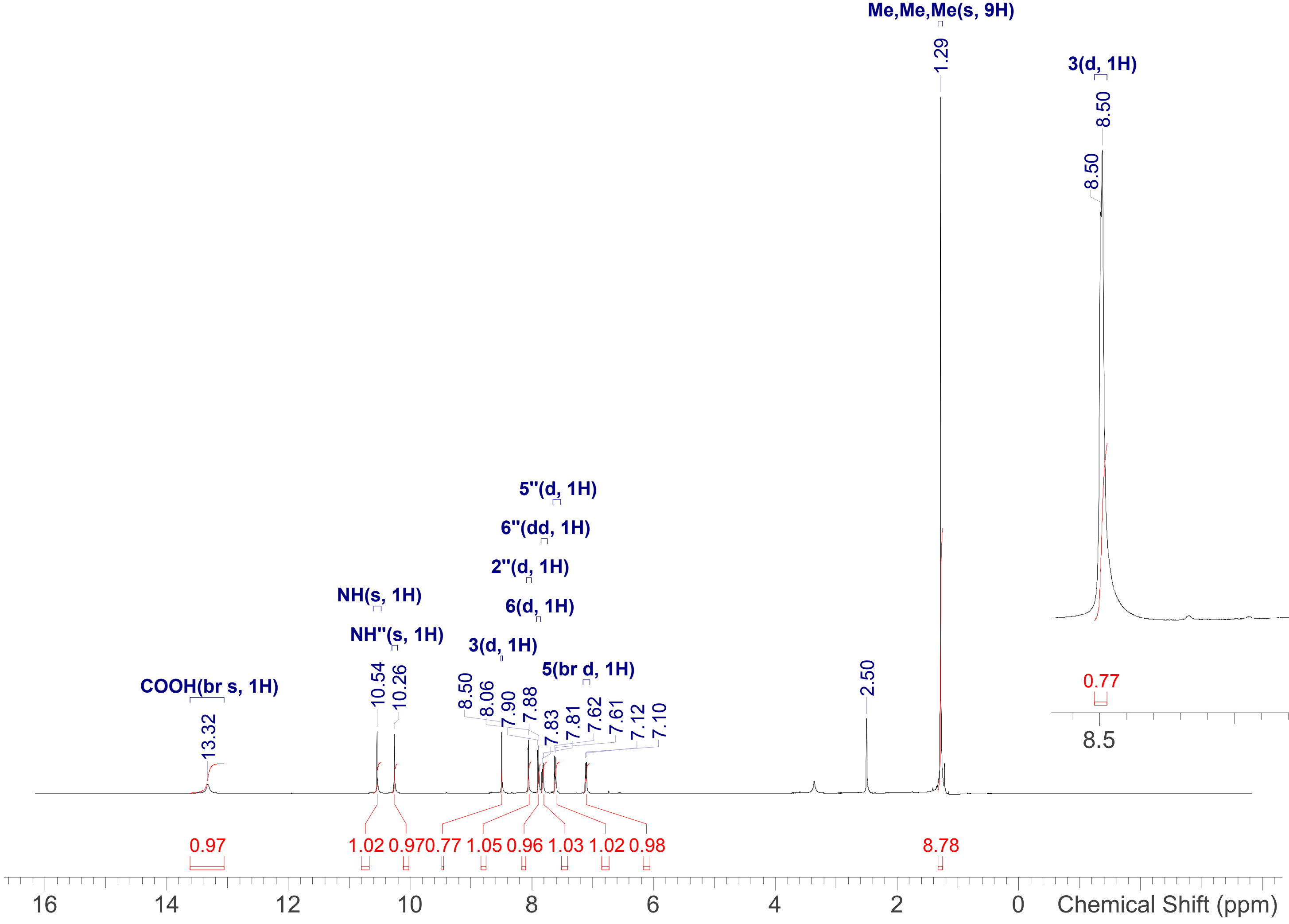

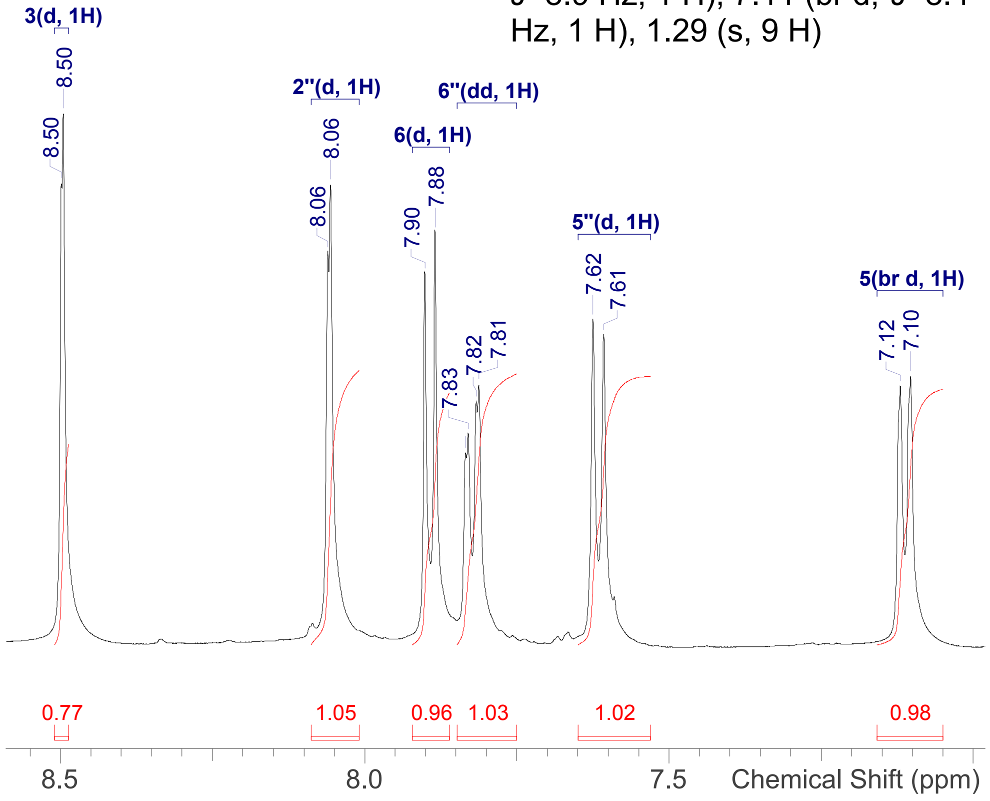

NVR-158_1H.spectrus

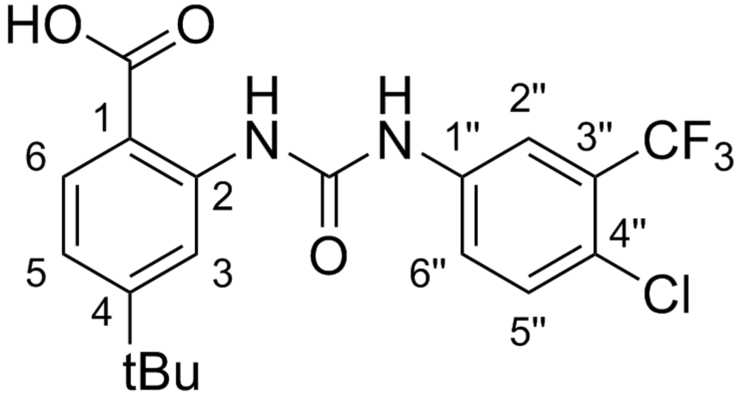

Shift (ppm)	C	m	J (Hz)	Assign
169.4	1	s	-	COOH
157.0	1	s	-	
157.0	1	s	-	4
152.2	1	s	-	CO
141.8	1	s	-	2
139.5	1	s	-	1''
132.0	1	s	-	6
130.8	1	s	-	5''
127.0	1	s	-	6''
126.6	1	q	30.8	3''
123.2	1	br s	-	4''
122.8	1	q	272.9	CF3
118.6	1	s	-	5
117.1	1	br q	4.9	2''
116.4	1	s	-	3
112.8	1	s	-	1
35.0	1	s	-	C(CH3)3
30.7	3	s	-	Me, Me, Me

Acquisition Time (sec)	2.0447
Date	24/10/2018 18:05:00
Date Stamp	24/10/2018 18:05:00
Frequency (MHz)	125.7870
Nucleus	13C
Number of Transients	32
Solvent	DMSO-d6

^{13}C NMR (126 MHz, *DMSO-d_6*) δ ppm 169.4 (s, 1 C), 157.0 (s, 1 C), 157.0 (s, 1 C), 152.2 (s, 1 C), 141.8 (s, 1 C), 139.5 (s, 1 C), 132.0 (s, 1 C), 130.8 (s, 1 C), 127.0 (s, 1 C), 126.6 (q, *J*=30.8 Hz, 1 C), 123.2 (br s, 1 C), 122.8 (q, *J*=272.9 Hz, 1 C), 118.6 (s, 1 C), 117.1 (br q, *J*=4.9 Hz, 1 C), 116.4 (s, 1 C), 112.8 (s, 1 C), 35.0 (s, 1 C), 30.7 (s, 3 C)

NVR-158_13C

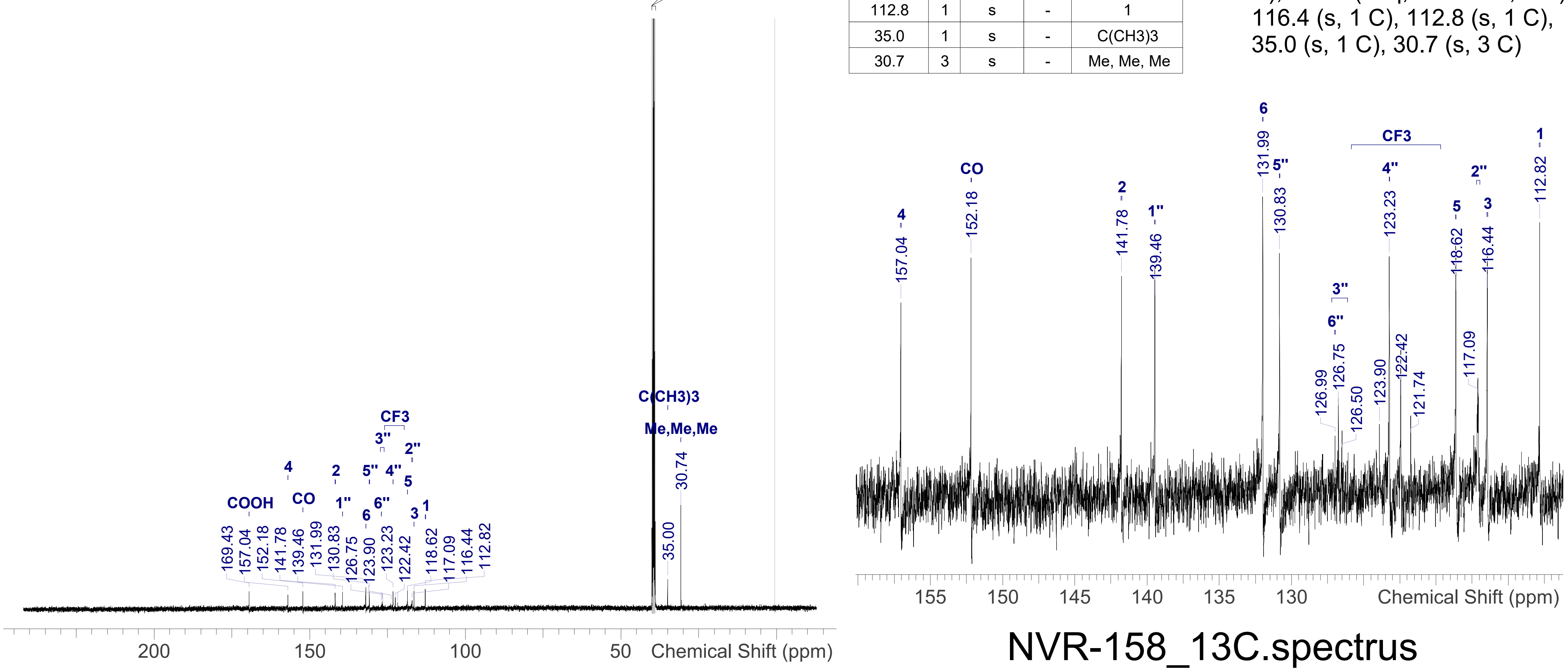

NVR-158_13C.spectrus

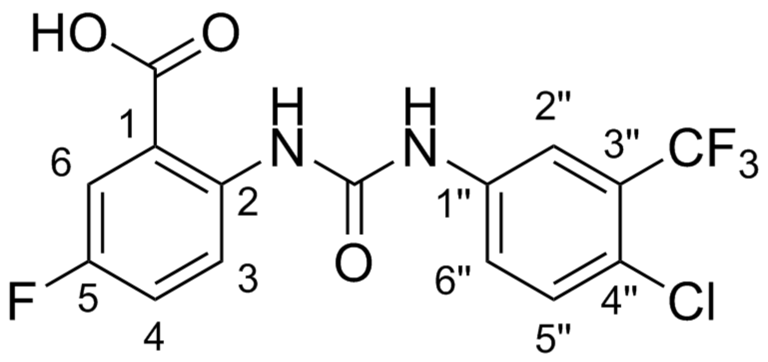

Shift (ppm)	H	m	J (Hz)	Assign
13.83	1	br s	-	COOH
10.36	1	s	-	NH''
10.28	1	s	-	NH
8.38	1	dd	9.3, 5.2	H3
8.09	1	d	2.6	H2''
7.75	1	dd	8.8, 2.5	H6''
7.67	1	dd	9.3, 3.2	H6
7.61	1	d	8.7	H5''
7.45	1	ddd	9.3, 7.9, 3.2	H4

Acquisition Time (sec)	3.2768
Date	04 Aug 2017 14:22:16
Date Stamp	04 Aug 2017 14:22:16
Frequency (MHz)	500.1900
Nucleus	1H
Number of Transients	16
Solvent	DMSO-d6
Temperature (degree C)	25.000

^{1}H NMR (500 MHz, *DMSO-d_6*) δ ppm 13.83 (br s, 1 H), 10.36 (s, 1 H), 10.28 (s, 1 H), 8.38 (dd, *J*=9.3, 5.2 Hz, 1 H), 8.09 (d, *J*=2.6 Hz, 1 H), 7.75 (dd, *J*=8.8, 2.5 Hz, 1 H), 7.67 (dd, *J*=9.3, 3.2 Hz, 1 H), 7.61 (d, *J*=8.7 Hz, 1 H), 7.45 (ddd, *J*=9.3, 7.9, 3.2 Hz, 1 H)

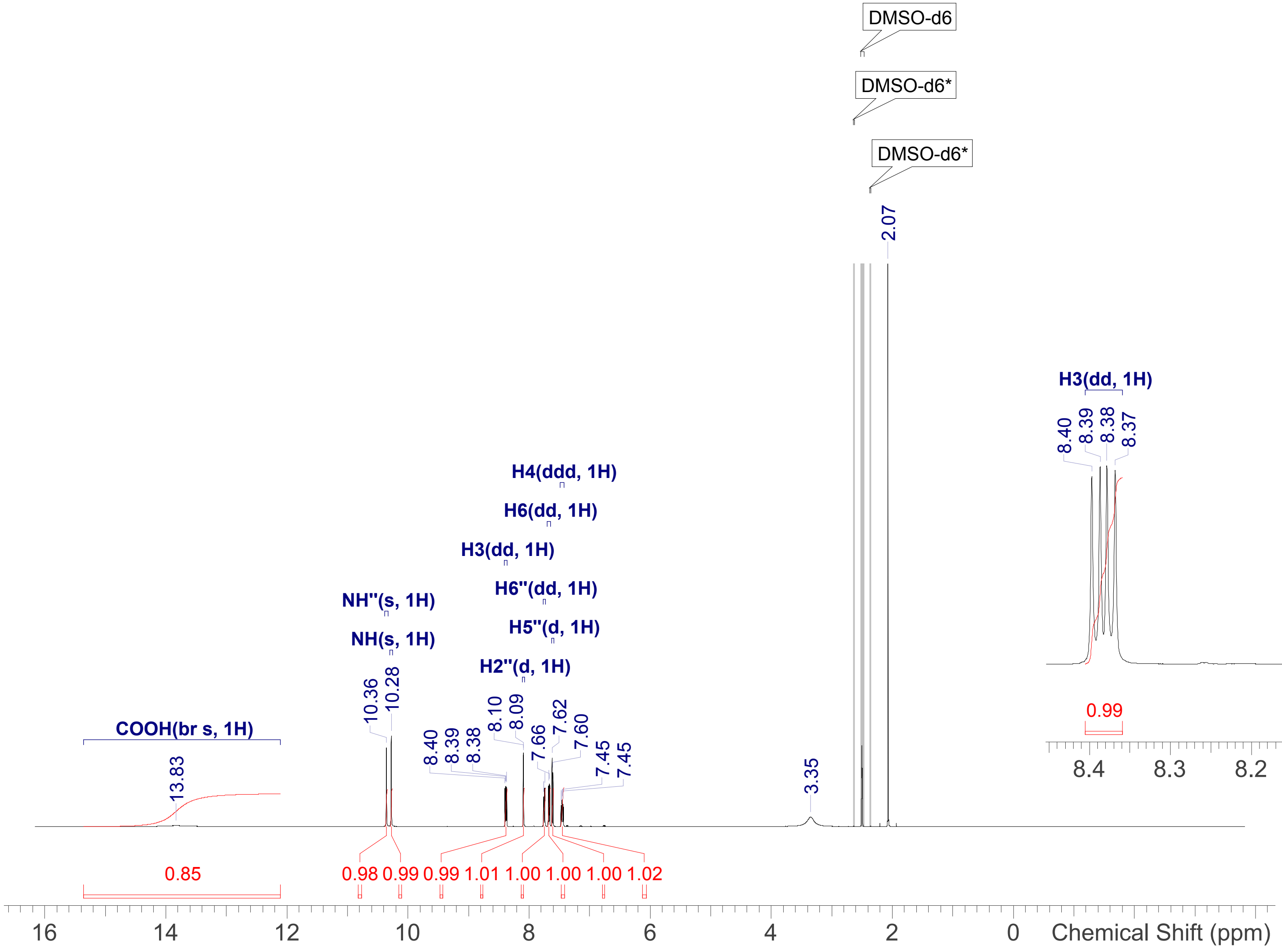

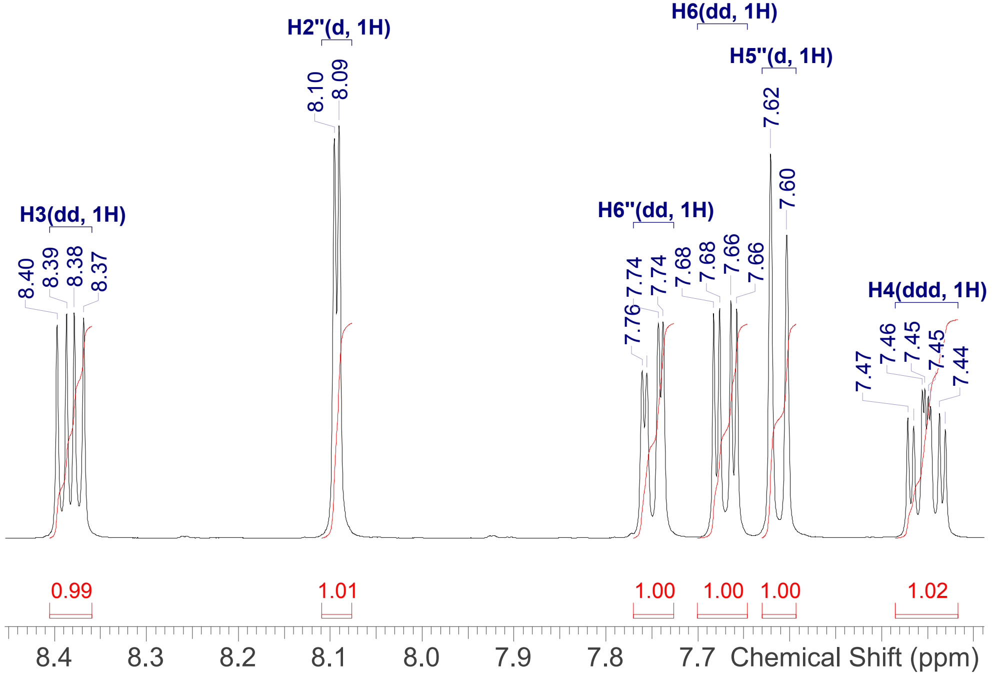

NVR-10_1H.spectrus

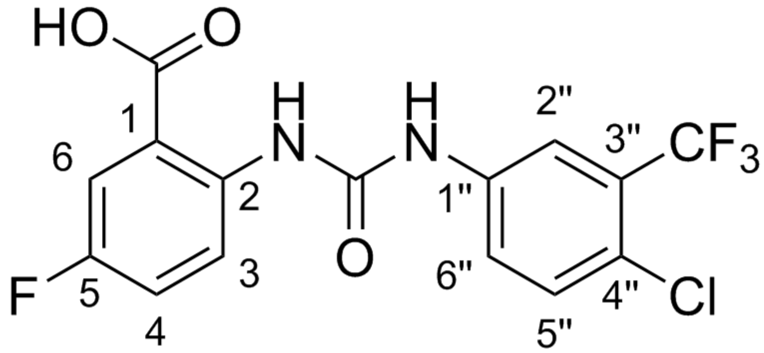

Shift (ppm)	C	m	J (Hz)	Assign
168.4	1	s	-	COOH
156.0	1	d	239.6	5
152.1	1	s	-	CO
139.4	1	s	-	1"
138.2	1	d	2.0	2
132.0	1	s	-	5"
126.7	1	q	30.3	3"
123.1	1	s	-	6"
122.8	1	q	272.9	CF3
122.5	1	br q	1.5	4"
122.0	1	d	7.3	3
120.9	1	d	22.0	4
117.2	1	d	6.8	1
117.0	1	q	5.7	2"
116.5	1	d	23.5	6

Acquisition Time (sec)	2.0447
Date	05/08/2017 14:42:00
Date Stamp	05/08/2017 14:42:00
Frequency (MHz)	125.7870
Nucleus	13C
Number of Transients	256
Solvent	DMSO-d6

^{13}C NMR (126 MHz, *DMSO-d_6*) δ ppm 168.4 (s, 1 C), 156.0 (d, *J*=239.6 Hz, 1 C), 152.1 (s, 1 C), 139.4 (s, 1 C), 138.2 (d, *J*=2.0 Hz, 1 C), 132.0 (s, 1 C), 126.7 (q, *J*=30.3 Hz, 1 C), 123.1 (s, 1 C), 122.5 (br q, *J*=1.5 Hz, 1 C), 122.0 (d, *J*=7.3 Hz, 1 C), 120.9 (d, *J*=22.0 Hz, 1 C), 122.8 (q, *J*=272.9 Hz, 1 C), 117.2 (d, *J*=6.8 Hz, 1 C), 117.0 (q, *J*=5.7 Hz, 1 C), 116.5 (d, *J*=23.5 Hz, 1 C)

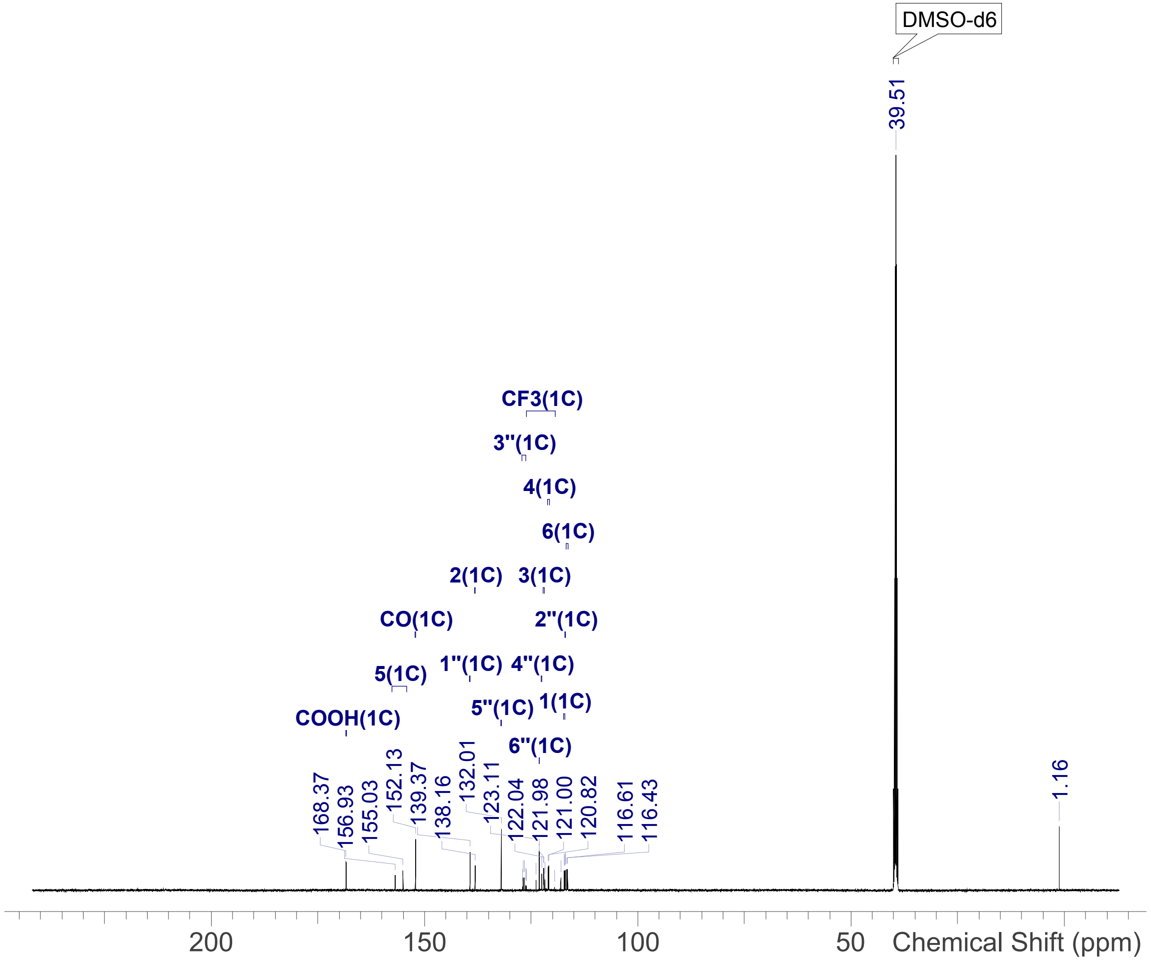

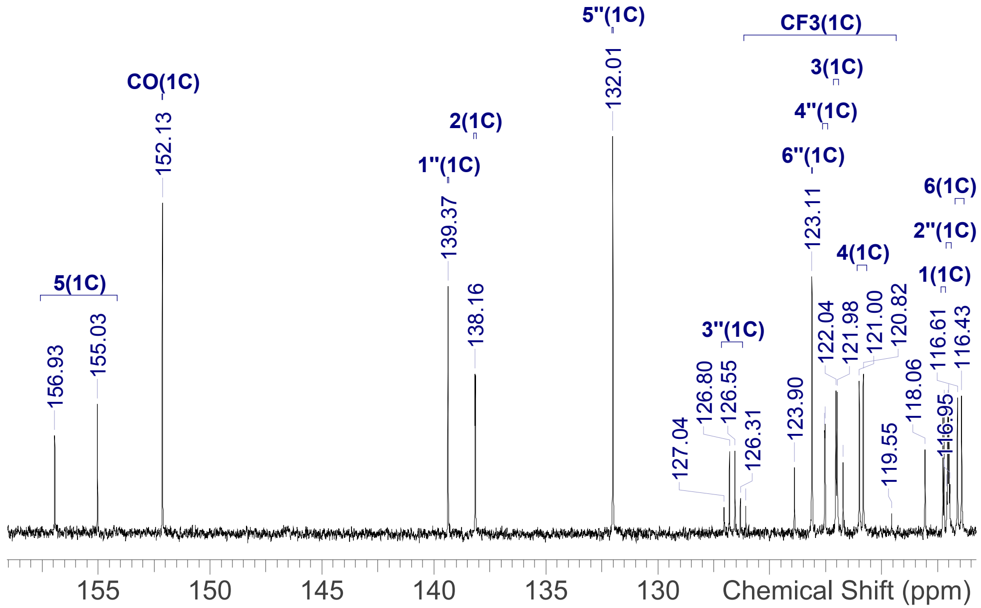

NVR-10_13C.spectrus

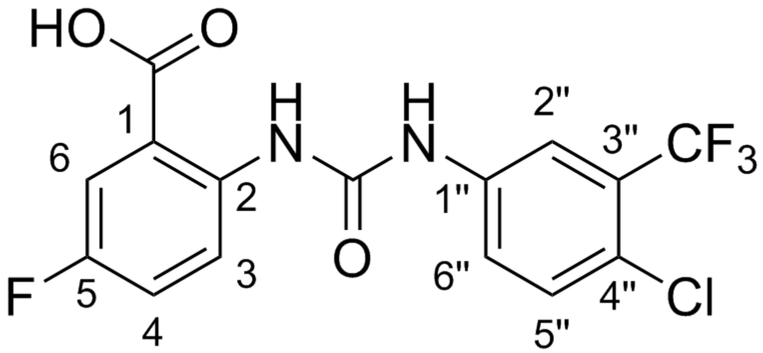

Shift (ppm)	F	m	J (Hz)
-61.54	3	s	-
-121.09	1	ddd	9.2, 8.2, 5.1

Acquisition Time (sec)	2.9360
Date	05/08/2017 15:17:00
Date Stamp	05/08/2017 15:17:00
Frequency (MHz)	470.6020
Nucleus	19F
Number of Transients	128
Solvent	DMSO-d6

^{19}F NMR (471 MHz, *DMSO-d_6*) δ ppm -61.54 (s, 3 F), -121.09 (ddd, *J*=9.2, 8.2, 5.1 Hz, 1 F)

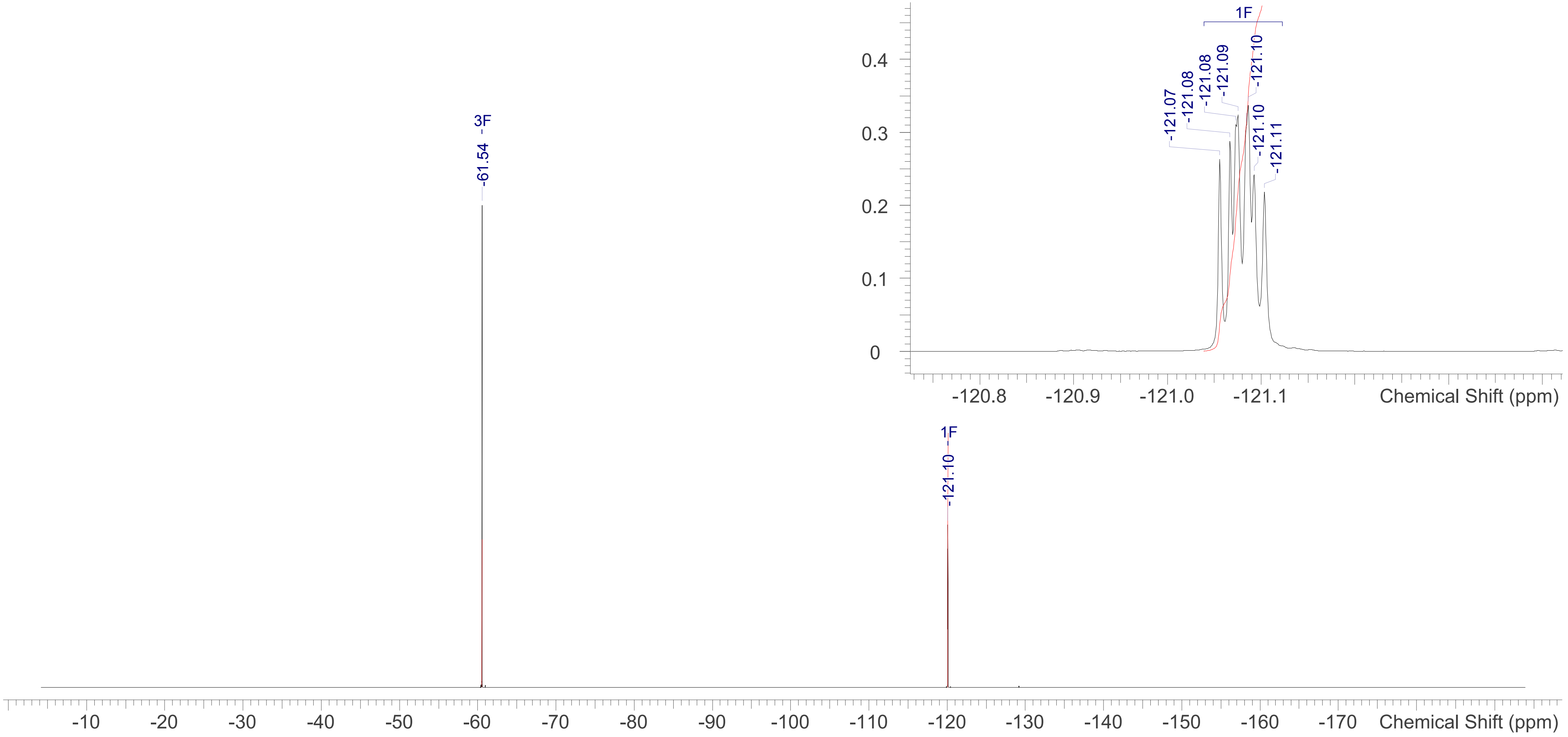

NVR-10_19F.spectrus

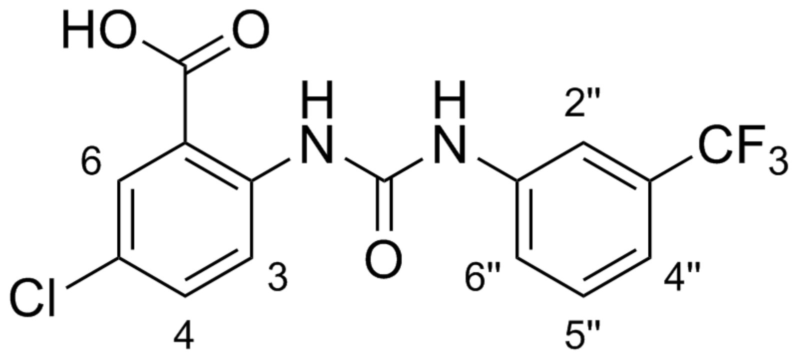

Shift (ppm)	H	m	J (Hz)	Assign
10.44	1	s	-	NH"
10.24	1	s	-	NH
8.43	1	d	9.2	3
8.00	1	s	-	2"
7.90	1	d	2.1	6
7.69	1	br d	8.4	6"
7.61	1	dd	9.1, 2.0	4
7.52	1	t	8.0	5"
7.33	1	br d	7.8	4"

Acquisition Time (sec)	5.2429
Date	22/10/2019 11:43:00
Date Stamp	22/10/2019 11:43:00
Frequency (MHz)	500.1870
Nucleus	1H
Number of Transients	128
Solvent	DMSO-d6

^{1}H NMR (500 MHz, *DMSO-d_6*) δ ppm 10.44 (s, 1 H), 10.24 (s, 1 H), 8.43 (d, *J*=9.2 Hz, 1 H), 8.00 (s, 1 H), 7.90 (d, *J*=2.1 Hz, 1 H), 7.69 (br d, *J*=8.4 Hz, 1 H), 7.61 (dd, *J*=9.1, 2.0 Hz, 1 H), 7.52 (t, *J*=8.0 Hz, 1 H), 7.33 (br d, *J*=7.8 Hz, 1 H)

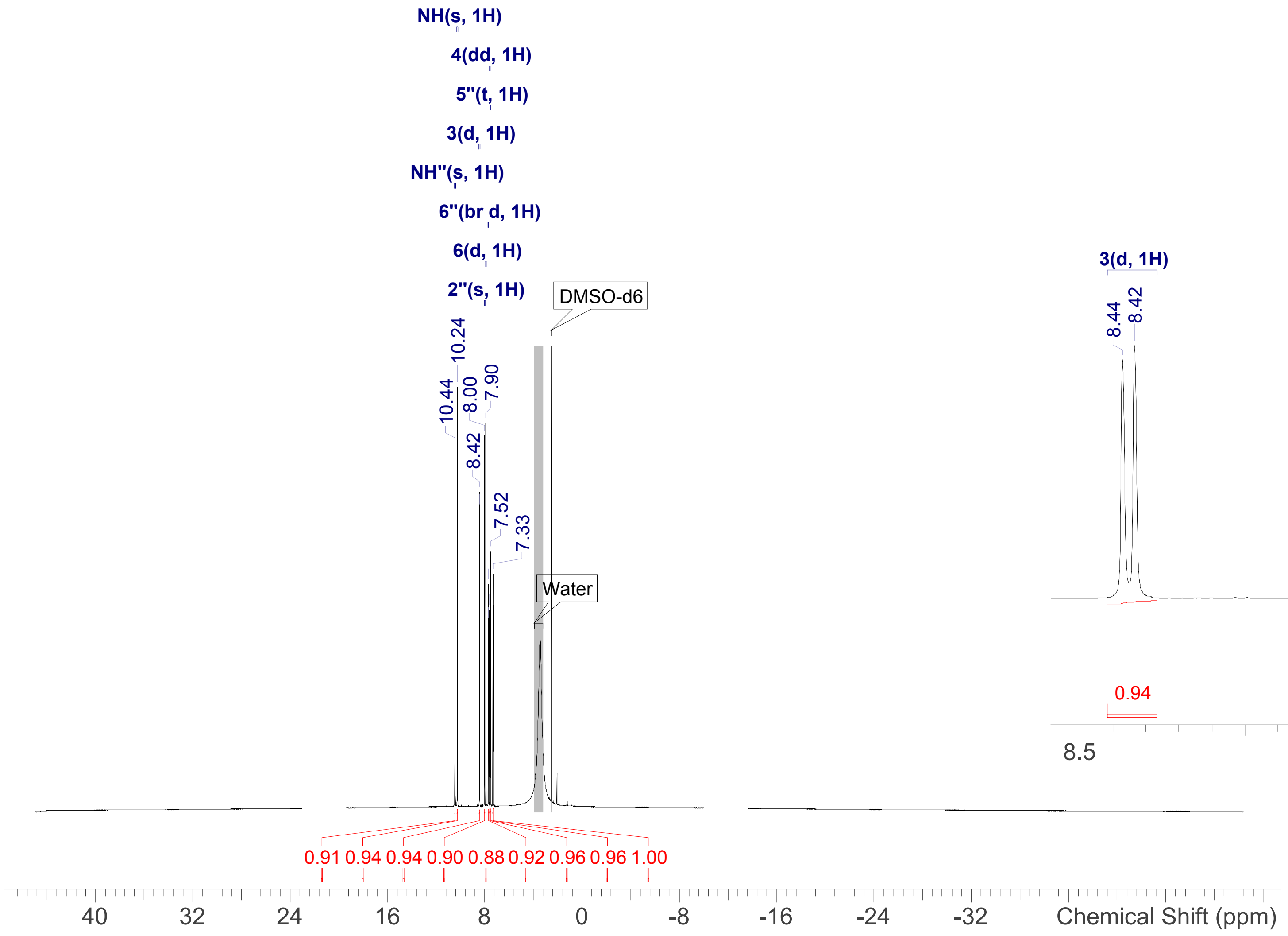

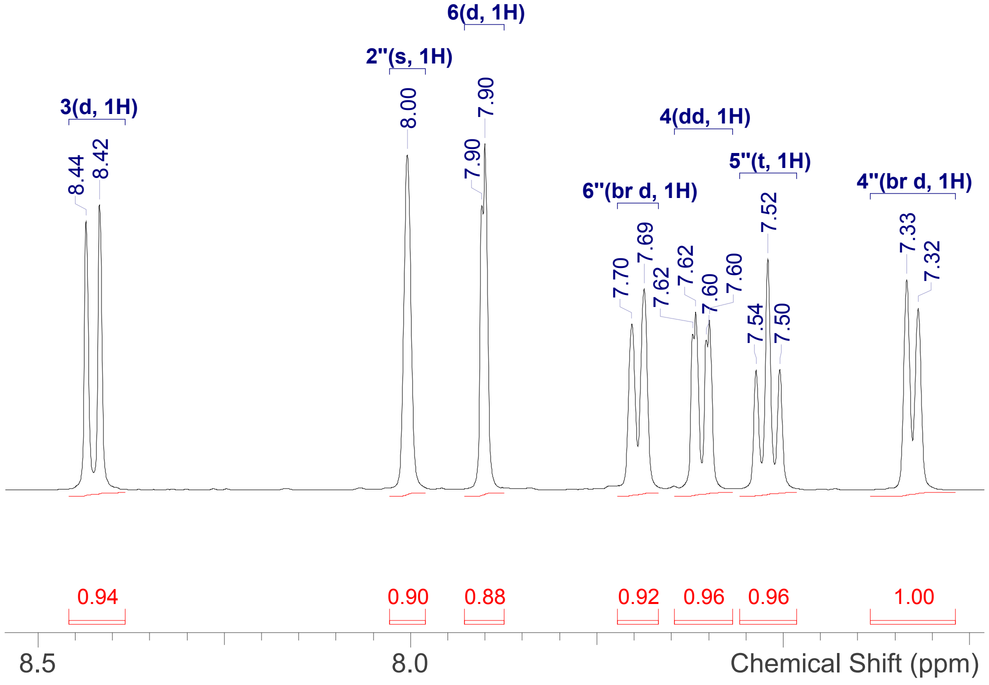

NVR-162_1H.spectrus

NVR-117 HRMS

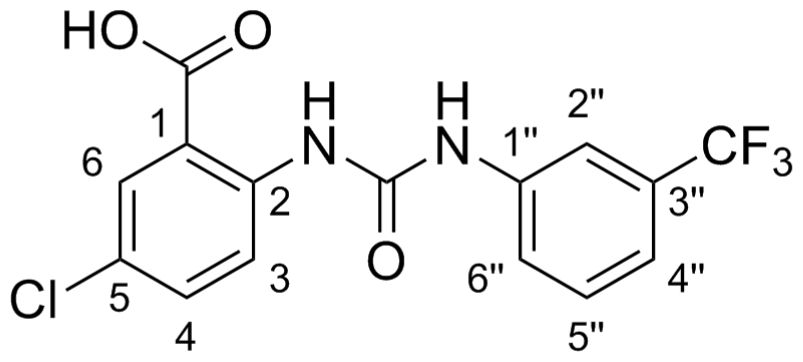

Shift (ppm)	C	m	J (Hz)	Assign
168.3	1	s	-	COOH
152.1	1	s	-	CO
140.8	1	s	-	1''
140.5	1	s	-	2
133.5	1	s	-	4
130.1	1	s	-	6
130.0	1	s	-	5''
129.5	1	q	31.6	3''
124.8	1	s	-	5
124.2	1	q	272.1	CF3
122.2	1	s	-	3
121.7	1	s	-	6''
118.5	1	br q	3.9	2''
117.3	1	s	-	1
114.6	1	br q	2.9	4''

Acquisition Time (sec)	2.0447
Date	22/10/2019 22:18:00
Date Stamp	22/10/2019 22:18:00
Frequency (MHz)	125.7870
Nucleus	13C
Number of Transients	2048
Solvent	DMSO-d6

^{13}C NMR (126 MHz, *DMSO-d_6*) δ ppm 168.3 (s, 1 C), 152.1 (s, 1 C), 140.8 (s, 1 C), 140.5 (s, 1 C), 133.5 (s, 1 C), 130.1 (s, 1 C), 130.0 (s, 1 C), 129.5 (q, *J*=31.6 Hz, 1 C), 124.8 (s, 1 C), 122.2 (s, 1 C), 121.7 (s, 1 C), 124.2 (q, *J*=272.1 Hz, 1 C), 118.5 (br q, *J*=3.9 Hz, 1 C), 117.3 (s, 1 C), 114.6 (br q, *J*=2.9 Hz, 1 C)

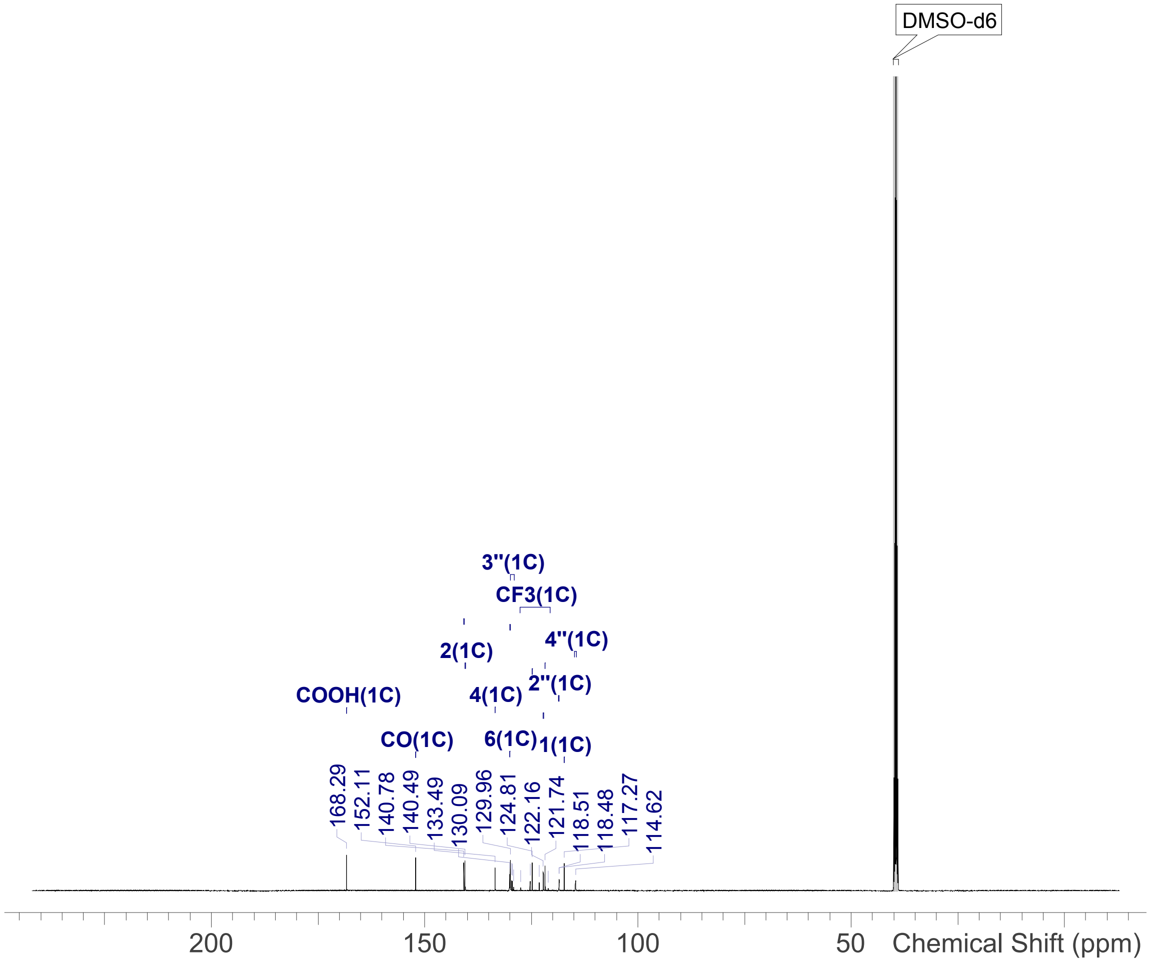

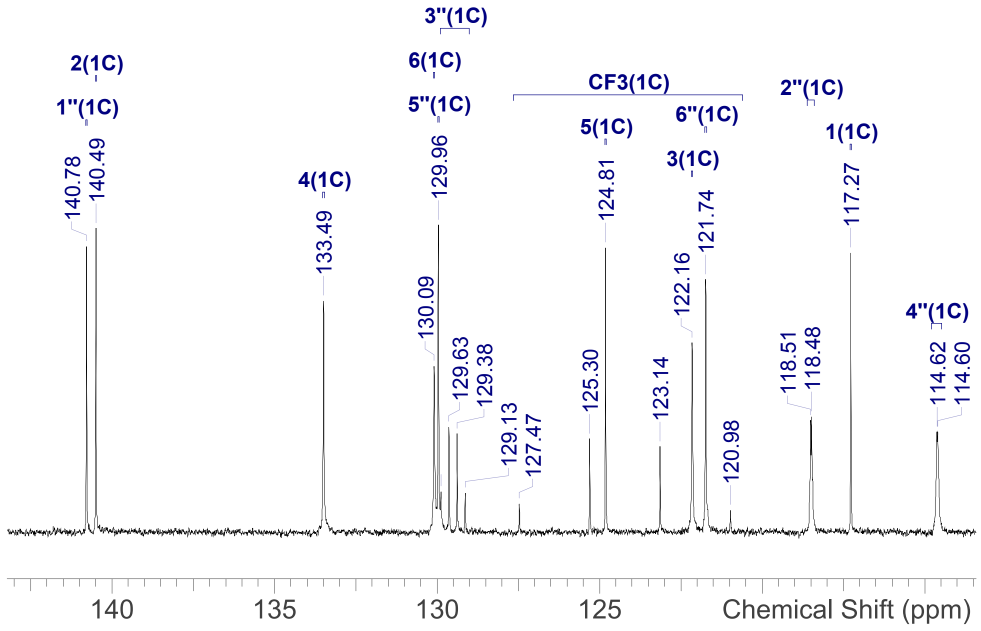

NVR-162_13C.spectrus

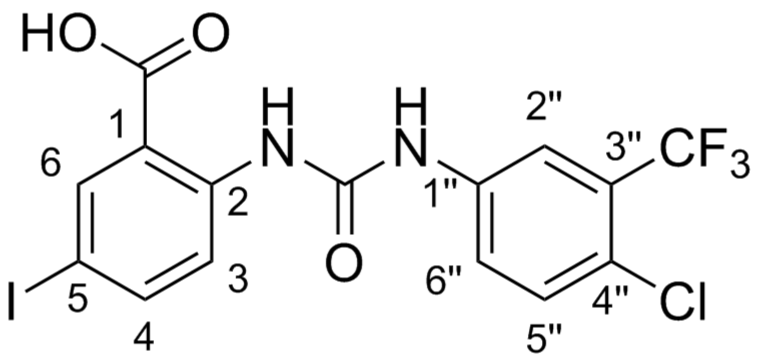

Shift (ppm)	H	m	J (Hz)	Assign
13.83	1	br s	-	COOH
10.48	1	s	-	NH
10.34	1	s	-	NH''
8.23	1	d	9.0	H3
8.20	1	br s	-	H6
8.09	1	br s	-	H2''
7.85	1	br d	9.0	H4
7.75	1	br d	8.9	H6''
7.61	1	d	8.9	H5''

Acquisition Time (sec)	6.5536
Date	08/08/2018 18:19:00
Date Stamp	08/08/2018 18:19:00
Frequency (MHz)	500.1930
Nucleus	1H
Number of Transients	16
Solvent	DMSO-d6

^{1}H NMR (500 MHz, *DMSO-d_6*) δ ppm 13.83 (br s, 1 H), 10.48 (s, 1 H), 10.34 (s, 1 H), 8.23 (d, *J*=9.0 Hz, 1 H), 8.20 (br s, 1 H), 8.09 (br s, 1 H), 7.85 (br d, *J*=9.0 Hz, 1 H), 7.75 (br d, *J*=8.9 Hz, 1 H), 7.61 (d, *J*=8.9 Hz, 1 H)

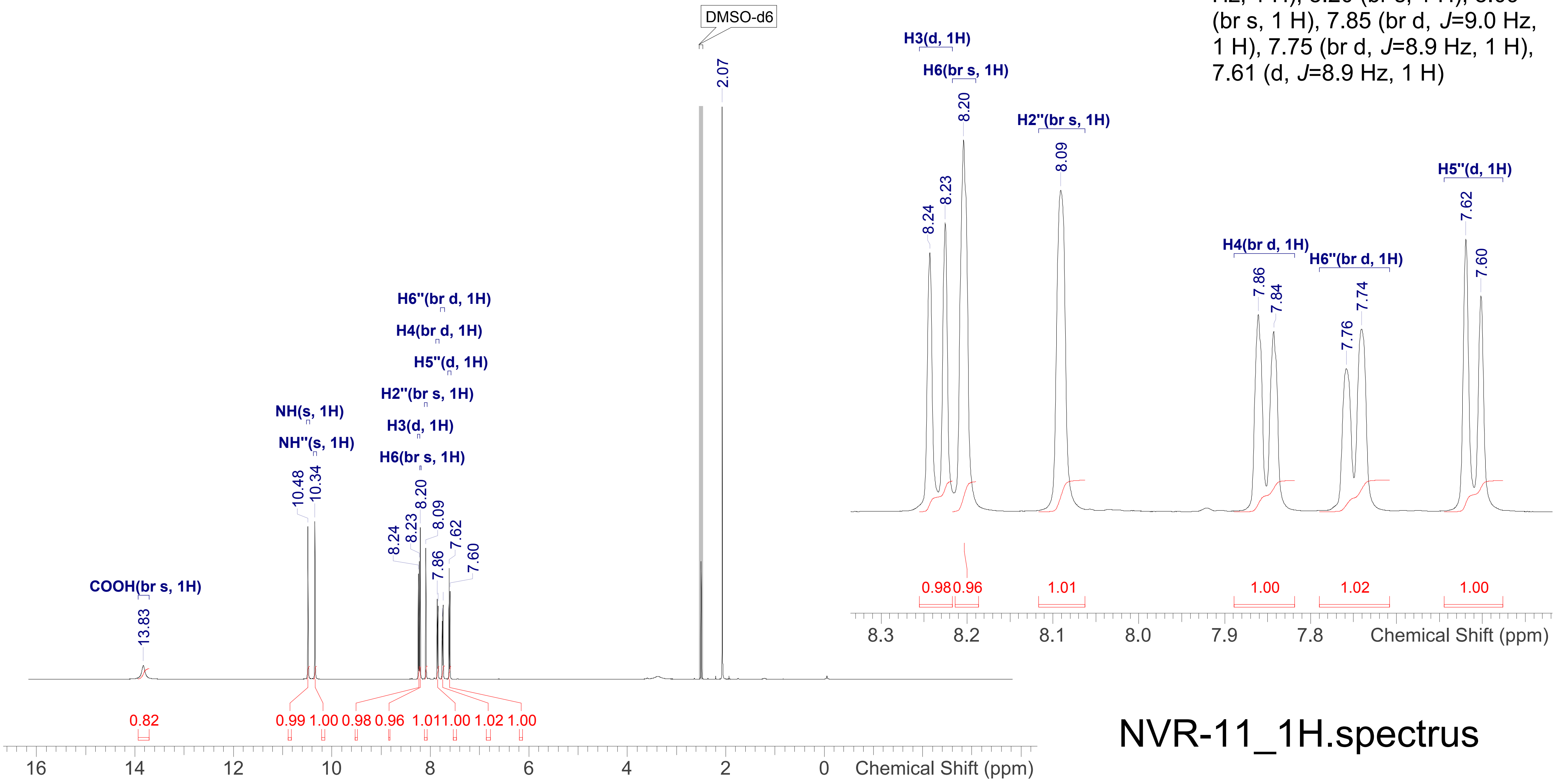

NVR-11_1H.spectrus

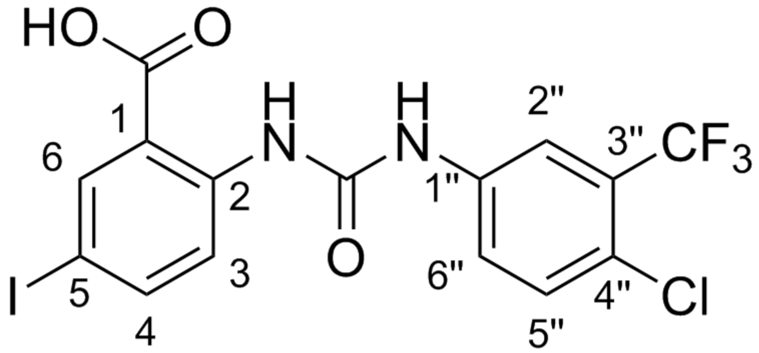

Shift (ppm)	C	m	J (Hz)	Assign
168.6	1	s	-	COOH
152.2	1	s	-	CO
141.3	1	s	-	4
140.2	1	s	-	1"
139.6	1	s	-	2
139.2	1	s	-	6
131.9	1	s	-	5"
126.6	1	q	30.5	3"
123.1	2	s	-	3, 4"
122.8	1	q	272.9	CF3
122.3	1	s	-	1
121.5	1	s	-	6"
117.1	1	q	5.4	2"
83.7	1	s	-	5

Acquisition Time (sec)	2.0447
Date	02/11/2017 14:47:00
Date Stamp	02/11/2017 14:47:00
Frequency (MHz)	125.7870
Nucleus	13C
Number of Transients	64
Solvent	DMSO-d6

^{13}C NMR (126 MHz, *DMSO-d_6*) δ ppm 168.6 (s, 1 C), 152.2 (s, 1 C), 141.3 (s, 1 C), 140.2 (s, 1 C), 139.6 (s, 1 C), 139.2 (s, 1 C), 131.9 (s, 1 C), 126.6 (q, *J*=30.5 Hz, 1 C), 123.1 (s, 2 C), 122.3 (s, 1 C), 121.5 (s, 1 C), 122.8 (q, *J*=272.9 Hz, 1 C), 117.1 (q, *J*=5.4 Hz, 1 C), 83.7 (s, 1 C)

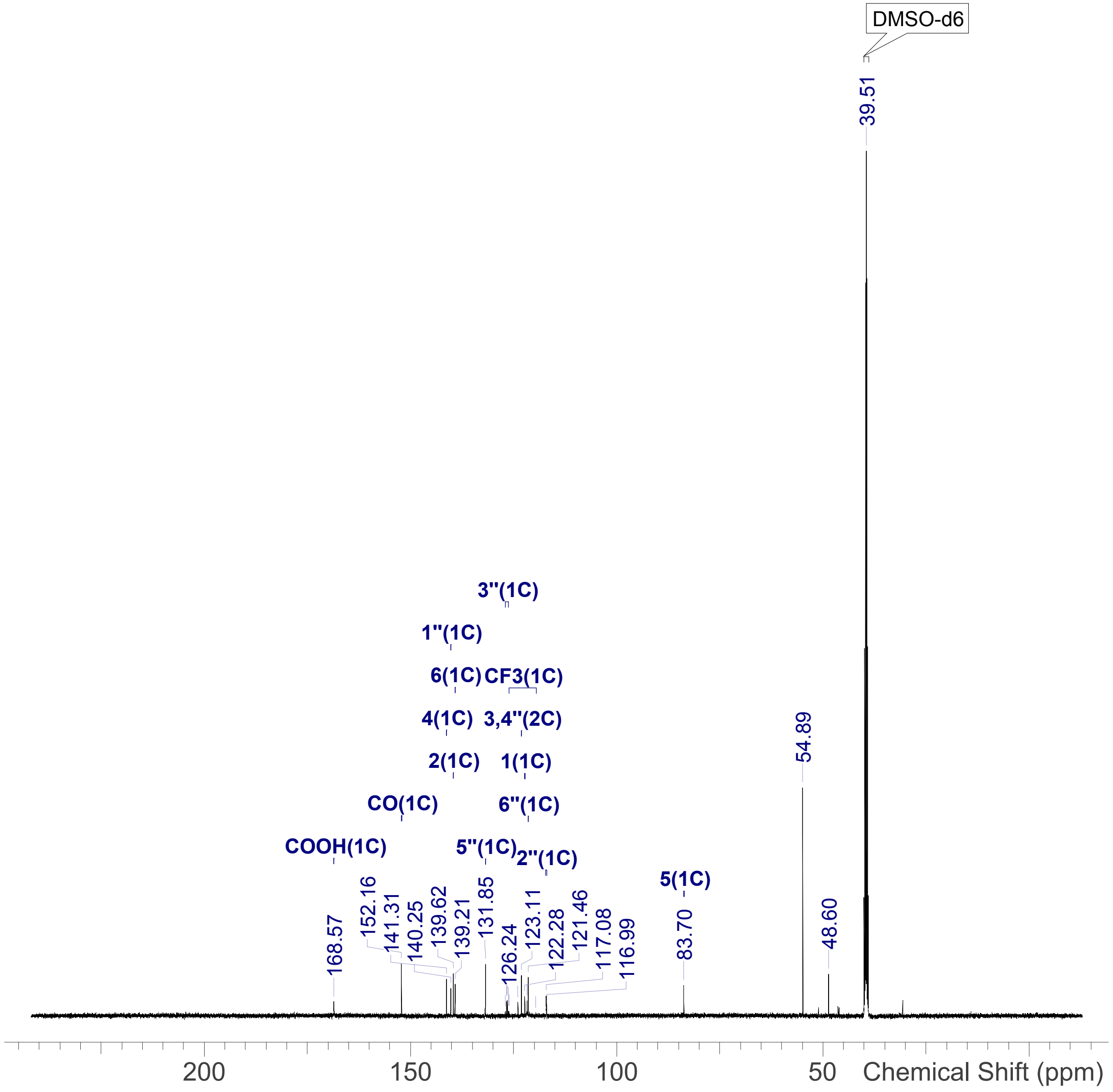

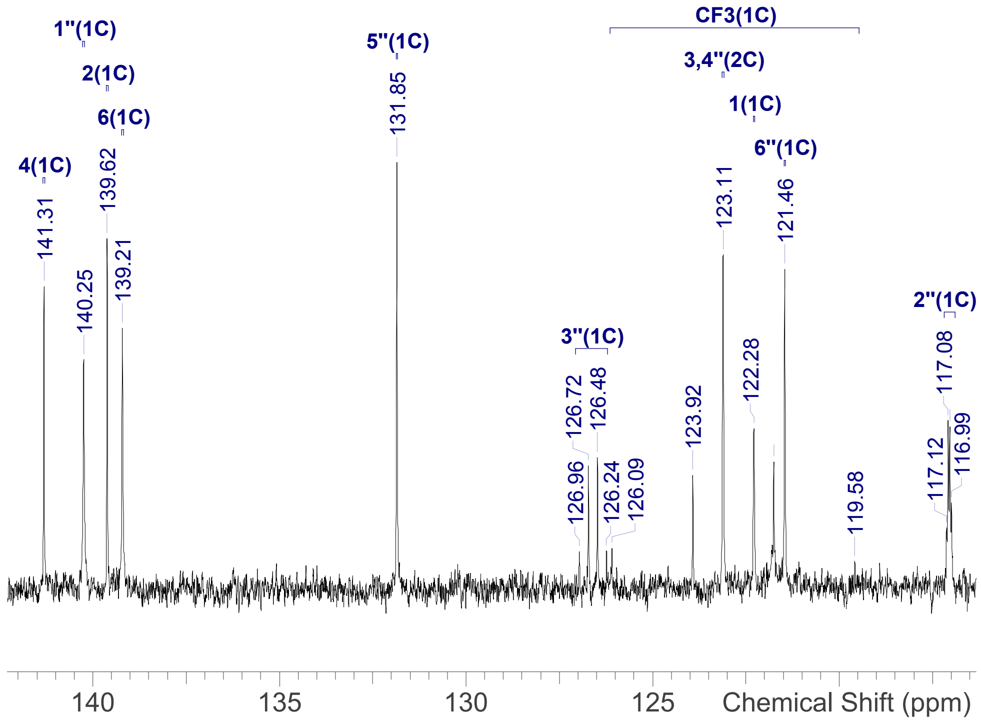

NVR-11_13C.spectrus

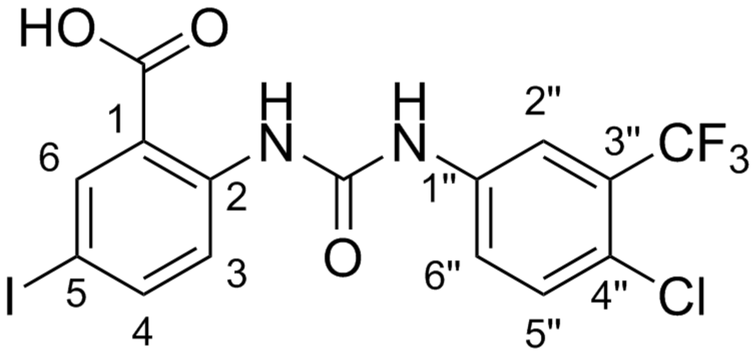

Shift (ppm)	F	m
-61.51	3	s

Acquisition Time (sec)	2.9360
Date	02/11/2017 14:54:00
Date Stamp	02/11/2017 14:54:00
Frequency (MHz)	470.6020
Nucleus	19F
Number of Transients	16
Solvent	DMSO-d6

^{19}F NMR (471 MHz, *DMSO-d_6*) δ ppm -61.51 (s, 3 F)

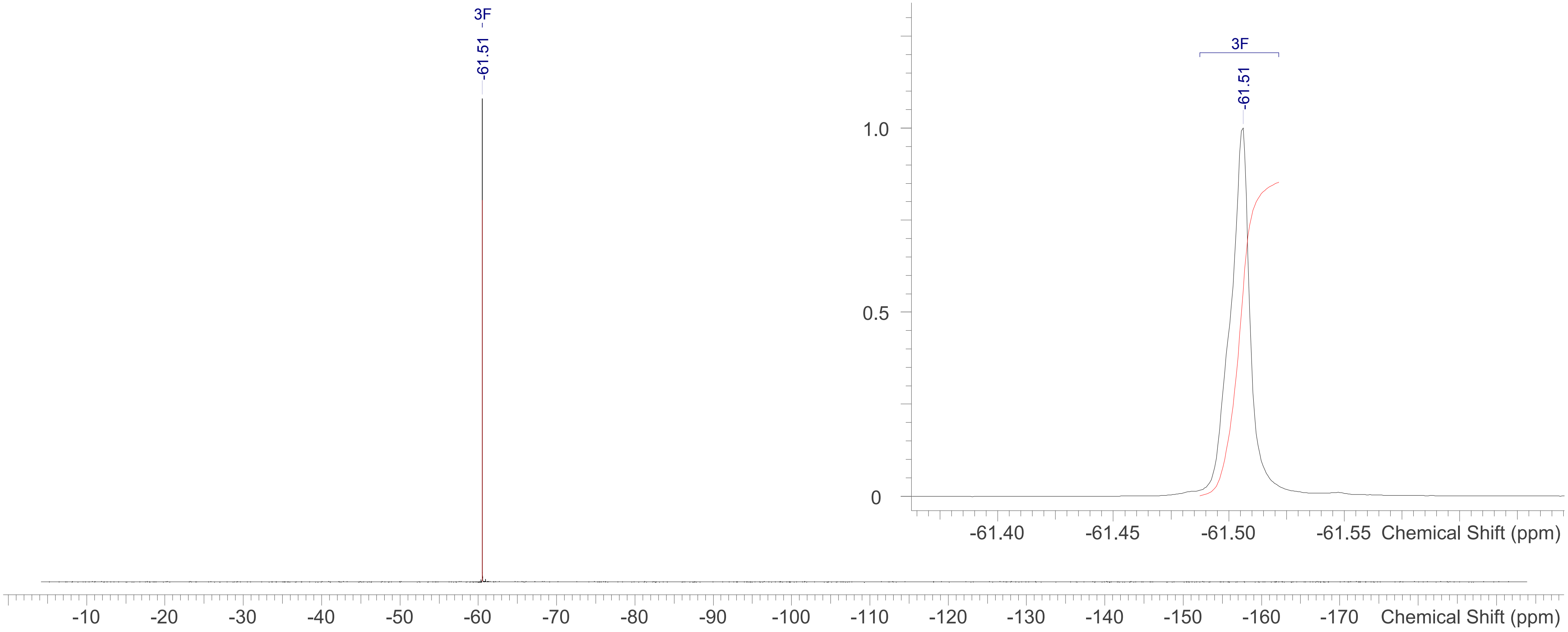

NVR-11_19F.spectrus

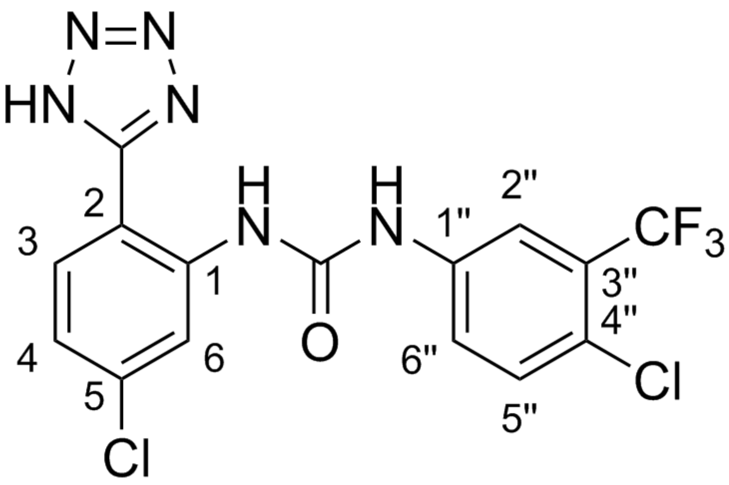

Shift (ppm)	H	m	J (Hz)	Assign
10.34	1	br s	-	NH
8.57	1	s	-	NH''
8.46	1	br s	-	H6
8.04	1	s	-	H2''
7.83	1	br d	8.4	H3
7.70	1	br d	8.7	H6''
7.50	1	br d	8.7	H5''
7.21	1	br d	8.5	H4

Acquisition Time (sec)	6.5536
Date	23/11/2017 15:51:00
Date Stamp	23/11/2017 15:51:00
Frequency (MHz)	500.1930
Nucleus	1H
Number of Transients	16
Solvent	ACETONITRILE-d3

^{1}H NMR (500 MHz, *ACETONITRILE-d$_3$*) δ ppm 10.34 (br s, 1 H), 8.57 (s, 1 H), 8.46 (br s, 1 H), 8.04 (s, 1 H), 7.83 (br d, *J*=8.4 Hz, 1 H), 7.70 (br d, *J*=8.7 Hz, 1 H), 7.50 (br d, *J*=8.7 Hz, 1 H), 7.21 (br d, *J*=8.5 Hz, 1 H)

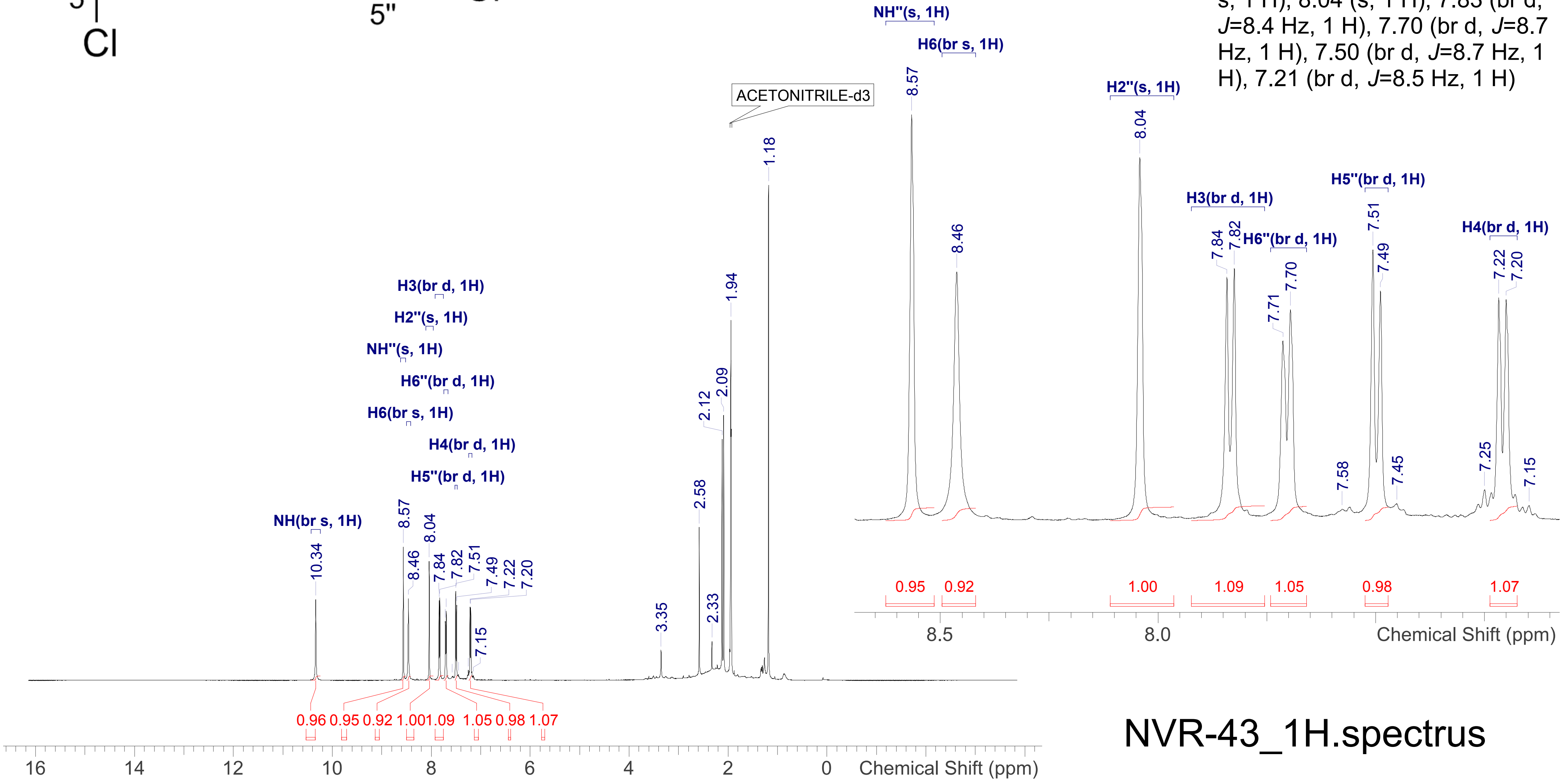

NVR-43_1H.spectrus

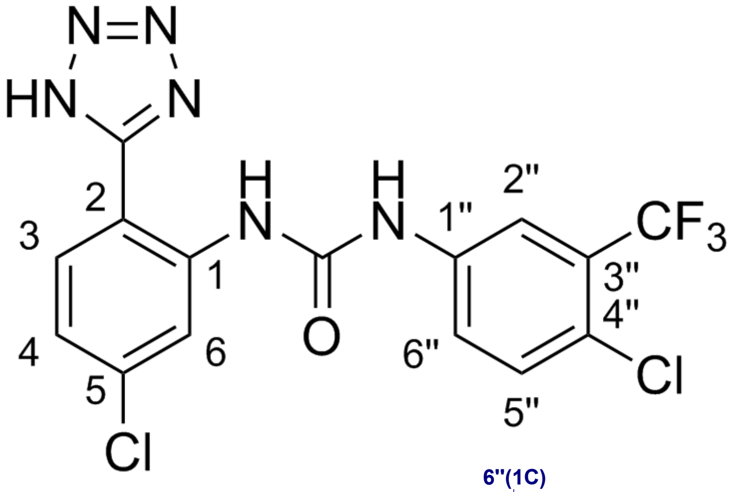

Shift (ppm)	C	m	J (Hz)	Assign
155.3	1	s	-	Tet-C
153.1	1	s	-	CO
140.6	1	s	-	1
139.8	1	s	-	1''
138.3	1	s	-	5
132.9	1	s	-	5''
130.7	1	s	-	3
124.6	1	s	-	6
123.5	1	s	-	4
121.4	1	s	-	6''
119.0	1	q	6.0	2''
110.8	1	s	-	2

Acquisition Time (sec)	2.0447
Date	23/11/2017 15:57:00
Date Stamp	23/11/2017 15:57:00
Frequency (MHz)	125.7870
Nucleus	13C
Number of Transients	64
Solvent	ACETONITRILE-d3

^{13}C NMR (126 MHz, *ACETONITRILE-d_3*) δ ppm 155.3 (s, 1 C), 153.1 (s, 1 C), 140.6 (s, 1 C), 139.8 (s, 1 C), 138.3 (s, 1 C), 132.9 (s, 1 C), 130.7 (s, 1 C), 124.6 (s, 1 C), 123.5 (s, 1 C), 121.4 (s, 1 C), 119.0 (q, *J*=6.0 Hz, 1 C), 110.8 (s, 1 C)

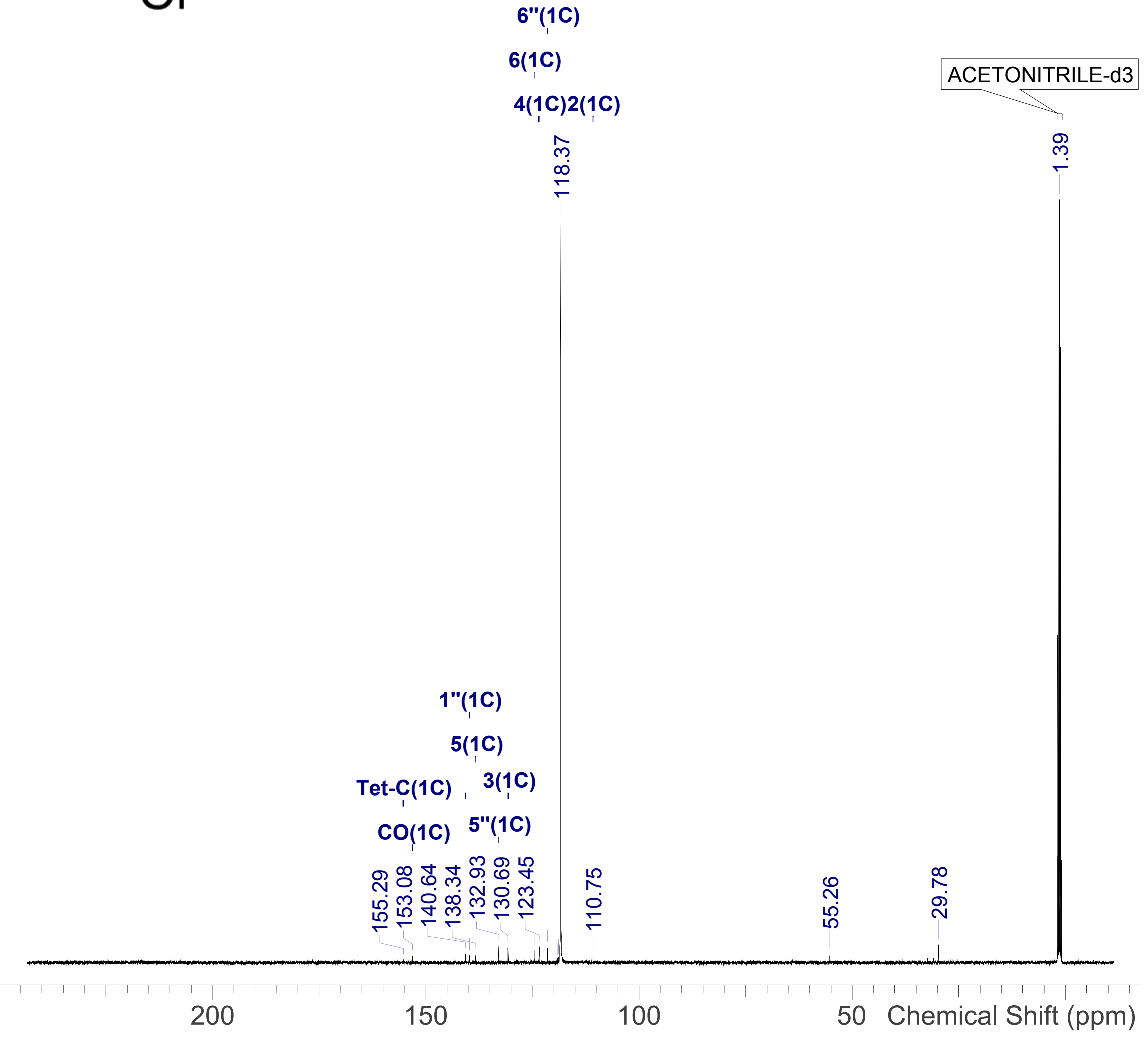

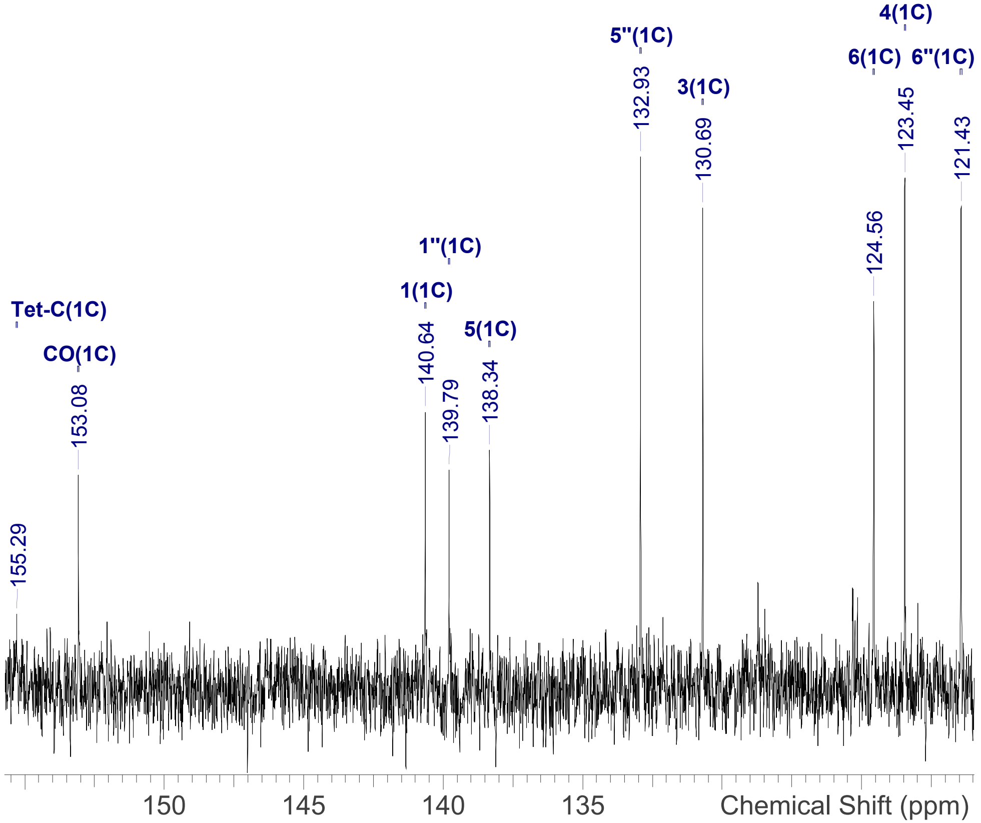

NVR-43_13C.spectrus

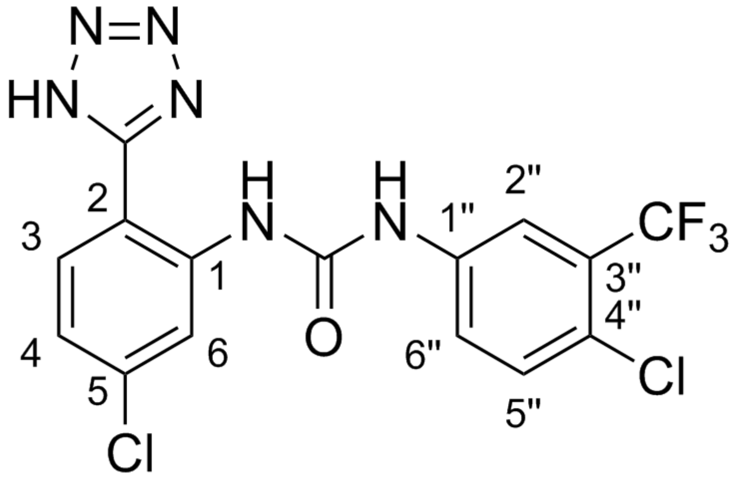

Shift (ppm)	F	m
-63.22	3	s

Acquisition Time (sec)	2.9360
Date	23/11/2017 16:06:00
Date Stamp	23/11/2017 16:06:00
Frequency (MHz)	470.6020
Nucleus	19F
Number of Transients	16
Solvent	ACETONITRILE-d3

^{19}F NMR (471 MHz, *ACETONITRILE-d_3*) δ ppm -63.22 (s, 3 F)

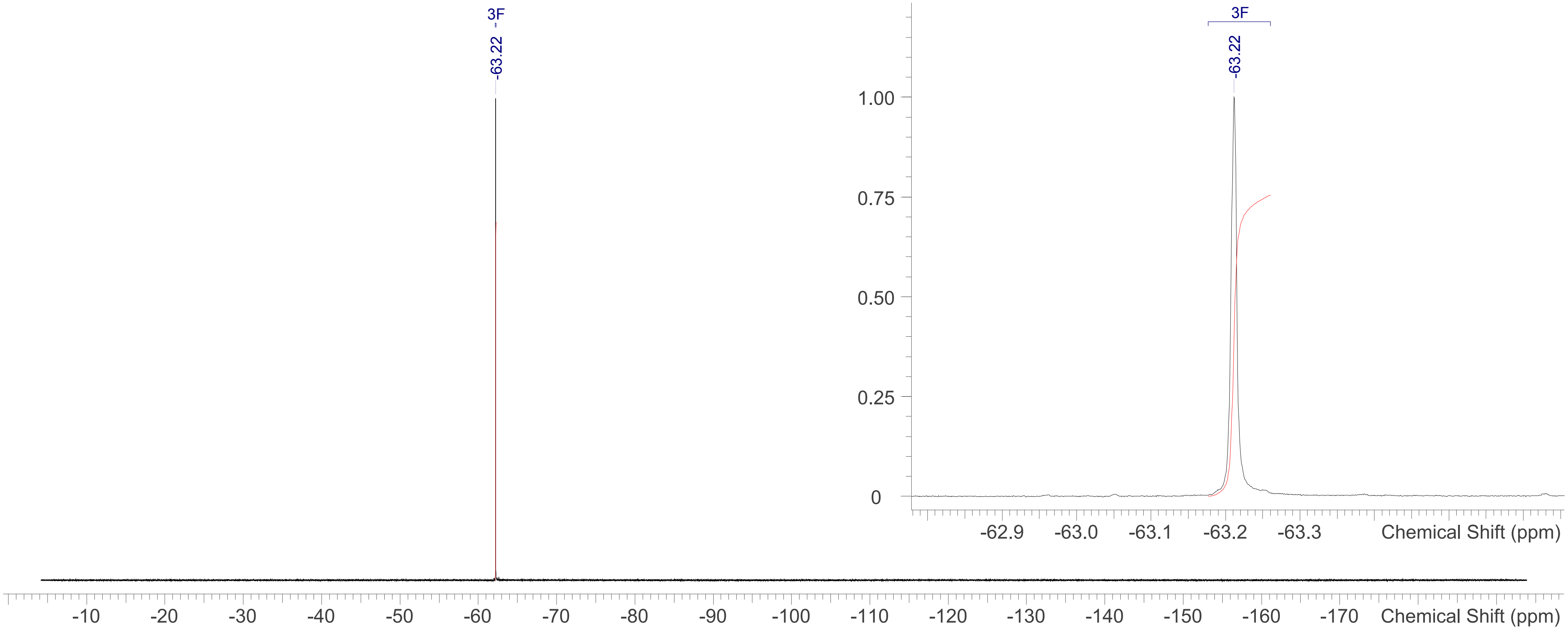

NVR-43_19F.spectrus

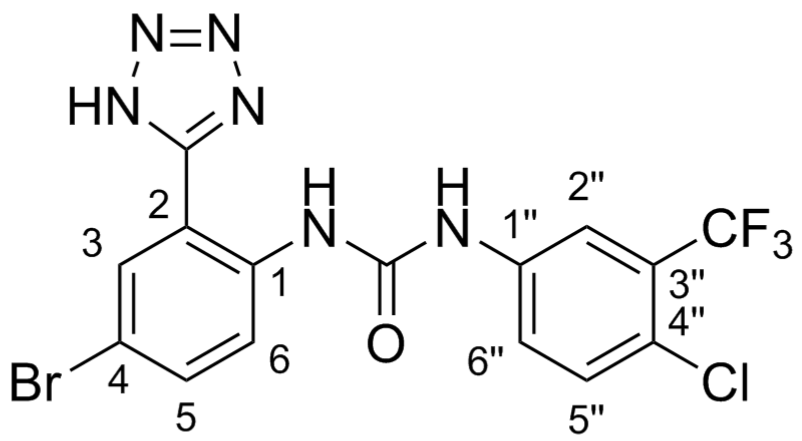

Shift (ppm)	H	m	J (Hz)	Assign
10.34	1	s	-	NH"
10.00	1	s	-	NH
8.27	1	d	9.0	H6
8.15	1	br d	1.7	H3
8.11	1	br s	-	H2"
7.75	1	br d	8.5	H6"
7.72	1	br d	8.9	H5
7.63	1	br d	8.7	H5"

Acquisition Time (sec)	6.5536
Date	10/04/2018 12:02:00
Date Stamp	10/04/2018 12:02:00
Frequency (MHz)	500.1930
Nucleus	1H
Number of Transients	16
Solvent	DMSO-d6

^{1}H NMR (500 MHz, *DMSO-d_6*) δ ppm 10.34 (s, 1 H), 10.00 (s, 1 H), 8.27 (d, *J*=9.0 Hz, 1 H), 8.15 (br d, *J*=1.7 Hz, 1 H), 8.11 (br s, 1 H), 7.75 (br d, *J*=8.5 Hz, 1 H), 7.72 (br d, *J*=8.9 Hz, 1 H), 7.63 (br d, *J*=8.7 Hz, 1 H)

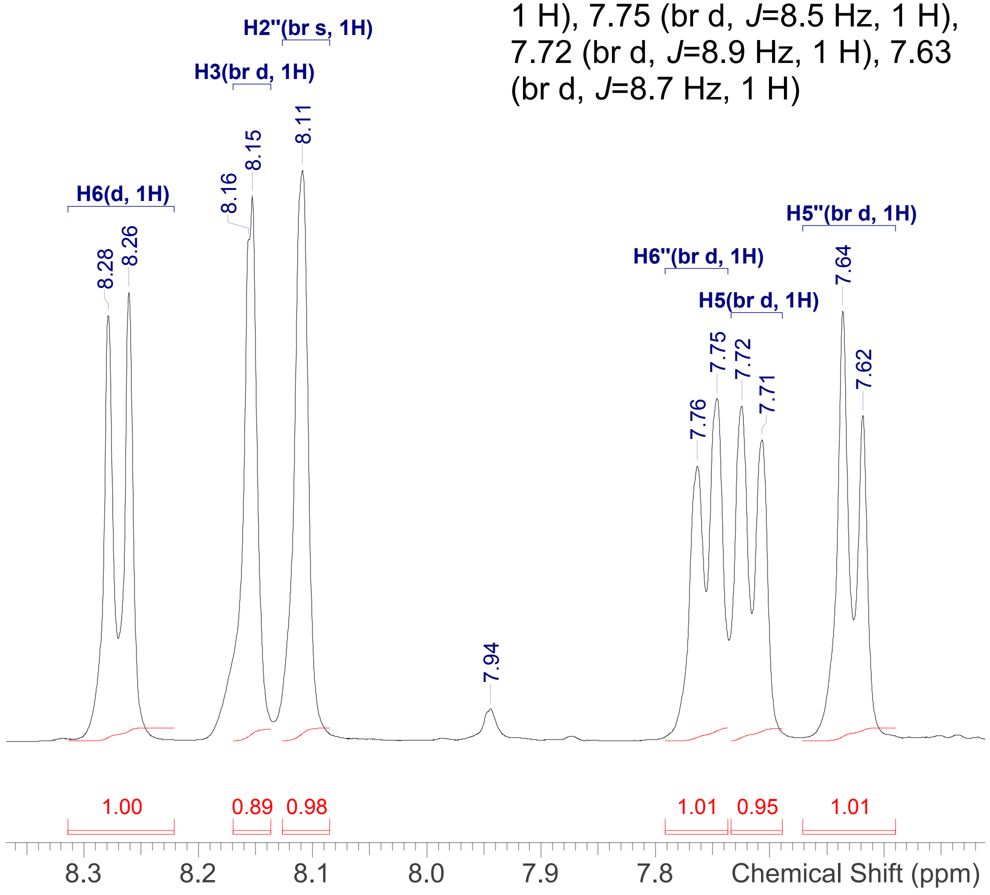

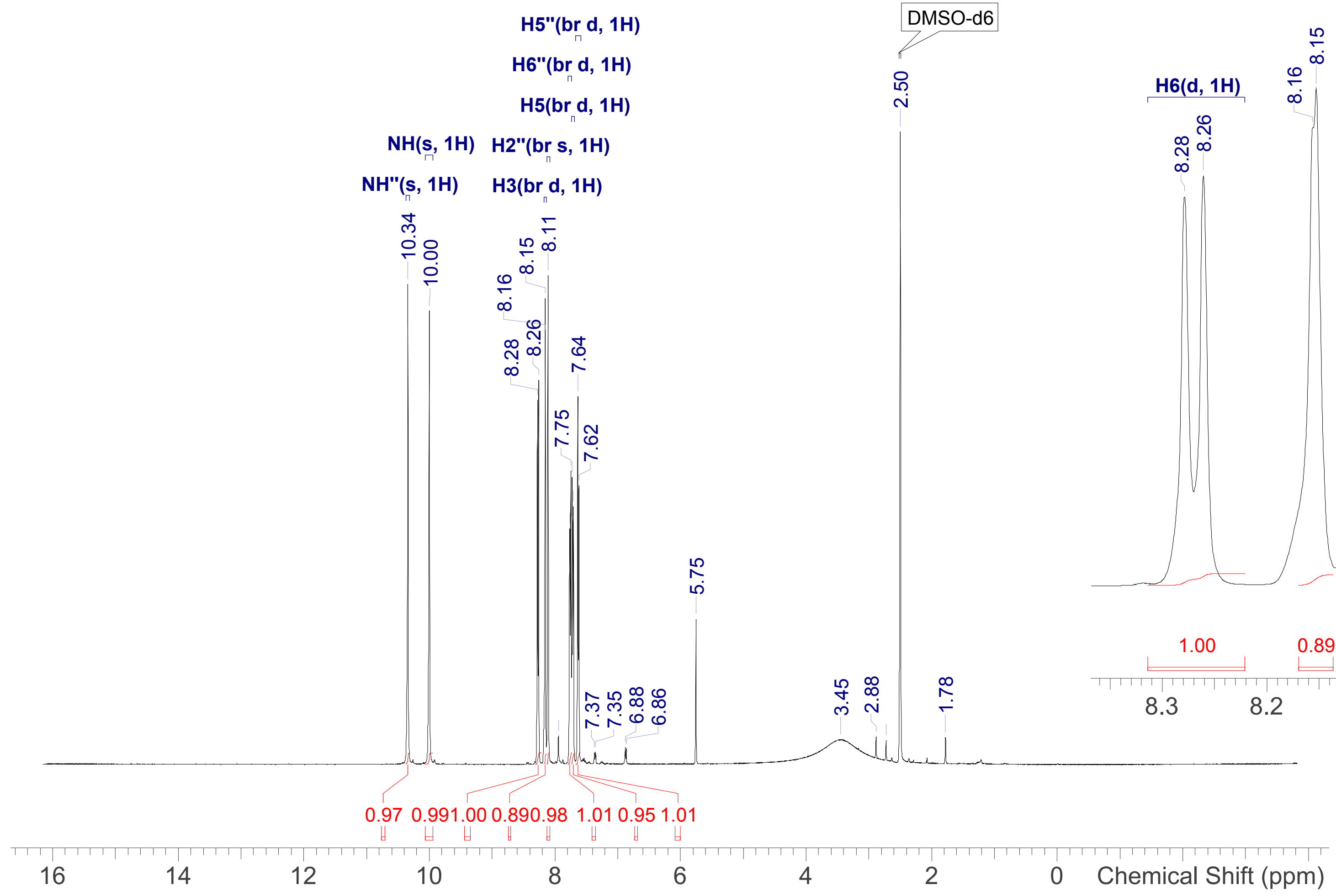

NVR-44_1H.spectrus

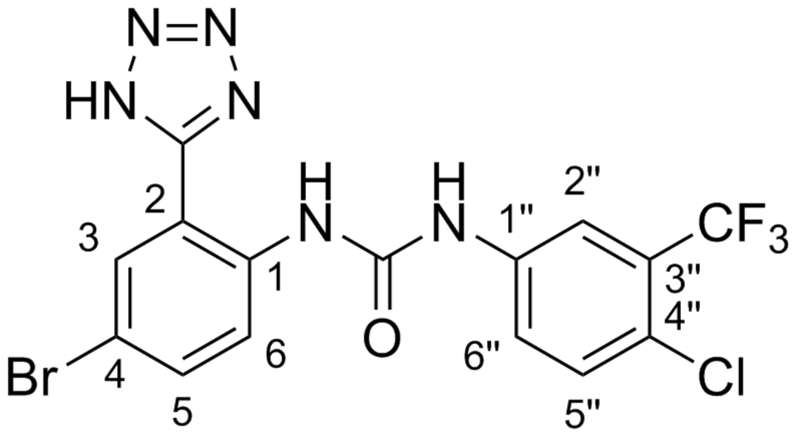

Shift (ppm)	C	m	J (Hz)	Assign
152.0	1	s	-	CO
139.2	1	s	-	1"
137.0	1	s	-	1
134.0	1	s	-	3
132.0	1	s	-	6"
130.8	1	s	-	5"
126.7	1	q	30.3	3"
123.4	1	s	-	6
123.3	1	s	-	5
122.8	1	q	272.9	CF3
122.7	1	br q	1.5	4"
117.2	1	q	6.2	2"
114.1	1	s	-	4
114.0	1	s	-	2

Acquisition Time (sec)	2.0447
Date	10/04/2018 12:07:00
Date Stamp	10/04/2018 12:07:00
Frequency (MHz)	125.7870
Nucleus	13C
Number of Transients	64
Solvent	DMSO-d6

^{13}C NMR (126 MHz, *DMSO-d_6*) δ ppm 152.0 (s, 1 C), 139.2 (s, 1 C), 137.0 (s, 1 C), 134.0 (s, 1 C), 132.0 (s, 1 C), 130.8 (s, 1 C), 126.7 (q, *J*=30.3 Hz, 1 C), 123.4 (s, 1 C), 123.3 (s, 1 C), 122.7 (br q, *J*=1.5 Hz, 1 C), 122.8 (q, *J*=272.9 Hz, 1 C), 117.2 (q, *J*=6.2 Hz, 1 C), 114.1 (s, 1 C), 114.0 (s, 1 C)

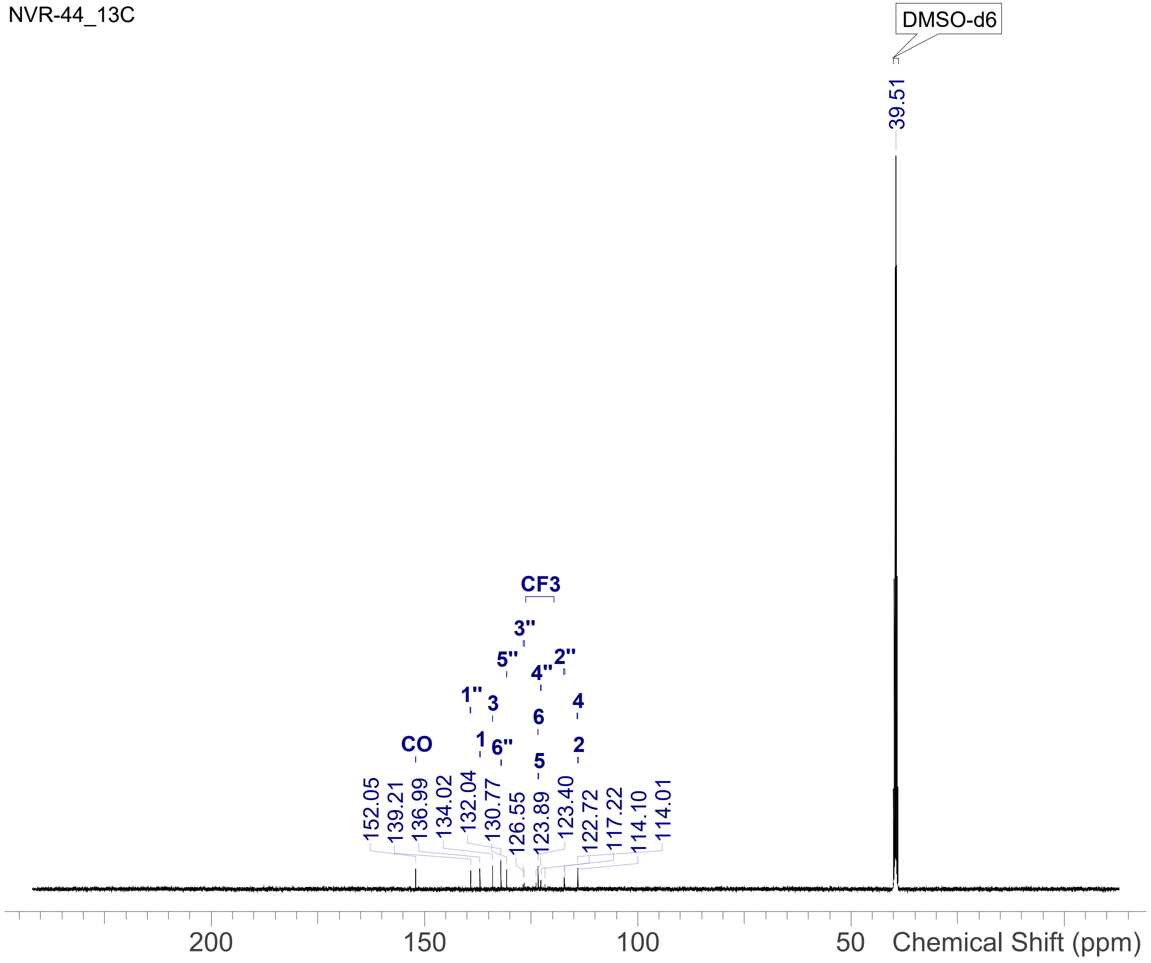

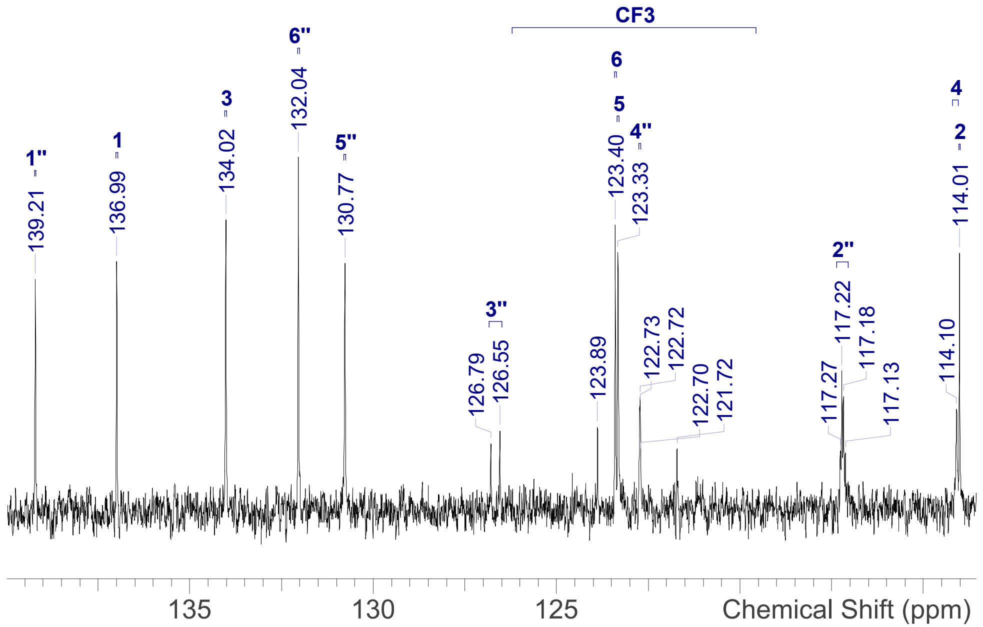

NVR-44_13C.spectrus

NVR-114 HRMS

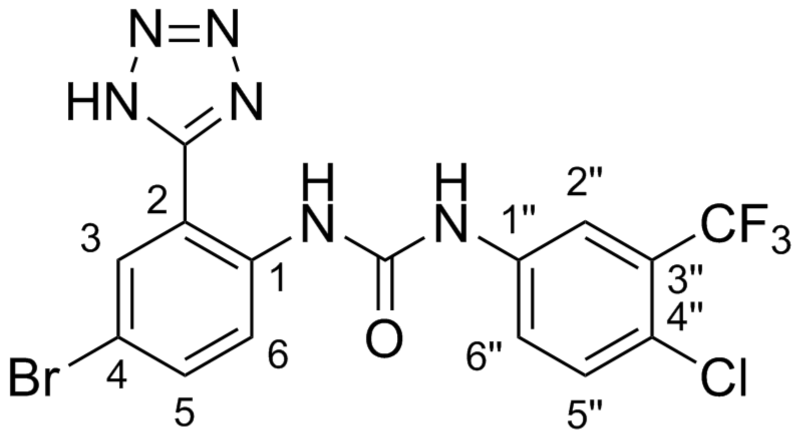

Shift (ppm)	F	m
-64.10	3	s

Acquisition Time (sec)	2.9360
Date	14/11/2017 15:08:00
Date Stamp	14/11/2017 15:08:00
Frequency (MHz)	470.6020
Nucleus	19F
Number of Transients	16
Solvent	METHANOL-d4

^{19}F NMR (471 MHz, *METHANOL-d_4*) δ ppm -64.10 (s, 3 F)

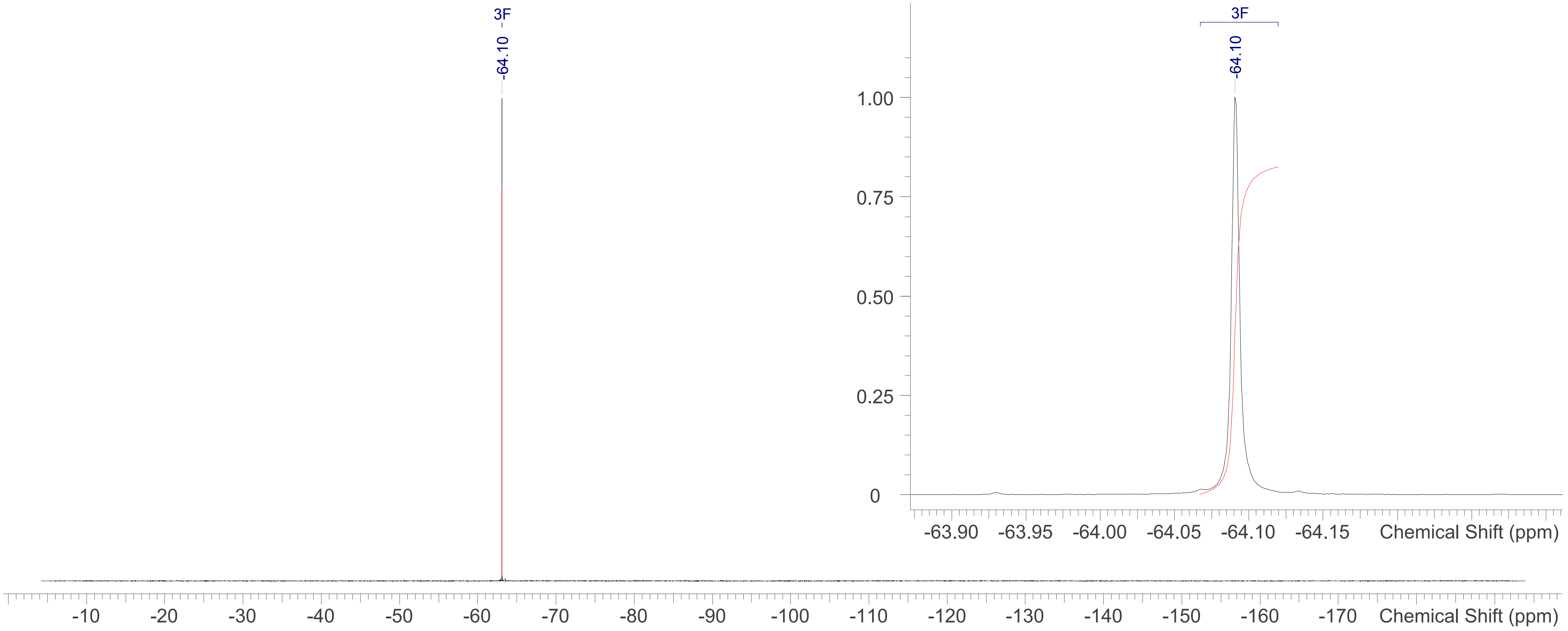

NVR-44_19F.spc

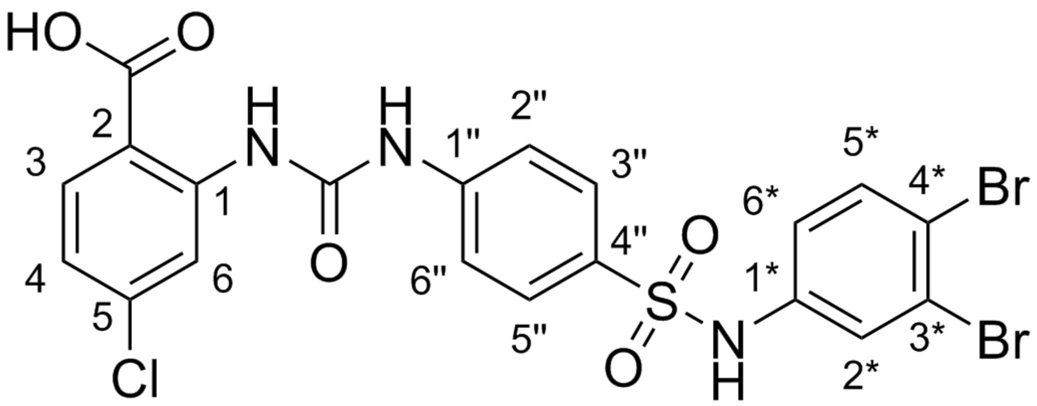

Shift (ppm)	H	m	J (Hz)	Assign
10.75	2	br s	-	NH, NH''
10.40	1	br s	-	NH*
8.48	1	br d	1.6	H6
7.96	1	br d	8.7	H3
7.70	4	m	-	H3'', H5'', H2'', H6''
7.60	1	br d	8.7	H5*
7.50	1	br d	1.6	H2*
7.38	1	d	6.3	H4
7.12	1	dd	8.1, 1.5	H6*

Acquisition Time (sec)	8.1920
Date	21/12/2017 12:28:00
Date Stamp	21/12/2017 12:28:00
Frequency (MHz)	400.1320
Nucleus	1H
Number of Transients	16
Solvent	DMSO-d6

^{1}H NMR (400 MHz, *DMSO-d_6*) δ ppm 10.75 (br s, 2 H), 10.40 (br s, 1 H), 8.48 (br d, *J*=1.6 Hz, 1 H), 7.96 (br d, *J*=8.7 Hz, 1 H), 7.65 - 7.77 (m, 4 H), 7.60 (br d, *J*=8.7 Hz, 1 H), 7.50 (br d, *J*=1.6 Hz, 1 H), 7.38 (d, *J*=6.3 Hz, 1 H), 7.12 (dd, *J*=8.1, 1.5 Hz, 1 H)

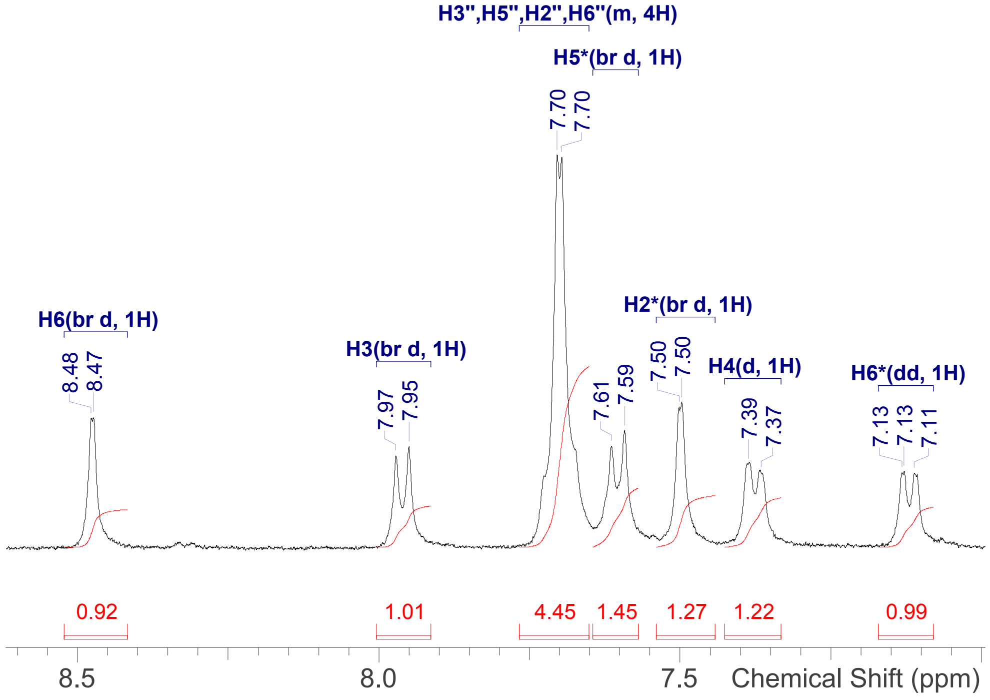

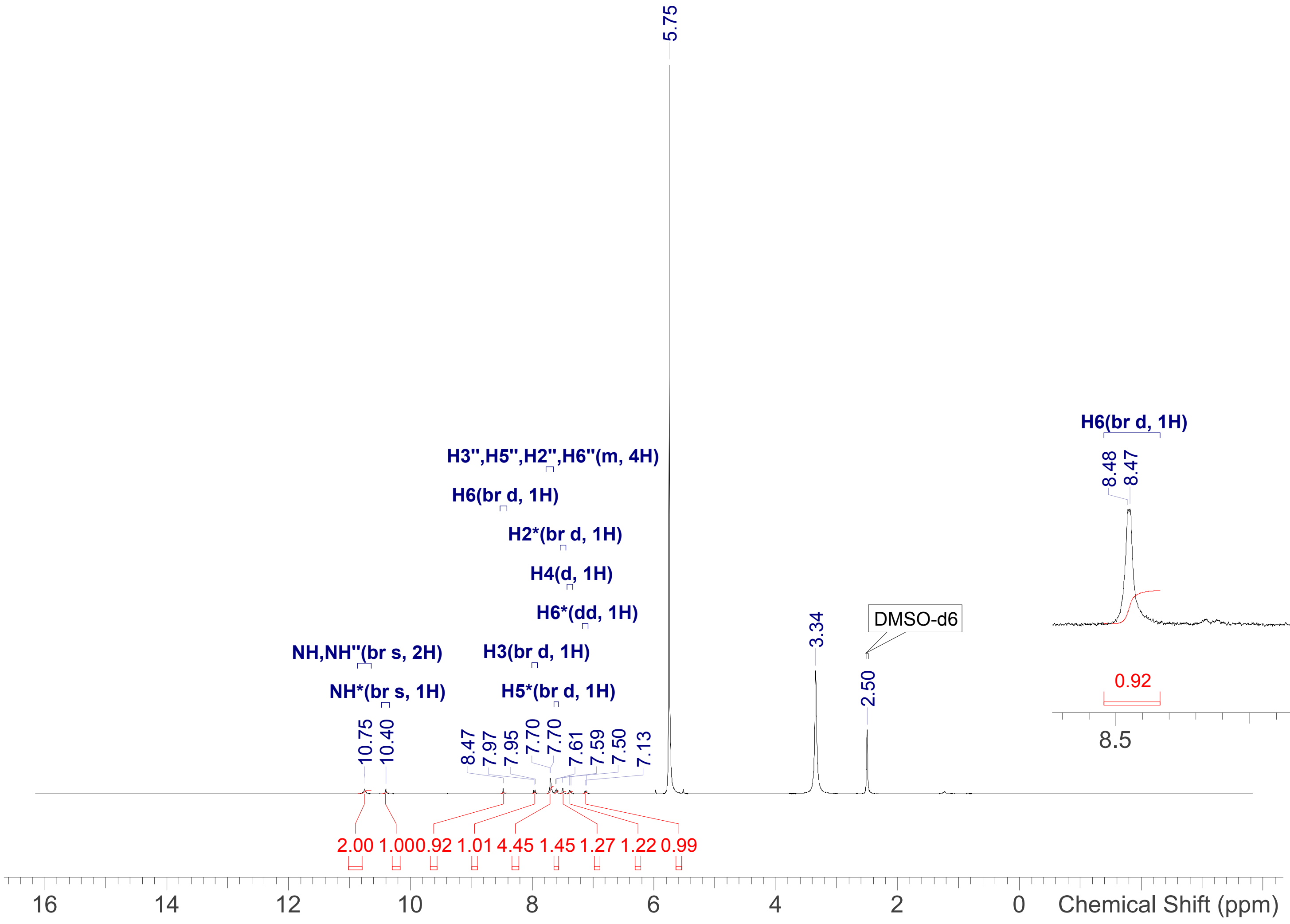

NVR-47_1H.spectrus

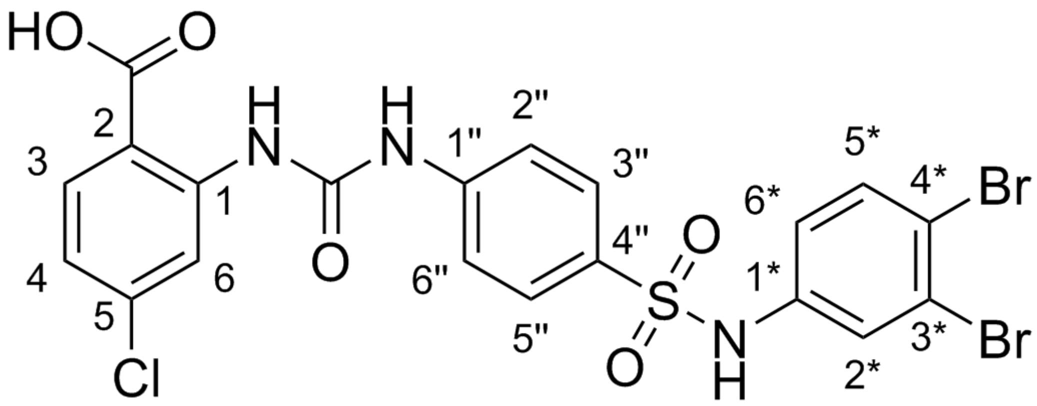

Shift (ppm)	C	m	Assign
168.9	1	s	COOH
152.4	1	s	CO
144.0	1	s	5
142.8	1	s	1
138.7	1	s	1*
138.4	1	s	1''
138.2	1	s	4''
134.2	1	s	5*
133.1	2	s	3'', 5''
131.5	1	s	3
124.1	1	s	4
123.7	1	s	3*
121.3	1	s	6
120.0	1	s	6*
118.4	1	s	2*
115.0	2	s	2'', 6''
114.5	1	s	4*
108.5	1	s	2

Acquisition Time (sec)	2.0447
Date	12/04/2018 09:36:00
Date Stamp	12/04/2018 09:36:00
Frequency (MHz)	125.7870
Nucleus	13C
Number of Transients	64
Solvent	DMSO-d6

^{13}C NMR (126 MHz, *DMSO-d_6*) δ ppm 168.9 (s, 1 C), 152.4 (s, 1 C), 144.0 (s, 1 C), 142.8 (s, 1 C), 138.7 (s, 1 C), 138.4 (s, 1 C), 138.2 (s, 1 C), 134.2 (s, 1 C), 133.1 (s, 2 C), 131.5 (s, 1 C), 124.1 (s, 1 C), 123.7 (s, 1 C), 121.3 (s, 1 C), 120.0 (s, 1 C), 118.4 (s, 1 C), 115.0 (s, 2 C), 114.5 (s, 1 C), 108.5 (s, 1 C)

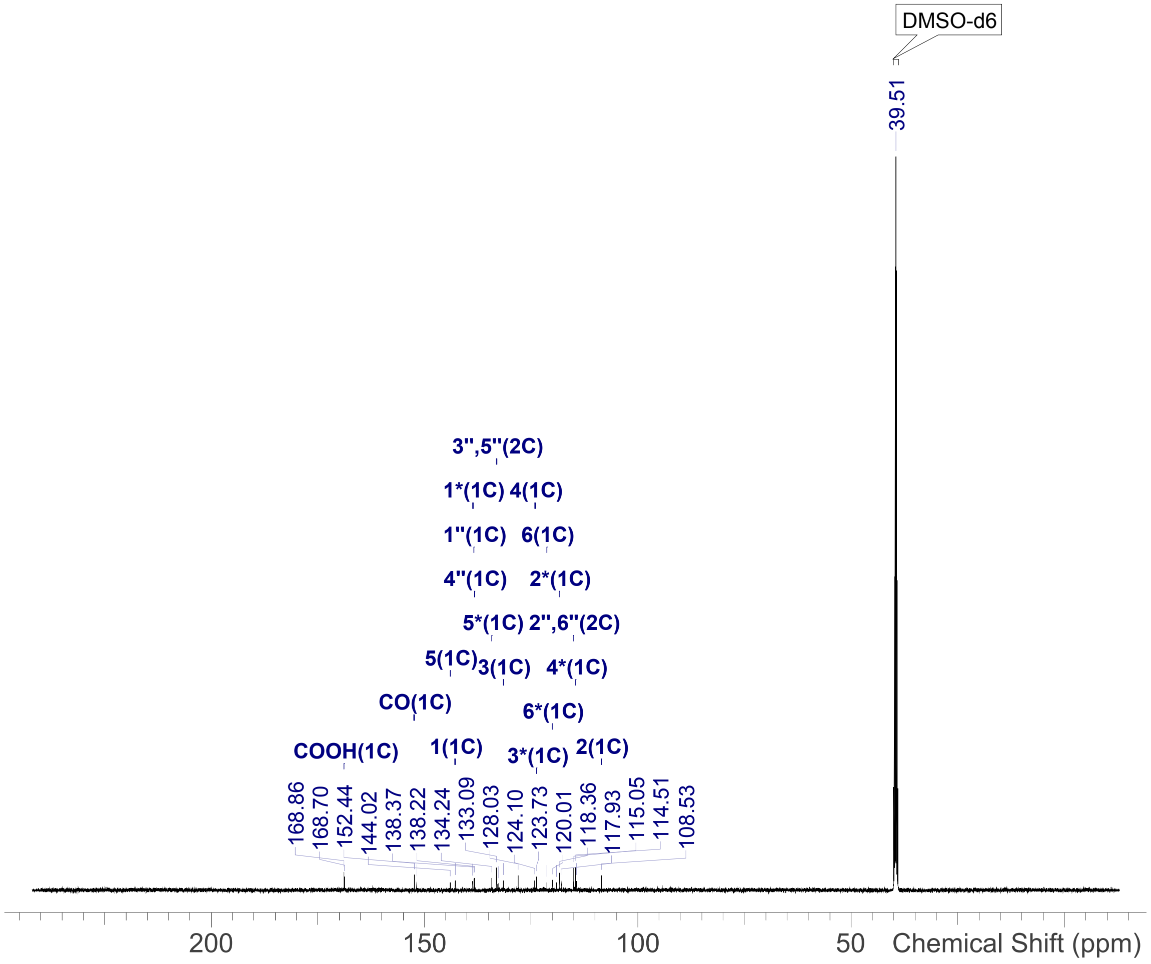

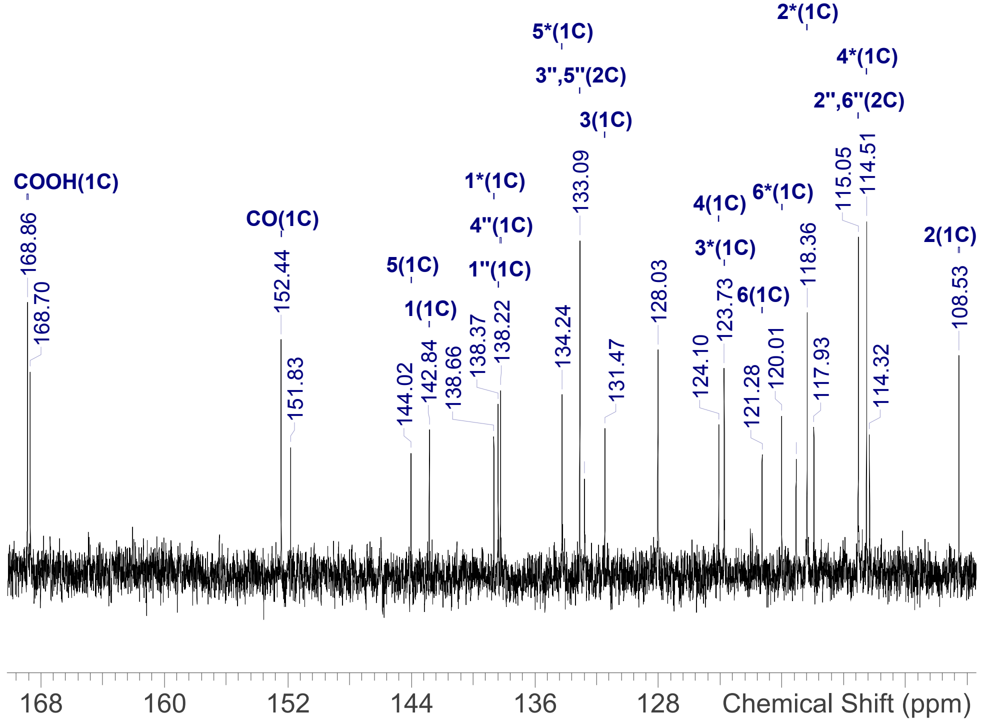

NVR-47_13C.spectrus

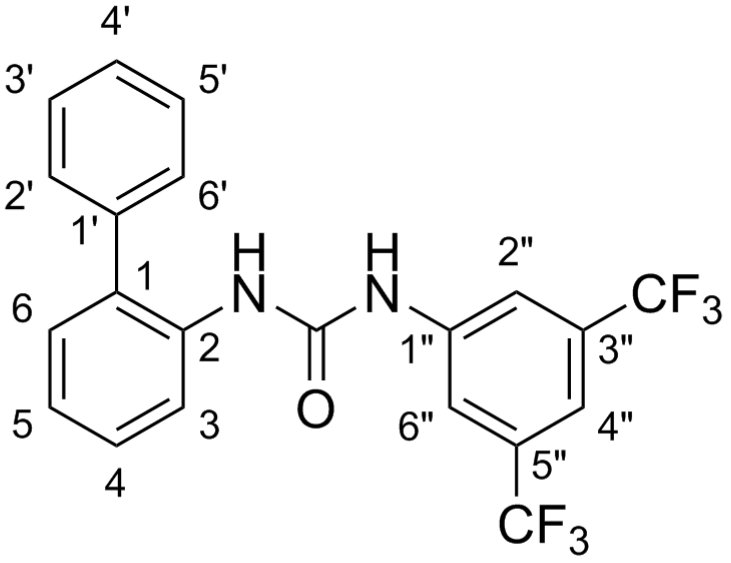

No.	Shift (ppm)	H	m	J (Hz)	Assign
1	9.67	1	br s	-	NH"
2	8.02	2	br s	-	6", 2"
3	7.88	1	s	-	NH
4	7.87	1	dd	8.3, 1.2	3
5	7.61	1	br s	-	4"
6	7.49	2	br t	7.5	3', 5'
7	7.42	3	m	-	4', 2', 6'
8	7.36	1	ddd	8.4, 6.9, 1.8	4
9	7.26	1	dd	7.5, 1.8	6
10	7.20	1	td	7.3, 1.3	5

Acquisition Time (sec)	3.9846
Date	05 Mar 2019 05:26:06
Date Stamp	05 Mar 2019 05:26:06
Frequency (MHz)	400.0700
Nucleus	1H
Number of Transients	16
Solvent	DMSO-d6
Temperature (degree C)	24.998

^{1}H NMR (400 MHz, *DMSO-d$_6$*) δ ppm 9.67 (br s, 1 H), 8.02 (br s, 2 H), 7.88 (s, 1 H), 7.87 (dd, *J*=8.3, 1.2 Hz, 1 H), 7.61 (br s, 1 H), 7.49 (br t, *J*=7.5 Hz, 2 H), 7.39 - 7.45 (m, 3 H), 7.36 (ddd, *J*=8.4, 6.9, 1.8 Hz, 1 H), 7.26 (dd, *J*=7.5, 1.8 Hz, 1 H), 7.20 (td, *J*=7.3, 1.3 Hz, 1 H)

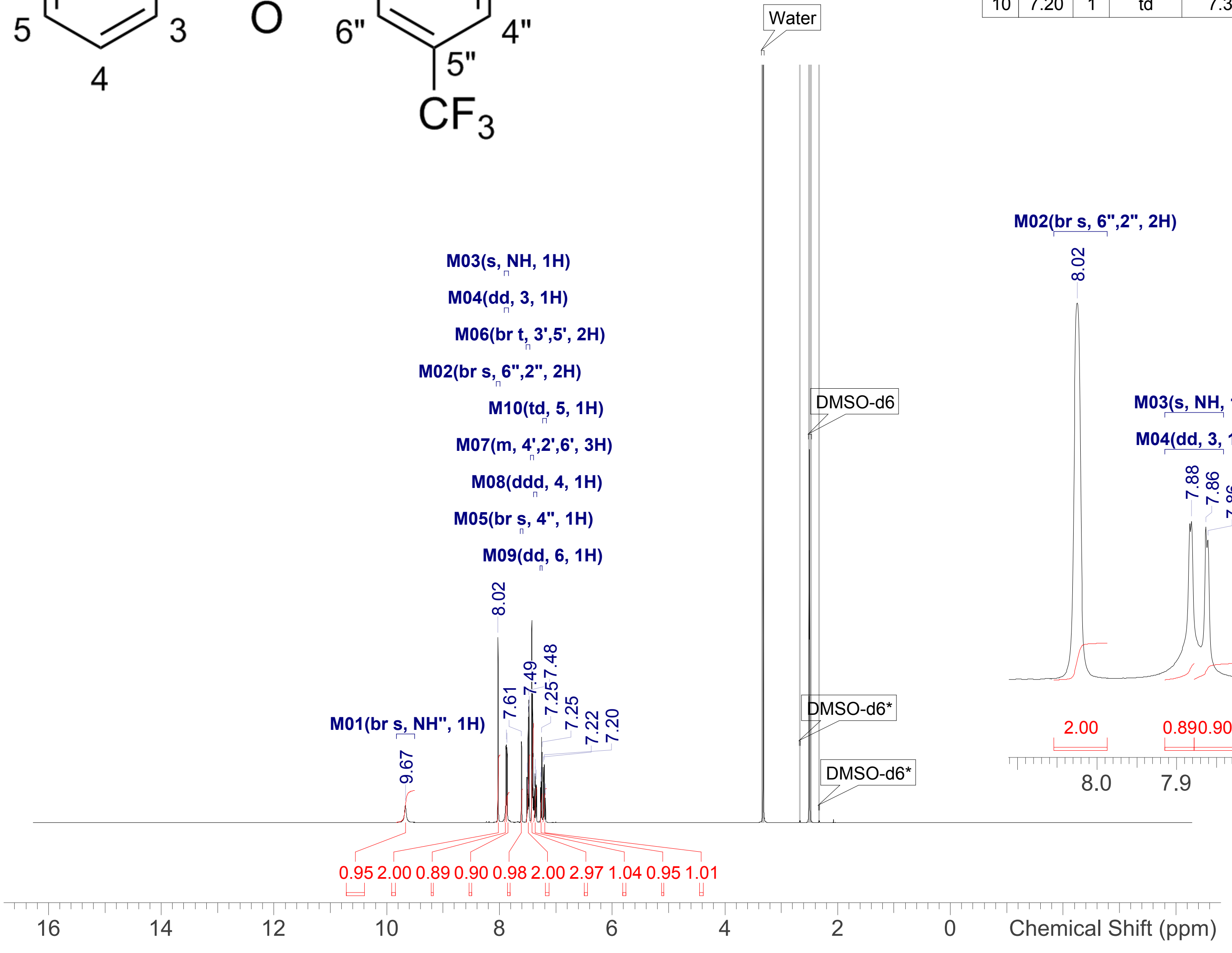

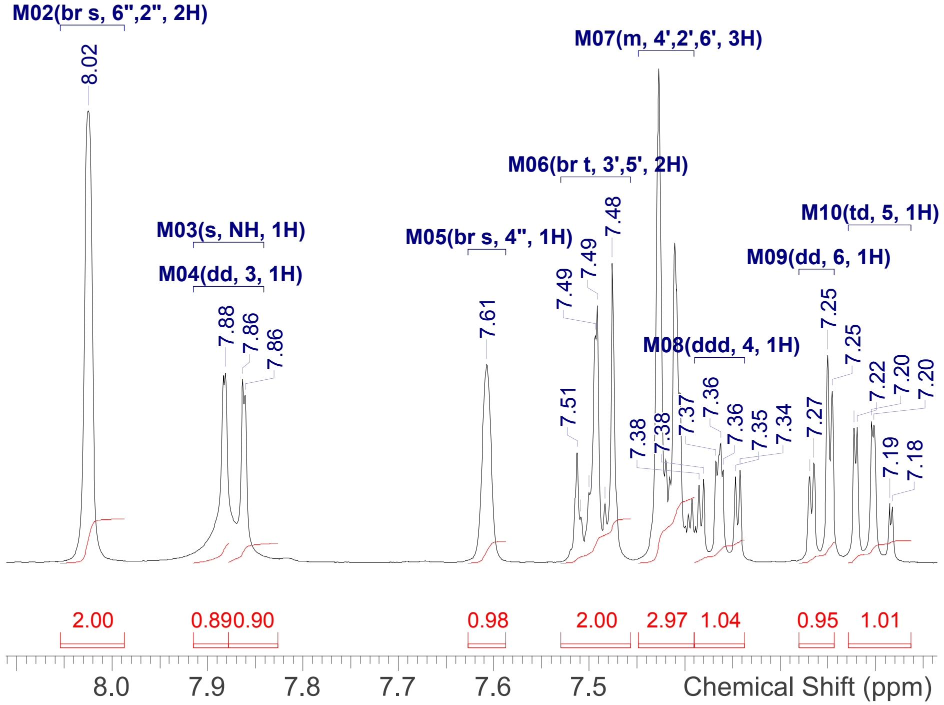

NVR-113_1H.spectrus

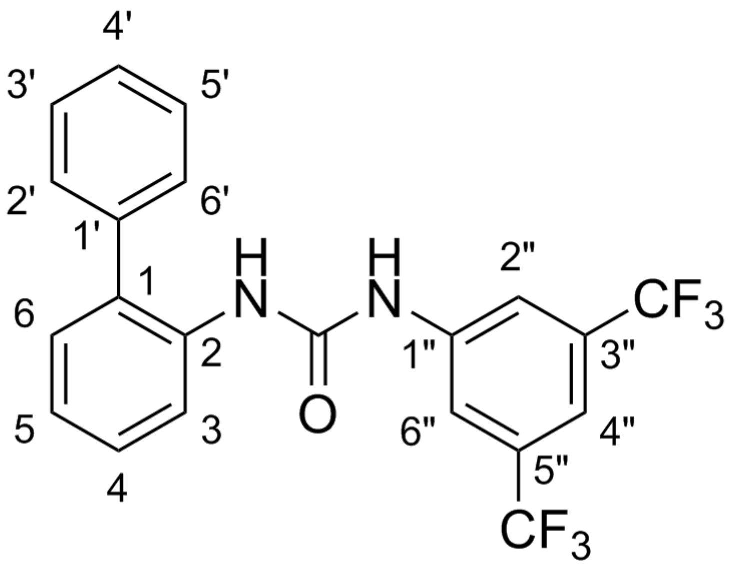

Shift (ppm)	C	m	J (Hz)	Assign
152.7	1	s	-	CO
141.9	1	s	-	1"
138.4	1	s	-	1'
134.9	1	s	-	2
133.8	1	s	-	1
130.7	2	q	32.6	5", 3"
130.4	1	s	-	6
129.1	2	s	-	2', 6'
128.8	2	s	-	3', 5'
127.9	1	s	-	4
127.5	1	s	-	4'
124.2	1	s	-	5
123.5	1	s	-	3
123.3	2	q	272.6	3"-CF3, 5"-CF3
117.6	2	br q	2.0	6", 2"
114.2	1	br spt	3.9	4"

Acquisition Time (sec)	1.0224
Date	28 Feb 2019 00:12:54
Date Stamp	28 Feb 2019 00:12:54
Frequency (MHz)	100.5977
Nucleus	13C
Number of Transients	256
Solvent	DMSO-d6
Temperature (degree C)	25.001

^{13}C NMR (101 MHz, *DMSO-d_6*) δ ppm 152.7 (s, 1 C), 141.9 (s, 1 C), 138.4 (s, 1 C), 134.9 (s, 1 C), 133.8 (s, 1 C), 130.4 (s, 1 C), 130.7 (q, *J*=32.6 Hz, 2 C), 129.1 (s, 2 C), 128.8 (s, 2 C), 127.9 (s, 1 C), 127.5 (s, 1 C), 124.2 (s, 1 C), 123.5 (s, 1 C), 123.3 (q, *J*=272.6 Hz, 2 C), 117.6 (br q, *J*=2.0 Hz, 2 C), 114.2 (br spt, *J*=3.9 Hz, 1 C)

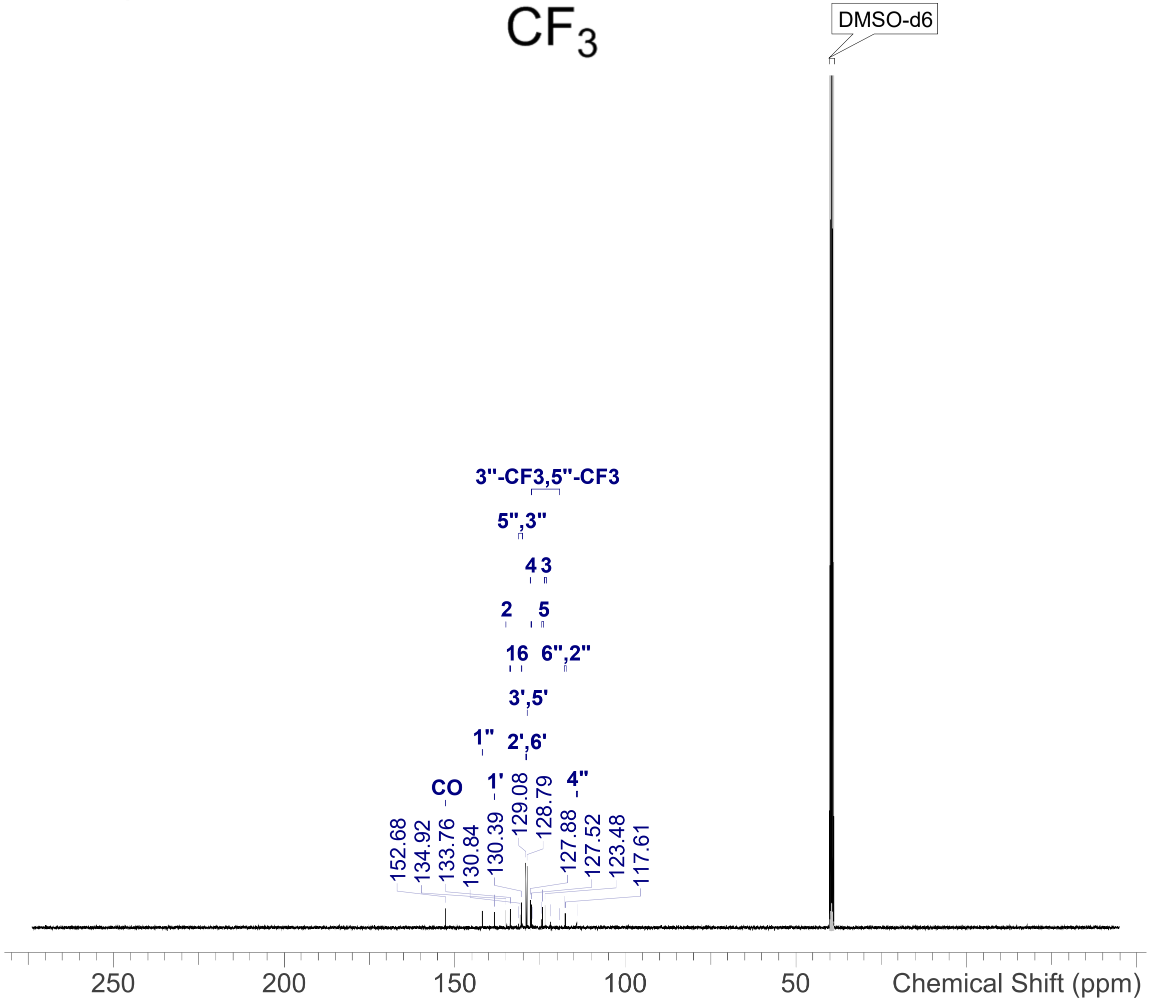

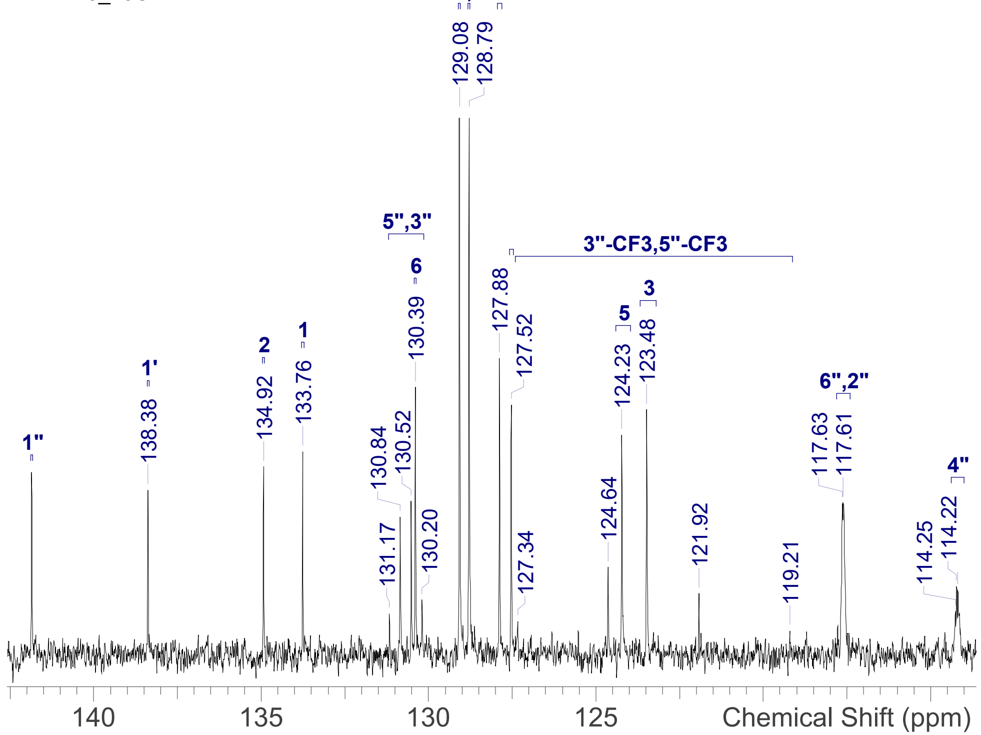

NVR-113_13C.spectrus

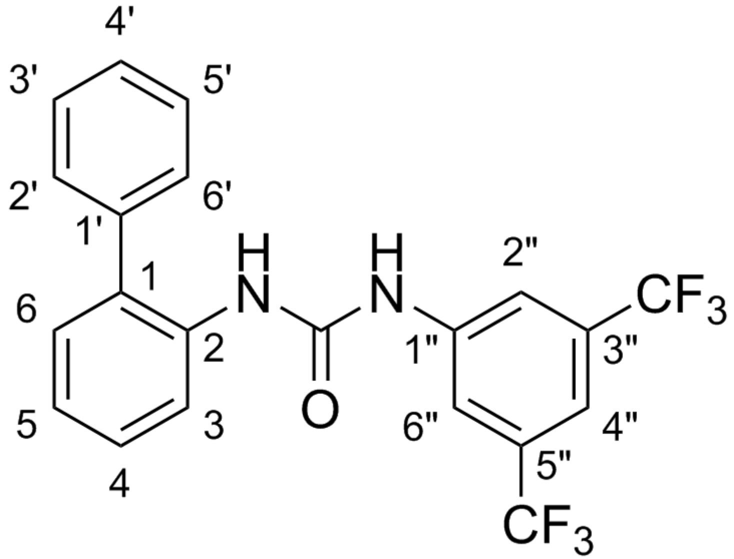

Shift (ppm)	F	m
-61.72	6	s

Acquisition Time (sec)	1.4680
Date	05 Mar 2019 05:40:51
Date Stamp	05 Mar 2019 05:40:51
Frequency (MHz)	376.4419
Nucleus	19F
Number of Transients	16
Solvent	DMSO-d6
Temperature (degree C)	25.000

^{19}F NMR (376 MHz, *DMSO-d_6*) δ ppm -61.72 (s, 6 F)

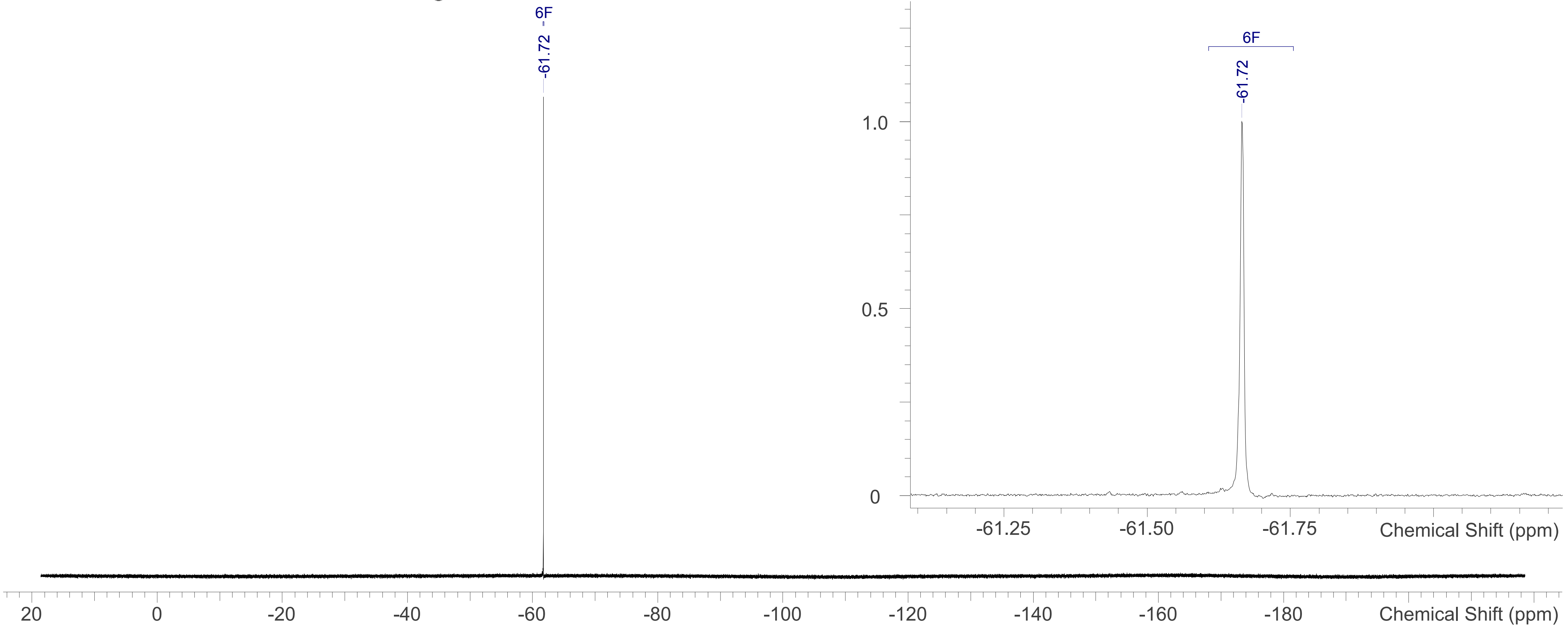

NVR-113_19F.spectrus

Openlynx Report - James B

Page 1

Sample: 1 | Vial:1:14 | ID:JB136 HRMS-1

File:JB136 HRMS-1 | Date:24-Sep-2019 | Time:15:19:51

Description:

Printed: Tue Sep 24 15:39:09 2019

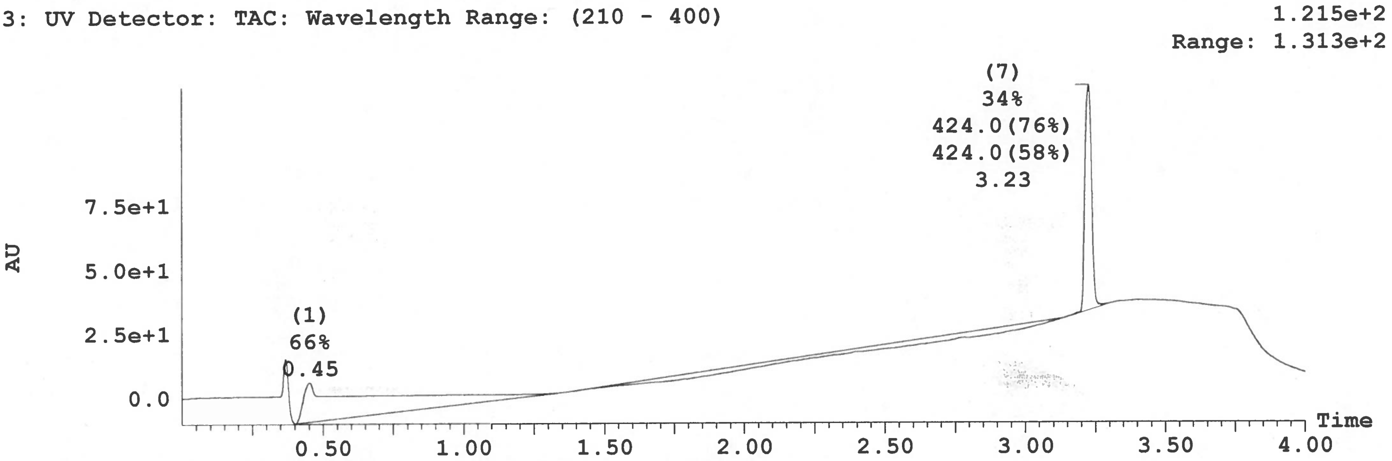

Peak Number	Compound	Time	Area %Total	Mass Found
1		0.45	66.35	Not Found
7	Found	3.23	33.65	424.0, 424.0

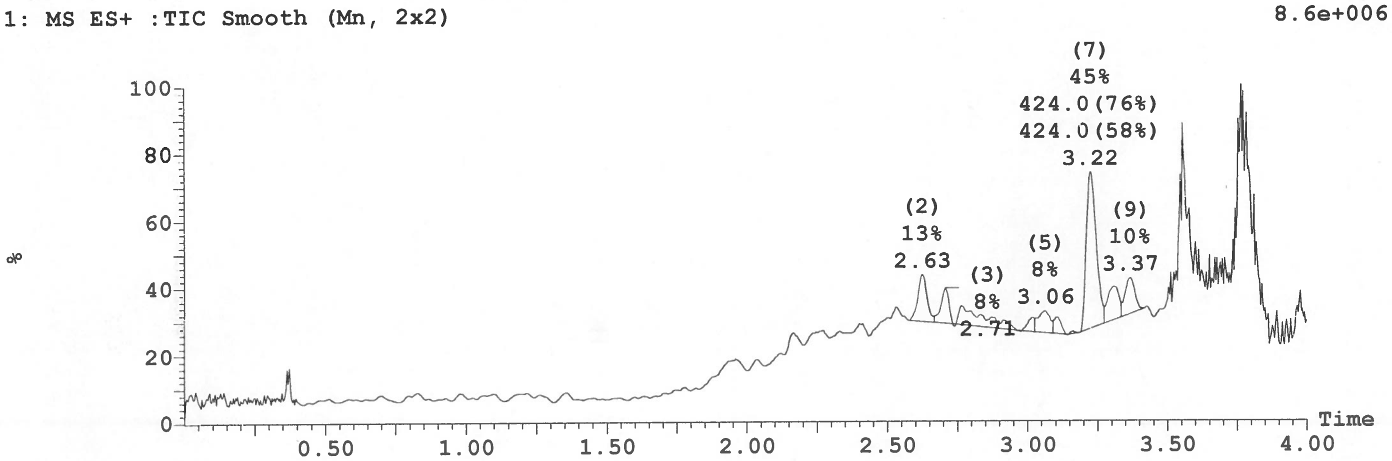

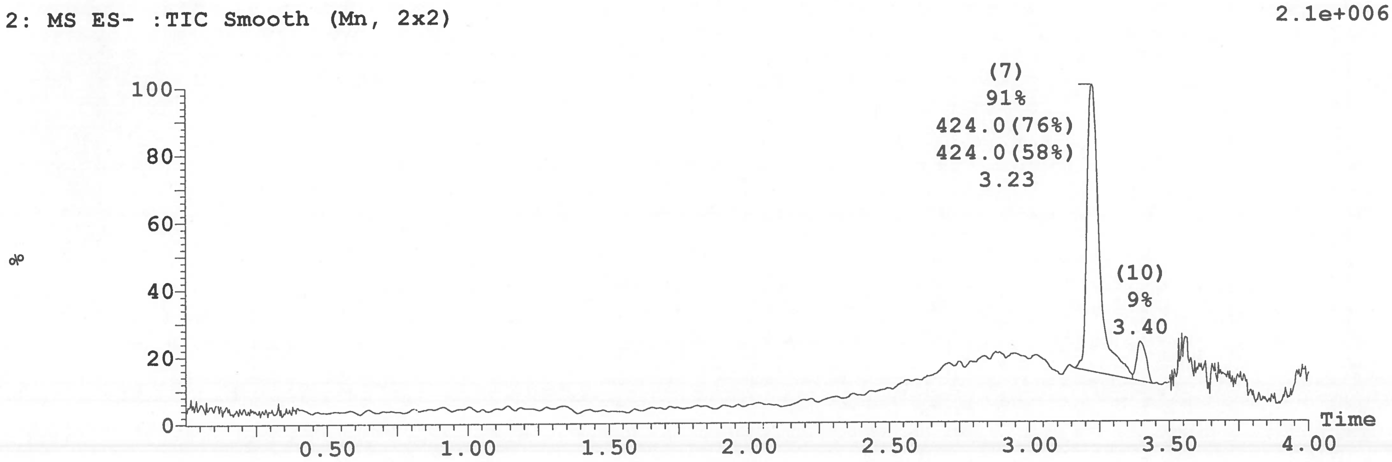

NVR-113 LCMS

Openlynx Report - James B

Sample: 1 | Vial:1:14 | ID:JB136 HRMS-1
File:JB136 HRMS-1 | Date:24-Sep-2019 | Time:15:19:51
Description:

Printed: Tue Sep 24 15:39:09 2019

Peak ID	Compound	Time	Mass Found
1		0.45	Not Found

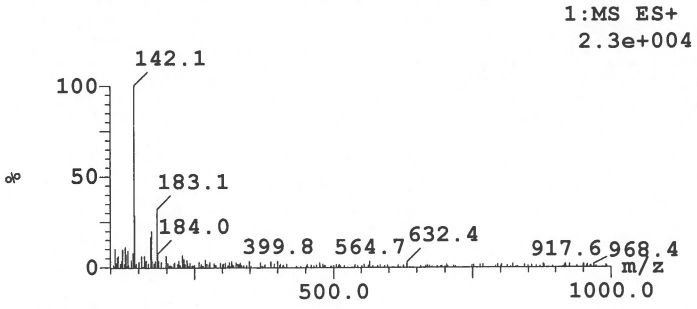

Peak ID	Compound	Time	Mass Found
1		0.45	Not Found

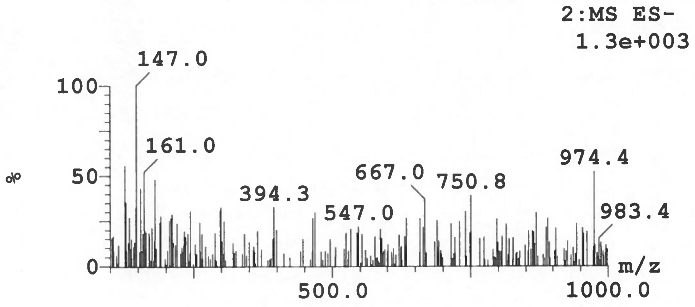

Peak ID	Compound	Time	Mass Found
1		0.45	Not Found

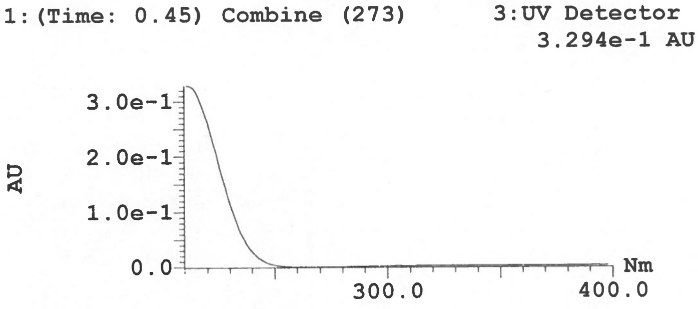

Peak ID	Compound	Time	Mass Found
7	Found	3.22	425

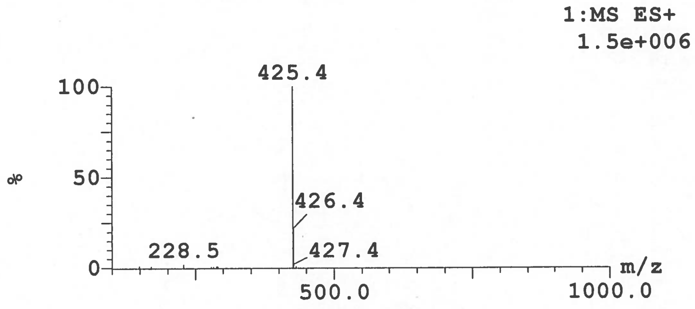

Peak ID	Compound	Time	Mass Found
7	Found	3.22	423

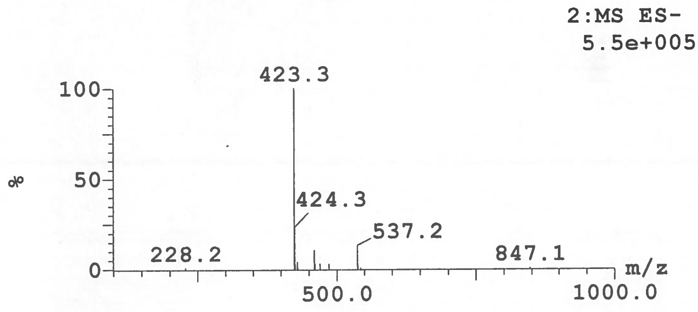

Peak ID	Compound	Time	Mass Found
7		3.22	Not Found

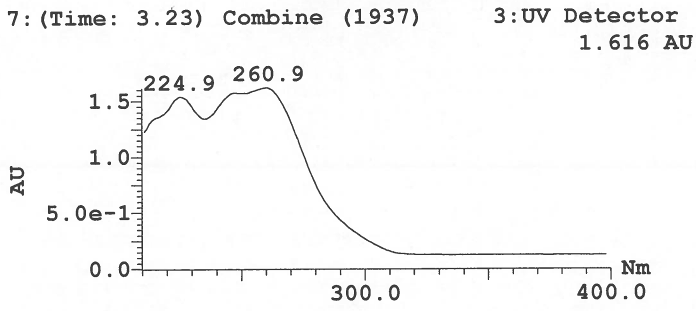

NVR-113 LCMS

Thermo Exactive Plus EMR Orbitrab ASAP neg

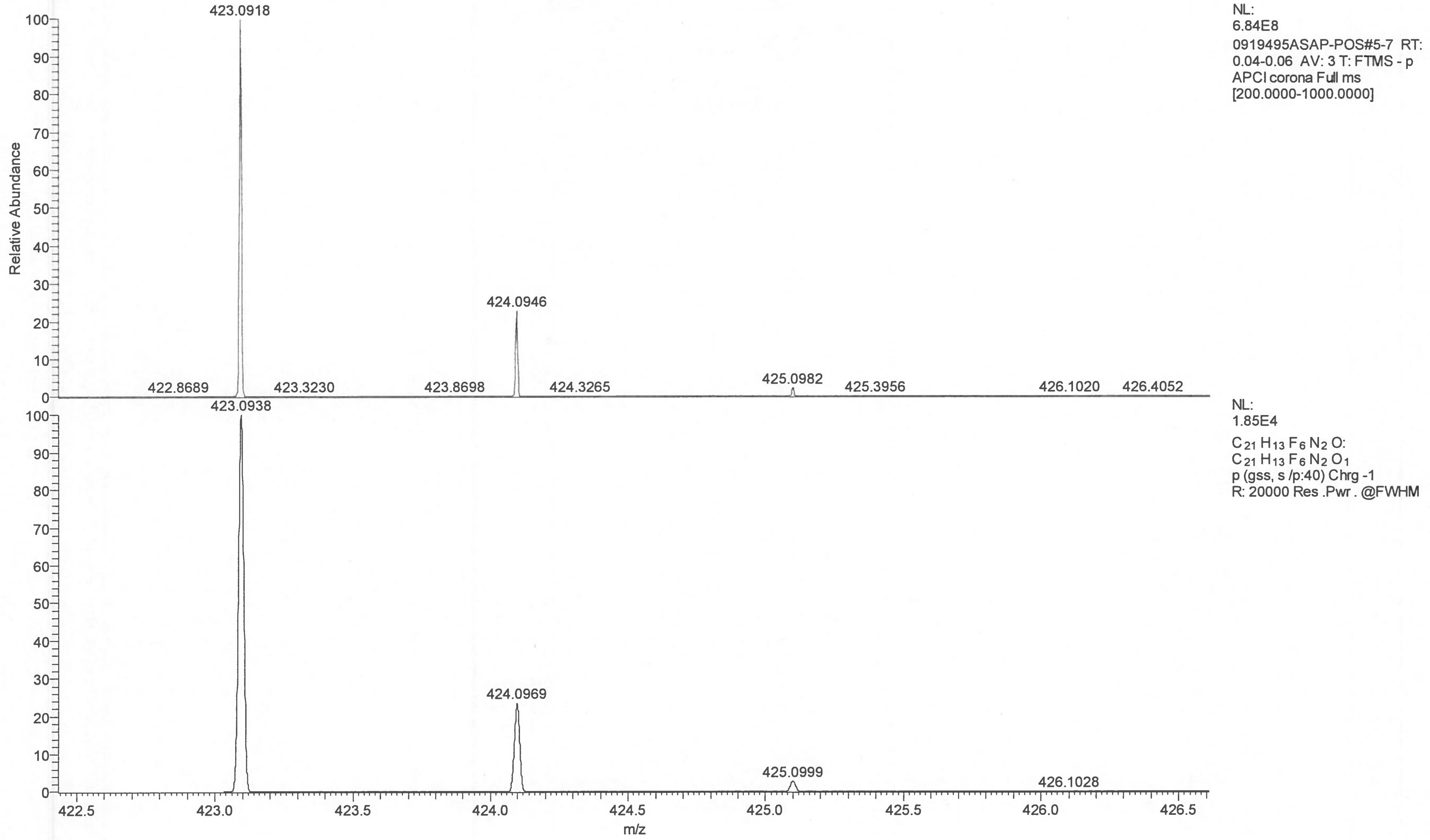

NVR-113 HRMS

Thermo Exactive Plus EMR Orbitrab ASAP neg

m/z	Theo. Mass	Delta (ppm)	RDB equiv.	Composition
423.0918	423.0924	-1.45	14.0	C19 H11 N5 F6
	423.0911	1.71	9.0	C18 H15 O4 N F6
	423.0929	-2.65	1.5	C5 H13 O6 N10 F6
	423.0938	-4.62	13.5	C21 H13 O N2 F6 ←
	423.0897	4.89	9.5	C16 H13 O3 N4 F6

NVR-113 HRMS

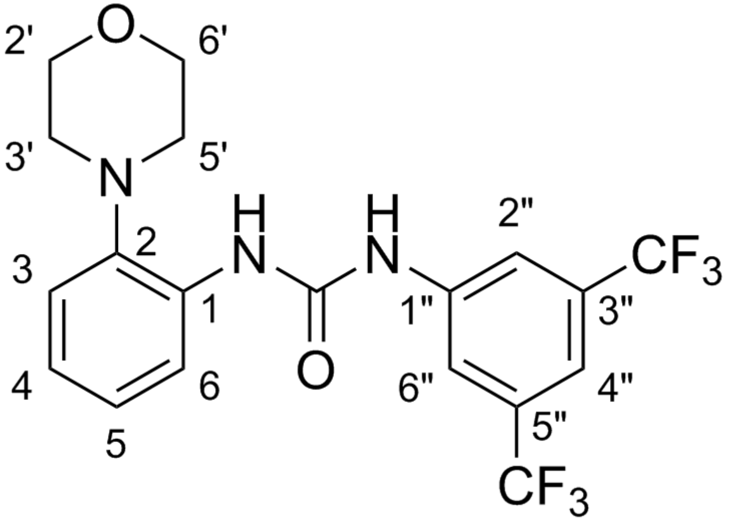

No.	Shift (ppm)	H	m	J (Hz)	Assign
1	10.22	1	s	-	NH"
2	8.26	1	s	-	NH
3	8.13	2	br s	-	6", 2"
4	8.06	1	dd	8.0, 1.5	6
5	7.65	1	br s	-	4"
6	7.23	1	dd	7.8, 1.5	3
7	7.10	1	td	7.8, 1.5	5
8	7.03	1	td	7.5, 1.8	4
9	3.85	4	m	-	6', 2'
10	2.81	4	m	-	5', 3'

Acquisition Time (sec)	3.9846
Date	05 Mar 2019 05:45:20
Date Stamp	05 Mar 2019 05:45:20
Frequency (MHz)	400.0700
Nucleus	1H
Number of Transients	16
Solvent	DMSO-d6
Temperature (degree C)	25.000

^{1}H NMR (400 MHz, *DMSO-d*$_6$) δ ppm 10.22 (s, 1 H), 8.26 (s, 1 H), 8.13 (br s, 2 H), 8.06 (dd, *J*=8.0, 1.5 Hz, 1 H), 7.65 (br s, 1 H), 7.23 (dd, *J*=7.8, 1.5 Hz, 1 H), 7.10 (td, *J*=7.8, 1.5 Hz, 1 H), 7.03 (td, *J*=7.5, 1.8 Hz, 1 H), 3.82 - 3.89 (m, 4 H), 2.77 - 2.86 (m, 4 H)

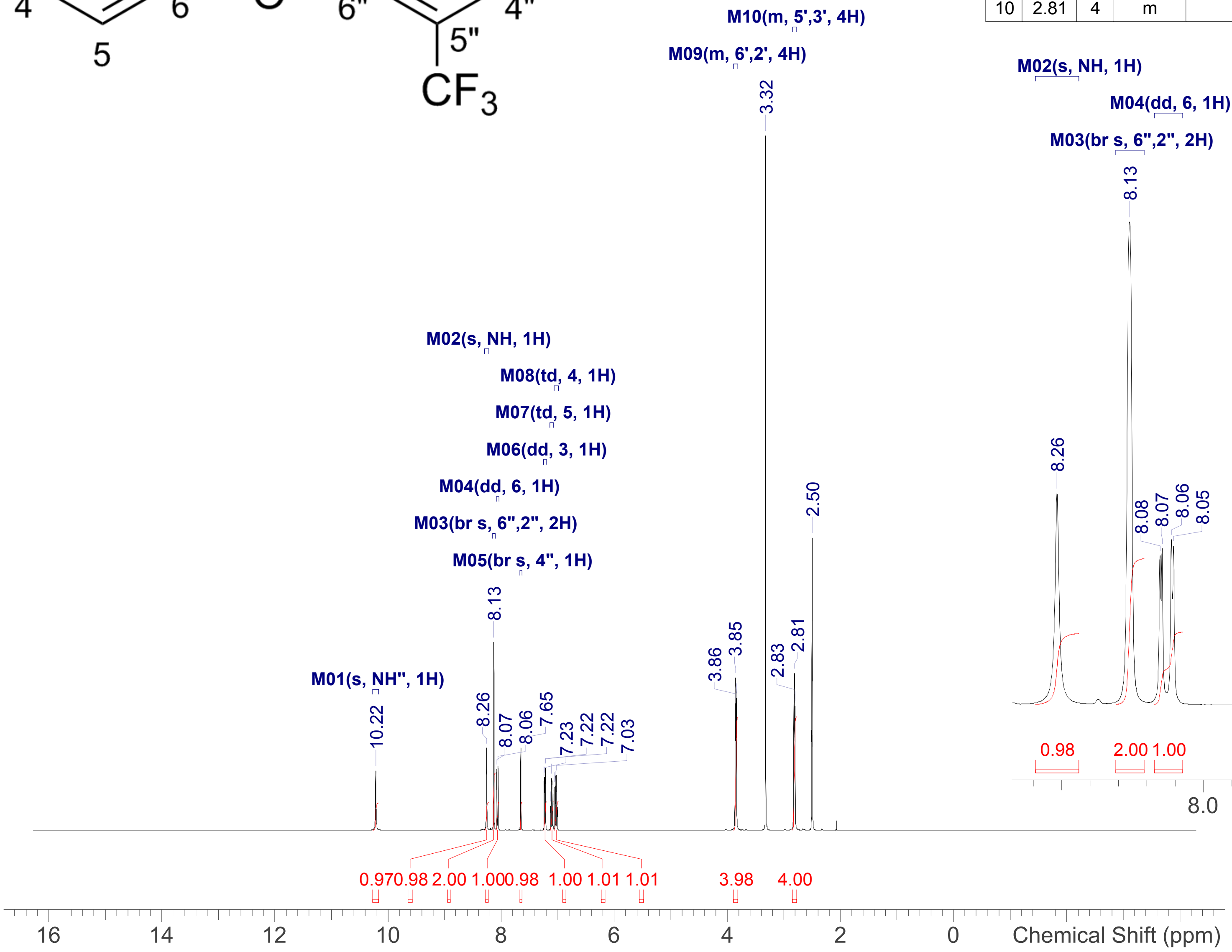

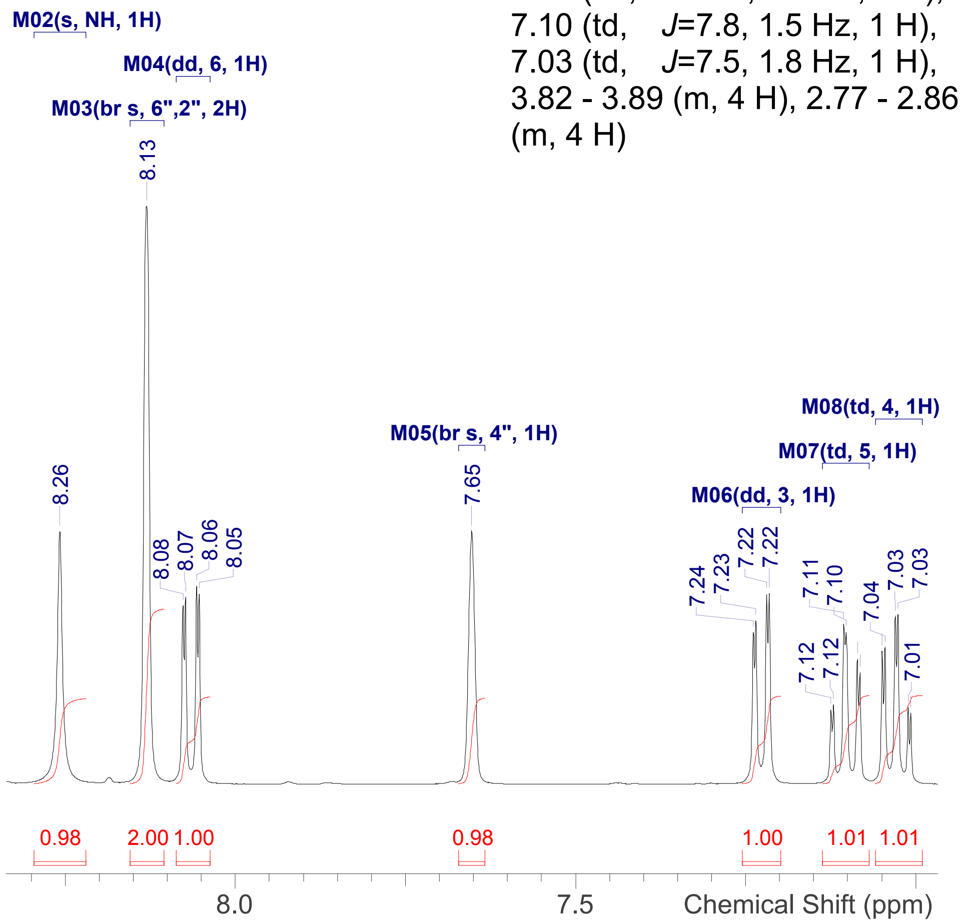

NVR-114_1H.spectrus

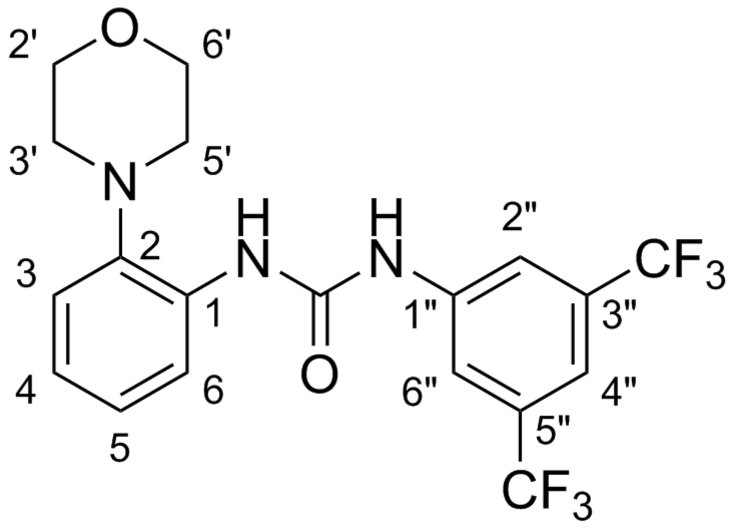

Shift (ppm)	C	m	J (Hz)	Assign
152.2	1	s	-	CO
141.9	1	s	-	1"
141.4	1	s	-	2
133.3	1	s	-	1
130.8	2	q	33.3	5", 3"
124.6	1	s	-	5
123.3	2	q	272.9	3"-CF3, 5"-CF3
122.9	1	s	-	4
120.4	1	s	-	3
119.3	1	s	-	6
117.6	2	br q	3.9	6", 2"
114.3	1	br spt	3.9	4"
66.3	2	s	-	6', 2'
52.0	2	s	-	5', 3'

Acquisition Time (sec)	1.0224
Date	28 Feb 2019 02:02:31
Date Stamp	28 Feb 2019 02:02:31
Frequency (MHz)	100.5977
Nucleus	13C
Number of Transients	256
Solvent	DMSO-d6
Temperature (degree C)	24.997

^{13}C NMR (101 MHz, *DMSO-d$_6$*) δ ppm 152.2 (s, 1 C), 141.9 (s, 1 C), 141.4 (s, 1 C), 133.3 (s, 1 C), 130.8 (q, *J*=33.3 Hz, 2 C), 124.6 (s, 1 C), 122.9 (s, 1 C), 120.4 (s, 1 C), 119.3 (s, 1 C), 123.3 (q, *J*=272.9 Hz, 2 C), 117.6 (br q, *J*=3.9 Hz, 2 C), 114.3 (br spt, *J*=3.9 Hz, 1 C), 66.3 (s, 2 C), 52.0 (s, 2 C)

NVR-114_13C

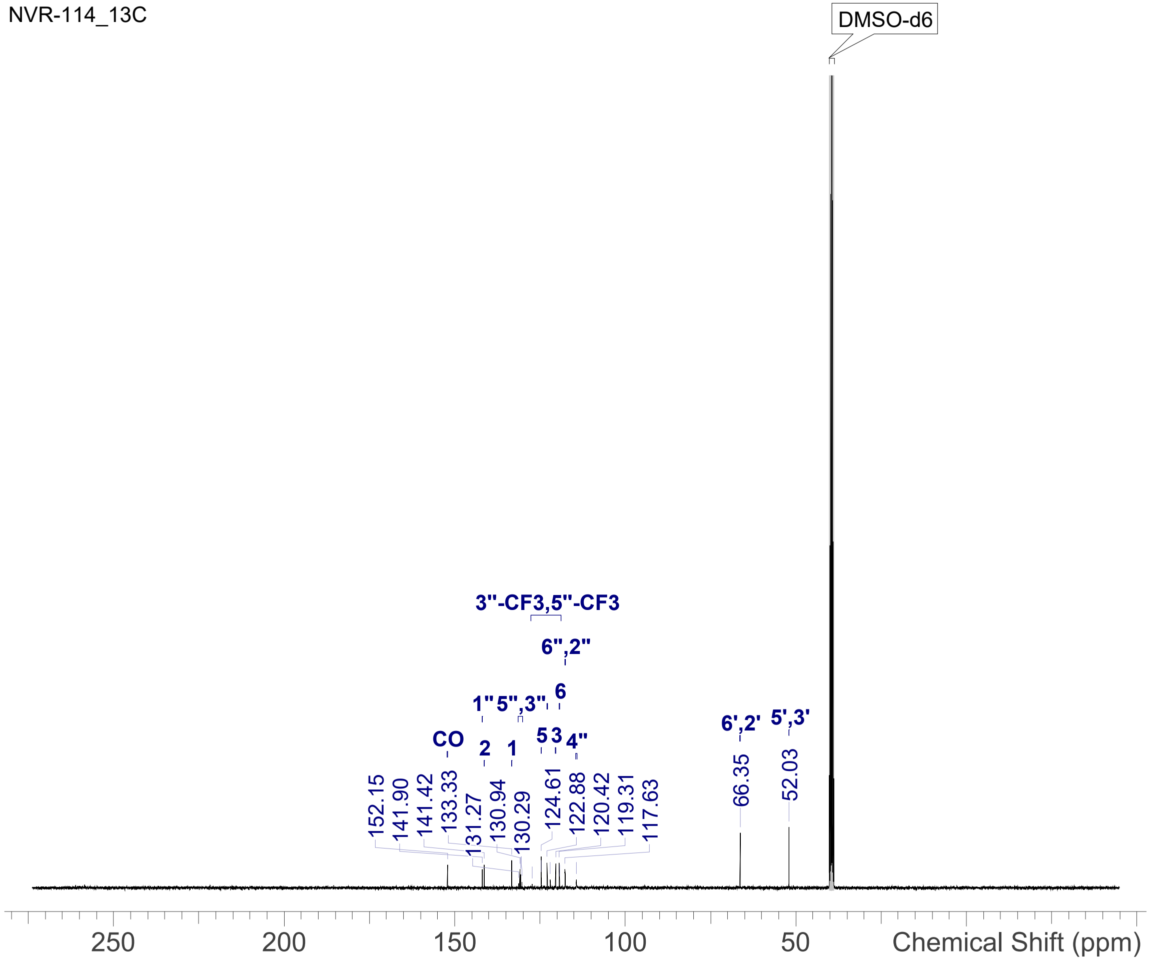

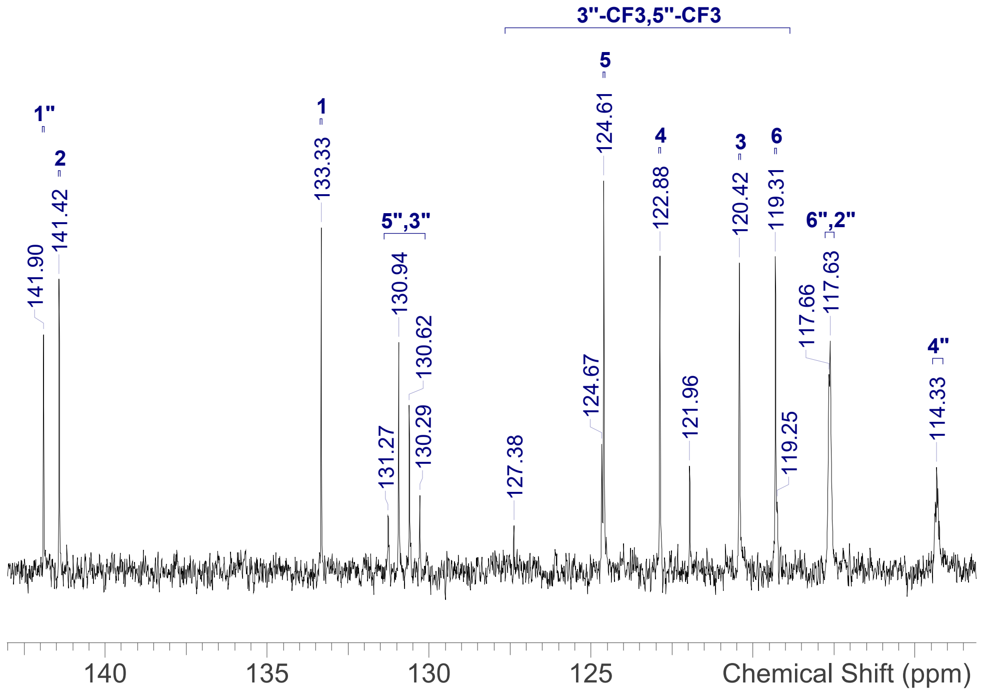

NVR-114_13C.spectrus

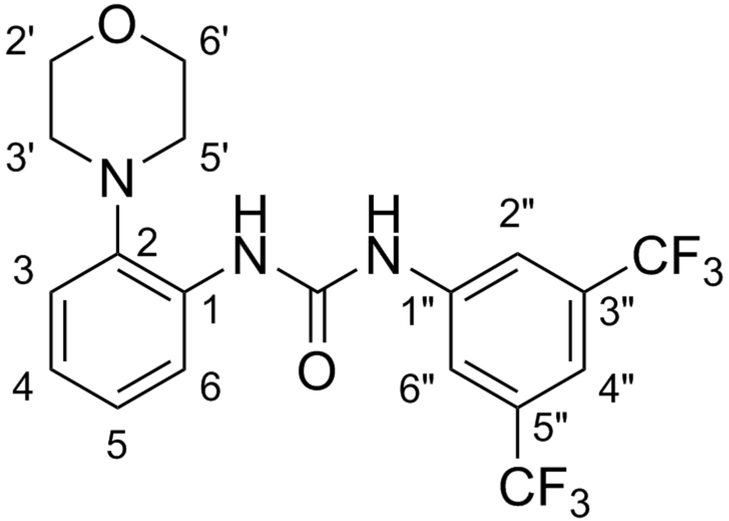

Shift (ppm)	F	m
-61.70	6	s

Acquisition Time (sec)	1.4680
Date	05 Mar 2019 06:35:53
Date Stamp	05 Mar 2019 06:35:53
Frequency (MHz)	376.4419
Nucleus	19F
Number of Transients	16
Solvent	DMSO-d6
Temperature (degree C)	24.999

^{19}F NMR (376 MHz, *DMSO-d_6*) δ ppm -61.70 (s, 6 F)

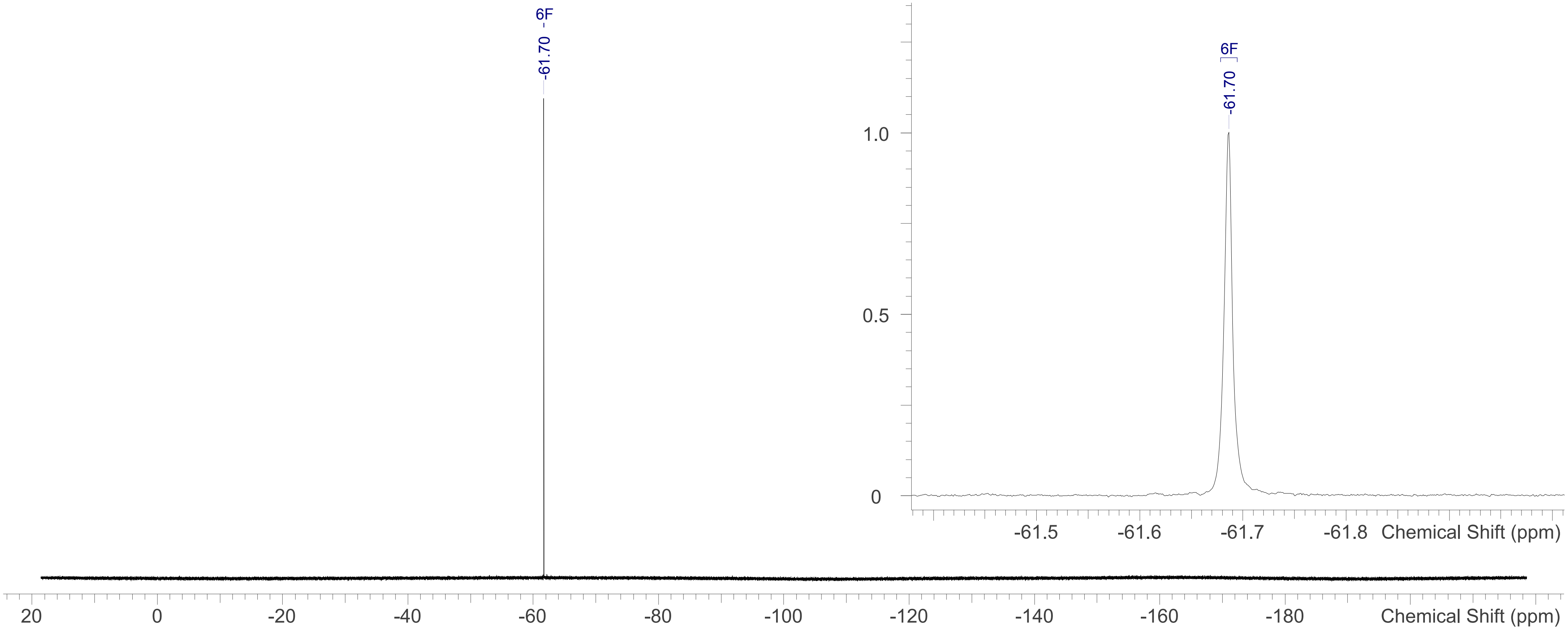

NVR-114_19F.spectrus

Openlynx Report - James B Page 1

Sample: 1	Vial:1:15	ID:JB137 HRMS-1
File:JB137 HRMS-1	Date:24-Sep-2019	Time:15:29:33
Description:		

Printed: Tue Sep 24 15:39:00 2019

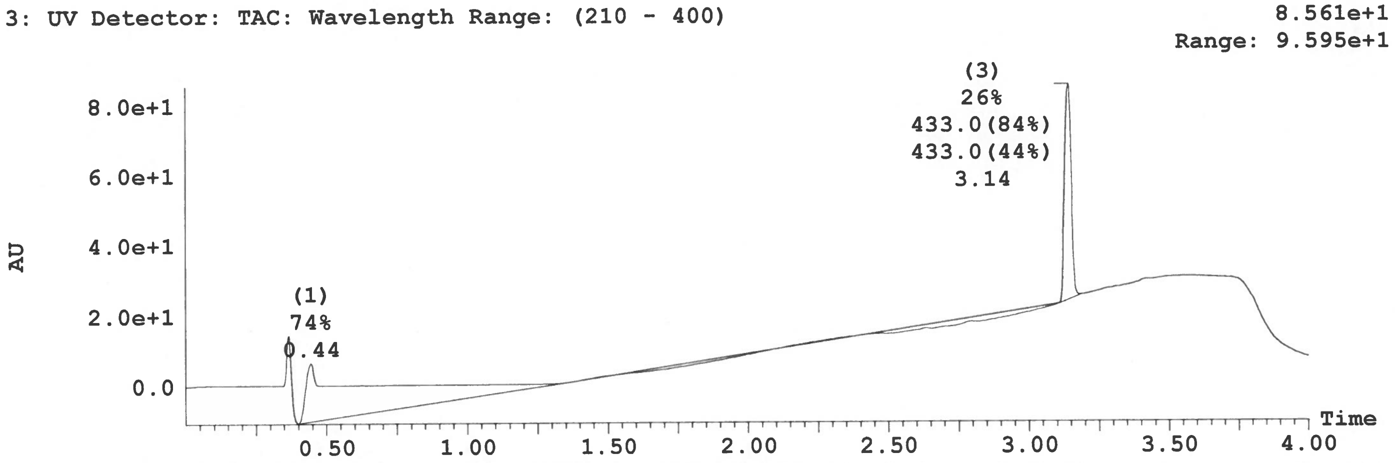

Peak Number	Compound	Time	Area %Total	Mass Found
1		0.44	74.00	Not Found
3	Found	3.14	26.00	433.0, 433.0

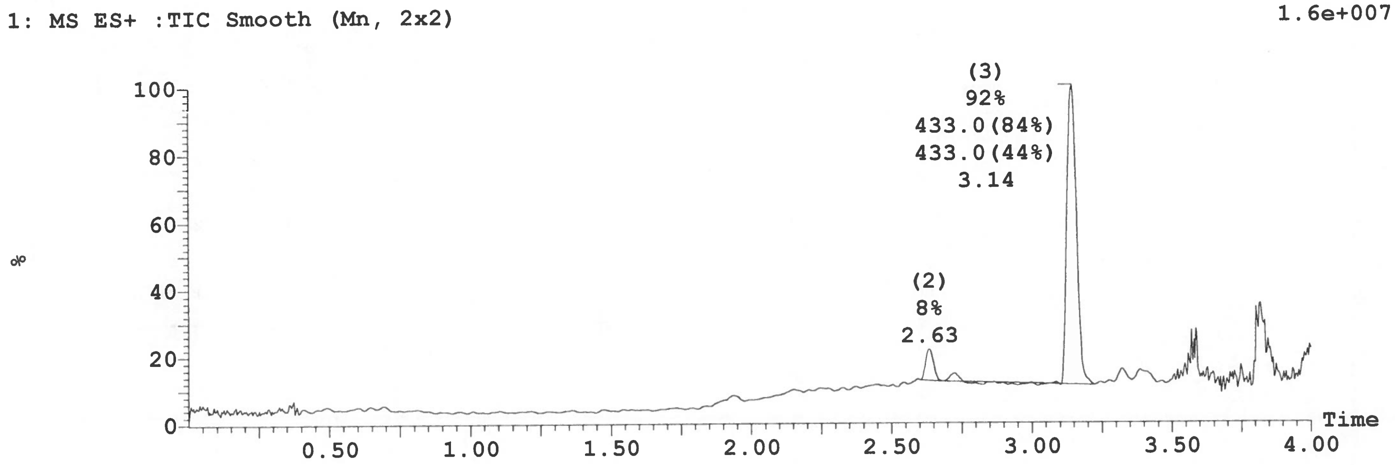

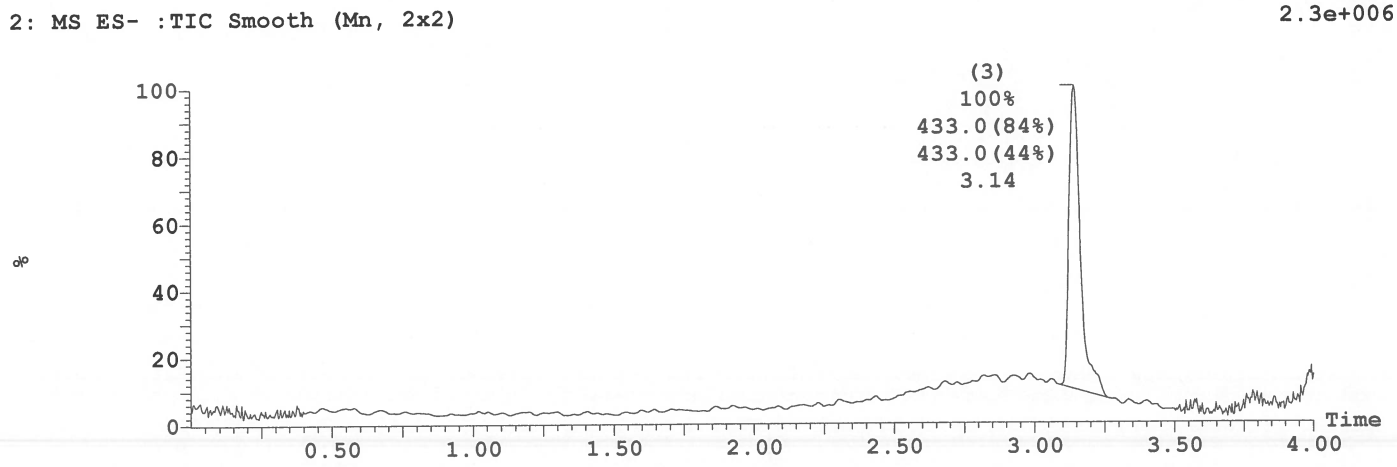

NVR-114 LCMS

Openlynx Report - James B Page 2

Sample: 1	Vial:1:15	ID:JB137 HRMS-1
File:JB137 HRMS-1	Date:24-Sep-2019	Time:15:29:33
Description:		

Printed: Tue Sep 24 15:39:00 2019

Peak ID	Compound	Time	Mass Found
1		0.44	Not Found

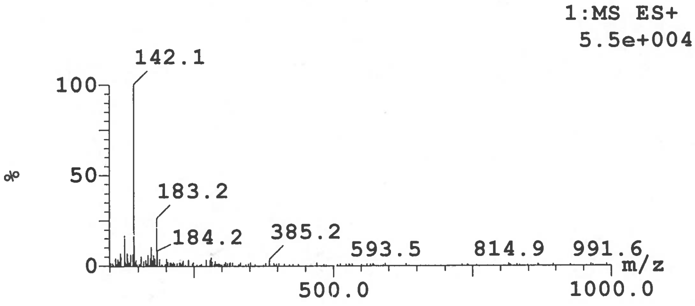

Peak ID	Compound	Time	Mass Found
1		0.44	Not Found

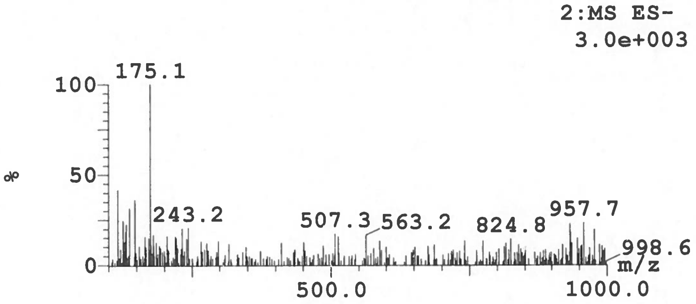

Peak ID	Compound	Time	Mass Found
1		0.44	Not Found

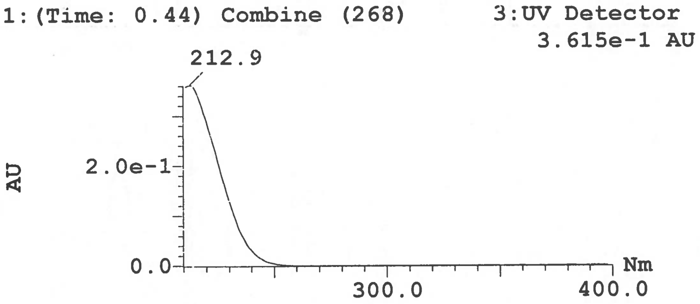

Peak ID	Compound	Time	Mass Found
3	Found	3.14	434

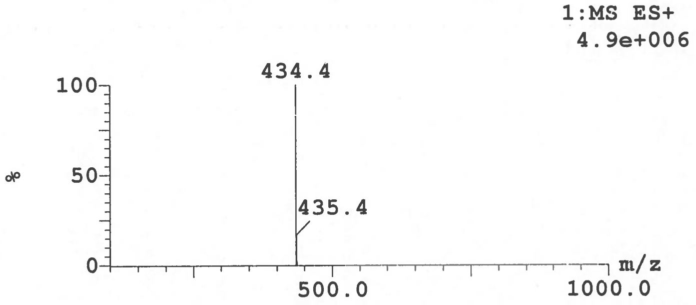

Peak ID	Compound	Time	Mass Found
3	Found	3.14	432

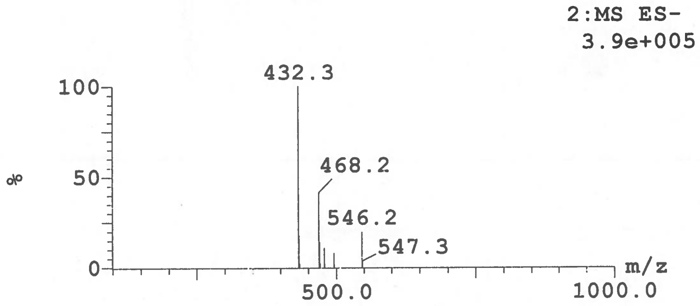

Peak ID	Compound	Time	Mass Found
3		3.14	Not Found

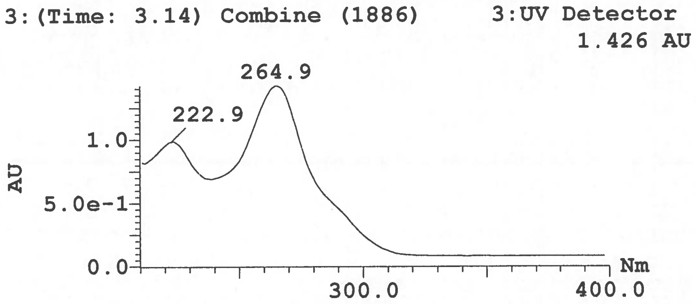

Thermo Exactive Plus EMR Orbitrab ASAP pos

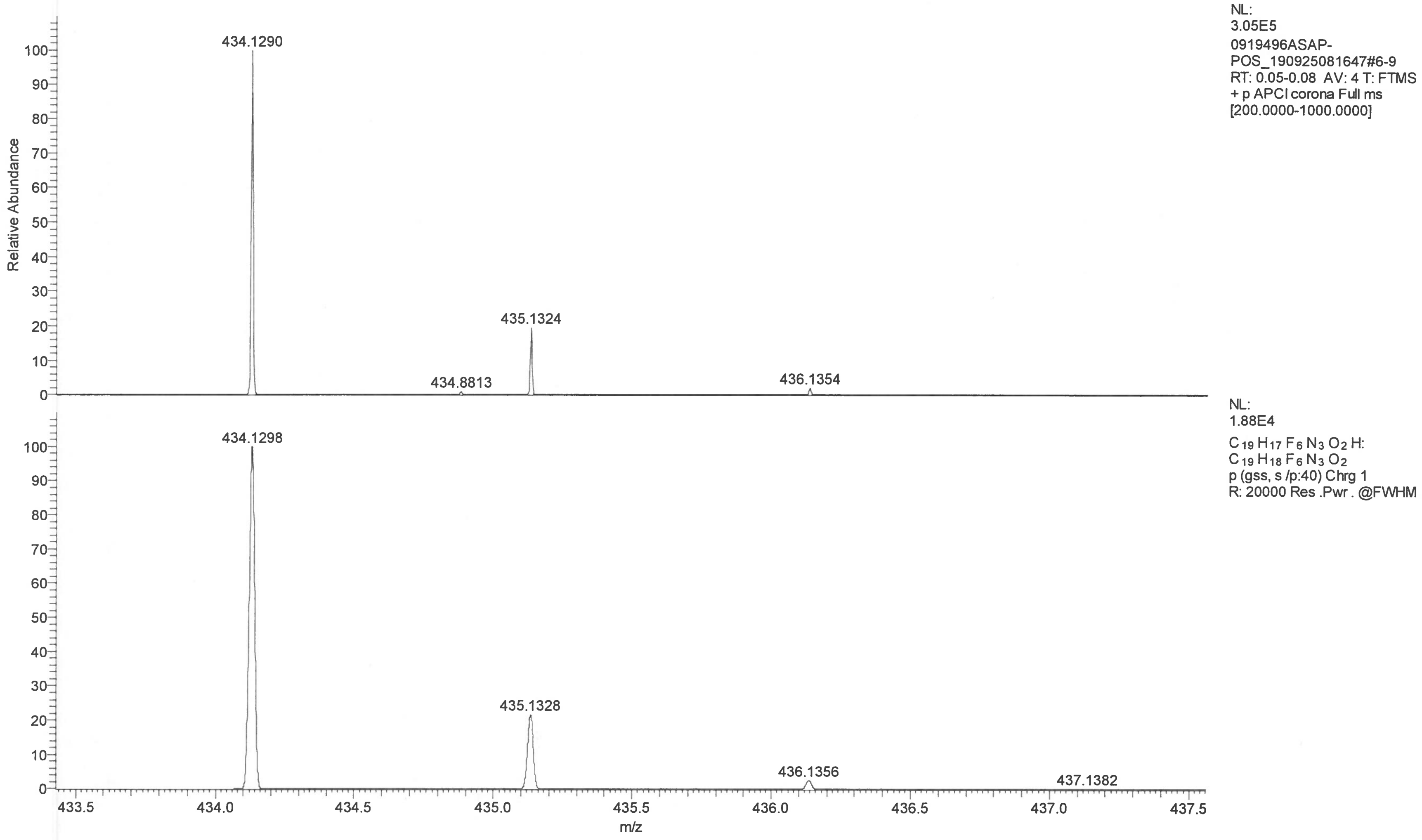

NVR-114 HRMS

Thermo Exactive Plus EMR Orbitrab ASAP pos

m/z	Theo. Mass	Delta (ppm)	RDB equiv.	Composition
434.1290	434.1284	1.31	10.0	C17 H16 O N6 F6
	434.1298	-1.78	9.5	C19 H18 O2 N3 F6 ←
	434.1271	4.39	5.0	C16 H20 O5 N2 F6
	434.1271	4.41	10.5	C15 H14 N9 F6
	434.1311	-4.87	9.0	C21 H20 O3 F6

NVR-114 HRMS

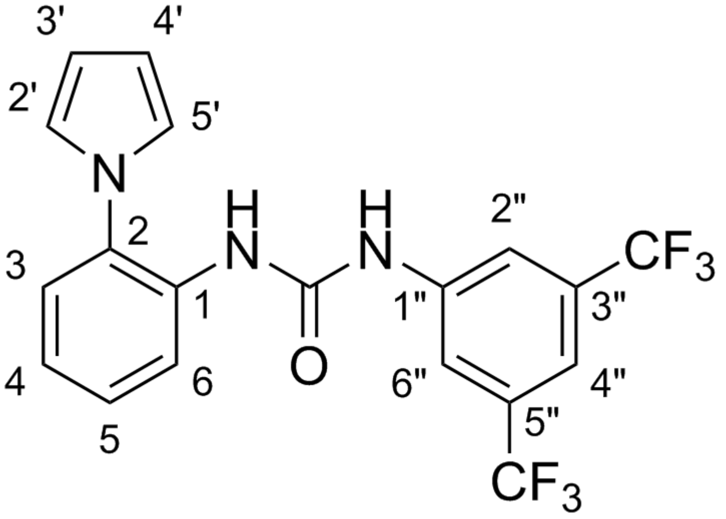

No.	Shift (ppm)	H	m	J (Hz)	Assign
1	9.85	1	s	-	NH"
2	8.05	2	br s	-	6", 2"
3	7.96	1	dd	8.0, 1.3	6
4	7.94	1	br s	-	NH
5	7.62	1	br s	-	4"
6	7.37	1	ddd	8.2, 7.5, 1.8	5
7	7.27	1	dd	7.8, 1.8	3
8	7.21	1	td	7.5, 1.3	4
9	6.99	2	t	2.1	2', 5'
10	6.29	2	t	2.1	3', 4'

Acquisition Time (sec)	3.9846
Date	10 May 2019 00:15:29
Date Stamp	10 May 2019 00:15:29
Frequency (MHz)	400.0700
Nucleus	1H
Number of Transients	16
Solvent	DMSO-d6
Temperature (degree C)	25.001

^{1}H NMR (400 MHz, *DMSO-d_6*) δ ppm 9.85 (s, 1 H), 8.05 (br s, 2 H), 7.96 (dd, *J*=8.0, 1.3 Hz, 1 H), 7.94 (br s, 1 H), 7.62 (br s, 1 H), 7.37 (ddd, *J*=8.2, 7.5, 1.8 Hz, 1 H), 7.27 (dd, *J*=7.8, 1.8 Hz, 1 H), 7.21 (td, *J*=7.5, 1.3 Hz, 1 H), 6.99 (t, *J*=2.1 Hz, 2 H), 6.29 (t, *J*=2.1 Hz, 2 H)

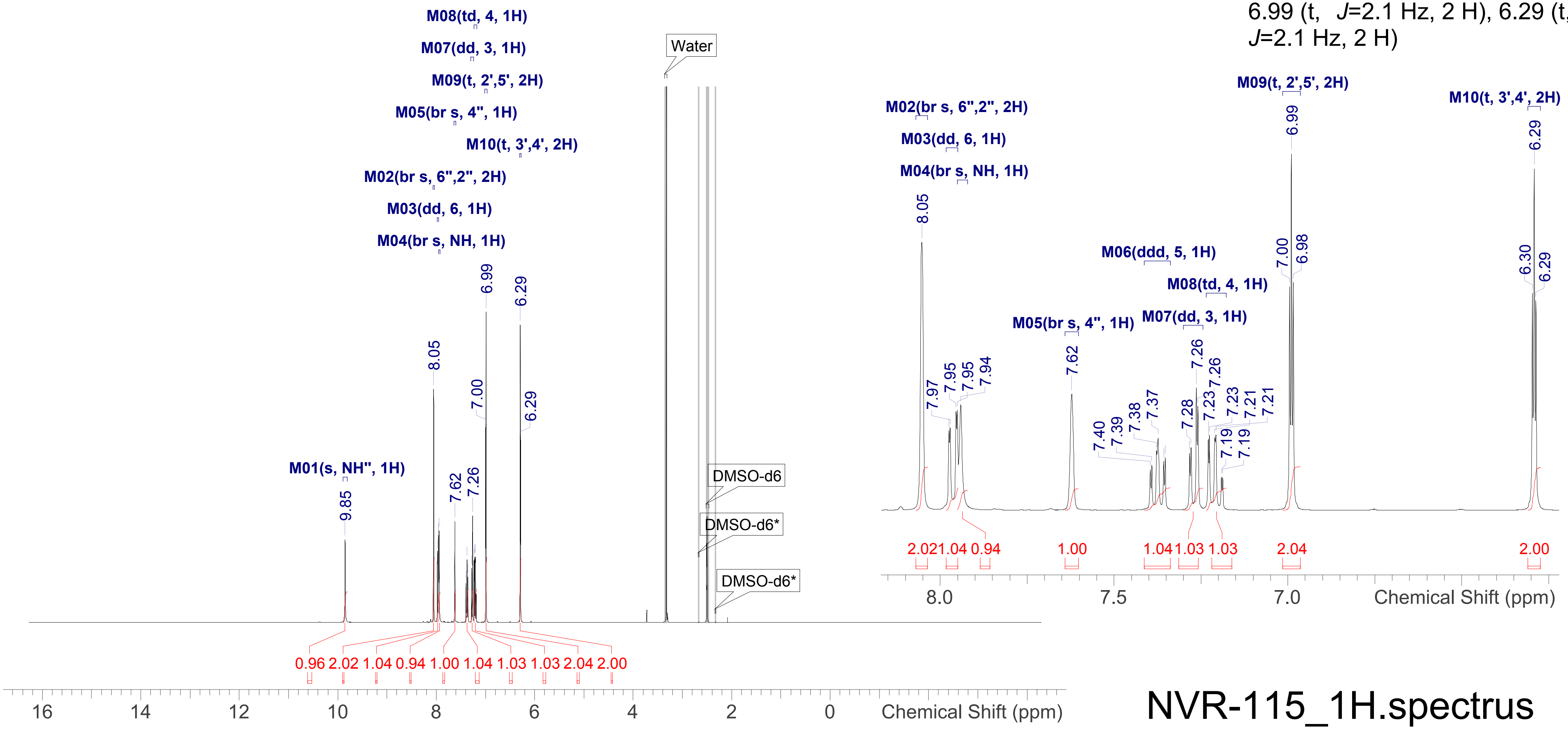

NVR-115_1H.spectrus

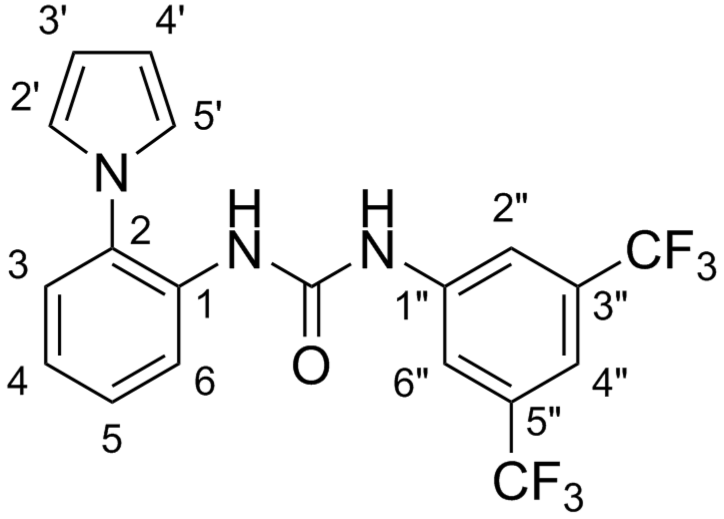

Shift (ppm)	C	m	J (Hz)	Assign
152.6	1	s	-	CO
141.7	1	s	-	1"
132.6	1	s	-	1
132.5	1	s	-	2
130.7	2	q	32.6	3", 5"
127.6	1	s	-	5
126.8	1	s	-	3
124.4	1	s	-	4
123.9	1	s	-	6
123.3	2	q	272.6	5"-CF3, 3"-CF3
122.0	2	s	-	5', 2'
117.7	2	br q	3.9	2", 6"
114.4	1	br spt	3.9	4"
109.7	2	s	-	4', 3'

Acquisition Time (sec)	1.0224
Date	10 May 2019 00:13:11
Date Stamp	10 May 2019 00:13:11
Frequency (MHz)	100.5977
Nucleus	13C
Number of Transients	256
Solvent	DMSO-d6
Temperature (degree C)	25.000

^{13}C NMR (101 MHz, *DMSO-d_6*) δ ppm 152.6 (s, 1 C), 141.7 (s, 1 C), 132.6 (s, 1 C), 132.5 (s, 1 C), 130.7 (q, *J*=32.6 Hz, 2 C), 127.6 (s, 1 C), 126.8 (s, 1 C), 124.4 (s, 1 C), 123.9 (s, 1 C), 122.0 (s, 2 C), 123.3 (q, *J*=272.6 Hz, 2 C), 117.7 (br q, *J*=3.9 Hz, 2 C), 114.4 (br spt, *J*=3.9 Hz, 1 C), 109.7 (s, 2 C)

NVR-115_13C

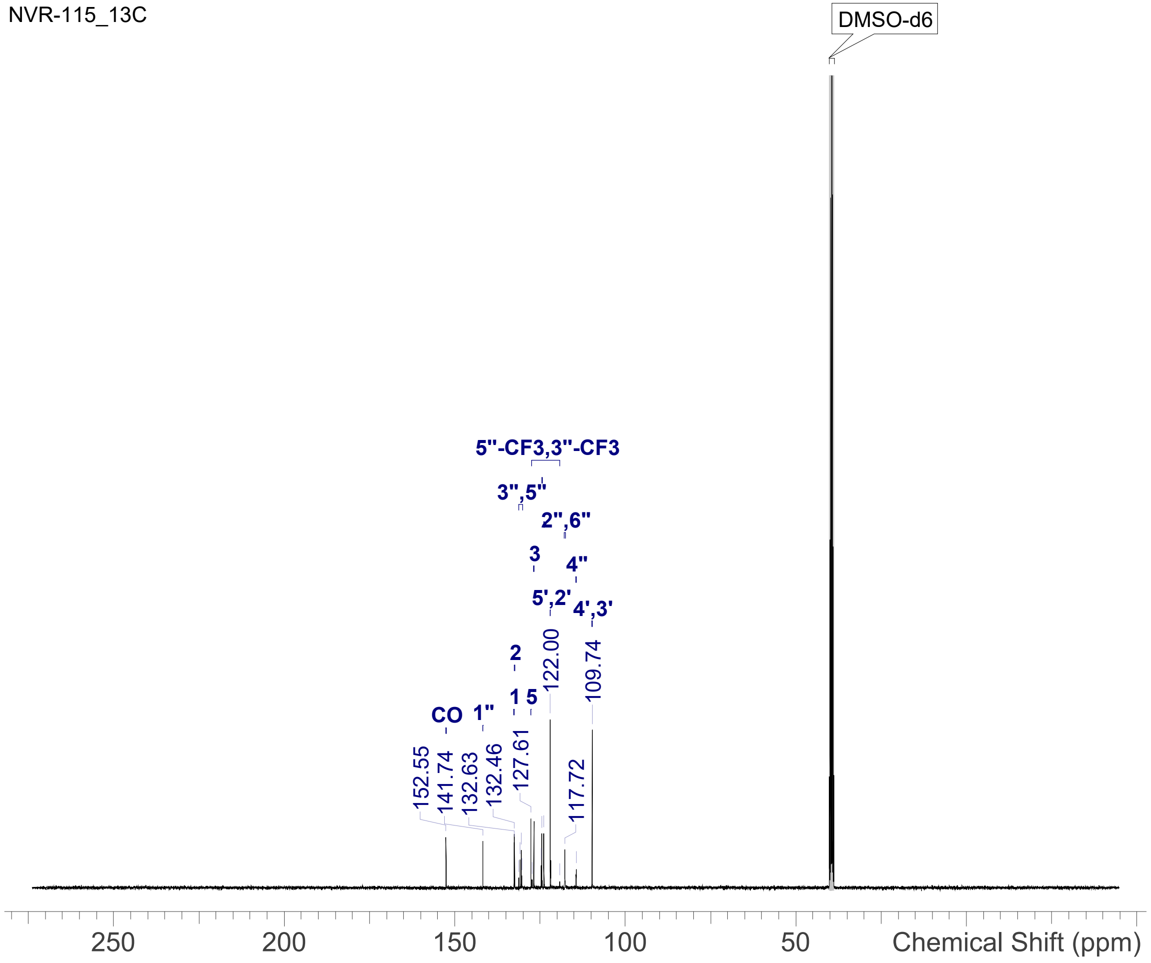

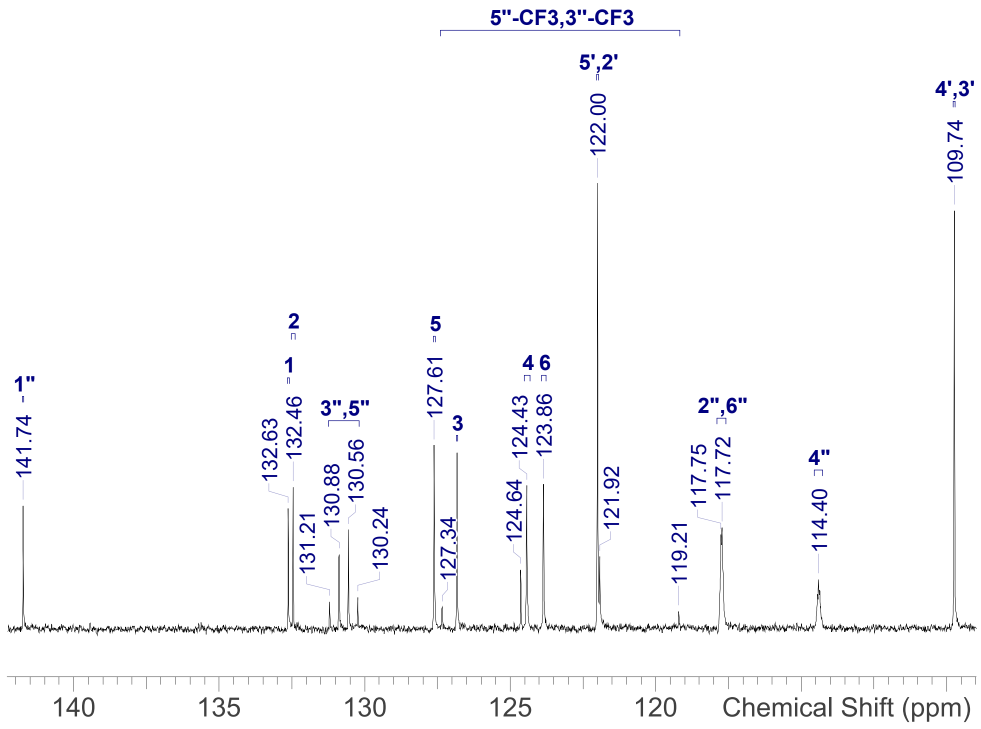

NVR-115_13C.spectrus

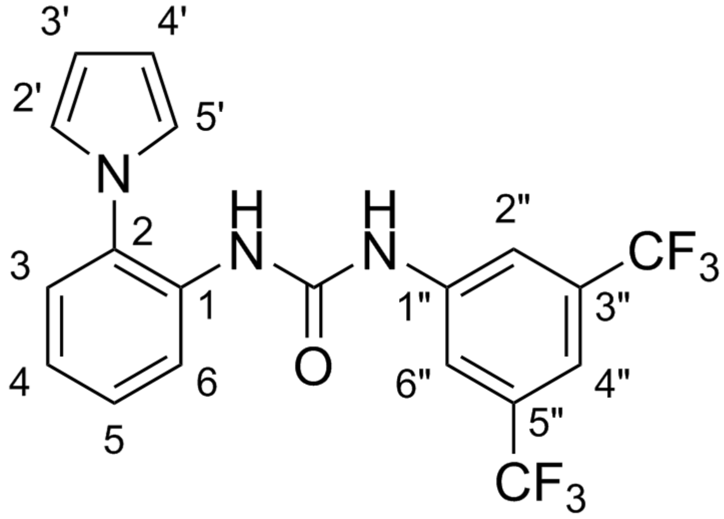

Shift (ppm)	F	m
-61.71	6	s

Acquisition Time (sec)	1.4680
Date	05 Mar 2019 20:54:43
Date Stamp	05 Mar 2019 20:54:43
Frequency (MHz)	376.4419
Nucleus	19F
Number of Transients	16
Solvent	DMSO-d6
Temperature (degree C)	25.001

^{19}F NMR (376 MHz, *DMSO-d_6*) δ ppm -61.71 (s, 6 F)

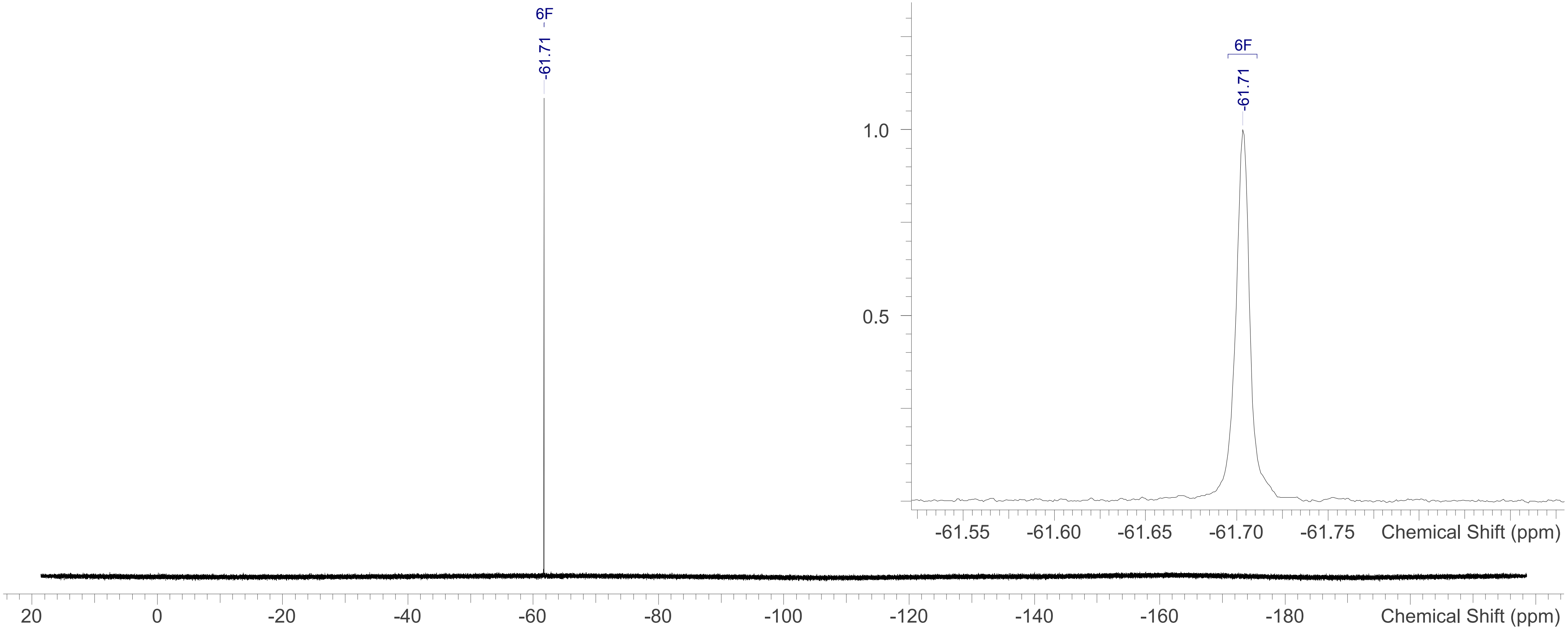

NVR-115_19F.spectrus

Openlynx Report - James B

Page 1

Sample: 1 | Vial:2:33 | ID:JB138 HRMS-1

File:JB138 HRMS-1 | Date:26-Sep-2019 | Time:11:58:24

Description:

Printed: Thu Sep 26 12:43:44 2019

3: UV Detector: TAC: Wavelength Range: (210 - 400)

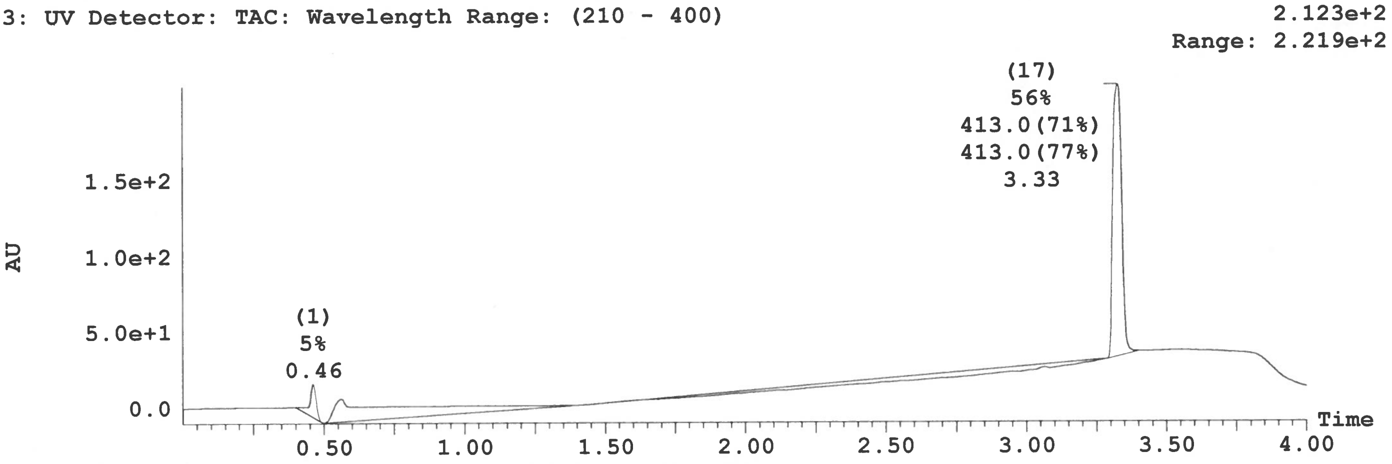

Peak Number	Compound	Time	Area %Total	Mass Found
1		0.46	4.94	Not Found
2		0.56	39.24	Not Found
17	Found	3.33	55.82	413.0, 413.0

1: MS ES+ :TIC Smooth (Mn, 2x2)

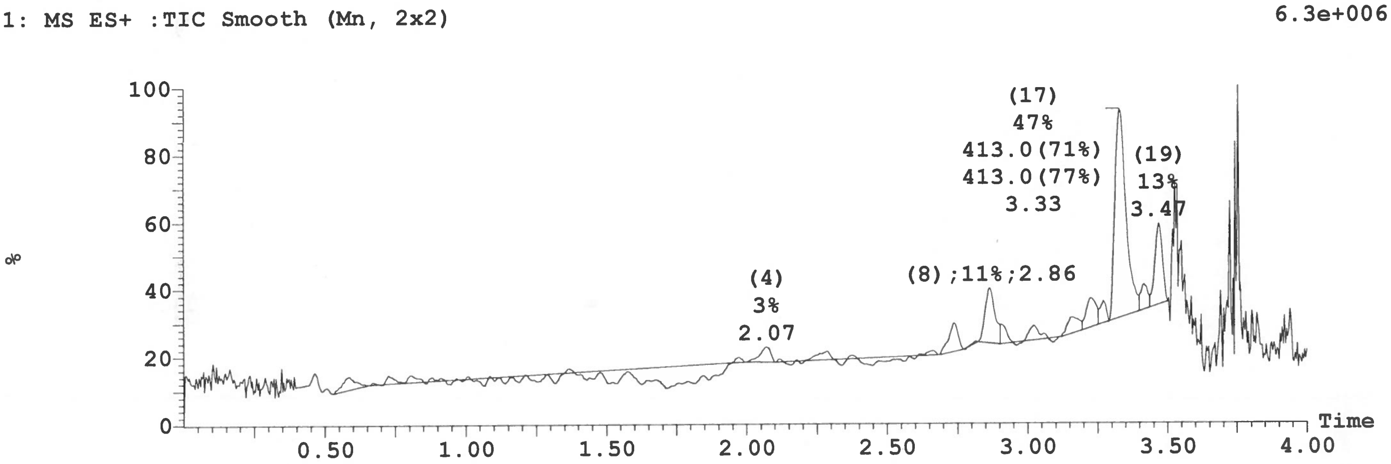

2: MS ES- :TIC Smooth (Mn, 2x2)

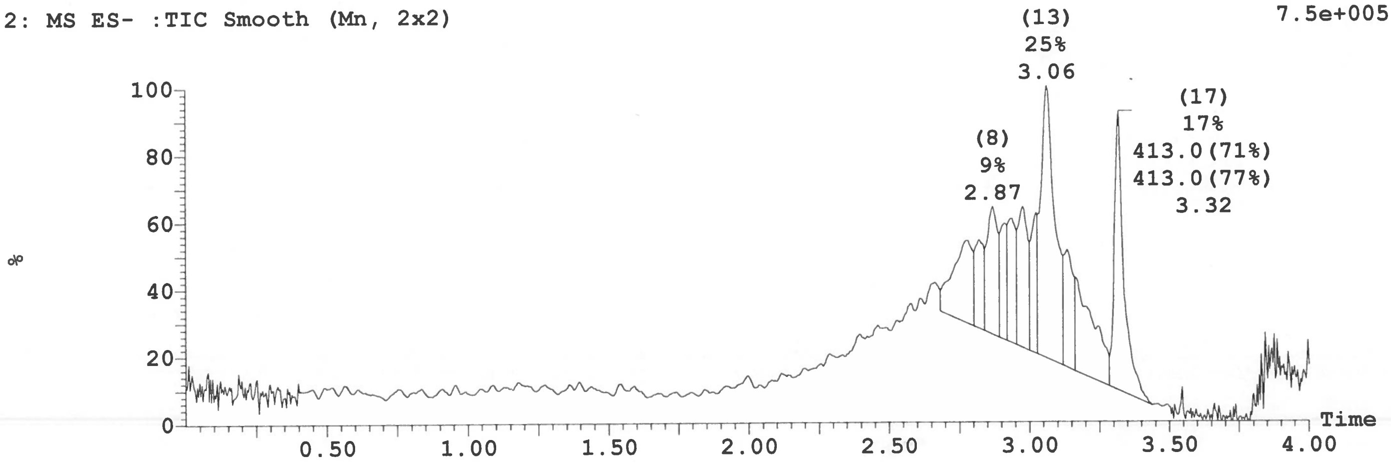

NVR-115 LCMS

Openlynx Report - James B

Sample: 1 | Vial:2:33 | ID:JB138 HRMS-1
File:JB138 HRMS-1 | Date:26-Sep-2019 | Time:11:58:24
Description:

Printed: Thu Sep 26 12:43:44 2019

Peak ID	Compound	Time	Mass Found
1		0.46	Not Found

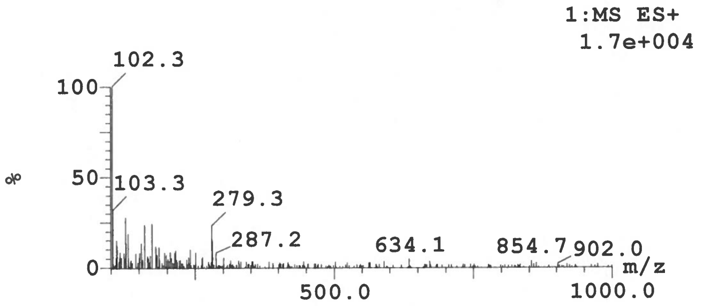

Peak ID	Compound	Time	Mass Found
1		0.46	Not Found

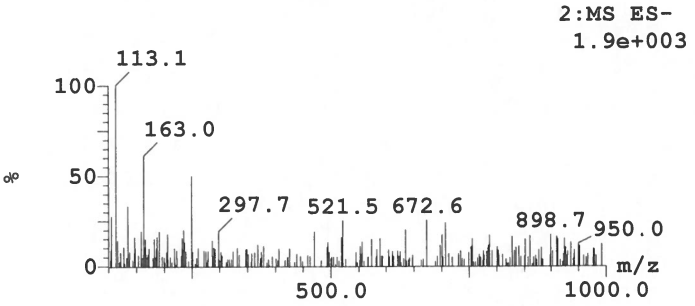

Peak ID	Compound	Time	Mass Found
1		0.46	Not Found

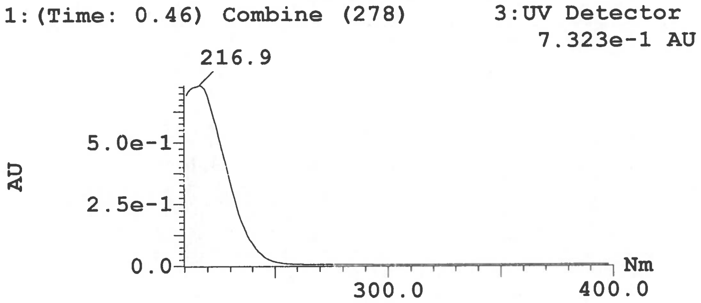

Peak ID	Compound	Time	Mass Found
2		0.56	Not Found

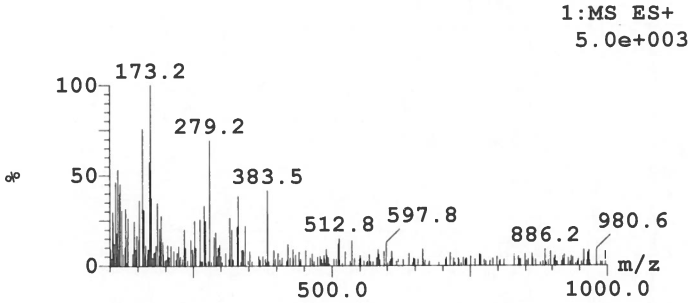

Peak ID	Compound	Time	Mass Found
2		0.56	Not Found

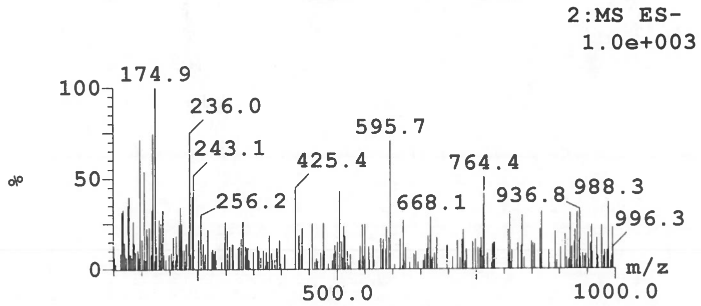

Peak ID	Compound	Time	Mass Found
2		0.56	Not Found

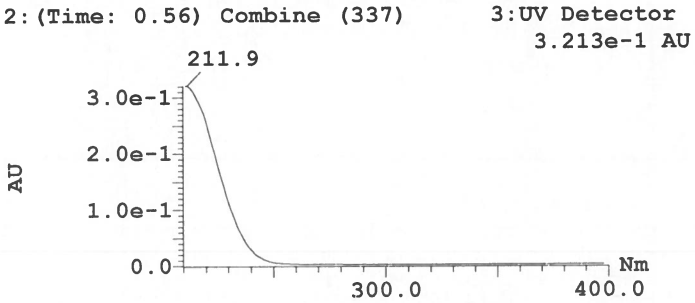

Peak ID	Compound	Time	Mass Found
17	Found	3.33	414

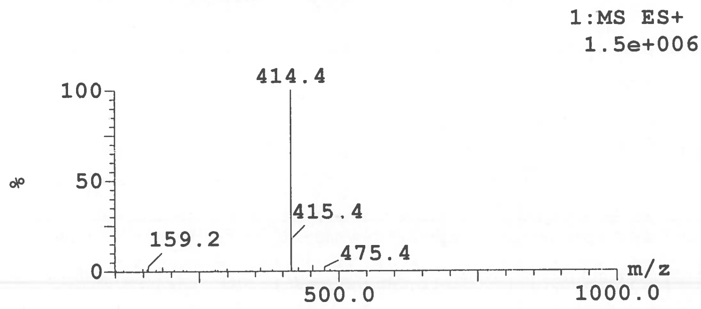

Peak ID	Compound	Time	Mass Found
17	Found	3.33	412

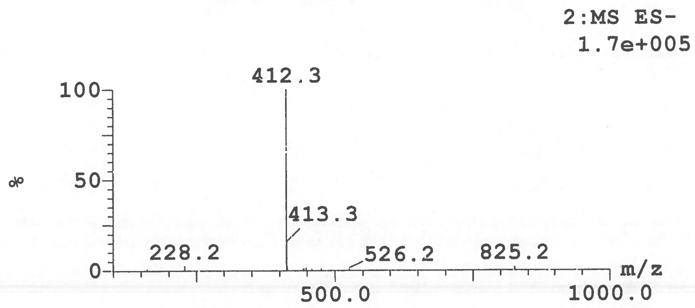

Sample: 1
File:JB138 HRMS-1
Description:

Vial:2:33
Date:26-Sep-2019

ID:JB138 HRMS-1
Time:11:58:24

Printed: Thu Sep 26 12:43:44 2019

Peak ID	Compound	Time	Mass Found
17		3.33	Not Found

17: (Time: 3.33) Combine (1997)   3:UV Detector
3.235 AU

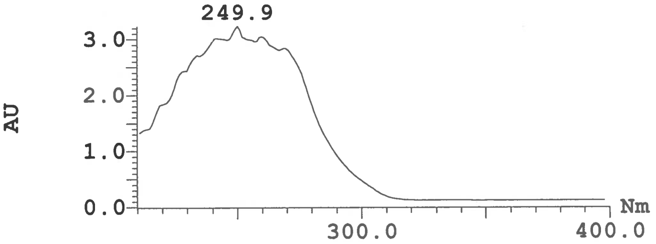

Thermo Exactive Plus EMR Orbitrab HESI neg

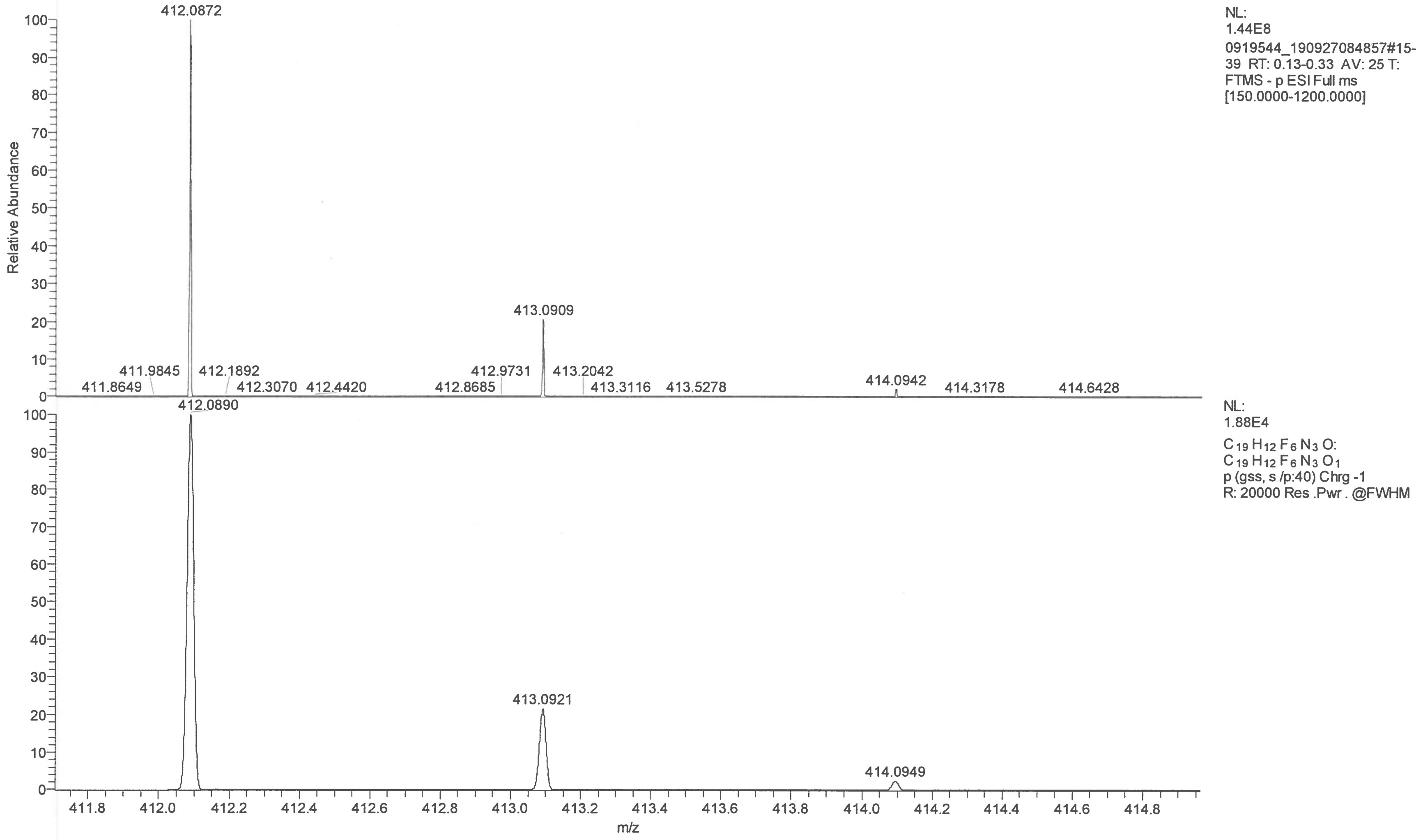

NVR-115 HRMS

Thermo Exactive Plus EMR Orbitrab HESI neg

m/z	Theo. Mass	Delta (ppm)	RDB equiv.	Composition
412.0872	412.0877	-1.12	13.0	C17 H10 N6 F6
	412.0863	2.12	8.0	C16 H14 O4 N2 F6
	412.0890	-4.38	12.5	C19 H12 O N3 F6←

NVR-115 HRMS

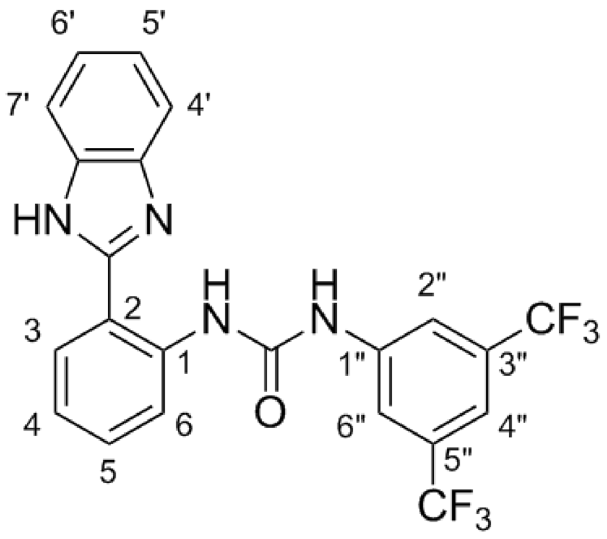

Shift (ppm)	H	m	J (Hz)	Assign
13.12	1	br s	-	Benz-NH
12.45	1	s	-	NH
10.42	1	s	-	NH"
8.44	1	dd	8.5, 1.0	6
8.29	2	br s	-	6", 2"
8.10	1	dd	7.9, 1.4	3
7.73	2	br s	-	7', 4'
7.66	1	br s	-	4"
7.48	1	ddd	8.5, 7.2, 1.4	5
7.30	2	m	-	6', 5'
7.22	1	ddd	7.9, 7.2, 1.0	4

Acquisition Time (sec)	3.9846
Date	09 May 2019 15:29:13
Date Stamp	09 May 2019 15:29:13
Frequency (MHz)	400.0700
Nucleus	1H
Number of Transients	4
Solvent	DMSO-d6
Temperature (degree C)	24.999

^{1}H NMR (400 MHz, *DMSO-d_6*) δ ppm 13.12 (br s, 1 H), 12.45 (s, 1 H), 10.42 (s, 1 H), 8.44 (dd, *J*=8.5, 1.0 Hz, 1 H), 8.29 (br s, 2 H), 8.10 (dd, *J*=7.9, 1.4 Hz, 1 H), 7.66 (br s, 1 H), 7.73 (br s, 2 H), 7.48 (ddd, *J*=8.5, 7.2, 1.4 Hz, 1 H), 7.26 - 7.34 (m, 2 H), 7.22 (ddd, *J*=7.9, 7.2, 1.0 Hz, 1 H)

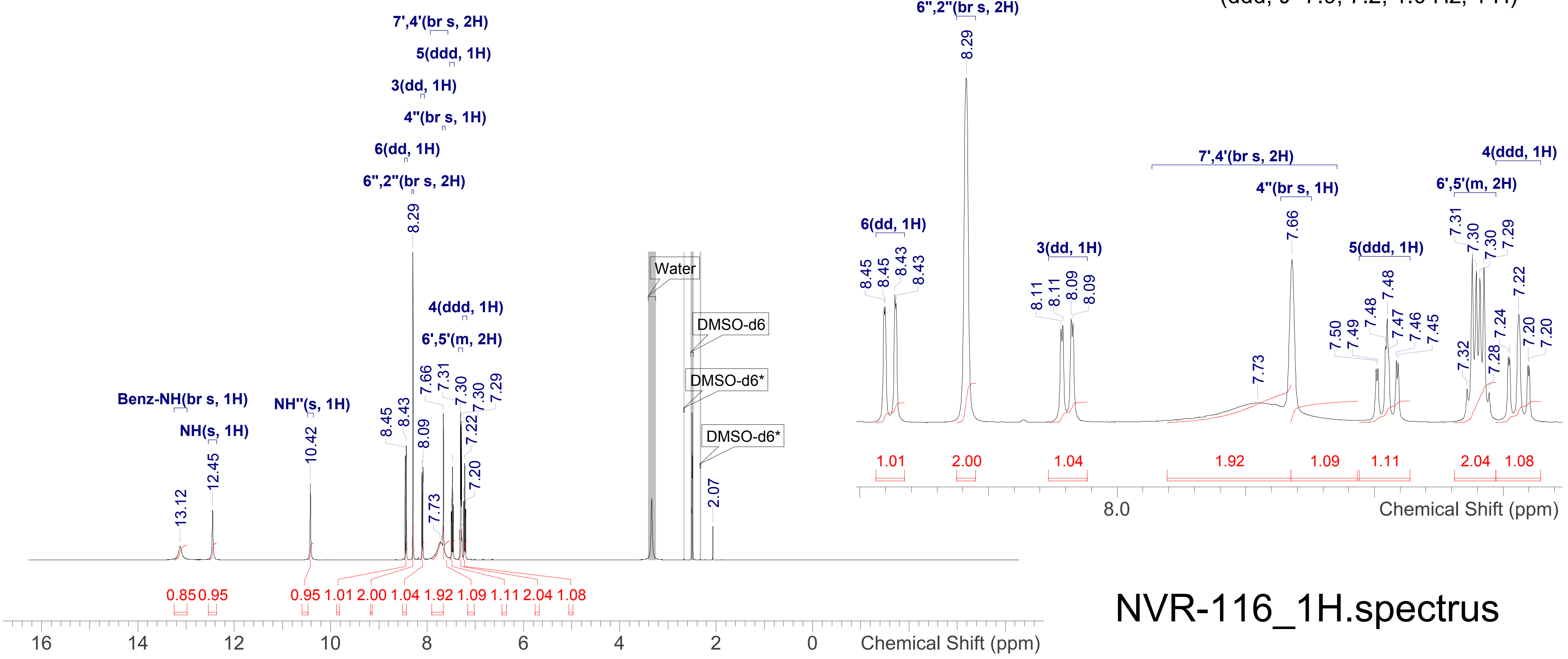

NVR-116_1H.spectrus

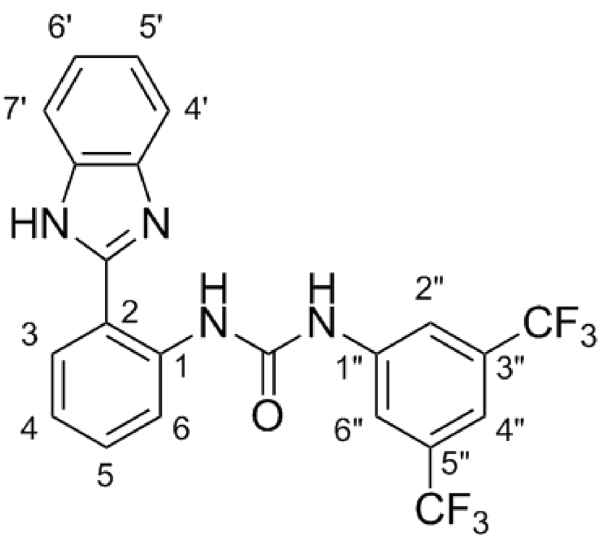

NVR-116_13C

Shift (ppm)	C	m	J (Hz)	Assign
152.5	1	s	-	CO
150.9	1	s	-	2'
142.1	1	s	-	1"
138.7	1	s	-	1
130.6	2	q	32.3	5", 3"
130.4	1	s	-	5
127.3	1	s	-	3
126.1	2	q	272.9	3"-CF3, 5"-CF3
122.7	2	br s	-	6', 5'
122.0	1	s	-	4
120.4	1	s	-	6
119.3	1	s	-	2
118.3	2	br q	3.9	6", 2"
115.4	2	s	-	7', 4'
114.4	1	br spt	3.9	4"

Acquisition Time (sec)	1.0224
Date	10 May 2019 01:21:35
Date Stamp	10 May 2019 01:21:35
Frequency (MHz)	100.5977
Nucleus	13C
Number of Transients	256
Solvent	DMSO-d6
Temperature (degree C)	24.999

^{13}C NMR (101 MHz, *DMSO-d_6*) δ ppm 152.5 (s, 1 C), 150.9 (s, 1 C), 142.1 (s, 1 C), 138.7 (s, 1 C), 130.4 (s, 1 C), 130.6 (q, *J*=32.3 Hz, 2 C), 127.3 (s, 1 C), 122.7 (br s, 2 C), 126.1 (q, *J*=272.9 Hz, 2 C), 122.0 (s, 1 C), 120.4 (s, 1 C), 119.3 (s, 1 C), 118.3 (br q, *J*=3.9 Hz, 2 C), 115.4 (s, 2 C), 114.4 (br spt, *J*=3.9 Hz, 1 C)

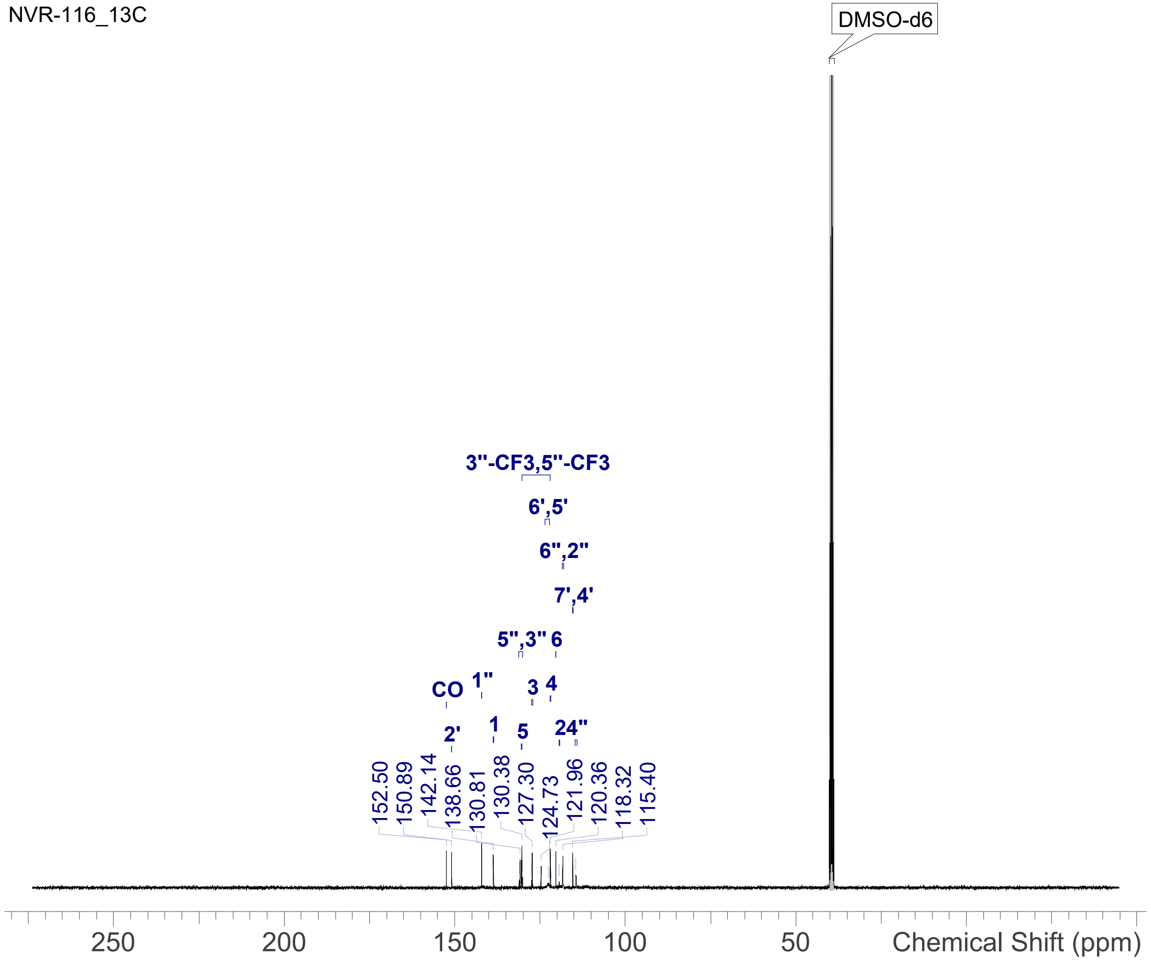

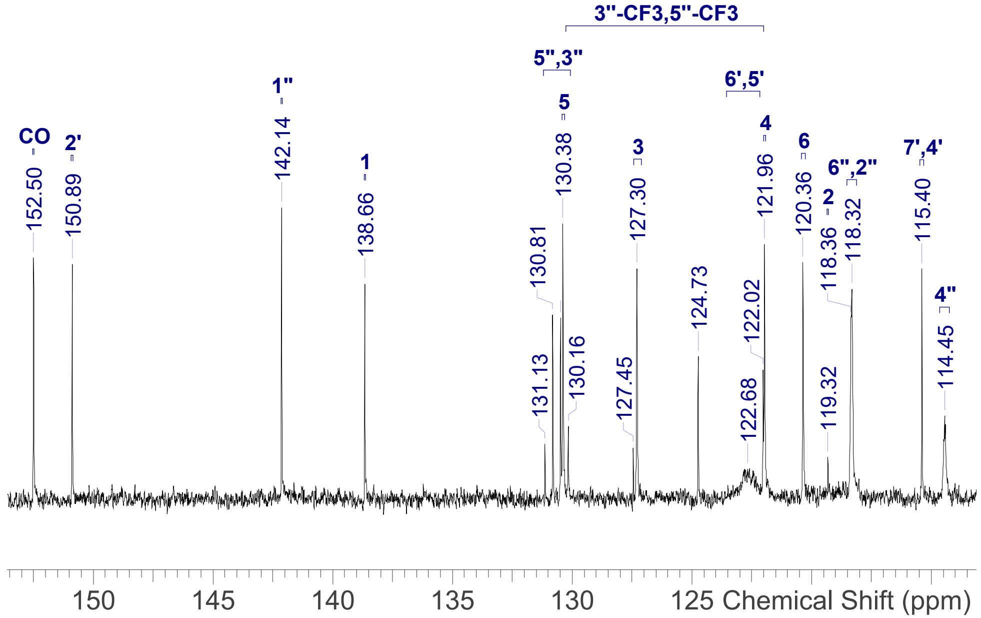

NVR-116_13C.spectrus

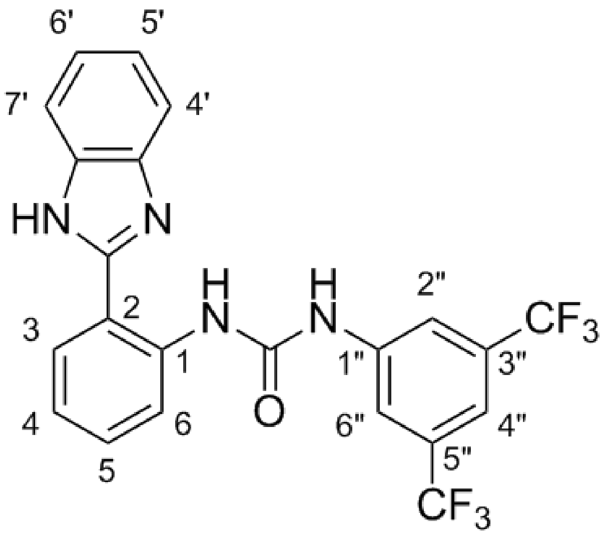

Shift (ppm)	F	m
-61.57	6	s

Acquisition Time (sec)	1.4680
Date	05 Mar 2019 22:24:19
Date Stamp	05 Mar 2019 22:24:19
Frequency (MHz)	376.4419
Nucleus	19F
Number of Transients	128
Solvent	DMSO-d6
Temperature (degree C)	25.000

^{19}F NMR (376 MHz, *DMSO-d_6*) δ ppm -61.57 (s, 6 F)

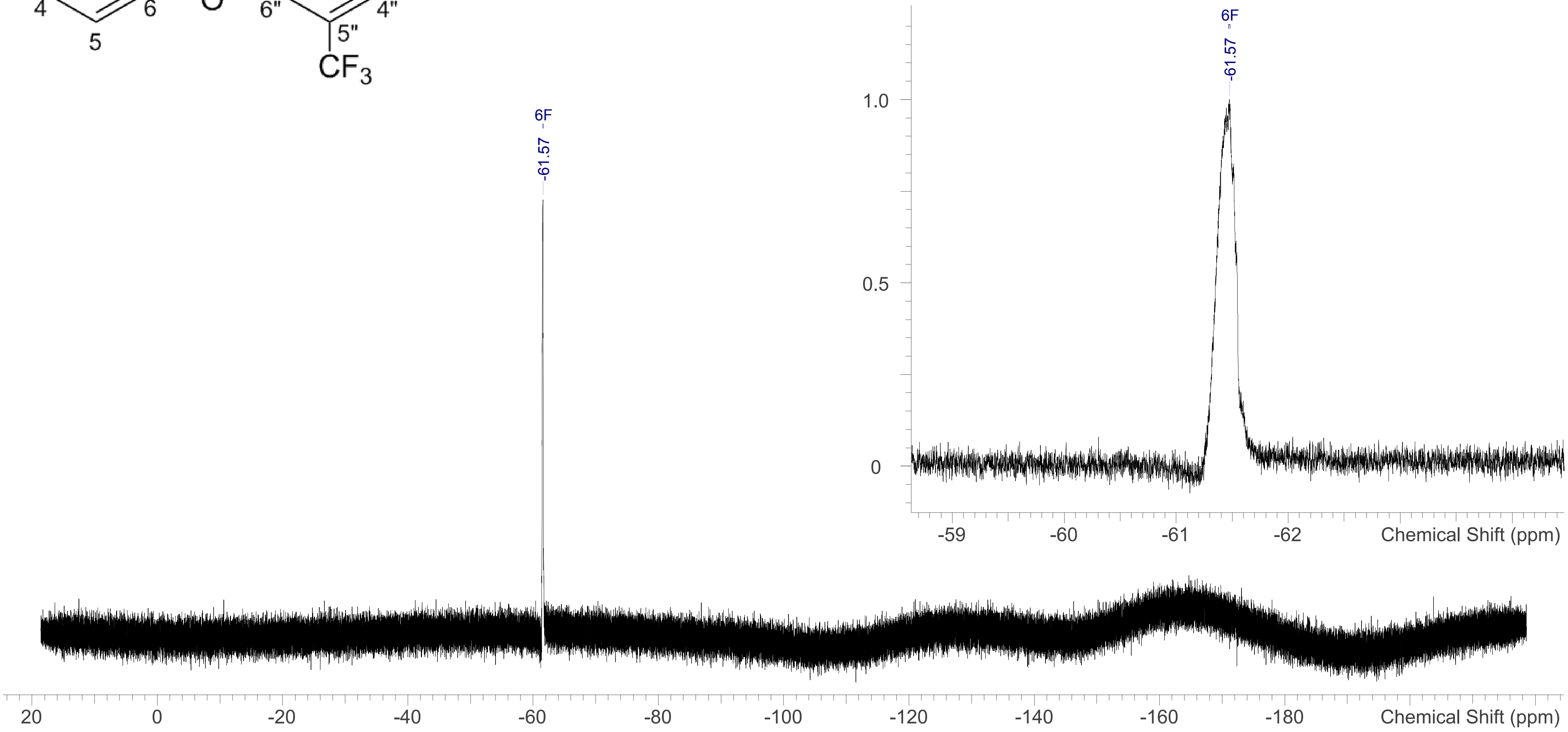

NVR-116_19F.spectrus

Openlynx Report - James B

Sample: 1 Vial:2:34 ID:JB139 HRMS-1

File:JB139 HRMS-1 Date:26-Sep-2019 Time:12:04:41

Description:

Printed: Thu Sep 26 12:44:25 2019

3: UV Detector: TAC: Wavelength Range: (210 - 400)

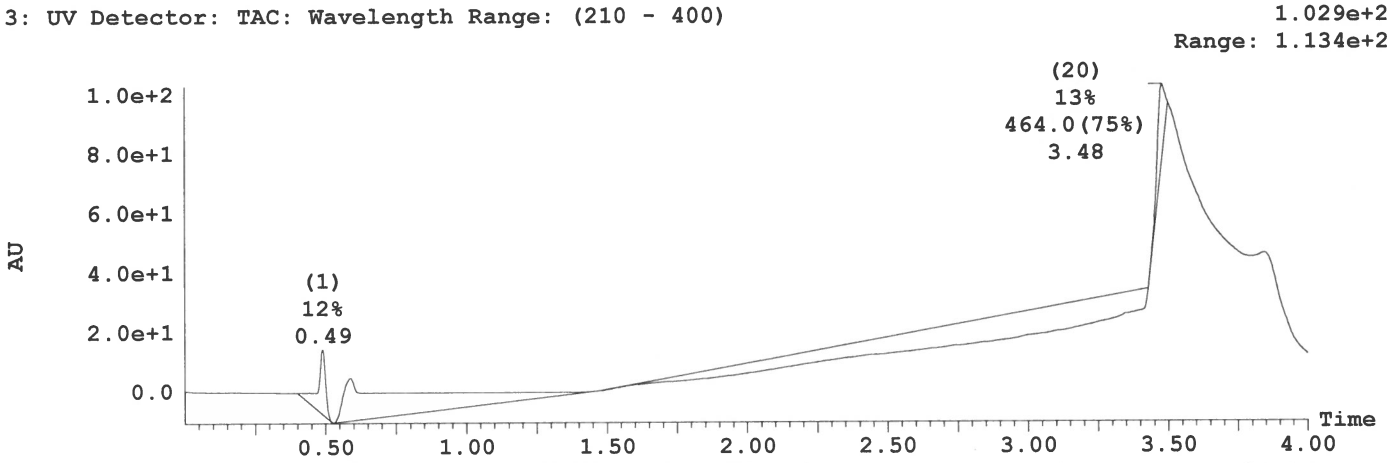

Peak Number	Compound	Time	Area %Total	Mass Found
1		0.49	11.90	Not Found
2		0.59	74.90	Not Found
20	Found	3.48	13.20	464.0

1: MS ES+ :TIC Smooth (Mn, 2x2)

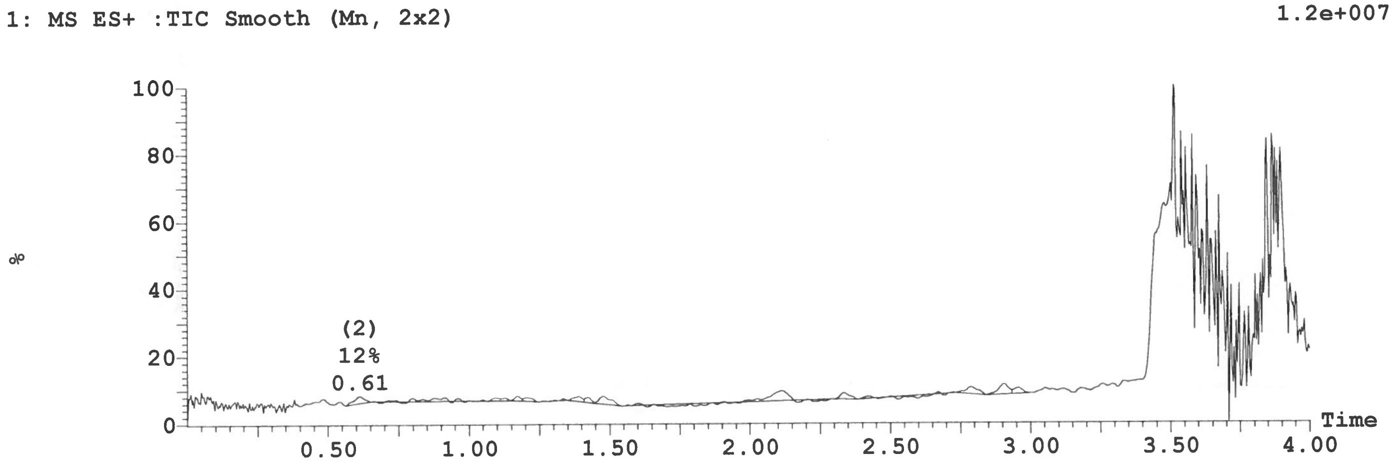

2: MS ES- :TIC Smooth (Mn, 2x2)

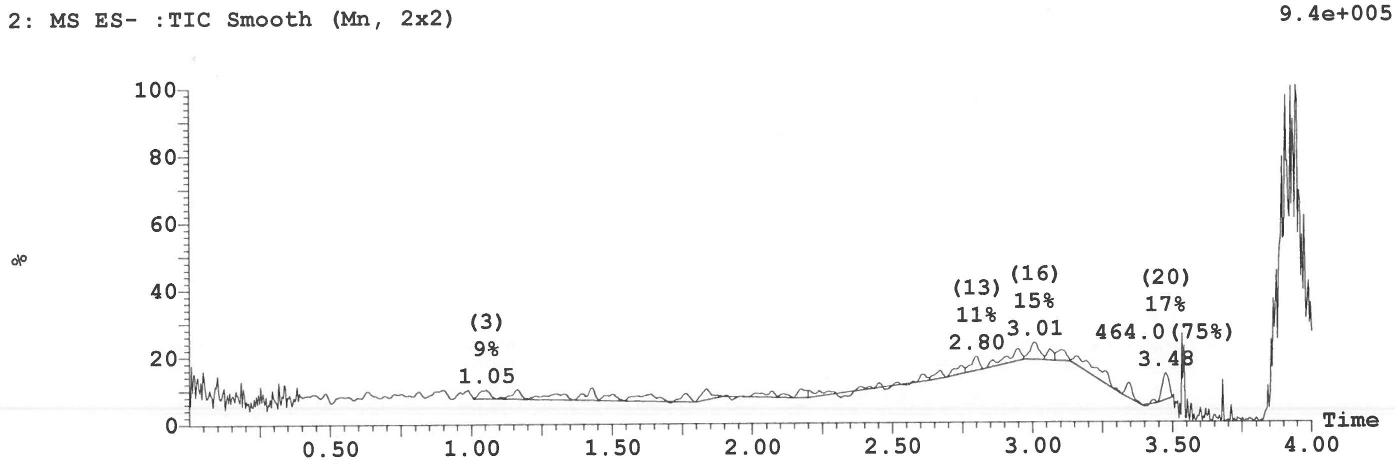

NVR-116 LCMS

Sample: 1 Vial:2:34 ID:JB139 HRMS-1
File:JB139 HRMS-1 Date:26-Sep-2019 Time:12:04:41
Description:

Printed: Thu Sep 26 12:44:25 2019

Peak ID	Compound	Time	Mass Found
1		0.49	Not Found

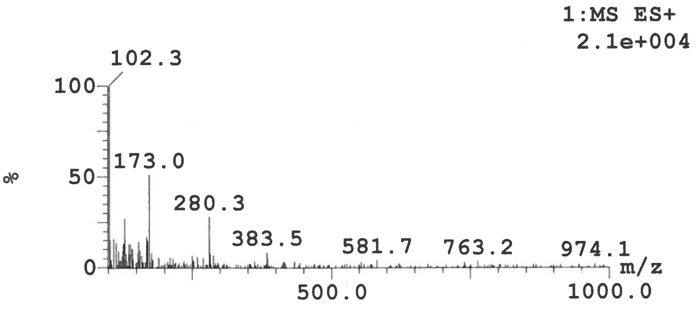

Peak ID	Compound	Time	Mass Found
1		0.49	Not Found

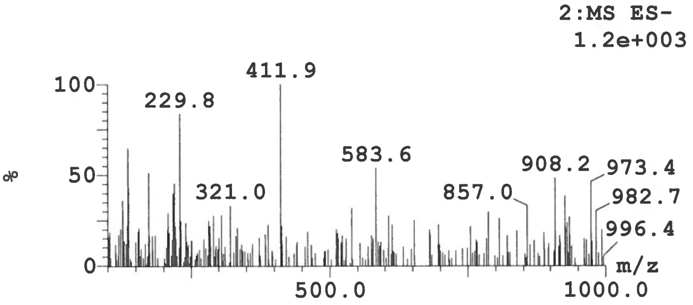

Peak ID	Compound	Time	Mass Found
1		0.49	Not Found

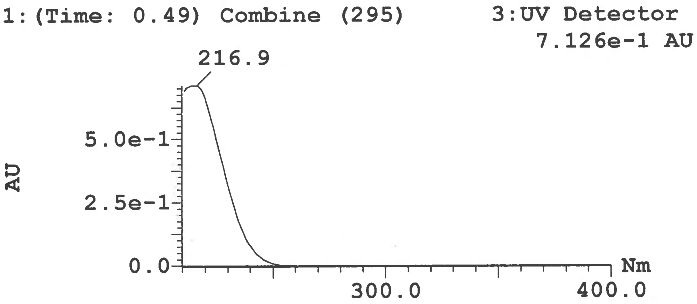

Peak ID	Compound	Time	Mass Found
2		0.61	Not Found

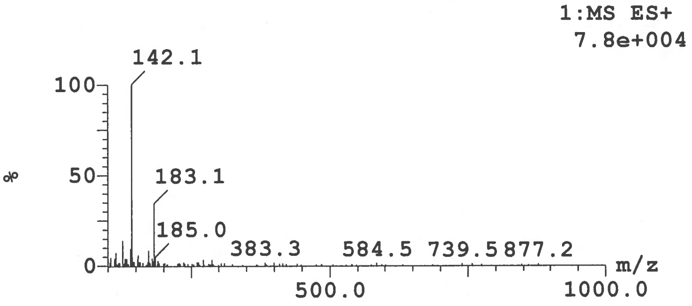

Peak ID	Compound	Time	Mass Found
2		0.61	Not Found

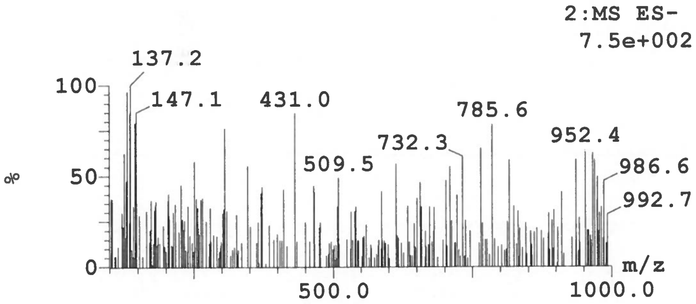

Peak ID	Compound	Time	Mass Found
2		0.61	Not Found

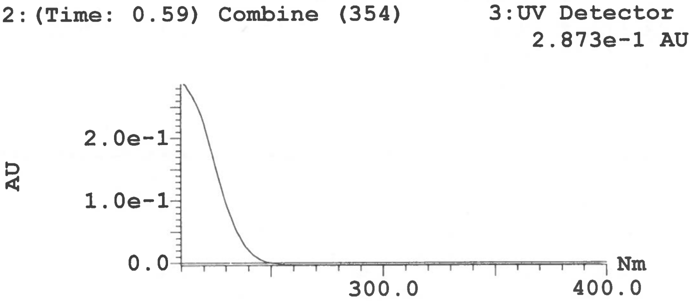

Peak ID	Compound	Time	Mass Found
20	Found	3.48	465

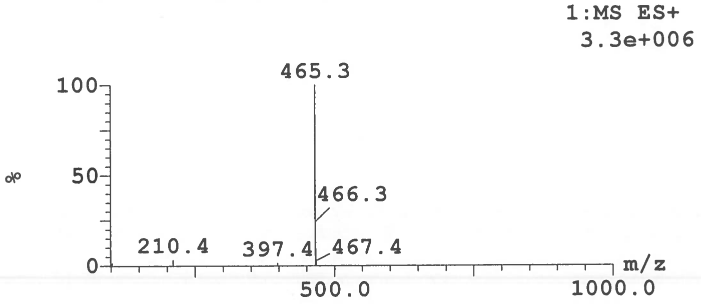

Peak ID	Compound	Time	Mass Found
20		3.48	Not Found

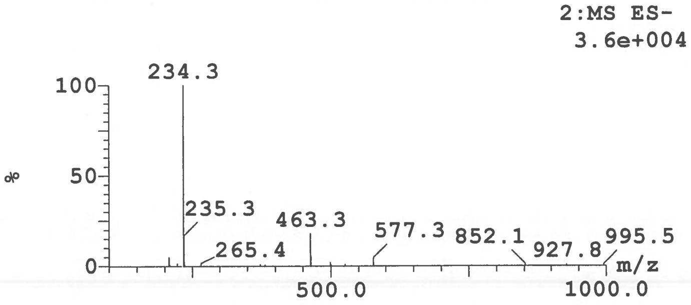

NVR-116 LCMS

Openlynx Report - James B

Sample: 1 | Vial:2:34 | ID:JB139 HRMS-1
File:JB139 HRMS-1 | Date:26-Sep-2019 | Time:12:04:41
Description:

Printed: Thu Sep 26 12:44:25 2019

Peak ID	Compound	Time	Mass Found
20		3.48	Not Found

20: (Time: 3.48) Combine (2088) 3:UV Detector
1.165 AU

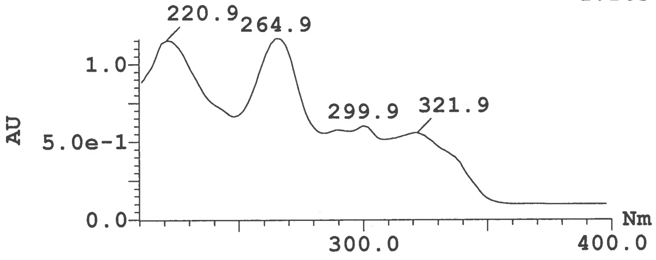

NVR-116 LCMS

Thermo Exactive Plus EMR Orbitrab HESI neg

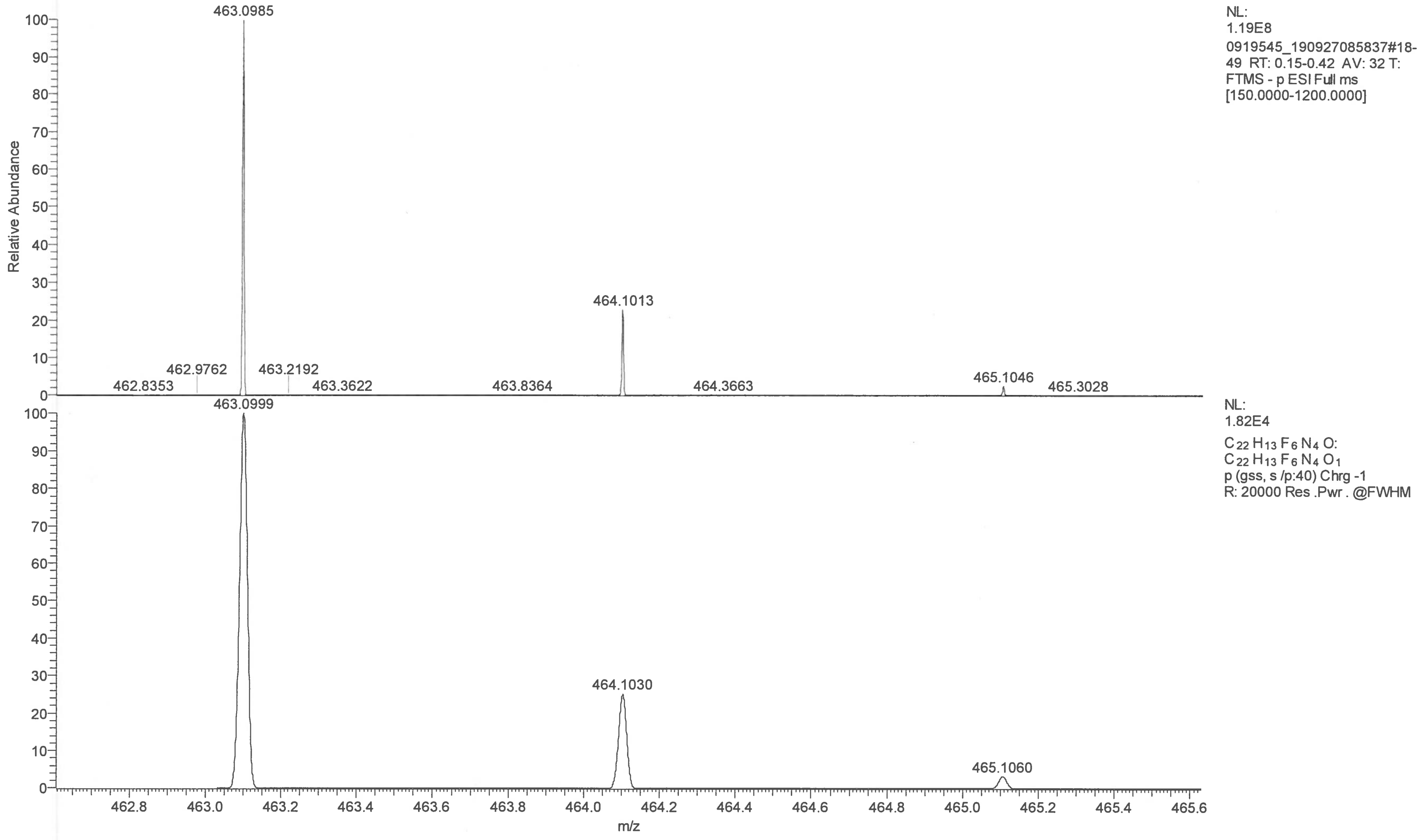

NVR-116 HRMS

Thermo Exactive Plus EMR Orbitrab HESI neg

m/z	Theo. Mass	Delta (ppm)	RDB equiv.	Composition
463.0985	463.0986	-0.13	16.0	C20 H11 N7 F6
	463.0986	-0.14	10.5	C21 H17 O5 F6
	463.0972	2.76	11.0	C19 H15 O4 N3 F6
	463.0999	-3.03	15.5	C22 H13 O N4 F6 ←
	463.1004	-4.13	3.0	C8 H15 O7 N9 F6

NVR-116 HRMS

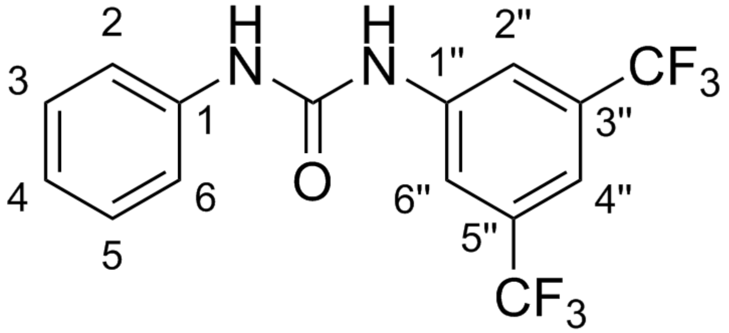

Shift (ppm)	H	m	J (Hz)	Assign
9.37	1	s	-	NH''
8.96	1	s	-	NH
8.13	2	s	-	H2'', H6''
7.61	1	s	-	H4''
7.48	2	br d	7.6	H2, H6
7.30	2	t	7.9	H5, H3
7.01	1	t	7.3	H4

Acquisition Time (sec)	6.5536
Date	20/07/2017 07:19:00
Date Stamp	20/07/2017 07:19:00
Frequency (MHz)	500.1930
Nucleus	1H
Number of Transients	128
Solvent	ACETONITRILE-d3

^{1}H NMR (500 MHz, *ACETONITRILE-d_3*) δ ppm 9.37 (s, 1 H), 8.96 (s, 1 H), 8.13 (s, 2 H), 7.61 (s, 1 H), 7.48 (br d, *J*=7.6 Hz, 2 H), 7.30 (t, *J*=7.9 Hz, 2 H), 7.01 (t, *J*=7.3 Hz, 1 H)

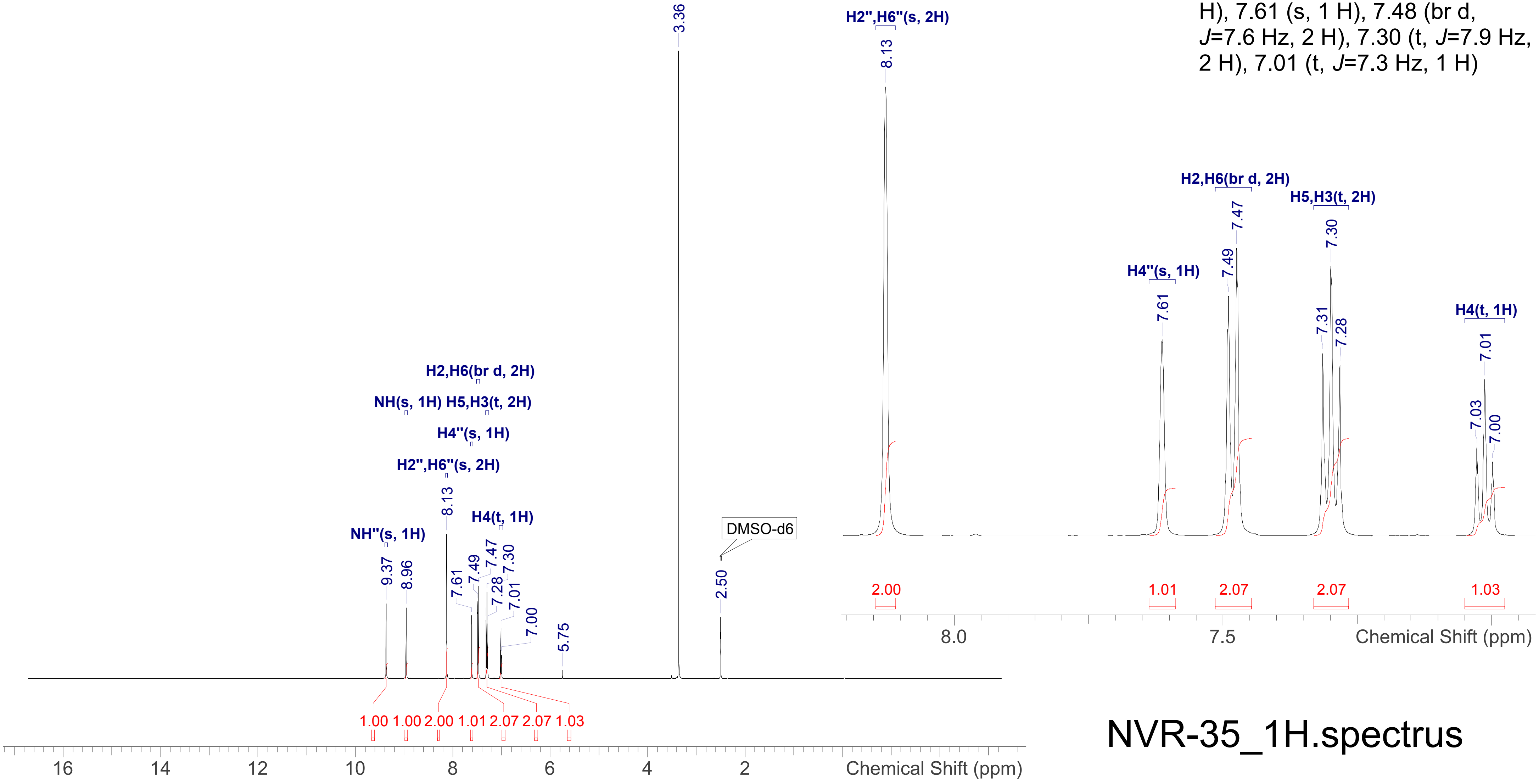

NVR-35_1H.spectrus

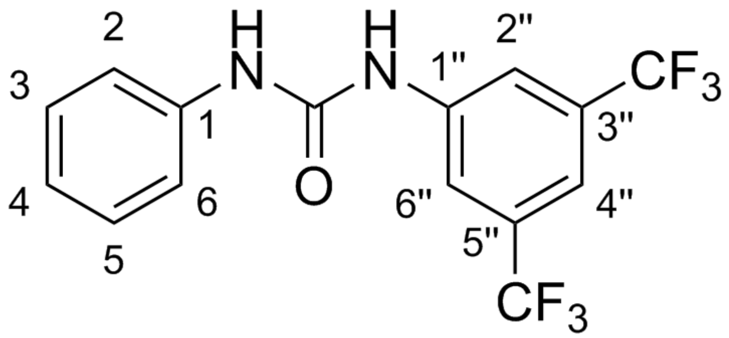

Shift (ppm)	C	m	J (Hz)	Assign
152.4	1	s	-	CO
141.9	1	s	-	1
139.0	1	s	-	1''
130.7	2	q	32.6	5'', 3''
128.8	2	s	-	5, 3
123.3	2	q	272.9	3''-CF3, 5''-CF3
122.5	1	s	-	4
118.9	2	s	-	2, 6
117.9	2	br q	3.9	6'', 2''
114.3	1	br spt	3.9	4''

Acquisition Time (sec)	2.0447
Date	25/07/2017 08:50:00
Date Stamp	25/07/2017 08:50:00
Frequency (MHz)	125.7870
Nucleus	13C
Number of Transients	2048
Solvent	DMSO-d6

^{13}C NMR (126 MHz, *DMSO-d_6*) δ ppm 152.4 (s, 1 C), 141.9 (s, 1 C), 139.0 (s, 1 C), 130.7 (q, *J*=32.6 Hz, 2 C), 128.8 (s, 2 C), 122.5 (s, 1 C), 123.3 (q, *J*=272.9 Hz, 2 C), 118.9 (s, 2 C), 117.9 (br q, *J*=3.9 Hz, 2 C), 114.3 (br spt, *J*=3.9 Hz, 1 C)

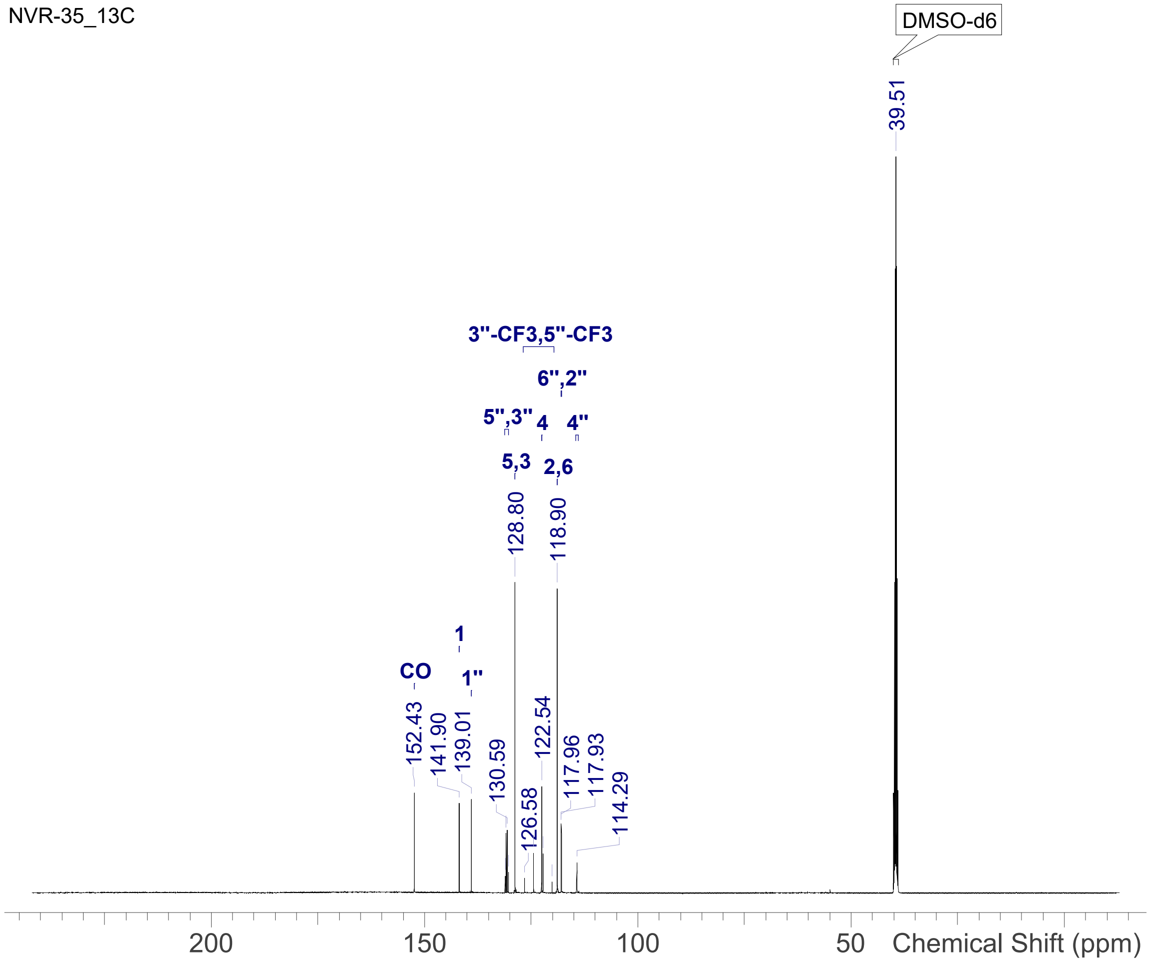

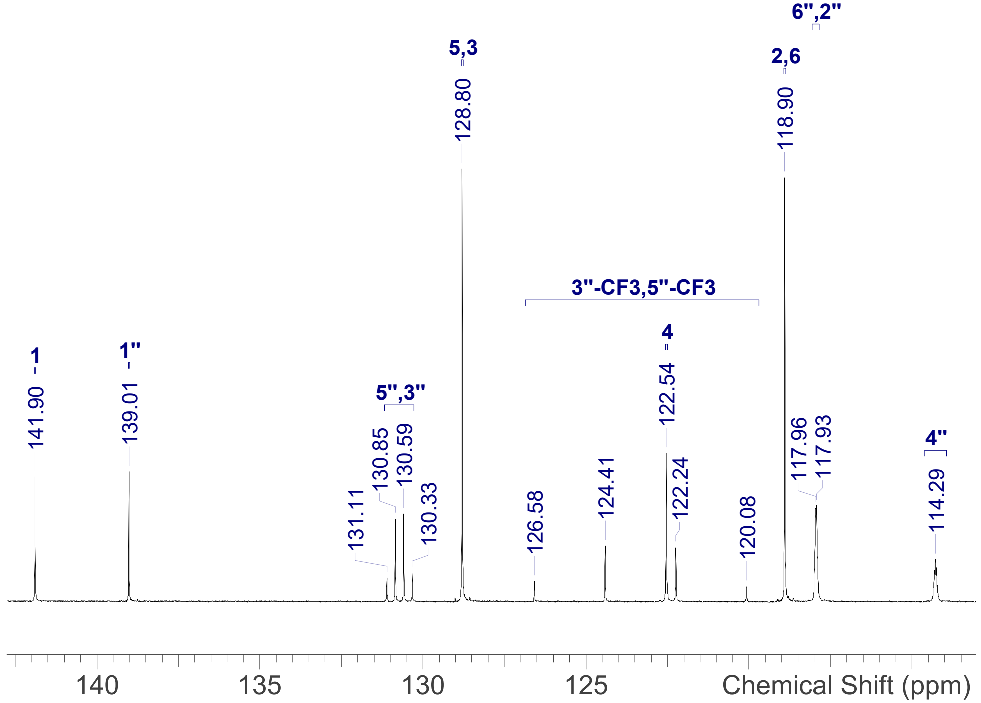

NVR-35_13C.spectrus

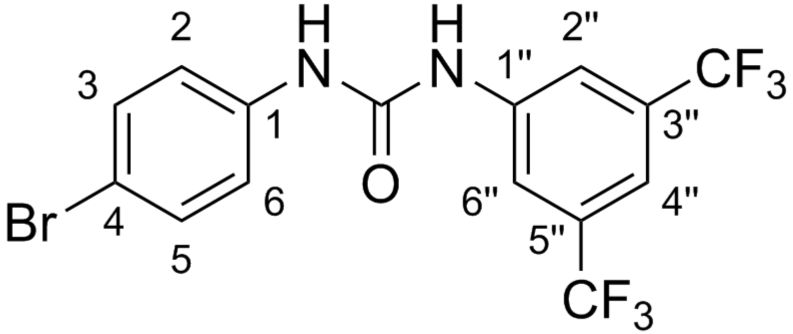

Shift (ppm)	H	m	Assign
9.41	1	s	NH''
9.12	1	s	NH
8.12	2	s	6'', 2''
7.62	1	s	4''
7.46	4	s	5, 3, 2, 6

Acquisition Time (sec)	6.5536
Date	20/07/2017 09:45:00
Date Stamp	20/07/2017 09:45:00
Frequency (MHz)	500.1930
Nucleus	1H
Number of Transients	128
Solvent	DMSO-d6

^{1}H NMR (500 MHz, *DMSO-d_6*) δ ppm 9.41 (s, 1 H), 9.12 (s, 1 H), 8.12 (s, 2 H), 7.62 (s, 1 H), 7.46 (s, 4 H)

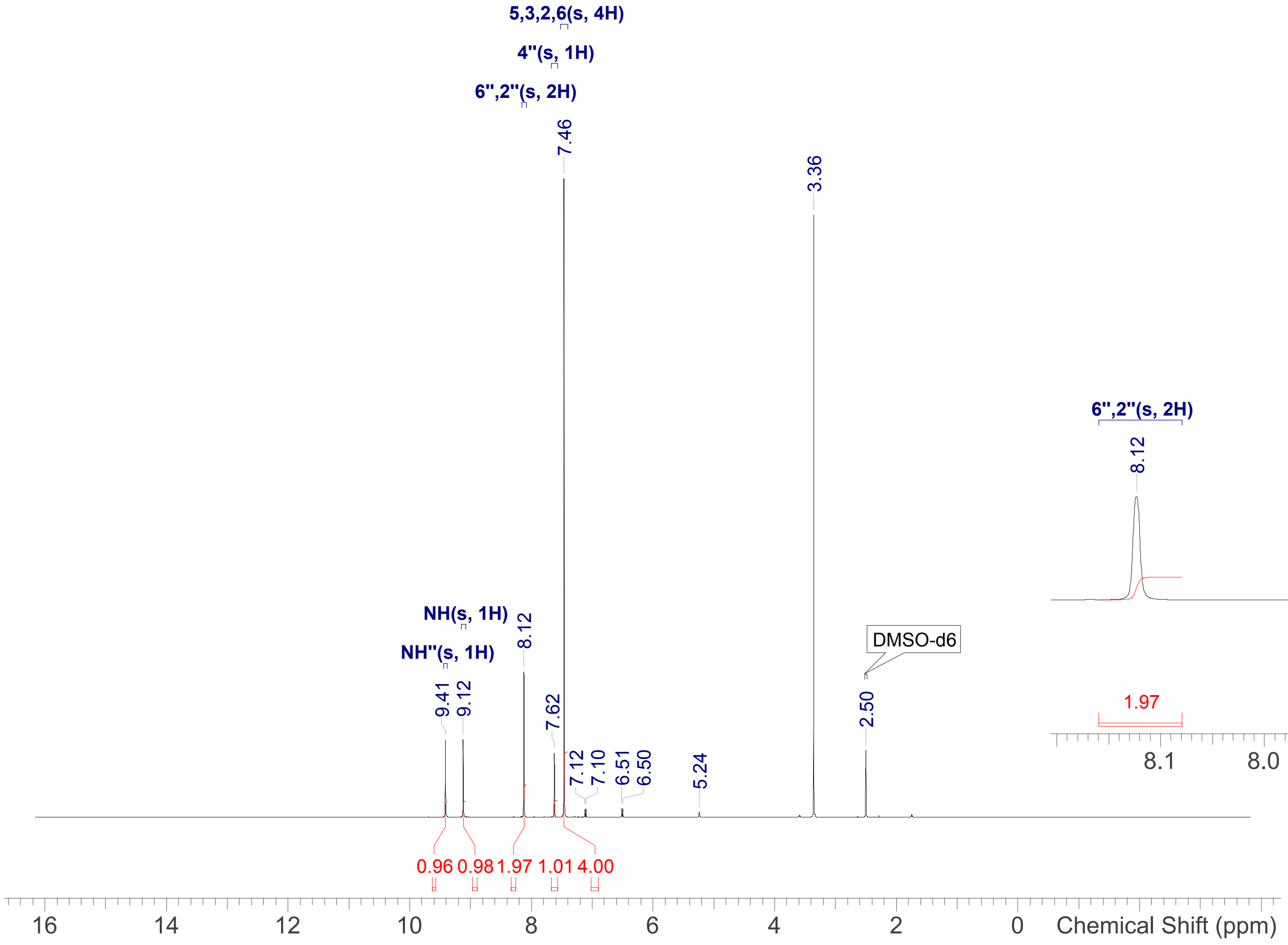

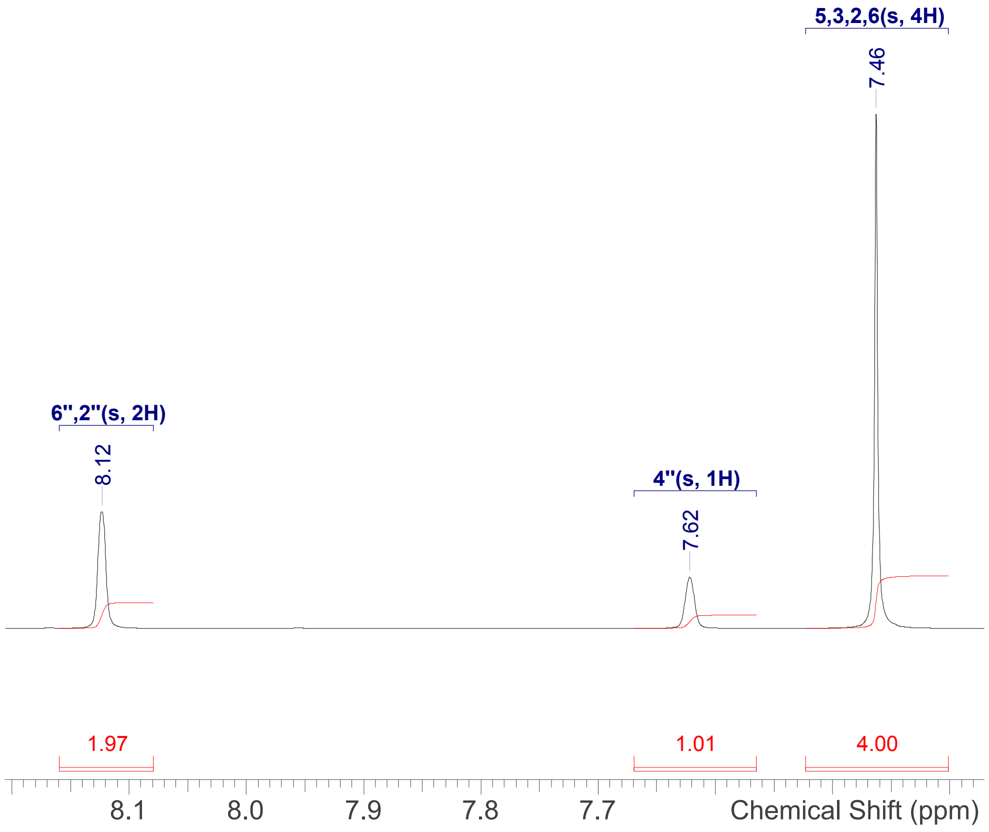

NVR-36_1H.spectrus

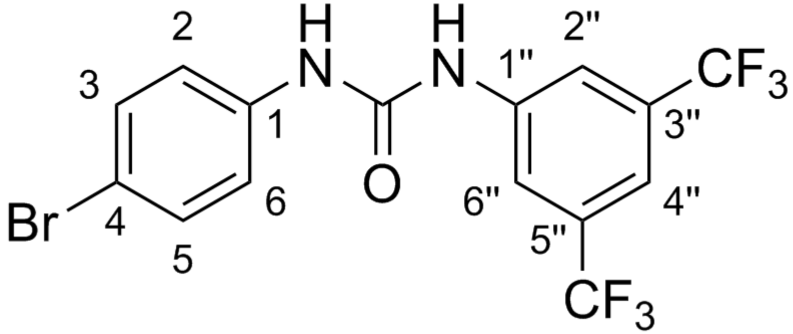

Shift (ppm)	C	m	J (Hz)	Assign
152.3	1	s	-	7''
141.7	1	s	-	1
138.5	1	s	-	1''
131.5	2	s	-	5, 3
130.7	2	q	32.6	3'', 5''
123.3	2	q	272.4	5''-CF3, 3''-CF3
120.8	2	s	-	2, 6
118.1	2	br q	3.9	2'', 6''
114.5	1	br spt	3.9	4''
114.0	1	s	-	4

Acquisition Time (sec)	2.0447
Date	20/07/2017 22:54:00
Date Stamp	20/07/2017 22:54:00
Frequency (MHz)	125.7870
Nucleus	13C
Number of Transients	2048
Solvent	DMSO-d6

^{13}C NMR (126 MHz, *DMSO-d_6*) δ ppm 152.3 (s, 1 C), 141.7 (s, 1 C), 138.5 (s, 1 C), 131.5 (s, 2 C), 130.7 (q, *J*=32.6 Hz, 2 C), 120.8 (s, 2 C), 123.3 (q, *J*=272.4 Hz, 2 C), 118.1 (br q, *J*=3.9 Hz, 2 C), 114.5 (br spt, *J*=3.9 Hz, 1 C), 114.0 (s, 1 C)

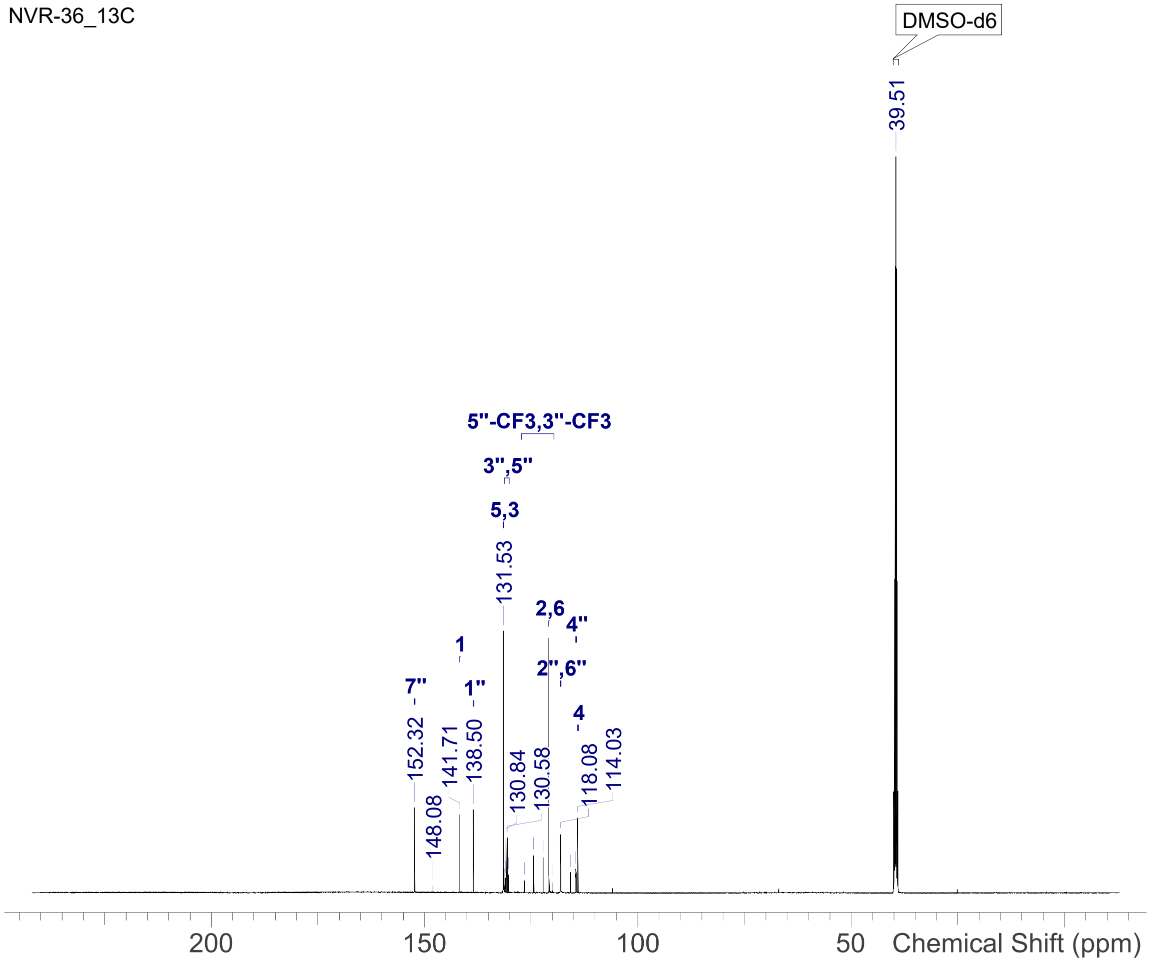

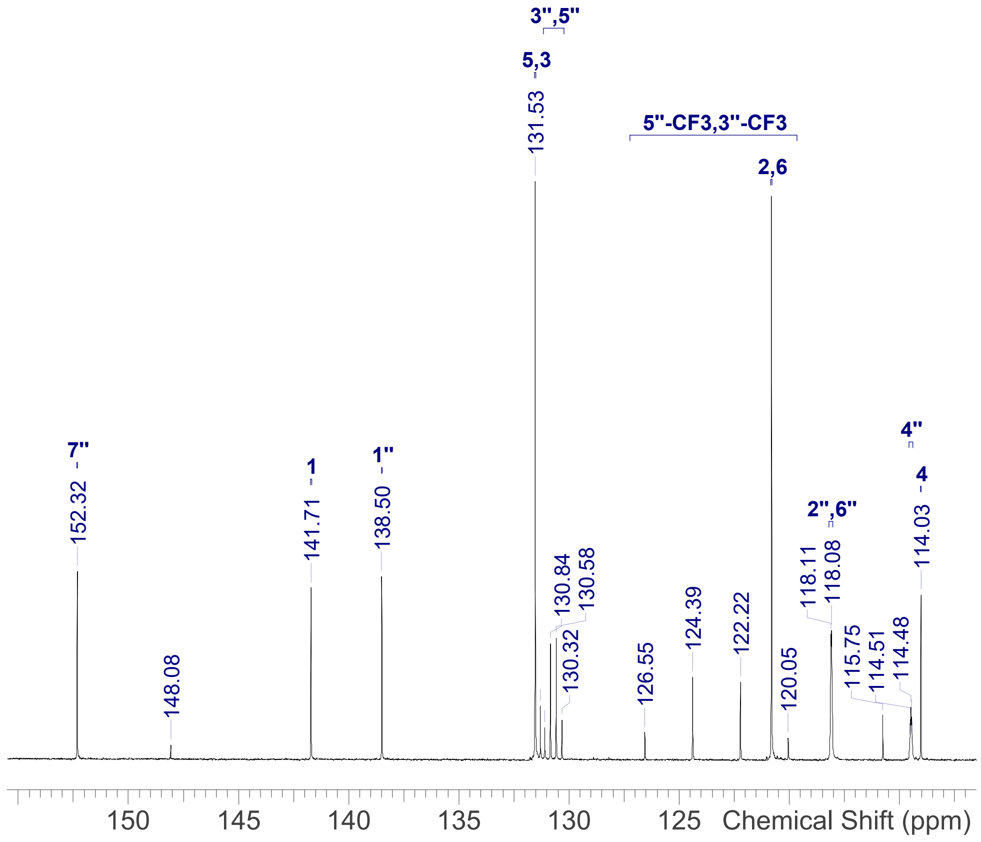

NVR-36_13C.spectrus

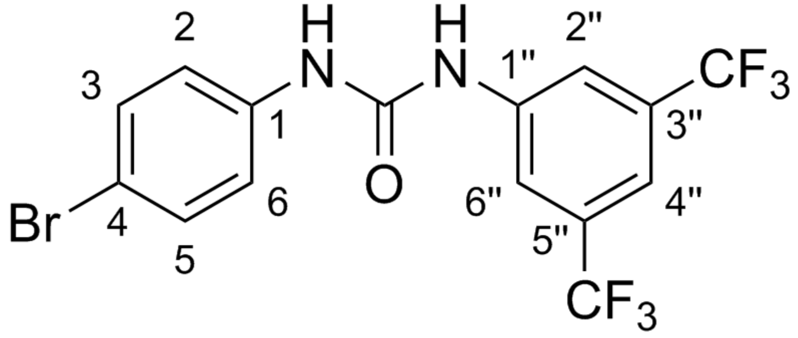

Shift (ppm)	F	m
-61.74	6	s

Acquisition Time (sec)	2.9360
Date	20/07/2017 23:01:00
Date Stamp	20/07/2017 23:01:00
Frequency (MHz)	470.6020
Nucleus	19F
Number of Transients	128
Solvent	DMSO-d6

^{19}F NMR (471 MHz, *DMSO-d_6*) δ ppm -61.74 (s, 6 F)

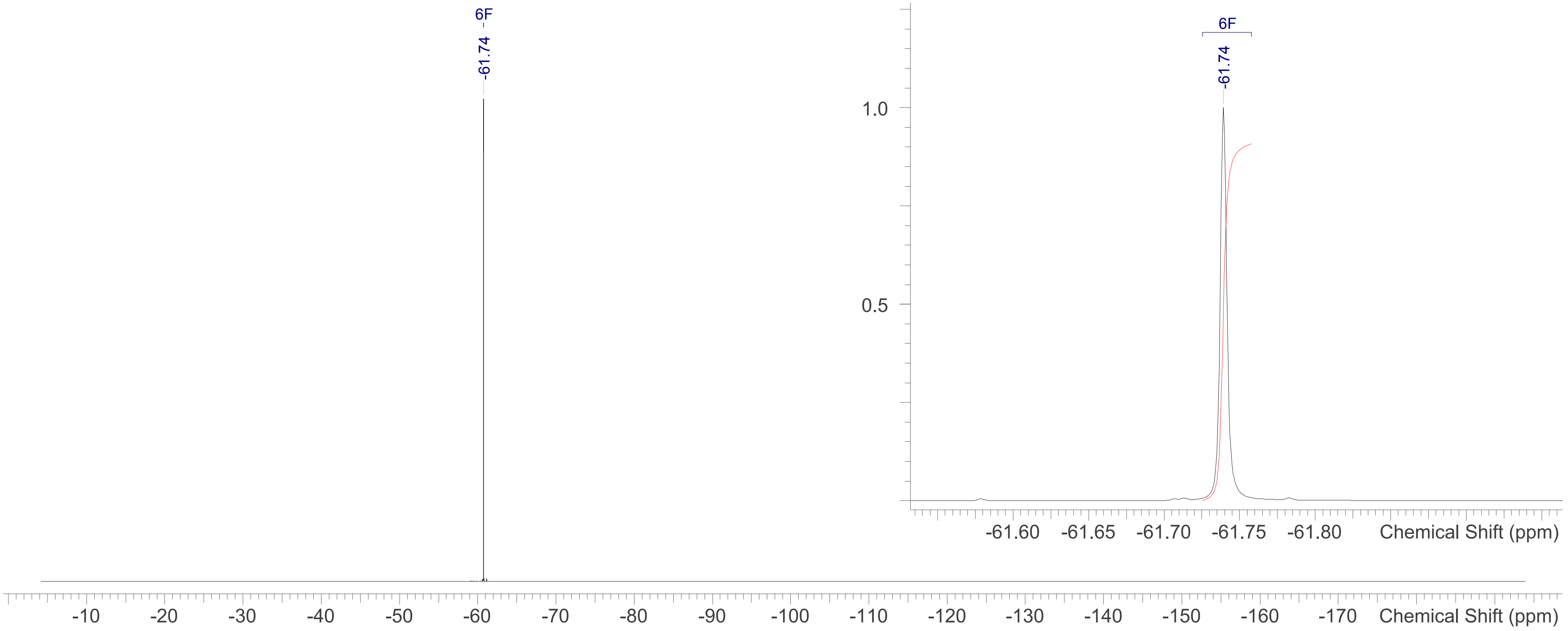

NVR-36_19F.spectrus

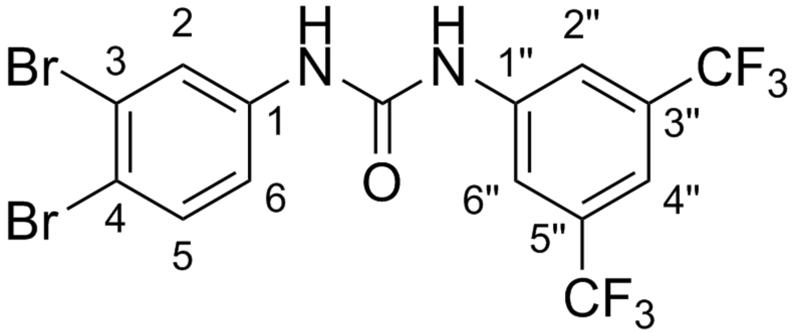

No.	Shift (ppm)	H	m	J (Hz)	Assign
1	9.51	1	s	-	NH
2	9.28	1	s	-	NH"
3	8.13	2	s	-	2", 6"
4	8.02	1	d	2.4	2
5	7.64	2	m	-	5, 4"
6	7.36	1	dd	8.8, 2.4	6

Acquisition Time (sec)	7.9692
Date	05/07/2019 15:49:00
Date Stamp	05/07/2019 15:49:00
Frequency (MHz)	400.0720
Nucleus	1H
Number of Transients	4
Solvent	DMSO-d6

^{1}H NMR (400 MHz, *DMSO-d_6*) δ ppm 9.51 (s, 1 H), 9.28 (s, 1 H), 8.13 (s, 2 H), 8.02 (d, *J*=2.4 Hz, 1 H), 7.62 - 7.67 (m, 2 H), 7.36 (dd, *J*=8.8, 2.4 Hz, 1 H)

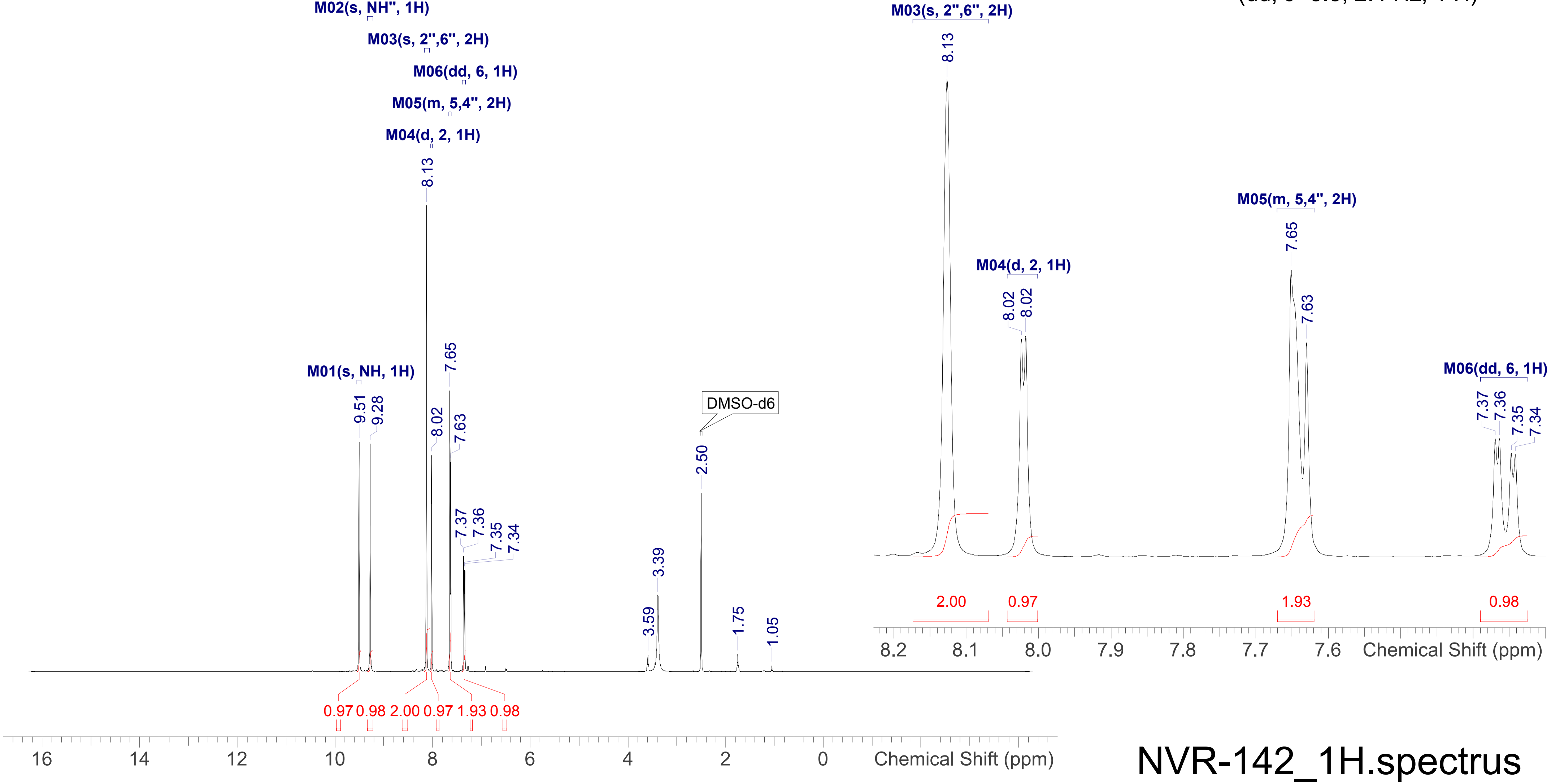

NVR-142_1H.spectrus

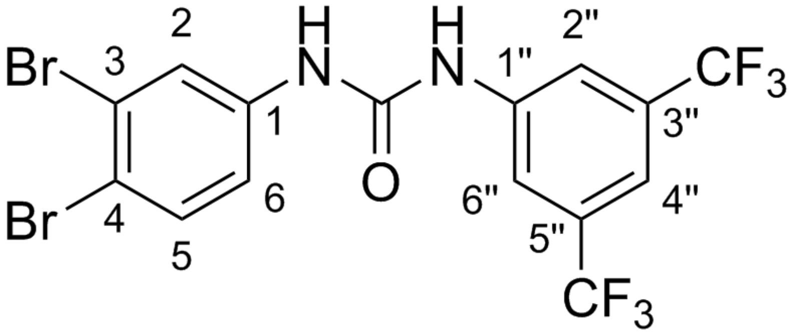

Shift (ppm)	C	m	J (Hz)	Assign
152.2	1	s	-	CO
141.5	1	s	-	1''
139.8	1	s	-	1
133.6	1	s	-	5
130.7	2	q	32.9	3'', 5''
123.8	1	s	-	3
123.3	2	q	272.6	5''-CF3, 3''-CF3
123.1	1	s	-	2
119.7	1	s	-	6
118.3	2	br q	3.9	2'', 6''
115.9	1	s	-	4
114.7	1	br spt	3.9	4''

Acquisition Time (sec)	1.0224
Date	05 Jul 2019 15:51:12
Date Stamp	05 Jul 2019 15:51:12
Frequency (MHz)	100.5977
Nucleus	13C
Number of Transients	16
Solvent	DMSO-d6
Temperature (degree C)	24.999

^{13}C NMR (101 MHz, *DMSO-d_6*) δ ppm 152.2 (s, 1 C), 141.5 (s, 1 C), 139.8 (s, 1 C), 133.6 (s, 1 C), 130.7 (q, *J*=32.9 Hz, 2 C), 123.8 (s, 1 C), 123.1 (s, 1 C), 119.7 (s, 1 C), 123.3 (q, *J*=272.6 Hz, 2 C), 118.3 (br q, *J*=3.9 Hz, 2 C), 115.9 (s, 1 C), 114.7 (br spt, *J*=3.9 Hz, 1 C)

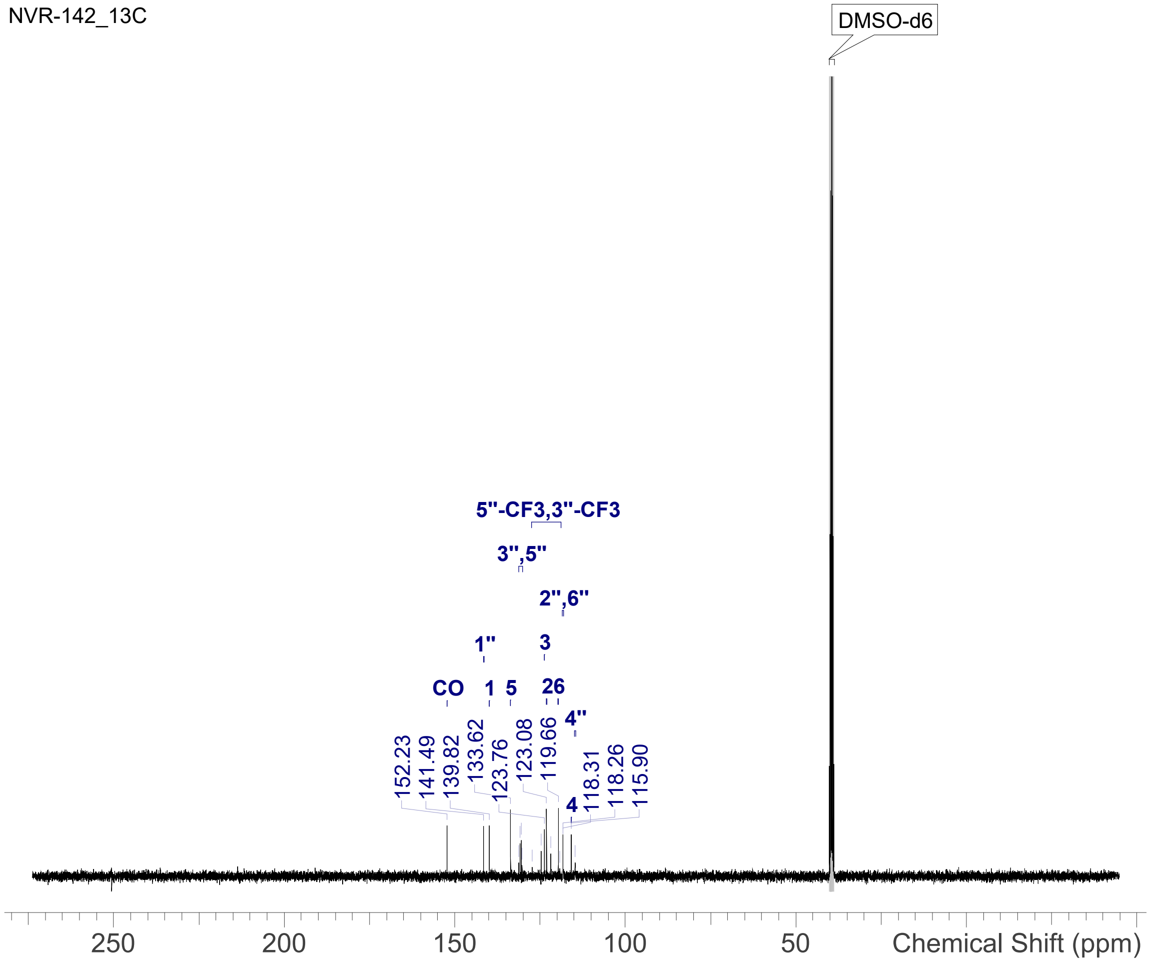

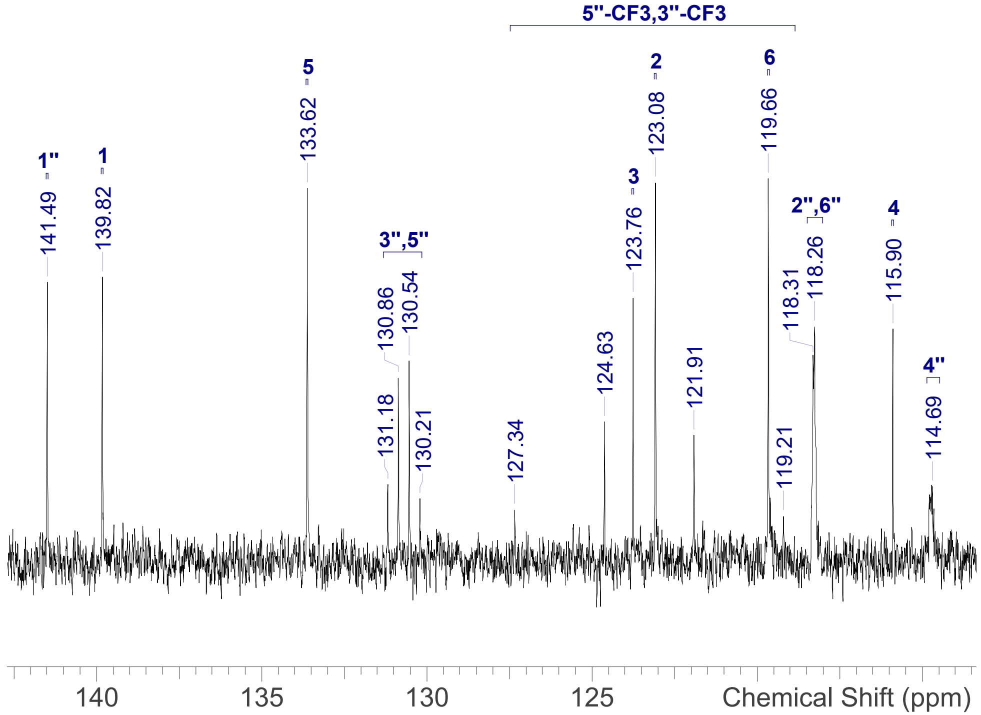

NVR-142_13C.spectrus

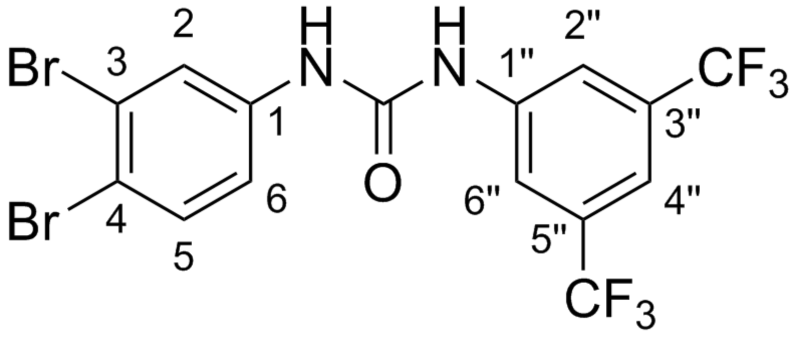

Shift (ppm)	F	m
-61.70	6	s

Acquisition Time (sec)	1.4680
Date	05 Jul 2019 15:57:44
Date Stamp	05 Jul 2019 15:57:44
Frequency (MHz)	376.4419
Nucleus	19F
Number of Transients	16
Solvent	DMSO-d6
Temperature (degree C)	25.001

^{19}F NMR (376 MHz, *DMSO-d$_6$*) δ ppm -61.70 (s, 6 F)

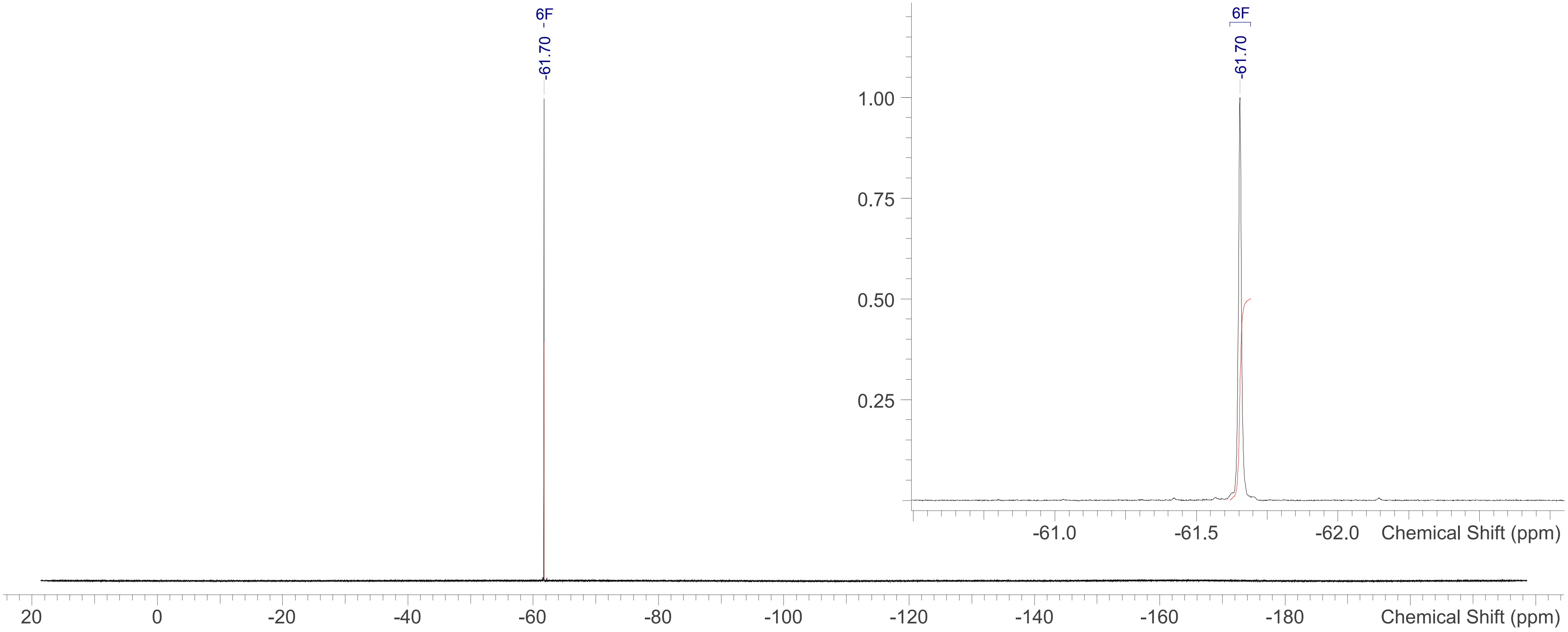

NVR-142_19F.spectrus

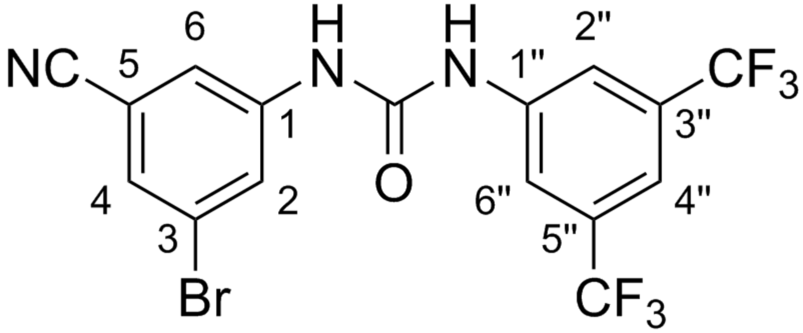

Shift (ppm)	H	m	Assign
9.68	1	br s	NH
9.46	1	br s	NH''
8.13	2	br s	2'', 6''
8.06	1	br s	4
7.88	1	br s	6
7.73	1	br s	2
7.67	1	br s	4''

Acquisition Time (sec)	3.9846
Date	05 Jul 2019 15:26:05
Date Stamp	05 Jul 2019 15:26:05
Frequency (MHz)	400.0700
Nucleus	1H
Number of Transients	4
Solvent	DMSO-d6
Temperature (degree C)	25.002

^{1}H NMR (400 MHz, *DMSO-d$_6$*) δ ppm 9.68 (br s, 1 H), 9.46 (br s, 1 H), 8.13 (br s, 2 H), 8.06 (br s, 1 H), 7.88 (br s, 1 H), 7.73 (br s, 1 H), 7.67 (br s, 1 H)

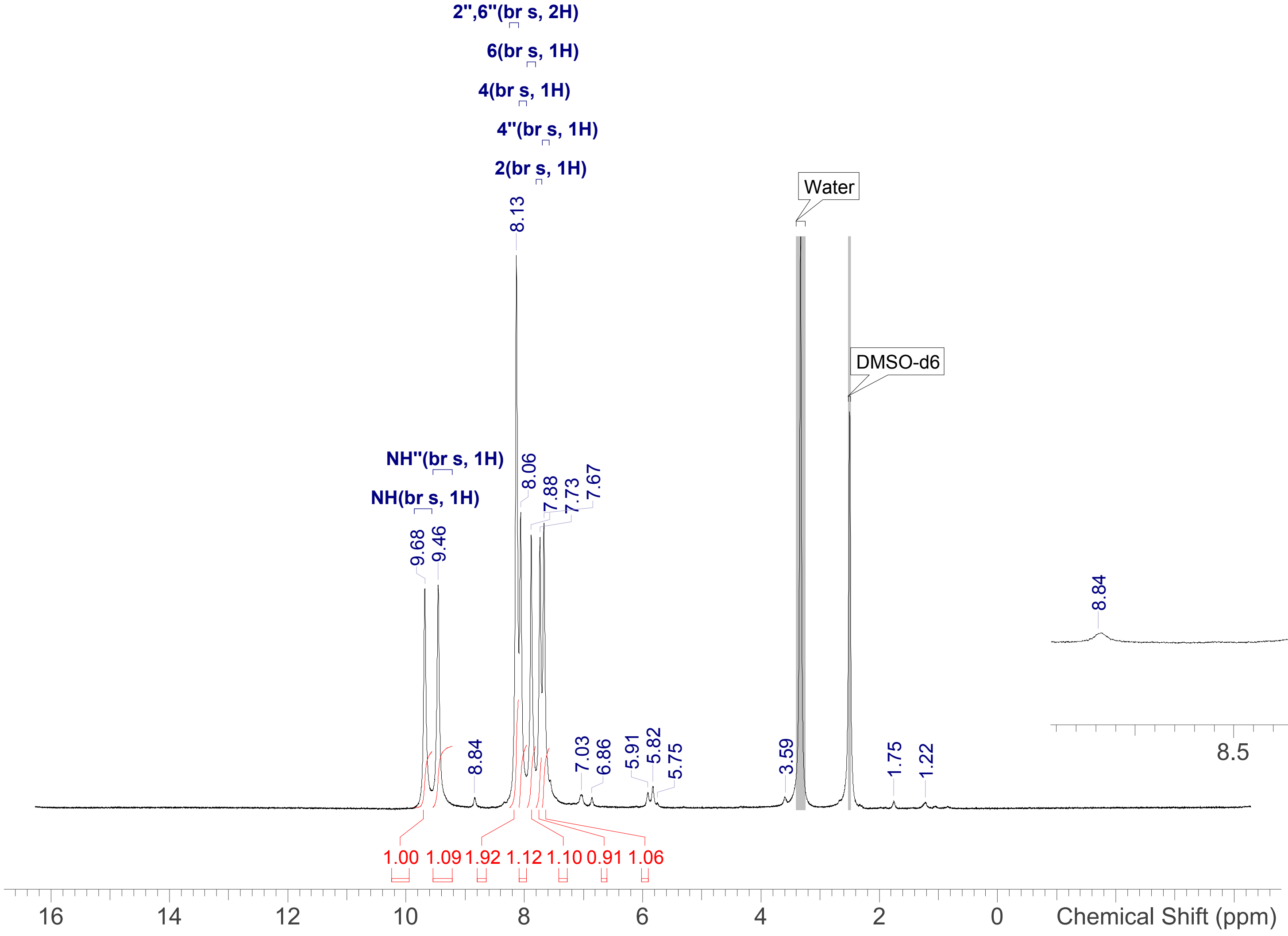

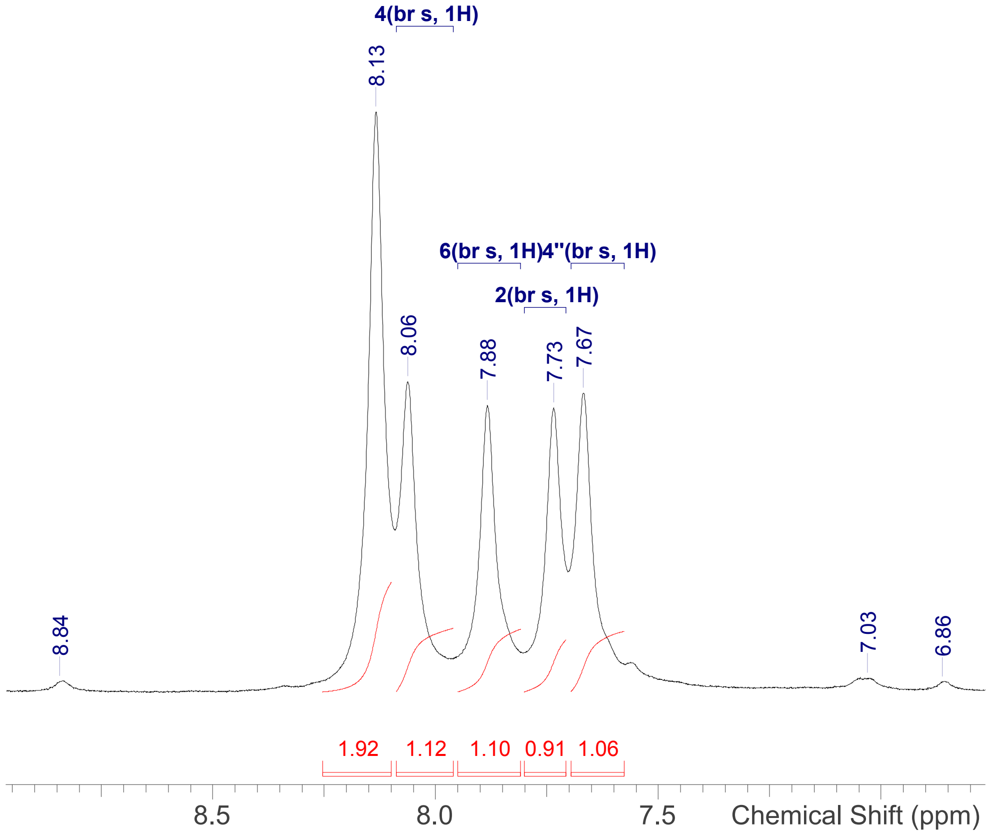

NVR-140_1H.spectrus

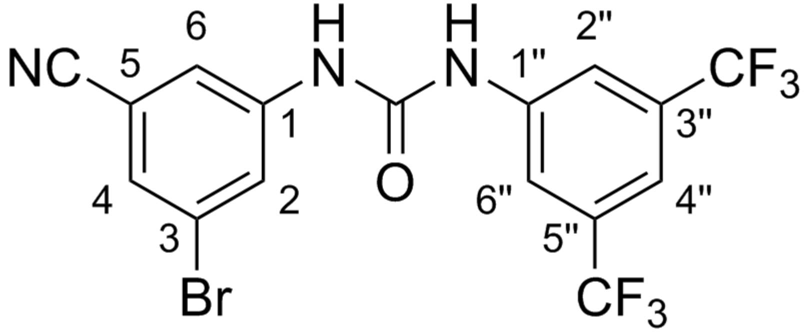

Shift (ppm)	C	m	J (Hz)	Assign
152.2	1	s	-	CO
141.4	1	s	-	1''
141.3	1	s	-	1
130.7	2	q	32.3	3'', 5''
127.8	1	s	-	4
125.7	1	s	-	2
123.4	2	br q	238.2	5''-CF3, 3''-CF3
122.3	1	s	-	3
120.8	1	s	-	6
118.4	2	br q	3.9	2'', 6''
117.3	1	br spt	3.9	CN
114.9	1	s	-	4''
113.4	1	s	-	5

Acquisition Time (sec)	1.0224
Date	05 Jul 2019 15:28:06
Date Stamp	05 Jul 2019 15:28:06
Frequency (MHz)	100.5977
Nucleus	13C
Number of Transients	16
Solvent	DMSO-d6
Temperature (degree C)	25.000

^{13}C NMR (101 MHz, *DMSO-d_6*) δ ppm 152.2 (s, 1 C), 141.4 (s, 1 C), 141.3 (s, 1 C), 130.7 (q, *J*=32.3 Hz, 2 C), 127.8 (s, 1 C), 125.7 (s, 1 C), 122.3 (s, 1 C), 120.8 (s, 1 C), 123.4 (br q, *J*=238.2 Hz, 2 C), 118.4 (br q, *J*=3.9 Hz, 2 C), 117.3 (br spt, *J*=3.9 Hz, 1 C), 114.9 (s, 1 C), 113.4 (s, 1 C)

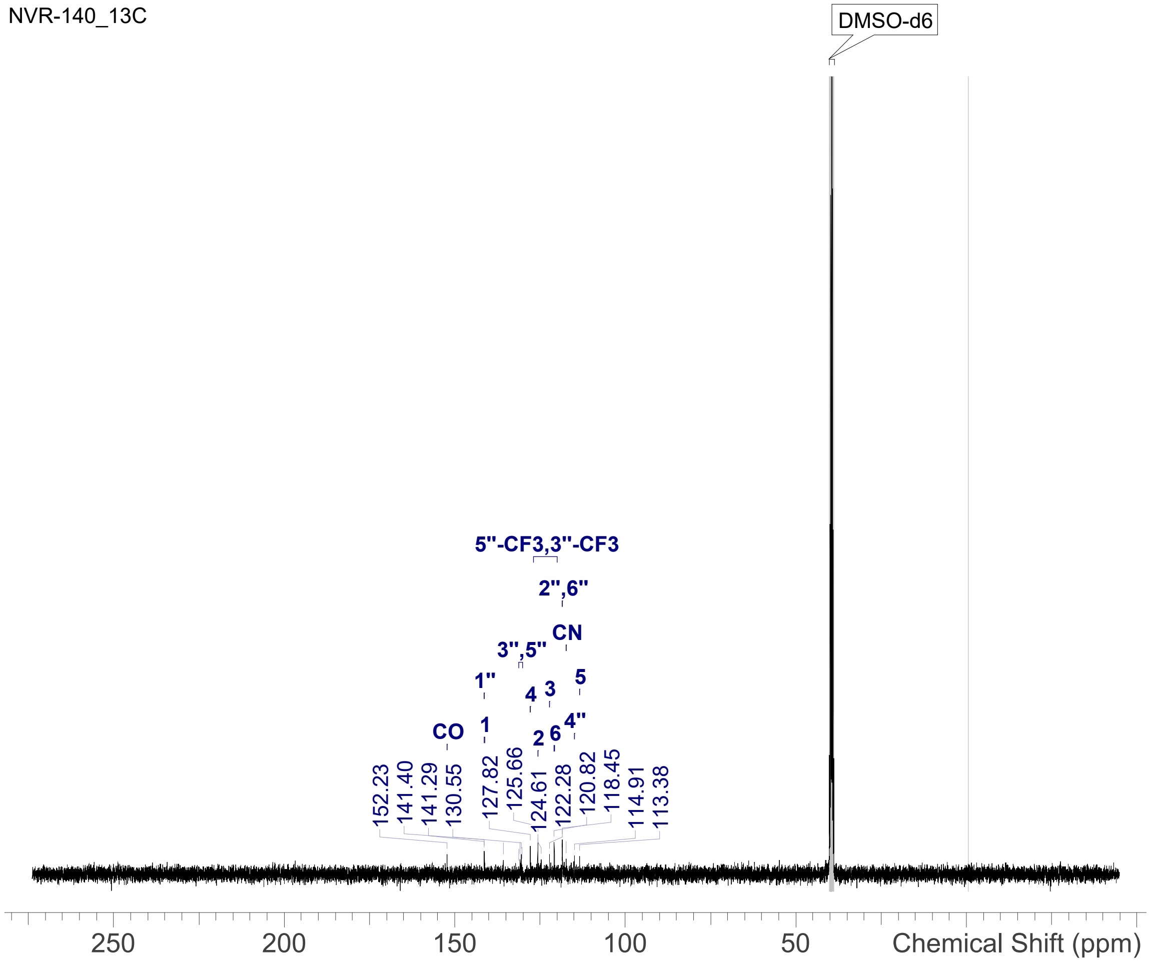

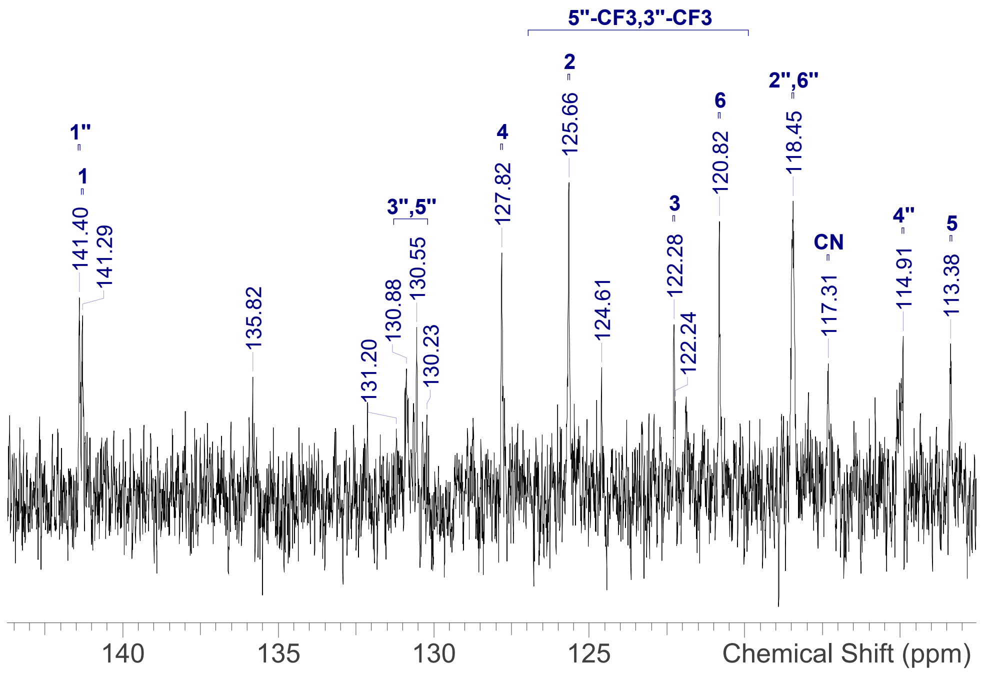

NVR-140_13C.spectrus

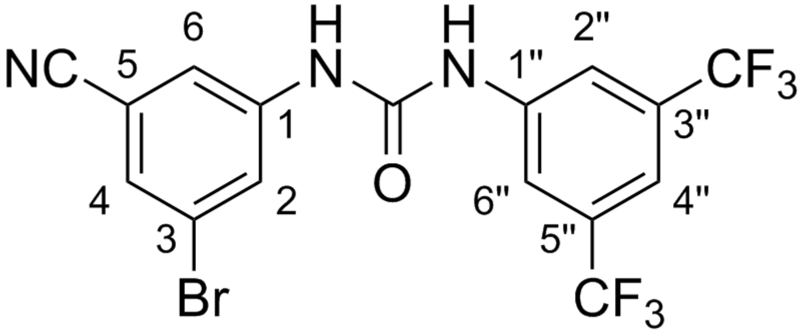

Shift (ppm)	F	m
-61.70	6	s

Acquisition Time (sec)	1.4680
Date	05 Jul 2019 15:34:37
Date Stamp	05 Jul 2019 15:34:37
Frequency (MHz)	376.4419
Nucleus	19F
Number of Transients	16
Solvent	DMSO-d6
Temperature (degree C)	24.999

^{19}F NMR (376 MHz, *DMSO-d$_6$*) δ ppm -61.70 (s, 6 F)

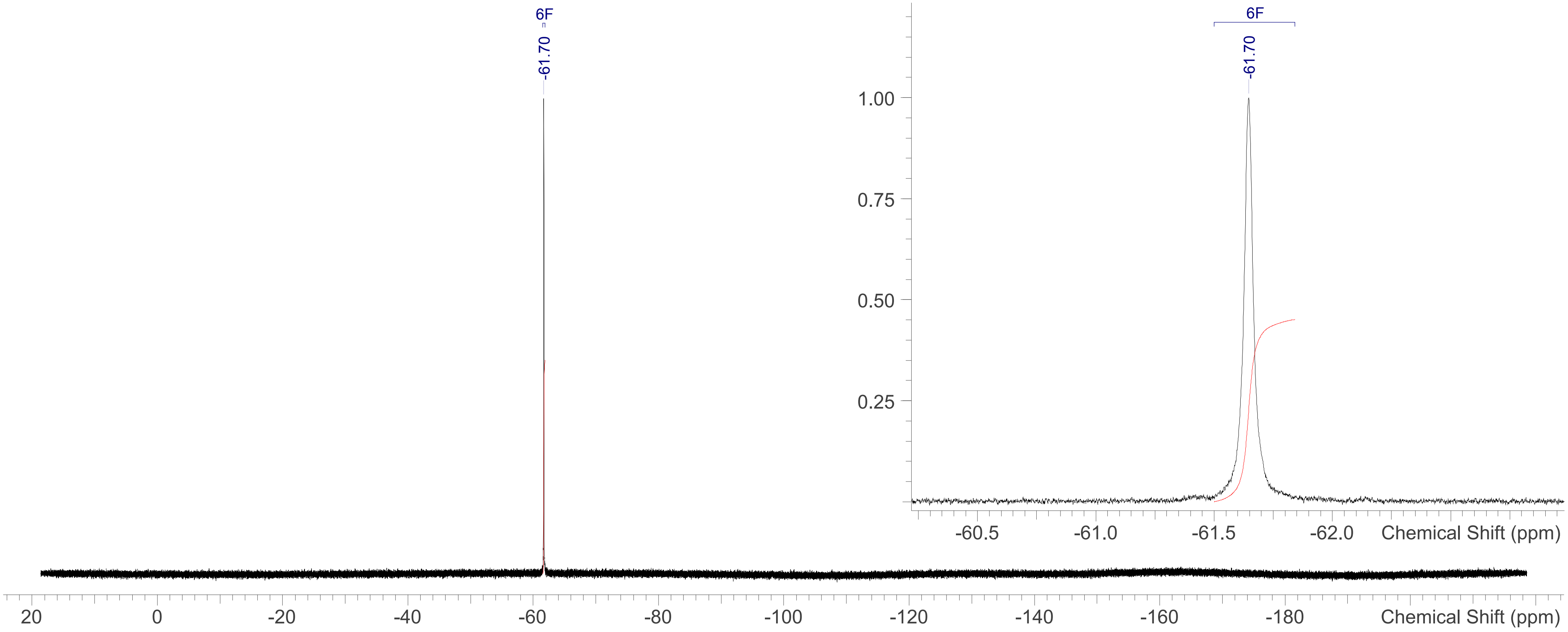

NVR-140_19F.spectrus

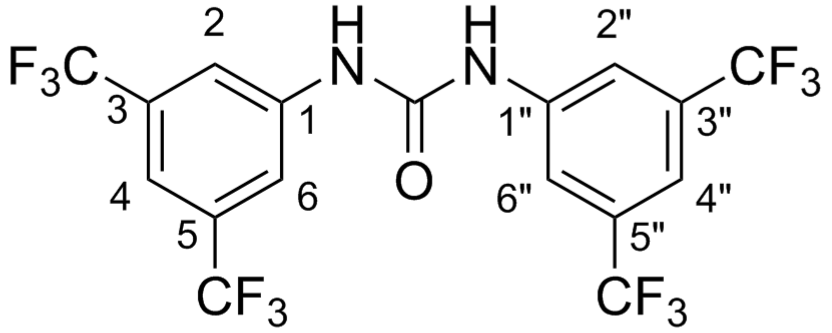

No.	Shift (ppm)	H	m	Assign
1	9.75	2	br s	NH", NH
2	8.18	4	br s	6, 2", 6", 2
3	7.71	2	br s	4, 4"

Acquisition Time (sec)	3.9846
Date	31 Jan 2019 20:08:36
Date Stamp	31 Jan 2019 20:08:36
Frequency (MHz)	400.0700
Nucleus	1H
Number of Transients	16
Solvent	DMSO-d6
Temperature (degree C)	25.001

^{1}H NMR (400 MHz, *DMSO-d_6*) δ ppm 9.75 (br s, 2 H), 8.18 (br s, 4 H), 7.71 (br s, 2 H)

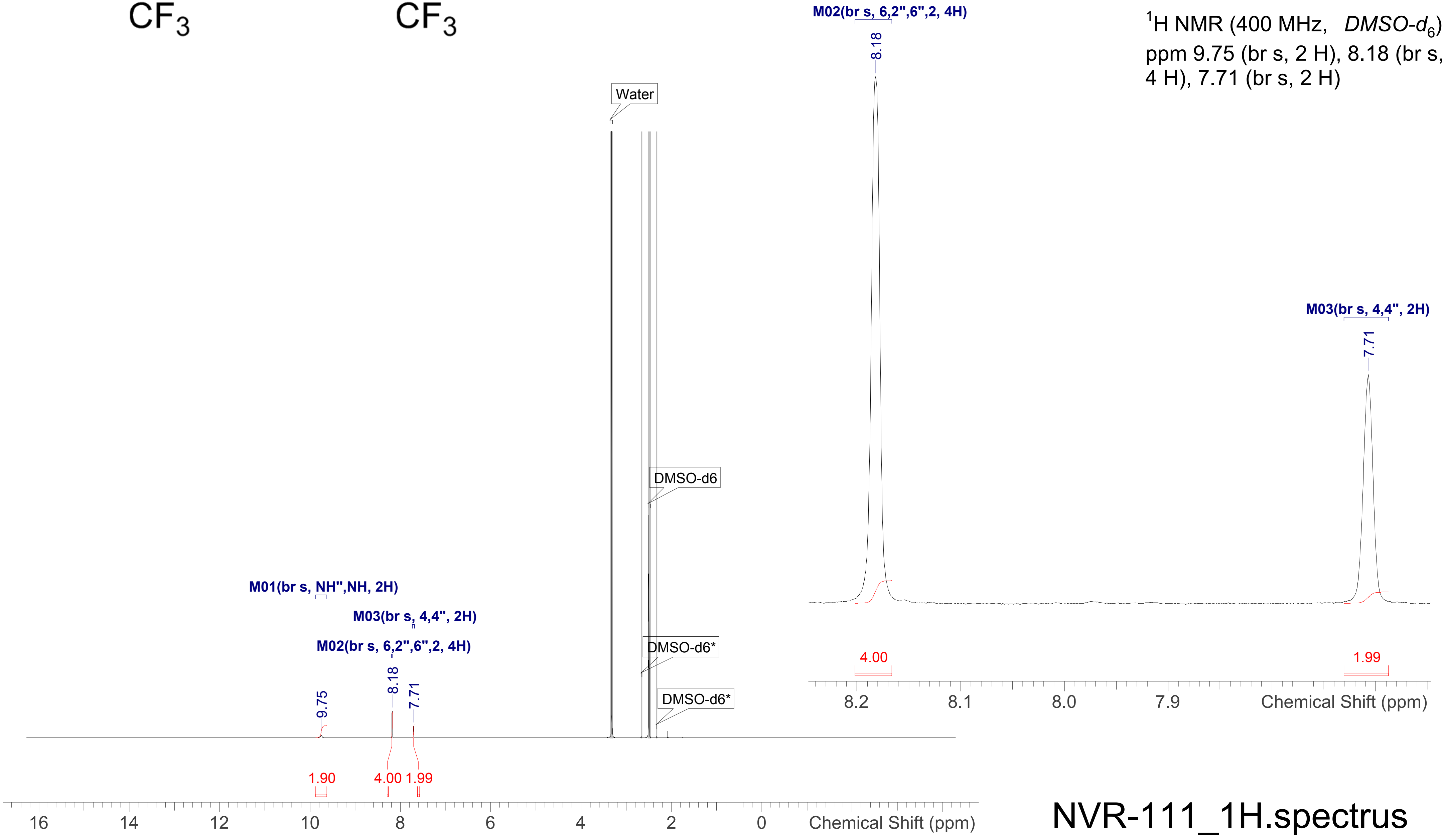

NVR-111_1H.spectrus

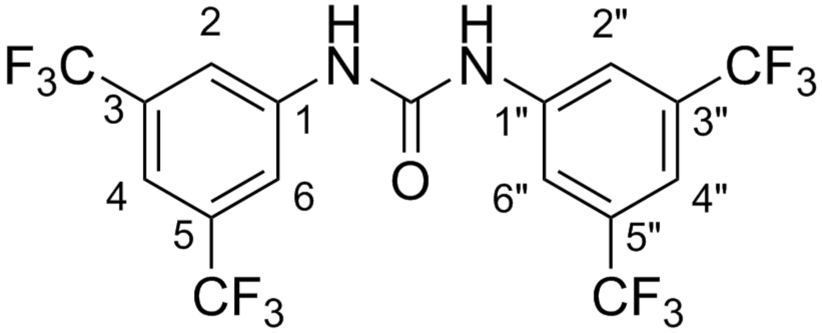

Shift (ppm)	F	m
-61.60	12	s

Acquisition Time (sec)	1.4680
Date	31 Jan 2019 21:12:03
Date Stamp	31 Jan 2019 21:12:03
Frequency (MHz)	376.4419
Nucleus	19F
Number of Transients	128
Solvent	DMSO-d6
Temperature (degree C)	25.001

^{19}F NMR (376 MHz, *DMSO-d_6*) δ ppm -61.60 (s, 12 F)

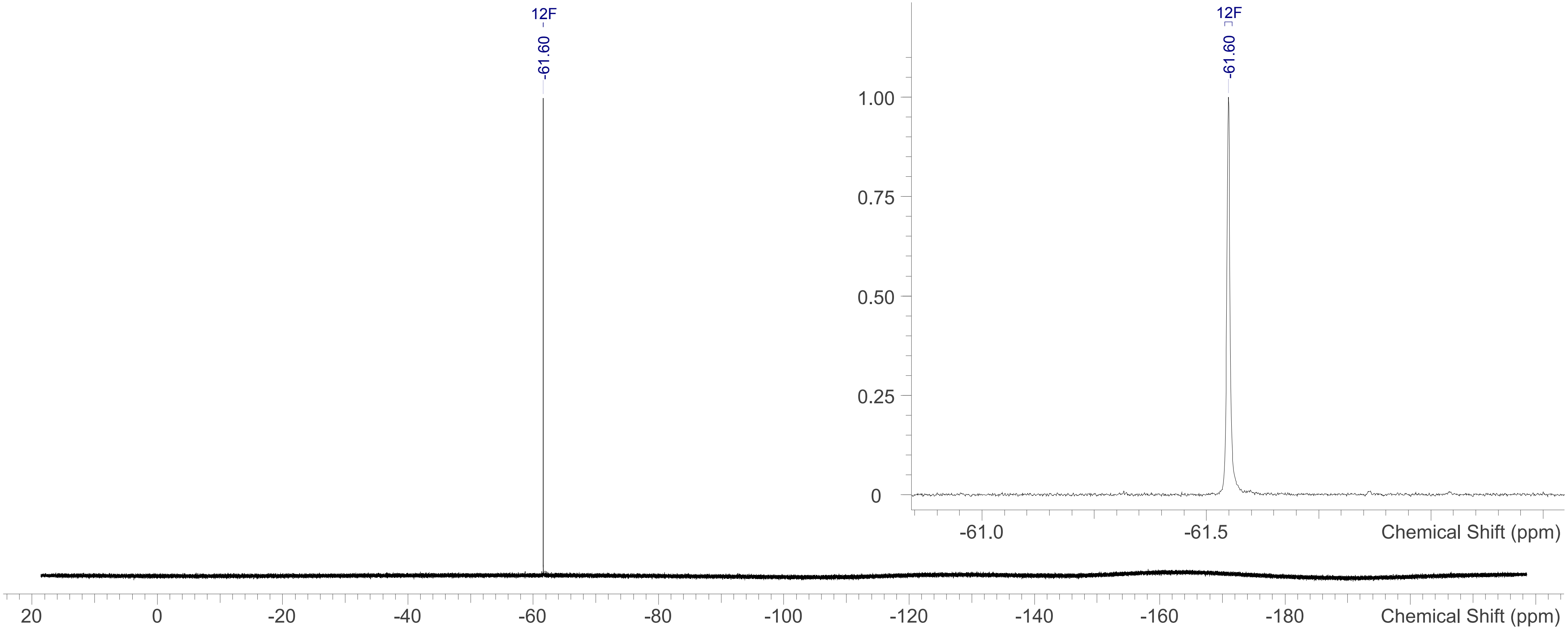

NVR-111_19F.spectrus

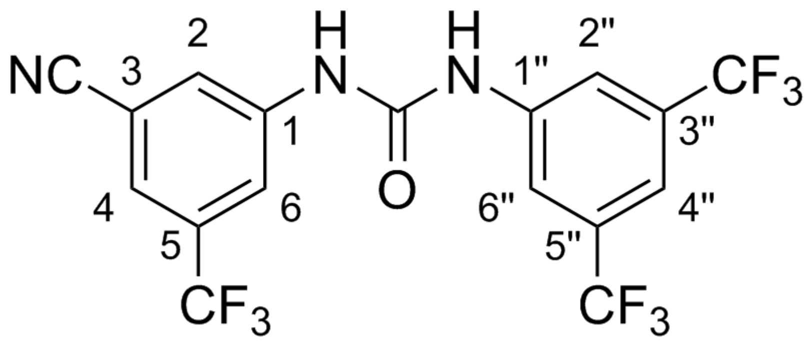

Shift (ppm)	H	m	Assign
9.74	1	s	NH"
9.63	1	s	NH
8.21	1	br s	6
8.15	3	m	4, 2", 6"
7.93	1	br s	2
7.68	1	br s	4"

Acquisition Time (sec)	3.2768
Date	15 Jul 2019 16:50:45
Date Stamp	15 Jul 2019 16:50:45
Frequency (MHz)	500.1900
Nucleus	1H
Number of Transients	16
Solvent	DMSO-d6
Temperature (degree C)	25.006

^{1}H NMR (500 MHz, *DMSO-d_6*) δ ppm 9.74 (s, 1 H), 9.63 (s, 1 H), 8.21 (br s, 1 H), 8.12 - 8.19 (m, 3 H), 7.93 (br s, 1 H), 7.68 (br s, 1 H)

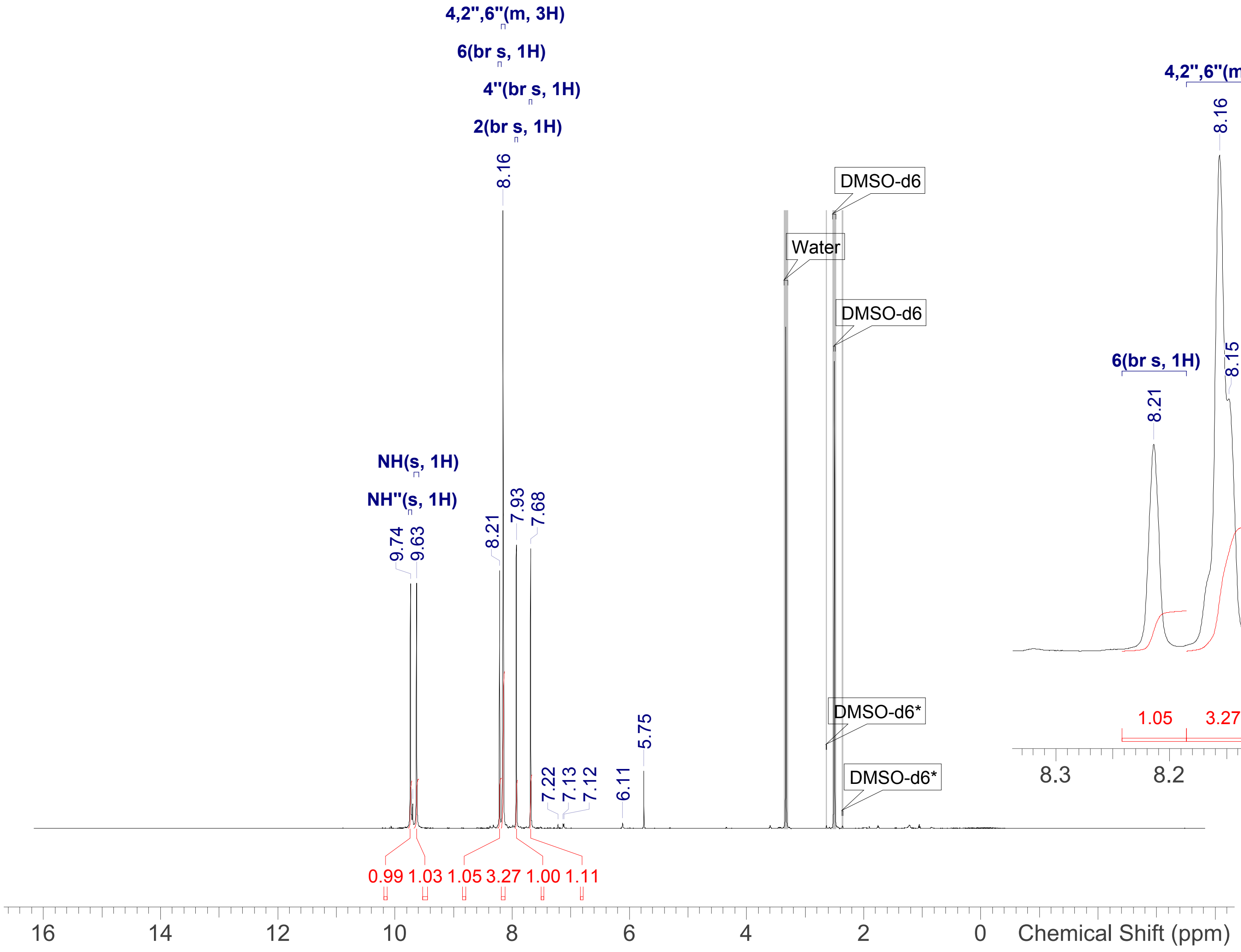

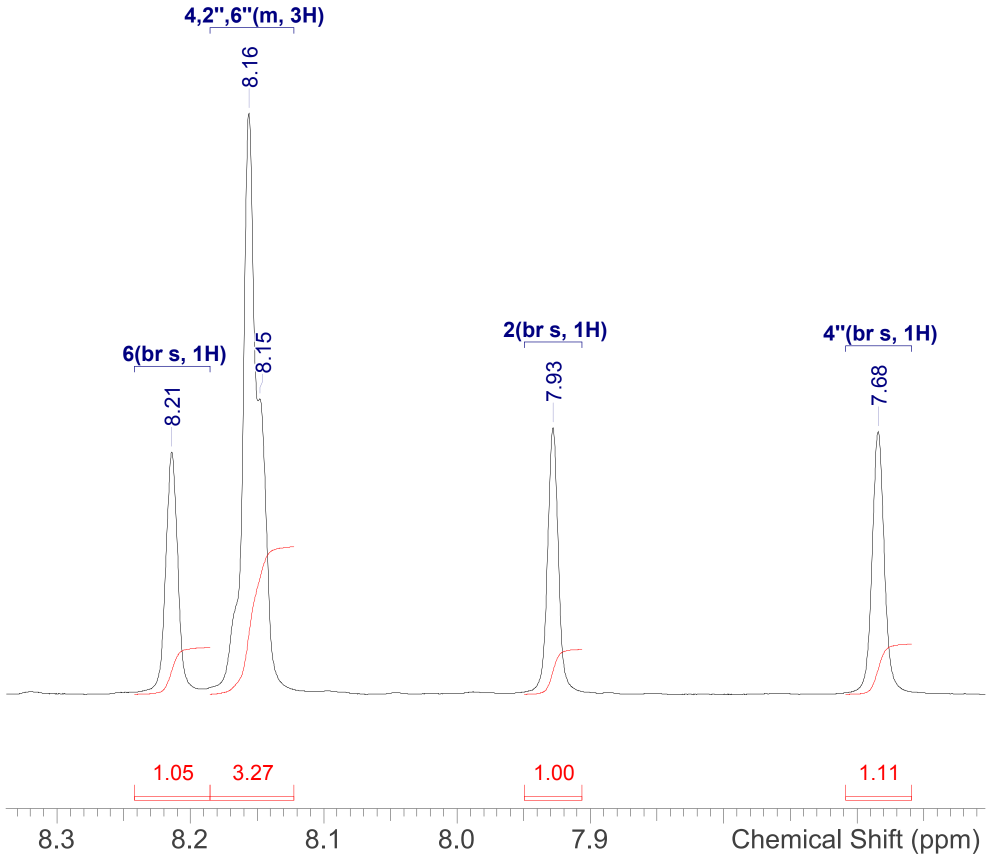

NVR-136_1H.spectrus

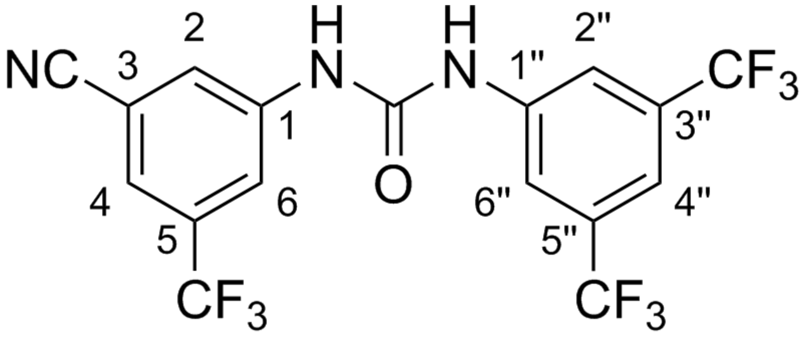

Shift (ppm)	C	m	J (Hz)	Assign
152.4	1	s	-	CO
141.3	1	s	-	1"
141.1	1	s	-	1
130.8	1	q	33.3	5
130.7	2	q	32.3	3", 5"
125.4	1	s	-	2
123.2	2	q	272.9	5"-CF3, 3"-CF3
123.0	1	q	272.9	5-CF3
122.3	1	br q	2.0	4
119.3	1	br q	4.9	6
118.6	2	br q	3.9	2", 6"
117.4	1	s	-	CN
115.1	1	br spt	3.9	4"
113.0	1	s	-	3

Acquisition Time (sec)	1.0224
Date	15 Jul 2019 16:53:44
Date Stamp	15 Jul 2019 16:53:44
Frequency (MHz)	125.7729
Nucleus	13C
Number of Transients	32
Solvent	DMSO-d6
Temperature (degree C)	24.995

^{13}C NMR (126 MHz, *DMSO-d$_6$*) δ ppm 152.4 (s, 1 C), 141.3 (s, 1 C), 141.1 (s, 1 C), 130.8 (q, *J*=33.3 Hz, 1 C), 130.7 (q, *J*=32.3 Hz, 2 C), 125.4 (s, 1 C), 122.3 (br q, *J*=2.0 Hz, 1 C), 123.2 (q, *J*=272.9 Hz, 2 C), 123.0 (q, *J*=272.9 Hz, 1 C), 119.3 (br q, *J*=4.9 Hz, 1 C), 118.6 (br q, *J*=3.9 Hz, 2 C), 117.4 (s, 1 C), 115.1 (br spt, *J*=3.9 Hz, 1 C), 113.0 (s, 1 C)

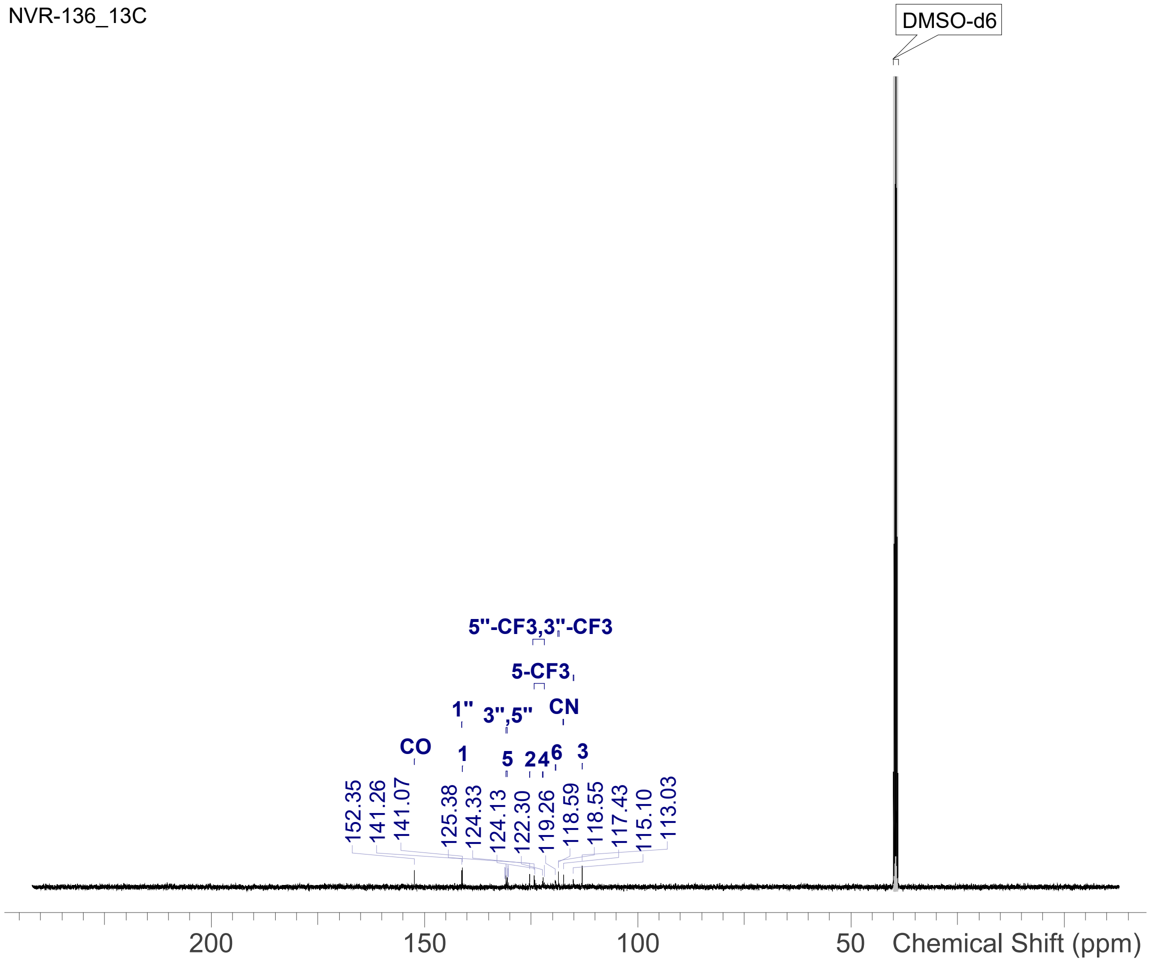

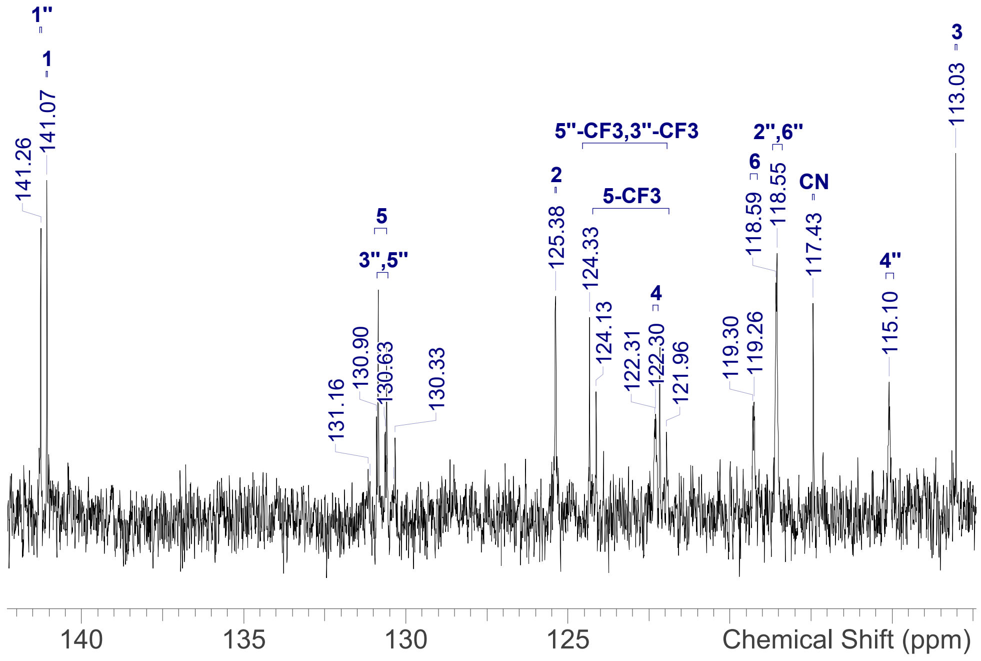

NVR-136_13C.spectrus

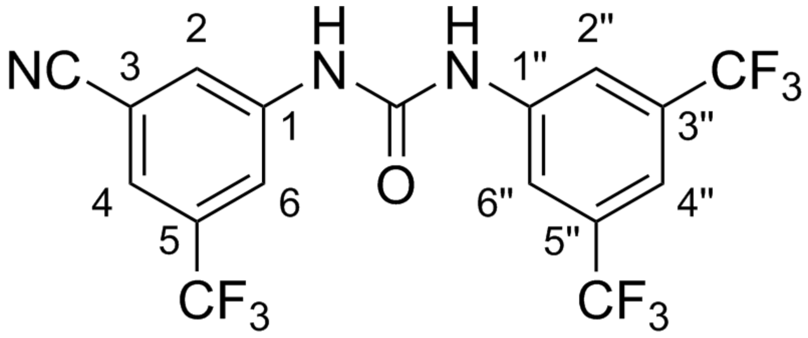

Shift (ppm)	F	m
-61.69	6	s
-61.82	3	s

Acquisition Time (sec)	0.3670
Date	15 Jul 2019 16:58:23
Date Stamp	15 Jul 2019 16:58:23
Frequency (MHz)	470.6488
Nucleus	19F
Number of Transients	16
Solvent	DMSO-d6
Temperature (degree C)	24.980

^{19}F NMR (471 MHz, *DMSO-d_6*) δ ppm -61.69 (s, 6 F), -61.82 (s, 3 F)

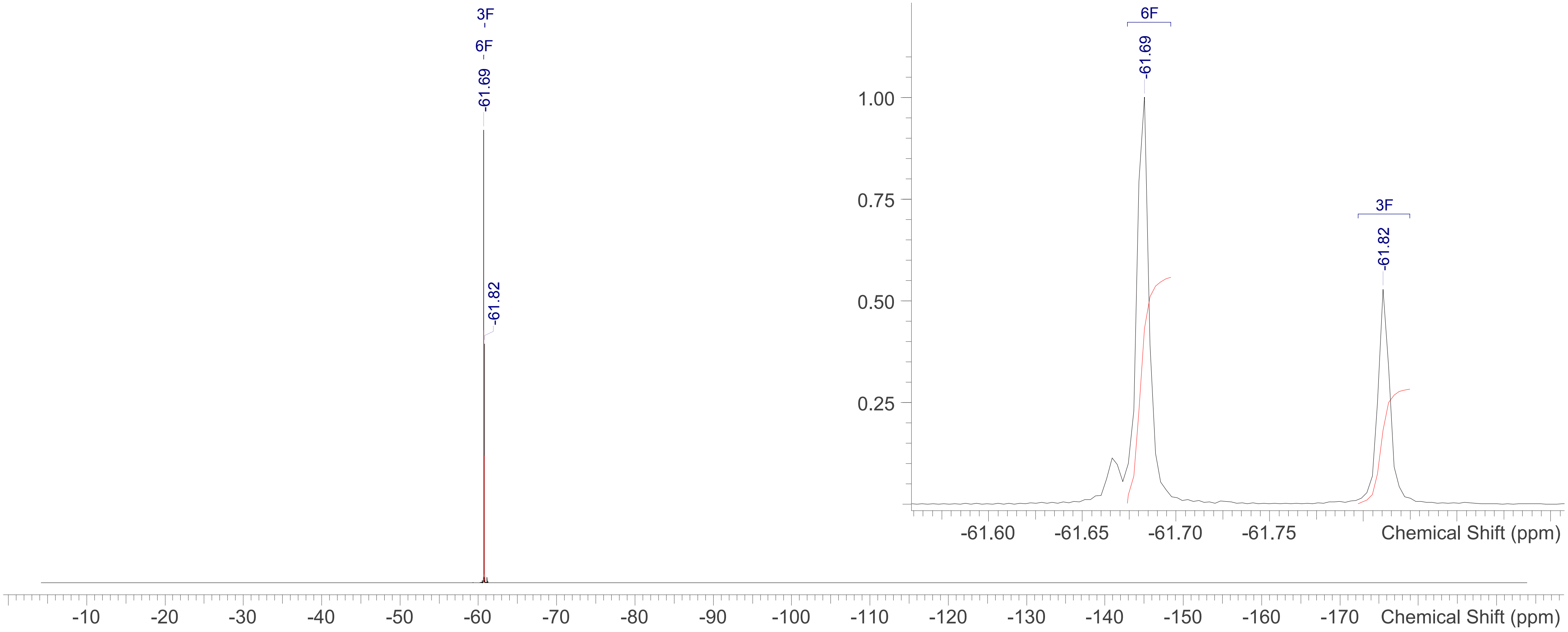

NVR-136_19F.spectrus

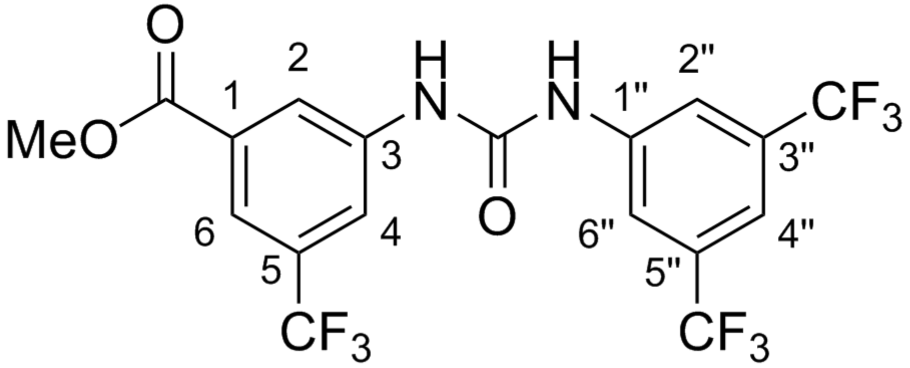

No.	Shift (ppm)	H	m	Assign
1	9.65	1	s	NH
2	9.59	1	s	NH"
3	8.34	1	br s	4
4	8.18	1	br s	6
5	8.16	2	s	2", 6"
6	7.80	1	br s	2
7	7.67	1	br s	4"
8	3.90	3	s	COOMe

Acquisition Time (sec)	8.1789
Date	21/06/2019 10:00:00
Date Stamp	21/06/2019 10:00:00
Frequency (MHz)	400.1320
Nucleus	1H
Number of Transients	16
Solvent	DMSO-d6

^{1}H NMR (400 MHz, *DMSO-d$_6$*) δ ppm 9.65 (s, 1 H), 9.59 (s, 1 H), 8.34 (br s, 1 H), 8.18 (br s, 1 H), 8.16 (s, 2 H), 7.80 (br s, 1 H), 7.67 (br s, 1 H), 3.90 (s, 3 H)

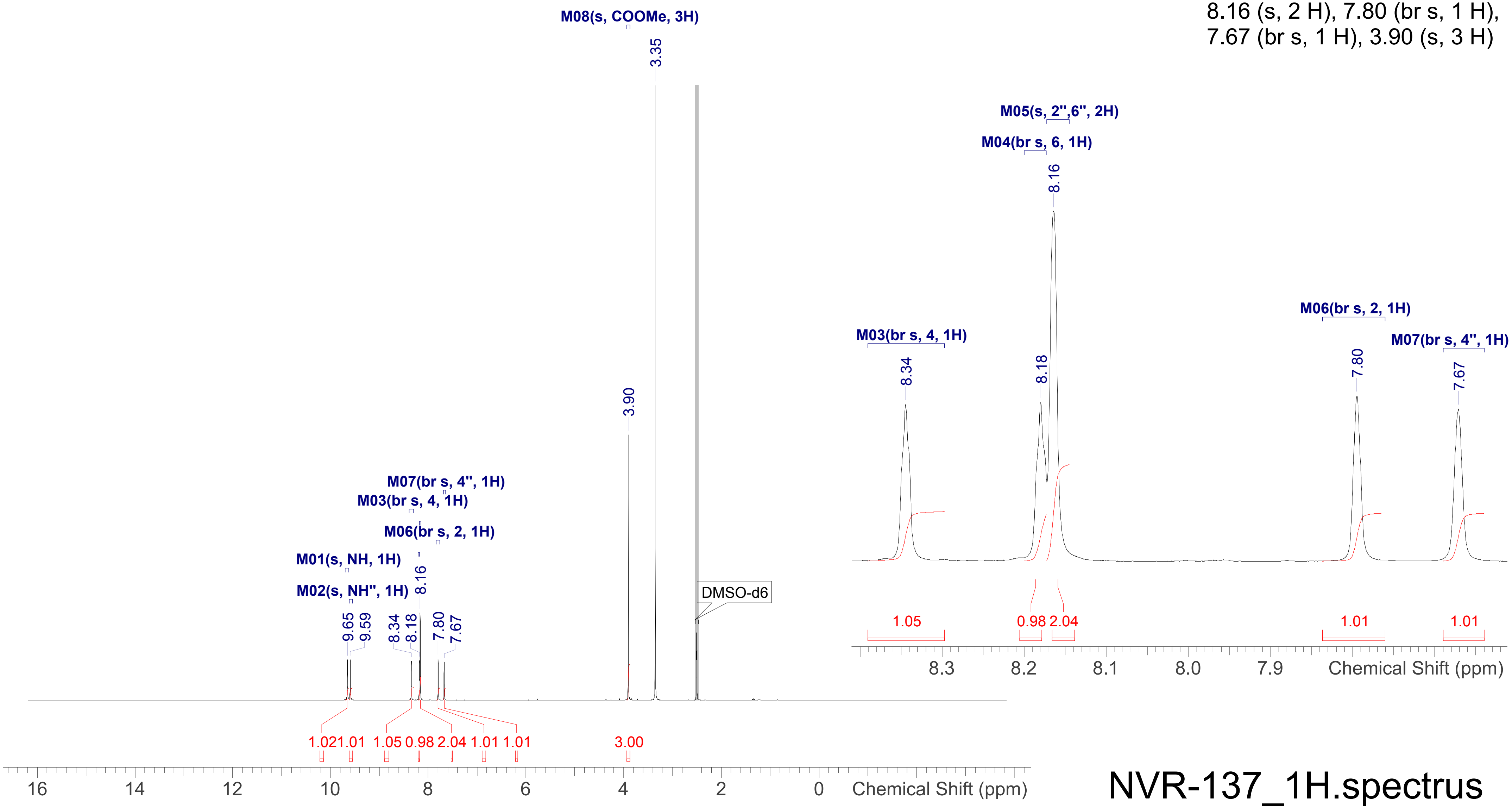

NVR-137_1H.spectrus

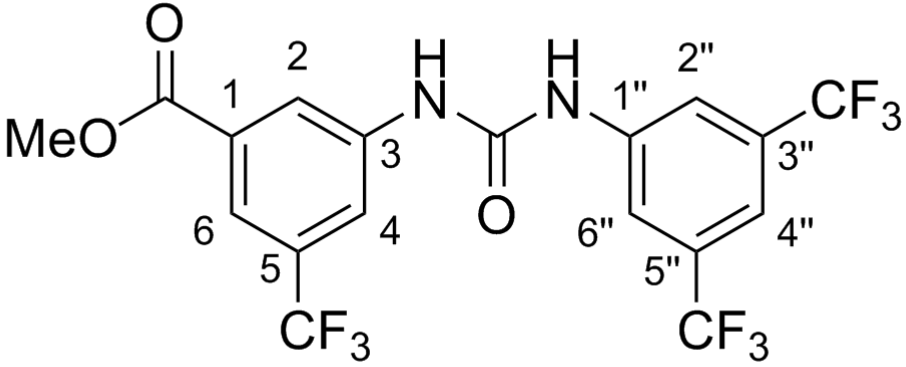

Shift (ppm)	C	m	J (Hz)	Assign
164.9	1	s	-	COOMe
152.4	1	s	-	CO
141.4	1	s	-	1''
140.8	1	s	-	3
131.4	1	s	-	1
130.7	2	q	32.3	3'', 5''
130.0	1	q	32.3	5
123.6	1	q	272.9	5-CF3
123.3	2	q	272.9	5''-CF3, 3''-CF3
122.7	1	s	-	2
119.0	1	br q	4.9	6
118.7	1	br q	3.9	4
118.5	2	br q	3.9	2'', 6''
114.9	1	br spt	3.9	4''
52.7	1	s	-	MeOOC

Acquisition Time (sec)	1.0224
Date	22 Jun 2019 20:28:02
Date Stamp	22 Jun 2019 20:28:02
Frequency (MHz)	100.6128
Nucleus	13C
Number of Transients	256
Solvent	DMSO-d6
Temperature (degree C)	23.000

^{13}C NMR (101 MHz, *DMSO-d_6*) δ ppm 164.9 (s, 1 C), 152.4 (s, 1 C), 141.4 (s, 1 C), 140.8 (s, 1 C), 131.4 (s, 1 C), 130.7 (q, *J*=32.3 Hz, 2 C), 130.0 (q, *J*=32.3 Hz, 1 C), 122.7 (s, 1 C), 123.6 (q, *J*=272.9 Hz, 1 C), 123.3 (q, *J*=272.9 Hz, 2 C), 119.0 (br q, *J*=4.9 Hz, 1 C), 118.7 (br q, *J*=3.9 Hz, 1 C), 118.5 (br q, *J*=3.9 Hz, 2 C), 114.9 (br spt, *J*=3.9 Hz, 1 C), 52.7 (s, 1 C)

NVR-137_13C

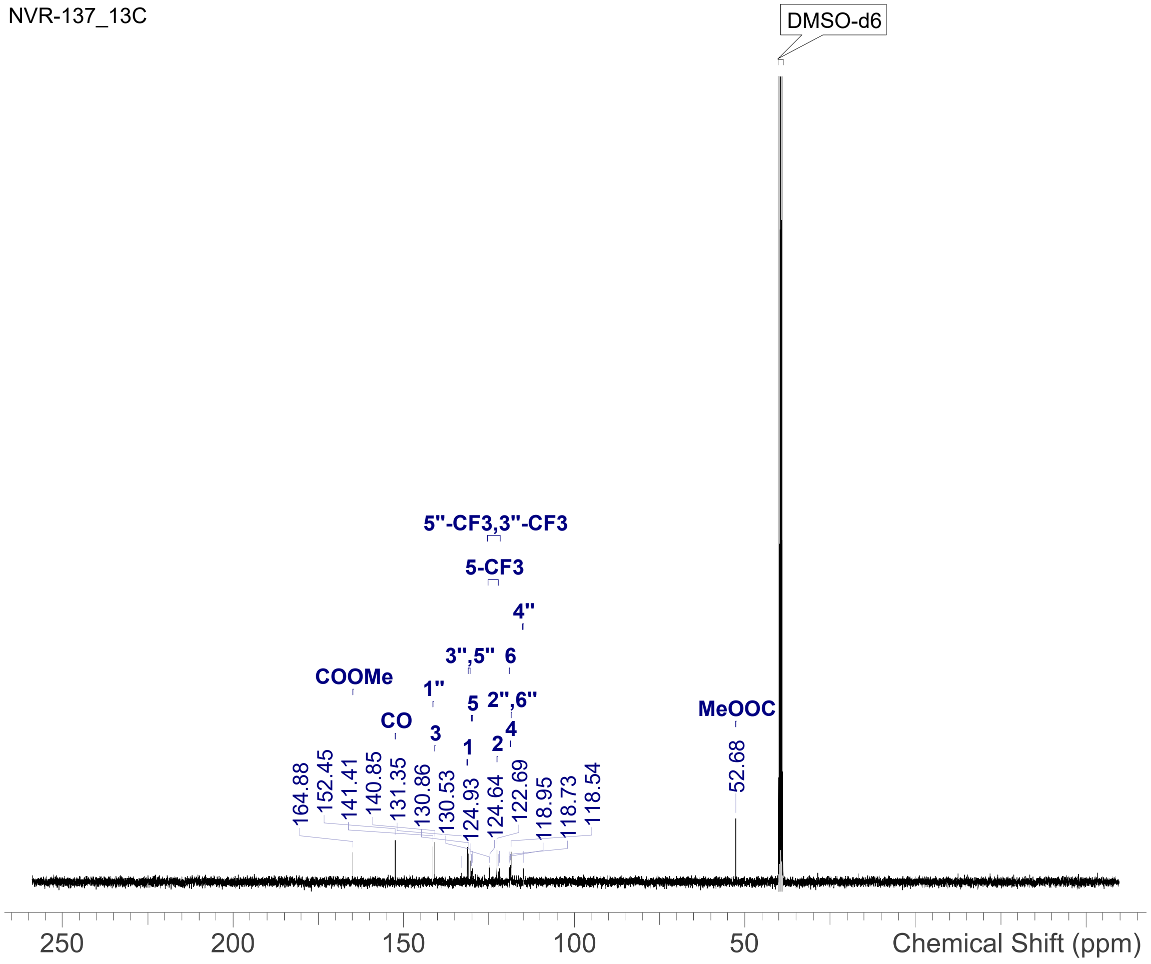

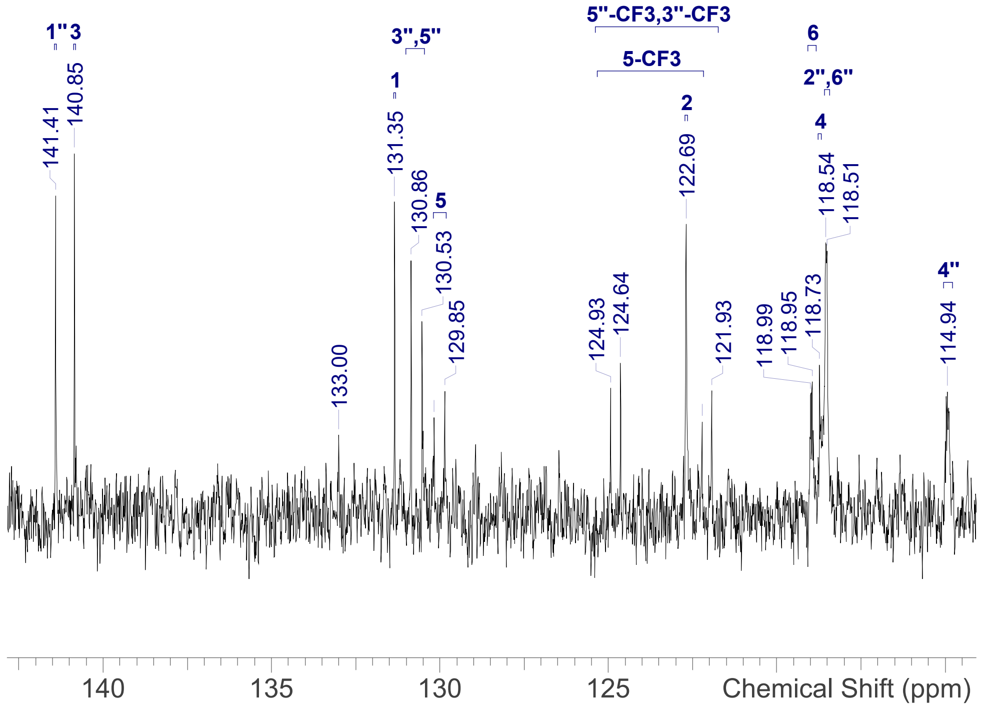

NVR-137_13C.spectrus

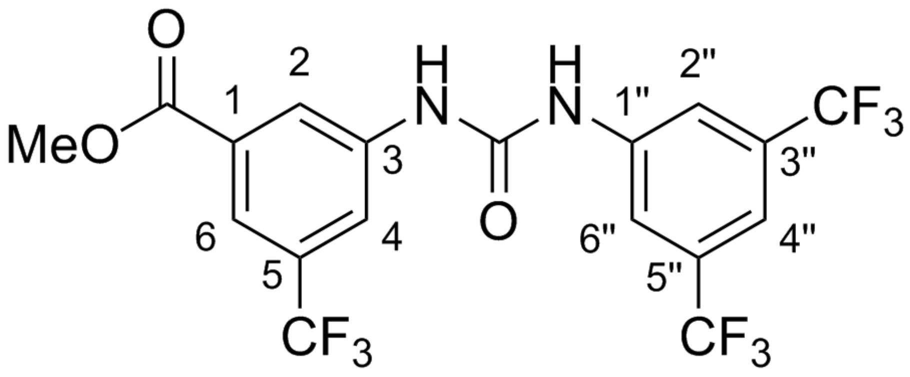

Shift (ppm)	F	m
-61.67	3	s
-61.68	6	s

Acquisition Time (sec)	1.4680
Date	22 Jun 2019 20:44:41
Date Stamp	22 Jun 2019 20:44:41
Frequency (MHz)	376.4984
Nucleus	19F
Number of Transients	128
Solvent	DMSO-d6
Temperature (degree C)	22.900

^{19}F NMR (376 MHz, *DMSO-d$_6$*) δ ppm -61.67 (s, 3 F), -61.68 (s, 6 F)

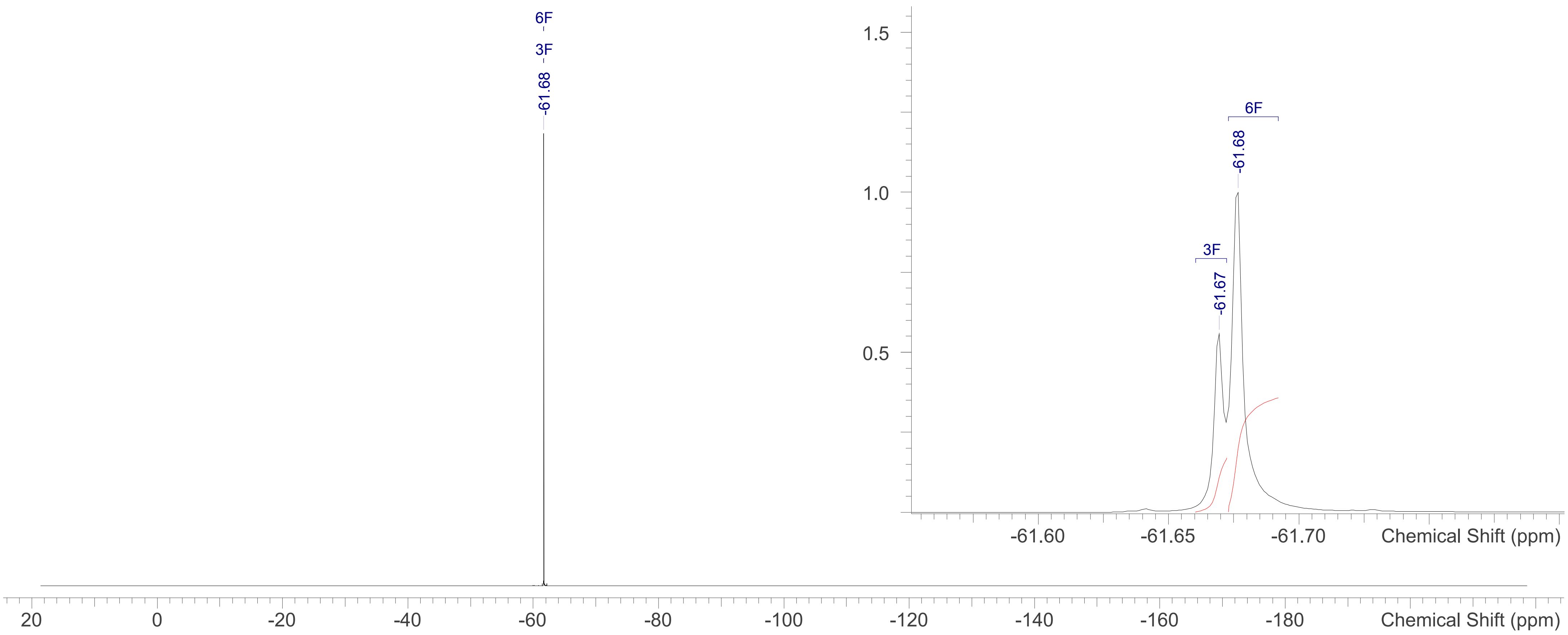

NVR-137_19F.spectrus

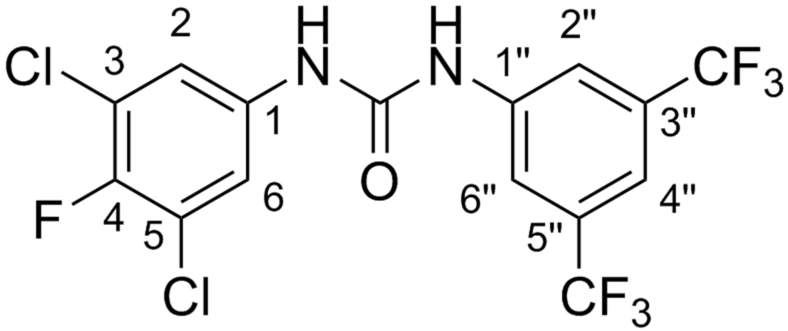

No.	Shift (ppm)	H	m	J (Hz)	Assign
1	9.55	1	br s	-	NH
2	9.23	1	br s	-	NH''
3	8.10	2	br s	-	2'', 6''
4	7.65	2	d	6.0	2, 6
5	7.61	1	br s	-	4''

Acquisition Time (sec)	3.2768
Date	29 Jul 2019 14:21:27
Date Stamp	29 Jul 2019 14:21:27
Frequency (MHz)	500.1900
Nucleus	1H
Number of Transients	16
Solvent	DMSO-d6
Temperature (degree C)	25.001

^{1}H NMR (500 MHz, *DMSO-d_6*) δ ppm 9.55 (br s, 1 H), 9.23 (br s, 1 H), 8.10 (br s, 2 H), 7.65 (d, *J*=6.0 Hz, 2 H), 7.61 (br s, 1 H)

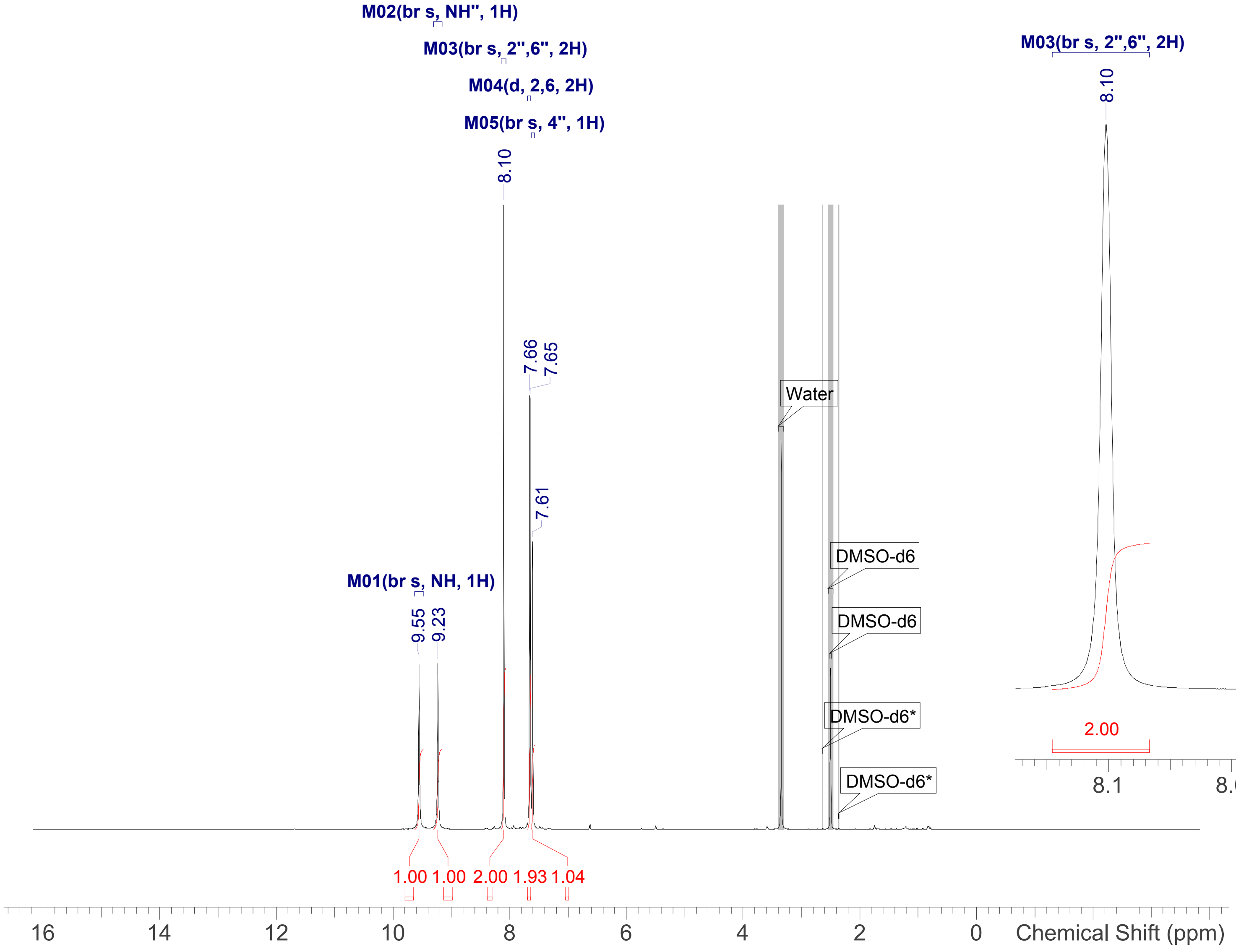

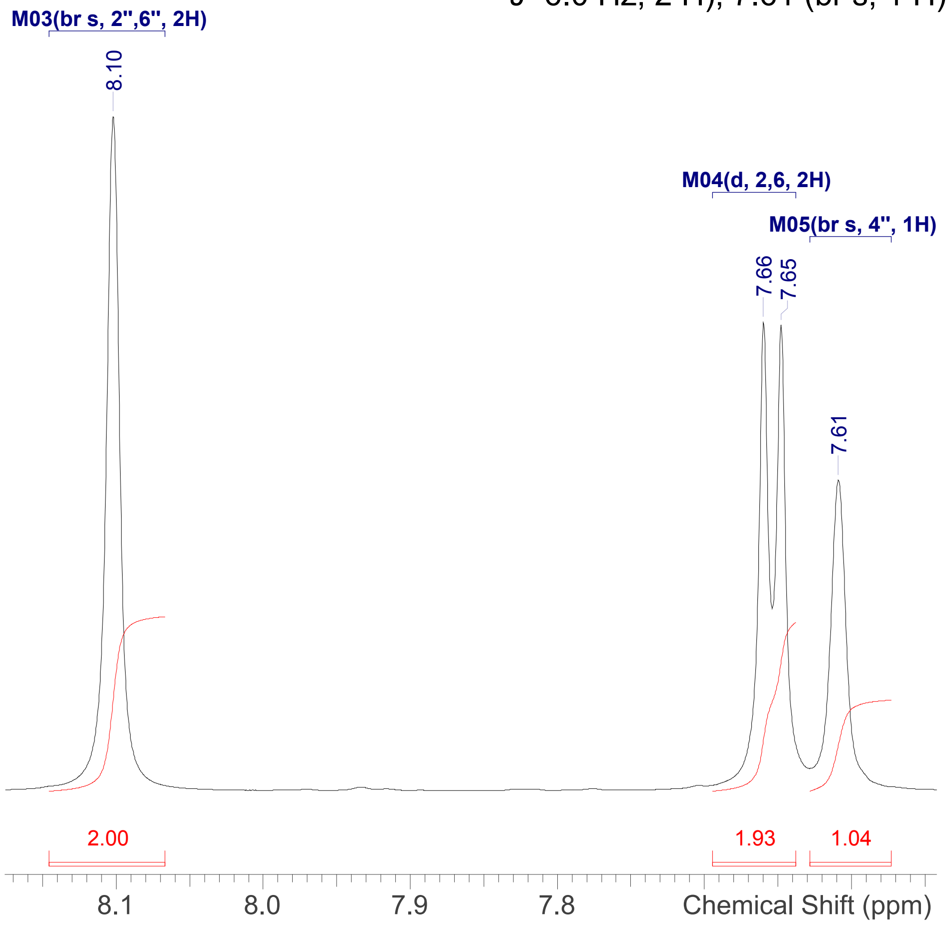

NVR-143_1H.spectrus

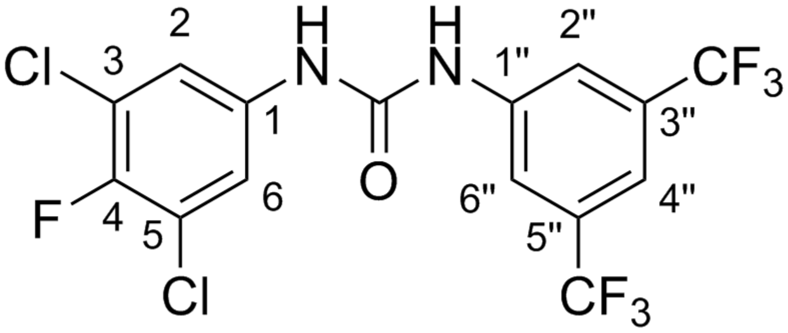

Shift (ppm)	C	m	J (Hz)	Assign
152.2	1	s	-	CO
148.5	1	d	242.6	4
141.4	1	s	-	1"
136.5	1	d	3.9	1
130.7	2	q	32.3	3", 5"
123.3	2	q	272.6	5"-CF3, 3"-CF3
120.7	2	d	17.6	5, 3
119.0	2	br s	-	2, 6
118.2	2	br q	3.9	2", 6"
114.7	1	br spt	3.9	4"

Acquisition Time (sec)	1.0224
Date	29 Jul 2019 14:24:26
Date Stamp	29 Jul 2019 14:24:26
Frequency (MHz)	125.7729
Nucleus	13C
Number of Transients	32
Solvent	DMSO-d6
Temperature (degree C)	24.999

^{13}C NMR (126 MHz, *DMSO-d_6*) δ ppm 152.2 (s, 1 C), 148.5 (d, *J*=242.6 Hz, 1 C), 141.4 (s, 1 C), 136.5 (d, *J*=3.9 Hz, 1 C), 130.7 (q, *J*=32.3 Hz, 2 C), 120.7 (d, *J*=17.6 Hz, 2 C), 123.3 (q, *J*=272.6 Hz, 2 C), 119.0 (br s, 2 C), 118.2 (br q, *J*=3.9 Hz, 2 C), 114.7 (br spt, *J*=3.9 Hz, 1 C)

NVR-143_13C

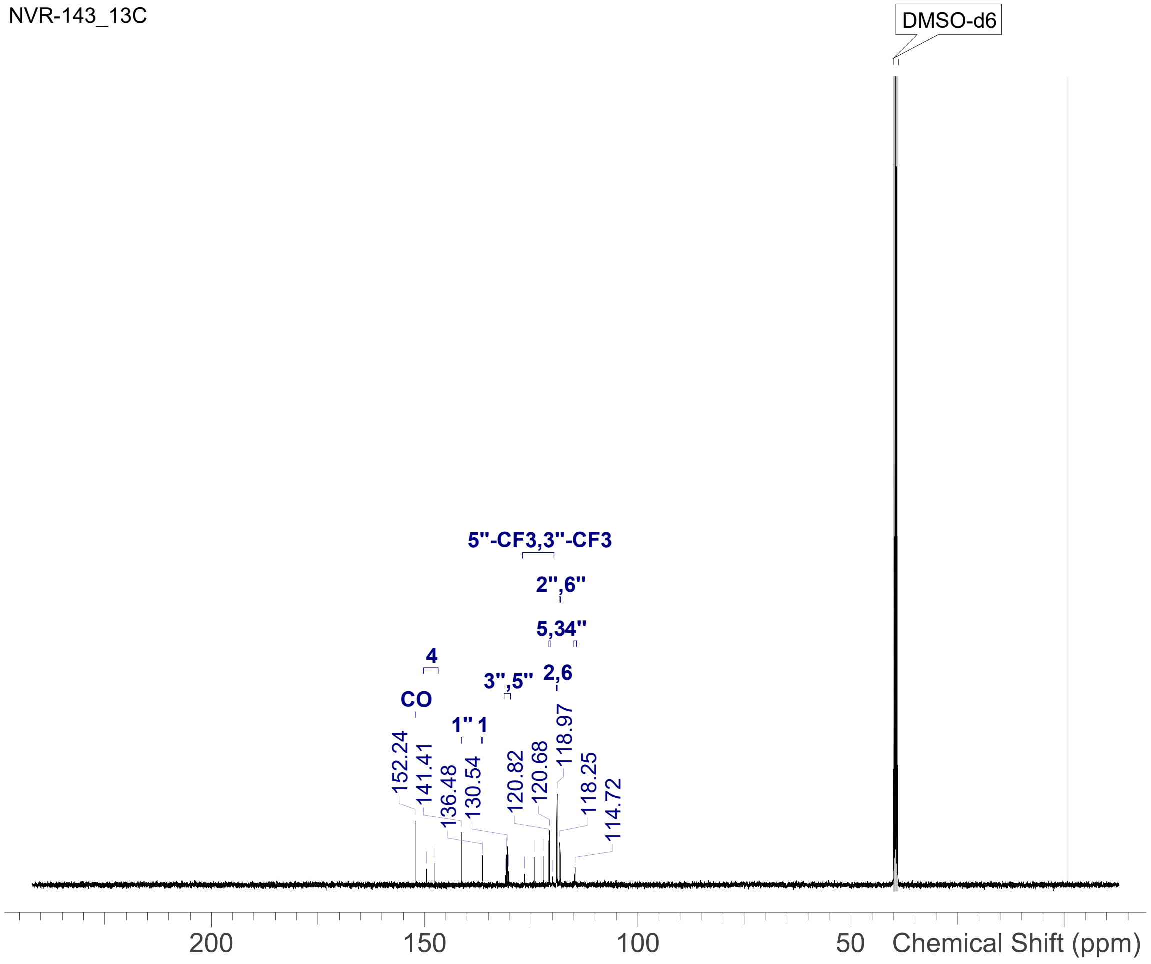

NVR-143_13C

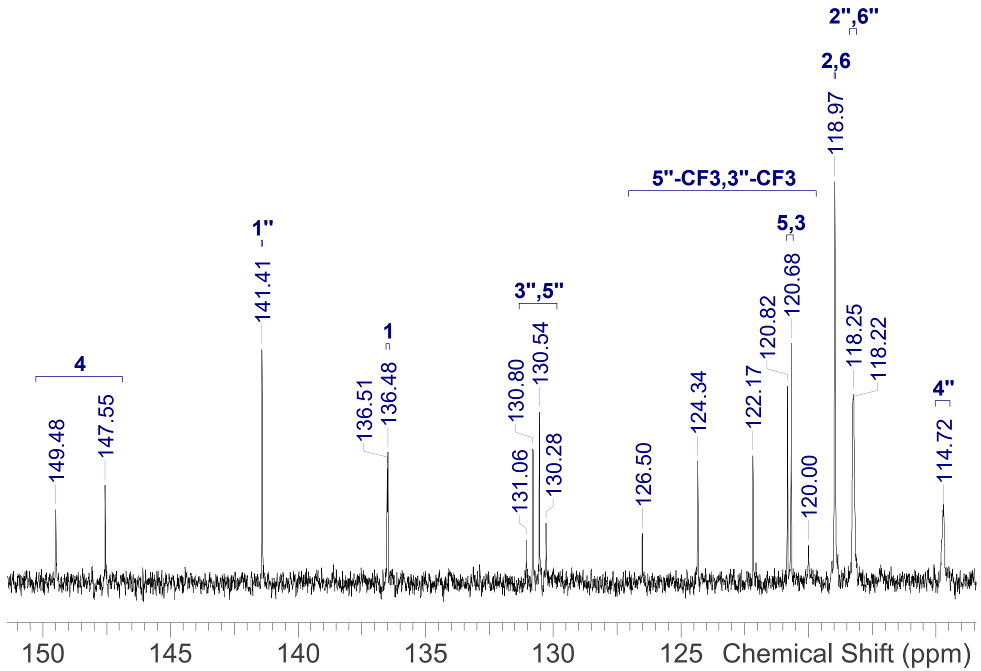

NVR-143_13C.spectrus

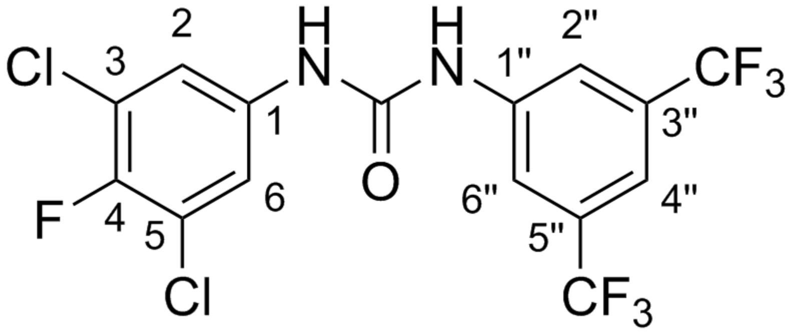

Shift (ppm)	F	m	J (Hz)
-61.72	6	s	-
-126.00	1	t	6.1

Acquisition Time (sec)	1.4680
Date	05 Jul 2019 16:10:29
Date Stamp	05 Jul 2019 16:10:29
Frequency (MHz)	376.4419
Nucleus	19F
Number of Transients	16
Solvent	DMSO-d6
Temperature (degree C)	24.998

^{19}F NMR (376 MHz, *DMSO-d_6*) δ ppm -61.72 (s, 6 F), -126.00 (t, *J*=6.1 Hz, 1 F)

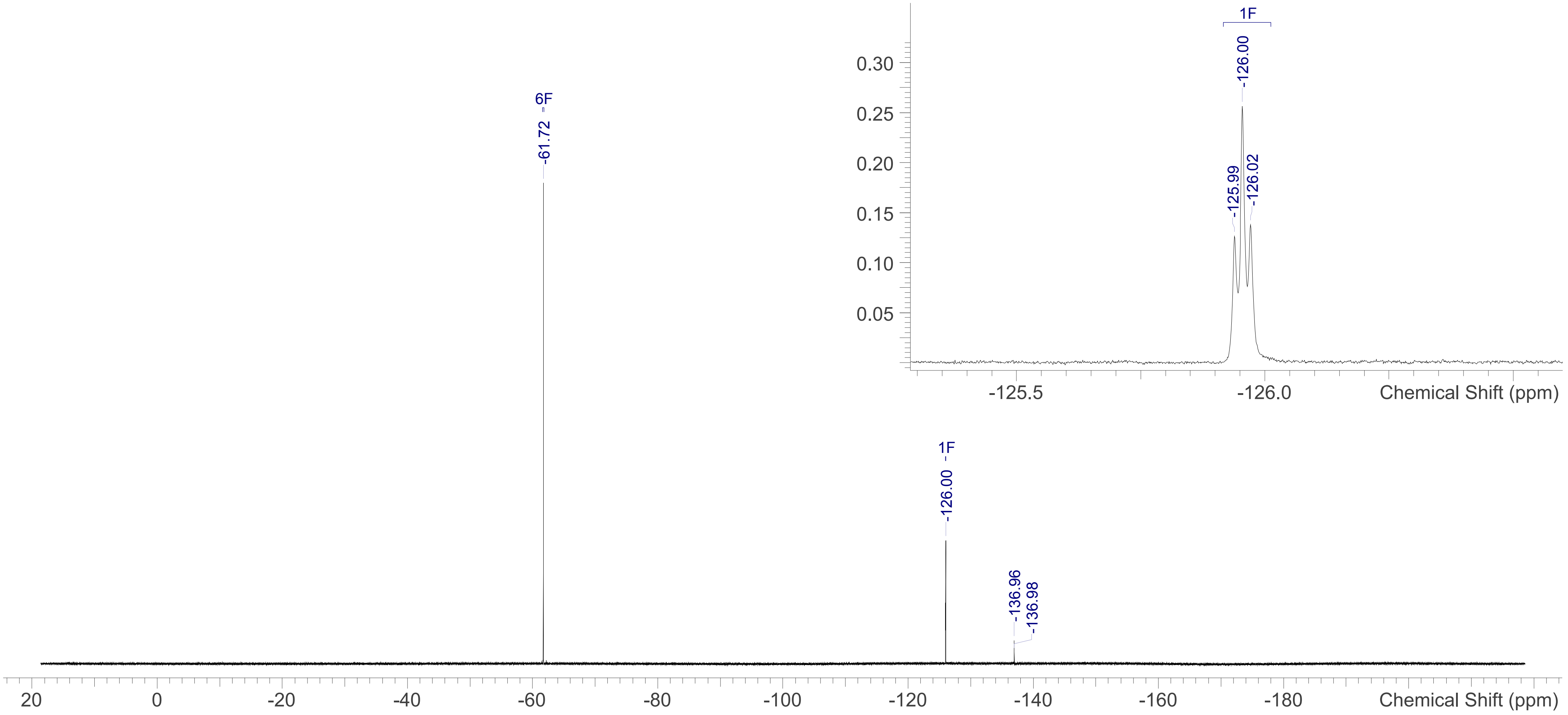

NVR-143_19F.spectrus

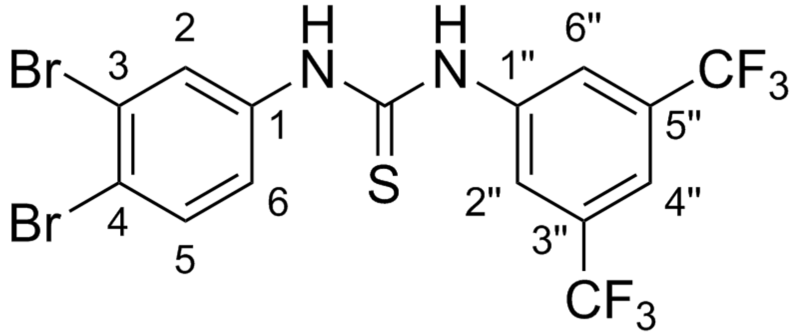

Shift (ppm)	H	m	J (Hz)	Assign
10.38	2	br s	-	NH, NH''
8.22	2	s	-	2'', 6''
7.94	1	d	2.4	2
7.82	1	br s	-	4''
7.74	1	d	8.7	5
7.40	1	dd	8.7, 2.4	6

Acquisition Time (sec)	7.9692
Date	05/07/2019 11:48:00
Date Stamp	05/07/2019 11:48:00
Frequency (MHz)	400.0720
Nucleus	1H
Number of Transients	4
Solvent	DMSO-d6

^{1}H NMR (400 MHz, *DMSO-d_6*) δ ppm 10.38 (br s, 2 H), 8.22 (s, 2 H), 7.94 (d, *J*=2.4 Hz, 1 H), 7.82 (br s, 1 H), 7.74 (d, *J*=8.7 Hz, 1 H), 7.40 (dd, *J*=8.7, 2.4 Hz, 1 H)

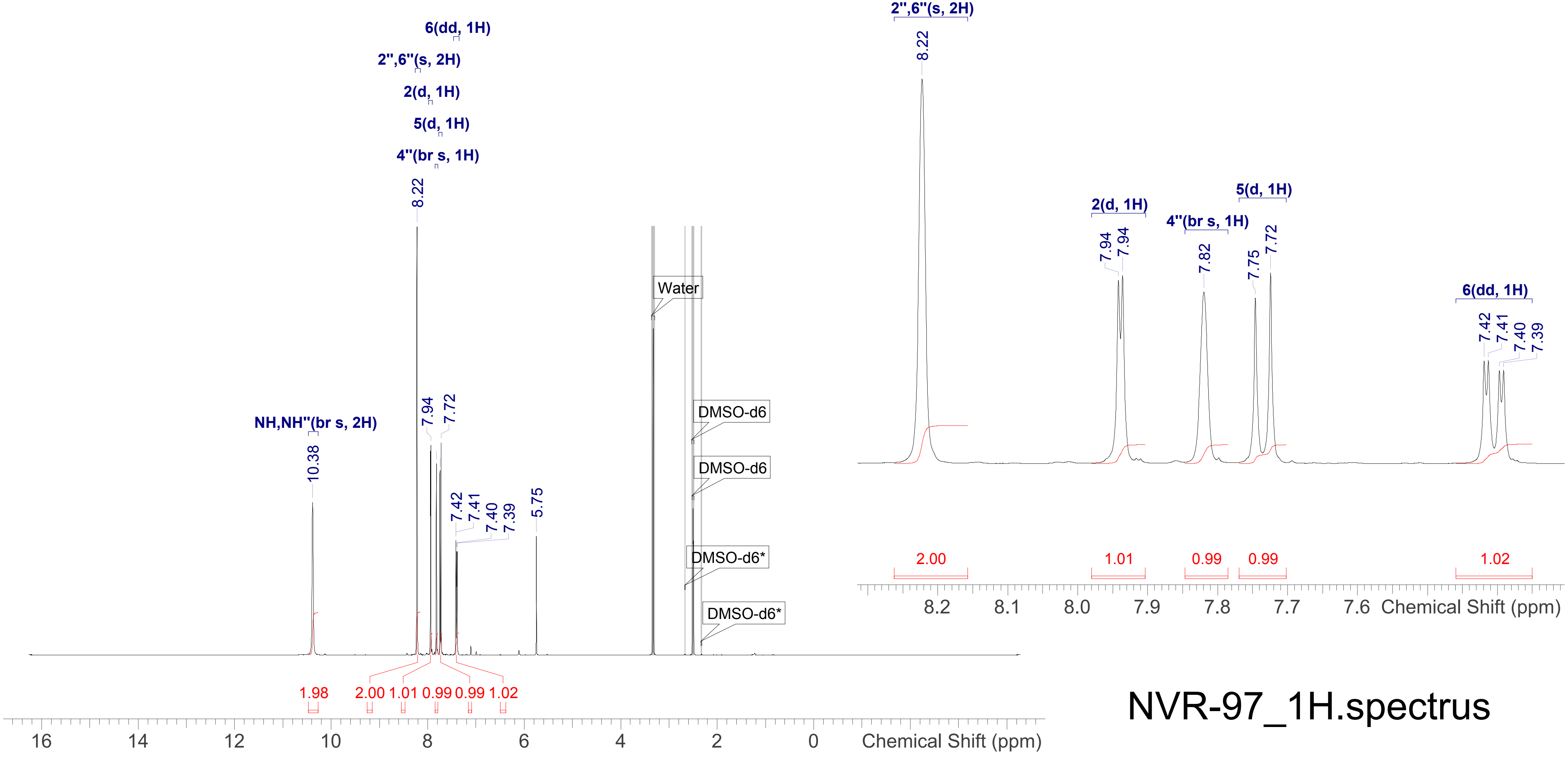

NVR-97_1H.spectrus

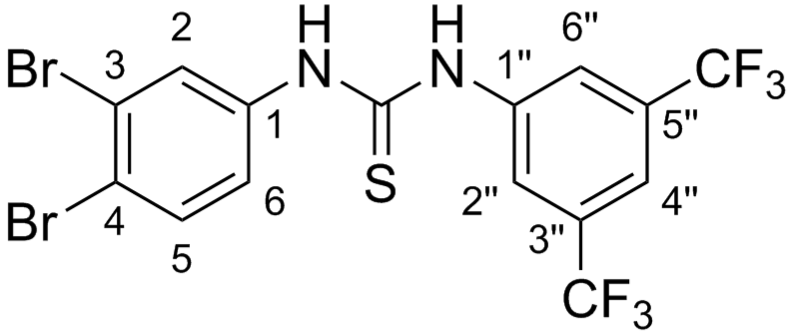

Shift (ppm)	C	m	J (Hz)	Assign
180.0	1	s	-	CS
141.5	1	s	-	1"
139.4	1	s	-	1
133.6	1	s	-	5
130.1	2	q	32.8	5", 3"
128.5	1	s	-	2
124.8	1	s	-	6
123.9	2	br q	3.9	6", 2"
123.5	1	s	-	3
123.2	2	q	272.7	3"-CF3, 5"-CF3
119.3	1	s	-	4
117.3	1	br spt	3.9	4"

Acquisition Time (sec)	1.0224
Date	05 Jul 2019 11:51:29
Date Stamp	05 Jul 2019 11:51:29
Frequency (MHz)	100.5977
Nucleus	13C
Number of Transients	16
Solvent	DMSO-d6
Temperature (degree C)	25.000

^{13}C NMR (101 MHz, *DMSO-d_6*) δ ppm 180.0 (s, 1 C), 141.5 (s, 1 C), 139.4 (s, 1 C), 133.6 (s, 1 C), 130.1 (q, *J*=32.8 Hz, 2 C), 128.5 (s, 1 C), 124.8 (s, 1 C), 123.9 (br q, *J*=3.9 Hz, 2 C), 123.5 (s, 1 C), 119.3 (s, 1 C), 123.2 (q, *J*=272.7 Hz, 2 C), 117.3 (br spt, *J*=3.9 Hz, 1 C)

NVR-97_13C

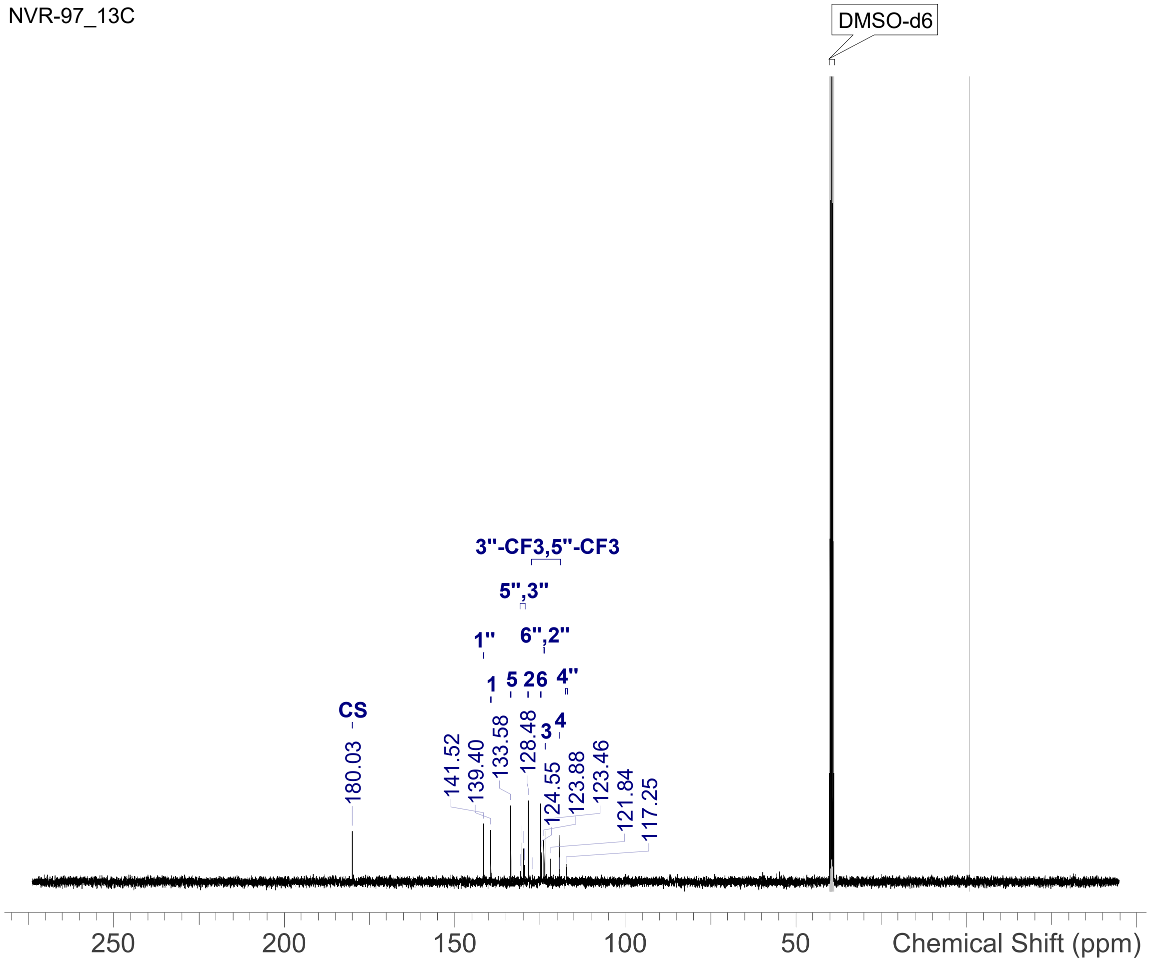

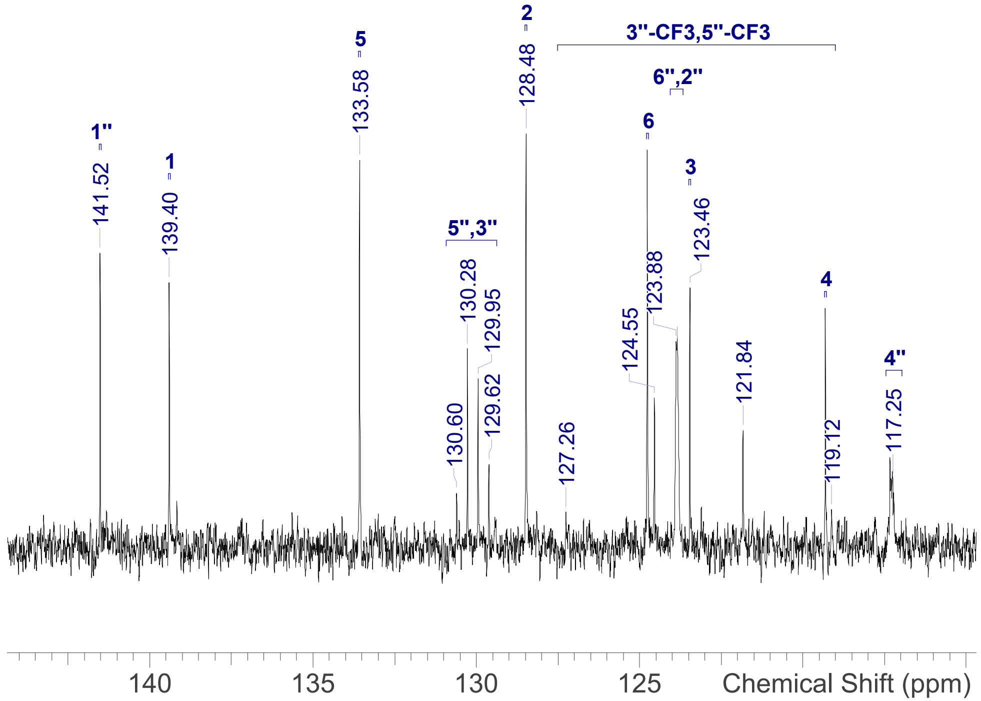

NVR-97_13C.spectrus

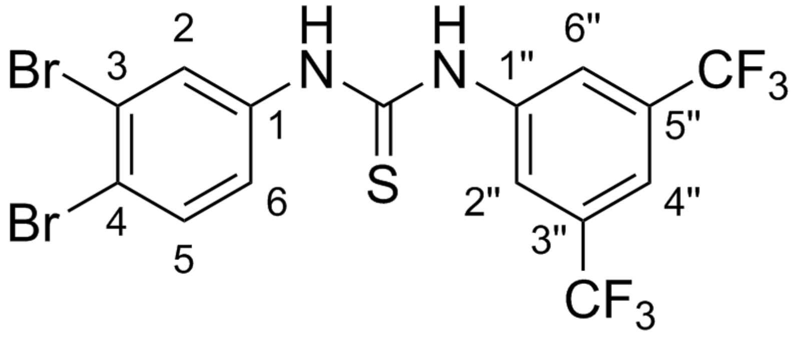

Shift (ppm)	F	m
-61.54	6	s

Acquisition Time (sec)	1.4680
Date	05 Jul 2019 18:46:10
Date Stamp	05 Jul 2019 18:46:10
Frequency (MHz)	376.4419
Nucleus	19F
Number of Transients	128
Solvent	DMSO-d6
Temperature (degree C)	25.001

^{19}F NMR (376 MHz, *DMSO-d_6*) δ ppm -61.54 (s, 6 F)

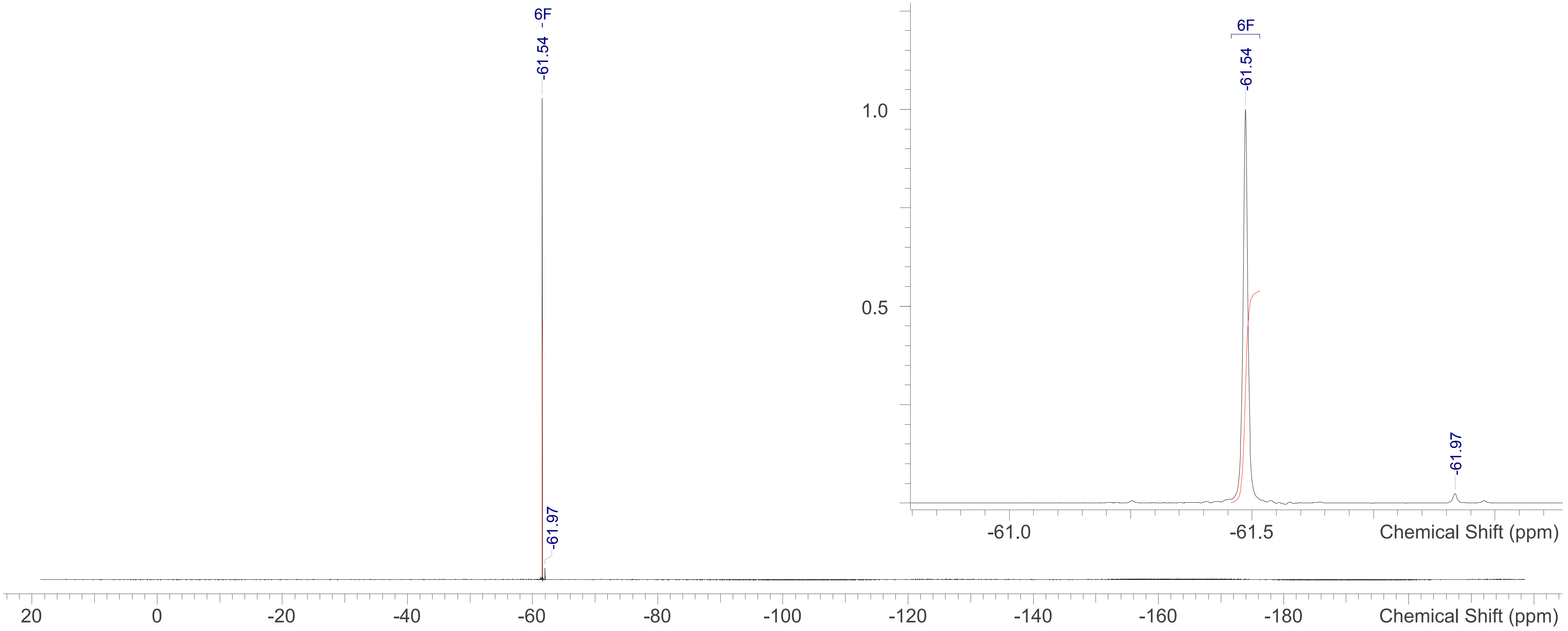

NVR-97_19F.spectrus

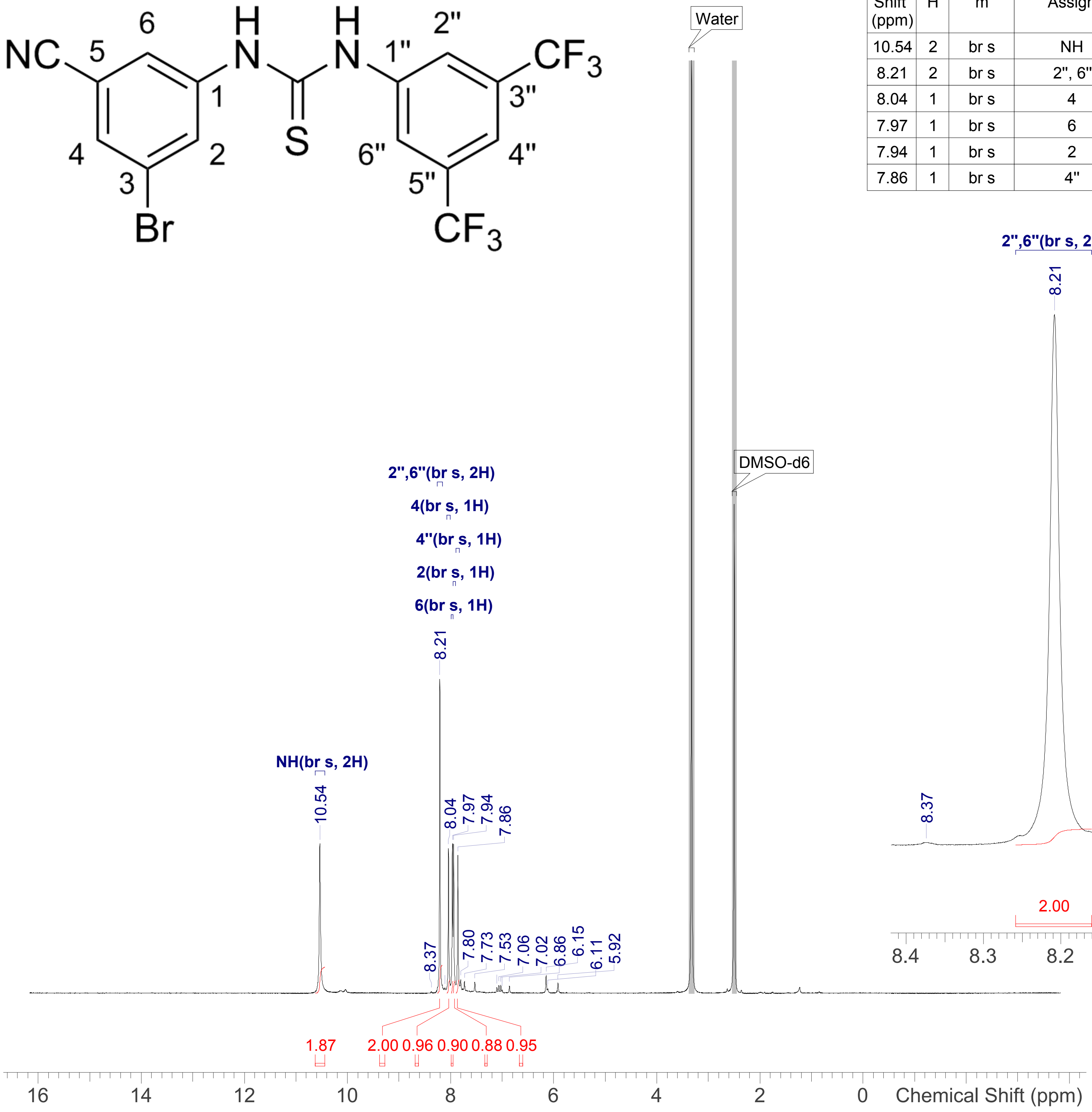

Shift (ppm)	H	m	Assign
10.54	2	br s	NH
8.21	2	br s	2'', 6''
8.04	1	br s	4
7.97	1	br s	6
7.94	1	br s	2
7.86	1	br s	4''

Acquisition Time (sec)	6.5536
Date	22/07/2019 10:37:00
Date Stamp	22/07/2019 10:37:00
Frequency (MHz)	500.1930
Nucleus	1H
Number of Transients	16
Solvent	DMSO-d6

^{1}H NMR (500 MHz, *DMSO-d$_6$*) δ ppm 10.54 (br s, 2 H), 8.21 (br s, 2 H), 8.04 (br s, 1 H), 7.97 (br s, 1 H), 7.94 (br s, 1 H), 7.86 (br s, 1 H)

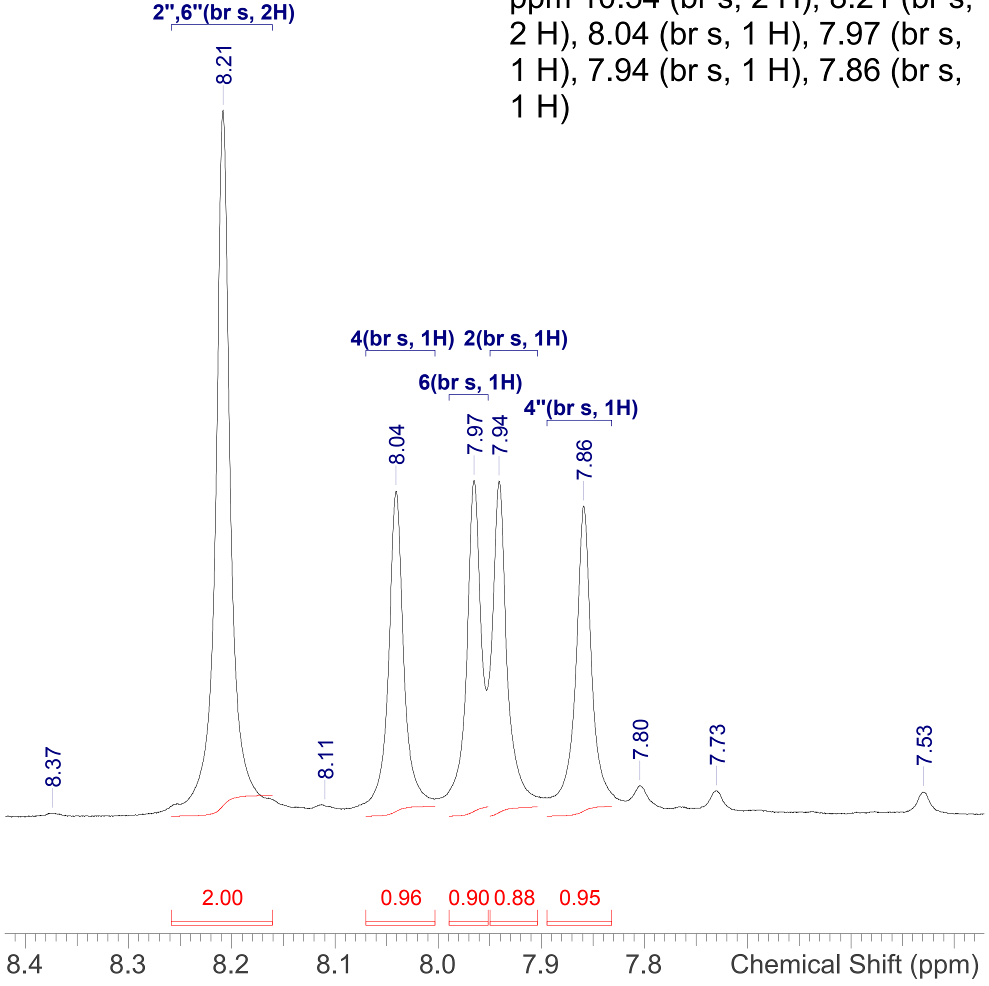

NVR-104_1H.spectrus

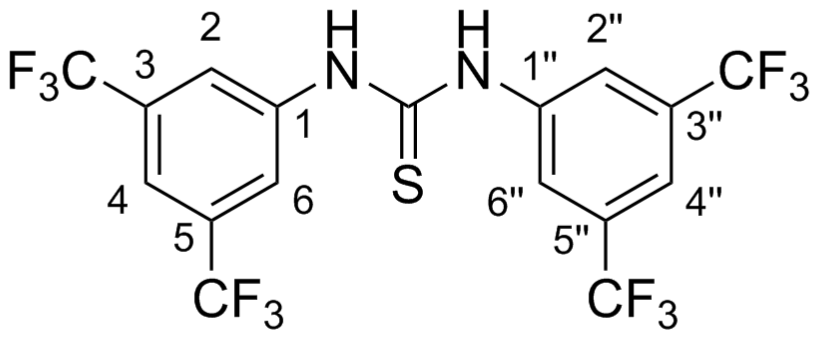

No.	Shift (ppm)	H	m	Assign
1	10.65	2	s	NH", NH
2	8.21	4	s	2", 6", 2, 6
3	7.83	2	s	4", 4

Acquisition Time (sec)	3.9846
Date	05 Jul 2019 11:59:29
Date Stamp	05 Jul 2019 11:59:29
Frequency (MHz)	400.0700
Nucleus	1H
Number of Transients	4
Solvent	DMSO-d6
Temperature (degree C)	25.000

^{1}H NMR (400 MHz, *DMSO-d_6*) δ ppm 10.65 (s, 2 H), 8.21 (s, 4 H), 7.83 (s, 2 H)

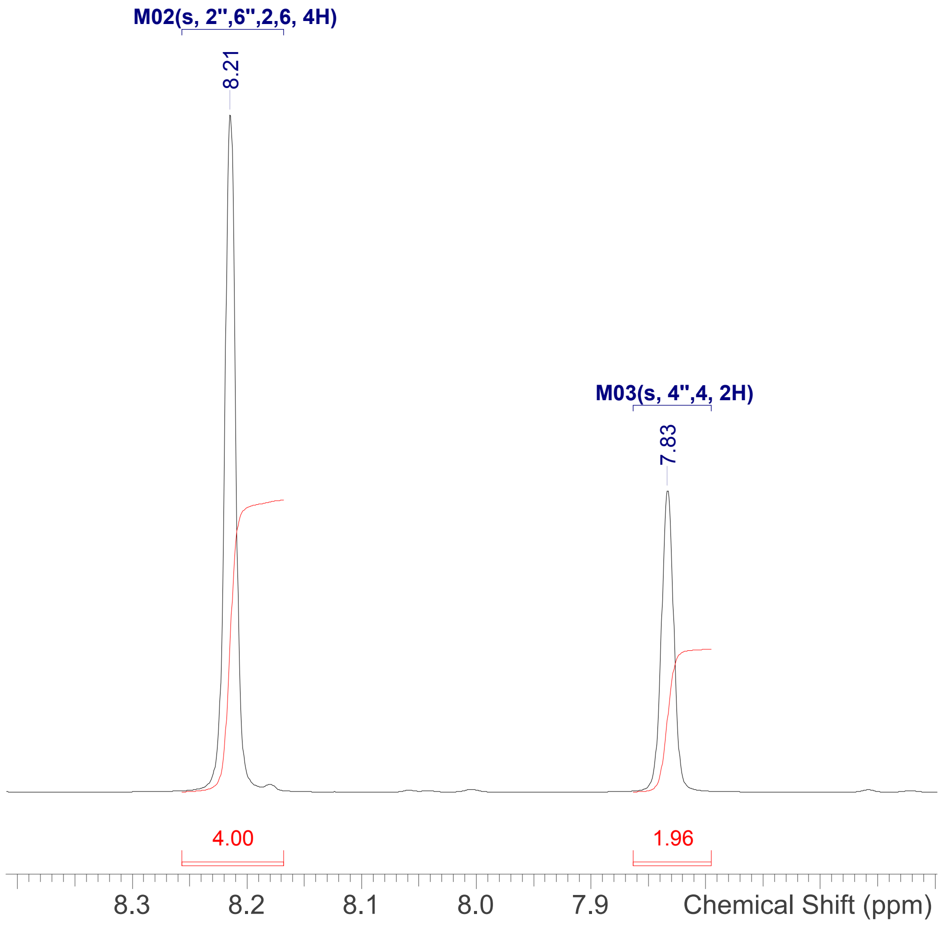

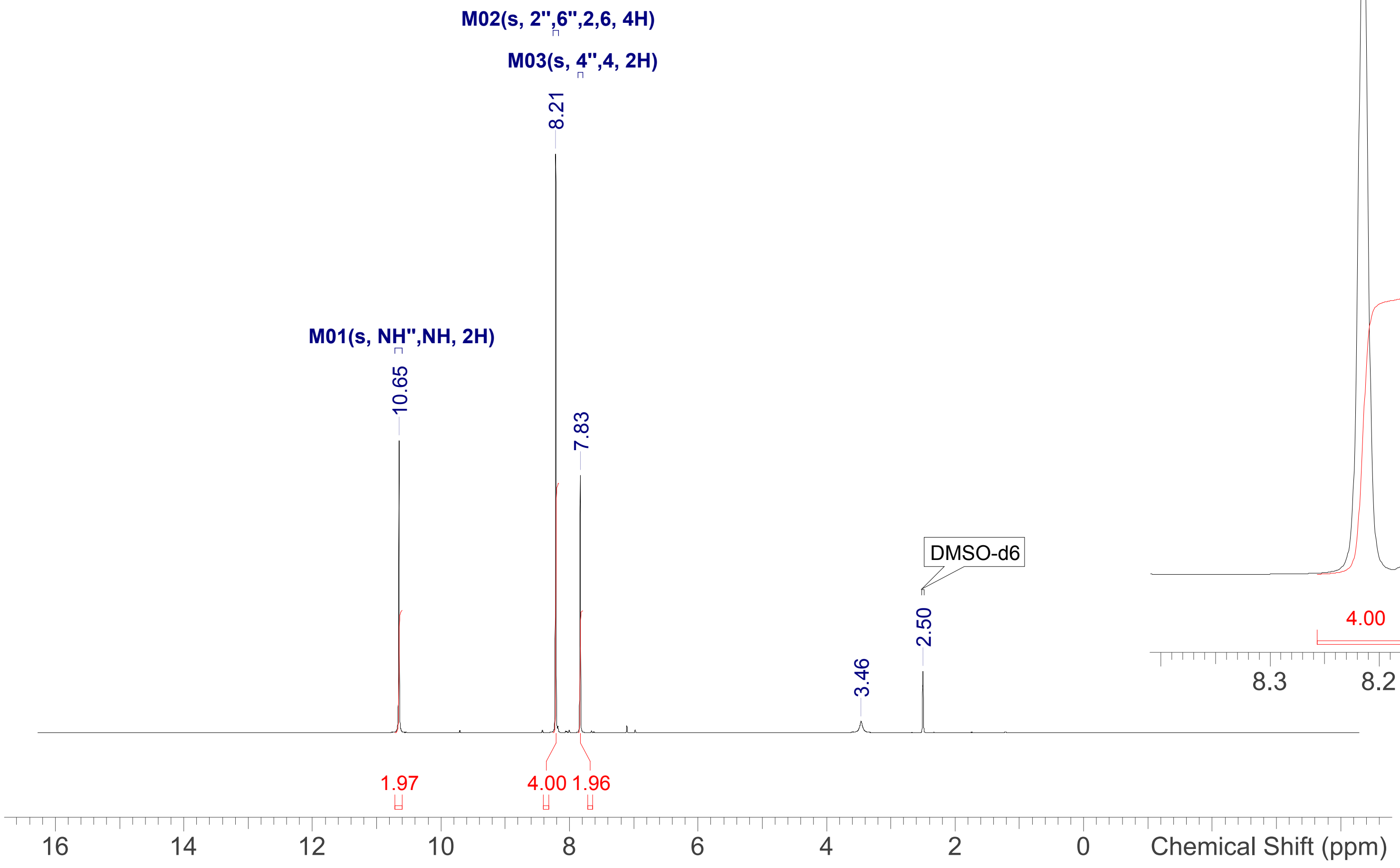

NVR-72_1H.spectrus

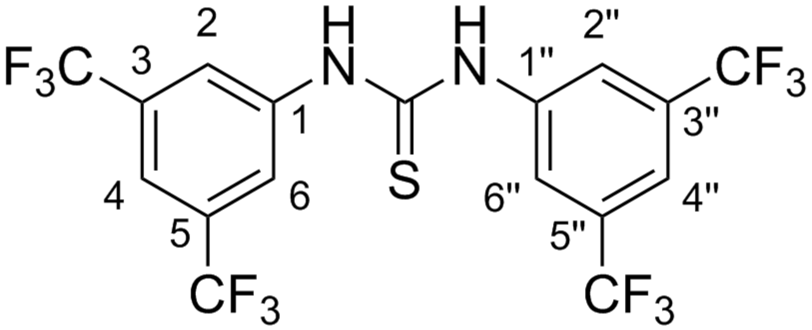

Shift (ppm)	F	m
-61.68	12	s

Acquisition Time (sec)	1.4680
Date	05 Jul 2019 12:07:55
Date Stamp	05 Jul 2019 12:07:55
Frequency (MHz)	376.4419
Nucleus	19F
Number of Transients	16
Solvent	DMSO-d6
Temperature (degree C)	24.997

^{19}F NMR (376 MHz, *DMSO-d_6*) δ ppm -61.68 (s, 12 F)

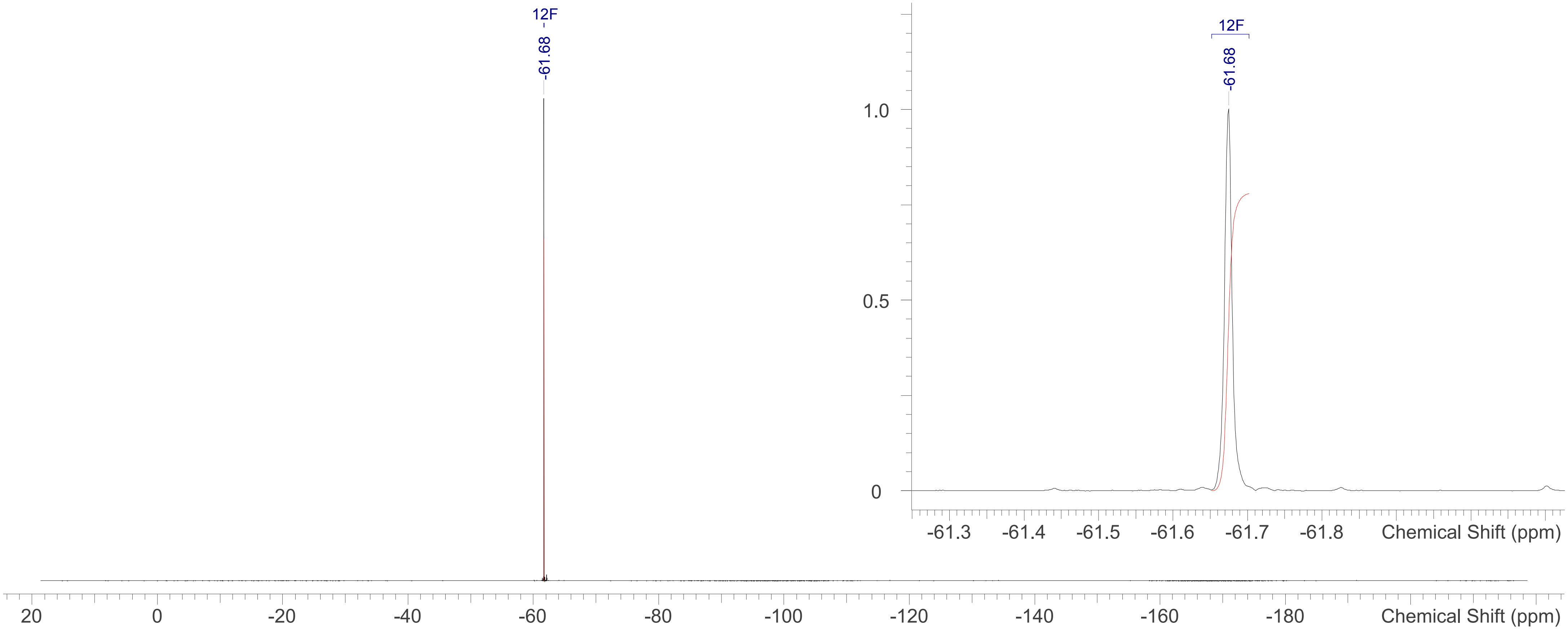

NVR-72_19F.spectrus

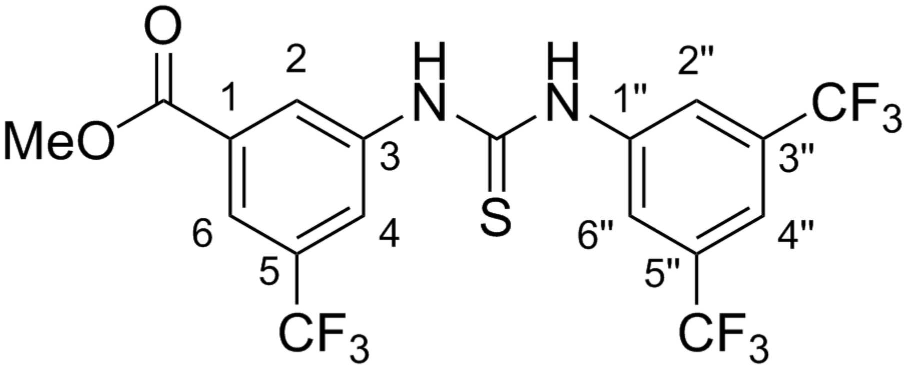

No.	Shift (ppm)	H	m	Assign
1	10.62	1	s	NH
2	10.59	1	s	NH"
3	8.34	1	br s	6
4	8.23	1	br s	4
5	8.21	2	s	2", 6"
6	7.95	1	br s	2
7	7.85	1	br s	4"
8	3.90	3	s	COOMe

Acquisition Time (sec)	4.0894
Date	21 Jun 2019 11:47:23
Date Stamp	21 Jun 2019 11:47:23
Frequency (MHz)	400.1300
Nucleus	1H
Number of Transients	16
Solvent	DMSO-d6
Temperature (degree C)	21.600

^{1}H NMR (400 MHz, *DMSO-d_6*) δ ppm 10.62 (s, 1 H), 10.59 (s, 1 H), 8.34 (br s, 1 H), 8.23 (br s, 1 H), 8.21 (s, 2 H), 7.95 (br s, 1 H), 7.85 (br s, 1 H), 3.90 (s, 3 H)

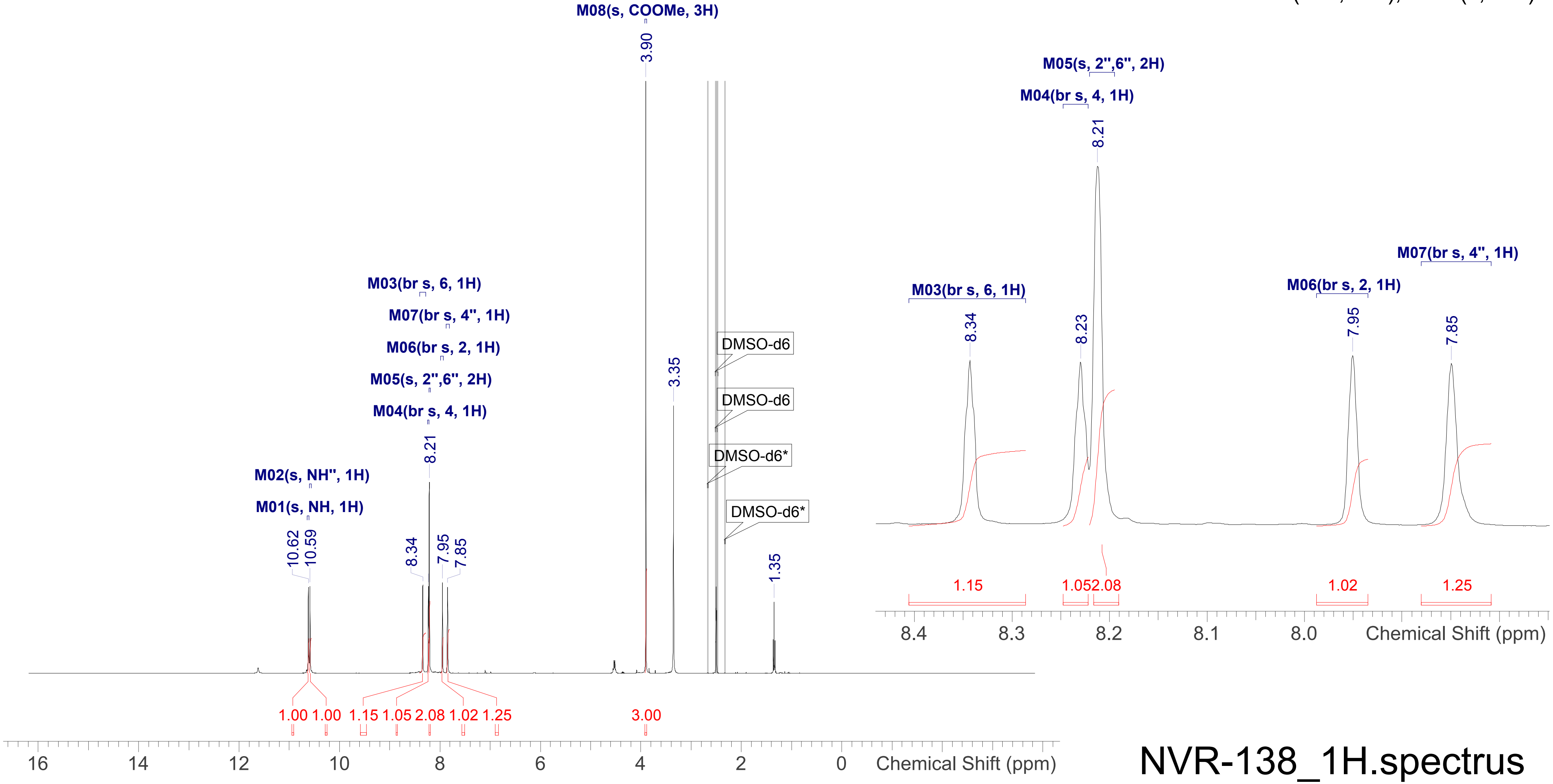

NVR-138_1H.spectrus

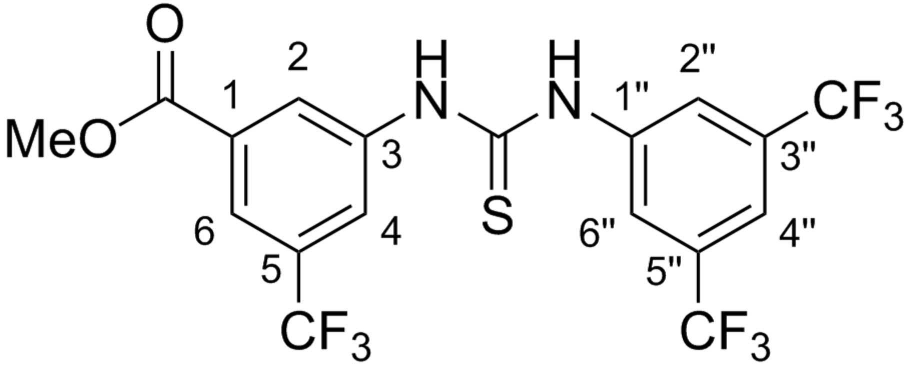

Shift (ppm)	C	m	J (Hz)	Assign
180.5	1	s	-	CS
164.7	1	s	-	COOMe
141.2	1	s	-	1''
140.7	1	s	-	3
131.1	1	s	-	1
130.3	2	q	33.3	3'', 5''
129.7	1	q	32.3	5
127.9	1	s	-	2
124.5	1	br q	3.9	6
124.0	2	br q	3.9	2'', 6''
123.4	1	q	271.9	5-CF3
123.2	2	q	271.9	5''-CF3, 3''-CF3
121.2	1	br q	3.9	4
117.6	1	br spt	3.9	4''
52.8	1	s	-	MeOOC

Acquisition Time (sec)	1.0224
Date	22 Jun 2019 21:00:52
Date Stamp	22 Jun 2019 21:00:52
Frequency (MHz)	100.6128
Nucleus	13C
Number of Transients	256
Solvent	DMSO-d6
Temperature (degree C)	23.100

^{13}C NMR (101 MHz, *DMSO-d_6*) δ ppm 180.5 (s, 1 C), 164.7 (s, 1 C), 141.2 (s, 1 C), 140.7 (s, 1 C), 131.1 (s, 1 C), 130.3 (q, *J*=33.3 Hz, 2 C), 129.7 (q, *J*=32.3 Hz, 1 C), 127.9 (s, 1 C), 124.5 (br q, *J*=3.9 Hz, 1 C), 124.0 (br q, *J*=3.9 Hz, 2 C), 121.2 (br q, *J*=3.9 Hz, 1 C), 123.4 (q, *J*=271.9 Hz, 1 C), 123.2 (q, *J*=271.9 Hz, 2 C), 117.6 (br spt, *J*=3.9 Hz, 1 C), 52.8 (s, 1 C)

NVR-138_13C

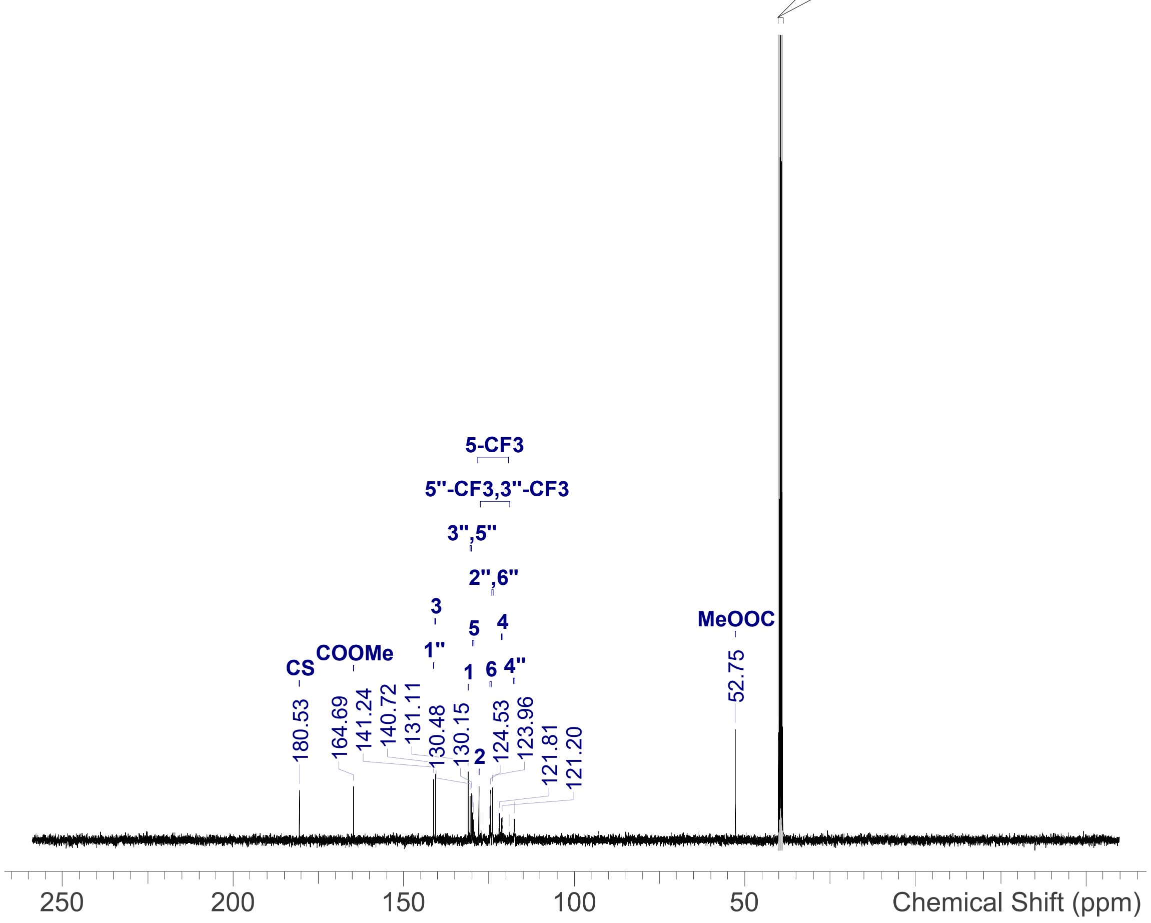

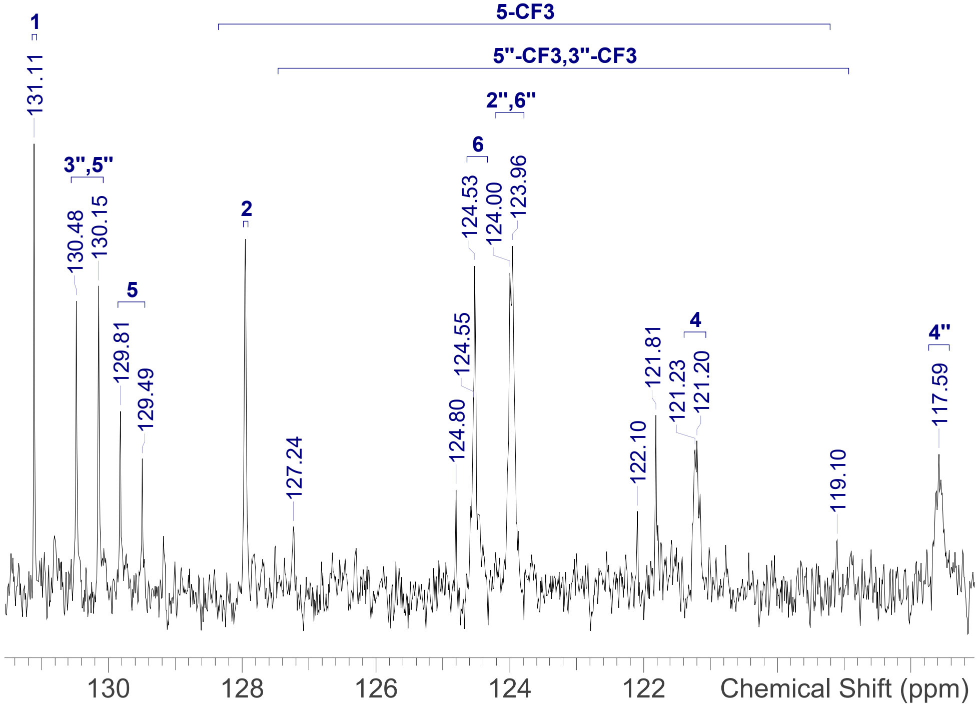

NVR-138_13C.spectrus

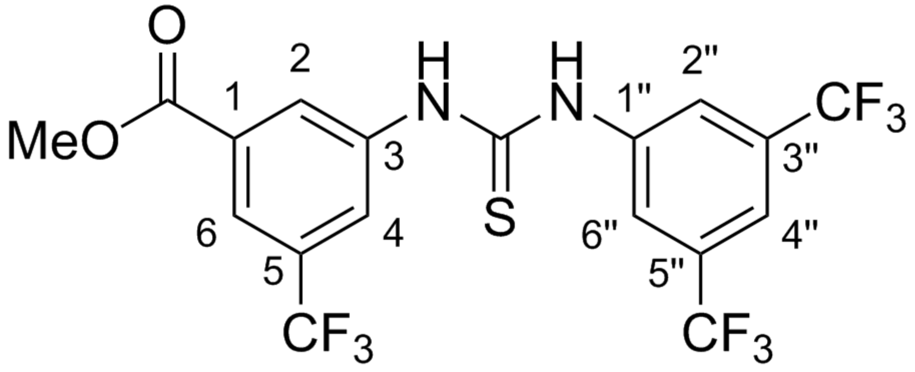

Shift (ppm)	F	m
-61.57	6	s
-61.58	3	s

Acquisition Time (sec)	2.9360
Date	22/06/2019 21:11:00
Date Stamp	22/06/2019 21:11:00
Frequency (MHz)	376.4610
Nucleus	19F
Number of Transients	16
Solvent	DMSO-d6

^{19}F NMR (376 MHz, *DMSO-d_6*) δ ppm -61.57 (s, 6 F), -61.58 (s, 3 F)

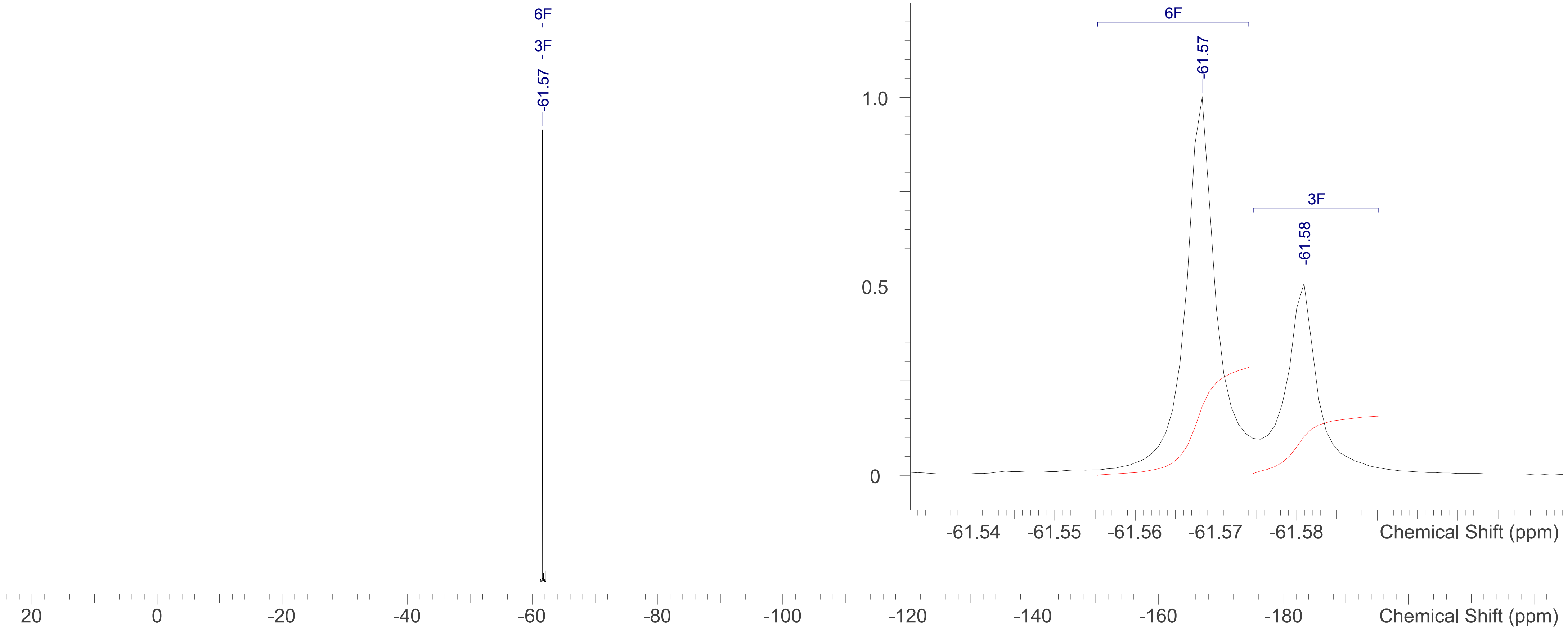

NVR-138_19F.spectrus

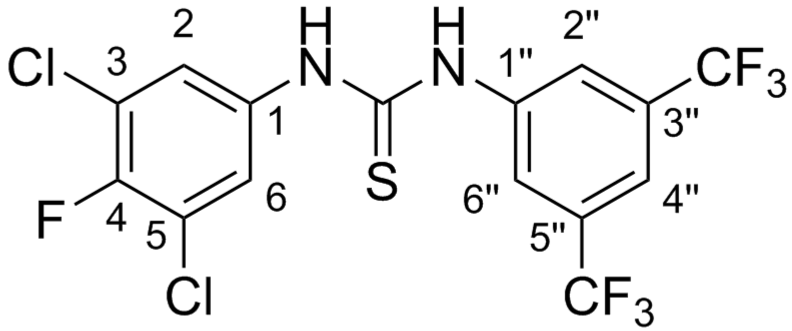

No.	Shift (ppm)	H	m	J (Hz)	Assign
1	10.39	1	s	-	NH
2	10.37	1	s	-	NH"
3	8.21	2	br s	-	2", 6"
4	7.84	1	br s	-	4"
5	7.70	2	d	6.3	2, 6

Acquisition Time (sec)	3.9846
Date	05 Jul 2019 16:25:19
Date Stamp	05 Jul 2019 16:25:19
Frequency (MHz)	400.0700
Nucleus	1H
Number of Transients	4
Solvent	DMSO-d6
Temperature (degree C)	25.001

^{1}H NMR (400 MHz, *DMSO-d_6*) δ ppm 10.39 (s, 1 H), 10.37 (s, 1 H), 8.21 (br s, 2 H), 7.84 (br s, 1 H), 7.70 (d, *J*=6.3 Hz, 2 H)

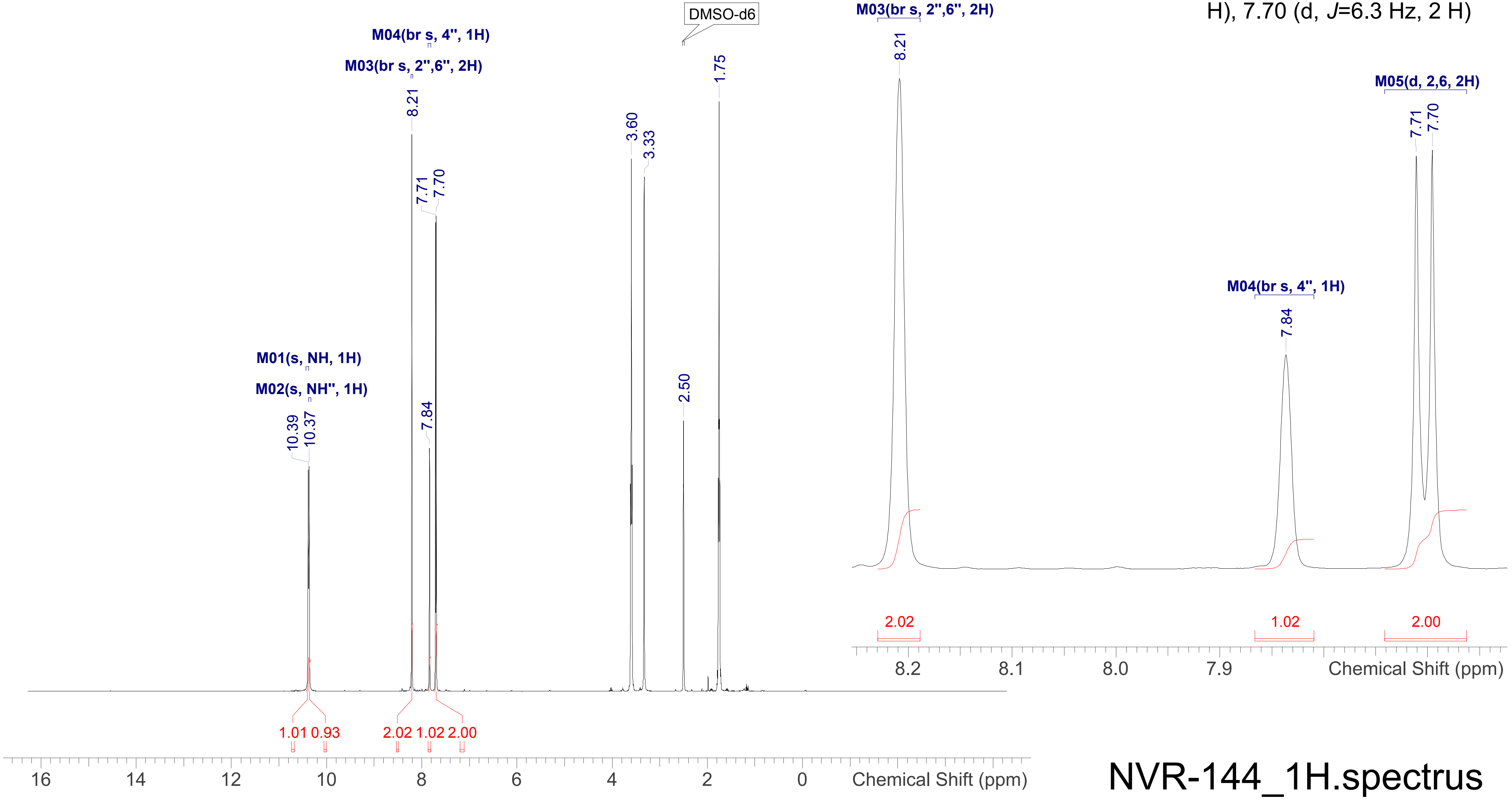

NVR-144_1H.spectrus

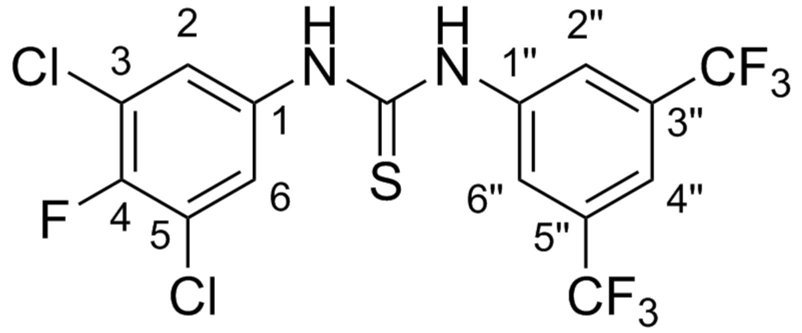

Shift (ppm)	C	m	J (Hz)	Assign
180.5	1	s	-	CS
150.6	1	d	245.5	4
141.4	1	s	-	1"
136.0	1	s	-	1
130.1	2	q	32.9	3", 5"
125.3	2	s	-	2, 6
124.2	2	br q	3.9	2", 6"
123.2	2	q	272.4	5"-CF3, 3"-CF3
120.6	2	d	18.6	5, 3
117.5	1	br spt	3.9	4"

Acquisition Time (sec)	1.0224
Date	05 Jul 2019 16:27:19
Date Stamp	05 Jul 2019 16:27:19
Frequency (MHz)	100.5977
Nucleus	13C
Number of Transients	16
Solvent	DMSO-d6
Temperature (degree C)	24.999

^{13}C NMR (101 MHz, *DMSO-d$_6$*) δ ppm 180.5 (s, 1 C), 150.6 (d, *J*=245.5 Hz, 1 C), 141.4 (s, 1 C), 136.0 (s, 1 C), 130.1 (q, *J*=32.9 Hz, 2 C), 125.3 (s, 2 C), 124.2 (br q, *J*=3.9 Hz, 2 C), 120.6 (d, *J*=18.6 Hz, 2 C), 123.2 (q, *J*=272.4 Hz, 2 C), 117.5 (br spt, *J*=3.9 Hz, 1 C)

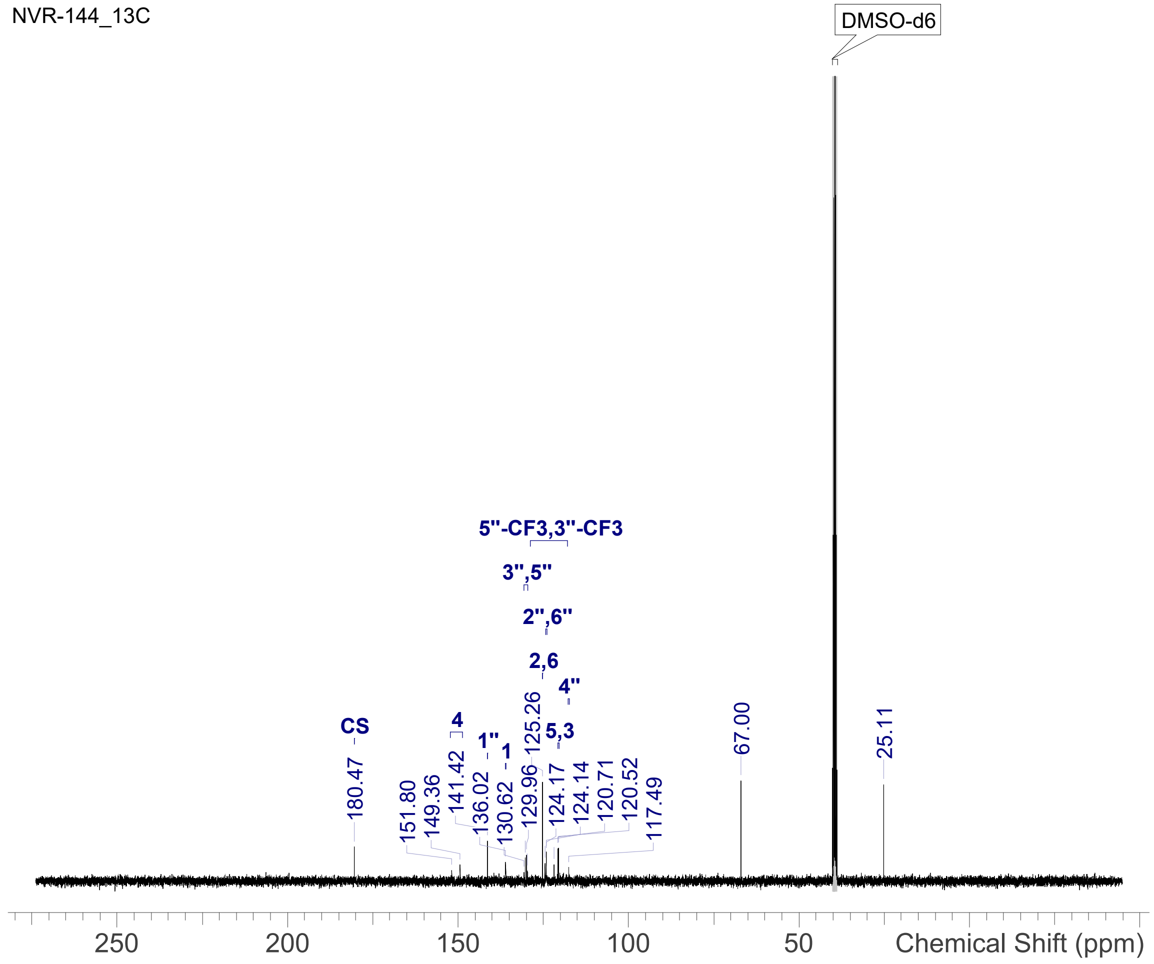

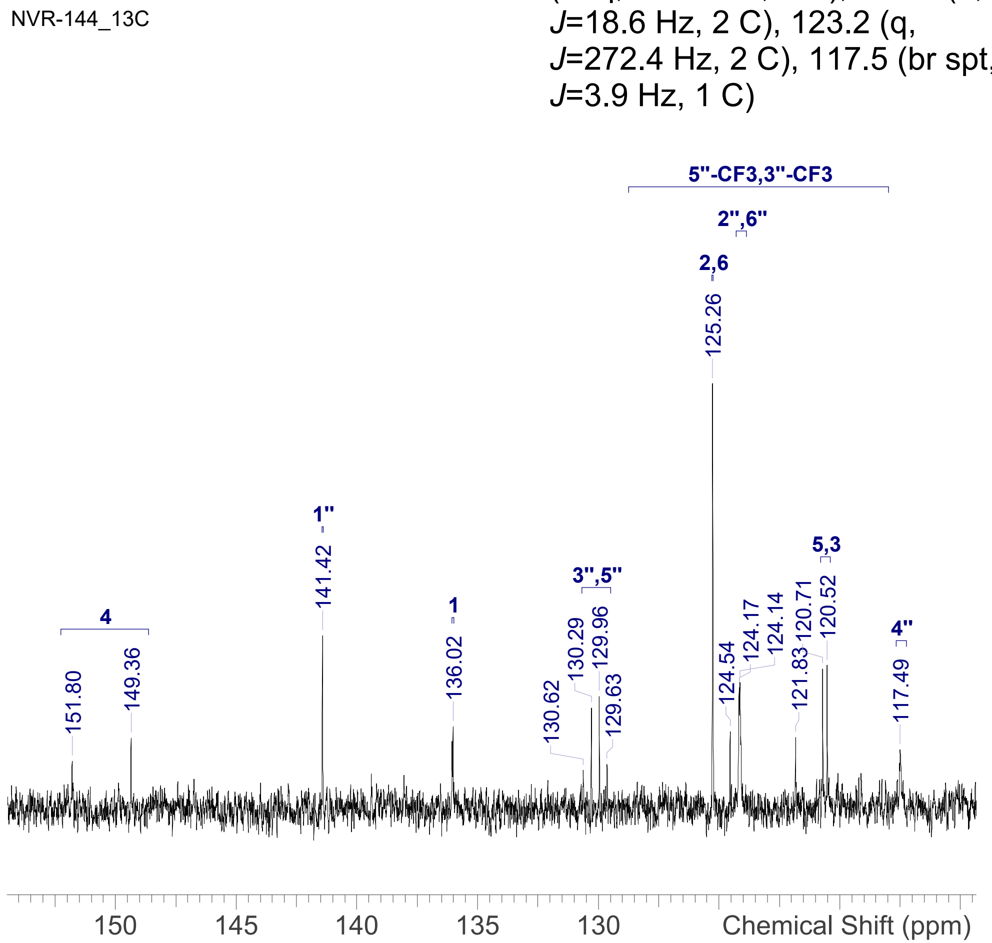

NVR-144_13C.spectrus

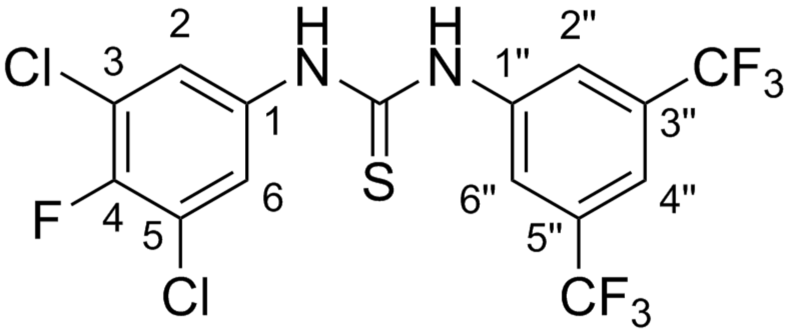

Shift (ppm)	F	m	J (Hz)
-61.53	6	s	-
-122.02	1	t	6.1

Acquisition Time (sec)	1.4680
Date	05 Jul 2019 16:33:51
Date Stamp	05 Jul 2019 16:33:51
Frequency (MHz)	376.4419
Nucleus	19F
Number of Transients	16
Solvent	DMSO-d6
Temperature (degree C)	24.999

^{19}F NMR (376 MHz, *DMSO-d$_6$*) δ ppm -61.53 (s, 6 F), -122.02 (t, *J*=6.1 Hz, 1 F)

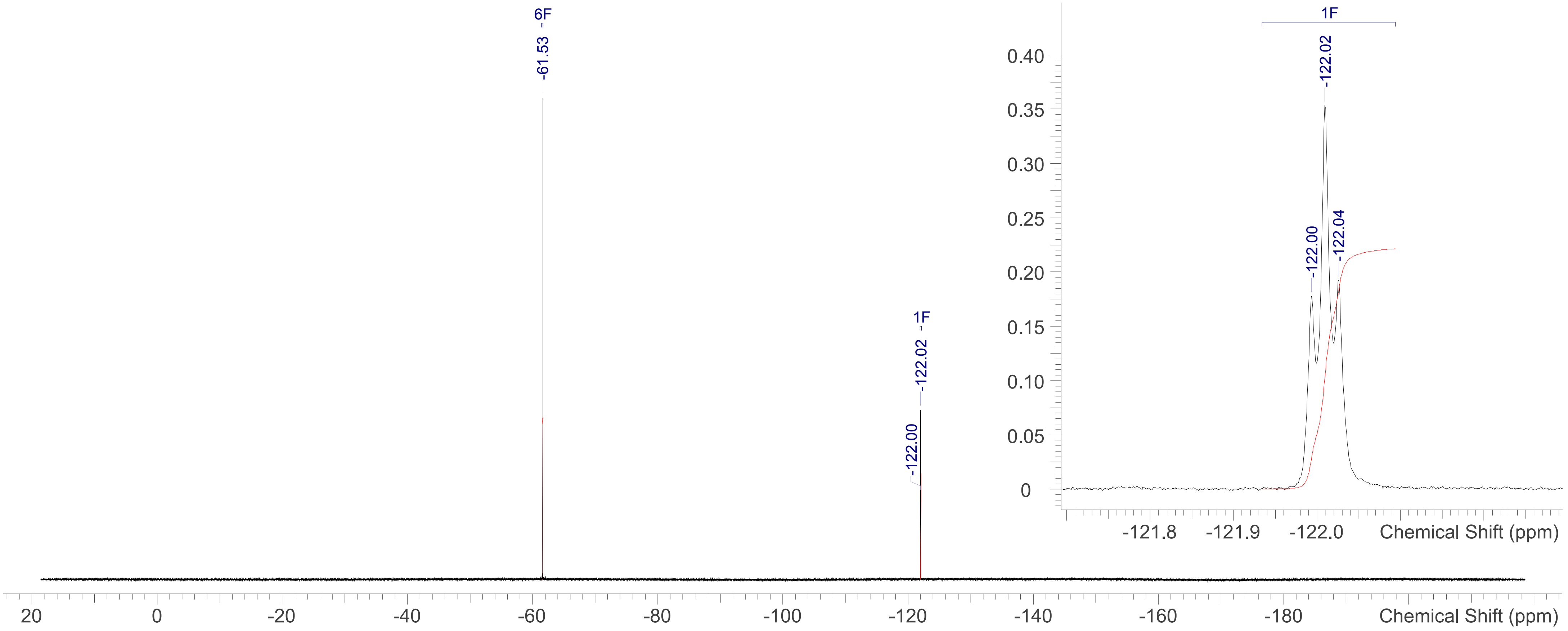

NVR-144_19F.spectrus

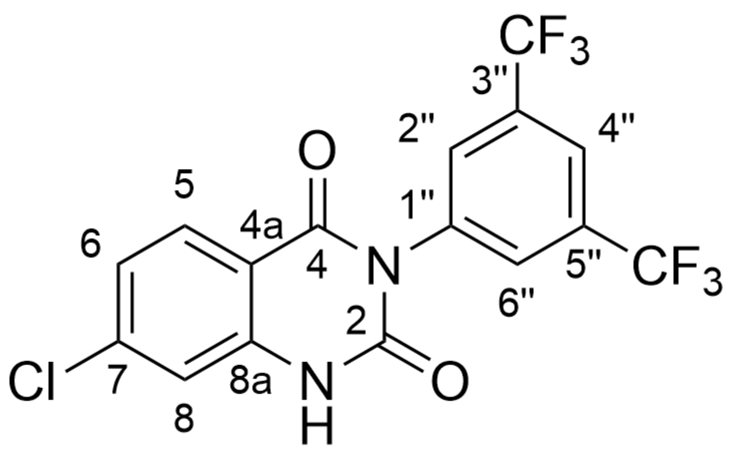

Shift (ppm)	H	m	J (Hz)	Assign
11.85	1	s	-	NH
8.24	3	s	-	2'', 6'', 4''
7.96	1	d	8.5	5
7.31	1	dd	8.5, 1.9	6
7.28	1	d	1.8	8

Acquisition Time (sec)	6.5536
Date	23/10/2018 15:36:00
Date Stamp	23/10/2018 15:36:00
Frequency (MHz)	500.1930
Nucleus	1H
Number of Transients	16
Solvent	DMSO-d6

^{1}H NMR (500 MHz, *DMSO-d_6*) δ ppm 11.85 (s, 1 H), 8.24 (s, 3 H), 7.96 (d, *J*=8.5 Hz, 1 H), 7.31 (dd, *J*=8.5, 1.9 Hz, 1 H), 7.28 (d, *J*=1.8 Hz, 1 H)

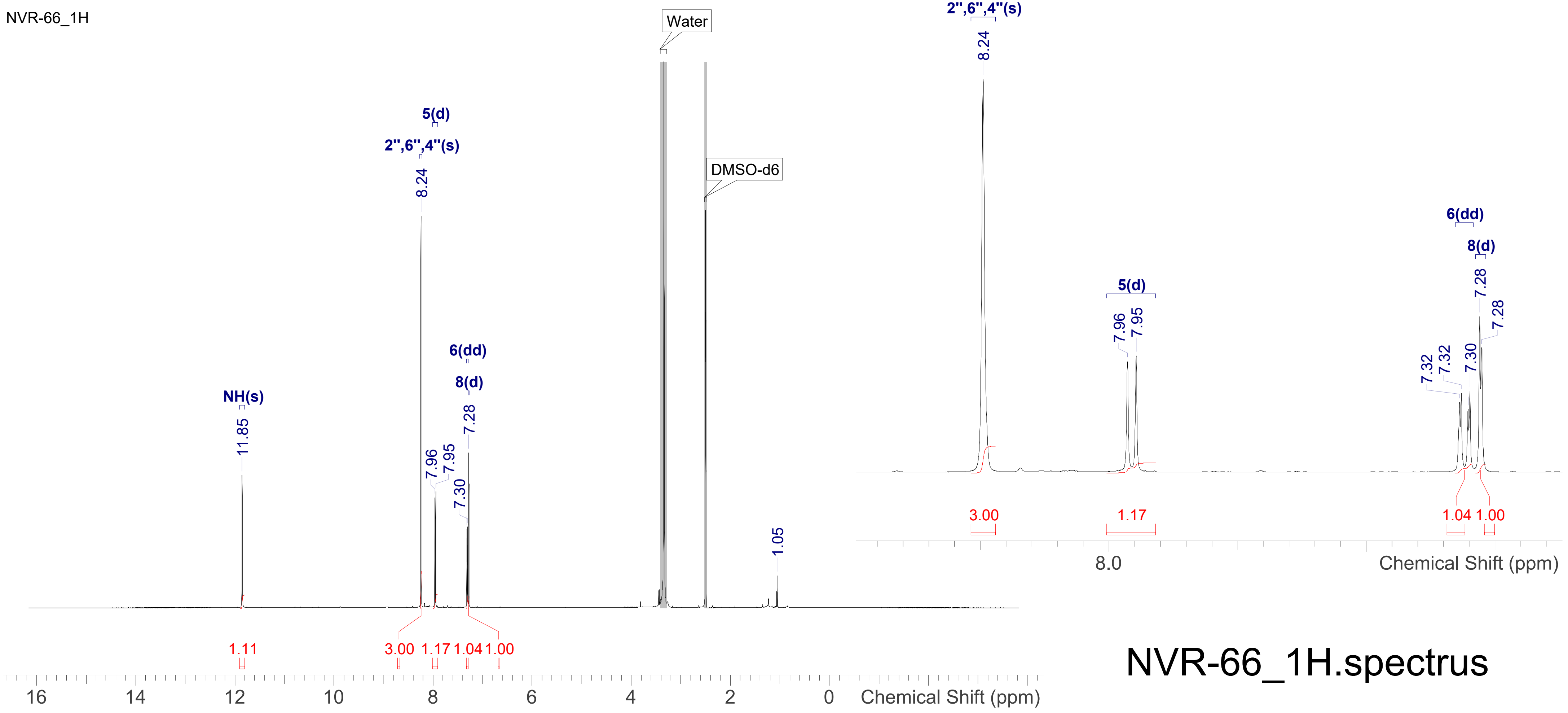

NVR-66_1H.spectrus

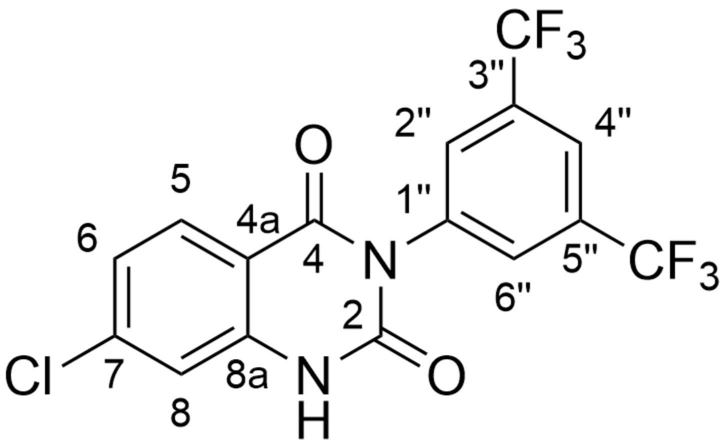

Shift (ppm)	C	m	J (Hz)	Assign
161.5	1	s	-	4
149.8	1	s	-	2
140.9	1	s	-	8a
139.8	1	s	-	7
137.7	1	s	-	1"
130.9	2	q	33.7	3", 5"
130.8	2	br q	3.9	2", 6"
129.6	1	s	-	5
123.0	2	q	272.9	5"-CF3, 3"-CF3
122.9	1	s	-	6
122.4	1	br spt	3.9	4"
114.7	1	s	-	8
113.3	1	s	-	4a

Acquisition Time (sec)	2.0447
Date	23/10/2018 15:39:00
Date Stamp	23/10/2018 15:39:00
Frequency (MHz)	125.7870
Nucleus	13C
Number of Transients	32
Solvent	DMSO-d6

^{13}C NMR (126 MHz, *DMSO-d$_6$*) δ ppm 161.5 (s, 1 C), 149.8 (s, 1 C), 140.9 (s, 1 C), 139.8 (s, 1 C), 137.7 (s, 1 C), 130.8 (br q, *J*=3.9 Hz, 2 C), 130.9 (q, *J*=33.7 Hz, 2 C), 129.6 (s, 1 C), 122.9 (s, 1 C), 122.4 (br spt, *J*=3.9 Hz, 1 C), 123.0 (q, *J*=272.9 Hz, 2 C), 114.7 (s, 1 C), 113.3 (s, 1 C)

NVR-66_13C

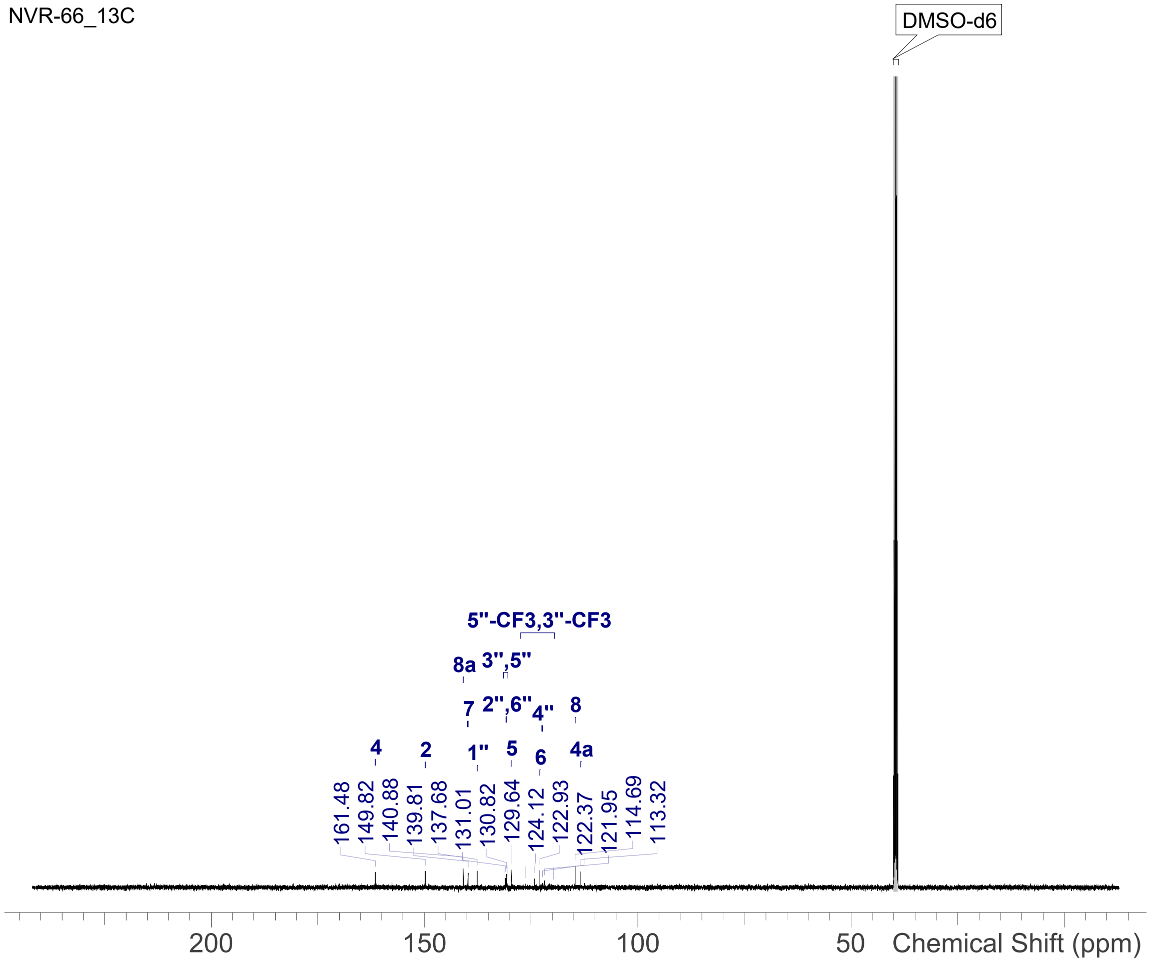

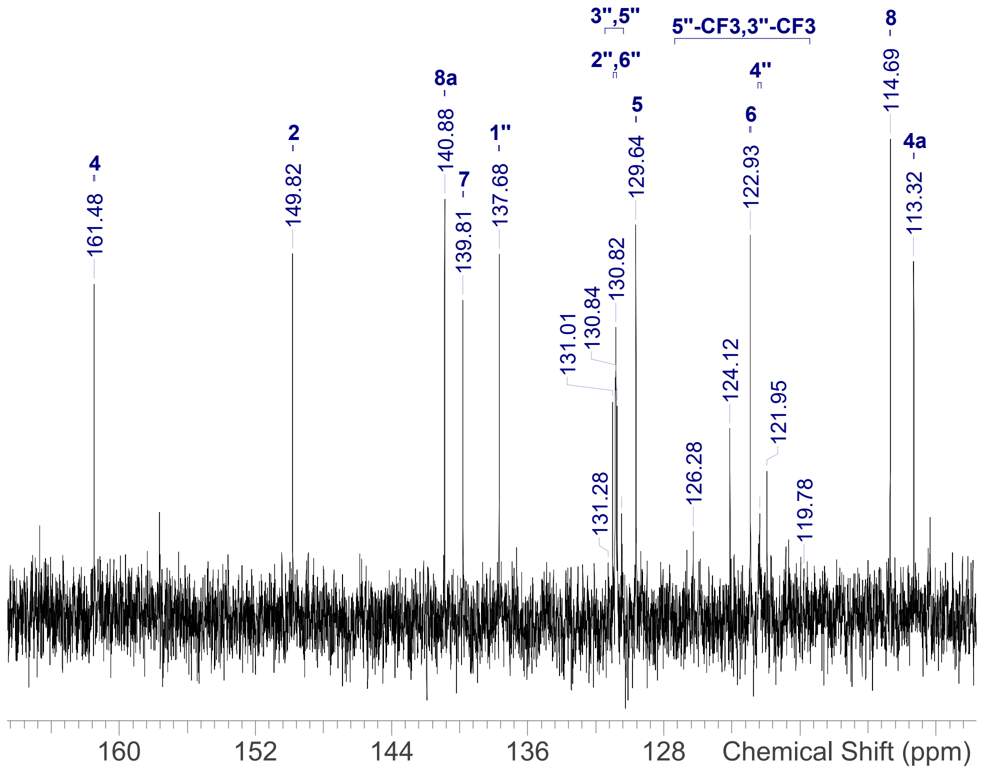

NVR-66_13C.spectrus

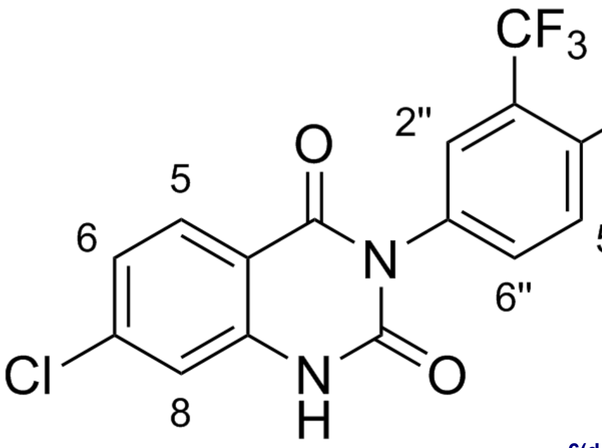

Shift (ppm)	H	m	J (Hz)	Assign
11.79	1	br s	-	NH
7.99	1	d	2.3	2''
7.94	1	d	8.4	5
7.88	1	d	8.4	5''
7.73	1	dd	8.5, 2.4	6
7.28	1	dd	8.5, 2.0	6''
7.25	1	d	1.8	8

Acquisition Time (sec)	6.5536
Date	21/11/2018 14:12:00
Date Stamp	21/11/2018 14:12:00
Frequency (MHz)	500.1930
Nucleus	1H
Number of Transients	16
Solvent	DMSO-d6

^{1}H NMR (500 MHz, *DMSO-d$_6$*) δ ppm 11.79 (br s, 1 H), 7.99 (d, *J*=2.3 Hz, 1 H), 7.94 (d, *J*=8.4 Hz, 1 H), 7.88 (d, *J*=8.4 Hz, 1 H), 7.73 (dd, *J*=8.5, 2.4 Hz, 1 H), 7.28 (dd, *J*=8.5, 2.0 Hz, 1 H), 7.25 (d, *J*=1.8 Hz, 1 H)

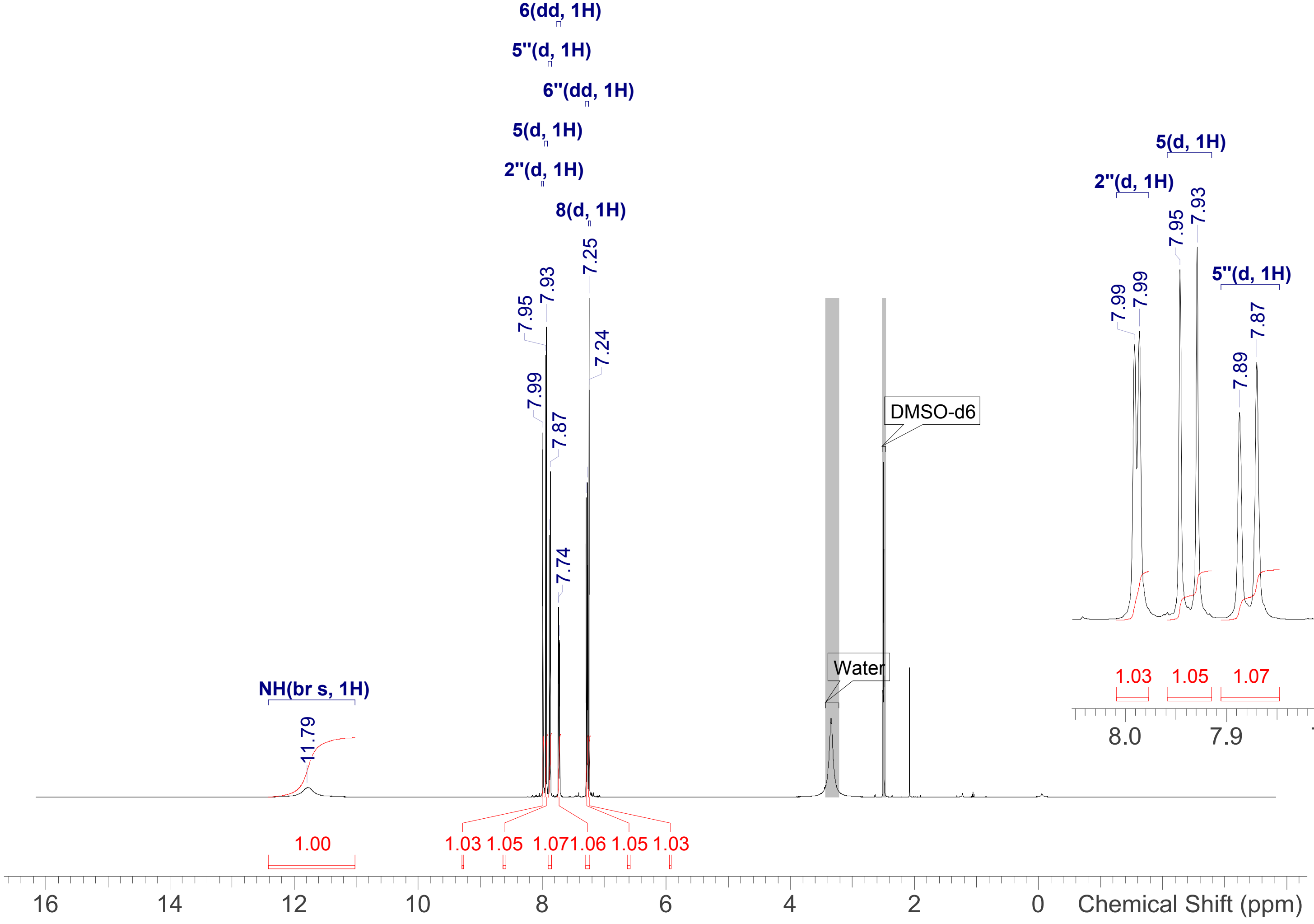

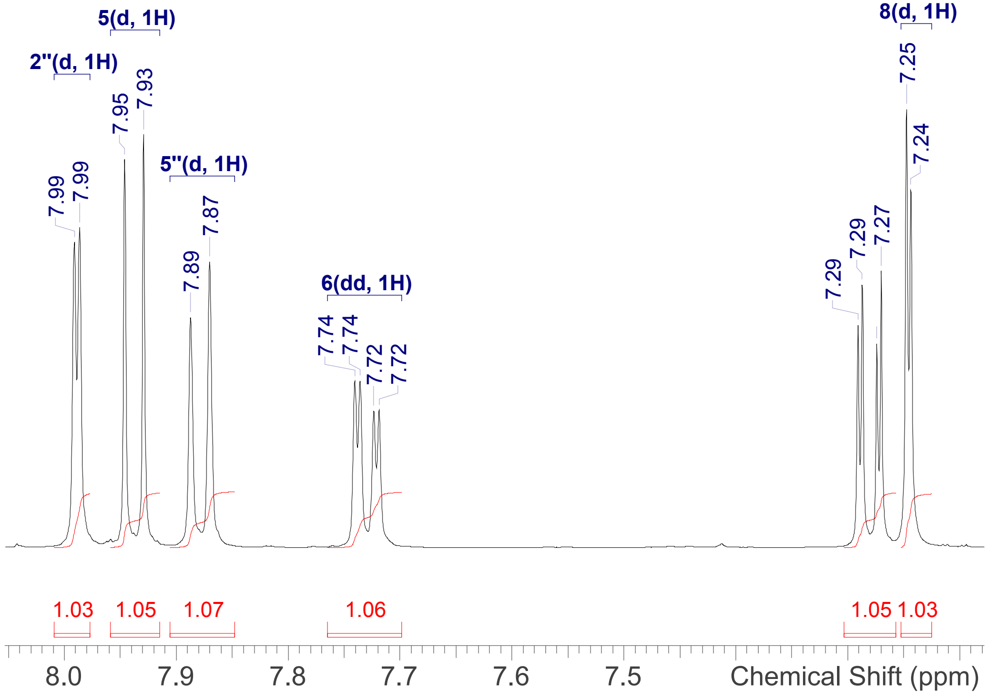

NVR-91_1H.spc

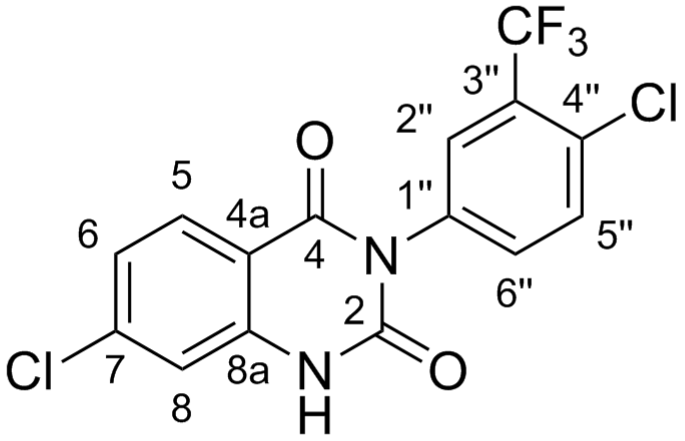

Shift (ppm)	C	m	J (Hz)	Assign
161.5	1	s	-	4
149.9	1	s	-	2
140.9	1	s	-	8a
139.7	1	s	-	7
135.2	1	s	-	6''
135.0	1	s	-	1''
132.3	1	s	-	5''
130.6	1	br q	2.0	4''
129.6	1	s	-	5
129.0	1	q	5.2	2''
127.1	1	q	31.1	3''
122.8	1	s	-	6
122.5	1	q	273.1	CF3
114.6	1	s	-	8
113.3	1	s	-	4a

Acquisition Time (sec)	2.0447
Date	21/11/2018 14:16:00
Date Stamp	21/11/2018 14:16:00
Frequency (MHz)	125.7870
Nucleus	13C
Number of Transients	32
Solvent	DMSO-d6

^{13}C NMR (126 MHz, *DMSO-d_6*) δ ppm 161.5 (s, 1 C), 149.9 (s, 1 C), 140.9 (s, 1 C), 139.7 (s, 1 C), 135.2 (s, 1 C), 135.0 (s, 1 C), 132.3 (s, 1 C), 130.6 (br q, *J*=2.0 Hz, 1 C), 129.6 (s, 1 C), 129.0 (q, *J*=5.2 Hz, 1 C), 127.1 (q, *J*=31.1 Hz, 1 C), 122.8 (s, 1 C), 122.5 (q, *J*=273.1 Hz, 1 C), 114.6 (s, 1 C), 113.3 (s, 1 C)

NVR-91_13C

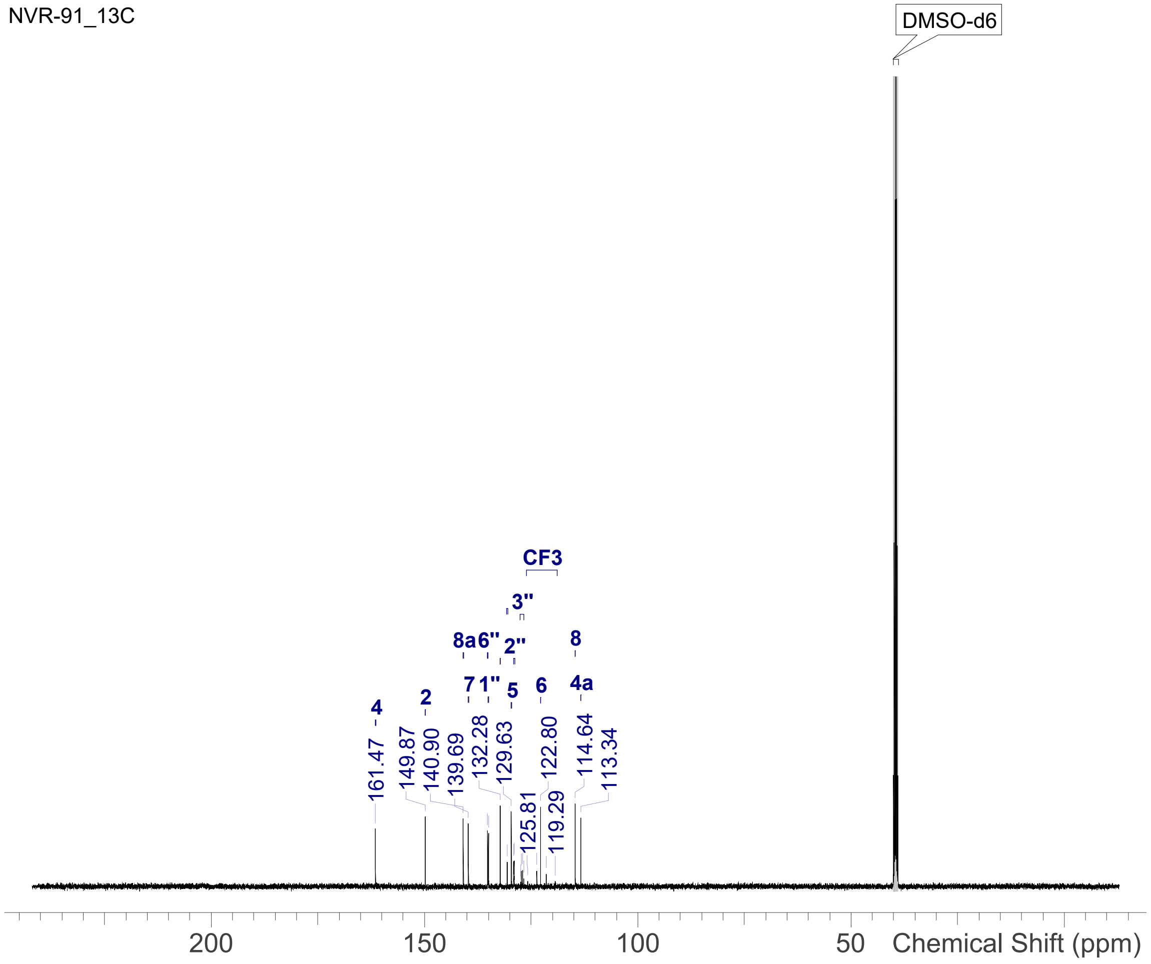

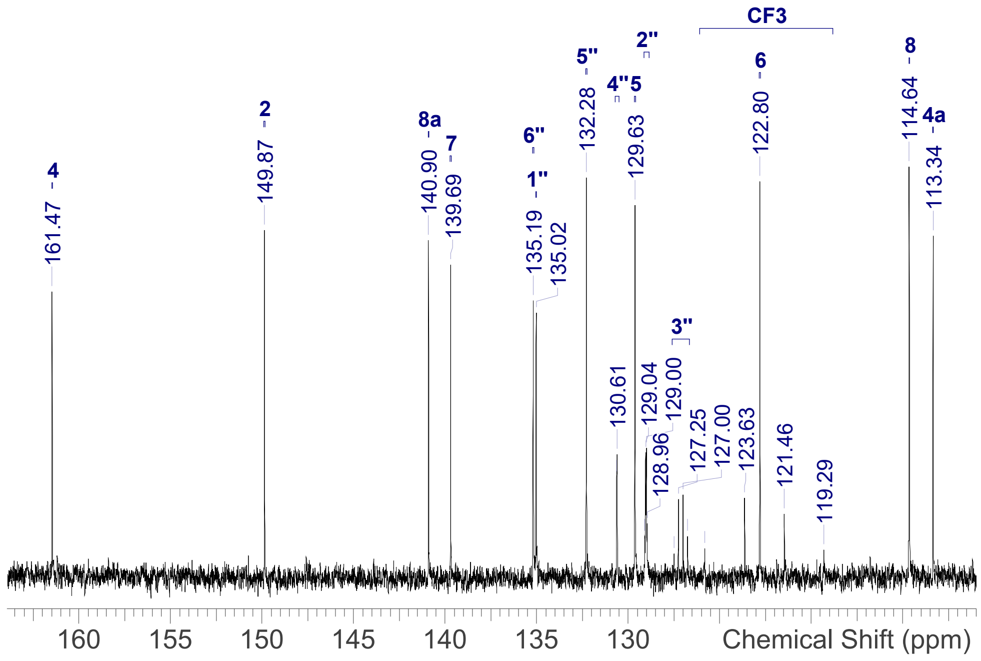

NVR-91_13C.spectrus

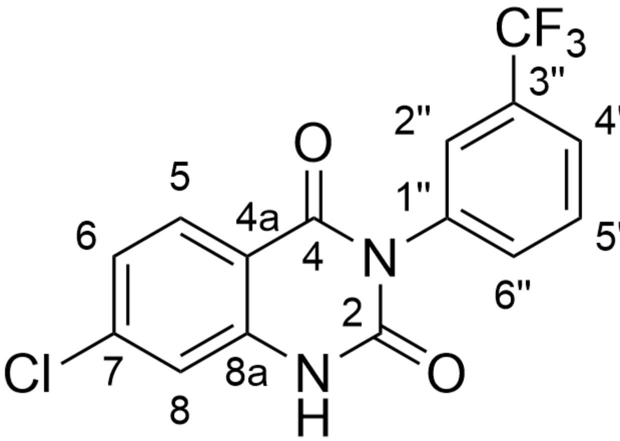

Shift (ppm)	H	m	J (Hz)	Assign
7.93	1	s	-	NH
7.80	2	m	-	8, 2''
7.73	1	t	8.1	5''
7.67	1	br d	7.9	6''
7.26	3	m	-	5, 4'', 6

Acquisition Time (sec)	3.2768
Date	21 Nov 2018 09:42:16
Date Stamp	21 Nov 2018 09:42:16
Frequency (MHz)	500.1900
Nucleus	1H
Number of Transients	16
Solvent	DMSO-d6
Temperature (degree C)	25.001

^{1}H NMR (500 MHz, *DMSO-d_6*) δ ppm 7.93 (s, 1 H), 7.78 - 7.83 (m, 2 H), 7.73 (t, *J*=8.1 Hz, 1 H), 7.67 (br d, *J*=7.9 Hz, 1 H), 7.25 - 7.30 (m, 3 H)

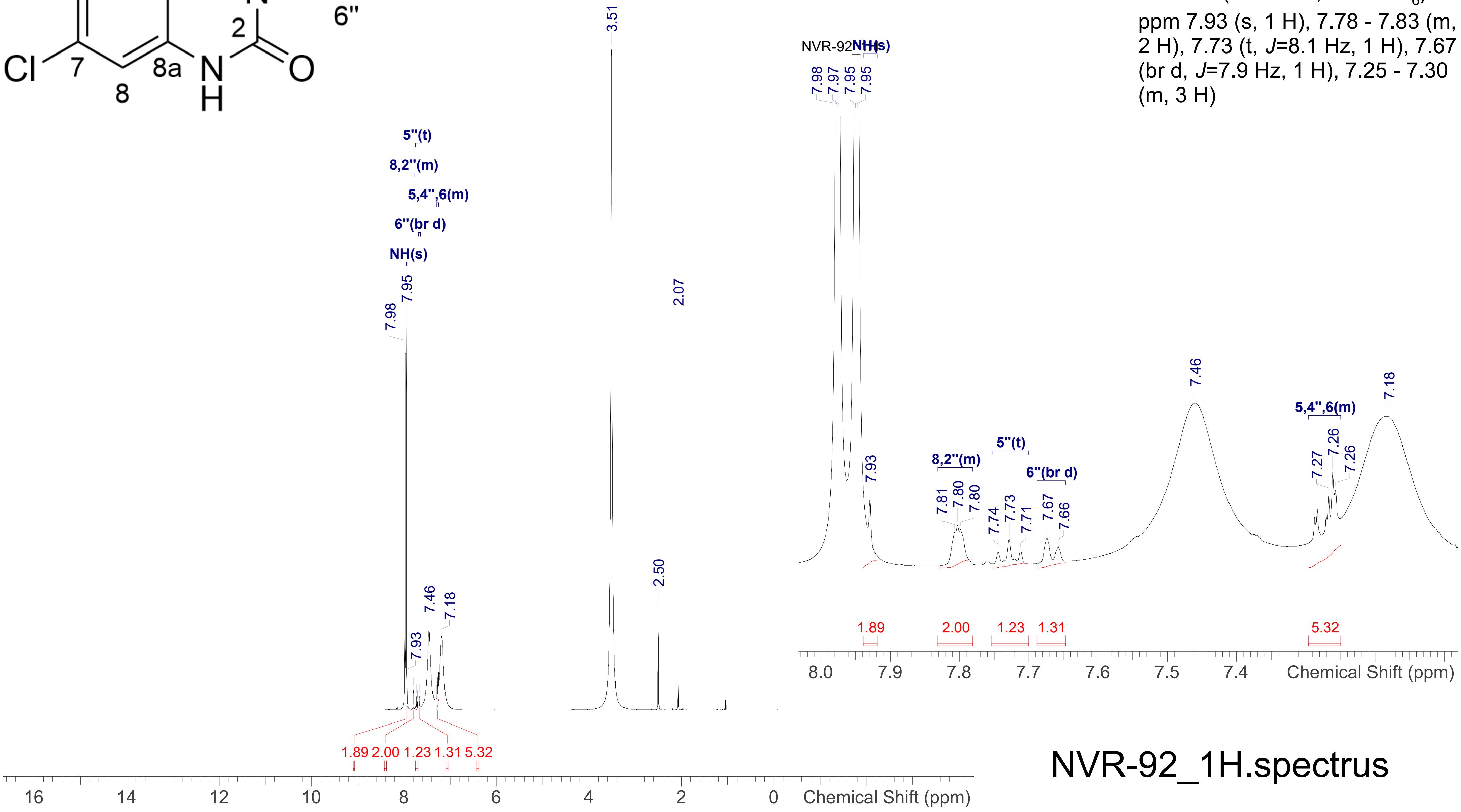

NVR-92_1H.spectrus

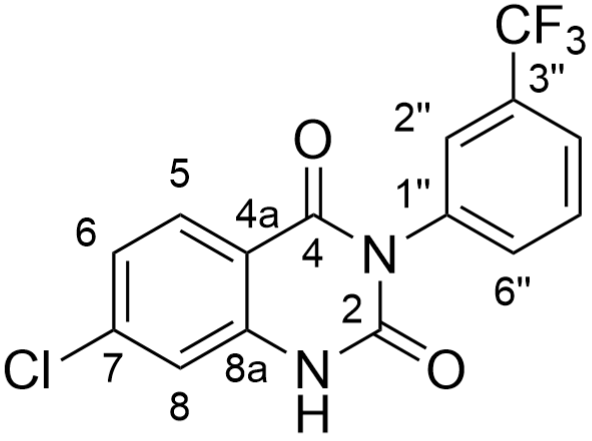

Shift (ppm)	C	m	J (Hz)	Assign
161.8	1	s	-	4
150.2	1	s	-	2
141.1	1	s	-	8a
139.9	1	s	-	7
136.5	1	s	-	1''
133.7	1	s	-	6''
130.3	1	s	-	5''
129.8	1	s	-	5
126.3	1	q	3.9	2''
125.3	1	q	3.9	4''
123.0	1	s	-	6
114.8	1	s	-	8
113.5	1	s	-	4a

Acquisition Time (sec)	1.0224
Date	21 Nov 2018 09:45:17
Date Stamp	21 Nov 2018 09:45:17
Frequency (MHz)	125.7729
Nucleus	13C
Number of Transients	32
Solvent	DMSO-d6
Temperature (degree C)	24.999

^{13}C NMR (126 MHz, *DMSO-d_6*) δ ppm 161.8 (s, 1 C), 150.2 (s, 1 C), 141.1 (s, 1 C), 139.9 (s, 1 C), 136.5 (s, 1 C), 133.7 (s, 1 C), 130.3 (s, 1 C), 129.8 (s, 1 C), 126.3 (q, *J*=3.9 Hz, 1 C), 125.3 (q, *J*=3.9 Hz, 1 C), 123.0 (s, 1 C), 114.8 (s, 1 C), 113.5 (s, 1 C)

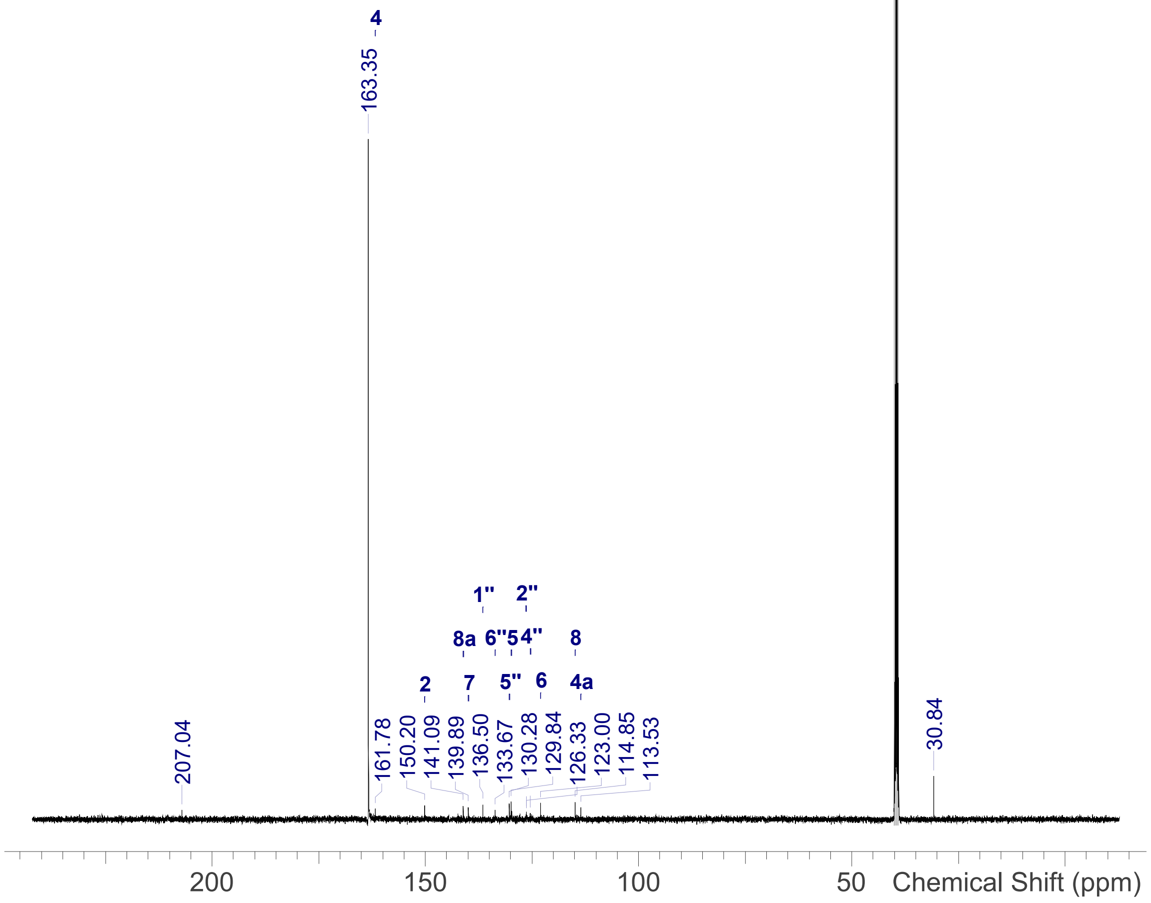

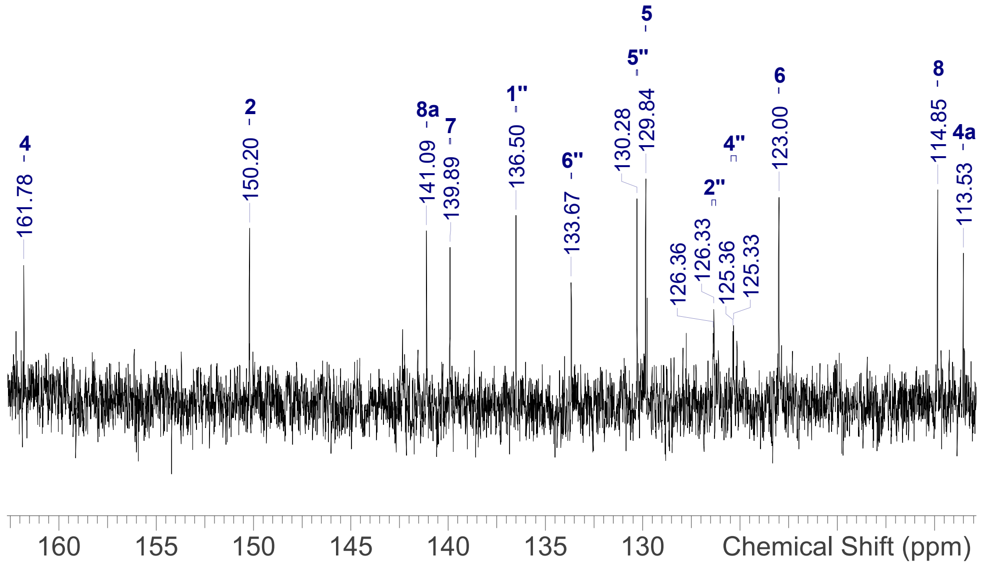

NVR-92_13C.spectrus

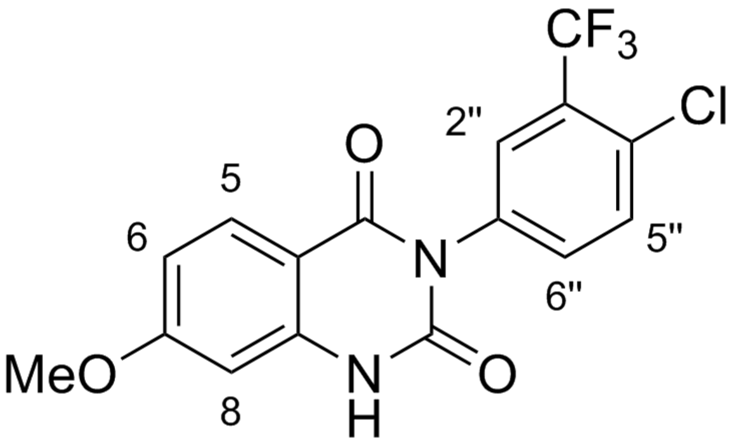

Shift (ppm)	H	m	J (Hz)	Assign
11.49	1	br s	-	NH
7.97	1	d	2.1	2''
7.86	1	br d	8.4	5
7.86	1	br d	8.9	5''
7.72	1	dd	8.5, 2.1	6''
6.84	1	dd	8.9, 2.1	6
6.69	1	d	2.3	8
3.86	3	s	-	OMe

Acquisition Time (sec)	6.5536
Date	21/11/2018 17:44:00
Date Stamp	21/11/2018 17:44:00
Frequency (MHz)	500.1930
Nucleus	1H
Number of Transients	16
Solvent	DMSO-d6

^{1}H NMR (500 MHz, *DMSO-d_6*) δ ppm 11.49 (br s, 1 H), 7.97 (d, *J*=2.1 Hz, 1 H), 7.86 (br d, *J*=8.4 Hz, 1 H), 7.86 (br d, *J*=8.9 Hz, 1 H), 7.72 (dd, *J*=8.5, 2.1 Hz, 1 H), 6.84 (dd, *J*=8.9, 2.1 Hz, 1 H), 6.69 (d, *J*=2.3 Hz, 1 H), 3.86 (s, 3 H)

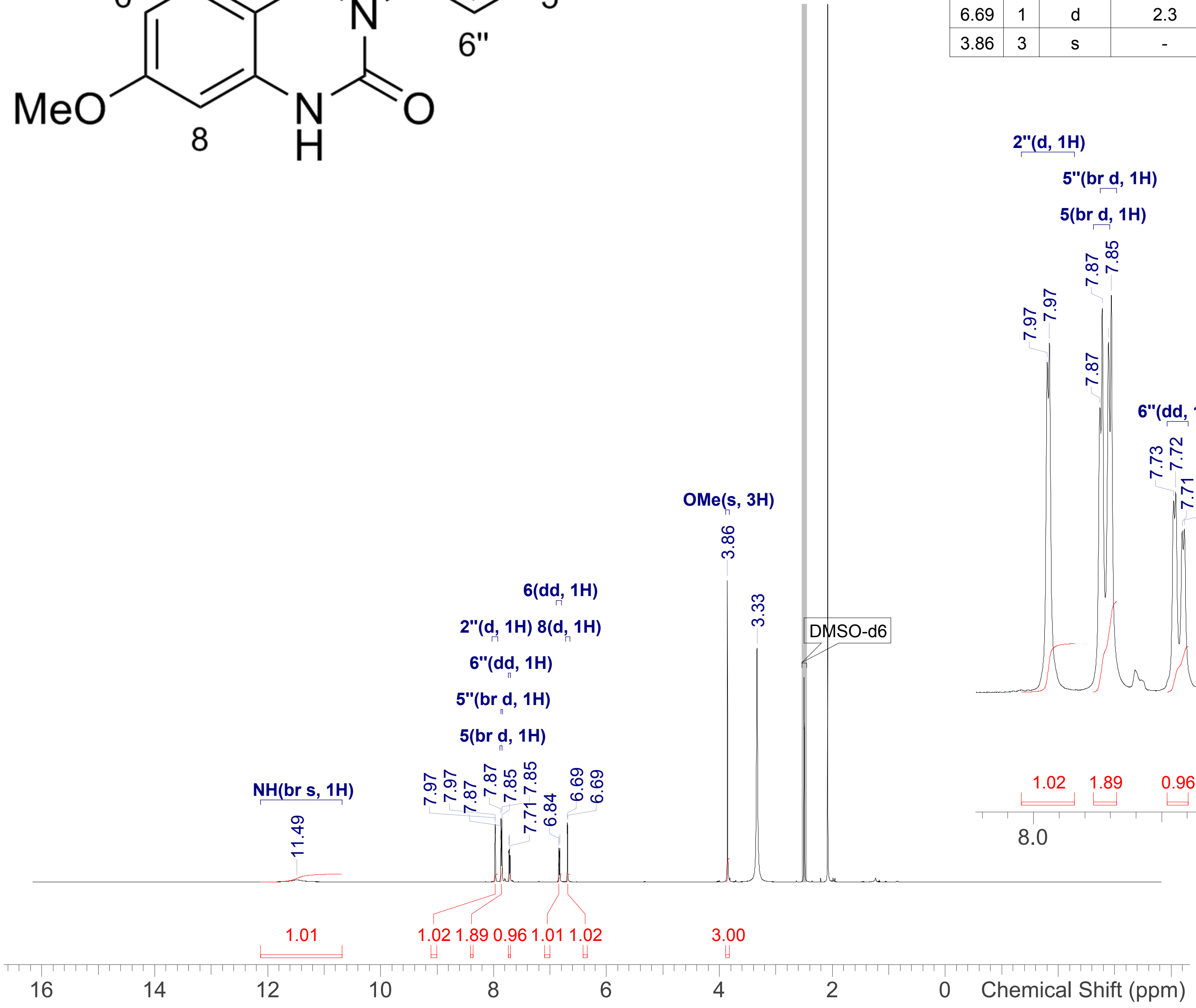

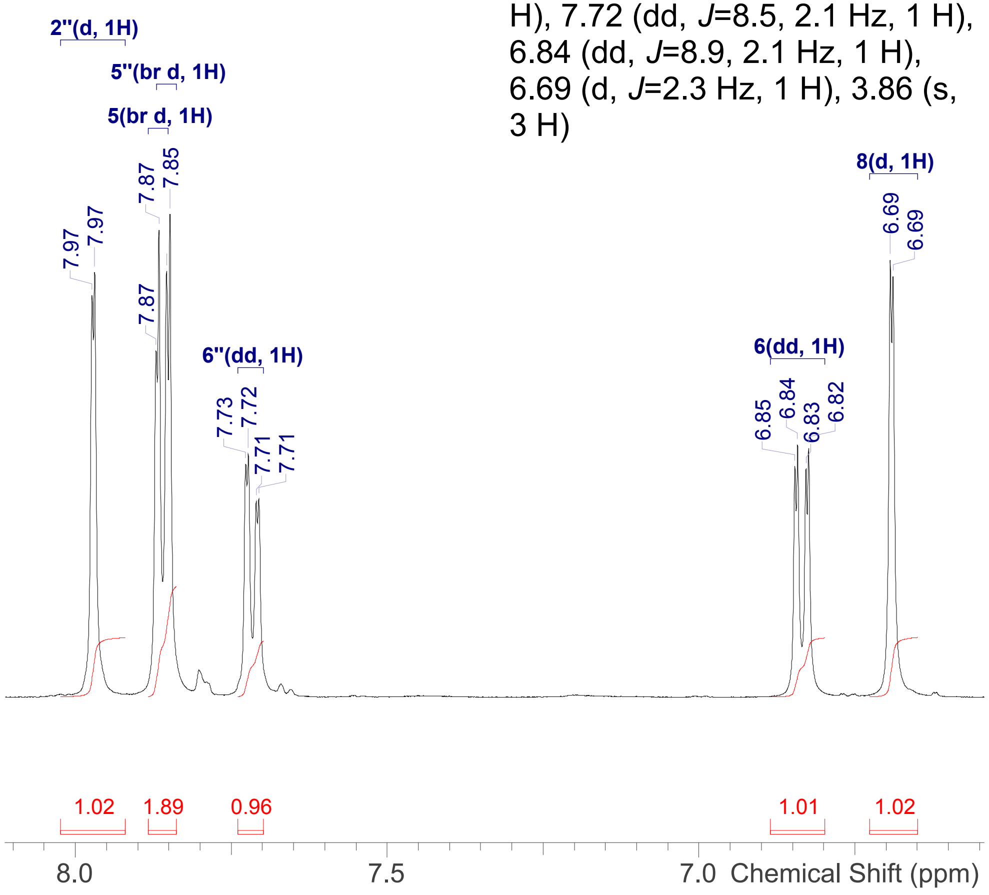

NVR-93_1H.spectrus

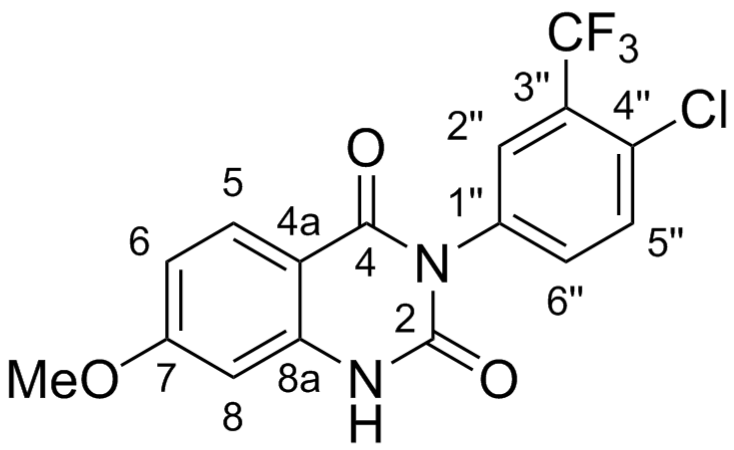

Shift (ppm)	C	m	J (Hz)	Assign
164.7	1	s	-	7
161.6	1	s	-	4
150.2	1	s	-	2
141.8	1	s	-	8a
135.3	1	s	-	6"
132.1	1	s	-	5"
130.3	1	s	-	1"
129.5	1	s	-	5
129.2	1	br q	3.9	2"
115.0	1	s	-	4"
110.8	1	s	-	6
107.6	1	s	-	4a
98.3	1	s	-	8
55.7	1	s	-	OMe

NVR-93_13C

Acquisition Time (sec)	2.0447
Date	21/11/2018 17:46:00
Date Stamp	21/11/2018 17:46:00
Frequency (MHz)	125.7870
Nucleus	13C
Number of Transients	32
Solvent	DMSO-d6

^{13}C NMR (126 MHz, *DMSO-d_6*) δ ppm 164.7 (s, 1 C), 161.6 (s, 1 C), 150.2 (s, 1 C), 141.8 (s, 1 C), 135.3 (s, 1 C), 132.1 (s, 1 C), 130.3 (s, 1 C), 129.5 (s, 1 C), 129.2 (br q, *J*=3.9 Hz, 1 C), 115.0 (s, 1 C), 110.8 (s, 1 C), 107.6 (s, 1 C), 98.3 (s, 1 C), 55.7 (s, 1 C)

NVR-93_13C

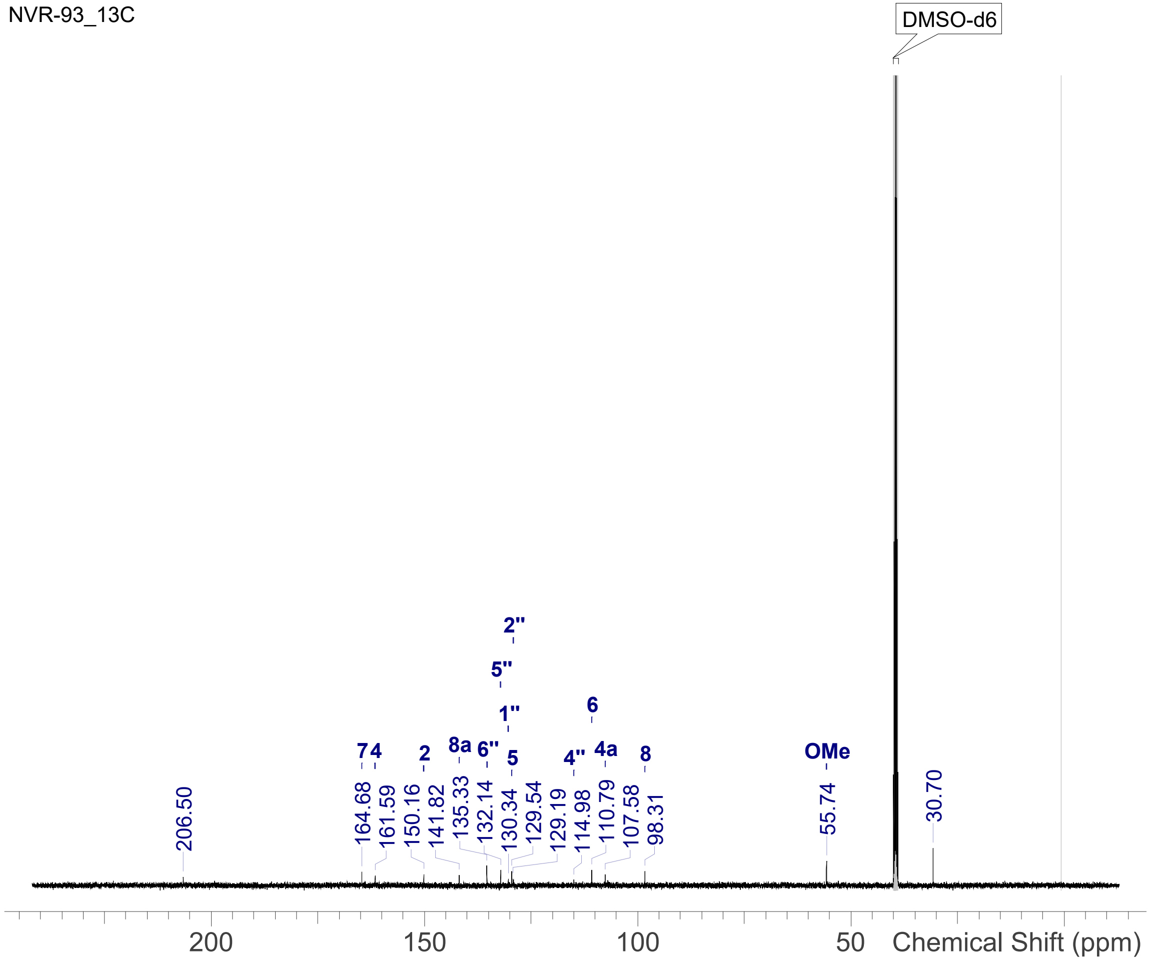

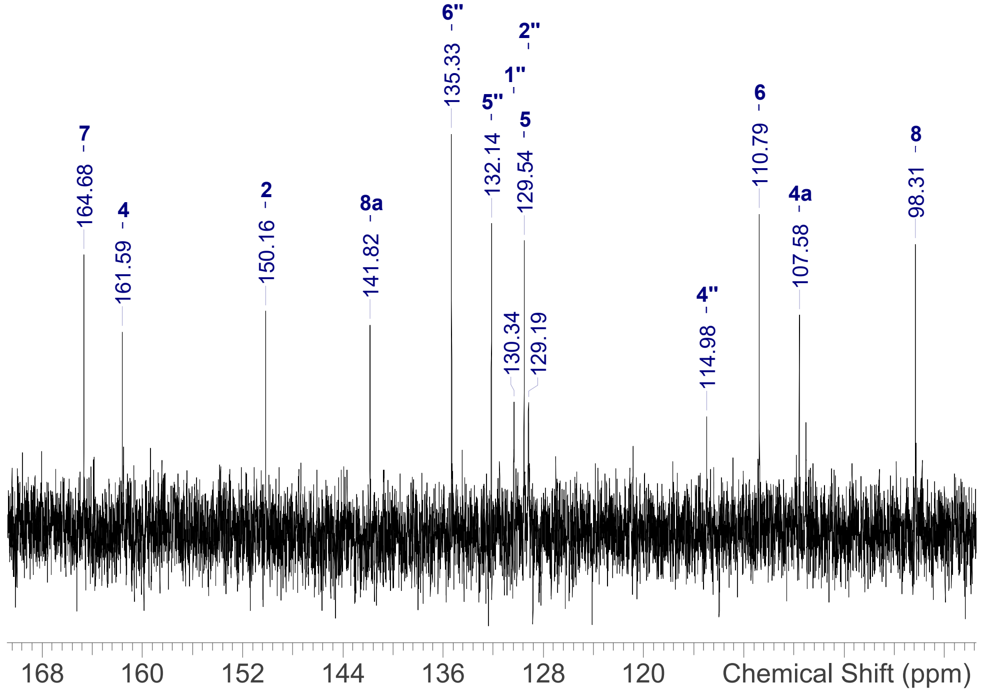

NVR-93_13C.spectrus

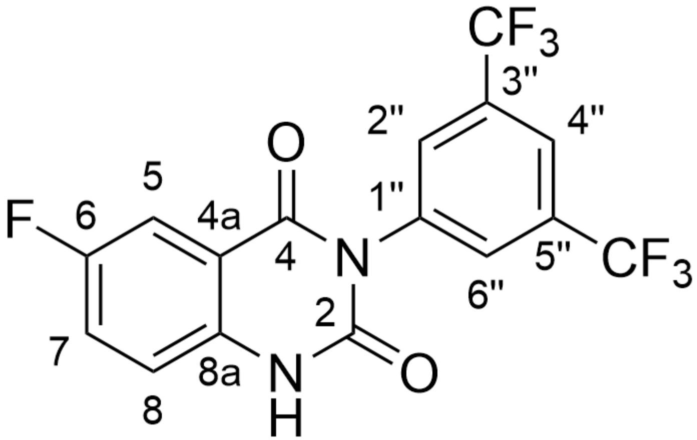

Shift (ppm)	H	m	J (Hz)	Assign
11.00	1	s	-	NH
8.47	2	s	-	6'', 2''
7.78	1	s	-	4''
7.75	1	m	-	5
7.69	1	dd	8.7, 3.2	8
7.46	1	dd	8.8, 4.8	7

Acquisition Time (sec)	5.3084
Date	08 Apr 2019 10:04:40
Date Stamp	08 Apr 2019 10:04:40
Frequency (MHz)	300.1300
Nucleus	1H
Number of Transients	16
Solvent	DMSO-d6
Temperature (degree C)	22.560

^{1}H NMR (300 MHz, *DMSO-d_6*) δ ppm 11.00 (s, 1 H), 8.47 (s, 2 H), 7.78 (s, 1 H), 7.73 - 7.77 (m, 1 H), 7.69 (dd, *J*=8.7, 3.2 Hz, 1 H), 7.46 (dd, *J*=8.8, 4.8 Hz, 1 H)

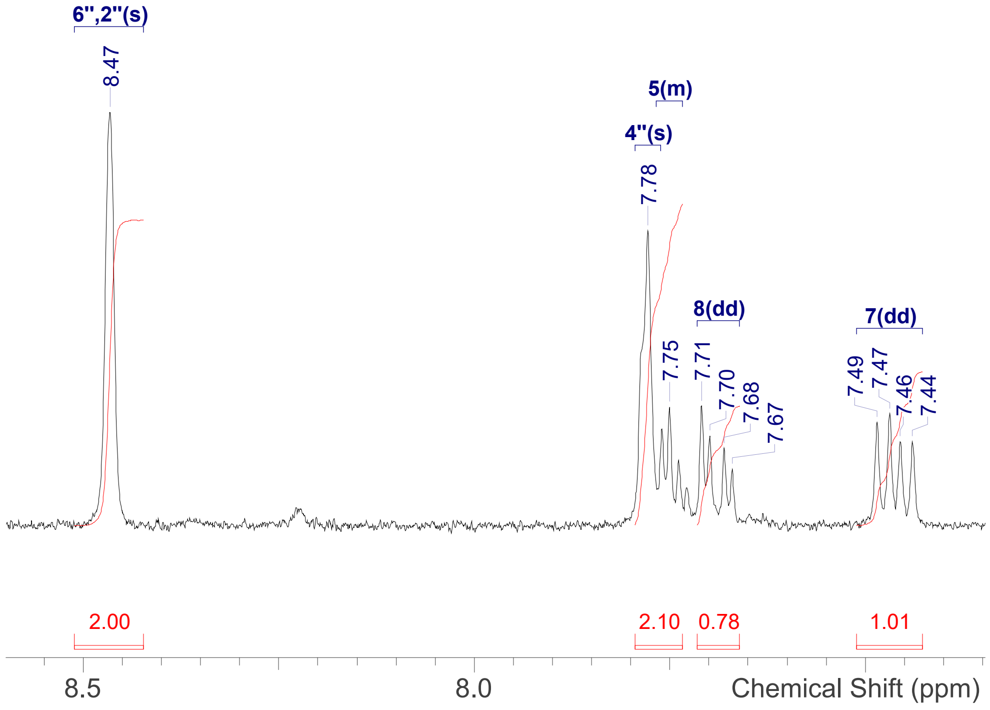

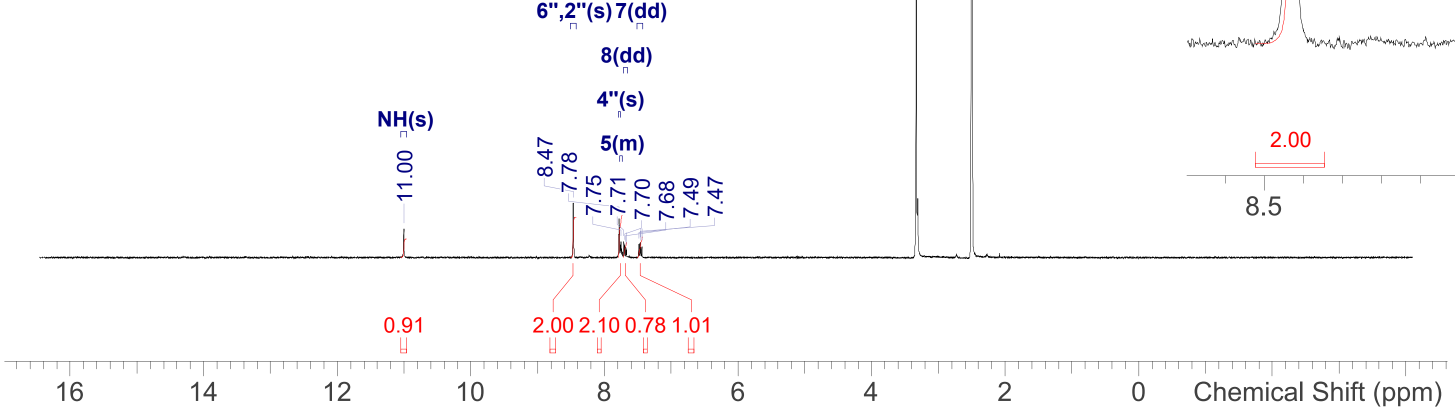

NVR-125_1H.spectrus

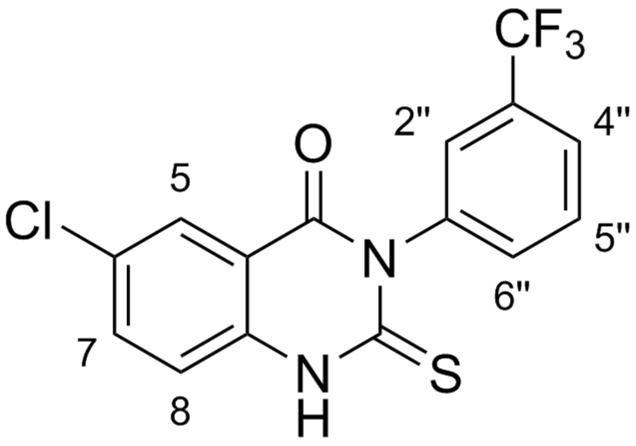

Shift (ppm)	H	m	J (Hz)	Assign
10.75	1	br s	-	NH
8.14	1	br s	-	5
7.78	1	br d	7.2	7
7.68	2	m	-	5", 2"
7.56	1	br s	-	8
7.48	1	br d	7.2	6"
7.14	1	br d	8.9	4"

Acquisition Time (sec)	10.6168
Date	06/08/2019 15:56:00
Date Stamp	06/08/2019 15:56:00
Frequency (MHz)	300.1320
Nucleus	1H
Number of Transients	16
Solvent	CHLOROFORM-d

^{1}H NMR (300 MHz, *CHLOROFORM-d*) δ ppm 10.75 (br s, 1 H), 8.14 (br s, 1 H), 7.78 (br d, *J*=7.2 Hz, 1 H), 7.60 - 7.74 (m, 2 H), 7.56 (br s, 1 H), 7.48 (br d, *J*=7.2 Hz, 1 H), 7.14 (br d, *J*=8.9 Hz, 1 H)

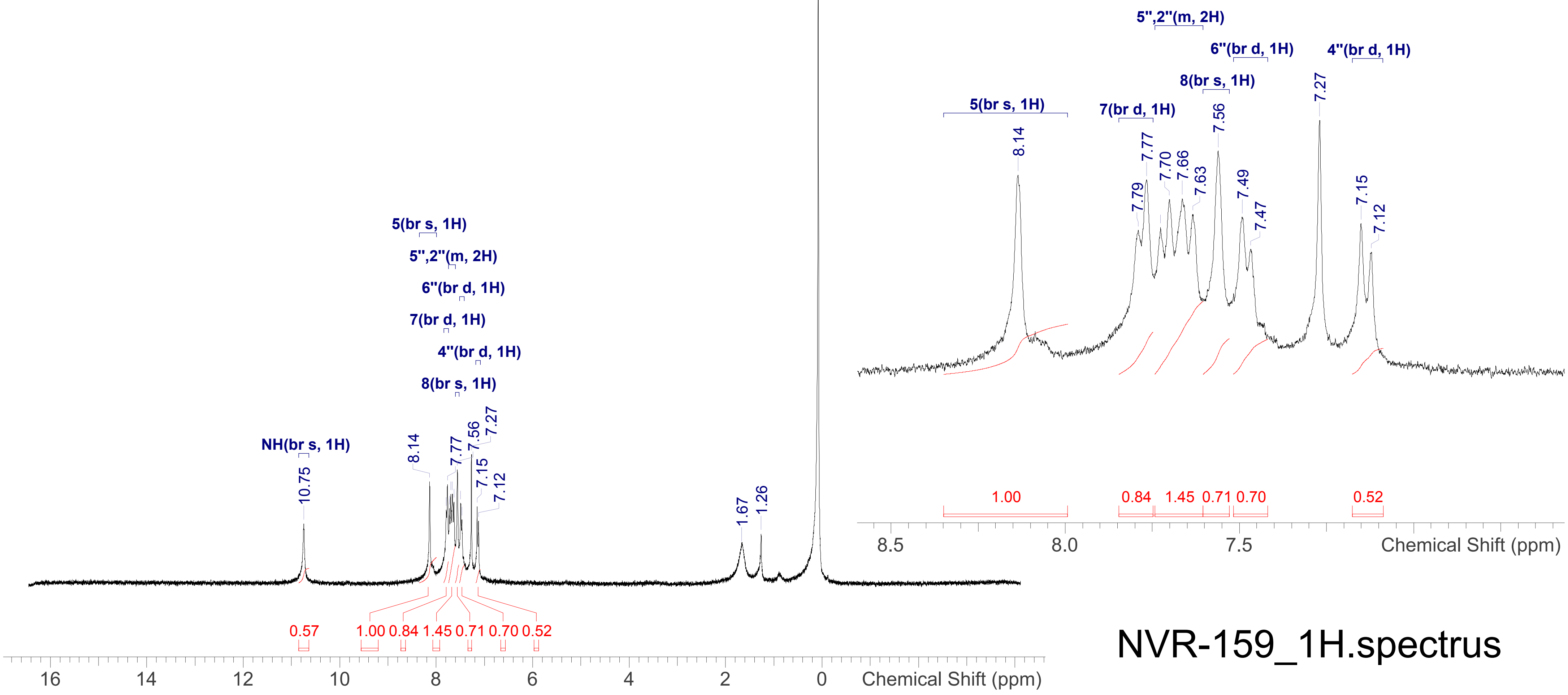

NVR-159_1H.spectrus

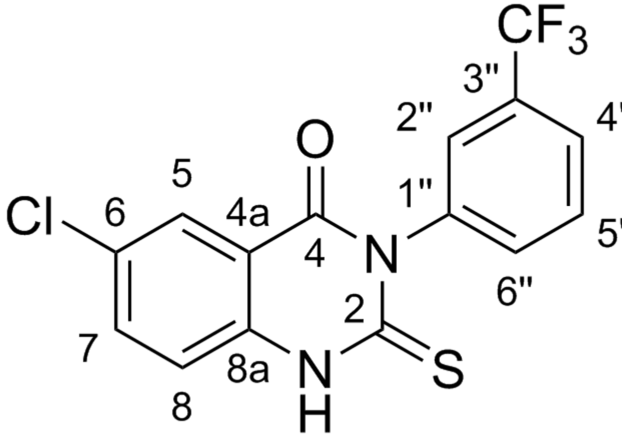

Shift (ppm)	C	m	J (Hz)	Assign
176.2	1	s	-	2
158.8	1	s	-	4
138.6	1	s	-	8a
137.2	1	s	-	1''
136.3	1	s	-	5''
132.2	1	br q	33.3	3''
132.1	1	s	-	6''
131.0	1	s	-	6
130.2	1	s	-	5
128.3	1	s	-	7
126.1	1	br q	2.9	2''
125.9	1	br q	2.9	4''
123.5	1	q	272.9	CF3
117.4	1	s	-	4a
116.4	1	s	-	8

Acquisition Time (sec)	1.0224
Date	16/08/2019 00:18:00
Date Stamp	16/08/2019 00:18:00
Frequency (MHz)	125.7730
Nucleus	13C
Number of Transients	2048
Solvent	CHLOROFORM-d

^{13}C NMR (126 MHz, *CHLOROFORM-d*) δ ppm 176.2 (s, 1 C), 158.8 (s, 1 C), 138.6 (s, 1 C), 137.2 (s, 1 C), 136.3 (s, 1 C), 132.1 (s, 1 C), 132.2 (br q, *J*=33.3 Hz, 1 C), 131.0 (s, 1 C), 130.2 (s, 1 C), 128.3 (s, 1 C), 126.1 (br q, *J*=2.9 Hz, 1 C), 125.9 (br q, *J*=2.9 Hz, 1 C), 123.5 (q, *J*=272.9 Hz, 1 C), 117.4 (s, 1 C), 116.4 (s, 1 C)

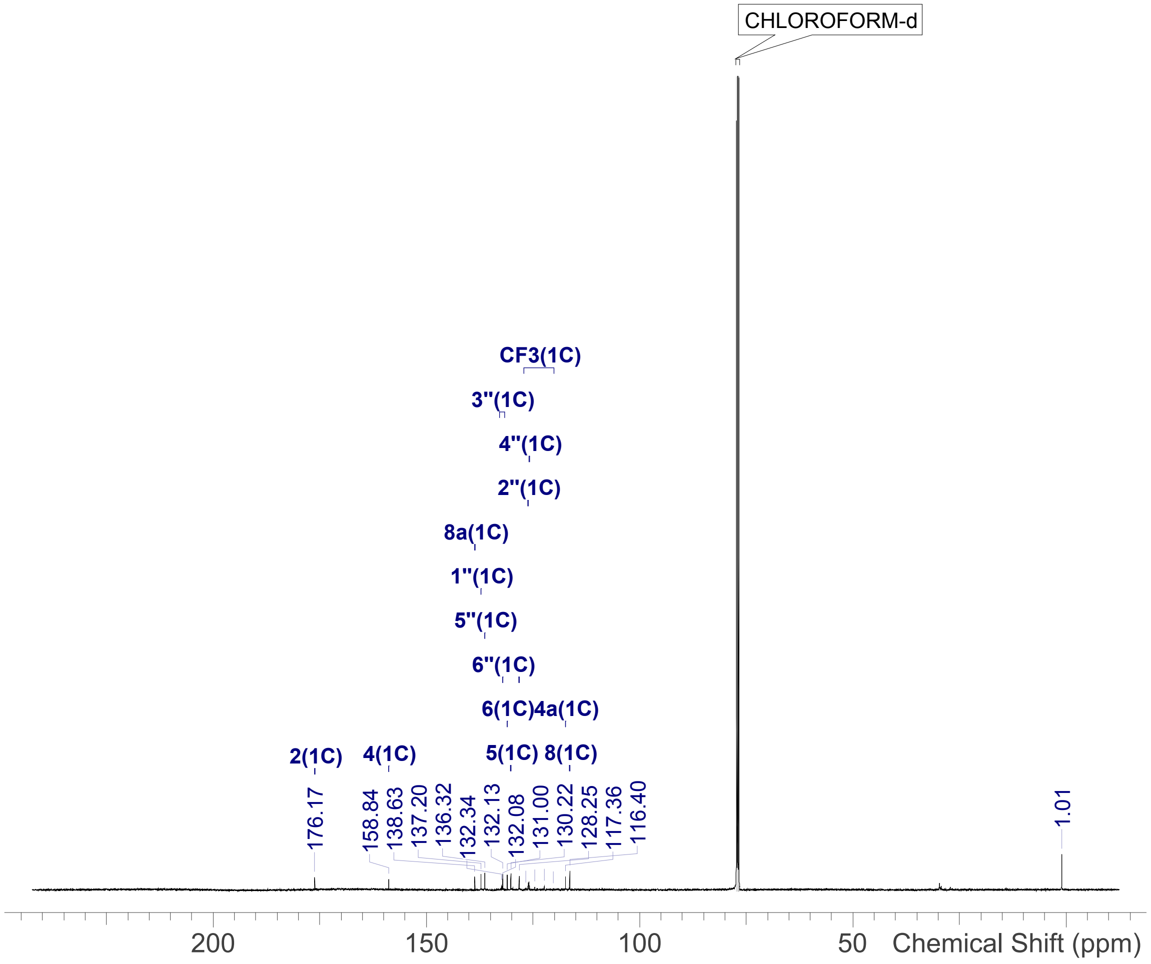

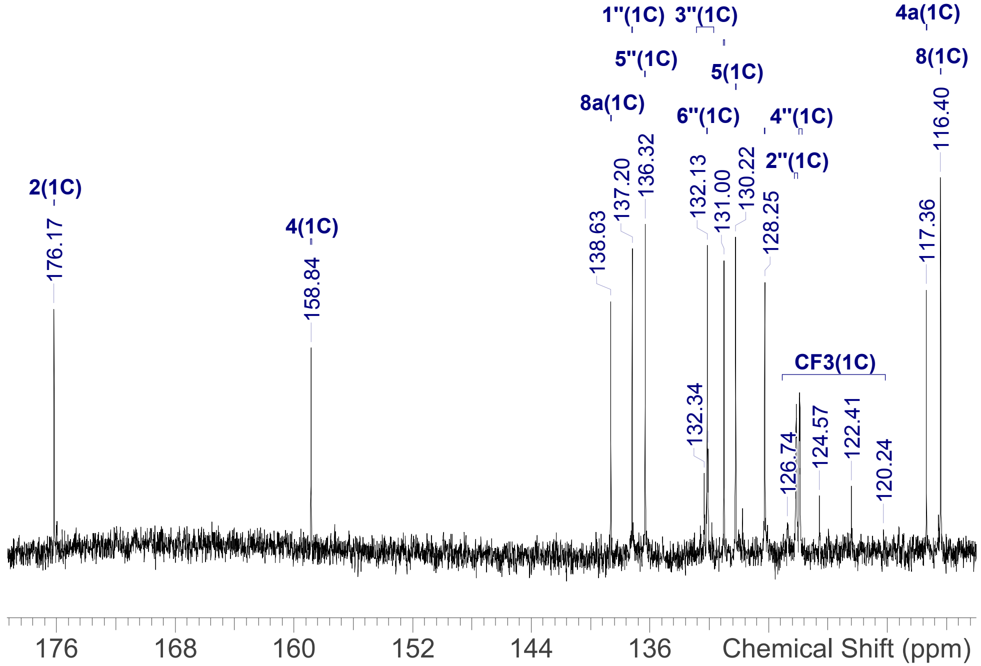

NVR-159_13C.spectrus

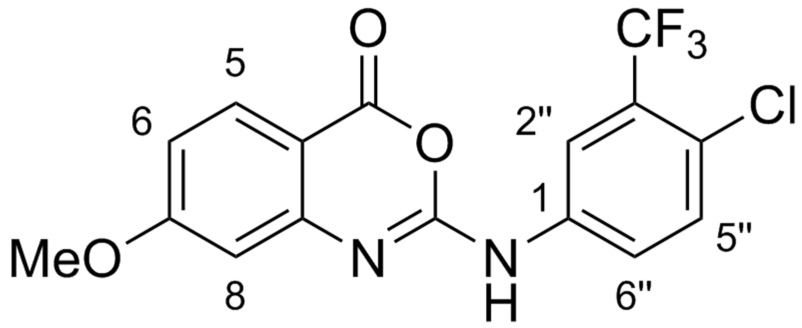

Shift (ppm)	H	m	J (Hz)	Assign
8.26	1	d	2.4	2''
8.10	1	dd	8.8, 2.4	6''
7.88	1	d	8.9	5
7.69	1	d	8.9	5''
6.89	1	dd	8.9, 2.4	6
6.79	1	d	2.4	8
3.89	3	s	-	OMe

Acquisition Time (sec)	6.5536
Date	23/10/2018 14:59:00
Date Stamp	23/10/2018 14:59:00
Frequency (MHz)	500.1930
Nucleus	1H
Number of Transients	16
Solvent	DMSO-d6

^{1}H NMR (500 MHz, *DMSO-d_6*) δ ppm 8.26 (d, *J*=2.4 Hz, 1 H), 8.10 (dd, *J*=8.8, 2.4 Hz, 1 H), 7.88 (d, *J*=8.9 Hz, 1 H), 7.69 (d, *J*=8.9 Hz, 1 H), 6.89 (dd, *J*=8.9, 2.4 Hz, 1 H), 6.79 (d, *J*=2.4 Hz, 1 H), 3.89 (s, 3 H)

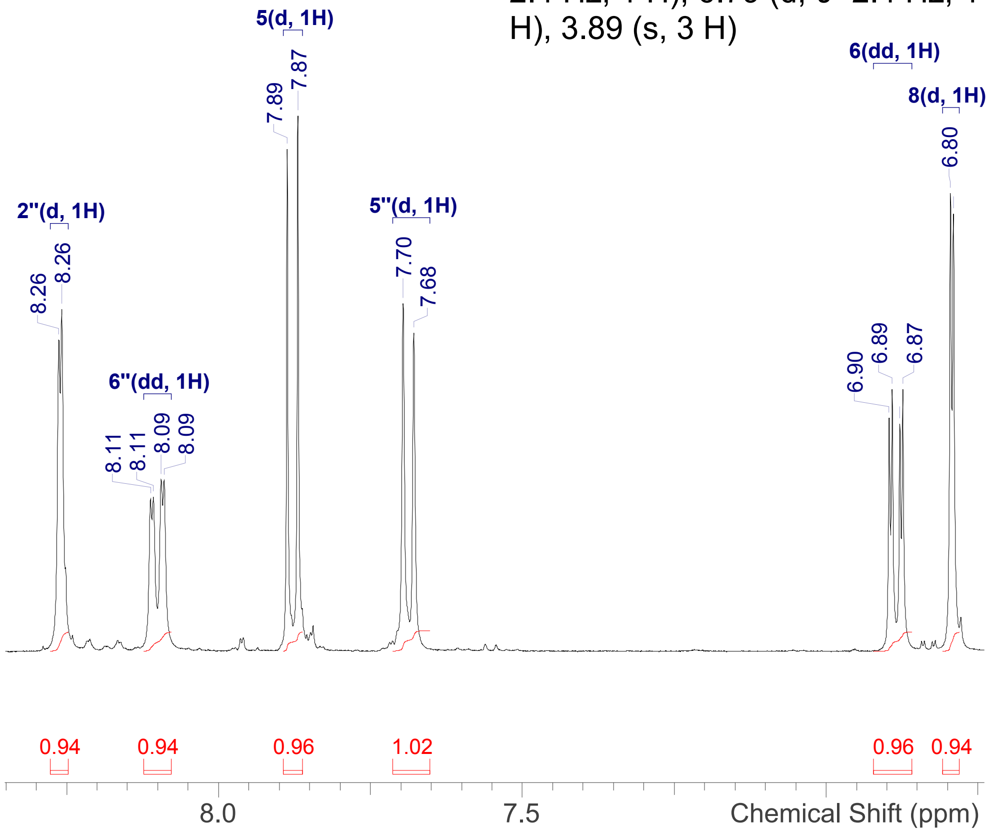

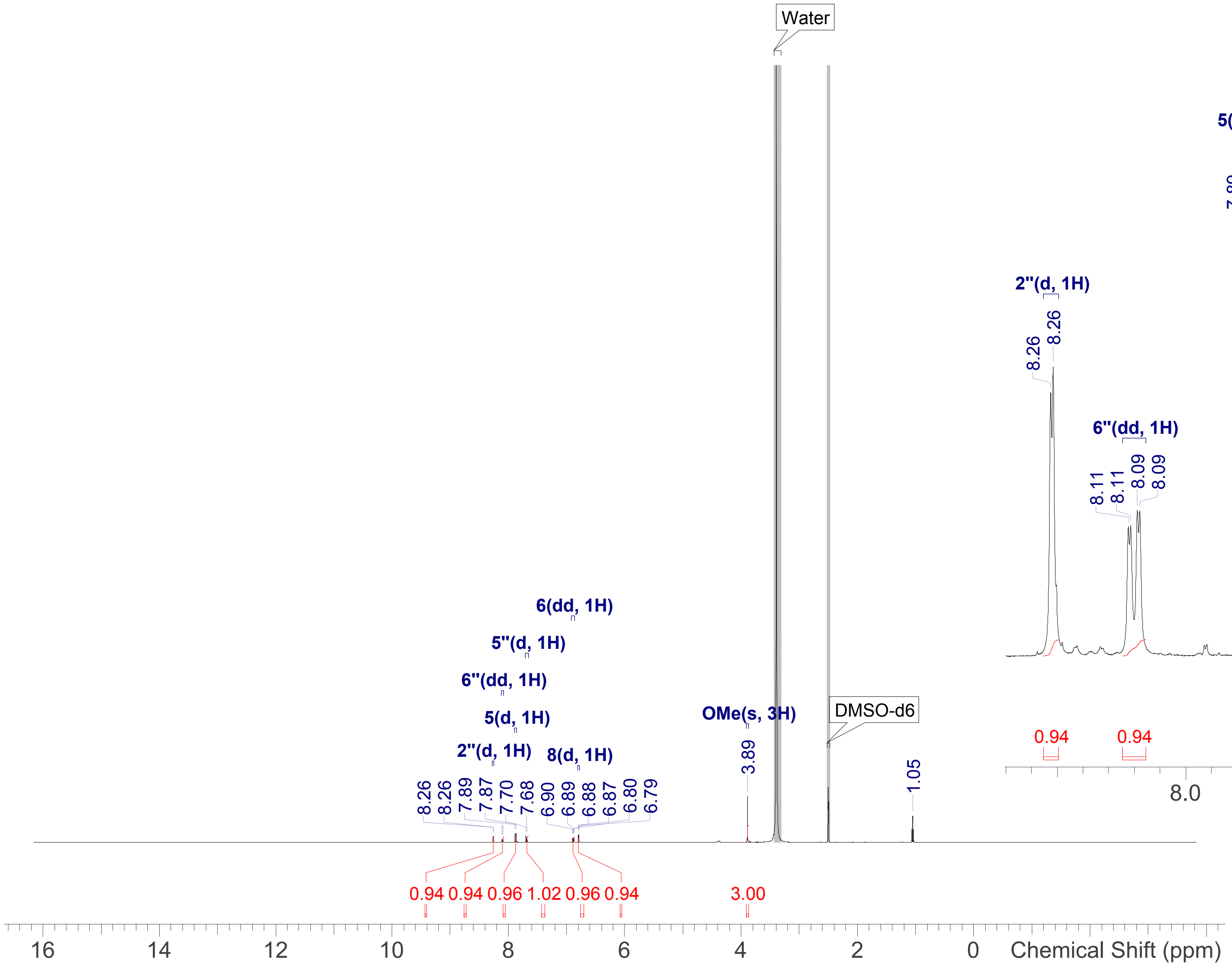

NVR-65_1H.spectrus

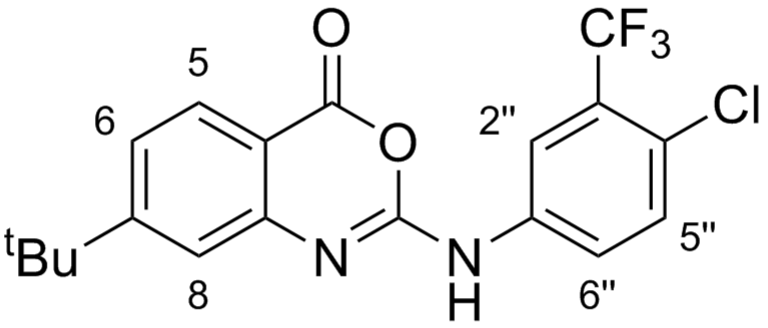

Shift (ppm)	H	m	J (Hz)	Assign
10.68	1	br s	-	NH
8.30	1	d	2.5	2"
8.16	1	dd	8.9, 2.4	6"
7.93	1	d	8.4	5
7.72	1	d	8.8	5"
7.44	1	dd	8.4, 1.9	6
7.32	1	d	1.7	8
1.33	9	s	-	Me, Me, Me

Acquisition Time (sec)	5.2429
Date	06/06/2019 21:11:00
Date Stamp	06/06/2019 21:11:00
Frequency (MHz)	400.0680
Nucleus	1H
Number of Transients	16
Solvent	DMSO-d6

^{1}H NMR (400 MHz, *DMSO-d*$_6$) δ ppm 10.68 (br s, 1 H), 8.30 (d, *J*=2.5 Hz, 1 H), 8.16 (dd, *J*=8.9, 2.4 Hz, 1 H), 7.93 (d, *J*=8.4 Hz, 1 H), 7.72 (d, *J*=8.8 Hz, 1 H), 7.44 (dd, *J*=8.4, 1.9 Hz, 1 H), 7.32 (d, *J*=1.7 Hz, 1 H), 1.33 (s, 9 H)

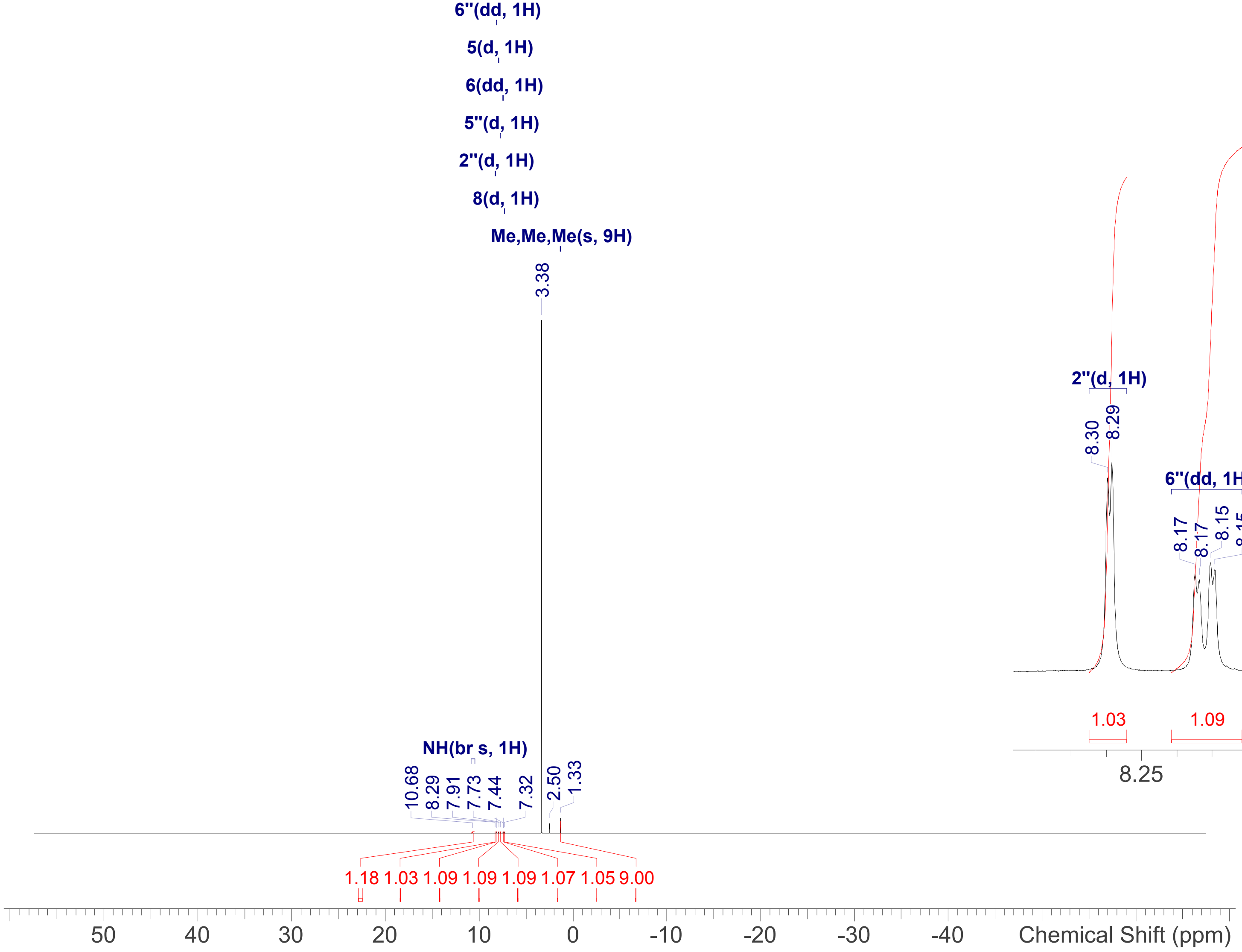

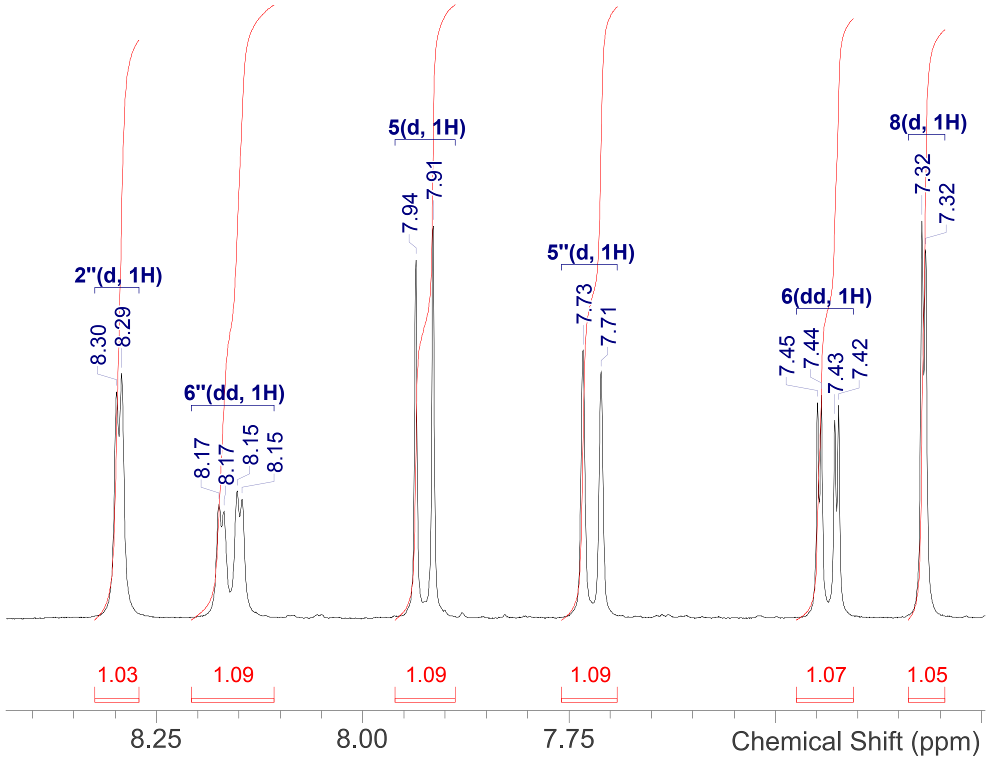

NVR-68_1H.spectrus

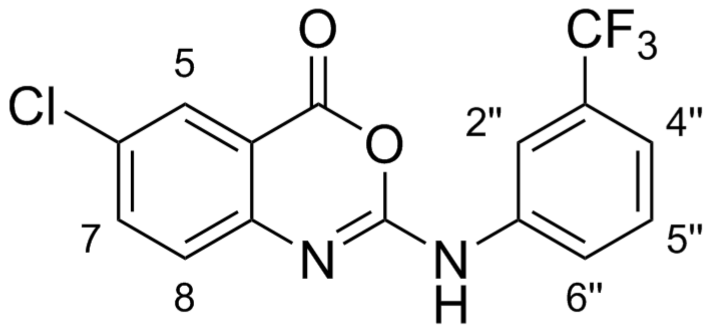

Shift (ppm)	H	m	J (Hz)	Assign
10.74	1	s	-	NH
8.24	1	s	-	2''
8.01	1	br d	8.1	4''
7.93	1	d	2.5	5
7.80	1	dd	8.7, 2.5	7
7.61	1	t	8.0	5''
7.43	1	d	7.8	6''
7.40	1	d	8.8	8

Acquisition Time (sec)	10.6168
Date	16/08/2019 20:46:00
Date Stamp	16/08/2019 20:46:00
Frequency (MHz)	300.1320
Nucleus	1H
Number of Transients	16
Solvent	DMSO-d6

^{1}H NMR (300 MHz, *DMSO-d$_6$*) δ ppm 10.74 (s, 1 H), 8.24 (s, 1 H), 8.01 (br d, *J*=8.1 Hz, 1 H), 7.93 (d, *J*=2.5 Hz, 1 H), 7.80 (dd, *J*=8.7, 2.5 Hz, 1 H), 7.61 (t, *J*=8.0 Hz, 1 H), 7.43 (d, *J*=7.8 Hz, 1 H), 7.40 (d, *J*=8.8 Hz, 1 H)

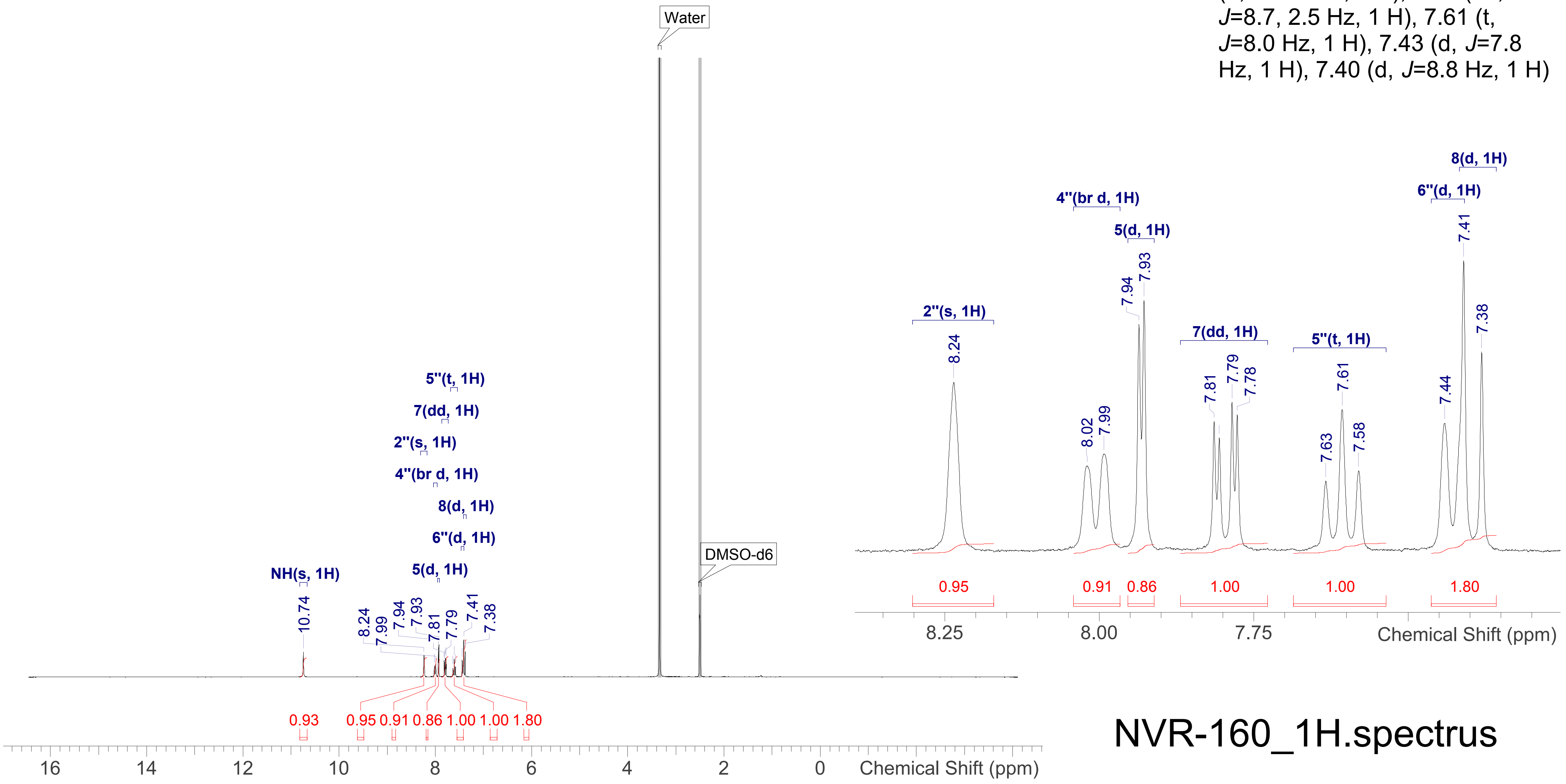

NVR-160_1H.spectrus

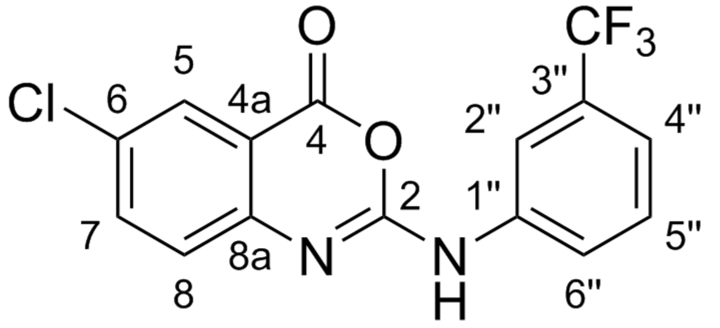

Shift (ppm)	C	m	J (Hz)	Assign
158.0	1	s	-	4
150.6	1	s	-	2
147.7	1	s	-	8a
138.8	1	s	-	1''
136.6	1	s	-	7
130.0	1	s	-	5
129.6	1	q	31.5	3''
128.2	1	s	-	6
126.9	1	q	2.0	5''
126.8	1	s	-	8
124.1	1	q	272.1	CF3
122.9	1	s	-	6''
119.4	1	br q	3.9	2''
115.7	1	s	-	4a
115.4	1	br q	3.9	4''

Acquisition Time (sec)	2.0447
Date	15/08/2019 22:00:00
Date Stamp	15/08/2019 22:00:00
Frequency (MHz)	125.7870
Nucleus	13C
Number of Transients	2048
Solvent	DMSO-d6

^{13}C NMR (126 MHz, *DMSO-d_6*) δ ppm 158.0 (s, 1 C), 150.6 (s, 1 C), 147.7 (s, 1 C), 138.8 (s, 1 C), 136.6 (s, 1 C), 130.0 (s, 1 C), 129.6 (q, *J*=31.5 Hz, 1 C), 128.2 (s, 1 C), 126.9 (q, *J*=2.0 Hz, 1 C), 126.8 (s, 1 C), 122.9 (s, 1 C), 124.1 (q, *J*=272.1 Hz, 1 C), 119.4 (br q, *J*=3.9 Hz, 1 C), 115.7 (s, 1 C), 115.4 (br q, *J*=3.9 Hz, 1 C)

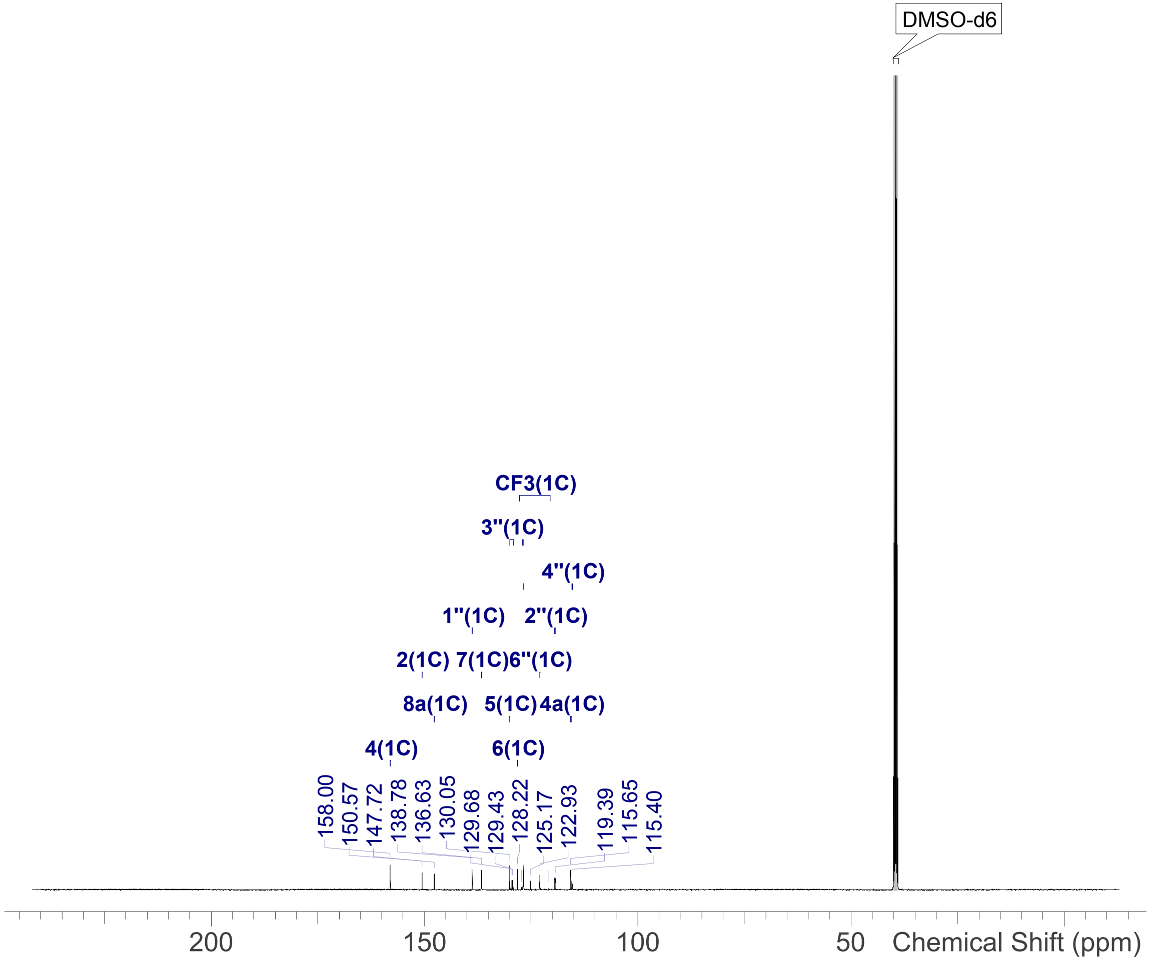

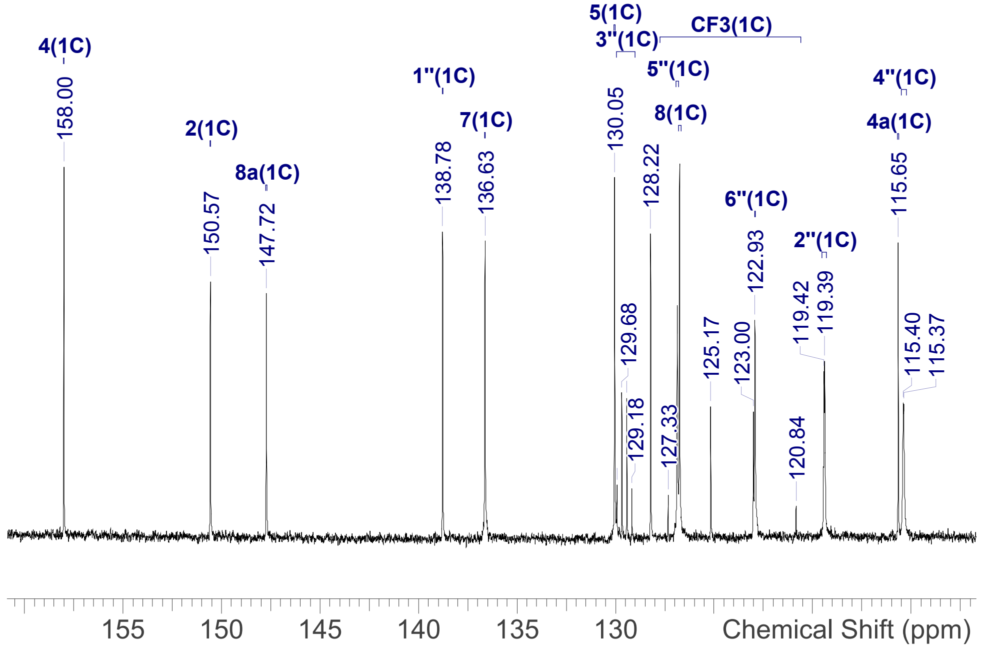

NVR-160_13C.spectrus

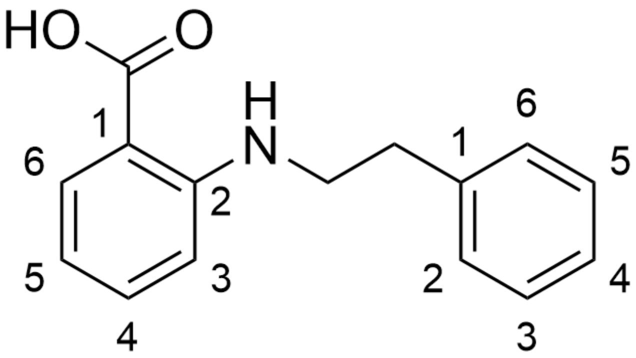

Shift (ppm)	H	m	J (Hz)	Assign
7.95	1	dd	8.1, 1.5	6
7.27	6	m	-	4", 3", 5", 2", 6", 4
6.69	1	d	8.5	3
6.59	1	br t	7.5	5
3.45	2	t	7.2	CH2NH
2.96	2	t	7.2	CH2Ph

Acquisition Time (sec)	10.6168
Date	11/04/2016 13:45:00
Date Stamp	11/04/2016 13:45:00
Frequency (MHz)	300.1310
Nucleus	1H
Number of Transients	16
Solvent	CHLOROFORM-d

^{1}H NMR (300 MHz, *CHLOROFORM-d*) δ ppm 7.95 (dd, *J*=8.1, 1.5 Hz, 1 H), 7.16 - 7.40 (m, 6 H), 6.69 (d, *J*=8.5 Hz, 1 H), 6.59 (br t, *J*=7.5 Hz, 1 H), 3.45 (t, *J*=7.2 Hz, 2 H), 2.96 (t, *J*=7.2 Hz, 2 H)

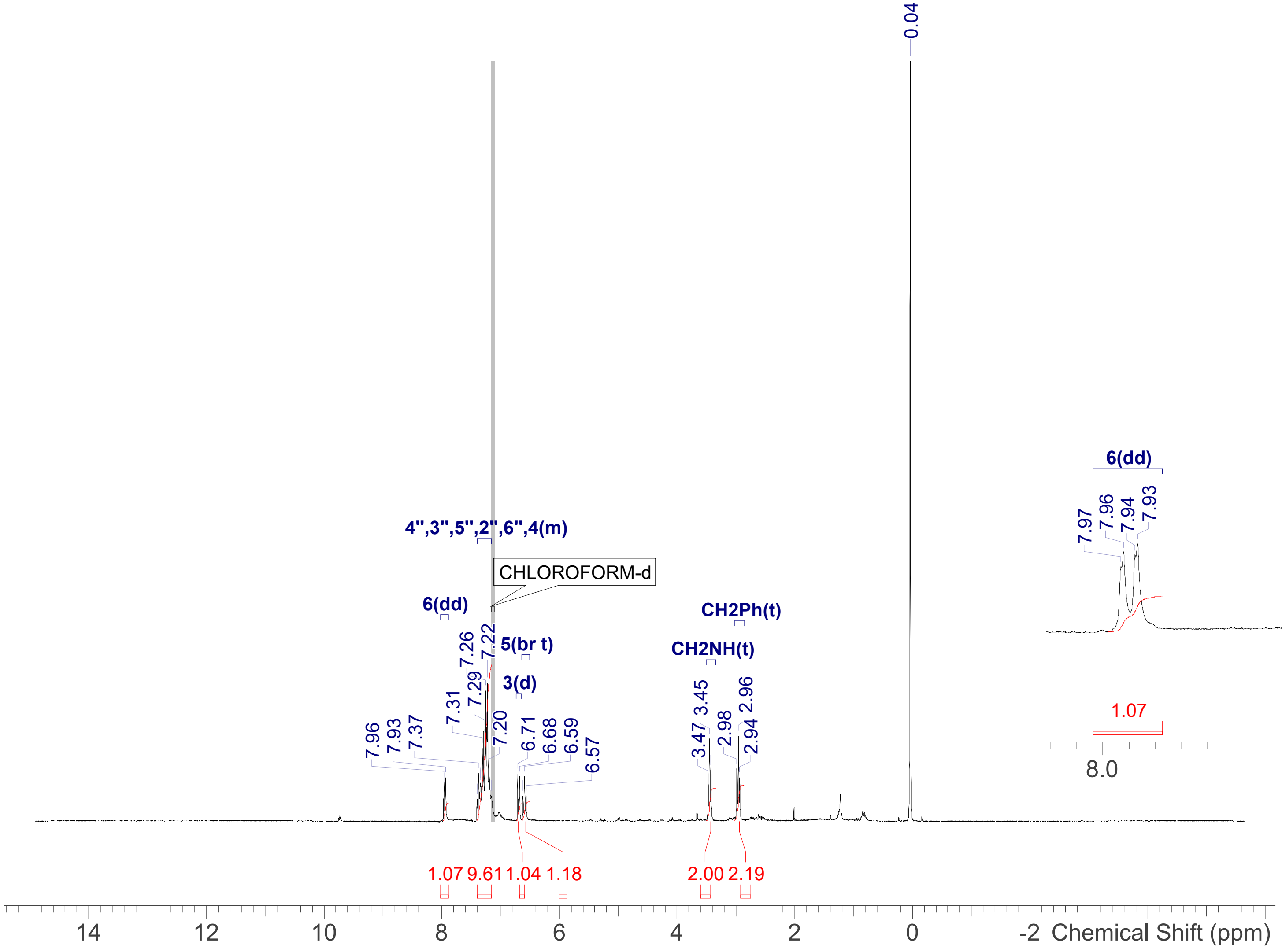

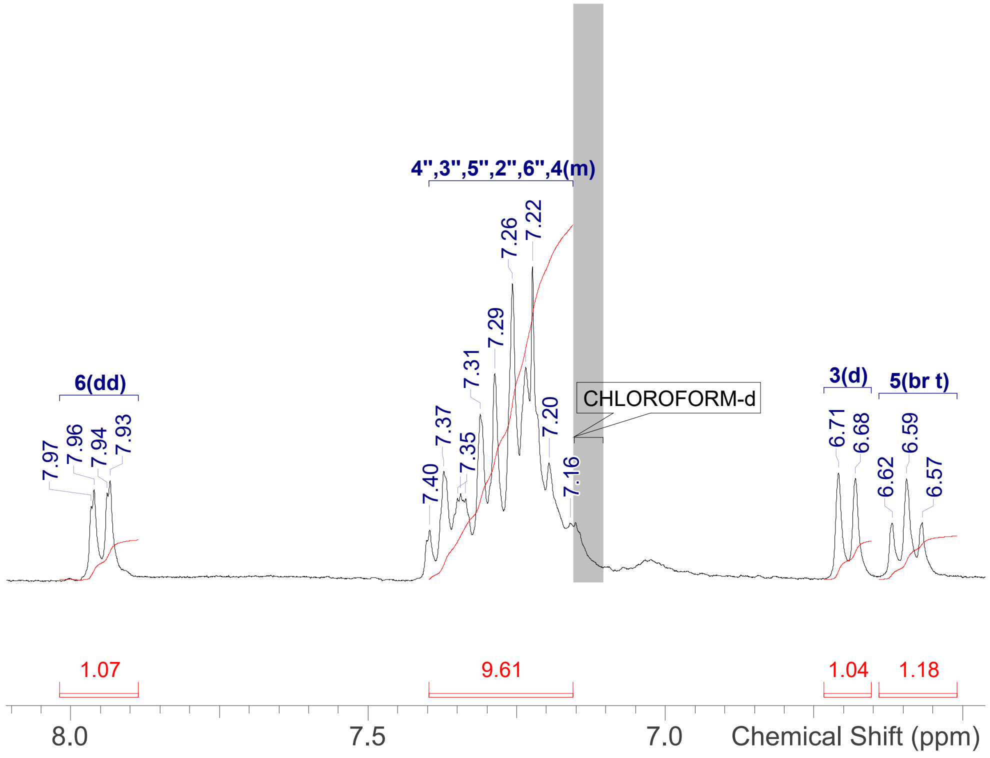

Tromaril_1H.spectrus

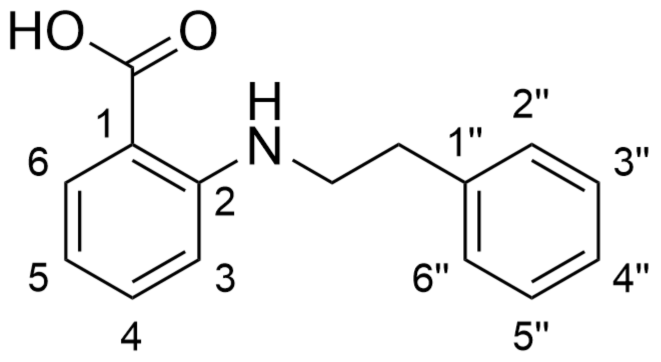

Shift (ppm)	C	m	Assign
173.0	1	s	COOH
151.5	1	s	2
139.1	1	s	1''
135.6	1	s	4
132.6	1	s	6
128.8	2	s	2'', 6''
128.6	2	s	3'', 5''
126.5	1	s	4''
114.7	1	s	5
111.3	1	s	3
108.6	1	s	1
44.4	1	s	CH2NH
35.5	1	s	CH2Ph

Acquisition Time (sec)	3.6438
Date	27/04/2016 21:10:00
Date Stamp	27/04/2016 21:10:00
Frequency (MHz)	75.4753
Nucleus	13C
Number of Transients	1200
Solvent	CHLOROFORM-d

^{13}C NMR (75 MHz, *CHLOROFORM-d*) δ ppm 173.0 (s, 1 C), 151.5 (s, 1 C), 139.1 (s, 1 C), 135.6 (s, 1 C), 132.6 (s, 1 C), 128.8 (s, 2 C), 128.6 (s, 2 C), 126.5 (s, 1 C), 114.7 (s, 1 C), 111.3 (s, 1 C), 108.6 (s, 1 C), 44.4 (s, 1 C), 35.5 (s, 1 C)

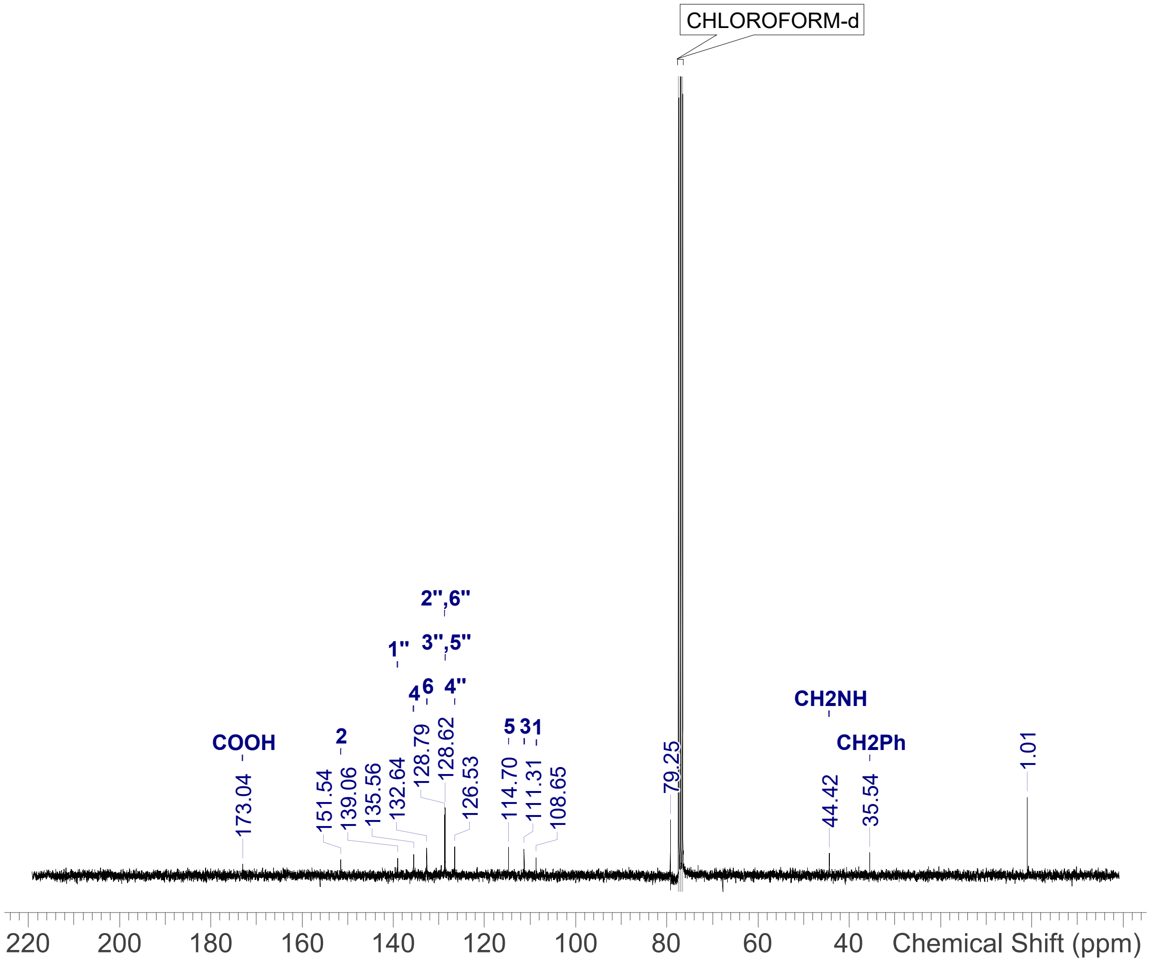

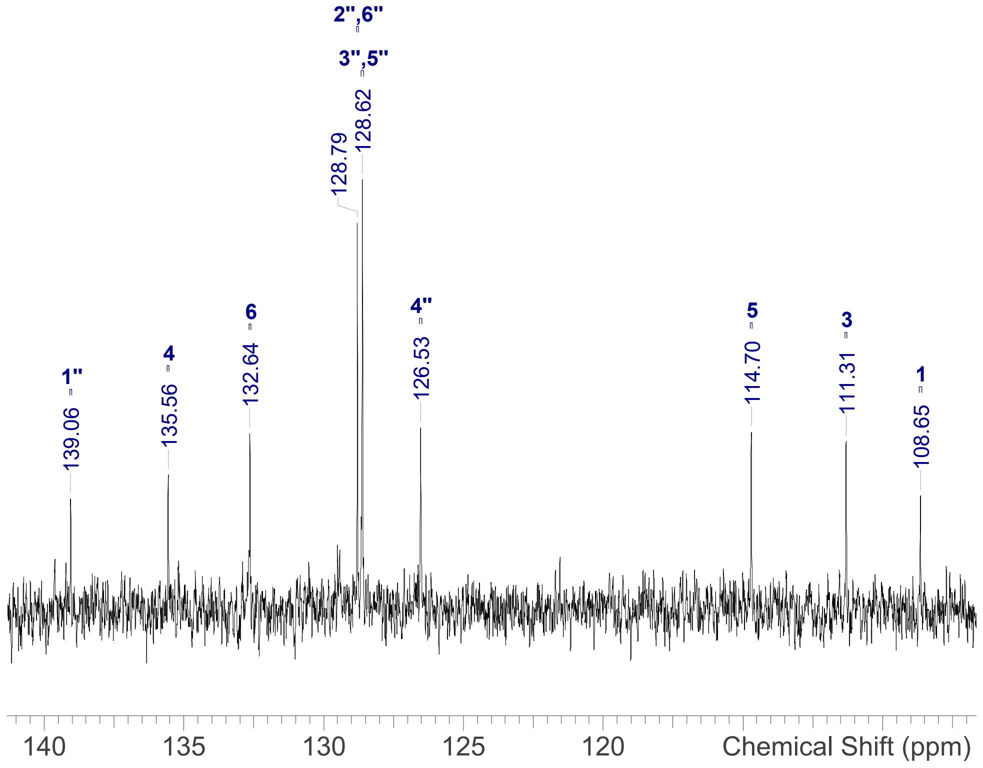

Tromaril_13C.spectrus

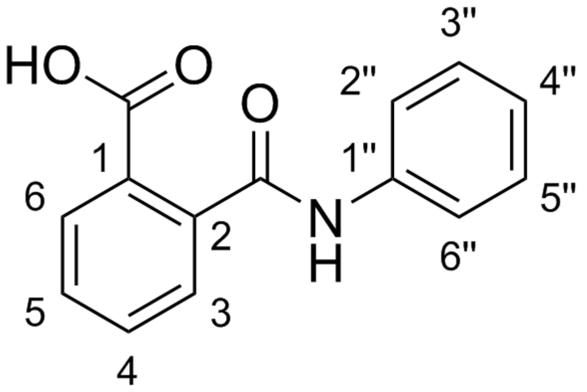

Shift (ppm)	H	m	J (Hz)	Assign
13.00	1	br s	-	COOH
10.32	1	s	-	CONH
7.88	1	dd	7.8, 0.8	6
7.69	2	br d	7.5	6'', 2''
7.65	1	dd	7.4, 1.1	3
7.57	1	td	7.5, 1.0	5
7.55	1	t	7.3	4
7.33	2	t	7.9	5'', 3''
7.07	1	t	7.3	4''

Acquisition Time (sec)	3.9846
Date	01 May 2018 15:53:15
Date Stamp	01 May 2018 15:53:15
Frequency (MHz)	400.0700
Nucleus	1H
Number of Transients	16
Solvent	DMSO-d6
Temperature (degree C)	25.003

^{1}H NMR (400 MHz, *DMSO-d_6*) δ ppm 13.00 (br s, 1 H), 10.32 (s, 1 H), 7.88 (dd, *J*=7.8, 0.8 Hz, 1 H), 7.69 (br d, *J*=7.5 Hz, 2 H), 7.65 (dd, *J*=7.4, 1.1 Hz, 1 H), 7.57 (td, *J*=7.5, 1.0 Hz, 1 H), 7.55 (t, *J*=7.3 Hz, 1 H), 7.33 (t, *J*=7.9 Hz, 2 H), 7.07 (t, *J*=7.3 Hz, 1 H)

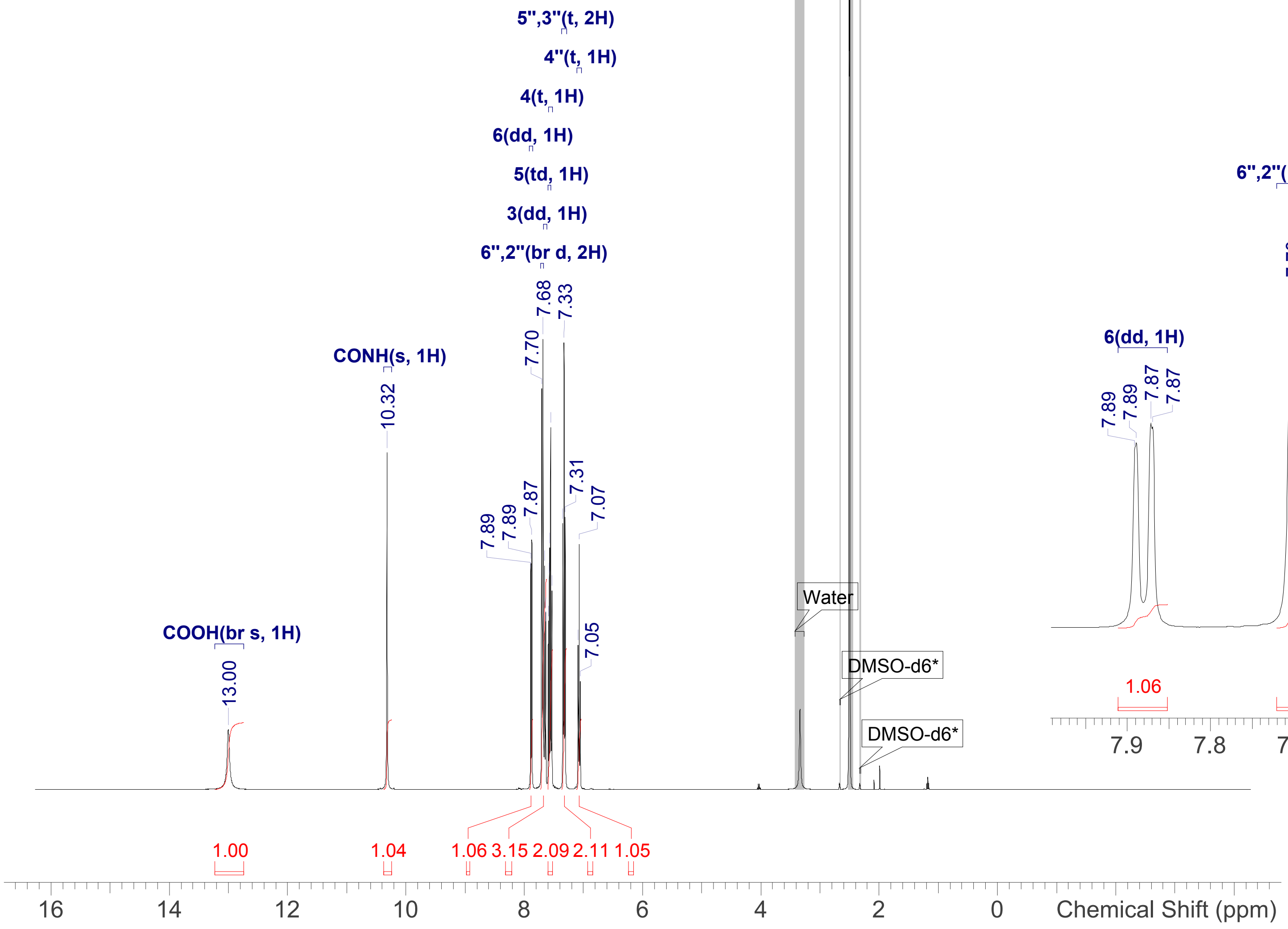

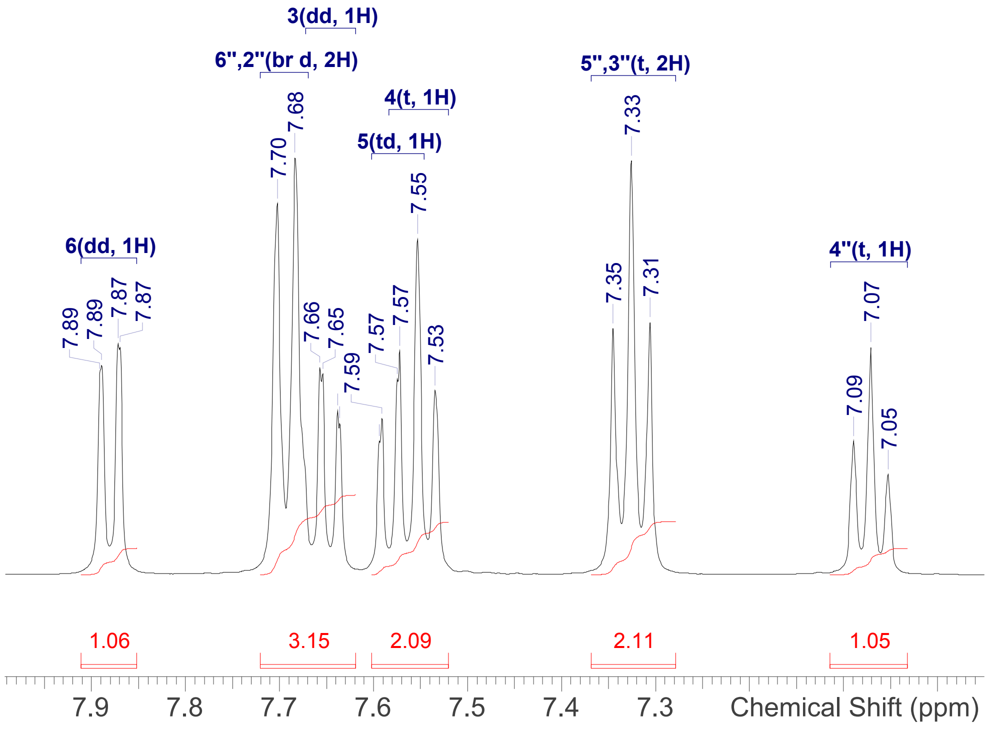

NVR-53_1H.spectrus

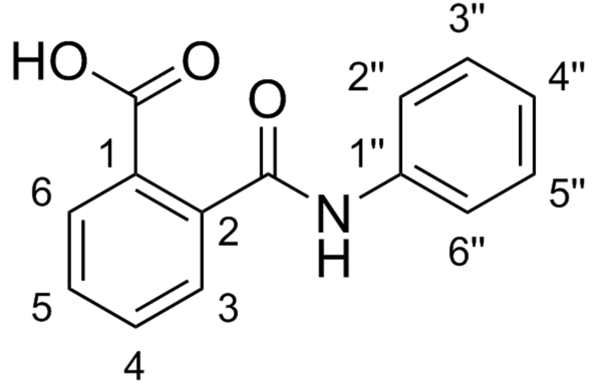

Shift (ppm)	C	m	Assign
167.5	1	s	COOH
167.4	1	s	CONH
139.6	1	s	1''
138.9	1	s	2
131.7	1	s	6
129.9	1	s	1
129.5	1	s	4
129.4	1	s	5
128.6	2	s	5'', 3''
127.8	1	s	3
123.3	1	s	4''
119.5	2	s	6'', 2''

Acquisition Time (sec)	1.0224
Date	01 May 2018 15:58:18
Date Stamp	01 May 2018 15:58:18
Frequency (MHz)	100.5977
Nucleus	13C
Number of Transients	64
Solvent	DMSO-d6
Temperature (degree C)	25.000

^{13}C NMR (101 MHz, *DMSO-d_6*) δ ppm 167.5 (s, 1 C), 167.4 (s, 1 C), 139.6 (s, 1 C), 138.9 (s, 1 C), 131.7 (s, 1 C), 129.9 (s, 1 C), 129.5 (s, 1 C), 129.4 (s, 1 C), 128.6 (s, 2 C), 127.8 (s, 1 C), 123.3 (s, 1 C), 119.5 (s, 2 C)

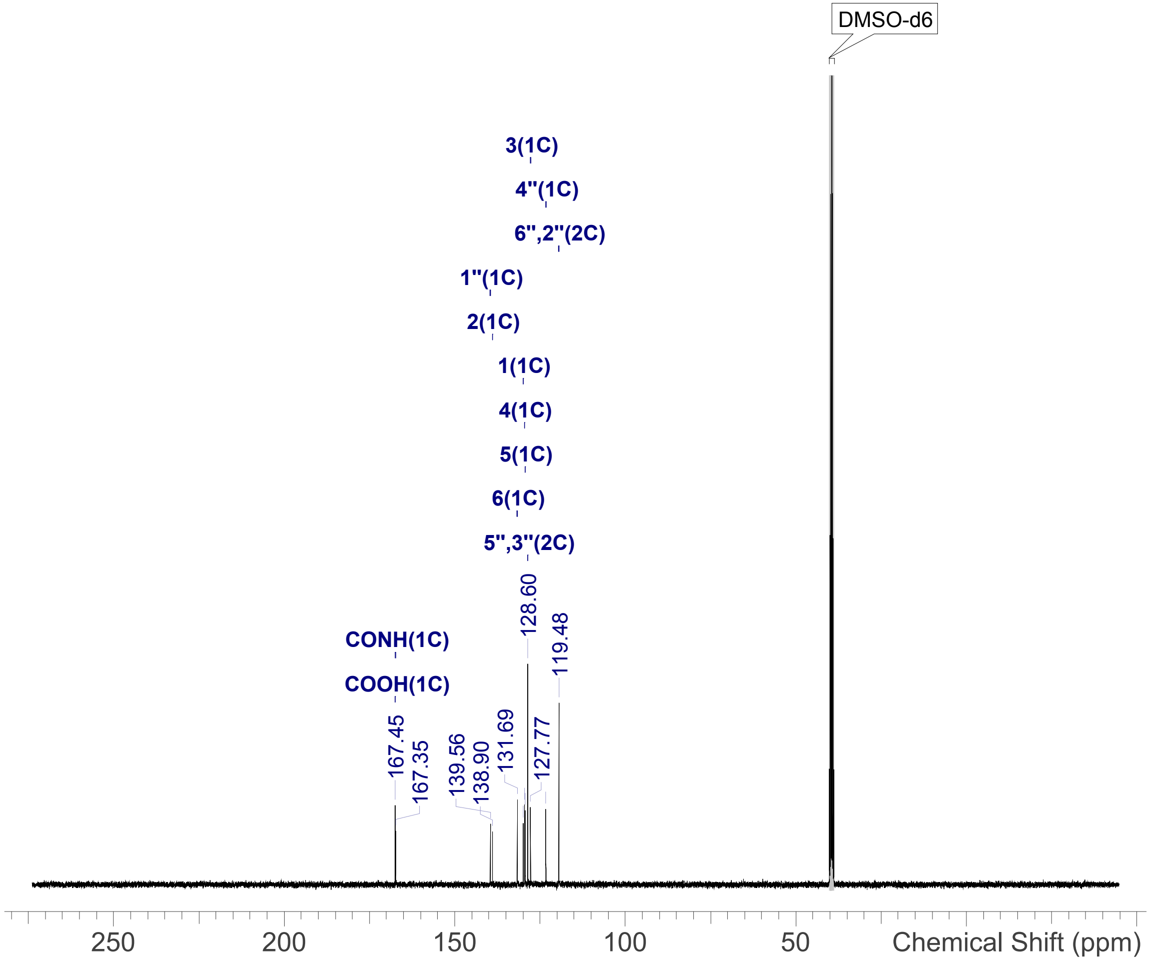

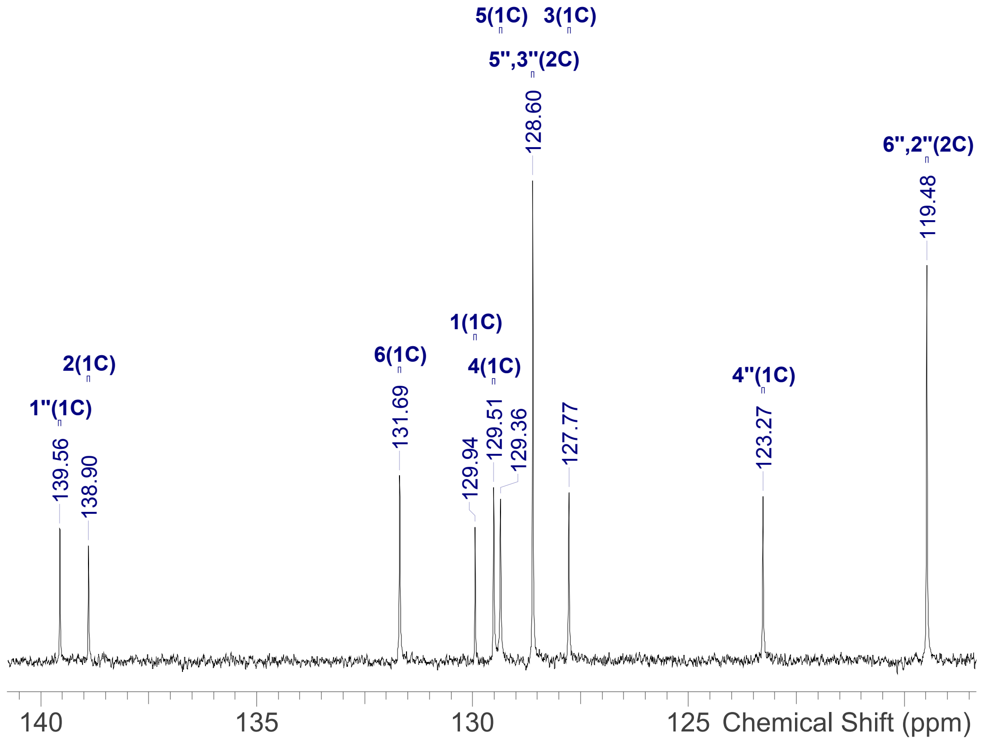

NVR-53_13C.spectrus

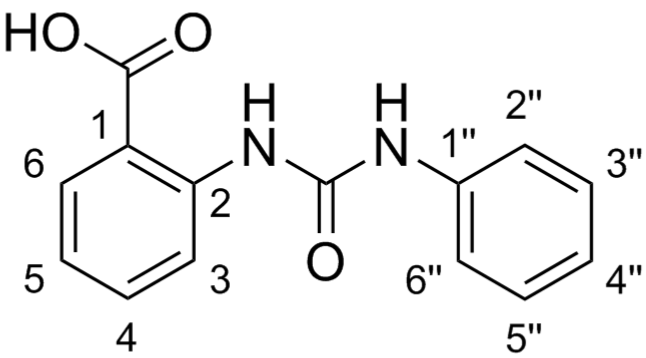

Shift (ppm)	H	m	J (Hz)	Assign
13.37	1	br s	-	COOH
10.34	1	s	-	NH
9.78	1	s	-	NH"
8.36	1	d	8.3	3
7.95	1	dd	7.8, 1.5	6
7.54	1	ddd	8.5, 7.3, 1.5	4
7.51	2	d	7.3	2", 6"
7.28	2	t	7.9	3", 5"
7.04	1	t	7.7	5
6.98	1	t	7.3	4"

Acquisition Time (sec)	3.9846
Date	01 May 2018 16:07:05
Date Stamp	01 May 2018 16:07:05
Frequency (MHz)	400.0700
Nucleus	1H
Number of Transients	16
Solvent	DMSO-d6
Temperature (degree C)	25.000

^{1}H NMR (400 MHz, *DMSO-d_6*) δ ppm 13.37 (br s, 1 H), 10.34 (s, 1 H), 9.78 (s, 1 H), 8.36 (d, *J*=8.3 Hz, 1 H), 7.95 (dd, *J*=7.8, 1.5 Hz, 1 H), 7.54 (ddd, *J*=8.5, 7.3, 1.5 Hz, 1 H), 7.51 (d, *J*=7.3 Hz, 2 H), 7.28 (t, *J*=7.9 Hz, 2 H), 7.04 (t, *J*=7.7 Hz, 1 H), 6.98 (t, *J*=7.3 Hz, 1 H)

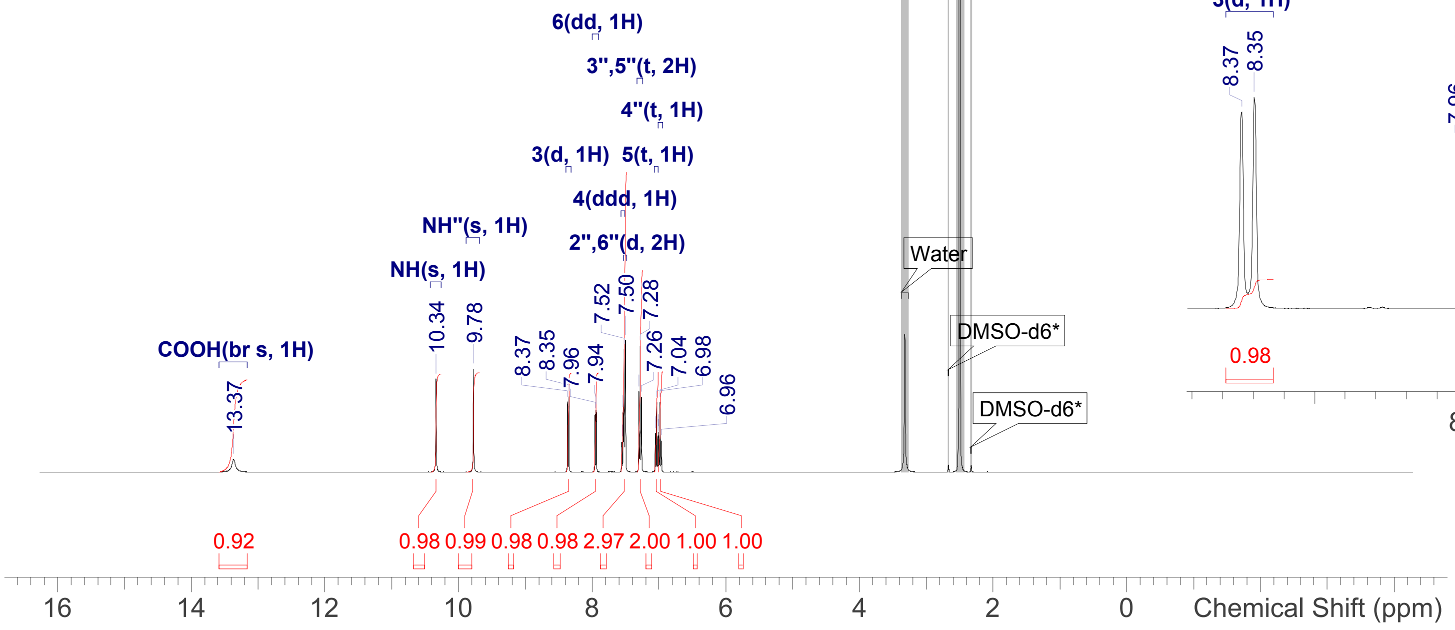

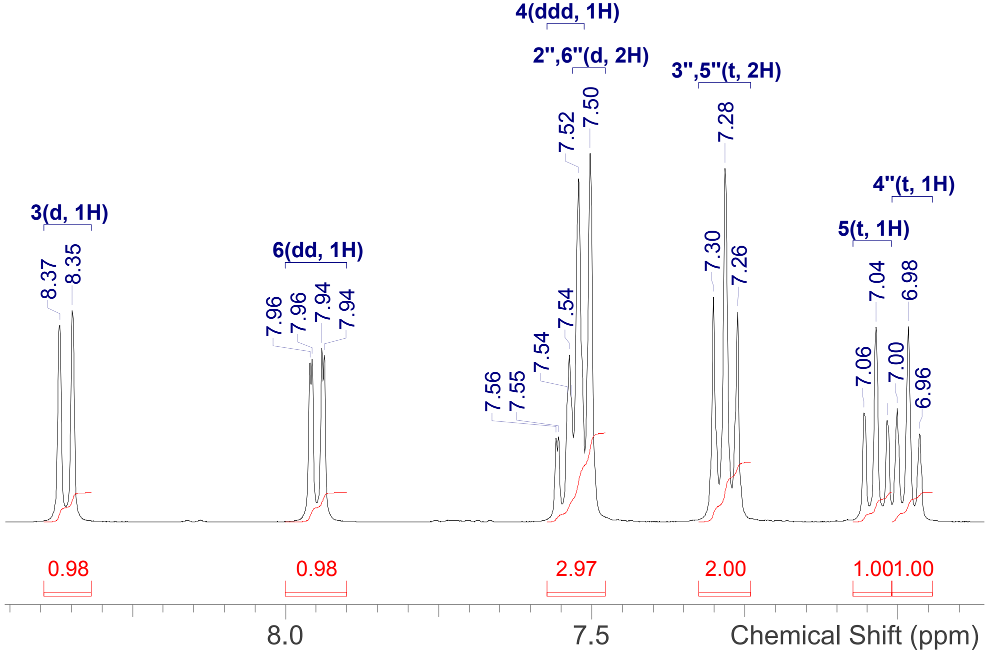

NVR-54_1H.spectrus

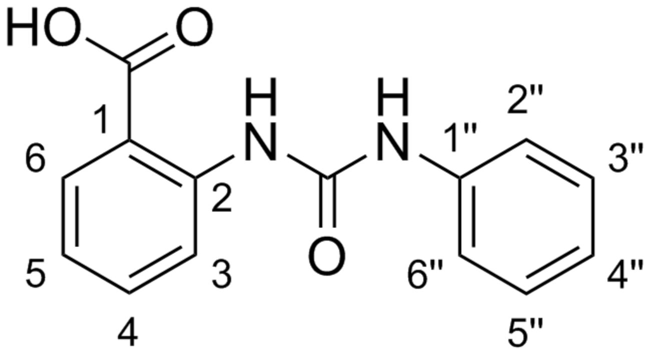

Shift (ppm)	C	m	Assign
169.4	1	s	COOH
152.3	1	s	CO
142.2	1	s	2
139.7	1	s	1''
133.7	1	s	4
131.0	1	s	6
128.7	2	s	3'', 5''
122.1	1	s	5
120.9	1	s	4''
119.8	1	s	3
118.8	2	s	2'', 6''
115.4	1	s	1

Acquisition Time (sec)	1.0224
Date	01 May 2018 16:11:28
Date Stamp	01 May 2018 16:11:28
Frequency (MHz)	100.5977
Nucleus	13C
Number of Transients	64
Solvent	DMSO-d6
Temperature (degree C)	24.999

^{13}C NMR (101 MHz, *DMSO-d_6*) δ ppm 169.4 (s, 1 C), 152.3 (s, 1 C), 142.2 (s, 1 C), 139.7 (s, 1 C), 133.7 (s, 1 C), 131.0 (s, 1 C), 128.7 (s, 2 C), 122.1 (s, 1 C), 120.9 (s, 1 C), 119.8 (s, 1 C), 118.8 (s, 2 C), 115.4 (s, 1 C)

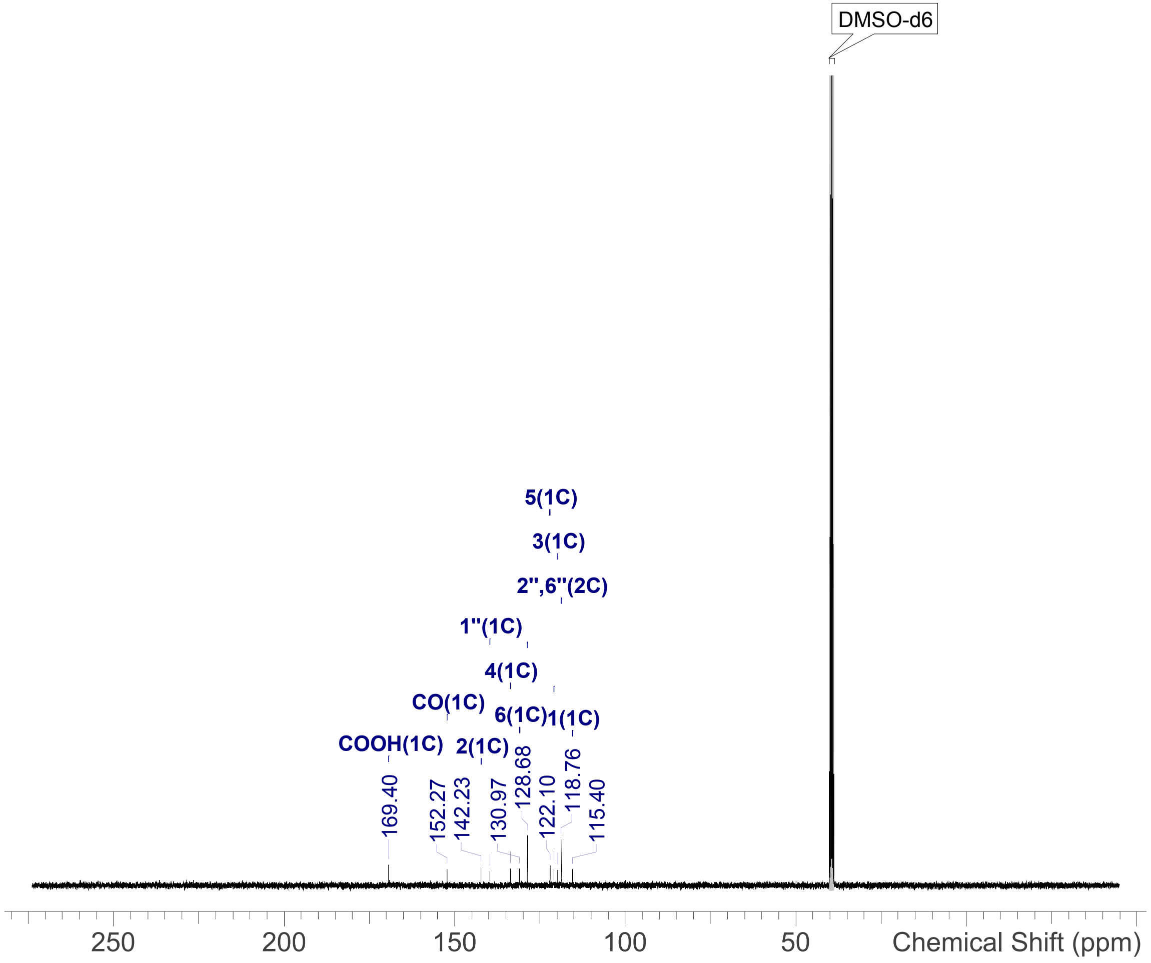

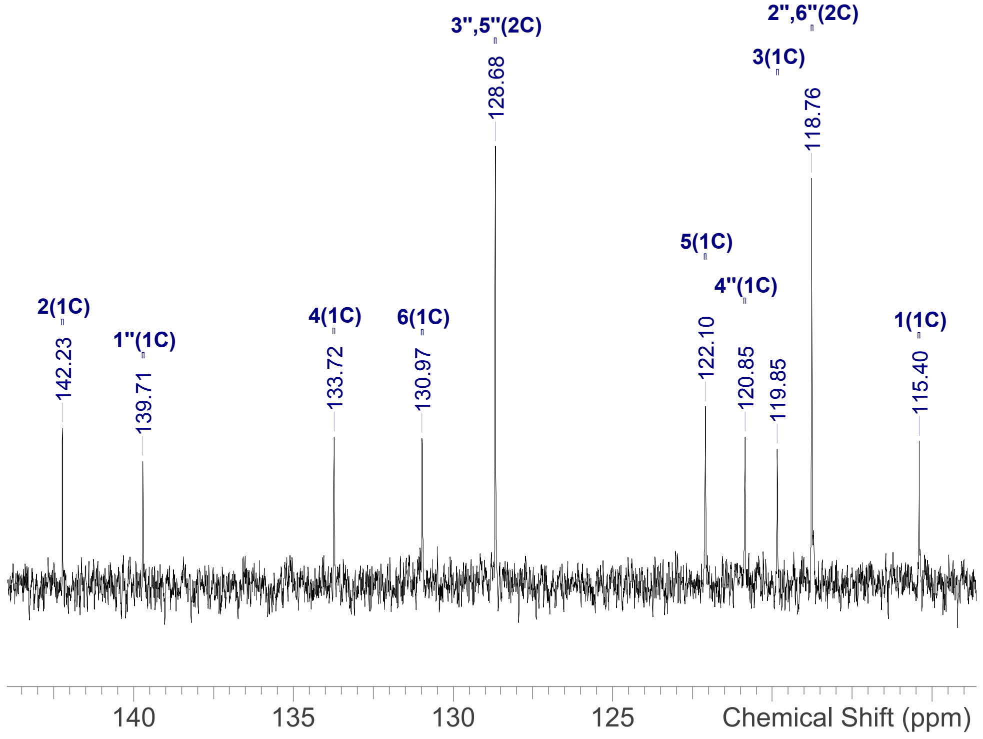

NVR-54_13C.spectrus

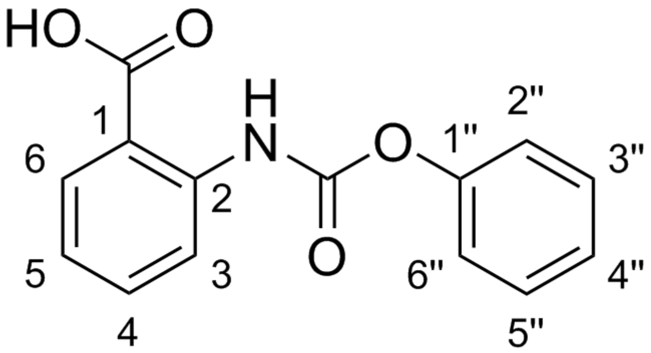

Shift (ppm)	H	m	J (Hz)	Assign
13.07	1	br s	-	COOH
9.65	1	br s	-	CONH
7.91	1	dd	7.9, 1.6	6
7.36	3	m	-	3", 5", 3
7.24	3	m	-	2", 6", 4
7.07	1	tt	7.3, 1.1	4"
6.78	1	td	7.5, 1.0	5

Acquisition Time (sec)	3.9846
Date	12 Jun 2018 14:48:18
Date Stamp	12 Jun 2018 14:48:18
Frequency (MHz)	400.0700
Nucleus	1H
Number of Transients	4
Solvent	DMSO-d6
Temperature (degree C)	24.999

^{1}H NMR (400 MHz, *DMSO-d_6*) δ ppm 13.07 (br s, 1 H), 9.65 (br s, 1 H), 7.91 (dd, *J*=7.9, 1.6 Hz, 1 H), 7.31 - 7.41 (m, 3 H), 7.20 - 7.28 (m, 3 H), 7.07 (tt, *J*=7.3, 1.1 Hz, 1 H), 6.78 (td, *J*=7.5, 1.0 Hz, 1 H)

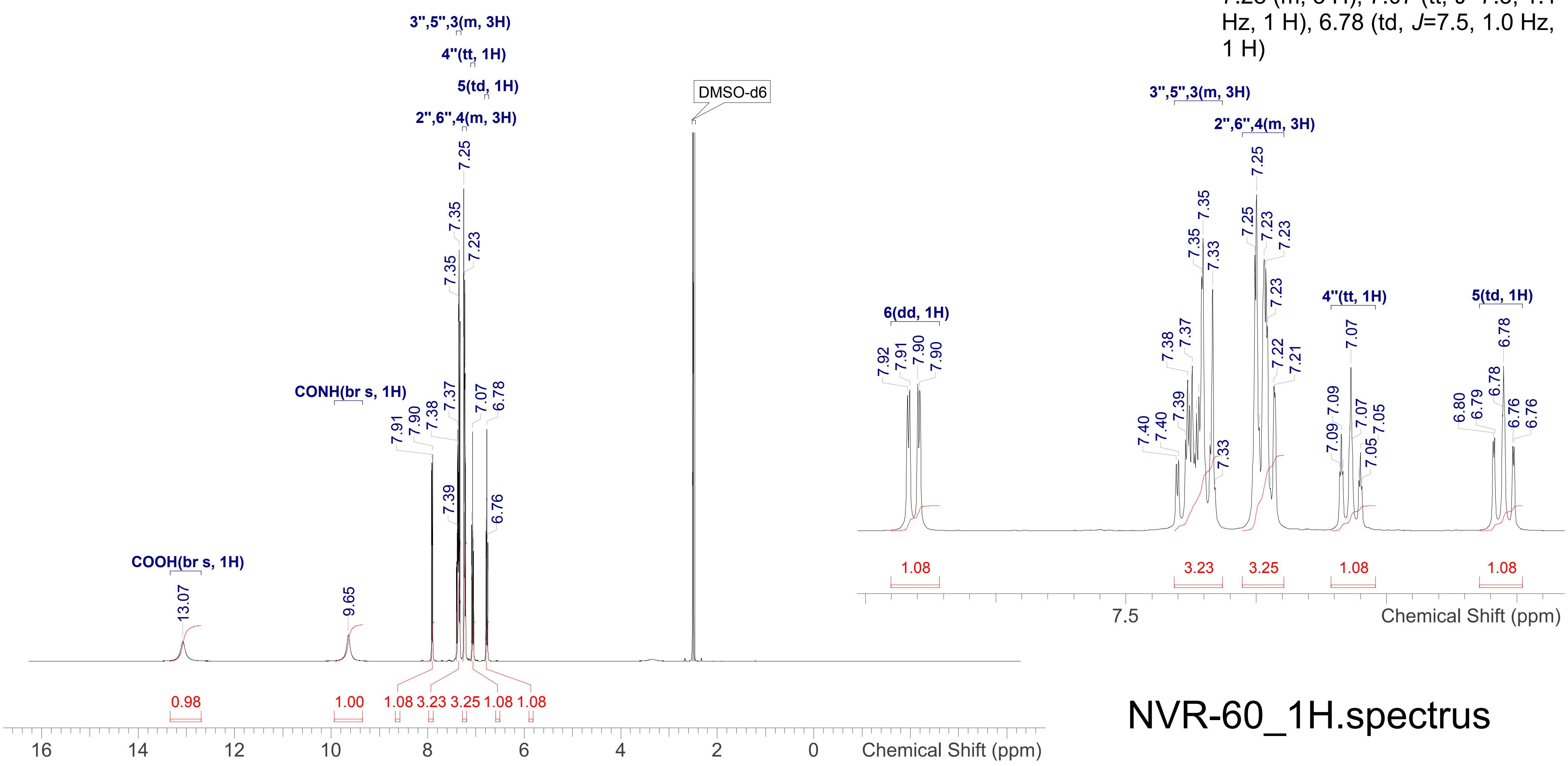

NVR-60_1H.spectrus

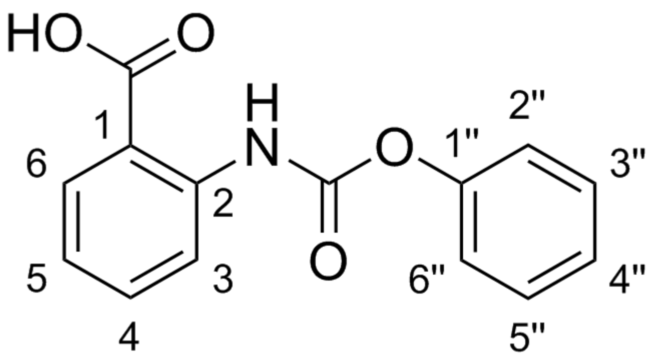

Shift (ppm)	C	m	Assign
170.0	1	s	COOH
147.0	2	s	1'', CONH
140.5	1	s	2
134.2	1	s	4
131.9	1	s	6
129.5	2	s	3'', 5''
123.1	1	s	4''
121.4	2	s	2'', 6''
117.4	1	s	5
113.7	1	s	3
112.6	1	s	1

Acquisition Time (sec)	1.0224
Date	12 Jun 2018 14:50:13
Date Stamp	12 Jun 2018 14:50:13
Frequency (MHz)	100.5977
Nucleus	13C
Number of Transients	16
Solvent	DMSO-d6
Temperature (degree C)	25.001

^{13}C NMR (101 MHz, *DMSO-d_6*) δ ppm 170.0 (s, 1 C), 147.0 (s, 2 C), 140.5 (s, 1 C), 134.2 (s, 1 C), 131.9 (s, 1 C), 129.5 (s, 2 C), 123.1 (s, 1 C), 121.4 (s, 2 C), 117.4 (s, 1 C), 113.7 (s, 1 C), 112.6 (s, 1 C)

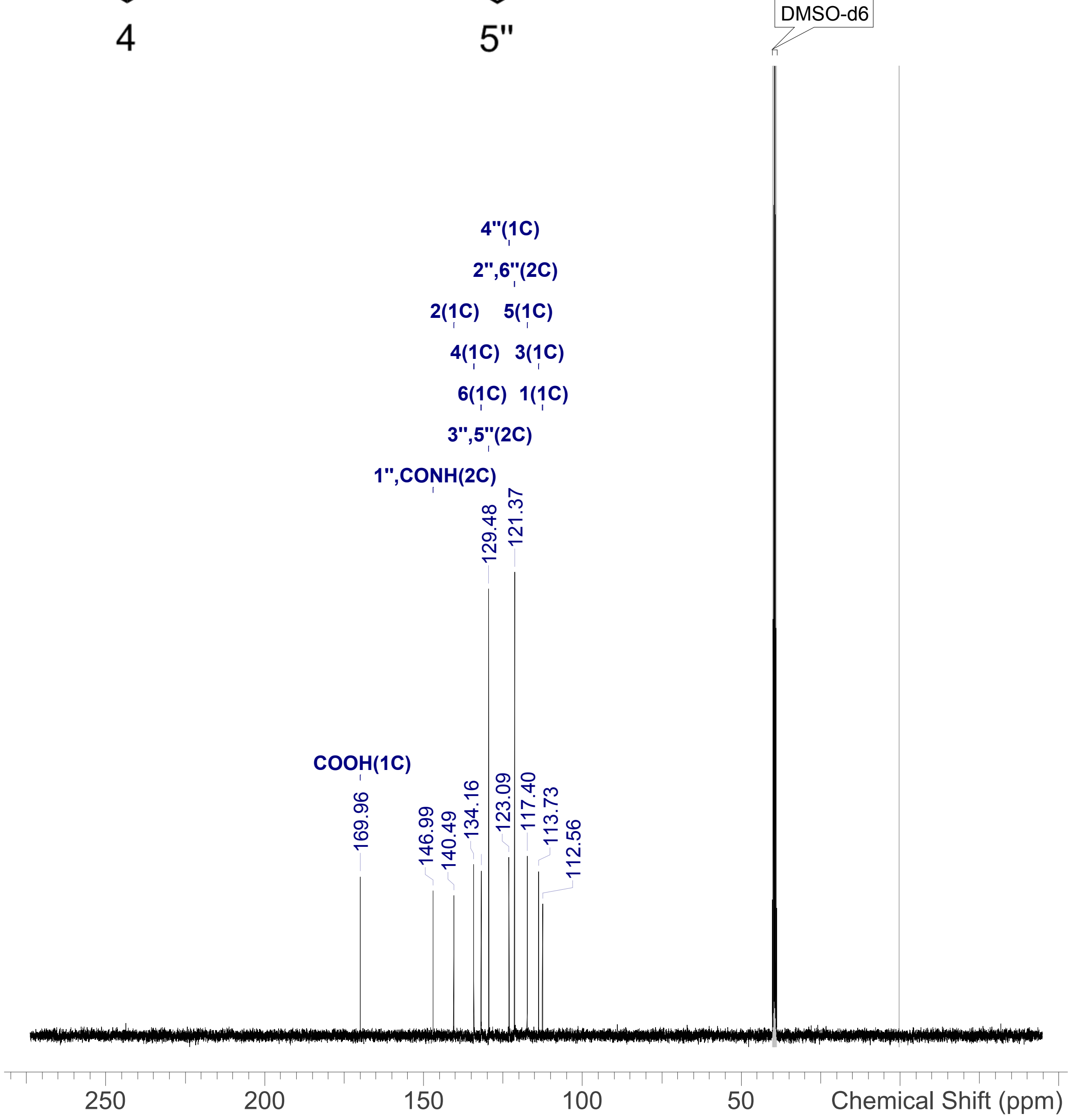

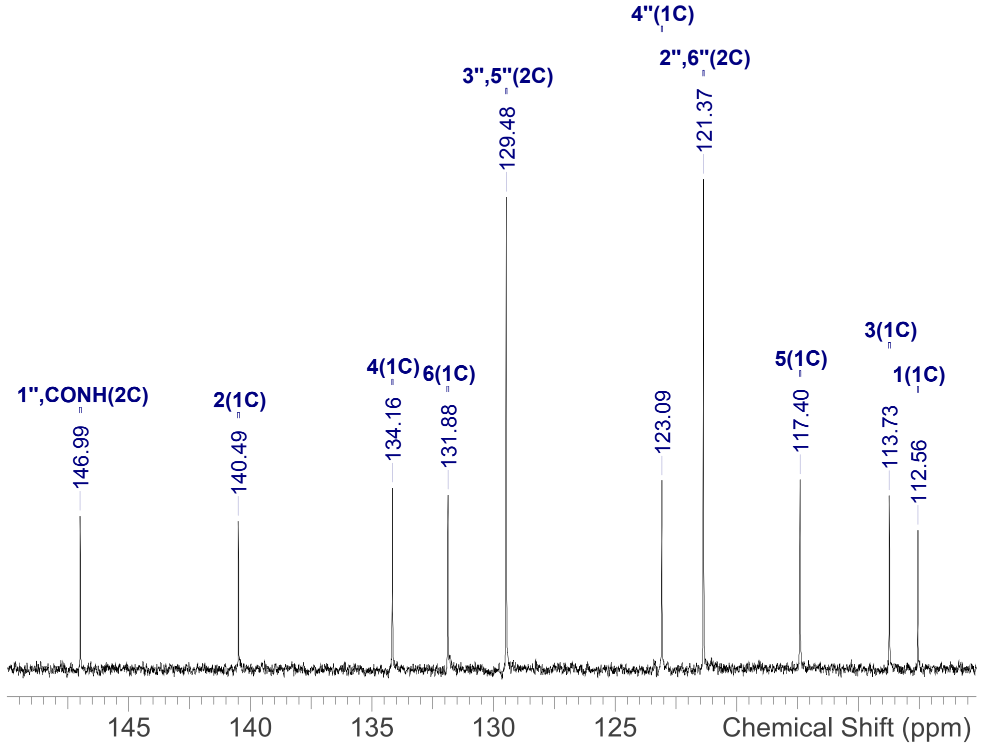

NVR-60_13C.spectrus

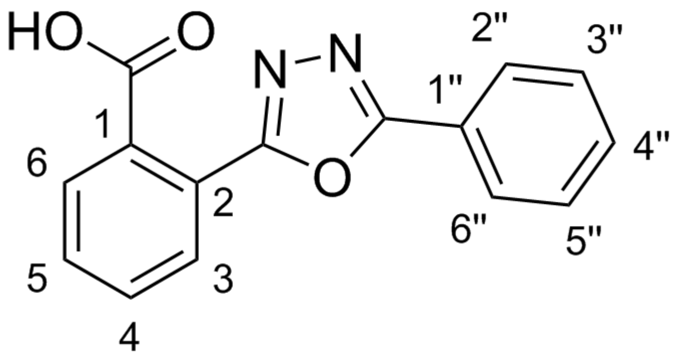

Shift (ppm)	H	m	J (Hz)	Assign
13.68	1	br s	-	COOH
8.04	2	dd	7.9, 1.6	2'', 6''
7.94	2	m	-	3, 6
7.78	2	m	-	4'', 4
7.64	3	m	-	5, 3'', 5''

Acquisition Time (sec)	3.9846
Date	09 Aug 2018 13:07:42
Date Stamp	09 Aug 2018 13:07:42
Frequency (MHz)	400.0700
Nucleus	1H
Number of Transients	16
Solvent	DMSO-d6
Temperature (degree C)	25.000

^{1}H NMR (400 MHz, *DMSO-d_6*) δ ppm 13.68 (br s, 1 H), 8.04 (dd, *J*=7.9, 1.6 Hz, 2 H), 7.89 - 7.97 (m, 2 H), 7.74 - 7.81 (m, 2 H), 7.60 - 7.68 (m, 3 H)

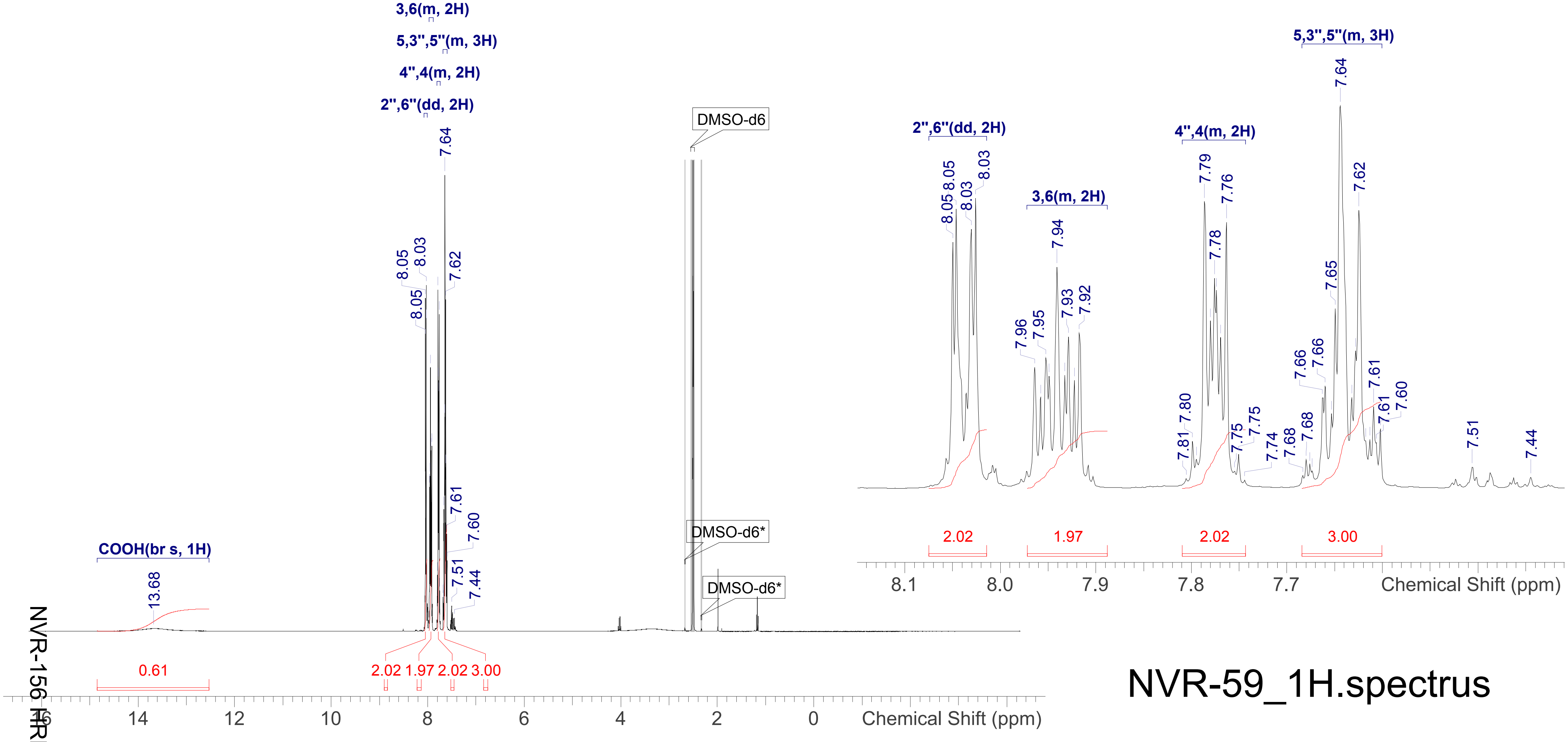

NVR-59_1H.spectrus

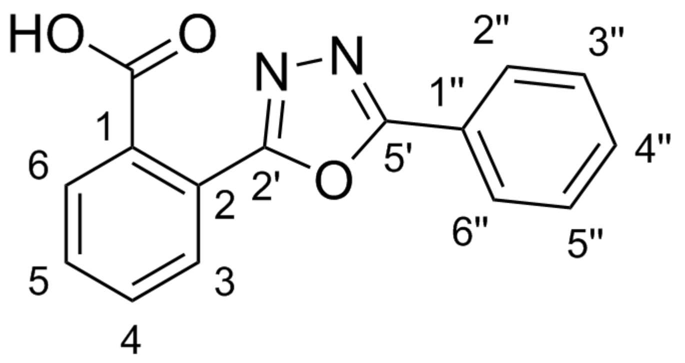

Shift (ppm)	C	m	Assign
167.7	1	s	COOH
164.3	1	s	2'
163.9	1	s	5'
132.9	1	s	1
132.1	1	s	4
131.9	1	s	6
131.7	1	s	4''
130.5	1	s	5
129.7	1	s	3
129.5	2	s	5'', 3''
126.5	2	s	6'', 2''
123.3	1	s	2
123.0	1	s	1''

Acquisition Time (sec)	1.0224
Date	10 Aug 2018 00:02:10
Date Stamp	10 Aug 2018 00:02:10
Frequency (MHz)	100.5977
Nucleus	13C
Number of Transients	256
Solvent	DMSO-d6
Temperature (degree C)	25.000

^{13}C NMR (101 MHz, *DMSO-d_6*) δ ppm 167.7 (s, 1 C), 164.3 (s, 1 C), 163.9 (s, 1 C), 132.9 (s, 1 C), 132.1 (s, 1 C), 131.9 (s, 1 C), 131.7 (s, 1 C), 130.5 (s, 1 C), 129.7 (s, 1 C), 129.5 (s, 2 C), 126.5 (s, 2 C), 123.3 (s, 1 C), 123.0 (s, 1 C)

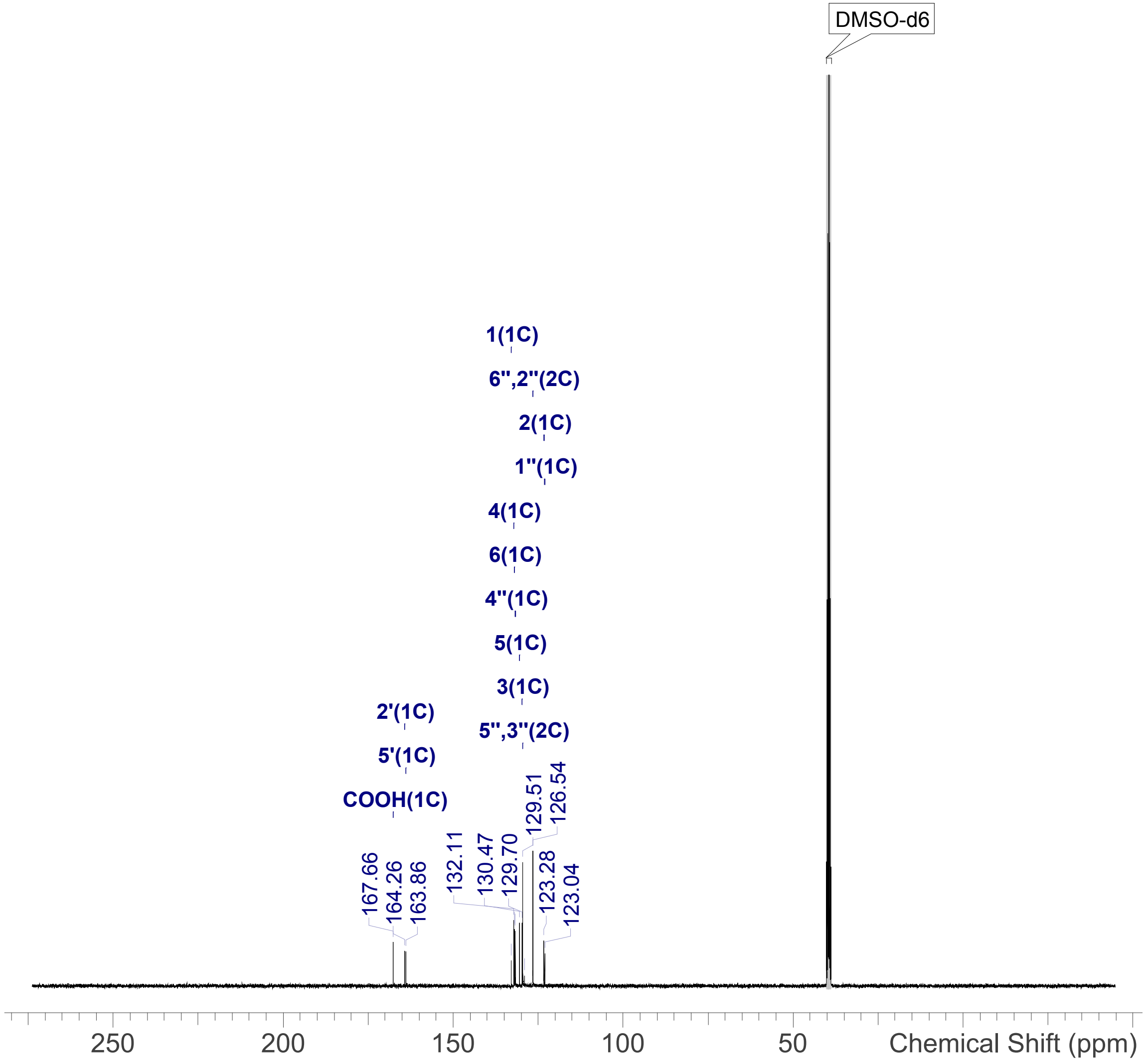

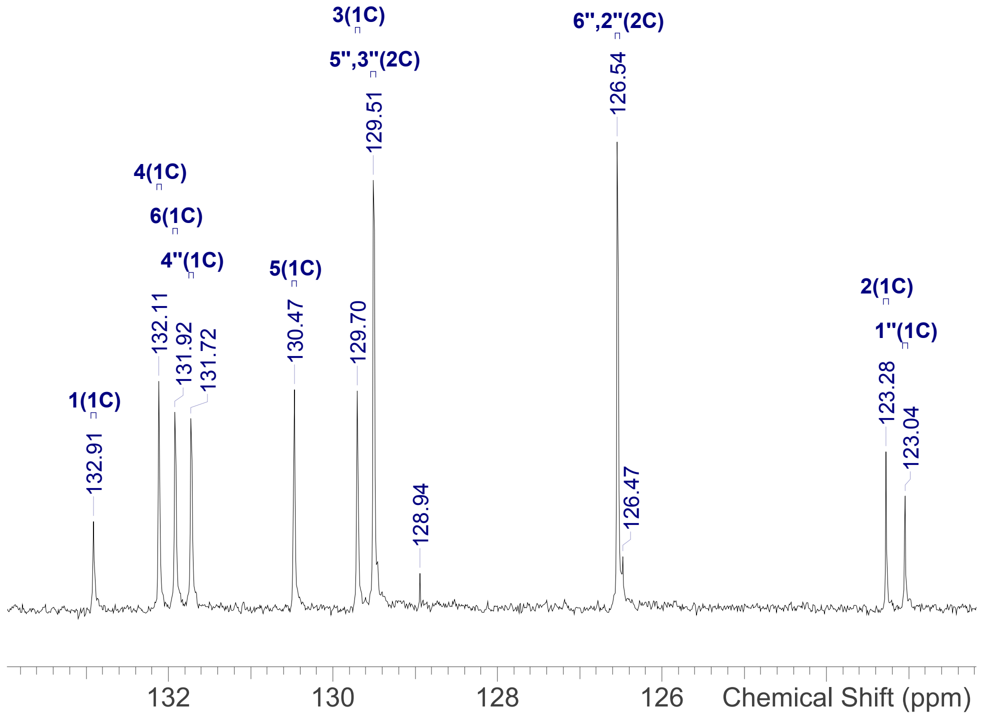

NVR-59_13C.spectrus

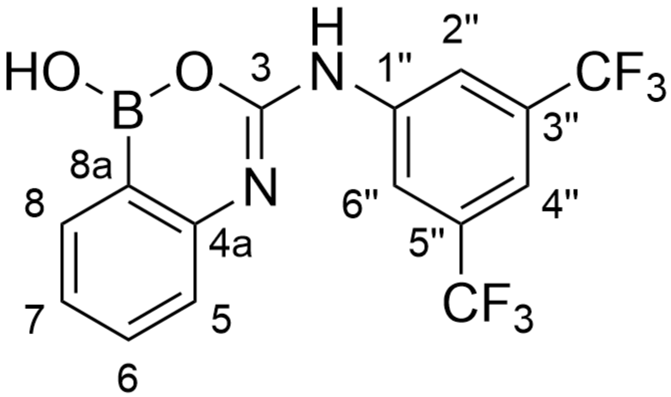

Shift (ppm)	H	m	J (Hz)	Assign
10.59	1	s	-	BOH
9.40	1	br s	-	NH
8.06	1	s	-	4"
8.02	2	s	-	2", 6"
7.99	1	br d	7.5	8
7.50	1	ddd	8.2, 7.1, 1.3	6
7.11	1	d	8.0	5
7.07	1	t	7.3	7

Acquisition Time (sec)	3.9846
Date	09 Mar 2020 15:52:38
Date Stamp	09 Mar 2020 15:52:38
Frequency (MHz)	400.0700
Nucleus	1H
Number of Transients	4
Solvent	DMSO-d6
Temperature (degree C)	20.263

^{1}H NMR (400 MHz, *DMSO-d_6*) δ ppm 10.59 (s, 1 H), 9.40 (br s, 1 H), 8.06 (s, 1 H), 8.02 (s, 2 H), 7.99 (br d, *J*=7.5 Hz, 1 H), 7.50 (ddd, *J*=8.2, 7.1, 1.3 Hz, 1 H), 7.11 (d, *J*=8.0 Hz, 1 H), 7.07 (t, *J*=7.3 Hz, 1 H)

NVR-154_1H

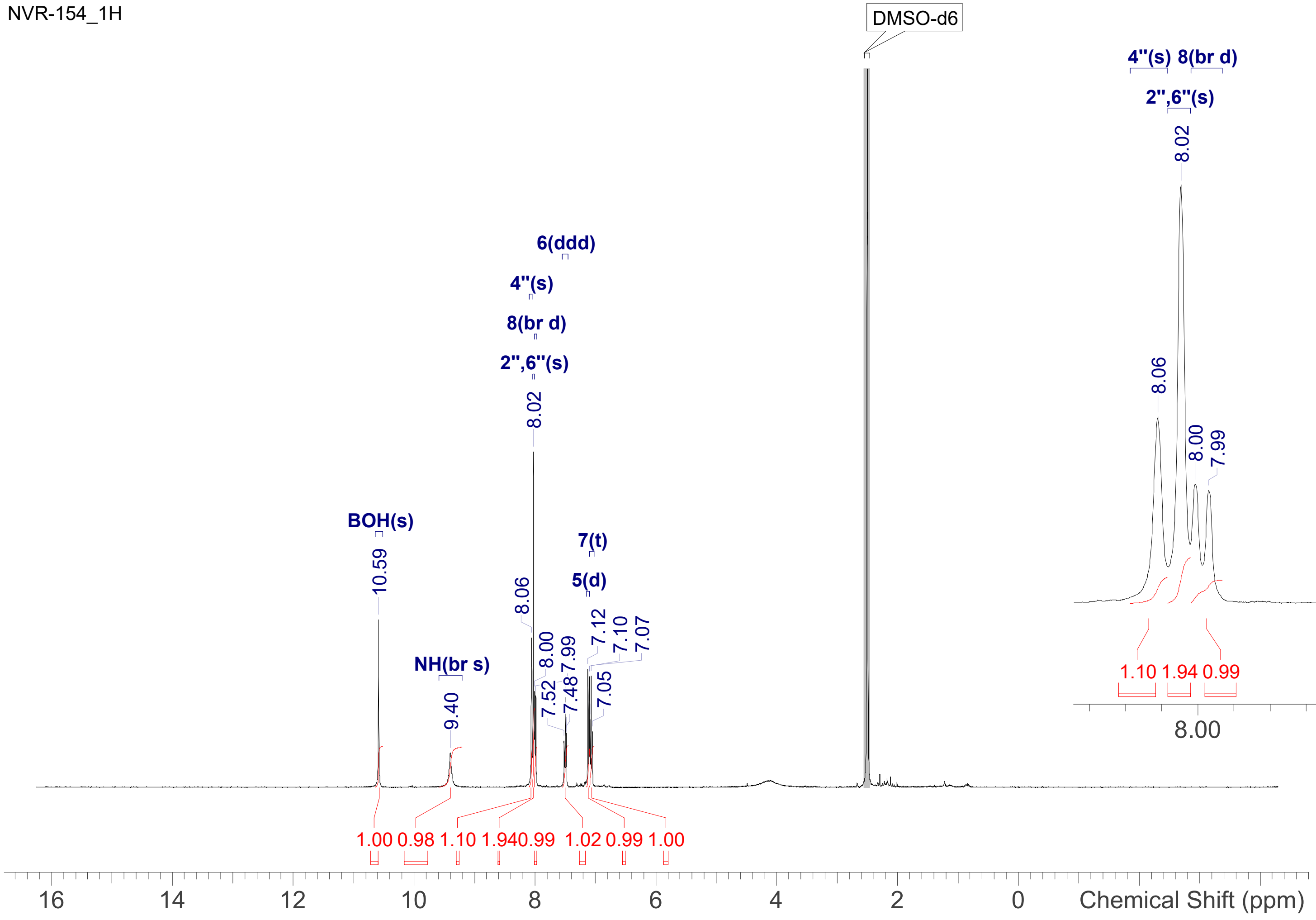

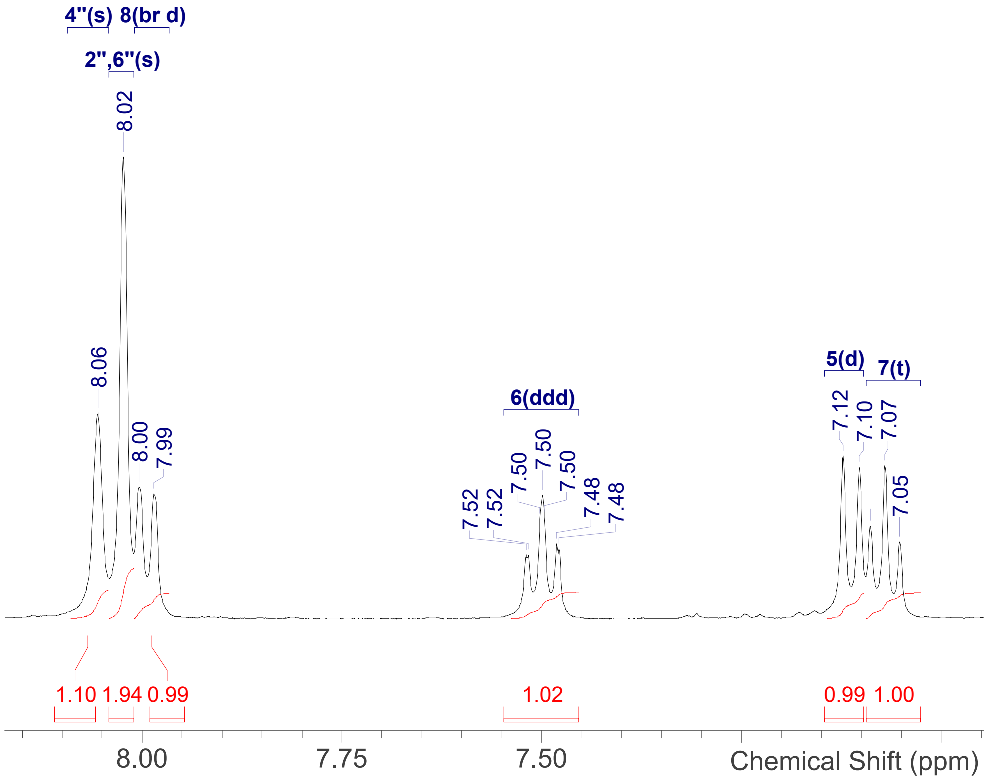

NVR-154_1H.spectrus

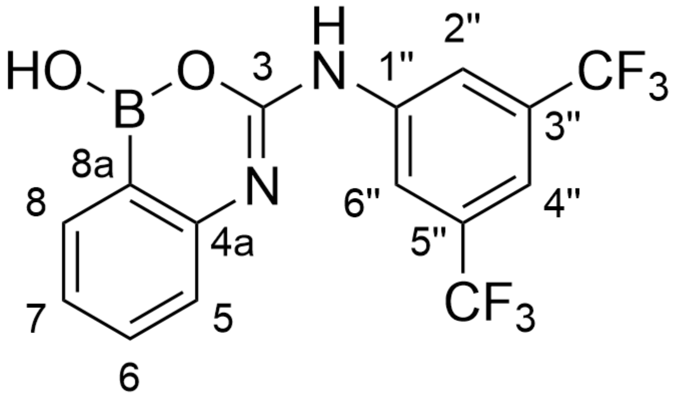

Shift (ppm)	C	m	J (Hz)	Assign
153.7	1	s	-	3
145.4	1	s	-	4a
141.5	1	s	-	1"
132.8	1	s	-	6
132.7	1	s	-	8
130.4	2	br q	2.9	2", 6"
130.3	2	q	32.9	3", 5"
123.3	2	q	272.2	5"-CF3, 3"-CF3
121.0	1	s	-	7
120.1	1	br spt	3.9	4"
114.5	1	s	-	5
114.2	1	br s	-	8a

Acquisition Time (sec)	1.0224
Date	10 Mar 2020 06:47:01
Date Stamp	10 Mar 2020 06:47:01
Frequency (MHz)	100.5977
Nucleus	13C
Number of Transients	256
Solvent	DMSO-d6
Temperature (degree C)	20.895

^{13}C NMR (101 MHz, *DMSO-d$_6$*) δ ppm 153.7 (s, 1 C), 145.4 (s, 1 C), 141.5 (s, 1 C), 132.8 (s, 1 C), 132.7 (s, 1 C), 130.4 (br q, *J*=2.9 Hz, 2 C), 130.3 (q, *J*=32.9 Hz, 2 C), 121.0 (s, 1 C), 120.1 (br spt, *J*=3.9 Hz, 1 C), 123.3 (q, *J*=272.2 Hz, 2 C), 114.5 (s, 1 C), 114.2 (br s, 1 C)

NVR-154_13C

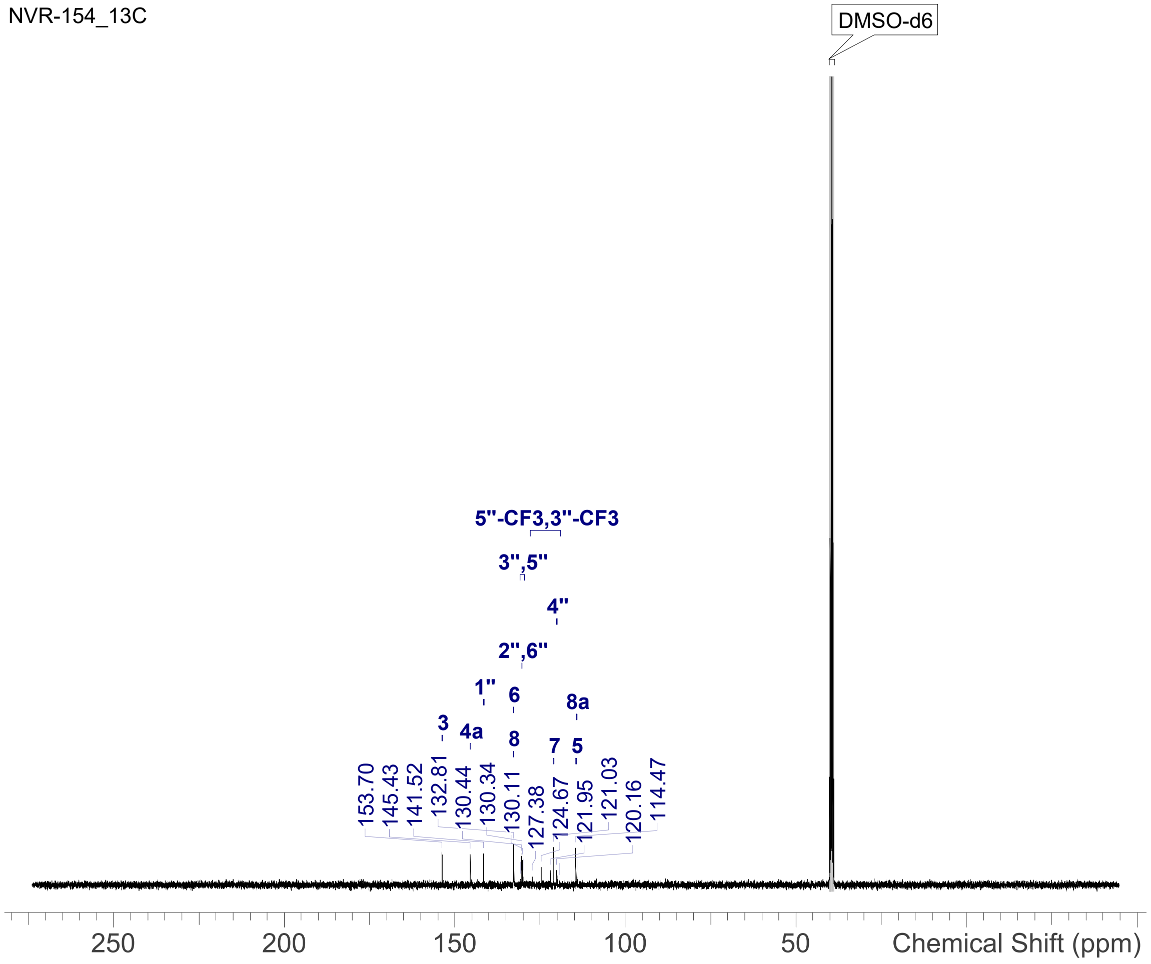

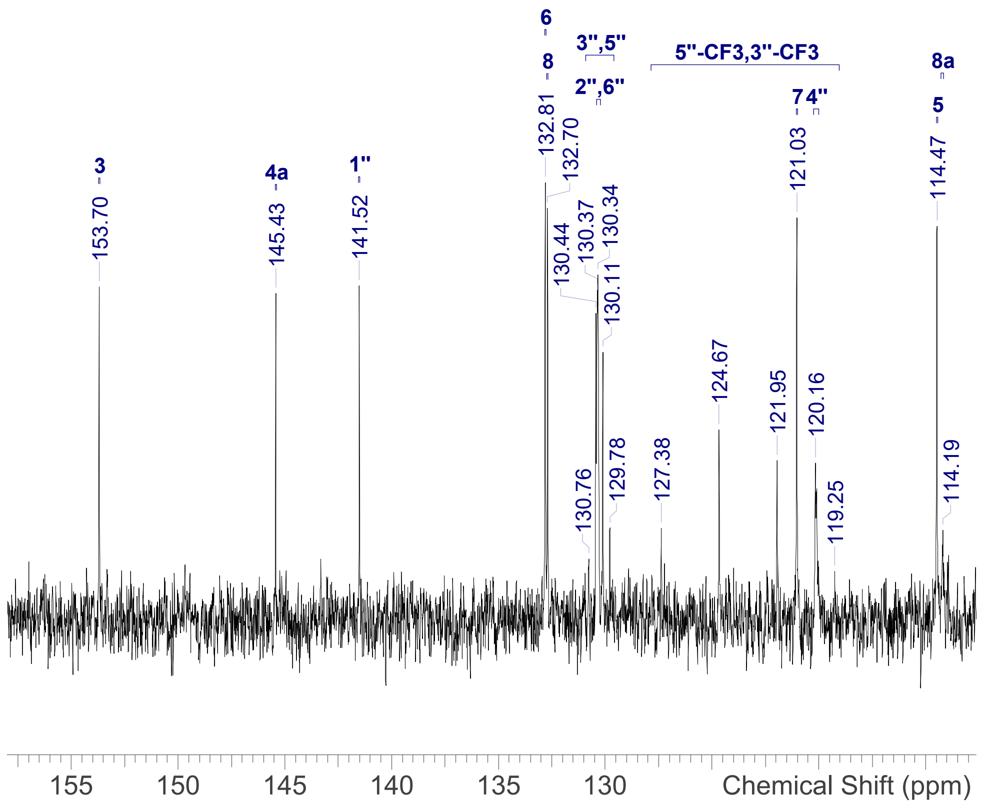

NVR-154_13C.spectrus

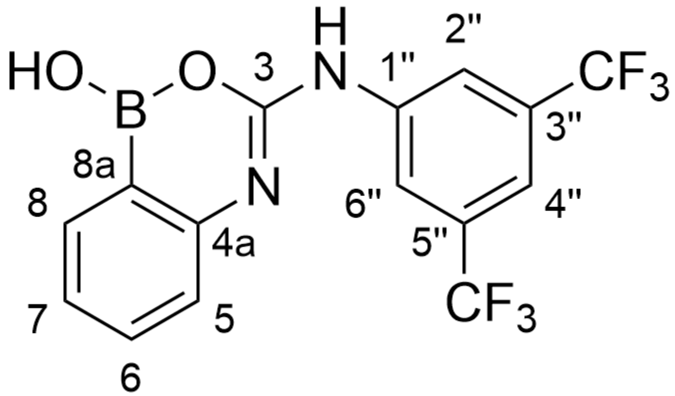

Shift (ppm)	F	m
-61.09	6	s

Acquisition Time (sec)	1.4680
Date	10 Mar 2020 06:30:05
Date Stamp	10 Mar 2020 06:30:05
Frequency (MHz)	376.4419
Nucleus	19F
Number of Transients	16
Solvent	DMSO-d6
Temperature (degree C)	20.092

^{19}F NMR (376 MHz, *DMSO-d_6*) δ ppm -61.09 (s, 6 F)

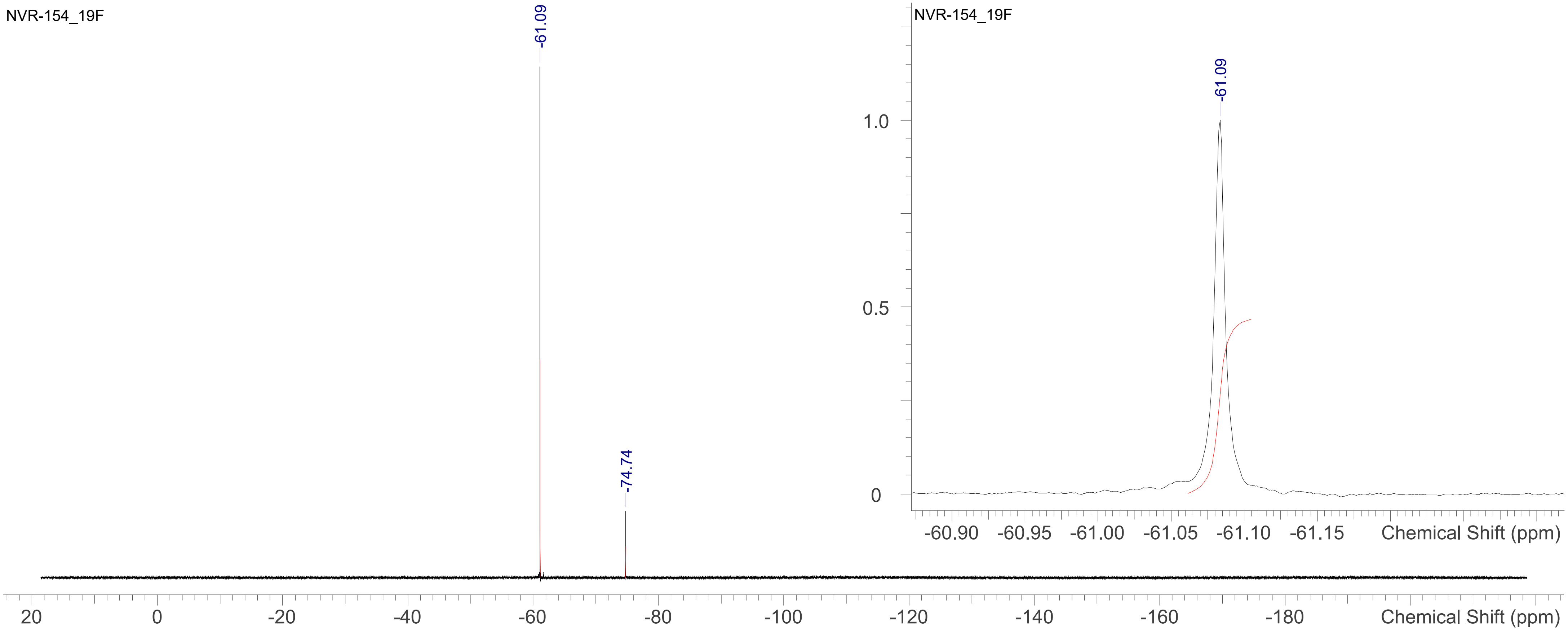

NVR-154_19F.spectrus

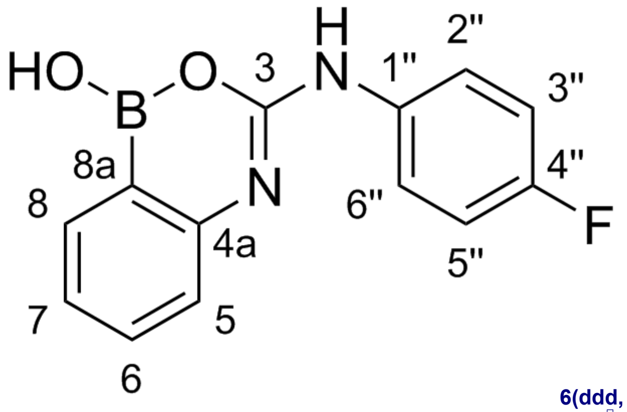

Shift (ppm)	H	m	J (Hz)	Assign
10.45	1	s	-	BOH
9.09	1	s	-	NH
7.99	1	d	6.8	8
7.47	1	ddd	8.3, 7.2, 1.4	6
7.20	4	d	7.0	2'', 6'', 3'', 5''
7.08	1	d	8.0	5
7.04	1	t	7.4	7

Acquisition Time (sec)	3.9846
Date	27 Feb 2020 03:22:41
Date Stamp	27 Feb 2020 03:22:41
Frequency (MHz)	400.0700
Nucleus	1H
Number of Transients	16
Solvent	DMSO-d6
Temperature (degree C)	19.591

^{1}H NMR (400 MHz, *DMSO-d_6*) δ ppm 10.45 (s, 1 H), 9.09 (s, 1 H), 7.99 (d, *J*=6.8 Hz, 1 H), 7.47 (ddd, *J*=8.3, 7.2, 1.4 Hz, 1 H), 7.20 (d, *J*=7.0 Hz, 4 H), 7.08 (d, *J*=8.0 Hz, 1 H), 7.04 (t, *J*=7.4 Hz, 1 H)

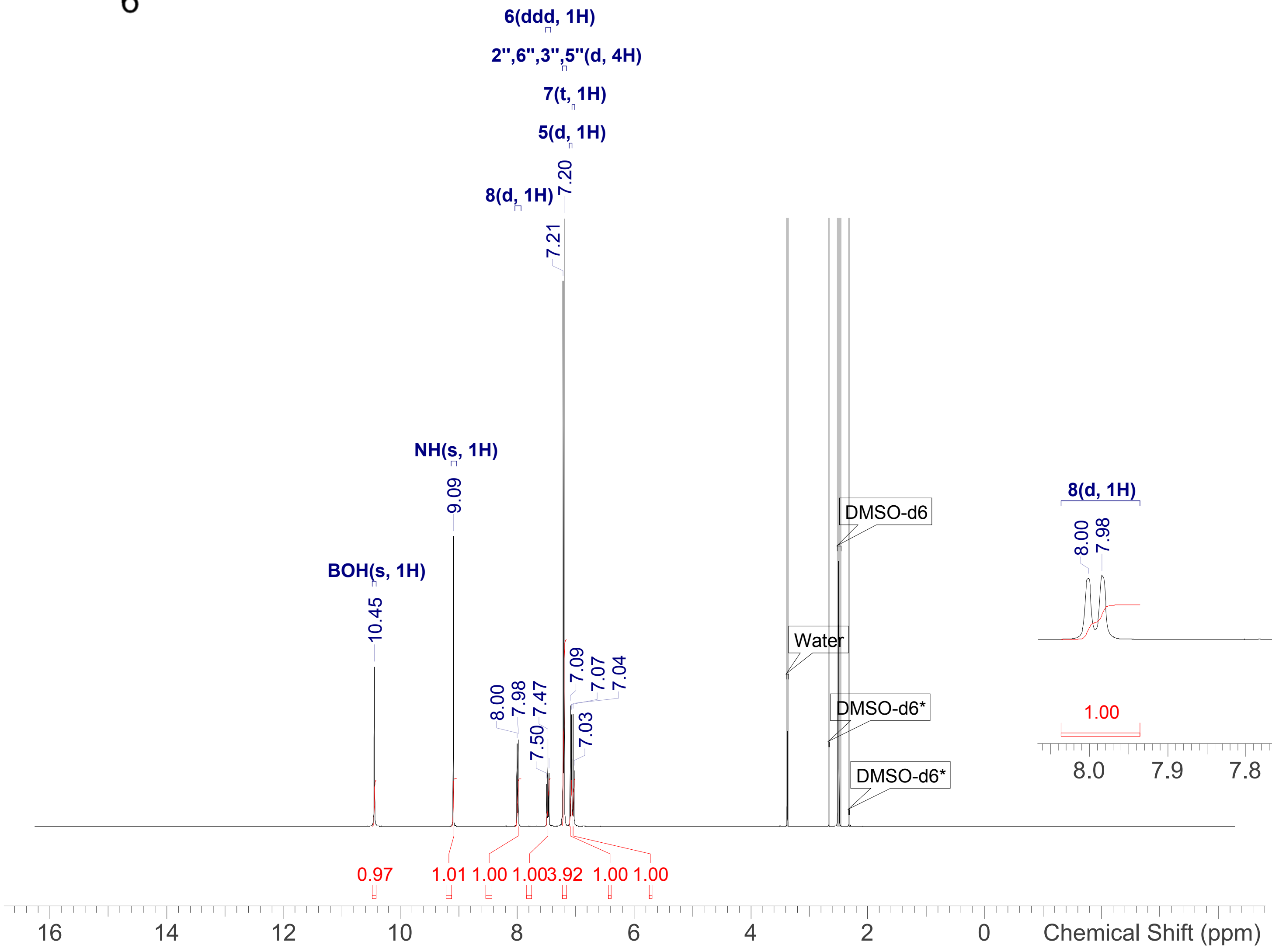

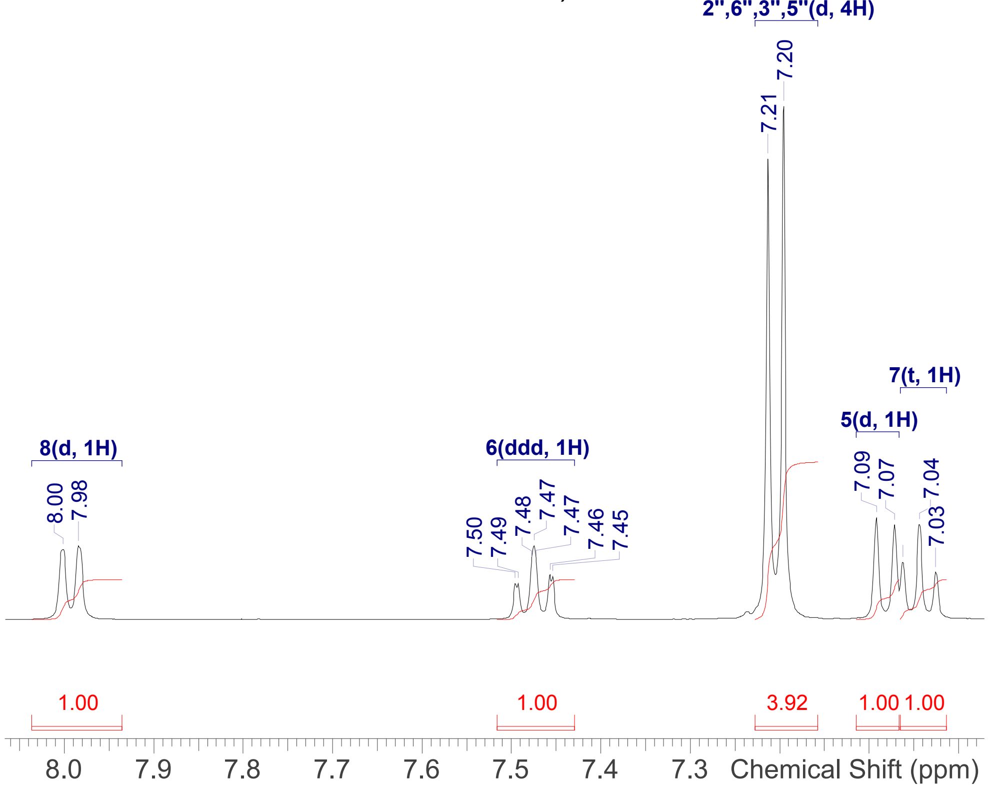

NVR-156_1H.spectrus

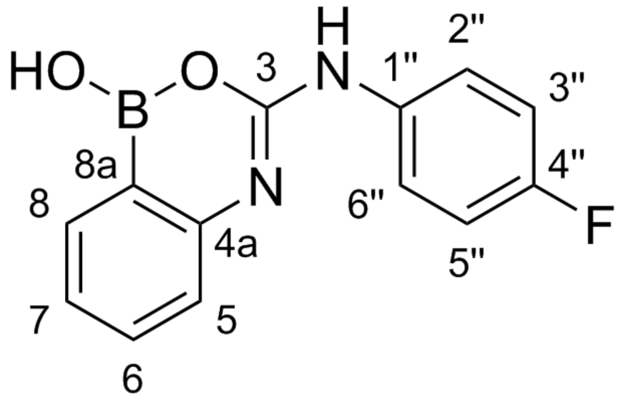

Shift (ppm)	C	m	J (Hz)	Assign
160.6	1	d	241.6	4"
154.2	1	s	-	3
145.6	1	s	-	4a
135.4	1	d	2.9	1"
132.7	2	s	-	8, 6
130.6	2	d	8.8	2", 6"
120.8	1	s	-	7
115.0	2	d	22.5	3", 5"
114.3	1	s	-	5

Acquisition Time (sec)	1.0224
Date	27 Feb 2020 03:37:08
Date Stamp	27 Feb 2020 03:37:08
Frequency (MHz)	100.5977
Nucleus	13C
Number of Transients	256
Solvent	DMSO-d6
Temperature (degree C)	20.458

^{13}C NMR (101 MHz, *DMSO-d$_6$*) δ ppm 160.6 (d, *J*=241.6 Hz, 1 C), 154.2 (s, 1 C), 145.6 (s, 1 C), 135.4 (d, *J*=2.9 Hz, 1 C), 132.7 (s, 2 C), 130.6 (d, *J*=8.8 Hz, 2 C), 120.8 (s, 1 C), 115.0 (d, *J*=22.5 Hz, 2 C), 114.3 (s, 1 C)

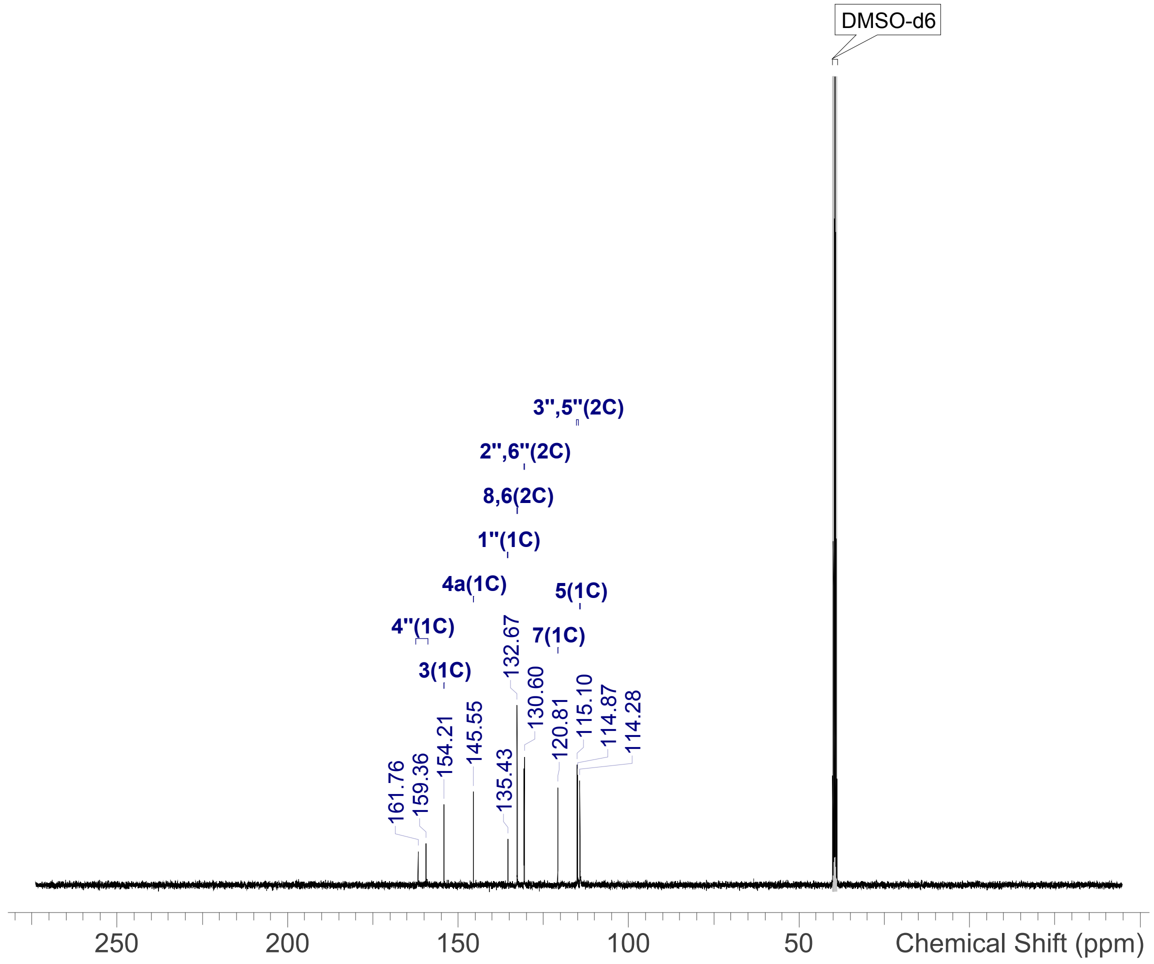

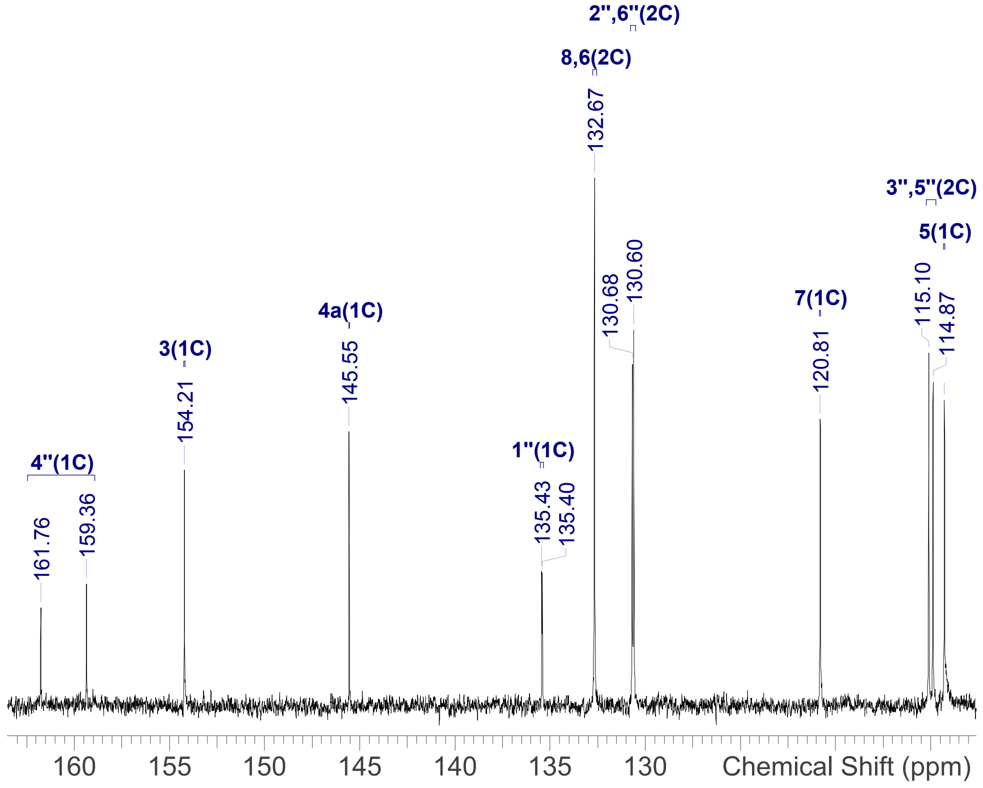

NVR-156_13C.spectrus

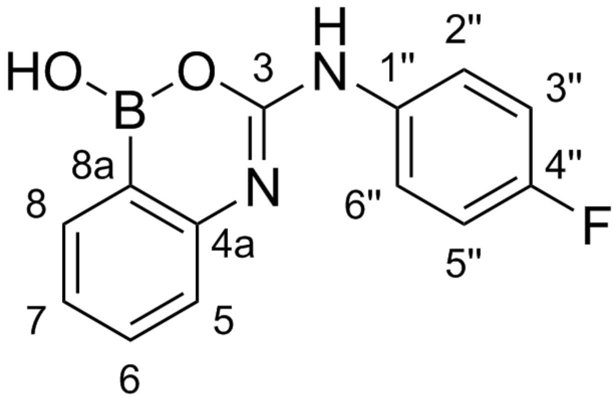

Shift (ppm)	F	m	J (Hz)
-117.05	1	dt	13.3, 6.8

Acquisition Time (sec)	1.4680
Date	27 Feb 2020 03:20:23
Date Stamp	27 Feb 2020 03:20:23
Frequency (MHz)	376.4419
Nucleus	19F
Number of Transients	128
Solvent	DMSO-d6
Temperature (degree C)	19.600

^{19}F NMR (376 MHz, *DMSO-d_6*) δ ppm -117.05 (dt, *J*=13.3, 6.8 Hz, 1 F)

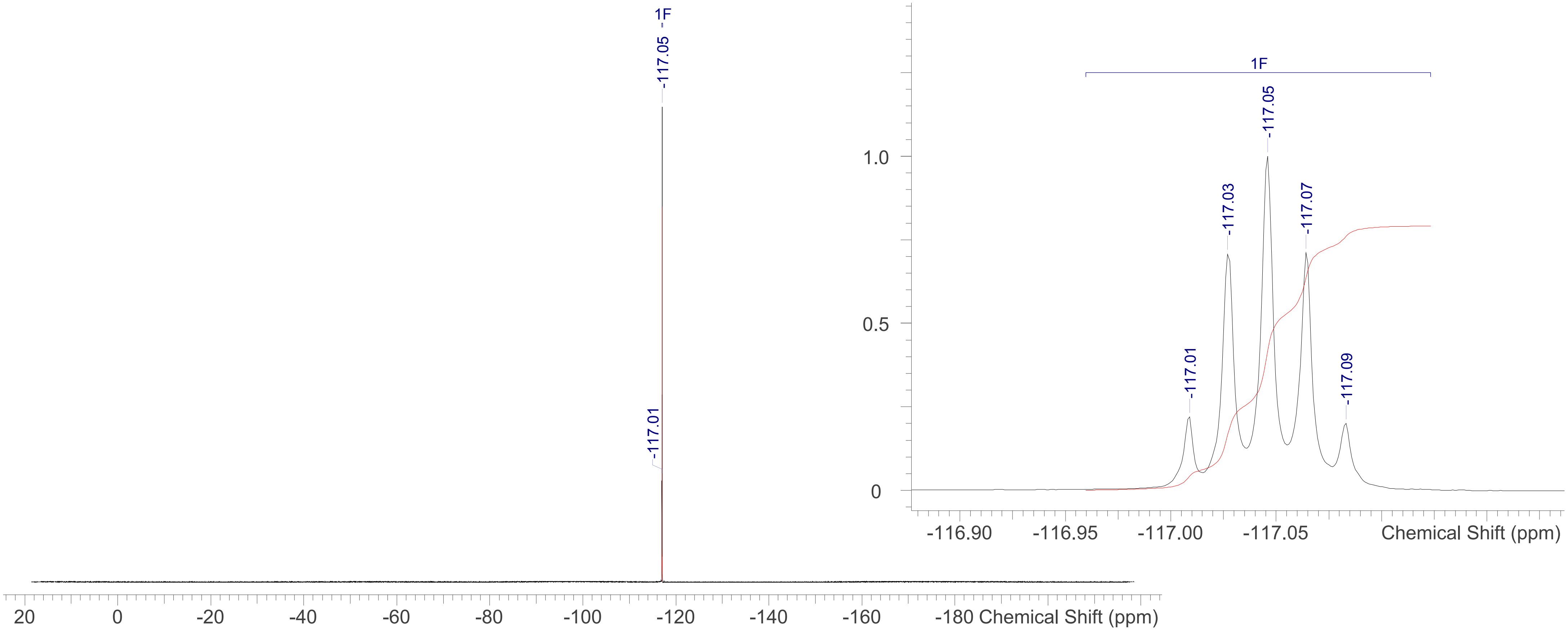

NVR-156_19F.spectrus

Openlynx Report - James B

Page 1

Sample: 1 | Vial:1:19 | ID:JB175 filtsol-1
File:JB175 filtsol-1 | Date:19-Jun-2019 | Time:18:29:26
Description:

Printed: Thu Jun 20 14:20:48 2019

3: UV Detector: TAC: Wavelength Range: (210 - 400)

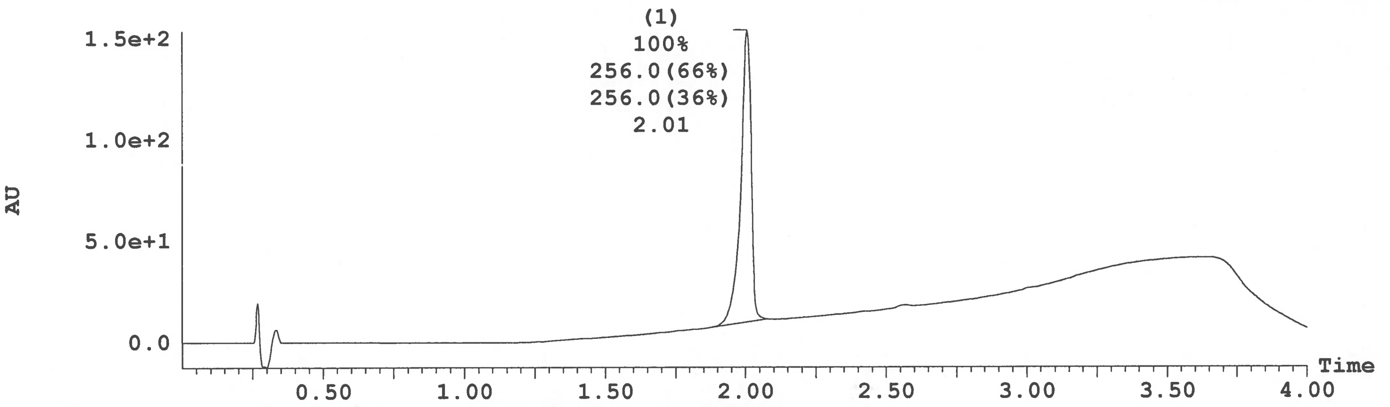

Peak Number	Compound	Time	Area %Total	Mass Found
1	Found	2.01	100.00	256.0, 256.0

1: MS ES+ :TIC Smooth (Mn, 2x2)

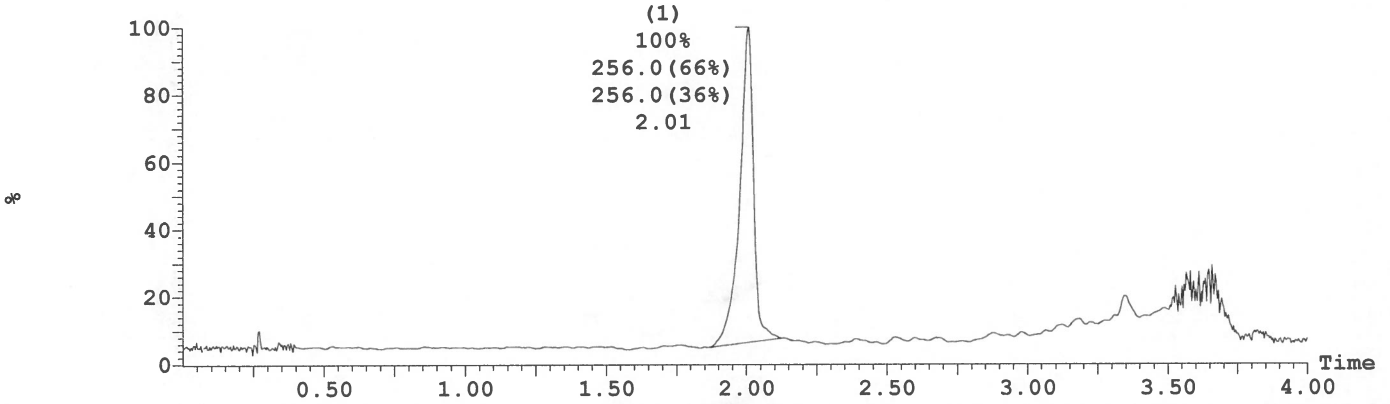

2: MS ES- :TIC Smooth (Mn, 2x2)

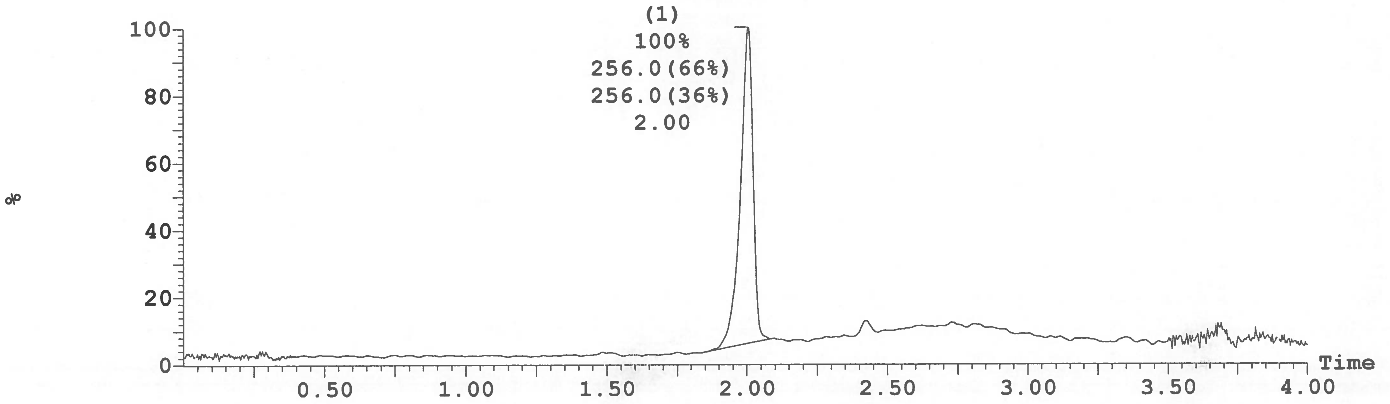

NVR-15NVR-15
NVR-156 LCMS

Sample: 1 | Vial:1:19 | ID:JB175 filtsol-1
File:JB175 filtsol-1 | Date:19-Jun-2019 | Time:18:29:26
Description:

Printed: Thu Jun 20 14:20:48 2019

Peak ID	Compound	Time	Mass Found
1	Found	2.01	257

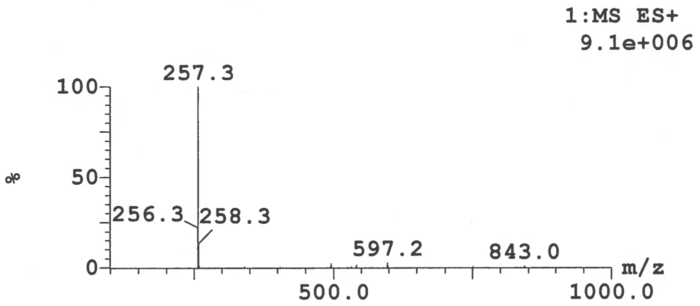

Peak ID	Compound	Time	Mass Found
1	Found	2.01	255

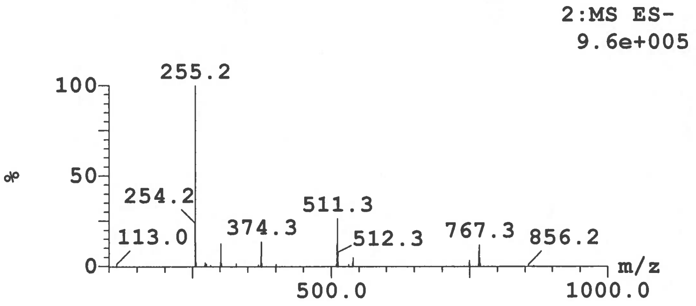

Peak ID	Compound	Time	Mass Found
1		2.01	Not Found

1:(Time: 2.01) Combine (1204) 3:UV Detector
3.017 AU

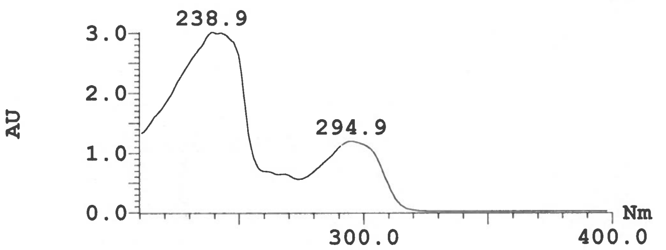

Thermo Exactive Plus EMR Orbitrab HESI pos

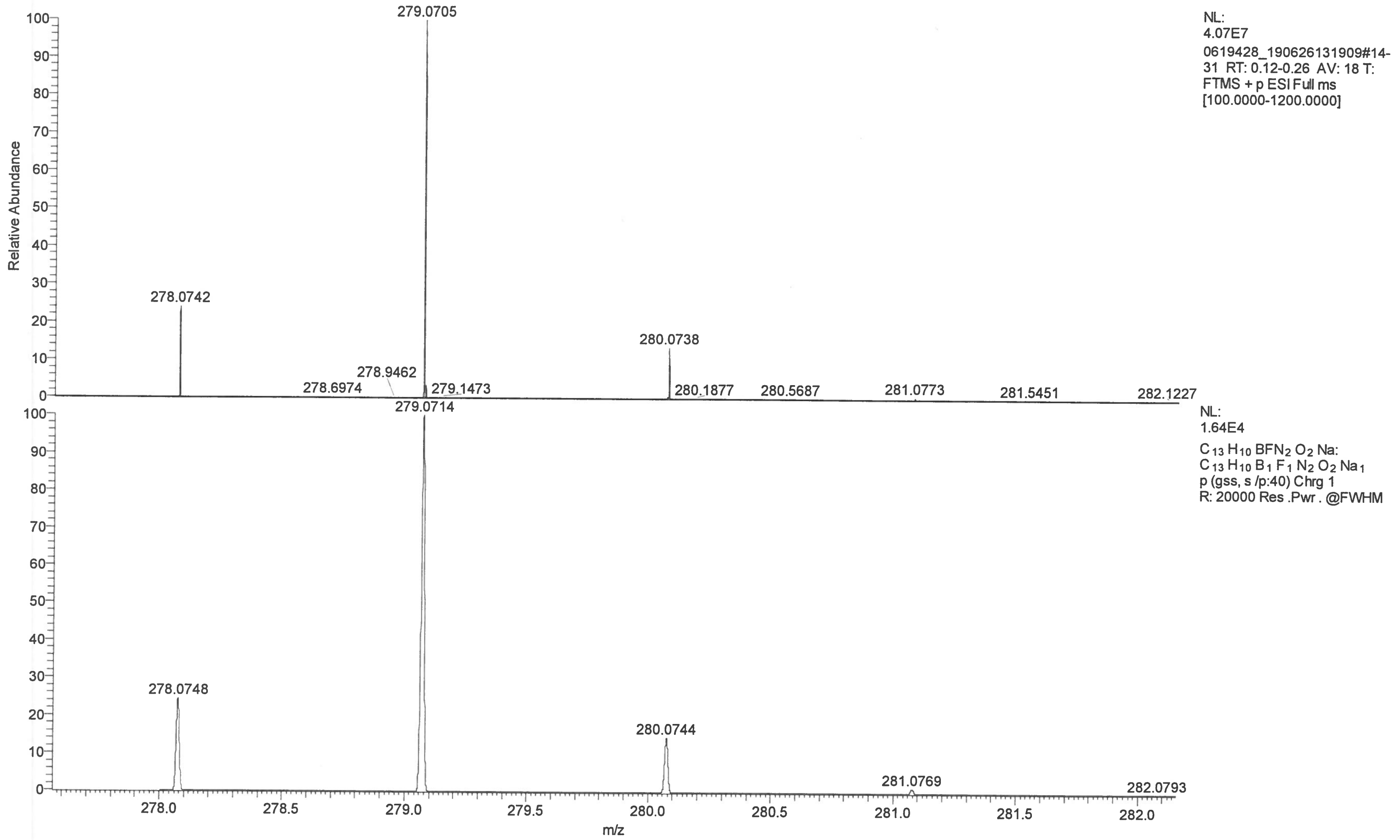

NVR-156 HRMS

Thermo Exactive Plus EMR Orbitrab HESI pos

m/z	Theo. Mass	Delta (ppm)	RDB equiv.	Composition
279.0705	279.0712	-2.36	9.5	C13 H10 O2 N2 B F Na ←
	279.0698	2.46	10.0	C11 H8 O N5 B F Na

NVR-156 HRMS

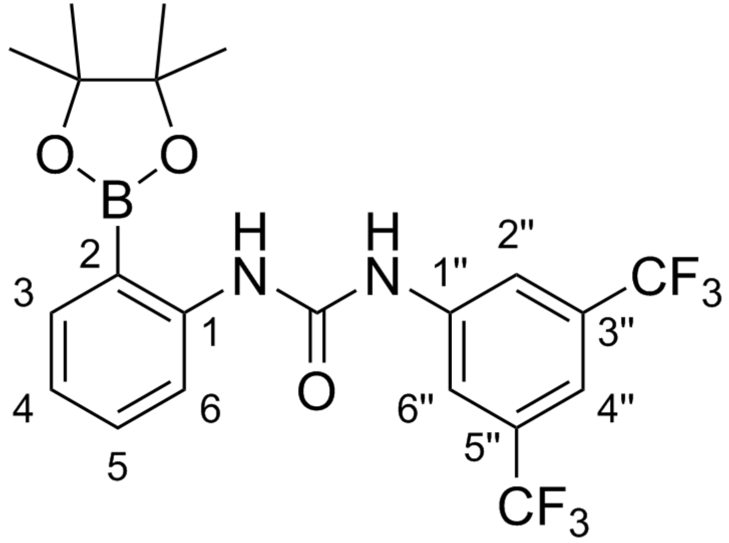

Shift (ppm)	H	m	J (Hz)	Assign
9.96	1	s	-	NH
9.25	1	s	-	NH''
8.15	2	s	-	2'', 6''
7.71	1	s	-	4''
7.51	1	d	7.0	3
7.37	2	m	-	5, 6
7.06	1	td	7.1, 1.4	4
1.23	12	s	-	Me, Me, Me, Me

Acquisition Time (sec)	3.9846
Date	27 Feb 2020 01:53:39
Date Stamp	27 Feb 2020 01:53:39
Frequency (MHz)	400.0700
Nucleus	1H
Number of Transients	16
Solvent	DMSO-d6
Temperature (degree C)	19.625

^{1}H NMR (400 MHz, *DMSO-d_6*) δ ppm 9.96 (s, 1 H), 9.25 (s, 1 H), 8.15 (s, 2 H), 7.71 (s, 1 H), 7.51 (d, *J*=7.0 Hz, 1 H), 7.32 - 7.43 (m, 2 H), 7.06 (td, *J*=7.1, 1.4 Hz, 1 H), 1.23 (s, 12 H)

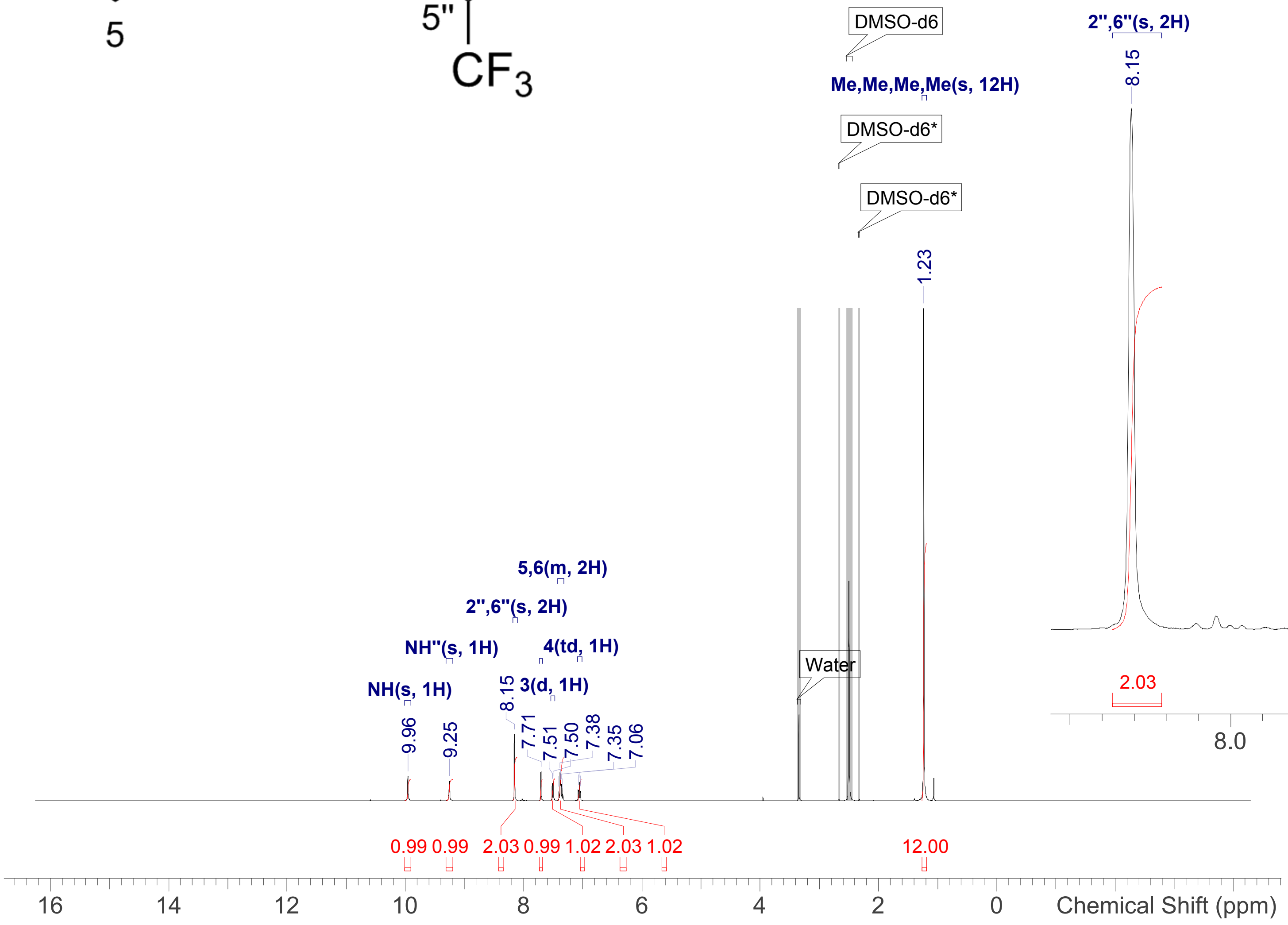

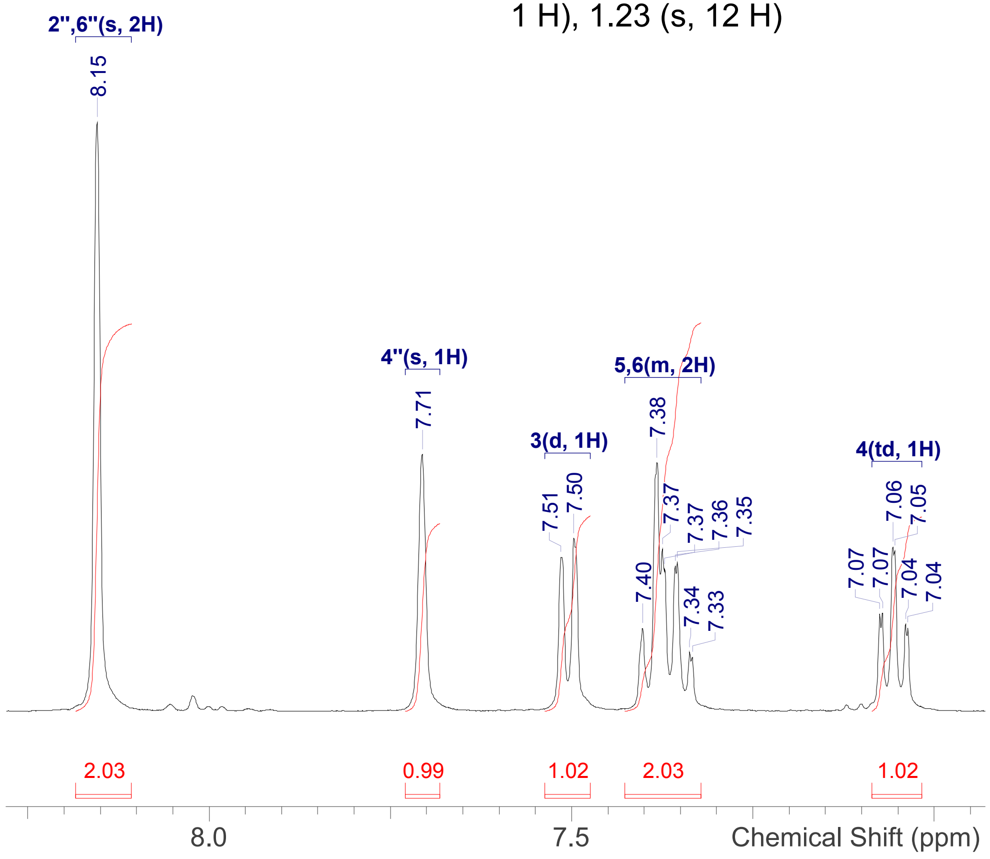

NVR-155_1H.spectrus

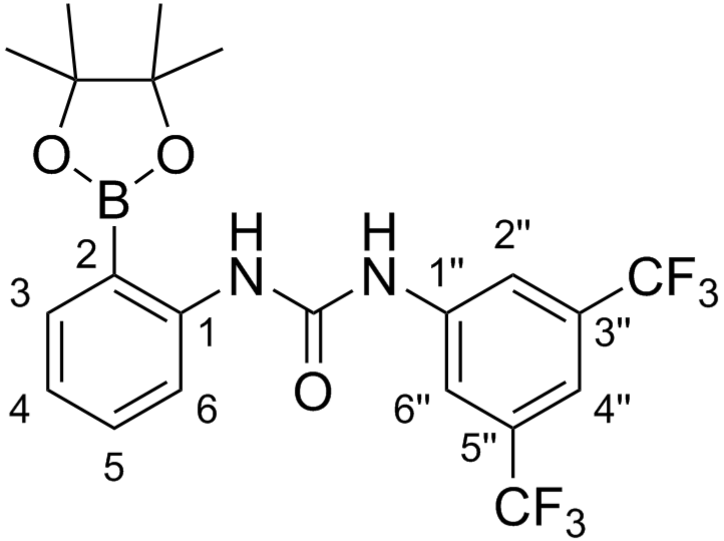

Shift (ppm)	C	m	J (Hz)	Assign
153.6	1	s	-	CO
141.7	1	s	-	1
141.2	1	s	-	1"
134.2	1	s	-	3
130.7	2	q	32.0	5", 3"
130.3	1	s	-	5
123.3	2	q	272.9	5"-CF3, 3"-CF3
122.9	1	s	-	4
119.2	1	s	-	6
119.0	2	br q	3.0	6", 2"
115.1	1	br spt	3.9	4"
82.4	2	s	-	5', 4'
25.0	4	s	-	Me, Me, Me, Me

Acquisition Time (sec)	1.0224
Date	27 Feb 2020 02:08:07
Date Stamp	27 Feb 2020 02:08:07
Frequency (MHz)	100.5977
Nucleus	13C
Number of Transients	256
Solvent	DMSO-d6
Temperature (degree C)	20.511

^{13}C NMR (101 MHz, *DMSO-d_6*) δ ppm 153.6 (s, 1 C), 141.7 (s, 1 C), 141.2 (s, 1 C), 134.2 (s, 1 C), 130.3 (s, 1 C), 130.7 (q, *J*=32.0 Hz, 2 C), 122.9 (s, 1 C), 119.2 (s, 1 C), 119.0 (br q, *J*=3.0 Hz, 2 C), 123.3 (q, *J*=272.9 Hz, 2 C), 115.1 (br spt, *J*=3.9 Hz, 1 C), 82.4 (s, 2 C), 25.0 (s, 4 C)

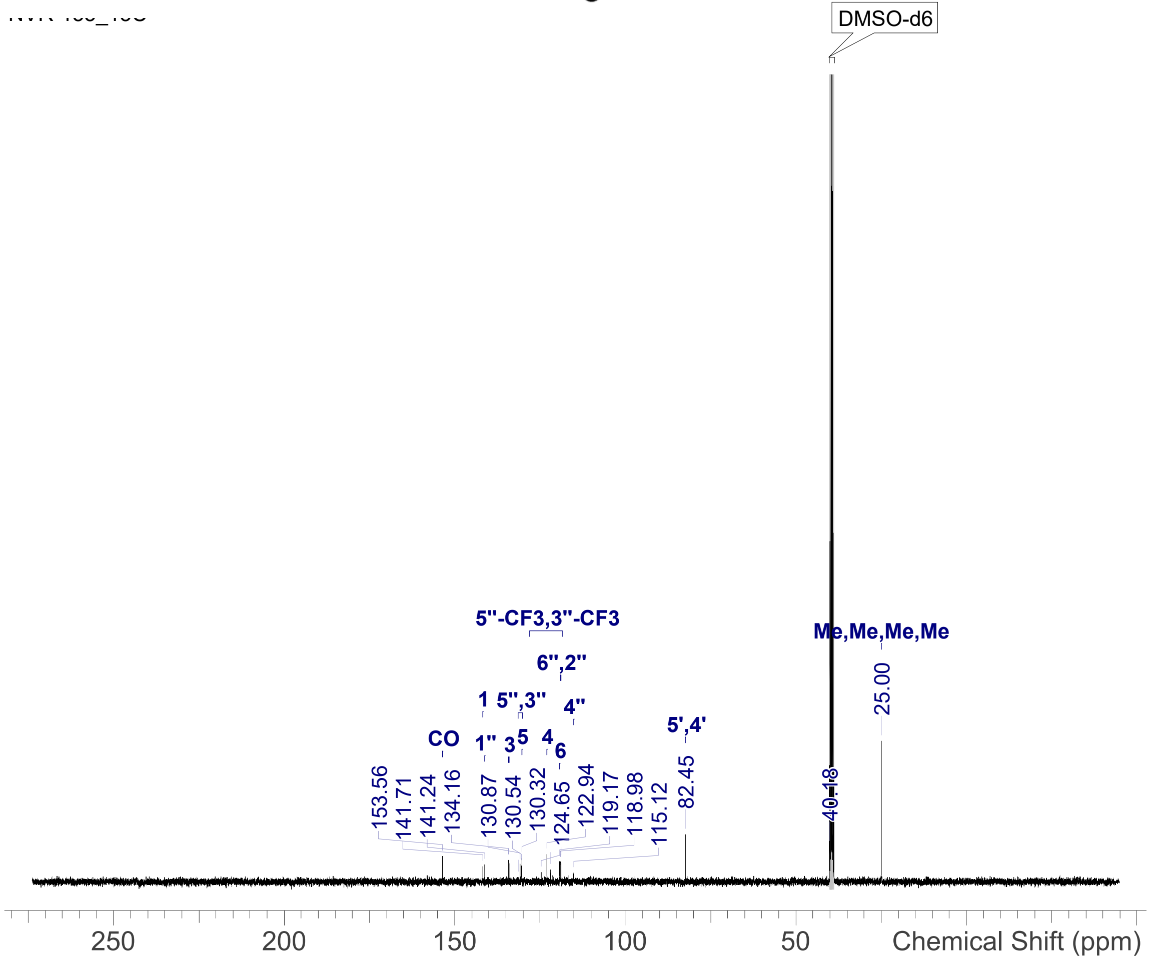

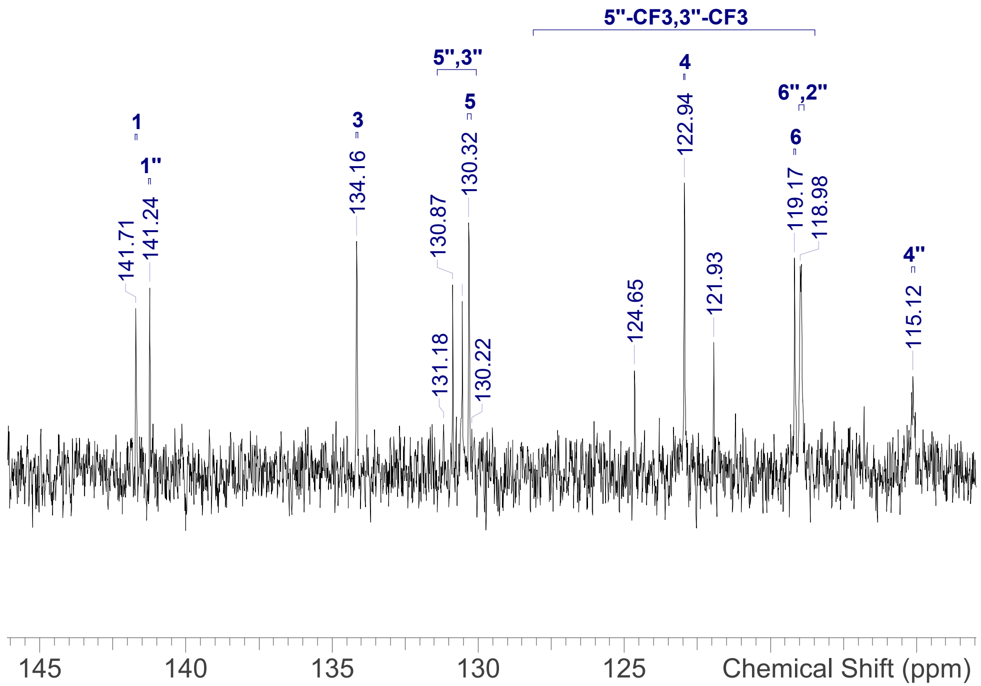

NVR-155_13C.spectrus

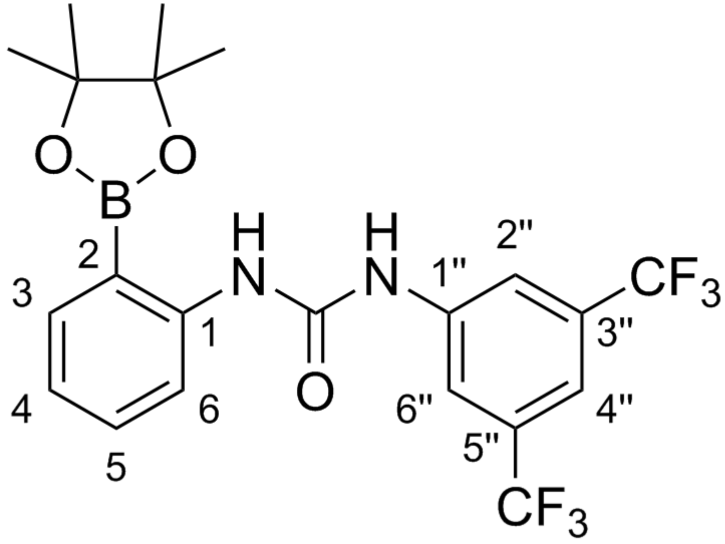

Shift (ppm)	F	m
-61.65	6	s

Acquisition Time (sec)	1.4680
Date	27 Feb 2020 01:51:09
Date Stamp	27 Feb 2020 01:51:09
Frequency (MHz)	376.4419
Nucleus	19F
Number of Transients	128
Solvent	DMSO-d6
Temperature (degree C)	19.639

^{19}F NMR (376 MHz, *DMSO-d_6*) δ ppm -61.65 (s, 6 F)

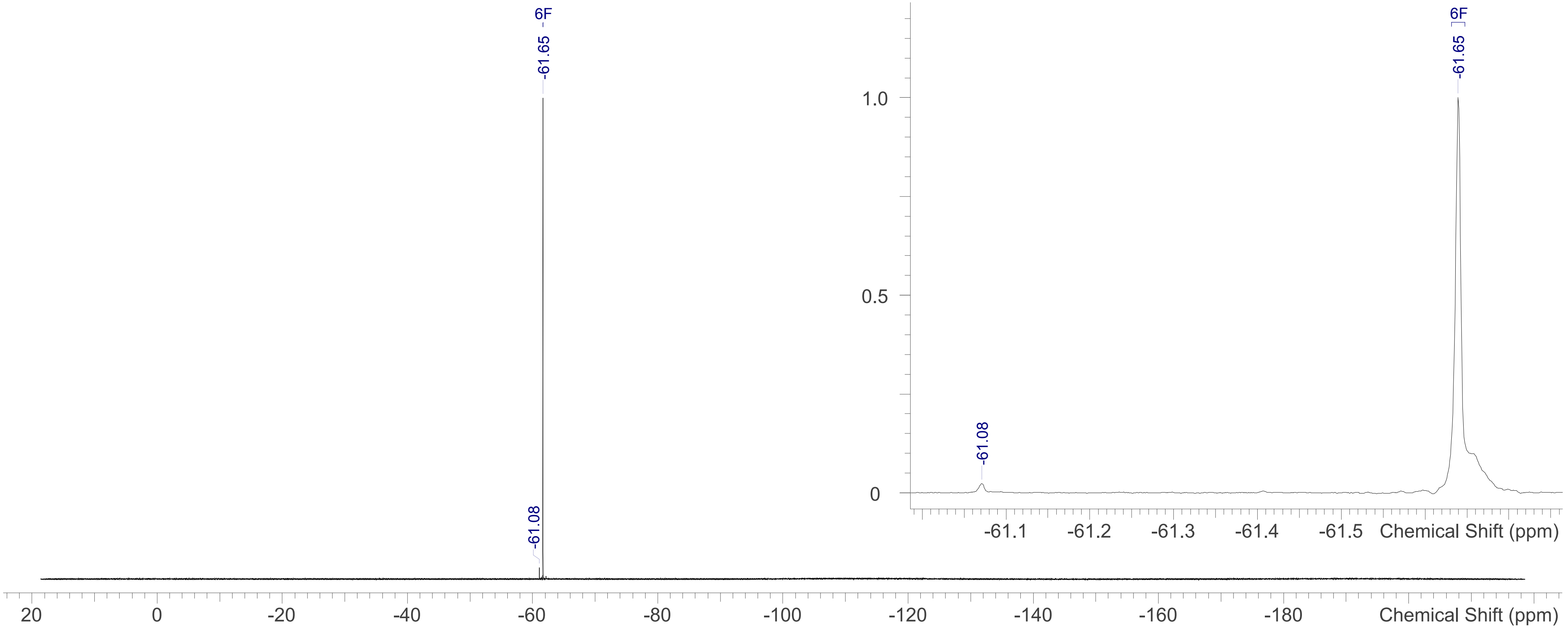

NVR-155_19F.spectrus

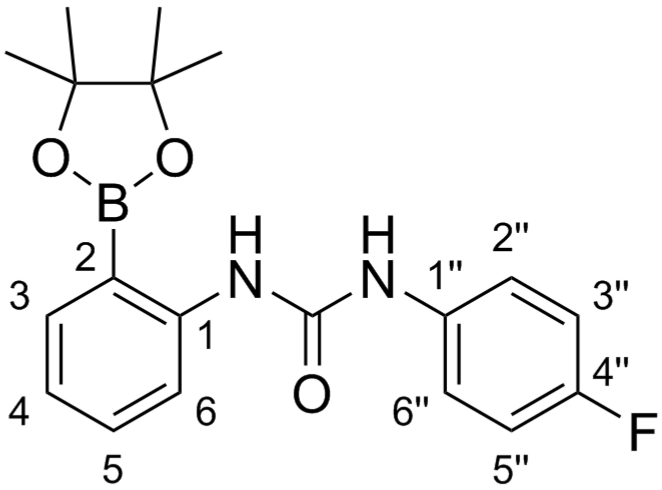

Shift (ppm)	H	m	J (Hz)	Assign
9.36	1	br s	-	NH''
9.35	1	br s	-	NH
7.44	3	m	-	6, 2'', 6''
7.26	1	m	-	3
7.23	1	m	-	5
7.18	2	br t	8.9	3'', 5''
7.00	1	td	7.1, 1.4	4
1.19	12	s	-	Me, Me, Me, Me

Acquisition Time (sec)	3.9846
Date	25 Feb 2020 13:44:41
Date Stamp	25 Feb 2020 13:44:41
Frequency (MHz)	400.0700
Nucleus	1H
Number of Transients	4
Solvent	DMSO-d6
Temperature (degree C)	25.004

^{1}H NMR (400 MHz, *DMSO-d*$_6$) δ ppm 9.36 (br s, 1 H), 9.35 (br s, 1 H), 7.37 - 7.50 (m, 3 H), 7.24 - 7.30 (m, 1 H), 7.21 - 7.24 (m, 1 H), 7.18 (br t, *J*=8.9 Hz, 2 H), 7.00 (td, *J*=7.1, 1.4 Hz, 1 H), 1.19 (s, 12 H)

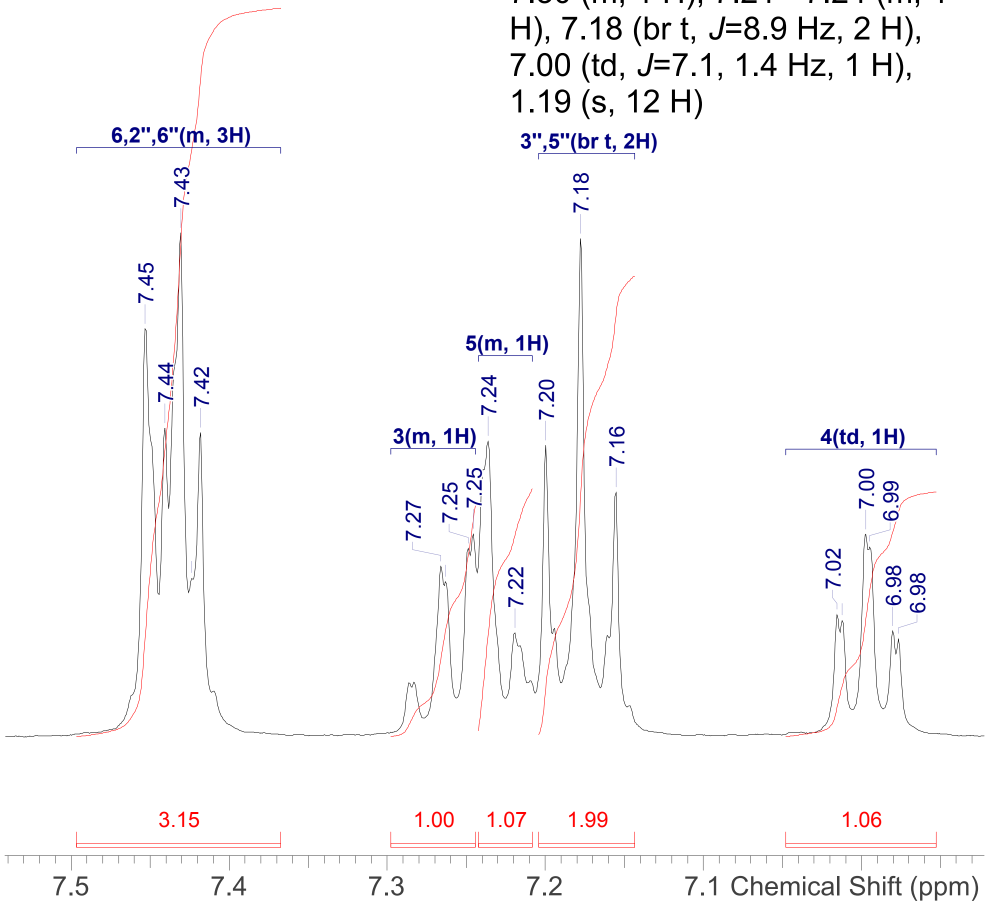

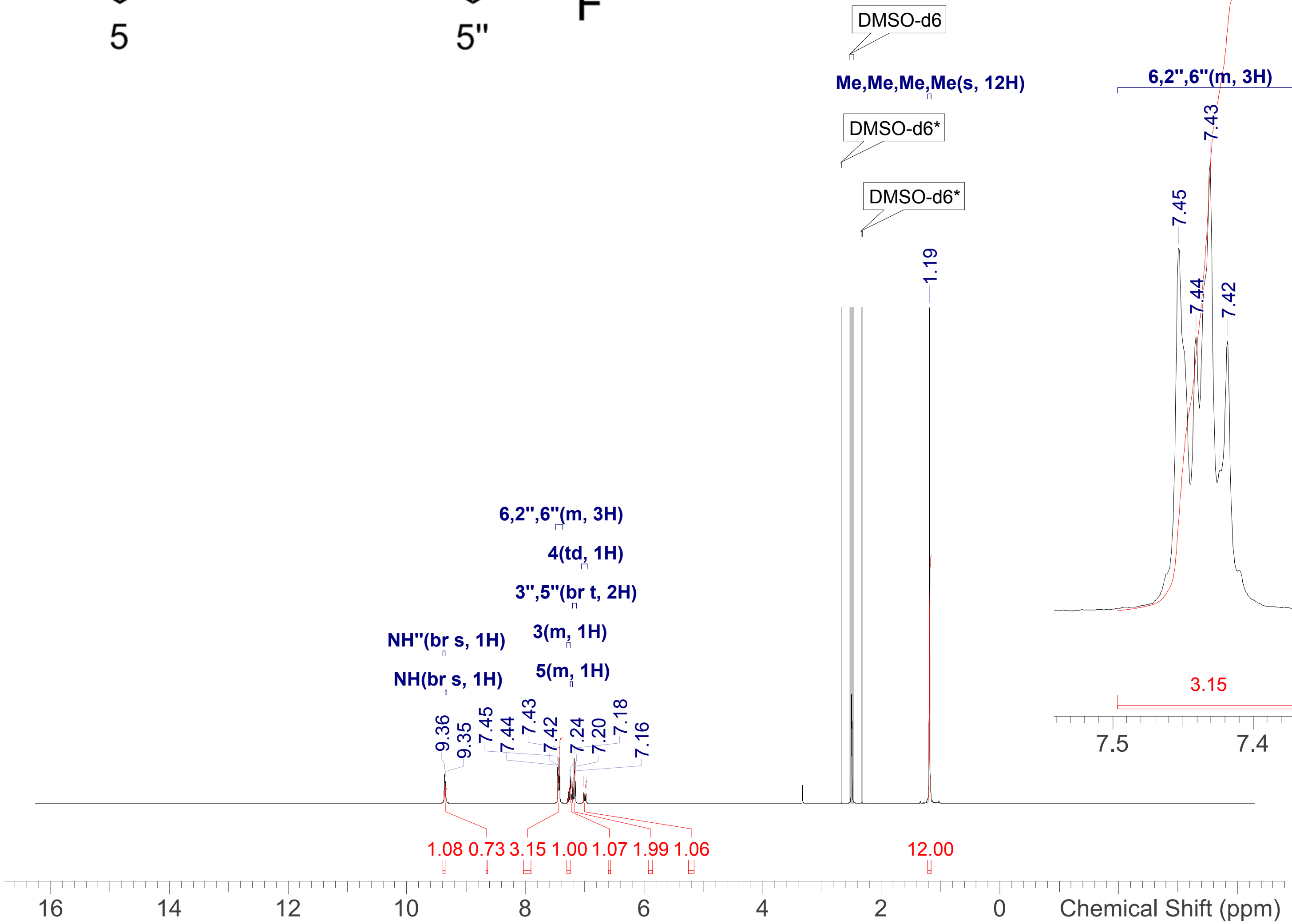

NVR-157_1H.spectrus

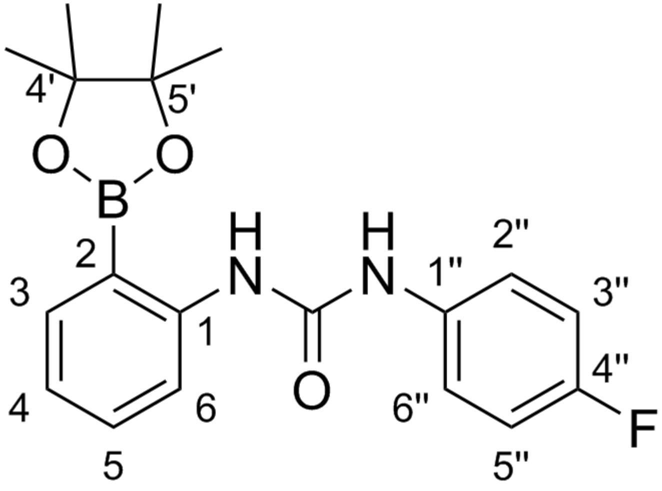

Shift (ppm)	C	m	J (Hz)	Assign
158.4	1	d	239.6	4"
154.2	1	s	-	7"
141.2	1	s	-	1
134.2	1	d	2.0	1"
133.4	1	s	-	6
129.1	1	s	-	3
122.6	1	s	-	4
122.5	2	br d	3.9	2", 6"
117.1	1	s	-	5
115.4	2	d	22.5	3", 5"
81.2	2	s	-	4', 5'
25.4	4	s	-	Me, Me, Me, Me

Acquisition Time (sec)	1.0224
Date	25 Feb 2020 18:18:10
Date Stamp	25 Feb 2020 18:18:10
Frequency (MHz)	100.5977
Nucleus	13C
Number of Transients	256
Solvent	DMSO-d6
Temperature (degree C)	25.008

^{13}C NMR (101 MHz, *DMSO-d$_6$*) δ ppm 158.4 (d, *J*=239.6 Hz, 1 C), 154.2 (s, 1 C), 141.2 (s, 1 C), 134.2 (d, *J*=2.0 Hz, 1 C), 133.4 (s, 1 C), 129.1 (s, 1 C), 122.6 (s, 1 C), 122.5 (br d, *J*=3.9 Hz, 2 C), 117.1 (s, 1 C), 115.4 (d, *J*=22.5 Hz, 2 C), 81.2 (s, 2 C), 25.4 (s, 4 C)

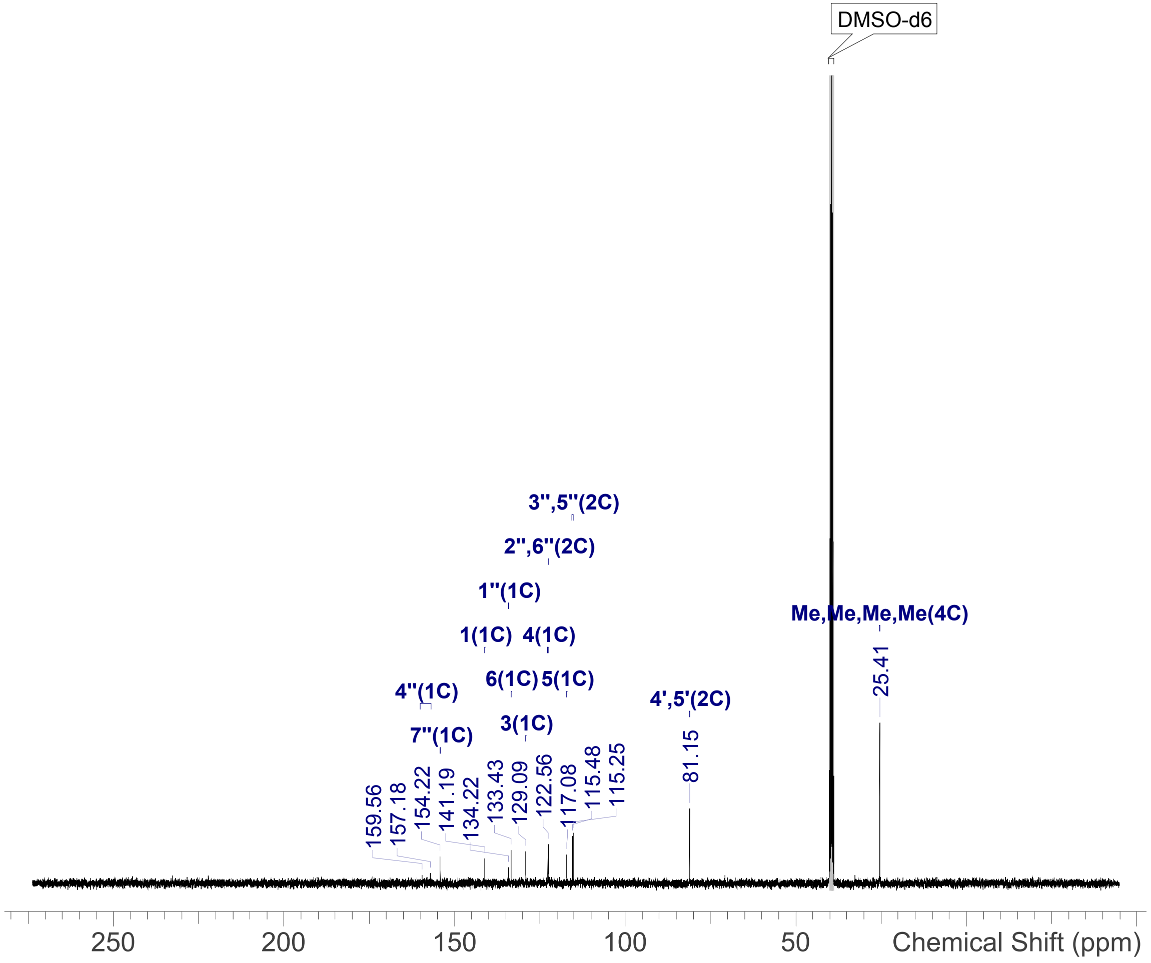

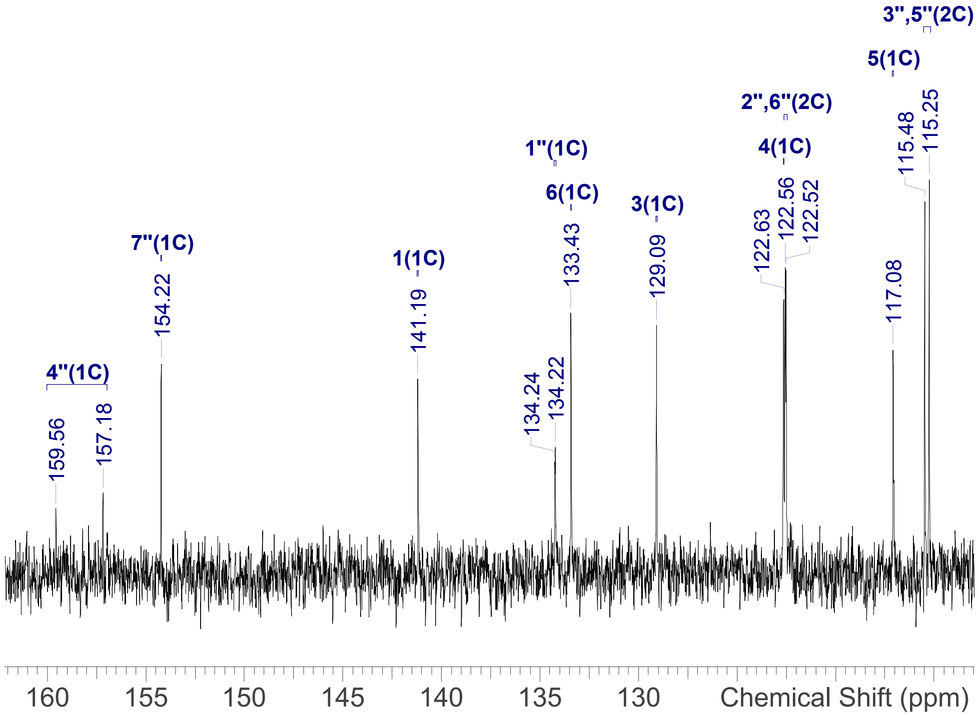

NVR-157_13C.spectrus

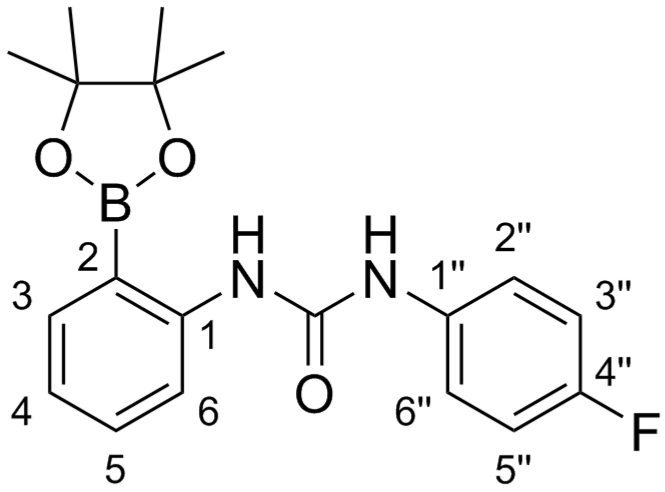

Shift (ppm)	F	m
-119.43	1	br s

Acquisition Time (sec)	1.4680
Date	25 Feb 2020 18:01:20
Date Stamp	25 Feb 2020 18:01:20
Frequency (MHz)	376.4419
Nucleus	19F
Number of Transients	16
Solvent	DMSO-d6
Temperature (degree C)	25.003

^{19}F NMR (376 MHz, *DMSO-d_6*) δ ppm -119.43 (br s, 1 F)

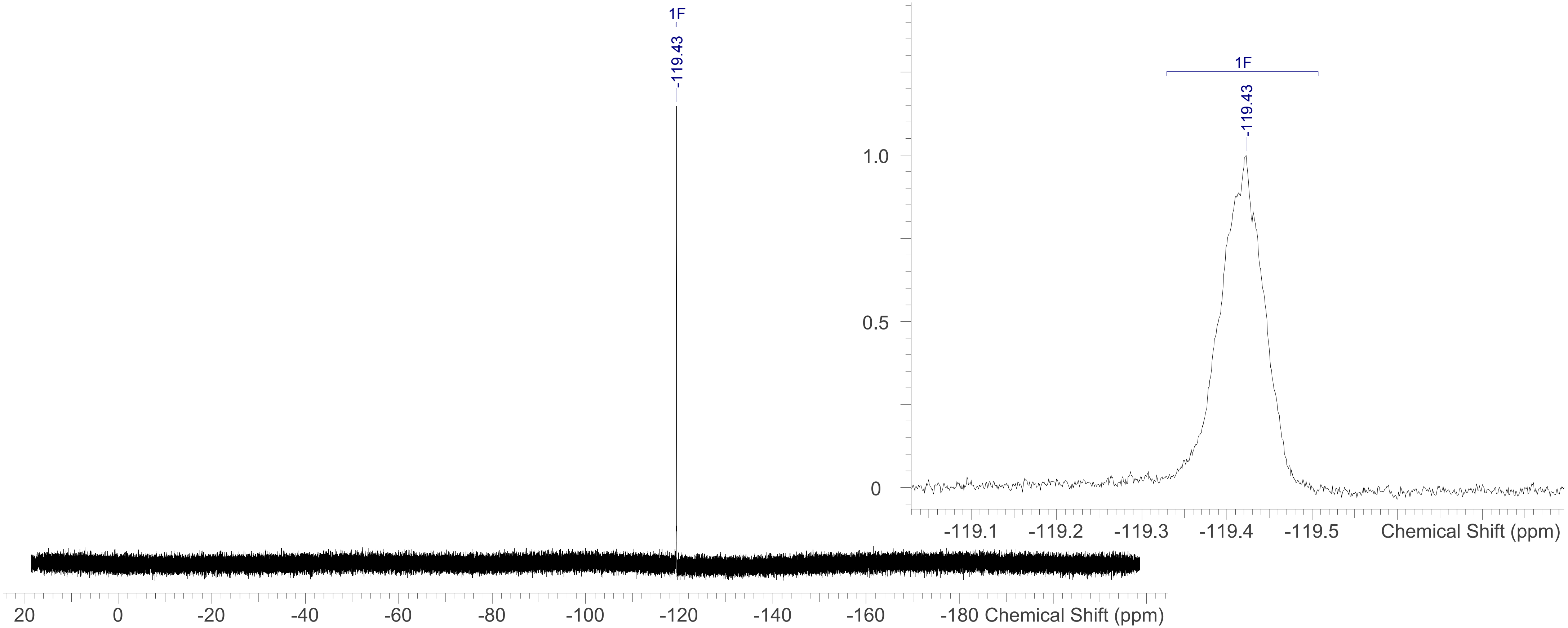

NVR-157_19F.spectrus

Openlynx Report - James B Page 1

Sample: 1	Vial:1:20	ID:JB176 filtsol-1
File:JB176 filtsol-1	Date:19-Jun-2019	Time:18:34:41
Description:		

Printed: Thu Jun 20 14:20:56 2019

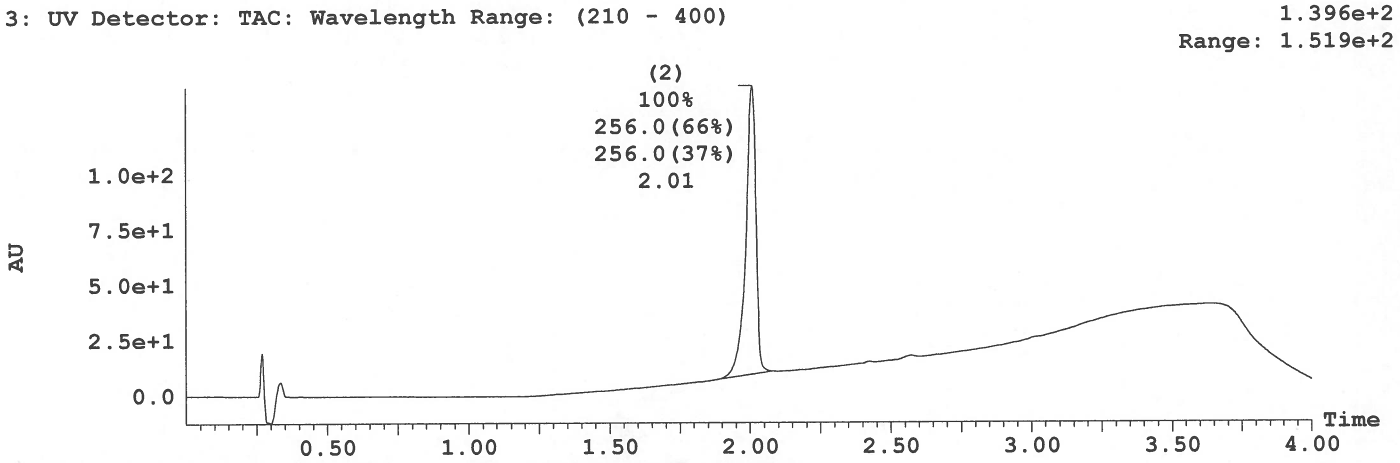

Peak Number	Compound	Time	Area %Total	Mass Found
2	Found	2.01	100.00	256.0, 256.0

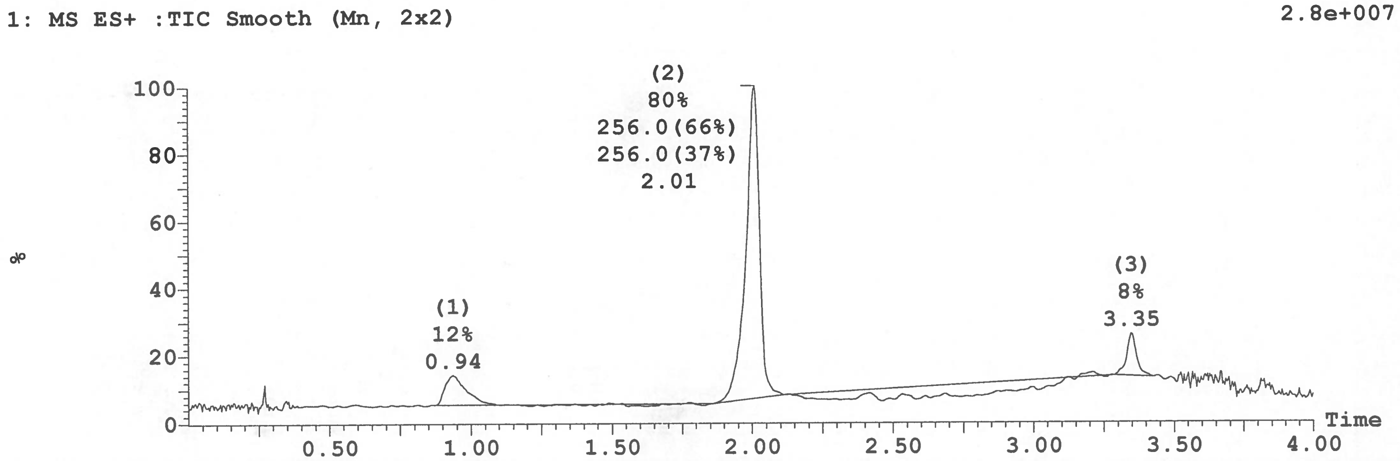

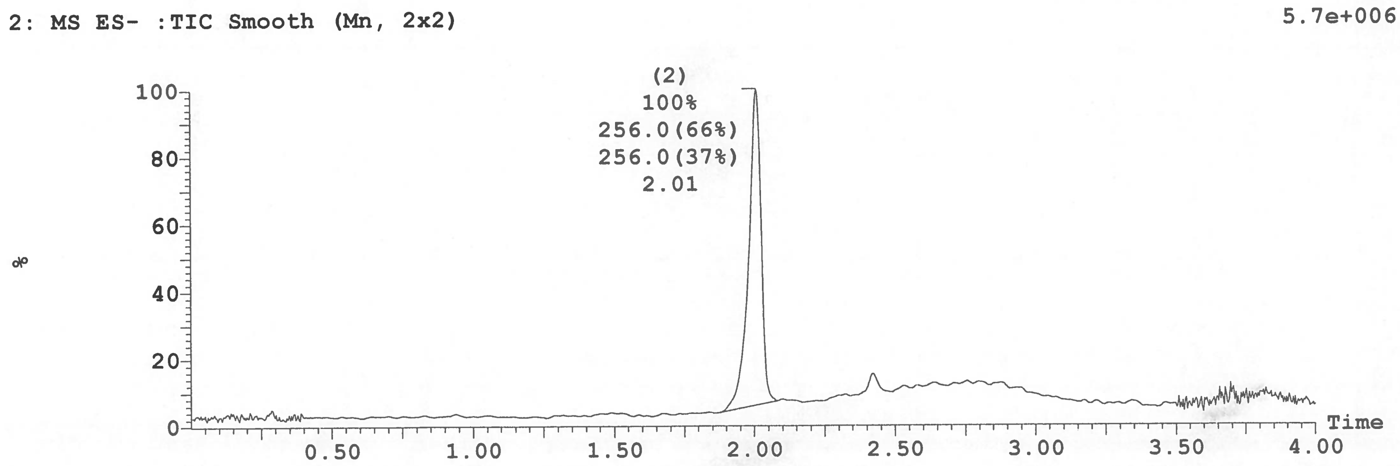

NVR-157 LCMS

Openlynx Report - James B
Sample: 1 Vial:1:20 ID:JB176 filtsol-1
File:JB176 filtsol-1 Date:19-Jun-2019 Time:18:34:41
Description:

Printed: Thu Jun 20 14:20:56 2019

Peak ID	Compound	Time	Mass Found
2	Found	2.01	257

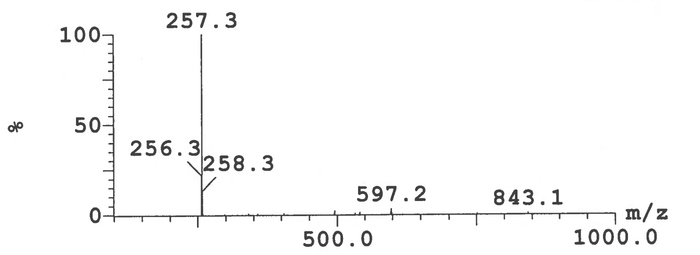

Peak ID	Compound	Time	Mass Found
2	Found	2.01	255

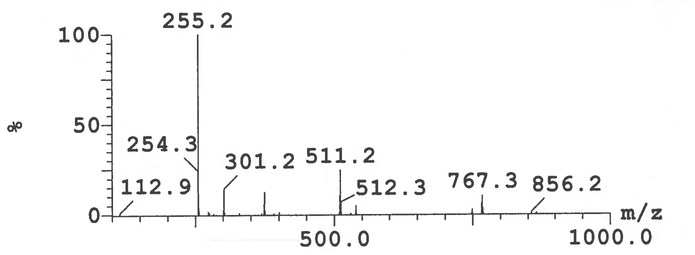

Peak ID	Compound	Time	Mass Found
2		2.01	Not Found

2: (Time: 2.01) Combine (1206) 3:UV Detector 2.88 AU

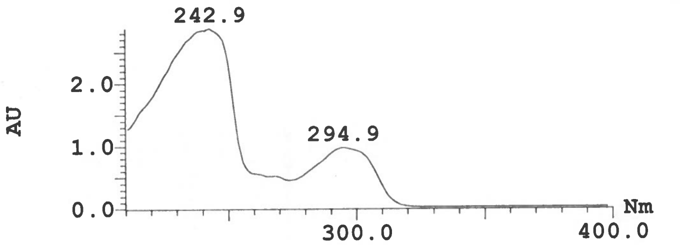

NVR-157 LCMS

Thermo Exactive Plus EMR Orbitrab HESI pos

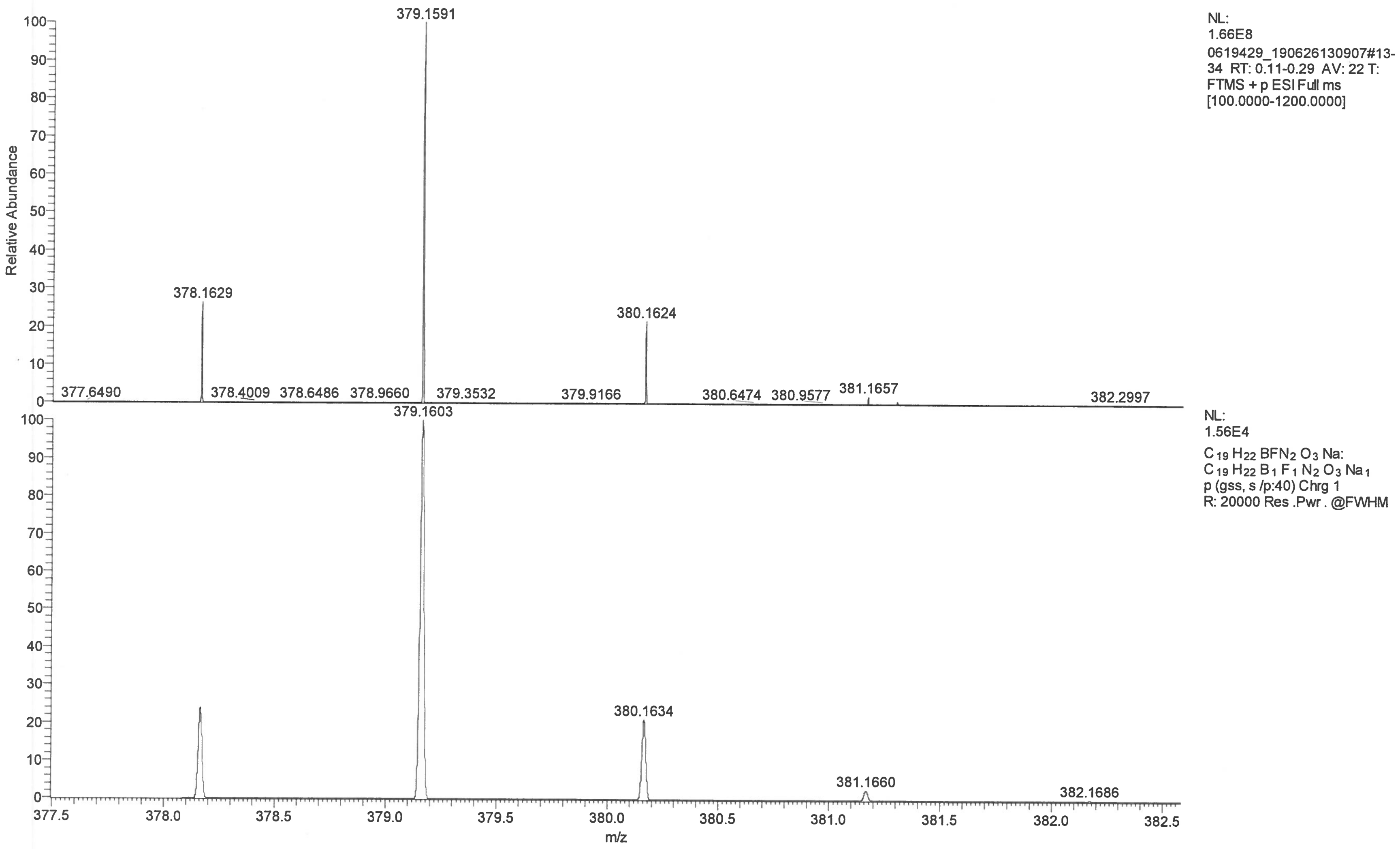

NVR-157 HRMS

Thermo Exactive Plus EMR Orbitrab HESI pos

m/z	Theo. Mass	Delta (ppm)	RDB equiv.	Composition
379.1591	379.1586	1.24	10.0	C17 H20 O2 N5 B F Na
	379.1600	-2.30	9.5	C19 H22 O3 N2 B F Na ←
	379.1573	4.77	5.0	C16 H24 O6 N B F Na
	379.1573	4.78	10.5	C15 H18 O N8 B F Na

NVR-157 HRMS
