# Supplementary material for: Selective inhibition of the K+ efflux sensitive NLRP3 pathway by Cl− channel modulation
Source: Chem Sci. 2020 Oct 12;11(43):11720–8. doi: 10.1039/d0sc03828h (PMC8162947; doi:10.1039/d0sc03828h)
Supplement: SC-011-D0SC03828H-s002 [file SC-011-D0SC03828H-s002.pdf]

Electronic Supplementary Material (ESI) for Chemical Science.
This journal is © The Royal Society of Chemistry 2020

Supplementary information

Selective inhibition of the K^+ efflux sensitive NLRP3 pathway by Cl^- channel modulation

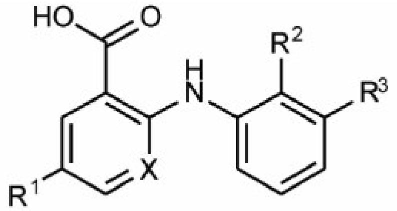

Entry	X	R^1	R^2	R^3	$\%_{inh}$
NVR-48	CH	H	H	H	13
FFA	CH	H	H	CF_3	-1
NFA	N	H	H	CF_3	29
NVR-1	N	H	H	CN	5
TFA	CH	H	Me	Cl	22
Clonixin	N	H	Me	Cl	8
NVR-3	CH	NO_2	H	CF_3	19
NVR-4	CH	CF_3	H	CF_3	2

Supplementary Figure 1: Structures of the fenamate NSAIDs Flufenamic acid (FFA), Niflumic acid (NFA), Tolfenamic acid (TFA) and Clonixin, and synthesised amine (fenamate) analogues, and their percentage inhibition of IL-1β release. LPS-primed (1 μg ml^{-1}, 4h) bone marrow-derived macrophages (BMDMs) were pre-treated with indicated drugs (10 μM, 15 min) or vehicle (DMSO, 0.5%) prior to ATP stimulation (5mM, 1h). Supernatants were harvested and IL-1β concentration was assessed by ELISA. Data are presented as mean percentage inhibition of IL-1β release vs. vehicle (*n*= *2-6*).

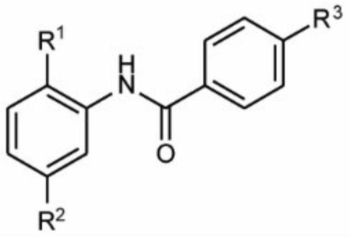

Entry	R^1	R^2	R^3	$\%_{inh}$
NVR-17	COOH	H	H	-4
NVR-15	Tet	H	H	58
NVR-29	Tet	H	OMe	15
NVR-16	Tet	Cl	H	55

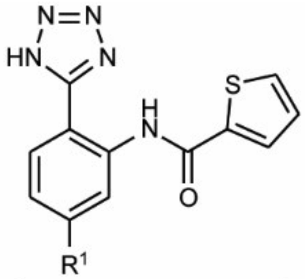

Entry	R^1	$\%_{inh}$
NVR-19	H	-2
NVR-20	Cl	21

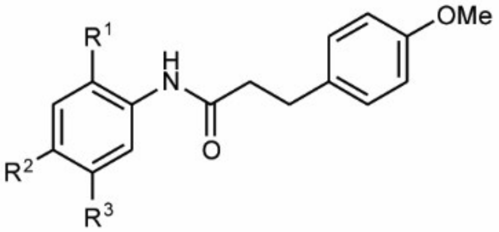

Entry	R^1	R^2	R^3	$\%_{inh}$
NVR-31	COOH	I	H	35
NVR-23	Tet	H	H	22
NVR-21	Tet	H	Cl	15
NVR-30	Tet	Br	H	50

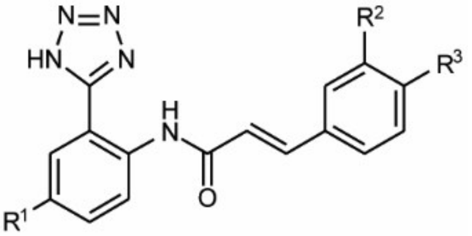

Entry	R^1	R^2	R^3	$\%_{inh}$
NVR-25	H	H	H	-2
NVR-38	H	H	OMe	0
NVR-27	H	OMe	OMe	12
NVR-32	Br	OMe	OMe	45

Supplementary Figure 2: Structures of synthesised amide analogues and their percentage inhibition of IL-1β release. LPS-primed (1 μg ml^{-1}, 4h) bone marrow-derived macrophages (BMDMs) were incubated with indicated drugs (10 μM, 15 min) or vehicle (DMSO, 0.5%) before stimulation with ATP (5mM, 1h). Supernatant IL-1β concentration was assessed by ELISA. Data are presented as mean percentage inhibition of IL-1β release vs. vehicle (n= 5). Tet = 1H-tetrazol-5-yl.

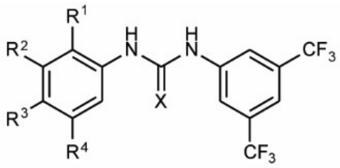

Entry	R1	R2	R3	R4	X	$\%_{inh}$
NVR-7	COOH	H	H	H	O	55
NVR-5	COOH	H	H	Cl	O	96
NVR-121	COOH	H	H	Me	O	83
NVR-120	COOH	H	H	F	O	79
NVR-123	COOH	H	H	OMe	O	87
NVR-119	COOH	H	Cl	H	O	97
NVR-122	COOH	H	Me	H	O	92
NVR-9	COOH	H	F	H	O	82
NVR-124	COOH	H	OMe	H	O	87
NVR-12	COOH	H	I	H	O	91
NVR-132	COOH	H	Cl	Cl	O	87
NVR-149	COOH	H	F	F	O	98
NVR-150	COOH	H	OMe	OMe	O	87
NVR-118	H	COOH	H	H	O	63
NVR-117	H	H	COOH	H	O	53
NVR-58	Tet	H	H	Cl	O	96
NVR-133	Tet	H	H	CF_3	O	90
NVR-41	Tet	H	Cl	H	O	94
NVR-42	Tet	H	F	H	O	87
NS3728	Tet	H	Br	H	O	86
NVR-40	Tet	H	t-Bu	H	O	88
NVR-131	Tet	H	Cl	F	O	68
NVR-135	H	Tet	H	CF_3	O	77
NVR-139	H	Tet	H	Br	O	49
NVR-134	Tet	H	H	CF_3	S	83
NVR-105	Tet	CF_3	H	CF_3	S	2
NVR-101	H	Tet	H	CF_3	S	81
NVR-96	H	CF_3	Tet	H	S	51
NVR-141	H	$B(OH)_2$	H	H	S	84

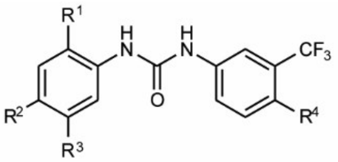

Entry	R1	R2	R3	R4	$\%_{inh}$
NVR-13	COOH	H	H	Cl	50
NVR-6	COOH	H	Cl	Cl	90
NVR-158	COOH	H	t-Bu	Cl	77
NVR-10	COOH	F	H	Cl	45
NVR-162	COOH	Cl	H	H	87
NVR-11	COOH	I	H	Cl	52
NVR-43	Tet	H	Cl	Cl	91
NVR-44	Tet	Br	H	Cl	91

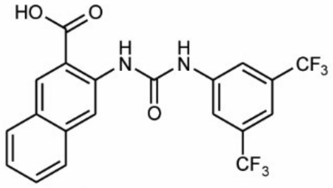

Entry	$\%_{inh}$
NVR-148	99

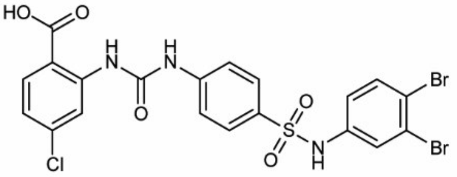

Entry	$\%_{inh}$
NVR-47	71

Supplementary Figure 3: Structures of synthesised urea analogues and their percentage inhibition of IL-1β release. LPS-primed (1 μg ml^{-1}, 4h) bone marrow-derived macrophages (BMDMs) were incubated with indicated drugs (10 μM, 15 min) or vehicle (DMSO, 0.5%) before stimulation with ATP (5mM, 1h). Supernatant IL-1β concentration was assessed by ELISA. Data are presented as mean percentage inhibition of IL-1β release vs. vehicle (*n*= *2-9*). Tet = 1H-tetrazol-5-yl

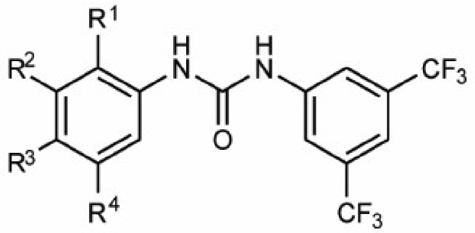

Entry	R^1	R^2	R^3	R^4	X	$\%_{inh}$
NVR-113	Ph	H	H	H	O	-26
NVR-114	Morph.	H	H	H	O	41
NVR-115	Py.	H	H	H	O	10
NVR-116	Benzimid.	H	H	H	O	18
NVR-35	H	H	H	H	O	58
NVR-36	H	H	Br	H	O	42
NVR-142	H	Br	Br	H	O	17
NVR-140	H	Br	H	CN	O	42
NVR-111	H	CF_3	H	CF_3	O	-1
NVR-136	H	CF_3	H	CN	O	70
NVR-137	H	CF_3	H	CO_2Me	O	30
NVR-143	H	Cl	F	Cl	O	37
NVR-97	H	Br	Br	H	S	81
NVR-104	H	Br	H	CN	S	63
NVR-72	H	CF_3	H	CF_3	S	80
NVR-138	H	CF_3	H	CO_2Me	S	69
NVR-144	H	Cl	F	Cl	S	77

Supplementary Figure 4: Structures of synthesised ‘acidless’ urea analogues and their percentage inhibition of IL-1β release. LPS-primed (1 µg ml^{-1}, 4h) bone marrow-derived macrophages (BMDMs) were incubated with indicated drugs (10 µM, 15 min) or vehicle (DMSO, 0.5%) before stimulation with ATP (5mM, 1h). Supernatant IL-1β concentration was assessed by ELISA. Data are presented as mean percentage inhibition of IL-1β release vs. vehicle (*n*= *3-7*). Ph = Phenyl; Morph. = 2-morpholino; Py. = 1H-pyrrol-1-yl; Benzimid. = 1H-benzo[d]imidazol-2-yl.

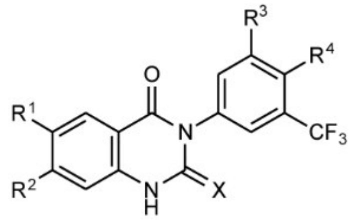

Entry	R^1	R^2	R^3	R^4	X	$\%_{inh}$
NVR-66	H	Cl	CF_3	H	O	13
NVR-91	H	Cl	H	Cl	O	-28
NVR-92	H	Cl	H	H	O	11
NVR-93	H	OMe	H	Cl	O	-6
NVR-125	F	H	CF_3	H	O	13
NVR-159	Cl	H	H	H	S	59

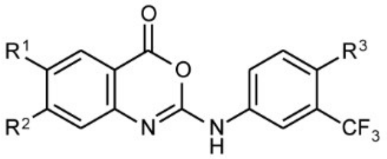

Entry	R^1	R^2	R^3	$\%_{inh}$
NVR-65	H	OMe	Cl	4
NVR-68	H	^{t}Bu	Cl	-14
NVR-160	Cl	H	H	23

Supplementary Figure 5: Structures of synthesised cyclised quinazoline-2,4(1H,3H)-dione (left) and 4H-benzo[d][1,3]oxazin-4-one (right) analogues and their percentage inhibition of IL-1β release. LPS-primed (1 μg ml^{-1}, 4h) bone marrow-derived macrophages (BMDMs) were incubated with indicated drugs (10 μM, 15 min) or vehicle (DMSO, 0.5%) before stimulation with ATP (5mM, 1h). Supernatant IL-1β concentration was assessed by ELISA. Data are presented as mean percentage inhibition of IL-1β release vs. vehicle (*n*= *3-8*).

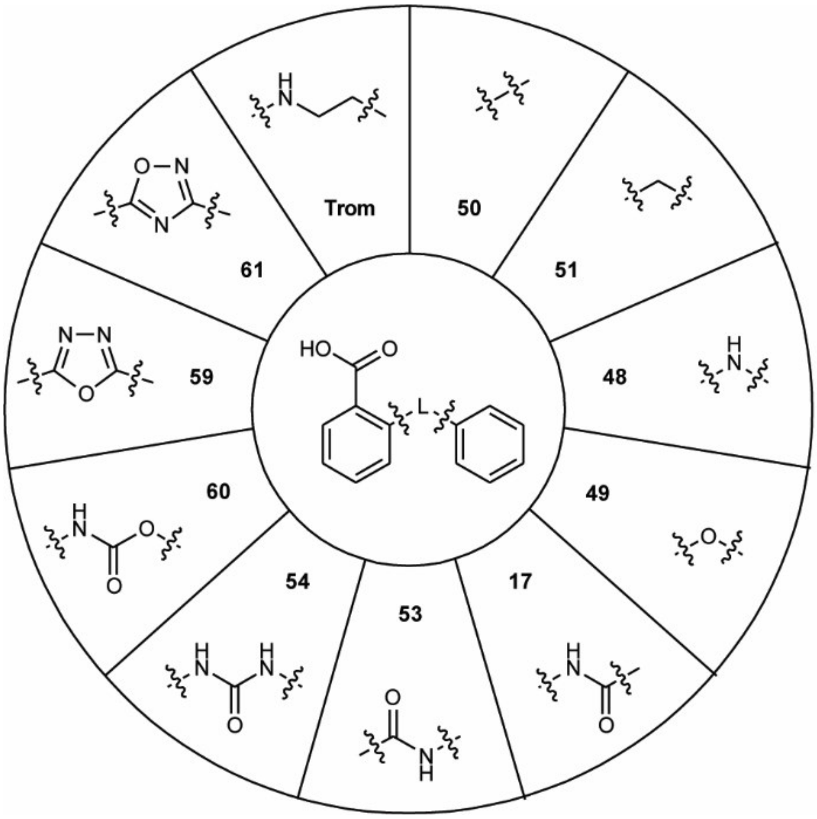

Entry	$\%_{inh}$
Tromaril	-55
NVR-50	2
NVR-51	-21
NVR-48	13
NVR-49	-26
NVR-17	-4
NVR-53	-19
NVR-54	-6
NVR-60	10
NVR-59	-1
NVR-61	7

Supplementary Figure 6: Structures of synthesised analogues with varied linkers, and their percentage inhibition of IL-1β release. LPS-primed (1 μg ml^{-1}, 4h) bone marrow-derived macrophages (BMDMs) were incubated with indicated drugs (10 μM, 15 min) or vehicle (DMSO, 0.5%) before stimulation with ATP (5mM, 1h). Supernatant IL-1β concentration was assessed by ELISA. Data are presented as mean percentage inhibition of IL-1β release vs. vehicle (*n*= *2-5*).

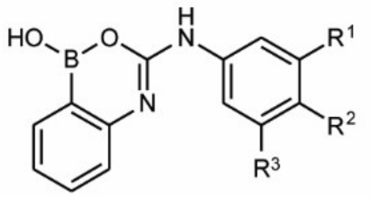

Entry	R^1	R^2	R^3	$\%_{inh}$
NVR-154	CF_3	H	CF_3	37
NVR-156	H	F	H	12

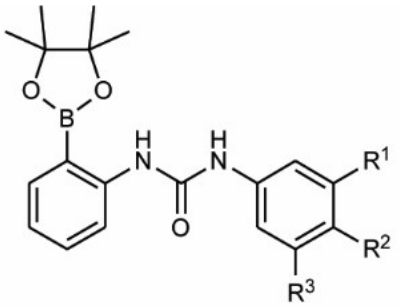

Entry	R^1	R^2	R^3	$\%_{inh}$
NVR-155	CF_3	H	CF_3	23
NVR-157	H	F	H	23

Supplementary Figure 7: Structures of synthesised boron containing analogues and their percentage inhibition of IL-1β release. LPS-primed (1 µg ml^{-1}, 4h) bone marrow-derived macrophages (BMDMs) were incubated with indicated drugs (10 µM, 15 min) or vehicle (DMSO, 0.5%) before stimulation with ATP (5mM, 1h). Supernatant IL-1β concentration was assessed by ELISA. Data are presented as mean percentage inhibition of IL-1β release vs. vehicle (*n=3*).

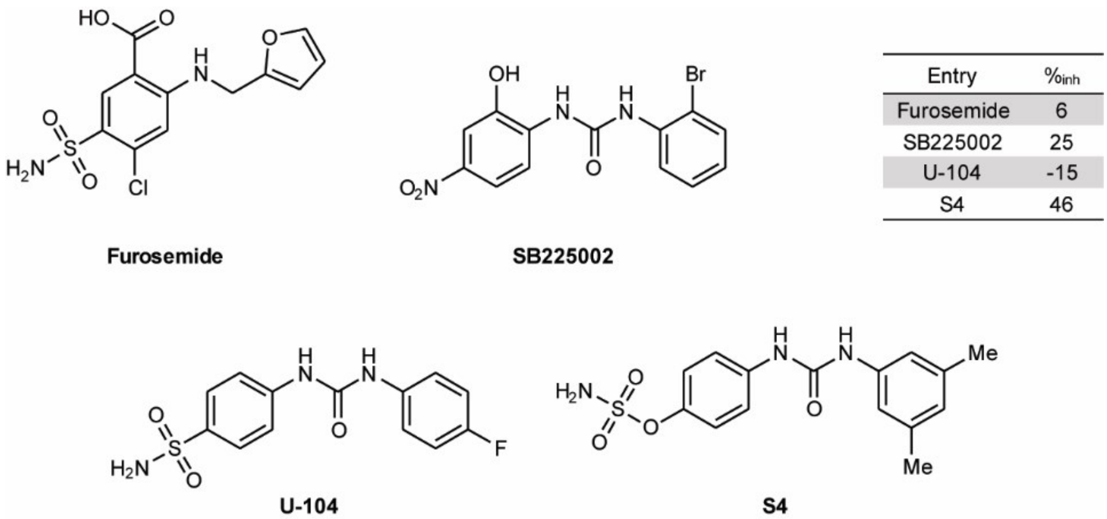

Entry	$\%_{inh}$
Furosemide	6
SB225002	25
U-104	-15
S4	46

Supplementary Figure 8: Commercial inhibitors and their percentage inhibition of IL-1β release. LPS-primed (1 μg ml^{-1}, 4h) bone marrow-derived macrophages (BMDMs) were incubated with indicated drugs (10 μM, 15 min) or vehicle (DMSO, 0.5%) before stimulation with ATP (5mM, 1h). Supernatant IL-1β concentration was assessed by ELISA. Data are presented as mean percentage inhibition of IL-1β release vs. vehicle (*n=4*).

Entry	Risk Category	Docking Score	MPO Score	logP	MW	HBD	HBA	logD	BBB Score	bpKa	TPSA	HAC	AR
NVR-1-1	MedHighRisk	-6.06	5.50	2.21	239.2	2	5	0.38	2.99	5.26	86.0	18	2
NVR-3-1	HighRisk	-8.38	4.42	5.19	326.2	2	5	1.75	3.46	-6.46	92.5	23	2
NVR-4-1	LowRisk	0.00	4.09	6.13	349.2	2	3	2.82	4.65	-2.31	49.3	24	2
NVR-5-1	LowRisk	0.00	3.48	5.79	426.7	3	3	2.42	4.03	-5.20	78.4	28	2
NVR-6-1	LowRisk	-1.66	3.85	5.51	393.1	3	3	2.15	4.08	-5.14	78.4	25	2
NVR-7-1	LowRisk	0.00	3.94	5.18	392.3	3	3	1.82	4.01	-5.14	78.4	27	2
NVR-9-1	LowRisk	0.00	3.81	5.32	410.2	3	3	1.88	4.01	-5.11	78.4	28	2
NVR-10-1	MedHighRisk	-6.46	4.05	5.05	376.7	3	3	1.60	4.05	-5.04	78.4	25	2
NVR-11-1	MedHighRisk	-5.45	3.06	5.84	484.6	3	3	2.43	4.20	-5.09	78.4	25	2
NVR-12-1	LowRisk	-2.74	2.81	6.11	518.2	3	3	2.71	4.13	-5.15	78.4	28	2
NVR-13-1	LowRisk	0.00	4.21	4.91	358.7	3	3	1.54	4.03	-5.08	78.4	24	2
NVR-15-1	MedHighRisk	-5.47	5.50	2.35	265.3	2	4	0.74	3.42	-1.03	83.6	20	3
NVR-16-1	MedHighRisk	-5.90	5.50	2.95	299.7	2	4	1.35	3.54	-1.03	83.6	21	3
NVR-17-1	HighRisk	-9.09	5.31	3.37	241.2	2	3	0.01	4.22	-4.00	66.4	18	2
NVR-19-1	HighRisk	-6.92	5.50	2.26	271.3	2	4	0.66	3.45	-1.03	83.6	19	3
NVR-20-1	LowRisk	0.00	5.50	2.86	305.7	2	4	1.26	3.57	-1.03	83.6	20	3
NVR-21-1	LowRisk	0.00	5.30	3.22	357.8	2	5	1.61	3.21	-1.03	92.8	25	3
NVR-23-1	HighRisk	-7.53	5.41	2.61	323.4	2	5	1.01	3.12	-1.03	92.8	24	3
NVR-25-1	MedLowRisk	-4.36	5.50	2.85	291.3	2	4	1.25	3.51	-0.99	83.6	22	3
NVR-27-1	LowRisk	0.00	5.10	2.54	351.4	2	6	0.93	2.72	-0.99	102	26	3
NVR-29-1	LowRisk	-2.47	5.41	2.19	295.3	2	5	0.59	3.05	-1.03	92.8	22	3
NVR-30-1	MedHighRisk	-6.53	4.92	3.38	402.3	2	5	1.78	3.33	-1.05	92.8	25	3
NVR-31-1	HighRisk	-8.82	4.25	4.57	425.2	2	4	1.17	4.21	-4.08	75.6	23	2
NVR-32-1	MedHighRisk	-6.10	4.45	3.30	430.3	2	6	1.70	2.93	-1.01	102	27	3
NVR-35-1	HighRisk	-9.68	3.57	4.87	348.2	2	1	4.87	4.81	-4.66	41.1	24	2
NVR-36-1	HighRisk	-9.05	3.02	5.64	427.1	2	1	5.64	4.73	-4.67	41.1	25	2
NVR-38-1	HighRisk	-8.43	5.41	2.69	321.3	2	5	1.09	3.11	-0.99	92.8	24	3
NVR-40-1	LowRisk	0.00	2.18	5.70	472.4	3	4	4.10	3.13	-1.04	95.6	33	3
NVR-41-1	LowRisk	-2.04	2.87	4.76	450.7	3	4	3.16	3.22	-1.06	95.6	30	3
NVR-42-1	LowRisk	0.00	3.45	4.30	434.3	3	4	2.69	3.18	-1.09	95.6	30	3
NVR-43-1	MedHighRisk	-6.61	3.39	4.49	417.2	3	4	2.88	3.26	-1.04	95.6	27	3
NVR-44-1	MedLowRisk	-4.01	2.90	4.65	461.6	3	4	3.05	3.35	-1.06	95.6	27	3
NVR-47-1	LowRisk	0.00	1.72	6.06	603.7	4	5	2.56	2.26	-5.31	125	32	3
NVR-48-1	HighRisk	-8.37	4.81	4.37	213.2	2	3	1.15	4.31	-1.64	49.3	16	2
NVR-49-1	HighRisk	-8.37	5.77	3.13	214.2	1	2	-0.21	4.81	-3.73	46.5	16	2
NVR-50-1	HighRisk	-8.14	5.56	3.28	198.2	1	2	-0.04	4.90	0.00	37.3	15	2
NVR-51-1	HighRisk	-8.54	5.34	3.72	212.2	1	2	0.47	4.93	0.00	37.3	16	2
NVR-53-1	HighRisk	-9.05	5.50	2.72	241.2	2	3	-0.76	4.22	-4.31	66.4	18	2
NVR-54-1	HighRisk	-8.54	4.95	3.43	256.3	3	3	0.06	3.77	-5.03	78.4	19	2
NVR-58-1	LowRisk	0.00	2.87	4.76	450.7	3	4	3.16	3.22	-1.04	95.6	30	3
NVR-59-1	HighRisk	-8.79	5.83	2.66	266.3	1	4	-0.82	3.86	-1.60	76.2	20	3
NVR-60-1	HighRisk	-8.38	5.43	3.15	257.2	2	4	-0.30	3.81	-2.09	75.6	19	2
NVR-61-1	HighRisk	-9.65	5.45	3.77	266.3	1	4	0.24	3.86	-1.45	76.2	20	3
NVR-65-1	HighRisk	-8.60	3.89	4.73	370.7	1	4	4.73	4.19	-0.14	59.9	25	3
NVR-66-1	HighRisk	-9.49	3.49	5.77	408.7	1	3	5.77	4.34	-0.13	50.7	27	3
NVR-68-1	LowRisk	0.00	3.57	6.44	396.8	1	3	6.44	4.34	0.54	50.7	27	3
NVR-72-1	LowRisk	0.00	1.70	7.52	500.3	2	0	7.52	4.33	0.00	24.1	32	2
NVR-91-1	LowRisk	0.00	3.87	4.71	375.1	1	2	4.71	4.37	-2.50	49.4	24	3
NVR-92-1	LowRisk	0.00	4.28	4.11	340.7	1	2	4.11	4.41	-2.50	49.4	23	3
NVR-93-1	MedHighRisk	-6.64	4.31	3.95	370.7	1	3	3.95	4.29	-2.40	58.6	25	3
NVR-96-1	LowRisk	0.00	2.17	5.92	500.4	3	3	4.32	3.59	-1.03	78.5	33	3
NVR-97-1	LowRisk	0.00	1.70	7.30	522.1	2	0	7.30	4.54	0.00	24.1	26	2
NVR-101-1	LowRisk	0.00	2.17	5.92	500.4	3	3	4.32	3.59	-0.94	78.5	33	3
NVR-104-1	HighRisk	-8.94	2.73	6.39	468.2	2	1	6.39	4.54	0.00	47.9	27	2
NVR-105-1	LowRisk	0.00	2.17	6.80	568.3	3	3	5.20	3.46	-1.18	78.5	37	3
NVR-111-1	LowRisk	0.00	2.61	6.63	484.2	2	1	6.63	4.42	-4.77	41.1	32	2
NVR-113-1	LowRisk	-3.36	3.04	6.52	424.3	2	1	6.52	4.24	-4.82	41.1	30	3
NVR-114-1	LowRisk	-0.05	3.10	4.76	433.4	2	3	4.76	4.46	0.01	53.6	30	2
NVR-115-1	HighRisk	-8.47	3.12	5.84	413.3	2	1	5.84	4.22	-4.84	46.1	29	3
NVR-116-1	LowRisk	0.00	2.42	6.19	464.4	3	2	6.18	3.48	4.95	69.8	33	4
NVR-117-1	HighRisk	-9.65	4.17	4.53	392.3	3	3	1.46	4.01	-4.85	78.4	27	2
NVR-118-1	HighRisk	-7.05	4.17	4.53	392.3	3	3	1.33	4.01	-4.75	78.4	27	2
NVR-119-1	LowRisk	0.00	3.51	5.79	426.7	3	3	2.37	4.03	-5.15	78.4	28	2
NVR-120-1	LowRisk	0.00	3.80	5.32	410.2	3	3	2.01	4.01	-5.25	78.4	28	2
NVR-121-1	LowRisk	0.00	3.64	5.70	406.3	3	3	2.40	4.00	-5.15	78.4	28	2
NVR-122-1	MedHighRisk	-5.66	3.67	5.70	406.3	3	3	2.33	4.00	-5.09	78.4	28	2
NVR-123-1	LowRisk	0.00	3.72	5.02	422.3	3	4	1.78	3.62	-4.70	87.7	29	2
NVR-124-1	LowRisk	0.00	3.72	5.02	422.3	3	4	1.59	3.62	-4.63	87.7	29	2
NVR-125-1	LowRisk	0.00	3.84	4.53	392.2	1	2	4.53	4.27	-2.61	49.4	27	3
NVR-131-1	LowRisk	4.80	2.60	4.90	468.7	3	4	3.30	3.21	-1.04	95.6	31	3
NVR-132-1	LowRisk	0.00	2.95	6.39	461.1	3	3	2.98	4.04	-5.20	78.4	29	2
NVR-133-1	LowRisk	0.00	2.38	5.03	484.3	3	4	3.43	3.15	-1.04	95.6	33	3
NVR-134-1	LowRisk	0.00	2.17	5.92	500.4	3	3	4.32	3.59	-1.03	78.5	33	3

NVR-135-1	LowRisk	0.00	2.38	5.03	484.3	3	4	3.43	3.15	-0.94	95.6	33	3
NVR-136-1	LowRisk	0.00	2.92	5.61	441.3	2	2	5.61	4.33	-4.80	64.9	30	2
NVR-137-1	MedHighRisk	-6.08	2.68	5.76	474.3	2	2	5.76	4.20	-4.81	67.4	32	2
NVR-138-1	HighRisk	-9.54	2.57	6.65	490.3	2	1	6.65	4.29	-6.89	50	32	2
NVR-139-1	MedLowRisk	-3.77	2.39	4.92	495.2	3	4	3.32	3.29	-0.94	95.6	30	3
NVR-140-1	HighRisk	-7.93	2.84	5.50	452.2	2	2	5.50	4.45	-4.80	64.9	27	2
NVR-141-1	MedHighRisk	-6.01	2.66	5.60	408.1	4	2	5.58	4.23	-5.43	64.5	27	2
NVR-142-1	LowRisk	0.00	2.50	6.41	506.0	2	1	6.41	4.64	-4.73	41.1	26	2
NVR-143-1	HighRisk	-9.07	2.96	6.23	435.1	2	1	6.23	4.66	-4.73	41.1	27	2
NVR-144-1	LowRisk	0.00	2.05	7.12	451.2	2	0	7.12	4.57	0.00	24.1	27	2
NVR-148-1	LowRisk	0.00	3.19	6.17	442.3	3	3	2.78	3.62	-5.17	78.4	31	3
NVR-149-1	LowRisk	0.00	3.65	5.47	428.2	3	3	2.06	4.00	-5.20	78.4	29	2
NVR-150-1	MedLowRisk	-4.46	3.34	4.87	452.3	3	5	1.52	3.20	-4.50	97	31	2
NVR-154-1	LowRisk	0.00	3.49	4.82	374.1	2	3	5.52	4.54	3.93	58.3	26	2
NVR-155-1	LowRisk	0.00	2.68	7.13	474.2	2	3	7.12	4.26	-4.41	59.6	33	2
NVR-156-1	MedHighRisk	-6.63	4.46	3.20	256.0	2	3	3.89	4.46	4.19	58.3	19	2
NVR-156-2	HighRisk	-8.40	4.46	3.20	256.0	2	3	3.89	4.46	4.19	58.3	19	2
NVR-157-1	LowRisk	0.60	3.50	5.50	356.2	2	3	5.50	4.46	-4.34	59.6	26	2
NVR-158-1	MedLowRisk	-3.62	3.21	6.45	414.8	3	3	3.14	4.01	-5.09	78.4	28	2
NVR-159-1	LowRisk	0.00	3.45	5.00	356.8	1	1	5.00	4.35	-2.10	32.3	23	3
NVR-159-2	LowRisk	0.00	3.45	5.00	356.8	1	1	5.00	4.35	-2.10	32.3	23	3
NVR-160-1	HighRisk	-8.35	3.89	4.89	340.7	1	3	4.89	4.45	0.63	50.7	23	3
NVR-162-1	MedHighRisk	-6.23	4.21	4.91	358.7	3	3	1.49	4.03	-5.09	78.4	24	2
NFA-1	HighRisk	-7.17	5.40	3.21	282.2	2	4	1.41	4.30	5.30	62.2	20	2
Clonixin-1	MedHighRisk	-6.24	5.29	3.42	262.7	2	4	1.67	4.23	5.37	62.2	18	2
FFA-1	HighRisk	-10.15	4.49	5.25	281.2	2	3	2.02	4.58	-2.14	49.3	20	2
TFA-1	HighRisk	-9.34	4.37	5.49	261.7	2	3	2.26	4.52	-2.10	49.3	18	2
Tromaril	HighRisk	-8.82	5.12	3.77	241.3	2	3	1.08	4.46	2.08	49.3	18	2
NS3728	LowRisk	-3.24	2.39	4.92	495.2	3	4	3.32	3.29	-1.06	95.6	30	3
Furosemide	MedHighRisk	-5.27	4.17	1.75	330.7	3	5	-1.25	2.48	-1.52	122.6	21	2
SB225002	LowRisk	0.00	3.88	3.52	352.1	3	4	3.08	3.41	-5.56	104.5	21	2
U-104	LowRisk	0.00	4.79	1.87	309.3	3	3	1.87	3.63	-4.70	101.3	21	2
S4	LowRisk	0.00	4.09	2.79	335.4	3	4	2.79	3.24	-4.51	110.5	23	2

Supplementary Figure 9: COX2 docking scores and physicochemical properties of the full panel of NVRs and commercial inhibitors. Each compound was docked into the COX2 crystal structure (PDB code 5IKQ) and sorted into a risk category according to their docking score. Risk categories were generated by calculating the mean docking score (-8.35) and standard deviation (σ) (1.61) of the NSAIDs Niflumic acid, Tolfenamic acid, Clonixin, Flufenamic acid and Tromaril. The criteria were as follows: High risk < -8.35 + 1σ, -8.35 + 1σ < Medium high < -8.35 + 2σ, -8.35 + 2σ < Medium low risk < -8.35 + 3σ, and Low risk > -8.35 + 3σ). ChemAxon software was used to calculate logP, molecular weight (MW), the number of hydrogen bond donors (HBD) and acceptors (HBA), logD, bpKa, topological polar surface area (TPSA), heavy atom count (HAC) and the number of aromatic rings (AR) for each compound. These physicochemical properties were used to obtain multi-parameter optimization (MPO) and blood-brain barrier (BBB) scores. The number following the dash represents the variant number of each compound, where -2 represents a different protonation state/ tautomeric form.

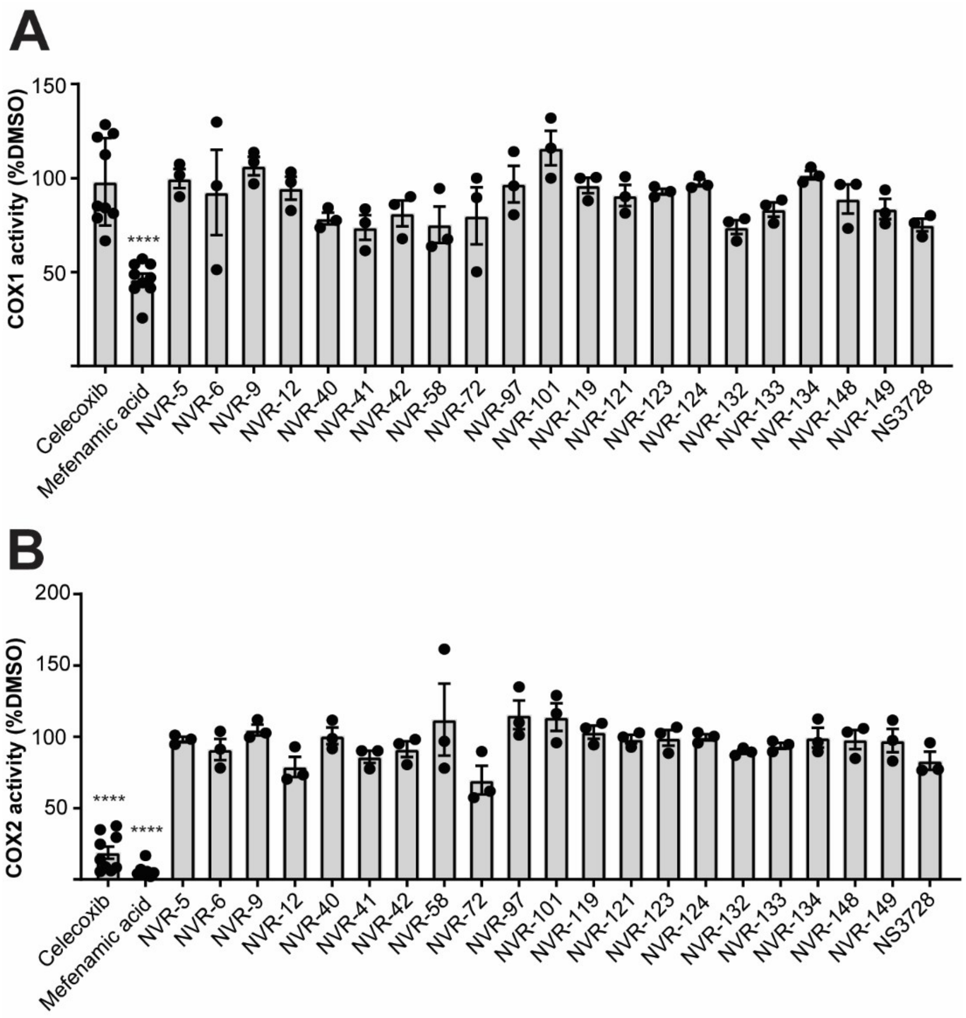

Supplementary Figure 10: The urea-based sub-set of NVR molecules do not inhibit COX enzymes. NVR molecules, celecoxib, mefenamic acid (10μM) or vehicle (DMSO (0.5%)) were incubated at room temperature with purified (A) COX1 or (2) COX2 enzymes for 30min prior to the addition of arachidonic acid, potassium hydroxide (KOH) and colorimetric substrate per manufacturer's instructions (Cayman Chemical). Drugs were assayed in duplicate in independent experiments, and celecoxib and mefenamic acid used as controls for each independent experiment. Absorbance values at 590nm were recorded, background absorbance from wells with no COX enzymes was subtracted from each value and COX activity was calculated as a percentage of vehicle. Data shown are mean ± S.E.M. (*n=3-9*). **** p <0.0001 determined by one-sample, one-tailed *t*-test versus 100% COX activity, with Holm-Sidak correction.

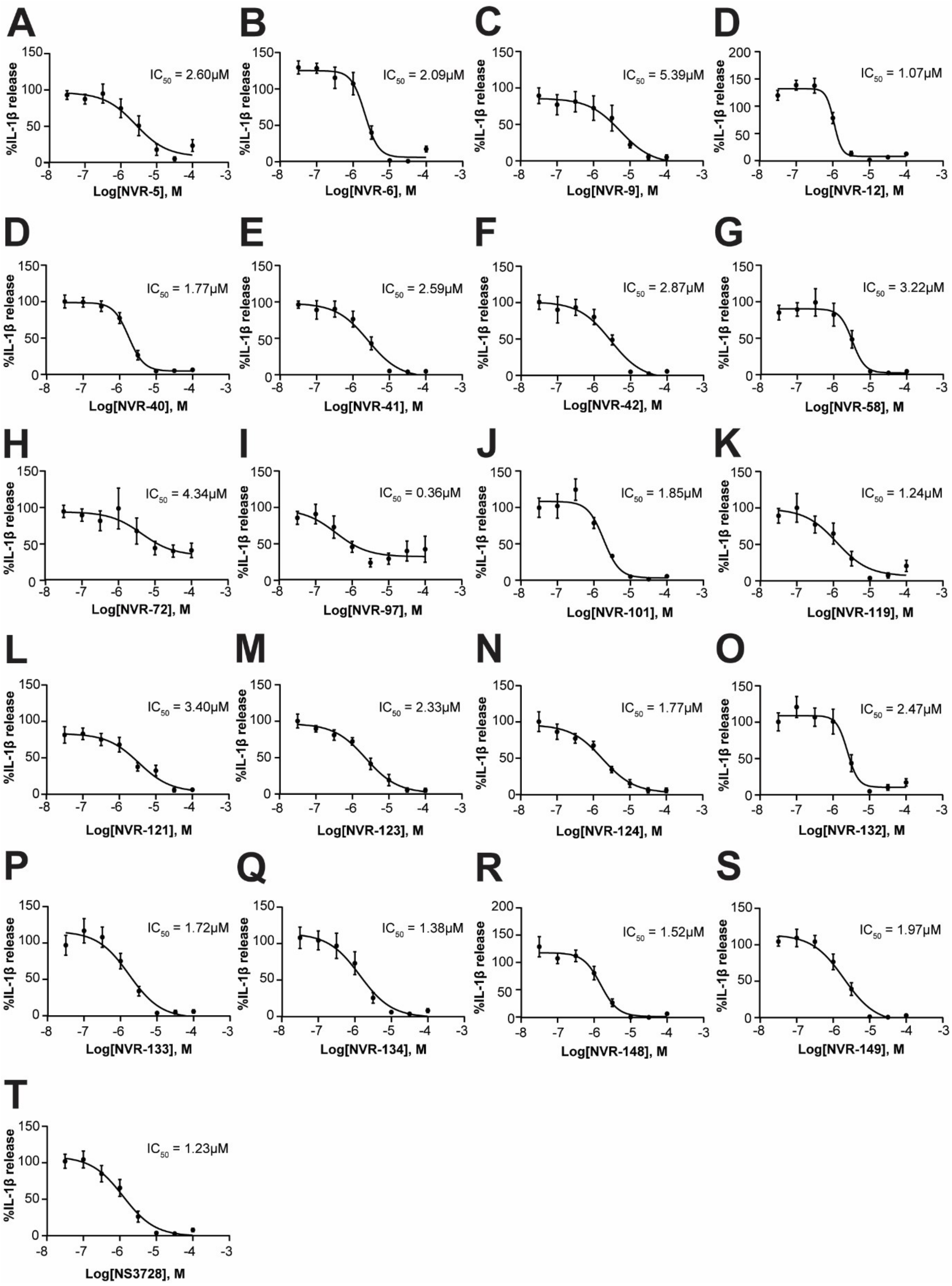

Supplementary Figure 11: Dose-response curves against IL-1β release. LPS-primed bone marrow-derived macrophages (BMDMs) (1 μg mL^{-1}; 4h) were pre-treated with either (A-S)

NVR, (T) NS3728 (0.03-100μM) or vehicle (DMSO, 0.5%) for 15 min before stimulation with ATP (5mM; 1h). IL-1β release was assessed by ELISA in the supernatants and data are expressed as a mean percentage of vehicle (± S.E.M.) of at least three independent experiments. Half-maximal inhibitory concentration (IC_{50}) values were obtained by fitting dose-response curves using either a 3- or 4-parameter logistical sigmoidal model.

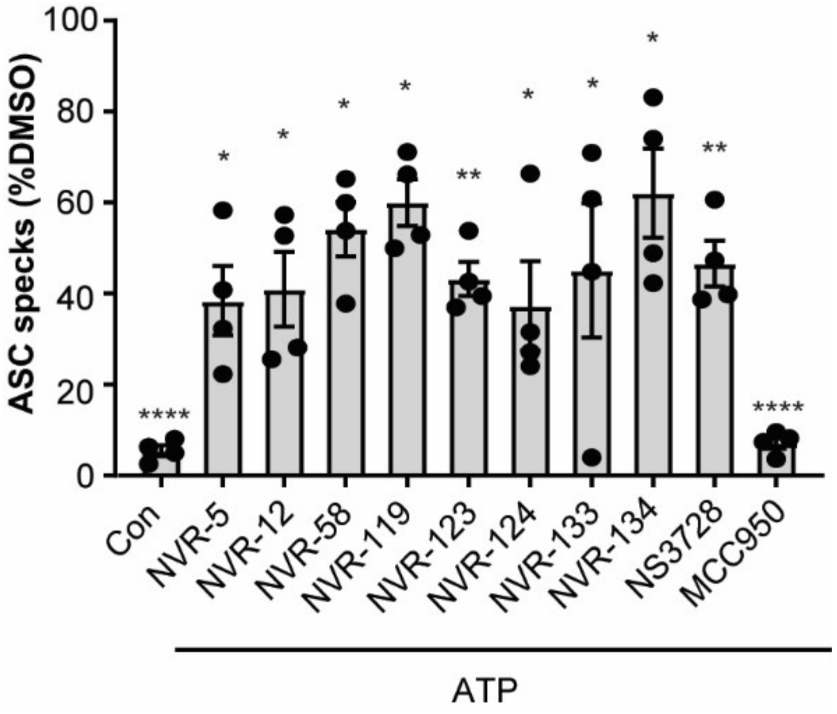

Supplementary Figure 12: Immortalised bone marrow-derived macrophages (iBMDMs) expressing ASC conjugated to mCherry protein were primed with LPS (1 µg mL^{-1}; 2h) before pre-treatment with NVR (10µM), NS3728 (10µM), MCC950 (10µM) or vehicle (DMSO; 0.5%), and Ac-YVAD-CMK (100µM) to prevent pyroptosis, for 15 min prior to the addition of ATP (5 mM; 90 min) under live microscopy. Control (Con) is no ATP stimulation (DMSO; 0.5%). Specks were quantified at the 90 min time point and presented as a percentage of ATP alone-treated cells (mean ± S.E.M (*n=4*)). ****$p<0.0001$, **$p<0.01$, *$p<0.05$ determined by one-sample, one-tailed t-test versus hypothetical value of 100% with Holm-Sidak *post-h*oc analysis. Data were assessed for normality using a Shapiro-Wilk's test.

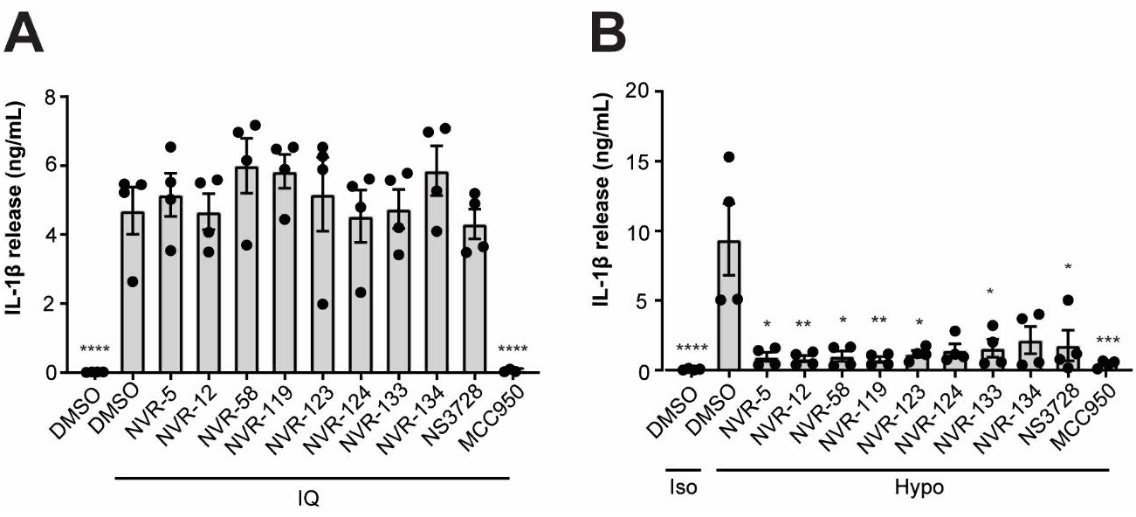

Supplementary Figure 13: (**A-B**) LPS-primed (1μg mL^{-1}; 4h) bone marrow-derived macrophages (BMDMs) were pre-treated with either NVR (10μM), MCC950 (10μM), NS3728 (10μM) or vehicle (DMSO; 0.5%) for 15 min before treatment with (**A**) imiquimod (75μM; 2h), (**B**) hypotonic (117 mOsm kg^{-1}; 4h) or isotonic buffer (340 mOsm kg^{-1}; 4h). IL-1β release was assessed by ELISA, where data are shown as mean ± S.E.M (*n=4*). **p*<0.05, ***p*<0.01, ****p*<0.001 and *****p*<0.0001, significant difference compared to vehicle determined by one-way ANOVA with Dunnett's *post hoc* analysis. Data were assessed for normality and homoscedasticity by performing a Shapiro-Wilks and Levene's test, respectively, and transformed where appropriate.

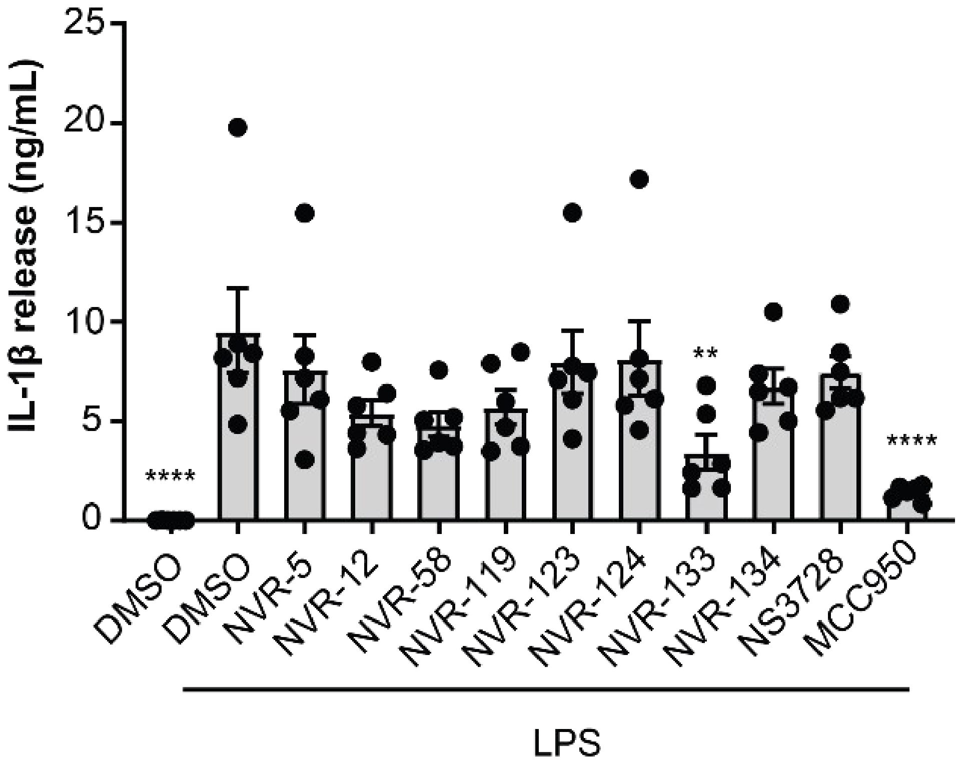

Supplementary Figure 14: LPS (1µg mL^{-1}; 20h) was added to human CD14^{+} monocytes alongside either NVR (10µM), NS3728 (10µM), MCC950 (10µM) or vehicle (DMSO; 0.5%). Supernatants were harvested and IL-1β release was assessed by ELISA. Data shown are mean ± S.E.M (*n=6*). ** $p<0.01$, **** $p<0.0001$ significant difference compared to vehicle determined by one-way ANOVA with Dunnett's *post hoc* analysis. Data were assessed for normality and homoscedasticity by performing a Shapiro-Wilks and Levene's test, respectively, and transformed where appropriate.

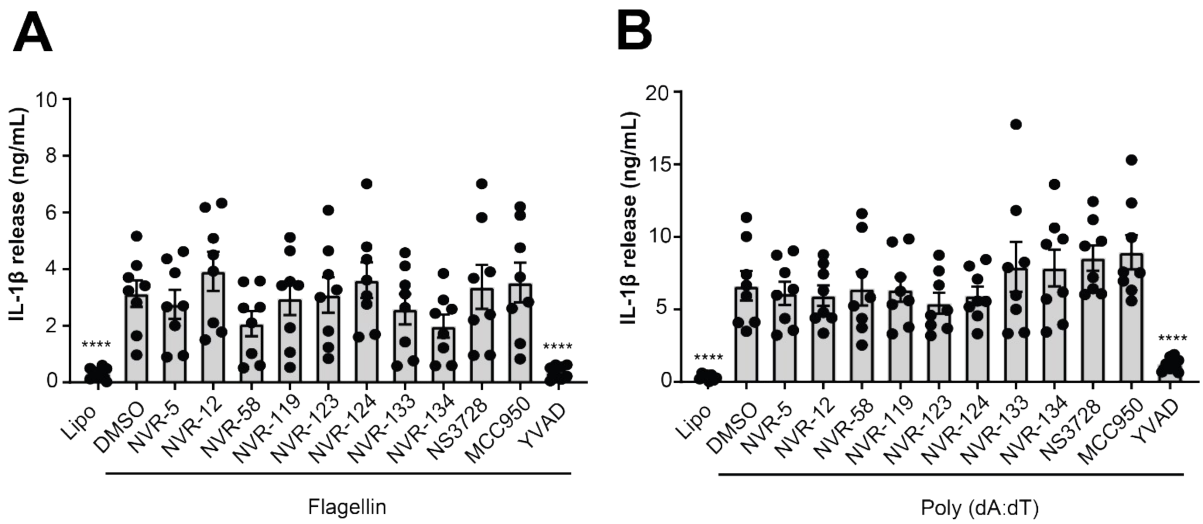

Supplementary Figure 15: For (**A**) NLRC4 and (**B**) AIM2 inflammasome activation, LPS-primed BMDMs (1µg mL^{-1}; 4h) were pre-treated with either NVR (10µM), NS3728 (10µM), MCC950 (10µM), Ac-YVAD-CMK (100µM) or vehicle (DMSO (0.5%) for 15 min followed by transfection with either (**A**) flagellin (1 µg ml^{-1}), (**B**) poly (dA:dT) (1 µg ml^{-1}) or lipofectamine alone (Lipo) for 4 h (*n=8*). ELISA was performed on the supernatants to detect IL-1β release, where data are shown as mean ± S.E.M (*n=8*). ****p<0.0001 significant difference compared to vehicle determined by one-way ANOVA with Dunnett's *post hoc* analysis. Data were assessed for normality and homoscedasticity by performing a Shapiro-Wilks and Levene's test, respectively, and transformed where appropriate.
